# Tracking industry pollution sources and health risks in China

**DOI:** 10.1038/s41598-023-49586-0

**Published:** 2023-12-14

**Authors:** Tien Foo Sing, Wenwen Wang, Changwei Zhan

**Affiliations:** 1 Department of Real Estate, Business School, National University of Singapore, 15 Kent Ridge Drive, Singapore, 119245 Singapore; 2 School of Finance, Zhejiang University of Finance and Economics, Xueyuan Street No. 18, Xiasha Higher Education Park, Hangzhou, 119245 China; 3 Institute of Real Estate and Urban Studies and Department of Real Estate, Business School, National University of Singapore, 15 Kent Ridge Drive, Singapore, 119245 Singapore

**Keywords:** Environmental economics, Environmental impact

## Abstract

Agglomeration of firms significantly increases pollution emission intensity and brings unintended consequences to public health. We develop the pollution emission indices using the firm-level pollutant emission data in China to track pollution intensities at the source using the locally weighted regression approach. Our constant-quality pollutant emission indices for three pollutants (wastewater discharge, COD, and SO_2_) and the pollution emission heatmaps show decreasing trends for the three pollutants from 1998 to 2012. We also show significant spatial clustering and regional variations in pollution emission trends. Industrial pollution mitigations in China's Eastern and Central regions have been neglected for decades since 2021, when driving economic growth took priority. The regime shifts in pollution controls from the 10th (2000–2005) to the 11th (2006–2010) Five-Year Plan period show the effects of tightening pollution emission controls. Failure to cut pollution emissions at source causes health consequences to residents living and working in nearby polluting industries. The latent environmental hazard could be a ticking time bomb, which could not be delinked from the emergence of cancer villages in the regions. Therefore, enforcing strict and uniform pollution controls and setting clear emission limits at sources can eliminate free-rider problems by polluting firms.

## Introduction

China's rapid economic growth comes with high environmental costs. Industrial firms discharge inorganic compounds and toxic gas into the water during production activities, resulting in serious water and air contamination^1,2^. The Ministry of Environmental Protection and the Academy of Engineering of China reported that over 3 million Chinese families did not have clean water, and one-third of the water system is below the safety standard.

Industrial pollution brings public health consequences. Ebenstein et al.^3,4^ found evidence of a high correlation between exposure to air- and water-borne pollution and cardiorespiratory mortality rate among residents in China. Ebenstein^5^ found that deteriorating water quality increases the digestive cancer death rate by 9% in China. More people died when exposed persistently to ambient particulate matter in the air, such as PM2.5 and PM10^6–8^. He et al.^9^ showed that a reduction of PM10 concentration in air by 10% reduces life mortality by 8%. Globally, 80% of the people in low- and middle-income countries with heavy reliance on polluting industries for economic growth live in unsafe areas with high exposure to PM2.5. People in these countries are highly vulnerable to health and mortality risks linked to industrial pollution^10^.

Local authorities collect real-time water and air quality data using sophisticated apparatus and sensors at monitoring stations distributed across the county. However, external conditions, such as wind and precipitation, topography, and river systems, can easily influence the environmental data collected onsite^11–14^. Pollutants at sources can be transmitted through wind or a river system to other regions. Using onsite data imprecisely measured and not reflective of true pollution levels at sources could misguide the government's efforts to mitigate pollution problems. The industrial emission data self-reported by firms offer an alternative but a more direct way to measure pollution at sources.

This study estimates the pollution emission intensity indices for three different pollutants (wastewater discharge, COD, and SO_2_) at the source from 1998 to 2012 using the data reported by firms, especially those in highly polluting industries. We apply the locally weighted regression (LWR) method to fit pollutant emission curves at the source, adjusting for the firm clustering effects on emission intensity at the local level. We plot the pollutant emission heatmaps to visually capture spatial autocorrelations in pollution intensity across China. We find significant regime shifts in pollution indices between the 10th Five-Year Plan (2001–2005) periods and the 11th Five-Year Plan (2006–2010) periods. China's mandates to clean out water and air in counties and cities in 2007 caused a significant reduction in pollutant emission intensities. We observed a high concentration of pollution emission sources in the 40-kilometer (km) zone of cancer villages in China (China's authority released a report in 2013 admitting the existence of cancer villages near industrial plants. About 400 cancer villages were identified, disproportionally distributed in the wealthier eastern region and near the mouth of the major river systems). Industrial pollution poses latent health risks to residents in these villages, and we can not delink the associations between cancer villages and pollutant emissions by industries^10^.

This paper makes two contributions to the literature. First, we use the LWR approach to construct time-varying pollution emission intensity indices adjusted for spatial heterogeneities and industry clustering effects at the raster level (at source). We construct the pollution emission intensity heatmaps (smoothened surfaces) for three pollutants. Second, most studies on environmental pollution used the onsite pollution data collected at local monitoring stations^12,14–17^; we add to the literature that uses firm-level data on production activities ^11,18^ and location choices^13,16,19,20^ to study firms' responses to the tightening of pollution emission regulations. With the panel of firm-year pollution emission data, the proposed quality-constant indices track time-varying pollution emissions in small geographical areas (rasters). We find firm-level evidence of regime shifts in the environmental regulations in China.

The remainder of the paper is organized as follows: “Data sources and analyses” section discusses data sources; “Pollutant emissions and cancer villages” section discusses the association between cancer villages and firm-level pollutant emissions; “Pollutant emission heatmaps” section discusses proposed LWR methodology used to construct the pollutant emission indices, conducts spatial analyses on pollutant emission indices and shows the regression results on pollution conditions before and after the regime switch; “Conclusion” section concludes the paper.

## Data sources and analyses

The data consisting of about 1.36 million self-reported firm-level pollution emission data from 1998 to 2012 were obtained from the Chinese Industrial Firm Pollution Database, the Ministry of Environmental Protection. The data include (1) firm profiles, such as firm name, year of establishment, industry code, administrative division code, and total output value; (2) the number of pollutant treatment equipment; (3) water consumption, wastewater discharge, and wastewater pollutant concentration (including chemical oxygen demand and ammonia nitrogen); (4) discharge and air pollutant concentration in waste gas, including sulfur dioxide, dust, and soot; (5) energy consumption; and (6) total amount of pollutants reduction.

The major pollutant emission data cover industrial firms nationwide. We measure three types of pollutants: wastewater, Chemical Oxygen Demand (COD), and sulfur dioxide (SO_2_) because (1) they are the most harmful pollutants in the manufacturing sector^11^; (2) many countries measure the same set of environmental pollutants; (3) the disclosure of these three pollutant concentrations were continuous and complete from 1998 to 2012, while other pollutants, such as soot and dust were not disclosed in 2011 and 2012. This is the most comprehensive dataset on Mainland Chinese industrial pollutant emissions, covering more than 85% of the total emissions and discharges in the regions except for Tibet.

We keep the sample Chinese firms in the two-digit SIC codes from 13 to 42. The industry codes changed several times in the past few years: The 1998–2002 industry code adopted the national economic industry classification in 1994 (GB/T4754-1994), the 2003–2012 code adopted the national economic industry classification in 2002(GB/T4754-2002). We unify and consolidate the 1998–2002 industry codes using the 2002 national economic industry classification codes. The administrative divisions have also undergone several major changes. The official versions of the *Code of Administrative Divisions of the People's Republic of China*" (GB/T2260.1995, 1999, 2002, and 2007 editions) were released in 1995, 1999, and 2002, respectively. We merge all the administrative division codes of the industrial firm database from 1998 to 2012 into the GB/T2260-2002 version of the code to keep the coding uniform.

**Figure 1:**
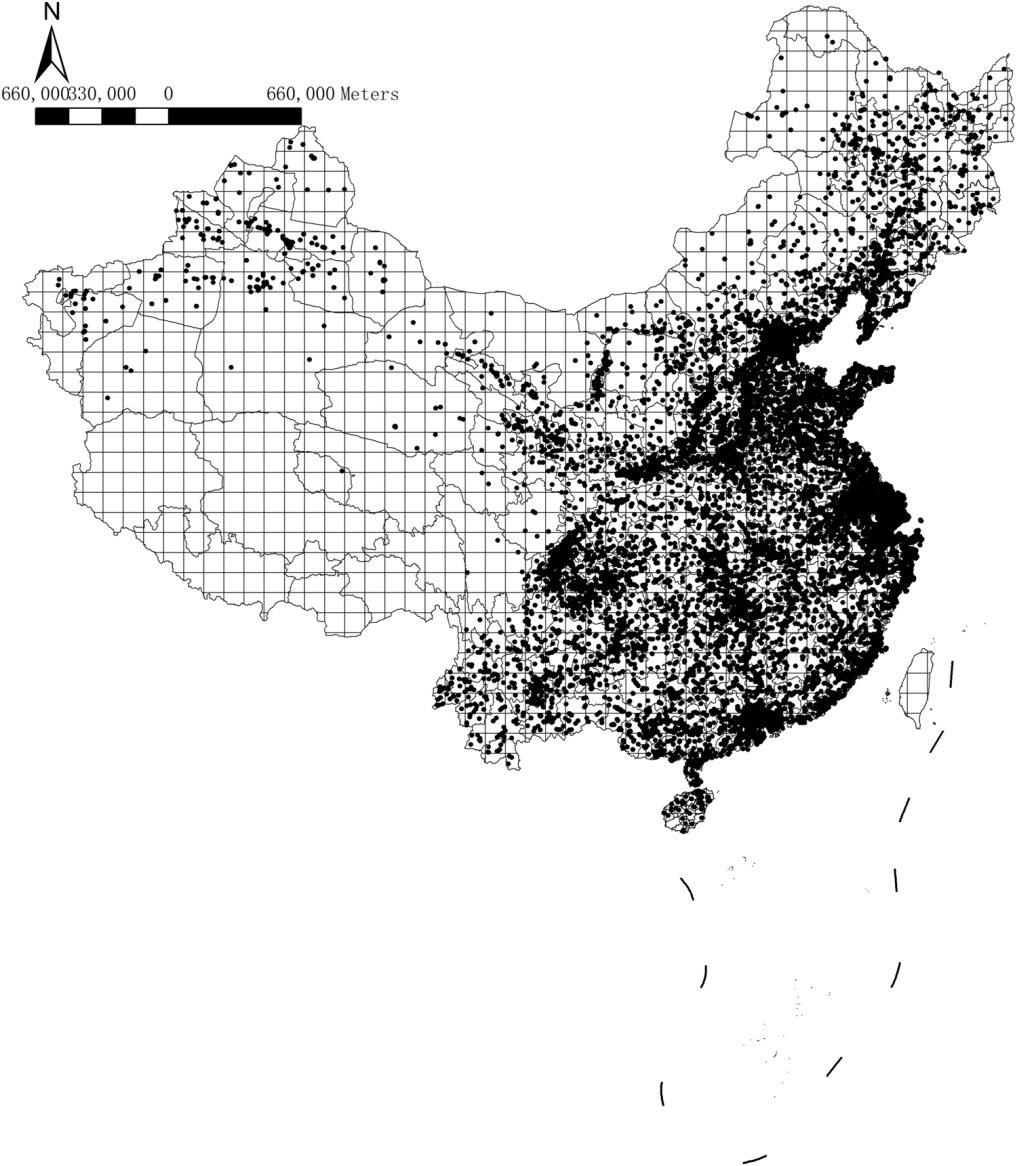
Distributions of firms and the local-area grids. Notes: this figure shows the distributions of firms in our sample, where each black dot represents one sample firm. The sample firms exclude firms from Hong Kong, Macau, and Taiwan. Other firms in the Western region's provinces, such as Xinjiang, Tibet, Qinghai, and Inner Mongolia, are also dropped due to the small number of firms in the area. The China map is divided by the 100 km × 100 km grids to define the local area into equal-size rasters, which create the pollution emission intensity heatmaps (the map was generated by ArcGIS 10.7 https://www.esri.com/en-us/arcgis/products/arcgis-desktop/resources).

The database contains domicile details of firms at the prefecture and county levels but not the provincial levels. We use the administrative division codes to identify the provincial-level details and extract the latitude and longitude coordinates of firm domiciles using the Baidu Application Programming Interface (API) and the location fields. We convert the latitude and longitude data and re-code them following the international coordinate system (WGS1984). The sample firms are sorted into rasters defined by 100 km × 100 km squared grids, where the local rasters pin down the pollution sources precisely. The database creates a more accurate and complete identification of the geographic locations of firms. Figure 1 shows the geographical distributions of sample firms. All the maps in base shapefiles are downloaded from the public resource (https://github.com/dongli/china-shapefiles) and no permission is needed.

## Pollutant emissions and cancer villages

Industrial pollution causes latent but long-term health risks to residents in neighboring villages. An article published in the Lancet raised "*serious health and social concerns*" linking soaring cancer rates in selected villages to air and water pollution from nearby industries^21^. The Chinese Government acknowledged the existence of cancer villages in a report released on February 21, 2013.

**Figure 2:**
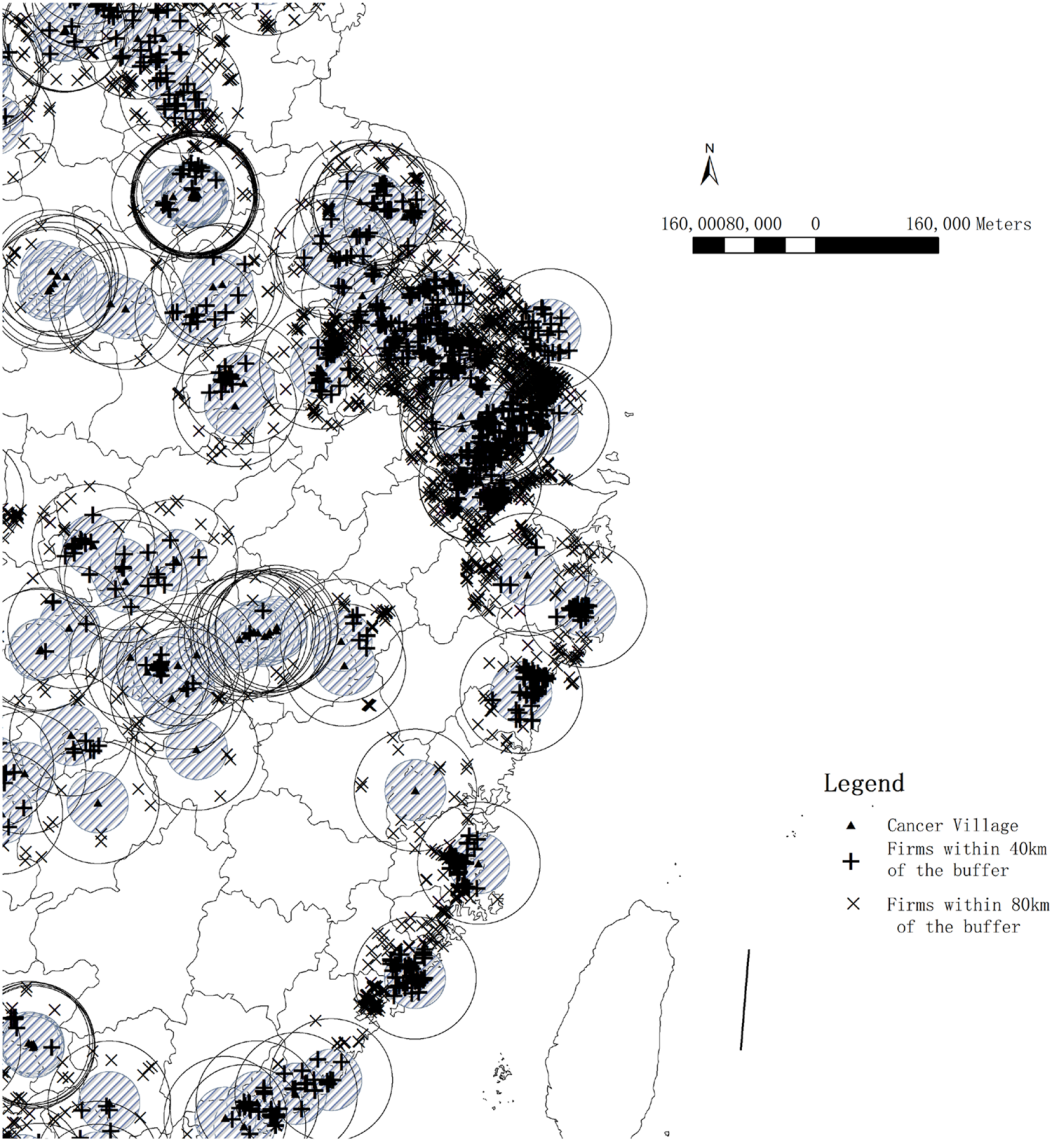
Distributions of cancer villages and the buffer zones. Notes: this figure shows the locations of about 440 cancer villages identified in the Chinese County Economy Yearbook. The areas surrounding the cancer villages are divided into two zones: the treatment zone covering the inner ring area of 40 kilometers (km) (radius) from the centroid and the control zone covering the outer ring area between 40 and 80 km (the map was generated by ArcGIS 10.7 https://www.esri.com/en-us/arcgis/products/arcgis-desktop/resources).

The locations of about 440 cancer villages in 81 counties across China were revealed in the Chinese County Economy Yearbook. To test the association between cancer villages and industrial pollution, we define two buffer rings, comprising an inner ring of a 40 kilometers (km) radius from the centroid of a cancer village denoted by a dummy, "*buffer*" = 1, and an outer ring between 40 and 80 km from the centroid denoted by a dummy, "*buffer*" = 0 (Fig. 2). The *t-tests* show that the socioeconomic attributes of the two buffer areas in 2007 were not significantly different (the t-test results are summarized in Table A1 in the Online Appendix 1). The three variables in the tests are (1) the proportion of primary and secondary school students in the registered population (*student*), (2) the proportion of employees in secondary industry in the registered population (*secondary_emp*), (3) government expenditures in general public budgets (*gov_expend*, unit: million RMB).

We regress pollution emissions by firms (wastewater, COD, and SO_2_) on the "*buffer*" dummy to test if inner ring areas surrounding cancer villages are correlated with high pollution intensity. The equation is given as follows:

$$\begin{aligned} {\text{Pollutant}}_{{{\text{it}}}} & = \upalpha _{{0}} + {{\upalpha }}_{{1}} {\text{buffer}}_{{{\text{it}}}} + {{\upalpha }}_{{2}} {\text{buffer}}_{{{\text{it}}}} \times {\text{k\_FYP}}_{it} + {{\upalpha }}_{{3}} {\text{Lnsize}}_{{{\text{it}}}} + {{\upalpha }}_{{4}} {\text{Lnage}}_{{{\text{it}}}} \\ & \quad + {{\upalpha }}_{{5}} {\text{Roa}}_{{{\text{it}}}} + {{\upalpha }}_{{6}} {\text{Leverage}}_{{{\text{it}}}} + {{\upalpha }}_{{7}} {\text{State\_owned}}_{{{\text{it}}}} + {{\upalpha }}_{{8}} {\text{Foreign\_owned}}_{{{\text{it}}}} \\ & \quad + {{\upalpha }}_{{9}} {\text{Pop\_density}}_{{{\text{it}}}} + {{\upalpha }}_{{{10}}} {\text{Unemployment}}_{{{\text{it}}}} + {{\upalpha }}_{{{11}}} {\text{Gdp\_per\_capita}}_{{{\text{it}}}} + \lambda_{{\text{i}}} + \mu_{t} \\ \end{aligned}$$

Pollutant_it_ refers to pollutant emission by firm *i* (in logarithm) (wastewater discharge, COD, and SO_2_) in year *t*. We include an interactive term "*buffer*_*it*_ × *k_FYP*_*it*_," where "k = 10" refers to the 10th FYP period from 2001 to 2005, and "k = 11" refers to the 11th FYP period after 2006, to test changes in pollution intensities between the two economic regimes. We control for the firm-level attributes, such as employee number (in logarithm) ("*Lnsize*"), log-firm age ("*Lnage*"), total export to revenue ratio ("*Export*"), return on asset ("*Roa*"), debt to equity ratio ("*Leverage*"), and the two firm dummies: a firm registered as a state-owned enterprise ("*State_Owned*"), and a foreign firm ("*Foreign_Owned*"), and the city-level attributes, such as population density("*Pop_intensity*") and GDP per capita ("*Gdp_per_capita*"). We also include the industrial and year-fixed effects for unobserved variations. The result is significant if the coefficient of the fixed effects model is significant at less than 10% level (the descriptive statistics of the key variables are summarized in Panel B of Table A2 in the Online Appendix 1).

Table 1 shows that the intensities of wastewater discharges (Column 1) and COD emissions (Column 2) are significantly higher in the inner ring areas of cancer villages. The interactive "*buffer* × *k_FYP*" coefficients have negative signs but are insignificant for the two FYP periods in the wastewater discharge models (Column 1). Column 2 shows that the coefficient is insignificant when interacting with the 10th FYP dummy but significantly negative when interacting with the 11th FYP dummy. The results imply that COD emissions were significantly reduced in areas near the cancer village after 2006. Column 3 shows that SO_2_ emission intensities near the cancer villages significantly increased during the 11th FYP period. The results could not delink the possibility that residents exposed to high concentrations of wastewater discharges, COD, and SO_2_ emissions for a long time were vulnerable to cancer risks. Industrial pollution brings negative externalities, increasing health risks for residents living and working near emission sources.

**Table 1:** Pollution emissions between cancer village counties and control counties.

Variables	(1)	(2)	(3)
	Lnwastewater	LnCOD	LnSO_2_
*buffer*	0.061***	0.199***	− 0.007
	(0.021)	(0.025)	(0.022)
*buffer* × *k_10*	− 0.001	− 0.051	0.032
	(0.035)	(0.042)	(0.036)
*buffer* × *k_11*	− 0.036	− 0.120***	0.152***
	(0.036)	(0.043)	(0.037)
*Lnsize*	0.985***	0.896***	0.797***
	(0.007)	(0.009)	(0.007)
*Lnage*	0.025***	− 0.002	− 0.008
	(0.009)	(0.011)	(0.009)
*Export*	− 0.008	0.027	− 0.151***
	(0.016)	(0.02)	(0.017)
*Roa*	− 0.05	0.168**	0.162***
	(0.061)	(0.073)	(0.063)
*Leverage*	2.56E−05	2.47E−05	− 3.42E−05
	(2.10−E05)	(2.53−E05)	(2.17−E05)
*State_Owned*	0.197***	0.0953***	− 0.083***
	(0.022)	(0.026)	(0.022)
*Foreign_Owned*	0.139***	0.035	− 0.191***
	(0.020)	(0.024)	(0.021)
*Pop_intensity*	7.74E−05***	− 6.75E−05***	− 0.0002***
	(1.67−E05)	(2.02E−05)	(1.73E−05)
*Gdp_per_capita*	0.0341***	− 0.019***	− 0.041***
	(0.003)	(0.004)	(0.004)
Constant	4.993***	3.600***	5.576***
	(0.047)	(0.056)	(0.0481)
Industry dummy	Yes	Yes	Yes
Year dummy	Yes	Yes	Yes
Observations	50,913	50,913	50,913
R-squared	0.399	0.33	0.406

This table shows the regression results of the pollutant emissions: wastewater, COD, and SO_2_ inside and outside cancer villages. The dummy "*buffer*" = 1 defines an area within the 40 kilometers (km) radius of a cancer village, "*buffer*" = 0 covers an outer ring area from 40 to 80 km. The interactive terms "*buffer* × *k_FYP*," where "k = 10" denotes the 10th FYP period from 2001 to 2005, and "k = 11" denotes the 11th FYP period after 2006. We control for the firm-level attributes, which include the number of employees (in logarithm) by the end of the year ("*Lnsize*"), log-firm age ("*Lnage*"), total export to revenue ratio ("*Export*"), return on asset ("*Roa*"), debt-to-equity ratio ("*Leverage*"), and the two firm dummies: a firm registered as a state-owned enterprise, including alliances of SOEs and unlisted state-owned limited companies ("*State_Owned*"), and a firm registered as a joint venture or cooperative with HK, Macau, Taiwan or foreign entities ("*Foreign_Owned*"), and the city-level attributes, which include population density in term of 10,000 population per 1 squared kilometers (km^2^) of land area ("*Pop_intensity*"), unemployment rate ("*Unemployment*") and GDP per capita ("*Gdp_per_capita*"). The model also includes the industry and year-fixed effects. The standard errors are in parentheses. ***p < 0.01; **p < 0.05; *p < 0.1.

## Pollutant emission heatmaps

We divide China's geographical area by the "100 km × 100 km" grids and aggregate the firm-level wastewater discharges within each grid to derive the grid-level wastewater discharge distributions (Fig. 1). The total wastewater discharges vary from 0 to 3.48 × 10^8^ tons, with an average of 229,470.2 tons. We estimate the grid-level COD emissions, which vary from 0 to 1.54 × 10^8^ Kilograms (kg), with an average of 53,260.78 kg. The grid-level SO_2_ emissions vary from 0 to 2.18 × 10^9^ kg, averaging 72,830.78 kg.

We use the locally weighted regression (LWR) approach to interpolate pollutant discharges relative to the centroid of each grid and fit the pollutant emission curves using the Modified Shepard's Algorithm (the discussions of the empirical methodology are covered in the “Online Appendix 1”). Due to the sparse distribution of firms in the Western regions, we dropped some Western provinces, which include Xinjiang, Tibet, Qinghai, and Inner Mongolia, from the map. Our data do not cover the Hong Kong, Macau, and Taiwan regions, which were dropped from the maps. We derive the emission indices by normalizing the estimated emissions at the grid level for wastewater, COD, and SO_2_ using the equation below:

$$Index_{ij} = \frac{{r_{ij} - \mathop {\min }\limits_{i} \left\{ {r_{ij} } \right\}}}{{\mathop {\max }\limits_{i} \left\{ {r_{ij} } \right\} - \mathop {\min }\limits_{i} \left\{ {r_{ij} } \right\}}}$$

where $r_{ij}$ is the interpolated pollution emission levels for pollutant *j* (wastewater, COD, or SO_2_) at grid *i*; and $\mathop {\max }\limits_{i} \left\{ {r_{ij} } \right\}$ and $\mathop {\min }\limits_{i} \left\{ {r_{ij} } \right\}$ are the maximum and minimum pollution emission levels for pollutant *j* in each grid from 1998 to 2012, respectively.

**Figure 3:**
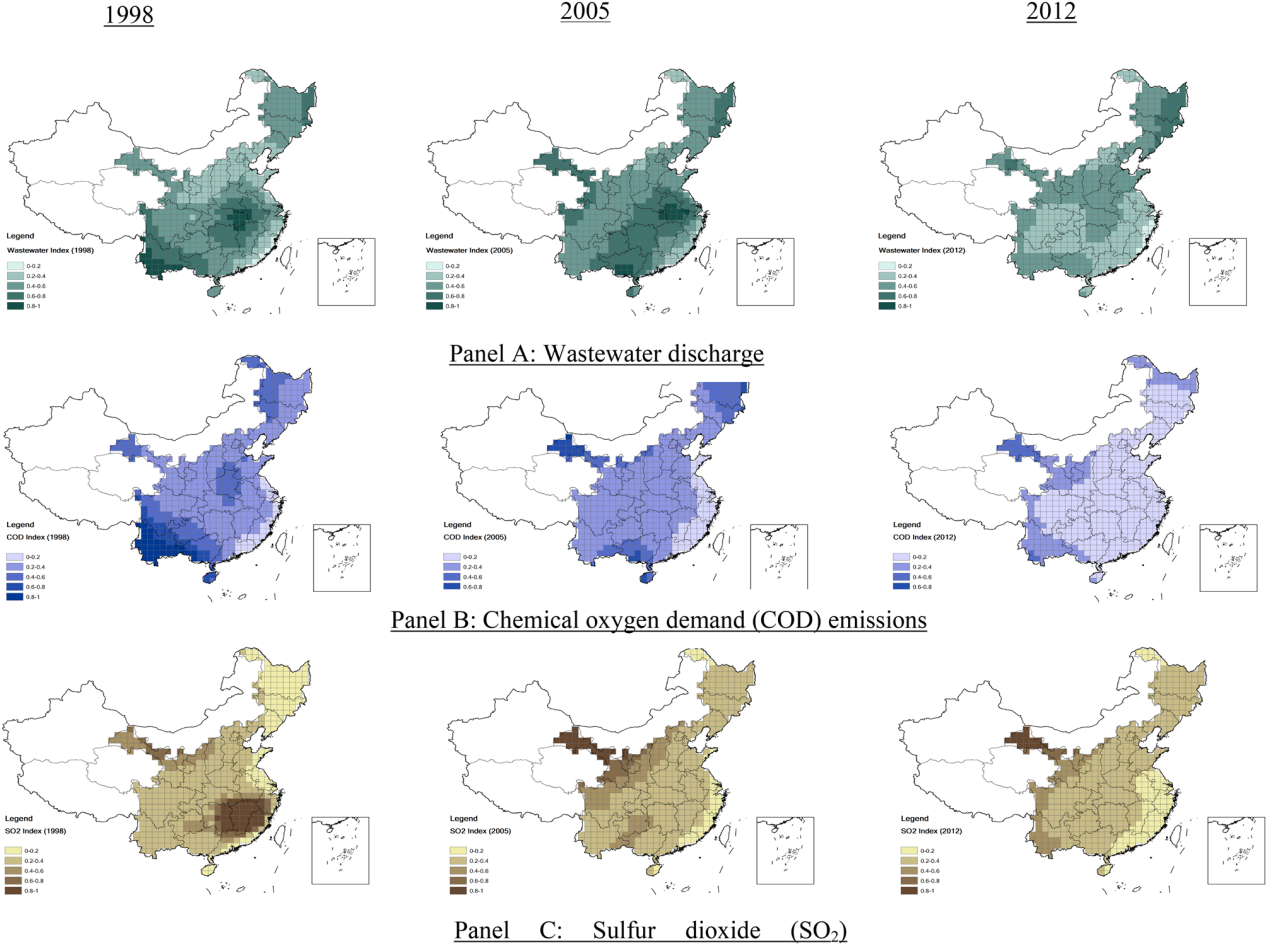
Spatial evolutions of the pollution emission intensities for wastewater, COD, and SO_2_. Notes: this figure shows the spatial distributions of pollution emissions estimated using the locally weighted regression (LWR) model, adjusted for spatial heterogeneity, where each local area is defined by a raster bounded by the 100 km × 100 km grids on the China map. The LWR model estimates the emission intensity for three pollutants: (**A**) wastewater discharge, (**B**) chemical oxygen demand (COD) emission, and (**C**) sulfur dioxide (SO_2_) each year from 1998 to 2012. The Figure shows only the pollution map for 1998, 2005, and 2012 due to space constraints (the map was generated by ArcGIS 10.7 https://www.esri.com/en-us/arcgis/products/arcgis-desktop/resources).

Figure 3 plots the multi-year pollutant emission "*heatmaps*" by geographical regions. We find wide variations in the distributions and pollution intensities across regions, and the high-pollution areas are mostly found in the Eastern and Southern regions, where a darker color represents a higher intensity of pollutants.

### Evolution and spatial variation of indices

**Figure 4:**
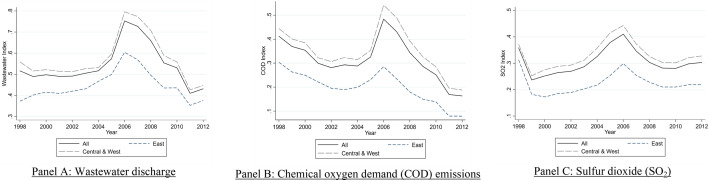
The pollution emission indices for wastewater, COD, and SO_2_ for the years 1998–2012. Notes: This figure shows the pollution emission indices: (**A**) wastewater discharge, (**B**) chemical oxygen demand (COD) emission, and (**C**) sulfur dioxide (SO_2_) from 1998 to 2012. The graphs contain three different indices, where "All" as represented by the dark black line, covers all the provinces in China; "East," as represented by the blue line, covers the provinces in the Eastern region; and "Central & West" as represented by the light black line, covers the provinces in the Western and Central regions (the map was generated by ArcGIS 10.7 https://www.esri.com/en-us/arcgis/products/arcgis-desktop/resources).

Figure 4 shows evolutions of the nationwide and regional indices of wastewater discharge, COD, and SO_2_ emissions from 1998 to 2012. The wastewater, COD, and SO_2_ emission indices declined by − 16.5%, − 60.4%, and − 15.5%, respectively, from 1998 to 2012. The three indices show clear regime switches in the pollution trends that increased from 1998 to 2006, peaked in 2006, and declined from 2006 to 2012. The declines in pollution emissions over the period coincided with the "top-down" pollution control policies during the 11th FYP. The Central Government set clear targets to reduce COD and SO_2_ but not for wastewater discharge. The local governments focus on reducing COD and SO_2_ emissions. Thus, larger declines were observed in the COD and SO_2_ emission indices relative to the wastewater discharge indices. The pollution indices for the Eastern region are also consistently lower than those for the Central and Western regions.

Our sample period covers the two major pollution-mitigating policies in China. The 10th Five-Year Plan (10th FYP) aimed to cut major pollutant emissions by 10% from the level in 2000 before 2005. The 10th FYP introduced a series of laws and regulations, including the Environmental Impact Assessment Law and the Cleaner Production Promotion Law. Chinese Officials charged firms with steeply rising penalties for almost all pollution discharges in 2003^18^. Except for the COD pollution index that declined after 2001, the wastewater and SO_2_ pollution indices show upward trends. The local government leaders prioritized economic growth over the emission reduction targets during the 10th FYP period^11^. As a result, the three pollution emission indices significantly increased and deviated from the 10th FYP pollution mitigation targets.

The 11th FYP strengthened air and water pollution emission controls by setting the target of reducing major pollutants by 10%. The Circular Economy Promotion Law was introduced in 2008 to tighten the enforcement. The Central Government put more pressure on the Local Governments to meet the pollution reduction targets. The top-down approach in pollution emission controls was effective, showing significant improvements in combating polluting activities in the 11th FYP. Figure 4 shows significant declines in the three pollutant emission indices (wastewater, COD, and SO_2_) after 2006, exceeding the targets by the end of 2010.

The 1998, 2005, and 2012 heatmaps in Fig. 3 show significant spatial variations in wastewater discharge, COD, and SO_2_ emissions in China. The wastewater discharges were concentrated in Hubei, Hunan, Anhui, and Jiangxi provinces in the Central region, Yunnan and Guangxi provinces in the Southwestern region, and Heilongjiang, Jilin, and Liaoning provinces in the Northeast region. The wastewater discharge indices significantly increased in these provinces, widening the pollution gap with other regions in 2005. Wastewater discharges have significantly reduced in most regions in 2012, except for the Northwestern and Northeastern regions.

COD emissions were concentrated in Yunnan and Guangxi provinces in the Southwestern region in 1998. The emission intensity shifted gradually from the East to the West between 2005 and 2012. COD emissions show the most significant decrease among the three pollutants. SO_2_ emissions in Hunan, Jiangxi, Zhejiang, and Fujian provinces in the Central and Southeastern regions disappeared after 2005. The 2012 emission heatmaps show lower intensity in the eastern region than in the western region.

We observe two patterns in the spatial emission intensity distributions. First, the declines in emission intensities were not uniform but varied across different regions. The Eastern region has a relatively lower emission level. The emission levels declined significantly in the Central but not in the Western regions. The lax pollution controls induced the "pollution haven" effects in the Western region. Second, more significant pollution reductions were observed in 2005–2012 than in 1998–2005, affirming that tightened environmental controls were more effective in reducing pollution emissions in the 11th FYP than the 10th FYP.

### Spatial autocorrelations in pollution emissions

Moran's I measures spatial autocorrelations in pollution emissions at the global and local levels. It ranges from − 1 to 1, where the negative and positive signs represent spatial autocorrelations in opposite directions, and 0 indicates no spatial autocorrelation. We calculate the global Moran's I value for pollution discharges from 1998 to 2012 and find a relatively large global Moran's I for the three pollutants. The pollution sources are not randomly distributed and have strong spatial dependence, suggesting that high-pollution and low-pollution emission areas cluster spatially. Wastewater discharge indices declined from 0.895 to 0.851, COD emission indices from 0.905 to 0.826, and SO_2_ emission indices from 0.935 to 0.902. The declining Moran's I value shows the spatial dispersion in pollution discharges and emissions (Table A3 in the Online Appendix 1 summarizes Moral's I of the three pollutants over the sample period).

**Figure 5:**
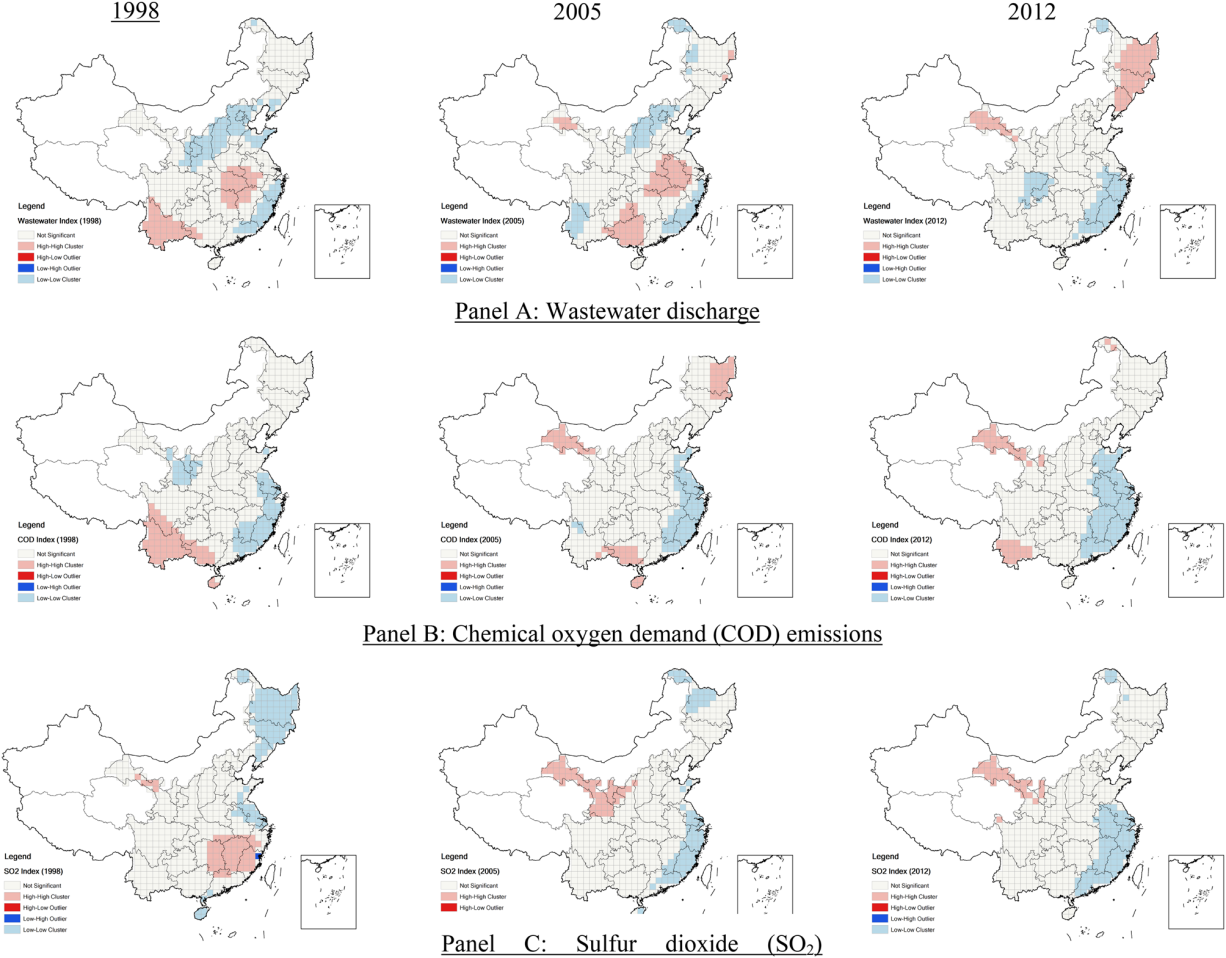
LISA cluster map of indices of wastewater, COD, and SO_2_. Notes: this figure computes the Anselin Local Moran's I to analyze spatial agglomeration for the discretized rasters defined by the 100 km × 100 km grids. The above colored-coded heatmaps, or the LISA ("Local Indicators of Spatial Association")^22^ cluster maps, the three pollution emission intensities: (**B**) chemical oxygen demand (COD) emission, and (**C**) sulfur dioxide (SO_2_), in 1998, 2005, and 2012 (the map was generated by ArcGIS 10.7 https://www.esri.com/en-us/arcgis/products/arcgis-desktop/resources).

We use the Anselin Local Moran's I to analyze spatial agglomeration for each discretized geographic unit represented by the 100 km × 100 km grids. Figure 5 shows the LISA ("Local Indicators of Spatial Association")^22^ colored-coded heatmaps for the three pollution emission intensities in 1998, 2005, and 2012. The 1998 LISA map for wastewater discharges shows that the "high-high" clusters were mainly found in five neighboring provinces in the Central region and Yunnan province in the Southwest region. The "low-low" clusters were found in Beijing-Tianjin-Hebei, Shandong, Shanxi, and Shaanxi provinces in the Northern region and Zhejiang, Fujian, and Guangdong provinces in the Southern coastal region. The 2005 LISA map shows that the distributions were more scattered but remained the same in the clustering areas. The 2012 LISA map shows that the "high-high" clusters moved to Gansu province in the Northwestern region and Heilongjiang, Jilin, and Liaoning provinces in the Northeastern region. The "low-low" clusters expanded in the Southeastern region in 2012.

The "high-high" COD pollutant clusters shrunk in Yunnan, Guangxi, and Hainan provinces in the Southwestern region between 1998 and 2012. The new "high-high" clusters appeared in Gansu and Heilongjiang provinces, whereas the "low-low" COD cluster in Gansu province disappeared. The "low-low" cluster expanded significantly in the Eastern coastal region, spreading to the Central regions. During the same periods, the "high-high" SO_2_ emission clusters moved from Hunan, Jiangxi, and Fujian provinces in the southern region to Gansu province in the northern region. Larger "low-low" clusters appeared in the Eastern coastal region, whereas smaller "low-low" clusters were found in the Northeastern region by 2012.

We observe three features of local spatial autocorrelations in pollution emission intensities. First, the global Moran's I show that distributions of "high-high" and "low-low" clusters are unevenly spread across the regions. Pollutive firms agglomerate close to each other spatially, implying that firms' choice of manufacturing plant location was not randomized. Second, firms in the high emission clusters shifted from the Central and Southern regions (in provinces of Hubei, Hunan, Jiangxi provinces, etc.) and the Southwestern region (in Yunnan and neighboring provinces) to the Western region (such as in Gansu province).

Third, the policy interventions were effective in cleaning out air and water in the highly pollutive provinces in the Eastern region. The "low-low" clusters expanded along the coastal belt from Shandong, Jiangsu, Zhejiang, Fujian, and Guangdong provinces in the Eastern and Southern regions to Anhui and Jiangxi provinces in the Central region. The pollution abatement efforts by the local regulators were unequal, pushing footloose polluting firms to seek shelters in pollution havens.

### Regime-Shifts in pollution emissions

We employ the ordinary least squares (OLS) regression models (is the linear regression model used in the empirical tests for the parameter estimations that provide the best-fit function for the data that minimizes squared errors) to test changes in wastewater discharge, COD, and SO_2_ indices between the 10th FYP and the 11th FYP regimes. We regress the dependent variables defined by wastewater discharge, COD, or SO_2_ emissions of grid *i* in year *t* against the time trend variable and run the regressions separately for the 10th FYP period and the 11th FYP period using the specification below:

$$Index_{it} = {\upalpha } + \beta_{1} year_{t} \times east_{i} + \beta_{2} year_{t} + X_{kt} {\Gamma } + \lambda_{k} + \varepsilon_{it}$$

where the interaction term, *year* × *East*, tests the regional difference, and the dummy *east* equals 1, if a grid is in the Eastern region; otherwise, 0. $X_{kt}$ is a vector of control variables in year *t* in province *k* bounded by the grids. Following the literature^23,24^, we include the controls on industry structure and demographic factors, such as GDP per capita (*gdppc*), contributions of secondary industry to GDP (*secondary*), contributions of tertiary industry to GDP (*tertiary*), and urbanization rate (*urbanrate*). The data are obtained from the China City Statistical Yearbook. The model includes the grid fixed effects, $\lambda_{k}$, to control for unobserved prefecture-level variations in pollution emissions. The summary statistics are included in Panel B of Table 2.

**Table 2:** Regressions on indices of wastewater, COD, and SO_2_ during the 10th FYP.

	(1)	(2)	(3)	(4)	(5)	(6)
	Wastewater	COD	SO_2_	Wastewater	COD	SO_2_
East*year				0.004**	− 0.005***	− 0.014***
				(0.002)	(0.002)	(0.002)
*year*	0.018***	0.008***	0.030***	0.017***	0.009***	0.032***
	(0.001)	(0.001)	(0.001)	(0.001)	(0.001)	(0.001)
*gdppc*	0.004	0.005	0.003	0.001	0.008*	0.011
	(0.003)	(0.004)	(0.005)	(0.003)	(0.005)	(0.008)
*secondary*	0.004***	− 0.002***	− 0.001**	0.004***	− 0.002***	− 0.001**
	(0.001)	(0.001)	(0.001)	(0.001)	(0.001)	(0.000)
*tertiary*	0.006***	0.000	0.001**	0.006***	0.000	0.001
	(0.001)	(0.001)	(0.001)	(0.001)	(0.001)	(0.001)
*urbanrate*	− 0.001	− 0.001*	− 0.002***	− 0.001*	− 0.001*	− 0.002***
	(0.000)	(0.000)	(0.001)	(0.000)	(0.000)	(0.001)
Grid FE	Yes	Yes	Yes	Yes	Yes	Yes
Observations	2352	2352	2352	2352	2352	2352
R-squared	0.878	0.864	0.951	0.878	0.865	0.954

This table summarizes the results of the regressions with the three pollution emissions indices as the dependent variables: wastewater, COD, and SO_2_. The regression covers the 10th FYP period from 2001 to 2005. The treatment variable is the interaction term, ["*year* × *East"*], where "year" indicates the year of the indices and "east" dummy equals 1 if a grid is located in the Eastern region; otherwise, 0. $X_{kt}$ is a vector of control variables in year *t* in city *k* (provincial-level) bounded by the grid in our map, which include GDP per capita (*gdppc*), contributions of secondary industry to GDP (*secondary*), contributions of tertiary industry to GDP (*tertiary*), and urbanization rate (*urbanrate*). The data are obtained from the China City Statistical Yearbook. The model also includes the grid fixed effects, $\lambda_{k}$. The standard errors are in parentheses and clustered at the grid level. ***p < 0.01; **p < 0.05; *p < 0.1.

Table 2 shows the first set of OLS regression results using the 10th FYP samples. In Columns (1)–(3) without the interaction term "*year* × *east,"* the estimated coefficients on *year* are positive and statistically significant at the 1% level. During the 10th FYP, the three pollutant sources increased sharply because the local governments prioritized economic growth at the expense of deteriorating water and air quality. In Columns (4) to (6) with the interaction term "*year* × *east,"* the "*year*" coefficient is positive and significant at 1% level, showing increasing trends in the pollution emissions for the three pollution sources. The interactive coefficients in the three pollution models are significant but with different signs. The coefficient is significantly positive in the wastewater model, but the coefficients are negative in the COD and SO_2_ models at less than 5% significance level. The results show that wastewater discharge increased at a faster rate, but COD and SO_2_ emissions increased at a slower rate in the Eastern region compared to other regions during 2001–2005. Firms emitting high levels of COD and SO_2_ were more footloose moving from the Eastern region to other regions, whereas other firms with activities highly dependent on water sources were more location-bound.

The results for the 11th FYP period in Table 3, Columns (1) to (3) without the interactive term, show significant decreasing trends in the three pollution indices. In Columns (4) to (6) with the interactive term, the negative and significant "*year*" coefficients show that the three pollution emission sources declined over the years. The interaction coefficients are negative in the wastewater model but positive in the COD and SO_2_ models. The Eastern region experienced a slower rate of increase in COD and SO_2_ during the 10th FYP but a faster rate of decrease in COD and SO_2_ during the 11th FYP.

**Table 3:** Regressions on indices of wastewater, COD, and SO_2_ during the 11th FYP.

	(1)	(2)	(3)	(4)	(5)	(6)
	Wastewater	COD	SO_2_	Wastewater	COD	SO_2_
east*year				− 0.011**	0.024***	0.023***
				(0.005)	(0.004)	(0.004)
*year*	− 0.035***	− 0.073***	− 0.052***	− 0.035***	− 0.074***	− 0.053***
	(0.004)	(0.003)	(0.004)	(0.004)	(0.003)	(0.003)
*gdppc*	− 0.016**	0.016***	0.018***	− 0.010	0.002	0.005
	(0.008)	(0.006)	(0.006)	(0.008)	(0.006)	(0.007)
*secondary*	− 0.006***	− 0.004**	− 0.005***	− 0.006***	− 0.005**	− 0.006***
	(0.002)	(0.002)	(0.002)	(0.002)	(0.002)	(0.002)
*tertiary*	− 0.004**	− 0.005**	− 0.000	− 0.003	− 0.007***	− 0.002
	(0.002)	(0.002)	(0.002)	(0.002)	(0.002)	(0.002)
*urbanrate*	− 0.012***	− 0.005*	− 0.007***	− 0.012***	− 0.006**	− 0.007***
	(0.004)	(0.003)	(0.002)	(0.004)	(0.003)	(0.002)
Grid FE	Yes	Yes	Yes	Yes	Yes	Yes
Observations	1413	1413	1413	1413	1413	1413
R-squared	0.921	0.969	0.933	0.922	0.971	0.935

This table summarizes the results of the regressions with the three pollution emissions indices as the dependent variables: wastewater, COD, and SO_2_. The regression covers the 11th FYP period from 2006 to 2010. The treatment variable is the interaction term, ["*year* × *East"*], where "year" indicates the year of the indices and "east" dummy equals 1 if a grid is located in the Eastern region; otherwise, 0. $X_{kt}$ is a vector of control variables in year *t* in city *k* (provincial-level) bounded by the grid in our map, which includes GDP per capita (*gdppc*), contributions of secondary industry to GDP (*secondary*), contributions of tertiary industry to GDP (*tertiary*), and urbanization rate (*urbanrate*). The data are obtained from the China City Statistical Yearbook. The model also includes the grid fixed effects, $\lambda_{k}$. The standard errors are in parentheses and clustered at the grid level. ***p < 0.01; **p < 0.05; *p < 0.1.

The emission intensities of the three pollutant sources decreased faster in the eastern region during the 11th FYP period. The different signs for the interactive coefficients imply significant regime shifts in COD and SO_2_ pollution controls between the 10th FYP and the 11th FYP periods. The Central Government's efforts to tighten pollution controls in the 11th FYP were effective in curbing the pollution haven effects caused by movements of footloose polluting firms. It incentivized firms to invest in more efficient equipment and install advanced pollution-mitigating measures to internalize pollution externalities, which is consistent with the pollution Porter hypothesis (See the distributions of firms in the 10 most pollutive industry sectors by the 2-digit SIC and the growth rates by of firms in the 10th and 11th FYPs are shown in Figure A1 in Online Appendix).

## Conclusion

Discharging contaminated wastewater and emitting toxic air are spatially dependent and autocorrelated locally^20,25–28^. The Intensity of emissions increases when firms agglomerate locally^28^. This paper constructs the pollution emission indices using the LWR approach with the firm-level pollution data. We generate the spatially adjusted pollution emission intensities for equal-sized discretized rasters. We plot the heatmaps to visually show spatial evolutions in pollution emission intensities for wastewater discharge, COD, and SO_2_ from 1998 to 2012.

We show the importance of tracking highly fluid pollution sources. Our study shows heterogeneity in polluting firms and spatial variations in pollution emissions. Two opposing forces exist in driving pollution sources. On the one hand, the agglomeration of firms increases the intensity of pollution emissions locally. On the other hand, pollution haven effects drive some pollution firms to areas with less stringent pollution controls.

Our policy implications are twofold. Local governments should tighten and unify pollution controls and standards to mitigate environmental degradation that harms public health. First, they can incentivize firms to internalize pollution externality by investing in technologies, switching to clean and renewable energy, and improving pollutant filtering systems. They can collaborate with local governments in neighboring counties and provinces to enforce consistent and uniform pollution controls to discourage the relocation of polluting firms. Instead, they should only attract value-added and technology-intensive firms with less pollutive production processes. Second, local governments can mandate and impose pollution targets at the source to eradicate free-riding problems, such as firms located along major rivers. This can prevent firms from causing negative externalities to residents near the downstream of rivers.

The study has some limitations. First, the study covers only the sample period from 1998 to 2012 due to the unavailability of the firm-level pollutant emission data. Second, we could not establish causal inferences between health outcomes and pollution sources due to the lack of micro-data on cancer villages. However, the LWR spatially distributed pollutant indices show high pollution intensity patterns near cancer villages. The study can be updated in the future subject to the accessibility to data sources in China.

### Supplementary Information

Supplementary Information 1.

Supplementary Information 2.

Supplementary Information 3.

## Data Availability

The data that support the findings of this study are available on request from the corresponding author.
