# Supplementary material for: Tracking industry pollution sources and health risks in China
Source: Sci Rep. 2023 Dec 14;13:22232. doi: 10.1038/s41598-023-49586-0 (PMC10721918; doi:10.1038/s41598-023-49586-0)
Supplement: Supplementary file 1 — Supplementary Information 1. [file 41598_2023_49586_MOESM1_ESM.docx]

**Online Appendix1**

Figure A1 shows the 10 most pollutive industry sectors by the 2-digit SIC and growth rates by the firm number in the 10^th^ and 11^th^ FYPs. Panel A shows a high concentration of firms in Raw Chemical Material and Chemical Products (26) and Textile Industry in the Eastern region, and firms in “Smelting and Pressing of Non-Ferrous (32)” and Ferrous Metal (33) and the “Papermaking and Paper Product (22)” in the Western and Central regions. Firms in “Smelting and Pressing (32 and 33)” and "Petroleum Processing, Cooking Products, Gas Production, and Supply (25)" grew at the fastest rate in the 10^th^ FYP in the Western and Central Regions. However, these three industries declined significantly by number during the 11^th^ FYP period. The firm growth rates were relatively slower in the Eastern region, and firms in "Smelting of Non-ferrous Metal (32)", "Plastic Products (30)", and "Leather, Fur, Down and Related Products (19)" industries grew the fastest during the 11^th^ FYP.


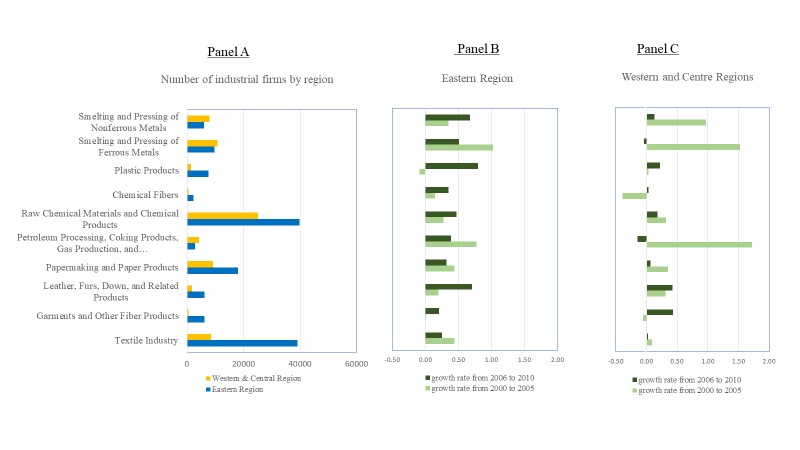


**Figure A1: Distributions and Growths of 10 Pollutive Industrial Firms by Type**

Notes: This figure shows the distributions of the 10 pollutive industries by the first 2 digits of the SIC Code from 2000-2012. Panel A shows the distributions of industrial firms by the number of firms registered in the sample by the Eastern Region, Western Region, and Central Region. Panels B and C show the growth in the number of industrial firms in the 10th (2000-2005) and 11th (2006-2010) Five Year Plan (FYP) periods and by Eastern, and Western & Central Regions, respectively.

**Table A1: T-tests on socioeconomic characteristics of cancer village counties and control counties**

| Variable | Mean  (cancer village counties) | Mean  (control counties) | t-value | p-value |
| --- | --- | --- | --- | --- |
| student | 0.137 | 0.141 | 0.832 | 0.406 |
| secondary_emp | 0.067 | 0.067 | 0.065 | 0.948 |
| gov_expend | 750.382 | 793.636 | 0.447 | 0.655 |

Notes: This table compares the socioeconomic characteristics of the two groups of counties, which include the cancer village counties located within the 40 km buffer ring of the centroid of the village and other counties located in the buffer rings between 40 km and 80 km. The table summarizes the t-test statistics on three variables, which include (1) the proportion of primary and secondary school students in the registered population (student), (2) the proportion of employees in secondary industry in the registered population (secondary_emp), (3) government expenditures in general public budgets (gov_expend, unit: million RMB). The p-value of less than 0.05 indicates that the socioeconomic characteristics are not significantly different between the two groups of counties.

**Table A2: Summary statistics**

| Variable | Meaning | | Unit | Mean | SD | Min | Max |
| --- | --- | --- | --- | --- | --- | --- | --- |
| Panel A: firm-level analysis (pollutant emissions and cancer villages) | | | | | | | |
| *Lnwastewater* | | Natural log of firm wastewater discharge | Ln(ton) | 11.308 | 2.103 | 0.000 | 19.254 |
| *LnCOD* | | Natural log of firm COD emission | Ln(Kg) | 9.051 | 2.407 | -2.303 | 17.217 |
| *LnSO2* | | Natural log of firm SO2 emission | Ln(Kg) | 9.994 | 2.185 | 0.000 | 21.503 |
| *buffer* | | =1 if a firm is within a 40 kilometers (km) radius from the centroid of a cancer village, otherwise it takes | / | 0.536 | 0.499 | 0.000 | 1.000 |
| *k_10* | | “k = 10” denotes the 10th FYP period from 2001-2005 | / | 0.270 | 0.444 | 0.000 | 1.000 |
| *k_11* | | “k=11” denotes the 11th FYP period after 2006 | / | 0.251 | 0.433 | 0.000 | 1.000 |
| *Lnsize* | | Natural Log of employee number | Ln(person) | 6.044 | 1.184 | 0.000 | 11.816 |
| *Lnage* | | Natural Log of Years of Business Running | / | 2.866 | 0.920 | 0.000 | 7.605 |
| *Export* | | Total export to revenue ratio | % | 0.150 | 0.462 | -9.752 | 61.790 |
| *Roa* | | Return on asset | % | 0.025 | 0.123 | -2.471 | 9.637 |
| *Leverage* | | Debt to equity ratio | % | 4.369 | 344.856 | -34044 | 44365 |
| *State_Owned* | | State_owned=1 if the firm is registered as a state-owned enterprise or unlisted state-owned limited company, otherwise State_owned=0 | / | 0.360 | 0.480 | 0.000 | 1.000 |
| *Foreign_Ownerd* | | Foreign_owned=1 if a firm is registered as a joint venture or cooperative with HK, Macau, Taiwan, or foreign entities | / | 0.395 | 0.489 | 0.000 | 1.000 |
| *Pop_intensity* | | 10,000 population per squared kilometer (km2) of land area | person/km2 | 762.917 | 470.991 | 16.250 | 4018 |
| *Gdp_per_capita* | | GDP per capita | RMB/person | 2.446 | 2.684 | 0.186 | 34.229 |
| Panel B: grid-level analysis | | | | | | | |
| *Wastewater* | Normalized index of wastewater emissions | | / | 0.543 | 0.160 | 0 | 1 |
| *COD* | Normalized index of wastewater emissions | | / | 0.317 | 0.177 | 0 | 1 |
| *SO2* | Normalized index of wastewater emissions | | / | 0.306 | 0.149 | 0 | 1 |
| *east* | =1 for eastern region, =0 for central and western regions | | / | 0.227 | 0.419 | 0 | 1 |
| *year* | Indicator of year | | / | 2005 | 4.321 | 1998 | 2012 |
| *gdppc* | GDP per capita | | 10,000 RMB/person | 1.737 | 2.093 | 0.164 | 45.202 |
| *secondary* | Share of secondary industries in GDP | | % | 44.173 | 11.221 | 9.000 | 89.700 |
| *tertiary* | Share of tertiary industries in GDP | | % | 37.066 | 8.364 | 8.500 | 85.340 |
| *urbanrate* | Urbanization rate | | % | 31.559 | 16.741 | 7.350 | 100.000 |

Notes: The table summarizes variables we employ in empirical analyses.

**Table A3: Summary of global Moran's I**

|  | (1) | (2) | (3) |
| --- | --- | --- | --- |
| Year | *Wastewater* | *COD* | *SO2* |
| 1998 | 0.895 | 0.905 | 0.935 |
| 1999 | 0.889 | 0.902 | 0.769 |
| 2000 | 0.865 | 0.870 | 0.777 |
| 2001 | 0.851 | 0.818 | 0.769 |
| 2002 | 0.854 | 0.795 | 0.763 |
| 2003 | 0.835 | 0.795 | 0.764 |
| 2004 | 0.792 | 0.796 | 0.749 |
| 2005 | 0.788 | 0.796 | 0.750 |
| 2006 | 0.718 | 0.855 | 0.755 |
| 2007 | 0.784 | 0.861 | 0.753 |
| 2008 | 0.824 | 0.872 | 0.762 |
| 2009 | 0.765 | 0.862 | 0.770 |
| 2010 | 0.792 | 0.872 | 0.756 |
| 2011 | 0.778 | 0.816 | 0.782 |
| 2012 | 0.851 | 0.826 | 0.902 |

Notes: This table summarizes global Moran's I of three pollutants (wastewater, COD, SO_2_) from 1998 to 2012.

**Unified Region Code and Industry Code**

The adjustments made to the Region Codes and Industry Codes are also appended separately in the Online Appendix for reference.

**China's Pollution Emission Mandates**

China's economic reform has driven rapid urbanization and industrialization since the 1970s (Ebenstein, 2012). The urbanization process causes severe degradation of environmental quality. Many Chinese cities reported an alarmingly high level of airborne particulate matter (PM) that causes serious health concerns (Zheng & Kahn, 2013, 2017). Industrial pollution brings latent long-term health risks to human beings.

With the decentralization of responsibilities in regulating environmental policies, local mayors and officials have no incentives to strictly enforce environmental standards, resulting in a "*race to the bottom*" in environmental protection. Instead, local mayors compete to outdo each other in luring investments from highly pollutive firms (Shen et al., 2017; Wang & Chen, 2014; Wang et al., 2019). Firms exploit privileges granted to them by the local authorities at the expense of inducing negative environmental externalities (Cai et al., 2016; Duvivier & Xiong, 2013; Kahn, 2004; Kahn et al., 2015; Sigman, 2002). These firms discharge inorganic compounds into the water, such as chemical oxygen demand (COD) and ammonia nitrogen pollutant concentrations, and emit toxic gas, such as sulfur dioxide (SO2), from their production plants (Jiang et al., 2014; Lindberg et al., 2014; Zhou et al., 2017).

The Chinese Government recognizes that pursuing economic growth at all costs is unsustainable (Shen et al., 2017). It could also cause long-term and irreversible damage to the health of residents (Ebenstein, 2012; Ebenstein et al., 2015; Ebenstein et al., 2017). In 2001, the Chinese Government mandated the environmental targets in the 10th Five Year Plan 2001-2005 to cut pollution discharges by 10% by the end of 2005 (The State Council, 2001). However, the effects of the pollution reduction mandates were far from satisfactory, with only meager improvements to environmental quality (Cai et al., 2016).

The Central Government of China changed its approach to setting the environmental mandates in the 11^th^ Five-Year Plan (2006-2010). The Chinese Government signaled its resolution to abate environmental degradation by changing the political rank promotion criteria that favor "*green*" leaders (Kahn et al., 2015). The Ministry of Environmental Protection (MEP) tightened the enforcement via the "*Pollution Reduction Performance Assessment*" system in 2007 to hold local officials accountable for the pollution reduction targets (Wu et al., 2017).

While the tightened environmental target regime reduced pollution emissions, the

outcomes were not uniformly distributed across counties and provinces. Pollution mandates that varied between the Eastern and Western regions motivated polluting firms to move upstream along the major rivers to the Western regions of China, with relatively less stringent environmental mandates (Cai et al., 2016; Shen et al., 2017; Wu et al., 2017). These firms continue to free-ride and smog villages at the border and discharge wastewater downstream of the rivers, a typical pollution haven dilemma (Duvivier & Xiong, 2013; Wu et al., 2017)（The transboundary pollution is also prevalent in bordered countries in other parts of the world Sigman, H. (2002). International Spillovers and Water Quality in Rivers: Do Countries Free Ride? American Economic Review, 92(4), 1152-1159. https://doi.org/10.1257/00028280260344687 and in the USA Kahn, M. E. (2004). Domestic pollution havens: Evidence from cancer deaths in border counties. Journal of Urban Economics, 56(1), 51-69. <https://doi.org/10.1016/j.jue.2004.02.001>）. However, not all firms relocate to pollution havens to avoid environmental compliance costs. The pollution Porter hypothesis predicts that firms in areas with strict environmental regulations internalize the environmental compliance costs of emitting airborne pollution.

**Constructing Pollution Emission Intensity Indices**

Environmental economists use two sources of empirical data to study free-riding and pollution haven problems. The first data source includes the county-level and prefectural-level pollution emission data, mostly available only after 2000, found in official publications, including the Urban Statistical Yearbooks of China and China Environmental Yearbooks. Public agencies, such as the Environmental Protection Bureau (EPB) in different counties and the Ministry of Environmental Protection (MEP), administer the data collection process and report the pollution statistics, usually aggregated at the provincial and county level. The data are collected in real-time at different monitoring stations distributed along rivers or custom-built stations across cities. However, the onsite pollution data provide timely but indirect reporting of pollution levels at destinations but not at sources. Many past studies used the pollution emission data to directly proxy pollution outcomes (Kahn et al., 2015; Wang & Chen, 2014), and others used the data to explain variations in economic outcomes (Cai et al., 2016; Ebenstein et al., 2015; Ebenstein et al., 2017; He et al., 2016).

The second data source consists of disaggregated data on production activities, locational choice, industry sector of firms, and mandatory self-reporting pollution emission data by firms. Some studies correlate firms' locations and production activities to public data on pollution emissions, which could not directly measure pollution emission intensity at the source. Due to industrial agglomeration (Wang & Chen, 2014), pollution emissions at the source are intensified by firms clustering in a small area. Pollution emission measurements collected at multiple stations distributed across geographical locations could vastly underestimate the pollution effects. Like Jiang et al. (2014), which used the firm-level self-reporting pollution data, we used the same data source from a larger sample of firms to measure pollution intensity at sources（The paper by Jiang, L., Lin, C., & Lin, P. (2014). The determinants of pollution levels: Firm-level evidence from Chinese manufacturing. Journal of Comparative Economics, 42(1), 118-142. https://doi.org/10.1016/j.jce.2013.07.007 is one of the few studies that uses the direct self-reporting pollution emission data by a sample of 2862 firms in 2006 and 4261 firms in 2007 in China. The national database is representative covering a large sample of manufacturing industries in all the provinces in China. The local EPBs will monitor and ensure the data reporting is reliable and accurate）. We collected the pollutant emission data from 1998 to 2012 from the National Bureau of Statistics. The original data contains 1.36 million observations reported by Chinese firms categorized by the SIC 4-digit industry sector. We use the Application Programming Interface (API) to crawl the coordinate data of firms (latitude and longitude) in batches from Baidu Maps and geocode them with the World Geodetic System (WGS 1984).

We use the locally weighted regression (LWR) （LWR estimates the kernel weight function for a set of target points, such that a higher weight is given to observations that are close to the target point, and then adjust the estimated values for other sample points in neighboring regions）methodology proposed by (Cleveland & Devlin, 1988), which is also widely used in the urban economic literature (McMillen & Redfearn, 2010; Meese & Wallace, 1991), to estimate pollution intensity accounting for firms clustering effects in a small area.

Contaminated water and toxic air are not static but move rapidly across cities and regions(Li, 2014; Wang et al., 2013). Pollution levels are dependent on firm distributions and spatial attributes at the source. Pollution concentration is not measured additively, which otherwise could ignore spatial attenuation in an area. The "local" pollution intensity changes continuously with the distance to a pollution center. However, few past studies, if any, have empirically and explicitly tested the spatially-dependent pollution intensity, which allows pollution levels to diminish with distance to the source. Some studies could not capture non-linear changes across neighborhoods by using discrete neighborhood dummies. The use of more sophisticated spatial regression models commonly found in the urban economics literature, such as spatial autoregressive models (SAR) and spatial autoregressive error term models (SARAR), are also scarce in the environmental literature.

The proposed LWR methodology adjusts spatial changes to the model parameters, revealing measurable and unmeasurable spatial heterogeneity in the environment, land, school, etc. The LWR coefficients in the model change continuously with the observation space (Cleveland & Devlin, 1988). The LWR uses a kernel weight function to estimate the spatial weights for a set of target points, where a higher weight is assigned to an observation point close to a target point, and an equal estimated value is given to the same region. The LWR performance is sensitive to the choice of the Kernel function and target points（Studies on improving the distance weighting scheme using different target systems are still limited）. The distance-decay kernel weighting scheme in LWR is optimized by setting the bandwidth, window size, and distance metric selection (i.e., straight-line distance, network distance, and travel time.

***1. Nonparametric estimation based on LWR***

The LWR is estimated by minimizing α and β in the equation below:

$$\sum_{i=1}^{n} \left(y_{i}-\alpha-\beta^{'}\left(X_{i}-X\right)\right)^{2}K\left(\frac{X_{i}-X}{h}\right)$$

where x_i_ and x represent the latitude and longitude coordinates of a polluting firm and a target point, respectively, and y_i_ is the value at the target point. K(ψ) determines the weight of the observation point x_i_ relative to the target point x. 𝜑 is the distance-decay expression, with 𝜑=$\frac{x_{i}-x}{h}$, so that [I (| 𝜑 | <1) > 0)], which defines the speed of weight decay with distance, and h is the bandwidth, which represents the number of observations with positive weights within a region close to a target point. We use a strategy that fixes the number of observations within a changing bandwidth (i.e., the "window" of observations versus a fixed bandwidth). This strategy is superior to a fixed bandwidth strategy because it simultaneously eliminates the "excessive-smoothing" and "excessive-volatility" problems of nearby observations with different densities around a target point (McMillen & Redfearn, 2010).

The choice of the kernel from among the many different kernel functions has no significant effect on the estimation results due to the decreasing weight for 𝜑. Like the literature, we use the Tri-Cube function（$\frac{70}{81}$（1- | 𝜑 | 2）3 *𝐼（| 𝜑 | <1）as the kernel weight.

***2. Target point selection***

There are three methods for selecting the target point (𝑥) when fitting the LWR model: (1) using a full sample of observations target points; (2) selecting a group of "artificial" target points based on a predefined algorithm; and (3) using a set of referenced locations, such as actual firm locations, as the target points. We interpret the estimated coefficients in Methods (2) and (3) with reference to selected target points.

In the target (evaluation) point selection, we need to make a trade-off between "interpolation cost" and "local fitting cost" to attain a more efficient LWR estimation. The interpolation cost is reduced if target points are kept as small as possible on the one hand. The "over smooth" problem (local fitting cost) is avoided by selecting target points with enough information to approximate the "true" local regression surface on the other hand. If the full set of target points is used, we retain as much information in the local fitting, but our estimation time significantly increases, thus reducing the model efficiency. Loader (1999) suggests that target points can be artificially defined based on distributions of the observations, or a subsample of these observations can be selected. The running speed is greatly improved, regardless of the target point method, relative to the full set of target points. Intuitively, using "artificial target points" that define a regular grid, such as the 100km x 100km rasters used in this study, can significantly reduce problems concerning the interpolation of regression surfaces.

We use the "adaptive tree" algorithm (Loader, 1999) to construct a grid of points partitioned by a bandwidth (also known as the smoothing parameter) containing positively weighted observations with the same density. A set of vertices on the grid is set as target points. The longitude and latitude coordinates of the vertices on the grid are used as the predictive variables in the local surface fitting.

More specifically, the adaptive tree method first groups the data into rectangular boxes of different shapes and then uses each box's vertices as target points. At the midpoint of each connected vertex pair, each rectangular box is divided into two equal-sized subsets, and we repeat the splitting process until each granulated box represents the level of a local pollutant emission surface. This method can significantly reduce the interpolation cost by selecting a region with a small bandwidth containing more continuous fitting points with easily distinguishable features. The segmentation is based on the score assigned to the edge of each box, which is defined as:

$$\rho_{i,j}=\frac{\left\|v_{i}-v_{j}\right\|}{\min\left(h_{i},h_{j}\right)}$$

where (ℎ_𝑖_, ℎ_𝑗_) is the bandwidth defined for vertices ν_i_ and ν_j_. When 𝜌_𝑖, 𝑗_ is less than a critical value, where the default setting is 0.8, the splitting process stops. The cubic polynomial interpolation is defined as:

$$\begin{matrix} \hat{\mu}(x)=(1-\lambda)^{2}(1+2\lambda)\hat{\mu}\left(v_{0}\right)+\lambda^{2}(3-2\lambda)\hat{\mu}\left(v_{1}\right) \\ \text{ }+\left(v_{1}-v_{0}\right)\left(\lambda(1-\lambda)^{2}\mu^{'}\left(v_{0}\right)-\lambda^{2}(1-\lambda)\mu^{'}\left(v_{1}\right)\right) \end{matrix}$$

where$\lambda=\frac{x-v_{0}}{v_{1}-v_{0}}$

Unlike the "artificial" target point method, this method selects the "real" target point at the convex envelope of the closest referenced positions. The "actual" target point selection method takes longer than the random "artificial" target point method. However, in our applications, the computation time in the interpolation process using the two methods is only marginally different.

Figure A1 illustrates the distribution of target points ("artificial " or "actual") selected by the adaptive tree model, where the X1 axis and X2 axis measure the geographic coordinates of an observation location. The fourteen red dots in the rectangular box are defined by the following vertices: [V0, V1, V2, and V3]. We split the box at the midpoint on the two sides of a box into smaller boxes such that each box contains an equal number of points. In a fixed bandwidth method (local bandwidth), the dotted line (edge) is split at the midpoint based on the vertex score. Artificial points represented by a red point circled by a purple-colored ring in the Figure are generated that are not constrained by observation points. The actual target points from the subset of the original data are the closest to the referenced target positions.

Figure A1 Distribution of artificial and actual target points (an example)

The actual target point method performs better in capturing the observed location distributions. The artificial target point method is too smooth by including low-density points, such as V6 in Figure A1. For the local bandwidth scheme with a fixed window size, a simple grid of artificial target points produces an excessively smooth surface when fitted to a region with a large bandwidth. In contrast, it creates an overly sensitive surface when fitted to a region with a small bandwidth.

***3. Interpolation***

By dividing China's geographical area into a square grid of 100km × 100km, we estimate the centroid value by interpolation using the modified Shephard algorithm (Franke & Nielson, 1980). This algorithm finds an approximate function of multidimensional Euclidean space from a limited set of data points (Thacker, 2010). The first step uses the Approximate Nearest Neighbor (ANN) method to find the "𝑘𝑛𝑢𝑚 nearest points," which are a set of "k" nearest neibors for a given location in a large dataset. The "𝑘𝑛𝑢𝑚 nearest points," Y_𝑖_, identified in a two-dimensional space with reference to a query point y_𝑖_, are organized into a binary k-dimensional tree (k-d tree) with a half-space splitting structure. The Euclidean distance between any two points is measured using the Minkowski metrics. The second step uses the following modified bivariate process to interpolate the previously defined target point to the reference locations (i.e., the grid centroid):

$$\frac{\sum_{i}^{knum} \omega_{i}p\text{ollu}tion_{i}}{\sum_{i}^{knum} \omega_{i}}$$

where ω_𝑖_ = ((𝑚𝑎𝑥𝑑-𝑑_𝑖_) 2) / (𝑚𝑎𝑥𝑑 * 𝑑_𝑖_); and 𝑚𝑎𝑥𝑑 = max (𝑑_1_, 𝑑_2_ ..., 𝑑_𝑘𝑛𝑢𝑚_), and the default setting is [𝑘𝑛𝑢𝑚 = 16). y_i_ is interpolated to a predefined centroid. Based on the Modified Shepard's Algorithm, we create a three-dimensional representation of pollutant emission values at the centroid of each grid.
