# Supplementary material for: Tracking industry pollution sources and health risks in China
Source: Sci Rep. 2023 Dec 14;13:22232. doi: 10.1038/s41598-023-49586-0 (PMC10721918; doi:10.1038/s41598-023-49586-0)
Supplement: Supplementary file 2 — Supplementary Information 2. [file 41598_2023_49586_MOESM2_ESM.doc]

Appendix 2-Industry code adjustment(Stata)

replace industrycode="1310" if year<=2002 & industrycode=="1311"

replace industrycode="1310" if year<=2002 & industrycode=="1312"

replace industrycode="1431" if year<=2002 & industrycode=="1313"

replace industrycode="1320" if year<=2002 & industrycode=="1314"

replace industrycode="1320" if year<=2002 & industrycode=="1315"

replace industrycode="1363" if year<=2002 & industrycode=="1317"

replace industrycode="1320" if year<=2002 & industrycode=="1319"

replace industrycode="1331" if year<=2002 & industrycode=="1321"

replace industrycode="1332" if year<=2002 & industrycode=="1322"

replace industrycode="1340" if year<=2002 & industrycode=="1331"

replace industrycode="1340" if year<=2002 & industrycode=="1332"

replace industrycode="1340" if year<=2002 & industrycode=="1334"

replace industrycode="1351" if year<=2002 & industrycode=="1341"

replace industrycode="1352" if year<=2002 & industrycode=="1342"

replace industrycode="1352" if year<=2002 & industrycode=="1343"

replace industrycode="1393" if year<=2002 & industrycode=="1344"

replace industrycode="1361" if year<=2002 & industrycode=="1351"

replace industrycode="1362" if year<=2002 & industrycode=="1352"

replace industrycode="1362" if year<=2002 & industrycode=="1353"

replace industrycode="1362" if year<=2002 & industrycode=="1354"

replace industrycode="1493" if year<=2002 & industrycode=="1360"

replace industrycode="1421" if year<=2002 & industrycode=="1411"

replace industrycode="1411" if year<=2002 & industrycode=="1412"

replace industrycode="1419" if year<=2002 & industrycode=="1413"

replace industrycode="1422" if year<=2002 & industrycode=="1415"

replace industrycode="1422" if year<=2002 & industrycode=="1419"

replace industrycode="1440" if year<=2002 & industrycode=="1420"

replace industrycode="1451" if year<=2002 & industrycode=="1431"

replace industrycode="1451" if year<=2002 & industrycode=="1432"

replace industrycode="1452" if year<=2002 & industrycode=="1433"

replace industrycode="1453" if year<=2002 & industrycode=="1434"

replace industrycode="1453" if year<=2002 & industrycode=="1435"

replace industrycode="1459" if year<=2002 & industrycode=="1439"

replace industrycode="1469" if year<=2002 & industrycode=="1441"

replace industrycode="1461" if year<=2002 & industrycode=="1442"

replace industrycode="1469" if year<=2002 & industrycode=="1443"

replace industrycode="1469" if year<=2002 & industrycode=="1444"

replace industrycode="1469" if year<=2002 & industrycode=="1445"

replace industrycode="1469" if year<=2002 & industrycode=="1449"

replace industrycode="1462" if year<=2002 & industrycode=="1451"

replace industrycode="1462" if year<=2002 & industrycode=="1452"

replace industrycode="1469" if year<=2002 & industrycode=="1453"

replace industrycode="1469" if year<=2002 & industrycode=="1454"

replace industrycode="1469" if year<=2002 & industrycode=="1459"

replace industrycode="1392" if year<=2002 & industrycode=="1491"

replace industrycode="1391" if year<=2002 & industrycode=="1492"

replace industrycode="1491" if year<=2002 & industrycode=="1493"

replace industrycode="1492" if year<=2002 & industrycode=="1495"

replace industrycode="1391" if year<=2002 & industrycode=="1497"

replace industrycode="1492" if year<=2002 & industrycode=="1498"

replace industrycode="1499" if year<=2002 & industrycode=="1499"

replace industrycode="1510" if year<=2002 & industrycode=="1511"

replace industrycode="1521" if year<=2002 & industrycode=="1512"

replace industrycode="1522" if year<=2002 & industrycode=="1513"

replace industrycode="1523" if year<=2002 & industrycode=="1514"

replace industrycode="1524" if year<=2002 & industrycode=="1515"

replace industrycode="1529" if year<=2002 & industrycode=="1516"

replace industrycode="1531" if year<=2002 & industrycode=="1521"

replace industrycode="1532" if year<=2002 & industrycode=="1522"

replace industrycode="1533" if year<=2002 & industrycode=="1523"

replace industrycode="1539" if year<=2002 & industrycode=="1529"

replace industrycode="1540" if year<=2002 & industrycode=="1550"

replace industrycode="1539" if year<=2002 & industrycode=="1590"

replace industrycode="1610" if year<=2002 & industrycode=="1610"

replace industrycode="1620" if year<=2002 & industrycode=="1620"

replace industrycode="1690" if year<=2002 & industrycode=="1690"

replace industrycode="1711" if year<=2002 & industrycode=="1711"

replace industrycode="1712" if year<=2002 & industrycode=="1712"

replace industrycode="1712" if year<=2002 & industrycode=="1713"

replace industrycode="1712" if year<=2002 & industrycode=="1714"

replace industrycode="1712" if year<=2002 & industrycode=="1719"

replace industrycode="1711" if year<=2002 & industrycode=="1721"

replace industrycode="1711" if year<=2002 & industrycode=="1722"

replace industrycode="1712" if year<=2002 & industrycode=="1723"

replace industrycode="1751" if year<=2002 & industrycode=="1724"

replace industrycode="1756" if year<=2002 & industrycode=="1725"

replace industrycode="1756" if year<=2002 & industrycode=="1726"

replace industrycode="1711" if year<=2002 & industrycode=="1729"

replace industrycode="1721" if year<=2002 & industrycode=="1741"

replace industrycode="1722" if year<=2002 & industrycode=="1742"

replace industrycode="1722" if year<=2002 & industrycode=="1743"

replace industrycode="1723" if year<=2002 & industrycode=="1744"

replace industrycode="1752" if year<=2002 & industrycode=="1745"

replace industrycode="1722" if year<=2002 & industrycode=="1749"

replace industrycode="1730" if year<=2002 & industrycode=="1761"

replace industrycode="1730" if year<=2002 & industrycode=="1762"

replace industrycode="1753" if year<=2002 & industrycode=="1763"

replace industrycode="1730" if year<=2002 & industrycode=="1769"

replace industrycode="1741" if year<=2002 & industrycode=="1771"

replace industrycode="1742" if year<=2002 & industrycode=="1772"

replace industrycode="1742" if year<=2002 & industrycode=="1773"

replace industrycode="1743" if year<=2002 & industrycode=="1774"

replace industrycode="1754" if year<=2002 & industrycode=="1775"

replace industrycode="1742" if year<=2002 & industrycode=="1779"

replace industrycode="1761" if year<=2002 & industrycode=="1781"

replace industrycode="1762" if year<=2002 & industrycode=="1782"

replace industrycode="1763" if year<=2002 & industrycode=="1783"

replace industrycode="1769" if year<=2002 & industrycode=="1789"

replace industrycode="1757" if year<=2002 & industrycode=="1790"

replace industrycode="1810" if year<=2002 & industrycode=="1810"

replace industrycode="1830" if year<=2002 & industrycode=="1820"

replace industrycode="1820" if year<=2002 & industrycode=="1830"

replace industrycode="1759" if year<=2002 & industrycode=="1890"

replace industrycode="1910" if year<=2002 & industrycode=="1911"

replace industrycode="1910" if year<=2002 & industrycode=="1912"

replace industrycode="1910" if year<=2002 & industrycode=="1919"

replace industrycode="1921" if year<=2002 & industrycode=="1921"

replace industrycode="1922" if year<=2002 & industrycode=="1923"

replace industrycode="1923" if year<=2002 & industrycode=="1924"

replace industrycode="1923" if year<=2002 & industrycode=="1925"

replace industrycode="1931" if year<=2002 & industrycode=="1931"

replace industrycode="1932" if year<=2002 & industrycode=="1932"

replace industrycode="1939" if year<=2002 & industrycode=="1939"

replace industrycode="1941" if year<=2002 & industrycode=="1951"

replace industrycode="1942" if year<=2002 & industrycode=="1952"

replace industrycode="2011" if year<=2002 & industrycode=="2011"

replace industrycode="2012" if year<=2002 & industrycode=="2012"

replace industrycode="2021" if year<=2002 & industrycode=="2021"

replace industrycode="2022" if year<=2002 & industrycode=="2022"

replace industrycode="2023" if year<=2002 & industrycode=="2023"

replace industrycode="2029" if year<=2002 & industrycode=="2029"

replace industrycode="2032" if year<=2002 & industrycode=="2033"

replace industrycode="2040" if year<=2002 & industrycode=="2040"

replace industrycode="2110" if year<=2002 & industrycode=="2110"

replace industrycode="2120" if year<=2002 & industrycode=="2120"

replace industrycode="2130" if year<=2002 & industrycode=="2130"

replace industrycode="2140" if year<=2002 & industrycode=="2140"

replace industrycode="2190" if year<=2002 & industrycode=="2190"

replace industrycode="2210" if year<=2002 & industrycode=="2210"

replace industrycode="2221" if year<=2002 & industrycode=="2221"

replace industrycode="2222" if year<=2002 & industrycode=="2223"

replace industrycode="2223" if year<=2002 & industrycode=="2224"

replace industrycode="2311" if year<=2002 & industrycode=="2311"

replace industrycode="2319" if year<=2002 & industrycode=="2312"

replace industrycode="2320" if year<=2002 & industrycode=="2319"

replace industrycode="2330" if year<=2002 & industrycode=="2320"

replace industrycode="2312" if year<=2002 & industrycode=="2413"

replace industrycode="2412" if year<=2002 & industrycode=="2415"

replace industrycode="2413" if year<=2002 & industrycode=="2417"

replace industrycode="2419" if year<=2002 & industrycode=="2419"

replace industrycode="2421" if year<=2002 & industrycode=="2421"

replace industrycode="2429" if year<=2002 & industrycode=="2429"

replace industrycode="2431" if year<=2002 & industrycode=="2431"

replace industrycode="2432" if year<=2002 & industrycode=="2433"

replace industrycode="2433" if year<=2002 & industrycode=="2435"

replace industrycode="2439" if year<=2002 & industrycode=="2439"

replace industrycode="2440" if year<=2002 & industrycode=="2440"

replace industrycode="2451" if year<=2002 & industrycode=="2450"

replace industrycode="2452" if year<=2002 & industrycode=="2490"

replace industrycode="2512" if year<=2002 & industrycode=="2510"

replace industrycode="2511" if year<=2002 & industrycode=="2520"

replace industrycode="2511" if year<=2002 & industrycode=="2530"

replace industrycode="2520" if year<=2002 & industrycode=="2570"

replace industrycode="2611" if year<=2002 & industrycode=="2611"

replace industrycode="2612" if year<=2002 & industrycode=="2613"

replace industrycode="2612" if year<=2002 & industrycode=="2615"

replace industrycode="2613" if year<=2002 & industrycode=="2617"

replace industrycode="2621" if year<=2002 & industrycode=="2621"

replace industrycode="2622" if year<=2002 & industrycode=="2622"

replace industrycode="2623" if year<=2002 & industrycode=="2623"

replace industrycode="2624" if year<=2002 & industrycode=="2624"

replace industrycode="2625" if year<=2002 & industrycode=="2625"

replace industrycode="2629" if year<=2002 & industrycode=="2629"

replace industrycode="2631" if year<=2002 & industrycode=="2631"

replace industrycode="2632" if year<=2002 & industrycode=="2633"

replace industrycode="2614" if year<=2002 & industrycode=="2651"

replace industrycode="2641" if year<=2002 & industrycode=="2652"

replace industrycode="2642" if year<=2002 & industrycode=="2653"

replace industrycode="2643" if year<=2002 & industrycode=="2654"

replace industrycode="2644" if year<=2002 & industrycode=="2655"

replace industrycode="2645" if year<=2002 & industrycode=="2659"

replace industrycode="2651" if year<=2002 & industrycode=="2661"

replace industrycode="2651" if year<=2002 & industrycode=="2662"

replace industrycode="2651" if year<=2002 & industrycode=="2663"

replace industrycode="2651" if year<=2002 & industrycode=="2664"

replace industrycode="2651" if year<=2002 & industrycode=="2665"

replace industrycode="2652" if year<=2002 & industrycode=="2666"

replace industrycode="2653" if year<=2002 & industrycode=="2667"

replace industrycode="2659" if year<=2002 & industrycode=="2669"

replace industrycode="2661" if year<=2002 & industrycode=="2671"

replace industrycode="2662" if year<=2002 & industrycode=="2672"

replace industrycode="2663" if year<=2002 & industrycode=="2673"

replace industrycode="2664" if year<=2002 & industrycode=="2674"

replace industrycode="2665" if year<=2002 & industrycode=="2675"

replace industrycode="2530" if year<=2002 & industrycode=="2676"

replace industrycode="1494" if year<=2002 & industrycode=="2677"

replace industrycode="2671" if year<=2002 & industrycode=="2681"

replace industrycode="2662" if year<=2002 & industrycode=="2682"

replace industrycode="2662" if year<=2002 & industrycode=="2683"

replace industrycode="2674" if year<=2002 & industrycode=="2684"

replace industrycode="2672" if year<=2002 & industrycode=="2685"

replace industrycode="2673" if year<=2002 & industrycode=="2686"

replace industrycode="2679" if year<=2002 & industrycode=="2687"

replace industrycode="2667" if year<=2002 & industrycode=="2688"

replace industrycode="2679" if year<=2002 & industrycode=="2689"

replace industrycode="2710" if year<=2002 & industrycode=="2710"

replace industrycode="2720" if year<=2002 & industrycode=="2720"

replace industrycode="2750" if year<=2002 & industrycode=="2740"

replace industrycode="2760" if year<=2002 & industrycode=="2750"

replace industrycode="2811" if year<=2002 & industrycode=="2811"

replace industrycode="2812" if year<=2002 & industrycode=="2812"

replace industrycode="2812" if year<=2002 & industrycode=="2819"

replace industrycode="2821" if year<=2002 & industrycode=="2821"

replace industrycode="2822" if year<=2002 & industrycode=="2822"

replace industrycode="2823" if year<=2002 & industrycode=="2823"

replace industrycode="2824" if year<=2002 & industrycode=="2824"

replace industrycode="2829" if year<=2002 & industrycode=="2829"

replace industrycode="1755" if year<=2002 & industrycode=="2851"

replace industrycode="1755" if year<=2002 & industrycode=="2852"

replace industrycode="1755" if year<=2002 & industrycode=="2853"

replace industrycode="1755" if year<=2002 & industrycode=="2854"

replace industrycode="2429" if year<=2002 & industrycode=="2859"

replace industrycode="2911" if year<=2002 & industrycode=="2910"

replace industrycode="2912" if year<=2002 & industrycode=="2920"

replace industrycode="2920" if year<=2002 & industrycode=="2930"

replace industrycode="2930" if year<=2002 & industrycode=="2940"

replace industrycode="2940" if year<=2002 & industrycode=="2950"

replace industrycode="2960" if year<=2002 & industrycode=="2960"

replace industrycode="2950" if year<=2002 & industrycode=="2970"

replace industrycode="2913" if year<=2002 & industrycode=="2981"

replace industrycode="2990" if year<=2002 & industrycode=="2989"

replace industrycode="2990" if year<=2002 & industrycode=="2990"

replace industrycode="3010" if year<=2002 & industrycode=="3010"

replace industrycode="3020" if year<=2002 & industrycode=="3020"

replace industrycode="3030" if year<=2002 & industrycode=="3030"

replace industrycode="3060" if year<=2002 & industrycode=="3050"

replace industrycode="3081" if year<=2002 & industrycode=="3060"

replace industrycode="3082" if year<=2002 & industrycode=="3070"

replace industrycode="3070" if year<=2002 & industrycode=="3080"

replace industrycode="3090" if year<=2002 & industrycode=="3090"

replace industrycode="3111" if year<=2002 & industrycode=="3110"

replace industrycode="3121" if year<=2002 & industrycode=="3121"

replace industrycode="3122" if year<=2002 & industrycode=="3123"

replace industrycode="3123" if year<=2002 & industrycode=="3124"

replace industrycode="3129" if year<=2002 & industrycode=="3129"

replace industrycode="3131" if year<=2002 & industrycode=="3131"

replace industrycode="3112" if year<=2002 & industrycode=="3132"

replace industrycode="3133" if year<=2002 & industrycode=="3133"

replace industrycode="3124" if year<=2002 & industrycode=="3134"

replace industrycode="3134" if year<=2002 & industrycode=="3135"

replace industrycode="3135" if year<=2002 & industrycode=="3136"

replace industrycode="3139" if year<=2002 & industrycode=="3139"

replace industrycode="3141" if year<=2002 & industrycode=="3141"

replace industrycode="3142" if year<=2002 & industrycode=="3142"

replace industrycode="3143" if year<=2002 & industrycode=="3143"

replace industrycode="3144" if year<=2002 & industrycode=="3145"

replace industrycode="3145" if year<=2002 & industrycode=="3147"

replace industrycode="3146" if year<=2002 & industrycode=="3148"

replace industrycode="3149" if year<=2002 & industrycode=="3149"

replace industrycode="3152" if year<=2002 & industrycode=="3153"

replace industrycode="3153" if year<=2002 & industrycode=="3155"

replace industrycode="3159" if year<=2002 & industrycode=="3159"

replace industrycode="3161" if year<=2002 & industrycode=="3161"

replace industrycode="3162" if year<=2002 & industrycode=="3163"

replace industrycode="3169" if year<=2002 & industrycode=="3169"

replace industrycode="3191" if year<=2002 & industrycode=="3171"

replace industrycode="3191" if year<=2002 & industrycode=="3172"

replace industrycode="3199" if year<=2002 & industrycode=="3179"

replace industrycode="3147" if year<=2002 & industrycode=="3181"

replace industrycode="3148" if year<=2002 & industrycode=="3182"

replace industrycode="3149" if year<=2002 & industrycode=="3189"

replace industrycode="3199" if year<=2002 & industrycode=="3190"

replace industrycode="3210" if year<=2002 & industrycode=="3210"

replace industrycode="3220" if year<=2002 & industrycode=="3220"

replace industrycode="3230" if year<=2002 & industrycode=="3240"

replace industrycode="3240" if year<=2002 & industrycode=="3260"

replace industrycode="3311" if year<=2002 & industrycode=="3311"

replace industrycode="3312" if year<=2002 & industrycode=="3312"

replace industrycode="3313" if year<=2002 & industrycode=="3314"

replace industrycode="3314" if year<=2002 & industrycode=="3316"

replace industrycode="3315" if year<=2002 & industrycode=="3317"

replace industrycode="3319" if year<=2002 & industrycode=="3318"

replace industrycode="3319" if year<=2002 & industrycode=="3319"

replace industrycode="3316" if year<=2002 & industrycode=="3321"

replace industrycode="3317" if year<=2002 & industrycode=="3322"

replace industrycode="3319" if year<=2002 & industrycode=="3323"

replace industrycode="3319" if year<=2002 & industrycode=="3329"

replace industrycode="3321" if year<=2002 & industrycode=="3331"

replace industrycode="3322" if year<=2002 & industrycode=="3332"

replace industrycode="3329" if year<=2002 & industrycode=="3339"

replace industrycode="3331" if year<=2002 & industrycode=="3341"

replace industrycode="3340" if year<=2002 & industrycode=="3360"

replace industrycode="3351" if year<=2002 & industrycode=="3381"

replace industrycode="3351" if year<=2002 & industrycode=="3383"

replace industrycode="3352" if year<=2002 & industrycode=="3385"

replace industrycode="3353" if year<=2002 & industrycode=="3387"

replace industrycode="3411" if year<=2002 & industrycode=="3410"

replace industrycode="3210" if year<=2002 & industrycode=="3420"

replace industrycode="3421" if year<=2002 & industrycode=="3431"

replace industrycode="3625" if year<=2002 & industrycode=="3434"

replace industrycode="3422" if year<=2002 & industrycode=="3435"

replace industrycode="3429" if year<=2002 & industrycode=="3439"

replace industrycode="3431" if year<=2002 & industrycode=="3441"

replace industrycode="3440" if year<=2002 & industrycode=="3450"

replace industrycode="3451" if year<=2002 & industrycode=="3461"

replace industrycode="3452" if year<=2002 & industrycode=="3463"

replace industrycode="3412" if year<=2002 & industrycode=="3465"

replace industrycode="3459" if year<=2002 & industrycode=="3469"

replace industrycode="3460" if year<=2002 & industrycode=="3470"

replace industrycode="3482" if year<=2002 & industrycode=="3482"

replace industrycode="3481" if year<=2002 & industrycode=="3483"

replace industrycode="3424" if year<=2002 & industrycode=="3484"

replace industrycode="3451" if year<=2002 & industrycode=="3485"

replace industrycode="3969" if year<=2002 & industrycode=="3486"

replace industrycode="3961" if year<=2002 & industrycode=="3487"

replace industrycode="3424" if year<=2002 & industrycode=="3488"

replace industrycode="3489" if year<=2002 & industrycode=="3489"

replace industrycode="3423" if year<=2002 & industrycode=="3491"

replace industrycode="3499" if year<=2002 & industrycode=="3495"

replace industrycode="3511" if year<=2002 & industrycode=="3511"

replace industrycode="3512" if year<=2002 & industrycode=="3512"

replace industrycode="3513" if year<=2002 & industrycode=="3513"

replace industrycode="3514" if year<=2002 & industrycode=="3514"

replace industrycode="3512" if year<=2002 & industrycode=="3515"

replace industrycode="3519" if year<=2002 & industrycode=="3519"

replace industrycode="3521" if year<=2002 & industrycode=="3521"

replace industrycode="3522" if year<=2002 & industrycode=="3523"

replace industrycode="3523" if year<=2002 & industrycode=="3525"

replace industrycode="3525" if year<=2002 & industrycode=="3526"

replace industrycode="3529" if year<=2002 & industrycode=="3529"

replace industrycode="3530" if year<=2002 & industrycode=="3531"

replace industrycode="3712" if year<=2002 & industrycode=="3532"

replace industrycode="3541" if year<=2002 & industrycode=="3533"

replace industrycode="3571" if year<=2002 & industrycode=="3534"

replace industrycode="3573" if year<=2002 & industrycode=="3536"

replace industrycode="3574" if year<=2002 & industrycode=="3537"

replace industrycode="3574" if year<=2002 & industrycode=="3538"

replace industrycode="3575" if year<=2002 & industrycode=="3539"

replace industrycode="3551" if year<=2002 & industrycode=="3541"

replace industrycode="3543" if year<=2002 & industrycode=="3542"

replace industrycode="3544" if year<=2002 & industrycode=="3561"

replace industrycode="3544" if year<=2002 & industrycode=="3562"

replace industrycode="3581" if year<=2002 & industrycode=="3563"

replace industrycode="3592" if year<=2002 & industrycode=="3564"

replace industrycode="3582" if year<=2002 & industrycode=="3565"

replace industrycode="3582" if year<=2002 & industrycode=="3566"

replace industrycode="3552" if year<=2002 & industrycode=="3567"

replace industrycode="3552" if year<=2002 & industrycode=="3568"

replace industrycode="3589" if year<=2002 & industrycode=="3569"

replace industrycode="3591" if year<=2002 & industrycode=="3571"

replace industrycode="3592" if year<=2002 & industrycode=="3572"

replace industrycode="3583" if year<=2002 & industrycode=="3580"

replace industrycode="3579" if year<=2002 & industrycode=="3590"

replace industrycode="3611" if year<=2002 & industrycode=="3611"

replace industrycode="3615" if year<=2002 & industrycode=="3613"

replace industrycode="3661" if year<=2002 & industrycode=="3615"

replace industrycode="3662" if year<=2002 & industrycode=="3617"

replace industrycode="3669" if year<=2002 & industrycode=="3619"

replace industrycode="3612" if year<=2002 & industrycode=="3621"

replace industrycode="3621" if year<=2002 & industrycode=="3622"

replace industrycode="3621" if year<=2002 & industrycode=="3623"

replace industrycode="3622" if year<=2002 & industrycode=="3624"

replace industrycode="3623" if year<=2002 & industrycode=="3625"

replace industrycode="3624" if year<=2002 & industrycode=="3626"

replace industrycode="3642" if year<=2002 & industrycode=="3627"

replace industrycode="3644" if year<=2002 & industrycode=="3628"

replace industrycode="3631" if year<=2002 & industrycode=="3631"

replace industrycode="3632" if year<=2002 & industrycode=="3632"

replace industrycode="3633" if year<=2002 & industrycode=="3633"

replace industrycode="3576" if year<=2002 & industrycode=="3634"

replace industrycode="3645" if year<=2002 & industrycode=="3636"

replace industrycode="3646" if year<=2002 & industrycode=="3637"

replace industrycode="3641" if year<=2002 & industrycode=="3638"

replace industrycode="3643" if year<=2002 & industrycode=="3639"

replace industrycode="3671" if year<=2002 & industrycode=="3641"

replace industrycode="3672" if year<=2002 & industrycode=="3642"

replace industrycode="3673" if year<=2002 & industrycode=="3643"

replace industrycode="3674" if year<=2002 & industrycode=="3644"

replace industrycode="3675" if year<=2002 & industrycode=="3645"

replace industrycode="3697" if year<=2002 & industrycode=="3646"

replace industrycode="3676" if year<=2002 & industrycode=="3647"

replace industrycode="3679" if year<=2002 & industrycode=="3649"

replace industrycode="3684" if year<=2002 & industrycode=="3651"

replace industrycode="3684" if year<=2002 & industrycode=="3653"

replace industrycode="2770" if year<=2002 & industrycode=="3654"

replace industrycode="3686" if year<=2002 & industrycode=="3655"

replace industrycode="3613" if year<=2002 & industrycode=="3671"

replace industrycode="3692" if year<=2002 & industrycode=="3672"

replace industrycode="3684" if year<=2002 & industrycode=="3673"

replace industrycode="3653" if year<=2002 & industrycode=="3674"

replace industrycode="3694" if year<=2002 & industrycode=="3675"

replace industrycode="3693" if year<=2002 & industrycode=="3676"

replace industrycode="3691" if year<=2002 & industrycode=="3677"

replace industrycode="3583" if year<=2002 & industrycode=="3681"

replace industrycode="3679" if year<=2002 & industrycode=="3683"

replace industrycode="3689" if year<=2002 & industrycode=="3685"

replace industrycode="3583" if year<=2002 & industrycode=="3689"

replace industrycode="3711" if year<=2002 & industrycode=="3711"

replace industrycode="3711" if year<=2002 & industrycode=="3712"

replace industrycode="3711" if year<=2002 & industrycode=="3713"

replace industrycode="3713" if year<=2002 & industrycode=="3714"

replace industrycode="3714" if year<=2002 & industrycode=="3715"

replace industrycode="3714" if year<=2002 & industrycode=="3716"

replace industrycode="3714" if year<=2002 & industrycode=="3717"

replace industrycode="3719" if year<=2002 & industrycode=="3719"

replace industrycode="3721" if year<=2002 & industrycode=="3721"

replace industrycode="3721" if year<=2002 & industrycode=="3722"

replace industrycode="3721" if year<=2002 & industrycode=="3723"

replace industrycode="3721" if year<=2002 & industrycode=="3724"

replace industrycode="3722" if year<=2002 & industrycode=="3725"

replace industrycode="3724" if year<=2002 & industrycode=="3726"

replace industrycode="3725" if year<=2002 & industrycode=="3727"

replace industrycode="3731" if year<=2002 & industrycode=="3731"

replace industrycode="3732" if year<=2002 & industrycode=="3732"

replace industrycode="3723" if year<=2002 & industrycode=="3750"

replace industrycode="3751" if year<=2002 & industrycode=="3761"

replace industrycode="3751" if year<=2002 & industrycode=="3762"

replace industrycode="3751" if year<=2002 & industrycode=="3763"

replace industrycode="3754" if year<=2002 & industrycode=="3764"

replace industrycode="3761" if year<=2002 & industrycode=="3771"

replace industrycode="3719" if year<=2002 & industrycode=="3781"

replace industrycode="3726" if year<=2002 & industrycode=="3782"

replace industrycode="3799" if year<=2002 & industrycode=="3783"

replace industrycode="3723" if year<=2002 & industrycode=="3784"

replace industrycode="3755" if year<=2002 & industrycode=="3785"

replace industrycode="3761" if year<=2002 & industrycode=="3786"

replace industrycode="3799" if year<=2002 & industrycode=="3789"

replace industrycode="3759" if year<=2002 & industrycode=="3791"

replace industrycode="3791" if year<=2002 & industrycode=="3792"

replace industrycode="3792" if year<=2002 & industrycode=="3793"

replace industrycode="3663" if year<=2002 & industrycode=="3900"

replace industrycode="3911" if year<=2002 & industrycode=="4011"

replace industrycode="3912" if year<=2002 & industrycode=="4012"

replace industrycode="3919" if year<=2002 & industrycode=="4013"

replace industrycode="3921" if year<=2002 & industrycode=="4021"

replace industrycode="3921" if year<=2002 & industrycode=="4022"

replace industrycode="3922" if year<=2002 & industrycode=="4023"

replace industrycode="3923" if year<=2002 & industrycode=="4024"

replace industrycode="3924" if year<=2002 & industrycode=="4027"

replace industrycode="3929" if year<=2002 & industrycode=="4029"

replace industrycode="3933" if year<=2002 & industrycode=="4043"

replace industrycode="3940" if year<=2002 & industrycode=="4045"

replace industrycode="3940" if year<=2002 & industrycode=="4046"

replace industrycode="3939" if year<=2002 & industrycode=="4049"

replace industrycode="3955" if year<=2002 & industrycode=="4061"

replace industrycode="3955" if year<=2002 & industrycode=="4062"

replace industrycode="3951" if year<=2002 & industrycode=="4063"

replace industrycode="3953" if year<=2002 & industrycode=="4064"

replace industrycode="3952" if year<=2002 & industrycode=="4065"

replace industrycode="3954" if year<=2002 & industrycode=="4066"

replace industrycode="3971" if year<=2002 & industrycode=="4071"

replace industrycode="3979" if year<=2002 & industrycode=="4072"

replace industrycode="3972" if year<=2002 & industrycode=="4073"

replace industrycode="3979" if year<=2002 & industrycode=="4074"

replace industrycode="3979" if year<=2002 & industrycode=="4079"

replace industrycode="3999" if year<=2002 & industrycode=="4080"

replace industrycode="3524" if year<=2002 & industrycode=="4091"

replace industrycode="3560" if year<=2002 & industrycode=="4092"

replace industrycode="3991" if year<=2002 & industrycode=="4099"

replace industrycode="4011" if year<=2002 & industrycode=="4111"

replace industrycode="4012" if year<=2002 & industrycode=="4112"

replace industrycode="4019" if year<=2002 & industrycode=="4119"

replace industrycode="4020" if year<=2002 & industrycode=="4121"

replace industrycode="4020" if year<=2002 & industrycode=="4122"

replace industrycode="4041" if year<=2002 & industrycode=="4141"

replace industrycode="4051" if year<=2002 & industrycode=="4151"

replace industrycode="4053" if year<=2002 & industrycode=="4155"

replace industrycode="4071" if year<=2002 & industrycode=="4171"

replace industrycode="4072" if year<=2002 & industrycode=="4172"

replace industrycode="4155" if year<=2002 & industrycode=="4173"

replace industrycode="4019" if year<=2002 & industrycode=="4181"

replace industrycode="4039" if year<=2002 & industrycode=="4182"

replace industrycode="4041" if year<=2002 & industrycode=="4183"

replace industrycode="4090" if year<=2002 & industrycode=="4189"

replace industrycode="4090" if year<=2002 & industrycode=="4190"

replace industrycode="4111" if year<=2002 & industrycode=="4211"

replace industrycode="4112" if year<=2002 & industrycode=="4212"

replace industrycode="4141" if year<=2002 & industrycode=="4213"

replace industrycode="4130" if year<=2002 & industrycode=="4214"

replace industrycode="4114" if year<=2002 & industrycode=="4215"

replace industrycode="4115" if year<=2002 & industrycode=="4216"

replace industrycode="4114" if year<=2002 & industrycode=="4217"

replace industrycode="4119" if year<=2002 & industrycode=="4218"

replace industrycode="4119" if year<=2002 & industrycode=="4219"

replace industrycode="4121" if year<=2002 & industrycode=="4221"

replace industrycode="4122" if year<=2002 & industrycode=="4222"

replace industrycode="4123" if year<=2002 & industrycode=="4223"

replace industrycode="4124" if year<=2002 & industrycode=="4224"

replace industrycode="4125" if year<=2002 & industrycode=="4225"

replace industrycode="4123" if year<=2002 & industrycode=="4226"

replace industrycode="4126" if year<=2002 & industrycode=="4227"

replace industrycode="4127" if year<=2002 & industrycode=="4228"

replace industrycode="4129" if year<=2002 & industrycode=="4229"

replace industrycode="4128" if year<=2002 & industrycode=="4230"

replace industrycode="4113" if year<=2002 & industrycode=="4241"

replace industrycode="4113" if year<=2002 & industrycode=="4242"

replace industrycode="3577" if year<=2002 & industrycode=="4243"

replace industrycode="4151" if year<=2002 & industrycode=="4251"

replace industrycode="4152" if year<=2002 & industrycode=="4252"

replace industrycode="4153" if year<=2002 & industrycode=="4254"

replace industrycode="4154" if year<=2002 & industrycode=="4256"

replace industrycode="4154" if year<=2002 & industrycode=="4257"

replace industrycode="4159" if year<=2002 & industrycode=="4259"

replace industrycode="4130" if year<=2002 & industrycode=="4260"

replace industrycode="4190" if year<=2002 & industrycode=="4280"

replace industrycode="4190" if year<=2002 & industrycode=="4290"

replace industrycode="4211" if year<=2002 & industrycode=="4311"

replace industrycode="4212" if year<=2002 & industrycode=="4312"

replace industrycode="4213" if year<=2002 & industrycode=="4313"

replace industrycode="4214" if year<=2002 & industrycode=="4314"

replace industrycode="4215" if year<=2002 & industrycode=="4315"

replace industrycode="4216" if year<=2002 & industrycode=="4316"

replace industrycode="4217" if year<=2002 & industrycode=="4317"

replace industrycode="4218" if year<=2002 & industrycode=="4318"

replace industrycode="4219" if year<=2002 & industrycode=="4319"

replace industrycode="4221" if year<=2002 & industrycode=="4351"

replace industrycode="4142" if year<=2002 & industrycode=="4353"

replace industrycode="4229" if year<=2002 & industrycode=="4355"

replace industrycode="4222" if year<=2002 & industrycode=="4357"

replace industrycode="1359" if year<=2002 & industrycode=="1359"

replace industrycode="1390" if year<=2002 & industrycode=="1390"

replace industrycode="1414" if year<=2002 & industrycode=="1414"

replace industrycode="1524" if year<=2002 & industrycode=="1524"

replace industrycode="1929" if year<=2002 & industrycode=="1929"

replace industrycode="2031" if year<=2002 & industrycode=="2031"

replace industrycode="2230" if year<=2002 & industrycode=="2230"

replace industrycode="2411" if year<=2002 & industrycode=="2411"

replace industrycode="2423" if year<=2002 & industrycode=="2423"

replace industrycode="2619" if year<=2002 & industrycode=="2619"

replace industrycode="2730" if year<=2002 & industrycode=="2730"

replace industrycode="3040" if year<=2002 & industrycode=="3040"

replace industrycode="3151" if year<=2002 & industrycode=="3151"

replace industrycode="3349" if year<=2002 & industrycode=="3349"

replace industrycode="3442" if year<=2002 & industrycode=="3442"

replace industrycode="3481" if year<=2002 & industrycode=="3481"

replace industrycode="3499" if year<=2002 & industrycode=="3499"

replace industrycode="3535" if year<=2002 & industrycode=="3535"

replace industrycode="3629" if year<=2002 & industrycode=="3629"

replace industrycode="3635" if year<=2002 & industrycode=="3635"

replace industrycode="3652" if year<=2002 & industrycode=="3652"

replace industrycode="3678" if year<=2002 & industrycode=="3678"

replace industrycode="3679" if year<=2002 & industrycode=="3679"

replace industrycode="3740" if year<=2002 & industrycode=="3740"

replace industrycode="3779" if year<=2002 & industrycode=="3779"

replace industrycode="4041" if year<=2002 & industrycode=="4041"

replace industrycode="4069" if year<=2002 & industrycode=="4069"

replace industrycode="4113" if year<=2002 & industrycode=="4113"

replace industrycode="4130" if year<=2002 & industrycode=="4130"

replace industrycode="4143" if year<=2002 & industrycode=="4143"

replace industrycode="4153" if year<=2002 & industrycode=="4153"

replace industrycode="4160" if year<=2002 & industrycode=="4160"
