# Supplementary material for: Tracking industry pollution sources and health risks in China
Source: Sci Rep. 2023 Dec 14;13:22232. doi: 10.1038/s41598-023-49586-0 (PMC10721918; doi:10.1038/s41598-023-49586-0)
Supplement: Supplementary file 3 — Supplementary Information 3. [file 41598_2023_49586_MOESM3_ESM.doc]

Appendix-Region code adjustment(Stata)

replace regioncode="110101" if regioncode=="110101" & year==1998

replace regioncode="110102" if regioncode=="110102" & year==1998

replace regioncode="110103" if regioncode=="110103" & year==1998

replace regioncode="110104" if regioncode=="110104" & year==1998

replace regioncode="110105" if regioncode=="110105" & year==1998

replace regioncode="110106" if regioncode=="110106" & year==1998

replace regioncode="110107" if regioncode=="110107" & year==1998

replace regioncode="110108" if regioncode=="110108" & year==1998

replace regioncode="110109" if regioncode=="110109" & year==1998

replace regioncode="110111" if regioncode=="110111" & year==1998

replace regioncode="110112" if regioncode=="110112" & year==1998

replace regioncode="110113" if regioncode=="110113" & year==1998

replace regioncode="110114" if regioncode=="110221" & year==1998

replace regioncode="110115" if regioncode=="110224" & year==1998

replace regioncode="110117" if regioncode=="110226" & year==1998

replace regioncode="110116" if regioncode=="110227" & year==1998

replace regioncode="110228" if regioncode=="110228" & year==1998

replace regioncode="110229" if regioncode=="110229" & year==1998

replace regioncode="120101" if regioncode=="120101" & year==1998

replace regioncode="120102" if regioncode=="120102" & year==1998

replace regioncode="120103" if regioncode=="120103" & year==1998

replace regioncode="120104" if regioncode=="120104" & year==1998

replace regioncode="120105" if regioncode=="120105" & year==1998

replace regioncode="120106" if regioncode=="120106" & year==1998

replace regioncode="120107" if regioncode=="120107" & year==1998

replace regioncode="120108" if regioncode=="120108" & year==1998

replace regioncode="120109" if regioncode=="120109" & year==1998

replace regioncode="120110" if regioncode=="120110" & year==1998

replace regioncode="120111" if regioncode=="120111" & year==1998

replace regioncode="120112" if regioncode=="120112" & year==1998

replace regioncode="120113" if regioncode=="120113" & year==1998

replace regioncode="120114" if regioncode=="120222" & year==1998

replace regioncode="120115" if regioncode=="120224" & year==1998

replace regioncode="120221" if regioncode=="120221" & year==1998

replace regioncode="120223" if regioncode=="120223" & year==1998

replace regioncode="120225" if regioncode=="120225" & year==1998

replace regioncode="130102" if regioncode=="130102" & year==1998

replace regioncode="130103" if regioncode=="130103" & year==1998

replace regioncode="130104" if regioncode=="130104" & year==1998

replace regioncode="130105" if regioncode=="130105" & year==1998

replace regioncode="130107" if regioncode=="130107" & year==1998

replace regioncode="130108" if regioncode=="130106" & year==1998

replace regioncode="130121" if regioncode=="130121" & year==1998

replace regioncode="130123" if regioncode=="130123" & year==1998

replace regioncode="130124" if regioncode=="130124" & year==1998

replace regioncode="130125" if regioncode=="130125" & year==1998

replace regioncode="130126" if regioncode=="130126" & year==1998

replace regioncode="130127" if regioncode=="130127" & year==1998

replace regioncode="130128" if regioncode=="130128" & year==1998

replace regioncode="130129" if regioncode=="130129" & year==1998

replace regioncode="130130" if regioncode=="130130" & year==1998

replace regioncode="130131" if regioncode=="130131" & year==1998

replace regioncode="130132" if regioncode=="130132" & year==1998

replace regioncode="130133" if regioncode=="130133" & year==1998

replace regioncode="130181" if regioncode=="130181" & year==1998

replace regioncode="130182" if regioncode=="130182" & year==1998

replace regioncode="130183" if regioncode=="130183" & year==1998

replace regioncode="130184" if regioncode=="130184" & year==1998

replace regioncode="130185" if regioncode=="130185" & year==1998

replace regioncode="130202" if regioncode=="130202" & year==1998

replace regioncode="130203" if regioncode=="130203" & year==1998

replace regioncode="130204" if regioncode=="130204" & year==1998

replace regioncode="130205" if regioncode=="130205" & year==1998

replace regioncode="130208" if regioncode=="130221" & year==1998

replace regioncode="130223" if regioncode=="130223" & year==1998

replace regioncode="130224" if regioncode=="130224" & year==1998

replace regioncode="130225" if regioncode=="130225" & year==1998

replace regioncode="130227" if regioncode=="130227" & year==1998

replace regioncode="130229" if regioncode=="130229" & year==1998

replace regioncode="130230" if regioncode=="130230" & year==1998

replace regioncode="130281" if regioncode=="130281" & year==1998

replace regioncode="130207" if regioncode=="130282" & year==1998

replace regioncode="130283" if regioncode=="130283" & year==1998

replace regioncode="130302" if regioncode=="130302" & year==1998

replace regioncode="130303" if regioncode=="130303" & year==1998

replace regioncode="130304" if regioncode=="130304" & year==1998

replace regioncode="130321" if regioncode=="130321" & year==1998

replace regioncode="130322" if regioncode=="130322" & year==1998

replace regioncode="130323" if regioncode=="130323" & year==1998

replace regioncode="130324" if regioncode=="130324" & year==1998

replace regioncode="130402" if regioncode=="130402" & year==1998

replace regioncode="130403" if regioncode=="130403" & year==1998

replace regioncode="130404" if regioncode=="130404" & year==1998

replace regioncode="130406" if regioncode=="130406" & year==1998

replace regioncode="130421" if regioncode=="130421" & year==1998

replace regioncode="130423" if regioncode=="130423" & year==1998

replace regioncode="130424" if regioncode=="130424" & year==1998

replace regioncode="130425" if regioncode=="130425" & year==1998

replace regioncode="130426" if regioncode=="130426" & year==1998

replace regioncode="130427" if regioncode=="130427" & year==1998

replace regioncode="130428" if regioncode=="130428" & year==1998

replace regioncode="130429" if regioncode=="130429" & year==1998

replace regioncode="130430" if regioncode=="130430" & year==1998

replace regioncode="130431" if regioncode=="130431" & year==1998

replace regioncode="130432" if regioncode=="130432" & year==1998

replace regioncode="130433" if regioncode=="130433" & year==1998

replace regioncode="130434" if regioncode=="130434" & year==1998

replace regioncode="130435" if regioncode=="130435" & year==1998

replace regioncode="130481" if regioncode=="130481" & year==1998

replace regioncode="130502" if regioncode=="130502" & year==1998

replace regioncode="130503" if regioncode=="130503" & year==1998

replace regioncode="130521" if regioncode=="130521" & year==1998

replace regioncode="130522" if regioncode=="130522" & year==1998

replace regioncode="130523" if regioncode=="130523" & year==1998

replace regioncode="130524" if regioncode=="130524" & year==1998

replace regioncode="130525" if regioncode=="130525" & year==1998

replace regioncode="130526" if regioncode=="130526" & year==1998

replace regioncode="130527" if regioncode=="130527" & year==1998

replace regioncode="130528" if regioncode=="130528" & year==1998

replace regioncode="130529" if regioncode=="130529" & year==1998

replace regioncode="130530" if regioncode=="130530" & year==1998

replace regioncode="130531" if regioncode=="130531" & year==1998

replace regioncode="130532" if regioncode=="130532" & year==1998

replace regioncode="130533" if regioncode=="130533" & year==1998

replace regioncode="130534" if regioncode=="130534" & year==1998

replace regioncode="130535" if regioncode=="130535" & year==1998

replace regioncode="130581" if regioncode=="130581" & year==1998

replace regioncode="130582" if regioncode=="130582" & year==1998

replace regioncode="130602" if regioncode=="130602" & year==1998

replace regioncode="130603" if regioncode=="130603" & year==1998

replace regioncode="130604" if regioncode=="130604" & year==1998

replace regioncode="130621" if regioncode=="130621" & year==1998

replace regioncode="130622" if regioncode=="130622" & year==1998

replace regioncode="130623" if regioncode=="130623" & year==1998

replace regioncode="130624" if regioncode=="130624" & year==1998

replace regioncode="130625" if regioncode=="130625" & year==1998

replace regioncode="130626" if regioncode=="130626" & year==1998

replace regioncode="130627" if regioncode=="130627" & year==1998

replace regioncode="130628" if regioncode=="130628" & year==1998

replace regioncode="130629" if regioncode=="130629" & year==1998

replace regioncode="130630" if regioncode=="130630" & year==1998

replace regioncode="130631" if regioncode=="130631" & year==1998

replace regioncode="130632" if regioncode=="130632" & year==1998

replace regioncode="130633" if regioncode=="130633" & year==1998

replace regioncode="130634" if regioncode=="130634" & year==1998

replace regioncode="130635" if regioncode=="130635" & year==1998

replace regioncode="130636" if regioncode=="130636" & year==1998

replace regioncode="130637" if regioncode=="130637" & year==1998

replace regioncode="130638" if regioncode=="130638" & year==1998

replace regioncode="130681" if regioncode=="130681" & year==1998

replace regioncode="130682" if regioncode=="130682" & year==1998

replace regioncode="130683" if regioncode=="130683" & year==1998

replace regioncode="130684" if regioncode=="130684" & year==1998

replace regioncode="130702" if regioncode=="130702" & year==1998

replace regioncode="130703" if regioncode=="130703" & year==1998

replace regioncode="130705" if regioncode=="130705" & year==1998

replace regioncode="130706" if regioncode=="130706" & year==1998

replace regioncode="130721" if regioncode=="130721" & year==1998

replace regioncode="130722" if regioncode=="130722" & year==1998

replace regioncode="130723" if regioncode=="130723" & year==1998

replace regioncode="130724" if regioncode=="130724" & year==1998

replace regioncode="130725" if regioncode=="130725" & year==1998

replace regioncode="130726" if regioncode=="130726" & year==1998

replace regioncode="130727" if regioncode=="130727" & year==1998

replace regioncode="130728" if regioncode=="130728" & year==1998

replace regioncode="130729" if regioncode=="130729" & year==1998

replace regioncode="130730" if regioncode=="130730" & year==1998

replace regioncode="130731" if regioncode=="130731" & year==1998

replace regioncode="130732" if regioncode=="130732" & year==1998

replace regioncode="130733" if regioncode=="130733" & year==1998

replace regioncode="130802" if regioncode=="130802" & year==1998

replace regioncode="130803" if regioncode=="130803" & year==1998

replace regioncode="130804" if regioncode=="130804" & year==1998

replace regioncode="130821" if regioncode=="130821" & year==1998

replace regioncode="130822" if regioncode=="130822" & year==1998

replace regioncode="130823" if regioncode=="130823" & year==1998

replace regioncode="130824" if regioncode=="130824" & year==1998

replace regioncode="130825" if regioncode=="130825" & year==1998

replace regioncode="130826" if regioncode=="130826" & year==1998

replace regioncode="130827" if regioncode=="130827" & year==1998

replace regioncode="130828" if regioncode=="130828" & year==1998

replace regioncode="130902" if regioncode=="130902" & year==1998

replace regioncode="130903" if regioncode=="130903" & year==1998

replace regioncode="130921" if regioncode=="130921" & year==1998

replace regioncode="130922" if regioncode=="130922" & year==1998

replace regioncode="130923" if regioncode=="130923" & year==1998

replace regioncode="130924" if regioncode=="130924" & year==1998

replace regioncode="130925" if regioncode=="130925" & year==1998

replace regioncode="130926" if regioncode=="130926" & year==1998

replace regioncode="130927" if regioncode=="130927" & year==1998

replace regioncode="130928" if regioncode=="130928" & year==1998

replace regioncode="130929" if regioncode=="130929" & year==1998

replace regioncode="130930" if regioncode=="130930" & year==1998

replace regioncode="130981" if regioncode=="130981" & year==1998

replace regioncode="130982" if regioncode=="130982" & year==1998

replace regioncode="130983" if regioncode=="130983" & year==1998

replace regioncode="130984" if regioncode=="130984" & year==1998

replace regioncode="131002" if regioncode=="131002" & year==1998

replace regioncode="131003" if regioncode=="131003" & year==1998

replace regioncode="131022" if regioncode=="131022" & year==1998

replace regioncode="131023" if regioncode=="131023" & year==1998

replace regioncode="131024" if regioncode=="131024" & year==1998

replace regioncode="131025" if regioncode=="131025" & year==1998

replace regioncode="131026" if regioncode=="131026" & year==1998

replace regioncode="131028" if regioncode=="131028" & year==1998

replace regioncode="131081" if regioncode=="131081" & year==1998

replace regioncode="131082" if regioncode=="131082" & year==1998

replace regioncode="131102" if regioncode=="131102" & year==1998

replace regioncode="131121" if regioncode=="131121" & year==1998

replace regioncode="131122" if regioncode=="131122" & year==1998

replace regioncode="131123" if regioncode=="131123" & year==1998

replace regioncode="131124" if regioncode=="131124" & year==1998

replace regioncode="131125" if regioncode=="131125" & year==1998

replace regioncode="131126" if regioncode=="131126" & year==1998

replace regioncode="131127" if regioncode=="131127" & year==1998

replace regioncode="131128" if regioncode=="131128" & year==1998

replace regioncode="131181" if regioncode=="131181" & year==1998

replace regioncode="131182" if regioncode=="131182" & year==1998

replace regioncode="140105" if regioncode=="140105" & year==1998

replace regioncode="140106" if regioncode=="140106" & year==1998

replace regioncode="140107" if regioncode=="140107" & year==1998

replace regioncode="140108" if regioncode=="140108" & year==1998

replace regioncode="140109" if regioncode=="140109" & year==1998

replace regioncode="140110" if regioncode=="140110" & year==1998

replace regioncode="140121" if regioncode=="140121" & year==1998

replace regioncode="140122" if regioncode=="140122" & year==1998

replace regioncode="140123" if regioncode=="140123" & year==1998

replace regioncode="140181" if regioncode=="140181" & year==1998

replace regioncode="140202" if regioncode=="140202" & year==1998

replace regioncode="140203" if regioncode=="140203" & year==1998

replace regioncode="140211" if regioncode=="140211" & year==1998

replace regioncode="140212" if regioncode=="140212" & year==1998

replace regioncode="140221" if regioncode=="140221" & year==1998

replace regioncode="140222" if regioncode=="140222" & year==1998

replace regioncode="140223" if regioncode=="140223" & year==1998

replace regioncode="140224" if regioncode=="140224" & year==1998

replace regioncode="140225" if regioncode=="140225" & year==1998

replace regioncode="140226" if regioncode=="140226" & year==1998

replace regioncode="140227" if regioncode=="140227" & year==1998

replace regioncode="140302" if regioncode=="140302" & year==1998

replace regioncode="140303" if regioncode=="140303" & year==1998

replace regioncode="140311" if regioncode=="140311" & year==1998

replace regioncode="140321" if regioncode=="140321" & year==1998

replace regioncode="140322" if regioncode=="140322" & year==1998

replace regioncode="140402" if regioncode=="140402" & year==1998

replace regioncode="140411" if regioncode=="140411" & year==1998

replace regioncode="140421" if regioncode=="140421" & year==1998

replace regioncode="140423" if regioncode=="140423" & year==1998

replace regioncode="140424" if regioncode=="140424" & year==1998

replace regioncode="140425" if regioncode=="140425" & year==1998

replace regioncode="140426" if regioncode=="140426" & year==1998

replace regioncode="140427" if regioncode=="140427" & year==1998

replace regioncode="140428" if regioncode=="140428" & year==1998

replace regioncode="140429" if regioncode=="140429" & year==1998

replace regioncode="140430" if regioncode=="140430" & year==1998

replace regioncode="140431" if regioncode=="140431" & year==1998

replace regioncode="140481" if regioncode=="140481" & year==1998

replace regioncode="140502" if regioncode=="140502" & year==1998

replace regioncode="140521" if regioncode=="140521" & year==1998

replace regioncode="140522" if regioncode=="140522" & year==1998

replace regioncode="140524" if regioncode=="140524" & year==1998

replace regioncode="140525" if regioncode=="140581" & year==1998

replace regioncode="140581" if regioncode=="140601" & year==1998

replace regioncode="140602" if regioncode=="140602" & year==1998

replace regioncode="140603" if regioncode=="140603" & year==1998

replace regioncode="140621" if regioncode=="140621" & year==1998

replace regioncode="140622" if regioncode=="140622" & year==1998

replace regioncode="140623" if regioncode=="140623" & year==1998

replace regioncode="140624" if regioncode=="140624" & year==1998

replace regioncode="140902" if regioncode=="142201" & year==1998

replace regioncode="140981" if regioncode=="142202" & year==1998

replace regioncode="140921" if regioncode=="142222" & year==1998

replace regioncode="140922" if regioncode=="142223" & year==1998

replace regioncode="140923" if regioncode=="142225" & year==1998

replace regioncode="140924" if regioncode=="142226" & year==1998

replace regioncode="140925" if regioncode=="142227" & year==1998

replace regioncode="140926" if regioncode=="142228" & year==1998

replace regioncode="140927" if regioncode=="142229" & year==1998

replace regioncode="140928" if regioncode=="142230" & year==1998

replace regioncode="140929" if regioncode=="142231" & year==1998

replace regioncode="140930" if regioncode=="142232" & year==1998

replace regioncode="140931" if regioncode=="142233" & year==1998

replace regioncode="140932" if regioncode=="142234" & year==1998

replace regioncode="141181" if regioncode=="142301" & year==1998

replace regioncode="141102" if regioncode=="142321" & year==1998

replace regioncode="141182" if regioncode=="142322" & year==1998

replace regioncode="141121" if regioncode=="142323" & year==1998

replace regioncode="141122" if regioncode=="142325" & year==1998

replace regioncode="141123" if regioncode=="142326" & year==1998

replace regioncode="141124" if regioncode=="142327" & year==1998

replace regioncode="141125" if regioncode=="142328" & year==1998

replace regioncode="141126" if regioncode=="142329" & year==1998

replace regioncode="141127" if regioncode=="142330" & year==1998

replace regioncode="141128" if regioncode=="142331" & year==1998

replace regioncode="141129" if regioncode=="142332" & year==1998

replace regioncode="141130" if regioncode=="142333" & year==1998

replace regioncode="140702" if regioncode=="142401" & year==1998

replace regioncode="140781" if regioncode=="142402" & year==1998

replace regioncode="140721" if regioncode=="142421" & year==1998

replace regioncode="140722" if regioncode=="142422" & year==1998

replace regioncode="140723" if regioncode=="142423" & year==1998

replace regioncode="140724" if regioncode=="142424" & year==1998

replace regioncode="140725" if regioncode=="142427" & year==1998

replace regioncode="140726" if regioncode=="142429" & year==1998

replace regioncode="140727" if regioncode=="142430" & year==1998

replace regioncode="140728" if regioncode=="142431" & year==1998

replace regioncode="140729" if regioncode=="142433" & year==1998

replace regioncode="141002" if regioncode=="142601" & year==1998

replace regioncode="141081" if regioncode=="142602" & year==1998

replace regioncode="141082" if regioncode=="142603" & year==1998

replace regioncode="141021" if regioncode=="142621" & year==1998

replace regioncode="141022" if regioncode=="142622" & year==1998

replace regioncode="141023" if regioncode=="142623" & year==1998

replace regioncode="141024" if regioncode=="142625" & year==1998

replace regioncode="141025" if regioncode=="142627" & year==1998

replace regioncode="141026" if regioncode=="142628" & year==1998

replace regioncode="141027" if regioncode=="142629" & year==1998

replace regioncode="141028" if regioncode=="142630" & year==1998

replace regioncode="141029" if regioncode=="142631" & year==1998

replace regioncode="141033" if regioncode=="142632" & year==1998

replace regioncode="141030" if regioncode=="142633" & year==1998

replace regioncode="141032" if regioncode=="142634" & year==1998

replace regioncode="141031" if regioncode=="142635" & year==1998

replace regioncode="141034" if regioncode=="142636" & year==1998

replace regioncode="140802" if regioncode=="142701" & year==1998

replace regioncode="140881" if regioncode=="142702" & year==1998

replace regioncode="140882" if regioncode=="142703" & year==1998

replace regioncode="140830" if regioncode=="142723" & year==1998

replace regioncode="140821" if regioncode=="142724" & year==1998

replace regioncode="140822" if regioncode=="142725" & year==1998

replace regioncode="140825" if regioncode=="142726" & year==1998

replace regioncode="140824" if regioncode=="142727" & year==1998

replace regioncode="140823" if regioncode=="142729" & year==1998

replace regioncode="140828" if regioncode=="142730" & year==1998

replace regioncode="140826" if regioncode=="142731" & year==1998

replace regioncode="140829" if regioncode=="142732" & year==1998

replace regioncode="140827" if regioncode=="142733" & year==1998

replace regioncode="150102" if regioncode=="150102" & year==1998

replace regioncode="150103" if regioncode=="150103" & year==1998

replace regioncode="150104" if regioncode=="150104" & year==1998

replace regioncode="150105" if regioncode=="150105" & year==1998

replace regioncode="150121" if regioncode=="150121" & year==1998

replace regioncode="150122" if regioncode=="150122" & year==1998

replace regioncode="150123" if regioncode=="150123" & year==1998

replace regioncode="150124" if regioncode=="150124" & year==1998

replace regioncode="150125" if regioncode=="150125" & year==1998

replace regioncode="150202" if regioncode=="150202" & year==1998

replace regioncode="150203" if regioncode=="150203" & year==1998

replace regioncode="150204" if regioncode=="150204" & year==1998

replace regioncode="150205" if regioncode=="150205" & year==1998

replace regioncode="150206" if regioncode=="150206" & year==1998

replace regioncode="150207" if regioncode=="150207" & year==1998

replace regioncode="150221" if regioncode=="150221" & year==1998

replace regioncode="150222" if regioncode=="150222" & year==1998

replace regioncode="150223" if regioncode=="150223" & year==1998

replace regioncode="150302" if regioncode=="150302" & year==1998

replace regioncode="150303" if regioncode=="150303" & year==1998

replace regioncode="150304" if regioncode=="150304" & year==1998

replace regioncode="150402" if regioncode=="150402" & year==1998

replace regioncode="150403" if regioncode=="150403" & year==1998

replace regioncode="150404" if regioncode=="150404" & year==1998

replace regioncode="150421" if regioncode=="150421" & year==1998

replace regioncode="150422" if regioncode=="150422" & year==1998

replace regioncode="150423" if regioncode=="150423" & year==1998

replace regioncode="150424" if regioncode=="150424" & year==1998

replace regioncode="150425" if regioncode=="150425" & year==1998

replace regioncode="150426" if regioncode=="150426" & year==1998

replace regioncode="150428" if regioncode=="150428" & year==1998

replace regioncode="150429" if regioncode=="150429" & year==1998

replace regioncode="150430" if regioncode=="150430" & year==1998

replace regioncode="150702" if regioncode=="152101" & year==1998

replace regioncode="150781" if regioncode=="152102" & year==1998

replace regioncode="150783" if regioncode=="152103" & year==1998

replace regioncode="150782" if regioncode=="152104" & year==1998

replace regioncode="150785" if regioncode=="152105" & year==1998

replace regioncode="150784" if regioncode=="152106" & year==1998

replace regioncode="150721" if regioncode=="152122" & year==1998

replace regioncode="150722" if regioncode=="152123" & year==1998

replace regioncode="150723" if regioncode=="152127" & year==1998

replace regioncode="150724" if regioncode=="152128" & year==1998

replace regioncode="150727" if regioncode=="152129" & year==1998

replace regioncode="150726" if regioncode=="152130" & year==1998

replace regioncode="150725" if regioncode=="152131" & year==1998

replace regioncode="152201" if regioncode=="152201" & year==1998

replace regioncode="152202" if regioncode=="152202" & year==1998

replace regioncode="152221" if regioncode=="152221" & year==1998

replace regioncode="152222" if regioncode=="152222" & year==1998

replace regioncode="152223" if regioncode=="152223" & year==1998

replace regioncode="152224" if regioncode=="152224" & year==1998

replace regioncode="150502" if regioncode=="152301" & year==1998

replace regioncode="150581" if regioncode=="152302" & year==1998

replace regioncode="150521" if regioncode=="152322" & year==1998

replace regioncode="150522" if regioncode=="152323" & year==1998

replace regioncode="150523" if regioncode=="152324" & year==1998

replace regioncode="150524" if regioncode=="152325" & year==1998

replace regioncode="150525" if regioncode=="152326" & year==1998

replace regioncode="150526" if regioncode=="152327" & year==1998

replace regioncode="152501" if regioncode=="152501" & year==1998

replace regioncode="152502" if regioncode=="152502" & year==1998

replace regioncode="152522" if regioncode=="152522" & year==1998

replace regioncode="152523" if regioncode=="152523" & year==1998

replace regioncode="152524" if regioncode=="152524" & year==1998

replace regioncode="152525" if regioncode=="152525" & year==1998

replace regioncode="152526" if regioncode=="152526" & year==1998

replace regioncode="152527" if regioncode=="152527" & year==1998

replace regioncode="152528" if regioncode=="152528" & year==1998

replace regioncode="152529" if regioncode=="152529" & year==1998

replace regioncode="152530" if regioncode=="152530" & year==1998

replace regioncode="152531" if regioncode=="152531" & year==1998

replace regioncode="152601" if regioncode=="152601" & year==1998

replace regioncode="152602" if regioncode=="152602" & year==1998

replace regioncode="152624" if regioncode=="152624" & year==1998

replace regioncode="152625" if regioncode=="152625" & year==1998

replace regioncode="152626" if regioncode=="152626" & year==1998

replace regioncode="152627" if regioncode=="152627" & year==1998

replace regioncode="152629" if regioncode=="152629" & year==1998

replace regioncode="152630" if regioncode=="152630" & year==1998

replace regioncode="152631" if regioncode=="152631" & year==1998

replace regioncode="152632" if regioncode=="152632" & year==1998

replace regioncode="152634" if regioncode=="152634" & year==1998

replace regioncode="150602" if regioncode=="152701" & year==1998

replace regioncode="150621" if regioncode=="152722" & year==1998

replace regioncode="150622" if regioncode=="152723" & year==1998

replace regioncode="150623" if regioncode=="152724" & year==1998

replace regioncode="150624" if regioncode=="152725" & year==1998

replace regioncode="150625" if regioncode=="152726" & year==1998

replace regioncode="150626" if regioncode=="152727" & year==1998

replace regioncode="150627" if regioncode=="152728" & year==1998

replace regioncode="152801" if regioncode=="152801" & year==1998

replace regioncode="152822" if regioncode=="152822" & year==1998

replace regioncode="152823" if regioncode=="152823" & year==1998

replace regioncode="152824" if regioncode=="152824" & year==1998

replace regioncode="152825" if regioncode=="152825" & year==1998

replace regioncode="152826" if regioncode=="152826" & year==1998

replace regioncode="152827" if regioncode=="152827" & year==1998

replace regioncode="152921" if regioncode=="152921" & year==1998

replace regioncode="152922" if regioncode=="152922" & year==1998

replace regioncode="152923" if regioncode=="152923" & year==1998

replace regioncode="210102" if regioncode=="210102" & year==1998

replace regioncode="210103" if regioncode=="210103" & year==1998

replace regioncode="210104" if regioncode=="210104" & year==1998

replace regioncode="210105" if regioncode=="210105" & year==1998

replace regioncode="210106" if regioncode=="210106" & year==1998

replace regioncode="210111" if regioncode=="210111" & year==1998

replace regioncode="210112" if regioncode=="210112" & year==1998

replace regioncode="210113" if regioncode=="210113" & year==1998

replace regioncode="210114" if regioncode=="210114" & year==1998

replace regioncode="210122" if regioncode=="210122" & year==1998

replace regioncode="210123" if regioncode=="210123" & year==1998

replace regioncode="210124" if regioncode=="210124" & year==1998

replace regioncode="210181" if regioncode=="210181" & year==1998

replace regioncode="210202" if regioncode=="210202" & year==1998

replace regioncode="210203" if regioncode=="210203" & year==1998

replace regioncode="210204" if regioncode=="210204" & year==1998

replace regioncode="210211" if regioncode=="210211" & year==1998

replace regioncode="210212" if regioncode=="210212" & year==1998

replace regioncode="210213" if regioncode=="210213" & year==1998

replace regioncode="210224" if regioncode=="210224" & year==1998

replace regioncode="210281" if regioncode=="210281" & year==1998

replace regioncode="210282" if regioncode=="210282" & year==1998

replace regioncode="210283" if regioncode=="210283" & year==1998

replace regioncode="210302" if regioncode=="210302" & year==1998

replace regioncode="210303" if regioncode=="210303" & year==1998

replace regioncode="210304" if regioncode=="210304" & year==1998

replace regioncode="210311" if regioncode=="210311" & year==1998

replace regioncode="210321" if regioncode=="210321" & year==1998

replace regioncode="210323" if regioncode=="210323" & year==1998

replace regioncode="210381" if regioncode=="210381" & year==1998

replace regioncode="210402" if regioncode=="210402" & year==1998

replace regioncode="210403" if regioncode=="210403" & year==1998

replace regioncode="210404" if regioncode=="210404" & year==1998

replace regioncode="210411" if regioncode=="210411" & year==1998

replace regioncode="210421" if regioncode=="210421" & year==1998

replace regioncode="210422" if regioncode=="210422" & year==1998

replace regioncode="210423" if regioncode=="210423" & year==1998

replace regioncode="210502" if regioncode=="210502" & year==1998

replace regioncode="210503" if regioncode=="210503" & year==1998

replace regioncode="210504" if regioncode=="210504" & year==1998

replace regioncode="210505" if regioncode=="210505" & year==1998

replace regioncode="210521" if regioncode=="210521" & year==1998

replace regioncode="210522" if regioncode=="210522" & year==1998

replace regioncode="210602" if regioncode=="210602" & year==1998

replace regioncode="210603" if regioncode=="210603" & year==1998

replace regioncode="210604" if regioncode=="210604" & year==1998

replace regioncode="210624" if regioncode=="210624" & year==1998

replace regioncode="210681" if regioncode=="210681" & year==1998

replace regioncode="210682" if regioncode=="210682" & year==1998

replace regioncode="210702" if regioncode=="210702" & year==1998

replace regioncode="210703" if regioncode=="210703" & year==1998

replace regioncode="210711" if regioncode=="210711" & year==1998

replace regioncode="210726" if regioncode=="210726" & year==1998

replace regioncode="210727" if regioncode=="210727" & year==1998

replace regioncode="210781" if regioncode=="210781" & year==1998

replace regioncode="210782" if regioncode=="210782" & year==1998

replace regioncode="210802" if regioncode=="210802" & year==1998

replace regioncode="210803" if regioncode=="210803" & year==1998

replace regioncode="210804" if regioncode=="210804" & year==1998

replace regioncode="210811" if regioncode=="210811" & year==1998

replace regioncode="210881" if regioncode=="210881" & year==1998

replace regioncode="210882" if regioncode=="210882" & year==1998

replace regioncode="210902" if regioncode=="210902" & year==1998

replace regioncode="210903" if regioncode=="210903" & year==1998

replace regioncode="210904" if regioncode=="210904" & year==1998

replace regioncode="210905" if regioncode=="210905" & year==1998

replace regioncode="210911" if regioncode=="210911" & year==1998

replace regioncode="210921" if regioncode=="210921" & year==1998

replace regioncode="210922" if regioncode=="210922" & year==1998

replace regioncode="211002" if regioncode=="211002" & year==1998

replace regioncode="211003" if regioncode=="211003" & year==1998

replace regioncode="211004" if regioncode=="211004" & year==1998

replace regioncode="211005" if regioncode=="211005" & year==1998

replace regioncode="211011" if regioncode=="211011" & year==1998

replace regioncode="211021" if regioncode=="211021" & year==1998

replace regioncode="211081" if regioncode=="211081" & year==1998

replace regioncode="211102" if regioncode=="211102" & year==1998

replace regioncode="211103" if regioncode=="211103" & year==1998

replace regioncode="211121" if regioncode=="211121" & year==1998

replace regioncode="211122" if regioncode=="211122" & year==1998

replace regioncode="211202" if regioncode=="211202" & year==1998

replace regioncode="211204" if regioncode=="211204" & year==1998

replace regioncode="211221" if regioncode=="211221" & year==1998

replace regioncode="211223" if regioncode=="211223" & year==1998

replace regioncode="211224" if regioncode=="211224" & year==1998

replace regioncode="211281" if regioncode=="211281" & year==1998

replace regioncode="211282" if regioncode=="211282" & year==1998

replace regioncode="211302" if regioncode=="211302" & year==1998

replace regioncode="211303" if regioncode=="211303" & year==1998

replace regioncode="211321" if regioncode=="211321" & year==1998

replace regioncode="211322" if regioncode=="211322" & year==1998

replace regioncode="211324" if regioncode=="211324" & year==1998

replace regioncode="211381" if regioncode=="211381" & year==1998

replace regioncode="211382" if regioncode=="211382" & year==1998

replace regioncode="211402" if regioncode=="211402" & year==1998

replace regioncode="211403" if regioncode=="211403" & year==1998

replace regioncode="211404" if regioncode=="211404" & year==1998

replace regioncode="211421" if regioncode=="211421" & year==1998

replace regioncode="211422" if regioncode=="211422" & year==1998

replace regioncode="211481" if regioncode=="211481" & year==1998

replace regioncode="220102" if regioncode=="220102" & year==1998

replace regioncode="220103" if regioncode=="220103" & year==1998

replace regioncode="220104" if regioncode=="220104" & year==1998

replace regioncode="220105" if regioncode=="220105" & year==1998

replace regioncode="220106" if regioncode=="220106" & year==1998

replace regioncode="220112" if regioncode=="220112" & year==1998

replace regioncode="220122" if regioncode=="220122" & year==1998

replace regioncode="220181" if regioncode=="220181" & year==1998

replace regioncode="220182" if regioncode=="220182" & year==1998

replace regioncode="220183" if regioncode=="220183" & year==1998

replace regioncode="220202" if regioncode=="220202" & year==1998

replace regioncode="220203" if regioncode=="220203" & year==1998

replace regioncode="220204" if regioncode=="220204" & year==1998

replace regioncode="220211" if regioncode=="220205" & year==1998

replace regioncode="220221" if regioncode=="220221" & year==1998

replace regioncode="220281" if regioncode=="220281" & year==1998

replace regioncode="220282" if regioncode=="220282" & year==1998

replace regioncode="220283" if regioncode=="220283" & year==1998

replace regioncode="220284" if regioncode=="220284" & year==1998

replace regioncode="220302" if regioncode=="220302" & year==1998

replace regioncode="220303" if regioncode=="220303" & year==1998

replace regioncode="220322" if regioncode=="220322" & year==1998

replace regioncode="220323" if regioncode=="220323" & year==1998

replace regioncode="220381" if regioncode=="220381" & year==1998

replace regioncode="220382" if regioncode=="220382" & year==1998

replace regioncode="220402" if regioncode=="220402" & year==1998

replace regioncode="220403" if regioncode=="220403" & year==1998

replace regioncode="220421" if regioncode=="220421" & year==1998

replace regioncode="220422" if regioncode=="220422" & year==1998

replace regioncode="220502" if regioncode=="220502" & year==1998

replace regioncode="220503" if regioncode=="220503" & year==1998

replace regioncode="220521" if regioncode=="220521" & year==1998

replace regioncode="220523" if regioncode=="220523" & year==1998

replace regioncode="220524" if regioncode=="220524" & year==1998

replace regioncode="220581" if regioncode=="220581" & year==1998

replace regioncode="220582" if regioncode=="220582" & year==1998

replace regioncode="220602" if regioncode=="220602" & year==1998

replace regioncode="220621" if regioncode=="220621" & year==1998

replace regioncode="220622" if regioncode=="220622" & year==1998

replace regioncode="220623" if regioncode=="220623" & year==1998

replace regioncode="220625" if regioncode=="220625" & year==1998

replace regioncode="220681" if regioncode=="220681" & year==1998

replace regioncode="220702" if regioncode=="220702" & year==1998

replace regioncode="220721" if regioncode=="220721" & year==1998

replace regioncode="220722" if regioncode=="220722" & year==1998

replace regioncode="220723" if regioncode=="220723" & year==1998

replace regioncode="220724" if regioncode=="220724" & year==1998

replace regioncode="220802" if regioncode=="220802" & year==1998

replace regioncode="220821" if regioncode=="220821" & year==1998

replace regioncode="220822" if regioncode=="220822" & year==1998

replace regioncode="220881" if regioncode=="220881" & year==1998

replace regioncode="220882" if regioncode=="220882" & year==1998

replace regioncode="222401" if regioncode=="222401" & year==1998

replace regioncode="222402" if regioncode=="222402" & year==1998

replace regioncode="222403" if regioncode=="222403" & year==1998

replace regioncode="222404" if regioncode=="222404" & year==1998

replace regioncode="222405" if regioncode=="222405" & year==1998

replace regioncode="222406" if regioncode=="222406" & year==1998

replace regioncode="222424" if regioncode=="222424" & year==1998

replace regioncode="222426" if regioncode=="222426" & year==1998

replace regioncode="230102" if regioncode=="230102" & year==1998

replace regioncode="230103" if regioncode=="230103" & year==1998

replace regioncode="230104" if regioncode=="230104" & year==1998

replace regioncode="230105" if regioncode=="230105" & year==1998

replace regioncode="230107" if regioncode=="230107" & year==1998

replace regioncode="230108" if regioncode=="230108" & year==1998

replace regioncode="230121" if regioncode=="230121" & year==1998

replace regioncode="230123" if regioncode=="230123" & year==1998

replace regioncode="230124" if regioncode=="230124" & year==1998

replace regioncode="230125" if regioncode=="230125" & year==1998

replace regioncode="230126" if regioncode=="230126" & year==1998

replace regioncode="230127" if regioncode=="230127" & year==1998

replace regioncode="230128" if regioncode=="230128" & year==1998

replace regioncode="230129" if regioncode=="230129" & year==1998

replace regioncode="230181" if regioncode=="230181" & year==1998

replace regioncode="230182" if regioncode=="230182" & year==1998

replace regioncode="230183" if regioncode=="230183" & year==1998

replace regioncode="230184" if regioncode=="230184" & year==1998

replace regioncode="230202" if regioncode=="230202" & year==1998

replace regioncode="230203" if regioncode=="230203" & year==1998

replace regioncode="230204" if regioncode=="230204" & year==1998

replace regioncode="230205" if regioncode=="230205" & year==1998

replace regioncode="230206" if regioncode=="230206" & year==1998

replace regioncode="230207" if regioncode=="230207" & year==1998

replace regioncode="230208" if regioncode=="230208" & year==1998

replace regioncode="230221" if regioncode=="230221" & year==1998

replace regioncode="230223" if regioncode=="230223" & year==1998

replace regioncode="230224" if regioncode=="230224" & year==1998

replace regioncode="230225" if regioncode=="230225" & year==1998

replace regioncode="230227" if regioncode=="230227" & year==1998

replace regioncode="230229" if regioncode=="230229" & year==1998

replace regioncode="230230" if regioncode=="230230" & year==1998

replace regioncode="230231" if regioncode=="230231" & year==1998

replace regioncode="230281" if regioncode=="230281" & year==1998

replace regioncode="230302" if regioncode=="230302" & year==1998

replace regioncode="230303" if regioncode=="230303" & year==1998

replace regioncode="230304" if regioncode=="230304" & year==1998

replace regioncode="230305" if regioncode=="230305" & year==1998

replace regioncode="230306" if regioncode=="230306" & year==1998

replace regioncode="230307" if regioncode=="230307" & year==1998

replace regioncode="230321" if regioncode=="230321" & year==1998

replace regioncode="230381" if regioncode=="230381" & year==1998

replace regioncode="230382" if regioncode=="230382" & year==1998

replace regioncode="230402" if regioncode=="230402" & year==1998

replace regioncode="230403" if regioncode=="230403" & year==1998

replace regioncode="230404" if regioncode=="230404" & year==1998

replace regioncode="230405" if regioncode=="230405" & year==1998

replace regioncode="230406" if regioncode=="230406" & year==1998

replace regioncode="230407" if regioncode=="230407" & year==1998

replace regioncode="230421" if regioncode=="230421" & year==1998

replace regioncode="230422" if regioncode=="230422" & year==1998

replace regioncode="230502" if regioncode=="230502" & year==1998

replace regioncode="230503" if regioncode=="230503" & year==1998

replace regioncode="230505" if regioncode=="230505" & year==1998

replace regioncode="230506" if regioncode=="230506" & year==1998

replace regioncode="230521" if regioncode=="230521" & year==1998

replace regioncode="230522" if regioncode=="230522" & year==1998

replace regioncode="230523" if regioncode=="230523" & year==1998

replace regioncode="230524" if regioncode=="230524" & year==1998

replace regioncode="230602" if regioncode=="230602" & year==1998

replace regioncode="230603" if regioncode=="230603" & year==1998

replace regioncode="230604" if regioncode=="230604" & year==1998

replace regioncode="230605" if regioncode=="230605" & year==1998

replace regioncode="230606" if regioncode=="230606" & year==1998

replace regioncode="230621" if regioncode=="230621" & year==1998

replace regioncode="230622" if regioncode=="230622" & year==1998

replace regioncode="230623" if regioncode=="230623" & year==1998

replace regioncode="230624" if regioncode=="230624" & year==1998

replace regioncode="230702" if regioncode=="230702" & year==1998

replace regioncode="230703" if regioncode=="230703" & year==1998

replace regioncode="230704" if regioncode=="230704" & year==1998

replace regioncode="230705" if regioncode=="230705" & year==1998

replace regioncode="230706" if regioncode=="230706" & year==1998

replace regioncode="230707" if regioncode=="230707" & year==1998

replace regioncode="230708" if regioncode=="230708" & year==1998

replace regioncode="230709" if regioncode=="230709" & year==1998

replace regioncode="230710" if regioncode=="230710" & year==1998

replace regioncode="230711" if regioncode=="230711" & year==1998

replace regioncode="230712" if regioncode=="230712" & year==1998

replace regioncode="230713" if regioncode=="230713" & year==1998

replace regioncode="230714" if regioncode=="230714" & year==1998

replace regioncode="230715" if regioncode=="230715" & year==1998

replace regioncode="230716" if regioncode=="230716" & year==1998

replace regioncode="230722" if regioncode=="230722" & year==1998

replace regioncode="230781" if regioncode=="230781" & year==1998

replace regioncode="230802" if regioncode=="230802" & year==1998

replace regioncode="230803" if regioncode=="230803" & year==1998

replace regioncode="230804" if regioncode=="230804" & year==1998

replace regioncode="230805" if regioncode=="230805" & year==1998

replace regioncode="230811" if regioncode=="230811" & year==1998

replace regioncode="230822" if regioncode=="230822" & year==1998

replace regioncode="230826" if regioncode=="230826" & year==1998

replace regioncode="230828" if regioncode=="230828" & year==1998

replace regioncode="230833" if regioncode=="230833" & year==1998

replace regioncode="230881" if regioncode=="230881" & year==1998

replace regioncode="230882" if regioncode=="230882" & year==1998

replace regioncode="230902" if regioncode=="230902" & year==1998

replace regioncode="230903" if regioncode=="230903" & year==1998

replace regioncode="230904" if regioncode=="230904" & year==1998

replace regioncode="230921" if regioncode=="230921" & year==1998

replace regioncode="231002" if regioncode=="231002" & year==1998

replace regioncode="231003" if regioncode=="231003" & year==1998

replace regioncode="231004" if regioncode=="231004" & year==1998

replace regioncode="231005" if regioncode=="231005" & year==1998

replace regioncode="231024" if regioncode=="231024" & year==1998

replace regioncode="231025" if regioncode=="231025" & year==1998

replace regioncode="231081" if regioncode=="231081" & year==1998

replace regioncode="231083" if regioncode=="231083" & year==1998

replace regioncode="231084" if regioncode=="231084" & year==1998

replace regioncode="231085" if regioncode=="231085" & year==1998

replace regioncode="231102" if regioncode=="231102" & year==1998

replace regioncode="231121" if regioncode=="231121" & year==1998

replace regioncode="231123" if regioncode=="231123" & year==1998

replace regioncode="231124" if regioncode=="231124" & year==1998

replace regioncode="231181" if regioncode=="231181" & year==1998

replace regioncode="231182" if regioncode=="231182" & year==1998

replace regioncode="231202" if regioncode=="232301" & year==1998

replace regioncode="231221" if regioncode=="232324" & year==1998

replace regioncode="231222" if regioncode=="232325" & year==1998

replace regioncode="231223" if regioncode=="232326" & year==1998

replace regioncode="231224" if regioncode=="232330" & year==1998

replace regioncode="231225" if regioncode=="232331" & year==1998

replace regioncode="231226" if regioncode=="232332" & year==1998

replace regioncode="231281" if regioncode=="232302" & year==1998

replace regioncode="231282" if regioncode=="232303" & year==1998

replace regioncode="231283" if regioncode=="232304" & year==1998

replace regioncode="232701" if regioncode=="232701" & year==1998

replace regioncode="232702" if regioncode=="232702" & year==1998

replace regioncode="232703" if regioncode=="232703" & year==1998

replace regioncode="232704" if regioncode=="232704" & year==1998

replace regioncode="232721" if regioncode=="232721" & year==1998

replace regioncode="232722" if regioncode=="232722" & year==1998

replace regioncode="232723" if regioncode=="232723" & year==1998

replace regioncode="310101" if regioncode=="310101" & year==1998

replace regioncode="310103" if regioncode=="310103" & year==1998

replace regioncode="310104" if regioncode=="310104" & year==1998

replace regioncode="310105" if regioncode=="310105" & year==1998

replace regioncode="310106" if regioncode=="310106" & year==1998

replace regioncode="310107" if regioncode=="310107" & year==1998

replace regioncode="310108" if regioncode=="310108" & year==1998

replace regioncode="310109" if regioncode=="310109" & year==1998

replace regioncode="310110" if regioncode=="310110" & year==1998

replace regioncode="310112" if regioncode=="310112" & year==1998

replace regioncode="310113" if regioncode=="310113" & year==1998

replace regioncode="310114" if regioncode=="310114" & year==1998

replace regioncode="310115" if regioncode=="310115" & year==1998

replace regioncode="310116" if regioncode=="310116" & year==1998

replace regioncode="310117" if regioncode=="310117" & year==1998

replace regioncode="310118" if regioncode=="310118" & year==1998

replace regioncode="310119" if regioncode=="310225" & year==1998

replace regioncode="310120" if regioncode=="310226" & year==1998

replace regioncode="310230" if regioncode=="310230" & year==1998

replace regioncode="320102" if regioncode=="320102" & year==1998

replace regioncode="320103" if regioncode=="320103" & year==1998

replace regioncode="320104" if regioncode=="320104" & year==1998

replace regioncode="320105" if regioncode=="320105" & year==1998

replace regioncode="320106" if regioncode=="320106" & year==1998

replace regioncode="320107" if regioncode=="320107" & year==1998

replace regioncode="320111" if regioncode=="320111" & year==1998

replace regioncode="320113" if regioncode=="320113" & year==1998

replace regioncode="320114" if regioncode=="320114" & year==1998

replace regioncode="320115" if regioncode=="320121" & year==1998

replace regioncode="320116" if regioncode=="320123" & year==1998

replace regioncode="320124" if regioncode=="320124" & year==1998

replace regioncode="320125" if regioncode=="320125" & year==1998

replace regioncode="320202" if regioncode=="320202" & year==1998

replace regioncode="320203" if regioncode=="320203" & year==1998

replace regioncode="320204" if regioncode=="320204" & year==1998

replace regioncode="320205" if regioncode=="320283" & year==1998

replace regioncode="320211" if regioncode=="320211" & year==1998

replace regioncode="320281" if regioncode=="320281" & year==1998

replace regioncode="320282" if regioncode=="320282" & year==1998

replace regioncode="320302" if regioncode=="320302" & year==1998

replace regioncode="320303" if regioncode=="320303" & year==1998

replace regioncode="320304" if regioncode=="320304" & year==1998

replace regioncode="320305" if regioncode=="320305" & year==1998

replace regioncode="320311" if regioncode=="320311" & year==1998

replace regioncode="320321" if regioncode=="320321" & year==1998

replace regioncode="320322" if regioncode=="320322" & year==1998

replace regioncode="320323" if regioncode=="320323" & year==1998

replace regioncode="320324" if regioncode=="320324" & year==1998

replace regioncode="320381" if regioncode=="320381" & year==1998

replace regioncode="320382" if regioncode=="320382" & year==1998

replace regioncode="320402" if regioncode=="320402" & year==1998

replace regioncode="320404" if regioncode=="320404" & year==1998

replace regioncode="320405" if regioncode=="320405" & year==1998

replace regioncode="320411" if regioncode=="320411" & year==1998

replace regioncode="320412" if regioncode=="320483" & year==1998

replace regioncode="320481" if regioncode=="320481" & year==1998

replace regioncode="320482" if regioncode=="320482" & year==1998

replace regioncode="320502" if regioncode=="320502" & year==1998

replace regioncode="320503" if regioncode=="320503" & year==1998

replace regioncode="320504" if regioncode=="320504" & year==1998

replace regioncode="320505" if regioncode=="320511" & year==1998

replace regioncode="320581" if regioncode=="320581" & year==1998

replace regioncode="320582" if regioncode=="320582" & year==1998

replace regioncode="320583" if regioncode=="320583" & year==1998

replace regioncode="320584" if regioncode=="320584" & year==1998

replace regioncode="320585" if regioncode=="320585" & year==1998

replace regioncode="320586" if regioncode=="320586" & year==1998

replace regioncode="320602" if regioncode=="320602" & year==1998

replace regioncode="320611" if regioncode=="320611" & year==1998

replace regioncode="320621" if regioncode=="320621" & year==1998

replace regioncode="320623" if regioncode=="320623" & year==1998

replace regioncode="320681" if regioncode=="320681" & year==1998

replace regioncode="320682" if regioncode=="320682" & year==1998

replace regioncode="320683" if regioncode=="320683" & year==1998

replace regioncode="320684" if regioncode=="320684" & year==1998

replace regioncode="320703" if regioncode=="320703" & year==1998

replace regioncode="320705" if regioncode=="320705" & year==1998

replace regioncode="320706" if regioncode=="320706" & year==1998

replace regioncode="320721" if regioncode=="320721" & year==1998

replace regioncode="320722" if regioncode=="320722" & year==1998

replace regioncode="320723" if regioncode=="320723" & year==1998

replace regioncode="320724" if regioncode=="320724" & year==1998

replace regioncode="320802" if regioncode=="320802" & year==1998

replace regioncode="320803" if regioncode=="320882" & year==1998

replace regioncode="320804" if regioncode=="320821" & year==1998

replace regioncode="320811" if regioncode=="320811" & year==1998

replace regioncode="320826" if regioncode=="320826" & year==1998

replace regioncode="320829" if regioncode=="320829" & year==1998

replace regioncode="320830" if regioncode=="320830" & year==1998

replace regioncode="320831" if regioncode=="320831" & year==1998

replace regioncode="320902" if regioncode=="320902" & year==1998

replace regioncode="320903" if regioncode=="320928" & year==1998

replace regioncode="320921" if regioncode=="320921" & year==1998

replace regioncode="320922" if regioncode=="320922" & year==1998

replace regioncode="320923" if regioncode=="320923" & year==1998

replace regioncode="320924" if regioncode=="320924" & year==1998

replace regioncode="320925" if regioncode=="320925" & year==1998

replace regioncode="320981" if regioncode=="320981" & year==1998

replace regioncode="320982" if regioncode=="320982" & year==1998

replace regioncode="321002" if regioncode=="321002" & year==1998

replace regioncode="321003" if regioncode=="321027" & year==1998

replace regioncode="321011" if regioncode=="321011" & year==1998

replace regioncode="321023" if regioncode=="321023" & year==1998

replace regioncode="321081" if regioncode=="321081" & year==1998

replace regioncode="321084" if regioncode=="321084" & year==1998

replace regioncode="321088" if regioncode=="321088" & year==1998

replace regioncode="321102" if regioncode=="321102" & year==1998

replace regioncode="321111" if regioncode=="321111" & year==1998

replace regioncode="321112" if regioncode=="321121" & year==1998

replace regioncode="321181" if regioncode=="321181" & year==1998

replace regioncode="321182" if regioncode=="321182" & year==1998

replace regioncode="321183" if regioncode=="321183" & year==1998

replace regioncode="321202" if regioncode=="321202" & year==1998

replace regioncode="321203" if regioncode=="321203" & year==1998

replace regioncode="321281" if regioncode=="321281" & year==1998

replace regioncode="321282" if regioncode=="321282" & year==1998

replace regioncode="321283" if regioncode=="321283" & year==1998

replace regioncode="321284" if regioncode=="321284" & year==1998

replace regioncode="321302" if regioncode=="321302" & year==1998

replace regioncode="321321" if regioncode=="321321" & year==1998

replace regioncode="321322" if regioncode=="321322" & year==1998

replace regioncode="321323" if regioncode=="321323" & year==1998

replace regioncode="321324" if regioncode=="321324" & year==1998

replace regioncode="330102" if regioncode=="330102" & year==1998

replace regioncode="330103" if regioncode=="330103" & year==1998

replace regioncode="330104" if regioncode=="330104" & year==1998

replace regioncode="330105" if regioncode=="330105" & year==1998

replace regioncode="330106" if regioncode=="330106" & year==1998

replace regioncode="330108" if regioncode=="330108" & year==1998

replace regioncode="330109" if regioncode=="330181" & year==1998

replace regioncode="330110" if regioncode=="330184" & year==1998

replace regioncode="330122" if regioncode=="330122" & year==1998

replace regioncode="330127" if regioncode=="330127" & year==1998

replace regioncode="330182" if regioncode=="330182" & year==1998

replace regioncode="330183" if regioncode=="330183" & year==1998

replace regioncode="330185" if regioncode=="330185" & year==1998

replace regioncode="330203" if regioncode=="330203" & year==1998

replace regioncode="330204" if regioncode=="330204" & year==1998

replace regioncode="330205" if regioncode=="330205" & year==1998

replace regioncode="330206" if regioncode=="330206" & year==1998

replace regioncode="330211" if regioncode=="330211" & year==1998

replace regioncode="330212" if regioncode=="330227" & year==1998

replace regioncode="330225" if regioncode=="330225" & year==1998

replace regioncode="330226" if regioncode=="330226" & year==1998

replace regioncode="330281" if regioncode=="330281" & year==1998

replace regioncode="330282" if regioncode=="330282" & year==1998

replace regioncode="330283" if regioncode=="330283" & year==1998

replace regioncode="330302" if regioncode=="330302" & year==1998

replace regioncode="330303" if regioncode=="330303" & year==1998

replace regioncode="330304" if regioncode=="330304" & year==1998

replace regioncode="330322" if regioncode=="330322" & year==1998

replace regioncode="330324" if regioncode=="330324" & year==1998

replace regioncode="330326" if regioncode=="330326" & year==1998

replace regioncode="330327" if regioncode=="330327" & year==1998

replace regioncode="330328" if regioncode=="330328" & year==1998

replace regioncode="330329" if regioncode=="330329" & year==1998

replace regioncode="330381" if regioncode=="330381" & year==1998

replace regioncode="330382" if regioncode=="330382" & year==1998

replace regioncode="330402" if regioncode=="330402" & year==1998

replace regioncode="330411" if regioncode=="330411" & year==1998

replace regioncode="330421" if regioncode=="330421" & year==1998

replace regioncode="330424" if regioncode=="330424" & year==1998

replace regioncode="330481" if regioncode=="330481" & year==1998

replace regioncode="330482" if regioncode=="330482" & year==1998

replace regioncode="330483" if regioncode=="330483" & year==1998

replace regioncode="330501" if regioncode=="330501" & year==1998

replace regioncode="330521" if regioncode=="330521" & year==1998

replace regioncode="330522" if regioncode=="330522" & year==1998

replace regioncode="330523" if regioncode=="330523" & year==1998

replace regioncode="330602" if regioncode=="330602" & year==1998

replace regioncode="330621" if regioncode=="330621" & year==1998

replace regioncode="330624" if regioncode=="330624" & year==1998

replace regioncode="330681" if regioncode=="330681" & year==1998

replace regioncode="330682" if regioncode=="330682" & year==1998

replace regioncode="330683" if regioncode=="330683" & year==1998

replace regioncode="330702" if regioncode=="330702" & year==1998

replace regioncode="330703" if regioncode=="330721" & year==1998

replace regioncode="330723" if regioncode=="330723" & year==1998

replace regioncode="330726" if regioncode=="330726" & year==1998

replace regioncode="330727" if regioncode=="330727" & year==1998

replace regioncode="330781" if regioncode=="330781" & year==1998

replace regioncode="330782" if regioncode=="330782" & year==1998

replace regioncode="330783" if regioncode=="330783" & year==1998

replace regioncode="330784" if regioncode=="330784" & year==1998

replace regioncode="330802" if regioncode=="330802" & year==1998

replace regioncode="330803" if regioncode=="330821" & year==1998

replace regioncode="330822" if regioncode=="330822" & year==1998

replace regioncode="330824" if regioncode=="330824" & year==1998

replace regioncode="330825" if regioncode=="330825" & year==1998

replace regioncode="330881" if regioncode=="330881" & year==1998

replace regioncode="330902" if regioncode=="330902" & year==1998

replace regioncode="330903" if regioncode=="330903" & year==1998

replace regioncode="330921" if regioncode=="330921" & year==1998

replace regioncode="330922" if regioncode=="330922" & year==1998

replace regioncode="331002" if regioncode=="331002" & year==1998

replace regioncode="331003" if regioncode=="331003" & year==1998

replace regioncode="331004" if regioncode=="331004" & year==1998

replace regioncode="331021" if regioncode=="331021" & year==1998

replace regioncode="331022" if regioncode=="331022" & year==1998

replace regioncode="331023" if regioncode=="331023" & year==1998

replace regioncode="331024" if regioncode=="331024" & year==1998

replace regioncode="331081" if regioncode=="331081" & year==1998

replace regioncode="331082" if regioncode=="331082" & year==1998

replace regioncode="331102" if regioncode=="332501" & year==1998

replace regioncode="331121" if regioncode=="332522" & year==1998

replace regioncode="331122" if regioncode=="332526" & year==1998

replace regioncode="331123" if regioncode=="332527" & year==1998

replace regioncode="331124" if regioncode=="332528" & year==1998

replace regioncode="331125" if regioncode=="332523" & year==1998

replace regioncode="331126" if regioncode=="332525" & year==1998

replace regioncode="331127" if regioncode=="332529" & year==1998

replace regioncode="331181" if regioncode=="332502" & year==1998

replace regioncode="340102" if regioncode=="340102" & year==1998

replace regioncode="340103" if regioncode=="340103" & year==1998

replace regioncode="340104" if regioncode=="340104" & year==1998

replace regioncode="340111" if regioncode=="340111" & year==1998

replace regioncode="340121" if regioncode=="340121" & year==1998

replace regioncode="340122" if regioncode=="340122" & year==1998

replace regioncode="340123" if regioncode=="340123" & year==1998

replace regioncode="340202" if regioncode=="340202" & year==1998

replace regioncode="340203" if regioncode=="340203" & year==1998

replace regioncode="340204" if regioncode=="340204" & year==1998

replace regioncode="340207" if regioncode=="340207" & year==1998

replace regioncode="340221" if regioncode=="340221" & year==1998

replace regioncode="340222" if regioncode=="340222" & year==1998

replace regioncode="340223" if regioncode=="340223" & year==1998

replace regioncode="340302" if regioncode=="340302" & year==1998

replace regioncode="340303" if regioncode=="340303" & year==1998

replace regioncode="340304" if regioncode=="340304" & year==1998

replace regioncode="340311" if regioncode=="340311" & year==1998

replace regioncode="340321" if regioncode=="340321" & year==1998

replace regioncode="340322" if regioncode=="340322" & year==1998

replace regioncode="340323" if regioncode=="340323" & year==1998

replace regioncode="340402" if regioncode=="340402" & year==1998

replace regioncode="340403" if regioncode=="340403" & year==1998

replace regioncode="340404" if regioncode=="340404" & year==1998

replace regioncode="340405" if regioncode=="340405" & year==1998

replace regioncode="340406" if regioncode=="340406" & year==1998

replace regioncode="340421" if regioncode=="340421" & year==1998

replace regioncode="340502" if regioncode=="340502" & year==1998

replace regioncode="340503" if regioncode=="340503" & year==1998

replace regioncode="340504" if regioncode=="340504" & year==1998

replace regioncode="340521" if regioncode=="340521" & year==1998

replace regioncode="340602" if regioncode=="340602" & year==1998

replace regioncode="340603" if regioncode=="340603" & year==1998

replace regioncode="340604" if regioncode=="340604" & year==1998

replace regioncode="340621" if regioncode=="340621" & year==1998

replace regioncode="340702" if regioncode=="340702" & year==1998

replace regioncode="340703" if regioncode=="340703" & year==1998

replace regioncode="340711" if regioncode=="340711" & year==1998

replace regioncode="340721" if regioncode=="340721" & year==1998

replace regioncode="340802" if regioncode=="340802" & year==1998

replace regioncode="340803" if regioncode=="340803" & year==1998

replace regioncode="340811" if regioncode=="340811" & year==1998

replace regioncode="340822" if regioncode=="340822" & year==1998

replace regioncode="340823" if regioncode=="340823" & year==1998

replace regioncode="340824" if regioncode=="340824" & year==1998

replace regioncode="340825" if regioncode=="340825" & year==1998

replace regioncode="340826" if regioncode=="340826" & year==1998

replace regioncode="340827" if regioncode=="340827" & year==1998

replace regioncode="340828" if regioncode=="340828" & year==1998

replace regioncode="340881" if regioncode=="340881" & year==1998

replace regioncode="341002" if regioncode=="341002" & year==1998

replace regioncode="341003" if regioncode=="341003" & year==1998

replace regioncode="341004" if regioncode=="341004" & year==1998

replace regioncode="341021" if regioncode=="341021" & year==1998

replace regioncode="341022" if regioncode=="341022" & year==1998

replace regioncode="341023" if regioncode=="341023" & year==1998

replace regioncode="341024" if regioncode=="341024" & year==1998

replace regioncode="341102" if regioncode=="341102" & year==1998

replace regioncode="341103" if regioncode=="341103" & year==1998

replace regioncode="341122" if regioncode=="341122" & year==1998

replace regioncode="341124" if regioncode=="341124" & year==1998

replace regioncode="341125" if regioncode=="341125" & year==1998

replace regioncode="341126" if regioncode=="341126" & year==1998

replace regioncode="341181" if regioncode=="341181" & year==1998

replace regioncode="341182" if regioncode=="341182" & year==1998

replace regioncode="341202" if regioncode=="341202" & year==1998

replace regioncode="341203" if regioncode=="341203" & year==1998

replace regioncode="341204" if regioncode=="341204" & year==1998

replace regioncode="341221" if regioncode=="341221" & year==1998

replace regioncode="341222" if regioncode=="341222" & year==1998

replace regioncode="341621" if regioncode=="341223" & year==1998

replace regioncode="341622" if regioncode=="341224" & year==1998

replace regioncode="341225" if regioncode=="341225" & year==1998

replace regioncode="341226" if regioncode=="341226" & year==1998

replace regioncode="341623" if regioncode=="341227" & year==1998

replace regioncode="341282" if regioncode=="341282" & year==1998

replace regioncode="341302" if regioncode=="342221" & year==1998

replace regioncode="341321" if regioncode=="342222" & year==1998

replace regioncode="341322" if regioncode=="342224" & year==1998

replace regioncode="341323" if regioncode=="342225" & year==1998

replace regioncode="341324" if regioncode=="342226" & year==1998

replace regioncode="341402" if regioncode=="342601" & year==1998

replace regioncode="341421" if regioncode=="342622" & year==1998

replace regioncode="341422" if regioncode=="342623" & year==1998

replace regioncode="341423" if regioncode=="342625" & year==1998

replace regioncode="341424" if regioncode=="342626" & year==1998

replace regioncode="341521" if regioncode=="342422" & year==1998

replace regioncode="341522" if regioncode=="342423" & year==1998

replace regioncode="341523" if regioncode=="342425" & year==1998

replace regioncode="341524" if regioncode=="342426" & year==1998

replace regioncode="341525" if regioncode=="342427" & year==1998

replace regioncode="341802" if regioncode=="342501" & year==1998

replace regioncode="341881" if regioncode=="342502" & year==1998

replace regioncode="341821" if regioncode=="342522" & year==1998

replace regioncode="341822" if regioncode=="342523" & year==1998

replace regioncode="341823" if regioncode=="342529" & year==1998

replace regioncode="341825" if regioncode=="342530" & year==1998

replace regioncode="341824" if regioncode=="342531" & year==1998

replace regioncode="341702" if regioncode=="342901" & year==1998

replace regioncode="341721" if regioncode=="342921" & year==1998

replace regioncode="341722" if regioncode=="342922" & year==1998

replace regioncode="341723" if regioncode=="342923" & year==1998

replace regioncode="341502" if regioncode=="342401" & year==1998

replace regioncode="341602" if regioncode=="341602" & year==1998

replace regioncode="350102" if regioncode=="350102" & year==1998

replace regioncode="350103" if regioncode=="350103" & year==1998

replace regioncode="350104" if regioncode=="350104" & year==1998

replace regioncode="350105" if regioncode=="350105" & year==1998

replace regioncode="350111" if regioncode=="350111" & year==1998

replace regioncode="350121" if regioncode=="350121" & year==1998

replace regioncode="350122" if regioncode=="350122" & year==1998

replace regioncode="350123" if regioncode=="350123" & year==1998

replace regioncode="350124" if regioncode=="350124" & year==1998

replace regioncode="350125" if regioncode=="350125" & year==1998

replace regioncode="350128" if regioncode=="350128" & year==1998

replace regioncode="350181" if regioncode=="350181" & year==1998

replace regioncode="350182" if regioncode=="350182" & year==1998

replace regioncode="350203" if regioncode=="350203" & year==1998

replace regioncode="350205" if regioncode=="350205" & year==1998

replace regioncode="350206" if regioncode=="350206" & year==1998

replace regioncode="350211" if regioncode=="350211" & year==1998

replace regioncode="350212" if regioncode=="350221" & year==1998

replace regioncode="350213" if regioncode=="350213" & year==1998

replace regioncode="350302" if regioncode=="350302" & year==1998

replace regioncode="350303" if regioncode=="350303" & year==1998

replace regioncode="350321" if regioncode=="350321" & year==1998

replace regioncode="350322" if regioncode=="350322" & year==1998

replace regioncode="350402" if regioncode=="350402" & year==1998

replace regioncode="350403" if regioncode=="350403" & year==1998

replace regioncode="350421" if regioncode=="350421" & year==1998

replace regioncode="350423" if regioncode=="350423" & year==1998

replace regioncode="350424" if regioncode=="350424" & year==1998

replace regioncode="350425" if regioncode=="350425" & year==1998

replace regioncode="350426" if regioncode=="350426" & year==1998

replace regioncode="350427" if regioncode=="350427" & year==1998

replace regioncode="350428" if regioncode=="350428" & year==1998

replace regioncode="350429" if regioncode=="350429" & year==1998

replace regioncode="350430" if regioncode=="350430" & year==1998

replace regioncode="350481" if regioncode=="350481" & year==1998

replace regioncode="350502" if regioncode=="350502" & year==1998

replace regioncode="350503" if regioncode=="350503" & year==1998

replace regioncode="350504" if regioncode=="350504" & year==1998

replace regioncode="350505" if regioncode=="350505" & year==1998

replace regioncode="350521" if regioncode=="350521" & year==1998

replace regioncode="350524" if regioncode=="350524" & year==1998

replace regioncode="350525" if regioncode=="350525" & year==1998

replace regioncode="350526" if regioncode=="350526" & year==1998

replace regioncode="350581" if regioncode=="350581" & year==1998

replace regioncode="350582" if regioncode=="350582" & year==1998

replace regioncode="350583" if regioncode=="350583" & year==1998

replace regioncode="350602" if regioncode=="350602" & year==1998

replace regioncode="350603" if regioncode=="350603" & year==1998

replace regioncode="350622" if regioncode=="350622" & year==1998

replace regioncode="350623" if regioncode=="350623" & year==1998

replace regioncode="350624" if regioncode=="350624" & year==1998

replace regioncode="350625" if regioncode=="350625" & year==1998

replace regioncode="350626" if regioncode=="350626" & year==1998

replace regioncode="350627" if regioncode=="350627" & year==1998

replace regioncode="350628" if regioncode=="350628" & year==1998

replace regioncode="350629" if regioncode=="350629" & year==1998

replace regioncode="350681" if regioncode=="350681" & year==1998

replace regioncode="350702" if regioncode=="350702" & year==1998

replace regioncode="350721" if regioncode=="350721" & year==1998

replace regioncode="350722" if regioncode=="350722" & year==1998

replace regioncode="350723" if regioncode=="350723" & year==1998

replace regioncode="350724" if regioncode=="350724" & year==1998

replace regioncode="350725" if regioncode=="350725" & year==1998

replace regioncode="350781" if regioncode=="350781" & year==1998

replace regioncode="350782" if regioncode=="350782" & year==1998

replace regioncode="350783" if regioncode=="350783" & year==1998

replace regioncode="350784" if regioncode=="350784" & year==1998

replace regioncode="350802" if regioncode=="350802" & year==1998

replace regioncode="350821" if regioncode=="350821" & year==1998

replace regioncode="350822" if regioncode=="350822" & year==1998

replace regioncode="350823" if regioncode=="350823" & year==1998

replace regioncode="350824" if regioncode=="350824" & year==1998

replace regioncode="350825" if regioncode=="350825" & year==1998

replace regioncode="350881" if regioncode=="350881" & year==1998

replace regioncode="350902" if regioncode=="352201" & year==1998

replace regioncode="350981" if regioncode=="352202" & year==1998

replace regioncode="350982" if regioncode=="352203" & year==1998

replace regioncode="350921" if regioncode=="352225" & year==1998

replace regioncode="350922" if regioncode=="352227" & year==1998

replace regioncode="350923" if regioncode=="352228" & year==1998

replace regioncode="350924" if regioncode=="352229" & year==1998

replace regioncode="350925" if regioncode=="352230" & year==1998

replace regioncode="350926" if regioncode=="352231" & year==1998

replace regioncode="360102" if regioncode=="360102" & year==1998

replace regioncode="360103" if regioncode=="360103" & year==1998

replace regioncode="360104" if regioncode=="360104" & year==1998

replace regioncode="360105" if regioncode=="360105" & year==1998

replace regioncode="360111" if regioncode=="360111" & year==1998

replace regioncode="360121" if regioncode=="360121" & year==1998

replace regioncode="360122" if regioncode=="360122" & year==1998

replace regioncode="360123" if regioncode=="360123" & year==1998

replace regioncode="360124" if regioncode=="360124" & year==1998

replace regioncode="360202" if regioncode=="360202" & year==1998

replace regioncode="360203" if regioncode=="360203" & year==1998

replace regioncode="360222" if regioncode=="360222" & year==1998

replace regioncode="360281" if regioncode=="360281" & year==1998

replace regioncode="360302" if regioncode=="360302" & year==1998

replace regioncode="360313" if regioncode=="360313" & year==1998

replace regioncode="360321" if regioncode=="360321" & year==1998

replace regioncode="360322" if regioncode=="360322" & year==1998

replace regioncode="360323" if regioncode=="360323" & year==1998

replace regioncode="360402" if regioncode=="360402" & year==1998

replace regioncode="360403" if regioncode=="360403" & year==1998

replace regioncode="360421" if regioncode=="360421" & year==1998

replace regioncode="360423" if regioncode=="360423" & year==1998

replace regioncode="360424" if regioncode=="360424" & year==1998

replace regioncode="360425" if regioncode=="360425" & year==1998

replace regioncode="360426" if regioncode=="360426" & year==1998

replace regioncode="360427" if regioncode=="360427" & year==1998

replace regioncode="360428" if regioncode=="360428" & year==1998

replace regioncode="360429" if regioncode=="360429" & year==1998

replace regioncode="360430" if regioncode=="360430" & year==1998

replace regioncode="360481" if regioncode=="360481" & year==1998

replace regioncode="360502" if regioncode=="360502" & year==1998

replace regioncode="360521" if regioncode=="360521" & year==1998

replace regioncode="360602" if regioncode=="360602" & year==1998

replace regioncode="360622" if regioncode=="360622" & year==1998

replace regioncode="360681" if regioncode=="360681" & year==1998

replace regioncode="360702" if regioncode=="362101" & year==1998

replace regioncode="360721" if regioncode=="362121" & year==1998

replace regioncode="360722" if regioncode=="362123" & year==1998

replace regioncode="360723" if regioncode=="362124" & year==1998

replace regioncode="360724" if regioncode=="362125" & year==1998

replace regioncode="360725" if regioncode=="362126" & year==1998

replace regioncode="360726" if regioncode=="362127" & year==1998

replace regioncode="360727" if regioncode=="362128" & year==1998

replace regioncode="360728" if regioncode=="362129" & year==1998

replace regioncode="360729" if regioncode=="362130" & year==1998

replace regioncode="360730" if regioncode=="362131" & year==1998

replace regioncode="360731" if regioncode=="362132" & year==1998

replace regioncode="360732" if regioncode=="362133" & year==1998

replace regioncode="360733" if regioncode=="362135" & year==1998

replace regioncode="360734" if regioncode=="362136" & year==1998

replace regioncode="360735" if regioncode=="362137" & year==1998

replace regioncode="360781" if regioncode=="362102" & year==1998

replace regioncode="360782" if regioncode=="362103" & year==1998

replace regioncode="360802" if regioncode=="362401" & year==1998

replace regioncode="360821" if regioncode=="362421" & year==1998

replace regioncode="360822" if regioncode=="362422" & year==1998

replace regioncode="360823" if regioncode=="362423" & year==1998

replace regioncode="360824" if regioncode=="362424" & year==1998

replace regioncode="360825" if regioncode=="362425" & year==1998

replace regioncode="360826" if regioncode=="362426" & year==1998

replace regioncode="360827" if regioncode=="362427" & year==1998

replace regioncode="360828" if regioncode=="362428" & year==1998

replace regioncode="360829" if regioncode=="362429" & year==1998

replace regioncode="360830" if regioncode=="362430" & year==1998

replace regioncode="360881" if regioncode=="362432" & year==1998

replace regioncode="360902" if regioncode=="362201" & year==1998

replace regioncode="360921" if regioncode=="362226" & year==1998

replace regioncode="360922" if regioncode=="362227" & year==1998

replace regioncode="360923" if regioncode=="362228" & year==1998

replace regioncode="360924" if regioncode=="362229" & year==1998

replace regioncode="360925" if regioncode=="362232" & year==1998

replace regioncode="360926" if regioncode=="362233" & year==1998

replace regioncode="360981" if regioncode=="362202" & year==1998

replace regioncode="360982" if regioncode=="362203" & year==1998

replace regioncode="360983" if regioncode=="362204" & year==1998

replace regioncode="361002" if regioncode=="362502" & year==1998

replace regioncode="361021" if regioncode=="362522" & year==1998

replace regioncode="361022" if regioncode=="362523" & year==1998

replace regioncode="361023" if regioncode=="362524" & year==1998

replace regioncode="361024" if regioncode=="362525" & year==1998

replace regioncode="361025" if regioncode=="362526" & year==1998

replace regioncode="361026" if regioncode=="362527" & year==1998

replace regioncode="361027" if regioncode=="362528" & year==1998

replace regioncode="361028" if regioncode=="362529" & year==1998

replace regioncode="361029" if regioncode=="362531" & year==1998

replace regioncode="361030" if regioncode=="362532" & year==1998

replace regioncode="361102" if regioncode=="362301" & year==1998

replace regioncode="361121" if regioncode=="362321" & year==1998

replace regioncode="361122" if regioncode=="362322" & year==1998

replace regioncode="361123" if regioncode=="362323" & year==1998

replace regioncode="361124" if regioncode=="362324" & year==1998

replace regioncode="361125" if regioncode=="362325" & year==1998

replace regioncode="361126" if regioncode=="362326" & year==1998

replace regioncode="361127" if regioncode=="362329" & year==1998

replace regioncode="361128" if regioncode=="362330" & year==1998

replace regioncode="361129" if regioncode=="362331" & year==1998

replace regioncode="361130" if regioncode=="362334" & year==1998

replace regioncode="361181" if regioncode=="362302" & year==1998

replace regioncode="370102" if regioncode=="370102" & year==1998

replace regioncode="370103" if regioncode=="370103" & year==1998

replace regioncode="370104" if regioncode=="370104" & year==1998

replace regioncode="370105" if regioncode=="370105" & year==1998

replace regioncode="370112" if regioncode=="370112" & year==1998

replace regioncode="370113" if regioncode=="370123" & year==1998

replace regioncode="370124" if regioncode=="370124" & year==1998

replace regioncode="370125" if regioncode=="370125" & year==1998

replace regioncode="370126" if regioncode=="370126" & year==1998

replace regioncode="370181" if regioncode=="370181" & year==1998

replace regioncode="370202" if regioncode=="370202" & year==1998

replace regioncode="370203" if regioncode=="370203" & year==1998

replace regioncode="370205" if regioncode=="370205" & year==1998

replace regioncode="370211" if regioncode=="370211" & year==1998

replace regioncode="370212" if regioncode=="370212" & year==1998

replace regioncode="370213" if regioncode=="370213" & year==1998

replace regioncode="370214" if regioncode=="370214" & year==1998

replace regioncode="370281" if regioncode=="370281" & year==1998

replace regioncode="370282" if regioncode=="370282" & year==1998

replace regioncode="370283" if regioncode=="370283" & year==1998

replace regioncode="370284" if regioncode=="370284" & year==1998

replace regioncode="370285" if regioncode=="370285" & year==1998

replace regioncode="370302" if regioncode=="370302" & year==1998

replace regioncode="370303" if regioncode=="370303" & year==1998

replace regioncode="370304" if regioncode=="370304" & year==1998

replace regioncode="370305" if regioncode=="370305" & year==1998

replace regioncode="370306" if regioncode=="370306" & year==1998

replace regioncode="370321" if regioncode=="370321" & year==1998

replace regioncode="370322" if regioncode=="370322" & year==1998

replace regioncode="370323" if regioncode=="370323" & year==1998

replace regioncode="370402" if regioncode=="370402" & year==1998

replace regioncode="370403" if regioncode=="370403" & year==1998

replace regioncode="370404" if regioncode=="370404" & year==1998

replace regioncode="370405" if regioncode=="370405" & year==1998

replace regioncode="370406" if regioncode=="370406" & year==1998

replace regioncode="370481" if regioncode=="370481" & year==1998

replace regioncode="370502" if regioncode=="370502" & year==1998

replace regioncode="370503" if regioncode=="370503" & year==1998

replace regioncode="370521" if regioncode=="370521" & year==1998

replace regioncode="370522" if regioncode=="370522" & year==1998

replace regioncode="370523" if regioncode=="370523" & year==1998

replace regioncode="370602" if regioncode=="370602" & year==1998

replace regioncode="370611" if regioncode=="370611" & year==1998

replace regioncode="370612" if regioncode=="370612" & year==1998

replace regioncode="370613" if regioncode=="370613" & year==1998

replace regioncode="370634" if regioncode=="370634" & year==1998

replace regioncode="370681" if regioncode=="370681" & year==1998

replace regioncode="370682" if regioncode=="370682" & year==1998

replace regioncode="370683" if regioncode=="370683" & year==1998

replace regioncode="370684" if regioncode=="370684" & year==1998

replace regioncode="370685" if regioncode=="370685" & year==1998

replace regioncode="370686" if regioncode=="370686" & year==1998

replace regioncode="370687" if regioncode=="370687" & year==1998

replace regioncode="370702" if regioncode=="370702" & year==1998

replace regioncode="370703" if regioncode=="370703" & year==1998

replace regioncode="370704" if regioncode=="370704" & year==1998

replace regioncode="370705" if regioncode=="370705" & year==1998

replace regioncode="370724" if regioncode=="370724" & year==1998

replace regioncode="370725" if regioncode=="370725" & year==1998

replace regioncode="370781" if regioncode=="370781" & year==1998

replace regioncode="370782" if regioncode=="370782" & year==1998

replace regioncode="370783" if regioncode=="370783" & year==1998

replace regioncode="370784" if regioncode=="370784" & year==1998

replace regioncode="370785" if regioncode=="370785" & year==1998

replace regioncode="370786" if regioncode=="370786" & year==1998

replace regioncode="370802" if regioncode=="370802" & year==1998

replace regioncode="370811" if regioncode=="370811" & year==1998

replace regioncode="370826" if regioncode=="370826" & year==1998

replace regioncode="370827" if regioncode=="370827" & year==1998

replace regioncode="370828" if regioncode=="370828" & year==1998

replace regioncode="370829" if regioncode=="370829" & year==1998

replace regioncode="370830" if regioncode=="370830" & year==1998

replace regioncode="370831" if regioncode=="370831" & year==1998

replace regioncode="370832" if regioncode=="370832" & year==1998

replace regioncode="370881" if regioncode=="370881" & year==1998

replace regioncode="370882" if regioncode=="370882" & year==1998

replace regioncode="370883" if regioncode=="370883" & year==1998

replace regioncode="370902" if regioncode=="370902" & year==1998

replace regioncode="370903" if regioncode=="370911" & year==1998

replace regioncode="370921" if regioncode=="370921" & year==1998

replace regioncode="370923" if regioncode=="370923" & year==1998

replace regioncode="370982" if regioncode=="370982" & year==1998

replace regioncode="370983" if regioncode=="370983" & year==1998

replace regioncode="371002" if regioncode=="371002" & year==1998

replace regioncode="371081" if regioncode=="371081" & year==1998

replace regioncode="371082" if regioncode=="371082" & year==1998

replace regioncode="371083" if regioncode=="371083" & year==1998

replace regioncode="371102" if regioncode=="371102" & year==1998

replace regioncode="371121" if regioncode=="371121" & year==1998

replace regioncode="371122" if regioncode=="371122" & year==1998

replace regioncode="371202" if regioncode=="371202" & year==1998

replace regioncode="371203" if regioncode=="371203" & year==1998

replace regioncode="371302" if regioncode=="371302" & year==1998

replace regioncode="371311" if regioncode=="371311" & year==1998

replace regioncode="371312" if regioncode=="371312" & year==1998

replace regioncode="371321" if regioncode=="371321" & year==1998

replace regioncode="371322" if regioncode=="371322" & year==1998

replace regioncode="371323" if regioncode=="371323" & year==1998

replace regioncode="371324" if regioncode=="371324" & year==1998

replace regioncode="371325" if regioncode=="371325" & year==1998

replace regioncode="371326" if regioncode=="371326" & year==1998

replace regioncode="371327" if regioncode=="371327" & year==1998

replace regioncode="371328" if regioncode=="371328" & year==1998

replace regioncode="371329" if regioncode=="371329" & year==1998

replace regioncode="371402" if regioncode=="371402" & year==1998

replace regioncode="371421" if regioncode=="371421" & year==1998

replace regioncode="371422" if regioncode=="371422" & year==1998

replace regioncode="371423" if regioncode=="371423" & year==1998

replace regioncode="371424" if regioncode=="371424" & year==1998

replace regioncode="371425" if regioncode=="371425" & year==1998

replace regioncode="371426" if regioncode=="371426" & year==1998

replace regioncode="371427" if regioncode=="371427" & year==1998

replace regioncode="371428" if regioncode=="371428" & year==1998

replace regioncode="371481" if regioncode=="371481" & year==1998

replace regioncode="371482" if regioncode=="371482" & year==1998

replace regioncode="371502" if regioncode=="371502" & year==1998

replace regioncode="371521" if regioncode=="371521" & year==1998

replace regioncode="371522" if regioncode=="371522" & year==1998

replace regioncode="371523" if regioncode=="371523" & year==1998

replace regioncode="371524" if regioncode=="371524" & year==1998

replace regioncode="371525" if regioncode=="371525" & year==1998

replace regioncode="371526" if regioncode=="371526" & year==1998

replace regioncode="371581" if regioncode=="371581" & year==1998

replace regioncode="371602" if regioncode=="372301" & year==1998

replace regioncode="371621" if regioncode=="372321" & year==1998

replace regioncode="371622" if regioncode=="372323" & year==1998

replace regioncode="371623" if regioncode=="372324" & year==1998

replace regioncode="371624" if regioncode=="372325" & year==1998

replace regioncode="371625" if regioncode=="372328" & year==1998

replace regioncode="371626" if regioncode=="372330" & year==1998

replace regioncode="371702" if regioncode=="372901" & year==1998

replace regioncode="371721" if regioncode=="372922" & year==1998

replace regioncode="371722" if regioncode=="372925" & year==1998

replace regioncode="371723" if regioncode=="372924" & year==1998

replace regioncode="371724" if regioncode=="372926" & year==1998

replace regioncode="371725" if regioncode=="372928" & year==1998

replace regioncode="371726" if regioncode=="372929" & year==1998

replace regioncode="371727" if regioncode=="372923" & year==1998

replace regioncode="371728" if regioncode=="372930" & year==1998

replace regioncode="410102" if regioncode=="410102" & year==1998

replace regioncode="410103" if regioncode=="410103" & year==1998

replace regioncode="410104" if regioncode=="410104" & year==1998

replace regioncode="410105" if regioncode=="410105" & year==1998

replace regioncode="410106" if regioncode=="410106" & year==1998

replace regioncode="410108" if regioncode=="410108" & year==1998

replace regioncode="410122" if regioncode=="410122" & year==1998

replace regioncode="410181" if regioncode=="410181" & year==1998

replace regioncode="410182" if regioncode=="410182" & year==1998

replace regioncode="410183" if regioncode=="410183" & year==1998

replace regioncode="410184" if regioncode=="410184" & year==1998

replace regioncode="410185" if regioncode=="410185" & year==1998

replace regioncode="410202" if regioncode=="410202" & year==1998

replace regioncode="410203" if regioncode=="410203" & year==1998

replace regioncode="410204" if regioncode=="410204" & year==1998

replace regioncode="410205" if regioncode=="410205" & year==1998

replace regioncode="410211" if regioncode=="410211" & year==1998

replace regioncode="410221" if regioncode=="410221" & year==1998

replace regioncode="410222" if regioncode=="410222" & year==1998

replace regioncode="410223" if regioncode=="410223" & year==1998

replace regioncode="410224" if regioncode=="410224" & year==1998

replace regioncode="410225" if regioncode=="410225" & year==1998

replace regioncode="410302" if regioncode=="410302" & year==1998

replace regioncode="410303" if regioncode=="410303" & year==1998

replace regioncode="410304" if regioncode=="410304" & year==1998

replace regioncode="410305" if regioncode=="410305" & year==1998

replace regioncode="410306" if regioncode=="410306" & year==1998

replace regioncode="410307" if regioncode=="410311" & year==1998

replace regioncode="410322" if regioncode=="410322" & year==1998

replace regioncode="410323" if regioncode=="410323" & year==1998

replace regioncode="410324" if regioncode=="410324" & year==1998

replace regioncode="410325" if regioncode=="410325" & year==1998

replace regioncode="410326" if regioncode=="410326" & year==1998

replace regioncode="410327" if regioncode=="410327" & year==1998

replace regioncode="410328" if regioncode=="410328" & year==1998

replace regioncode="410329" if regioncode=="410329" & year==1998

replace regioncode="410381" if regioncode=="410381" & year==1998

replace regioncode="410402" if regioncode=="410402" & year==1998

replace regioncode="410403" if regioncode=="410403" & year==1998

replace regioncode="410404" if regioncode=="410404" & year==1998

replace regioncode="410411" if regioncode=="410411" & year==1998

replace regioncode="410421" if regioncode=="410421" & year==1998

replace regioncode="410422" if regioncode=="410422" & year==1998

replace regioncode="410423" if regioncode=="410423" & year==1998

replace regioncode="410425" if regioncode=="410425" & year==1998

replace regioncode="410481" if regioncode=="410481" & year==1998

replace regioncode="410482" if regioncode=="410482" & year==1998

replace regioncode="410502" if regioncode=="410502" & year==1998

replace regioncode="410503" if regioncode=="410503" & year==1998

replace regioncode="410504" if regioncode=="410504" & year==1998

replace regioncode="410511" if regioncode=="410511" & year==1998

replace regioncode="410522" if regioncode=="410522" & year==1998

replace regioncode="410523" if regioncode=="410523" & year==1998

replace regioncode="410526" if regioncode=="410526" & year==1998

replace regioncode="410527" if regioncode=="410527" & year==1998

replace regioncode="410581" if regioncode=="410581" & year==1998

replace regioncode="410602" if regioncode=="410602" & year==1998

replace regioncode="410603" if regioncode=="410603" & year==1998

replace regioncode="410611" if regioncode=="410611" & year==1998

replace regioncode="410621" if regioncode=="410621" & year==1998

replace regioncode="410622" if regioncode=="410622" & year==1998

replace regioncode="410702" if regioncode=="410702" & year==1998

replace regioncode="410703" if regioncode=="410703" & year==1998

replace regioncode="410704" if regioncode=="410704" & year==1998

replace regioncode="410711" if regioncode=="410711" & year==1998

replace regioncode="410721" if regioncode=="410721" & year==1998

replace regioncode="410724" if regioncode=="410724" & year==1998

replace regioncode="410725" if regioncode=="410725" & year==1998

replace regioncode="410726" if regioncode=="410726" & year==1998

replace regioncode="410727" if regioncode=="410727" & year==1998

replace regioncode="410728" if regioncode=="410728" & year==1998

replace regioncode="410781" if regioncode=="410781" & year==1998

replace regioncode="410782" if regioncode=="410782" & year==1998

replace regioncode="410802" if regioncode=="410802" & year==1998

replace regioncode="410803" if regioncode=="410803" & year==1998

replace regioncode="410804" if regioncode=="410804" & year==1998

replace regioncode="410811" if regioncode=="410811" & year==1998

replace regioncode="410821" if regioncode=="410821" & year==1998

replace regioncode="410822" if regioncode=="410822" & year==1998

replace regioncode="410823" if regioncode=="410823" & year==1998

replace regioncode="410825" if regioncode=="410825" & year==1998

replace regioncode="410881" if regioncode=="410881" & year==1998

replace regioncode="410882" if regioncode=="410882" & year==1998

replace regioncode="410883" if regioncode=="410883" & year==1998

replace regioncode="410902" if regioncode=="410902" & year==1998

replace regioncode="410922" if regioncode=="410922" & year==1998

replace regioncode="410923" if regioncode=="410923" & year==1998

replace regioncode="410926" if regioncode=="410926" & year==1998

replace regioncode="410927" if regioncode=="410927" & year==1998

replace regioncode="410928" if regioncode=="410928" & year==1998

replace regioncode="411002" if regioncode=="411002" & year==1998

replace regioncode="411023" if regioncode=="411023" & year==1998

replace regioncode="411024" if regioncode=="411024" & year==1998

replace regioncode="411025" if regioncode=="411025" & year==1998

replace regioncode="411081" if regioncode=="411081" & year==1998

replace regioncode="411082" if regioncode=="411082" & year==1998

replace regioncode="411102" if regioncode=="411102" & year==1998

replace regioncode="411123" if regioncode=="411123" & year==1998

replace regioncode="411121" if regioncode=="411121" & year==1998

replace regioncode="411122" if regioncode=="411122" & year==1998

replace regioncode="411202" if regioncode=="411202" & year==1998

replace regioncode="411221" if regioncode=="411221" & year==1998

replace regioncode="411222" if regioncode=="411222" & year==1998

replace regioncode="411224" if regioncode=="411224" & year==1998

replace regioncode="411281" if regioncode=="411281" & year==1998

replace regioncode="411282" if regioncode=="411282" & year==1998

replace regioncode="411302" if regioncode=="411302" & year==1998

replace regioncode="411303" if regioncode=="411303" & year==1998

replace regioncode="411321" if regioncode=="411321" & year==1998

replace regioncode="411322" if regioncode=="411322" & year==1998

replace regioncode="411323" if regioncode=="411323" & year==1998

replace regioncode="411324" if regioncode=="411324" & year==1998

replace regioncode="411325" if regioncode=="411325" & year==1998

replace regioncode="411326" if regioncode=="411326" & year==1998

replace regioncode="411327" if regioncode=="411327" & year==1998

replace regioncode="411328" if regioncode=="411328" & year==1998

replace regioncode="411329" if regioncode=="411329" & year==1998

replace regioncode="411330" if regioncode=="411330" & year==1998

replace regioncode="411381" if regioncode=="411381" & year==1998

replace regioncode="411402" if regioncode=="411402" & year==1998

replace regioncode="411403" if regioncode=="411403" & year==1998

replace regioncode="411421" if regioncode=="411421" & year==1998

replace regioncode="411422" if regioncode=="411422" & year==1998

replace regioncode="411423" if regioncode=="411423" & year==1998

replace regioncode="411424" if regioncode=="411424" & year==1998

replace regioncode="411425" if regioncode=="411425" & year==1998

replace regioncode="411426" if regioncode=="411426" & year==1998

replace regioncode="411481" if regioncode=="411481" & year==1998

replace regioncode="411502" if regioncode=="411502" & year==1998

replace regioncode="411503" if regioncode=="411503" & year==1998

replace regioncode="411521" if regioncode=="411521" & year==1998

replace regioncode="411522" if regioncode=="411522" & year==1998

replace regioncode="411523" if regioncode=="411523" & year==1998

replace regioncode="411524" if regioncode=="411524" & year==1998

replace regioncode="411525" if regioncode=="411525" & year==1998

replace regioncode="411526" if regioncode=="411526" & year==1998

replace regioncode="411527" if regioncode=="411527" & year==1998

replace regioncode="411528" if regioncode=="411528" & year==1998

replace regioncode="411602" if regioncode=="412701" & year==1998

replace regioncode="411621" if regioncode=="412721" & year==1998

replace regioncode="411622" if regioncode=="412722" & year==1998

replace regioncode="411623" if regioncode=="412723" & year==1998

replace regioncode="411624" if regioncode=="412728" & year==1998

replace regioncode="411625" if regioncode=="412726" & year==1998

replace regioncode="411626" if regioncode=="412727" & year==1998

replace regioncode="411627" if regioncode=="412724" & year==1998

replace regioncode="411628" if regioncode=="412725" & year==1998

replace regioncode="411681" if regioncode=="412702" & year==1998

replace regioncode="411702" if regioncode=="412801" & year==1998

replace regioncode="411721" if regioncode=="412824" & year==1998

replace regioncode="411722" if regioncode=="412825" & year==1998

replace regioncode="411723" if regioncode=="412827" & year==1998

replace regioncode="411724" if regioncode=="412829" & year==1998

replace regioncode="411725" if regioncode=="412821" & year==1998

replace regioncode="411726" if regioncode=="412822" & year==1998

replace regioncode="411727" if regioncode=="412826" & year==1998

replace regioncode="411728" if regioncode=="412823" & year==1998

replace regioncode="411729" if regioncode=="412828" & year==1998

replace regioncode="420102" if regioncode=="420102" & year==1998

replace regioncode="420103" if regioncode=="420103" & year==1998

replace regioncode="420104" if regioncode=="420104" & year==1998

replace regioncode="420105" if regioncode=="420105" & year==1998

replace regioncode="420106" if regioncode=="420106" & year==1998

replace regioncode="420107" if regioncode=="420107" & year==1998

replace regioncode="420111" if regioncode=="420111" & year==1998

replace regioncode="420112" if regioncode=="420112" & year==1998

replace regioncode="420113" if regioncode=="420113" & year==1998

replace regioncode="420114" if regioncode=="420114" & year==1998

replace regioncode="420115" if regioncode=="420115" & year==1998

replace regioncode="420116" if regioncode=="420123" & year==1998

replace regioncode="420117" if regioncode=="420124" & year==1998

replace regioncode="420202" if regioncode=="420202" & year==1998

replace regioncode="420203" if regioncode=="420203" & year==1998

replace regioncode="420204" if regioncode=="420204" & year==1998

replace regioncode="420205" if regioncode=="420205" & year==1998

replace regioncode="420222" if regioncode=="420221" & year==1998

replace regioncode="420281" if regioncode=="420281" & year==1998

replace regioncode="420302" if regioncode=="420302" & year==1998

replace regioncode="420303" if regioncode=="420303" & year==1998

replace regioncode="420321" if regioncode=="420321" & year==1998

replace regioncode="420322" if regioncode=="420322" & year==1998

replace regioncode="420323" if regioncode=="420323" & year==1998

replace regioncode="420324" if regioncode=="420324" & year==1998

replace regioncode="420325" if regioncode=="420325" & year==1998

replace regioncode="420381" if regioncode=="420381" & year==1998

replace regioncode="420502" if regioncode=="420502" & year==1998

replace regioncode="420503" if regioncode=="420503" & year==1998

replace regioncode="420504" if regioncode=="420504" & year==1998

replace regioncode="420505" if regioncode=="420505" & year==1998

replace regioncode="420506" if regioncode=="420521" & year==1998

replace regioncode="420525" if regioncode=="420525" & year==1998

replace regioncode="420526" if regioncode=="420526" & year==1998

replace regioncode="420527" if regioncode=="420527" & year==1998

replace regioncode="420528" if regioncode=="420528" & year==1998

replace regioncode="420529" if regioncode=="420529" & year==1998

replace regioncode="420581" if regioncode=="420581" & year==1998

replace regioncode="420582" if regioncode=="420582" & year==1998

replace regioncode="420583" if regioncode=="420583" & year==1998

replace regioncode="420602" if regioncode=="420602" & year==1998

replace regioncode="420606" if regioncode=="420606" & year==1998

replace regioncode="420607" if regioncode=="420607" & year==1998

replace regioncode="420624" if regioncode=="420624" & year==1998

replace regioncode="420625" if regioncode=="420625" & year==1998

replace regioncode="420626" if regioncode=="420626" & year==1998

replace regioncode="420682" if regioncode=="420682" & year==1998

replace regioncode="420683" if regioncode=="420683" & year==1998

replace regioncode="420684" if regioncode=="420684" & year==1998

replace regioncode="420702" if regioncode=="420702" & year==1998

replace regioncode="420703" if regioncode=="420703" & year==1998

replace regioncode="420704" if regioncode=="420704" & year==1998

replace regioncode="420802" if regioncode=="420802" & year==1998

replace regioncode="420804" if regioncode=="420804" & year==1998

replace regioncode="420821" if regioncode=="420821" & year==1998

replace regioncode="420822" if regioncode=="420803" & year==1998

replace regioncode="420881" if regioncode=="420881" & year==1998

replace regioncode="420902" if regioncode=="420902" & year==1998

replace regioncode="420921" if regioncode=="420921" & year==1998

replace regioncode="420922" if regioncode=="420922" & year==1998

replace regioncode="420923" if regioncode=="420923" & year==1998

replace regioncode="420981" if regioncode=="420981" & year==1998

replace regioncode="420982" if regioncode=="420982" & year==1998

replace regioncode="421381" if regioncode=="420983" & year==1998

replace regioncode="420984" if regioncode=="420924" & year==1998

replace regioncode="421002" if regioncode=="421002" & year==1998

replace regioncode="421003" if regioncode=="421003" & year==1998

replace regioncode="421022" if regioncode=="421022" & year==1998

replace regioncode="421023" if regioncode=="421023" & year==1998

replace regioncode="421024" if regioncode=="421004" & year==1998

replace regioncode="421081" if regioncode=="421081" & year==1998

replace regioncode="421083" if regioncode=="421083" & year==1998

replace regioncode="421087" if regioncode=="421087" & year==1998

replace regioncode="421102" if regioncode=="421102" & year==1998

replace regioncode="421121" if regioncode=="421121" & year==1998

replace regioncode="421122" if regioncode=="421122" & year==1998

replace regioncode="421123" if regioncode=="421123" & year==1998

replace regioncode="421124" if regioncode=="421124" & year==1998

replace regioncode="421125" if regioncode=="421125" & year==1998

replace regioncode="421126" if regioncode=="421126" & year==1998

replace regioncode="421127" if regioncode=="421127" & year==1998

replace regioncode="421181" if regioncode=="421181" & year==1998

replace regioncode="421182" if regioncode=="421182" & year==1998

replace regioncode="421202" if regioncode=="422301" & year==1998

replace regioncode="421221" if regioncode=="422322" & year==1998

replace regioncode="421222" if regioncode=="422324" & year==1998

replace regioncode="421223" if regioncode=="422325" & year==1998

replace regioncode="421224" if regioncode=="422326" & year==1998

replace regioncode="421281" if regioncode=="422302" & year==1998

replace regioncode="421302" if regioncode=="429001" & year==1998

replace regioncode="422801" if regioncode=="422801" & year==1998

replace regioncode="422802" if regioncode=="422802" & year==1998

replace regioncode="422822" if regioncode=="422822" & year==1998

replace regioncode="422823" if regioncode=="422823" & year==1998

replace regioncode="422825" if regioncode=="422825" & year==1998

replace regioncode="422826" if regioncode=="422826" & year==1998

replace regioncode="422827" if regioncode=="422827" & year==1998

replace regioncode="422828" if regioncode=="422828" & year==1998

replace regioncode="429004" if regioncode=="429004" & year==1998

replace regioncode="429005" if regioncode=="429005" & year==1998

replace regioncode="429006" if regioncode=="429006" & year==1998

replace regioncode="429021" if regioncode=="429021" & year==1998

replace regioncode="430102" if regioncode=="430102" & year==1998

replace regioncode="430103" if regioncode=="430103" & year==1998

replace regioncode="430104" if regioncode=="430104" & year==1998

replace regioncode="430105" if regioncode=="430105" & year==1998

replace regioncode="430111" if regioncode=="430111" & year==1998

replace regioncode="430121" if regioncode=="430121" & year==1998

replace regioncode="430122" if regioncode=="430122" & year==1998

replace regioncode="430124" if regioncode=="430124" & year==1998

replace regioncode="430181" if regioncode=="430181" & year==1998

replace regioncode="430202" if regioncode=="430202" & year==1998

replace regioncode="430203" if regioncode=="430203" & year==1998

replace regioncode="430204" if regioncode=="430204" & year==1998

replace regioncode="430211" if regioncode=="430211" & year==1998

replace regioncode="430221" if regioncode=="430221" & year==1998

replace regioncode="430223" if regioncode=="430223" & year==1998

replace regioncode="430224" if regioncode=="430224" & year==1998

replace regioncode="430225" if regioncode=="430225" & year==1998

replace regioncode="430281" if regioncode=="430281" & year==1998

replace regioncode="430302" if regioncode=="430302" & year==1998

replace regioncode="430304" if regioncode=="430304" & year==1998

replace regioncode="430321" if regioncode=="430321" & year==1998

replace regioncode="430381" if regioncode=="430381" & year==1998

replace regioncode="430382" if regioncode=="430382" & year==1998

replace regioncode="430405" if regioncode=="430402" & year==1998

replace regioncode="430406" if regioncode=="430403" & year==1998

replace regioncode="430407" if regioncode=="430404" & year==1998

replace regioncode="430408" if regioncode=="430411" & year==1998

replace regioncode="430412" if regioncode=="430412" & year==1998

replace regioncode="430421" if regioncode=="430421" & year==1998

replace regioncode="430422" if regioncode=="430422" & year==1998

replace regioncode="430423" if regioncode=="430423" & year==1998

replace regioncode="430424" if regioncode=="430424" & year==1998

replace regioncode="430426" if regioncode=="430426" & year==1998

replace regioncode="430481" if regioncode=="430481" & year==1998

replace regioncode="430482" if regioncode=="430482" & year==1998

replace regioncode="430502" if regioncode=="430502" & year==1998

replace regioncode="430503" if regioncode=="430503" & year==1998

replace regioncode="430511" if regioncode=="430511" & year==1998

replace regioncode="430521" if regioncode=="430521" & year==1998

replace regioncode="430522" if regioncode=="430522" & year==1998

replace regioncode="430523" if regioncode=="430523" & year==1998

replace regioncode="430524" if regioncode=="430524" & year==1998

replace regioncode="430525" if regioncode=="430525" & year==1998

replace regioncode="430527" if regioncode=="430527" & year==1998

replace regioncode="430528" if regioncode=="430528" & year==1998

replace regioncode="430529" if regioncode=="430529" & year==1998

replace regioncode="430581" if regioncode=="430581" & year==1998

replace regioncode="430602" if regioncode=="430602" & year==1998

replace regioncode="430603" if regioncode=="430603" & year==1998

replace regioncode="430611" if regioncode=="430611" & year==1998

replace regioncode="430621" if regioncode=="430621" & year==1998

replace regioncode="430623" if regioncode=="430623" & year==1998

replace regioncode="430624" if regioncode=="430624" & year==1998

replace regioncode="430626" if regioncode=="430626" & year==1998

replace regioncode="430681" if regioncode=="430681" & year==1998

replace regioncode="430682" if regioncode=="430682" & year==1998

replace regioncode="430702" if regioncode=="430702" & year==1998

replace regioncode="430703" if regioncode=="430703" & year==1998

replace regioncode="430721" if regioncode=="430721" & year==1998

replace regioncode="430722" if regioncode=="430722" & year==1998

replace regioncode="430723" if regioncode=="430723" & year==1998

replace regioncode="430724" if regioncode=="430724" & year==1998

replace regioncode="430725" if regioncode=="430725" & year==1998

replace regioncode="430726" if regioncode=="430726" & year==1998

replace regioncode="430781" if regioncode=="430781" & year==1998

replace regioncode="430802" if regioncode=="430802" & year==1998

replace regioncode="430811" if regioncode=="430811" & year==1998

replace regioncode="430821" if regioncode=="430821" & year==1998

replace regioncode="430822" if regioncode=="430822" & year==1998

replace regioncode="430902" if regioncode=="430902" & year==1998

replace regioncode="430903" if regioncode=="430903" & year==1998

replace regioncode="430921" if regioncode=="430921" & year==1998

replace regioncode="430922" if regioncode=="430922" & year==1998

replace regioncode="430923" if regioncode=="430923" & year==1998

replace regioncode="430981" if regioncode=="430981" & year==1998

replace regioncode="431002" if regioncode=="431002" & year==1998

replace regioncode="431003" if regioncode=="431003" & year==1998

replace regioncode="431021" if regioncode=="431021" & year==1998

replace regioncode="431022" if regioncode=="431022" & year==1998

replace regioncode="431023" if regioncode=="431023" & year==1998

replace regioncode="431024" if regioncode=="431024" & year==1998

replace regioncode="431025" if regioncode=="431025" & year==1998

replace regioncode="431026" if regioncode=="431026" & year==1998

replace regioncode="431027" if regioncode=="431027" & year==1998

replace regioncode="431028" if regioncode=="431028" & year==1998

replace regioncode="431081" if regioncode=="431081" & year==1998

replace regioncode="431102" if regioncode=="431102" & year==1998

replace regioncode="431103" if regioncode=="431103" & year==1998

replace regioncode="431121" if regioncode=="431121" & year==1998

replace regioncode="431122" if regioncode=="431122" & year==1998

replace regioncode="431123" if regioncode=="431123" & year==1998

replace regioncode="431124" if regioncode=="431124" & year==1998

replace regioncode="431125" if regioncode=="431125" & year==1998

replace regioncode="431126" if regioncode=="431126" & year==1998

replace regioncode="431127" if regioncode=="431127" & year==1998

replace regioncode="431128" if regioncode=="431128" & year==1998

replace regioncode="431129" if regioncode=="431129" & year==1998

replace regioncode="431202" if regioncode=="431202" & year==1998

replace regioncode="431221" if regioncode=="431221" & year==1998

replace regioncode="431222" if regioncode=="431222" & year==1998

replace regioncode="431223" if regioncode=="431223" & year==1998

replace regioncode="431224" if regioncode=="431224" & year==1998

replace regioncode="431225" if regioncode=="431225" & year==1998

replace regioncode="431226" if regioncode=="431226" & year==1998

replace regioncode="431227" if regioncode=="431227" & year==1998

replace regioncode="431228" if regioncode=="431228" & year==1998

replace regioncode="431229" if regioncode=="431229" & year==1998

replace regioncode="431230" if regioncode=="431230" & year==1998

replace regioncode="431281" if regioncode=="431281" & year==1998

replace regioncode="431302" if regioncode=="432501" & year==1998

replace regioncode="431321" if regioncode=="432522" & year==1998

replace regioncode="431322" if regioncode=="432524" & year==1998

replace regioncode="431381" if regioncode=="432502" & year==1998

replace regioncode="431382" if regioncode=="432503" & year==1998

replace regioncode="433101" if regioncode=="433101" & year==1998

replace regioncode="433122" if regioncode=="433122" & year==1998

replace regioncode="433123" if regioncode=="433123" & year==1998

replace regioncode="433124" if regioncode=="433124" & year==1998

replace regioncode="433125" if regioncode=="433125" & year==1998

replace regioncode="433126" if regioncode=="433126" & year==1998

replace regioncode="433127" if regioncode=="433127" & year==1998

replace regioncode="433130" if regioncode=="433130" & year==1998

replace regioncode="440102" if regioncode=="440102" & year==1998

replace regioncode="440103" if regioncode=="440103" & year==1998

replace regioncode="440104" if regioncode=="440104" & year==1998

replace regioncode="440105" if regioncode=="440105" & year==1998

replace regioncode="440106" if regioncode=="440106" & year==1998

replace regioncode="440107" if regioncode=="440107" & year==1998

replace regioncode="440111" if regioncode=="440111" & year==1998

replace regioncode="440112" if regioncode=="440112" & year==1998

replace regioncode="440113" if regioncode=="440181" & year==1998

replace regioncode="440114" if regioncode=="440182" & year==1998

replace regioncode="440183" if regioncode=="440183" & year==1998

replace regioncode="440184" if regioncode=="440184" & year==1998

replace regioncode="440203" if regioncode=="440203" & year==1998

replace regioncode="440204" if regioncode=="440204" & year==1998

replace regioncode="440221" if regioncode=="440221" & year==1998

replace regioncode="440222" if regioncode=="440222" & year==1998

replace regioncode="440224" if regioncode=="440224" & year==1998

replace regioncode="440229" if regioncode=="440229" & year==1998

replace regioncode="440232" if regioncode=="440232" & year==1998

replace regioncode="440233" if regioncode=="440233" & year==1998

replace regioncode="440281" if regioncode=="440281" & year==1998

replace regioncode="440282" if regioncode=="440282" & year==1998

replace regioncode="440303" if regioncode=="440303" & year==1998

replace regioncode="440304" if regioncode=="440304" & year==1998

replace regioncode="440305" if regioncode=="440305" & year==1998

replace regioncode="440306" if regioncode=="440306" & year==1998

replace regioncode="440307" if regioncode=="440307" & year==1998

replace regioncode="440308" if regioncode=="440308" & year==1998

replace regioncode="440402" if regioncode=="440402" & year==1998

replace regioncode="440403" if regioncode=="440421" & year==1998

replace regioncode="440404" if regioncode=="440404" & year==1998

replace regioncode="440507" if regioncode=="440507" & year==1998

replace regioncode="440511" if regioncode=="440509" & year==1998

replace regioncode="440512" if regioncode=="440510" & year==1998

replace regioncode="440514" if regioncode=="440582" & year==1998

replace regioncode="440515" if regioncode=="440583" & year==1998

replace regioncode="440523" if regioncode=="440523" & year==1998

replace regioncode="440604" if regioncode=="440603" & year==1998

replace regioncode="440605" if regioncode=="440682" & year==1998

replace regioncode="440606" if regioncode=="440681" & year==1998

replace regioncode="440607" if regioncode=="440683" & year==1998

replace regioncode="440608" if regioncode=="440684" & year==1998

replace regioncode="440703" if regioncode=="440703" & year==1998

replace regioncode="440704" if regioncode=="440704" & year==1998

replace regioncode="440781" if regioncode=="440781" & year==1998

replace regioncode="440705" if regioncode=="440782" & year==1998

replace regioncode="440783" if regioncode=="440783" & year==1998

replace regioncode="440784" if regioncode=="440784" & year==1998

replace regioncode="440785" if regioncode=="440785" & year==1998

replace regioncode="440802" if regioncode=="440802" & year==1998

replace regioncode="440803" if regioncode=="440803" & year==1998

replace regioncode="440804" if regioncode=="440804" & year==1998

replace regioncode="440811" if regioncode=="440811" & year==1998

replace regioncode="440823" if regioncode=="440823" & year==1998

replace regioncode="440825" if regioncode=="440825" & year==1998

replace regioncode="440881" if regioncode=="440881" & year==1998

replace regioncode="440882" if regioncode=="440882" & year==1998

replace regioncode="440883" if regioncode=="440883" & year==1998

replace regioncode="440902" if regioncode=="440902" & year==1998

replace regioncode="440903" if regioncode=="440903" & year==1998

replace regioncode="440923" if regioncode=="440923" & year==1998

replace regioncode="440981" if regioncode=="440981" & year==1998

replace regioncode="440982" if regioncode=="440982" & year==1998

replace regioncode="440983" if regioncode=="440983" & year==1998

replace regioncode="441202" if regioncode=="441202" & year==1998

replace regioncode="441203" if regioncode=="441203" & year==1998

replace regioncode="441223" if regioncode=="441223" & year==1998

replace regioncode="441224" if regioncode=="441224" & year==1998

replace regioncode="441225" if regioncode=="441225" & year==1998

replace regioncode="441226" if regioncode=="441226" & year==1998

replace regioncode="441283" if regioncode=="441283" & year==1998

replace regioncode="441284" if regioncode=="441284" & year==1998

replace regioncode="441302" if regioncode=="441302" & year==1998

replace regioncode="441303" if regioncode=="441381" & year==1998

replace regioncode="441322" if regioncode=="441322" & year==1998

replace regioncode="441323" if regioncode=="441323" & year==1998

replace regioncode="441324" if regioncode=="441324" & year==1998

replace regioncode="441402" if regioncode=="441402" & year==1998

replace regioncode="441421" if regioncode=="441421" & year==1998

replace regioncode="441422" if regioncode=="441422" & year==1998

replace regioncode="441423" if regioncode=="441423" & year==1998

replace regioncode="441424" if regioncode=="441424" & year==1998

replace regioncode="441426" if regioncode=="441426" & year==1998

replace regioncode="441427" if regioncode=="441427" & year==1998

replace regioncode="441481" if regioncode=="441481" & year==1998

replace regioncode="441502" if regioncode=="441502" & year==1998

replace regioncode="441521" if regioncode=="441521" & year==1998

replace regioncode="441523" if regioncode=="441523" & year==1998

replace regioncode="441581" if regioncode=="441581" & year==1998

replace regioncode="441602" if regioncode=="441602" & year==1998

replace regioncode="441621" if regioncode=="441621" & year==1998

replace regioncode="441622" if regioncode=="441622" & year==1998

replace regioncode="441623" if regioncode=="441623" & year==1998

replace regioncode="441624" if regioncode=="441624" & year==1998

replace regioncode="441625" if regioncode=="441625" & year==1998

replace regioncode="441702" if regioncode=="441702" & year==1998

replace regioncode="441721" if regioncode=="441721" & year==1998

replace regioncode="441723" if regioncode=="441723" & year==1998

replace regioncode="441781" if regioncode=="441781" & year==1998

replace regioncode="441802" if regioncode=="441802" & year==1998

replace regioncode="441821" if regioncode=="441821" & year==1998

replace regioncode="441823" if regioncode=="441823" & year==1998

replace regioncode="441825" if regioncode=="441825" & year==1998

replace regioncode="441826" if regioncode=="441826" & year==1998

replace regioncode="441827" if regioncode=="441827" & year==1998

replace regioncode="441881" if regioncode=="441881" & year==1998

replace regioncode="441882" if regioncode=="441882" & year==1998

replace regioncode="441900" if regioncode=="441900" & year==1998

replace regioncode="442000" if regioncode=="442000" & year==1998

replace regioncode="445102" if regioncode=="445102" & year==1998

replace regioncode="445121" if regioncode=="445121" & year==1998

replace regioncode="445122" if regioncode=="445122" & year==1998

replace regioncode="445202" if regioncode=="445202" & year==1998

replace regioncode="445221" if regioncode=="445221" & year==1998

replace regioncode="445222" if regioncode=="445222" & year==1998

replace regioncode="445224" if regioncode=="445224" & year==1998

replace regioncode="445281" if regioncode=="445281" & year==1998

replace regioncode="445302" if regioncode=="445302" & year==1998

replace regioncode="445321" if regioncode=="445321" & year==1998

replace regioncode="445322" if regioncode=="445322" & year==1998

replace regioncode="445323" if regioncode=="445323" & year==1998

replace regioncode="445381" if regioncode=="445381" & year==1998

replace regioncode="450102" if regioncode=="450102" & year==1998

replace regioncode="450103" if regioncode=="450103" & year==1998

replace regioncode="450104" if regioncode=="450104" & year==1998

replace regioncode="450105" if regioncode=="450105" & year==1998

replace regioncode="450106" if regioncode=="450106" & year==1998

replace regioncode="450121" if regioncode=="450121" & year==1998

replace regioncode="450122" if regioncode=="450122" & year==1998

replace regioncode="450123" if regioncode=="452126" & year==1998

replace regioncode="450124" if regioncode=="452127" & year==1998

replace regioncode="450125" if regioncode=="452124" & year==1998

replace regioncode="450126" if regioncode=="452123" & year==1998

replace regioncode="450127" if regioncode=="452122" & year==1998

replace regioncode="450202" if regioncode=="450202" & year==1998

replace regioncode="450203" if regioncode=="450203" & year==1998

replace regioncode="450204" if regioncode=="450204" & year==1998

replace regioncode="450205" if regioncode=="450205" & year==1998

replace regioncode="450221" if regioncode=="450221" & year==1998

replace regioncode="450222" if regioncode=="450222" & year==1998

replace regioncode="450223" if regioncode=="452223" & year==1998

replace regioncode="450224" if regioncode=="452227" & year==1998

replace regioncode="450225" if regioncode=="452229" & year==1998

replace regioncode="450226" if regioncode=="452228" & year==1998

replace regioncode="450302" if regioncode=="450302" & year==1998

replace regioncode="450303" if regioncode=="450303" & year==1998

replace regioncode="450304" if regioncode=="450304" & year==1998

replace regioncode="450305" if regioncode=="450305" & year==1998

replace regioncode="450311" if regioncode=="450311" & year==1998

replace regioncode="450321" if regioncode=="450321" & year==1998

replace regioncode="450322" if regioncode=="450322" & year==1998

replace regioncode="450323" if regioncode=="452322" & year==1998

replace regioncode="450324" if regioncode=="452323" & year==1998

replace regioncode="450325" if regioncode=="452324" & year==1998

replace regioncode="450326" if regioncode=="452325" & year==1998

replace regioncode="450327" if regioncode=="452327" & year==1998

replace regioncode="450328" if regioncode=="452328" & year==1998

replace regioncode="450329" if regioncode=="452329" & year==1998

replace regioncode="450330" if regioncode=="452330" & year==1998

replace regioncode="450331" if regioncode=="452331" & year==1998

replace regioncode="450332" if regioncode=="452332" & year==1998

replace regioncode="450403" if regioncode=="450403" & year==1998

replace regioncode="450404" if regioncode=="450404" & year==1998

replace regioncode="450421" if regioncode=="450421" & year==1998

replace regioncode="450422" if regioncode=="450422" & year==1998

replace regioncode="450423" if regioncode=="450423" & year==1998

replace regioncode="450481" if regioncode=="450481" & year==1998

replace regioncode="450502" if regioncode=="450502" & year==1998

replace regioncode="450503" if regioncode=="450503" & year==1998

replace regioncode="450512" if regioncode=="450504" & year==1998

replace regioncode="450521" if regioncode=="450521" & year==1998

replace regioncode="450602" if regioncode=="450602" & year==1998

replace regioncode="450603" if regioncode=="450603" & year==1998

replace regioncode="450621" if regioncode=="450621" & year==1998

replace regioncode="450681" if regioncode=="450681" & year==1998

replace regioncode="450702" if regioncode=="450702" & year==1998

replace regioncode="450703" if regioncode=="450703" & year==1998

replace regioncode="450721" if regioncode=="450721" & year==1998

replace regioncode="450722" if regioncode=="450722" & year==1998

replace regioncode="450802" if regioncode=="450802" & year==1998

replace regioncode="450803" if regioncode=="450803" & year==1998

replace regioncode="450804" if regioncode=="450804" & year==1998

replace regioncode="450821" if regioncode=="450821" & year==1998

replace regioncode="450881" if regioncode=="450881" & year==1998

replace regioncode="450902" if regioncode=="450902" & year==1998

replace regioncode="450921" if regioncode=="450921" & year==1998

replace regioncode="450922" if regioncode=="450922" & year==1998

replace regioncode="450923" if regioncode=="450923" & year==1998

replace regioncode="450924" if regioncode=="450924" & year==1998

replace regioncode="450981" if regioncode=="450981" & year==1998

replace regioncode="451002" if regioncode=="452601" & year==1998

replace regioncode="451021" if regioncode=="452622" & year==1998

replace regioncode="451022" if regioncode=="452623" & year==1998

replace regioncode="451023" if regioncode=="452624" & year==1998

replace regioncode="451024" if regioncode=="452625" & year==1998

replace regioncode="451025" if regioncode=="452626" & year==1998

replace regioncode="451026" if regioncode=="452627" & year==1998

replace regioncode="451027" if regioncode=="452628" & year==1998

replace regioncode="451028" if regioncode=="452629" & year==1998

replace regioncode="451029" if regioncode=="452630" & year==1998

replace regioncode="451030" if regioncode=="452632" & year==1998

replace regioncode="451031" if regioncode=="452631" & year==1998

replace regioncode="451102" if regioncode=="452402" & year==1998

replace regioncode="451121" if regioncode=="452424" & year==1998

replace regioncode="451122" if regioncode=="452427" & year==1998

replace regioncode="451123" if regioncode=="452428" & year==1998

replace regioncode="451202" if regioncode=="452701" & year==1998

replace regioncode="451221" if regioncode=="452725" & year==1998

replace regioncode="451222" if regioncode=="452726" & year==1998

replace regioncode="451223" if regioncode=="452727" & year==1998

replace regioncode="451224" if regioncode=="452728" & year==1998

replace regioncode="451225" if regioncode=="452723" & year==1998

replace regioncode="451226" if regioncode=="452724" & year==1998

replace regioncode="451227" if regioncode=="452729" & year==1998

replace regioncode="451228" if regioncode=="452730" & year==1998

replace regioncode="451229" if regioncode=="452731" & year==1998

replace regioncode="451281" if regioncode=="452702" & year==1998

replace regioncode="451302" if regioncode=="452226" & year==1998

replace regioncode="451321" if regioncode=="452231" & year==1998

replace regioncode="451322" if regioncode=="452224" & year==1998

replace regioncode="451323" if regioncode=="452225" & year==1998

replace regioncode="451324" if regioncode=="452230" & year==1998

replace regioncode="451381" if regioncode=="452201" & year==1998

replace regioncode="451402" if regioncode=="452129" & year==1998

replace regioncode="451421" if regioncode=="452128" & year==1998

replace regioncode="451422" if regioncode=="452132" & year==1998

replace regioncode="451423" if regioncode=="452133" & year==1998

replace regioncode="451424" if regioncode=="452130" & year==1998

replace regioncode="451425" if regioncode=="452131" & year==1998

replace regioncode="451481" if regioncode=="452101" & year==1998

replace regioncode="460105" if regioncode=="460102" & year==1998

replace regioncode="460106" if regioncode=="460103" & year==1998

replace regioncode="460107" if regioncode=="460104" & year==1998

replace regioncode="460108" if regioncode=="469004" & year==1998

replace regioncode="460001" if regioncode=="469001" & year==1998

replace regioncode="460002" if regioncode=="469002" & year==1998

replace regioncode="460003" if regioncode=="460003" & year==1998

replace regioncode="460005" if regioncode=="469005" & year==1998

replace regioncode="460006" if regioncode=="469006" & year==1998

replace regioncode="460007" if regioncode=="469007" & year==1998

replace regioncode="460030" if regioncode=="469025" & year==1998

replace regioncode="460031" if regioncode=="469026" & year==1998

replace regioncode="460033" if regioncode=="469027" & year==1998

replace regioncode="460034" if regioncode=="469028" & year==1998

replace regioncode="460036" if regioncode=="469030" & year==1998

replace regioncode="500101" if regioncode=="500101" & year==1998

replace regioncode="500102" if regioncode=="500102" & year==1998

replace regioncode="500103" if regioncode=="500103" & year==1998

replace regioncode="500104" if regioncode=="500104" & year==1998

replace regioncode="500105" if regioncode=="500105" & year==1998

replace regioncode="500106" if regioncode=="500106" & year==1998

replace regioncode="500107" if regioncode=="500107" & year==1998

replace regioncode="500108" if regioncode=="500108" & year==1998

replace regioncode="500109" if regioncode=="500109" & year==1998

replace regioncode="500110" if regioncode=="500110" & year==1998

replace regioncode="500111" if regioncode=="500111" & year==1998

replace regioncode="500112" if regioncode=="500112" & year==1998

replace regioncode="500113" if regioncode=="500113" & year==1998

replace regioncode="500114" if regioncode=="500239" & year==1998

replace regioncode="500115" if regioncode=="500221" & year==1998

replace regioncode="500381" if regioncode=="500381" & year==1998

replace regioncode="500382" if regioncode=="500382" & year==1998

replace regioncode="500383" if regioncode=="500383" & year==1998

replace regioncode="500384" if regioncode=="500384" & year==1998

replace regioncode="500222" if regioncode=="500222" & year==1998

replace regioncode="500223" if regioncode=="500223" & year==1998

replace regioncode="500224" if regioncode=="500224" & year==1998

replace regioncode="500225" if regioncode=="500225" & year==1998

replace regioncode="500226" if regioncode=="500226" & year==1998

replace regioncode="500227" if regioncode=="500227" & year==1998

replace regioncode="500228" if regioncode=="500228" & year==1998

replace regioncode="500229" if regioncode=="500229" & year==1998

replace regioncode="500230" if regioncode=="500230" & year==1998

replace regioncode="500231" if regioncode=="500231" & year==1998

replace regioncode="500232" if regioncode=="500232" & year==1998

replace regioncode="500233" if regioncode=="500233" & year==1998

replace regioncode="500234" if regioncode=="500234" & year==1998

replace regioncode="500235" if regioncode=="500235" & year==1998

replace regioncode="500236" if regioncode=="500236" & year==1998

replace regioncode="500237" if regioncode=="500237" & year==1998

replace regioncode="500238" if regioncode=="500238" & year==1998

replace regioncode="500240" if regioncode=="500240" & year==1998

replace regioncode="500241" if regioncode=="500241" & year==1998

replace regioncode="500242" if regioncode=="500242" & year==1998

replace regioncode="500243" if regioncode=="500243" & year==1998

replace regioncode="510104" if regioncode=="510104" & year==1998

replace regioncode="510105" if regioncode=="510105" & year==1998

replace regioncode="510106" if regioncode=="510106" & year==1998

replace regioncode="510107" if regioncode=="510107" & year==1998

replace regioncode="510108" if regioncode=="510108" & year==1998

replace regioncode="510112" if regioncode=="510112" & year==1998

replace regioncode="510113" if regioncode=="510113" & year==1998

replace regioncode="510114" if regioncode=="510125" & year==1998

replace regioncode="510115" if regioncode=="510123" & year==1998

replace regioncode="510121" if regioncode=="510121" & year==1998

replace regioncode="510122" if regioncode=="510122" & year==1998

replace regioncode="510124" if regioncode=="510124" & year==1998

replace regioncode="510129" if regioncode=="510129" & year==1998

replace regioncode="510131" if regioncode=="510131" & year==1998

replace regioncode="510132" if regioncode=="510132" & year==1998

replace regioncode="510181" if regioncode=="510181" & year==1998

replace regioncode="510182" if regioncode=="510182" & year==1998

replace regioncode="510183" if regioncode=="510183" & year==1998

replace regioncode="510184" if regioncode=="510184" & year==1998

replace regioncode="510302" if regioncode=="510302" & year==1998

replace regioncode="510303" if regioncode=="510303" & year==1998

replace regioncode="510304" if regioncode=="510304" & year==1998

replace regioncode="510311" if regioncode=="510311" & year==1998

replace regioncode="510321" if regioncode=="510321" & year==1998

replace regioncode="510322" if regioncode=="510322" & year==1998

replace regioncode="510402" if regioncode=="510402" & year==1998

replace regioncode="510403" if regioncode=="510403" & year==1998

replace regioncode="510411" if regioncode=="510411" & year==1998

replace regioncode="510421" if regioncode=="510421" & year==1998

replace regioncode="510422" if regioncode=="510422" & year==1998

replace regioncode="510502" if regioncode=="510502" & year==1998

replace regioncode="510503" if regioncode=="510503" & year==1998

replace regioncode="510504" if regioncode=="510504" & year==1998

replace regioncode="510521" if regioncode=="510521" & year==1998

replace regioncode="510522" if regioncode=="510522" & year==1998

replace regioncode="510524" if regioncode=="510524" & year==1998

replace regioncode="510525" if regioncode=="510525" & year==1998

replace regioncode="510603" if regioncode=="510603" & year==1998

replace regioncode="510623" if regioncode=="510623" & year==1998

replace regioncode="510626" if regioncode=="510626" & year==1998

replace regioncode="510681" if regioncode=="510681" & year==1998

replace regioncode="510682" if regioncode=="510682" & year==1998

replace regioncode="510683" if regioncode=="510683" & year==1998

replace regioncode="510703" if regioncode=="510703" & year==1998

replace regioncode="510704" if regioncode=="510704" & year==1998

replace regioncode="510722" if regioncode=="510722" & year==1998

replace regioncode="510723" if regioncode=="510723" & year==1998

replace regioncode="510724" if regioncode=="510724" & year==1998

replace regioncode="510725" if regioncode=="510725" & year==1998

replace regioncode="510726" if regioncode=="510726" & year==1998

replace regioncode="510727" if regioncode=="510727" & year==1998

replace regioncode="510781" if regioncode=="510781" & year==1998

replace regioncode="510802" if regioncode=="510802" & year==1998

replace regioncode="510811" if regioncode=="510811" & year==1998

replace regioncode="510812" if regioncode=="510812" & year==1998

replace regioncode="510821" if regioncode=="510821" & year==1998

replace regioncode="510822" if regioncode=="510822" & year==1998

replace regioncode="510823" if regioncode=="510823" & year==1998

replace regioncode="510824" if regioncode=="510824" & year==1998

replace regioncode="510902" if regioncode=="510902" & year==1998

replace regioncode="510921" if regioncode=="510921" & year==1998

replace regioncode="510922" if regioncode=="510922" & year==1998

replace regioncode="510923" if regioncode=="510923" & year==1998

replace regioncode="511002" if regioncode=="511002" & year==1998

replace regioncode="511011" if regioncode=="511011" & year==1998

replace regioncode="511024" if regioncode=="511024" & year==1998

replace regioncode="511025" if regioncode=="511025" & year==1998

replace regioncode="511028" if regioncode=="511028" & year==1998

replace regioncode="511102" if regioncode=="511102" & year==1998

replace regioncode="511111" if regioncode=="511111" & year==1998

replace regioncode="511112" if regioncode=="511112" & year==1998

replace regioncode="511113" if regioncode=="511113" & year==1998

replace regioncode="511123" if regioncode=="511123" & year==1998

replace regioncode="511124" if regioncode=="511124" & year==1998

replace regioncode="511126" if regioncode=="511126" & year==1998

replace regioncode="511129" if regioncode=="511129" & year==1998

replace regioncode="511132" if regioncode=="511132" & year==1998

replace regioncode="511133" if regioncode=="511133" & year==1998

replace regioncode="511181" if regioncode=="511181" & year==1998

replace regioncode="511302" if regioncode=="511302" & year==1998

replace regioncode="511303" if regioncode=="511303" & year==1998

replace regioncode="511304" if regioncode=="511304" & year==1998

replace regioncode="511321" if regioncode=="511321" & year==1998

replace regioncode="511322" if regioncode=="511322" & year==1998

replace regioncode="511323" if regioncode=="511323" & year==1998

replace regioncode="511324" if regioncode=="511324" & year==1998

replace regioncode="511325" if regioncode=="511325" & year==1998

replace regioncode="511381" if regioncode=="511381" & year==1998

replace regioncode="511402" if regioncode=="513821" & year==1998

replace regioncode="511421" if regioncode=="513822" & year==1998

replace regioncode="511422" if regioncode=="513823" & year==1998

replace regioncode="511423" if regioncode=="513824" & year==1998

replace regioncode="511424" if regioncode=="513825" & year==1998

replace regioncode="511425" if regioncode=="513826" & year==1998

replace regioncode="511502" if regioncode=="512501" & year==1998

replace regioncode="511521" if regioncode=="512527" & year==1998

replace regioncode="511522" if regioncode=="512528" & year==1998

replace regioncode="511523" if regioncode=="512529" & year==1998

replace regioncode="511524" if regioncode=="512530" & year==1998

replace regioncode="511525" if regioncode=="512531" & year==1998

replace regioncode="511526" if regioncode=="512533" & year==1998

replace regioncode="511527" if regioncode=="512532" & year==1998

replace regioncode="511528" if regioncode=="512534" & year==1998

replace regioncode="511529" if regioncode=="512535" & year==1998

replace regioncode="511602" if regioncode=="513622" & year==1998

replace regioncode="511621" if regioncode=="513621" & year==1998

replace regioncode="511622" if regioncode=="513623" & year==1998

replace regioncode="511623" if regioncode=="513624" & year==1998

replace regioncode="511681" if regioncode=="513601" & year==1998

replace regioncode="511702" if regioncode=="513001" & year==1998

replace regioncode="511721" if regioncode=="513021" & year==1998

replace regioncode="511722" if regioncode=="513022" & year==1998

replace regioncode="511723" if regioncode=="513023" & year==1998

replace regioncode="511724" if regioncode=="513029" & year==1998

replace regioncode="511725" if regioncode=="513030" & year==1998

replace regioncode="511781" if regioncode=="513002" & year==1998

replace regioncode="511802" if regioncode=="513101" & year==1998

replace regioncode="511821" if regioncode=="513122" & year==1998

replace regioncode="511822" if regioncode=="513123" & year==1998

replace regioncode="511823" if regioncode=="513124" & year==1998

replace regioncode="511824" if regioncode=="513125" & year==1998

replace regioncode="511825" if regioncode=="513126" & year==1998

replace regioncode="511826" if regioncode=="513127" & year==1998

replace regioncode="511827" if regioncode=="513128" & year==1998

replace regioncode="511902" if regioncode=="513701" & year==1998

replace regioncode="511921" if regioncode=="513721" & year==1998

replace regioncode="511922" if regioncode=="513722" & year==1998

replace regioncode="511923" if regioncode=="513723" & year==1998

replace regioncode="512002" if regioncode=="513901" & year==1998

replace regioncode="512021" if regioncode=="513921" & year==1998

replace regioncode="512022" if regioncode=="513922" & year==1998

replace regioncode="512081" if regioncode=="513902" & year==1998

replace regioncode="513221" if regioncode=="513221" & year==1998

replace regioncode="513222" if regioncode=="513222" & year==1998

replace regioncode="513223" if regioncode=="513223" & year==1998

replace regioncode="513224" if regioncode=="513224" & year==1998

replace regioncode="513225" if regioncode=="513225" & year==1998

replace regioncode="513226" if regioncode=="513226" & year==1998

replace regioncode="513227" if regioncode=="513227" & year==1998

replace regioncode="513228" if regioncode=="513228" & year==1998

replace regioncode="513229" if regioncode=="513229" & year==1998

replace regioncode="513230" if regioncode=="513230" & year==1998

replace regioncode="513231" if regioncode=="513231" & year==1998

replace regioncode="513232" if regioncode=="513232" & year==1998

replace regioncode="513233" if regioncode=="513233" & year==1998

replace regioncode="513321" if regioncode=="513321" & year==1998

replace regioncode="513322" if regioncode=="513322" & year==1998

replace regioncode="513323" if regioncode=="513323" & year==1998

replace regioncode="513324" if regioncode=="513324" & year==1998

replace regioncode="513325" if regioncode=="513325" & year==1998

replace regioncode="513326" if regioncode=="513326" & year==1998

replace regioncode="513327" if regioncode=="513327" & year==1998

replace regioncode="513328" if regioncode=="513328" & year==1998

replace regioncode="513329" if regioncode=="513329" & year==1998

replace regioncode="513330" if regioncode=="513330" & year==1998

replace regioncode="513331" if regioncode=="513331" & year==1998

replace regioncode="513332" if regioncode=="513332" & year==1998

replace regioncode="513333" if regioncode=="513333" & year==1998

replace regioncode="513334" if regioncode=="513334" & year==1998

replace regioncode="513335" if regioncode=="513335" & year==1998

replace regioncode="513336" if regioncode=="513336" & year==1998

replace regioncode="513337" if regioncode=="513337" & year==1998

replace regioncode="513338" if regioncode=="513338" & year==1998

replace regioncode="513401" if regioncode=="513401" & year==1998

replace regioncode="513422" if regioncode=="513422" & year==1998

replace regioncode="513423" if regioncode=="513423" & year==1998

replace regioncode="513424" if regioncode=="513424" & year==1998

replace regioncode="513425" if regioncode=="513425" & year==1998

replace regioncode="513426" if regioncode=="513426" & year==1998

replace regioncode="513427" if regioncode=="513427" & year==1998

replace regioncode="513428" if regioncode=="513428" & year==1998

replace regioncode="513429" if regioncode=="513429" & year==1998

replace regioncode="513430" if regioncode=="513430" & year==1998

replace regioncode="513431" if regioncode=="513431" & year==1998

replace regioncode="513432" if regioncode=="513432" & year==1998

replace regioncode="513433" if regioncode=="513433" & year==1998

replace regioncode="513434" if regioncode=="513434" & year==1998

replace regioncode="513435" if regioncode=="513435" & year==1998

replace regioncode="513436" if regioncode=="513436" & year==1998

replace regioncode="513437" if regioncode=="513437" & year==1998

replace regioncode="520102" if regioncode=="520102" & year==1998

replace regioncode="520103" if regioncode=="520103" & year==1998

replace regioncode="520111" if regioncode=="520111" & year==1998

replace regioncode="520112" if regioncode=="520112" & year==1998

replace regioncode="520113" if regioncode=="520113" & year==1998

replace regioncode="520114" if regioncode=="520114" & year==1998

replace regioncode="520121" if regioncode=="520121" & year==1998

replace regioncode="520122" if regioncode=="520122" & year==1998

replace regioncode="520123" if regioncode=="520123" & year==1998

replace regioncode="520181" if regioncode=="520181" & year==1998

replace regioncode="520201" if regioncode=="520201" & year==1998

replace regioncode="520222" if regioncode=="520202" & year==1998

replace regioncode="520203" if regioncode=="520203" & year==1998

replace regioncode="520221" if regioncode=="520221" & year==1998

replace regioncode="520302" if regioncode=="520302" & year==1998

replace regioncode="520321" if regioncode=="520321" & year==1998

replace regioncode="520322" if regioncode=="520322" & year==1998

replace regioncode="520323" if regioncode=="520323" & year==1998

replace regioncode="520324" if regioncode=="520324" & year==1998

replace regioncode="520325" if regioncode=="520325" & year==1998

replace regioncode="520326" if regioncode=="520326" & year==1998

replace regioncode="520327" if regioncode=="520327" & year==1998

replace regioncode="520328" if regioncode=="520328" & year==1998

replace regioncode="520329" if regioncode=="520329" & year==1998

replace regioncode="520330" if regioncode=="520330" & year==1998

replace regioncode="520381" if regioncode=="520381" & year==1998

replace regioncode="520382" if regioncode=="520382" & year==1998

replace regioncode="520402" if regioncode=="522501" & year==1998

replace regioncode="520421" if regioncode=="522526" & year==1998

replace regioncode="520422" if regioncode=="522527" & year==1998

replace regioncode="520423" if regioncode=="522529" & year==1998

replace regioncode="520424" if regioncode=="522528" & year==1998

replace regioncode="520425" if regioncode=="522530" & year==1998

replace regioncode="522201" if regioncode=="522201" & year==1998

replace regioncode="522222" if regioncode=="522222" & year==1998

replace regioncode="522223" if regioncode=="522223" & year==1998

replace regioncode="522224" if regioncode=="522224" & year==1998

replace regioncode="522225" if regioncode=="522225" & year==1998

replace regioncode="522226" if regioncode=="522226" & year==1998

replace regioncode="522227" if regioncode=="522227" & year==1998

replace regioncode="522228" if regioncode=="522228" & year==1998

replace regioncode="522229" if regioncode=="522229" & year==1998

replace regioncode="522230" if regioncode=="522230" & year==1998

replace regioncode="522301" if regioncode=="522301" & year==1998

replace regioncode="522322" if regioncode=="522322" & year==1998

replace regioncode="522323" if regioncode=="522323" & year==1998

replace regioncode="522324" if regioncode=="522324" & year==1998

replace regioncode="522325" if regioncode=="522325" & year==1998

replace regioncode="522326" if regioncode=="522326" & year==1998

replace regioncode="522327" if regioncode=="522327" & year==1998

replace regioncode="522328" if regioncode=="522328" & year==1998

replace regioncode="522401" if regioncode=="522401" & year==1998

replace regioncode="522422" if regioncode=="522422" & year==1998

replace regioncode="522423" if regioncode=="522423" & year==1998

replace regioncode="522424" if regioncode=="522424" & year==1998

replace regioncode="522425" if regioncode=="522425" & year==1998

replace regioncode="522426" if regioncode=="522426" & year==1998

replace regioncode="522427" if regioncode=="522427" & year==1998

replace regioncode="522428" if regioncode=="522428" & year==1998

replace regioncode="522601" if regioncode=="522601" & year==1998

replace regioncode="522622" if regioncode=="522622" & year==1998

replace regioncode="522623" if regioncode=="522623" & year==1998

replace regioncode="522624" if regioncode=="522624" & year==1998

replace regioncode="522625" if regioncode=="522625" & year==1998

replace regioncode="522626" if regioncode=="522626" & year==1998

replace regioncode="522627" if regioncode=="522627" & year==1998

replace regioncode="522628" if regioncode=="522628" & year==1998

replace regioncode="522629" if regioncode=="522629" & year==1998

replace regioncode="522630" if regioncode=="522630" & year==1998

replace regioncode="522631" if regioncode=="522631" & year==1998

replace regioncode="522632" if regioncode=="522632" & year==1998

replace regioncode="522633" if regioncode=="522633" & year==1998

replace regioncode="522634" if regioncode=="522634" & year==1998

replace regioncode="522635" if regioncode=="522635" & year==1998

replace regioncode="522636" if regioncode=="522636" & year==1998

replace regioncode="522701" if regioncode=="522701" & year==1998

replace regioncode="522702" if regioncode=="522702" & year==1998

replace regioncode="522722" if regioncode=="522722" & year==1998

replace regioncode="522723" if regioncode=="522723" & year==1998

replace regioncode="522725" if regioncode=="522725" & year==1998

replace regioncode="522726" if regioncode=="522726" & year==1998

replace regioncode="522727" if regioncode=="522727" & year==1998

replace regioncode="522728" if regioncode=="522728" & year==1998

replace regioncode="522729" if regioncode=="522729" & year==1998

replace regioncode="522730" if regioncode=="522730" & year==1998

replace regioncode="522731" if regioncode=="522731" & year==1998

replace regioncode="522732" if regioncode=="522732" & year==1998

replace regioncode="530102" if regioncode=="530102" & year==1998

replace regioncode="530103" if regioncode=="530103" & year==1998

replace regioncode="530111" if regioncode=="530111" & year==1998

replace regioncode="530112" if regioncode=="530112" & year==1998

replace regioncode="530113" if regioncode=="530113" & year==1998

replace regioncode="530121" if regioncode=="530121" & year==1998

replace regioncode="530122" if regioncode=="530122" & year==1998

replace regioncode="530124" if regioncode=="530124" & year==1998

replace regioncode="530125" if regioncode=="530125" & year==1998

replace regioncode="530126" if regioncode=="530126" & year==1998

replace regioncode="530127" if regioncode=="530127" & year==1998

replace regioncode="530128" if regioncode=="530128" & year==1998

replace regioncode="530129" if regioncode=="530129" & year==1998

replace regioncode="530181" if regioncode=="530181" & year==1998

replace regioncode="530302" if regioncode=="530302" & year==1998

replace regioncode="530321" if regioncode=="530321" & year==1998

replace regioncode="530322" if regioncode=="530322" & year==1998

replace regioncode="530323" if regioncode=="530323" & year==1998

replace regioncode="530324" if regioncode=="530324" & year==1998

replace regioncode="530325" if regioncode=="530325" & year==1998

replace regioncode="530326" if regioncode=="530326" & year==1998

replace regioncode="530328" if regioncode=="530328" & year==1998

replace regioncode="530381" if regioncode=="530381" & year==1998

replace regioncode="530402" if regioncode=="530402" & year==1998

replace regioncode="530421" if regioncode=="530421" & year==1998

replace regioncode="530422" if regioncode=="530422" & year==1998

replace regioncode="530423" if regioncode=="530423" & year==1998

replace regioncode="530424" if regioncode=="530424" & year==1998

replace regioncode="530425" if regioncode=="530425" & year==1998

replace regioncode="530426" if regioncode=="530426" & year==1998

replace regioncode="530427" if regioncode=="530427" & year==1998

replace regioncode="530428" if regioncode=="530428" & year==1998

replace regioncode="530502" if regioncode=="533001" & year==1998

replace regioncode="530521" if regioncode=="533022" & year==1998

replace regioncode="530522" if regioncode=="533023" & year==1998

replace regioncode="530523" if regioncode=="533024" & year==1998

replace regioncode="530524" if regioncode=="533025" & year==1998

replace regioncode="530602" if regioncode=="532101" & year==1998

replace regioncode="530621" if regioncode=="532122" & year==1998

replace regioncode="530622" if regioncode=="532123" & year==1998

replace regioncode="530623" if regioncode=="532124" & year==1998

replace regioncode="530624" if regioncode=="532125" & year==1998

replace regioncode="530625" if regioncode=="532126" & year==1998

replace regioncode="530626" if regioncode=="532127" & year==1998

replace regioncode="530627" if regioncode=="532128" & year==1998

replace regioncode="530628" if regioncode=="532129" & year==1998

replace regioncode="530629" if regioncode=="532130" & year==1998

replace regioncode="530630" if regioncode=="532131" & year==1998

replace regioncode="530721" if regioncode=="533221" & year==1998

replace regioncode="530722" if regioncode=="533222" & year==1998

replace regioncode="530723" if regioncode=="533223" & year==1998

replace regioncode="530724" if regioncode=="533224" & year==1998

replace regioncode="532701" if regioncode=="532701" & year==1998

replace regioncode="532722" if regioncode=="532722" & year==1998

replace regioncode="532723" if regioncode=="532723" & year==1998

replace regioncode="532724" if regioncode=="532724" & year==1998

replace regioncode="532725" if regioncode=="532725" & year==1998

replace regioncode="532726" if regioncode=="532726" & year==1998

replace regioncode="532727" if regioncode=="532727" & year==1998

replace regioncode="532728" if regioncode=="532728" & year==1998

replace regioncode="532729" if regioncode=="532729" & year==1998

replace regioncode="532730" if regioncode=="532730" & year==1998

replace regioncode="533521" if regioncode=="533521" & year==1998

replace regioncode="533522" if regioncode=="533522" & year==1998

replace regioncode="533523" if regioncode=="533523" & year==1998

replace regioncode="533524" if regioncode=="533524" & year==1998

replace regioncode="533525" if regioncode=="533525" & year==1998

replace regioncode="533526" if regioncode=="533526" & year==1998

replace regioncode="533527" if regioncode=="533527" & year==1998

replace regioncode="533528" if regioncode=="533528" & year==1998

replace regioncode="532301" if regioncode=="532301" & year==1998

replace regioncode="532322" if regioncode=="532322" & year==1998

replace regioncode="532323" if regioncode=="532323" & year==1998

replace regioncode="532324" if regioncode=="532324" & year==1998

replace regioncode="532325" if regioncode=="532325" & year==1998

replace regioncode="532326" if regioncode=="532326" & year==1998

replace regioncode="532327" if regioncode=="532327" & year==1998

replace regioncode="532328" if regioncode=="532328" & year==1998

replace regioncode="532329" if regioncode=="532329" & year==1998

replace regioncode="532331" if regioncode=="532331" & year==1998

replace regioncode="532501" if regioncode=="532501" & year==1998

replace regioncode="532502" if regioncode=="532502" & year==1998

replace regioncode="532522" if regioncode=="532522" & year==1998

replace regioncode="532523" if regioncode=="532523" & year==1998

replace regioncode="532524" if regioncode=="532524" & year==1998

replace regioncode="532525" if regioncode=="532525" & year==1998

replace regioncode="532526" if regioncode=="532526" & year==1998

replace regioncode="532527" if regioncode=="532527" & year==1998

replace regioncode="532528" if regioncode=="532528" & year==1998

replace regioncode="532529" if regioncode=="532529" & year==1998

replace regioncode="532530" if regioncode=="532530" & year==1998

replace regioncode="532531" if regioncode=="532531" & year==1998

replace regioncode="532532" if regioncode=="532532" & year==1998

replace regioncode="532621" if regioncode=="532621" & year==1998

replace regioncode="532622" if regioncode=="532622" & year==1998

replace regioncode="532623" if regioncode=="532623" & year==1998

replace regioncode="532624" if regioncode=="532624" & year==1998

replace regioncode="532625" if regioncode=="532625" & year==1998

replace regioncode="532626" if regioncode=="532626" & year==1998

replace regioncode="532627" if regioncode=="532627" & year==1998

replace regioncode="532628" if regioncode=="532628" & year==1998

replace regioncode="532801" if regioncode=="532801" & year==1998

replace regioncode="532822" if regioncode=="532822" & year==1998

replace regioncode="532823" if regioncode=="532823" & year==1998

replace regioncode="532901" if regioncode=="532901" & year==1998

replace regioncode="532922" if regioncode=="532922" & year==1998

replace regioncode="532923" if regioncode=="532923" & year==1998

replace regioncode="532924" if regioncode=="532924" & year==1998

replace regioncode="532925" if regioncode=="532925" & year==1998

replace regioncode="532926" if regioncode=="532926" & year==1998

replace regioncode="532927" if regioncode=="532927" & year==1998

replace regioncode="532928" if regioncode=="532928" & year==1998

replace regioncode="532929" if regioncode=="532929" & year==1998

replace regioncode="532930" if regioncode=="532930" & year==1998

replace regioncode="532931" if regioncode=="532931" & year==1998

replace regioncode="532932" if regioncode=="532932" & year==1998

replace regioncode="533102" if regioncode=="533102" & year==1998

replace regioncode="533103" if regioncode=="533103" & year==1998

replace regioncode="533122" if regioncode=="533122" & year==1998

replace regioncode="533123" if regioncode=="533123" & year==1998

replace regioncode="533124" if regioncode=="533124" & year==1998

replace regioncode="533321" if regioncode=="533321" & year==1998

replace regioncode="533323" if regioncode=="533323" & year==1998

replace regioncode="533324" if regioncode=="533324" & year==1998

replace regioncode="533325" if regioncode=="533325" & year==1998

replace regioncode="533421" if regioncode=="533421" & year==1998

replace regioncode="533422" if regioncode=="533422" & year==1998

replace regioncode="533423" if regioncode=="533423" & year==1998

replace regioncode="540102" if regioncode=="540102" & year==1998

replace regioncode="540121" if regioncode=="540121" & year==1998

replace regioncode="540122" if regioncode=="540122" & year==1998

replace regioncode="540123" if regioncode=="540123" & year==1998

replace regioncode="540124" if regioncode=="540124" & year==1998

replace regioncode="540125" if regioncode=="540125" & year==1998

replace regioncode="540126" if regioncode=="540126" & year==1998

replace regioncode="540127" if regioncode=="540127" & year==1998

replace regioncode="542121" if regioncode=="542121" & year==1998

replace regioncode="542122" if regioncode=="542122" & year==1998

replace regioncode="542123" if regioncode=="542123" & year==1998

replace regioncode="542124" if regioncode=="542124" & year==1998

replace regioncode="542125" if regioncode=="542125" & year==1998

replace regioncode="542126" if regioncode=="542126" & year==1998

replace regioncode="542127" if regioncode=="542127" & year==1998

replace regioncode="542128" if regioncode=="542128" & year==1998

replace regioncode="542129" if regioncode=="542129" & year==1998

replace regioncode="542132" if regioncode=="542132" & year==1998

replace regioncode="542133" if regioncode=="542133" & year==1998

replace regioncode="542221" if regioncode=="542221" & year==1998

replace regioncode="542223" if regioncode=="542223" & year==1998

replace regioncode="542224" if regioncode=="542224" & year==1998

replace regioncode="542225" if regioncode=="542225" & year==1998

replace regioncode="542227" if regioncode=="542227" & year==1998

replace regioncode="542228" if regioncode=="542228" & year==1998

replace regioncode="542229" if regioncode=="542229" & year==1998

replace regioncode="542231" if regioncode=="542231" & year==1998

replace regioncode="542232" if regioncode=="542232" & year==1998

replace regioncode="542233" if regioncode=="542233" & year==1998

replace regioncode="542301" if regioncode=="542301" & year==1998

replace regioncode="542322" if regioncode=="542322" & year==1998

replace regioncode="542323" if regioncode=="542323" & year==1998

replace regioncode="542324" if regioncode=="542324" & year==1998

replace regioncode="542325" if regioncode=="542325" & year==1998

replace regioncode="542326" if regioncode=="542326" & year==1998

replace regioncode="542327" if regioncode=="542327" & year==1998

replace regioncode="542328" if regioncode=="542328" & year==1998

replace regioncode="542329" if regioncode=="542329" & year==1998

replace regioncode="542330" if regioncode=="542330" & year==1998

replace regioncode="542331" if regioncode=="542331" & year==1998

replace regioncode="542332" if regioncode=="542332" & year==1998

replace regioncode="542334" if regioncode=="542334" & year==1998

replace regioncode="542336" if regioncode=="542336" & year==1998

replace regioncode="542338" if regioncode=="542338" & year==1998

replace regioncode="542421" if regioncode=="542421" & year==1998

replace regioncode="542422" if regioncode=="542422" & year==1998

replace regioncode="542423" if regioncode=="542423" & year==1998

replace regioncode="542424" if regioncode=="542424" & year==1998

replace regioncode="542425" if regioncode=="542425" & year==1998

replace regioncode="542426" if regioncode=="542426" & year==1998

replace regioncode="542427" if regioncode=="542427" & year==1998

replace regioncode="542429" if regioncode=="542429" & year==1998

replace regioncode="542430" if regioncode=="542430" & year==1998

replace regioncode="542523" if regioncode=="542523" & year==1998

replace regioncode="542621" if regioncode=="542621" & year==1998

replace regioncode="542622" if regioncode=="542622" & year==1998

replace regioncode="542623" if regioncode=="542623" & year==1998

replace regioncode="542625" if regioncode=="542625" & year==1998

replace regioncode="542626" if regioncode=="542626" & year==1998

replace regioncode="610102" if regioncode=="610102" & year==1998

replace regioncode="610103" if regioncode=="610103" & year==1998

replace regioncode="610104" if regioncode=="610104" & year==1998

replace regioncode="610111" if regioncode=="610111" & year==1998

replace regioncode="610112" if regioncode=="610112" & year==1998

replace regioncode="610113" if regioncode=="610113" & year==1998

replace regioncode="610114" if regioncode=="610114" & year==1998

replace regioncode="610115" if regioncode=="610123" & year==1998

replace regioncode="610116" if regioncode=="610121" & year==1998

replace regioncode="610122" if regioncode=="610122" & year==1998

replace regioncode="610124" if regioncode=="610124" & year==1998

replace regioncode="610125" if regioncode=="610125" & year==1998

replace regioncode="610126" if regioncode=="610126" & year==1998

replace regioncode="610202" if regioncode=="610202" & year==1998

replace regioncode="610203" if regioncode=="610203" & year==1998

replace regioncode="610204" if regioncode=="610221" & year==1998

replace regioncode="610222" if regioncode=="610222" & year==1998

replace regioncode="610302" if regioncode=="610302" & year==1998

replace regioncode="610303" if regioncode=="610303" & year==1998

replace regioncode="610304" if regioncode=="610321" & year==1998

replace regioncode="610322" if regioncode=="610322" & year==1998

replace regioncode="610323" if regioncode=="610323" & year==1998

replace regioncode="610324" if regioncode=="610324" & year==1998

replace regioncode="610326" if regioncode=="610326" & year==1998

replace regioncode="610327" if regioncode=="610327" & year==1998

replace regioncode="610328" if regioncode=="610328" & year==1998

replace regioncode="610329" if regioncode=="610329" & year==1998

replace regioncode="610330" if regioncode=="610330" & year==1998

replace regioncode="610331" if regioncode=="610331" & year==1998

replace regioncode="610402" if regioncode=="610402" & year==1998

replace regioncode="610403" if regioncode=="610403" & year==1998

replace regioncode="610404" if regioncode=="610404" & year==1998

replace regioncode="610422" if regioncode=="610422" & year==1998

replace regioncode="610423" if regioncode=="610423" & year==1998

replace regioncode="610424" if regioncode=="610424" & year==1998

replace regioncode="610425" if regioncode=="610425" & year==1998

replace regioncode="610426" if regioncode=="610426" & year==1998

replace regioncode="610427" if regioncode=="610427" & year==1998

replace regioncode="610428" if regioncode=="610428" & year==1998

replace regioncode="610429" if regioncode=="610429" & year==1998

replace regioncode="610430" if regioncode=="610430" & year==1998

replace regioncode="610431" if regioncode=="610431" & year==1998

replace regioncode="610481" if regioncode=="610481" & year==1998

replace regioncode="610502" if regioncode=="610502" & year==1998

replace regioncode="610521" if regioncode=="610521" & year==1998

replace regioncode="610522" if regioncode=="610522" & year==1998

replace regioncode="610523" if regioncode=="610523" & year==1998

replace regioncode="610524" if regioncode=="610524" & year==1998

replace regioncode="610525" if regioncode=="610525" & year==1998

replace regioncode="610526" if regioncode=="610526" & year==1998

replace regioncode="610527" if regioncode=="610527" & year==1998

replace regioncode="610528" if regioncode=="610528" & year==1998

replace regioncode="610581" if regioncode=="610581" & year==1998

replace regioncode="610582" if regioncode=="610582" & year==1998

replace regioncode="610602" if regioncode=="612601" & year==1998

replace regioncode="610621" if regioncode=="612621" & year==1998

replace regioncode="610622" if regioncode=="612622" & year==1998

replace regioncode="610623" if regioncode=="612623" & year==1998

replace regioncode="610624" if regioncode=="612624" & year==1998

replace regioncode="610625" if regioncode=="612625" & year==1998

replace regioncode="610626" if regioncode=="612626" & year==1998

replace regioncode="610627" if regioncode=="612627" & year==1998

replace regioncode="610628" if regioncode=="612628" & year==1998

replace regioncode="610629" if regioncode=="612629" & year==1998

replace regioncode="610630" if regioncode=="612630" & year==1998

replace regioncode="610631" if regioncode=="612631" & year==1998

replace regioncode="610632" if regioncode=="612632" & year==1998

replace regioncode="610702" if regioncode=="612301" & year==1998

replace regioncode="610721" if regioncode=="612321" & year==1998

replace regioncode="610722" if regioncode=="612322" & year==1998

replace regioncode="610723" if regioncode=="612323" & year==1998

replace regioncode="610724" if regioncode=="612324" & year==1998

replace regioncode="610725" if regioncode=="612325" & year==1998

replace regioncode="610726" if regioncode=="612326" & year==1998

replace regioncode="610727" if regioncode=="612327" & year==1998

replace regioncode="610728" if regioncode=="612328" & year==1998

replace regioncode="610729" if regioncode=="612329" & year==1998

replace regioncode="610730" if regioncode=="612330" & year==1998

replace regioncode="610802" if regioncode=="612701" & year==1998

replace regioncode="610821" if regioncode=="612722" & year==1998

replace regioncode="610822" if regioncode=="612723" & year==1998

replace regioncode="610823" if regioncode=="612724" & year==1998

replace regioncode="610824" if regioncode=="612725" & year==1998

replace regioncode="610825" if regioncode=="612726" & year==1998

replace regioncode="610826" if regioncode=="612727" & year==1998

replace regioncode="610827" if regioncode=="612728" & year==1998

replace regioncode="610828" if regioncode=="612729" & year==1998

replace regioncode="610829" if regioncode=="612730" & year==1998

replace regioncode="610830" if regioncode=="612731" & year==1998

replace regioncode="610831" if regioncode=="612732" & year==1998

replace regioncode="610902" if regioncode=="612401" & year==1998

replace regioncode="610921" if regioncode=="612422" & year==1998

replace regioncode="610922" if regioncode=="612423" & year==1998

replace regioncode="610923" if regioncode=="612424" & year==1998

replace regioncode="610924" if regioncode=="612425" & year==1998

replace regioncode="610925" if regioncode=="612426" & year==1998

replace regioncode="610926" if regioncode=="612427" & year==1998

replace regioncode="610927" if regioncode=="612428" & year==1998

replace regioncode="610928" if regioncode=="612429" & year==1998

replace regioncode="610929" if regioncode=="612430" & year==1998

replace regioncode="611002" if regioncode=="612501" & year==1998

replace regioncode="611021" if regioncode=="612522" & year==1998

replace regioncode="611022" if regioncode=="612523" & year==1998

replace regioncode="611023" if regioncode=="612524" & year==1998

replace regioncode="611024" if regioncode=="612525" & year==1998

replace regioncode="611025" if regioncode=="612526" & year==1998

replace regioncode="611026" if regioncode=="612527" & year==1998

replace regioncode="620102" if regioncode=="620102" & year==1998

replace regioncode="620103" if regioncode=="620103" & year==1998

replace regioncode="620104" if regioncode=="620104" & year==1998

replace regioncode="620105" if regioncode=="620105" & year==1998

replace regioncode="620111" if regioncode=="620111" & year==1998

replace regioncode="620121" if regioncode=="620121" & year==1998

replace regioncode="620122" if regioncode=="620122" & year==1998

replace regioncode="620123" if regioncode=="620123" & year==1998

replace regioncode="620201" if regioncode=="620202" & year==1998

replace regioncode="620302" if regioncode=="620302" & year==1998

replace regioncode="620321" if regioncode=="620321" & year==1998

replace regioncode="620402" if regioncode=="620402" & year==1998

replace regioncode="620403" if regioncode=="620403" & year==1998

replace regioncode="620421" if regioncode=="620421" & year==1998

replace regioncode="620422" if regioncode=="620422" & year==1998

replace regioncode="620423" if regioncode=="620423" & year==1998

replace regioncode="620502" if regioncode=="620502" & year==1998

replace regioncode="620503" if regioncode=="620503" & year==1998

replace regioncode="620521" if regioncode=="620521" & year==1998

replace regioncode="620522" if regioncode=="620522" & year==1998

replace regioncode="620523" if regioncode=="620523" & year==1998

replace regioncode="620524" if regioncode=="620524" & year==1998

replace regioncode="620525" if regioncode=="620525" & year==1998

replace regioncode="622301" if regioncode=="622301" & year==1998

replace regioncode="622322" if regioncode=="622322" & year==1998

replace regioncode="622323" if regioncode=="622323" & year==1998

replace regioncode="622326" if regioncode=="622326" & year==1998

replace regioncode="622201" if regioncode=="622201" & year==1998

replace regioncode="622222" if regioncode=="622222" & year==1998

replace regioncode="622223" if regioncode=="622223" & year==1998

replace regioncode="622224" if regioncode=="622224" & year==1998

replace regioncode="622225" if regioncode=="622225" & year==1998

replace regioncode="622226" if regioncode=="622226" & year==1998

replace regioncode="622701" if regioncode=="622701" & year==1998

replace regioncode="622722" if regioncode=="622722" & year==1998

replace regioncode="622723" if regioncode=="622723" & year==1998

replace regioncode="622724" if regioncode=="622724" & year==1998

replace regioncode="622725" if regioncode=="622725" & year==1998

replace regioncode="622726" if regioncode=="622726" & year==1998

replace regioncode="622727" if regioncode=="622727" & year==1998

replace regioncode="622102" if regioncode=="622102" & year==1998

replace regioncode="622123" if regioncode=="622123" & year==1998

replace regioncode="622126" if regioncode=="622126" & year==1998

replace regioncode="622124" if regioncode=="622124" & year==1998

replace regioncode="622125" if regioncode=="622125" & year==1998

replace regioncode="622101" if regioncode=="622101" & year==1998

replace regioncode="622103" if regioncode=="622103" & year==1998

replace regioncode="622801" if regioncode=="622801" & year==1998

replace regioncode="622821" if regioncode=="622821" & year==1998

replace regioncode="622822" if regioncode=="622822" & year==1998

replace regioncode="622823" if regioncode=="622823" & year==1998

replace regioncode="622824" if regioncode=="622824" & year==1998

replace regioncode="622825" if regioncode=="622825" & year==1998

replace regioncode="622826" if regioncode=="622826" & year==1998

replace regioncode="622827" if regioncode=="622827" & year==1998

replace regioncode="622421" if regioncode=="622421" & year==1998

replace regioncode="622424" if regioncode=="622424" & year==1998

replace regioncode="622425" if regioncode=="622425" & year==1998

replace regioncode="622426" if regioncode=="622426" & year==1998

replace regioncode="622427" if regioncode=="622427" & year==1998

replace regioncode="622428" if regioncode=="622428" & year==1998

replace regioncode="622429" if regioncode=="622429" & year==1998

replace regioncode="622621" if regioncode=="622621" & year==1998

replace regioncode="622624" if regioncode=="622624" & year==1998

replace regioncode="622626" if regioncode=="622626" & year==1998

replace regioncode="622623" if regioncode=="622623" & year==1998

replace regioncode="622625" if regioncode=="622625" & year==1998

replace regioncode="622627" if regioncode=="622627" & year==1998

replace regioncode="622628" if regioncode=="622628" & year==1998

replace regioncode="622630" if regioncode=="622630" & year==1998

replace regioncode="622629" if regioncode=="622629" & year==1998

replace regioncode="622901" if regioncode=="622901" & year==1998

replace regioncode="622921" if regioncode=="622921" & year==1998

replace regioncode="622922" if regioncode=="622922" & year==1998

replace regioncode="622923" if regioncode=="622923" & year==1998

replace regioncode="622924" if regioncode=="622924" & year==1998

replace regioncode="622925" if regioncode=="622925" & year==1998

replace regioncode="622926" if regioncode=="622926" & year==1998

replace regioncode="622927" if regioncode=="622927" & year==1998

replace regioncode="623001" if regioncode=="623001" & year==1998

replace regioncode="623021" if regioncode=="623021" & year==1998

replace regioncode="623022" if regioncode=="623022" & year==1998

replace regioncode="623023" if regioncode=="623023" & year==1998

replace regioncode="623024" if regioncode=="623024" & year==1998

replace regioncode="623025" if regioncode=="623025" & year==1998

replace regioncode="623026" if regioncode=="623026" & year==1998

replace regioncode="623027" if regioncode=="623027" & year==1998

replace regioncode="630102" if regioncode=="630102" & year==1998

replace regioncode="630103" if regioncode=="630103" & year==1998

replace regioncode="630104" if regioncode=="630104" & year==1998

replace regioncode="630105" if regioncode=="630105" & year==1998

replace regioncode="630121" if regioncode=="630121" & year==1998

replace regioncode="630122" if regioncode=="632124" & year==1998

replace regioncode="630123" if regioncode=="632125" & year==1998

replace regioncode="632121" if regioncode=="632121" & year==1998

replace regioncode="632122" if regioncode=="632122" & year==1998

replace regioncode="632123" if regioncode=="632123" & year==1998

replace regioncode="632126" if regioncode=="632126" & year==1998

replace regioncode="632127" if regioncode=="632127" & year==1998

replace regioncode="632128" if regioncode=="632128" & year==1998

replace regioncode="632221" if regioncode=="632221" & year==1998

replace regioncode="632222" if regioncode=="632222" & year==1998

replace regioncode="632223" if regioncode=="632223" & year==1998

replace regioncode="632224" if regioncode=="632224" & year==1998

replace regioncode="632321" if regioncode=="632321" & year==1998

replace regioncode="632322" if regioncode=="632322" & year==1998

replace regioncode="632521" if regioncode=="632521" & year==1998

replace regioncode="632522" if regioncode=="632522" & year==1998

replace regioncode="632523" if regioncode=="632523" & year==1998

replace regioncode="632524" if regioncode=="632524" & year==1998

replace regioncode="632525" if regioncode=="632525" & year==1998

replace regioncode="632621" if regioncode=="632621" & year==1998

replace regioncode="632622" if regioncode=="632622" & year==1998

replace regioncode="632623" if regioncode=="632623" & year==1998

replace regioncode="632624" if regioncode=="632624" & year==1998

replace regioncode="632625" if regioncode=="632625" & year==1998

replace regioncode="632626" if regioncode=="632626" & year==1998

replace regioncode="632721" if regioncode=="632721" & year==1998

replace regioncode="632723" if regioncode=="632723" & year==1998

replace regioncode="632724" if regioncode=="632724" & year==1998

replace regioncode="632725" if regioncode=="632725" & year==1998

replace regioncode="632726" if regioncode=="632726" & year==1998

replace regioncode="632801" if regioncode=="632801" & year==1998

replace regioncode="632802" if regioncode=="632802" & year==1998

replace regioncode="632821" if regioncode=="632821" & year==1998

replace regioncode="632822" if regioncode=="632822" & year==1998

replace regioncode="640104" if regioncode=="640102" & year==1998

replace regioncode="640105" if regioncode=="640103" & year==1998

replace regioncode="640106" if regioncode=="640111" & year==1998

replace regioncode="640121" if regioncode=="640121" & year==1998

replace regioncode="640122" if regioncode=="640122" & year==1998

replace regioncode="640181" if regioncode=="640382" & year==1998

replace regioncode="640202" if regioncode=="640202" & year==1998

replace regioncode="640203" if regioncode=="640203" & year==1998

replace regioncode="640221" if regioncode=="640221" & year==1998

replace regioncode="640302" if regioncode=="642101" & year==1998

replace regioncode="640321" if regioncode=="642123" & year==1998

replace regioncode="640322" if regioncode=="642124" & year==1998

replace regioncode="640323" if regioncode=="642126" & year==1998

replace regioncode="640324" if regioncode=="642127" & year==1998

replace regioncode="640381" if regioncode=="642102" & year==1998

replace regioncode="640402" if regioncode=="642221" & year==1998

replace regioncode="640421" if regioncode=="642222" & year==1998

replace regioncode="640422" if regioncode=="642223" & year==1998

replace regioncode="640423" if regioncode=="642224" & year==1998

replace regioncode="640424" if regioncode=="642225" & year==1998

replace regioncode="640425" if regioncode=="642226" & year==1998

replace regioncode="650102" if regioncode=="650102" & year==1998

replace regioncode="650103" if regioncode=="650103" & year==1998

replace regioncode="650104" if regioncode=="650104" & year==1998

replace regioncode="650105" if regioncode=="650105" & year==1998

replace regioncode="650106" if regioncode=="650106" & year==1998

replace regioncode="650107" if regioncode=="650107" & year==1998

replace regioncode="650108" if regioncode=="650108" & year==1998

replace regioncode="650121" if regioncode=="650121" & year==1998

replace regioncode="650202" if regioncode=="650202" & year==1998

replace regioncode="650203" if regioncode=="650203" & year==1998

replace regioncode="650204" if regioncode=="650204" & year==1998

replace regioncode="650205" if regioncode=="650205" & year==1998

replace regioncode="652101" if regioncode=="652101" & year==1998

replace regioncode="652122" if regioncode=="652122" & year==1998

replace regioncode="652123" if regioncode=="652123" & year==1998

replace regioncode="652201" if regioncode=="652201" & year==1998

replace regioncode="652222" if regioncode=="652222" & year==1998

replace regioncode="652223" if regioncode=="652223" & year==1998

replace regioncode="652301" if regioncode=="652301" & year==1998

replace regioncode="652302" if regioncode=="652302" & year==1998

replace regioncode="652303" if regioncode=="652322" & year==1998

replace regioncode="652323" if regioncode=="652323" & year==1998

replace regioncode="652324" if regioncode=="652324" & year==1998

replace regioncode="652325" if regioncode=="652325" & year==1998

replace regioncode="652327" if regioncode=="652327" & year==1998

replace regioncode="652328" if regioncode=="652328" & year==1998

replace regioncode="652701" if regioncode=="652701" & year==1998

replace regioncode="652722" if regioncode=="652722" & year==1998

replace regioncode="652723" if regioncode=="652723" & year==1998

replace regioncode="652801" if regioncode=="652801" & year==1998

replace regioncode="652822" if regioncode=="652822" & year==1998

replace regioncode="652823" if regioncode=="652823" & year==1998

replace regioncode="652824" if regioncode=="652824" & year==1998

replace regioncode="652825" if regioncode=="652825" & year==1998

replace regioncode="652826" if regioncode=="652826" & year==1998

replace regioncode="652827" if regioncode=="652827" & year==1998

replace regioncode="652828" if regioncode=="652828" & year==1998

replace regioncode="652829" if regioncode=="652829" & year==1998

replace regioncode="652901" if regioncode=="652901" & year==1998

replace regioncode="652922" if regioncode=="652922" & year==1998

replace regioncode="652923" if regioncode=="652923" & year==1998

replace regioncode="652924" if regioncode=="652924" & year==1998

replace regioncode="652925" if regioncode=="652925" & year==1998

replace regioncode="652926" if regioncode=="652926" & year==1998

replace regioncode="652928" if regioncode=="652928" & year==1998

replace regioncode="652929" if regioncode=="652929" & year==1998

replace regioncode="653001" if regioncode=="653001" & year==1998

replace regioncode="653022" if regioncode=="653022" & year==1998

replace regioncode="653023" if regioncode=="653023" & year==1998

replace regioncode="653024" if regioncode=="653024" & year==1998

replace regioncode="653101" if regioncode=="653101" & year==1998

replace regioncode="653121" if regioncode=="653121" & year==1998

replace regioncode="653122" if regioncode=="653122" & year==1998

replace regioncode="653123" if regioncode=="653123" & year==1998

replace regioncode="653124" if regioncode=="653124" & year==1998

replace regioncode="653125" if regioncode=="653125" & year==1998

replace regioncode="653126" if regioncode=="653126" & year==1998

replace regioncode="653127" if regioncode=="653127" & year==1998

replace regioncode="653128" if regioncode=="653128" & year==1998

replace regioncode="653129" if regioncode=="653129" & year==1998

replace regioncode="653130" if regioncode=="653130" & year==1998

replace regioncode="653131" if regioncode=="653131" & year==1998

replace regioncode="653201" if regioncode=="653201" & year==1998

replace regioncode="653221" if regioncode=="653221" & year==1998

replace regioncode="653222" if regioncode=="653222" & year==1998

replace regioncode="653223" if regioncode=="653223" & year==1998

replace regioncode="653224" if regioncode=="653224" & year==1998

replace regioncode="653225" if regioncode=="653225" & year==1998

replace regioncode="653226" if regioncode=="653226" & year==1998

replace regioncode="653227" if regioncode=="653227" & year==1998

replace regioncode="654002" if regioncode=="654101" & year==1998

replace regioncode="654003" if regioncode=="654001" & year==1998

replace regioncode="654021" if regioncode=="654121" & year==1998

replace regioncode="654022" if regioncode=="654122" & year==1998

replace regioncode="654023" if regioncode=="654123" & year==1998

replace regioncode="654024" if regioncode=="654124" & year==1998

replace regioncode="654025" if regioncode=="654125" & year==1998

replace regioncode="654026" if regioncode=="654126" & year==1998

replace regioncode="654027" if regioncode=="654127" & year==1998

replace regioncode="654028" if regioncode=="654128" & year==1998

replace regioncode="654201" if regioncode=="654201" & year==1998

replace regioncode="654202" if regioncode=="654202" & year==1998

replace regioncode="654221" if regioncode=="654221" & year==1998

replace regioncode="654223" if regioncode=="654223" & year==1998

replace regioncode="654224" if regioncode=="654224" & year==1998

replace regioncode="654225" if regioncode=="654225" & year==1998

replace regioncode="654226" if regioncode=="654226" & year==1998

replace regioncode="654301" if regioncode=="654301" & year==1998

replace regioncode="654321" if regioncode=="654321" & year==1998

replace regioncode="654322" if regioncode=="654322" & year==1998

replace regioncode="654323" if regioncode=="654323" & year==1998

replace regioncode="654324" if regioncode=="654324" & year==1998

replace regioncode="654325" if regioncode=="654325" & year==1998

replace regioncode="654326" if regioncode=="654326" & year==1998

replace regioncode="659001" if regioncode=="659001" & year==1998

replace regioncode="110101" if regioncode=="110101" & year==1999

replace regioncode="110102" if regioncode=="110102" & year==1999

replace regioncode="110103" if regioncode=="110103" & year==1999

replace regioncode="110104" if regioncode=="110104" & year==1999

replace regioncode="110105" if regioncode=="110105" & year==1999

replace regioncode="110106" if regioncode=="110106" & year==1999

replace regioncode="110107" if regioncode=="110107" & year==1999

replace regioncode="110108" if regioncode=="110108" & year==1999

replace regioncode="110109" if regioncode=="110109" & year==1999

replace regioncode="110111" if regioncode=="110111" & year==1999

replace regioncode="110112" if regioncode=="110112" & year==1999

replace regioncode="110113" if regioncode=="110113" & year==1999

replace regioncode="110114" if regioncode=="110221" & year==1999

replace regioncode="110115" if regioncode=="110224" & year==1999

replace regioncode="110117" if regioncode=="110226" & year==1999

replace regioncode="110116" if regioncode=="110227" & year==1999

replace regioncode="110228" if regioncode=="110228" & year==1999

replace regioncode="110229" if regioncode=="110229" & year==1999

replace regioncode="120101" if regioncode=="120101" & year==1999

replace regioncode="120102" if regioncode=="120102" & year==1999

replace regioncode="120103" if regioncode=="120103" & year==1999

replace regioncode="120104" if regioncode=="120104" & year==1999

replace regioncode="120105" if regioncode=="120105" & year==1999

replace regioncode="120106" if regioncode=="120106" & year==1999

replace regioncode="120107" if regioncode=="120107" & year==1999

replace regioncode="120108" if regioncode=="120108" & year==1999

replace regioncode="120109" if regioncode=="120109" & year==1999

replace regioncode="120110" if regioncode=="120110" & year==1999

replace regioncode="120111" if regioncode=="120111" & year==1999

replace regioncode="120112" if regioncode=="120112" & year==1999

replace regioncode="120113" if regioncode=="120113" & year==1999

replace regioncode="120114" if regioncode=="120222" & year==1999

replace regioncode="120115" if regioncode=="120224" & year==1999

replace regioncode="120221" if regioncode=="120221" & year==1999

replace regioncode="120223" if regioncode=="120223" & year==1999

replace regioncode="120225" if regioncode=="120225" & year==1999

replace regioncode="130102" if regioncode=="130102" & year==1999

replace regioncode="130103" if regioncode=="130103" & year==1999

replace regioncode="130104" if regioncode=="130104" & year==1999

replace regioncode="130105" if regioncode=="130105" & year==1999

replace regioncode="130107" if regioncode=="130107" & year==1999

replace regioncode="130108" if regioncode=="130106" & year==1999

replace regioncode="130121" if regioncode=="130121" & year==1999

replace regioncode="130123" if regioncode=="130123" & year==1999

replace regioncode="130124" if regioncode=="130124" & year==1999

replace regioncode="130125" if regioncode=="130125" & year==1999

replace regioncode="130126" if regioncode=="130126" & year==1999

replace regioncode="130127" if regioncode=="130127" & year==1999

replace regioncode="130128" if regioncode=="130128" & year==1999

replace regioncode="130129" if regioncode=="130129" & year==1999

replace regioncode="130130" if regioncode=="130130" & year==1999

replace regioncode="130131" if regioncode=="130131" & year==1999

replace regioncode="130132" if regioncode=="130132" & year==1999

replace regioncode="130133" if regioncode=="130133" & year==1999

replace regioncode="130181" if regioncode=="130181" & year==1999

replace regioncode="130182" if regioncode=="130182" & year==1999

replace regioncode="130183" if regioncode=="130183" & year==1999

replace regioncode="130184" if regioncode=="130184" & year==1999

replace regioncode="130185" if regioncode=="130185" & year==1999

replace regioncode="130202" if regioncode=="130202" & year==1999

replace regioncode="130203" if regioncode=="130203" & year==1999

replace regioncode="130204" if regioncode=="130204" & year==1999

replace regioncode="130205" if regioncode=="130205" & year==1999

replace regioncode="130208" if regioncode=="130221" & year==1999

replace regioncode="130223" if regioncode=="130223" & year==1999

replace regioncode="130224" if regioncode=="130224" & year==1999

replace regioncode="130225" if regioncode=="130225" & year==1999

replace regioncode="130227" if regioncode=="130227" & year==1999

replace regioncode="130229" if regioncode=="130229" & year==1999

replace regioncode="130230" if regioncode=="130230" & year==1999

replace regioncode="130281" if regioncode=="130281" & year==1999

replace regioncode="130207" if regioncode=="130282" & year==1999

replace regioncode="130283" if regioncode=="130283" & year==1999

replace regioncode="130302" if regioncode=="130302" & year==1999

replace regioncode="130303" if regioncode=="130303" & year==1999

replace regioncode="130304" if regioncode=="130304" & year==1999

replace regioncode="130321" if regioncode=="130321" & year==1999

replace regioncode="130322" if regioncode=="130322" & year==1999

replace regioncode="130323" if regioncode=="130323" & year==1999

replace regioncode="130324" if regioncode=="130324" & year==1999

replace regioncode="130402" if regioncode=="130402" & year==1999

replace regioncode="130403" if regioncode=="130403" & year==1999

replace regioncode="130404" if regioncode=="130404" & year==1999

replace regioncode="130406" if regioncode=="130406" & year==1999

replace regioncode="130421" if regioncode=="130421" & year==1999

replace regioncode="130423" if regioncode=="130423" & year==1999

replace regioncode="130424" if regioncode=="130424" & year==1999

replace regioncode="130425" if regioncode=="130425" & year==1999

replace regioncode="130426" if regioncode=="130426" & year==1999

replace regioncode="130427" if regioncode=="130427" & year==1999

replace regioncode="130428" if regioncode=="130428" & year==1999

replace regioncode="130429" if regioncode=="130429" & year==1999

replace regioncode="130430" if regioncode=="130430" & year==1999

replace regioncode="130431" if regioncode=="130431" & year==1999

replace regioncode="130432" if regioncode=="130432" & year==1999

replace regioncode="130433" if regioncode=="130433" & year==1999

replace regioncode="130434" if regioncode=="130434" & year==1999

replace regioncode="130435" if regioncode=="130435" & year==1999

replace regioncode="130481" if regioncode=="130481" & year==1999

replace regioncode="130502" if regioncode=="130502" & year==1999

replace regioncode="130503" if regioncode=="130503" & year==1999

replace regioncode="130521" if regioncode=="130521" & year==1999

replace regioncode="130522" if regioncode=="130522" & year==1999

replace regioncode="130523" if regioncode=="130523" & year==1999

replace regioncode="130524" if regioncode=="130524" & year==1999

replace regioncode="130525" if regioncode=="130525" & year==1999

replace regioncode="130526" if regioncode=="130526" & year==1999

replace regioncode="130527" if regioncode=="130527" & year==1999

replace regioncode="130528" if regioncode=="130528" & year==1999

replace regioncode="130529" if regioncode=="130529" & year==1999

replace regioncode="130530" if regioncode=="130530" & year==1999

replace regioncode="130531" if regioncode=="130531" & year==1999

replace regioncode="130532" if regioncode=="130532" & year==1999

replace regioncode="130533" if regioncode=="130533" & year==1999

replace regioncode="130534" if regioncode=="130534" & year==1999

replace regioncode="130535" if regioncode=="130535" & year==1999

replace regioncode="130581" if regioncode=="130581" & year==1999

replace regioncode="130582" if regioncode=="130582" & year==1999

replace regioncode="130602" if regioncode=="130602" & year==1999

replace regioncode="130603" if regioncode=="130603" & year==1999

replace regioncode="130604" if regioncode=="130604" & year==1999

replace regioncode="130621" if regioncode=="130621" & year==1999

replace regioncode="130622" if regioncode=="130622" & year==1999

replace regioncode="130623" if regioncode=="130623" & year==1999

replace regioncode="130624" if regioncode=="130624" & year==1999

replace regioncode="130625" if regioncode=="130625" & year==1999

replace regioncode="130626" if regioncode=="130626" & year==1999

replace regioncode="130627" if regioncode=="130627" & year==1999

replace regioncode="130628" if regioncode=="130628" & year==1999

replace regioncode="130629" if regioncode=="130629" & year==1999

replace regioncode="130630" if regioncode=="130630" & year==1999

replace regioncode="130631" if regioncode=="130631" & year==1999

replace regioncode="130632" if regioncode=="130632" & year==1999

replace regioncode="130633" if regioncode=="130633" & year==1999

replace regioncode="130634" if regioncode=="130634" & year==1999

replace regioncode="130635" if regioncode=="130635" & year==1999

replace regioncode="130636" if regioncode=="130636" & year==1999

replace regioncode="130637" if regioncode=="130637" & year==1999

replace regioncode="130638" if regioncode=="130638" & year==1999

replace regioncode="130681" if regioncode=="130681" & year==1999

replace regioncode="130682" if regioncode=="130682" & year==1999

replace regioncode="130683" if regioncode=="130683" & year==1999

replace regioncode="130684" if regioncode=="130684" & year==1999

replace regioncode="130702" if regioncode=="130702" & year==1999

replace regioncode="130703" if regioncode=="130703" & year==1999

replace regioncode="130705" if regioncode=="130705" & year==1999

replace regioncode="130706" if regioncode=="130706" & year==1999

replace regioncode="130721" if regioncode=="130721" & year==1999

replace regioncode="130722" if regioncode=="130722" & year==1999

replace regioncode="130723" if regioncode=="130723" & year==1999

replace regioncode="130724" if regioncode=="130724" & year==1999

replace regioncode="130725" if regioncode=="130725" & year==1999

replace regioncode="130726" if regioncode=="130726" & year==1999

replace regioncode="130727" if regioncode=="130727" & year==1999

replace regioncode="130728" if regioncode=="130728" & year==1999

replace regioncode="130729" if regioncode=="130729" & year==1999

replace regioncode="130730" if regioncode=="130730" & year==1999

replace regioncode="130731" if regioncode=="130731" & year==1999

replace regioncode="130732" if regioncode=="130732" & year==1999

replace regioncode="130733" if regioncode=="130733" & year==1999

replace regioncode="130802" if regioncode=="130802" & year==1999

replace regioncode="130803" if regioncode=="130803" & year==1999

replace regioncode="130804" if regioncode=="130804" & year==1999

replace regioncode="130821" if regioncode=="130821" & year==1999

replace regioncode="130822" if regioncode=="130822" & year==1999

replace regioncode="130823" if regioncode=="130823" & year==1999

replace regioncode="130824" if regioncode=="130824" & year==1999

replace regioncode="130825" if regioncode=="130825" & year==1999

replace regioncode="130826" if regioncode=="130826" & year==1999

replace regioncode="130827" if regioncode=="130827" & year==1999

replace regioncode="130828" if regioncode=="130828" & year==1999

replace regioncode="130902" if regioncode=="130902" & year==1999

replace regioncode="130903" if regioncode=="130903" & year==1999

replace regioncode="130921" if regioncode=="130921" & year==1999

replace regioncode="130922" if regioncode=="130922" & year==1999

replace regioncode="130923" if regioncode=="130923" & year==1999

replace regioncode="130924" if regioncode=="130924" & year==1999

replace regioncode="130925" if regioncode=="130925" & year==1999

replace regioncode="130926" if regioncode=="130926" & year==1999

replace regioncode="130927" if regioncode=="130927" & year==1999

replace regioncode="130928" if regioncode=="130928" & year==1999

replace regioncode="130929" if regioncode=="130929" & year==1999

replace regioncode="130930" if regioncode=="130930" & year==1999

replace regioncode="130981" if regioncode=="130981" & year==1999

replace regioncode="130982" if regioncode=="130982" & year==1999

replace regioncode="130983" if regioncode=="130983" & year==1999

replace regioncode="130984" if regioncode=="130984" & year==1999

replace regioncode="131002" if regioncode=="131002" & year==1999

replace regioncode="131003" if regioncode=="131003" & year==1999

replace regioncode="131022" if regioncode=="131022" & year==1999

replace regioncode="131023" if regioncode=="131023" & year==1999

replace regioncode="131024" if regioncode=="131024" & year==1999

replace regioncode="131025" if regioncode=="131025" & year==1999

replace regioncode="131026" if regioncode=="131026" & year==1999

replace regioncode="131028" if regioncode=="131028" & year==1999

replace regioncode="131081" if regioncode=="131081" & year==1999

replace regioncode="131082" if regioncode=="131082" & year==1999

replace regioncode="131102" if regioncode=="131102" & year==1999

replace regioncode="131121" if regioncode=="131121" & year==1999

replace regioncode="131122" if regioncode=="131122" & year==1999

replace regioncode="131123" if regioncode=="131123" & year==1999

replace regioncode="131124" if regioncode=="131124" & year==1999

replace regioncode="131125" if regioncode=="131125" & year==1999

replace regioncode="131126" if regioncode=="131126" & year==1999

replace regioncode="131127" if regioncode=="131127" & year==1999

replace regioncode="131128" if regioncode=="131128" & year==1999

replace regioncode="131181" if regioncode=="131181" & year==1999

replace regioncode="131182" if regioncode=="131182" & year==1999

replace regioncode="140105" if regioncode=="140105" & year==1999

replace regioncode="140106" if regioncode=="140106" & year==1999

replace regioncode="140107" if regioncode=="140107" & year==1999

replace regioncode="140108" if regioncode=="140108" & year==1999

replace regioncode="140109" if regioncode=="140109" & year==1999

replace regioncode="140110" if regioncode=="140110" & year==1999

replace regioncode="140121" if regioncode=="140121" & year==1999

replace regioncode="140122" if regioncode=="140122" & year==1999

replace regioncode="140123" if regioncode=="140123" & year==1999

replace regioncode="140181" if regioncode=="140181" & year==1999

replace regioncode="140202" if regioncode=="140202" & year==1999

replace regioncode="140203" if regioncode=="140203" & year==1999

replace regioncode="140211" if regioncode=="140211" & year==1999

replace regioncode="140212" if regioncode=="140212" & year==1999

replace regioncode="140221" if regioncode=="140221" & year==1999

replace regioncode="140222" if regioncode=="140222" & year==1999

replace regioncode="140223" if regioncode=="140223" & year==1999

replace regioncode="140224" if regioncode=="140224" & year==1999

replace regioncode="140225" if regioncode=="140225" & year==1999

replace regioncode="140226" if regioncode=="140226" & year==1999

replace regioncode="140227" if regioncode=="140227" & year==1999

replace regioncode="140302" if regioncode=="140301" & year==1999

replace regioncode="140303" if regioncode=="140303" & year==1999

replace regioncode="140311" if regioncode=="140311" & year==1999

replace regioncode="140321" if regioncode=="140321" & year==1999

replace regioncode="140322" if regioncode=="140322" & year==1999

replace regioncode="140402" if regioncode=="140402" & year==1999

replace regioncode="140411" if regioncode=="140411" & year==1999

replace regioncode="140421" if regioncode=="140421" & year==1999

replace regioncode="140423" if regioncode=="140423" & year==1999

replace regioncode="140424" if regioncode=="140424" & year==1999

replace regioncode="140425" if regioncode=="140425" & year==1999

replace regioncode="140426" if regioncode=="140426" & year==1999

replace regioncode="140427" if regioncode=="140427" & year==1999

replace regioncode="140428" if regioncode=="140428" & year==1999

replace regioncode="140429" if regioncode=="140429" & year==1999

replace regioncode="140430" if regioncode=="140430" & year==1999

replace regioncode="140431" if regioncode=="140431" & year==1999

replace regioncode="140481" if regioncode=="140481" & year==1999

replace regioncode="140502" if regioncode=="140502" & year==1999

replace regioncode="140521" if regioncode=="140521" & year==1999

replace regioncode="140522" if regioncode=="140522" & year==1999

replace regioncode="140524" if regioncode=="140524" & year==1999

replace regioncode="140525" if regioncode=="140525" & year==1999

replace regioncode="140581" if regioncode=="140581" & year==1999

replace regioncode="140602" if regioncode=="140602" & year==1999

replace regioncode="140603" if regioncode=="140603" & year==1999

replace regioncode="140621" if regioncode=="140621" & year==1999

replace regioncode="140622" if regioncode=="140622" & year==1999

replace regioncode="140623" if regioncode=="140623" & year==1999

replace regioncode="140624" if regioncode=="140624" & year==1999

replace regioncode="140902" if regioncode=="142201" & year==1999

replace regioncode="140981" if regioncode=="142202" & year==1999

replace regioncode="140921" if regioncode=="142222" & year==1999

replace regioncode="140922" if regioncode=="142223" & year==1999

replace regioncode="140923" if regioncode=="142225" & year==1999

replace regioncode="140924" if regioncode=="142226" & year==1999

replace regioncode="140925" if regioncode=="142227" & year==1999

replace regioncode="140926" if regioncode=="142228" & year==1999

replace regioncode="140927" if regioncode=="142229" & year==1999

replace regioncode="140928" if regioncode=="142230" & year==1999

replace regioncode="140929" if regioncode=="142231" & year==1999

replace regioncode="140930" if regioncode=="142232" & year==1999

replace regioncode="140931" if regioncode=="142233" & year==1999

replace regioncode="140932" if regioncode=="142234" & year==1999

replace regioncode="141181" if regioncode=="142301" & year==1999

replace regioncode="141102" if regioncode=="142302" & year==1999

replace regioncode="141182" if regioncode=="142303" & year==1999

replace regioncode="141121" if regioncode=="142322" & year==1999

replace regioncode="141122" if regioncode=="142323" & year==1999

replace regioncode="141123" if regioncode=="142325" & year==1999

replace regioncode="141124" if regioncode=="142326" & year==1999

replace regioncode="141125" if regioncode=="142327" & year==1999

replace regioncode="141126" if regioncode=="142328" & year==1999

replace regioncode="141127" if regioncode=="142329" & year==1999

replace regioncode="141128" if regioncode=="142330" & year==1999

replace regioncode="141129" if regioncode=="142332" & year==1999

replace regioncode="141130" if regioncode=="142333" & year==1999

replace regioncode="140702" if regioncode=="142401" & year==1999

replace regioncode="140781" if regioncode=="142402" & year==1999

replace regioncode="140721" if regioncode=="142421" & year==1999

replace regioncode="140722" if regioncode=="142422" & year==1999

replace regioncode="140723" if regioncode=="142423" & year==1999

replace regioncode="140724" if regioncode=="142424" & year==1999

replace regioncode="140725" if regioncode=="142427" & year==1999

replace regioncode="140726" if regioncode=="142429" & year==1999

replace regioncode="140727" if regioncode=="142430" & year==1999

replace regioncode="140728" if regioncode=="142431" & year==1999

replace regioncode="140729" if regioncode=="142433" & year==1999

replace regioncode="141002" if regioncode=="142601" & year==1999

replace regioncode="141081" if regioncode=="142602" & year==1999

replace regioncode="141082" if regioncode=="142603" & year==1999

replace regioncode="141021" if regioncode=="142621" & year==1999

replace regioncode="141022" if regioncode=="142622" & year==1999

replace regioncode="141023" if regioncode=="142623" & year==1999

replace regioncode="141024" if regioncode=="142625" & year==1999

replace regioncode="141025" if regioncode=="142627" & year==1999

replace regioncode="141026" if regioncode=="142628" & year==1999

replace regioncode="141027" if regioncode=="142629" & year==1999

replace regioncode="141028" if regioncode=="142630" & year==1999

replace regioncode="141029" if regioncode=="142631" & year==1999

replace regioncode="141033" if regioncode=="142632" & year==1999

replace regioncode="141030" if regioncode=="142633" & year==1999

replace regioncode="141032" if regioncode=="142634" & year==1999

replace regioncode="141031" if regioncode=="142635" & year==1999

replace regioncode="141034" if regioncode=="142636" & year==1999

replace regioncode="140802" if regioncode=="142701" & year==1999

replace regioncode="140881" if regioncode=="142702" & year==1999

replace regioncode="140882" if regioncode=="142703" & year==1999

replace regioncode="140830" if regioncode=="142723" & year==1999

replace regioncode="140821" if regioncode=="142724" & year==1999

replace regioncode="140822" if regioncode=="142725" & year==1999

replace regioncode="140825" if regioncode=="142726" & year==1999

replace regioncode="140824" if regioncode=="142727" & year==1999

replace regioncode="140823" if regioncode=="142729" & year==1999

replace regioncode="140828" if regioncode=="142730" & year==1999

replace regioncode="140826" if regioncode=="142731" & year==1999

replace regioncode="140829" if regioncode=="142732" & year==1999

replace regioncode="140827" if regioncode=="142733" & year==1999

replace regioncode="150102" if regioncode=="150102" & year==1999

replace regioncode="150103" if regioncode=="150103" & year==1999

replace regioncode="150104" if regioncode=="150104" & year==1999

replace regioncode="150105" if regioncode=="150105" & year==1999

replace regioncode="150121" if regioncode=="150121" & year==1999

replace regioncode="150122" if regioncode=="150122" & year==1999

replace regioncode="150123" if regioncode=="150123" & year==1999

replace regioncode="150124" if regioncode=="150124" & year==1999

replace regioncode="150125" if regioncode=="150125" & year==1999

replace regioncode="150202" if regioncode=="150202" & year==1999

replace regioncode="150203" if regioncode=="150203" & year==1999

replace regioncode="150204" if regioncode=="150204" & year==1999

replace regioncode="150205" if regioncode=="150205" & year==1999

replace regioncode="150206" if regioncode=="150206" & year==1999

replace regioncode="150207" if regioncode=="150207" & year==1999

replace regioncode="150221" if regioncode=="150221" & year==1999

replace regioncode="150222" if regioncode=="150222" & year==1999

replace regioncode="150223" if regioncode=="150223" & year==1999

replace regioncode="150302" if regioncode=="150302" & year==1999

replace regioncode="150303" if regioncode=="150303" & year==1999

replace regioncode="150304" if regioncode=="150304" & year==1999

replace regioncode="150402" if regioncode=="150402" & year==1999

replace regioncode="150403" if regioncode=="150403" & year==1999

replace regioncode="150404" if regioncode=="150404" & year==1999

replace regioncode="150421" if regioncode=="150421" & year==1999

replace regioncode="150422" if regioncode=="150422" & year==1999

replace regioncode="150423" if regioncode=="150423" & year==1999

replace regioncode="150424" if regioncode=="150424" & year==1999

replace regioncode="150425" if regioncode=="150425" & year==1999

replace regioncode="150426" if regioncode=="150426" & year==1999

replace regioncode="150428" if regioncode=="150428" & year==1999

replace regioncode="150429" if regioncode=="150429" & year==1999

replace regioncode="150430" if regioncode=="150430" & year==1999

replace regioncode="150702" if regioncode=="152101" & year==1999

replace regioncode="150781" if regioncode=="152102" & year==1999

replace regioncode="150783" if regioncode=="152103" & year==1999

replace regioncode="150782" if regioncode=="152104" & year==1999

replace regioncode="150785" if regioncode=="152105" & year==1999

replace regioncode="150784" if regioncode=="152106" & year==1999

replace regioncode="150721" if regioncode=="152122" & year==1999

replace regioncode="150722" if regioncode=="152123" & year==1999

replace regioncode="150723" if regioncode=="152127" & year==1999

replace regioncode="150724" if regioncode=="152128" & year==1999

replace regioncode="150727" if regioncode=="152129" & year==1999

replace regioncode="150726" if regioncode=="152130" & year==1999

replace regioncode="150725" if regioncode=="152131" & year==1999

replace regioncode="152201" if regioncode=="152201" & year==1999

replace regioncode="152202" if regioncode=="152202" & year==1999

replace regioncode="152221" if regioncode=="152221" & year==1999

replace regioncode="152222" if regioncode=="152222" & year==1999

replace regioncode="152223" if regioncode=="152223" & year==1999

replace regioncode="152224" if regioncode=="152224" & year==1999

replace regioncode="150502" if regioncode=="150502" & year==1999

replace regioncode="150581" if regioncode=="150581" & year==1999

replace regioncode="150521" if regioncode=="150521" & year==1999

replace regioncode="150522" if regioncode=="150522" & year==1999

replace regioncode="150523" if regioncode=="150523" & year==1999

replace regioncode="150524" if regioncode=="150524" & year==1999

replace regioncode="150525" if regioncode=="150525" & year==1999

replace regioncode="150526" if regioncode=="150526" & year==1999

replace regioncode="152501" if regioncode=="152501" & year==1999

replace regioncode="152502" if regioncode=="152502" & year==1999

replace regioncode="152522" if regioncode=="152522" & year==1999

replace regioncode="152523" if regioncode=="152523" & year==1999

replace regioncode="152524" if regioncode=="152524" & year==1999

replace regioncode="152525" if regioncode=="152525" & year==1999

replace regioncode="152526" if regioncode=="152526" & year==1999

replace regioncode="152527" if regioncode=="152527" & year==1999

replace regioncode="152528" if regioncode=="152528" & year==1999

replace regioncode="152529" if regioncode=="152529" & year==1999

replace regioncode="152530" if regioncode=="152530" & year==1999

replace regioncode="152531" if regioncode=="152531" & year==1999

replace regioncode="152601" if regioncode=="152601" & year==1999

replace regioncode="152602" if regioncode=="152602" & year==1999

replace regioncode="152624" if regioncode=="152624" & year==1999

replace regioncode="152625" if regioncode=="152625" & year==1999

replace regioncode="152626" if regioncode=="152626" & year==1999

replace regioncode="152627" if regioncode=="152627" & year==1999

replace regioncode="152629" if regioncode=="152629" & year==1999

replace regioncode="152630" if regioncode=="152630" & year==1999

replace regioncode="152631" if regioncode=="152631" & year==1999

replace regioncode="152632" if regioncode=="152632" & year==1999

replace regioncode="152634" if regioncode=="152634" & year==1999

replace regioncode="150602" if regioncode=="152701" & year==1999

replace regioncode="150621" if regioncode=="152722" & year==1999

replace regioncode="150622" if regioncode=="152723" & year==1999

replace regioncode="150623" if regioncode=="152724" & year==1999

replace regioncode="150624" if regioncode=="152725" & year==1999

replace regioncode="150625" if regioncode=="152726" & year==1999

replace regioncode="150626" if regioncode=="152727" & year==1999

replace regioncode="150627" if regioncode=="152728" & year==1999

replace regioncode="152801" if regioncode=="152801" & year==1999

replace regioncode="152822" if regioncode=="152822" & year==1999

replace regioncode="152823" if regioncode=="152823" & year==1999

replace regioncode="152824" if regioncode=="152824" & year==1999

replace regioncode="152825" if regioncode=="152825" & year==1999

replace regioncode="152826" if regioncode=="152826" & year==1999

replace regioncode="152827" if regioncode=="152827" & year==1999

replace regioncode="152921" if regioncode=="152921" & year==1999

replace regioncode="152922" if regioncode=="152922" & year==1999

replace regioncode="152923" if regioncode=="152923" & year==1999

replace regioncode="210102" if regioncode=="210102" & year==1999

replace regioncode="210103" if regioncode=="210103" & year==1999

replace regioncode="210104" if regioncode=="210104" & year==1999

replace regioncode="210105" if regioncode=="210105" & year==1999

replace regioncode="210106" if regioncode=="210106" & year==1999

replace regioncode="210111" if regioncode=="210111" & year==1999

replace regioncode="210112" if regioncode=="210112" & year==1999

replace regioncode="210113" if regioncode=="210113" & year==1999

replace regioncode="210114" if regioncode=="210114" & year==1999

replace regioncode="210122" if regioncode=="210122" & year==1999

replace regioncode="210123" if regioncode=="210123" & year==1999

replace regioncode="210124" if regioncode=="210124" & year==1999

replace regioncode="210181" if regioncode=="210181" & year==1999

replace regioncode="210202" if regioncode=="210202" & year==1999

replace regioncode="210203" if regioncode=="210203" & year==1999

replace regioncode="210204" if regioncode=="210204" & year==1999

replace regioncode="210211" if regioncode=="210211" & year==1999

replace regioncode="210212" if regioncode=="210212" & year==1999

replace regioncode="210213" if regioncode=="210213" & year==1999

replace regioncode="210224" if regioncode=="210224" & year==1999

replace regioncode="210281" if regioncode=="210281" & year==1999

replace regioncode="210282" if regioncode=="210282" & year==1999

replace regioncode="210283" if regioncode=="210283" & year==1999

replace regioncode="210302" if regioncode=="210302" & year==1999

replace regioncode="210303" if regioncode=="210303" & year==1999

replace regioncode="210304" if regioncode=="210304" & year==1999

replace regioncode="210311" if regioncode=="210311" & year==1999

replace regioncode="210321" if regioncode=="210321" & year==1999

replace regioncode="210323" if regioncode=="210323" & year==1999

replace regioncode="210381" if regioncode=="210381" & year==1999

replace regioncode="210402" if regioncode=="210402" & year==1999

replace regioncode="210403" if regioncode=="210403" & year==1999

replace regioncode="210404" if regioncode=="210404" & year==1999

replace regioncode="210411" if regioncode=="210411" & year==1999

replace regioncode="210421" if regioncode=="210421" & year==1999

replace regioncode="210422" if regioncode=="210422" & year==1999

replace regioncode="210423" if regioncode=="210423" & year==1999

replace regioncode="210502" if regioncode=="210502" & year==1999

replace regioncode="210503" if regioncode=="210503" & year==1999

replace regioncode="210504" if regioncode=="210504" & year==1999

replace regioncode="210505" if regioncode=="210505" & year==1999

replace regioncode="210521" if regioncode=="210521" & year==1999

replace regioncode="210522" if regioncode=="210522" & year==1999

replace regioncode="210602" if regioncode=="210602" & year==1999

replace regioncode="210603" if regioncode=="210603" & year==1999

replace regioncode="210604" if regioncode=="210604" & year==1999

replace regioncode="210624" if regioncode=="210624" & year==1999

replace regioncode="210681" if regioncode=="210681" & year==1999

replace regioncode="210682" if regioncode=="210682" & year==1999

replace regioncode="210702" if regioncode=="210702" & year==1999

replace regioncode="210703" if regioncode=="210703" & year==1999

replace regioncode="210711" if regioncode=="210711" & year==1999

replace regioncode="210726" if regioncode=="210726" & year==1999

replace regioncode="210727" if regioncode=="210727" & year==1999

replace regioncode="210781" if regioncode=="210781" & year==1999

replace regioncode="210782" if regioncode=="210782" & year==1999

replace regioncode="210802" if regioncode=="210802" & year==1999

replace regioncode="210803" if regioncode=="210803" & year==1999

replace regioncode="210804" if regioncode=="210804" & year==1999

replace regioncode="210811" if regioncode=="210811" & year==1999

replace regioncode="210881" if regioncode=="210881" & year==1999

replace regioncode="210882" if regioncode=="210882" & year==1999

replace regioncode="210902" if regioncode=="210902" & year==1999

replace regioncode="210903" if regioncode=="210903" & year==1999

replace regioncode="210904" if regioncode=="210904" & year==1999

replace regioncode="210905" if regioncode=="210905" & year==1999

replace regioncode="210911" if regioncode=="210911" & year==1999

replace regioncode="210921" if regioncode=="210921" & year==1999

replace regioncode="210922" if regioncode=="210922" & year==1999

replace regioncode="211002" if regioncode=="211002" & year==1999

replace regioncode="211003" if regioncode=="211003" & year==1999

replace regioncode="211004" if regioncode=="211004" & year==1999

replace regioncode="211005" if regioncode=="211005" & year==1999

replace regioncode="211011" if regioncode=="211011" & year==1999

replace regioncode="211021" if regioncode=="211021" & year==1999

replace regioncode="211081" if regioncode=="211081" & year==1999

replace regioncode="211102" if regioncode=="211102" & year==1999

replace regioncode="211103" if regioncode=="211103" & year==1999

replace regioncode="211121" if regioncode=="211121" & year==1999

replace regioncode="211122" if regioncode=="211122" & year==1999

replace regioncode="211202" if regioncode=="211202" & year==1999

replace regioncode="211204" if regioncode=="211204" & year==1999

replace regioncode="211221" if regioncode=="211221" & year==1999

replace regioncode="211223" if regioncode=="211223" & year==1999

replace regioncode="211224" if regioncode=="211224" & year==1999

replace regioncode="211281" if regioncode=="211281" & year==1999

replace regioncode="211282" if regioncode=="211282" & year==1999

replace regioncode="211302" if regioncode=="211302" & year==1999

replace regioncode="211303" if regioncode=="211303" & year==1999

replace regioncode="211321" if regioncode=="211321" & year==1999

replace regioncode="211322" if regioncode=="211322" & year==1999

replace regioncode="211324" if regioncode=="211324" & year==1999

replace regioncode="211381" if regioncode=="211381" & year==1999

replace regioncode="211382" if regioncode=="211382" & year==1999

replace regioncode="211402" if regioncode=="211402" & year==1999

replace regioncode="211403" if regioncode=="211403" & year==1999

replace regioncode="211404" if regioncode=="211404" & year==1999

replace regioncode="211421" if regioncode=="211421" & year==1999

replace regioncode="211422" if regioncode=="211422" & year==1999

replace regioncode="211481" if regioncode=="211481" & year==1999

replace regioncode="220102" if regioncode=="220102" & year==1999

replace regioncode="220103" if regioncode=="220103" & year==1999

replace regioncode="220104" if regioncode=="220104" & year==1999

replace regioncode="220105" if regioncode=="220105" & year==1999

replace regioncode="220106" if regioncode=="220106" & year==1999

replace regioncode="220112" if regioncode=="220112" & year==1999

replace regioncode="220122" if regioncode=="220122" & year==1999

replace regioncode="220181" if regioncode=="220181" & year==1999

replace regioncode="220182" if regioncode=="220182" & year==1999

replace regioncode="220183" if regioncode=="220183" & year==1999

replace regioncode="220202" if regioncode=="220202" & year==1999

replace regioncode="220203" if regioncode=="220203" & year==1999

replace regioncode="220204" if regioncode=="220204" & year==1999

replace regioncode="220211" if regioncode=="220205" & year==1999

replace regioncode="220221" if regioncode=="220221" & year==1999

replace regioncode="220281" if regioncode=="220281" & year==1999

replace regioncode="220282" if regioncode=="220282" & year==1999

replace regioncode="220283" if regioncode=="220283" & year==1999

replace regioncode="220284" if regioncode=="220284" & year==1999

replace regioncode="220302" if regioncode=="220302" & year==1999

replace regioncode="220303" if regioncode=="220303" & year==1999

replace regioncode="220322" if regioncode=="220322" & year==1999

replace regioncode="220323" if regioncode=="220323" & year==1999

replace regioncode="220381" if regioncode=="220381" & year==1999

replace regioncode="220382" if regioncode=="220382" & year==1999

replace regioncode="220402" if regioncode=="220402" & year==1999

replace regioncode="220403" if regioncode=="220403" & year==1999

replace regioncode="220421" if regioncode=="220421" & year==1999

replace regioncode="220422" if regioncode=="220422" & year==1999

replace regioncode="220502" if regioncode=="220502" & year==1999

replace regioncode="220503" if regioncode=="220503" & year==1999

replace regioncode="220521" if regioncode=="220521" & year==1999

replace regioncode="220523" if regioncode=="220523" & year==1999

replace regioncode="220524" if regioncode=="220524" & year==1999

replace regioncode="220581" if regioncode=="220581" & year==1999

replace regioncode="220582" if regioncode=="220582" & year==1999

replace regioncode="220602" if regioncode=="220602" & year==1999

replace regioncode="220621" if regioncode=="220621" & year==1999

replace regioncode="220622" if regioncode=="220622" & year==1999

replace regioncode="220623" if regioncode=="220623" & year==1999

replace regioncode="220625" if regioncode=="220624" & year==1999

replace regioncode="220681" if regioncode=="220681" & year==1999

replace regioncode="220702" if regioncode=="220702" & year==1999

replace regioncode="220721" if regioncode=="220721" & year==1999

replace regioncode="220722" if regioncode=="220722" & year==1999

replace regioncode="220723" if regioncode=="220723" & year==1999

replace regioncode="220724" if regioncode=="220724" & year==1999

replace regioncode="220802" if regioncode=="220802" & year==1999

replace regioncode="220821" if regioncode=="220821" & year==1999

replace regioncode="220822" if regioncode=="220822" & year==1999

replace regioncode="220881" if regioncode=="220881" & year==1999

replace regioncode="220882" if regioncode=="220882" & year==1999

replace regioncode="222401" if regioncode=="222401" & year==1999

replace regioncode="222402" if regioncode=="222402" & year==1999

replace regioncode="222403" if regioncode=="222403" & year==1999

replace regioncode="222404" if regioncode=="222404" & year==1999

replace regioncode="222405" if regioncode=="222405" & year==1999

replace regioncode="222406" if regioncode=="222406" & year==1999

replace regioncode="222424" if regioncode=="222424" & year==1999

replace regioncode="222426" if regioncode=="222426" & year==1999

replace regioncode="230102" if regioncode=="230102" & year==1999

replace regioncode="230103" if regioncode=="230103" & year==1999

replace regioncode="230104" if regioncode=="230104" & year==1999

replace regioncode="230105" if regioncode=="230105" & year==1999

replace regioncode="230107" if regioncode=="230107" & year==1999

replace regioncode="230108" if regioncode=="230108" & year==1999

replace regioncode="230121" if regioncode=="230121" & year==1999

replace regioncode="230123" if regioncode=="230123" & year==1999

replace regioncode="230124" if regioncode=="230124" & year==1999

replace regioncode="230125" if regioncode=="230125" & year==1999

replace regioncode="230126" if regioncode=="230126" & year==1999

replace regioncode="230127" if regioncode=="230127" & year==1999

replace regioncode="230128" if regioncode=="230128" & year==1999

replace regioncode="230129" if regioncode=="230129" & year==1999

replace regioncode="230181" if regioncode=="230181" & year==1999

replace regioncode="230182" if regioncode=="230182" & year==1999

replace regioncode="230183" if regioncode=="230183" & year==1999

replace regioncode="230184" if regioncode=="230184" & year==1999

replace regioncode="230202" if regioncode=="230202" & year==1999

replace regioncode="230203" if regioncode=="230203" & year==1999

replace regioncode="230204" if regioncode=="230204" & year==1999

replace regioncode="230205" if regioncode=="230205" & year==1999

replace regioncode="230206" if regioncode=="230206" & year==1999

replace regioncode="230207" if regioncode=="230207" & year==1999

replace regioncode="230208" if regioncode=="230208" & year==1999

replace regioncode="230221" if regioncode=="230221" & year==1999

replace regioncode="230223" if regioncode=="230223" & year==1999

replace regioncode="230224" if regioncode=="230224" & year==1999

replace regioncode="230225" if regioncode=="230225" & year==1999

replace regioncode="230227" if regioncode=="230227" & year==1999

replace regioncode="230229" if regioncode=="230229" & year==1999

replace regioncode="230230" if regioncode=="230230" & year==1999

replace regioncode="230231" if regioncode=="230231" & year==1999

replace regioncode="230281" if regioncode=="230281" & year==1999

replace regioncode="230302" if regioncode=="230302" & year==1999

replace regioncode="230303" if regioncode=="230303" & year==1999

replace regioncode="230304" if regioncode=="230304" & year==1999

replace regioncode="230305" if regioncode=="230305" & year==1999

replace regioncode="230306" if regioncode=="230306" & year==1999

replace regioncode="230307" if regioncode=="230307" & year==1999

replace regioncode="230321" if regioncode=="230321" & year==1999

replace regioncode="230381" if regioncode=="230381" & year==1999

replace regioncode="230382" if regioncode=="230382" & year==1999

replace regioncode="230402" if regioncode=="230402" & year==1999

replace regioncode="230403" if regioncode=="230403" & year==1999

replace regioncode="230404" if regioncode=="230404" & year==1999

replace regioncode="230405" if regioncode=="230405" & year==1999

replace regioncode="230406" if regioncode=="230406" & year==1999

replace regioncode="230407" if regioncode=="230407" & year==1999

replace regioncode="230421" if regioncode=="230421" & year==1999

replace regioncode="230422" if regioncode=="230422" & year==1999

replace regioncode="230502" if regioncode=="230502" & year==1999

replace regioncode="230503" if regioncode=="230503" & year==1999

replace regioncode="230505" if regioncode=="230505" & year==1999

replace regioncode="230506" if regioncode=="230506" & year==1999

replace regioncode="230521" if regioncode=="230521" & year==1999

replace regioncode="230522" if regioncode=="230522" & year==1999

replace regioncode="230523" if regioncode=="230523" & year==1999

replace regioncode="230524" if regioncode=="230524" & year==1999

replace regioncode="230602" if regioncode=="230602" & year==1999

replace regioncode="230603" if regioncode=="230603" & year==1999

replace regioncode="230604" if regioncode=="230604" & year==1999

replace regioncode="230605" if regioncode=="230605" & year==1999

replace regioncode="230606" if regioncode=="230606" & year==1999

replace regioncode="230621" if regioncode=="230621" & year==1999

replace regioncode="230622" if regioncode=="230622" & year==1999

replace regioncode="230623" if regioncode=="230623" & year==1999

replace regioncode="230624" if regioncode=="230624" & year==1999

replace regioncode="230702" if regioncode=="230702" & year==1999

replace regioncode="230703" if regioncode=="230703" & year==1999

replace regioncode="230704" if regioncode=="230704" & year==1999

replace regioncode="230705" if regioncode=="230705" & year==1999

replace regioncode="230706" if regioncode=="230706" & year==1999

replace regioncode="230707" if regioncode=="230707" & year==1999

replace regioncode="230708" if regioncode=="230708" & year==1999

replace regioncode="230709" if regioncode=="230709" & year==1999

replace regioncode="230710" if regioncode=="230710" & year==1999

replace regioncode="230711" if regioncode=="230711" & year==1999

replace regioncode="230712" if regioncode=="230712" & year==1999

replace regioncode="230713" if regioncode=="230713" & year==1999

replace regioncode="230714" if regioncode=="230714" & year==1999

replace regioncode="230715" if regioncode=="230715" & year==1999

replace regioncode="230716" if regioncode=="230716" & year==1999

replace regioncode="230722" if regioncode=="230722" & year==1999

replace regioncode="230781" if regioncode=="230781" & year==1999

replace regioncode="230802" if regioncode=="230802" & year==1999

replace regioncode="230803" if regioncode=="230803" & year==1999

replace regioncode="230804" if regioncode=="230804" & year==1999

replace regioncode="230805" if regioncode=="230805" & year==1999

replace regioncode="230811" if regioncode=="230811" & year==1999

replace regioncode="230822" if regioncode=="230822" & year==1999

replace regioncode="230826" if regioncode=="230826" & year==1999

replace regioncode="230828" if regioncode=="230828" & year==1999

replace regioncode="230833" if regioncode=="230833" & year==1999

replace regioncode="230881" if regioncode=="230881" & year==1999

replace regioncode="230882" if regioncode=="230882" & year==1999

replace regioncode="230902" if regioncode=="230902" & year==1999

replace regioncode="230903" if regioncode=="230903" & year==1999

replace regioncode="230904" if regioncode=="230904" & year==1999

replace regioncode="230921" if regioncode=="230921" & year==1999

replace regioncode="231002" if regioncode=="231002" & year==1999

replace regioncode="231003" if regioncode=="231003" & year==1999

replace regioncode="231004" if regioncode=="231004" & year==1999

replace regioncode="231005" if regioncode=="231005" & year==1999

replace regioncode="231024" if regioncode=="231024" & year==1999

replace regioncode="231025" if regioncode=="231025" & year==1999

replace regioncode="231081" if regioncode=="231081" & year==1999

replace regioncode="231083" if regioncode=="231083" & year==1999

replace regioncode="231084" if regioncode=="231084" & year==1999

replace regioncode="231085" if regioncode=="231085" & year==1999

replace regioncode="231102" if regioncode=="231102" & year==1999

replace regioncode="231121" if regioncode=="231121" & year==1999

replace regioncode="231123" if regioncode=="231123" & year==1999

replace regioncode="231124" if regioncode=="231124" & year==1999

replace regioncode="231181" if regioncode=="231181" & year==1999

replace regioncode="231182" if regioncode=="231182" & year==1999

replace regioncode="231202" if regioncode=="232301" & year==1999

replace regioncode="231221" if regioncode=="232324" & year==1999

replace regioncode="231222" if regioncode=="232325" & year==1999

replace regioncode="231223" if regioncode=="232326" & year==1999

replace regioncode="231224" if regioncode=="232330" & year==1999

replace regioncode="231225" if regioncode=="232331" & year==1999

replace regioncode="231226" if regioncode=="232332" & year==1999

replace regioncode="231281" if regioncode=="232302" & year==1999

replace regioncode="231282" if regioncode=="232303" & year==1999

replace regioncode="231283" if regioncode=="232304" & year==1999

replace regioncode="232701" if regioncode=="232701" & year==1999

replace regioncode="232702" if regioncode=="232702" & year==1999

replace regioncode="232703" if regioncode=="232703" & year==1999

replace regioncode="232704" if regioncode=="232704" & year==1999

replace regioncode="232721" if regioncode=="232721" & year==1999

replace regioncode="232722" if regioncode=="232722" & year==1999

replace regioncode="232723" if regioncode=="232723" & year==1999

replace regioncode="310101" if regioncode=="310101" & year==1999

replace regioncode="310103" if regioncode=="310103" & year==1999

replace regioncode="310104" if regioncode=="310104" & year==1999

replace regioncode="310105" if regioncode=="310105" & year==1999

replace regioncode="310106" if regioncode=="310106" & year==1999

replace regioncode="310107" if regioncode=="310107" & year==1999

replace regioncode="310108" if regioncode=="310108" & year==1999

replace regioncode="310109" if regioncode=="310109" & year==1999

replace regioncode="310110" if regioncode=="310110" & year==1999

replace regioncode="310112" if regioncode=="310112" & year==1999

replace regioncode="310113" if regioncode=="310113" & year==1999

replace regioncode="310114" if regioncode=="310114" & year==1999

replace regioncode="310115" if regioncode=="310115" & year==1999

replace regioncode="310116" if regioncode=="310116" & year==1999

replace regioncode="310117" if regioncode=="310117" & year==1999

replace regioncode="310118" if regioncode=="310118" & year==1999

replace regioncode="310119" if regioncode=="310225" & year==1999

replace regioncode="310120" if regioncode=="310226" & year==1999

replace regioncode="310230" if regioncode=="310230" & year==1999

replace regioncode="320102" if regioncode=="320102" & year==1999

replace regioncode="320103" if regioncode=="320103" & year==1999

replace regioncode="320104" if regioncode=="320104" & year==1999

replace regioncode="320105" if regioncode=="320105" & year==1999

replace regioncode="320106" if regioncode=="320106" & year==1999

replace regioncode="320107" if regioncode=="320107" & year==1999

replace regioncode="320111" if regioncode=="320111" & year==1999

replace regioncode="320113" if regioncode=="320113" & year==1999

replace regioncode="320114" if regioncode=="320114" & year==1999

replace regioncode="320115" if regioncode=="320121" & year==1999

replace regioncode="320116" if regioncode=="320123" & year==1999

replace regioncode="320124" if regioncode=="320124" & year==1999

replace regioncode="320125" if regioncode=="320125" & year==1999

replace regioncode="320202" if regioncode=="320202" & year==1999

replace regioncode="320203" if regioncode=="320203" & year==1999

replace regioncode="320204" if regioncode=="320204" & year==1999

replace regioncode="320205" if regioncode=="320283" & year==1999

replace regioncode="320211" if regioncode=="320211" & year==1999

replace regioncode="320281" if regioncode=="320281" & year==1999

replace regioncode="320282" if regioncode=="320282" & year==1999

replace regioncode="320302" if regioncode=="320302" & year==1999

replace regioncode="320303" if regioncode=="320303" & year==1999

replace regioncode="320304" if regioncode=="320304" & year==1999

replace regioncode="320305" if regioncode=="320305" & year==1999

replace regioncode="320311" if regioncode=="320311" & year==1999

replace regioncode="320321" if regioncode=="320321" & year==1999

replace regioncode="320322" if regioncode=="320322" & year==1999

replace regioncode="320323" if regioncode=="320323" & year==1999

replace regioncode="320324" if regioncode=="320324" & year==1999

replace regioncode="320381" if regioncode=="320381" & year==1999

replace regioncode="320382" if regioncode=="320382" & year==1999

replace regioncode="320402" if regioncode=="320402" & year==1999

replace regioncode="320404" if regioncode=="320404" & year==1999

replace regioncode="320405" if regioncode=="320405" & year==1999

replace regioncode="320411" if regioncode=="320411" & year==1999

replace regioncode="320412" if regioncode=="320483" & year==1999

replace regioncode="320481" if regioncode=="320481" & year==1999

replace regioncode="320482" if regioncode=="320482" & year==1999

replace regioncode="320502" if regioncode=="320502" & year==1999

replace regioncode="320503" if regioncode=="320503" & year==1999

replace regioncode="320504" if regioncode=="320504" & year==1999

replace regioncode="320505" if regioncode=="320511" & year==1999

replace regioncode="320581" if regioncode=="320581" & year==1999

replace regioncode="320582" if regioncode=="320582" & year==1999

replace regioncode="320583" if regioncode=="320583" & year==1999

replace regioncode="320584" if regioncode=="320584" & year==1999

replace regioncode="320585" if regioncode=="320585" & year==1999

replace regioncode="320586" if regioncode=="320586" & year==1999

replace regioncode="320602" if regioncode=="320602" & year==1999

replace regioncode="320611" if regioncode=="320611" & year==1999

replace regioncode="320621" if regioncode=="320621" & year==1999

replace regioncode="320623" if regioncode=="320623" & year==1999

replace regioncode="320681" if regioncode=="320681" & year==1999

replace regioncode="320682" if regioncode=="320682" & year==1999

replace regioncode="320683" if regioncode=="320683" & year==1999

replace regioncode="320684" if regioncode=="320684" & year==1999

replace regioncode="320703" if regioncode=="320703" & year==1999

replace regioncode="320705" if regioncode=="320705" & year==1999

replace regioncode="320706" if regioncode=="320706" & year==1999

replace regioncode="320721" if regioncode=="320721" & year==1999

replace regioncode="320722" if regioncode=="320722" & year==1999

replace regioncode="320723" if regioncode=="320723" & year==1999

replace regioncode="320724" if regioncode=="320724" & year==1999

replace regioncode="320802" if regioncode=="320802" & year==1999

replace regioncode="320803" if regioncode=="320882" & year==1999

replace regioncode="320804" if regioncode=="320821" & year==1999

replace regioncode="320811" if regioncode=="320811" & year==1999

replace regioncode="320826" if regioncode=="320826" & year==1999

replace regioncode="320829" if regioncode=="320829" & year==1999

replace regioncode="320830" if regioncode=="320830" & year==1999

replace regioncode="320831" if regioncode=="320831" & year==1999

replace regioncode="320902" if regioncode=="320902" & year==1999

replace regioncode="320903" if regioncode=="320928" & year==1999

replace regioncode="320921" if regioncode=="320921" & year==1999

replace regioncode="320922" if regioncode=="320922" & year==1999

replace regioncode="320923" if regioncode=="320923" & year==1999

replace regioncode="320924" if regioncode=="320924" & year==1999

replace regioncode="320925" if regioncode=="320925" & year==1999

replace regioncode="320981" if regioncode=="320981" & year==1999

replace regioncode="320982" if regioncode=="320982" & year==1999

replace regioncode="321002" if regioncode=="321002" & year==1999

replace regioncode="321003" if regioncode=="321027" & year==1999

replace regioncode="321011" if regioncode=="321011" & year==1999

replace regioncode="321023" if regioncode=="321023" & year==1999

replace regioncode="321081" if regioncode=="321081" & year==1999

replace regioncode="321084" if regioncode=="321084" & year==1999

replace regioncode="321088" if regioncode=="321088" & year==1999

replace regioncode="321102" if regioncode=="321102" & year==1999

replace regioncode="321111" if regioncode=="321111" & year==1999

replace regioncode="321112" if regioncode=="321121" & year==1999

replace regioncode="321181" if regioncode=="321181" & year==1999

replace regioncode="321182" if regioncode=="321182" & year==1999

replace regioncode="321183" if regioncode=="321183" & year==1999

replace regioncode="321202" if regioncode=="321202" & year==1999

replace regioncode="321203" if regioncode=="321203" & year==1999

replace regioncode="321281" if regioncode=="321281" & year==1999

replace regioncode="321282" if regioncode=="321282" & year==1999

replace regioncode="321283" if regioncode=="321283" & year==1999

replace regioncode="321284" if regioncode=="321284" & year==1999

replace regioncode="321302" if regioncode=="321302" & year==1999

replace regioncode="321321" if regioncode=="321321" & year==1999

replace regioncode="321322" if regioncode=="321322" & year==1999

replace regioncode="321323" if regioncode=="321323" & year==1999

replace regioncode="321324" if regioncode=="321324" & year==1999

replace regioncode="330102" if regioncode=="330102" & year==1999

replace regioncode="330103" if regioncode=="330103" & year==1999

replace regioncode="330104" if regioncode=="330104" & year==1999

replace regioncode="330105" if regioncode=="330105" & year==1999

replace regioncode="330106" if regioncode=="330106" & year==1999

replace regioncode="330108" if regioncode=="330108" & year==1999

replace regioncode="330109" if regioncode=="330181" & year==1999

replace regioncode="330110" if regioncode=="330184" & year==1999

replace regioncode="330122" if regioncode=="330122" & year==1999

replace regioncode="330127" if regioncode=="330127" & year==1999

replace regioncode="330182" if regioncode=="330182" & year==1999

replace regioncode="330183" if regioncode=="330183" & year==1999

replace regioncode="330185" if regioncode=="330185" & year==1999

replace regioncode="330203" if regioncode=="330203" & year==1999

replace regioncode="330204" if regioncode=="330204" & year==1999

replace regioncode="330205" if regioncode=="330205" & year==1999

replace regioncode="330206" if regioncode=="330206" & year==1999

replace regioncode="330211" if regioncode=="330211" & year==1999

replace regioncode="330212" if regioncode=="330227" & year==1999

replace regioncode="330225" if regioncode=="330225" & year==1999

replace regioncode="330226" if regioncode=="330226" & year==1999

replace regioncode="330281" if regioncode=="330281" & year==1999

replace regioncode="330282" if regioncode=="330282" & year==1999

replace regioncode="330283" if regioncode=="330283" & year==1999

replace regioncode="330302" if regioncode=="330302" & year==1999

replace regioncode="330303" if regioncode=="330303" & year==1999

replace regioncode="330304" if regioncode=="330304" & year==1999

replace regioncode="330322" if regioncode=="330322" & year==1999

replace regioncode="330324" if regioncode=="330324" & year==1999

replace regioncode="330326" if regioncode=="330326" & year==1999

replace regioncode="330327" if regioncode=="330327" & year==1999

replace regioncode="330328" if regioncode=="330328" & year==1999

replace regioncode="330329" if regioncode=="330329" & year==1999

replace regioncode="330381" if regioncode=="330381" & year==1999

replace regioncode="330382" if regioncode=="330382" & year==1999

replace regioncode="330402" if regioncode=="330402" & year==1999

replace regioncode="330411" if regioncode=="330411" & year==1999

replace regioncode="330421" if regioncode=="330421" & year==1999

replace regioncode="330424" if regioncode=="330424" & year==1999

replace regioncode="330481" if regioncode=="330481" & year==1999

replace regioncode="330482" if regioncode=="330482" & year==1999

replace regioncode="330483" if regioncode=="330483" & year==1999

replace regioncode="330501" if regioncode=="330501" & year==1999

replace regioncode="330521" if regioncode=="330521" & year==1999

replace regioncode="330522" if regioncode=="330522" & year==1999

replace regioncode="330523" if regioncode=="330523" & year==1999

replace regioncode="330602" if regioncode=="330602" & year==1999

replace regioncode="330621" if regioncode=="330621" & year==1999

replace regioncode="330624" if regioncode=="330624" & year==1999

replace regioncode="330681" if regioncode=="330681" & year==1999

replace regioncode="330682" if regioncode=="330682" & year==1999

replace regioncode="330683" if regioncode=="330683" & year==1999

replace regioncode="330702" if regioncode=="330702" & year==1999

replace regioncode="330703" if regioncode=="330721" & year==1999

replace regioncode="330723" if regioncode=="330723" & year==1999

replace regioncode="330726" if regioncode=="330726" & year==1999

replace regioncode="330727" if regioncode=="330727" & year==1999

replace regioncode="330781" if regioncode=="330781" & year==1999

replace regioncode="330782" if regioncode=="330782" & year==1999

replace regioncode="330783" if regioncode=="330783" & year==1999

replace regioncode="330784" if regioncode=="330784" & year==1999

replace regioncode="330802" if regioncode=="330802" & year==1999

replace regioncode="330803" if regioncode=="330821" & year==1999

replace regioncode="330822" if regioncode=="330822" & year==1999

replace regioncode="330824" if regioncode=="330824" & year==1999

replace regioncode="330825" if regioncode=="330825" & year==1999

replace regioncode="330881" if regioncode=="330881" & year==1999

replace regioncode="330902" if regioncode=="330902" & year==1999

replace regioncode="330903" if regioncode=="330903" & year==1999

replace regioncode="330921" if regioncode=="330921" & year==1999

replace regioncode="330922" if regioncode=="330922" & year==1999

replace regioncode="331002" if regioncode=="331002" & year==1999

replace regioncode="331003" if regioncode=="331003" & year==1999

replace regioncode="331004" if regioncode=="331004" & year==1999

replace regioncode="331021" if regioncode=="331021" & year==1999

replace regioncode="331022" if regioncode=="331022" & year==1999

replace regioncode="331023" if regioncode=="331023" & year==1999

replace regioncode="331024" if regioncode=="331024" & year==1999

replace regioncode="331081" if regioncode=="331081" & year==1999

replace regioncode="331082" if regioncode=="331082" & year==1999

replace regioncode="331102" if regioncode=="332501" & year==1999

replace regioncode="331121" if regioncode=="332522" & year==1999

replace regioncode="331122" if regioncode=="332526" & year==1999

replace regioncode="331123" if regioncode=="332527" & year==1999

replace regioncode="331124" if regioncode=="332528" & year==1999

replace regioncode="331125" if regioncode=="332523" & year==1999

replace regioncode="331126" if regioncode=="332525" & year==1999

replace regioncode="331127" if regioncode=="332529" & year==1999

replace regioncode="331181" if regioncode=="332502" & year==1999

replace regioncode="340102" if regioncode=="340102" & year==1999

replace regioncode="340103" if regioncode=="340103" & year==1999

replace regioncode="340104" if regioncode=="340104" & year==1999

replace regioncode="340111" if regioncode=="340111" & year==1999

replace regioncode="340121" if regioncode=="340121" & year==1999

replace regioncode="340122" if regioncode=="340122" & year==1999

replace regioncode="340123" if regioncode=="340123" & year==1999

replace regioncode="340202" if regioncode=="340202" & year==1999

replace regioncode="340203" if regioncode=="340203" & year==1999

replace regioncode="340204" if regioncode=="340204" & year==1999

replace regioncode="340207" if regioncode=="340207" & year==1999

replace regioncode="340221" if regioncode=="340221" & year==1999

replace regioncode="340222" if regioncode=="340222" & year==1999

replace regioncode="340223" if regioncode=="340223" & year==1999

replace regioncode="340302" if regioncode=="340302" & year==1999

replace regioncode="340303" if regioncode=="340303" & year==1999

replace regioncode="340304" if regioncode=="340304" & year==1999

replace regioncode="340311" if regioncode=="340311" & year==1999

replace regioncode="340321" if regioncode=="340321" & year==1999

replace regioncode="340322" if regioncode=="340322" & year==1999

replace regioncode="340323" if regioncode=="340323" & year==1999

replace regioncode="340402" if regioncode=="340402" & year==1999

replace regioncode="340403" if regioncode=="340403" & year==1999

replace regioncode="340404" if regioncode=="340404" & year==1999

replace regioncode="340405" if regioncode=="340405" & year==1999

replace regioncode="340406" if regioncode=="340406" & year==1999

replace regioncode="340421" if regioncode=="340421" & year==1999

replace regioncode="340502" if regioncode=="340502" & year==1999

replace regioncode="340503" if regioncode=="340503" & year==1999

replace regioncode="340504" if regioncode=="340504" & year==1999

replace regioncode="340521" if regioncode=="340521" & year==1999

replace regioncode="340602" if regioncode=="340602" & year==1999

replace regioncode="340603" if regioncode=="340603" & year==1999

replace regioncode="340604" if regioncode=="340604" & year==1999

replace regioncode="340621" if regioncode=="340621" & year==1999

replace regioncode="340702" if regioncode=="340702" & year==1999

replace regioncode="340703" if regioncode=="340703" & year==1999

replace regioncode="340711" if regioncode=="340711" & year==1999

replace regioncode="340721" if regioncode=="340721" & year==1999

replace regioncode="340802" if regioncode=="340802" & year==1999

replace regioncode="340803" if regioncode=="340803" & year==1999

replace regioncode="340811" if regioncode=="340811" & year==1999

replace regioncode="340822" if regioncode=="340822" & year==1999

replace regioncode="340823" if regioncode=="340823" & year==1999

replace regioncode="340824" if regioncode=="340824" & year==1999

replace regioncode="340825" if regioncode=="340825" & year==1999

replace regioncode="340826" if regioncode=="340826" & year==1999

replace regioncode="340827" if regioncode=="340827" & year==1999

replace regioncode="340828" if regioncode=="340828" & year==1999

replace regioncode="340881" if regioncode=="340881" & year==1999

replace regioncode="341002" if regioncode=="341002" & year==1999

replace regioncode="341003" if regioncode=="341003" & year==1999

replace regioncode="341004" if regioncode=="341004" & year==1999

replace regioncode="341021" if regioncode=="341021" & year==1999

replace regioncode="341022" if regioncode=="341022" & year==1999

replace regioncode="341023" if regioncode=="341023" & year==1999

replace regioncode="341024" if regioncode=="341024" & year==1999

replace regioncode="341102" if regioncode=="341102" & year==1999

replace regioncode="341103" if regioncode=="341103" & year==1999

replace regioncode="341122" if regioncode=="341122" & year==1999

replace regioncode="341124" if regioncode=="341124" & year==1999

replace regioncode="341125" if regioncode=="341125" & year==1999

replace regioncode="341126" if regioncode=="341126" & year==1999

replace regioncode="341181" if regioncode=="341181" & year==1999

replace regioncode="341182" if regioncode=="341182" & year==1999

replace regioncode="341202" if regioncode=="341202" & year==1999

replace regioncode="341203" if regioncode=="341203" & year==1999

replace regioncode="341204" if regioncode=="341204" & year==1999

replace regioncode="341221" if regioncode=="341221" & year==1999

replace regioncode="341222" if regioncode=="341222" & year==1999

replace regioncode="341621" if regioncode=="341223" & year==1999

replace regioncode="341622" if regioncode=="341224" & year==1999

replace regioncode="341225" if regioncode=="341225" & year==1999

replace regioncode="341226" if regioncode=="341226" & year==1999

replace regioncode="341623" if regioncode=="341227" & year==1999

replace regioncode="341282" if regioncode=="341282" & year==1999

replace regioncode="341302" if regioncode=="341302" & year==1999

replace regioncode="341321" if regioncode=="341321" & year==1999

replace regioncode="341322" if regioncode=="341322" & year==1999

replace regioncode="341323" if regioncode=="341323" & year==1999

replace regioncode="341324" if regioncode=="341324" & year==1999

replace regioncode="341402" if regioncode=="341402" & year==1999

replace regioncode="341421" if regioncode=="341421" & year==1999

replace regioncode="341422" if regioncode=="341422" & year==1999

replace regioncode="341423" if regioncode=="341423" & year==1999

replace regioncode="341424" if regioncode=="341424" & year==1999

replace regioncode="341521" if regioncode=="341522" & year==1999

replace regioncode="341522" if regioncode=="341523" & year==1999

replace regioncode="341523" if regioncode=="341525" & year==1999

replace regioncode="341524" if regioncode=="341526" & year==1999

replace regioncode="341525" if regioncode=="341527" & year==1999

replace regioncode="341802" if regioncode=="342501" & year==1999

replace regioncode="341881" if regioncode=="342502" & year==1999

replace regioncode="341821" if regioncode=="342522" & year==1999

replace regioncode="341822" if regioncode=="342523" & year==1999

replace regioncode="341823" if regioncode=="342529" & year==1999

replace regioncode="341825" if regioncode=="342530" & year==1999

replace regioncode="341824" if regioncode=="342531" & year==1999

replace regioncode="341702" if regioncode=="342901" & year==1999

replace regioncode="341721" if regioncode=="342921" & year==1999

replace regioncode="341722" if regioncode=="342922" & year==1999

replace regioncode="341723" if regioncode=="342923" & year==1999

replace regioncode="341502" if regioncode=="342401" & year==1999

replace regioncode="341602" if regioncode=="341602" & year==1999

replace regioncode="350102" if regioncode=="350102" & year==1999

replace regioncode="350103" if regioncode=="350103" & year==1999

replace regioncode="350104" if regioncode=="350104" & year==1999

replace regioncode="350105" if regioncode=="350105" & year==1999

replace regioncode="350111" if regioncode=="350111" & year==1999

replace regioncode="350121" if regioncode=="350121" & year==1999

replace regioncode="350122" if regioncode=="350122" & year==1999

replace regioncode="350123" if regioncode=="350123" & year==1999

replace regioncode="350124" if regioncode=="350124" & year==1999

replace regioncode="350125" if regioncode=="350125" & year==1999

replace regioncode="350128" if regioncode=="350128" & year==1999

replace regioncode="350181" if regioncode=="350181" & year==1999

replace regioncode="350182" if regioncode=="350182" & year==1999

replace regioncode="350203" if regioncode=="350203" & year==1999

replace regioncode="350205" if regioncode=="350205" & year==1999

replace regioncode="350206" if regioncode=="350206" & year==1999

replace regioncode="350211" if regioncode=="350211" & year==1999

replace regioncode="350212" if regioncode=="350221" & year==1999

replace regioncode="350213" if regioncode=="350213" & year==1999

replace regioncode="350302" if regioncode=="350302" & year==1999

replace regioncode="350303" if regioncode=="350303" & year==1999

replace regioncode="350321" if regioncode=="350321" & year==1999

replace regioncode="350322" if regioncode=="350322" & year==1999

replace regioncode="350402" if regioncode=="350402" & year==1999

replace regioncode="350403" if regioncode=="350403" & year==1999

replace regioncode="350421" if regioncode=="350421" & year==1999

replace regioncode="350423" if regioncode=="350423" & year==1999

replace regioncode="350424" if regioncode=="350424" & year==1999

replace regioncode="350425" if regioncode=="350425" & year==1999

replace regioncode="350426" if regioncode=="350426" & year==1999

replace regioncode="350427" if regioncode=="350427" & year==1999

replace regioncode="350428" if regioncode=="350428" & year==1999

replace regioncode="350429" if regioncode=="350429" & year==1999

replace regioncode="350430" if regioncode=="350430" & year==1999

replace regioncode="350481" if regioncode=="350481" & year==1999

replace regioncode="350502" if regioncode=="350502" & year==1999

replace regioncode="350503" if regioncode=="350503" & year==1999

replace regioncode="350504" if regioncode=="350504" & year==1999

replace regioncode="350505" if regioncode=="350505" & year==1999

replace regioncode="350521" if regioncode=="350521" & year==1999

replace regioncode="350524" if regioncode=="350524" & year==1999

replace regioncode="350525" if regioncode=="350525" & year==1999

replace regioncode="350526" if regioncode=="350526" & year==1999

replace regioncode="350581" if regioncode=="350581" & year==1999

replace regioncode="350582" if regioncode=="350582" & year==1999

replace regioncode="350583" if regioncode=="350583" & year==1999

replace regioncode="350602" if regioncode=="350602" & year==1999

replace regioncode="350603" if regioncode=="350603" & year==1999

replace regioncode="350622" if regioncode=="350622" & year==1999

replace regioncode="350623" if regioncode=="350623" & year==1999

replace regioncode="350624" if regioncode=="350624" & year==1999

replace regioncode="350625" if regioncode=="350625" & year==1999

replace regioncode="350626" if regioncode=="350626" & year==1999

replace regioncode="350627" if regioncode=="350627" & year==1999

replace regioncode="350628" if regioncode=="350628" & year==1999

replace regioncode="350629" if regioncode=="350629" & year==1999

replace regioncode="350681" if regioncode=="350681" & year==1999

replace regioncode="350702" if regioncode=="350702" & year==1999

replace regioncode="350721" if regioncode=="350721" & year==1999

replace regioncode="350722" if regioncode=="350722" & year==1999

replace regioncode="350723" if regioncode=="350723" & year==1999

replace regioncode="350724" if regioncode=="350724" & year==1999

replace regioncode="350725" if regioncode=="350725" & year==1999

replace regioncode="350781" if regioncode=="350781" & year==1999

replace regioncode="350782" if regioncode=="350782" & year==1999

replace regioncode="350783" if regioncode=="350783" & year==1999

replace regioncode="350784" if regioncode=="350784" & year==1999

replace regioncode="350802" if regioncode=="350802" & year==1999

replace regioncode="350821" if regioncode=="350821" & year==1999

replace regioncode="350822" if regioncode=="350822" & year==1999

replace regioncode="350823" if regioncode=="350823" & year==1999

replace regioncode="350824" if regioncode=="350824" & year==1999

replace regioncode="350825" if regioncode=="350825" & year==1999

replace regioncode="350881" if regioncode=="350881" & year==1999

replace regioncode="350902" if regioncode=="352201" & year==1999

replace regioncode="350981" if regioncode=="352202" & year==1999

replace regioncode="350982" if regioncode=="352203" & year==1999

replace regioncode="350921" if regioncode=="352225" & year==1999

replace regioncode="350922" if regioncode=="352227" & year==1999

replace regioncode="350923" if regioncode=="352228" & year==1999

replace regioncode="350924" if regioncode=="352229" & year==1999

replace regioncode="350925" if regioncode=="352230" & year==1999

replace regioncode="350926" if regioncode=="352231" & year==1999

replace regioncode="360102" if regioncode=="360102" & year==1999

replace regioncode="360103" if regioncode=="360103" & year==1999

replace regioncode="360104" if regioncode=="360104" & year==1999

replace regioncode="360105" if regioncode=="360105" & year==1999

replace regioncode="360111" if regioncode=="360111" & year==1999

replace regioncode="360121" if regioncode=="360121" & year==1999

replace regioncode="360122" if regioncode=="360122" & year==1999

replace regioncode="360123" if regioncode=="360123" & year==1999

replace regioncode="360124" if regioncode=="360124" & year==1999

replace regioncode="360202" if regioncode=="360202" & year==1999

replace regioncode="360203" if regioncode=="360203" & year==1999

replace regioncode="360222" if regioncode=="360222" & year==1999

replace regioncode="360281" if regioncode=="360281" & year==1999

replace regioncode="360302" if regioncode=="360302" & year==1999

replace regioncode="360313" if regioncode=="360313" & year==1999

replace regioncode="360321" if regioncode=="360321" & year==1999

replace regioncode="360322" if regioncode=="360322" & year==1999

replace regioncode="360323" if regioncode=="360323" & year==1999

replace regioncode="360402" if regioncode=="360402" & year==1999

replace regioncode="360403" if regioncode=="360403" & year==1999

replace regioncode="360421" if regioncode=="360421" & year==1999

replace regioncode="360423" if regioncode=="360423" & year==1999

replace regioncode="360424" if regioncode=="360424" & year==1999

replace regioncode="360425" if regioncode=="360425" & year==1999

replace regioncode="360426" if regioncode=="360426" & year==1999

replace regioncode="360427" if regioncode=="360427" & year==1999

replace regioncode="360428" if regioncode=="360428" & year==1999

replace regioncode="360429" if regioncode=="360429" & year==1999

replace regioncode="360430" if regioncode=="360430" & year==1999

replace regioncode="360481" if regioncode=="360481" & year==1999

replace regioncode="360502" if regioncode=="360502" & year==1999

replace regioncode="360521" if regioncode=="360521" & year==1999

replace regioncode="360602" if regioncode=="360602" & year==1999

replace regioncode="360622" if regioncode=="360622" & year==1999

replace regioncode="360681" if regioncode=="360681" & year==1999

replace regioncode="360702" if regioncode=="360702" & year==1999

replace regioncode="360721" if regioncode=="360721" & year==1999

replace regioncode="360722" if regioncode=="360722" & year==1999

replace regioncode="360723" if regioncode=="360723" & year==1999

replace regioncode="360724" if regioncode=="360724" & year==1999

replace regioncode="360725" if regioncode=="360725" & year==1999

replace regioncode="360726" if regioncode=="360726" & year==1999

replace regioncode="360727" if regioncode=="360727" & year==1999

replace regioncode="360728" if regioncode=="360728" & year==1999

replace regioncode="360729" if regioncode=="360729" & year==1999

replace regioncode="360730" if regioncode=="360730" & year==1999

replace regioncode="360731" if regioncode=="360731" & year==1999

replace regioncode="360732" if regioncode=="360732" & year==1999

replace regioncode="360733" if regioncode=="360733" & year==1999

replace regioncode="360734" if regioncode=="360734" & year==1999

replace regioncode="360735" if regioncode=="360735" & year==1999

replace regioncode="360781" if regioncode=="360781" & year==1999

replace regioncode="360782" if regioncode=="360782" & year==1999

replace regioncode="360802" if regioncode=="362401" & year==1999

replace regioncode="360821" if regioncode=="362421" & year==1999

replace regioncode="360822" if regioncode=="362422" & year==1999

replace regioncode="360823" if regioncode=="362423" & year==1999

replace regioncode="360824" if regioncode=="362424" & year==1999

replace regioncode="360825" if regioncode=="362425" & year==1999

replace regioncode="360826" if regioncode=="362426" & year==1999

replace regioncode="360827" if regioncode=="362427" & year==1999

replace regioncode="360828" if regioncode=="362428" & year==1999

replace regioncode="360829" if regioncode=="362429" & year==1999

replace regioncode="360830" if regioncode=="362430" & year==1999

replace regioncode="360881" if regioncode=="362432" & year==1999

replace regioncode="360902" if regioncode=="362201" & year==1999

replace regioncode="360921" if regioncode=="362226" & year==1999

replace regioncode="360922" if regioncode=="362227" & year==1999

replace regioncode="360923" if regioncode=="362228" & year==1999

replace regioncode="360924" if regioncode=="362229" & year==1999

replace regioncode="360925" if regioncode=="362232" & year==1999

replace regioncode="360926" if regioncode=="362233" & year==1999

replace regioncode="360981" if regioncode=="362202" & year==1999

replace regioncode="360982" if regioncode=="362203" & year==1999

replace regioncode="360983" if regioncode=="362204" & year==1999

replace regioncode="361002" if regioncode=="362502" & year==1999

replace regioncode="361021" if regioncode=="362522" & year==1999

replace regioncode="361022" if regioncode=="362523" & year==1999

replace regioncode="361023" if regioncode=="362524" & year==1999

replace regioncode="361024" if regioncode=="362525" & year==1999

replace regioncode="361025" if regioncode=="362526" & year==1999

replace regioncode="361026" if regioncode=="362527" & year==1999

replace regioncode="361027" if regioncode=="362528" & year==1999

replace regioncode="361028" if regioncode=="362529" & year==1999

replace regioncode="361029" if regioncode=="362531" & year==1999

replace regioncode="361030" if regioncode=="362532" & year==1999

replace regioncode="361102" if regioncode=="362301" & year==1999

replace regioncode="361121" if regioncode=="362321" & year==1999

replace regioncode="361122" if regioncode=="362322" & year==1999

replace regioncode="361123" if regioncode=="362323" & year==1999

replace regioncode="361124" if regioncode=="362324" & year==1999

replace regioncode="361125" if regioncode=="362325" & year==1999

replace regioncode="361126" if regioncode=="362326" & year==1999

replace regioncode="361127" if regioncode=="362329" & year==1999

replace regioncode="361128" if regioncode=="362330" & year==1999

replace regioncode="361129" if regioncode=="362331" & year==1999

replace regioncode="361130" if regioncode=="362334" & year==1999

replace regioncode="361181" if regioncode=="362302" & year==1999

replace regioncode="370102" if regioncode=="370102" & year==1999

replace regioncode="370103" if regioncode=="370103" & year==1999

replace regioncode="370104" if regioncode=="370104" & year==1999

replace regioncode="370105" if regioncode=="370105" & year==1999

replace regioncode="370112" if regioncode=="370112" & year==1999

replace regioncode="370113" if regioncode=="370123" & year==1999

replace regioncode="370124" if regioncode=="370124" & year==1999

replace regioncode="370125" if regioncode=="370125" & year==1999

replace regioncode="370126" if regioncode=="370126" & year==1999

replace regioncode="370181" if regioncode=="370181" & year==1999

replace regioncode="370202" if regioncode=="370202" & year==1999

replace regioncode="370203" if regioncode=="370203" & year==1999

replace regioncode="370205" if regioncode=="370205" & year==1999

replace regioncode="370211" if regioncode=="370211" & year==1999

replace regioncode="370212" if regioncode=="370212" & year==1999

replace regioncode="370213" if regioncode=="370213" & year==1999

replace regioncode="370214" if regioncode=="370214" & year==1999

replace regioncode="370281" if regioncode=="370281" & year==1999

replace regioncode="370282" if regioncode=="370282" & year==1999

replace regioncode="370283" if regioncode=="370283" & year==1999

replace regioncode="370284" if regioncode=="370284" & year==1999

replace regioncode="370285" if regioncode=="370285" & year==1999

replace regioncode="370302" if regioncode=="370302" & year==1999

replace regioncode="370303" if regioncode=="370303" & year==1999

replace regioncode="370304" if regioncode=="370304" & year==1999

replace regioncode="370305" if regioncode=="370305" & year==1999

replace regioncode="370306" if regioncode=="370306" & year==1999

replace regioncode="370321" if regioncode=="370321" & year==1999

replace regioncode="370322" if regioncode=="370322" & year==1999

replace regioncode="370323" if regioncode=="370323" & year==1999

replace regioncode="370402" if regioncode=="370402" & year==1999

replace regioncode="370403" if regioncode=="370403" & year==1999

replace regioncode="370404" if regioncode=="370404" & year==1999

replace regioncode="370405" if regioncode=="370405" & year==1999

replace regioncode="370406" if regioncode=="370406" & year==1999

replace regioncode="370481" if regioncode=="370481" & year==1999

replace regioncode="370502" if regioncode=="370502" & year==1999

replace regioncode="370503" if regioncode=="370503" & year==1999

replace regioncode="370521" if regioncode=="370521" & year==1999

replace regioncode="370522" if regioncode=="370522" & year==1999

replace regioncode="370523" if regioncode=="370523" & year==1999

replace regioncode="370602" if regioncode=="370602" & year==1999

replace regioncode="370611" if regioncode=="370611" & year==1999

replace regioncode="370612" if regioncode=="370612" & year==1999

replace regioncode="370613" if regioncode=="370613" & year==1999

replace regioncode="370634" if regioncode=="370634" & year==1999

replace regioncode="370681" if regioncode=="370681" & year==1999

replace regioncode="370682" if regioncode=="370682" & year==1999

replace regioncode="370683" if regioncode=="370683" & year==1999

replace regioncode="370684" if regioncode=="370684" & year==1999

replace regioncode="370685" if regioncode=="370685" & year==1999

replace regioncode="370686" if regioncode=="370686" & year==1999

replace regioncode="370687" if regioncode=="370687" & year==1999

replace regioncode="370702" if regioncode=="370702" & year==1999

replace regioncode="370703" if regioncode=="370703" & year==1999

replace regioncode="370704" if regioncode=="370704" & year==1999

replace regioncode="370705" if regioncode=="370705" & year==1999

replace regioncode="370724" if regioncode=="370724" & year==1999

replace regioncode="370725" if regioncode=="370725" & year==1999

replace regioncode="370781" if regioncode=="370781" & year==1999

replace regioncode="370782" if regioncode=="370782" & year==1999

replace regioncode="370783" if regioncode=="370783" & year==1999

replace regioncode="370784" if regioncode=="370784" & year==1999

replace regioncode="370785" if regioncode=="370785" & year==1999

replace regioncode="370786" if regioncode=="370786" & year==1999

replace regioncode="370802" if regioncode=="370802" & year==1999

replace regioncode="370811" if regioncode=="370811" & year==1999

replace regioncode="370826" if regioncode=="370826" & year==1999

replace regioncode="370827" if regioncode=="370827" & year==1999

replace regioncode="370828" if regioncode=="370828" & year==1999

replace regioncode="370829" if regioncode=="370829" & year==1999

replace regioncode="370830" if regioncode=="370830" & year==1999

replace regioncode="370831" if regioncode=="370831" & year==1999

replace regioncode="370832" if regioncode=="370832" & year==1999

replace regioncode="370881" if regioncode=="370881" & year==1999

replace regioncode="370882" if regioncode=="370882" & year==1999

replace regioncode="370883" if regioncode=="370883" & year==1999

replace regioncode="370902" if regioncode=="370902" & year==1999

replace regioncode="370903" if regioncode=="370911" & year==1999

replace regioncode="370921" if regioncode=="370921" & year==1999

replace regioncode="370923" if regioncode=="370923" & year==1999

replace regioncode="370982" if regioncode=="370982" & year==1999

replace regioncode="370983" if regioncode=="370983" & year==1999

replace regioncode="371002" if regioncode=="371002" & year==1999

replace regioncode="371081" if regioncode=="371081" & year==1999

replace regioncode="371082" if regioncode=="371082" & year==1999

replace regioncode="371083" if regioncode=="371083" & year==1999

replace regioncode="371102" if regioncode=="371102" & year==1999

replace regioncode="371121" if regioncode=="371121" & year==1999

replace regioncode="371122" if regioncode=="371122" & year==1999

replace regioncode="371202" if regioncode=="371202" & year==1999

replace regioncode="371203" if regioncode=="371203" & year==1999

replace regioncode="371302" if regioncode=="371302" & year==1999

replace regioncode="371311" if regioncode=="371311" & year==1999

replace regioncode="371312" if regioncode=="371312" & year==1999

replace regioncode="371321" if regioncode=="371321" & year==1999

replace regioncode="371322" if regioncode=="371322" & year==1999

replace regioncode="371323" if regioncode=="371323" & year==1999

replace regioncode="371324" if regioncode=="371324" & year==1999

replace regioncode="371325" if regioncode=="371325" & year==1999

replace regioncode="371326" if regioncode=="371326" & year==1999

replace regioncode="371327" if regioncode=="371327" & year==1999

replace regioncode="371328" if regioncode=="371328" & year==1999

replace regioncode="371329" if regioncode=="371329" & year==1999

replace regioncode="371402" if regioncode=="371402" & year==1999

replace regioncode="371421" if regioncode=="371421" & year==1999

replace regioncode="371422" if regioncode=="371422" & year==1999

replace regioncode="371423" if regioncode=="371423" & year==1999

replace regioncode="371424" if regioncode=="371424" & year==1999

replace regioncode="371425" if regioncode=="371425" & year==1999

replace regioncode="371426" if regioncode=="371426" & year==1999

replace regioncode="371427" if regioncode=="371427" & year==1999

replace regioncode="371428" if regioncode=="371428" & year==1999

replace regioncode="371481" if regioncode=="371481" & year==1999

replace regioncode="371482" if regioncode=="371482" & year==1999

replace regioncode="371502" if regioncode=="371502" & year==1999

replace regioncode="371521" if regioncode=="371521" & year==1999

replace regioncode="371522" if regioncode=="371522" & year==1999

replace regioncode="371523" if regioncode=="371523" & year==1999

replace regioncode="371524" if regioncode=="371524" & year==1999

replace regioncode="371525" if regioncode=="371525" & year==1999

replace regioncode="371526" if regioncode=="371526" & year==1999

replace regioncode="371581" if regioncode=="371581" & year==1999

replace regioncode="371602" if regioncode=="372301" & year==1999

replace regioncode="371621" if regioncode=="372321" & year==1999

replace regioncode="371622" if regioncode=="372323" & year==1999

replace regioncode="371623" if regioncode=="372324" & year==1999

replace regioncode="371624" if regioncode=="372325" & year==1999

replace regioncode="371625" if regioncode=="372328" & year==1999

replace regioncode="371626" if regioncode=="372330" & year==1999

replace regioncode="371702" if regioncode=="372901" & year==1999

replace regioncode="371721" if regioncode=="372922" & year==1999

replace regioncode="371722" if regioncode=="372925" & year==1999

replace regioncode="371723" if regioncode=="372924" & year==1999

replace regioncode="371724" if regioncode=="372926" & year==1999

replace regioncode="371725" if regioncode=="372928" & year==1999

replace regioncode="371726" if regioncode=="372929" & year==1999

replace regioncode="371727" if regioncode=="372923" & year==1999

replace regioncode="371728" if regioncode=="372930" & year==1999

replace regioncode="410102" if regioncode=="410102" & year==1999

replace regioncode="410103" if regioncode=="410103" & year==1999

replace regioncode="410104" if regioncode=="410104" & year==1999

replace regioncode="410105" if regioncode=="410105" & year==1999

replace regioncode="410106" if regioncode=="410106" & year==1999

replace regioncode="410108" if regioncode=="410108" & year==1999

replace regioncode="410122" if regioncode=="410122" & year==1999

replace regioncode="410181" if regioncode=="410181" & year==1999

replace regioncode="410182" if regioncode=="410182" & year==1999

replace regioncode="410183" if regioncode=="410183" & year==1999

replace regioncode="410184" if regioncode=="410184" & year==1999

replace regioncode="410185" if regioncode=="410185" & year==1999

replace regioncode="410202" if regioncode=="410202" & year==1999

replace regioncode="410203" if regioncode=="410203" & year==1999

replace regioncode="410204" if regioncode=="410204" & year==1999

replace regioncode="410205" if regioncode=="410205" & year==1999

replace regioncode="410211" if regioncode=="410211" & year==1999

replace regioncode="410221" if regioncode=="410221" & year==1999

replace regioncode="410222" if regioncode=="410222" & year==1999

replace regioncode="410223" if regioncode=="410223" & year==1999

replace regioncode="410224" if regioncode=="410224" & year==1999

replace regioncode="410225" if regioncode=="410225" & year==1999

replace regioncode="410302" if regioncode=="410302" & year==1999

replace regioncode="410303" if regioncode=="410303" & year==1999

replace regioncode="410304" if regioncode=="410304" & year==1999

replace regioncode="410305" if regioncode=="410305" & year==1999

replace regioncode="410306" if regioncode=="410306" & year==1999

replace regioncode="410307" if regioncode=="410311" & year==1999

replace regioncode="410322" if regioncode=="410322" & year==1999

replace regioncode="410323" if regioncode=="410323" & year==1999

replace regioncode="410324" if regioncode=="410324" & year==1999

replace regioncode="410325" if regioncode=="410325" & year==1999

replace regioncode="410326" if regioncode=="410326" & year==1999

replace regioncode="410327" if regioncode=="410327" & year==1999

replace regioncode="410328" if regioncode=="410328" & year==1999

replace regioncode="410329" if regioncode=="410329" & year==1999

replace regioncode="410381" if regioncode=="410381" & year==1999

replace regioncode="410402" if regioncode=="410402" & year==1999

replace regioncode="410403" if regioncode=="410403" & year==1999

replace regioncode="410404" if regioncode=="410404" & year==1999

replace regioncode="410411" if regioncode=="410411" & year==1999

replace regioncode="410421" if regioncode=="410421" & year==1999

replace regioncode="410422" if regioncode=="410422" & year==1999

replace regioncode="410423" if regioncode=="410423" & year==1999

replace regioncode="410425" if regioncode=="410425" & year==1999

replace regioncode="410481" if regioncode=="410481" & year==1999

replace regioncode="410482" if regioncode=="410482" & year==1999

replace regioncode="410502" if regioncode=="410502" & year==1999

replace regioncode="410503" if regioncode=="410503" & year==1999

replace regioncode="410504" if regioncode=="410504" & year==1999

replace regioncode="410511" if regioncode=="410511" & year==1999

replace regioncode="410522" if regioncode=="410522" & year==1999

replace regioncode="410523" if regioncode=="410523" & year==1999

replace regioncode="410526" if regioncode=="410526" & year==1999

replace regioncode="410527" if regioncode=="410527" & year==1999

replace regioncode="410581" if regioncode=="410581" & year==1999

replace regioncode="410602" if regioncode=="410602" & year==1999

replace regioncode="410603" if regioncode=="410603" & year==1999

replace regioncode="410611" if regioncode=="410611" & year==1999

replace regioncode="410621" if regioncode=="410621" & year==1999

replace regioncode="410622" if regioncode=="410622" & year==1999

replace regioncode="410702" if regioncode=="410702" & year==1999

replace regioncode="410703" if regioncode=="410703" & year==1999

replace regioncode="410704" if regioncode=="410704" & year==1999

replace regioncode="410711" if regioncode=="410711" & year==1999

replace regioncode="410721" if regioncode=="410721" & year==1999

replace regioncode="410724" if regioncode=="410724" & year==1999

replace regioncode="410725" if regioncode=="410725" & year==1999

replace regioncode="410726" if regioncode=="410726" & year==1999

replace regioncode="410727" if regioncode=="410727" & year==1999

replace regioncode="410728" if regioncode=="410728" & year==1999

replace regioncode="410781" if regioncode=="410781" & year==1999

replace regioncode="410782" if regioncode=="410782" & year==1999

replace regioncode="410802" if regioncode=="410802" & year==1999

replace regioncode="410803" if regioncode=="410803" & year==1999

replace regioncode="410804" if regioncode=="410804" & year==1999

replace regioncode="410811" if regioncode=="410811" & year==1999

replace regioncode="410821" if regioncode=="410821" & year==1999

replace regioncode="410822" if regioncode=="410822" & year==1999

replace regioncode="410823" if regioncode=="410823" & year==1999

replace regioncode="410825" if regioncode=="410825" & year==1999

replace regioncode="410881" if regioncode=="410881" & year==1999

replace regioncode="410882" if regioncode=="410882" & year==1999

replace regioncode="410883" if regioncode=="410883" & year==1999

replace regioncode="410902" if regioncode=="410902" & year==1999

replace regioncode="410922" if regioncode=="410922" & year==1999

replace regioncode="410923" if regioncode=="410923" & year==1999

replace regioncode="410926" if regioncode=="410926" & year==1999

replace regioncode="410927" if regioncode=="410927" & year==1999

replace regioncode="410928" if regioncode=="410928" & year==1999

replace regioncode="411002" if regioncode=="411002" & year==1999

replace regioncode="411023" if regioncode=="411023" & year==1999

replace regioncode="411024" if regioncode=="411024" & year==1999

replace regioncode="411025" if regioncode=="411025" & year==1999

replace regioncode="411081" if regioncode=="411081" & year==1999

replace regioncode="411082" if regioncode=="411082" & year==1999

replace regioncode="411102" if regioncode=="411102" & year==1999

replace regioncode="411123" if regioncode=="411123" & year==1999

replace regioncode="411121" if regioncode=="411121" & year==1999

replace regioncode="411122" if regioncode=="411122" & year==1999

replace regioncode="411202" if regioncode=="411202" & year==1999

replace regioncode="411221" if regioncode=="411221" & year==1999

replace regioncode="411222" if regioncode=="411222" & year==1999

replace regioncode="411224" if regioncode=="411224" & year==1999

replace regioncode="411281" if regioncode=="411281" & year==1999

replace regioncode="411282" if regioncode=="411282" & year==1999

replace regioncode="411302" if regioncode=="411302" & year==1999

replace regioncode="411303" if regioncode=="411303" & year==1999

replace regioncode="411321" if regioncode=="411321" & year==1999

replace regioncode="411322" if regioncode=="411322" & year==1999

replace regioncode="411323" if regioncode=="411323" & year==1999

replace regioncode="411324" if regioncode=="411324" & year==1999

replace regioncode="411325" if regioncode=="411325" & year==1999

replace regioncode="411326" if regioncode=="411326" & year==1999

replace regioncode="411327" if regioncode=="411327" & year==1999

replace regioncode="411328" if regioncode=="411328" & year==1999

replace regioncode="411329" if regioncode=="411329" & year==1999

replace regioncode="411330" if regioncode=="411330" & year==1999

replace regioncode="411381" if regioncode=="411381" & year==1999

replace regioncode="411402" if regioncode=="411402" & year==1999

replace regioncode="411403" if regioncode=="411403" & year==1999

replace regioncode="411421" if regioncode=="411421" & year==1999

replace regioncode="411422" if regioncode=="411422" & year==1999

replace regioncode="411423" if regioncode=="411423" & year==1999

replace regioncode="411424" if regioncode=="411424" & year==1999

replace regioncode="411425" if regioncode=="411425" & year==1999

replace regioncode="411426" if regioncode=="411426" & year==1999

replace regioncode="411481" if regioncode=="411481" & year==1999

replace regioncode="411502" if regioncode=="411502" & year==1999

replace regioncode="411503" if regioncode=="411503" & year==1999

replace regioncode="411521" if regioncode=="411521" & year==1999

replace regioncode="411522" if regioncode=="411522" & year==1999

replace regioncode="411523" if regioncode=="411523" & year==1999

replace regioncode="411524" if regioncode=="411524" & year==1999

replace regioncode="411525" if regioncode=="411525" & year==1999

replace regioncode="411526" if regioncode=="411526" & year==1999

replace regioncode="411527" if regioncode=="411527" & year==1999

replace regioncode="411528" if regioncode=="411528" & year==1999

replace regioncode="411602" if regioncode=="412701" & year==1999

replace regioncode="411621" if regioncode=="412721" & year==1999

replace regioncode="411622" if regioncode=="412722" & year==1999

replace regioncode="411623" if regioncode=="412723" & year==1999

replace regioncode="411624" if regioncode=="412728" & year==1999

replace regioncode="411625" if regioncode=="412726" & year==1999

replace regioncode="411626" if regioncode=="412727" & year==1999

replace regioncode="411627" if regioncode=="412724" & year==1999

replace regioncode="411628" if regioncode=="412725" & year==1999

replace regioncode="411681" if regioncode=="412702" & year==1999

replace regioncode="411702" if regioncode=="412801" & year==1999

replace regioncode="411721" if regioncode=="412824" & year==1999

replace regioncode="411722" if regioncode=="412825" & year==1999

replace regioncode="411723" if regioncode=="412827" & year==1999

replace regioncode="411724" if regioncode=="412829" & year==1999

replace regioncode="411725" if regioncode=="412821" & year==1999

replace regioncode="411726" if regioncode=="412822" & year==1999

replace regioncode="411727" if regioncode=="412826" & year==1999

replace regioncode="411728" if regioncode=="412823" & year==1999

replace regioncode="411729" if regioncode=="412828" & year==1999

replace regioncode="420102" if regioncode=="420102" & year==1999

replace regioncode="420103" if regioncode=="420103" & year==1999

replace regioncode="420104" if regioncode=="420104" & year==1999

replace regioncode="420105" if regioncode=="420105" & year==1999

replace regioncode="420106" if regioncode=="420106" & year==1999

replace regioncode="420107" if regioncode=="420107" & year==1999

replace regioncode="420111" if regioncode=="420111" & year==1999

replace regioncode="420112" if regioncode=="420112" & year==1999

replace regioncode="420113" if regioncode=="420113" & year==1999

replace regioncode="420114" if regioncode=="420114" & year==1999

replace regioncode="420115" if regioncode=="420115" & year==1999

replace regioncode="420116" if regioncode=="420123" & year==1999

replace regioncode="420117" if regioncode=="420124" & year==1999

replace regioncode="420202" if regioncode=="420202" & year==1999

replace regioncode="420203" if regioncode=="420203" & year==1999

replace regioncode="420204" if regioncode=="420204" & year==1999

replace regioncode="420205" if regioncode=="420205" & year==1999

replace regioncode="420222" if regioncode=="420222" & year==1999

replace regioncode="420281" if regioncode=="420281" & year==1999

replace regioncode="420302" if regioncode=="420302" & year==1999

replace regioncode="420303" if regioncode=="420303" & year==1999

replace regioncode="420321" if regioncode=="420321" & year==1999

replace regioncode="420322" if regioncode=="420322" & year==1999

replace regioncode="420323" if regioncode=="420323" & year==1999

replace regioncode="420324" if regioncode=="420324" & year==1999

replace regioncode="420325" if regioncode=="420325" & year==1999

replace regioncode="420381" if regioncode=="420381" & year==1999

replace regioncode="420502" if regioncode=="420502" & year==1999

replace regioncode="420503" if regioncode=="420503" & year==1999

replace regioncode="420504" if regioncode=="420504" & year==1999

replace regioncode="420505" if regioncode=="420505" & year==1999

replace regioncode="420506" if regioncode=="420521" & year==1999

replace regioncode="420525" if regioncode=="420525" & year==1999

replace regioncode="420526" if regioncode=="420526" & year==1999

replace regioncode="420527" if regioncode=="420527" & year==1999

replace regioncode="420528" if regioncode=="420528" & year==1999

replace regioncode="420529" if regioncode=="420529" & year==1999

replace regioncode="420581" if regioncode=="420581" & year==1999

replace regioncode="420582" if regioncode=="420582" & year==1999

replace regioncode="420583" if regioncode=="420583" & year==1999

replace regioncode="420602" if regioncode=="420602" & year==1999

replace regioncode="420606" if regioncode=="420606" & year==1999

replace regioncode="420607" if regioncode=="420607" & year==1999

replace regioncode="420624" if regioncode=="420624" & year==1999

replace regioncode="420625" if regioncode=="420625" & year==1999

replace regioncode="420626" if regioncode=="420626" & year==1999

replace regioncode="420682" if regioncode=="420682" & year==1999

replace regioncode="420683" if regioncode=="420683" & year==1999

replace regioncode="420684" if regioncode=="420684" & year==1999

replace regioncode="420702" if regioncode=="420702" & year==1999

replace regioncode="420703" if regioncode=="420703" & year==1999

replace regioncode="420704" if regioncode=="420704" & year==1999

replace regioncode="420802" if regioncode=="420802" & year==1999

replace regioncode="420804" if regioncode=="420804" & year==1999

replace regioncode="420821" if regioncode=="420821" & year==1999

replace regioncode="420822" if regioncode=="420822" & year==1999

replace regioncode="420881" if regioncode=="420881" & year==1999

replace regioncode="420902" if regioncode=="420902" & year==1999

replace regioncode="420921" if regioncode=="420921" & year==1999

replace regioncode="420922" if regioncode=="420922" & year==1999

replace regioncode="420923" if regioncode=="420923" & year==1999

replace regioncode="420981" if regioncode=="420981" & year==1999

replace regioncode="420982" if regioncode=="420982" & year==1999

replace regioncode="421381" if regioncode=="420983" & year==1999

replace regioncode="420984" if regioncode=="420984" & year==1999

replace regioncode="421002" if regioncode=="421002" & year==1999

replace regioncode="421003" if regioncode=="421003" & year==1999

replace regioncode="421022" if regioncode=="421022" & year==1999

replace regioncode="421023" if regioncode=="421023" & year==1999

replace regioncode="421024" if regioncode=="421024" & year==1999

replace regioncode="421081" if regioncode=="421081" & year==1999

replace regioncode="421083" if regioncode=="421083" & year==1999

replace regioncode="421087" if regioncode=="421087" & year==1999

replace regioncode="421102" if regioncode=="421102" & year==1999

replace regioncode="421121" if regioncode=="421121" & year==1999

replace regioncode="421122" if regioncode=="421122" & year==1999

replace regioncode="421123" if regioncode=="421123" & year==1999

replace regioncode="421124" if regioncode=="421124" & year==1999

replace regioncode="421125" if regioncode=="421125" & year==1999

replace regioncode="421126" if regioncode=="421126" & year==1999

replace regioncode="421127" if regioncode=="421127" & year==1999

replace regioncode="421181" if regioncode=="421181" & year==1999

replace regioncode="421182" if regioncode=="421182" & year==1999

replace regioncode="421202" if regioncode=="421202" & year==1999

replace regioncode="421221" if regioncode=="421221" & year==1999

replace regioncode="421222" if regioncode=="421222" & year==1999

replace regioncode="421223" if regioncode=="421223" & year==1999

replace regioncode="421224" if regioncode=="421224" & year==1999

replace regioncode="421281" if regioncode=="421281" & year==1999

replace regioncode="421302" if regioncode=="429001" & year==1999

replace regioncode="422801" if regioncode=="422801" & year==1999

replace regioncode="422802" if regioncode=="422802" & year==1999

replace regioncode="422822" if regioncode=="422822" & year==1999

replace regioncode="422823" if regioncode=="422823" & year==1999

replace regioncode="422825" if regioncode=="422825" & year==1999

replace regioncode="422826" if regioncode=="422826" & year==1999

replace regioncode="422827" if regioncode=="422827" & year==1999

replace regioncode="422828" if regioncode=="422828" & year==1999

replace regioncode="429004" if regioncode=="429004" & year==1999

replace regioncode="429005" if regioncode=="429005" & year==1999

replace regioncode="429006" if regioncode=="429006" & year==1999

replace regioncode="429021" if regioncode=="429021" & year==1999

replace regioncode="430102" if regioncode=="430102" & year==1999

replace regioncode="430103" if regioncode=="430103" & year==1999

replace regioncode="430104" if regioncode=="430104" & year==1999

replace regioncode="430105" if regioncode=="430105" & year==1999

replace regioncode="430111" if regioncode=="430111" & year==1999

replace regioncode="430121" if regioncode=="430121" & year==1999

replace regioncode="430122" if regioncode=="430122" & year==1999

replace regioncode="430124" if regioncode=="430124" & year==1999

replace regioncode="430181" if regioncode=="430181" & year==1999

replace regioncode="430202" if regioncode=="430202" & year==1999

replace regioncode="430203" if regioncode=="430203" & year==1999

replace regioncode="430204" if regioncode=="430204" & year==1999

replace regioncode="430211" if regioncode=="430211" & year==1999

replace regioncode="430221" if regioncode=="430221" & year==1999

replace regioncode="430223" if regioncode=="430223" & year==1999

replace regioncode="430224" if regioncode=="430224" & year==1999

replace regioncode="430225" if regioncode=="430225" & year==1999

replace regioncode="430281" if regioncode=="430281" & year==1999

replace regioncode="430302" if regioncode=="430302" & year==1999

replace regioncode="430304" if regioncode=="430304" & year==1999

replace regioncode="430321" if regioncode=="430321" & year==1999

replace regioncode="430381" if regioncode=="430381" & year==1999

replace regioncode="430382" if regioncode=="430382" & year==1999

replace regioncode="430405" if regioncode=="430402" & year==1999

replace regioncode="430406" if regioncode=="430403" & year==1999

replace regioncode="430407" if regioncode=="430404" & year==1999

replace regioncode="430408" if regioncode=="430411" & year==1999

replace regioncode="430412" if regioncode=="430412" & year==1999

replace regioncode="430421" if regioncode=="430421" & year==1999

replace regioncode="430422" if regioncode=="430422" & year==1999

replace regioncode="430423" if regioncode=="430423" & year==1999

replace regioncode="430424" if regioncode=="430424" & year==1999

replace regioncode="430426" if regioncode=="430426" & year==1999

replace regioncode="430481" if regioncode=="430481" & year==1999

replace regioncode="430482" if regioncode=="430482" & year==1999

replace regioncode="430502" if regioncode=="430502" & year==1999

replace regioncode="430503" if regioncode=="430503" & year==1999

replace regioncode="430511" if regioncode=="430511" & year==1999

replace regioncode="430521" if regioncode=="430521" & year==1999

replace regioncode="430522" if regioncode=="430522" & year==1999

replace regioncode="430523" if regioncode=="430523" & year==1999

replace regioncode="430524" if regioncode=="430524" & year==1999

replace regioncode="430525" if regioncode=="430525" & year==1999

replace regioncode="430527" if regioncode=="430527" & year==1999

replace regioncode="430528" if regioncode=="430528" & year==1999

replace regioncode="430529" if regioncode=="430529" & year==1999

replace regioncode="430581" if regioncode=="430581" & year==1999

replace regioncode="430602" if regioncode=="430602" & year==1999

replace regioncode="430603" if regioncode=="430603" & year==1999

replace regioncode="430611" if regioncode=="430611" & year==1999

replace regioncode="430621" if regioncode=="430621" & year==1999

replace regioncode="430623" if regioncode=="430623" & year==1999

replace regioncode="430624" if regioncode=="430624" & year==1999

replace regioncode="430626" if regioncode=="430626" & year==1999

replace regioncode="430681" if regioncode=="430681" & year==1999

replace regioncode="430682" if regioncode=="430682" & year==1999

replace regioncode="430702" if regioncode=="430702" & year==1999

replace regioncode="430703" if regioncode=="430703" & year==1999

replace regioncode="430721" if regioncode=="430721" & year==1999

replace regioncode="430722" if regioncode=="430722" & year==1999

replace regioncode="430723" if regioncode=="430723" & year==1999

replace regioncode="430724" if regioncode=="430724" & year==1999

replace regioncode="430725" if regioncode=="430725" & year==1999

replace regioncode="430726" if regioncode=="430726" & year==1999

replace regioncode="430781" if regioncode=="430781" & year==1999

replace regioncode="430802" if regioncode=="430802" & year==1999

replace regioncode="430811" if regioncode=="430811" & year==1999

replace regioncode="430821" if regioncode=="430821" & year==1999

replace regioncode="430822" if regioncode=="430822" & year==1999

replace regioncode="430902" if regioncode=="430902" & year==1999

replace regioncode="430903" if regioncode=="430903" & year==1999

replace regioncode="430921" if regioncode=="430921" & year==1999

replace regioncode="430922" if regioncode=="430922" & year==1999

replace regioncode="430923" if regioncode=="430923" & year==1999

replace regioncode="430981" if regioncode=="430981" & year==1999

replace regioncode="431002" if regioncode=="431002" & year==1999

replace regioncode="431003" if regioncode=="431003" & year==1999

replace regioncode="431021" if regioncode=="431021" & year==1999

replace regioncode="431022" if regioncode=="431022" & year==1999

replace regioncode="431023" if regioncode=="431023" & year==1999

replace regioncode="431024" if regioncode=="431024" & year==1999

replace regioncode="431025" if regioncode=="431025" & year==1999

replace regioncode="431026" if regioncode=="431026" & year==1999

replace regioncode="431027" if regioncode=="431027" & year==1999

replace regioncode="431028" if regioncode=="431028" & year==1999

replace regioncode="431081" if regioncode=="431081" & year==1999

replace regioncode="431102" if regioncode=="431102" & year==1999

replace regioncode="431103" if regioncode=="431103" & year==1999

replace regioncode="431121" if regioncode=="431121" & year==1999

replace regioncode="431122" if regioncode=="431122" & year==1999

replace regioncode="431123" if regioncode=="431123" & year==1999

replace regioncode="431124" if regioncode=="431124" & year==1999

replace regioncode="431125" if regioncode=="431125" & year==1999

replace regioncode="431126" if regioncode=="431126" & year==1999

replace regioncode="431127" if regioncode=="431127" & year==1999

replace regioncode="431128" if regioncode=="431128" & year==1999

replace regioncode="431129" if regioncode=="431129" & year==1999

replace regioncode="431202" if regioncode=="431202" & year==1999

replace regioncode="431221" if regioncode=="431221" & year==1999

replace regioncode="431222" if regioncode=="431222" & year==1999

replace regioncode="431223" if regioncode=="431223" & year==1999

replace regioncode="431224" if regioncode=="431224" & year==1999

replace regioncode="431225" if regioncode=="431225" & year==1999

replace regioncode="431226" if regioncode=="431226" & year==1999

replace regioncode="431227" if regioncode=="431227" & year==1999

replace regioncode="431228" if regioncode=="431228" & year==1999

replace regioncode="431229" if regioncode=="431229" & year==1999

replace regioncode="431230" if regioncode=="431230" & year==1999

replace regioncode="431281" if regioncode=="431281" & year==1999

replace regioncode="431302" if regioncode=="431302" & year==1999

replace regioncode="431321" if regioncode=="431321" & year==1999

replace regioncode="431322" if regioncode=="431322" & year==1999

replace regioncode="431381" if regioncode=="431381" & year==1999

replace regioncode="431382" if regioncode=="431382" & year==1999

replace regioncode="433101" if regioncode=="433101" & year==1999

replace regioncode="433122" if regioncode=="433122" & year==1999

replace regioncode="433123" if regioncode=="433123" & year==1999

replace regioncode="433124" if regioncode=="433124" & year==1999

replace regioncode="433125" if regioncode=="433125" & year==1999

replace regioncode="433126" if regioncode=="433126" & year==1999

replace regioncode="433127" if regioncode=="433127" & year==1999

replace regioncode="433130" if regioncode=="433130" & year==1999

replace regioncode="440102" if regioncode=="440102" & year==1999

replace regioncode="440103" if regioncode=="440103" & year==1999

replace regioncode="440104" if regioncode=="440104" & year==1999

replace regioncode="440105" if regioncode=="440105" & year==1999

replace regioncode="440106" if regioncode=="440106" & year==1999

replace regioncode="440107" if regioncode=="440107" & year==1999

replace regioncode="440111" if regioncode=="440111" & year==1999

replace regioncode="440112" if regioncode=="440112" & year==1999

replace regioncode="440113" if regioncode=="440181" & year==1999

replace regioncode="440114" if regioncode=="440182" & year==1999

replace regioncode="440183" if regioncode=="440183" & year==1999

replace regioncode="440184" if regioncode=="440184" & year==1999

replace regioncode="440203" if regioncode=="440203" & year==1999

replace regioncode="440204" if regioncode=="440204" & year==1999

replace regioncode="440221" if regioncode=="440221" & year==1999

replace regioncode="440222" if regioncode=="440222" & year==1999

replace regioncode="440224" if regioncode=="440224" & year==1999

replace regioncode="440229" if regioncode=="440229" & year==1999

replace regioncode="440232" if regioncode=="440232" & year==1999

replace regioncode="440233" if regioncode=="440233" & year==1999

replace regioncode="440281" if regioncode=="440281" & year==1999

replace regioncode="440282" if regioncode=="440282" & year==1999

replace regioncode="440303" if regioncode=="440303" & year==1999

replace regioncode="440304" if regioncode=="440304" & year==1999

replace regioncode="440305" if regioncode=="440305" & year==1999

replace regioncode="440306" if regioncode=="440306" & year==1999

replace regioncode="440307" if regioncode=="440307" & year==1999

replace regioncode="440308" if regioncode=="440308" & year==1999

replace regioncode="440402" if regioncode=="440402" & year==1999

replace regioncode="440403" if regioncode=="440421" & year==1999

replace regioncode="440404" if regioncode=="440404" & year==1999

replace regioncode="440507" if regioncode=="440507" & year==1999

replace regioncode="440511" if regioncode=="440509" & year==1999

replace regioncode="440512" if regioncode=="440510" & year==1999

replace regioncode="440514" if regioncode=="440582" & year==1999

replace regioncode="440515" if regioncode=="440583" & year==1999

replace regioncode="440523" if regioncode=="440523" & year==1999

replace regioncode="440604" if regioncode=="440603" & year==1999

replace regioncode="440605" if regioncode=="440682" & year==1999

replace regioncode="440606" if regioncode=="440681" & year==1999

replace regioncode="440607" if regioncode=="440683" & year==1999

replace regioncode="440608" if regioncode=="440684" & year==1999

replace regioncode="440703" if regioncode=="440703" & year==1999

replace regioncode="440704" if regioncode=="440704" & year==1999

replace regioncode="440781" if regioncode=="440781" & year==1999

replace regioncode="440705" if regioncode=="440782" & year==1999

replace regioncode="440783" if regioncode=="440783" & year==1999

replace regioncode="440784" if regioncode=="440784" & year==1999

replace regioncode="440785" if regioncode=="440785" & year==1999

replace regioncode="440802" if regioncode=="440802" & year==1999

replace regioncode="440803" if regioncode=="440803" & year==1999

replace regioncode="440804" if regioncode=="440804" & year==1999

replace regioncode="440811" if regioncode=="440811" & year==1999

replace regioncode="440823" if regioncode=="440823" & year==1999

replace regioncode="440825" if regioncode=="440825" & year==1999

replace regioncode="440881" if regioncode=="440881" & year==1999

replace regioncode="440882" if regioncode=="440882" & year==1999

replace regioncode="440883" if regioncode=="440883" & year==1999

replace regioncode="440902" if regioncode=="440902" & year==1999

replace regioncode="440903" if regioncode=="440903" & year==1999

replace regioncode="440923" if regioncode=="440923" & year==1999

replace regioncode="440981" if regioncode=="440981" & year==1999

replace regioncode="440982" if regioncode=="440982" & year==1999

replace regioncode="440983" if regioncode=="440983" & year==1999

replace regioncode="441202" if regioncode=="441202" & year==1999

replace regioncode="441203" if regioncode=="441203" & year==1999

replace regioncode="441223" if regioncode=="441223" & year==1999

replace regioncode="441224" if regioncode=="441224" & year==1999

replace regioncode="441225" if regioncode=="441225" & year==1999

replace regioncode="441226" if regioncode=="441226" & year==1999

replace regioncode="441283" if regioncode=="441283" & year==1999

replace regioncode="441284" if regioncode=="441284" & year==1999

replace regioncode="441302" if regioncode=="441302" & year==1999

replace regioncode="441303" if regioncode=="441381" & year==1999

replace regioncode="441322" if regioncode=="441322" & year==1999

replace regioncode="441323" if regioncode=="441323" & year==1999

replace regioncode="441324" if regioncode=="441324" & year==1999

replace regioncode="441402" if regioncode=="441402" & year==1999

replace regioncode="441421" if regioncode=="441421" & year==1999

replace regioncode="441422" if regioncode=="441422" & year==1999

replace regioncode="441423" if regioncode=="441423" & year==1999

replace regioncode="441424" if regioncode=="441424" & year==1999

replace regioncode="441426" if regioncode=="441426" & year==1999

replace regioncode="441427" if regioncode=="441427" & year==1999

replace regioncode="441481" if regioncode=="441481" & year==1999

replace regioncode="441502" if regioncode=="441502" & year==1999

replace regioncode="441521" if regioncode=="441521" & year==1999

replace regioncode="441523" if regioncode=="441523" & year==1999

replace regioncode="441581" if regioncode=="441581" & year==1999

replace regioncode="441602" if regioncode=="441602" & year==1999

replace regioncode="441621" if regioncode=="441621" & year==1999

replace regioncode="441622" if regioncode=="441622" & year==1999

replace regioncode="441623" if regioncode=="441623" & year==1999

replace regioncode="441624" if regioncode=="441624" & year==1999

replace regioncode="441625" if regioncode=="441625" & year==1999

replace regioncode="441702" if regioncode=="441702" & year==1999

replace regioncode="441721" if regioncode=="441721" & year==1999

replace regioncode="441723" if regioncode=="441723" & year==1999

replace regioncode="441781" if regioncode=="441781" & year==1999

replace regioncode="441802" if regioncode=="441802" & year==1999

replace regioncode="441821" if regioncode=="441821" & year==1999

replace regioncode="441823" if regioncode=="441823" & year==1999

replace regioncode="441825" if regioncode=="441825" & year==1999

replace regioncode="441826" if regioncode=="441826" & year==1999

replace regioncode="441827" if regioncode=="441827" & year==1999

replace regioncode="441881" if regioncode=="441881" & year==1999

replace regioncode="441882" if regioncode=="441882" & year==1999

replace regioncode="441900" if regioncode=="441900" & year==1999

replace regioncode="442000" if regioncode=="442098" & year==1999

replace regioncode="445102" if regioncode=="445102" & year==1999

replace regioncode="445121" if regioncode=="445121" & year==1999

replace regioncode="445122" if regioncode=="445122" & year==1999

replace regioncode="445202" if regioncode=="445202" & year==1999

replace regioncode="445221" if regioncode=="445221" & year==1999

replace regioncode="445222" if regioncode=="445222" & year==1999

replace regioncode="445224" if regioncode=="445224" & year==1999

replace regioncode="445281" if regioncode=="445281" & year==1999

replace regioncode="445302" if regioncode=="445302" & year==1999

replace regioncode="445321" if regioncode=="445321" & year==1999

replace regioncode="445322" if regioncode=="445322" & year==1999

replace regioncode="445323" if regioncode=="445323" & year==1999

replace regioncode="445381" if regioncode=="445381" & year==1999

replace regioncode="450102" if regioncode=="450102" & year==1999

replace regioncode="450103" if regioncode=="450103" & year==1999

replace regioncode="450104" if regioncode=="450104" & year==1999

replace regioncode="450105" if regioncode=="450105" & year==1999

replace regioncode="450106" if regioncode=="450106" & year==1999

replace regioncode="450121" if regioncode=="450121" & year==1999

replace regioncode="450122" if regioncode=="450122" & year==1999

replace regioncode="450123" if regioncode=="452126" & year==1999

replace regioncode="450124" if regioncode=="452127" & year==1999

replace regioncode="450125" if regioncode=="452124" & year==1999

replace regioncode="450126" if regioncode=="452123" & year==1999

replace regioncode="450127" if regioncode=="452122" & year==1999

replace regioncode="450202" if regioncode=="450202" & year==1999

replace regioncode="450203" if regioncode=="450203" & year==1999

replace regioncode="450204" if regioncode=="450204" & year==1999

replace regioncode="450205" if regioncode=="450205" & year==1999

replace regioncode="450221" if regioncode=="450221" & year==1999

replace regioncode="450222" if regioncode=="450222" & year==1999

replace regioncode="450223" if regioncode=="452223" & year==1999

replace regioncode="450224" if regioncode=="452227" & year==1999

replace regioncode="450225" if regioncode=="452229" & year==1999

replace regioncode="450226" if regioncode=="452228" & year==1999

replace regioncode="450302" if regioncode=="450302" & year==1999

replace regioncode="450303" if regioncode=="450303" & year==1999

replace regioncode="450304" if regioncode=="450304" & year==1999

replace regioncode="450305" if regioncode=="450305" & year==1999

replace regioncode="450311" if regioncode=="450311" & year==1999

replace regioncode="450321" if regioncode=="450321" & year==1999

replace regioncode="450322" if regioncode=="450322" & year==1999

replace regioncode="450323" if regioncode=="450323" & year==1999

replace regioncode="450324" if regioncode=="450324" & year==1999

replace regioncode="450325" if regioncode=="450325" & year==1999

replace regioncode="450326" if regioncode=="450326" & year==1999

replace regioncode="450327" if regioncode=="450327" & year==1999

replace regioncode="450328" if regioncode=="450328" & year==1999

replace regioncode="450329" if regioncode=="450329" & year==1999

replace regioncode="450330" if regioncode=="450330" & year==1999

replace regioncode="450331" if regioncode=="450331" & year==1999

replace regioncode="450332" if regioncode=="450332" & year==1999

replace regioncode="450403" if regioncode=="450403" & year==1999

replace regioncode="450404" if regioncode=="450404" & year==1999

replace regioncode="450421" if regioncode=="450421" & year==1999

replace regioncode="450422" if regioncode=="450422" & year==1999

replace regioncode="450423" if regioncode=="450423" & year==1999

replace regioncode="450481" if regioncode=="450481" & year==1999

replace regioncode="450502" if regioncode=="450502" & year==1999

replace regioncode="450503" if regioncode=="450503" & year==1999

replace regioncode="450512" if regioncode=="450512" & year==1999

replace regioncode="450521" if regioncode=="450521" & year==1999

replace regioncode="450602" if regioncode=="450602" & year==1999

replace regioncode="450603" if regioncode=="450603" & year==1999

replace regioncode="450621" if regioncode=="450621" & year==1999

replace regioncode="450681" if regioncode=="450681" & year==1999

replace regioncode="450702" if regioncode=="450702" & year==1999

replace regioncode="450703" if regioncode=="450703" & year==1999

replace regioncode="450721" if regioncode=="450721" & year==1999

replace regioncode="450722" if regioncode=="450722" & year==1999

replace regioncode="450802" if regioncode=="450802" & year==1999

replace regioncode="450803" if regioncode=="450803" & year==1999

replace regioncode="450804" if regioncode=="450804" & year==1999

replace regioncode="450821" if regioncode=="450821" & year==1999

replace regioncode="450881" if regioncode=="450881" & year==1999

replace regioncode="450902" if regioncode=="450902" & year==1999

replace regioncode="450921" if regioncode=="450921" & year==1999

replace regioncode="450922" if regioncode=="450922" & year==1999

replace regioncode="450923" if regioncode=="450923" & year==1999

replace regioncode="450924" if regioncode=="450924" & year==1999

replace regioncode="450981" if regioncode=="450981" & year==1999

replace regioncode="451002" if regioncode=="452601" & year==1999

replace regioncode="451021" if regioncode=="452622" & year==1999

replace regioncode="451022" if regioncode=="452623" & year==1999

replace regioncode="451023" if regioncode=="452624" & year==1999

replace regioncode="451024" if regioncode=="452625" & year==1999

replace regioncode="451025" if regioncode=="452626" & year==1999

replace regioncode="451026" if regioncode=="452627" & year==1999

replace regioncode="451027" if regioncode=="452628" & year==1999

replace regioncode="451028" if regioncode=="452629" & year==1999

replace regioncode="451029" if regioncode=="452630" & year==1999

replace regioncode="451030" if regioncode=="452632" & year==1999

replace regioncode="451031" if regioncode=="452631" & year==1999

replace regioncode="451102" if regioncode=="452402" & year==1999

replace regioncode="451121" if regioncode=="452424" & year==1999

replace regioncode="451122" if regioncode=="452427" & year==1999

replace regioncode="451123" if regioncode=="452428" & year==1999

replace regioncode="451202" if regioncode=="452701" & year==1999

replace regioncode="451221" if regioncode=="452725" & year==1999

replace regioncode="451222" if regioncode=="452726" & year==1999

replace regioncode="451223" if regioncode=="452727" & year==1999

replace regioncode="451224" if regioncode=="452728" & year==1999

replace regioncode="451225" if regioncode=="452723" & year==1999

replace regioncode="451226" if regioncode=="452724" & year==1999

replace regioncode="451227" if regioncode=="452729" & year==1999

replace regioncode="451228" if regioncode=="452730" & year==1999

replace regioncode="451229" if regioncode=="452731" & year==1999

replace regioncode="451281" if regioncode=="452702" & year==1999

replace regioncode="451302" if regioncode=="452226" & year==1999

replace regioncode="451321" if regioncode=="452231" & year==1999

replace regioncode="451322" if regioncode=="452224" & year==1999

replace regioncode="451323" if regioncode=="452225" & year==1999

replace regioncode="451324" if regioncode=="452230" & year==1999

replace regioncode="451381" if regioncode=="452201" & year==1999

replace regioncode="451402" if regioncode=="452129" & year==1999

replace regioncode="451421" if regioncode=="452128" & year==1999

replace regioncode="451422" if regioncode=="452132" & year==1999

replace regioncode="451423" if regioncode=="452133" & year==1999

replace regioncode="451424" if regioncode=="452130" & year==1999

replace regioncode="451425" if regioncode=="452131" & year==1999

replace regioncode="451481" if regioncode=="452101" & year==1999

replace regioncode="460105" if regioncode=="460102" & year==1999

replace regioncode="460106" if regioncode=="460103" & year==1999

replace regioncode="460107" if regioncode=="460104" & year==1999

replace regioncode="460108" if regioncode=="469004" & year==1999

replace regioncode="460001" if regioncode=="460001" & year==1999

replace regioncode="460002" if regioncode=="460002" & year==1999

replace regioncode="460003" if regioncode=="460003" & year==1999

replace regioncode="460005" if regioncode=="460005" & year==1999

replace regioncode="460006" if regioncode=="460006" & year==1999

replace regioncode="460007" if regioncode=="460007" & year==1999

replace regioncode="460030" if regioncode=="460030" & year==1999

replace regioncode="460031" if regioncode=="460031" & year==1999

replace regioncode="460033" if regioncode=="460033" & year==1999

replace regioncode="460034" if regioncode=="460034" & year==1999

replace regioncode="460036" if regioncode=="460036" & year==1999

replace regioncode="500101" if regioncode=="500101" & year==1999

replace regioncode="500102" if regioncode=="500102" & year==1999

replace regioncode="500103" if regioncode=="500103" & year==1999

replace regioncode="500104" if regioncode=="500104" & year==1999

replace regioncode="500105" if regioncode=="500105" & year==1999

replace regioncode="500106" if regioncode=="500106" & year==1999

replace regioncode="500107" if regioncode=="500107" & year==1999

replace regioncode="500108" if regioncode=="500108" & year==1999

replace regioncode="500109" if regioncode=="500109" & year==1999

replace regioncode="500110" if regioncode=="500110" & year==1999

replace regioncode="500111" if regioncode=="500111" & year==1999

replace regioncode="500112" if regioncode=="500112" & year==1999

replace regioncode="500113" if regioncode=="500113" & year==1999

replace regioncode="500114" if regioncode=="500239" & year==1999

replace regioncode="500115" if regioncode=="500221" & year==1999

replace regioncode="500381" if regioncode=="500381" & year==1999

replace regioncode="500382" if regioncode=="500382" & year==1999

replace regioncode="500383" if regioncode=="500383" & year==1999

replace regioncode="500384" if regioncode=="500384" & year==1999

replace regioncode="500222" if regioncode=="500222" & year==1999

replace regioncode="500223" if regioncode=="500223" & year==1999

replace regioncode="500224" if regioncode=="500224" & year==1999

replace regioncode="500225" if regioncode=="500225" & year==1999

replace regioncode="500226" if regioncode=="500226" & year==1999

replace regioncode="500227" if regioncode=="500227" & year==1999

replace regioncode="500228" if regioncode=="500228" & year==1999

replace regioncode="500229" if regioncode=="500229" & year==1999

replace regioncode="500230" if regioncode=="500230" & year==1999

replace regioncode="500231" if regioncode=="500231" & year==1999

replace regioncode="500232" if regioncode=="500232" & year==1999

replace regioncode="500233" if regioncode=="500233" & year==1999

replace regioncode="500234" if regioncode=="500234" & year==1999

replace regioncode="500235" if regioncode=="500235" & year==1999

replace regioncode="500236" if regioncode=="500236" & year==1999

replace regioncode="500237" if regioncode=="500237" & year==1999

replace regioncode="500238" if regioncode=="500238" & year==1999

replace regioncode="500240" if regioncode=="500240" & year==1999

replace regioncode="500241" if regioncode=="500241" & year==1999

replace regioncode="500242" if regioncode=="500242" & year==1999

replace regioncode="500243" if regioncode=="500243" & year==1999

replace regioncode="510104" if regioncode=="510104" & year==1999

replace regioncode="510105" if regioncode=="510105" & year==1999

replace regioncode="510106" if regioncode=="510106" & year==1999

replace regioncode="510107" if regioncode=="510107" & year==1999

replace regioncode="510108" if regioncode=="510108" & year==1999

replace regioncode="510112" if regioncode=="510112" & year==1999

replace regioncode="510113" if regioncode=="510113" & year==1999

replace regioncode="510114" if regioncode=="510125" & year==1999

replace regioncode="510115" if regioncode=="510123" & year==1999

replace regioncode="510121" if regioncode=="510121" & year==1999

replace regioncode="510122" if regioncode=="510122" & year==1999

replace regioncode="510124" if regioncode=="510124" & year==1999

replace regioncode="510129" if regioncode=="510129" & year==1999

replace regioncode="510131" if regioncode=="510131" & year==1999

replace regioncode="510132" if regioncode=="510132" & year==1999

replace regioncode="510181" if regioncode=="510181" & year==1999

replace regioncode="510182" if regioncode=="510182" & year==1999

replace regioncode="510183" if regioncode=="510183" & year==1999

replace regioncode="510184" if regioncode=="510184" & year==1999

replace regioncode="510302" if regioncode=="510302" & year==1999

replace regioncode="510303" if regioncode=="510303" & year==1999

replace regioncode="510304" if regioncode=="510304" & year==1999

replace regioncode="510311" if regioncode=="510311" & year==1999

replace regioncode="510321" if regioncode=="510321" & year==1999

replace regioncode="510322" if regioncode=="510322" & year==1999

replace regioncode="510402" if regioncode=="510402" & year==1999

replace regioncode="510403" if regioncode=="510403" & year==1999

replace regioncode="510411" if regioncode=="510411" & year==1999

replace regioncode="510421" if regioncode=="510421" & year==1999

replace regioncode="510422" if regioncode=="510422" & year==1999

replace regioncode="510502" if regioncode=="510502" & year==1999

replace regioncode="510503" if regioncode=="510503" & year==1999

replace regioncode="510504" if regioncode=="510504" & year==1999

replace regioncode="510521" if regioncode=="510521" & year==1999

replace regioncode="510522" if regioncode=="510522" & year==1999

replace regioncode="510524" if regioncode=="510524" & year==1999

replace regioncode="510525" if regioncode=="510525" & year==1999

replace regioncode="510603" if regioncode=="510603" & year==1999

replace regioncode="510623" if regioncode=="510623" & year==1999

replace regioncode="510626" if regioncode=="510626" & year==1999

replace regioncode="510681" if regioncode=="510681" & year==1999

replace regioncode="510682" if regioncode=="510682" & year==1999

replace regioncode="510683" if regioncode=="510683" & year==1999

replace regioncode="510703" if regioncode=="510703" & year==1999

replace regioncode="510704" if regioncode=="510704" & year==1999

replace regioncode="510722" if regioncode=="510722" & year==1999

replace regioncode="510723" if regioncode=="510723" & year==1999

replace regioncode="510724" if regioncode=="510724" & year==1999

replace regioncode="510725" if regioncode=="510725" & year==1999

replace regioncode="510726" if regioncode=="510726" & year==1999

replace regioncode="510727" if regioncode=="510727" & year==1999

replace regioncode="510781" if regioncode=="510781" & year==1999

replace regioncode="510802" if regioncode=="510802" & year==1999

replace regioncode="510811" if regioncode=="510811" & year==1999

replace regioncode="510812" if regioncode=="510812" & year==1999

replace regioncode="510821" if regioncode=="510821" & year==1999

replace regioncode="510822" if regioncode=="510822" & year==1999

replace regioncode="510823" if regioncode=="510823" & year==1999

replace regioncode="510824" if regioncode=="510824" & year==1999

replace regioncode="510902" if regioncode=="510902" & year==1999

replace regioncode="510921" if regioncode=="510921" & year==1999

replace regioncode="510922" if regioncode=="510922" & year==1999

replace regioncode="510923" if regioncode=="510923" & year==1999

replace regioncode="511002" if regioncode=="511002" & year==1999

replace regioncode="511011" if regioncode=="511011" & year==1999

replace regioncode="511024" if regioncode=="511024" & year==1999

replace regioncode="511025" if regioncode=="511025" & year==1999

replace regioncode="511028" if regioncode=="511028" & year==1999

replace regioncode="511102" if regioncode=="511102" & year==1999

replace regioncode="511111" if regioncode=="511111" & year==1999

replace regioncode="511112" if regioncode=="511112" & year==1999

replace regioncode="511113" if regioncode=="511113" & year==1999

replace regioncode="511123" if regioncode=="511123" & year==1999

replace regioncode="511124" if regioncode=="511124" & year==1999

replace regioncode="511126" if regioncode=="511126" & year==1999

replace regioncode="511129" if regioncode=="511129" & year==1999

replace regioncode="511132" if regioncode=="511132" & year==1999

replace regioncode="511133" if regioncode=="511133" & year==1999

replace regioncode="511181" if regioncode=="511181" & year==1999

replace regioncode="511302" if regioncode=="511302" & year==1999

replace regioncode="511303" if regioncode=="511303" & year==1999

replace regioncode="511304" if regioncode=="511304" & year==1999

replace regioncode="511321" if regioncode=="511321" & year==1999

replace regioncode="511322" if regioncode=="511322" & year==1999

replace regioncode="511323" if regioncode=="511323" & year==1999

replace regioncode="511324" if regioncode=="511324" & year==1999

replace regioncode="511325" if regioncode=="511325" & year==1999

replace regioncode="511381" if regioncode=="511381" & year==1999

replace regioncode="511402" if regioncode=="513821" & year==1999

replace regioncode="511421" if regioncode=="513822" & year==1999

replace regioncode="511422" if regioncode=="513823" & year==1999

replace regioncode="511423" if regioncode=="513824" & year==1999

replace regioncode="511424" if regioncode=="513825" & year==1999

replace regioncode="511425" if regioncode=="513826" & year==1999

replace regioncode="511502" if regioncode=="511502" & year==1999

replace regioncode="511521" if regioncode=="511521" & year==1999

replace regioncode="511522" if regioncode=="511522" & year==1999

replace regioncode="511523" if regioncode=="511523" & year==1999

replace regioncode="511524" if regioncode=="511524" & year==1999

replace regioncode="511525" if regioncode=="511525" & year==1999

replace regioncode="511526" if regioncode=="511526" & year==1999

replace regioncode="511527" if regioncode=="511527" & year==1999

replace regioncode="511528" if regioncode=="511528" & year==1999

replace regioncode="511529" if regioncode=="511529" & year==1999

replace regioncode="511602" if regioncode=="511602" & year==1999

replace regioncode="511621" if regioncode=="511621" & year==1999

replace regioncode="511622" if regioncode=="511622" & year==1999

replace regioncode="511623" if regioncode=="511623" & year==1999

replace regioncode="511681" if regioncode=="511681" & year==1999

replace regioncode="511702" if regioncode=="511702" & year==1999

replace regioncode="511721" if regioncode=="511721" & year==1999

replace regioncode="511722" if regioncode=="511722" & year==1999

replace regioncode="511723" if regioncode=="511723" & year==1999

replace regioncode="511724" if regioncode=="511724" & year==1999

replace regioncode="511725" if regioncode=="511725" & year==1999

replace regioncode="511781" if regioncode=="511781" & year==1999

replace regioncode="511802" if regioncode=="513101" & year==1999

replace regioncode="511821" if regioncode=="513122" & year==1999

replace regioncode="511822" if regioncode=="513123" & year==1999

replace regioncode="511823" if regioncode=="513124" & year==1999

replace regioncode="511824" if regioncode=="513125" & year==1999

replace regioncode="511825" if regioncode=="513126" & year==1999

replace regioncode="511826" if regioncode=="513127" & year==1999

replace regioncode="511827" if regioncode=="513128" & year==1999

replace regioncode="511902" if regioncode=="513701" & year==1999

replace regioncode="511921" if regioncode=="513721" & year==1999

replace regioncode="511922" if regioncode=="513722" & year==1999

replace regioncode="511923" if regioncode=="513723" & year==1999

replace regioncode="512002" if regioncode=="513901" & year==1999

replace regioncode="512021" if regioncode=="513921" & year==1999

replace regioncode="512022" if regioncode=="513922" & year==1999

replace regioncode="512081" if regioncode=="513902" & year==1999

replace regioncode="513221" if regioncode=="513221" & year==1999

replace regioncode="513222" if regioncode=="513222" & year==1999

replace regioncode="513223" if regioncode=="513223" & year==1999

replace regioncode="513224" if regioncode=="513224" & year==1999

replace regioncode="513225" if regioncode=="513225" & year==1999

replace regioncode="513226" if regioncode=="513226" & year==1999

replace regioncode="513227" if regioncode=="513227" & year==1999

replace regioncode="513228" if regioncode=="513228" & year==1999

replace regioncode="513229" if regioncode=="513229" & year==1999

replace regioncode="513230" if regioncode=="513230" & year==1999

replace regioncode="513231" if regioncode=="513231" & year==1999

replace regioncode="513232" if regioncode=="513232" & year==1999

replace regioncode="513233" if regioncode=="513233" & year==1999

replace regioncode="513321" if regioncode=="513321" & year==1999

replace regioncode="513322" if regioncode=="513322" & year==1999

replace regioncode="513323" if regioncode=="513323" & year==1999

replace regioncode="513324" if regioncode=="513324" & year==1999

replace regioncode="513325" if regioncode=="513325" & year==1999

replace regioncode="513326" if regioncode=="513326" & year==1999

replace regioncode="513327" if regioncode=="513327" & year==1999

replace regioncode="513328" if regioncode=="513328" & year==1999

replace regioncode="513329" if regioncode=="513329" & year==1999

replace regioncode="513330" if regioncode=="513330" & year==1999

replace regioncode="513331" if regioncode=="513331" & year==1999

replace regioncode="513332" if regioncode=="513332" & year==1999

replace regioncode="513333" if regioncode=="513333" & year==1999

replace regioncode="513334" if regioncode=="513334" & year==1999

replace regioncode="513335" if regioncode=="513335" & year==1999

replace regioncode="513336" if regioncode=="513336" & year==1999

replace regioncode="513337" if regioncode=="513337" & year==1999

replace regioncode="513338" if regioncode=="513338" & year==1999

replace regioncode="513401" if regioncode=="513401" & year==1999

replace regioncode="513422" if regioncode=="513422" & year==1999

replace regioncode="513423" if regioncode=="513423" & year==1999

replace regioncode="513424" if regioncode=="513424" & year==1999

replace regioncode="513425" if regioncode=="513425" & year==1999

replace regioncode="513426" if regioncode=="513426" & year==1999

replace regioncode="513427" if regioncode=="513427" & year==1999

replace regioncode="513428" if regioncode=="513428" & year==1999

replace regioncode="513429" if regioncode=="513429" & year==1999

replace regioncode="513430" if regioncode=="513430" & year==1999

replace regioncode="513431" if regioncode=="513431" & year==1999

replace regioncode="513432" if regioncode=="513432" & year==1999

replace regioncode="513433" if regioncode=="513433" & year==1999

replace regioncode="513434" if regioncode=="513434" & year==1999

replace regioncode="513435" if regioncode=="513435" & year==1999

replace regioncode="513436" if regioncode=="513436" & year==1999

replace regioncode="513437" if regioncode=="513437" & year==1999

replace regioncode="520102" if regioncode=="520102" & year==1999

replace regioncode="520103" if regioncode=="520103" & year==1999

replace regioncode="520111" if regioncode=="520111" & year==1999

replace regioncode="520112" if regioncode=="520112" & year==1999

replace regioncode="520113" if regioncode=="520113" & year==1999

replace regioncode="520114" if regioncode=="520114" & year==1999

replace regioncode="520121" if regioncode=="520121" & year==1999

replace regioncode="520122" if regioncode=="520122" & year==1999

replace regioncode="520123" if regioncode=="520123" & year==1999

replace regioncode="520181" if regioncode=="520181" & year==1999

replace regioncode="520201" if regioncode=="520201" & year==1999

replace regioncode="520222" if regioncode=="520222" & year==1999

replace regioncode="520203" if regioncode=="520203" & year==1999

replace regioncode="520221" if regioncode=="520221" & year==1999

replace regioncode="520302" if regioncode=="520302" & year==1999

replace regioncode="520321" if regioncode=="520321" & year==1999

replace regioncode="520322" if regioncode=="520322" & year==1999

replace regioncode="520323" if regioncode=="520323" & year==1999

replace regioncode="520324" if regioncode=="520324" & year==1999

replace regioncode="520325" if regioncode=="520325" & year==1999

replace regioncode="520326" if regioncode=="520326" & year==1999

replace regioncode="520327" if regioncode=="520327" & year==1999

replace regioncode="520328" if regioncode=="520328" & year==1999

replace regioncode="520329" if regioncode=="520329" & year==1999

replace regioncode="520330" if regioncode=="520330" & year==1999

replace regioncode="520381" if regioncode=="520381" & year==1999

replace regioncode="520382" if regioncode=="520382" & year==1999

replace regioncode="520402" if regioncode=="522501" & year==1999

replace regioncode="520421" if regioncode=="522526" & year==1999

replace regioncode="520422" if regioncode=="522527" & year==1999

replace regioncode="520423" if regioncode=="522529" & year==1999

replace regioncode="520424" if regioncode=="522528" & year==1999

replace regioncode="520425" if regioncode=="522530" & year==1999

replace regioncode="522201" if regioncode=="522201" & year==1999

replace regioncode="522222" if regioncode=="522222" & year==1999

replace regioncode="522223" if regioncode=="522223" & year==1999

replace regioncode="522224" if regioncode=="522224" & year==1999

replace regioncode="522225" if regioncode=="522225" & year==1999

replace regioncode="522226" if regioncode=="522226" & year==1999

replace regioncode="522227" if regioncode=="522227" & year==1999

replace regioncode="522228" if regioncode=="522228" & year==1999

replace regioncode="522229" if regioncode=="522229" & year==1999

replace regioncode="522230" if regioncode=="522230" & year==1999

replace regioncode="522301" if regioncode=="522301" & year==1999

replace regioncode="522322" if regioncode=="522322" & year==1999

replace regioncode="522323" if regioncode=="522323" & year==1999

replace regioncode="522324" if regioncode=="522324" & year==1999

replace regioncode="522325" if regioncode=="522325" & year==1999

replace regioncode="522326" if regioncode=="522326" & year==1999

replace regioncode="522327" if regioncode=="522327" & year==1999

replace regioncode="522328" if regioncode=="522328" & year==1999

replace regioncode="522401" if regioncode=="522401" & year==1999

replace regioncode="522422" if regioncode=="522422" & year==1999

replace regioncode="522423" if regioncode=="522423" & year==1999

replace regioncode="522424" if regioncode=="522424" & year==1999

replace regioncode="522425" if regioncode=="522425" & year==1999

replace regioncode="522426" if regioncode=="522426" & year==1999

replace regioncode="522427" if regioncode=="522427" & year==1999

replace regioncode="522428" if regioncode=="522428" & year==1999

replace regioncode="522601" if regioncode=="522601" & year==1999

replace regioncode="522622" if regioncode=="522622" & year==1999

replace regioncode="522623" if regioncode=="522623" & year==1999

replace regioncode="522624" if regioncode=="522624" & year==1999

replace regioncode="522625" if regioncode=="522625" & year==1999

replace regioncode="522626" if regioncode=="522626" & year==1999

replace regioncode="522627" if regioncode=="522627" & year==1999

replace regioncode="522628" if regioncode=="522628" & year==1999

replace regioncode="522629" if regioncode=="522629" & year==1999

replace regioncode="522630" if regioncode=="522630" & year==1999

replace regioncode="522631" if regioncode=="522631" & year==1999

replace regioncode="522632" if regioncode=="522632" & year==1999

replace regioncode="522633" if regioncode=="522633" & year==1999

replace regioncode="522634" if regioncode=="522634" & year==1999

replace regioncode="522635" if regioncode=="522635" & year==1999

replace regioncode="522636" if regioncode=="522636" & year==1999

replace regioncode="522701" if regioncode=="522701" & year==1999

replace regioncode="522702" if regioncode=="522702" & year==1999

replace regioncode="522722" if regioncode=="522722" & year==1999

replace regioncode="522723" if regioncode=="522723" & year==1999

replace regioncode="522725" if regioncode=="522725" & year==1999

replace regioncode="522726" if regioncode=="522726" & year==1999

replace regioncode="522727" if regioncode=="522727" & year==1999

replace regioncode="522728" if regioncode=="522728" & year==1999

replace regioncode="522729" if regioncode=="522729" & year==1999

replace regioncode="522730" if regioncode=="522730" & year==1999

replace regioncode="522731" if regioncode=="522731" & year==1999

replace regioncode="522732" if regioncode=="522732" & year==1999

replace regioncode="530102" if regioncode=="530102" & year==1999

replace regioncode="530103" if regioncode=="530103" & year==1999

replace regioncode="530111" if regioncode=="530111" & year==1999

replace regioncode="530112" if regioncode=="530112" & year==1999

replace regioncode="530113" if regioncode=="530113" & year==1999

replace regioncode="530121" if regioncode=="530121" & year==1999

replace regioncode="530122" if regioncode=="530122" & year==1999

replace regioncode="530124" if regioncode=="530124" & year==1999

replace regioncode="530125" if regioncode=="530125" & year==1999

replace regioncode="530126" if regioncode=="530126" & year==1999

replace regioncode="530127" if regioncode=="530127" & year==1999

replace regioncode="530128" if regioncode=="530128" & year==1999

replace regioncode="530129" if regioncode=="530129" & year==1999

replace regioncode="530181" if regioncode=="530181" & year==1999

replace regioncode="530302" if regioncode=="530302" & year==1999

replace regioncode="530321" if regioncode=="530321" & year==1999

replace regioncode="530322" if regioncode=="530322" & year==1999

replace regioncode="530323" if regioncode=="530323" & year==1999

replace regioncode="530324" if regioncode=="530324" & year==1999

replace regioncode="530325" if regioncode=="530325" & year==1999

replace regioncode="530326" if regioncode=="530326" & year==1999

replace regioncode="530328" if regioncode=="530328" & year==1999

replace regioncode="530381" if regioncode=="530381" & year==1999

replace regioncode="530402" if regioncode=="530402" & year==1999

replace regioncode="530421" if regioncode=="530421" & year==1999

replace regioncode="530422" if regioncode=="530422" & year==1999

replace regioncode="530423" if regioncode=="530423" & year==1999

replace regioncode="530424" if regioncode=="530424" & year==1999

replace regioncode="530425" if regioncode=="530425" & year==1999

replace regioncode="530426" if regioncode=="530426" & year==1999

replace regioncode="530427" if regioncode=="530427" & year==1999

replace regioncode="530428" if regioncode=="530428" & year==1999

replace regioncode="530502" if regioncode=="533001" & year==1999

replace regioncode="530521" if regioncode=="533022" & year==1999

replace regioncode="530522" if regioncode=="533023" & year==1999

replace regioncode="530523" if regioncode=="533024" & year==1999

replace regioncode="530524" if regioncode=="533025" & year==1999

replace regioncode="530602" if regioncode=="532101" & year==1999

replace regioncode="530621" if regioncode=="532122" & year==1999

replace regioncode="530622" if regioncode=="532123" & year==1999

replace regioncode="530623" if regioncode=="532124" & year==1999

replace regioncode="530624" if regioncode=="532125" & year==1999

replace regioncode="530625" if regioncode=="532126" & year==1999

replace regioncode="530626" if regioncode=="532127" & year==1999

replace regioncode="530627" if regioncode=="532128" & year==1999

replace regioncode="530628" if regioncode=="532129" & year==1999

replace regioncode="530629" if regioncode=="532130" & year==1999

replace regioncode="530630" if regioncode=="532131" & year==1999

replace regioncode="530721" if regioncode=="533221" & year==1999

replace regioncode="530722" if regioncode=="533222" & year==1999

replace regioncode="530723" if regioncode=="533223" & year==1999

replace regioncode="530724" if regioncode=="533224" & year==1999

replace regioncode="532701" if regioncode=="532701" & year==1999

replace regioncode="532722" if regioncode=="532722" & year==1999

replace regioncode="532723" if regioncode=="532723" & year==1999

replace regioncode="532724" if regioncode=="532724" & year==1999

replace regioncode="532725" if regioncode=="532725" & year==1999

replace regioncode="532726" if regioncode=="532726" & year==1999

replace regioncode="532727" if regioncode=="532727" & year==1999

replace regioncode="532728" if regioncode=="532728" & year==1999

replace regioncode="532729" if regioncode=="532729" & year==1999

replace regioncode="532730" if regioncode=="532730" & year==1999

replace regioncode="533521" if regioncode=="533521" & year==1999

replace regioncode="533522" if regioncode=="533522" & year==1999

replace regioncode="533523" if regioncode=="533523" & year==1999

replace regioncode="533524" if regioncode=="533524" & year==1999

replace regioncode="533525" if regioncode=="533525" & year==1999

replace regioncode="533526" if regioncode=="533526" & year==1999

replace regioncode="533527" if regioncode=="533527" & year==1999

replace regioncode="533528" if regioncode=="533528" & year==1999

replace regioncode="532301" if regioncode=="532301" & year==1999

replace regioncode="532322" if regioncode=="532322" & year==1999

replace regioncode="532323" if regioncode=="532323" & year==1999

replace regioncode="532324" if regioncode=="532324" & year==1999

replace regioncode="532325" if regioncode=="532325" & year==1999

replace regioncode="532326" if regioncode=="532326" & year==1999

replace regioncode="532327" if regioncode=="532327" & year==1999

replace regioncode="532328" if regioncode=="532328" & year==1999

replace regioncode="532329" if regioncode=="532329" & year==1999

replace regioncode="532331" if regioncode=="532331" & year==1999

replace regioncode="532501" if regioncode=="532501" & year==1999

replace regioncode="532502" if regioncode=="532502" & year==1999

replace regioncode="532522" if regioncode=="532522" & year==1999

replace regioncode="532523" if regioncode=="532523" & year==1999

replace regioncode="532524" if regioncode=="532524" & year==1999

replace regioncode="532525" if regioncode=="532525" & year==1999

replace regioncode="532526" if regioncode=="532526" & year==1999

replace regioncode="532527" if regioncode=="532527" & year==1999

replace regioncode="532528" if regioncode=="532528" & year==1999

replace regioncode="532529" if regioncode=="532529" & year==1999

replace regioncode="532530" if regioncode=="532530" & year==1999

replace regioncode="532531" if regioncode=="532531" & year==1999

replace regioncode="532532" if regioncode=="532532" & year==1999

replace regioncode="532621" if regioncode=="532621" & year==1999

replace regioncode="532622" if regioncode=="532622" & year==1999

replace regioncode="532623" if regioncode=="532623" & year==1999

replace regioncode="532624" if regioncode=="532624" & year==1999

replace regioncode="532625" if regioncode=="532625" & year==1999

replace regioncode="532626" if regioncode=="532626" & year==1999

replace regioncode="532627" if regioncode=="532627" & year==1999

replace regioncode="532628" if regioncode=="532628" & year==1999

replace regioncode="532801" if regioncode=="532801" & year==1999

replace regioncode="532822" if regioncode=="532822" & year==1999

replace regioncode="532823" if regioncode=="532823" & year==1999

replace regioncode="532901" if regioncode=="532901" & year==1999

replace regioncode="532922" if regioncode=="532922" & year==1999

replace regioncode="532923" if regioncode=="532923" & year==1999

replace regioncode="532924" if regioncode=="532924" & year==1999

replace regioncode="532925" if regioncode=="532925" & year==1999

replace regioncode="532926" if regioncode=="532926" & year==1999

replace regioncode="532927" if regioncode=="532927" & year==1999

replace regioncode="532928" if regioncode=="532928" & year==1999

replace regioncode="532929" if regioncode=="532929" & year==1999

replace regioncode="532930" if regioncode=="532930" & year==1999

replace regioncode="532931" if regioncode=="532931" & year==1999

replace regioncode="532932" if regioncode=="532932" & year==1999

replace regioncode="533102" if regioncode=="533102" & year==1999

replace regioncode="533103" if regioncode=="533103" & year==1999

replace regioncode="533122" if regioncode=="533122" & year==1999

replace regioncode="533123" if regioncode=="533123" & year==1999

replace regioncode="533124" if regioncode=="533124" & year==1999

replace regioncode="533321" if regioncode=="533321" & year==1999

replace regioncode="533323" if regioncode=="533323" & year==1999

replace regioncode="533324" if regioncode=="533324" & year==1999

replace regioncode="533325" if regioncode=="533325" & year==1999

replace regioncode="533421" if regioncode=="533421" & year==1999

replace regioncode="533422" if regioncode=="533422" & year==1999

replace regioncode="533423" if regioncode=="533423" & year==1999

replace regioncode="540102" if regioncode=="540102" & year==1999

replace regioncode="540121" if regioncode=="540121" & year==1999

replace regioncode="540122" if regioncode=="540122" & year==1999

replace regioncode="540123" if regioncode=="540123" & year==1999

replace regioncode="540124" if regioncode=="540124" & year==1999

replace regioncode="540125" if regioncode=="540125" & year==1999

replace regioncode="540126" if regioncode=="540126" & year==1999

replace regioncode="540127" if regioncode=="540127" & year==1999

replace regioncode="542121" if regioncode=="542121" & year==1999

replace regioncode="542122" if regioncode=="542122" & year==1999

replace regioncode="542123" if regioncode=="542123" & year==1999

replace regioncode="542124" if regioncode=="542124" & year==1999

replace regioncode="542125" if regioncode=="542125" & year==1999

replace regioncode="542126" if regioncode=="542126" & year==1999

replace regioncode="542127" if regioncode=="542127" & year==1999

replace regioncode="542128" if regioncode=="542128" & year==1999

replace regioncode="542129" if regioncode=="542129" & year==1999

replace regioncode="542132" if regioncode=="542132" & year==1999

replace regioncode="542133" if regioncode=="542133" & year==1999

replace regioncode="542221" if regioncode=="542221" & year==1999

replace regioncode="542223" if regioncode=="542223" & year==1999

replace regioncode="542224" if regioncode=="542224" & year==1999

replace regioncode="542225" if regioncode=="542225" & year==1999

replace regioncode="542227" if regioncode=="542227" & year==1999

replace regioncode="542228" if regioncode=="542228" & year==1999

replace regioncode="542229" if regioncode=="542229" & year==1999

replace regioncode="542231" if regioncode=="542231" & year==1999

replace regioncode="542232" if regioncode=="542232" & year==1999

replace regioncode="542233" if regioncode=="542233" & year==1999

replace regioncode="542301" if regioncode=="542301" & year==1999

replace regioncode="542322" if regioncode=="542322" & year==1999

replace regioncode="542323" if regioncode=="542323" & year==1999

replace regioncode="542324" if regioncode=="542324" & year==1999

replace regioncode="542325" if regioncode=="542325" & year==1999

replace regioncode="542326" if regioncode=="542326" & year==1999

replace regioncode="542327" if regioncode=="542327" & year==1999

replace regioncode="542328" if regioncode=="542328" & year==1999

replace regioncode="542329" if regioncode=="542329" & year==1999

replace regioncode="542330" if regioncode=="542330" & year==1999

replace regioncode="542331" if regioncode=="542331" & year==1999

replace regioncode="542332" if regioncode=="542332" & year==1999

replace regioncode="542334" if regioncode=="542334" & year==1999

replace regioncode="542336" if regioncode=="542336" & year==1999

replace regioncode="542338" if regioncode=="542338" & year==1999

replace regioncode="542421" if regioncode=="542421" & year==1999

replace regioncode="542422" if regioncode=="542422" & year==1999

replace regioncode="542423" if regioncode=="542423" & year==1999

replace regioncode="542424" if regioncode=="542424" & year==1999

replace regioncode="542425" if regioncode=="542425" & year==1999

replace regioncode="542426" if regioncode=="542426" & year==1999

replace regioncode="542427" if regioncode=="542427" & year==1999

replace regioncode="542429" if regioncode=="542429" & year==1999

replace regioncode="542430" if regioncode=="542430" & year==1999

replace regioncode="542523" if regioncode=="542523" & year==1999

replace regioncode="542621" if regioncode=="542621" & year==1999

replace regioncode="542622" if regioncode=="542622" & year==1999

replace regioncode="542623" if regioncode=="542623" & year==1999

replace regioncode="542625" if regioncode=="542625" & year==1999

replace regioncode="542626" if regioncode=="542626" & year==1999

replace regioncode="610102" if regioncode=="610102" & year==1999

replace regioncode="610103" if regioncode=="610103" & year==1999

replace regioncode="610104" if regioncode=="610104" & year==1999

replace regioncode="610111" if regioncode=="610111" & year==1999

replace regioncode="610112" if regioncode=="610112" & year==1999

replace regioncode="610113" if regioncode=="610113" & year==1999

replace regioncode="610114" if regioncode=="610114" & year==1999

replace regioncode="610115" if regioncode=="610115" & year==1999

replace regioncode="610116" if regioncode=="610121" & year==1999

replace regioncode="610122" if regioncode=="610122" & year==1999

replace regioncode="610124" if regioncode=="610124" & year==1999

replace regioncode="610125" if regioncode=="610125" & year==1999

replace regioncode="610126" if regioncode=="610126" & year==1999

replace regioncode="610202" if regioncode=="610202" & year==1999

replace regioncode="610203" if regioncode=="610203" & year==1999

replace regioncode="610204" if regioncode=="610221" & year==1999

replace regioncode="610222" if regioncode=="610222" & year==1999

replace regioncode="610302" if regioncode=="610302" & year==1999

replace regioncode="610303" if regioncode=="610303" & year==1999

replace regioncode="610304" if regioncode=="610321" & year==1999

replace regioncode="610322" if regioncode=="610322" & year==1999

replace regioncode="610323" if regioncode=="610323" & year==1999

replace regioncode="610324" if regioncode=="610324" & year==1999

replace regioncode="610326" if regioncode=="610326" & year==1999

replace regioncode="610327" if regioncode=="610327" & year==1999

replace regioncode="610328" if regioncode=="610328" & year==1999

replace regioncode="610329" if regioncode=="610329" & year==1999

replace regioncode="610330" if regioncode=="610330" & year==1999

replace regioncode="610331" if regioncode=="610331" & year==1999

replace regioncode="610402" if regioncode=="610402" & year==1999

replace regioncode="610403" if regioncode=="610403" & year==1999

replace regioncode="610404" if regioncode=="610404" & year==1999

replace regioncode="610422" if regioncode=="610422" & year==1999

replace regioncode="610423" if regioncode=="610423" & year==1999

replace regioncode="610424" if regioncode=="610424" & year==1999

replace regioncode="610425" if regioncode=="610425" & year==1999

replace regioncode="610426" if regioncode=="610426" & year==1999

replace regioncode="610427" if regioncode=="610427" & year==1999

replace regioncode="610428" if regioncode=="610428" & year==1999

replace regioncode="610429" if regioncode=="610429" & year==1999

replace regioncode="610430" if regioncode=="610430" & year==1999

replace regioncode="610431" if regioncode=="610431" & year==1999

replace regioncode="610481" if regioncode=="610481" & year==1999

replace regioncode="610502" if regioncode=="610502" & year==1999

replace regioncode="610521" if regioncode=="610521" & year==1999

replace regioncode="610522" if regioncode=="610522" & year==1999

replace regioncode="610523" if regioncode=="610523" & year==1999

replace regioncode="610524" if regioncode=="610524" & year==1999

replace regioncode="610525" if regioncode=="610525" & year==1999

replace regioncode="610526" if regioncode=="610526" & year==1999

replace regioncode="610527" if regioncode=="610527" & year==1999

replace regioncode="610528" if regioncode=="610528" & year==1999

replace regioncode="610581" if regioncode=="610581" & year==1999

replace regioncode="610582" if regioncode=="610582" & year==1999

replace regioncode="610602" if regioncode=="612601" & year==1999

replace regioncode="610621" if regioncode=="612621" & year==1999

replace regioncode="610622" if regioncode=="612622" & year==1999

replace regioncode="610623" if regioncode=="612623" & year==1999

replace regioncode="610624" if regioncode=="612624" & year==1999

replace regioncode="610625" if regioncode=="612625" & year==1999

replace regioncode="610626" if regioncode=="612626" & year==1999

replace regioncode="610627" if regioncode=="612627" & year==1999

replace regioncode="610628" if regioncode=="612628" & year==1999

replace regioncode="610629" if regioncode=="612629" & year==1999

replace regioncode="610630" if regioncode=="612630" & year==1999

replace regioncode="610631" if regioncode=="612631" & year==1999

replace regioncode="610632" if regioncode=="612632" & year==1999

replace regioncode="610702" if regioncode=="612301" & year==1999

replace regioncode="610721" if regioncode=="612321" & year==1999

replace regioncode="610722" if regioncode=="612322" & year==1999

replace regioncode="610723" if regioncode=="612323" & year==1999

replace regioncode="610724" if regioncode=="612324" & year==1999

replace regioncode="610725" if regioncode=="612325" & year==1999

replace regioncode="610726" if regioncode=="612326" & year==1999

replace regioncode="610727" if regioncode=="612327" & year==1999

replace regioncode="610728" if regioncode=="612328" & year==1999

replace regioncode="610729" if regioncode=="612329" & year==1999

replace regioncode="610730" if regioncode=="612330" & year==1999

replace regioncode="610802" if regioncode=="610801" & year==1999

replace regioncode="610821" if regioncode=="610821" & year==1999

replace regioncode="610822" if regioncode=="610822" & year==1999

replace regioncode="610823" if regioncode=="610823" & year==1999

replace regioncode="610824" if regioncode=="610824" & year==1999

replace regioncode="610825" if regioncode=="610825" & year==1999

replace regioncode="610826" if regioncode=="610826" & year==1999

replace regioncode="610827" if regioncode=="610827" & year==1999

replace regioncode="610828" if regioncode=="610828" & year==1999

replace regioncode="610829" if regioncode=="610829" & year==1999

replace regioncode="610830" if regioncode=="610830" & year==1999

replace regioncode="610831" if regioncode=="610831" & year==1999

replace regioncode="610902" if regioncode=="612401" & year==1999

replace regioncode="610921" if regioncode=="612422" & year==1999

replace regioncode="610922" if regioncode=="612423" & year==1999

replace regioncode="610923" if regioncode=="612424" & year==1999

replace regioncode="610924" if regioncode=="612425" & year==1999

replace regioncode="610925" if regioncode=="612426" & year==1999

replace regioncode="610926" if regioncode=="612427" & year==1999

replace regioncode="610927" if regioncode=="612428" & year==1999

replace regioncode="610928" if regioncode=="612429" & year==1999

replace regioncode="610929" if regioncode=="612430" & year==1999

replace regioncode="611002" if regioncode=="612501" & year==1999

replace regioncode="611021" if regioncode=="612522" & year==1999

replace regioncode="611022" if regioncode=="612523" & year==1999

replace regioncode="611023" if regioncode=="612524" & year==1999

replace regioncode="611024" if regioncode=="612525" & year==1999

replace regioncode="611025" if regioncode=="612526" & year==1999

replace regioncode="611026" if regioncode=="612527" & year==1999

replace regioncode="620102" if regioncode=="620102" & year==1999

replace regioncode="620103" if regioncode=="620103" & year==1999

replace regioncode="620104" if regioncode=="620104" & year==1999

replace regioncode="620105" if regioncode=="620105" & year==1999

replace regioncode="620111" if regioncode=="620111" & year==1999

replace regioncode="620121" if regioncode=="620121" & year==1999

replace regioncode="620122" if regioncode=="620122" & year==1999

replace regioncode="620123" if regioncode=="620123" & year==1999

replace regioncode="620201" if regioncode=="620202" & year==1999

replace regioncode="620302" if regioncode=="620302" & year==1999

replace regioncode="620321" if regioncode=="620321" & year==1999

replace regioncode="620402" if regioncode=="620402" & year==1999

replace regioncode="620403" if regioncode=="620403" & year==1999

replace regioncode="620421" if regioncode=="620421" & year==1999

replace regioncode="620422" if regioncode=="620422" & year==1999

replace regioncode="620423" if regioncode=="620423" & year==1999

replace regioncode="620502" if regioncode=="620502" & year==1999

replace regioncode="620503" if regioncode=="620503" & year==1999

replace regioncode="620521" if regioncode=="620521" & year==1999

replace regioncode="620522" if regioncode=="620522" & year==1999

replace regioncode="620523" if regioncode=="620523" & year==1999

replace regioncode="620524" if regioncode=="620524" & year==1999

replace regioncode="620525" if regioncode=="620525" & year==1999

replace regioncode="622301" if regioncode=="622301" & year==1999

replace regioncode="622322" if regioncode=="622322" & year==1999

replace regioncode="622323" if regioncode=="622323" & year==1999

replace regioncode="622326" if regioncode=="622326" & year==1999

replace regioncode="622201" if regioncode=="622201" & year==1999

replace regioncode="622222" if regioncode=="622222" & year==1999

replace regioncode="622223" if regioncode=="622223" & year==1999

replace regioncode="622224" if regioncode=="622224" & year==1999

replace regioncode="622225" if regioncode=="622225" & year==1999

replace regioncode="622226" if regioncode=="622226" & year==1999

replace regioncode="622701" if regioncode=="622701" & year==1999

replace regioncode="622722" if regioncode=="622722" & year==1999

replace regioncode="622723" if regioncode=="622723" & year==1999

replace regioncode="622724" if regioncode=="622724" & year==1999

replace regioncode="622725" if regioncode=="622725" & year==1999

replace regioncode="622726" if regioncode=="622726" & year==1999

replace regioncode="622727" if regioncode=="622727" & year==1999

replace regioncode="622102" if regioncode=="622102" & year==1999

replace regioncode="622123" if regioncode=="622123" & year==1999

replace regioncode="622126" if regioncode=="622126" & year==1999

replace regioncode="622124" if regioncode=="622124" & year==1999

replace regioncode="622125" if regioncode=="622125" & year==1999

replace regioncode="622101" if regioncode=="622101" & year==1999

replace regioncode="622103" if regioncode=="622103" & year==1999

replace regioncode="622801" if regioncode=="622801" & year==1999

replace regioncode="622821" if regioncode=="622821" & year==1999

replace regioncode="622822" if regioncode=="622822" & year==1999

replace regioncode="622823" if regioncode=="622823" & year==1999

replace regioncode="622824" if regioncode=="622824" & year==1999

replace regioncode="622825" if regioncode=="622825" & year==1999

replace regioncode="622826" if regioncode=="622826" & year==1999

replace regioncode="622827" if regioncode=="622827" & year==1999

replace regioncode="622421" if regioncode=="622421" & year==1999

replace regioncode="622424" if regioncode=="622424" & year==1999

replace regioncode="622425" if regioncode=="622425" & year==1999

replace regioncode="622426" if regioncode=="622426" & year==1999

replace regioncode="622427" if regioncode=="622427" & year==1999

replace regioncode="622428" if regioncode=="622428" & year==1999

replace regioncode="622429" if regioncode=="622429" & year==1999

replace regioncode="622621" if regioncode=="622621" & year==1999

replace regioncode="622624" if regioncode=="622624" & year==1999

replace regioncode="622626" if regioncode=="622626" & year==1999

replace regioncode="622623" if regioncode=="622623" & year==1999

replace regioncode="622625" if regioncode=="622625" & year==1999

replace regioncode="622627" if regioncode=="622627" & year==1999

replace regioncode="622628" if regioncode=="622628" & year==1999

replace regioncode="622630" if regioncode=="622630" & year==1999

replace regioncode="622629" if regioncode=="622629" & year==1999

replace regioncode="622901" if regioncode=="622901" & year==1999

replace regioncode="622921" if regioncode=="622921" & year==1999

replace regioncode="622922" if regioncode=="622922" & year==1999

replace regioncode="622923" if regioncode=="622923" & year==1999

replace regioncode="622924" if regioncode=="622924" & year==1999

replace regioncode="622925" if regioncode=="622925" & year==1999

replace regioncode="622926" if regioncode=="622926" & year==1999

replace regioncode="622927" if regioncode=="622927" & year==1999

replace regioncode="623001" if regioncode=="623001" & year==1999

replace regioncode="623021" if regioncode=="623021" & year==1999

replace regioncode="623022" if regioncode=="623022" & year==1999

replace regioncode="623023" if regioncode=="623023" & year==1999

replace regioncode="623024" if regioncode=="623024" & year==1999

replace regioncode="623025" if regioncode=="623025" & year==1999

replace regioncode="623026" if regioncode=="623026" & year==1999

replace regioncode="623027" if regioncode=="623027" & year==1999

replace regioncode="630102" if regioncode=="630102" & year==1999

replace regioncode="630103" if regioncode=="630103" & year==1999

replace regioncode="630104" if regioncode=="630104" & year==1999

replace regioncode="630105" if regioncode=="630105" & year==1999

replace regioncode="630121" if regioncode=="630121" & year==1999

replace regioncode="630122" if regioncode=="632124" & year==1999

replace regioncode="630123" if regioncode=="632125" & year==1999

replace regioncode="632121" if regioncode=="632121" & year==1999

replace regioncode="632122" if regioncode=="632122" & year==1999

replace regioncode="632123" if regioncode=="632123" & year==1999

replace regioncode="632126" if regioncode=="632126" & year==1999

replace regioncode="632127" if regioncode=="632127" & year==1999

replace regioncode="632128" if regioncode=="632128" & year==1999

replace regioncode="632221" if regioncode=="632221" & year==1999

replace regioncode="632222" if regioncode=="632222" & year==1999

replace regioncode="632223" if regioncode=="632223" & year==1999

replace regioncode="632224" if regioncode=="632224" & year==1999

replace regioncode="632321" if regioncode=="632321" & year==1999

replace regioncode="632322" if regioncode=="632322" & year==1999

replace regioncode="632521" if regioncode=="632521" & year==1999

replace regioncode="632522" if regioncode=="632522" & year==1999

replace regioncode="632523" if regioncode=="632523" & year==1999

replace regioncode="632524" if regioncode=="632524" & year==1999

replace regioncode="632525" if regioncode=="632525" & year==1999

replace regioncode="632621" if regioncode=="632621" & year==1999

replace regioncode="632622" if regioncode=="632622" & year==1999

replace regioncode="632623" if regioncode=="632623" & year==1999

replace regioncode="632624" if regioncode=="632624" & year==1999

replace regioncode="632625" if regioncode=="632625" & year==1999

replace regioncode="632626" if regioncode=="632626" & year==1999

replace regioncode="632721" if regioncode=="632721" & year==1999

replace regioncode="632723" if regioncode=="632723" & year==1999

replace regioncode="632724" if regioncode=="632724" & year==1999

replace regioncode="632725" if regioncode=="632725" & year==1999

replace regioncode="632726" if regioncode=="632726" & year==1999

replace regioncode="632801" if regioncode=="632801" & year==1999

replace regioncode="632802" if regioncode=="632802" & year==1999

replace regioncode="632821" if regioncode=="632821" & year==1999

replace regioncode="632822" if regioncode=="632822" & year==1999

replace regioncode="640104" if regioncode=="640102" & year==1999

replace regioncode="640105" if regioncode=="640103" & year==1999

replace regioncode="640106" if regioncode=="640111" & year==1999

replace regioncode="640121" if regioncode=="640121" & year==1999

replace regioncode="640122" if regioncode=="640122" & year==1999

replace regioncode="640181" if regioncode=="640382" & year==1999

replace regioncode="640202" if regioncode=="640202" & year==1999

replace regioncode="640203" if regioncode=="640203" & year==1999

replace regioncode="640221" if regioncode=="640221" & year==1999

replace regioncode="640302" if regioncode=="640302" & year==1999

replace regioncode="640321" if regioncode=="640321" & year==1999

replace regioncode="640322" if regioncode=="640322" & year==1999

replace regioncode="640323" if regioncode=="640323" & year==1999

replace regioncode="640324" if regioncode=="642127" & year==1999

replace regioncode="640381" if regioncode=="640381" & year==1999

replace regioncode="640402" if regioncode=="642221" & year==1999

replace regioncode="640421" if regioncode=="642222" & year==1999

replace regioncode="640422" if regioncode=="642223" & year==1999

replace regioncode="640423" if regioncode=="642224" & year==1999

replace regioncode="640424" if regioncode=="642225" & year==1999

replace regioncode="640425" if regioncode=="642226" & year==1999

replace regioncode="650102" if regioncode=="650102" & year==1999

replace regioncode="650103" if regioncode=="650103" & year==1999

replace regioncode="650104" if regioncode=="650104" & year==1999

replace regioncode="650105" if regioncode=="650105" & year==1999

replace regioncode="650106" if regioncode=="650106" & year==1999

replace regioncode="650107" if regioncode=="650107" & year==1999

replace regioncode="650108" if regioncode=="650108" & year==1999

replace regioncode="650121" if regioncode=="650121" & year==1999

replace regioncode="650202" if regioncode=="650202" & year==1999

replace regioncode="650203" if regioncode=="650203" & year==1999

replace regioncode="650204" if regioncode=="650204" & year==1999

replace regioncode="650205" if regioncode=="650205" & year==1999

replace regioncode="652101" if regioncode=="652101" & year==1999

replace regioncode="652122" if regioncode=="652122" & year==1999

replace regioncode="652123" if regioncode=="652123" & year==1999

replace regioncode="652201" if regioncode=="652201" & year==1999

replace regioncode="652222" if regioncode=="652222" & year==1999

replace regioncode="652223" if regioncode=="652223" & year==1999

replace regioncode="652301" if regioncode=="652301" & year==1999

replace regioncode="652302" if regioncode=="652302" & year==1999

replace regioncode="652303" if regioncode=="652303" & year==1999

replace regioncode="652323" if regioncode=="652323" & year==1999

replace regioncode="652324" if regioncode=="652324" & year==1999

replace regioncode="652325" if regioncode=="652325" & year==1999

replace regioncode="652327" if regioncode=="652327" & year==1999

replace regioncode="652328" if regioncode=="652328" & year==1999

replace regioncode="652701" if regioncode=="652701" & year==1999

replace regioncode="652722" if regioncode=="652722" & year==1999

replace regioncode="652723" if regioncode=="652723" & year==1999

replace regioncode="652801" if regioncode=="652801" & year==1999

replace regioncode="652822" if regioncode=="652822" & year==1999

replace regioncode="652823" if regioncode=="652823" & year==1999

replace regioncode="652824" if regioncode=="652824" & year==1999

replace regioncode="652825" if regioncode=="652825" & year==1999

replace regioncode="652826" if regioncode=="652826" & year==1999

replace regioncode="652827" if regioncode=="652827" & year==1999

replace regioncode="652828" if regioncode=="652828" & year==1999

replace regioncode="652829" if regioncode=="652829" & year==1999

replace regioncode="652901" if regioncode=="652901" & year==1999

replace regioncode="652922" if regioncode=="652922" & year==1999

replace regioncode="652923" if regioncode=="652923" & year==1999

replace regioncode="652924" if regioncode=="652924" & year==1999

replace regioncode="652925" if regioncode=="652925" & year==1999

replace regioncode="652926" if regioncode=="652926" & year==1999

replace regioncode="652928" if regioncode=="652928" & year==1999

replace regioncode="652929" if regioncode=="652929" & year==1999

replace regioncode="653001" if regioncode=="653001" & year==1999

replace regioncode="653022" if regioncode=="653022" & year==1999

replace regioncode="653023" if regioncode=="653023" & year==1999

replace regioncode="653024" if regioncode=="653024" & year==1999

replace regioncode="653101" if regioncode=="653101" & year==1999

replace regioncode="653121" if regioncode=="653121" & year==1999

replace regioncode="653122" if regioncode=="653122" & year==1999

replace regioncode="653123" if regioncode=="653123" & year==1999

replace regioncode="653124" if regioncode=="653124" & year==1999

replace regioncode="653125" if regioncode=="653125" & year==1999

replace regioncode="653126" if regioncode=="653126" & year==1999

replace regioncode="653127" if regioncode=="653127" & year==1999

replace regioncode="653128" if regioncode=="653128" & year==1999

replace regioncode="653129" if regioncode=="653129" & year==1999

replace regioncode="653130" if regioncode=="653130" & year==1999

replace regioncode="653131" if regioncode=="653131" & year==1999

replace regioncode="653201" if regioncode=="653201" & year==1999

replace regioncode="653221" if regioncode=="653221" & year==1999

replace regioncode="653222" if regioncode=="653222" & year==1999

replace regioncode="653223" if regioncode=="653223" & year==1999

replace regioncode="653224" if regioncode=="653224" & year==1999

replace regioncode="653225" if regioncode=="653225" & year==1999

replace regioncode="653226" if regioncode=="653226" & year==1999

replace regioncode="653227" if regioncode=="653227" & year==1999

replace regioncode="654002" if regioncode=="654101" & year==1999

replace regioncode="654003" if regioncode=="654001" & year==1999

replace regioncode="654021" if regioncode=="654121" & year==1999

replace regioncode="654022" if regioncode=="654122" & year==1999

replace regioncode="654023" if regioncode=="654123" & year==1999

replace regioncode="654024" if regioncode=="654124" & year==1999

replace regioncode="654025" if regioncode=="654125" & year==1999

replace regioncode="654026" if regioncode=="654126" & year==1999

replace regioncode="654027" if regioncode=="654127" & year==1999

replace regioncode="654028" if regioncode=="654128" & year==1999

replace regioncode="654201" if regioncode=="654201" & year==1999

replace regioncode="654202" if regioncode=="654202" & year==1999

replace regioncode="654221" if regioncode=="654221" & year==1999

replace regioncode="654223" if regioncode=="654223" & year==1999

replace regioncode="654224" if regioncode=="654224" & year==1999

replace regioncode="654225" if regioncode=="654225" & year==1999

replace regioncode="654226" if regioncode=="654226" & year==1999

replace regioncode="654301" if regioncode=="654301" & year==1999

replace regioncode="654321" if regioncode=="654321" & year==1999

replace regioncode="654322" if regioncode=="654322" & year==1999

replace regioncode="654323" if regioncode=="654323" & year==1999

replace regioncode="654324" if regioncode=="654324" & year==1999

replace regioncode="654325" if regioncode=="654325" & year==1999

replace regioncode="654326" if regioncode=="654326" & year==1999

replace regioncode="659001" if regioncode=="659001" & year==1999

replace regioncode="110101" if regioncode=="110101" & year==2000

replace regioncode="110102" if regioncode=="110102" & year==2000

replace regioncode="110103" if regioncode=="110103" & year==2000

replace regioncode="110104" if regioncode=="110104" & year==2000

replace regioncode="110105" if regioncode=="110105" & year==2000

replace regioncode="110106" if regioncode=="110106" & year==2000

replace regioncode="110107" if regioncode=="110107" & year==2000

replace regioncode="110108" if regioncode=="110108" & year==2000

replace regioncode="110109" if regioncode=="110109" & year==2000

replace regioncode="110111" if regioncode=="110111" & year==2000

replace regioncode="110112" if regioncode=="110112" & year==2000

replace regioncode="110113" if regioncode=="110113" & year==2000

replace regioncode="110114" if regioncode=="110221" & year==2000

replace regioncode="110115" if regioncode=="110224" & year==2000

replace regioncode="110117" if regioncode=="110226" & year==2000

replace regioncode="110116" if regioncode=="110227" & year==2000

replace regioncode="110228" if regioncode=="110228" & year==2000

replace regioncode="110229" if regioncode=="110229" & year==2000

replace regioncode="120101" if regioncode=="120101" & year==2000

replace regioncode="120102" if regioncode=="120102" & year==2000

replace regioncode="120103" if regioncode=="120103" & year==2000

replace regioncode="120104" if regioncode=="120104" & year==2000

replace regioncode="120105" if regioncode=="120105" & year==2000

replace regioncode="120106" if regioncode=="120106" & year==2000

replace regioncode="120107" if regioncode=="120107" & year==2000

replace regioncode="120108" if regioncode=="120108" & year==2000

replace regioncode="120109" if regioncode=="120109" & year==2000

replace regioncode="120110" if regioncode=="120110" & year==2000

replace regioncode="120111" if regioncode=="120111" & year==2000

replace regioncode="120112" if regioncode=="120112" & year==2000

replace regioncode="120113" if regioncode=="120113" & year==2000

replace regioncode="120114" if regioncode=="120222" & year==2000

replace regioncode="120115" if regioncode=="120224" & year==2000

replace regioncode="120221" if regioncode=="120221" & year==2000

replace regioncode="120223" if regioncode=="120223" & year==2000

replace regioncode="120225" if regioncode=="120225" & year==2000

replace regioncode="130102" if regioncode=="130102" & year==2000

replace regioncode="130103" if regioncode=="130103" & year==2000

replace regioncode="130104" if regioncode=="130104" & year==2000

replace regioncode="130105" if regioncode=="130105" & year==2000

replace regioncode="130107" if regioncode=="130107" & year==2000

replace regioncode="130108" if regioncode=="130106" & year==2000

replace regioncode="130121" if regioncode=="130121" & year==2000

replace regioncode="130123" if regioncode=="130123" & year==2000

replace regioncode="130124" if regioncode=="130124" & year==2000

replace regioncode="130125" if regioncode=="130125" & year==2000

replace regioncode="130126" if regioncode=="130126" & year==2000

replace regioncode="130127" if regioncode=="130127" & year==2000

replace regioncode="130128" if regioncode=="130128" & year==2000

replace regioncode="130129" if regioncode=="130129" & year==2000

replace regioncode="130130" if regioncode=="130130" & year==2000

replace regioncode="130131" if regioncode=="130131" & year==2000

replace regioncode="130132" if regioncode=="130132" & year==2000

replace regioncode="130133" if regioncode=="130133" & year==2000

replace regioncode="130181" if regioncode=="130181" & year==2000

replace regioncode="130182" if regioncode=="130182" & year==2000

replace regioncode="130183" if regioncode=="130183" & year==2000

replace regioncode="130184" if regioncode=="130184" & year==2000

replace regioncode="130185" if regioncode=="130185" & year==2000

replace regioncode="130202" if regioncode=="130202" & year==2000

replace regioncode="130203" if regioncode=="130203" & year==2000

replace regioncode="130204" if regioncode=="130204" & year==2000

replace regioncode="130205" if regioncode=="130205" & year==2000

replace regioncode="130208" if regioncode=="130221" & year==2000

replace regioncode="130223" if regioncode=="130223" & year==2000

replace regioncode="130224" if regioncode=="130224" & year==2000

replace regioncode="130225" if regioncode=="130225" & year==2000

replace regioncode="130227" if regioncode=="130227" & year==2000

replace regioncode="130229" if regioncode=="130229" & year==2000

replace regioncode="130230" if regioncode=="130230" & year==2000

replace regioncode="130281" if regioncode=="130281" & year==2000

replace regioncode="130207" if regioncode=="130282" & year==2000

replace regioncode="130283" if regioncode=="130283" & year==2000

replace regioncode="130302" if regioncode=="130302" & year==2000

replace regioncode="130303" if regioncode=="130303" & year==2000

replace regioncode="130304" if regioncode=="130304" & year==2000

replace regioncode="130321" if regioncode=="130321" & year==2000

replace regioncode="130322" if regioncode=="130322" & year==2000

replace regioncode="130323" if regioncode=="130323" & year==2000

replace regioncode="130324" if regioncode=="130324" & year==2000

replace regioncode="130402" if regioncode=="130402" & year==2000

replace regioncode="130403" if regioncode=="130403" & year==2000

replace regioncode="130404" if regioncode=="130404" & year==2000

replace regioncode="130406" if regioncode=="130406" & year==2000

replace regioncode="130421" if regioncode=="130421" & year==2000

replace regioncode="130423" if regioncode=="130423" & year==2000

replace regioncode="130424" if regioncode=="130424" & year==2000

replace regioncode="130425" if regioncode=="130425" & year==2000

replace regioncode="130426" if regioncode=="130426" & year==2000

replace regioncode="130427" if regioncode=="130427" & year==2000

replace regioncode="130428" if regioncode=="130428" & year==2000

replace regioncode="130429" if regioncode=="130429" & year==2000

replace regioncode="130430" if regioncode=="130430" & year==2000

replace regioncode="130431" if regioncode=="130431" & year==2000

replace regioncode="130432" if regioncode=="130432" & year==2000

replace regioncode="130433" if regioncode=="130433" & year==2000

replace regioncode="130434" if regioncode=="130434" & year==2000

replace regioncode="130435" if regioncode=="130435" & year==2000

replace regioncode="130481" if regioncode=="130481" & year==2000

replace regioncode="130502" if regioncode=="130502" & year==2000

replace regioncode="130503" if regioncode=="130503" & year==2000

replace regioncode="130521" if regioncode=="130521" & year==2000

replace regioncode="130522" if regioncode=="130522" & year==2000

replace regioncode="130523" if regioncode=="130523" & year==2000

replace regioncode="130524" if regioncode=="130524" & year==2000

replace regioncode="130525" if regioncode=="130525" & year==2000

replace regioncode="130526" if regioncode=="130526" & year==2000

replace regioncode="130527" if regioncode=="130527" & year==2000

replace regioncode="130528" if regioncode=="130528" & year==2000

replace regioncode="130529" if regioncode=="130529" & year==2000

replace regioncode="130530" if regioncode=="130530" & year==2000

replace regioncode="130531" if regioncode=="130531" & year==2000

replace regioncode="130532" if regioncode=="130532" & year==2000

replace regioncode="130533" if regioncode=="130533" & year==2000

replace regioncode="130534" if regioncode=="130534" & year==2000

replace regioncode="130535" if regioncode=="130535" & year==2000

replace regioncode="130581" if regioncode=="130581" & year==2000

replace regioncode="130582" if regioncode=="130582" & year==2000

replace regioncode="130602" if regioncode=="130602" & year==2000

replace regioncode="130603" if regioncode=="130603" & year==2000

replace regioncode="130604" if regioncode=="130604" & year==2000

replace regioncode="130621" if regioncode=="130621" & year==2000

replace regioncode="130622" if regioncode=="130622" & year==2000

replace regioncode="130623" if regioncode=="130623" & year==2000

replace regioncode="130624" if regioncode=="130624" & year==2000

replace regioncode="130625" if regioncode=="130625" & year==2000

replace regioncode="130626" if regioncode=="130626" & year==2000

replace regioncode="130627" if regioncode=="130627" & year==2000

replace regioncode="130628" if regioncode=="130628" & year==2000

replace regioncode="130629" if regioncode=="130629" & year==2000

replace regioncode="130630" if regioncode=="130630" & year==2000

replace regioncode="130631" if regioncode=="130631" & year==2000

replace regioncode="130632" if regioncode=="130632" & year==2000

replace regioncode="130633" if regioncode=="130633" & year==2000

replace regioncode="130634" if regioncode=="130634" & year==2000

replace regioncode="130635" if regioncode=="130635" & year==2000

replace regioncode="130636" if regioncode=="130636" & year==2000

replace regioncode="130637" if regioncode=="130637" & year==2000

replace regioncode="130638" if regioncode=="130638" & year==2000

replace regioncode="130681" if regioncode=="130681" & year==2000

replace regioncode="130682" if regioncode=="130682" & year==2000

replace regioncode="130683" if regioncode=="130683" & year==2000

replace regioncode="130684" if regioncode=="130684" & year==2000

replace regioncode="130702" if regioncode=="130702" & year==2000

replace regioncode="130703" if regioncode=="130703" & year==2000

replace regioncode="130705" if regioncode=="130705" & year==2000

replace regioncode="130706" if regioncode=="130706" & year==2000

replace regioncode="130721" if regioncode=="130721" & year==2000

replace regioncode="130722" if regioncode=="130722" & year==2000

replace regioncode="130723" if regioncode=="130723" & year==2000

replace regioncode="130724" if regioncode=="130724" & year==2000

replace regioncode="130725" if regioncode=="130725" & year==2000

replace regioncode="130726" if regioncode=="130726" & year==2000

replace regioncode="130727" if regioncode=="130727" & year==2000

replace regioncode="130728" if regioncode=="130728" & year==2000

replace regioncode="130729" if regioncode=="130729" & year==2000

replace regioncode="130730" if regioncode=="130730" & year==2000

replace regioncode="130731" if regioncode=="130731" & year==2000

replace regioncode="130732" if regioncode=="130732" & year==2000

replace regioncode="130733" if regioncode=="130733" & year==2000

replace regioncode="130802" if regioncode=="130802" & year==2000

replace regioncode="130803" if regioncode=="130803" & year==2000

replace regioncode="130804" if regioncode=="130804" & year==2000

replace regioncode="130821" if regioncode=="130821" & year==2000

replace regioncode="130822" if regioncode=="130822" & year==2000

replace regioncode="130823" if regioncode=="130823" & year==2000

replace regioncode="130824" if regioncode=="130824" & year==2000

replace regioncode="130825" if regioncode=="130825" & year==2000

replace regioncode="130826" if regioncode=="130826" & year==2000

replace regioncode="130827" if regioncode=="130827" & year==2000

replace regioncode="130828" if regioncode=="130828" & year==2000

replace regioncode="130902" if regioncode=="130902" & year==2000

replace regioncode="130903" if regioncode=="130903" & year==2000

replace regioncode="130921" if regioncode=="130921" & year==2000

replace regioncode="130922" if regioncode=="130922" & year==2000

replace regioncode="130923" if regioncode=="130923" & year==2000

replace regioncode="130924" if regioncode=="130924" & year==2000

replace regioncode="130925" if regioncode=="130925" & year==2000

replace regioncode="130926" if regioncode=="130926" & year==2000

replace regioncode="130927" if regioncode=="130927" & year==2000

replace regioncode="130928" if regioncode=="130928" & year==2000

replace regioncode="130929" if regioncode=="130929" & year==2000

replace regioncode="130930" if regioncode=="130930" & year==2000

replace regioncode="130981" if regioncode=="130981" & year==2000

replace regioncode="130982" if regioncode=="130982" & year==2000

replace regioncode="130983" if regioncode=="130983" & year==2000

replace regioncode="130984" if regioncode=="130984" & year==2000

replace regioncode="131002" if regioncode=="131002" & year==2000

replace regioncode="131003" if regioncode=="131003" & year==2000

replace regioncode="131022" if regioncode=="131022" & year==2000

replace regioncode="131023" if regioncode=="131023" & year==2000

replace regioncode="131024" if regioncode=="131024" & year==2000

replace regioncode="131025" if regioncode=="131025" & year==2000

replace regioncode="131026" if regioncode=="131026" & year==2000

replace regioncode="131028" if regioncode=="131028" & year==2000

replace regioncode="131081" if regioncode=="131081" & year==2000

replace regioncode="131082" if regioncode=="131082" & year==2000

replace regioncode="131102" if regioncode=="131102" & year==2000

replace regioncode="131121" if regioncode=="131121" & year==2000

replace regioncode="131122" if regioncode=="131122" & year==2000

replace regioncode="131123" if regioncode=="131123" & year==2000

replace regioncode="131124" if regioncode=="131124" & year==2000

replace regioncode="131125" if regioncode=="131125" & year==2000

replace regioncode="131126" if regioncode=="131126" & year==2000

replace regioncode="131127" if regioncode=="131127" & year==2000

replace regioncode="131128" if regioncode=="131128" & year==2000

replace regioncode="131181" if regioncode=="131181" & year==2000

replace regioncode="131182" if regioncode=="131182" & year==2000

replace regioncode="140105" if regioncode=="140105" & year==2000

replace regioncode="140106" if regioncode=="140106" & year==2000

replace regioncode="140107" if regioncode=="140107" & year==2000

replace regioncode="140108" if regioncode=="140108" & year==2000

replace regioncode="140109" if regioncode=="140109" & year==2000

replace regioncode="140110" if regioncode=="140110" & year==2000

replace regioncode="140121" if regioncode=="140121" & year==2000

replace regioncode="140122" if regioncode=="140122" & year==2000

replace regioncode="140123" if regioncode=="140123" & year==2000

replace regioncode="140181" if regioncode=="140181" & year==2000

replace regioncode="140202" if regioncode=="140202" & year==2000

replace regioncode="140203" if regioncode=="140203" & year==2000

replace regioncode="140211" if regioncode=="140211" & year==2000

replace regioncode="140212" if regioncode=="140212" & year==2000

replace regioncode="140221" if regioncode=="140221" & year==2000

replace regioncode="140222" if regioncode=="140222" & year==2000

replace regioncode="140223" if regioncode=="140223" & year==2000

replace regioncode="140224" if regioncode=="140224" & year==2000

replace regioncode="140225" if regioncode=="140225" & year==2000

replace regioncode="140226" if regioncode=="140226" & year==2000

replace regioncode="140227" if regioncode=="140227" & year==2000

replace regioncode="140302" if regioncode=="140301" & year==2000

replace regioncode="140303" if regioncode=="140303" & year==2000

replace regioncode="140311" if regioncode=="140311" & year==2000

replace regioncode="140321" if regioncode=="140321" & year==2000

replace regioncode="140322" if regioncode=="140322" & year==2000

replace regioncode="140402" if regioncode=="140402" & year==2000

replace regioncode="140411" if regioncode=="140411" & year==2000

replace regioncode="140421" if regioncode=="140421" & year==2000

replace regioncode="140423" if regioncode=="140423" & year==2000

replace regioncode="140424" if regioncode=="140424" & year==2000

replace regioncode="140425" if regioncode=="140425" & year==2000

replace regioncode="140426" if regioncode=="140426" & year==2000

replace regioncode="140427" if regioncode=="140427" & year==2000

replace regioncode="140428" if regioncode=="140428" & year==2000

replace regioncode="140429" if regioncode=="140429" & year==2000

replace regioncode="140430" if regioncode=="140430" & year==2000

replace regioncode="140431" if regioncode=="140431" & year==2000

replace regioncode="140481" if regioncode=="140481" & year==2000

replace regioncode="140502" if regioncode=="140502" & year==2000

replace regioncode="140521" if regioncode=="140521" & year==2000

replace regioncode="140522" if regioncode=="140522" & year==2000

replace regioncode="140524" if regioncode=="140524" & year==2000

replace regioncode="140525" if regioncode=="140525" & year==2000

replace regioncode="140581" if regioncode=="140581" & year==2000

replace regioncode="140602" if regioncode=="140602" & year==2000

replace regioncode="140603" if regioncode=="140603" & year==2000

replace regioncode="140621" if regioncode=="140621" & year==2000

replace regioncode="140622" if regioncode=="140622" & year==2000

replace regioncode="140623" if regioncode=="140623" & year==2000

replace regioncode="140624" if regioncode=="140624" & year==2000

replace regioncode="140902" if regioncode=="140902" & year==2000

replace regioncode="140981" if regioncode=="140981" & year==2000

replace regioncode="140921" if regioncode=="140921" & year==2000

replace regioncode="140922" if regioncode=="140922" & year==2000

replace regioncode="140923" if regioncode=="140923" & year==2000

replace regioncode="140924" if regioncode=="140924" & year==2000

replace regioncode="140925" if regioncode=="140925" & year==2000

replace regioncode="140926" if regioncode=="140926" & year==2000

replace regioncode="140927" if regioncode=="140927" & year==2000

replace regioncode="140928" if regioncode=="140928" & year==2000

replace regioncode="140929" if regioncode=="140929" & year==2000

replace regioncode="140930" if regioncode=="140930" & year==2000

replace regioncode="140931" if regioncode=="140931" & year==2000

replace regioncode="140932" if regioncode=="140932" & year==2000

replace regioncode="141181" if regioncode=="142301" & year==2000

replace regioncode="141102" if regioncode=="142302" & year==2000

replace regioncode="141182" if regioncode=="142303" & year==2000

replace regioncode="141121" if regioncode=="142322" & year==2000

replace regioncode="141122" if regioncode=="142323" & year==2000

replace regioncode="141123" if regioncode=="142325" & year==2000

replace regioncode="141124" if regioncode=="142326" & year==2000

replace regioncode="141125" if regioncode=="142327" & year==2000

replace regioncode="141126" if regioncode=="142328" & year==2000

replace regioncode="141127" if regioncode=="142329" & year==2000

replace regioncode="141128" if regioncode=="142330" & year==2000

replace regioncode="141129" if regioncode=="142332" & year==2000

replace regioncode="141130" if regioncode=="142333" & year==2000

replace regioncode="140702" if regioncode=="140702" & year==2000

replace regioncode="140781" if regioncode=="140781" & year==2000

replace regioncode="140721" if regioncode=="140721" & year==2000

replace regioncode="140722" if regioncode=="140722" & year==2000

replace regioncode="140723" if regioncode=="140723" & year==2000

replace regioncode="140724" if regioncode=="140724" & year==2000

replace regioncode="140725" if regioncode=="140725" & year==2000

replace regioncode="140726" if regioncode=="140726" & year==2000

replace regioncode="140727" if regioncode=="140727" & year==2000

replace regioncode="140728" if regioncode=="140728" & year==2000

replace regioncode="140729" if regioncode=="140729" & year==2000

replace regioncode="141002" if regioncode=="141002" & year==2000

replace regioncode="141081" if regioncode=="141081" & year==2000

replace regioncode="141082" if regioncode=="141082" & year==2000

replace regioncode="141021" if regioncode=="141021" & year==2000

replace regioncode="141022" if regioncode=="141022" & year==2000

replace regioncode="141023" if regioncode=="141023" & year==2000

replace regioncode="141024" if regioncode=="141024" & year==2000

replace regioncode="141025" if regioncode=="141025" & year==2000

replace regioncode="141026" if regioncode=="141026" & year==2000

replace regioncode="141027" if regioncode=="141027" & year==2000

replace regioncode="141028" if regioncode=="141028" & year==2000

replace regioncode="141029" if regioncode=="141029" & year==2000

replace regioncode="141033" if regioncode=="141033" & year==2000

replace regioncode="141030" if regioncode=="141030" & year==2000

replace regioncode="141032" if regioncode=="141032" & year==2000

replace regioncode="141031" if regioncode=="141031" & year==2000

replace regioncode="141034" if regioncode=="141034" & year==2000

replace regioncode="140802" if regioncode=="140802" & year==2000

replace regioncode="140881" if regioncode=="140881" & year==2000

replace regioncode="140882" if regioncode=="140882" & year==2000

replace regioncode="140830" if regioncode=="140830" & year==2000

replace regioncode="140821" if regioncode=="140821" & year==2000

replace regioncode="140822" if regioncode=="140822" & year==2000

replace regioncode="140825" if regioncode=="140825" & year==2000

replace regioncode="140824" if regioncode=="140824" & year==2000

replace regioncode="140823" if regioncode=="140823" & year==2000

replace regioncode="140828" if regioncode=="140828" & year==2000

replace regioncode="140826" if regioncode=="140826" & year==2000

replace regioncode="140829" if regioncode=="140829" & year==2000

replace regioncode="140827" if regioncode=="140827" & year==2000

replace regioncode="150102" if regioncode=="150102" & year==2000

replace regioncode="150103" if regioncode=="150103" & year==2000

replace regioncode="150104" if regioncode=="150104" & year==2000

replace regioncode="150105" if regioncode=="150105" & year==2000

replace regioncode="150121" if regioncode=="150121" & year==2000

replace regioncode="150122" if regioncode=="150122" & year==2000

replace regioncode="150123" if regioncode=="150123" & year==2000

replace regioncode="150124" if regioncode=="150124" & year==2000

replace regioncode="150125" if regioncode=="150125" & year==2000

replace regioncode="150202" if regioncode=="150202" & year==2000

replace regioncode="150203" if regioncode=="150203" & year==2000

replace regioncode="150204" if regioncode=="150204" & year==2000

replace regioncode="150205" if regioncode=="150205" & year==2000

replace regioncode="150206" if regioncode=="150206" & year==2000

replace regioncode="150207" if regioncode=="150207" & year==2000

replace regioncode="150221" if regioncode=="150221" & year==2000

replace regioncode="150222" if regioncode=="150222" & year==2000

replace regioncode="150223" if regioncode=="150223" & year==2000

replace regioncode="150302" if regioncode=="150302" & year==2000

replace regioncode="150303" if regioncode=="150303" & year==2000

replace regioncode="150304" if regioncode=="150304" & year==2000

replace regioncode="150402" if regioncode=="150402" & year==2000

replace regioncode="150403" if regioncode=="150403" & year==2000

replace regioncode="150404" if regioncode=="150404" & year==2000

replace regioncode="150421" if regioncode=="150421" & year==2000

replace regioncode="150422" if regioncode=="150422" & year==2000

replace regioncode="150423" if regioncode=="150423" & year==2000

replace regioncode="150424" if regioncode=="150424" & year==2000

replace regioncode="150425" if regioncode=="150425" & year==2000

replace regioncode="150426" if regioncode=="150426" & year==2000

replace regioncode="150428" if regioncode=="150428" & year==2000

replace regioncode="150429" if regioncode=="150429" & year==2000

replace regioncode="150430" if regioncode=="150430" & year==2000

replace regioncode="150702" if regioncode=="152101" & year==2000

replace regioncode="150781" if regioncode=="152102" & year==2000

replace regioncode="150783" if regioncode=="152103" & year==2000

replace regioncode="150782" if regioncode=="152104" & year==2000

replace regioncode="150785" if regioncode=="152105" & year==2000

replace regioncode="150784" if regioncode=="152106" & year==2000

replace regioncode="150721" if regioncode=="152122" & year==2000

replace regioncode="150722" if regioncode=="152123" & year==2000

replace regioncode="150723" if regioncode=="152127" & year==2000

replace regioncode="150724" if regioncode=="152128" & year==2000

replace regioncode="150727" if regioncode=="152129" & year==2000

replace regioncode="150726" if regioncode=="152130" & year==2000

replace regioncode="150725" if regioncode=="152131" & year==2000

replace regioncode="152201" if regioncode=="152201" & year==2000

replace regioncode="152202" if regioncode=="152202" & year==2000

replace regioncode="152221" if regioncode=="152221" & year==2000

replace regioncode="152222" if regioncode=="152222" & year==2000

replace regioncode="152223" if regioncode=="152223" & year==2000

replace regioncode="152224" if regioncode=="152224" & year==2000

replace regioncode="150502" if regioncode=="150502" & year==2000

replace regioncode="150581" if regioncode=="150581" & year==2000

replace regioncode="150521" if regioncode=="150521" & year==2000

replace regioncode="150522" if regioncode=="150522" & year==2000

replace regioncode="150523" if regioncode=="150523" & year==2000

replace regioncode="150524" if regioncode=="150524" & year==2000

replace regioncode="150525" if regioncode=="150525" & year==2000

replace regioncode="150526" if regioncode=="150526" & year==2000

replace regioncode="152501" if regioncode=="152501" & year==2000

replace regioncode="152502" if regioncode=="152502" & year==2000

replace regioncode="152522" if regioncode=="152522" & year==2000

replace regioncode="152523" if regioncode=="152523" & year==2000

replace regioncode="152524" if regioncode=="152524" & year==2000

replace regioncode="152525" if regioncode=="152525" & year==2000

replace regioncode="152526" if regioncode=="152526" & year==2000

replace regioncode="152527" if regioncode=="152527" & year==2000

replace regioncode="152528" if regioncode=="152528" & year==2000

replace regioncode="152529" if regioncode=="152529" & year==2000

replace regioncode="152530" if regioncode=="152530" & year==2000

replace regioncode="152531" if regioncode=="152531" & year==2000

replace regioncode="152601" if regioncode=="152601" & year==2000

replace regioncode="152602" if regioncode=="152602" & year==2000

replace regioncode="152624" if regioncode=="152624" & year==2000

replace regioncode="152625" if regioncode=="152625" & year==2000

replace regioncode="152626" if regioncode=="152626" & year==2000

replace regioncode="152627" if regioncode=="152627" & year==2000

replace regioncode="152629" if regioncode=="152629" & year==2000

replace regioncode="152630" if regioncode=="152630" & year==2000

replace regioncode="152631" if regioncode=="152631" & year==2000

replace regioncode="152632" if regioncode=="152632" & year==2000

replace regioncode="152634" if regioncode=="152634" & year==2000

replace regioncode="150602" if regioncode=="152701" & year==2000

replace regioncode="150621" if regioncode=="152722" & year==2000

replace regioncode="150622" if regioncode=="152723" & year==2000

replace regioncode="150623" if regioncode=="152724" & year==2000

replace regioncode="150624" if regioncode=="152725" & year==2000

replace regioncode="150625" if regioncode=="152726" & year==2000

replace regioncode="150626" if regioncode=="152727" & year==2000

replace regioncode="150627" if regioncode=="152728" & year==2000

replace regioncode="152801" if regioncode=="152801" & year==2000

replace regioncode="152822" if regioncode=="152822" & year==2000

replace regioncode="152823" if regioncode=="152823" & year==2000

replace regioncode="152824" if regioncode=="152824" & year==2000

replace regioncode="152825" if regioncode=="152825" & year==2000

replace regioncode="152826" if regioncode=="152826" & year==2000

replace regioncode="152827" if regioncode=="152827" & year==2000

replace regioncode="152921" if regioncode=="152921" & year==2000

replace regioncode="152922" if regioncode=="152922" & year==2000

replace regioncode="152923" if regioncode=="152923" & year==2000

replace regioncode="210102" if regioncode=="210102" & year==2000

replace regioncode="210103" if regioncode=="210103" & year==2000

replace regioncode="210104" if regioncode=="210104" & year==2000

replace regioncode="210105" if regioncode=="210105" & year==2000

replace regioncode="210106" if regioncode=="210106" & year==2000

replace regioncode="210111" if regioncode=="210111" & year==2000

replace regioncode="210112" if regioncode=="210112" & year==2000

replace regioncode="210113" if regioncode=="210113" & year==2000

replace regioncode="210114" if regioncode=="210114" & year==2000

replace regioncode="210122" if regioncode=="210122" & year==2000

replace regioncode="210123" if regioncode=="210123" & year==2000

replace regioncode="210124" if regioncode=="210124" & year==2000

replace regioncode="210181" if regioncode=="210181" & year==2000

replace regioncode="210202" if regioncode=="210202" & year==2000

replace regioncode="210203" if regioncode=="210203" & year==2000

replace regioncode="210204" if regioncode=="210204" & year==2000

replace regioncode="210211" if regioncode=="210211" & year==2000

replace regioncode="210212" if regioncode=="210212" & year==2000

replace regioncode="210213" if regioncode=="210213" & year==2000

replace regioncode="210224" if regioncode=="210224" & year==2000

replace regioncode="210281" if regioncode=="210281" & year==2000

replace regioncode="210282" if regioncode=="210282" & year==2000

replace regioncode="210283" if regioncode=="210283" & year==2000

replace regioncode="210302" if regioncode=="210302" & year==2000

replace regioncode="210303" if regioncode=="210303" & year==2000

replace regioncode="210304" if regioncode=="210304" & year==2000

replace regioncode="210311" if regioncode=="210311" & year==2000

replace regioncode="210321" if regioncode=="210321" & year==2000

replace regioncode="210323" if regioncode=="210323" & year==2000

replace regioncode="210381" if regioncode=="210381" & year==2000

replace regioncode="210402" if regioncode=="210402" & year==2000

replace regioncode="210403" if regioncode=="210403" & year==2000

replace regioncode="210404" if regioncode=="210404" & year==2000

replace regioncode="210411" if regioncode=="210411" & year==2000

replace regioncode="210421" if regioncode=="210421" & year==2000

replace regioncode="210422" if regioncode=="210422" & year==2000

replace regioncode="210423" if regioncode=="210423" & year==2000

replace regioncode="210502" if regioncode=="210502" & year==2000

replace regioncode="210503" if regioncode=="210503" & year==2000

replace regioncode="210504" if regioncode=="210504" & year==2000

replace regioncode="210505" if regioncode=="210505" & year==2000

replace regioncode="210521" if regioncode=="210521" & year==2000

replace regioncode="210522" if regioncode=="210522" & year==2000

replace regioncode="210602" if regioncode=="210602" & year==2000

replace regioncode="210603" if regioncode=="210603" & year==2000

replace regioncode="210604" if regioncode=="210604" & year==2000

replace regioncode="210624" if regioncode=="210624" & year==2000

replace regioncode="210681" if regioncode=="210681" & year==2000

replace regioncode="210682" if regioncode=="210682" & year==2000

replace regioncode="210702" if regioncode=="210702" & year==2000

replace regioncode="210703" if regioncode=="210703" & year==2000

replace regioncode="210711" if regioncode=="210711" & year==2000

replace regioncode="210726" if regioncode=="210726" & year==2000

replace regioncode="210727" if regioncode=="210727" & year==2000

replace regioncode="210781" if regioncode=="210781" & year==2000

replace regioncode="210782" if regioncode=="210782" & year==2000

replace regioncode="210802" if regioncode=="210802" & year==2000

replace regioncode="210803" if regioncode=="210803" & year==2000

replace regioncode="210804" if regioncode=="210804" & year==2000

replace regioncode="210811" if regioncode=="210811" & year==2000

replace regioncode="210881" if regioncode=="210881" & year==2000

replace regioncode="210882" if regioncode=="210882" & year==2000

replace regioncode="210902" if regioncode=="210902" & year==2000

replace regioncode="210903" if regioncode=="210903" & year==2000

replace regioncode="210904" if regioncode=="210904" & year==2000

replace regioncode="210905" if regioncode=="210905" & year==2000

replace regioncode="210911" if regioncode=="210911" & year==2000

replace regioncode="210921" if regioncode=="210921" & year==2000

replace regioncode="210922" if regioncode=="210922" & year==2000

replace regioncode="211002" if regioncode=="211002" & year==2000

replace regioncode="211003" if regioncode=="211003" & year==2000

replace regioncode="211004" if regioncode=="211004" & year==2000

replace regioncode="211005" if regioncode=="211005" & year==2000

replace regioncode="211011" if regioncode=="211011" & year==2000

replace regioncode="211021" if regioncode=="211021" & year==2000

replace regioncode="211081" if regioncode=="211081" & year==2000

replace regioncode="211102" if regioncode=="211102" & year==2000

replace regioncode="211103" if regioncode=="211103" & year==2000

replace regioncode="211121" if regioncode=="211121" & year==2000

replace regioncode="211122" if regioncode=="211122" & year==2000

replace regioncode="211202" if regioncode=="211202" & year==2000

replace regioncode="211204" if regioncode=="211204" & year==2000

replace regioncode="211221" if regioncode=="211221" & year==2000

replace regioncode="211223" if regioncode=="211223" & year==2000

replace regioncode="211224" if regioncode=="211224" & year==2000

replace regioncode="211281" if regioncode=="211281" & year==2000

replace regioncode="211282" if regioncode=="211282" & year==2000

replace regioncode="211302" if regioncode=="211302" & year==2000

replace regioncode="211303" if regioncode=="211303" & year==2000

replace regioncode="211321" if regioncode=="211321" & year==2000

replace regioncode="211322" if regioncode=="211322" & year==2000

replace regioncode="211324" if regioncode=="211324" & year==2000

replace regioncode="211381" if regioncode=="211381" & year==2000

replace regioncode="211382" if regioncode=="211382" & year==2000

replace regioncode="211402" if regioncode=="211402" & year==2000

replace regioncode="211403" if regioncode=="211403" & year==2000

replace regioncode="211404" if regioncode=="211404" & year==2000

replace regioncode="211421" if regioncode=="211421" & year==2000

replace regioncode="211422" if regioncode=="211422" & year==2000

replace regioncode="211481" if regioncode=="211481" & year==2000

replace regioncode="220102" if regioncode=="220102" & year==2000

replace regioncode="220103" if regioncode=="220103" & year==2000

replace regioncode="220104" if regioncode=="220104" & year==2000

replace regioncode="220105" if regioncode=="220105" & year==2000

replace regioncode="220106" if regioncode=="220106" & year==2000

replace regioncode="220112" if regioncode=="220112" & year==2000

replace regioncode="220122" if regioncode=="220122" & year==2000

replace regioncode="220181" if regioncode=="220181" & year==2000

replace regioncode="220182" if regioncode=="220182" & year==2000

replace regioncode="220183" if regioncode=="220183" & year==2000

replace regioncode="220202" if regioncode=="220202" & year==2000

replace regioncode="220203" if regioncode=="220203" & year==2000

replace regioncode="220204" if regioncode=="220204" & year==2000

replace regioncode="220211" if regioncode=="220205" & year==2000

replace regioncode="220221" if regioncode=="220221" & year==2000

replace regioncode="220281" if regioncode=="220281" & year==2000

replace regioncode="220282" if regioncode=="220282" & year==2000

replace regioncode="220283" if regioncode=="220283" & year==2000

replace regioncode="220284" if regioncode=="220284" & year==2000

replace regioncode="220302" if regioncode=="220302" & year==2000

replace regioncode="220303" if regioncode=="220303" & year==2000

replace regioncode="220322" if regioncode=="220322" & year==2000

replace regioncode="220323" if regioncode=="220323" & year==2000

replace regioncode="220381" if regioncode=="220381" & year==2000

replace regioncode="220382" if regioncode=="220382" & year==2000

replace regioncode="220402" if regioncode=="220402" & year==2000

replace regioncode="220403" if regioncode=="220403" & year==2000

replace regioncode="220421" if regioncode=="220421" & year==2000

replace regioncode="220422" if regioncode=="220422" & year==2000

replace regioncode="220502" if regioncode=="220502" & year==2000

replace regioncode="220503" if regioncode=="220503" & year==2000

replace regioncode="220521" if regioncode=="220521" & year==2000

replace regioncode="220523" if regioncode=="220523" & year==2000

replace regioncode="220524" if regioncode=="220524" & year==2000

replace regioncode="220581" if regioncode=="220581" & year==2000

replace regioncode="220582" if regioncode=="220582" & year==2000

replace regioncode="220602" if regioncode=="220602" & year==2000

replace regioncode="220621" if regioncode=="220621" & year==2000

replace regioncode="220622" if regioncode=="220622" & year==2000

replace regioncode="220623" if regioncode=="220623" & year==2000

replace regioncode="220625" if regioncode=="220624" & year==2000

replace regioncode="220681" if regioncode=="220681" & year==2000

replace regioncode="220702" if regioncode=="220702" & year==2000

replace regioncode="220721" if regioncode=="220721" & year==2000

replace regioncode="220722" if regioncode=="220722" & year==2000

replace regioncode="220723" if regioncode=="220723" & year==2000

replace regioncode="220724" if regioncode=="220724" & year==2000

replace regioncode="220802" if regioncode=="220802" & year==2000

replace regioncode="220821" if regioncode=="220821" & year==2000

replace regioncode="220822" if regioncode=="220822" & year==2000

replace regioncode="220881" if regioncode=="220881" & year==2000

replace regioncode="220882" if regioncode=="220882" & year==2000

replace regioncode="222401" if regioncode=="222401" & year==2000

replace regioncode="222402" if regioncode=="222402" & year==2000

replace regioncode="222403" if regioncode=="222403" & year==2000

replace regioncode="222404" if regioncode=="222404" & year==2000

replace regioncode="222405" if regioncode=="222405" & year==2000

replace regioncode="222406" if regioncode=="222406" & year==2000

replace regioncode="222424" if regioncode=="222424" & year==2000

replace regioncode="222426" if regioncode=="222426" & year==2000

replace regioncode="230102" if regioncode=="230102" & year==2000

replace regioncode="230103" if regioncode=="230103" & year==2000

replace regioncode="230104" if regioncode=="230104" & year==2000

replace regioncode="230105" if regioncode=="230105" & year==2000

replace regioncode="230107" if regioncode=="230107" & year==2000

replace regioncode="230108" if regioncode=="230108" & year==2000

replace regioncode="230121" if regioncode=="230121" & year==2000

replace regioncode="230123" if regioncode=="230123" & year==2000

replace regioncode="230124" if regioncode=="230124" & year==2000

replace regioncode="230125" if regioncode=="230125" & year==2000

replace regioncode="230126" if regioncode=="230126" & year==2000

replace regioncode="230127" if regioncode=="230127" & year==2000

replace regioncode="230128" if regioncode=="230128" & year==2000

replace regioncode="230129" if regioncode=="230129" & year==2000

replace regioncode="230181" if regioncode=="230181" & year==2000

replace regioncode="230182" if regioncode=="230182" & year==2000

replace regioncode="230183" if regioncode=="230183" & year==2000

replace regioncode="230184" if regioncode=="230184" & year==2000

replace regioncode="230202" if regioncode=="230202" & year==2000

replace regioncode="230203" if regioncode=="230203" & year==2000

replace regioncode="230204" if regioncode=="230204" & year==2000

replace regioncode="230205" if regioncode=="230205" & year==2000

replace regioncode="230206" if regioncode=="230206" & year==2000

replace regioncode="230207" if regioncode=="230207" & year==2000

replace regioncode="230208" if regioncode=="230208" & year==2000

replace regioncode="230221" if regioncode=="230221" & year==2000

replace regioncode="230223" if regioncode=="230223" & year==2000

replace regioncode="230224" if regioncode=="230224" & year==2000

replace regioncode="230225" if regioncode=="230225" & year==2000

replace regioncode="230227" if regioncode=="230227" & year==2000

replace regioncode="230229" if regioncode=="230229" & year==2000

replace regioncode="230230" if regioncode=="230230" & year==2000

replace regioncode="230231" if regioncode=="230231" & year==2000

replace regioncode="230281" if regioncode=="230281" & year==2000

replace regioncode="230302" if regioncode=="230302" & year==2000

replace regioncode="230303" if regioncode=="230303" & year==2000

replace regioncode="230304" if regioncode=="230304" & year==2000

replace regioncode="230305" if regioncode=="230305" & year==2000

replace regioncode="230306" if regioncode=="230306" & year==2000

replace regioncode="230307" if regioncode=="230307" & year==2000

replace regioncode="230321" if regioncode=="230321" & year==2000

replace regioncode="230381" if regioncode=="230381" & year==2000

replace regioncode="230382" if regioncode=="230382" & year==2000

replace regioncode="230402" if regioncode=="230402" & year==2000

replace regioncode="230403" if regioncode=="230403" & year==2000

replace regioncode="230404" if regioncode=="230404" & year==2000

replace regioncode="230405" if regioncode=="230405" & year==2000

replace regioncode="230406" if regioncode=="230406" & year==2000

replace regioncode="230407" if regioncode=="230407" & year==2000

replace regioncode="230421" if regioncode=="230421" & year==2000

replace regioncode="230422" if regioncode=="230422" & year==2000

replace regioncode="230502" if regioncode=="230502" & year==2000

replace regioncode="230503" if regioncode=="230503" & year==2000

replace regioncode="230505" if regioncode=="230505" & year==2000

replace regioncode="230506" if regioncode=="230506" & year==2000

replace regioncode="230521" if regioncode=="230521" & year==2000

replace regioncode="230522" if regioncode=="230522" & year==2000

replace regioncode="230523" if regioncode=="230523" & year==2000

replace regioncode="230524" if regioncode=="230524" & year==2000

replace regioncode="230602" if regioncode=="230602" & year==2000

replace regioncode="230603" if regioncode=="230603" & year==2000

replace regioncode="230604" if regioncode=="230604" & year==2000

replace regioncode="230605" if regioncode=="230605" & year==2000

replace regioncode="230606" if regioncode=="230606" & year==2000

replace regioncode="230621" if regioncode=="230621" & year==2000

replace regioncode="230622" if regioncode=="230622" & year==2000

replace regioncode="230623" if regioncode=="230623" & year==2000

replace regioncode="230624" if regioncode=="230624" & year==2000

replace regioncode="230702" if regioncode=="230702" & year==2000

replace regioncode="230703" if regioncode=="230703" & year==2000

replace regioncode="230704" if regioncode=="230704" & year==2000

replace regioncode="230705" if regioncode=="230705" & year==2000

replace regioncode="230706" if regioncode=="230706" & year==2000

replace regioncode="230707" if regioncode=="230707" & year==2000

replace regioncode="230708" if regioncode=="230708" & year==2000

replace regioncode="230709" if regioncode=="230709" & year==2000

replace regioncode="230710" if regioncode=="230710" & year==2000

replace regioncode="230711" if regioncode=="230711" & year==2000

replace regioncode="230712" if regioncode=="230712" & year==2000

replace regioncode="230713" if regioncode=="230713" & year==2000

replace regioncode="230714" if regioncode=="230714" & year==2000

replace regioncode="230715" if regioncode=="230715" & year==2000

replace regioncode="230716" if regioncode=="230716" & year==2000

replace regioncode="230722" if regioncode=="230722" & year==2000

replace regioncode="230781" if regioncode=="230781" & year==2000

replace regioncode="230802" if regioncode=="230802" & year==2000

replace regioncode="230803" if regioncode=="230803" & year==2000

replace regioncode="230804" if regioncode=="230804" & year==2000

replace regioncode="230805" if regioncode=="230805" & year==2000

replace regioncode="230811" if regioncode=="230811" & year==2000

replace regioncode="230822" if regioncode=="230822" & year==2000

replace regioncode="230826" if regioncode=="230826" & year==2000

replace regioncode="230828" if regioncode=="230828" & year==2000

replace regioncode="230833" if regioncode=="230833" & year==2000

replace regioncode="230881" if regioncode=="230881" & year==2000

replace regioncode="230882" if regioncode=="230882" & year==2000

replace regioncode="230902" if regioncode=="230902" & year==2000

replace regioncode="230903" if regioncode=="230903" & year==2000

replace regioncode="230904" if regioncode=="230904" & year==2000

replace regioncode="230921" if regioncode=="230921" & year==2000

replace regioncode="231002" if regioncode=="231002" & year==2000

replace regioncode="231003" if regioncode=="231003" & year==2000

replace regioncode="231004" if regioncode=="231004" & year==2000

replace regioncode="231005" if regioncode=="231005" & year==2000

replace regioncode="231024" if regioncode=="231024" & year==2000

replace regioncode="231025" if regioncode=="231025" & year==2000

replace regioncode="231081" if regioncode=="231081" & year==2000

replace regioncode="231083" if regioncode=="231083" & year==2000

replace regioncode="231084" if regioncode=="231084" & year==2000

replace regioncode="231085" if regioncode=="231085" & year==2000

replace regioncode="231102" if regioncode=="231102" & year==2000

replace regioncode="231121" if regioncode=="231121" & year==2000

replace regioncode="231123" if regioncode=="231123" & year==2000

replace regioncode="231124" if regioncode=="231124" & year==2000

replace regioncode="231181" if regioncode=="231181" & year==2000

replace regioncode="231182" if regioncode=="231182" & year==2000

replace regioncode="231202" if regioncode=="231202" & year==2000

replace regioncode="231221" if regioncode=="231221" & year==2000

replace regioncode="231222" if regioncode=="231222" & year==2000

replace regioncode="231223" if regioncode=="231223" & year==2000

replace regioncode="231224" if regioncode=="231224" & year==2000

replace regioncode="231225" if regioncode=="231225" & year==2000

replace regioncode="231226" if regioncode=="231226" & year==2000

replace regioncode="231281" if regioncode=="231281" & year==2000

replace regioncode="231282" if regioncode=="231282" & year==2000

replace regioncode="231283" if regioncode=="231283" & year==2000

replace regioncode="232701" if regioncode=="232701" & year==2000

replace regioncode="232702" if regioncode=="232702" & year==2000

replace regioncode="232703" if regioncode=="232703" & year==2000

replace regioncode="232704" if regioncode=="232704" & year==2000

replace regioncode="232721" if regioncode=="232721" & year==2000

replace regioncode="232722" if regioncode=="232722" & year==2000

replace regioncode="232723" if regioncode=="232723" & year==2000

replace regioncode="310101" if regioncode=="310101" & year==2000

replace regioncode="310103" if regioncode=="310103" & year==2000

replace regioncode="310104" if regioncode=="310104" & year==2000

replace regioncode="310105" if regioncode=="310105" & year==2000

replace regioncode="310106" if regioncode=="310106" & year==2000

replace regioncode="310107" if regioncode=="310107" & year==2000

replace regioncode="310108" if regioncode=="310108" & year==2000

replace regioncode="310109" if regioncode=="310109" & year==2000

replace regioncode="310110" if regioncode=="310110" & year==2000

replace regioncode="310112" if regioncode=="310112" & year==2000

replace regioncode="310113" if regioncode=="310113" & year==2000

replace regioncode="310114" if regioncode=="310114" & year==2000

replace regioncode="310115" if regioncode=="310115" & year==2000

replace regioncode="310116" if regioncode=="310116" & year==2000

replace regioncode="310117" if regioncode=="310117" & year==2000

replace regioncode="310118" if regioncode=="310118" & year==2000

replace regioncode="310119" if regioncode=="310225" & year==2000

replace regioncode="310120" if regioncode=="310226" & year==2000

replace regioncode="310230" if regioncode=="310230" & year==2000

replace regioncode="320102" if regioncode=="320102" & year==2000

replace regioncode="320103" if regioncode=="320103" & year==2000

replace regioncode="320104" if regioncode=="320104" & year==2000

replace regioncode="320105" if regioncode=="320105" & year==2000

replace regioncode="320106" if regioncode=="320106" & year==2000

replace regioncode="320107" if regioncode=="320107" & year==2000

replace regioncode="320111" if regioncode=="320111" & year==2000

replace regioncode="320113" if regioncode=="320113" & year==2000

replace regioncode="320114" if regioncode=="320114" & year==2000

replace regioncode="320115" if regioncode=="320121" & year==2000

replace regioncode="320116" if regioncode=="320123" & year==2000

replace regioncode="320124" if regioncode=="320124" & year==2000

replace regioncode="320125" if regioncode=="320125" & year==2000

replace regioncode="320202" if regioncode=="320202" & year==2000

replace regioncode="320203" if regioncode=="320203" & year==2000

replace regioncode="320204" if regioncode=="320204" & year==2000

replace regioncode="320205" if regioncode=="320283" & year==2000

replace regioncode="320211" if regioncode=="320211" & year==2000

replace regioncode="320281" if regioncode=="320281" & year==2000

replace regioncode="320282" if regioncode=="320282" & year==2000

replace regioncode="320302" if regioncode=="320302" & year==2000

replace regioncode="320303" if regioncode=="320303" & year==2000

replace regioncode="320304" if regioncode=="320304" & year==2000

replace regioncode="320305" if regioncode=="320305" & year==2000

replace regioncode="320311" if regioncode=="320311" & year==2000

replace regioncode="320321" if regioncode=="320321" & year==2000

replace regioncode="320322" if regioncode=="320322" & year==2000

replace regioncode="320323" if regioncode=="320323" & year==2000

replace regioncode="320324" if regioncode=="320324" & year==2000

replace regioncode="320381" if regioncode=="320381" & year==2000

replace regioncode="320382" if regioncode=="320382" & year==2000

replace regioncode="320402" if regioncode=="320402" & year==2000

replace regioncode="320404" if regioncode=="320404" & year==2000

replace regioncode="320405" if regioncode=="320405" & year==2000

replace regioncode="320411" if regioncode=="320411" & year==2000

replace regioncode="320412" if regioncode=="320483" & year==2000

replace regioncode="320481" if regioncode=="320481" & year==2000

replace regioncode="320482" if regioncode=="320482" & year==2000

replace regioncode="320502" if regioncode=="320502" & year==2000

replace regioncode="320503" if regioncode=="320503" & year==2000

replace regioncode="320504" if regioncode=="320504" & year==2000

replace regioncode="320505" if regioncode=="320505" & year==2000

replace regioncode="320581" if regioncode=="320581" & year==2000

replace regioncode="320582" if regioncode=="320582" & year==2000

replace regioncode="320583" if regioncode=="320583" & year==2000

replace regioncode="320584" if regioncode=="320584" & year==2000

replace regioncode="320585" if regioncode=="320585" & year==2000

replace regioncode="320586" if regioncode=="320586" & year==2000

replace regioncode="320602" if regioncode=="320602" & year==2000

replace regioncode="320611" if regioncode=="320611" & year==2000

replace regioncode="320621" if regioncode=="320621" & year==2000

replace regioncode="320623" if regioncode=="320623" & year==2000

replace regioncode="320681" if regioncode=="320681" & year==2000

replace regioncode="320682" if regioncode=="320682" & year==2000

replace regioncode="320683" if regioncode=="320683" & year==2000

replace regioncode="320684" if regioncode=="320684" & year==2000

replace regioncode="320703" if regioncode=="320703" & year==2000

replace regioncode="320705" if regioncode=="320705" & year==2000

replace regioncode="320706" if regioncode=="320706" & year==2000

replace regioncode="320721" if regioncode=="320721" & year==2000

replace regioncode="320722" if regioncode=="320722" & year==2000

replace regioncode="320723" if regioncode=="320723" & year==2000

replace regioncode="320724" if regioncode=="320724" & year==2000

replace regioncode="320802" if regioncode=="320802" & year==2000

replace regioncode="320803" if regioncode=="320882" & year==2000

replace regioncode="320804" if regioncode=="320821" & year==2000

replace regioncode="320811" if regioncode=="320811" & year==2000

replace regioncode="320826" if regioncode=="320826" & year==2000

replace regioncode="320829" if regioncode=="320829" & year==2000

replace regioncode="320830" if regioncode=="320830" & year==2000

replace regioncode="320831" if regioncode=="320831" & year==2000

replace regioncode="320902" if regioncode=="320902" & year==2000

replace regioncode="320903" if regioncode=="320928" & year==2000

replace regioncode="320921" if regioncode=="320921" & year==2000

replace regioncode="320922" if regioncode=="320922" & year==2000

replace regioncode="320923" if regioncode=="320923" & year==2000

replace regioncode="320924" if regioncode=="320924" & year==2000

replace regioncode="320925" if regioncode=="320925" & year==2000

replace regioncode="320981" if regioncode=="320981" & year==2000

replace regioncode="320982" if regioncode=="320982" & year==2000

replace regioncode="321002" if regioncode=="321002" & year==2000

replace regioncode="321003" if regioncode=="321027" & year==2000

replace regioncode="321011" if regioncode=="321011" & year==2000

replace regioncode="321023" if regioncode=="321023" & year==2000

replace regioncode="321081" if regioncode=="321081" & year==2000

replace regioncode="321084" if regioncode=="321084" & year==2000

replace regioncode="321088" if regioncode=="321088" & year==2000

replace regioncode="321102" if regioncode=="321102" & year==2000

replace regioncode="321111" if regioncode=="321111" & year==2000

replace regioncode="321112" if regioncode=="321121" & year==2000

replace regioncode="321181" if regioncode=="321181" & year==2000

replace regioncode="321182" if regioncode=="321182" & year==2000

replace regioncode="321183" if regioncode=="321183" & year==2000

replace regioncode="321202" if regioncode=="321202" & year==2000

replace regioncode="321203" if regioncode=="321203" & year==2000

replace regioncode="321281" if regioncode=="321281" & year==2000

replace regioncode="321282" if regioncode=="321282" & year==2000

replace regioncode="321283" if regioncode=="321283" & year==2000

replace regioncode="321284" if regioncode=="321284" & year==2000

replace regioncode="321302" if regioncode=="321302" & year==2000

replace regioncode="321321" if regioncode=="321321" & year==2000

replace regioncode="321322" if regioncode=="321322" & year==2000

replace regioncode="321323" if regioncode=="321323" & year==2000

replace regioncode="321324" if regioncode=="321324" & year==2000

replace regioncode="330102" if regioncode=="330102" & year==2000

replace regioncode="330103" if regioncode=="330103" & year==2000

replace regioncode="330104" if regioncode=="330104" & year==2000

replace regioncode="330105" if regioncode=="330105" & year==2000

replace regioncode="330106" if regioncode=="330106" & year==2000

replace regioncode="330108" if regioncode=="330108" & year==2000

replace regioncode="330109" if regioncode=="330181" & year==2000

replace regioncode="330110" if regioncode=="330184" & year==2000

replace regioncode="330122" if regioncode=="330122" & year==2000

replace regioncode="330127" if regioncode=="330127" & year==2000

replace regioncode="330182" if regioncode=="330182" & year==2000

replace regioncode="330183" if regioncode=="330183" & year==2000

replace regioncode="330185" if regioncode=="330185" & year==2000

replace regioncode="330203" if regioncode=="330203" & year==2000

replace regioncode="330204" if regioncode=="330204" & year==2000

replace regioncode="330205" if regioncode=="330205" & year==2000

replace regioncode="330206" if regioncode=="330206" & year==2000

replace regioncode="330211" if regioncode=="330211" & year==2000

replace regioncode="330212" if regioncode=="330227" & year==2000

replace regioncode="330225" if regioncode=="330225" & year==2000

replace regioncode="330226" if regioncode=="330226" & year==2000

replace regioncode="330281" if regioncode=="330281" & year==2000

replace regioncode="330282" if regioncode=="330282" & year==2000

replace regioncode="330283" if regioncode=="330283" & year==2000

replace regioncode="330302" if regioncode=="330302" & year==2000

replace regioncode="330303" if regioncode=="330303" & year==2000

replace regioncode="330304" if regioncode=="330304" & year==2000

replace regioncode="330322" if regioncode=="330322" & year==2000

replace regioncode="330324" if regioncode=="330324" & year==2000

replace regioncode="330326" if regioncode=="330326" & year==2000

replace regioncode="330327" if regioncode=="330327" & year==2000

replace regioncode="330328" if regioncode=="330328" & year==2000

replace regioncode="330329" if regioncode=="330329" & year==2000

replace regioncode="330381" if regioncode=="330381" & year==2000

replace regioncode="330382" if regioncode=="330382" & year==2000

replace regioncode="330402" if regioncode=="330402" & year==2000

replace regioncode="330411" if regioncode=="330411" & year==2000

replace regioncode="330421" if regioncode=="330421" & year==2000

replace regioncode="330424" if regioncode=="330424" & year==2000

replace regioncode="330481" if regioncode=="330481" & year==2000

replace regioncode="330482" if regioncode=="330482" & year==2000

replace regioncode="330483" if regioncode=="330483" & year==2000

replace regioncode="330501" if regioncode=="330501" & year==2000

replace regioncode="330521" if regioncode=="330521" & year==2000

replace regioncode="330522" if regioncode=="330522" & year==2000

replace regioncode="330523" if regioncode=="330523" & year==2000

replace regioncode="330602" if regioncode=="330602" & year==2000

replace regioncode="330621" if regioncode=="330621" & year==2000

replace regioncode="330624" if regioncode=="330624" & year==2000

replace regioncode="330681" if regioncode=="330681" & year==2000

replace regioncode="330682" if regioncode=="330682" & year==2000

replace regioncode="330683" if regioncode=="330683" & year==2000

replace regioncode="330702" if regioncode=="330702" & year==2000

replace regioncode="330703" if regioncode=="330721" & year==2000

replace regioncode="330723" if regioncode=="330723" & year==2000

replace regioncode="330726" if regioncode=="330726" & year==2000

replace regioncode="330727" if regioncode=="330727" & year==2000

replace regioncode="330781" if regioncode=="330781" & year==2000

replace regioncode="330782" if regioncode=="330782" & year==2000

replace regioncode="330783" if regioncode=="330783" & year==2000

replace regioncode="330784" if regioncode=="330784" & year==2000

replace regioncode="330802" if regioncode=="330802" & year==2000

replace regioncode="330803" if regioncode=="330821" & year==2000

replace regioncode="330822" if regioncode=="330822" & year==2000

replace regioncode="330824" if regioncode=="330824" & year==2000

replace regioncode="330825" if regioncode=="330825" & year==2000

replace regioncode="330881" if regioncode=="330881" & year==2000

replace regioncode="330902" if regioncode=="330902" & year==2000

replace regioncode="330903" if regioncode=="330903" & year==2000

replace regioncode="330921" if regioncode=="330921" & year==2000

replace regioncode="330922" if regioncode=="330922" & year==2000

replace regioncode="331002" if regioncode=="331002" & year==2000

replace regioncode="331003" if regioncode=="331003" & year==2000

replace regioncode="331004" if regioncode=="331004" & year==2000

replace regioncode="331021" if regioncode=="331021" & year==2000

replace regioncode="331022" if regioncode=="331022" & year==2000

replace regioncode="331023" if regioncode=="331023" & year==2000

replace regioncode="331024" if regioncode=="331024" & year==2000

replace regioncode="331081" if regioncode=="331081" & year==2000

replace regioncode="331082" if regioncode=="331082" & year==2000

replace regioncode="331102" if regioncode=="331102" & year==2000

replace regioncode="331121" if regioncode=="331121" & year==2000

replace regioncode="331122" if regioncode=="331122" & year==2000

replace regioncode="331123" if regioncode=="331123" & year==2000

replace regioncode="331124" if regioncode=="331124" & year==2000

replace regioncode="331125" if regioncode=="331125" & year==2000

replace regioncode="331126" if regioncode=="331126" & year==2000

replace regioncode="331127" if regioncode=="331127" & year==2000

replace regioncode="331181" if regioncode=="331181" & year==2000

replace regioncode="340102" if regioncode=="340102" & year==2000

replace regioncode="340103" if regioncode=="340103" & year==2000

replace regioncode="340104" if regioncode=="340104" & year==2000

replace regioncode="340111" if regioncode=="340111" & year==2000

replace regioncode="340121" if regioncode=="340121" & year==2000

replace regioncode="340122" if regioncode=="340122" & year==2000

replace regioncode="340123" if regioncode=="340123" & year==2000

replace regioncode="340202" if regioncode=="340202" & year==2000

replace regioncode="340203" if regioncode=="340203" & year==2000

replace regioncode="340204" if regioncode=="340204" & year==2000

replace regioncode="340207" if regioncode=="340207" & year==2000

replace regioncode="340221" if regioncode=="340221" & year==2000

replace regioncode="340222" if regioncode=="340222" & year==2000

replace regioncode="340223" if regioncode=="340223" & year==2000

replace regioncode="340302" if regioncode=="340302" & year==2000

replace regioncode="340303" if regioncode=="340303" & year==2000

replace regioncode="340304" if regioncode=="340304" & year==2000

replace regioncode="340311" if regioncode=="340311" & year==2000

replace regioncode="340321" if regioncode=="340321" & year==2000

replace regioncode="340322" if regioncode=="340322" & year==2000

replace regioncode="340323" if regioncode=="340323" & year==2000

replace regioncode="340402" if regioncode=="340402" & year==2000

replace regioncode="340403" if regioncode=="340403" & year==2000

replace regioncode="340404" if regioncode=="340404" & year==2000

replace regioncode="340405" if regioncode=="340405" & year==2000

replace regioncode="340406" if regioncode=="340406" & year==2000

replace regioncode="340421" if regioncode=="340421" & year==2000

replace regioncode="340502" if regioncode=="340502" & year==2000

replace regioncode="340503" if regioncode=="340503" & year==2000

replace regioncode="340504" if regioncode=="340504" & year==2000

replace regioncode="340521" if regioncode=="340521" & year==2000

replace regioncode="340602" if regioncode=="340602" & year==2000

replace regioncode="340603" if regioncode=="340603" & year==2000

replace regioncode="340604" if regioncode=="340604" & year==2000

replace regioncode="340621" if regioncode=="340621" & year==2000

replace regioncode="340702" if regioncode=="340702" & year==2000

replace regioncode="340703" if regioncode=="340703" & year==2000

replace regioncode="340711" if regioncode=="340711" & year==2000

replace regioncode="340721" if regioncode=="340721" & year==2000

replace regioncode="340802" if regioncode=="340802" & year==2000

replace regioncode="340803" if regioncode=="340803" & year==2000

replace regioncode="340811" if regioncode=="340811" & year==2000

replace regioncode="340822" if regioncode=="340822" & year==2000

replace regioncode="340823" if regioncode=="340823" & year==2000

replace regioncode="340824" if regioncode=="340824" & year==2000

replace regioncode="340825" if regioncode=="340825" & year==2000

replace regioncode="340826" if regioncode=="340826" & year==2000

replace regioncode="340827" if regioncode=="340827" & year==2000

replace regioncode="340828" if regioncode=="340828" & year==2000

replace regioncode="340881" if regioncode=="340881" & year==2000

replace regioncode="341002" if regioncode=="341002" & year==2000

replace regioncode="341003" if regioncode=="341003" & year==2000

replace regioncode="341004" if regioncode=="341004" & year==2000

replace regioncode="341021" if regioncode=="341021" & year==2000

replace regioncode="341022" if regioncode=="341022" & year==2000

replace regioncode="341023" if regioncode=="341023" & year==2000

replace regioncode="341024" if regioncode=="341024" & year==2000

replace regioncode="341102" if regioncode=="341102" & year==2000

replace regioncode="341103" if regioncode=="341103" & year==2000

replace regioncode="341122" if regioncode=="341122" & year==2000

replace regioncode="341124" if regioncode=="341124" & year==2000

replace regioncode="341125" if regioncode=="341125" & year==2000

replace regioncode="341126" if regioncode=="341126" & year==2000

replace regioncode="341181" if regioncode=="341181" & year==2000

replace regioncode="341182" if regioncode=="341182" & year==2000

replace regioncode="341202" if regioncode=="341202" & year==2000

replace regioncode="341203" if regioncode=="341203" & year==2000

replace regioncode="341204" if regioncode=="341204" & year==2000

replace regioncode="341221" if regioncode=="341221" & year==2000

replace regioncode="341222" if regioncode=="341222" & year==2000

replace regioncode="341621" if regioncode=="341621" & year==2000

replace regioncode="341622" if regioncode=="341622" & year==2000

replace regioncode="341225" if regioncode=="341225" & year==2000

replace regioncode="341226" if regioncode=="341226" & year==2000

replace regioncode="341623" if regioncode=="341623" & year==2000

replace regioncode="341282" if regioncode=="341282" & year==2000

replace regioncode="341302" if regioncode=="341302" & year==2000

replace regioncode="341321" if regioncode=="341321" & year==2000

replace regioncode="341322" if regioncode=="341322" & year==2000

replace regioncode="341323" if regioncode=="341323" & year==2000

replace regioncode="341324" if regioncode=="341324" & year==2000

replace regioncode="341402" if regioncode=="341402" & year==2000

replace regioncode="341421" if regioncode=="341421" & year==2000

replace regioncode="341422" if regioncode=="341422" & year==2000

replace regioncode="341423" if regioncode=="341423" & year==2000

replace regioncode="341424" if regioncode=="341424" & year==2000

replace regioncode="341521" if regioncode=="341521" & year==2000

replace regioncode="341522" if regioncode=="341522" & year==2000

replace regioncode="341523" if regioncode=="341523" & year==2000

replace regioncode="341524" if regioncode=="341524" & year==2000

replace regioncode="341525" if regioncode=="341525" & year==2000

replace regioncode="341802" if regioncode=="341802" & year==2000

replace regioncode="341881" if regioncode=="341881" & year==2000

replace regioncode="341821" if regioncode=="341821" & year==2000

replace regioncode="341822" if regioncode=="341822" & year==2000

replace regioncode="341823" if regioncode=="341823" & year==2000

replace regioncode="341825" if regioncode=="341825" & year==2000

replace regioncode="341824" if regioncode=="341824" & year==2000

replace regioncode="341702" if regioncode=="341702" & year==2000

replace regioncode="341721" if regioncode=="341721" & year==2000

replace regioncode="341722" if regioncode=="341722" & year==2000

replace regioncode="341723" if regioncode=="341723" & year==2000

replace regioncode="341502" if regioncode=="341503" & year==2000

replace regioncode="341602" if regioncode=="341602" & year==2000

replace regioncode="350102" if regioncode=="350102" & year==2000

replace regioncode="350103" if regioncode=="350103" & year==2000

replace regioncode="350104" if regioncode=="350104" & year==2000

replace regioncode="350105" if regioncode=="350105" & year==2000

replace regioncode="350111" if regioncode=="350111" & year==2000

replace regioncode="350121" if regioncode=="350121" & year==2000

replace regioncode="350122" if regioncode=="350122" & year==2000

replace regioncode="350123" if regioncode=="350123" & year==2000

replace regioncode="350124" if regioncode=="350124" & year==2000

replace regioncode="350125" if regioncode=="350125" & year==2000

replace regioncode="350128" if regioncode=="350128" & year==2000

replace regioncode="350181" if regioncode=="350181" & year==2000

replace regioncode="350182" if regioncode=="350182" & year==2000

replace regioncode="350203" if regioncode=="350203" & year==2000

replace regioncode="350205" if regioncode=="350205" & year==2000

replace regioncode="350206" if regioncode=="350206" & year==2000

replace regioncode="350211" if regioncode=="350211" & year==2000

replace regioncode="350212" if regioncode=="350212" & year==2000

replace regioncode="350213" if regioncode=="350213" & year==2000

replace regioncode="350302" if regioncode=="350302" & year==2000

replace regioncode="350303" if regioncode=="350303" & year==2000

replace regioncode="350321" if regioncode=="350321" & year==2000

replace regioncode="350322" if regioncode=="350322" & year==2000

replace regioncode="350402" if regioncode=="350402" & year==2000

replace regioncode="350403" if regioncode=="350403" & year==2000

replace regioncode="350421" if regioncode=="350421" & year==2000

replace regioncode="350423" if regioncode=="350423" & year==2000

replace regioncode="350424" if regioncode=="350424" & year==2000

replace regioncode="350425" if regioncode=="350425" & year==2000

replace regioncode="350426" if regioncode=="350426" & year==2000

replace regioncode="350427" if regioncode=="350427" & year==2000

replace regioncode="350428" if regioncode=="350428" & year==2000

replace regioncode="350429" if regioncode=="350429" & year==2000

replace regioncode="350430" if regioncode=="350430" & year==2000

replace regioncode="350481" if regioncode=="350481" & year==2000

replace regioncode="350502" if regioncode=="350502" & year==2000

replace regioncode="350503" if regioncode=="350503" & year==2000

replace regioncode="350504" if regioncode=="350504" & year==2000

replace regioncode="350505" if regioncode=="350505" & year==2000

replace regioncode="350521" if regioncode=="350521" & year==2000

replace regioncode="350524" if regioncode=="350524" & year==2000

replace regioncode="350525" if regioncode=="350525" & year==2000

replace regioncode="350526" if regioncode=="350526" & year==2000

replace regioncode="350581" if regioncode=="350581" & year==2000

replace regioncode="350582" if regioncode=="350582" & year==2000

replace regioncode="350583" if regioncode=="350583" & year==2000

replace regioncode="350602" if regioncode=="350602" & year==2000

replace regioncode="350603" if regioncode=="350603" & year==2000

replace regioncode="350622" if regioncode=="350622" & year==2000

replace regioncode="350623" if regioncode=="350623" & year==2000

replace regioncode="350624" if regioncode=="350624" & year==2000

replace regioncode="350625" if regioncode=="350625" & year==2000

replace regioncode="350626" if regioncode=="350626" & year==2000

replace regioncode="350627" if regioncode=="350627" & year==2000

replace regioncode="350628" if regioncode=="350628" & year==2000

replace regioncode="350629" if regioncode=="350629" & year==2000

replace regioncode="350681" if regioncode=="350681" & year==2000

replace regioncode="350702" if regioncode=="350702" & year==2000

replace regioncode="350721" if regioncode=="350721" & year==2000

replace regioncode="350722" if regioncode=="350722" & year==2000

replace regioncode="350723" if regioncode=="350723" & year==2000

replace regioncode="350724" if regioncode=="350724" & year==2000

replace regioncode="350725" if regioncode=="350725" & year==2000

replace regioncode="350781" if regioncode=="350781" & year==2000

replace regioncode="350782" if regioncode=="350782" & year==2000

replace regioncode="350783" if regioncode=="350783" & year==2000

replace regioncode="350784" if regioncode=="350784" & year==2000

replace regioncode="350802" if regioncode=="350802" & year==2000

replace regioncode="350821" if regioncode=="350821" & year==2000

replace regioncode="350822" if regioncode=="350822" & year==2000

replace regioncode="350823" if regioncode=="350823" & year==2000

replace regioncode="350824" if regioncode=="350824" & year==2000

replace regioncode="350825" if regioncode=="350825" & year==2000

replace regioncode="350881" if regioncode=="350881" & year==2000

replace regioncode="350902" if regioncode=="350902" & year==2000

replace regioncode="350981" if regioncode=="350981" & year==2000

replace regioncode="350982" if regioncode=="350982" & year==2000

replace regioncode="350921" if regioncode=="350921" & year==2000

replace regioncode="350922" if regioncode=="350922" & year==2000

replace regioncode="350923" if regioncode=="350923" & year==2000

replace regioncode="350924" if regioncode=="350924" & year==2000

replace regioncode="350925" if regioncode=="350925" & year==2000

replace regioncode="350926" if regioncode=="350926" & year==2000

replace regioncode="360102" if regioncode=="360102" & year==2000

replace regioncode="360103" if regioncode=="360103" & year==2000

replace regioncode="360104" if regioncode=="360104" & year==2000

replace regioncode="360105" if regioncode=="360105" & year==2000

replace regioncode="360111" if regioncode=="360111" & year==2000

replace regioncode="360121" if regioncode=="360121" & year==2000

replace regioncode="360122" if regioncode=="360122" & year==2000

replace regioncode="360123" if regioncode=="360123" & year==2000

replace regioncode="360124" if regioncode=="360124" & year==2000

replace regioncode="360202" if regioncode=="360202" & year==2000

replace regioncode="360203" if regioncode=="360203" & year==2000

replace regioncode="360222" if regioncode=="360222" & year==2000

replace regioncode="360281" if regioncode=="360281" & year==2000

replace regioncode="360302" if regioncode=="360302" & year==2000

replace regioncode="360313" if regioncode=="360313" & year==2000

replace regioncode="360321" if regioncode=="360321" & year==2000

replace regioncode="360322" if regioncode=="360322" & year==2000

replace regioncode="360323" if regioncode=="360323" & year==2000

replace regioncode="360402" if regioncode=="360402" & year==2000

replace regioncode="360403" if regioncode=="360403" & year==2000

replace regioncode="360421" if regioncode=="360421" & year==2000

replace regioncode="360423" if regioncode=="360423" & year==2000

replace regioncode="360424" if regioncode=="360424" & year==2000

replace regioncode="360425" if regioncode=="360425" & year==2000

replace regioncode="360426" if regioncode=="360426" & year==2000

replace regioncode="360427" if regioncode=="360427" & year==2000

replace regioncode="360428" if regioncode=="360428" & year==2000

replace regioncode="360429" if regioncode=="360429" & year==2000

replace regioncode="360430" if regioncode=="360430" & year==2000

replace regioncode="360481" if regioncode=="360481" & year==2000

replace regioncode="360502" if regioncode=="360502" & year==2000

replace regioncode="360521" if regioncode=="360521" & year==2000

replace regioncode="360602" if regioncode=="360602" & year==2000

replace regioncode="360622" if regioncode=="360622" & year==2000

replace regioncode="360681" if regioncode=="360681" & year==2000

replace regioncode="360702" if regioncode=="360702" & year==2000

replace regioncode="360721" if regioncode=="360721" & year==2000

replace regioncode="360722" if regioncode=="360722" & year==2000

replace regioncode="360723" if regioncode=="360723" & year==2000

replace regioncode="360724" if regioncode=="360724" & year==2000

replace regioncode="360725" if regioncode=="360725" & year==2000

replace regioncode="360726" if regioncode=="360726" & year==2000

replace regioncode="360727" if regioncode=="360727" & year==2000

replace regioncode="360728" if regioncode=="360728" & year==2000

replace regioncode="360729" if regioncode=="360729" & year==2000

replace regioncode="360730" if regioncode=="360730" & year==2000

replace regioncode="360731" if regioncode=="360731" & year==2000

replace regioncode="360732" if regioncode=="360732" & year==2000

replace regioncode="360733" if regioncode=="360733" & year==2000

replace regioncode="360734" if regioncode=="360734" & year==2000

replace regioncode="360735" if regioncode=="360735" & year==2000

replace regioncode="360781" if regioncode=="360781" & year==2000

replace regioncode="360782" if regioncode=="360782" & year==2000

replace regioncode="360802" if regioncode=="360802" & year==2000

replace regioncode="360821" if regioncode=="360821" & year==2000

replace regioncode="360822" if regioncode=="360822" & year==2000

replace regioncode="360823" if regioncode=="360823" & year==2000

replace regioncode="360824" if regioncode=="360824" & year==2000

replace regioncode="360825" if regioncode=="360825" & year==2000

replace regioncode="360826" if regioncode=="360826" & year==2000

replace regioncode="360827" if regioncode=="360827" & year==2000

replace regioncode="360828" if regioncode=="360828" & year==2000

replace regioncode="360829" if regioncode=="360829" & year==2000

replace regioncode="360830" if regioncode=="360830" & year==2000

replace regioncode="360881" if regioncode=="360881" & year==2000

replace regioncode="360902" if regioncode=="360902" & year==2000

replace regioncode="360921" if regioncode=="360921" & year==2000

replace regioncode="360922" if regioncode=="360922" & year==2000

replace regioncode="360923" if regioncode=="360923" & year==2000

replace regioncode="360924" if regioncode=="360924" & year==2000

replace regioncode="360925" if regioncode=="360925" & year==2000

replace regioncode="360926" if regioncode=="360926" & year==2000

replace regioncode="360981" if regioncode=="360981" & year==2000

replace regioncode="360982" if regioncode=="360982" & year==2000

replace regioncode="360983" if regioncode=="360983" & year==2000

replace regioncode="361002" if regioncode=="361002" & year==2000

replace regioncode="361021" if regioncode=="361021" & year==2000

replace regioncode="361022" if regioncode=="361022" & year==2000

replace regioncode="361023" if regioncode=="361023" & year==2000

replace regioncode="361024" if regioncode=="361024" & year==2000

replace regioncode="361025" if regioncode=="361025" & year==2000

replace regioncode="361026" if regioncode=="361026" & year==2000

replace regioncode="361027" if regioncode=="361027" & year==2000

replace regioncode="361028" if regioncode=="361028" & year==2000

replace regioncode="361029" if regioncode=="361029" & year==2000

replace regioncode="361030" if regioncode=="361030" & year==2000

replace regioncode="361102" if regioncode=="361102" & year==2000

replace regioncode="361121" if regioncode=="361121" & year==2000

replace regioncode="361122" if regioncode=="361122" & year==2000

replace regioncode="361123" if regioncode=="361123" & year==2000

replace regioncode="361124" if regioncode=="361124" & year==2000

replace regioncode="361125" if regioncode=="361125" & year==2000

replace regioncode="361126" if regioncode=="361126" & year==2000

replace regioncode="361127" if regioncode=="361127" & year==2000

replace regioncode="361128" if regioncode=="361128" & year==2000

replace regioncode="361129" if regioncode=="361129" & year==2000

replace regioncode="361130" if regioncode=="361130" & year==2000

replace regioncode="361181" if regioncode=="361181" & year==2000

replace regioncode="370102" if regioncode=="370102" & year==2000

replace regioncode="370103" if regioncode=="370103" & year==2000

replace regioncode="370104" if regioncode=="370104" & year==2000

replace regioncode="370105" if regioncode=="370105" & year==2000

replace regioncode="370112" if regioncode=="370112" & year==2000

replace regioncode="370113" if regioncode=="370123" & year==2000

replace regioncode="370124" if regioncode=="370124" & year==2000

replace regioncode="370125" if regioncode=="370125" & year==2000

replace regioncode="370126" if regioncode=="370126" & year==2000

replace regioncode="370181" if regioncode=="370181" & year==2000

replace regioncode="370202" if regioncode=="370202" & year==2000

replace regioncode="370203" if regioncode=="370203" & year==2000

replace regioncode="370205" if regioncode=="370205" & year==2000

replace regioncode="370211" if regioncode=="370211" & year==2000

replace regioncode="370212" if regioncode=="370212" & year==2000

replace regioncode="370213" if regioncode=="370213" & year==2000

replace regioncode="370214" if regioncode=="370214" & year==2000

replace regioncode="370281" if regioncode=="370281" & year==2000

replace regioncode="370282" if regioncode=="370282" & year==2000

replace regioncode="370283" if regioncode=="370283" & year==2000

replace regioncode="370284" if regioncode=="370284" & year==2000

replace regioncode="370285" if regioncode=="370285" & year==2000

replace regioncode="370302" if regioncode=="370302" & year==2000

replace regioncode="370303" if regioncode=="370303" & year==2000

replace regioncode="370304" if regioncode=="370304" & year==2000

replace regioncode="370305" if regioncode=="370305" & year==2000

replace regioncode="370306" if regioncode=="370306" & year==2000

replace regioncode="370321" if regioncode=="370321" & year==2000

replace regioncode="370322" if regioncode=="370322" & year==2000

replace regioncode="370323" if regioncode=="370323" & year==2000

replace regioncode="370402" if regioncode=="370402" & year==2000

replace regioncode="370403" if regioncode=="370403" & year==2000

replace regioncode="370404" if regioncode=="370404" & year==2000

replace regioncode="370405" if regioncode=="370405" & year==2000

replace regioncode="370406" if regioncode=="370406" & year==2000

replace regioncode="370481" if regioncode=="370481" & year==2000

replace regioncode="370502" if regioncode=="370502" & year==2000

replace regioncode="370503" if regioncode=="370503" & year==2000

replace regioncode="370521" if regioncode=="370521" & year==2000

replace regioncode="370522" if regioncode=="370522" & year==2000

replace regioncode="370523" if regioncode=="370523" & year==2000

replace regioncode="370602" if regioncode=="370602" & year==2000

replace regioncode="370611" if regioncode=="370611" & year==2000

replace regioncode="370612" if regioncode=="370612" & year==2000

replace regioncode="370613" if regioncode=="370613" & year==2000

replace regioncode="370634" if regioncode=="370634" & year==2000

replace regioncode="370681" if regioncode=="370681" & year==2000

replace regioncode="370682" if regioncode=="370682" & year==2000

replace regioncode="370683" if regioncode=="370683" & year==2000

replace regioncode="370684" if regioncode=="370684" & year==2000

replace regioncode="370685" if regioncode=="370685" & year==2000

replace regioncode="370686" if regioncode=="370686" & year==2000

replace regioncode="370687" if regioncode=="370687" & year==2000

replace regioncode="370702" if regioncode=="370702" & year==2000

replace regioncode="370703" if regioncode=="370703" & year==2000

replace regioncode="370704" if regioncode=="370704" & year==2000

replace regioncode="370705" if regioncode=="370705" & year==2000

replace regioncode="370724" if regioncode=="370724" & year==2000

replace regioncode="370725" if regioncode=="370725" & year==2000

replace regioncode="370781" if regioncode=="370781" & year==2000

replace regioncode="370782" if regioncode=="370782" & year==2000

replace regioncode="370783" if regioncode=="370783" & year==2000

replace regioncode="370784" if regioncode=="370784" & year==2000

replace regioncode="370785" if regioncode=="370785" & year==2000

replace regioncode="370786" if regioncode=="370786" & year==2000

replace regioncode="370802" if regioncode=="370802" & year==2000

replace regioncode="370811" if regioncode=="370811" & year==2000

replace regioncode="370826" if regioncode=="370826" & year==2000

replace regioncode="370827" if regioncode=="370827" & year==2000

replace regioncode="370828" if regioncode=="370828" & year==2000

replace regioncode="370829" if regioncode=="370829" & year==2000

replace regioncode="370830" if regioncode=="370830" & year==2000

replace regioncode="370831" if regioncode=="370831" & year==2000

replace regioncode="370832" if regioncode=="370832" & year==2000

replace regioncode="370881" if regioncode=="370881" & year==2000

replace regioncode="370882" if regioncode=="370882" & year==2000

replace regioncode="370883" if regioncode=="370883" & year==2000

replace regioncode="370902" if regioncode=="370902" & year==2000

replace regioncode="370903" if regioncode=="370903" & year==2000

replace regioncode="370921" if regioncode=="370921" & year==2000

replace regioncode="370923" if regioncode=="370923" & year==2000

replace regioncode="370982" if regioncode=="370982" & year==2000

replace regioncode="370983" if regioncode=="370983" & year==2000

replace regioncode="371002" if regioncode=="371002" & year==2000

replace regioncode="371081" if regioncode=="371081" & year==2000

replace regioncode="371082" if regioncode=="371082" & year==2000

replace regioncode="371083" if regioncode=="371083" & year==2000

replace regioncode="371102" if regioncode=="371102" & year==2000

replace regioncode="371121" if regioncode=="371121" & year==2000

replace regioncode="371122" if regioncode=="371122" & year==2000

replace regioncode="371202" if regioncode=="371202" & year==2000

replace regioncode="371203" if regioncode=="371203" & year==2000

replace regioncode="371302" if regioncode=="371302" & year==2000

replace regioncode="371311" if regioncode=="371311" & year==2000

replace regioncode="371312" if regioncode=="371312" & year==2000

replace regioncode="371321" if regioncode=="371321" & year==2000

replace regioncode="371322" if regioncode=="371322" & year==2000

replace regioncode="371323" if regioncode=="371323" & year==2000

replace regioncode="371324" if regioncode=="371324" & year==2000

replace regioncode="371325" if regioncode=="371325" & year==2000

replace regioncode="371326" if regioncode=="371326" & year==2000

replace regioncode="371327" if regioncode=="371327" & year==2000

replace regioncode="371328" if regioncode=="371328" & year==2000

replace regioncode="371329" if regioncode=="371329" & year==2000

replace regioncode="371402" if regioncode=="371402" & year==2000

replace regioncode="371421" if regioncode=="371421" & year==2000

replace regioncode="371422" if regioncode=="371422" & year==2000

replace regioncode="371423" if regioncode=="371423" & year==2000

replace regioncode="371424" if regioncode=="371424" & year==2000

replace regioncode="371425" if regioncode=="371425" & year==2000

replace regioncode="371426" if regioncode=="371426" & year==2000

replace regioncode="371427" if regioncode=="371427" & year==2000

replace regioncode="371428" if regioncode=="371428" & year==2000

replace regioncode="371481" if regioncode=="371481" & year==2000

replace regioncode="371482" if regioncode=="371482" & year==2000

replace regioncode="371502" if regioncode=="371502" & year==2000

replace regioncode="371521" if regioncode=="371521" & year==2000

replace regioncode="371522" if regioncode=="371522" & year==2000

replace regioncode="371523" if regioncode=="371523" & year==2000

replace regioncode="371524" if regioncode=="371524" & year==2000

replace regioncode="371525" if regioncode=="371525" & year==2000

replace regioncode="371526" if regioncode=="371526" & year==2000

replace regioncode="371581" if regioncode=="371581" & year==2000

replace regioncode="371602" if regioncode=="372301" & year==2000

replace regioncode="371621" if regioncode=="372321" & year==2000

replace regioncode="371622" if regioncode=="372322" & year==2000

replace regioncode="371623" if regioncode=="372324" & year==2000

replace regioncode="371624" if regioncode=="372325" & year==2000

replace regioncode="371625" if regioncode=="372328" & year==2000

replace regioncode="371626" if regioncode=="372330" & year==2000

replace regioncode="371702" if regioncode=="371702" & year==2000

replace regioncode="371721" if regioncode=="371721" & year==2000

replace regioncode="371722" if regioncode=="371722" & year==2000

replace regioncode="371723" if regioncode=="371723" & year==2000

replace regioncode="371724" if regioncode=="371724" & year==2000

replace regioncode="371725" if regioncode=="371725" & year==2000

replace regioncode="371726" if regioncode=="371726" & year==2000

replace regioncode="371727" if regioncode=="371727" & year==2000

replace regioncode="371728" if regioncode=="371728" & year==2000

replace regioncode="410102" if regioncode=="410102" & year==2000

replace regioncode="410103" if regioncode=="410103" & year==2000

replace regioncode="410104" if regioncode=="410104" & year==2000

replace regioncode="410105" if regioncode=="410105" & year==2000

replace regioncode="410106" if regioncode=="410106" & year==2000

replace regioncode="410108" if regioncode=="410108" & year==2000

replace regioncode="410122" if regioncode=="410122" & year==2000

replace regioncode="410181" if regioncode=="410181" & year==2000

replace regioncode="410182" if regioncode=="410182" & year==2000

replace regioncode="410183" if regioncode=="410183" & year==2000

replace regioncode="410184" if regioncode=="410184" & year==2000

replace regioncode="410185" if regioncode=="410185" & year==2000

replace regioncode="410202" if regioncode=="410202" & year==2000

replace regioncode="410203" if regioncode=="410203" & year==2000

replace regioncode="410204" if regioncode=="410204" & year==2000

replace regioncode="410205" if regioncode=="410205" & year==2000

replace regioncode="410211" if regioncode=="410211" & year==2000

replace regioncode="410221" if regioncode=="410221" & year==2000

replace regioncode="410222" if regioncode=="410222" & year==2000

replace regioncode="410223" if regioncode=="410223" & year==2000

replace regioncode="410224" if regioncode=="410224" & year==2000

replace regioncode="410225" if regioncode=="410225" & year==2000

replace regioncode="410302" if regioncode=="410302" & year==2000

replace regioncode="410303" if regioncode=="410303" & year==2000

replace regioncode="410304" if regioncode=="410304" & year==2000

replace regioncode="410305" if regioncode=="410305" & year==2000

replace regioncode="410306" if regioncode=="410306" & year==2000

replace regioncode="410307" if regioncode=="410307" & year==2000

replace regioncode="410322" if regioncode=="410322" & year==2000

replace regioncode="410323" if regioncode=="410323" & year==2000

replace regioncode="410324" if regioncode=="410324" & year==2000

replace regioncode="410325" if regioncode=="410325" & year==2000

replace regioncode="410326" if regioncode=="410326" & year==2000

replace regioncode="410327" if regioncode=="410327" & year==2000

replace regioncode="410328" if regioncode=="410328" & year==2000

replace regioncode="410329" if regioncode=="410329" & year==2000

replace regioncode="410381" if regioncode=="410381" & year==2000

replace regioncode="410402" if regioncode=="410402" & year==2000

replace regioncode="410403" if regioncode=="410403" & year==2000

replace regioncode="410404" if regioncode=="410404" & year==2000

replace regioncode="410411" if regioncode=="410411" & year==2000

replace regioncode="410421" if regioncode=="410421" & year==2000

replace regioncode="410422" if regioncode=="410422" & year==2000

replace regioncode="410423" if regioncode=="410423" & year==2000

replace regioncode="410425" if regioncode=="410425" & year==2000

replace regioncode="410481" if regioncode=="410481" & year==2000

replace regioncode="410482" if regioncode=="410482" & year==2000

replace regioncode="410502" if regioncode=="410502" & year==2000

replace regioncode="410503" if regioncode=="410503" & year==2000

replace regioncode="410504" if regioncode=="410504" & year==2000

replace regioncode="410511" if regioncode=="410511" & year==2000

replace regioncode="410522" if regioncode=="410522" & year==2000

replace regioncode="410523" if regioncode=="410523" & year==2000

replace regioncode="410526" if regioncode=="410526" & year==2000

replace regioncode="410527" if regioncode=="410527" & year==2000

replace regioncode="410581" if regioncode=="410581" & year==2000

replace regioncode="410602" if regioncode=="410602" & year==2000

replace regioncode="410603" if regioncode=="410603" & year==2000

replace regioncode="410611" if regioncode=="410611" & year==2000

replace regioncode="410621" if regioncode=="410621" & year==2000

replace regioncode="410622" if regioncode=="410622" & year==2000

replace regioncode="410702" if regioncode=="410702" & year==2000

replace regioncode="410703" if regioncode=="410703" & year==2000

replace regioncode="410704" if regioncode=="410704" & year==2000

replace regioncode="410711" if regioncode=="410711" & year==2000

replace regioncode="410721" if regioncode=="410721" & year==2000

replace regioncode="410724" if regioncode=="410724" & year==2000

replace regioncode="410725" if regioncode=="410725" & year==2000

replace regioncode="410726" if regioncode=="410726" & year==2000

replace regioncode="410727" if regioncode=="410727" & year==2000

replace regioncode="410728" if regioncode=="410728" & year==2000

replace regioncode="410781" if regioncode=="410781" & year==2000

replace regioncode="410782" if regioncode=="410782" & year==2000

replace regioncode="410802" if regioncode=="410802" & year==2000

replace regioncode="410803" if regioncode=="410803" & year==2000

replace regioncode="410804" if regioncode=="410804" & year==2000

replace regioncode="410811" if regioncode=="410811" & year==2000

replace regioncode="410821" if regioncode=="410821" & year==2000

replace regioncode="410822" if regioncode=="410822" & year==2000

replace regioncode="410823" if regioncode=="410823" & year==2000

replace regioncode="410825" if regioncode=="410825" & year==2000

replace regioncode="410881" if regioncode=="410881" & year==2000

replace regioncode="410882" if regioncode=="410882" & year==2000

replace regioncode="410883" if regioncode=="410883" & year==2000

replace regioncode="410902" if regioncode=="410902" & year==2000

replace regioncode="410922" if regioncode=="410922" & year==2000

replace regioncode="410923" if regioncode=="410923" & year==2000

replace regioncode="410926" if regioncode=="410926" & year==2000

replace regioncode="410927" if regioncode=="410927" & year==2000

replace regioncode="410928" if regioncode=="410928" & year==2000

replace regioncode="411002" if regioncode=="411002" & year==2000

replace regioncode="411023" if regioncode=="411023" & year==2000

replace regioncode="411024" if regioncode=="411024" & year==2000

replace regioncode="411025" if regioncode=="411025" & year==2000

replace regioncode="411081" if regioncode=="411081" & year==2000

replace regioncode="411082" if regioncode=="411082" & year==2000

replace regioncode="411102" if regioncode=="411102" & year==2000

replace regioncode="411123" if regioncode=="411123" & year==2000

replace regioncode="411121" if regioncode=="411121" & year==2000

replace regioncode="411122" if regioncode=="411122" & year==2000

replace regioncode="411202" if regioncode=="411202" & year==2000

replace regioncode="411221" if regioncode=="411221" & year==2000

replace regioncode="411222" if regioncode=="411222" & year==2000

replace regioncode="411224" if regioncode=="411224" & year==2000

replace regioncode="411281" if regioncode=="411281" & year==2000

replace regioncode="411282" if regioncode=="411282" & year==2000

replace regioncode="411302" if regioncode=="411302" & year==2000

replace regioncode="411303" if regioncode=="411303" & year==2000

replace regioncode="411321" if regioncode=="411321" & year==2000

replace regioncode="411322" if regioncode=="411322" & year==2000

replace regioncode="411323" if regioncode=="411323" & year==2000

replace regioncode="411324" if regioncode=="411324" & year==2000

replace regioncode="411325" if regioncode=="411325" & year==2000

replace regioncode="411326" if regioncode=="411326" & year==2000

replace regioncode="411327" if regioncode=="411327" & year==2000

replace regioncode="411328" if regioncode=="411328" & year==2000

replace regioncode="411329" if regioncode=="411329" & year==2000

replace regioncode="411330" if regioncode=="411330" & year==2000

replace regioncode="411381" if regioncode=="411381" & year==2000

replace regioncode="411402" if regioncode=="411402" & year==2000

replace regioncode="411403" if regioncode=="411403" & year==2000

replace regioncode="411421" if regioncode=="411421" & year==2000

replace regioncode="411422" if regioncode=="411422" & year==2000

replace regioncode="411423" if regioncode=="411423" & year==2000

replace regioncode="411424" if regioncode=="411424" & year==2000

replace regioncode="411425" if regioncode=="411425" & year==2000

replace regioncode="411426" if regioncode=="411426" & year==2000

replace regioncode="411481" if regioncode=="411481" & year==2000

replace regioncode="411502" if regioncode=="411502" & year==2000

replace regioncode="411503" if regioncode=="411503" & year==2000

replace regioncode="411521" if regioncode=="411521" & year==2000

replace regioncode="411522" if regioncode=="411522" & year==2000

replace regioncode="411523" if regioncode=="411523" & year==2000

replace regioncode="411524" if regioncode=="411524" & year==2000

replace regioncode="411525" if regioncode=="411525" & year==2000

replace regioncode="411526" if regioncode=="411526" & year==2000

replace regioncode="411527" if regioncode=="411527" & year==2000

replace regioncode="411528" if regioncode=="411528" & year==2000

replace regioncode="411602" if regioncode=="411602" & year==2000

replace regioncode="411621" if regioncode=="411621" & year==2000

replace regioncode="411622" if regioncode=="411622" & year==2000

replace regioncode="411623" if regioncode=="411623" & year==2000

replace regioncode="411624" if regioncode=="411624" & year==2000

replace regioncode="411625" if regioncode=="411625" & year==2000

replace regioncode="411626" if regioncode=="411626" & year==2000

replace regioncode="411627" if regioncode=="411627" & year==2000

replace regioncode="411628" if regioncode=="411628" & year==2000

replace regioncode="411681" if regioncode=="411681" & year==2000

replace regioncode="411702" if regioncode=="411702" & year==2000

replace regioncode="411721" if regioncode=="411721" & year==2000

replace regioncode="411722" if regioncode=="411722" & year==2000

replace regioncode="411723" if regioncode=="411723" & year==2000

replace regioncode="411724" if regioncode=="411724" & year==2000

replace regioncode="411725" if regioncode=="411725" & year==2000

replace regioncode="411726" if regioncode=="411726" & year==2000

replace regioncode="411727" if regioncode=="411727" & year==2000

replace regioncode="411728" if regioncode=="411728" & year==2000

replace regioncode="411729" if regioncode=="411729" & year==2000

replace regioncode="420102" if regioncode=="420102" & year==2000

replace regioncode="420103" if regioncode=="420103" & year==2000

replace regioncode="420104" if regioncode=="420104" & year==2000

replace regioncode="420105" if regioncode=="420105" & year==2000

replace regioncode="420106" if regioncode=="420106" & year==2000

replace regioncode="420107" if regioncode=="420107" & year==2000

replace regioncode="420111" if regioncode=="420111" & year==2000

replace regioncode="420112" if regioncode=="420112" & year==2000

replace regioncode="420113" if regioncode=="420113" & year==2000

replace regioncode="420114" if regioncode=="420114" & year==2000

replace regioncode="420115" if regioncode=="420115" & year==2000

replace regioncode="420116" if regioncode=="420123" & year==2000

replace regioncode="420117" if regioncode=="420124" & year==2000

replace regioncode="420202" if regioncode=="420202" & year==2000

replace regioncode="420203" if regioncode=="420203" & year==2000

replace regioncode="420204" if regioncode=="420204" & year==2000

replace regioncode="420205" if regioncode=="420205" & year==2000

replace regioncode="420222" if regioncode=="420222" & year==2000

replace regioncode="420281" if regioncode=="420281" & year==2000

replace regioncode="420302" if regioncode=="420302" & year==2000

replace regioncode="420303" if regioncode=="420303" & year==2000

replace regioncode="420321" if regioncode=="420321" & year==2000

replace regioncode="420322" if regioncode=="420322" & year==2000

replace regioncode="420323" if regioncode=="420323" & year==2000

replace regioncode="420324" if regioncode=="420324" & year==2000

replace regioncode="420325" if regioncode=="420325" & year==2000

replace regioncode="420381" if regioncode=="420381" & year==2000

replace regioncode="420502" if regioncode=="420502" & year==2000

replace regioncode="420503" if regioncode=="420503" & year==2000

replace regioncode="420504" if regioncode=="420504" & year==2000

replace regioncode="420505" if regioncode=="420505" & year==2000

replace regioncode="420506" if regioncode=="420521" & year==2000

replace regioncode="420525" if regioncode=="420525" & year==2000

replace regioncode="420526" if regioncode=="420526" & year==2000

replace regioncode="420527" if regioncode=="420527" & year==2000

replace regioncode="420528" if regioncode=="420528" & year==2000

replace regioncode="420529" if regioncode=="420529" & year==2000

replace regioncode="420581" if regioncode=="420581" & year==2000

replace regioncode="420582" if regioncode=="420582" & year==2000

replace regioncode="420583" if regioncode=="420583" & year==2000

replace regioncode="420602" if regioncode=="420602" & year==2000

replace regioncode="420606" if regioncode=="420606" & year==2000

replace regioncode="420607" if regioncode=="420607" & year==2000

replace regioncode="420624" if regioncode=="420624" & year==2000

replace regioncode="420625" if regioncode=="420625" & year==2000

replace regioncode="420626" if regioncode=="420626" & year==2000

replace regioncode="420682" if regioncode=="420682" & year==2000

replace regioncode="420683" if regioncode=="420683" & year==2000

replace regioncode="420684" if regioncode=="420684" & year==2000

replace regioncode="420702" if regioncode=="420702" & year==2000

replace regioncode="420703" if regioncode=="420703" & year==2000

replace regioncode="420704" if regioncode=="420704" & year==2000

replace regioncode="420802" if regioncode=="420802" & year==2000

replace regioncode="420804" if regioncode=="420804" & year==2000

replace regioncode="420821" if regioncode=="420821" & year==2000

replace regioncode="420822" if regioncode=="420822" & year==2000

replace regioncode="420881" if regioncode=="420881" & year==2000

replace regioncode="420902" if regioncode=="420902" & year==2000

replace regioncode="420921" if regioncode=="420921" & year==2000

replace regioncode="420922" if regioncode=="420922" & year==2000

replace regioncode="420923" if regioncode=="420923" & year==2000

replace regioncode="420981" if regioncode=="420981" & year==2000

replace regioncode="420982" if regioncode=="420982" & year==2000

replace regioncode="421381" if regioncode=="421381" & year==2000

replace regioncode="420984" if regioncode=="420984" & year==2000

replace regioncode="421002" if regioncode=="421002" & year==2000

replace regioncode="421003" if regioncode=="421003" & year==2000

replace regioncode="421022" if regioncode=="421022" & year==2000

replace regioncode="421023" if regioncode=="421023" & year==2000

replace regioncode="421024" if regioncode=="421024" & year==2000

replace regioncode="421081" if regioncode=="421081" & year==2000

replace regioncode="421083" if regioncode=="421083" & year==2000

replace regioncode="421087" if regioncode=="421087" & year==2000

replace regioncode="421102" if regioncode=="421102" & year==2000

replace regioncode="421121" if regioncode=="421121" & year==2000

replace regioncode="421122" if regioncode=="421122" & year==2000

replace regioncode="421123" if regioncode=="421123" & year==2000

replace regioncode="421124" if regioncode=="421124" & year==2000

replace regioncode="421125" if regioncode=="421125" & year==2000

replace regioncode="421126" if regioncode=="421126" & year==2000

replace regioncode="421127" if regioncode=="421127" & year==2000

replace regioncode="421181" if regioncode=="421181" & year==2000

replace regioncode="421182" if regioncode=="421182" & year==2000

replace regioncode="421202" if regioncode=="421202" & year==2000

replace regioncode="421221" if regioncode=="421221" & year==2000

replace regioncode="421222" if regioncode=="421222" & year==2000

replace regioncode="421223" if regioncode=="421223" & year==2000

replace regioncode="421224" if regioncode=="421224" & year==2000

replace regioncode="421281" if regioncode=="421281" & year==2000

replace regioncode="421302" if regioncode=="421302" & year==2000

replace regioncode="422801" if regioncode=="422801" & year==2000

replace regioncode="422802" if regioncode=="422802" & year==2000

replace regioncode="422822" if regioncode=="422822" & year==2000

replace regioncode="422823" if regioncode=="422823" & year==2000

replace regioncode="422825" if regioncode=="422825" & year==2000

replace regioncode="422826" if regioncode=="422826" & year==2000

replace regioncode="422827" if regioncode=="422827" & year==2000

replace regioncode="422828" if regioncode=="422828" & year==2000

replace regioncode="429004" if regioncode=="429004" & year==2000

replace regioncode="429005" if regioncode=="429005" & year==2000

replace regioncode="429006" if regioncode=="429006" & year==2000

replace regioncode="429021" if regioncode=="429021" & year==2000

replace regioncode="430102" if regioncode=="430102" & year==2000

replace regioncode="430103" if regioncode=="430103" & year==2000

replace regioncode="430104" if regioncode=="430104" & year==2000

replace regioncode="430105" if regioncode=="430105" & year==2000

replace regioncode="430111" if regioncode=="430111" & year==2000

replace regioncode="430121" if regioncode=="430121" & year==2000

replace regioncode="430122" if regioncode=="430122" & year==2000

replace regioncode="430124" if regioncode=="430124" & year==2000

replace regioncode="430181" if regioncode=="430181" & year==2000

replace regioncode="430202" if regioncode=="430202" & year==2000

replace regioncode="430203" if regioncode=="430203" & year==2000

replace regioncode="430204" if regioncode=="430204" & year==2000

replace regioncode="430211" if regioncode=="430211" & year==2000

replace regioncode="430221" if regioncode=="430221" & year==2000

replace regioncode="430223" if regioncode=="430223" & year==2000

replace regioncode="430224" if regioncode=="430224" & year==2000

replace regioncode="430225" if regioncode=="430225" & year==2000

replace regioncode="430281" if regioncode=="430281" & year==2000

replace regioncode="430302" if regioncode=="430302" & year==2000

replace regioncode="430304" if regioncode=="430304" & year==2000

replace regioncode="430321" if regioncode=="430321" & year==2000

replace regioncode="430381" if regioncode=="430381" & year==2000

replace regioncode="430382" if regioncode=="430382" & year==2000

replace regioncode="430405" if regioncode=="430402" & year==2000

replace regioncode="430406" if regioncode=="430403" & year==2000

replace regioncode="430407" if regioncode=="430404" & year==2000

replace regioncode="430408" if regioncode=="430411" & year==2000

replace regioncode="430412" if regioncode=="430412" & year==2000

replace regioncode="430421" if regioncode=="430421" & year==2000

replace regioncode="430422" if regioncode=="430422" & year==2000

replace regioncode="430423" if regioncode=="430423" & year==2000

replace regioncode="430424" if regioncode=="430424" & year==2000

replace regioncode="430426" if regioncode=="430426" & year==2000

replace regioncode="430481" if regioncode=="430481" & year==2000

replace regioncode="430482" if regioncode=="430482" & year==2000

replace regioncode="430502" if regioncode=="430502" & year==2000

replace regioncode="430503" if regioncode=="430503" & year==2000

replace regioncode="430511" if regioncode=="430511" & year==2000

replace regioncode="430521" if regioncode=="430521" & year==2000

replace regioncode="430522" if regioncode=="430522" & year==2000

replace regioncode="430523" if regioncode=="430523" & year==2000

replace regioncode="430524" if regioncode=="430524" & year==2000

replace regioncode="430525" if regioncode=="430525" & year==2000

replace regioncode="430527" if regioncode=="430527" & year==2000

replace regioncode="430528" if regioncode=="430528" & year==2000

replace regioncode="430529" if regioncode=="430529" & year==2000

replace regioncode="430581" if regioncode=="430581" & year==2000

replace regioncode="430602" if regioncode=="430602" & year==2000

replace regioncode="430603" if regioncode=="430603" & year==2000

replace regioncode="430611" if regioncode=="430611" & year==2000

replace regioncode="430621" if regioncode=="430621" & year==2000

replace regioncode="430623" if regioncode=="430623" & year==2000

replace regioncode="430624" if regioncode=="430624" & year==2000

replace regioncode="430626" if regioncode=="430626" & year==2000

replace regioncode="430681" if regioncode=="430681" & year==2000

replace regioncode="430682" if regioncode=="430682" & year==2000

replace regioncode="430702" if regioncode=="430702" & year==2000

replace regioncode="430703" if regioncode=="430703" & year==2000

replace regioncode="430721" if regioncode=="430721" & year==2000

replace regioncode="430722" if regioncode=="430722" & year==2000

replace regioncode="430723" if regioncode=="430723" & year==2000

replace regioncode="430724" if regioncode=="430724" & year==2000

replace regioncode="430725" if regioncode=="430725" & year==2000

replace regioncode="430726" if regioncode=="430726" & year==2000

replace regioncode="430781" if regioncode=="430781" & year==2000

replace regioncode="430802" if regioncode=="430802" & year==2000

replace regioncode="430811" if regioncode=="430811" & year==2000

replace regioncode="430821" if regioncode=="430821" & year==2000

replace regioncode="430822" if regioncode=="430822" & year==2000

replace regioncode="430902" if regioncode=="430902" & year==2000

replace regioncode="430903" if regioncode=="430903" & year==2000

replace regioncode="430921" if regioncode=="430921" & year==2000

replace regioncode="430922" if regioncode=="430922" & year==2000

replace regioncode="430923" if regioncode=="430923" & year==2000

replace regioncode="430981" if regioncode=="430981" & year==2000

replace regioncode="431002" if regioncode=="431002" & year==2000

replace regioncode="431003" if regioncode=="431003" & year==2000

replace regioncode="431021" if regioncode=="431021" & year==2000

replace regioncode="431022" if regioncode=="431022" & year==2000

replace regioncode="431023" if regioncode=="431023" & year==2000

replace regioncode="431024" if regioncode=="431024" & year==2000

replace regioncode="431025" if regioncode=="431025" & year==2000

replace regioncode="431026" if regioncode=="431026" & year==2000

replace regioncode="431027" if regioncode=="431027" & year==2000

replace regioncode="431028" if regioncode=="431028" & year==2000

replace regioncode="431081" if regioncode=="431081" & year==2000

replace regioncode="431102" if regioncode=="431102" & year==2000

replace regioncode="431103" if regioncode=="431103" & year==2000

replace regioncode="431121" if regioncode=="431121" & year==2000

replace regioncode="431122" if regioncode=="431122" & year==2000

replace regioncode="431123" if regioncode=="431123" & year==2000

replace regioncode="431124" if regioncode=="431124" & year==2000

replace regioncode="431125" if regioncode=="431125" & year==2000

replace regioncode="431126" if regioncode=="431126" & year==2000

replace regioncode="431127" if regioncode=="431127" & year==2000

replace regioncode="431128" if regioncode=="431128" & year==2000

replace regioncode="431129" if regioncode=="431129" & year==2000

replace regioncode="431202" if regioncode=="431202" & year==2000

replace regioncode="431221" if regioncode=="431221" & year==2000

replace regioncode="431222" if regioncode=="431222" & year==2000

replace regioncode="431223" if regioncode=="431223" & year==2000

replace regioncode="431224" if regioncode=="431224" & year==2000

replace regioncode="431225" if regioncode=="431225" & year==2000

replace regioncode="431226" if regioncode=="431226" & year==2000

replace regioncode="431227" if regioncode=="431227" & year==2000

replace regioncode="431228" if regioncode=="431228" & year==2000

replace regioncode="431229" if regioncode=="431229" & year==2000

replace regioncode="431230" if regioncode=="431230" & year==2000

replace regioncode="431281" if regioncode=="431281" & year==2000

replace regioncode="431302" if regioncode=="431302" & year==2000

replace regioncode="431321" if regioncode=="431321" & year==2000

replace regioncode="431322" if regioncode=="431322" & year==2000

replace regioncode="431381" if regioncode=="431381" & year==2000

replace regioncode="431382" if regioncode=="431382" & year==2000

replace regioncode="433101" if regioncode=="433101" & year==2000

replace regioncode="433122" if regioncode=="433122" & year==2000

replace regioncode="433123" if regioncode=="433123" & year==2000

replace regioncode="433124" if regioncode=="433124" & year==2000

replace regioncode="433125" if regioncode=="433125" & year==2000

replace regioncode="433126" if regioncode=="433126" & year==2000

replace regioncode="433127" if regioncode=="433127" & year==2000

replace regioncode="433130" if regioncode=="433130" & year==2000

replace regioncode="440102" if regioncode=="440102" & year==2000

replace regioncode="440103" if regioncode=="440103" & year==2000

replace regioncode="440104" if regioncode=="440104" & year==2000

replace regioncode="440105" if regioncode=="440105" & year==2000

replace regioncode="440106" if regioncode=="440106" & year==2000

replace regioncode="440107" if regioncode=="440107" & year==2000

replace regioncode="440111" if regioncode=="440111" & year==2000

replace regioncode="440112" if regioncode=="440112" & year==2000

replace regioncode="440113" if regioncode=="440113" & year==2000

replace regioncode="440114" if regioncode=="440114" & year==2000

replace regioncode="440183" if regioncode=="440183" & year==2000

replace regioncode="440184" if regioncode=="440184" & year==2000

replace regioncode="440203" if regioncode=="440203" & year==2000

replace regioncode="440204" if regioncode=="440204" & year==2000

replace regioncode="440221" if regioncode=="440221" & year==2000

replace regioncode="440222" if regioncode=="440222" & year==2000

replace regioncode="440224" if regioncode=="440224" & year==2000

replace regioncode="440229" if regioncode=="440229" & year==2000

replace regioncode="440232" if regioncode=="440232" & year==2000

replace regioncode="440233" if regioncode=="440233" & year==2000

replace regioncode="440281" if regioncode=="440281" & year==2000

replace regioncode="440282" if regioncode=="440282" & year==2000

replace regioncode="440303" if regioncode=="440303" & year==2000

replace regioncode="440304" if regioncode=="440304" & year==2000

replace regioncode="440305" if regioncode=="440305" & year==2000

replace regioncode="440306" if regioncode=="440306" & year==2000

replace regioncode="440307" if regioncode=="440307" & year==2000

replace regioncode="440308" if regioncode=="440308" & year==2000

replace regioncode="440402" if regioncode=="440402" & year==2000

replace regioncode="440403" if regioncode=="440421" & year==2000

replace regioncode="440404" if regioncode=="440404" & year==2000

replace regioncode="440507" if regioncode=="440507" & year==2000

replace regioncode="440511" if regioncode=="440509" & year==2000

replace regioncode="440512" if regioncode=="440510" & year==2000

replace regioncode="440514" if regioncode=="440582" & year==2000

replace regioncode="440515" if regioncode=="440583" & year==2000

replace regioncode="440523" if regioncode=="440523" & year==2000

replace regioncode="440604" if regioncode=="440603" & year==2000

replace regioncode="440605" if regioncode=="440682" & year==2000

replace regioncode="440606" if regioncode=="440681" & year==2000

replace regioncode="440607" if regioncode=="440683" & year==2000

replace regioncode="440608" if regioncode=="440684" & year==2000

replace regioncode="440703" if regioncode=="440703" & year==2000

replace regioncode="440704" if regioncode=="440704" & year==2000

replace regioncode="440781" if regioncode=="440781" & year==2000

replace regioncode="440705" if regioncode=="440782" & year==2000

replace regioncode="440783" if regioncode=="440783" & year==2000

replace regioncode="440784" if regioncode=="440784" & year==2000

replace regioncode="440785" if regioncode=="440785" & year==2000

replace regioncode="440802" if regioncode=="440802" & year==2000

replace regioncode="440803" if regioncode=="440803" & year==2000

replace regioncode="440804" if regioncode=="440804" & year==2000

replace regioncode="440811" if regioncode=="440811" & year==2000

replace regioncode="440823" if regioncode=="440823" & year==2000

replace regioncode="440825" if regioncode=="440825" & year==2000

replace regioncode="440881" if regioncode=="440881" & year==2000

replace regioncode="440882" if regioncode=="440882" & year==2000

replace regioncode="440883" if regioncode=="440883" & year==2000

replace regioncode="440902" if regioncode=="440902" & year==2000

replace regioncode="440903" if regioncode=="440903" & year==2000

replace regioncode="440923" if regioncode=="440923" & year==2000

replace regioncode="440981" if regioncode=="440981" & year==2000

replace regioncode="440982" if regioncode=="440982" & year==2000

replace regioncode="440983" if regioncode=="440983" & year==2000

replace regioncode="441202" if regioncode=="441202" & year==2000

replace regioncode="441203" if regioncode=="441203" & year==2000

replace regioncode="441223" if regioncode=="441223" & year==2000

replace regioncode="441224" if regioncode=="441224" & year==2000

replace regioncode="441225" if regioncode=="441225" & year==2000

replace regioncode="441226" if regioncode=="441226" & year==2000

replace regioncode="441283" if regioncode=="441283" & year==2000

replace regioncode="441284" if regioncode=="441284" & year==2000

replace regioncode="441302" if regioncode=="441302" & year==2000

replace regioncode="441303" if regioncode=="441381" & year==2000

replace regioncode="441322" if regioncode=="441322" & year==2000

replace regioncode="441323" if regioncode=="441323" & year==2000

replace regioncode="441324" if regioncode=="441324" & year==2000

replace regioncode="441402" if regioncode=="441402" & year==2000

replace regioncode="441421" if regioncode=="441421" & year==2000

replace regioncode="441422" if regioncode=="441422" & year==2000

replace regioncode="441423" if regioncode=="441423" & year==2000

replace regioncode="441424" if regioncode=="441424" & year==2000

replace regioncode="441426" if regioncode=="441426" & year==2000

replace regioncode="441427" if regioncode=="441427" & year==2000

replace regioncode="441481" if regioncode=="441481" & year==2000

replace regioncode="441502" if regioncode=="441502" & year==2000

replace regioncode="441521" if regioncode=="441521" & year==2000

replace regioncode="441523" if regioncode=="441523" & year==2000

replace regioncode="441581" if regioncode=="441581" & year==2000

replace regioncode="441602" if regioncode=="441602" & year==2000

replace regioncode="441621" if regioncode=="441621" & year==2000

replace regioncode="441622" if regioncode=="441622" & year==2000

replace regioncode="441623" if regioncode=="441623" & year==2000

replace regioncode="441624" if regioncode=="441624" & year==2000

replace regioncode="441625" if regioncode=="441625" & year==2000

replace regioncode="441702" if regioncode=="441702" & year==2000

replace regioncode="441721" if regioncode=="441721" & year==2000

replace regioncode="441723" if regioncode=="441723" & year==2000

replace regioncode="441781" if regioncode=="441781" & year==2000

replace regioncode="441802" if regioncode=="441802" & year==2000

replace regioncode="441821" if regioncode=="441821" & year==2000

replace regioncode="441823" if regioncode=="441823" & year==2000

replace regioncode="441825" if regioncode=="441825" & year==2000

replace regioncode="441826" if regioncode=="441826" & year==2000

replace regioncode="441827" if regioncode=="441827" & year==2000

replace regioncode="441881" if regioncode=="441881" & year==2000

replace regioncode="441882" if regioncode=="441882" & year==2000

replace regioncode="441900" if regioncode=="441900" & year==2000

replace regioncode="442000" if regioncode=="442000" & year==2000

replace regioncode="445102" if regioncode=="445102" & year==2000

replace regioncode="445121" if regioncode=="445121" & year==2000

replace regioncode="445122" if regioncode=="445122" & year==2000

replace regioncode="445202" if regioncode=="445202" & year==2000

replace regioncode="445221" if regioncode=="445221" & year==2000

replace regioncode="445222" if regioncode=="445222" & year==2000

replace regioncode="445224" if regioncode=="445224" & year==2000

replace regioncode="445281" if regioncode=="445281" & year==2000

replace regioncode="445302" if regioncode=="445302" & year==2000

replace regioncode="445321" if regioncode=="445321" & year==2000

replace regioncode="445322" if regioncode=="445322" & year==2000

replace regioncode="445323" if regioncode=="445323" & year==2000

replace regioncode="445381" if regioncode=="445381" & year==2000

replace regioncode="450102" if regioncode=="450102" & year==2000

replace regioncode="450103" if regioncode=="450103" & year==2000

replace regioncode="450104" if regioncode=="450104" & year==2000

replace regioncode="450105" if regioncode=="450105" & year==2000

replace regioncode="450106" if regioncode=="450106" & year==2000

replace regioncode="450121" if regioncode=="450121" & year==2000

replace regioncode="450122" if regioncode=="450122" & year==2000

replace regioncode="450123" if regioncode=="452126" & year==2000

replace regioncode="450124" if regioncode=="452127" & year==2000

replace regioncode="450125" if regioncode=="452124" & year==2000

replace regioncode="450126" if regioncode=="452123" & year==2000

replace regioncode="450127" if regioncode=="452122" & year==2000

replace regioncode="450202" if regioncode=="450202" & year==2000

replace regioncode="450203" if regioncode=="450203" & year==2000

replace regioncode="450204" if regioncode=="450204" & year==2000

replace regioncode="450205" if regioncode=="450205" & year==2000

replace regioncode="450221" if regioncode=="450221" & year==2000

replace regioncode="450222" if regioncode=="450222" & year==2000

replace regioncode="450223" if regioncode=="452223" & year==2000

replace regioncode="450224" if regioncode=="452227" & year==2000

replace regioncode="450225" if regioncode=="452229" & year==2000

replace regioncode="450226" if regioncode=="452228" & year==2000

replace regioncode="450302" if regioncode=="450302" & year==2000

replace regioncode="450303" if regioncode=="450303" & year==2000

replace regioncode="450304" if regioncode=="450304" & year==2000

replace regioncode="450305" if regioncode=="450305" & year==2000

replace regioncode="450311" if regioncode=="450311" & year==2000

replace regioncode="450321" if regioncode=="450321" & year==2000

replace regioncode="450322" if regioncode=="450322" & year==2000

replace regioncode="450323" if regioncode=="450323" & year==2000

replace regioncode="450324" if regioncode=="450324" & year==2000

replace regioncode="450325" if regioncode=="450325" & year==2000

replace regioncode="450326" if regioncode=="450326" & year==2000

replace regioncode="450327" if regioncode=="450327" & year==2000

replace regioncode="450328" if regioncode=="450328" & year==2000

replace regioncode="450329" if regioncode=="450329" & year==2000

replace regioncode="450330" if regioncode=="450330" & year==2000

replace regioncode="450331" if regioncode=="450331" & year==2000

replace regioncode="450332" if regioncode=="450332" & year==2000

replace regioncode="450403" if regioncode=="450403" & year==2000

replace regioncode="450404" if regioncode=="450404" & year==2000

replace regioncode="450421" if regioncode=="450421" & year==2000

replace regioncode="450422" if regioncode=="450422" & year==2000

replace regioncode="450423" if regioncode=="450423" & year==2000

replace regioncode="450481" if regioncode=="450481" & year==2000

replace regioncode="450502" if regioncode=="450502" & year==2000

replace regioncode="450503" if regioncode=="450503" & year==2000

replace regioncode="450512" if regioncode=="450504" & year==2000

replace regioncode="450521" if regioncode=="450521" & year==2000

replace regioncode="450602" if regioncode=="450602" & year==2000

replace regioncode="450603" if regioncode=="450603" & year==2000

replace regioncode="450621" if regioncode=="450621" & year==2000

replace regioncode="450681" if regioncode=="450681" & year==2000

replace regioncode="450702" if regioncode=="450702" & year==2000

replace regioncode="450703" if regioncode=="450703" & year==2000

replace regioncode="450721" if regioncode=="450721" & year==2000

replace regioncode="450722" if regioncode=="450722" & year==2000

replace regioncode="450802" if regioncode=="450802" & year==2000

replace regioncode="450803" if regioncode=="450803" & year==2000

replace regioncode="450804" if regioncode=="450804" & year==2000

replace regioncode="450821" if regioncode=="450821" & year==2000

replace regioncode="450881" if regioncode=="450881" & year==2000

replace regioncode="450902" if regioncode=="450902" & year==2000

replace regioncode="450921" if regioncode=="450921" & year==2000

replace regioncode="450922" if regioncode=="450922" & year==2000

replace regioncode="450923" if regioncode=="450923" & year==2000

replace regioncode="450924" if regioncode=="450924" & year==2000

replace regioncode="450981" if regioncode=="450981" & year==2000

replace regioncode="451002" if regioncode=="452601" & year==2000

replace regioncode="451021" if regioncode=="452622" & year==2000

replace regioncode="451022" if regioncode=="452623" & year==2000

replace regioncode="451023" if regioncode=="452624" & year==2000

replace regioncode="451024" if regioncode=="452625" & year==2000

replace regioncode="451025" if regioncode=="452626" & year==2000

replace regioncode="451026" if regioncode=="452627" & year==2000

replace regioncode="451027" if regioncode=="452628" & year==2000

replace regioncode="451028" if regioncode=="452629" & year==2000

replace regioncode="451029" if regioncode=="452630" & year==2000

replace regioncode="451030" if regioncode=="452632" & year==2000

replace regioncode="451031" if regioncode=="452631" & year==2000

replace regioncode="451102" if regioncode=="452402" & year==2000

replace regioncode="451121" if regioncode=="452424" & year==2000

replace regioncode="451122" if regioncode=="452427" & year==2000

replace regioncode="451123" if regioncode=="452428" & year==2000

replace regioncode="451202" if regioncode=="452701" & year==2000

replace regioncode="451221" if regioncode=="452725" & year==2000

replace regioncode="451222" if regioncode=="452726" & year==2000

replace regioncode="451223" if regioncode=="452727" & year==2000

replace regioncode="451224" if regioncode=="452728" & year==2000

replace regioncode="451225" if regioncode=="452723" & year==2000

replace regioncode="451226" if regioncode=="452724" & year==2000

replace regioncode="451227" if regioncode=="452729" & year==2000

replace regioncode="451228" if regioncode=="452730" & year==2000

replace regioncode="451229" if regioncode=="452731" & year==2000

replace regioncode="451281" if regioncode=="452702" & year==2000

replace regioncode="451302" if regioncode=="452226" & year==2000

replace regioncode="451321" if regioncode=="452231" & year==2000

replace regioncode="451322" if regioncode=="452224" & year==2000

replace regioncode="451323" if regioncode=="452225" & year==2000

replace regioncode="451324" if regioncode=="452230" & year==2000

replace regioncode="451381" if regioncode=="452201" & year==2000

replace regioncode="451402" if regioncode=="452129" & year==2000

replace regioncode="451421" if regioncode=="452128" & year==2000

replace regioncode="451422" if regioncode=="452132" & year==2000

replace regioncode="451423" if regioncode=="452133" & year==2000

replace regioncode="451424" if regioncode=="452130" & year==2000

replace regioncode="451425" if regioncode=="452131" & year==2000

replace regioncode="451481" if regioncode=="452101" & year==2000

replace regioncode="460105" if regioncode=="460102" & year==2000

replace regioncode="460106" if regioncode=="460103" & year==2000

replace regioncode="460107" if regioncode=="460104" & year==2000

replace regioncode="460108" if regioncode=="469004" & year==2000

replace regioncode="460001" if regioncode=="460001" & year==2000

replace regioncode="460002" if regioncode=="460002" & year==2000

replace regioncode="460003" if regioncode=="460003" & year==2000

replace regioncode="460005" if regioncode=="460005" & year==2000

replace regioncode="460006" if regioncode=="460006" & year==2000

replace regioncode="460007" if regioncode=="460007" & year==2000

replace regioncode="460030" if regioncode=="460030" & year==2000

replace regioncode="460031" if regioncode=="460031" & year==2000

replace regioncode="460033" if regioncode=="460033" & year==2000

replace regioncode="460034" if regioncode=="460034" & year==2000

replace regioncode="460036" if regioncode=="460036" & year==2000

replace regioncode="500101" if regioncode=="500101" & year==2000

replace regioncode="500102" if regioncode=="500102" & year==2000

replace regioncode="500103" if regioncode=="500103" & year==2000

replace regioncode="500104" if regioncode=="500104" & year==2000

replace regioncode="500105" if regioncode=="500105" & year==2000

replace regioncode="500106" if regioncode=="500106" & year==2000

replace regioncode="500107" if regioncode=="500107" & year==2000

replace regioncode="500108" if regioncode=="500108" & year==2000

replace regioncode="500109" if regioncode=="500109" & year==2000

replace regioncode="500110" if regioncode=="500110" & year==2000

replace regioncode="500111" if regioncode=="500111" & year==2000

replace regioncode="500112" if regioncode=="500112" & year==2000

replace regioncode="500113" if regioncode=="500113" & year==2000

replace regioncode="500114" if regioncode=="500114" & year==2000

replace regioncode="500115" if regioncode=="500221" & year==2000

replace regioncode="500381" if regioncode=="500381" & year==2000

replace regioncode="500382" if regioncode=="500382" & year==2000

replace regioncode="500383" if regioncode=="500383" & year==2000

replace regioncode="500384" if regioncode=="500384" & year==2000

replace regioncode="500222" if regioncode=="500222" & year==2000

replace regioncode="500223" if regioncode=="500223" & year==2000

replace regioncode="500224" if regioncode=="500224" & year==2000

replace regioncode="500225" if regioncode=="500225" & year==2000

replace regioncode="500226" if regioncode=="500226" & year==2000

replace regioncode="500227" if regioncode=="500227" & year==2000

replace regioncode="500228" if regioncode=="500228" & year==2000

replace regioncode="500229" if regioncode=="500229" & year==2000

replace regioncode="500230" if regioncode=="500230" & year==2000

replace regioncode="500231" if regioncode=="500231" & year==2000

replace regioncode="500232" if regioncode=="500232" & year==2000

replace regioncode="500233" if regioncode=="500233" & year==2000

replace regioncode="500234" if regioncode=="500234" & year==2000

replace regioncode="500235" if regioncode=="500235" & year==2000

replace regioncode="500236" if regioncode=="500236" & year==2000

replace regioncode="500237" if regioncode=="500237" & year==2000

replace regioncode="500238" if regioncode=="500238" & year==2000

replace regioncode="500240" if regioncode=="500240" & year==2000

replace regioncode="500241" if regioncode=="500241" & year==2000

replace regioncode="500242" if regioncode=="500242" & year==2000

replace regioncode="500243" if regioncode=="500243" & year==2000

replace regioncode="510104" if regioncode=="510104" & year==2000

replace regioncode="510105" if regioncode=="510105" & year==2000

replace regioncode="510106" if regioncode=="510106" & year==2000

replace regioncode="510107" if regioncode=="510107" & year==2000

replace regioncode="510108" if regioncode=="510108" & year==2000

replace regioncode="510112" if regioncode=="510112" & year==2000

replace regioncode="510113" if regioncode=="510113" & year==2000

replace regioncode="510114" if regioncode=="510125" & year==2000

replace regioncode="510115" if regioncode=="510123" & year==2000

replace regioncode="510121" if regioncode=="510121" & year==2000

replace regioncode="510122" if regioncode=="510122" & year==2000

replace regioncode="510124" if regioncode=="510124" & year==2000

replace regioncode="510129" if regioncode=="510129" & year==2000

replace regioncode="510131" if regioncode=="510131" & year==2000

replace regioncode="510132" if regioncode=="510132" & year==2000

replace regioncode="510181" if regioncode=="510181" & year==2000

replace regioncode="510182" if regioncode=="510182" & year==2000

replace regioncode="510183" if regioncode=="510183" & year==2000

replace regioncode="510184" if regioncode=="510184" & year==2000

replace regioncode="510302" if regioncode=="510302" & year==2000

replace regioncode="510303" if regioncode=="510303" & year==2000

replace regioncode="510304" if regioncode=="510304" & year==2000

replace regioncode="510311" if regioncode=="510311" & year==2000

replace regioncode="510321" if regioncode=="510321" & year==2000

replace regioncode="510322" if regioncode=="510322" & year==2000

replace regioncode="510402" if regioncode=="510402" & year==2000

replace regioncode="510403" if regioncode=="510403" & year==2000

replace regioncode="510411" if regioncode=="510411" & year==2000

replace regioncode="510421" if regioncode=="510421" & year==2000

replace regioncode="510422" if regioncode=="510422" & year==2000

replace regioncode="510502" if regioncode=="510502" & year==2000

replace regioncode="510503" if regioncode=="510503" & year==2000

replace regioncode="510504" if regioncode=="510504" & year==2000

replace regioncode="510521" if regioncode=="510521" & year==2000

replace regioncode="510522" if regioncode=="510522" & year==2000

replace regioncode="510524" if regioncode=="510524" & year==2000

replace regioncode="510525" if regioncode=="510525" & year==2000

replace regioncode="510603" if regioncode=="510603" & year==2000

replace regioncode="510623" if regioncode=="510623" & year==2000

replace regioncode="510626" if regioncode=="510626" & year==2000

replace regioncode="510681" if regioncode=="510681" & year==2000

replace regioncode="510682" if regioncode=="510682" & year==2000

replace regioncode="510683" if regioncode=="510683" & year==2000

replace regioncode="510703" if regioncode=="510703" & year==2000

replace regioncode="510704" if regioncode=="510704" & year==2000

replace regioncode="510722" if regioncode=="510722" & year==2000

replace regioncode="510723" if regioncode=="510723" & year==2000

replace regioncode="510724" if regioncode=="510724" & year==2000

replace regioncode="510725" if regioncode=="510725" & year==2000

replace regioncode="510726" if regioncode=="510726" & year==2000

replace regioncode="510727" if regioncode=="510727" & year==2000

replace regioncode="510781" if regioncode=="510781" & year==2000

replace regioncode="510802" if regioncode=="510802" & year==2000

replace regioncode="510811" if regioncode=="510811" & year==2000

replace regioncode="510812" if regioncode=="510812" & year==2000

replace regioncode="510821" if regioncode=="510821" & year==2000

replace regioncode="510822" if regioncode=="510822" & year==2000

replace regioncode="510823" if regioncode=="510823" & year==2000

replace regioncode="510824" if regioncode=="510824" & year==2000

replace regioncode="510902" if regioncode=="510902" & year==2000

replace regioncode="510921" if regioncode=="510921" & year==2000

replace regioncode="510922" if regioncode=="510922" & year==2000

replace regioncode="510923" if regioncode=="510923" & year==2000

replace regioncode="511002" if regioncode=="511002" & year==2000

replace regioncode="511011" if regioncode=="511011" & year==2000

replace regioncode="511024" if regioncode=="511024" & year==2000

replace regioncode="511025" if regioncode=="511025" & year==2000

replace regioncode="511028" if regioncode=="511028" & year==2000

replace regioncode="511102" if regioncode=="511102" & year==2000

replace regioncode="511111" if regioncode=="511111" & year==2000

replace regioncode="511112" if regioncode=="511112" & year==2000

replace regioncode="511113" if regioncode=="511113" & year==2000

replace regioncode="511123" if regioncode=="511123" & year==2000

replace regioncode="511124" if regioncode=="511124" & year==2000

replace regioncode="511126" if regioncode=="511126" & year==2000

replace regioncode="511129" if regioncode=="511129" & year==2000

replace regioncode="511132" if regioncode=="511132" & year==2000

replace regioncode="511133" if regioncode=="511133" & year==2000

replace regioncode="511181" if regioncode=="511181" & year==2000

replace regioncode="511302" if regioncode=="511302" & year==2000

replace regioncode="511303" if regioncode=="511303" & year==2000

replace regioncode="511304" if regioncode=="511304" & year==2000

replace regioncode="511321" if regioncode=="511321" & year==2000

replace regioncode="511322" if regioncode=="511322" & year==2000

replace regioncode="511323" if regioncode=="511323" & year==2000

replace regioncode="511324" if regioncode=="511324" & year==2000

replace regioncode="511325" if regioncode=="511325" & year==2000

replace regioncode="511381" if regioncode=="511381" & year==2000

replace regioncode="511402" if regioncode=="511402" & year==2000

replace regioncode="511421" if regioncode=="511421" & year==2000

replace regioncode="511422" if regioncode=="511422" & year==2000

replace regioncode="511423" if regioncode=="511423" & year==2000

replace regioncode="511424" if regioncode=="511424" & year==2000

replace regioncode="511425" if regioncode=="511425" & year==2000

replace regioncode="511502" if regioncode=="511502" & year==2000

replace regioncode="511521" if regioncode=="511521" & year==2000

replace regioncode="511522" if regioncode=="511522" & year==2000

replace regioncode="511523" if regioncode=="511523" & year==2000

replace regioncode="511524" if regioncode=="511524" & year==2000

replace regioncode="511525" if regioncode=="511525" & year==2000

replace regioncode="511526" if regioncode=="511526" & year==2000

replace regioncode="511527" if regioncode=="511527" & year==2000

replace regioncode="511528" if regioncode=="511528" & year==2000

replace regioncode="511529" if regioncode=="511529" & year==2000

replace regioncode="511602" if regioncode=="511602" & year==2000

replace regioncode="511621" if regioncode=="511621" & year==2000

replace regioncode="511622" if regioncode=="511622" & year==2000

replace regioncode="511623" if regioncode=="511623" & year==2000

replace regioncode="511681" if regioncode=="511681" & year==2000

replace regioncode="511702" if regioncode=="511702" & year==2000

replace regioncode="511721" if regioncode=="511721" & year==2000

replace regioncode="511722" if regioncode=="511722" & year==2000

replace regioncode="511723" if regioncode=="511723" & year==2000

replace regioncode="511724" if regioncode=="511724" & year==2000

replace regioncode="511725" if regioncode=="511725" & year==2000

replace regioncode="511781" if regioncode=="511781" & year==2000

replace regioncode="511802" if regioncode=="511802" & year==2000

replace regioncode="511821" if regioncode=="511821" & year==2000

replace regioncode="511822" if regioncode=="511822" & year==2000

replace regioncode="511823" if regioncode=="511823" & year==2000

replace regioncode="511824" if regioncode=="511824" & year==2000

replace regioncode="511825" if regioncode=="511825" & year==2000

replace regioncode="511826" if regioncode=="511826" & year==2000

replace regioncode="511827" if regioncode=="511827" & year==2000

replace regioncode="511902" if regioncode=="511902" & year==2000

replace regioncode="511921" if regioncode=="511921" & year==2000

replace regioncode="511922" if regioncode=="511922" & year==2000

replace regioncode="511923" if regioncode=="511923" & year==2000

replace regioncode="512002" if regioncode=="512002" & year==2000

replace regioncode="512021" if regioncode=="512021" & year==2000

replace regioncode="512022" if regioncode=="512022" & year==2000

replace regioncode="512081" if regioncode=="512081" & year==2000

replace regioncode="513221" if regioncode=="513221" & year==2000

replace regioncode="513222" if regioncode=="513222" & year==2000

replace regioncode="513223" if regioncode=="513223" & year==2000

replace regioncode="513224" if regioncode=="513224" & year==2000

replace regioncode="513225" if regioncode=="513225" & year==2000

replace regioncode="513226" if regioncode=="513226" & year==2000

replace regioncode="513227" if regioncode=="513227" & year==2000

replace regioncode="513228" if regioncode=="513228" & year==2000

replace regioncode="513229" if regioncode=="513229" & year==2000

replace regioncode="513230" if regioncode=="513230" & year==2000

replace regioncode="513231" if regioncode=="513231" & year==2000

replace regioncode="513232" if regioncode=="513232" & year==2000

replace regioncode="513233" if regioncode=="513233" & year==2000

replace regioncode="513321" if regioncode=="513321" & year==2000

replace regioncode="513322" if regioncode=="513322" & year==2000

replace regioncode="513323" if regioncode=="513323" & year==2000

replace regioncode="513324" if regioncode=="513324" & year==2000

replace regioncode="513325" if regioncode=="513325" & year==2000

replace regioncode="513326" if regioncode=="513326" & year==2000

replace regioncode="513327" if regioncode=="513327" & year==2000

replace regioncode="513328" if regioncode=="513328" & year==2000

replace regioncode="513329" if regioncode=="513329" & year==2000

replace regioncode="513330" if regioncode=="513330" & year==2000

replace regioncode="513331" if regioncode=="513331" & year==2000

replace regioncode="513332" if regioncode=="513332" & year==2000

replace regioncode="513333" if regioncode=="513333" & year==2000

replace regioncode="513334" if regioncode=="513334" & year==2000

replace regioncode="513335" if regioncode=="513335" & year==2000

replace regioncode="513336" if regioncode=="513336" & year==2000

replace regioncode="513337" if regioncode=="513337" & year==2000

replace regioncode="513338" if regioncode=="513338" & year==2000

replace regioncode="513401" if regioncode=="513401" & year==2000

replace regioncode="513422" if regioncode=="513422" & year==2000

replace regioncode="513423" if regioncode=="513423" & year==2000

replace regioncode="513424" if regioncode=="513424" & year==2000

replace regioncode="513425" if regioncode=="513425" & year==2000

replace regioncode="513426" if regioncode=="513426" & year==2000

replace regioncode="513427" if regioncode=="513427" & year==2000

replace regioncode="513428" if regioncode=="513428" & year==2000

replace regioncode="513429" if regioncode=="513429" & year==2000

replace regioncode="513430" if regioncode=="513430" & year==2000

replace regioncode="513431" if regioncode=="513431" & year==2000

replace regioncode="513432" if regioncode=="513432" & year==2000

replace regioncode="513433" if regioncode=="513433" & year==2000

replace regioncode="513434" if regioncode=="513434" & year==2000

replace regioncode="513435" if regioncode=="513435" & year==2000

replace regioncode="513436" if regioncode=="513436" & year==2000

replace regioncode="513437" if regioncode=="513437" & year==2000

replace regioncode="520102" if regioncode=="520102" & year==2000

replace regioncode="520103" if regioncode=="520103" & year==2000

replace regioncode="520111" if regioncode=="520111" & year==2000

replace regioncode="520112" if regioncode=="520112" & year==2000

replace regioncode="520113" if regioncode=="520113" & year==2000

replace regioncode="520114" if regioncode=="520114" & year==2000

replace regioncode="520121" if regioncode=="520121" & year==2000

replace regioncode="520122" if regioncode=="520122" & year==2000

replace regioncode="520123" if regioncode=="520123" & year==2000

replace regioncode="520181" if regioncode=="520181" & year==2000

replace regioncode="520201" if regioncode=="520201" & year==2000

replace regioncode="520222" if regioncode=="520222" & year==2000

replace regioncode="520203" if regioncode=="520203" & year==2000

replace regioncode="520221" if regioncode=="520221" & year==2000

replace regioncode="520302" if regioncode=="520302" & year==2000

replace regioncode="520321" if regioncode=="520321" & year==2000

replace regioncode="520322" if regioncode=="520322" & year==2000

replace regioncode="520323" if regioncode=="520323" & year==2000

replace regioncode="520324" if regioncode=="520324" & year==2000

replace regioncode="520325" if regioncode=="520325" & year==2000

replace regioncode="520326" if regioncode=="520326" & year==2000

replace regioncode="520327" if regioncode=="520327" & year==2000

replace regioncode="520328" if regioncode=="520328" & year==2000

replace regioncode="520329" if regioncode=="520329" & year==2000

replace regioncode="520330" if regioncode=="520330" & year==2000

replace regioncode="520381" if regioncode=="520381" & year==2000

replace regioncode="520382" if regioncode=="520382" & year==2000

replace regioncode="520402" if regioncode=="520402" & year==2000

replace regioncode="520421" if regioncode=="520421" & year==2000

replace regioncode="520422" if regioncode=="520422" & year==2000

replace regioncode="520423" if regioncode=="520423" & year==2000

replace regioncode="520424" if regioncode=="520424" & year==2000

replace regioncode="520425" if regioncode=="520425" & year==2000

replace regioncode="522201" if regioncode=="522201" & year==2000

replace regioncode="522222" if regioncode=="522222" & year==2000

replace regioncode="522223" if regioncode=="522223" & year==2000

replace regioncode="522224" if regioncode=="522224" & year==2000

replace regioncode="522225" if regioncode=="522225" & year==2000

replace regioncode="522226" if regioncode=="522226" & year==2000

replace regioncode="522227" if regioncode=="522227" & year==2000

replace regioncode="522228" if regioncode=="522228" & year==2000

replace regioncode="522229" if regioncode=="522229" & year==2000

replace regioncode="522230" if regioncode=="522230" & year==2000

replace regioncode="522301" if regioncode=="522301" & year==2000

replace regioncode="522322" if regioncode=="522322" & year==2000

replace regioncode="522323" if regioncode=="522323" & year==2000

replace regioncode="522324" if regioncode=="522324" & year==2000

replace regioncode="522325" if regioncode=="522325" & year==2000

replace regioncode="522326" if regioncode=="522326" & year==2000

replace regioncode="522327" if regioncode=="522327" & year==2000

replace regioncode="522328" if regioncode=="522328" & year==2000

replace regioncode="522401" if regioncode=="522401" & year==2000

replace regioncode="522422" if regioncode=="522422" & year==2000

replace regioncode="522423" if regioncode=="522423" & year==2000

replace regioncode="522424" if regioncode=="522424" & year==2000

replace regioncode="522425" if regioncode=="522425" & year==2000

replace regioncode="522426" if regioncode=="522426" & year==2000

replace regioncode="522427" if regioncode=="522427" & year==2000

replace regioncode="522428" if regioncode=="522428" & year==2000

replace regioncode="522601" if regioncode=="522601" & year==2000

replace regioncode="522622" if regioncode=="522622" & year==2000

replace regioncode="522623" if regioncode=="522623" & year==2000

replace regioncode="522624" if regioncode=="522624" & year==2000

replace regioncode="522625" if regioncode=="522625" & year==2000

replace regioncode="522626" if regioncode=="522626" & year==2000

replace regioncode="522627" if regioncode=="522627" & year==2000

replace regioncode="522628" if regioncode=="522628" & year==2000

replace regioncode="522629" if regioncode=="522629" & year==2000

replace regioncode="522630" if regioncode=="522630" & year==2000

replace regioncode="522631" if regioncode=="522631" & year==2000

replace regioncode="522632" if regioncode=="522632" & year==2000

replace regioncode="522633" if regioncode=="522633" & year==2000

replace regioncode="522634" if regioncode=="522634" & year==2000

replace regioncode="522635" if regioncode=="522635" & year==2000

replace regioncode="522636" if regioncode=="522636" & year==2000

replace regioncode="522701" if regioncode=="522701" & year==2000

replace regioncode="522702" if regioncode=="522702" & year==2000

replace regioncode="522722" if regioncode=="522722" & year==2000

replace regioncode="522723" if regioncode=="522723" & year==2000

replace regioncode="522725" if regioncode=="522725" & year==2000

replace regioncode="522726" if regioncode=="522726" & year==2000

replace regioncode="522727" if regioncode=="522727" & year==2000

replace regioncode="522728" if regioncode=="522728" & year==2000

replace regioncode="522729" if regioncode=="522729" & year==2000

replace regioncode="522730" if regioncode=="522730" & year==2000

replace regioncode="522731" if regioncode=="522731" & year==2000

replace regioncode="522732" if regioncode=="522732" & year==2000

replace regioncode="530102" if regioncode=="530102" & year==2000

replace regioncode="530103" if regioncode=="530103" & year==2000

replace regioncode="530111" if regioncode=="530111" & year==2000

replace regioncode="530112" if regioncode=="530112" & year==2000

replace regioncode="530113" if regioncode=="530113" & year==2000

replace regioncode="530121" if regioncode=="530121" & year==2000

replace regioncode="530122" if regioncode=="530122" & year==2000

replace regioncode="530124" if regioncode=="530124" & year==2000

replace regioncode="530125" if regioncode=="530125" & year==2000

replace regioncode="530126" if regioncode=="530126" & year==2000

replace regioncode="530127" if regioncode=="530127" & year==2000

replace regioncode="530128" if regioncode=="530128" & year==2000

replace regioncode="530129" if regioncode=="530129" & year==2000

replace regioncode="530181" if regioncode=="530181" & year==2000

replace regioncode="530302" if regioncode=="530302" & year==2000

replace regioncode="530321" if regioncode=="530321" & year==2000

replace regioncode="530322" if regioncode=="530322" & year==2000

replace regioncode="530323" if regioncode=="530323" & year==2000

replace regioncode="530324" if regioncode=="530324" & year==2000

replace regioncode="530325" if regioncode=="530325" & year==2000

replace regioncode="530326" if regioncode=="530326" & year==2000

replace regioncode="530328" if regioncode=="530328" & year==2000

replace regioncode="530381" if regioncode=="530381" & year==2000

replace regioncode="530402" if regioncode=="530402" & year==2000

replace regioncode="530421" if regioncode=="530421" & year==2000

replace regioncode="530422" if regioncode=="530422" & year==2000

replace regioncode="530423" if regioncode=="530423" & year==2000

replace regioncode="530424" if regioncode=="530424" & year==2000

replace regioncode="530425" if regioncode=="530425" & year==2000

replace regioncode="530426" if regioncode=="530426" & year==2000

replace regioncode="530427" if regioncode=="530427" & year==2000

replace regioncode="530428" if regioncode=="530428" & year==2000

replace regioncode="530502" if regioncode=="533001" & year==2000

replace regioncode="530521" if regioncode=="533022" & year==2000

replace regioncode="530522" if regioncode=="533023" & year==2000

replace regioncode="530523" if regioncode=="533024" & year==2000

replace regioncode="530524" if regioncode=="533025" & year==2000

replace regioncode="530602" if regioncode=="532101" & year==2000

replace regioncode="530621" if regioncode=="532122" & year==2000

replace regioncode="530622" if regioncode=="532123" & year==2000

replace regioncode="530623" if regioncode=="532124" & year==2000

replace regioncode="530624" if regioncode=="532125" & year==2000

replace regioncode="530625" if regioncode=="532126" & year==2000

replace regioncode="530626" if regioncode=="532127" & year==2000

replace regioncode="530627" if regioncode=="532128" & year==2000

replace regioncode="530628" if regioncode=="532129" & year==2000

replace regioncode="530629" if regioncode=="532130" & year==2000

replace regioncode="530630" if regioncode=="532131" & year==2000

replace regioncode="530721" if regioncode=="533221" & year==2000

replace regioncode="530722" if regioncode=="533222" & year==2000

replace regioncode="530723" if regioncode=="533223" & year==2000

replace regioncode="530724" if regioncode=="533224" & year==2000

replace regioncode="532701" if regioncode=="532701" & year==2000

replace regioncode="532722" if regioncode=="532722" & year==2000

replace regioncode="532723" if regioncode=="532723" & year==2000

replace regioncode="532724" if regioncode=="532724" & year==2000

replace regioncode="532725" if regioncode=="532725" & year==2000

replace regioncode="532726" if regioncode=="532726" & year==2000

replace regioncode="532727" if regioncode=="532727" & year==2000

replace regioncode="532728" if regioncode=="532728" & year==2000

replace regioncode="532729" if regioncode=="532729" & year==2000

replace regioncode="532730" if regioncode=="532730" & year==2000

replace regioncode="533521" if regioncode=="533521" & year==2000

replace regioncode="533522" if regioncode=="533522" & year==2000

replace regioncode="533523" if regioncode=="533523" & year==2000

replace regioncode="533524" if regioncode=="533524" & year==2000

replace regioncode="533525" if regioncode=="533525" & year==2000

replace regioncode="533526" if regioncode=="533526" & year==2000

replace regioncode="533527" if regioncode=="533527" & year==2000

replace regioncode="533528" if regioncode=="533528" & year==2000

replace regioncode="532301" if regioncode=="532301" & year==2000

replace regioncode="532322" if regioncode=="532322" & year==2000

replace regioncode="532323" if regioncode=="532323" & year==2000

replace regioncode="532324" if regioncode=="532324" & year==2000

replace regioncode="532325" if regioncode=="532325" & year==2000

replace regioncode="532326" if regioncode=="532326" & year==2000

replace regioncode="532327" if regioncode=="532327" & year==2000

replace regioncode="532328" if regioncode=="532328" & year==2000

replace regioncode="532329" if regioncode=="532329" & year==2000

replace regioncode="532331" if regioncode=="532331" & year==2000

replace regioncode="532501" if regioncode=="532501" & year==2000

replace regioncode="532502" if regioncode=="532502" & year==2000

replace regioncode="532522" if regioncode=="532522" & year==2000

replace regioncode="532523" if regioncode=="532523" & year==2000

replace regioncode="532524" if regioncode=="532524" & year==2000

replace regioncode="532525" if regioncode=="532525" & year==2000

replace regioncode="532526" if regioncode=="532526" & year==2000

replace regioncode="532527" if regioncode=="532527" & year==2000

replace regioncode="532528" if regioncode=="532528" & year==2000

replace regioncode="532529" if regioncode=="532529" & year==2000

replace regioncode="532530" if regioncode=="532530" & year==2000

replace regioncode="532531" if regioncode=="532531" & year==2000

replace regioncode="532532" if regioncode=="532532" & year==2000

replace regioncode="532621" if regioncode=="532621" & year==2000

replace regioncode="532622" if regioncode=="532622" & year==2000

replace regioncode="532623" if regioncode=="532623" & year==2000

replace regioncode="532624" if regioncode=="532624" & year==2000

replace regioncode="532625" if regioncode=="532625" & year==2000

replace regioncode="532626" if regioncode=="532626" & year==2000

replace regioncode="532627" if regioncode=="532627" & year==2000

replace regioncode="532628" if regioncode=="532628" & year==2000

replace regioncode="532801" if regioncode=="532801" & year==2000

replace regioncode="532822" if regioncode=="532822" & year==2000

replace regioncode="532823" if regioncode=="532823" & year==2000

replace regioncode="532901" if regioncode=="532901" & year==2000

replace regioncode="532922" if regioncode=="532922" & year==2000

replace regioncode="532923" if regioncode=="532923" & year==2000

replace regioncode="532924" if regioncode=="532924" & year==2000

replace regioncode="532925" if regioncode=="532925" & year==2000

replace regioncode="532926" if regioncode=="532926" & year==2000

replace regioncode="532927" if regioncode=="532927" & year==2000

replace regioncode="532928" if regioncode=="532928" & year==2000

replace regioncode="532929" if regioncode=="532929" & year==2000

replace regioncode="532930" if regioncode=="532930" & year==2000

replace regioncode="532931" if regioncode=="532931" & year==2000

replace regioncode="532932" if regioncode=="532932" & year==2000

replace regioncode="533102" if regioncode=="533102" & year==2000

replace regioncode="533103" if regioncode=="533103" & year==2000

replace regioncode="533122" if regioncode=="533122" & year==2000

replace regioncode="533123" if regioncode=="533123" & year==2000

replace regioncode="533124" if regioncode=="533124" & year==2000

replace regioncode="533321" if regioncode=="533321" & year==2000

replace regioncode="533323" if regioncode=="533323" & year==2000

replace regioncode="533324" if regioncode=="533324" & year==2000

replace regioncode="533325" if regioncode=="533325" & year==2000

replace regioncode="533421" if regioncode=="533421" & year==2000

replace regioncode="533422" if regioncode=="533422" & year==2000

replace regioncode="533423" if regioncode=="533423" & year==2000

replace regioncode="540102" if regioncode=="540102" & year==2000

replace regioncode="540121" if regioncode=="540121" & year==2000

replace regioncode="540122" if regioncode=="540122" & year==2000

replace regioncode="540123" if regioncode=="540123" & year==2000

replace regioncode="540124" if regioncode=="540124" & year==2000

replace regioncode="540125" if regioncode=="540125" & year==2000

replace regioncode="540126" if regioncode=="540126" & year==2000

replace regioncode="540127" if regioncode=="540127" & year==2000

replace regioncode="542121" if regioncode=="542121" & year==2000

replace regioncode="542122" if regioncode=="542122" & year==2000

replace regioncode="542123" if regioncode=="542123" & year==2000

replace regioncode="542124" if regioncode=="542124" & year==2000

replace regioncode="542125" if regioncode=="542125" & year==2000

replace regioncode="542126" if regioncode=="542126" & year==2000

replace regioncode="542127" if regioncode=="542127" & year==2000

replace regioncode="542128" if regioncode=="542128" & year==2000

replace regioncode="542129" if regioncode=="542129" & year==2000

replace regioncode="542132" if regioncode=="542132" & year==2000

replace regioncode="542133" if regioncode=="542133" & year==2000

replace regioncode="542221" if regioncode=="542221" & year==2000

replace regioncode="542223" if regioncode=="542223" & year==2000

replace regioncode="542224" if regioncode=="542224" & year==2000

replace regioncode="542225" if regioncode=="542225" & year==2000

replace regioncode="542227" if regioncode=="542227" & year==2000

replace regioncode="542228" if regioncode=="542228" & year==2000

replace regioncode="542229" if regioncode=="542229" & year==2000

replace regioncode="542231" if regioncode=="542231" & year==2000

replace regioncode="542232" if regioncode=="542232" & year==2000

replace regioncode="542233" if regioncode=="542233" & year==2000

replace regioncode="542301" if regioncode=="542301" & year==2000

replace regioncode="542322" if regioncode=="542322" & year==2000

replace regioncode="542323" if regioncode=="542323" & year==2000

replace regioncode="542324" if regioncode=="542324" & year==2000

replace regioncode="542325" if regioncode=="542325" & year==2000

replace regioncode="542326" if regioncode=="542326" & year==2000

replace regioncode="542327" if regioncode=="542327" & year==2000

replace regioncode="542328" if regioncode=="542328" & year==2000

replace regioncode="542329" if regioncode=="542329" & year==2000

replace regioncode="542330" if regioncode=="542330" & year==2000

replace regioncode="542331" if regioncode=="542331" & year==2000

replace regioncode="542332" if regioncode=="542332" & year==2000

replace regioncode="542334" if regioncode=="542334" & year==2000

replace regioncode="542336" if regioncode=="542336" & year==2000

replace regioncode="542338" if regioncode=="542338" & year==2000

replace regioncode="542421" if regioncode=="542421" & year==2000

replace regioncode="542422" if regioncode=="542422" & year==2000

replace regioncode="542423" if regioncode=="542423" & year==2000

replace regioncode="542424" if regioncode=="542424" & year==2000

replace regioncode="542425" if regioncode=="542425" & year==2000

replace regioncode="542426" if regioncode=="542426" & year==2000

replace regioncode="542427" if regioncode=="542427" & year==2000

replace regioncode="542429" if regioncode=="542429" & year==2000

replace regioncode="542430" if regioncode=="542430" & year==2000

replace regioncode="542523" if regioncode=="542523" & year==2000

replace regioncode="542621" if regioncode=="542621" & year==2000

replace regioncode="542622" if regioncode=="542622" & year==2000

replace regioncode="542623" if regioncode=="542623" & year==2000

replace regioncode="542625" if regioncode=="542625" & year==2000

replace regioncode="542626" if regioncode=="542626" & year==2000

replace regioncode="610102" if regioncode=="610102" & year==2000

replace regioncode="610103" if regioncode=="610103" & year==2000

replace regioncode="610104" if regioncode=="610104" & year==2000

replace regioncode="610111" if regioncode=="610111" & year==2000

replace regioncode="610112" if regioncode=="610112" & year==2000

replace regioncode="610113" if regioncode=="610113" & year==2000

replace regioncode="610114" if regioncode=="610114" & year==2000

replace regioncode="610115" if regioncode=="610115" & year==2000

replace regioncode="610116" if regioncode=="610121" & year==2000

replace regioncode="610122" if regioncode=="610122" & year==2000

replace regioncode="610124" if regioncode=="610124" & year==2000

replace regioncode="610125" if regioncode=="610125" & year==2000

replace regioncode="610126" if regioncode=="610126" & year==2000

replace regioncode="610202" if regioncode=="610202" & year==2000

replace regioncode="610203" if regioncode=="610203" & year==2000

replace regioncode="610204" if regioncode=="610221" & year==2000

replace regioncode="610222" if regioncode=="610222" & year==2000

replace regioncode="610302" if regioncode=="610302" & year==2000

replace regioncode="610303" if regioncode=="610303" & year==2000

replace regioncode="610304" if regioncode=="610321" & year==2000

replace regioncode="610322" if regioncode=="610322" & year==2000

replace regioncode="610323" if regioncode=="610323" & year==2000

replace regioncode="610324" if regioncode=="610324" & year==2000

replace regioncode="610326" if regioncode=="610326" & year==2000

replace regioncode="610327" if regioncode=="610327" & year==2000

replace regioncode="610328" if regioncode=="610328" & year==2000

replace regioncode="610329" if regioncode=="610329" & year==2000

replace regioncode="610330" if regioncode=="610330" & year==2000

replace regioncode="610331" if regioncode=="610331" & year==2000

replace regioncode="610402" if regioncode=="610402" & year==2000

replace regioncode="610403" if regioncode=="610403" & year==2000

replace regioncode="610404" if regioncode=="610404" & year==2000

replace regioncode="610422" if regioncode=="610422" & year==2000

replace regioncode="610423" if regioncode=="610423" & year==2000

replace regioncode="610424" if regioncode=="610424" & year==2000

replace regioncode="610425" if regioncode=="610425" & year==2000

replace regioncode="610426" if regioncode=="610426" & year==2000

replace regioncode="610427" if regioncode=="610427" & year==2000

replace regioncode="610428" if regioncode=="610428" & year==2000

replace regioncode="610429" if regioncode=="610429" & year==2000

replace regioncode="610430" if regioncode=="610430" & year==2000

replace regioncode="610431" if regioncode=="610431" & year==2000

replace regioncode="610481" if regioncode=="610481" & year==2000

replace regioncode="610502" if regioncode=="610502" & year==2000

replace regioncode="610521" if regioncode=="610521" & year==2000

replace regioncode="610522" if regioncode=="610522" & year==2000

replace regioncode="610523" if regioncode=="610523" & year==2000

replace regioncode="610524" if regioncode=="610524" & year==2000

replace regioncode="610525" if regioncode=="610525" & year==2000

replace regioncode="610526" if regioncode=="610526" & year==2000

replace regioncode="610527" if regioncode=="610527" & year==2000

replace regioncode="610528" if regioncode=="610528" & year==2000

replace regioncode="610581" if regioncode=="610581" & year==2000

replace regioncode="610582" if regioncode=="610582" & year==2000

replace regioncode="610602" if regioncode=="612601" & year==2000

replace regioncode="610621" if regioncode=="610621" & year==2000

replace regioncode="610622" if regioncode=="610622" & year==2000

replace regioncode="610623" if regioncode=="610623" & year==2000

replace regioncode="610624" if regioncode=="610624" & year==2000

replace regioncode="610625" if regioncode=="610625" & year==2000

replace regioncode="610626" if regioncode=="610626" & year==2000

replace regioncode="610627" if regioncode=="610627" & year==2000

replace regioncode="610628" if regioncode=="610628" & year==2000

replace regioncode="610629" if regioncode=="610629" & year==2000

replace regioncode="610630" if regioncode=="610630" & year==2000

replace regioncode="610631" if regioncode=="610631" & year==2000

replace regioncode="610632" if regioncode=="610632" & year==2000

replace regioncode="610702" if regioncode=="610702" & year==2000

replace regioncode="610721" if regioncode=="610721" & year==2000

replace regioncode="610722" if regioncode=="610722" & year==2000

replace regioncode="610723" if regioncode=="610723" & year==2000

replace regioncode="610724" if regioncode=="610724" & year==2000

replace regioncode="610725" if regioncode=="610725" & year==2000

replace regioncode="610726" if regioncode=="610726" & year==2000

replace regioncode="610727" if regioncode=="610727" & year==2000

replace regioncode="610728" if regioncode=="610728" & year==2000

replace regioncode="610729" if regioncode=="610729" & year==2000

replace regioncode="610730" if regioncode=="610730" & year==2000

replace regioncode="610802" if regioncode=="610801" & year==2000

replace regioncode="610821" if regioncode=="610821" & year==2000

replace regioncode="610822" if regioncode=="610822" & year==2000

replace regioncode="610823" if regioncode=="610823" & year==2000

replace regioncode="610824" if regioncode=="610824" & year==2000

replace regioncode="610825" if regioncode=="610825" & year==2000

replace regioncode="610826" if regioncode=="610826" & year==2000

replace regioncode="610827" if regioncode=="610827" & year==2000

replace regioncode="610828" if regioncode=="610828" & year==2000

replace regioncode="610829" if regioncode=="610829" & year==2000

replace regioncode="610830" if regioncode=="610830" & year==2000

replace regioncode="610831" if regioncode=="610831" & year==2000

replace regioncode="610902" if regioncode=="610902" & year==2000

replace regioncode="610921" if regioncode=="610921" & year==2000

replace regioncode="610922" if regioncode=="610922" & year==2000

replace regioncode="610923" if regioncode=="610923" & year==2000

replace regioncode="610924" if regioncode=="610924" & year==2000

replace regioncode="610925" if regioncode=="610925" & year==2000

replace regioncode="610926" if regioncode=="610926" & year==2000

replace regioncode="610927" if regioncode=="610927" & year==2000

replace regioncode="610928" if regioncode=="610928" & year==2000

replace regioncode="610929" if regioncode=="610929" & year==2000

replace regioncode="611002" if regioncode=="612501" & year==2000

replace regioncode="611021" if regioncode=="612522" & year==2000

replace regioncode="611022" if regioncode=="612523" & year==2000

replace regioncode="611023" if regioncode=="612524" & year==2000

replace regioncode="611024" if regioncode=="612525" & year==2000

replace regioncode="611025" if regioncode=="612526" & year==2000

replace regioncode="611026" if regioncode=="612527" & year==2000

replace regioncode="620102" if regioncode=="620102" & year==2000

replace regioncode="620103" if regioncode=="620103" & year==2000

replace regioncode="620104" if regioncode=="620104" & year==2000

replace regioncode="620105" if regioncode=="620105" & year==2000

replace regioncode="620111" if regioncode=="620111" & year==2000

replace regioncode="620121" if regioncode=="620121" & year==2000

replace regioncode="620122" if regioncode=="620122" & year==2000

replace regioncode="620123" if regioncode=="620123" & year==2000

replace regioncode="620201" if regioncode=="620201" & year==2000

replace regioncode="620302" if regioncode=="620302" & year==2000

replace regioncode="620321" if regioncode=="620321" & year==2000

replace regioncode="620402" if regioncode=="620402" & year==2000

replace regioncode="620403" if regioncode=="620403" & year==2000

replace regioncode="620421" if regioncode=="620421" & year==2000

replace regioncode="620422" if regioncode=="620422" & year==2000

replace regioncode="620423" if regioncode=="620423" & year==2000

replace regioncode="620502" if regioncode=="620502" & year==2000

replace regioncode="620503" if regioncode=="620503" & year==2000

replace regioncode="620521" if regioncode=="620521" & year==2000

replace regioncode="620522" if regioncode=="620522" & year==2000

replace regioncode="620523" if regioncode=="620523" & year==2000

replace regioncode="620524" if regioncode=="620524" & year==2000

replace regioncode="620525" if regioncode=="620525" & year==2000

replace regioncode="622301" if regioncode=="622301" & year==2000

replace regioncode="622322" if regioncode=="622322" & year==2000

replace regioncode="622323" if regioncode=="622323" & year==2000

replace regioncode="622326" if regioncode=="622326" & year==2000

replace regioncode="622201" if regioncode=="622201" & year==2000

replace regioncode="622222" if regioncode=="622222" & year==2000

replace regioncode="622223" if regioncode=="622223" & year==2000

replace regioncode="622224" if regioncode=="622224" & year==2000

replace regioncode="622225" if regioncode=="622225" & year==2000

replace regioncode="622226" if regioncode=="622226" & year==2000

replace regioncode="622701" if regioncode=="622701" & year==2000

replace regioncode="622722" if regioncode=="622722" & year==2000

replace regioncode="622723" if regioncode=="622723" & year==2000

replace regioncode="622724" if regioncode=="622724" & year==2000

replace regioncode="622725" if regioncode=="622725" & year==2000

replace regioncode="622726" if regioncode=="622726" & year==2000

replace regioncode="622727" if regioncode=="622727" & year==2000

replace regioncode="622102" if regioncode=="622102" & year==2000

replace regioncode="622123" if regioncode=="622123" & year==2000

replace regioncode="622126" if regioncode=="622126" & year==2000

replace regioncode="622124" if regioncode=="622124" & year==2000

replace regioncode="622125" if regioncode=="622125" & year==2000

replace regioncode="622101" if regioncode=="622101" & year==2000

replace regioncode="622103" if regioncode=="622103" & year==2000

replace regioncode="622801" if regioncode=="622801" & year==2000

replace regioncode="622821" if regioncode=="622821" & year==2000

replace regioncode="622822" if regioncode=="622822" & year==2000

replace regioncode="622823" if regioncode=="622823" & year==2000

replace regioncode="622824" if regioncode=="622824" & year==2000

replace regioncode="622825" if regioncode=="622825" & year==2000

replace regioncode="622826" if regioncode=="622826" & year==2000

replace regioncode="622827" if regioncode=="622827" & year==2000

replace regioncode="622421" if regioncode=="622421" & year==2000

replace regioncode="622424" if regioncode=="622424" & year==2000

replace regioncode="622425" if regioncode=="622425" & year==2000

replace regioncode="622426" if regioncode=="622426" & year==2000

replace regioncode="622427" if regioncode=="622427" & year==2000

replace regioncode="622428" if regioncode=="622428" & year==2000

replace regioncode="622429" if regioncode=="622429" & year==2000

replace regioncode="622621" if regioncode=="622621" & year==2000

replace regioncode="622624" if regioncode=="622624" & year==2000

replace regioncode="622626" if regioncode=="622626" & year==2000

replace regioncode="622623" if regioncode=="622623" & year==2000

replace regioncode="622625" if regioncode=="622625" & year==2000

replace regioncode="622627" if regioncode=="622627" & year==2000

replace regioncode="622628" if regioncode=="622628" & year==2000

replace regioncode="622630" if regioncode=="622630" & year==2000

replace regioncode="622629" if regioncode=="622629" & year==2000

replace regioncode="622901" if regioncode=="622901" & year==2000

replace regioncode="622921" if regioncode=="622921" & year==2000

replace regioncode="622922" if regioncode=="622922" & year==2000

replace regioncode="622923" if regioncode=="622923" & year==2000

replace regioncode="622924" if regioncode=="622924" & year==2000

replace regioncode="622925" if regioncode=="622925" & year==2000

replace regioncode="622926" if regioncode=="622926" & year==2000

replace regioncode="622927" if regioncode=="622927" & year==2000

replace regioncode="623001" if regioncode=="623001" & year==2000

replace regioncode="623021" if regioncode=="623021" & year==2000

replace regioncode="623022" if regioncode=="623022" & year==2000

replace regioncode="623023" if regioncode=="623023" & year==2000

replace regioncode="623024" if regioncode=="623024" & year==2000

replace regioncode="623025" if regioncode=="623025" & year==2000

replace regioncode="623026" if regioncode=="623026" & year==2000

replace regioncode="623027" if regioncode=="623027" & year==2000

replace regioncode="630102" if regioncode=="630102" & year==2000

replace regioncode="630103" if regioncode=="630103" & year==2000

replace regioncode="630104" if regioncode=="630104" & year==2000

replace regioncode="630105" if regioncode=="630105" & year==2000

replace regioncode="630121" if regioncode=="630121" & year==2000

replace regioncode="630122" if regioncode=="630122" & year==2000

replace regioncode="630123" if regioncode=="630123" & year==2000

replace regioncode="632121" if regioncode=="632121" & year==2000

replace regioncode="632122" if regioncode=="632122" & year==2000

replace regioncode="632123" if regioncode=="632123" & year==2000

replace regioncode="632126" if regioncode=="632126" & year==2000

replace regioncode="632127" if regioncode=="632127" & year==2000

replace regioncode="632128" if regioncode=="632128" & year==2000

replace regioncode="632221" if regioncode=="632221" & year==2000

replace regioncode="632222" if regioncode=="632222" & year==2000

replace regioncode="632223" if regioncode=="632223" & year==2000

replace regioncode="632224" if regioncode=="632224" & year==2000

replace regioncode="632321" if regioncode=="632321" & year==2000

replace regioncode="632322" if regioncode=="632322" & year==2000

replace regioncode="632521" if regioncode=="632521" & year==2000

replace regioncode="632522" if regioncode=="632522" & year==2000

replace regioncode="632523" if regioncode=="632523" & year==2000

replace regioncode="632524" if regioncode=="632524" & year==2000

replace regioncode="632525" if regioncode=="632525" & year==2000

replace regioncode="632621" if regioncode=="632621" & year==2000

replace regioncode="632622" if regioncode=="632622" & year==2000

replace regioncode="632623" if regioncode=="632623" & year==2000

replace regioncode="632624" if regioncode=="632624" & year==2000

replace regioncode="632625" if regioncode=="632625" & year==2000

replace regioncode="632626" if regioncode=="632626" & year==2000

replace regioncode="632721" if regioncode=="632721" & year==2000

replace regioncode="632723" if regioncode=="632723" & year==2000

replace regioncode="632724" if regioncode=="632724" & year==2000

replace regioncode="632725" if regioncode=="632725" & year==2000

replace regioncode="632726" if regioncode=="632726" & year==2000

replace regioncode="632801" if regioncode=="632801" & year==2000

replace regioncode="632802" if regioncode=="632802" & year==2000

replace regioncode="632821" if regioncode=="632821" & year==2000

replace regioncode="632822" if regioncode=="632822" & year==2000

replace regioncode="640104" if regioncode=="640102" & year==2000

replace regioncode="640105" if regioncode=="640103" & year==2000

replace regioncode="640106" if regioncode=="640111" & year==2000

replace regioncode="640121" if regioncode=="640121" & year==2000

replace regioncode="640122" if regioncode=="640122" & year==2000

replace regioncode="640181" if regioncode=="640382" & year==2000

replace regioncode="640202" if regioncode=="640202" & year==2000

replace regioncode="640203" if regioncode=="640203" & year==2000

replace regioncode="640221" if regioncode=="640221" & year==2000

replace regioncode="640302" if regioncode=="640302" & year==2000

replace regioncode="640321" if regioncode=="640321" & year==2000

replace regioncode="640322" if regioncode=="640322" & year==2000

replace regioncode="640323" if regioncode=="640323" & year==2000

replace regioncode="640324" if regioncode=="640324" & year==2000

replace regioncode="640381" if regioncode=="640381" & year==2000

replace regioncode="640402" if regioncode=="642221" & year==2000

replace regioncode="640421" if regioncode=="642222" & year==2000

replace regioncode="640422" if regioncode=="642223" & year==2000

replace regioncode="640423" if regioncode=="642224" & year==2000

replace regioncode="640424" if regioncode=="642225" & year==2000

replace regioncode="640425" if regioncode=="642226" & year==2000

replace regioncode="650102" if regioncode=="650102" & year==2000

replace regioncode="650103" if regioncode=="650103" & year==2000

replace regioncode="650104" if regioncode=="650104" & year==2000

replace regioncode="650105" if regioncode=="650105" & year==2000

replace regioncode="650106" if regioncode=="650106" & year==2000

replace regioncode="650107" if regioncode=="650107" & year==2000

replace regioncode="650108" if regioncode=="650108" & year==2000

replace regioncode="650121" if regioncode=="650121" & year==2000

replace regioncode="650202" if regioncode=="650202" & year==2000

replace regioncode="650203" if regioncode=="650203" & year==2000

replace regioncode="650204" if regioncode=="650204" & year==2000

replace regioncode="650205" if regioncode=="650205" & year==2000

replace regioncode="652101" if regioncode=="652101" & year==2000

replace regioncode="652122" if regioncode=="652122" & year==2000

replace regioncode="652123" if regioncode=="652123" & year==2000

replace regioncode="652201" if regioncode=="652201" & year==2000

replace regioncode="652222" if regioncode=="652222" & year==2000

replace regioncode="652223" if regioncode=="652223" & year==2000

replace regioncode="652301" if regioncode=="652301" & year==2000

replace regioncode="652302" if regioncode=="652302" & year==2000

replace regioncode="652303" if regioncode=="652303" & year==2000

replace regioncode="652323" if regioncode=="652323" & year==2000

replace regioncode="652324" if regioncode=="652324" & year==2000

replace regioncode="652325" if regioncode=="652325" & year==2000

replace regioncode="652327" if regioncode=="652327" & year==2000

replace regioncode="652328" if regioncode=="652328" & year==2000

replace regioncode="652701" if regioncode=="652701" & year==2000

replace regioncode="652722" if regioncode=="652722" & year==2000

replace regioncode="652723" if regioncode=="652723" & year==2000

replace regioncode="652801" if regioncode=="652801" & year==2000

replace regioncode="652822" if regioncode=="652822" & year==2000

replace regioncode="652823" if regioncode=="652823" & year==2000

replace regioncode="652824" if regioncode=="652824" & year==2000

replace regioncode="652825" if regioncode=="652825" & year==2000

replace regioncode="652826" if regioncode=="652826" & year==2000

replace regioncode="652827" if regioncode=="652827" & year==2000

replace regioncode="652828" if regioncode=="652828" & year==2000

replace regioncode="652829" if regioncode=="652829" & year==2000

replace regioncode="652901" if regioncode=="652901" & year==2000

replace regioncode="652922" if regioncode=="652922" & year==2000

replace regioncode="652923" if regioncode=="652923" & year==2000

replace regioncode="652924" if regioncode=="652924" & year==2000

replace regioncode="652925" if regioncode=="652925" & year==2000

replace regioncode="652926" if regioncode=="652926" & year==2000

replace regioncode="652928" if regioncode=="652928" & year==2000

replace regioncode="652929" if regioncode=="652929" & year==2000

replace regioncode="653001" if regioncode=="653001" & year==2000

replace regioncode="653022" if regioncode=="653022" & year==2000

replace regioncode="653023" if regioncode=="653023" & year==2000

replace regioncode="653024" if regioncode=="653024" & year==2000

replace regioncode="653101" if regioncode=="653101" & year==2000

replace regioncode="653121" if regioncode=="653121" & year==2000

replace regioncode="653122" if regioncode=="653122" & year==2000

replace regioncode="653123" if regioncode=="653123" & year==2000

replace regioncode="653124" if regioncode=="653124" & year==2000

replace regioncode="653125" if regioncode=="653125" & year==2000

replace regioncode="653126" if regioncode=="653126" & year==2000

replace regioncode="653127" if regioncode=="653127" & year==2000

replace regioncode="653128" if regioncode=="653128" & year==2000

replace regioncode="653129" if regioncode=="653129" & year==2000

replace regioncode="653130" if regioncode=="653130" & year==2000

replace regioncode="653131" if regioncode=="653131" & year==2000

replace regioncode="653201" if regioncode=="653201" & year==2000

replace regioncode="653221" if regioncode=="653221" & year==2000

replace regioncode="653222" if regioncode=="653222" & year==2000

replace regioncode="653223" if regioncode=="653223" & year==2000

replace regioncode="653224" if regioncode=="653224" & year==2000

replace regioncode="653225" if regioncode=="653225" & year==2000

replace regioncode="653226" if regioncode=="653226" & year==2000

replace regioncode="653227" if regioncode=="653227" & year==2000

replace regioncode="654002" if regioncode=="654101" & year==2000

replace regioncode="654003" if regioncode=="654001" & year==2000

replace regioncode="654021" if regioncode=="654121" & year==2000

replace regioncode="654022" if regioncode=="654122" & year==2000

replace regioncode="654023" if regioncode=="654123" & year==2000

replace regioncode="654024" if regioncode=="654124" & year==2000

replace regioncode="654025" if regioncode=="654125" & year==2000

replace regioncode="654026" if regioncode=="654126" & year==2000

replace regioncode="654027" if regioncode=="654127" & year==2000

replace regioncode="654028" if regioncode=="654128" & year==2000

replace regioncode="654201" if regioncode=="654201" & year==2000

replace regioncode="654202" if regioncode=="654202" & year==2000

replace regioncode="654221" if regioncode=="654221" & year==2000

replace regioncode="654223" if regioncode=="654223" & year==2000

replace regioncode="654224" if regioncode=="654224" & year==2000

replace regioncode="654225" if regioncode=="654225" & year==2000

replace regioncode="654226" if regioncode=="654226" & year==2000

replace regioncode="654301" if regioncode=="654301" & year==2000

replace regioncode="654321" if regioncode=="654321" & year==2000

replace regioncode="654322" if regioncode=="654322" & year==2000

replace regioncode="654323" if regioncode=="654323" & year==2000

replace regioncode="654324" if regioncode=="654324" & year==2000

replace regioncode="654325" if regioncode=="654325" & year==2000

replace regioncode="654326" if regioncode=="654326" & year==2000

replace regioncode="659001" if regioncode=="659001" & year==2000

replace regioncode="110101" if regioncode=="110101" & year==2001

replace regioncode="110102" if regioncode=="110102" & year==2001

replace regioncode="110103" if regioncode=="110103" & year==2001

replace regioncode="110104" if regioncode=="110104" & year==2001

replace regioncode="110105" if regioncode=="110105" & year==2001

replace regioncode="110106" if regioncode=="110106" & year==2001

replace regioncode="110107" if regioncode=="110107" & year==2001

replace regioncode="110108" if regioncode=="110108" & year==2001

replace regioncode="110109" if regioncode=="110109" & year==2001

replace regioncode="110111" if regioncode=="110111" & year==2001

replace regioncode="110112" if regioncode=="110112" & year==2001

replace regioncode="110113" if regioncode=="110113" & year==2001

replace regioncode="110114" if regioncode=="110114" & year==2001

replace regioncode="110115" if regioncode=="110115" & year==2001

replace regioncode="110117" if regioncode=="110226" & year==2001

replace regioncode="110116" if regioncode=="110227" & year==2001

replace regioncode="110228" if regioncode=="110228" & year==2001

replace regioncode="110229" if regioncode=="110229" & year==2001

replace regioncode="120101" if regioncode=="120101" & year==2001

replace regioncode="120102" if regioncode=="120102" & year==2001

replace regioncode="120103" if regioncode=="120103" & year==2001

replace regioncode="120104" if regioncode=="120104" & year==2001

replace regioncode="120105" if regioncode=="120105" & year==2001

replace regioncode="120106" if regioncode=="120106" & year==2001

replace regioncode="120107" if regioncode=="120107" & year==2001

replace regioncode="120108" if regioncode=="120108" & year==2001

replace regioncode="120109" if regioncode=="120109" & year==2001

replace regioncode="120110" if regioncode=="120110" & year==2001

replace regioncode="120111" if regioncode=="120111" & year==2001

replace regioncode="120112" if regioncode=="120112" & year==2001

replace regioncode="120113" if regioncode=="120113" & year==2001

replace regioncode="120114" if regioncode=="120114" & year==2001

replace regioncode="120115" if regioncode=="120115" & year==2001

replace regioncode="120221" if regioncode=="120221" & year==2001

replace regioncode="120223" if regioncode=="120223" & year==2001

replace regioncode="120225" if regioncode=="120225" & year==2001

replace regioncode="130102" if regioncode=="130102" & year==2001

replace regioncode="130103" if regioncode=="130103" & year==2001

replace regioncode="130104" if regioncode=="130104" & year==2001

replace regioncode="130105" if regioncode=="130105" & year==2001

replace regioncode="130107" if regioncode=="130107" & year==2001

replace regioncode="130108" if regioncode=="130108" & year==2001

replace regioncode="130121" if regioncode=="130121" & year==2001

replace regioncode="130123" if regioncode=="130123" & year==2001

replace regioncode="130124" if regioncode=="130124" & year==2001

replace regioncode="130125" if regioncode=="130125" & year==2001

replace regioncode="130126" if regioncode=="130126" & year==2001

replace regioncode="130127" if regioncode=="130127" & year==2001

replace regioncode="130128" if regioncode=="130128" & year==2001

replace regioncode="130129" if regioncode=="130129" & year==2001

replace regioncode="130130" if regioncode=="130130" & year==2001

replace regioncode="130131" if regioncode=="130131" & year==2001

replace regioncode="130132" if regioncode=="130132" & year==2001

replace regioncode="130133" if regioncode=="130133" & year==2001

replace regioncode="130181" if regioncode=="130181" & year==2001

replace regioncode="130182" if regioncode=="130182" & year==2001

replace regioncode="130183" if regioncode=="130183" & year==2001

replace regioncode="130184" if regioncode=="130184" & year==2001

replace regioncode="130185" if regioncode=="130185" & year==2001

replace regioncode="130202" if regioncode=="130202" & year==2001

replace regioncode="130203" if regioncode=="130203" & year==2001

replace regioncode="130204" if regioncode=="130204" & year==2001

replace regioncode="130205" if regioncode=="130205" & year==2001

replace regioncode="130208" if regioncode=="130221" & year==2001

replace regioncode="130223" if regioncode=="130223" & year==2001

replace regioncode="130224" if regioncode=="130224" & year==2001

replace regioncode="130225" if regioncode=="130225" & year==2001

replace regioncode="130227" if regioncode=="130227" & year==2001

replace regioncode="130229" if regioncode=="130229" & year==2001

replace regioncode="130230" if regioncode=="130230" & year==2001

replace regioncode="130281" if regioncode=="130281" & year==2001

replace regioncode="130207" if regioncode=="130282" & year==2001

replace regioncode="130283" if regioncode=="130283" & year==2001

replace regioncode="130302" if regioncode=="130302" & year==2001

replace regioncode="130303" if regioncode=="130303" & year==2001

replace regioncode="130304" if regioncode=="130304" & year==2001

replace regioncode="130321" if regioncode=="130321" & year==2001

replace regioncode="130322" if regioncode=="130322" & year==2001

replace regioncode="130323" if regioncode=="130323" & year==2001

replace regioncode="130324" if regioncode=="130324" & year==2001

replace regioncode="130402" if regioncode=="130402" & year==2001

replace regioncode="130403" if regioncode=="130403" & year==2001

replace regioncode="130404" if regioncode=="130404" & year==2001

replace regioncode="130406" if regioncode=="130406" & year==2001

replace regioncode="130421" if regioncode=="130421" & year==2001

replace regioncode="130423" if regioncode=="130423" & year==2001

replace regioncode="130424" if regioncode=="130424" & year==2001

replace regioncode="130425" if regioncode=="130425" & year==2001

replace regioncode="130426" if regioncode=="130426" & year==2001

replace regioncode="130427" if regioncode=="130427" & year==2001

replace regioncode="130428" if regioncode=="130428" & year==2001

replace regioncode="130429" if regioncode=="130429" & year==2001

replace regioncode="130430" if regioncode=="130430" & year==2001

replace regioncode="130431" if regioncode=="130431" & year==2001

replace regioncode="130432" if regioncode=="130432" & year==2001

replace regioncode="130433" if regioncode=="130433" & year==2001

replace regioncode="130434" if regioncode=="130434" & year==2001

replace regioncode="130435" if regioncode=="130435" & year==2001

replace regioncode="130481" if regioncode=="130481" & year==2001

replace regioncode="130502" if regioncode=="130502" & year==2001

replace regioncode="130503" if regioncode=="130503" & year==2001

replace regioncode="130521" if regioncode=="130521" & year==2001

replace regioncode="130522" if regioncode=="130522" & year==2001

replace regioncode="130523" if regioncode=="130523" & year==2001

replace regioncode="130524" if regioncode=="130524" & year==2001

replace regioncode="130525" if regioncode=="130525" & year==2001

replace regioncode="130526" if regioncode=="130526" & year==2001

replace regioncode="130527" if regioncode=="130527" & year==2001

replace regioncode="130528" if regioncode=="130528" & year==2001

replace regioncode="130529" if regioncode=="130529" & year==2001

replace regioncode="130530" if regioncode=="130530" & year==2001

replace regioncode="130531" if regioncode=="130531" & year==2001

replace regioncode="130532" if regioncode=="130532" & year==2001

replace regioncode="130533" if regioncode=="130533" & year==2001

replace regioncode="130534" if regioncode=="130534" & year==2001

replace regioncode="130535" if regioncode=="130535" & year==2001

replace regioncode="130581" if regioncode=="130581" & year==2001

replace regioncode="130582" if regioncode=="130582" & year==2001

replace regioncode="130602" if regioncode=="130602" & year==2001

replace regioncode="130603" if regioncode=="130603" & year==2001

replace regioncode="130604" if regioncode=="130604" & year==2001

replace regioncode="130621" if regioncode=="130621" & year==2001

replace regioncode="130622" if regioncode=="130622" & year==2001

replace regioncode="130623" if regioncode=="130623" & year==2001

replace regioncode="130624" if regioncode=="130624" & year==2001

replace regioncode="130625" if regioncode=="130625" & year==2001

replace regioncode="130626" if regioncode=="130626" & year==2001

replace regioncode="130627" if regioncode=="130627" & year==2001

replace regioncode="130628" if regioncode=="130628" & year==2001

replace regioncode="130629" if regioncode=="130629" & year==2001

replace regioncode="130630" if regioncode=="130630" & year==2001

replace regioncode="130631" if regioncode=="130631" & year==2001

replace regioncode="130632" if regioncode=="130632" & year==2001

replace regioncode="130633" if regioncode=="130633" & year==2001

replace regioncode="130634" if regioncode=="130634" & year==2001

replace regioncode="130635" if regioncode=="130635" & year==2001

replace regioncode="130636" if regioncode=="130636" & year==2001

replace regioncode="130637" if regioncode=="130637" & year==2001

replace regioncode="130638" if regioncode=="130638" & year==2001

replace regioncode="130681" if regioncode=="130681" & year==2001

replace regioncode="130682" if regioncode=="130682" & year==2001

replace regioncode="130683" if regioncode=="130683" & year==2001

replace regioncode="130684" if regioncode=="130684" & year==2001

replace regioncode="130702" if regioncode=="130702" & year==2001

replace regioncode="130703" if regioncode=="130703" & year==2001

replace regioncode="130705" if regioncode=="130705" & year==2001

replace regioncode="130706" if regioncode=="130706" & year==2001

replace regioncode="130721" if regioncode=="130721" & year==2001

replace regioncode="130722" if regioncode=="130722" & year==2001

replace regioncode="130723" if regioncode=="130723" & year==2001

replace regioncode="130724" if regioncode=="130724" & year==2001

replace regioncode="130725" if regioncode=="130725" & year==2001

replace regioncode="130726" if regioncode=="130726" & year==2001

replace regioncode="130727" if regioncode=="130727" & year==2001

replace regioncode="130728" if regioncode=="130728" & year==2001

replace regioncode="130729" if regioncode=="130729" & year==2001

replace regioncode="130730" if regioncode=="130730" & year==2001

replace regioncode="130731" if regioncode=="130731" & year==2001

replace regioncode="130732" if regioncode=="130732" & year==2001

replace regioncode="130733" if regioncode=="130733" & year==2001

replace regioncode="130802" if regioncode=="130802" & year==2001

replace regioncode="130803" if regioncode=="130803" & year==2001

replace regioncode="130804" if regioncode=="130804" & year==2001

replace regioncode="130821" if regioncode=="130821" & year==2001

replace regioncode="130822" if regioncode=="130822" & year==2001

replace regioncode="130823" if regioncode=="130823" & year==2001

replace regioncode="130824" if regioncode=="130824" & year==2001

replace regioncode="130825" if regioncode=="130825" & year==2001

replace regioncode="130826" if regioncode=="130826" & year==2001

replace regioncode="130827" if regioncode=="130827" & year==2001

replace regioncode="130828" if regioncode=="130828" & year==2001

replace regioncode="130902" if regioncode=="130902" & year==2001

replace regioncode="130903" if regioncode=="130903" & year==2001

replace regioncode="130921" if regioncode=="130921" & year==2001

replace regioncode="130922" if regioncode=="130922" & year==2001

replace regioncode="130923" if regioncode=="130923" & year==2001

replace regioncode="130924" if regioncode=="130924" & year==2001

replace regioncode="130925" if regioncode=="130925" & year==2001

replace regioncode="130926" if regioncode=="130926" & year==2001

replace regioncode="130927" if regioncode=="130927" & year==2001

replace regioncode="130928" if regioncode=="130928" & year==2001

replace regioncode="130929" if regioncode=="130929" & year==2001

replace regioncode="130930" if regioncode=="130930" & year==2001

replace regioncode="130981" if regioncode=="130981" & year==2001

replace regioncode="130982" if regioncode=="130982" & year==2001

replace regioncode="130983" if regioncode=="130983" & year==2001

replace regioncode="130984" if regioncode=="130984" & year==2001

replace regioncode="131002" if regioncode=="131002" & year==2001

replace regioncode="131003" if regioncode=="131003" & year==2001

replace regioncode="131022" if regioncode=="131022" & year==2001

replace regioncode="131023" if regioncode=="131023" & year==2001

replace regioncode="131024" if regioncode=="131024" & year==2001

replace regioncode="131025" if regioncode=="131025" & year==2001

replace regioncode="131026" if regioncode=="131026" & year==2001

replace regioncode="131028" if regioncode=="131028" & year==2001

replace regioncode="131081" if regioncode=="131081" & year==2001

replace regioncode="131082" if regioncode=="131082" & year==2001

replace regioncode="131102" if regioncode=="131102" & year==2001

replace regioncode="131121" if regioncode=="131121" & year==2001

replace regioncode="131122" if regioncode=="131122" & year==2001

replace regioncode="131123" if regioncode=="131123" & year==2001

replace regioncode="131124" if regioncode=="131124" & year==2001

replace regioncode="131125" if regioncode=="131125" & year==2001

replace regioncode="131126" if regioncode=="131126" & year==2001

replace regioncode="131127" if regioncode=="131127" & year==2001

replace regioncode="131128" if regioncode=="131128" & year==2001

replace regioncode="131181" if regioncode=="131181" & year==2001

replace regioncode="131182" if regioncode=="131182" & year==2001

replace regioncode="140105" if regioncode=="140105" & year==2001

replace regioncode="140106" if regioncode=="140106" & year==2001

replace regioncode="140107" if regioncode=="140107" & year==2001

replace regioncode="140108" if regioncode=="140108" & year==2001

replace regioncode="140109" if regioncode=="140109" & year==2001

replace regioncode="140110" if regioncode=="140110" & year==2001

replace regioncode="140121" if regioncode=="140121" & year==2001

replace regioncode="140122" if regioncode=="140122" & year==2001

replace regioncode="140123" if regioncode=="140123" & year==2001

replace regioncode="140181" if regioncode=="140181" & year==2001

replace regioncode="140202" if regioncode=="140202" & year==2001

replace regioncode="140203" if regioncode=="140203" & year==2001

replace regioncode="140211" if regioncode=="140211" & year==2001

replace regioncode="140212" if regioncode=="140212" & year==2001

replace regioncode="140221" if regioncode=="140221" & year==2001

replace regioncode="140222" if regioncode=="140222" & year==2001

replace regioncode="140223" if regioncode=="140223" & year==2001

replace regioncode="140224" if regioncode=="140224" & year==2001

replace regioncode="140225" if regioncode=="140225" & year==2001

replace regioncode="140226" if regioncode=="140226" & year==2001

replace regioncode="140227" if regioncode=="140227" & year==2001

replace regioncode="140302" if regioncode=="140301" & year==2001

replace regioncode="140303" if regioncode=="140303" & year==2001

replace regioncode="140311" if regioncode=="140311" & year==2001

replace regioncode="140321" if regioncode=="140321" & year==2001

replace regioncode="140322" if regioncode=="140322" & year==2001

replace regioncode="140402" if regioncode=="140402" & year==2001

replace regioncode="140411" if regioncode=="140411" & year==2001

replace regioncode="140421" if regioncode=="140421" & year==2001

replace regioncode="140423" if regioncode=="140423" & year==2001

replace regioncode="140424" if regioncode=="140424" & year==2001

replace regioncode="140425" if regioncode=="140425" & year==2001

replace regioncode="140426" if regioncode=="140426" & year==2001

replace regioncode="140427" if regioncode=="140427" & year==2001

replace regioncode="140428" if regioncode=="140428" & year==2001

replace regioncode="140429" if regioncode=="140429" & year==2001

replace regioncode="140430" if regioncode=="140430" & year==2001

replace regioncode="140431" if regioncode=="140431" & year==2001

replace regioncode="140481" if regioncode=="140481" & year==2001

replace regioncode="140502" if regioncode=="140502" & year==2001

replace regioncode="140521" if regioncode=="140521" & year==2001

replace regioncode="140522" if regioncode=="140522" & year==2001

replace regioncode="140524" if regioncode=="140524" & year==2001

replace regioncode="140525" if regioncode=="140525" & year==2001

replace regioncode="140581" if regioncode=="140581" & year==2001

replace regioncode="140602" if regioncode=="140602" & year==2001

replace regioncode="140603" if regioncode=="140603" & year==2001

replace regioncode="140621" if regioncode=="140621" & year==2001

replace regioncode="140622" if regioncode=="140622" & year==2001

replace regioncode="140623" if regioncode=="140623" & year==2001

replace regioncode="140624" if regioncode=="140624" & year==2001

replace regioncode="140902" if regioncode=="140902" & year==2001

replace regioncode="140981" if regioncode=="140981" & year==2001

replace regioncode="140921" if regioncode=="140921" & year==2001

replace regioncode="140922" if regioncode=="140922" & year==2001

replace regioncode="140923" if regioncode=="140923" & year==2001

replace regioncode="140924" if regioncode=="140924" & year==2001

replace regioncode="140925" if regioncode=="140925" & year==2001

replace regioncode="140926" if regioncode=="140926" & year==2001

replace regioncode="140927" if regioncode=="140927" & year==2001

replace regioncode="140928" if regioncode=="140928" & year==2001

replace regioncode="140929" if regioncode=="140929" & year==2001

replace regioncode="140930" if regioncode=="140930" & year==2001

replace regioncode="140931" if regioncode=="140931" & year==2001

replace regioncode="140932" if regioncode=="140932" & year==2001

replace regioncode="141181" if regioncode=="142301" & year==2001

replace regioncode="141102" if regioncode=="142302" & year==2001

replace regioncode="141182" if regioncode=="142303" & year==2001

replace regioncode="141121" if regioncode=="142322" & year==2001

replace regioncode="141122" if regioncode=="142323" & year==2001

replace regioncode="141123" if regioncode=="142325" & year==2001

replace regioncode="141124" if regioncode=="142326" & year==2001

replace regioncode="141125" if regioncode=="142327" & year==2001

replace regioncode="141126" if regioncode=="142328" & year==2001

replace regioncode="141127" if regioncode=="142329" & year==2001

replace regioncode="141128" if regioncode=="142330" & year==2001

replace regioncode="141129" if regioncode=="142332" & year==2001

replace regioncode="141130" if regioncode=="142333" & year==2001

replace regioncode="140702" if regioncode=="140702" & year==2001

replace regioncode="140781" if regioncode=="140781" & year==2001

replace regioncode="140721" if regioncode=="140721" & year==2001

replace regioncode="140722" if regioncode=="140722" & year==2001

replace regioncode="140723" if regioncode=="140723" & year==2001

replace regioncode="140724" if regioncode=="140724" & year==2001

replace regioncode="140725" if regioncode=="140725" & year==2001

replace regioncode="140726" if regioncode=="140726" & year==2001

replace regioncode="140727" if regioncode=="140727" & year==2001

replace regioncode="140728" if regioncode=="140728" & year==2001

replace regioncode="140729" if regioncode=="140729" & year==2001

replace regioncode="141002" if regioncode=="141002" & year==2001

replace regioncode="141081" if regioncode=="141081" & year==2001

replace regioncode="141082" if regioncode=="141082" & year==2001

replace regioncode="141021" if regioncode=="141021" & year==2001

replace regioncode="141022" if regioncode=="141022" & year==2001

replace regioncode="141023" if regioncode=="141023" & year==2001

replace regioncode="141024" if regioncode=="141024" & year==2001

replace regioncode="141025" if regioncode=="141025" & year==2001

replace regioncode="141026" if regioncode=="141026" & year==2001

replace regioncode="141027" if regioncode=="141027" & year==2001

replace regioncode="141028" if regioncode=="141028" & year==2001

replace regioncode="141029" if regioncode=="141029" & year==2001

replace regioncode="141033" if regioncode=="141033" & year==2001

replace regioncode="141030" if regioncode=="141030" & year==2001

replace regioncode="141032" if regioncode=="141032" & year==2001

replace regioncode="141031" if regioncode=="141031" & year==2001

replace regioncode="141034" if regioncode=="141034" & year==2001

replace regioncode="140802" if regioncode=="140802" & year==2001

replace regioncode="140881" if regioncode=="140881" & year==2001

replace regioncode="140882" if regioncode=="140882" & year==2001

replace regioncode="140830" if regioncode=="140830" & year==2001

replace regioncode="140821" if regioncode=="140821" & year==2001

replace regioncode="140822" if regioncode=="140822" & year==2001

replace regioncode="140825" if regioncode=="140825" & year==2001

replace regioncode="140824" if regioncode=="140824" & year==2001

replace regioncode="140823" if regioncode=="140823" & year==2001

replace regioncode="140828" if regioncode=="140828" & year==2001

replace regioncode="140826" if regioncode=="140826" & year==2001

replace regioncode="140829" if regioncode=="140829" & year==2001

replace regioncode="140827" if regioncode=="140827" & year==2001

replace regioncode="150102" if regioncode=="150102" & year==2001

replace regioncode="150103" if regioncode=="150103" & year==2001

replace regioncode="150104" if regioncode=="150104" & year==2001

replace regioncode="150105" if regioncode=="150105" & year==2001

replace regioncode="150121" if regioncode=="150121" & year==2001

replace regioncode="150122" if regioncode=="150122" & year==2001

replace regioncode="150123" if regioncode=="150123" & year==2001

replace regioncode="150124" if regioncode=="150124" & year==2001

replace regioncode="150125" if regioncode=="150125" & year==2001

replace regioncode="150202" if regioncode=="150202" & year==2001

replace regioncode="150203" if regioncode=="150203" & year==2001

replace regioncode="150204" if regioncode=="150204" & year==2001

replace regioncode="150205" if regioncode=="150205" & year==2001

replace regioncode="150206" if regioncode=="150206" & year==2001

replace regioncode="150207" if regioncode=="150207" & year==2001

replace regioncode="150221" if regioncode=="150221" & year==2001

replace regioncode="150222" if regioncode=="150222" & year==2001

replace regioncode="150223" if regioncode=="150223" & year==2001

replace regioncode="150302" if regioncode=="150302" & year==2001

replace regioncode="150303" if regioncode=="150303" & year==2001

replace regioncode="150304" if regioncode=="150304" & year==2001

replace regioncode="150402" if regioncode=="150402" & year==2001

replace regioncode="150403" if regioncode=="150403" & year==2001

replace regioncode="150404" if regioncode=="150404" & year==2001

replace regioncode="150421" if regioncode=="150421" & year==2001

replace regioncode="150422" if regioncode=="150422" & year==2001

replace regioncode="150423" if regioncode=="150423" & year==2001

replace regioncode="150424" if regioncode=="150424" & year==2001

replace regioncode="150425" if regioncode=="150425" & year==2001

replace regioncode="150426" if regioncode=="150426" & year==2001

replace regioncode="150428" if regioncode=="150428" & year==2001

replace regioncode="150429" if regioncode=="150429" & year==2001

replace regioncode="150430" if regioncode=="150430" & year==2001

replace regioncode="150702" if regioncode=="150702" & year==2001

replace regioncode="150781" if regioncode=="150781" & year==2001

replace regioncode="150783" if regioncode=="150783" & year==2001

replace regioncode="150782" if regioncode=="150782" & year==2001

replace regioncode="150785" if regioncode=="150785" & year==2001

replace regioncode="150784" if regioncode=="150784" & year==2001

replace regioncode="150721" if regioncode=="150721" & year==2001

replace regioncode="150722" if regioncode=="150722" & year==2001

replace regioncode="150723" if regioncode=="150723" & year==2001

replace regioncode="150724" if regioncode=="150724" & year==2001

replace regioncode="150727" if regioncode=="150727" & year==2001

replace regioncode="150726" if regioncode=="150726" & year==2001

replace regioncode="150725" if regioncode=="150725" & year==2001

replace regioncode="152201" if regioncode=="152201" & year==2001

replace regioncode="152202" if regioncode=="152202" & year==2001

replace regioncode="152221" if regioncode=="152221" & year==2001

replace regioncode="152222" if regioncode=="152222" & year==2001

replace regioncode="152223" if regioncode=="152223" & year==2001

replace regioncode="152224" if regioncode=="152224" & year==2001

replace regioncode="150502" if regioncode=="150502" & year==2001

replace regioncode="150581" if regioncode=="150581" & year==2001

replace regioncode="150521" if regioncode=="150521" & year==2001

replace regioncode="150522" if regioncode=="150522" & year==2001

replace regioncode="150523" if regioncode=="150523" & year==2001

replace regioncode="150524" if regioncode=="150524" & year==2001

replace regioncode="150525" if regioncode=="150525" & year==2001

replace regioncode="150526" if regioncode=="150526" & year==2001

replace regioncode="152501" if regioncode=="152501" & year==2001

replace regioncode="152502" if regioncode=="152502" & year==2001

replace regioncode="152522" if regioncode=="152522" & year==2001

replace regioncode="152523" if regioncode=="152523" & year==2001

replace regioncode="152524" if regioncode=="152524" & year==2001

replace regioncode="152525" if regioncode=="152525" & year==2001

replace regioncode="152526" if regioncode=="152526" & year==2001

replace regioncode="152527" if regioncode=="152527" & year==2001

replace regioncode="152528" if regioncode=="152528" & year==2001

replace regioncode="152529" if regioncode=="152529" & year==2001

replace regioncode="152530" if regioncode=="152530" & year==2001

replace regioncode="152531" if regioncode=="152531" & year==2001

replace regioncode="152601" if regioncode=="152601" & year==2001

replace regioncode="152602" if regioncode=="152602" & year==2001

replace regioncode="152624" if regioncode=="152624" & year==2001

replace regioncode="152625" if regioncode=="152625" & year==2001

replace regioncode="152626" if regioncode=="152626" & year==2001

replace regioncode="152627" if regioncode=="152627" & year==2001

replace regioncode="152629" if regioncode=="152629" & year==2001

replace regioncode="152630" if regioncode=="152630" & year==2001

replace regioncode="152631" if regioncode=="152631" & year==2001

replace regioncode="152632" if regioncode=="152632" & year==2001

replace regioncode="152634" if regioncode=="152634" & year==2001

replace regioncode="150602" if regioncode=="150602" & year==2001

replace regioncode="150621" if regioncode=="150621" & year==2001

replace regioncode="150622" if regioncode=="150622" & year==2001

replace regioncode="150623" if regioncode=="150623" & year==2001

replace regioncode="150624" if regioncode=="150624" & year==2001

replace regioncode="150625" if regioncode=="150625" & year==2001

replace regioncode="150626" if regioncode=="150626" & year==2001

replace regioncode="150627" if regioncode=="150627" & year==2001

replace regioncode="152801" if regioncode=="152801" & year==2001

replace regioncode="152822" if regioncode=="152822" & year==2001

replace regioncode="152823" if regioncode=="152823" & year==2001

replace regioncode="152824" if regioncode=="152824" & year==2001

replace regioncode="152825" if regioncode=="152825" & year==2001

replace regioncode="152826" if regioncode=="152826" & year==2001

replace regioncode="152827" if regioncode=="152827" & year==2001

replace regioncode="152921" if regioncode=="152921" & year==2001

replace regioncode="152922" if regioncode=="152922" & year==2001

replace regioncode="152923" if regioncode=="152923" & year==2001

replace regioncode="210102" if regioncode=="210102" & year==2001

replace regioncode="210103" if regioncode=="210103" & year==2001

replace regioncode="210104" if regioncode=="210104" & year==2001

replace regioncode="210105" if regioncode=="210105" & year==2001

replace regioncode="210106" if regioncode=="210106" & year==2001

replace regioncode="210111" if regioncode=="210111" & year==2001

replace regioncode="210112" if regioncode=="210112" & year==2001

replace regioncode="210113" if regioncode=="210113" & year==2001

replace regioncode="210114" if regioncode=="210114" & year==2001

replace regioncode="210122" if regioncode=="210122" & year==2001

replace regioncode="210123" if regioncode=="210123" & year==2001

replace regioncode="210124" if regioncode=="210124" & year==2001

replace regioncode="210181" if regioncode=="210181" & year==2001

replace regioncode="210202" if regioncode=="210202" & year==2001

replace regioncode="210203" if regioncode=="210203" & year==2001

replace regioncode="210204" if regioncode=="210204" & year==2001

replace regioncode="210211" if regioncode=="210211" & year==2001

replace regioncode="210212" if regioncode=="210212" & year==2001

replace regioncode="210213" if regioncode=="210213" & year==2001

replace regioncode="210224" if regioncode=="210224" & year==2001

replace regioncode="210281" if regioncode=="210281" & year==2001

replace regioncode="210282" if regioncode=="210282" & year==2001

replace regioncode="210283" if regioncode=="210283" & year==2001

replace regioncode="210302" if regioncode=="210302" & year==2001

replace regioncode="210303" if regioncode=="210303" & year==2001

replace regioncode="210304" if regioncode=="210304" & year==2001

replace regioncode="210311" if regioncode=="210311" & year==2001

replace regioncode="210321" if regioncode=="210321" & year==2001

replace regioncode="210323" if regioncode=="210323" & year==2001

replace regioncode="210381" if regioncode=="210381" & year==2001

replace regioncode="210402" if regioncode=="210402" & year==2001

replace regioncode="210403" if regioncode=="210403" & year==2001

replace regioncode="210404" if regioncode=="210404" & year==2001

replace regioncode="210411" if regioncode=="210411" & year==2001

replace regioncode="210421" if regioncode=="210421" & year==2001

replace regioncode="210422" if regioncode=="210422" & year==2001

replace regioncode="210423" if regioncode=="210423" & year==2001

replace regioncode="210502" if regioncode=="210502" & year==2001

replace regioncode="210503" if regioncode=="210503" & year==2001

replace regioncode="210504" if regioncode=="210504" & year==2001

replace regioncode="210505" if regioncode=="210505" & year==2001

replace regioncode="210521" if regioncode=="210521" & year==2001

replace regioncode="210522" if regioncode=="210522" & year==2001

replace regioncode="210602" if regioncode=="210602" & year==2001

replace regioncode="210603" if regioncode=="210603" & year==2001

replace regioncode="210604" if regioncode=="210604" & year==2001

replace regioncode="210624" if regioncode=="210624" & year==2001

replace regioncode="210681" if regioncode=="210681" & year==2001

replace regioncode="210682" if regioncode=="210682" & year==2001

replace regioncode="210702" if regioncode=="210702" & year==2001

replace regioncode="210703" if regioncode=="210703" & year==2001

replace regioncode="210711" if regioncode=="210711" & year==2001

replace regioncode="210726" if regioncode=="210726" & year==2001

replace regioncode="210727" if regioncode=="210727" & year==2001

replace regioncode="210781" if regioncode=="210781" & year==2001

replace regioncode="210782" if regioncode=="210782" & year==2001

replace regioncode="210802" if regioncode=="210802" & year==2001

replace regioncode="210803" if regioncode=="210803" & year==2001

replace regioncode="210804" if regioncode=="210804" & year==2001

replace regioncode="210811" if regioncode=="210811" & year==2001

replace regioncode="210881" if regioncode=="210881" & year==2001

replace regioncode="210882" if regioncode=="210882" & year==2001

replace regioncode="210902" if regioncode=="210902" & year==2001

replace regioncode="210903" if regioncode=="210903" & year==2001

replace regioncode="210904" if regioncode=="210904" & year==2001

replace regioncode="210905" if regioncode=="210905" & year==2001

replace regioncode="210911" if regioncode=="210911" & year==2001

replace regioncode="210921" if regioncode=="210921" & year==2001

replace regioncode="210922" if regioncode=="210922" & year==2001

replace regioncode="211002" if regioncode=="211002" & year==2001

replace regioncode="211003" if regioncode=="211003" & year==2001

replace regioncode="211004" if regioncode=="211004" & year==2001

replace regioncode="211005" if regioncode=="211005" & year==2001

replace regioncode="211011" if regioncode=="211011" & year==2001

replace regioncode="211021" if regioncode=="211021" & year==2001

replace regioncode="211081" if regioncode=="211081" & year==2001

replace regioncode="211102" if regioncode=="211102" & year==2001

replace regioncode="211103" if regioncode=="211103" & year==2001

replace regioncode="211121" if regioncode=="211121" & year==2001

replace regioncode="211122" if regioncode=="211122" & year==2001

replace regioncode="211202" if regioncode=="211202" & year==2001

replace regioncode="211204" if regioncode=="211204" & year==2001

replace regioncode="211221" if regioncode=="211221" & year==2001

replace regioncode="211223" if regioncode=="211223" & year==2001

replace regioncode="211224" if regioncode=="211224" & year==2001

replace regioncode="211281" if regioncode=="211281" & year==2001

replace regioncode="211282" if regioncode=="211282" & year==2001

replace regioncode="211302" if regioncode=="211302" & year==2001

replace regioncode="211303" if regioncode=="211303" & year==2001

replace regioncode="211321" if regioncode=="211321" & year==2001

replace regioncode="211322" if regioncode=="211322" & year==2001

replace regioncode="211324" if regioncode=="211324" & year==2001

replace regioncode="211381" if regioncode=="211381" & year==2001

replace regioncode="211382" if regioncode=="211382" & year==2001

replace regioncode="211402" if regioncode=="211402" & year==2001

replace regioncode="211403" if regioncode=="211403" & year==2001

replace regioncode="211404" if regioncode=="211404" & year==2001

replace regioncode="211421" if regioncode=="211421" & year==2001

replace regioncode="211422" if regioncode=="211422" & year==2001

replace regioncode="211481" if regioncode=="211481" & year==2001

replace regioncode="220102" if regioncode=="220102" & year==2001

replace regioncode="220103" if regioncode=="220103" & year==2001

replace regioncode="220104" if regioncode=="220104" & year==2001

replace regioncode="220105" if regioncode=="220105" & year==2001

replace regioncode="220106" if regioncode=="220106" & year==2001

replace regioncode="220112" if regioncode=="220112" & year==2001

replace regioncode="220122" if regioncode=="220122" & year==2001

replace regioncode="220181" if regioncode=="220181" & year==2001

replace regioncode="220182" if regioncode=="220182" & year==2001

replace regioncode="220183" if regioncode=="220183" & year==2001

replace regioncode="220202" if regioncode=="220202" & year==2001

replace regioncode="220203" if regioncode=="220203" & year==2001

replace regioncode="220204" if regioncode=="220204" & year==2001

replace regioncode="220211" if regioncode=="220211" & year==2001

replace regioncode="220221" if regioncode=="220221" & year==2001

replace regioncode="220281" if regioncode=="220281" & year==2001

replace regioncode="220282" if regioncode=="220282" & year==2001

replace regioncode="220283" if regioncode=="220283" & year==2001

replace regioncode="220284" if regioncode=="220284" & year==2001

replace regioncode="220302" if regioncode=="220302" & year==2001

replace regioncode="220303" if regioncode=="220303" & year==2001

replace regioncode="220322" if regioncode=="220322" & year==2001

replace regioncode="220323" if regioncode=="220323" & year==2001

replace regioncode="220381" if regioncode=="220381" & year==2001

replace regioncode="220382" if regioncode=="220382" & year==2001

replace regioncode="220402" if regioncode=="220402" & year==2001

replace regioncode="220403" if regioncode=="220403" & year==2001

replace regioncode="220421" if regioncode=="220421" & year==2001

replace regioncode="220422" if regioncode=="220422" & year==2001

replace regioncode="220502" if regioncode=="220502" & year==2001

replace regioncode="220503" if regioncode=="220503" & year==2001

replace regioncode="220521" if regioncode=="220521" & year==2001

replace regioncode="220523" if regioncode=="220523" & year==2001

replace regioncode="220524" if regioncode=="220524" & year==2001

replace regioncode="220581" if regioncode=="220581" & year==2001

replace regioncode="220582" if regioncode=="220582" & year==2001

replace regioncode="220602" if regioncode=="220602" & year==2001

replace regioncode="220621" if regioncode=="220621" & year==2001

replace regioncode="220622" if regioncode=="220622" & year==2001

replace regioncode="220623" if regioncode=="220623" & year==2001

replace regioncode="220625" if regioncode=="220625" & year==2001

replace regioncode="220681" if regioncode=="220681" & year==2001

replace regioncode="220702" if regioncode=="220702" & year==2001

replace regioncode="220721" if regioncode=="220721" & year==2001

replace regioncode="220722" if regioncode=="220722" & year==2001

replace regioncode="220723" if regioncode=="220723" & year==2001

replace regioncode="220724" if regioncode=="220724" & year==2001

replace regioncode="220802" if regioncode=="220802" & year==2001

replace regioncode="220821" if regioncode=="220821" & year==2001

replace regioncode="220822" if regioncode=="220822" & year==2001

replace regioncode="220881" if regioncode=="220881" & year==2001

replace regioncode="220882" if regioncode=="220882" & year==2001

replace regioncode="222401" if regioncode=="222401" & year==2001

replace regioncode="222402" if regioncode=="222402" & year==2001

replace regioncode="222403" if regioncode=="222403" & year==2001

replace regioncode="222404" if regioncode=="222404" & year==2001

replace regioncode="222405" if regioncode=="222405" & year==2001

replace regioncode="222406" if regioncode=="222406" & year==2001

replace regioncode="222424" if regioncode=="222424" & year==2001

replace regioncode="222426" if regioncode=="222426" & year==2001

replace regioncode="230102" if regioncode=="230102" & year==2001

replace regioncode="230103" if regioncode=="230103" & year==2001

replace regioncode="230104" if regioncode=="230104" & year==2001

replace regioncode="230105" if regioncode=="230105" & year==2001

replace regioncode="230107" if regioncode=="230107" & year==2001

replace regioncode="230108" if regioncode=="230108" & year==2001

replace regioncode="230121" if regioncode=="230121" & year==2001

replace regioncode="230123" if regioncode=="230123" & year==2001

replace regioncode="230124" if regioncode=="230124" & year==2001

replace regioncode="230125" if regioncode=="230125" & year==2001

replace regioncode="230126" if regioncode=="230126" & year==2001

replace regioncode="230127" if regioncode=="230127" & year==2001

replace regioncode="230128" if regioncode=="230128" & year==2001

replace regioncode="230129" if regioncode=="230129" & year==2001

replace regioncode="230181" if regioncode=="230181" & year==2001

replace regioncode="230182" if regioncode=="230182" & year==2001

replace regioncode="230183" if regioncode=="230183" & year==2001

replace regioncode="230184" if regioncode=="230184" & year==2001

replace regioncode="230202" if regioncode=="230202" & year==2001

replace regioncode="230203" if regioncode=="230203" & year==2001

replace regioncode="230204" if regioncode=="230204" & year==2001

replace regioncode="230205" if regioncode=="230205" & year==2001

replace regioncode="230206" if regioncode=="230206" & year==2001

replace regioncode="230207" if regioncode=="230207" & year==2001

replace regioncode="230208" if regioncode=="230208" & year==2001

replace regioncode="230221" if regioncode=="230221" & year==2001

replace regioncode="230223" if regioncode=="230223" & year==2001

replace regioncode="230224" if regioncode=="230224" & year==2001

replace regioncode="230225" if regioncode=="230225" & year==2001

replace regioncode="230227" if regioncode=="230227" & year==2001

replace regioncode="230229" if regioncode=="230229" & year==2001

replace regioncode="230230" if regioncode=="230230" & year==2001

replace regioncode="230231" if regioncode=="230231" & year==2001

replace regioncode="230281" if regioncode=="230281" & year==2001

replace regioncode="230302" if regioncode=="230302" & year==2001

replace regioncode="230303" if regioncode=="230303" & year==2001

replace regioncode="230304" if regioncode=="230304" & year==2001

replace regioncode="230305" if regioncode=="230305" & year==2001

replace regioncode="230306" if regioncode=="230306" & year==2001

replace regioncode="230307" if regioncode=="230307" & year==2001

replace regioncode="230321" if regioncode=="230321" & year==2001

replace regioncode="230381" if regioncode=="230381" & year==2001

replace regioncode="230382" if regioncode=="230382" & year==2001

replace regioncode="230402" if regioncode=="230402" & year==2001

replace regioncode="230403" if regioncode=="230403" & year==2001

replace regioncode="230404" if regioncode=="230404" & year==2001

replace regioncode="230405" if regioncode=="230405" & year==2001

replace regioncode="230406" if regioncode=="230406" & year==2001

replace regioncode="230407" if regioncode=="230407" & year==2001

replace regioncode="230421" if regioncode=="230421" & year==2001

replace regioncode="230422" if regioncode=="230422" & year==2001

replace regioncode="230502" if regioncode=="230502" & year==2001

replace regioncode="230503" if regioncode=="230503" & year==2001

replace regioncode="230505" if regioncode=="230505" & year==2001

replace regioncode="230506" if regioncode=="230506" & year==2001

replace regioncode="230521" if regioncode=="230521" & year==2001

replace regioncode="230522" if regioncode=="230522" & year==2001

replace regioncode="230523" if regioncode=="230523" & year==2001

replace regioncode="230524" if regioncode=="230524" & year==2001

replace regioncode="230602" if regioncode=="230602" & year==2001

replace regioncode="230603" if regioncode=="230603" & year==2001

replace regioncode="230604" if regioncode=="230604" & year==2001

replace regioncode="230605" if regioncode=="230605" & year==2001

replace regioncode="230606" if regioncode=="230606" & year==2001

replace regioncode="230621" if regioncode=="230621" & year==2001

replace regioncode="230622" if regioncode=="230622" & year==2001

replace regioncode="230623" if regioncode=="230623" & year==2001

replace regioncode="230624" if regioncode=="230624" & year==2001

replace regioncode="230702" if regioncode=="230702" & year==2001

replace regioncode="230703" if regioncode=="230703" & year==2001

replace regioncode="230704" if regioncode=="230704" & year==2001

replace regioncode="230705" if regioncode=="230705" & year==2001

replace regioncode="230706" if regioncode=="230706" & year==2001

replace regioncode="230707" if regioncode=="230707" & year==2001

replace regioncode="230708" if regioncode=="230708" & year==2001

replace regioncode="230709" if regioncode=="230709" & year==2001

replace regioncode="230710" if regioncode=="230710" & year==2001

replace regioncode="230711" if regioncode=="230711" & year==2001

replace regioncode="230712" if regioncode=="230712" & year==2001

replace regioncode="230713" if regioncode=="230713" & year==2001

replace regioncode="230714" if regioncode=="230714" & year==2001

replace regioncode="230715" if regioncode=="230715" & year==2001

replace regioncode="230716" if regioncode=="230716" & year==2001

replace regioncode="230722" if regioncode=="230722" & year==2001

replace regioncode="230781" if regioncode=="230781" & year==2001

replace regioncode="230802" if regioncode=="230802" & year==2001

replace regioncode="230803" if regioncode=="230803" & year==2001

replace regioncode="230804" if regioncode=="230804" & year==2001

replace regioncode="230805" if regioncode=="230805" & year==2001

replace regioncode="230811" if regioncode=="230811" & year==2001

replace regioncode="230822" if regioncode=="230822" & year==2001

replace regioncode="230826" if regioncode=="230826" & year==2001

replace regioncode="230828" if regioncode=="230828" & year==2001

replace regioncode="230833" if regioncode=="230833" & year==2001

replace regioncode="230881" if regioncode=="230881" & year==2001

replace regioncode="230882" if regioncode=="230882" & year==2001

replace regioncode="230902" if regioncode=="230902" & year==2001

replace regioncode="230903" if regioncode=="230903" & year==2001

replace regioncode="230904" if regioncode=="230904" & year==2001

replace regioncode="230921" if regioncode=="230921" & year==2001

replace regioncode="231002" if regioncode=="231002" & year==2001

replace regioncode="231003" if regioncode=="231003" & year==2001

replace regioncode="231004" if regioncode=="231004" & year==2001

replace regioncode="231005" if regioncode=="231005" & year==2001

replace regioncode="231024" if regioncode=="231024" & year==2001

replace regioncode="231025" if regioncode=="231025" & year==2001

replace regioncode="231081" if regioncode=="231081" & year==2001

replace regioncode="231083" if regioncode=="231083" & year==2001

replace regioncode="231084" if regioncode=="231084" & year==2001

replace regioncode="231085" if regioncode=="231085" & year==2001

replace regioncode="231102" if regioncode=="231102" & year==2001

replace regioncode="231121" if regioncode=="231121" & year==2001

replace regioncode="231123" if regioncode=="231123" & year==2001

replace regioncode="231124" if regioncode=="231124" & year==2001

replace regioncode="231181" if regioncode=="231181" & year==2001

replace regioncode="231182" if regioncode=="231182" & year==2001

replace regioncode="231202" if regioncode=="231202" & year==2001

replace regioncode="231221" if regioncode=="231221" & year==2001

replace regioncode="231222" if regioncode=="231222" & year==2001

replace regioncode="231223" if regioncode=="231223" & year==2001

replace regioncode="231224" if regioncode=="231224" & year==2001

replace regioncode="231225" if regioncode=="231225" & year==2001

replace regioncode="231226" if regioncode=="231226" & year==2001

replace regioncode="231281" if regioncode=="231281" & year==2001

replace regioncode="231282" if regioncode=="231282" & year==2001

replace regioncode="231283" if regioncode=="231283" & year==2001

replace regioncode="232701" if regioncode=="232701" & year==2001

replace regioncode="232702" if regioncode=="232702" & year==2001

replace regioncode="232703" if regioncode=="232703" & year==2001

replace regioncode="232704" if regioncode=="232704" & year==2001

replace regioncode="232721" if regioncode=="232721" & year==2001

replace regioncode="232722" if regioncode=="232722" & year==2001

replace regioncode="232723" if regioncode=="232723" & year==2001

replace regioncode="310101" if regioncode=="310101" & year==2001

replace regioncode="310103" if regioncode=="310103" & year==2001

replace regioncode="310104" if regioncode=="310104" & year==2001

replace regioncode="310105" if regioncode=="310105" & year==2001

replace regioncode="310106" if regioncode=="310106" & year==2001

replace regioncode="310107" if regioncode=="310107" & year==2001

replace regioncode="310108" if regioncode=="310108" & year==2001

replace regioncode="310109" if regioncode=="310109" & year==2001

replace regioncode="310110" if regioncode=="310110" & year==2001

replace regioncode="310112" if regioncode=="310112" & year==2001

replace regioncode="310113" if regioncode=="310113" & year==2001

replace regioncode="310114" if regioncode=="310114" & year==2001

replace regioncode="310115" if regioncode=="310115" & year==2001

replace regioncode="310116" if regioncode=="310116" & year==2001

replace regioncode="310117" if regioncode=="310117" & year==2001

replace regioncode="310118" if regioncode=="310118" & year==2001

replace regioncode="310119" if regioncode=="310119" & year==2001

replace regioncode="310120" if regioncode=="310120" & year==2001

replace regioncode="310230" if regioncode=="310230" & year==2001

replace regioncode="320102" if regioncode=="320102" & year==2001

replace regioncode="320103" if regioncode=="320103" & year==2001

replace regioncode="320104" if regioncode=="320104" & year==2001

replace regioncode="320105" if regioncode=="320105" & year==2001

replace regioncode="320106" if regioncode=="320106" & year==2001

replace regioncode="320107" if regioncode=="320107" & year==2001

replace regioncode="320111" if regioncode=="320111" & year==2001

replace regioncode="320113" if regioncode=="320113" & year==2001

replace regioncode="320114" if regioncode=="320114" & year==2001

replace regioncode="320115" if regioncode=="320115" & year==2001

replace regioncode="320116" if regioncode=="320123" & year==2001

replace regioncode="320124" if regioncode=="320124" & year==2001

replace regioncode="320125" if regioncode=="320125" & year==2001

replace regioncode="320202" if regioncode=="320202" & year==2001

replace regioncode="320203" if regioncode=="320203" & year==2001

replace regioncode="320204" if regioncode=="320204" & year==2001

replace regioncode="320205" if regioncode=="320205" & year==2001

replace regioncode="320211" if regioncode=="320211" & year==2001

replace regioncode="320281" if regioncode=="320281" & year==2001

replace regioncode="320282" if regioncode=="320282" & year==2001

replace regioncode="320302" if regioncode=="320302" & year==2001

replace regioncode="320303" if regioncode=="320303" & year==2001

replace regioncode="320304" if regioncode=="320304" & year==2001

replace regioncode="320305" if regioncode=="320305" & year==2001

replace regioncode="320311" if regioncode=="320311" & year==2001

replace regioncode="320321" if regioncode=="320321" & year==2001

replace regioncode="320322" if regioncode=="320322" & year==2001

replace regioncode="320323" if regioncode=="320323" & year==2001

replace regioncode="320324" if regioncode=="320324" & year==2001

replace regioncode="320381" if regioncode=="320381" & year==2001

replace regioncode="320382" if regioncode=="320382" & year==2001

replace regioncode="320402" if regioncode=="320402" & year==2001

replace regioncode="320404" if regioncode=="320404" & year==2001

replace regioncode="320405" if regioncode=="320405" & year==2001

replace regioncode="320411" if regioncode=="320411" & year==2001

replace regioncode="320412" if regioncode=="320483" & year==2001

replace regioncode="320481" if regioncode=="320481" & year==2001

replace regioncode="320482" if regioncode=="320482" & year==2001

replace regioncode="320502" if regioncode=="320502" & year==2001

replace regioncode="320503" if regioncode=="320503" & year==2001

replace regioncode="320504" if regioncode=="320504" & year==2001

replace regioncode="320505" if regioncode=="320505" & year==2001

replace regioncode="320581" if regioncode=="320581" & year==2001

replace regioncode="320582" if regioncode=="320582" & year==2001

replace regioncode="320583" if regioncode=="320583" & year==2001

replace regioncode="320584" if regioncode=="320584" & year==2001

replace regioncode="320585" if regioncode=="320585" & year==2001

replace regioncode="320586" if regioncode=="320586" & year==2001

replace regioncode="320602" if regioncode=="320602" & year==2001

replace regioncode="320611" if regioncode=="320611" & year==2001

replace regioncode="320621" if regioncode=="320621" & year==2001

replace regioncode="320623" if regioncode=="320623" & year==2001

replace regioncode="320681" if regioncode=="320681" & year==2001

replace regioncode="320682" if regioncode=="320682" & year==2001

replace regioncode="320683" if regioncode=="320683" & year==2001

replace regioncode="320684" if regioncode=="320684" & year==2001

replace regioncode="320703" if regioncode=="320703" & year==2001

replace regioncode="320705" if regioncode=="320705" & year==2001

replace regioncode="320706" if regioncode=="320706" & year==2001

replace regioncode="320721" if regioncode=="320721" & year==2001

replace regioncode="320722" if regioncode=="320722" & year==2001

replace regioncode="320723" if regioncode=="320723" & year==2001

replace regioncode="320724" if regioncode=="320724" & year==2001

replace regioncode="320802" if regioncode=="320802" & year==2001

replace regioncode="320803" if regioncode=="320803" & year==2001

replace regioncode="320804" if regioncode=="320804" & year==2001

replace regioncode="320811" if regioncode=="320811" & year==2001

replace regioncode="320826" if regioncode=="320826" & year==2001

replace regioncode="320829" if regioncode=="320829" & year==2001

replace regioncode="320830" if regioncode=="320830" & year==2001

replace regioncode="320831" if regioncode=="320831" & year==2001

replace regioncode="320902" if regioncode=="320902" & year==2001

replace regioncode="320903" if regioncode=="320928" & year==2001

replace regioncode="320921" if regioncode=="320921" & year==2001

replace regioncode="320922" if regioncode=="320922" & year==2001

replace regioncode="320923" if regioncode=="320923" & year==2001

replace regioncode="320924" if regioncode=="320924" & year==2001

replace regioncode="320925" if regioncode=="320925" & year==2001

replace regioncode="320981" if regioncode=="320981" & year==2001

replace regioncode="320982" if regioncode=="320982" & year==2001

replace regioncode="321002" if regioncode=="321002" & year==2001

replace regioncode="321003" if regioncode=="321003" & year==2001

replace regioncode="321011" if regioncode=="321011" & year==2001

replace regioncode="321023" if regioncode=="321023" & year==2001

replace regioncode="321081" if regioncode=="321081" & year==2001

replace regioncode="321084" if regioncode=="321084" & year==2001

replace regioncode="321088" if regioncode=="321088" & year==2001

replace regioncode="321102" if regioncode=="321102" & year==2001

replace regioncode="321111" if regioncode=="321111" & year==2001

replace regioncode="321112" if regioncode=="321121" & year==2001

replace regioncode="321181" if regioncode=="321181" & year==2001

replace regioncode="321182" if regioncode=="321182" & year==2001

replace regioncode="321183" if regioncode=="321183" & year==2001

replace regioncode="321202" if regioncode=="321202" & year==2001

replace regioncode="321203" if regioncode=="321203" & year==2001

replace regioncode="321281" if regioncode=="321281" & year==2001

replace regioncode="321282" if regioncode=="321282" & year==2001

replace regioncode="321283" if regioncode=="321283" & year==2001

replace regioncode="321284" if regioncode=="321284" & year==2001

replace regioncode="321302" if regioncode=="321302" & year==2001

replace regioncode="321321" if regioncode=="321321" & year==2001

replace regioncode="321322" if regioncode=="321322" & year==2001

replace regioncode="321323" if regioncode=="321323" & year==2001

replace regioncode="321324" if regioncode=="321324" & year==2001

replace regioncode="330102" if regioncode=="330102" & year==2001

replace regioncode="330103" if regioncode=="330103" & year==2001

replace regioncode="330104" if regioncode=="330104" & year==2001

replace regioncode="330105" if regioncode=="330105" & year==2001

replace regioncode="330106" if regioncode=="330106" & year==2001

replace regioncode="330108" if regioncode=="330108" & year==2001

replace regioncode="330109" if regioncode=="330109" & year==2001

replace regioncode="330110" if regioncode=="330110" & year==2001

replace regioncode="330122" if regioncode=="330122" & year==2001

replace regioncode="330127" if regioncode=="330127" & year==2001

replace regioncode="330182" if regioncode=="330182" & year==2001

replace regioncode="330183" if regioncode=="330183" & year==2001

replace regioncode="330185" if regioncode=="330185" & year==2001

replace regioncode="330203" if regioncode=="330203" & year==2001

replace regioncode="330204" if regioncode=="330204" & year==2001

replace regioncode="330205" if regioncode=="330205" & year==2001

replace regioncode="330206" if regioncode=="330206" & year==2001

replace regioncode="330211" if regioncode=="330211" & year==2001

replace regioncode="330212" if regioncode=="330227" & year==2001

replace regioncode="330225" if regioncode=="330225" & year==2001

replace regioncode="330226" if regioncode=="330226" & year==2001

replace regioncode="330281" if regioncode=="330281" & year==2001

replace regioncode="330282" if regioncode=="330282" & year==2001

replace regioncode="330283" if regioncode=="330283" & year==2001

replace regioncode="330302" if regioncode=="330302" & year==2001

replace regioncode="330303" if regioncode=="330303" & year==2001

replace regioncode="330304" if regioncode=="330304" & year==2001

replace regioncode="330322" if regioncode=="330322" & year==2001

replace regioncode="330324" if regioncode=="330324" & year==2001

replace regioncode="330326" if regioncode=="330326" & year==2001

replace regioncode="330327" if regioncode=="330327" & year==2001

replace regioncode="330328" if regioncode=="330328" & year==2001

replace regioncode="330329" if regioncode=="330329" & year==2001

replace regioncode="330381" if regioncode=="330381" & year==2001

replace regioncode="330382" if regioncode=="330382" & year==2001

replace regioncode="330402" if regioncode=="330402" & year==2001

replace regioncode="330411" if regioncode=="330411" & year==2001

replace regioncode="330421" if regioncode=="330421" & year==2001

replace regioncode="330424" if regioncode=="330424" & year==2001

replace regioncode="330481" if regioncode=="330481" & year==2001

replace regioncode="330482" if regioncode=="330482" & year==2001

replace regioncode="330483" if regioncode=="330483" & year==2001

replace regioncode="330501" if regioncode=="330501" & year==2001

replace regioncode="330521" if regioncode=="330521" & year==2001

replace regioncode="330522" if regioncode=="330522" & year==2001

replace regioncode="330523" if regioncode=="330523" & year==2001

replace regioncode="330602" if regioncode=="330602" & year==2001

replace regioncode="330621" if regioncode=="330621" & year==2001

replace regioncode="330624" if regioncode=="330624" & year==2001

replace regioncode="330681" if regioncode=="330681" & year==2001

replace regioncode="330682" if regioncode=="330682" & year==2001

replace regioncode="330683" if regioncode=="330683" & year==2001

replace regioncode="330702" if regioncode=="330702" & year==2001

replace regioncode="330703" if regioncode=="330703" & year==2001

replace regioncode="330723" if regioncode=="330723" & year==2001

replace regioncode="330726" if regioncode=="330726" & year==2001

replace regioncode="330727" if regioncode=="330727" & year==2001

replace regioncode="330781" if regioncode=="330781" & year==2001

replace regioncode="330782" if regioncode=="330782" & year==2001

replace regioncode="330783" if regioncode=="330783" & year==2001

replace regioncode="330784" if regioncode=="330784" & year==2001

replace regioncode="330802" if regioncode=="330802" & year==2001

replace regioncode="330803" if regioncode=="330821" & year==2001

replace regioncode="330822" if regioncode=="330822" & year==2001

replace regioncode="330824" if regioncode=="330824" & year==2001

replace regioncode="330825" if regioncode=="330825" & year==2001

replace regioncode="330881" if regioncode=="330881" & year==2001

replace regioncode="330902" if regioncode=="330902" & year==2001

replace regioncode="330903" if regioncode=="330903" & year==2001

replace regioncode="330921" if regioncode=="330921" & year==2001

replace regioncode="330922" if regioncode=="330922" & year==2001

replace regioncode="331002" if regioncode=="331002" & year==2001

replace regioncode="331003" if regioncode=="331003" & year==2001

replace regioncode="331004" if regioncode=="331004" & year==2001

replace regioncode="331021" if regioncode=="331021" & year==2001

replace regioncode="331022" if regioncode=="331022" & year==2001

replace regioncode="331023" if regioncode=="331023" & year==2001

replace regioncode="331024" if regioncode=="331024" & year==2001

replace regioncode="331081" if regioncode=="331081" & year==2001

replace regioncode="331082" if regioncode=="331082" & year==2001

replace regioncode="331102" if regioncode=="331102" & year==2001

replace regioncode="331121" if regioncode=="331121" & year==2001

replace regioncode="331122" if regioncode=="331122" & year==2001

replace regioncode="331123" if regioncode=="331123" & year==2001

replace regioncode="331124" if regioncode=="331124" & year==2001

replace regioncode="331125" if regioncode=="331125" & year==2001

replace regioncode="331126" if regioncode=="331126" & year==2001

replace regioncode="331127" if regioncode=="331127" & year==2001

replace regioncode="331181" if regioncode=="331181" & year==2001

replace regioncode="340102" if regioncode=="340102" & year==2001

replace regioncode="340103" if regioncode=="340103" & year==2001

replace regioncode="340104" if regioncode=="340104" & year==2001

replace regioncode="340111" if regioncode=="340111" & year==2001

replace regioncode="340121" if regioncode=="340121" & year==2001

replace regioncode="340122" if regioncode=="340122" & year==2001

replace regioncode="340123" if regioncode=="340123" & year==2001

replace regioncode="340202" if regioncode=="340202" & year==2001

replace regioncode="340203" if regioncode=="340203" & year==2001

replace regioncode="340204" if regioncode=="340204" & year==2001

replace regioncode="340207" if regioncode=="340207" & year==2001

replace regioncode="340221" if regioncode=="340221" & year==2001

replace regioncode="340222" if regioncode=="340222" & year==2001

replace regioncode="340223" if regioncode=="340223" & year==2001

replace regioncode="340302" if regioncode=="340302" & year==2001

replace regioncode="340303" if regioncode=="340303" & year==2001

replace regioncode="340304" if regioncode=="340304" & year==2001

replace regioncode="340311" if regioncode=="340311" & year==2001

replace regioncode="340321" if regioncode=="340321" & year==2001

replace regioncode="340322" if regioncode=="340322" & year==2001

replace regioncode="340323" if regioncode=="340323" & year==2001

replace regioncode="340402" if regioncode=="340402" & year==2001

replace regioncode="340403" if regioncode=="340403" & year==2001

replace regioncode="340404" if regioncode=="340404" & year==2001

replace regioncode="340405" if regioncode=="340405" & year==2001

replace regioncode="340406" if regioncode=="340406" & year==2001

replace regioncode="340421" if regioncode=="340421" & year==2001

replace regioncode="340502" if regioncode=="340502" & year==2001

replace regioncode="340503" if regioncode=="340503" & year==2001

replace regioncode="340504" if regioncode=="340504" & year==2001

replace regioncode="340521" if regioncode=="340521" & year==2001

replace regioncode="340602" if regioncode=="340602" & year==2001

replace regioncode="340603" if regioncode=="340603" & year==2001

replace regioncode="340604" if regioncode=="340604" & year==2001

replace regioncode="340621" if regioncode=="340621" & year==2001

replace regioncode="340702" if regioncode=="340702" & year==2001

replace regioncode="340703" if regioncode=="340703" & year==2001

replace regioncode="340711" if regioncode=="340711" & year==2001

replace regioncode="340721" if regioncode=="340721" & year==2001

replace regioncode="340802" if regioncode=="340802" & year==2001

replace regioncode="340803" if regioncode=="340803" & year==2001

replace regioncode="340811" if regioncode=="340811" & year==2001

replace regioncode="340822" if regioncode=="340822" & year==2001

replace regioncode="340823" if regioncode=="340823" & year==2001

replace regioncode="340824" if regioncode=="340824" & year==2001

replace regioncode="340825" if regioncode=="340825" & year==2001

replace regioncode="340826" if regioncode=="340826" & year==2001

replace regioncode="340827" if regioncode=="340827" & year==2001

replace regioncode="340828" if regioncode=="340828" & year==2001

replace regioncode="340881" if regioncode=="340881" & year==2001

replace regioncode="341002" if regioncode=="341002" & year==2001

replace regioncode="341003" if regioncode=="341003" & year==2001

replace regioncode="341004" if regioncode=="341004" & year==2001

replace regioncode="341021" if regioncode=="341021" & year==2001

replace regioncode="341022" if regioncode=="341022" & year==2001

replace regioncode="341023" if regioncode=="341023" & year==2001

replace regioncode="341024" if regioncode=="341024" & year==2001

replace regioncode="341102" if regioncode=="341102" & year==2001

replace regioncode="341103" if regioncode=="341103" & year==2001

replace regioncode="341122" if regioncode=="341122" & year==2001

replace regioncode="341124" if regioncode=="341124" & year==2001

replace regioncode="341125" if regioncode=="341125" & year==2001

replace regioncode="341126" if regioncode=="341126" & year==2001

replace regioncode="341181" if regioncode=="341181" & year==2001

replace regioncode="341182" if regioncode=="341182" & year==2001

replace regioncode="341202" if regioncode=="341202" & year==2001

replace regioncode="341203" if regioncode=="341203" & year==2001

replace regioncode="341204" if regioncode=="341204" & year==2001

replace regioncode="341221" if regioncode=="341221" & year==2001

replace regioncode="341222" if regioncode=="341222" & year==2001

replace regioncode="341621" if regioncode=="341621" & year==2001

replace regioncode="341622" if regioncode=="341622" & year==2001

replace regioncode="341225" if regioncode=="341225" & year==2001

replace regioncode="341226" if regioncode=="341226" & year==2001

replace regioncode="341623" if regioncode=="341623" & year==2001

replace regioncode="341282" if regioncode=="341282" & year==2001

replace regioncode="341302" if regioncode=="341302" & year==2001

replace regioncode="341321" if regioncode=="341321" & year==2001

replace regioncode="341322" if regioncode=="341322" & year==2001

replace regioncode="341323" if regioncode=="341323" & year==2001

replace regioncode="341324" if regioncode=="341324" & year==2001

replace regioncode="341402" if regioncode=="341402" & year==2001

replace regioncode="341421" if regioncode=="341421" & year==2001

replace regioncode="341422" if regioncode=="341422" & year==2001

replace regioncode="341423" if regioncode=="341423" & year==2001

replace regioncode="341424" if regioncode=="341424" & year==2001

replace regioncode="341521" if regioncode=="341521" & year==2001

replace regioncode="341522" if regioncode=="341522" & year==2001

replace regioncode="341523" if regioncode=="341523" & year==2001

replace regioncode="341524" if regioncode=="341524" & year==2001

replace regioncode="341525" if regioncode=="341525" & year==2001

replace regioncode="341802" if regioncode=="341802" & year==2001

replace regioncode="341881" if regioncode=="341881" & year==2001

replace regioncode="341821" if regioncode=="341821" & year==2001

replace regioncode="341822" if regioncode=="341822" & year==2001

replace regioncode="341823" if regioncode=="341823" & year==2001

replace regioncode="341825" if regioncode=="341825" & year==2001

replace regioncode="341824" if regioncode=="341824" & year==2001

replace regioncode="341702" if regioncode=="341702" & year==2001

replace regioncode="341721" if regioncode=="341721" & year==2001

replace regioncode="341722" if regioncode=="341722" & year==2001

replace regioncode="341723" if regioncode=="341723" & year==2001

replace regioncode="341502" if regioncode=="342401" & year==2001

replace regioncode="341602" if regioncode=="341602" & year==2001

replace regioncode="350102" if regioncode=="350102" & year==2001

replace regioncode="350103" if regioncode=="350103" & year==2001

replace regioncode="350104" if regioncode=="350104" & year==2001

replace regioncode="350105" if regioncode=="350105" & year==2001

replace regioncode="350111" if regioncode=="350111" & year==2001

replace regioncode="350121" if regioncode=="350121" & year==2001

replace regioncode="350122" if regioncode=="350122" & year==2001

replace regioncode="350123" if regioncode=="350123" & year==2001

replace regioncode="350124" if regioncode=="350124" & year==2001

replace regioncode="350125" if regioncode=="350125" & year==2001

replace regioncode="350128" if regioncode=="350128" & year==2001

replace regioncode="350181" if regioncode=="350181" & year==2001

replace regioncode="350182" if regioncode=="350182" & year==2001

replace regioncode="350203" if regioncode=="350203" & year==2001

replace regioncode="350205" if regioncode=="350205" & year==2001

replace regioncode="350206" if regioncode=="350206" & year==2001

replace regioncode="350211" if regioncode=="350211" & year==2001

replace regioncode="350212" if regioncode=="350212" & year==2001

replace regioncode="350213" if regioncode=="350213" & year==2001

replace regioncode="350302" if regioncode=="350302" & year==2001

replace regioncode="350303" if regioncode=="350303" & year==2001

replace regioncode="350321" if regioncode=="350321" & year==2001

replace regioncode="350322" if regioncode=="350322" & year==2001

replace regioncode="350402" if regioncode=="350402" & year==2001

replace regioncode="350403" if regioncode=="350403" & year==2001

replace regioncode="350421" if regioncode=="350421" & year==2001

replace regioncode="350423" if regioncode=="350423" & year==2001

replace regioncode="350424" if regioncode=="350424" & year==2001

replace regioncode="350425" if regioncode=="350425" & year==2001

replace regioncode="350426" if regioncode=="350426" & year==2001

replace regioncode="350427" if regioncode=="350427" & year==2001

replace regioncode="350428" if regioncode=="350428" & year==2001

replace regioncode="350429" if regioncode=="350429" & year==2001

replace regioncode="350430" if regioncode=="350430" & year==2001

replace regioncode="350481" if regioncode=="350481" & year==2001

replace regioncode="350502" if regioncode=="350502" & year==2001

replace regioncode="350503" if regioncode=="350503" & year==2001

replace regioncode="350504" if regioncode=="350504" & year==2001

replace regioncode="350505" if regioncode=="350505" & year==2001

replace regioncode="350521" if regioncode=="350521" & year==2001

replace regioncode="350524" if regioncode=="350524" & year==2001

replace regioncode="350525" if regioncode=="350525" & year==2001

replace regioncode="350526" if regioncode=="350526" & year==2001

replace regioncode="350581" if regioncode=="350581" & year==2001

replace regioncode="350582" if regioncode=="350582" & year==2001

replace regioncode="350583" if regioncode=="350583" & year==2001

replace regioncode="350602" if regioncode=="350602" & year==2001

replace regioncode="350603" if regioncode=="350603" & year==2001

replace regioncode="350622" if regioncode=="350622" & year==2001

replace regioncode="350623" if regioncode=="350623" & year==2001

replace regioncode="350624" if regioncode=="350624" & year==2001

replace regioncode="350625" if regioncode=="350625" & year==2001

replace regioncode="350626" if regioncode=="350626" & year==2001

replace regioncode="350627" if regioncode=="350627" & year==2001

replace regioncode="350628" if regioncode=="350628" & year==2001

replace regioncode="350629" if regioncode=="350629" & year==2001

replace regioncode="350681" if regioncode=="350681" & year==2001

replace regioncode="350702" if regioncode=="350702" & year==2001

replace regioncode="350721" if regioncode=="350721" & year==2001

replace regioncode="350722" if regioncode=="350722" & year==2001

replace regioncode="350723" if regioncode=="350723" & year==2001

replace regioncode="350724" if regioncode=="350724" & year==2001

replace regioncode="350725" if regioncode=="350725" & year==2001

replace regioncode="350781" if regioncode=="350781" & year==2001

replace regioncode="350782" if regioncode=="350782" & year==2001

replace regioncode="350783" if regioncode=="350783" & year==2001

replace regioncode="350784" if regioncode=="350784" & year==2001

replace regioncode="350802" if regioncode=="350802" & year==2001

replace regioncode="350821" if regioncode=="350821" & year==2001

replace regioncode="350822" if regioncode=="350822" & year==2001

replace regioncode="350823" if regioncode=="350823" & year==2001

replace regioncode="350824" if regioncode=="350824" & year==2001

replace regioncode="350825" if regioncode=="350825" & year==2001

replace regioncode="350881" if regioncode=="350881" & year==2001

replace regioncode="350902" if regioncode=="350902" & year==2001

replace regioncode="350981" if regioncode=="350981" & year==2001

replace regioncode="350982" if regioncode=="350982" & year==2001

replace regioncode="350921" if regioncode=="350921" & year==2001

replace regioncode="350922" if regioncode=="350922" & year==2001

replace regioncode="350923" if regioncode=="350923" & year==2001

replace regioncode="350924" if regioncode=="350924" & year==2001

replace regioncode="350925" if regioncode=="350925" & year==2001

replace regioncode="350926" if regioncode=="350926" & year==2001

replace regioncode="360102" if regioncode=="360102" & year==2001

replace regioncode="360103" if regioncode=="360103" & year==2001

replace regioncode="360104" if regioncode=="360104" & year==2001

replace regioncode="360105" if regioncode=="360105" & year==2001

replace regioncode="360111" if regioncode=="360111" & year==2001

replace regioncode="360121" if regioncode=="360121" & year==2001

replace regioncode="360122" if regioncode=="360122" & year==2001

replace regioncode="360123" if regioncode=="360123" & year==2001

replace regioncode="360124" if regioncode=="360124" & year==2001

replace regioncode="360202" if regioncode=="360202" & year==2001

replace regioncode="360203" if regioncode=="360203" & year==2001

replace regioncode="360222" if regioncode=="360222" & year==2001

replace regioncode="360281" if regioncode=="360281" & year==2001

replace regioncode="360302" if regioncode=="360302" & year==2001

replace regioncode="360313" if regioncode=="360313" & year==2001

replace regioncode="360321" if regioncode=="360321" & year==2001

replace regioncode="360322" if regioncode=="360322" & year==2001

replace regioncode="360323" if regioncode=="360323" & year==2001

replace regioncode="360402" if regioncode=="360402" & year==2001

replace regioncode="360403" if regioncode=="360403" & year==2001

replace regioncode="360421" if regioncode=="360421" & year==2001

replace regioncode="360423" if regioncode=="360423" & year==2001

replace regioncode="360424" if regioncode=="360424" & year==2001

replace regioncode="360425" if regioncode=="360425" & year==2001

replace regioncode="360426" if regioncode=="360426" & year==2001

replace regioncode="360427" if regioncode=="360427" & year==2001

replace regioncode="360428" if regioncode=="360428" & year==2001

replace regioncode="360429" if regioncode=="360429" & year==2001

replace regioncode="360430" if regioncode=="360430" & year==2001

replace regioncode="360481" if regioncode=="360481" & year==2001

replace regioncode="360502" if regioncode=="360502" & year==2001

replace regioncode="360521" if regioncode=="360521" & year==2001

replace regioncode="360602" if regioncode=="360602" & year==2001

replace regioncode="360622" if regioncode=="360622" & year==2001

replace regioncode="360681" if regioncode=="360681" & year==2001

replace regioncode="360702" if regioncode=="360702" & year==2001

replace regioncode="360721" if regioncode=="360721" & year==2001

replace regioncode="360722" if regioncode=="360722" & year==2001

replace regioncode="360723" if regioncode=="360723" & year==2001

replace regioncode="360724" if regioncode=="360724" & year==2001

replace regioncode="360725" if regioncode=="360725" & year==2001

replace regioncode="360726" if regioncode=="360726" & year==2001

replace regioncode="360727" if regioncode=="360727" & year==2001

replace regioncode="360728" if regioncode=="360728" & year==2001

replace regioncode="360729" if regioncode=="360729" & year==2001

replace regioncode="360730" if regioncode=="360730" & year==2001

replace regioncode="360731" if regioncode=="360731" & year==2001

replace regioncode="360732" if regioncode=="360732" & year==2001

replace regioncode="360733" if regioncode=="360733" & year==2001

replace regioncode="360734" if regioncode=="360734" & year==2001

replace regioncode="360735" if regioncode=="360735" & year==2001

replace regioncode="360781" if regioncode=="360781" & year==2001

replace regioncode="360782" if regioncode=="360782" & year==2001

replace regioncode="360802" if regioncode=="360802" & year==2001

replace regioncode="360821" if regioncode=="360821" & year==2001

replace regioncode="360822" if regioncode=="360822" & year==2001

replace regioncode="360823" if regioncode=="360823" & year==2001

replace regioncode="360824" if regioncode=="360824" & year==2001

replace regioncode="360825" if regioncode=="360825" & year==2001

replace regioncode="360826" if regioncode=="360826" & year==2001

replace regioncode="360827" if regioncode=="360827" & year==2001

replace regioncode="360828" if regioncode=="360828" & year==2001

replace regioncode="360829" if regioncode=="360829" & year==2001

replace regioncode="360830" if regioncode=="360830" & year==2001

replace regioncode="360881" if regioncode=="360881" & year==2001

replace regioncode="360902" if regioncode=="360902" & year==2001

replace regioncode="360921" if regioncode=="360921" & year==2001

replace regioncode="360922" if regioncode=="360922" & year==2001

replace regioncode="360923" if regioncode=="360923" & year==2001

replace regioncode="360924" if regioncode=="360924" & year==2001

replace regioncode="360925" if regioncode=="360925" & year==2001

replace regioncode="360926" if regioncode=="360926" & year==2001

replace regioncode="360981" if regioncode=="360981" & year==2001

replace regioncode="360982" if regioncode=="360982" & year==2001

replace regioncode="360983" if regioncode=="360983" & year==2001

replace regioncode="361002" if regioncode=="361002" & year==2001

replace regioncode="361021" if regioncode=="361021" & year==2001

replace regioncode="361022" if regioncode=="361022" & year==2001

replace regioncode="361023" if regioncode=="361023" & year==2001

replace regioncode="361024" if regioncode=="361024" & year==2001

replace regioncode="361025" if regioncode=="361025" & year==2001

replace regioncode="361026" if regioncode=="361026" & year==2001

replace regioncode="361027" if regioncode=="361027" & year==2001

replace regioncode="361028" if regioncode=="361028" & year==2001

replace regioncode="361029" if regioncode=="361029" & year==2001

replace regioncode="361030" if regioncode=="361030" & year==2001

replace regioncode="361102" if regioncode=="361102" & year==2001

replace regioncode="361121" if regioncode=="361121" & year==2001

replace regioncode="361122" if regioncode=="361122" & year==2001

replace regioncode="361123" if regioncode=="361123" & year==2001

replace regioncode="361124" if regioncode=="361124" & year==2001

replace regioncode="361125" if regioncode=="361125" & year==2001

replace regioncode="361126" if regioncode=="361126" & year==2001

replace regioncode="361127" if regioncode=="361127" & year==2001

replace regioncode="361128" if regioncode=="361128" & year==2001

replace regioncode="361129" if regioncode=="361129" & year==2001

replace regioncode="361130" if regioncode=="361130" & year==2001

replace regioncode="361181" if regioncode=="361181" & year==2001

replace regioncode="370102" if regioncode=="370102" & year==2001

replace regioncode="370103" if regioncode=="370103" & year==2001

replace regioncode="370104" if regioncode=="370104" & year==2001

replace regioncode="370105" if regioncode=="370105" & year==2001

replace regioncode="370112" if regioncode=="370112" & year==2001

replace regioncode="370113" if regioncode=="370113" & year==2001

replace regioncode="370124" if regioncode=="370124" & year==2001

replace regioncode="370125" if regioncode=="370125" & year==2001

replace regioncode="370126" if regioncode=="370126" & year==2001

replace regioncode="370181" if regioncode=="370181" & year==2001

replace regioncode="370202" if regioncode=="370202" & year==2001

replace regioncode="370203" if regioncode=="370203" & year==2001

replace regioncode="370205" if regioncode=="370205" & year==2001

replace regioncode="370211" if regioncode=="370211" & year==2001

replace regioncode="370212" if regioncode=="370212" & year==2001

replace regioncode="370213" if regioncode=="370213" & year==2001

replace regioncode="370214" if regioncode=="370214" & year==2001

replace regioncode="370281" if regioncode=="370281" & year==2001

replace regioncode="370282" if regioncode=="370282" & year==2001

replace regioncode="370283" if regioncode=="370283" & year==2001

replace regioncode="370284" if regioncode=="370284" & year==2001

replace regioncode="370285" if regioncode=="370285" & year==2001

replace regioncode="370302" if regioncode=="370302" & year==2001

replace regioncode="370303" if regioncode=="370303" & year==2001

replace regioncode="370304" if regioncode=="370304" & year==2001

replace regioncode="370305" if regioncode=="370305" & year==2001

replace regioncode="370306" if regioncode=="370306" & year==2001

replace regioncode="370321" if regioncode=="370321" & year==2001

replace regioncode="370322" if regioncode=="370322" & year==2001

replace regioncode="370323" if regioncode=="370323" & year==2001

replace regioncode="370402" if regioncode=="370402" & year==2001

replace regioncode="370403" if regioncode=="370403" & year==2001

replace regioncode="370404" if regioncode=="370404" & year==2001

replace regioncode="370405" if regioncode=="370405" & year==2001

replace regioncode="370406" if regioncode=="370406" & year==2001

replace regioncode="370481" if regioncode=="370481" & year==2001

replace regioncode="370502" if regioncode=="370502" & year==2001

replace regioncode="370503" if regioncode=="370503" & year==2001

replace regioncode="370521" if regioncode=="370521" & year==2001

replace regioncode="370522" if regioncode=="370522" & year==2001

replace regioncode="370523" if regioncode=="370523" & year==2001

replace regioncode="370602" if regioncode=="370602" & year==2001

replace regioncode="370611" if regioncode=="370611" & year==2001

replace regioncode="370612" if regioncode=="370612" & year==2001

replace regioncode="370613" if regioncode=="370613" & year==2001

replace regioncode="370634" if regioncode=="370634" & year==2001

replace regioncode="370681" if regioncode=="370681" & year==2001

replace regioncode="370682" if regioncode=="370682" & year==2001

replace regioncode="370683" if regioncode=="370683" & year==2001

replace regioncode="370684" if regioncode=="370684" & year==2001

replace regioncode="370685" if regioncode=="370685" & year==2001

replace regioncode="370686" if regioncode=="370686" & year==2001

replace regioncode="370687" if regioncode=="370687" & year==2001

replace regioncode="370702" if regioncode=="370702" & year==2001

replace regioncode="370703" if regioncode=="370703" & year==2001

replace regioncode="370704" if regioncode=="370704" & year==2001

replace regioncode="370705" if regioncode=="370705" & year==2001

replace regioncode="370724" if regioncode=="370724" & year==2001

replace regioncode="370725" if regioncode=="370725" & year==2001

replace regioncode="370781" if regioncode=="370781" & year==2001

replace regioncode="370782" if regioncode=="370782" & year==2001

replace regioncode="370783" if regioncode=="370783" & year==2001

replace regioncode="370784" if regioncode=="370784" & year==2001

replace regioncode="370785" if regioncode=="370785" & year==2001

replace regioncode="370786" if regioncode=="370786" & year==2001

replace regioncode="370802" if regioncode=="370802" & year==2001

replace regioncode="370811" if regioncode=="370811" & year==2001

replace regioncode="370826" if regioncode=="370826" & year==2001

replace regioncode="370827" if regioncode=="370827" & year==2001

replace regioncode="370828" if regioncode=="370828" & year==2001

replace regioncode="370829" if regioncode=="370829" & year==2001

replace regioncode="370830" if regioncode=="370830" & year==2001

replace regioncode="370831" if regioncode=="370831" & year==2001

replace regioncode="370832" if regioncode=="370832" & year==2001

replace regioncode="370881" if regioncode=="370881" & year==2001

replace regioncode="370882" if regioncode=="370882" & year==2001

replace regioncode="370883" if regioncode=="370883" & year==2001

replace regioncode="370902" if regioncode=="370902" & year==2001

replace regioncode="370903" if regioncode=="370903" & year==2001

replace regioncode="370921" if regioncode=="370921" & year==2001

replace regioncode="370923" if regioncode=="370923" & year==2001

replace regioncode="370982" if regioncode=="370982" & year==2001

replace regioncode="370983" if regioncode=="370983" & year==2001

replace regioncode="371002" if regioncode=="371002" & year==2001

replace regioncode="371081" if regioncode=="371081" & year==2001

replace regioncode="371082" if regioncode=="371082" & year==2001

replace regioncode="371083" if regioncode=="371083" & year==2001

replace regioncode="371102" if regioncode=="371102" & year==2001

replace regioncode="371121" if regioncode=="371121" & year==2001

replace regioncode="371122" if regioncode=="371122" & year==2001

replace regioncode="371202" if regioncode=="371202" & year==2001

replace regioncode="371203" if regioncode=="371203" & year==2001

replace regioncode="371302" if regioncode=="371302" & year==2001

replace regioncode="371311" if regioncode=="371311" & year==2001

replace regioncode="371312" if regioncode=="371312" & year==2001

replace regioncode="371321" if regioncode=="371321" & year==2001

replace regioncode="371322" if regioncode=="371322" & year==2001

replace regioncode="371323" if regioncode=="371323" & year==2001

replace regioncode="371324" if regioncode=="371324" & year==2001

replace regioncode="371325" if regioncode=="371325" & year==2001

replace regioncode="371326" if regioncode=="371326" & year==2001

replace regioncode="371327" if regioncode=="371327" & year==2001

replace regioncode="371328" if regioncode=="371328" & year==2001

replace regioncode="371329" if regioncode=="371329" & year==2001

replace regioncode="371402" if regioncode=="371402" & year==2001

replace regioncode="371421" if regioncode=="371421" & year==2001

replace regioncode="371422" if regioncode=="371422" & year==2001

replace regioncode="371423" if regioncode=="371423" & year==2001

replace regioncode="371424" if regioncode=="371424" & year==2001

replace regioncode="371425" if regioncode=="371425" & year==2001

replace regioncode="371426" if regioncode=="371426" & year==2001

replace regioncode="371427" if regioncode=="371427" & year==2001

replace regioncode="371428" if regioncode=="371428" & year==2001

replace regioncode="371481" if regioncode=="371481" & year==2001

replace regioncode="371482" if regioncode=="371482" & year==2001

replace regioncode="371502" if regioncode=="371502" & year==2001

replace regioncode="371521" if regioncode=="371521" & year==2001

replace regioncode="371522" if regioncode=="371522" & year==2001

replace regioncode="371523" if regioncode=="371523" & year==2001

replace regioncode="371524" if regioncode=="371524" & year==2001

replace regioncode="371525" if regioncode=="371525" & year==2001

replace regioncode="371526" if regioncode=="371526" & year==2001

replace regioncode="371581" if regioncode=="371581" & year==2001

replace regioncode="371602" if regioncode=="371602" & year==2001

replace regioncode="371621" if regioncode=="371621" & year==2001

replace regioncode="371622" if regioncode=="371622" & year==2001

replace regioncode="371623" if regioncode=="371623" & year==2001

replace regioncode="371624" if regioncode=="371624" & year==2001

replace regioncode="371625" if regioncode=="371625" & year==2001

replace regioncode="371626" if regioncode=="371626" & year==2001

replace regioncode="371702" if regioncode=="371702" & year==2001

replace regioncode="371721" if regioncode=="371721" & year==2001

replace regioncode="371722" if regioncode=="371722" & year==2001

replace regioncode="371723" if regioncode=="371723" & year==2001

replace regioncode="371724" if regioncode=="371724" & year==2001

replace regioncode="371725" if regioncode=="371725" & year==2001

replace regioncode="371726" if regioncode=="371726" & year==2001

replace regioncode="371727" if regioncode=="371727" & year==2001

replace regioncode="371728" if regioncode=="371728" & year==2001

replace regioncode="410102" if regioncode=="410102" & year==2001

replace regioncode="410103" if regioncode=="410103" & year==2001

replace regioncode="410104" if regioncode=="410104" & year==2001

replace regioncode="410105" if regioncode=="410105" & year==2001

replace regioncode="410106" if regioncode=="410106" & year==2001

replace regioncode="410108" if regioncode=="410108" & year==2001

replace regioncode="410122" if regioncode=="410122" & year==2001

replace regioncode="410181" if regioncode=="410181" & year==2001

replace regioncode="410182" if regioncode=="410182" & year==2001

replace regioncode="410183" if regioncode=="410183" & year==2001

replace regioncode="410184" if regioncode=="410184" & year==2001

replace regioncode="410185" if regioncode=="410185" & year==2001

replace regioncode="410202" if regioncode=="410202" & year==2001

replace regioncode="410203" if regioncode=="410203" & year==2001

replace regioncode="410204" if regioncode=="410204" & year==2001

replace regioncode="410205" if regioncode=="410205" & year==2001

replace regioncode="410211" if regioncode=="410211" & year==2001

replace regioncode="410221" if regioncode=="410221" & year==2001

replace regioncode="410222" if regioncode=="410222" & year==2001

replace regioncode="410223" if regioncode=="410223" & year==2001

replace regioncode="410224" if regioncode=="410224" & year==2001

replace regioncode="410225" if regioncode=="410225" & year==2001

replace regioncode="410302" if regioncode=="410302" & year==2001

replace regioncode="410303" if regioncode=="410303" & year==2001

replace regioncode="410304" if regioncode=="410304" & year==2001

replace regioncode="410305" if regioncode=="410305" & year==2001

replace regioncode="410306" if regioncode=="410306" & year==2001

replace regioncode="410307" if regioncode=="410307" & year==2001

replace regioncode="410322" if regioncode=="410322" & year==2001

replace regioncode="410323" if regioncode=="410323" & year==2001

replace regioncode="410324" if regioncode=="410324" & year==2001

replace regioncode="410325" if regioncode=="410325" & year==2001

replace regioncode="410326" if regioncode=="410326" & year==2001

replace regioncode="410327" if regioncode=="410327" & year==2001

replace regioncode="410328" if regioncode=="410328" & year==2001

replace regioncode="410329" if regioncode=="410329" & year==2001

replace regioncode="410381" if regioncode=="410381" & year==2001

replace regioncode="410402" if regioncode=="410402" & year==2001

replace regioncode="410403" if regioncode=="410403" & year==2001

replace regioncode="410404" if regioncode=="410404" & year==2001

replace regioncode="410411" if regioncode=="410411" & year==2001

replace regioncode="410421" if regioncode=="410421" & year==2001

replace regioncode="410422" if regioncode=="410422" & year==2001

replace regioncode="410423" if regioncode=="410423" & year==2001

replace regioncode="410425" if regioncode=="410425" & year==2001

replace regioncode="410481" if regioncode=="410481" & year==2001

replace regioncode="410482" if regioncode=="410482" & year==2001

replace regioncode="410502" if regioncode=="410502" & year==2001

replace regioncode="410503" if regioncode=="410503" & year==2001

replace regioncode="410504" if regioncode=="410504" & year==2001

replace regioncode="410511" if regioncode=="410511" & year==2001

replace regioncode="410522" if regioncode=="410522" & year==2001

replace regioncode="410523" if regioncode=="410523" & year==2001

replace regioncode="410526" if regioncode=="410526" & year==2001

replace regioncode="410527" if regioncode=="410527" & year==2001

replace regioncode="410581" if regioncode=="410581" & year==2001

replace regioncode="410602" if regioncode=="410602" & year==2001

replace regioncode="410603" if regioncode=="410603" & year==2001

replace regioncode="410611" if regioncode=="410611" & year==2001

replace regioncode="410621" if regioncode=="410621" & year==2001

replace regioncode="410622" if regioncode=="410622" & year==2001

replace regioncode="410702" if regioncode=="410702" & year==2001

replace regioncode="410703" if regioncode=="410703" & year==2001

replace regioncode="410704" if regioncode=="410704" & year==2001

replace regioncode="410711" if regioncode=="410711" & year==2001

replace regioncode="410721" if regioncode=="410721" & year==2001

replace regioncode="410724" if regioncode=="410724" & year==2001

replace regioncode="410725" if regioncode=="410725" & year==2001

replace regioncode="410726" if regioncode=="410726" & year==2001

replace regioncode="410727" if regioncode=="410727" & year==2001

replace regioncode="410728" if regioncode=="410728" & year==2001

replace regioncode="410781" if regioncode=="410781" & year==2001

replace regioncode="410782" if regioncode=="410782" & year==2001

replace regioncode="410802" if regioncode=="410802" & year==2001

replace regioncode="410803" if regioncode=="410803" & year==2001

replace regioncode="410804" if regioncode=="410804" & year==2001

replace regioncode="410811" if regioncode=="410811" & year==2001

replace regioncode="410821" if regioncode=="410821" & year==2001

replace regioncode="410822" if regioncode=="410822" & year==2001

replace regioncode="410823" if regioncode=="410823" & year==2001

replace regioncode="410825" if regioncode=="410825" & year==2001

replace regioncode="410881" if regioncode=="410881" & year==2001

replace regioncode="410882" if regioncode=="410882" & year==2001

replace regioncode="410883" if regioncode=="410883" & year==2001

replace regioncode="410902" if regioncode=="410902" & year==2001

replace regioncode="410922" if regioncode=="410922" & year==2001

replace regioncode="410923" if regioncode=="410923" & year==2001

replace regioncode="410926" if regioncode=="410926" & year==2001

replace regioncode="410927" if regioncode=="410927" & year==2001

replace regioncode="410928" if regioncode=="410928" & year==2001

replace regioncode="411002" if regioncode=="411002" & year==2001

replace regioncode="411023" if regioncode=="411023" & year==2001

replace regioncode="411024" if regioncode=="411024" & year==2001

replace regioncode="411025" if regioncode=="411025" & year==2001

replace regioncode="411081" if regioncode=="411081" & year==2001

replace regioncode="411082" if regioncode=="411082" & year==2001

replace regioncode="411102" if regioncode=="411102" & year==2001

replace regioncode="411123" if regioncode=="411123" & year==2001

replace regioncode="411121" if regioncode=="411121" & year==2001

replace regioncode="411122" if regioncode=="411122" & year==2001

replace regioncode="411202" if regioncode=="411202" & year==2001

replace regioncode="411221" if regioncode=="411221" & year==2001

replace regioncode="411222" if regioncode=="411222" & year==2001

replace regioncode="411224" if regioncode=="411224" & year==2001

replace regioncode="411281" if regioncode=="411281" & year==2001

replace regioncode="411282" if regioncode=="411282" & year==2001

replace regioncode="411302" if regioncode=="411302" & year==2001

replace regioncode="411303" if regioncode=="411303" & year==2001

replace regioncode="411321" if regioncode=="411321" & year==2001

replace regioncode="411322" if regioncode=="411322" & year==2001

replace regioncode="411323" if regioncode=="411323" & year==2001

replace regioncode="411324" if regioncode=="411324" & year==2001

replace regioncode="411325" if regioncode=="411325" & year==2001

replace regioncode="411326" if regioncode=="411326" & year==2001

replace regioncode="411327" if regioncode=="411327" & year==2001

replace regioncode="411328" if regioncode=="411328" & year==2001

replace regioncode="411329" if regioncode=="411329" & year==2001

replace regioncode="411330" if regioncode=="411330" & year==2001

replace regioncode="411381" if regioncode=="411381" & year==2001

replace regioncode="411402" if regioncode=="411402" & year==2001

replace regioncode="411403" if regioncode=="411403" & year==2001

replace regioncode="411421" if regioncode=="411421" & year==2001

replace regioncode="411422" if regioncode=="411422" & year==2001

replace regioncode="411423" if regioncode=="411423" & year==2001

replace regioncode="411424" if regioncode=="411424" & year==2001

replace regioncode="411425" if regioncode=="411425" & year==2001

replace regioncode="411426" if regioncode=="411426" & year==2001

replace regioncode="411481" if regioncode=="411481" & year==2001

replace regioncode="411502" if regioncode=="411502" & year==2001

replace regioncode="411503" if regioncode=="411503" & year==2001

replace regioncode="411521" if regioncode=="411521" & year==2001

replace regioncode="411522" if regioncode=="411522" & year==2001

replace regioncode="411523" if regioncode=="411523" & year==2001

replace regioncode="411524" if regioncode=="411524" & year==2001

replace regioncode="411525" if regioncode=="411525" & year==2001

replace regioncode="411526" if regioncode=="411526" & year==2001

replace regioncode="411527" if regioncode=="411527" & year==2001

replace regioncode="411528" if regioncode=="411528" & year==2001

replace regioncode="411602" if regioncode=="411602" & year==2001

replace regioncode="411621" if regioncode=="411621" & year==2001

replace regioncode="411622" if regioncode=="411622" & year==2001

replace regioncode="411623" if regioncode=="411623" & year==2001

replace regioncode="411624" if regioncode=="411624" & year==2001

replace regioncode="411625" if regioncode=="411625" & year==2001

replace regioncode="411626" if regioncode=="411626" & year==2001

replace regioncode="411627" if regioncode=="411627" & year==2001

replace regioncode="411628" if regioncode=="411628" & year==2001

replace regioncode="411681" if regioncode=="411681" & year==2001

replace regioncode="411702" if regioncode=="411702" & year==2001

replace regioncode="411721" if regioncode=="411721" & year==2001

replace regioncode="411722" if regioncode=="411722" & year==2001

replace regioncode="411723" if regioncode=="411723" & year==2001

replace regioncode="411724" if regioncode=="411724" & year==2001

replace regioncode="411725" if regioncode=="411725" & year==2001

replace regioncode="411726" if regioncode=="411726" & year==2001

replace regioncode="411727" if regioncode=="411727" & year==2001

replace regioncode="411728" if regioncode=="411728" & year==2001

replace regioncode="411729" if regioncode=="411729" & year==2001

replace regioncode="420102" if regioncode=="420102" & year==2001

replace regioncode="420103" if regioncode=="420103" & year==2001

replace regioncode="420104" if regioncode=="420104" & year==2001

replace regioncode="420105" if regioncode=="420105" & year==2001

replace regioncode="420106" if regioncode=="420106" & year==2001

replace regioncode="420107" if regioncode=="420107" & year==2001

replace regioncode="420111" if regioncode=="420111" & year==2001

replace regioncode="420112" if regioncode=="420112" & year==2001

replace regioncode="420113" if regioncode=="420113" & year==2001

replace regioncode="420114" if regioncode=="420114" & year==2001

replace regioncode="420115" if regioncode=="420115" & year==2001

replace regioncode="420116" if regioncode=="420116" & year==2001

replace regioncode="420117" if regioncode=="420117" & year==2001

replace regioncode="420202" if regioncode=="420202" & year==2001

replace regioncode="420203" if regioncode=="420203" & year==2001

replace regioncode="420204" if regioncode=="420204" & year==2001

replace regioncode="420205" if regioncode=="420205" & year==2001

replace regioncode="420222" if regioncode=="420222" & year==2001

replace regioncode="420281" if regioncode=="420281" & year==2001

replace regioncode="420302" if regioncode=="420302" & year==2001

replace regioncode="420303" if regioncode=="420303" & year==2001

replace regioncode="420321" if regioncode=="420321" & year==2001

replace regioncode="420322" if regioncode=="420322" & year==2001

replace regioncode="420323" if regioncode=="420323" & year==2001

replace regioncode="420324" if regioncode=="420324" & year==2001

replace regioncode="420325" if regioncode=="420325" & year==2001

replace regioncode="420381" if regioncode=="420381" & year==2001

replace regioncode="420502" if regioncode=="420502" & year==2001

replace regioncode="420503" if regioncode=="420503" & year==2001

replace regioncode="420504" if regioncode=="420504" & year==2001

replace regioncode="420505" if regioncode=="420505" & year==2001

replace regioncode="420506" if regioncode=="420521" & year==2001

replace regioncode="420525" if regioncode=="420525" & year==2001

replace regioncode="420526" if regioncode=="420526" & year==2001

replace regioncode="420527" if regioncode=="420527" & year==2001

replace regioncode="420528" if regioncode=="420528" & year==2001

replace regioncode="420529" if regioncode=="420529" & year==2001

replace regioncode="420581" if regioncode=="420581" & year==2001

replace regioncode="420582" if regioncode=="420582" & year==2001

replace regioncode="420583" if regioncode=="420583" & year==2001

replace regioncode="420602" if regioncode=="420602" & year==2001

replace regioncode="420606" if regioncode=="420606" & year==2001

replace regioncode="420607" if regioncode=="420607" & year==2001

replace regioncode="420624" if regioncode=="420624" & year==2001

replace regioncode="420625" if regioncode=="420625" & year==2001

replace regioncode="420626" if regioncode=="420626" & year==2001

replace regioncode="420682" if regioncode=="420682" & year==2001

replace regioncode="420683" if regioncode=="420683" & year==2001

replace regioncode="420684" if regioncode=="420684" & year==2001

replace regioncode="420702" if regioncode=="420702" & year==2001

replace regioncode="420703" if regioncode=="420703" & year==2001

replace regioncode="420704" if regioncode=="420704" & year==2001

replace regioncode="420802" if regioncode=="420802" & year==2001

replace regioncode="420804" if regioncode=="420804" & year==2001

replace regioncode="420821" if regioncode=="420821" & year==2001

replace regioncode="420822" if regioncode=="420822" & year==2001

replace regioncode="420881" if regioncode=="420881" & year==2001

replace regioncode="420902" if regioncode=="420902" & year==2001

replace regioncode="420921" if regioncode=="420921" & year==2001

replace regioncode="420922" if regioncode=="420922" & year==2001

replace regioncode="420923" if regioncode=="420923" & year==2001

replace regioncode="420981" if regioncode=="420981" & year==2001

replace regioncode="420982" if regioncode=="420982" & year==2001

replace regioncode="421381" if regioncode=="421381" & year==2001

replace regioncode="420984" if regioncode=="420984" & year==2001

replace regioncode="421002" if regioncode=="421002" & year==2001

replace regioncode="421003" if regioncode=="421003" & year==2001

replace regioncode="421022" if regioncode=="421022" & year==2001

replace regioncode="421023" if regioncode=="421023" & year==2001

replace regioncode="421024" if regioncode=="421024" & year==2001

replace regioncode="421081" if regioncode=="421081" & year==2001

replace regioncode="421083" if regioncode=="421083" & year==2001

replace regioncode="421087" if regioncode=="421087" & year==2001

replace regioncode="421102" if regioncode=="421102" & year==2001

replace regioncode="421121" if regioncode=="421121" & year==2001

replace regioncode="421122" if regioncode=="421122" & year==2001

replace regioncode="421123" if regioncode=="421123" & year==2001

replace regioncode="421124" if regioncode=="421124" & year==2001

replace regioncode="421125" if regioncode=="421125" & year==2001

replace regioncode="421126" if regioncode=="421126" & year==2001

replace regioncode="421127" if regioncode=="421127" & year==2001

replace regioncode="421181" if regioncode=="421181" & year==2001

replace regioncode="421182" if regioncode=="421182" & year==2001

replace regioncode="421202" if regioncode=="421202" & year==2001

replace regioncode="421221" if regioncode=="421221" & year==2001

replace regioncode="421222" if regioncode=="421222" & year==2001

replace regioncode="421223" if regioncode=="421223" & year==2001

replace regioncode="421224" if regioncode=="421224" & year==2001

replace regioncode="421281" if regioncode=="421281" & year==2001

replace regioncode="421302" if regioncode=="421302" & year==2001

replace regioncode="422801" if regioncode=="422801" & year==2001

replace regioncode="422802" if regioncode=="422802" & year==2001

replace regioncode="422822" if regioncode=="422822" & year==2001

replace regioncode="422823" if regioncode=="422823" & year==2001

replace regioncode="422825" if regioncode=="422825" & year==2001

replace regioncode="422826" if regioncode=="422826" & year==2001

replace regioncode="422827" if regioncode=="422827" & year==2001

replace regioncode="422828" if regioncode=="422828" & year==2001

replace regioncode="429004" if regioncode=="429004" & year==2001

replace regioncode="429005" if regioncode=="429005" & year==2001

replace regioncode="429006" if regioncode=="429006" & year==2001

replace regioncode="429021" if regioncode=="429021" & year==2001

replace regioncode="430102" if regioncode=="430102" & year==2001

replace regioncode="430103" if regioncode=="430103" & year==2001

replace regioncode="430104" if regioncode=="430104" & year==2001

replace regioncode="430105" if regioncode=="430105" & year==2001

replace regioncode="430111" if regioncode=="430111" & year==2001

replace regioncode="430121" if regioncode=="430121" & year==2001

replace regioncode="430122" if regioncode=="430122" & year==2001

replace regioncode="430124" if regioncode=="430124" & year==2001

replace regioncode="430181" if regioncode=="430181" & year==2001

replace regioncode="430202" if regioncode=="430202" & year==2001

replace regioncode="430203" if regioncode=="430203" & year==2001

replace regioncode="430204" if regioncode=="430204" & year==2001

replace regioncode="430211" if regioncode=="430211" & year==2001

replace regioncode="430221" if regioncode=="430221" & year==2001

replace regioncode="430223" if regioncode=="430223" & year==2001

replace regioncode="430224" if regioncode=="430224" & year==2001

replace regioncode="430225" if regioncode=="430225" & year==2001

replace regioncode="430281" if regioncode=="430281" & year==2001

replace regioncode="430302" if regioncode=="430302" & year==2001

replace regioncode="430304" if regioncode=="430304" & year==2001

replace regioncode="430321" if regioncode=="430321" & year==2001

replace regioncode="430381" if regioncode=="430381" & year==2001

replace regioncode="430382" if regioncode=="430382" & year==2001

replace regioncode="430405" if regioncode=="430405" & year==2001

replace regioncode="430406" if regioncode=="430406" & year==2001

replace regioncode="430407" if regioncode=="430407" & year==2001

replace regioncode="430408" if regioncode=="430408" & year==2001

replace regioncode="430412" if regioncode=="430412" & year==2001

replace regioncode="430421" if regioncode=="430421" & year==2001

replace regioncode="430422" if regioncode=="430422" & year==2001

replace regioncode="430423" if regioncode=="430423" & year==2001

replace regioncode="430424" if regioncode=="430424" & year==2001

replace regioncode="430426" if regioncode=="430426" & year==2001

replace regioncode="430481" if regioncode=="430481" & year==2001

replace regioncode="430482" if regioncode=="430482" & year==2001

replace regioncode="430502" if regioncode=="430502" & year==2001

replace regioncode="430503" if regioncode=="430503" & year==2001

replace regioncode="430511" if regioncode=="430511" & year==2001

replace regioncode="430521" if regioncode=="430521" & year==2001

replace regioncode="430522" if regioncode=="430522" & year==2001

replace regioncode="430523" if regioncode=="430523" & year==2001

replace regioncode="430524" if regioncode=="430524" & year==2001

replace regioncode="430525" if regioncode=="430525" & year==2001

replace regioncode="430527" if regioncode=="430527" & year==2001

replace regioncode="430528" if regioncode=="430528" & year==2001

replace regioncode="430529" if regioncode=="430529" & year==2001

replace regioncode="430581" if regioncode=="430581" & year==2001

replace regioncode="430602" if regioncode=="430602" & year==2001

replace regioncode="430603" if regioncode=="430603" & year==2001

replace regioncode="430611" if regioncode=="430611" & year==2001

replace regioncode="430621" if regioncode=="430621" & year==2001

replace regioncode="430623" if regioncode=="430623" & year==2001

replace regioncode="430624" if regioncode=="430624" & year==2001

replace regioncode="430626" if regioncode=="430626" & year==2001

replace regioncode="430681" if regioncode=="430681" & year==2001

replace regioncode="430682" if regioncode=="430682" & year==2001

replace regioncode="430702" if regioncode=="430702" & year==2001

replace regioncode="430703" if regioncode=="430703" & year==2001

replace regioncode="430721" if regioncode=="430721" & year==2001

replace regioncode="430722" if regioncode=="430722" & year==2001

replace regioncode="430723" if regioncode=="430723" & year==2001

replace regioncode="430724" if regioncode=="430724" & year==2001

replace regioncode="430725" if regioncode=="430725" & year==2001

replace regioncode="430726" if regioncode=="430726" & year==2001

replace regioncode="430781" if regioncode=="430781" & year==2001

replace regioncode="430802" if regioncode=="430802" & year==2001

replace regioncode="430811" if regioncode=="430811" & year==2001

replace regioncode="430821" if regioncode=="430821" & year==2001

replace regioncode="430822" if regioncode=="430822" & year==2001

replace regioncode="430902" if regioncode=="430902" & year==2001

replace regioncode="430903" if regioncode=="430903" & year==2001

replace regioncode="430921" if regioncode=="430921" & year==2001

replace regioncode="430922" if regioncode=="430922" & year==2001

replace regioncode="430923" if regioncode=="430923" & year==2001

replace regioncode="430981" if regioncode=="430981" & year==2001

replace regioncode="431002" if regioncode=="431002" & year==2001

replace regioncode="431003" if regioncode=="431003" & year==2001

replace regioncode="431021" if regioncode=="431021" & year==2001

replace regioncode="431022" if regioncode=="431022" & year==2001

replace regioncode="431023" if regioncode=="431023" & year==2001

replace regioncode="431024" if regioncode=="431024" & year==2001

replace regioncode="431025" if regioncode=="431025" & year==2001

replace regioncode="431026" if regioncode=="431026" & year==2001

replace regioncode="431027" if regioncode=="431027" & year==2001

replace regioncode="431028" if regioncode=="431028" & year==2001

replace regioncode="431081" if regioncode=="431081" & year==2001

replace regioncode="431102" if regioncode=="431102" & year==2001

replace regioncode="431103" if regioncode=="431103" & year==2001

replace regioncode="431121" if regioncode=="431121" & year==2001

replace regioncode="431122" if regioncode=="431122" & year==2001

replace regioncode="431123" if regioncode=="431123" & year==2001

replace regioncode="431124" if regioncode=="431124" & year==2001

replace regioncode="431125" if regioncode=="431125" & year==2001

replace regioncode="431126" if regioncode=="431126" & year==2001

replace regioncode="431127" if regioncode=="431127" & year==2001

replace regioncode="431128" if regioncode=="431128" & year==2001

replace regioncode="431129" if regioncode=="431129" & year==2001

replace regioncode="431202" if regioncode=="431202" & year==2001

replace regioncode="431221" if regioncode=="431221" & year==2001

replace regioncode="431222" if regioncode=="431222" & year==2001

replace regioncode="431223" if regioncode=="431223" & year==2001

replace regioncode="431224" if regioncode=="431224" & year==2001

replace regioncode="431225" if regioncode=="431225" & year==2001

replace regioncode="431226" if regioncode=="431226" & year==2001

replace regioncode="431227" if regioncode=="431227" & year==2001

replace regioncode="431228" if regioncode=="431228" & year==2001

replace regioncode="431229" if regioncode=="431229" & year==2001

replace regioncode="431230" if regioncode=="431230" & year==2001

replace regioncode="431281" if regioncode=="431281" & year==2001

replace regioncode="431302" if regioncode=="431302" & year==2001

replace regioncode="431321" if regioncode=="431321" & year==2001

replace regioncode="431322" if regioncode=="431322" & year==2001

replace regioncode="431381" if regioncode=="431381" & year==2001

replace regioncode="431382" if regioncode=="431382" & year==2001

replace regioncode="433101" if regioncode=="433101" & year==2001

replace regioncode="433122" if regioncode=="433122" & year==2001

replace regioncode="433123" if regioncode=="433123" & year==2001

replace regioncode="433124" if regioncode=="433124" & year==2001

replace regioncode="433125" if regioncode=="433125" & year==2001

replace regioncode="433126" if regioncode=="433126" & year==2001

replace regioncode="433127" if regioncode=="433127" & year==2001

replace regioncode="433130" if regioncode=="433130" & year==2001

replace regioncode="440102" if regioncode=="440102" & year==2001

replace regioncode="440103" if regioncode=="440103" & year==2001

replace regioncode="440104" if regioncode=="440104" & year==2001

replace regioncode="440105" if regioncode=="440105" & year==2001

replace regioncode="440106" if regioncode=="440106" & year==2001

replace regioncode="440107" if regioncode=="440107" & year==2001

replace regioncode="440111" if regioncode=="440111" & year==2001

replace regioncode="440112" if regioncode=="440112" & year==2001

replace regioncode="440113" if regioncode=="440113" & year==2001

replace regioncode="440114" if regioncode=="440114" & year==2001

replace regioncode="440183" if regioncode=="440183" & year==2001

replace regioncode="440184" if regioncode=="440184" & year==2001

replace regioncode="440203" if regioncode=="440203" & year==2001

replace regioncode="440204" if regioncode=="440204" & year==2001

replace regioncode="440221" if regioncode=="440221" & year==2001

replace regioncode="440222" if regioncode=="440222" & year==2001

replace regioncode="440224" if regioncode=="440224" & year==2001

replace regioncode="440229" if regioncode=="440229" & year==2001

replace regioncode="440232" if regioncode=="440232" & year==2001

replace regioncode="440233" if regioncode=="440233" & year==2001

replace regioncode="440281" if regioncode=="440281" & year==2001

replace regioncode="440282" if regioncode=="440282" & year==2001

replace regioncode="440303" if regioncode=="440303" & year==2001

replace regioncode="440304" if regioncode=="440304" & year==2001

replace regioncode="440305" if regioncode=="440305" & year==2001

replace regioncode="440306" if regioncode=="440306" & year==2001

replace regioncode="440307" if regioncode=="440307" & year==2001

replace regioncode="440308" if regioncode=="440308" & year==2001

replace regioncode="440402" if regioncode=="440402" & year==2001

replace regioncode="440403" if regioncode=="440403" & year==2001

replace regioncode="440404" if regioncode=="440404" & year==2001

replace regioncode="440507" if regioncode=="440507" & year==2001

replace regioncode="440511" if regioncode=="440509" & year==2001

replace regioncode="440512" if regioncode=="440510" & year==2001

replace regioncode="440514" if regioncode=="440582" & year==2001

replace regioncode="440515" if regioncode=="440583" & year==2001

replace regioncode="440523" if regioncode=="440523" & year==2001

replace regioncode="440604" if regioncode=="440603" & year==2001

replace regioncode="440605" if regioncode=="440682" & year==2001

replace regioncode="440606" if regioncode=="440681" & year==2001

replace regioncode="440607" if regioncode=="440683" & year==2001

replace regioncode="440608" if regioncode=="440684" & year==2001

replace regioncode="440703" if regioncode=="440703" & year==2001

replace regioncode="440704" if regioncode=="440704" & year==2001

replace regioncode="440781" if regioncode=="440781" & year==2001

replace regioncode="440705" if regioncode=="440782" & year==2001

replace regioncode="440783" if regioncode=="440783" & year==2001

replace regioncode="440784" if regioncode=="440784" & year==2001

replace regioncode="440785" if regioncode=="440785" & year==2001

replace regioncode="440802" if regioncode=="440802" & year==2001

replace regioncode="440803" if regioncode=="440803" & year==2001

replace regioncode="440804" if regioncode=="440804" & year==2001

replace regioncode="440811" if regioncode=="440811" & year==2001

replace regioncode="440823" if regioncode=="440823" & year==2001

replace regioncode="440825" if regioncode=="440825" & year==2001

replace regioncode="440881" if regioncode=="440881" & year==2001

replace regioncode="440882" if regioncode=="440882" & year==2001

replace regioncode="440883" if regioncode=="440883" & year==2001

replace regioncode="440902" if regioncode=="440902" & year==2001

replace regioncode="440903" if regioncode=="440903" & year==2001

replace regioncode="440923" if regioncode=="440923" & year==2001

replace regioncode="440981" if regioncode=="440981" & year==2001

replace regioncode="440982" if regioncode=="440982" & year==2001

replace regioncode="440983" if regioncode=="440983" & year==2001

replace regioncode="441202" if regioncode=="441202" & year==2001

replace regioncode="441203" if regioncode=="441203" & year==2001

replace regioncode="441223" if regioncode=="441223" & year==2001

replace regioncode="441224" if regioncode=="441224" & year==2001

replace regioncode="441225" if regioncode=="441225" & year==2001

replace regioncode="441226" if regioncode=="441226" & year==2001

replace regioncode="441283" if regioncode=="441283" & year==2001

replace regioncode="441284" if regioncode=="441284" & year==2001

replace regioncode="441302" if regioncode=="441302" & year==2001

replace regioncode="441303" if regioncode=="441381" & year==2001

replace regioncode="441322" if regioncode=="441322" & year==2001

replace regioncode="441323" if regioncode=="441323" & year==2001

replace regioncode="441324" if regioncode=="441324" & year==2001

replace regioncode="441402" if regioncode=="441402" & year==2001

replace regioncode="441421" if regioncode=="441421" & year==2001

replace regioncode="441422" if regioncode=="441422" & year==2001

replace regioncode="441423" if regioncode=="441423" & year==2001

replace regioncode="441424" if regioncode=="441424" & year==2001

replace regioncode="441426" if regioncode=="441426" & year==2001

replace regioncode="441427" if regioncode=="441427" & year==2001

replace regioncode="441481" if regioncode=="441481" & year==2001

replace regioncode="441502" if regioncode=="441502" & year==2001

replace regioncode="441521" if regioncode=="441521" & year==2001

replace regioncode="441523" if regioncode=="441523" & year==2001

replace regioncode="441581" if regioncode=="441581" & year==2001

replace regioncode="441602" if regioncode=="441602" & year==2001

replace regioncode="441621" if regioncode=="441621" & year==2001

replace regioncode="441622" if regioncode=="441622" & year==2001

replace regioncode="441623" if regioncode=="441623" & year==2001

replace regioncode="441624" if regioncode=="441624" & year==2001

replace regioncode="441625" if regioncode=="441625" & year==2001

replace regioncode="441702" if regioncode=="441702" & year==2001

replace regioncode="441721" if regioncode=="441721" & year==2001

replace regioncode="441723" if regioncode=="441723" & year==2001

replace regioncode="441781" if regioncode=="441781" & year==2001

replace regioncode="441802" if regioncode=="441802" & year==2001

replace regioncode="441821" if regioncode=="441821" & year==2001

replace regioncode="441823" if regioncode=="441823" & year==2001

replace regioncode="441825" if regioncode=="441825" & year==2001

replace regioncode="441826" if regioncode=="441826" & year==2001

replace regioncode="441827" if regioncode=="441827" & year==2001

replace regioncode="441881" if regioncode=="441881" & year==2001

replace regioncode="441882" if regioncode=="441882" & year==2001

replace regioncode="441900" if regioncode=="441900" & year==2001

replace regioncode="442000" if regioncode=="442000" & year==2001

replace regioncode="445102" if regioncode=="445102" & year==2001

replace regioncode="445121" if regioncode=="445121" & year==2001

replace regioncode="445122" if regioncode=="445122" & year==2001

replace regioncode="445202" if regioncode=="445202" & year==2001

replace regioncode="445221" if regioncode=="445221" & year==2001

replace regioncode="445222" if regioncode=="445222" & year==2001

replace regioncode="445224" if regioncode=="445224" & year==2001

replace regioncode="445281" if regioncode=="445281" & year==2001

replace regioncode="445302" if regioncode=="445302" & year==2001

replace regioncode="445321" if regioncode=="445321" & year==2001

replace regioncode="445322" if regioncode=="445322" & year==2001

replace regioncode="445323" if regioncode=="445323" & year==2001

replace regioncode="445381" if regioncode=="445381" & year==2001

replace regioncode="450102" if regioncode=="450102" & year==2001

replace regioncode="450103" if regioncode=="450103" & year==2001

replace regioncode="450104" if regioncode=="450104" & year==2001

replace regioncode="450105" if regioncode=="450105" & year==2001

replace regioncode="450106" if regioncode=="450106" & year==2001

replace regioncode="450121" if regioncode=="450121" & year==2001

replace regioncode="450122" if regioncode=="450122" & year==2001

replace regioncode="450123" if regioncode=="452126" & year==2001

replace regioncode="450124" if regioncode=="452127" & year==2001

replace regioncode="450125" if regioncode=="452124" & year==2001

replace regioncode="450126" if regioncode=="452123" & year==2001

replace regioncode="450127" if regioncode=="452122" & year==2001

replace regioncode="450202" if regioncode=="450202" & year==2001

replace regioncode="450203" if regioncode=="450203" & year==2001

replace regioncode="450204" if regioncode=="450204" & year==2001

replace regioncode="450205" if regioncode=="450205" & year==2001

replace regioncode="450221" if regioncode=="450221" & year==2001

replace regioncode="450222" if regioncode=="450222" & year==2001

replace regioncode="450223" if regioncode=="452223" & year==2001

replace regioncode="450224" if regioncode=="452227" & year==2001

replace regioncode="450225" if regioncode=="452229" & year==2001

replace regioncode="450226" if regioncode=="452228" & year==2001

replace regioncode="450302" if regioncode=="450302" & year==2001

replace regioncode="450303" if regioncode=="450303" & year==2001

replace regioncode="450304" if regioncode=="450304" & year==2001

replace regioncode="450305" if regioncode=="450305" & year==2001

replace regioncode="450311" if regioncode=="450311" & year==2001

replace regioncode="450321" if regioncode=="450321" & year==2001

replace regioncode="450322" if regioncode=="450322" & year==2001

replace regioncode="450323" if regioncode=="450323" & year==2001

replace regioncode="450324" if regioncode=="450324" & year==2001

replace regioncode="450325" if regioncode=="450325" & year==2001

replace regioncode="450326" if regioncode=="450326" & year==2001

replace regioncode="450327" if regioncode=="450327" & year==2001

replace regioncode="450328" if regioncode=="450328" & year==2001

replace regioncode="450329" if regioncode=="450329" & year==2001

replace regioncode="450330" if regioncode=="450330" & year==2001

replace regioncode="450331" if regioncode=="450331" & year==2001

replace regioncode="450332" if regioncode=="450332" & year==2001

replace regioncode="450403" if regioncode=="450403" & year==2001

replace regioncode="450404" if regioncode=="450404" & year==2001

replace regioncode="450421" if regioncode=="450421" & year==2001

replace regioncode="450422" if regioncode=="450422" & year==2001

replace regioncode="450423" if regioncode=="450423" & year==2001

replace regioncode="450481" if regioncode=="450481" & year==2001

replace regioncode="450502" if regioncode=="450502" & year==2001

replace regioncode="450503" if regioncode=="450503" & year==2001

replace regioncode="450512" if regioncode=="450504" & year==2001

replace regioncode="450521" if regioncode=="450521" & year==2001

replace regioncode="450602" if regioncode=="450602" & year==2001

replace regioncode="450603" if regioncode=="450603" & year==2001

replace regioncode="450621" if regioncode=="450621" & year==2001

replace regioncode="450681" if regioncode=="450681" & year==2001

replace regioncode="450702" if regioncode=="450702" & year==2001

replace regioncode="450703" if regioncode=="450703" & year==2001

replace regioncode="450721" if regioncode=="450721" & year==2001

replace regioncode="450722" if regioncode=="450722" & year==2001

replace regioncode="450802" if regioncode=="450802" & year==2001

replace regioncode="450803" if regioncode=="450803" & year==2001

replace regioncode="450804" if regioncode=="450804" & year==2001

replace regioncode="450821" if regioncode=="450821" & year==2001

replace regioncode="450881" if regioncode=="450881" & year==2001

replace regioncode="450902" if regioncode=="450902" & year==2001

replace regioncode="450921" if regioncode=="450921" & year==2001

replace regioncode="450922" if regioncode=="450922" & year==2001

replace regioncode="450923" if regioncode=="450923" & year==2001

replace regioncode="450924" if regioncode=="450924" & year==2001

replace regioncode="450981" if regioncode=="450981" & year==2001

replace regioncode="451002" if regioncode=="452601" & year==2001

replace regioncode="451021" if regioncode=="452622" & year==2001

replace regioncode="451022" if regioncode=="452623" & year==2001

replace regioncode="451023" if regioncode=="452624" & year==2001

replace regioncode="451024" if regioncode=="452625" & year==2001

replace regioncode="451025" if regioncode=="452626" & year==2001

replace regioncode="451026" if regioncode=="452627" & year==2001

replace regioncode="451027" if regioncode=="452628" & year==2001

replace regioncode="451028" if regioncode=="452629" & year==2001

replace regioncode="451029" if regioncode=="452630" & year==2001

replace regioncode="451030" if regioncode=="452632" & year==2001

replace regioncode="451031" if regioncode=="452631" & year==2001

replace regioncode="451102" if regioncode=="452402" & year==2001

replace regioncode="451121" if regioncode=="452424" & year==2001

replace regioncode="451122" if regioncode=="452427" & year==2001

replace regioncode="451123" if regioncode=="452428" & year==2001

replace regioncode="451202" if regioncode=="452701" & year==2001

replace regioncode="451221" if regioncode=="452725" & year==2001

replace regioncode="451222" if regioncode=="452726" & year==2001

replace regioncode="451223" if regioncode=="452727" & year==2001

replace regioncode="451224" if regioncode=="452728" & year==2001

replace regioncode="451225" if regioncode=="452723" & year==2001

replace regioncode="451226" if regioncode=="452724" & year==2001

replace regioncode="451227" if regioncode=="452729" & year==2001

replace regioncode="451228" if regioncode=="452730" & year==2001

replace regioncode="451229" if regioncode=="452731" & year==2001

replace regioncode="451281" if regioncode=="452702" & year==2001

replace regioncode="451302" if regioncode=="452226" & year==2001

replace regioncode="451321" if regioncode=="452231" & year==2001

replace regioncode="451322" if regioncode=="452224" & year==2001

replace regioncode="451323" if regioncode=="452225" & year==2001

replace regioncode="451324" if regioncode=="452230" & year==2001

replace regioncode="451381" if regioncode=="452201" & year==2001

replace regioncode="451402" if regioncode=="452129" & year==2001

replace regioncode="451421" if regioncode=="452128" & year==2001

replace regioncode="451422" if regioncode=="452132" & year==2001

replace regioncode="451423" if regioncode=="452133" & year==2001

replace regioncode="451424" if regioncode=="452130" & year==2001

replace regioncode="451425" if regioncode=="452131" & year==2001

replace regioncode="451481" if regioncode=="452101" & year==2001

replace regioncode="460105" if regioncode=="460102" & year==2001

replace regioncode="460106" if regioncode=="460103" & year==2001

replace regioncode="460107" if regioncode=="460104" & year==2001

replace regioncode="460108" if regioncode=="469004" & year==2001

replace regioncode="460001" if regioncode=="460001" & year==2001

replace regioncode="460002" if regioncode=="460002" & year==2001

replace regioncode="460003" if regioncode=="460003" & year==2001

replace regioncode="460005" if regioncode=="460005" & year==2001

replace regioncode="460006" if regioncode=="460006" & year==2001

replace regioncode="460007" if regioncode=="460007" & year==2001

replace regioncode="460030" if regioncode=="460030" & year==2001

replace regioncode="460031" if regioncode=="460031" & year==2001

replace regioncode="460033" if regioncode=="460033" & year==2001

replace regioncode="460034" if regioncode=="460034" & year==2001

replace regioncode="460036" if regioncode=="460036" & year==2001

replace regioncode="500101" if regioncode=="500101" & year==2001

replace regioncode="500102" if regioncode=="500102" & year==2001

replace regioncode="500103" if regioncode=="500103" & year==2001

replace regioncode="500104" if regioncode=="500104" & year==2001

replace regioncode="500105" if regioncode=="500105" & year==2001

replace regioncode="500106" if regioncode=="500106" & year==2001

replace regioncode="500107" if regioncode=="500107" & year==2001

replace regioncode="500108" if regioncode=="500108" & year==2001

replace regioncode="500109" if regioncode=="500109" & year==2001

replace regioncode="500110" if regioncode=="500110" & year==2001

replace regioncode="500111" if regioncode=="500111" & year==2001

replace regioncode="500112" if regioncode=="500112" & year==2001

replace regioncode="500113" if regioncode=="500113" & year==2001

replace regioncode="500114" if regioncode=="500114" & year==2001

replace regioncode="500115" if regioncode=="500221" & year==2001

replace regioncode="500381" if regioncode=="500381" & year==2001

replace regioncode="500382" if regioncode=="500382" & year==2001

replace regioncode="500383" if regioncode=="500383" & year==2001

replace regioncode="500384" if regioncode=="500384" & year==2001

replace regioncode="500222" if regioncode=="500222" & year==2001

replace regioncode="500223" if regioncode=="500223" & year==2001

replace regioncode="500224" if regioncode=="500224" & year==2001

replace regioncode="500225" if regioncode=="500225" & year==2001

replace regioncode="500226" if regioncode=="500226" & year==2001

replace regioncode="500227" if regioncode=="500227" & year==2001

replace regioncode="500228" if regioncode=="500228" & year==2001

replace regioncode="500229" if regioncode=="500229" & year==2001

replace regioncode="500230" if regioncode=="500230" & year==2001

replace regioncode="500231" if regioncode=="500231" & year==2001

replace regioncode="500232" if regioncode=="500232" & year==2001

replace regioncode="500233" if regioncode=="500233" & year==2001

replace regioncode="500234" if regioncode=="500234" & year==2001

replace regioncode="500235" if regioncode=="500235" & year==2001

replace regioncode="500236" if regioncode=="500236" & year==2001

replace regioncode="500237" if regioncode=="500237" & year==2001

replace regioncode="500238" if regioncode=="500238" & year==2001

replace regioncode="500240" if regioncode=="500240" & year==2001

replace regioncode="500241" if regioncode=="500241" & year==2001

replace regioncode="500242" if regioncode=="500242" & year==2001

replace regioncode="500243" if regioncode=="500243" & year==2001

replace regioncode="510104" if regioncode=="510104" & year==2001

replace regioncode="510105" if regioncode=="510105" & year==2001

replace regioncode="510106" if regioncode=="510106" & year==2001

replace regioncode="510107" if regioncode=="510107" & year==2001

replace regioncode="510108" if regioncode=="510108" & year==2001

replace regioncode="510112" if regioncode=="510112" & year==2001

replace regioncode="510113" if regioncode=="510113" & year==2001

replace regioncode="510114" if regioncode=="510125" & year==2001

replace regioncode="510115" if regioncode=="510123" & year==2001

replace regioncode="510121" if regioncode=="510121" & year==2001

replace regioncode="510122" if regioncode=="510122" & year==2001

replace regioncode="510124" if regioncode=="510124" & year==2001

replace regioncode="510129" if regioncode=="510129" & year==2001

replace regioncode="510131" if regioncode=="510131" & year==2001

replace regioncode="510132" if regioncode=="510132" & year==2001

replace regioncode="510181" if regioncode=="510181" & year==2001

replace regioncode="510182" if regioncode=="510182" & year==2001

replace regioncode="510183" if regioncode=="510183" & year==2001

replace regioncode="510184" if regioncode=="510184" & year==2001

replace regioncode="510302" if regioncode=="510302" & year==2001

replace regioncode="510303" if regioncode=="510303" & year==2001

replace regioncode="510304" if regioncode=="510304" & year==2001

replace regioncode="510311" if regioncode=="510311" & year==2001

replace regioncode="510321" if regioncode=="510321" & year==2001

replace regioncode="510322" if regioncode=="510322" & year==2001

replace regioncode="510402" if regioncode=="510402" & year==2001

replace regioncode="510403" if regioncode=="510403" & year==2001

replace regioncode="510411" if regioncode=="510411" & year==2001

replace regioncode="510421" if regioncode=="510421" & year==2001

replace regioncode="510422" if regioncode=="510422" & year==2001

replace regioncode="510502" if regioncode=="510502" & year==2001

replace regioncode="510503" if regioncode=="510503" & year==2001

replace regioncode="510504" if regioncode=="510504" & year==2001

replace regioncode="510521" if regioncode=="510521" & year==2001

replace regioncode="510522" if regioncode=="510522" & year==2001

replace regioncode="510524" if regioncode=="510524" & year==2001

replace regioncode="510525" if regioncode=="510525" & year==2001

replace regioncode="510603" if regioncode=="510603" & year==2001

replace regioncode="510623" if regioncode=="510623" & year==2001

replace regioncode="510626" if regioncode=="510626" & year==2001

replace regioncode="510681" if regioncode=="510681" & year==2001

replace regioncode="510682" if regioncode=="510682" & year==2001

replace regioncode="510683" if regioncode=="510683" & year==2001

replace regioncode="510703" if regioncode=="510703" & year==2001

replace regioncode="510704" if regioncode=="510704" & year==2001

replace regioncode="510722" if regioncode=="510722" & year==2001

replace regioncode="510723" if regioncode=="510723" & year==2001

replace regioncode="510724" if regioncode=="510724" & year==2001

replace regioncode="510725" if regioncode=="510725" & year==2001

replace regioncode="510726" if regioncode=="510726" & year==2001

replace regioncode="510727" if regioncode=="510727" & year==2001

replace regioncode="510781" if regioncode=="510781" & year==2001

replace regioncode="510802" if regioncode=="510802" & year==2001

replace regioncode="510811" if regioncode=="510811" & year==2001

replace regioncode="510812" if regioncode=="510812" & year==2001

replace regioncode="510821" if regioncode=="510821" & year==2001

replace regioncode="510822" if regioncode=="510822" & year==2001

replace regioncode="510823" if regioncode=="510823" & year==2001

replace regioncode="510824" if regioncode=="510824" & year==2001

replace regioncode="510902" if regioncode=="510902" & year==2001

replace regioncode="510921" if regioncode=="510921" & year==2001

replace regioncode="510922" if regioncode=="510922" & year==2001

replace regioncode="510923" if regioncode=="510923" & year==2001

replace regioncode="511002" if regioncode=="511002" & year==2001

replace regioncode="511011" if regioncode=="511011" & year==2001

replace regioncode="511024" if regioncode=="511024" & year==2001

replace regioncode="511025" if regioncode=="511025" & year==2001

replace regioncode="511028" if regioncode=="511028" & year==2001

replace regioncode="511102" if regioncode=="511102" & year==2001

replace regioncode="511111" if regioncode=="511111" & year==2001

replace regioncode="511112" if regioncode=="511112" & year==2001

replace regioncode="511113" if regioncode=="511113" & year==2001

replace regioncode="511123" if regioncode=="511123" & year==2001

replace regioncode="511124" if regioncode=="511124" & year==2001

replace regioncode="511126" if regioncode=="511126" & year==2001

replace regioncode="511129" if regioncode=="511129" & year==2001

replace regioncode="511132" if regioncode=="511132" & year==2001

replace regioncode="511133" if regioncode=="511133" & year==2001

replace regioncode="511181" if regioncode=="511181" & year==2001

replace regioncode="511302" if regioncode=="511302" & year==2001

replace regioncode="511303" if regioncode=="511303" & year==2001

replace regioncode="511304" if regioncode=="511304" & year==2001

replace regioncode="511321" if regioncode=="511321" & year==2001

replace regioncode="511322" if regioncode=="511322" & year==2001

replace regioncode="511323" if regioncode=="511323" & year==2001

replace regioncode="511324" if regioncode=="511324" & year==2001

replace regioncode="511325" if regioncode=="511325" & year==2001

replace regioncode="511381" if regioncode=="511381" & year==2001

replace regioncode="511402" if regioncode=="511402" & year==2001

replace regioncode="511421" if regioncode=="511421" & year==2001

replace regioncode="511422" if regioncode=="511422" & year==2001

replace regioncode="511423" if regioncode=="511423" & year==2001

replace regioncode="511424" if regioncode=="511424" & year==2001

replace regioncode="511425" if regioncode=="511425" & year==2001

replace regioncode="511502" if regioncode=="511502" & year==2001

replace regioncode="511521" if regioncode=="511521" & year==2001

replace regioncode="511522" if regioncode=="511522" & year==2001

replace regioncode="511523" if regioncode=="511523" & year==2001

replace regioncode="511524" if regioncode=="511524" & year==2001

replace regioncode="511525" if regioncode=="511525" & year==2001

replace regioncode="511526" if regioncode=="511526" & year==2001

replace regioncode="511527" if regioncode=="511527" & year==2001

replace regioncode="511528" if regioncode=="511528" & year==2001

replace regioncode="511529" if regioncode=="511529" & year==2001

replace regioncode="511602" if regioncode=="511602" & year==2001

replace regioncode="511621" if regioncode=="511621" & year==2001

replace regioncode="511622" if regioncode=="511622" & year==2001

replace regioncode="511623" if regioncode=="511623" & year==2001

replace regioncode="511681" if regioncode=="511681" & year==2001

replace regioncode="511702" if regioncode=="511702" & year==2001

replace regioncode="511721" if regioncode=="511721" & year==2001

replace regioncode="511722" if regioncode=="511722" & year==2001

replace regioncode="511723" if regioncode=="511723" & year==2001

replace regioncode="511724" if regioncode=="511724" & year==2001

replace regioncode="511725" if regioncode=="511725" & year==2001

replace regioncode="511781" if regioncode=="511781" & year==2001

replace regioncode="511802" if regioncode=="511802" & year==2001

replace regioncode="511821" if regioncode=="511821" & year==2001

replace regioncode="511822" if regioncode=="511822" & year==2001

replace regioncode="511823" if regioncode=="511823" & year==2001

replace regioncode="511824" if regioncode=="511824" & year==2001

replace regioncode="511825" if regioncode=="511825" & year==2001

replace regioncode="511826" if regioncode=="511826" & year==2001

replace regioncode="511827" if regioncode=="511827" & year==2001

replace regioncode="511902" if regioncode=="511902" & year==2001

replace regioncode="511921" if regioncode=="511921" & year==2001

replace regioncode="511922" if regioncode=="511922" & year==2001

replace regioncode="511923" if regioncode=="511923" & year==2001

replace regioncode="512002" if regioncode=="512002" & year==2001

replace regioncode="512021" if regioncode=="512021" & year==2001

replace regioncode="512022" if regioncode=="512022" & year==2001

replace regioncode="512081" if regioncode=="512081" & year==2001

replace regioncode="513221" if regioncode=="513221" & year==2001

replace regioncode="513222" if regioncode=="513222" & year==2001

replace regioncode="513223" if regioncode=="513223" & year==2001

replace regioncode="513224" if regioncode=="513224" & year==2001

replace regioncode="513225" if regioncode=="513225" & year==2001

replace regioncode="513226" if regioncode=="513226" & year==2001

replace regioncode="513227" if regioncode=="513227" & year==2001

replace regioncode="513228" if regioncode=="513228" & year==2001

replace regioncode="513229" if regioncode=="513229" & year==2001

replace regioncode="513230" if regioncode=="513230" & year==2001

replace regioncode="513231" if regioncode=="513231" & year==2001

replace regioncode="513232" if regioncode=="513232" & year==2001

replace regioncode="513233" if regioncode=="513233" & year==2001

replace regioncode="513321" if regioncode=="513321" & year==2001

replace regioncode="513322" if regioncode=="513322" & year==2001

replace regioncode="513323" if regioncode=="513323" & year==2001

replace regioncode="513324" if regioncode=="513324" & year==2001

replace regioncode="513325" if regioncode=="513325" & year==2001

replace regioncode="513326" if regioncode=="513326" & year==2001

replace regioncode="513327" if regioncode=="513327" & year==2001

replace regioncode="513328" if regioncode=="513328" & year==2001

replace regioncode="513329" if regioncode=="513329" & year==2001

replace regioncode="513330" if regioncode=="513330" & year==2001

replace regioncode="513331" if regioncode=="513331" & year==2001

replace regioncode="513332" if regioncode=="513332" & year==2001

replace regioncode="513333" if regioncode=="513333" & year==2001

replace regioncode="513334" if regioncode=="513334" & year==2001

replace regioncode="513335" if regioncode=="513335" & year==2001

replace regioncode="513336" if regioncode=="513336" & year==2001

replace regioncode="513337" if regioncode=="513337" & year==2001

replace regioncode="513338" if regioncode=="513338" & year==2001

replace regioncode="513401" if regioncode=="513401" & year==2001

replace regioncode="513422" if regioncode=="513422" & year==2001

replace regioncode="513423" if regioncode=="513423" & year==2001

replace regioncode="513424" if regioncode=="513424" & year==2001

replace regioncode="513425" if regioncode=="513425" & year==2001

replace regioncode="513426" if regioncode=="513426" & year==2001

replace regioncode="513427" if regioncode=="513427" & year==2001

replace regioncode="513428" if regioncode=="513428" & year==2001

replace regioncode="513429" if regioncode=="513429" & year==2001

replace regioncode="513430" if regioncode=="513430" & year==2001

replace regioncode="513431" if regioncode=="513431" & year==2001

replace regioncode="513432" if regioncode=="513432" & year==2001

replace regioncode="513433" if regioncode=="513433" & year==2001

replace regioncode="513434" if regioncode=="513434" & year==2001

replace regioncode="513435" if regioncode=="513435" & year==2001

replace regioncode="513436" if regioncode=="513436" & year==2001

replace regioncode="513437" if regioncode=="513437" & year==2001

replace regioncode="520102" if regioncode=="520102" & year==2001

replace regioncode="520103" if regioncode=="520103" & year==2001

replace regioncode="520111" if regioncode=="520111" & year==2001

replace regioncode="520112" if regioncode=="520112" & year==2001

replace regioncode="520113" if regioncode=="520113" & year==2001

replace regioncode="520114" if regioncode=="520114" & year==2001

replace regioncode="520121" if regioncode=="520121" & year==2001

replace regioncode="520122" if regioncode=="520122" & year==2001

replace regioncode="520123" if regioncode=="520123" & year==2001

replace regioncode="520181" if regioncode=="520181" & year==2001

replace regioncode="520201" if regioncode=="520201" & year==2001

replace regioncode="520222" if regioncode=="520222" & year==2001

replace regioncode="520203" if regioncode=="520203" & year==2001

replace regioncode="520221" if regioncode=="520221" & year==2001

replace regioncode="520302" if regioncode=="520302" & year==2001

replace regioncode="520321" if regioncode=="520321" & year==2001

replace regioncode="520322" if regioncode=="520322" & year==2001

replace regioncode="520323" if regioncode=="520323" & year==2001

replace regioncode="520324" if regioncode=="520324" & year==2001

replace regioncode="520325" if regioncode=="520325" & year==2001

replace regioncode="520326" if regioncode=="520326" & year==2001

replace regioncode="520327" if regioncode=="520327" & year==2001

replace regioncode="520328" if regioncode=="520328" & year==2001

replace regioncode="520329" if regioncode=="520329" & year==2001

replace regioncode="520330" if regioncode=="520330" & year==2001

replace regioncode="520381" if regioncode=="520381" & year==2001

replace regioncode="520382" if regioncode=="520382" & year==2001

replace regioncode="520402" if regioncode=="520402" & year==2001

replace regioncode="520421" if regioncode=="520421" & year==2001

replace regioncode="520422" if regioncode=="520422" & year==2001

replace regioncode="520423" if regioncode=="520423" & year==2001

replace regioncode="520424" if regioncode=="520424" & year==2001

replace regioncode="520425" if regioncode=="520425" & year==2001

replace regioncode="522201" if regioncode=="522201" & year==2001

replace regioncode="522222" if regioncode=="522222" & year==2001

replace regioncode="522223" if regioncode=="522223" & year==2001

replace regioncode="522224" if regioncode=="522224" & year==2001

replace regioncode="522225" if regioncode=="522225" & year==2001

replace regioncode="522226" if regioncode=="522226" & year==2001

replace regioncode="522227" if regioncode=="522227" & year==2001

replace regioncode="522228" if regioncode=="522228" & year==2001

replace regioncode="522229" if regioncode=="522229" & year==2001

replace regioncode="522230" if regioncode=="522230" & year==2001

replace regioncode="522301" if regioncode=="522301" & year==2001

replace regioncode="522322" if regioncode=="522322" & year==2001

replace regioncode="522323" if regioncode=="522323" & year==2001

replace regioncode="522324" if regioncode=="522324" & year==2001

replace regioncode="522325" if regioncode=="522325" & year==2001

replace regioncode="522326" if regioncode=="522326" & year==2001

replace regioncode="522327" if regioncode=="522327" & year==2001

replace regioncode="522328" if regioncode=="522328" & year==2001

replace regioncode="522401" if regioncode=="522401" & year==2001

replace regioncode="522422" if regioncode=="522422" & year==2001

replace regioncode="522423" if regioncode=="522423" & year==2001

replace regioncode="522424" if regioncode=="522424" & year==2001

replace regioncode="522425" if regioncode=="522425" & year==2001

replace regioncode="522426" if regioncode=="522426" & year==2001

replace regioncode="522427" if regioncode=="522427" & year==2001

replace regioncode="522428" if regioncode=="522428" & year==2001

replace regioncode="522601" if regioncode=="522601" & year==2001

replace regioncode="522622" if regioncode=="522622" & year==2001

replace regioncode="522623" if regioncode=="522623" & year==2001

replace regioncode="522624" if regioncode=="522624" & year==2001

replace regioncode="522625" if regioncode=="522625" & year==2001

replace regioncode="522626" if regioncode=="522626" & year==2001

replace regioncode="522627" if regioncode=="522627" & year==2001

replace regioncode="522628" if regioncode=="522628" & year==2001

replace regioncode="522629" if regioncode=="522629" & year==2001

replace regioncode="522630" if regioncode=="522630" & year==2001

replace regioncode="522631" if regioncode=="522631" & year==2001

replace regioncode="522632" if regioncode=="522632" & year==2001

replace regioncode="522633" if regioncode=="522633" & year==2001

replace regioncode="522634" if regioncode=="522634" & year==2001

replace regioncode="522635" if regioncode=="522635" & year==2001

replace regioncode="522636" if regioncode=="522636" & year==2001

replace regioncode="522701" if regioncode=="522701" & year==2001

replace regioncode="522702" if regioncode=="522702" & year==2001

replace regioncode="522722" if regioncode=="522722" & year==2001

replace regioncode="522723" if regioncode=="522723" & year==2001

replace regioncode="522725" if regioncode=="522725" & year==2001

replace regioncode="522726" if regioncode=="522726" & year==2001

replace regioncode="522727" if regioncode=="522727" & year==2001

replace regioncode="522728" if regioncode=="522728" & year==2001

replace regioncode="522729" if regioncode=="522729" & year==2001

replace regioncode="522730" if regioncode=="522730" & year==2001

replace regioncode="522731" if regioncode=="522731" & year==2001

replace regioncode="522732" if regioncode=="522732" & year==2001

replace regioncode="530102" if regioncode=="530102" & year==2001

replace regioncode="530103" if regioncode=="530103" & year==2001

replace regioncode="530111" if regioncode=="530111" & year==2001

replace regioncode="530112" if regioncode=="530112" & year==2001

replace regioncode="530113" if regioncode=="530113" & year==2001

replace regioncode="530121" if regioncode=="530121" & year==2001

replace regioncode="530122" if regioncode=="530122" & year==2001

replace regioncode="530124" if regioncode=="530124" & year==2001

replace regioncode="530125" if regioncode=="530125" & year==2001

replace regioncode="530126" if regioncode=="530126" & year==2001

replace regioncode="530127" if regioncode=="530127" & year==2001

replace regioncode="530128" if regioncode=="530128" & year==2001

replace regioncode="530129" if regioncode=="530129" & year==2001

replace regioncode="530181" if regioncode=="530181" & year==2001

replace regioncode="530302" if regioncode=="530302" & year==2001

replace regioncode="530321" if regioncode=="530321" & year==2001

replace regioncode="530322" if regioncode=="530322" & year==2001

replace regioncode="530323" if regioncode=="530323" & year==2001

replace regioncode="530324" if regioncode=="530324" & year==2001

replace regioncode="530325" if regioncode=="530325" & year==2001

replace regioncode="530326" if regioncode=="530326" & year==2001

replace regioncode="530328" if regioncode=="530328" & year==2001

replace regioncode="530381" if regioncode=="530381" & year==2001

replace regioncode="530402" if regioncode=="530402" & year==2001

replace regioncode="530421" if regioncode=="530421" & year==2001

replace regioncode="530422" if regioncode=="530422" & year==2001

replace regioncode="530423" if regioncode=="530423" & year==2001

replace regioncode="530424" if regioncode=="530424" & year==2001

replace regioncode="530425" if regioncode=="530425" & year==2001

replace regioncode="530426" if regioncode=="530426" & year==2001

replace regioncode="530427" if regioncode=="530427" & year==2001

replace regioncode="530428" if regioncode=="530428" & year==2001

replace regioncode="530502" if regioncode=="530502" & year==2001

replace regioncode="530521" if regioncode=="530521" & year==2001

replace regioncode="530522" if regioncode=="530522" & year==2001

replace regioncode="530523" if regioncode=="530523" & year==2001

replace regioncode="530524" if regioncode=="530524" & year==2001

replace regioncode="530602" if regioncode=="530602" & year==2001

replace regioncode="530621" if regioncode=="530621" & year==2001

replace regioncode="530622" if regioncode=="530622" & year==2001

replace regioncode="530623" if regioncode=="530623" & year==2001

replace regioncode="530624" if regioncode=="530624" & year==2001

replace regioncode="530625" if regioncode=="530625" & year==2001

replace regioncode="530626" if regioncode=="530626" & year==2001

replace regioncode="530627" if regioncode=="530627" & year==2001

replace regioncode="530628" if regioncode=="530628" & year==2001

replace regioncode="530629" if regioncode=="530629" & year==2001

replace regioncode="530630" if regioncode=="530630" & year==2001

replace regioncode="530721" if regioncode=="533221" & year==2001

replace regioncode="530722" if regioncode=="533222" & year==2001

replace regioncode="530723" if regioncode=="533223" & year==2001

replace regioncode="530724" if regioncode=="533224" & year==2001

replace regioncode="532701" if regioncode=="532701" & year==2001

replace regioncode="532722" if regioncode=="532722" & year==2001

replace regioncode="532723" if regioncode=="532723" & year==2001

replace regioncode="532724" if regioncode=="532724" & year==2001

replace regioncode="532725" if regioncode=="532725" & year==2001

replace regioncode="532726" if regioncode=="532726" & year==2001

replace regioncode="532727" if regioncode=="532727" & year==2001

replace regioncode="532728" if regioncode=="532728" & year==2001

replace regioncode="532729" if regioncode=="532729" & year==2001

replace regioncode="532730" if regioncode=="532730" & year==2001

replace regioncode="533521" if regioncode=="533521" & year==2001

replace regioncode="533522" if regioncode=="533522" & year==2001

replace regioncode="533523" if regioncode=="533523" & year==2001

replace regioncode="533524" if regioncode=="533524" & year==2001

replace regioncode="533525" if regioncode=="533525" & year==2001

replace regioncode="533526" if regioncode=="533526" & year==2001

replace regioncode="533527" if regioncode=="533527" & year==2001

replace regioncode="533528" if regioncode=="533528" & year==2001

replace regioncode="532301" if regioncode=="532301" & year==2001

replace regioncode="532322" if regioncode=="532322" & year==2001

replace regioncode="532323" if regioncode=="532323" & year==2001

replace regioncode="532324" if regioncode=="532324" & year==2001

replace regioncode="532325" if regioncode=="532325" & year==2001

replace regioncode="532326" if regioncode=="532326" & year==2001

replace regioncode="532327" if regioncode=="532327" & year==2001

replace regioncode="532328" if regioncode=="532328" & year==2001

replace regioncode="532329" if regioncode=="532329" & year==2001

replace regioncode="532331" if regioncode=="532331" & year==2001

replace regioncode="532501" if regioncode=="532501" & year==2001

replace regioncode="532502" if regioncode=="532502" & year==2001

replace regioncode="532522" if regioncode=="532522" & year==2001

replace regioncode="532523" if regioncode=="532523" & year==2001

replace regioncode="532524" if regioncode=="532524" & year==2001

replace regioncode="532525" if regioncode=="532525" & year==2001

replace regioncode="532526" if regioncode=="532526" & year==2001

replace regioncode="532527" if regioncode=="532527" & year==2001

replace regioncode="532528" if regioncode=="532528" & year==2001

replace regioncode="532529" if regioncode=="532529" & year==2001

replace regioncode="532530" if regioncode=="532530" & year==2001

replace regioncode="532531" if regioncode=="532531" & year==2001

replace regioncode="532532" if regioncode=="532532" & year==2001

replace regioncode="532621" if regioncode=="532621" & year==2001

replace regioncode="532622" if regioncode=="532622" & year==2001

replace regioncode="532623" if regioncode=="532623" & year==2001

replace regioncode="532624" if regioncode=="532624" & year==2001

replace regioncode="532625" if regioncode=="532625" & year==2001

replace regioncode="532626" if regioncode=="532626" & year==2001

replace regioncode="532627" if regioncode=="532627" & year==2001

replace regioncode="532628" if regioncode=="532628" & year==2001

replace regioncode="532801" if regioncode=="532801" & year==2001

replace regioncode="532822" if regioncode=="532822" & year==2001

replace regioncode="532823" if regioncode=="532823" & year==2001

replace regioncode="532901" if regioncode=="532901" & year==2001

replace regioncode="532922" if regioncode=="532922" & year==2001

replace regioncode="532923" if regioncode=="532923" & year==2001

replace regioncode="532924" if regioncode=="532924" & year==2001

replace regioncode="532925" if regioncode=="532925" & year==2001

replace regioncode="532926" if regioncode=="532926" & year==2001

replace regioncode="532927" if regioncode=="532927" & year==2001

replace regioncode="532928" if regioncode=="532928" & year==2001

replace regioncode="532929" if regioncode=="532929" & year==2001

replace regioncode="532930" if regioncode=="532930" & year==2001

replace regioncode="532931" if regioncode=="532931" & year==2001

replace regioncode="532932" if regioncode=="532932" & year==2001

replace regioncode="533102" if regioncode=="533102" & year==2001

replace regioncode="533103" if regioncode=="533103" & year==2001

replace regioncode="533122" if regioncode=="533122" & year==2001

replace regioncode="533123" if regioncode=="533123" & year==2001

replace regioncode="533124" if regioncode=="533124" & year==2001

replace regioncode="533321" if regioncode=="533321" & year==2001

replace regioncode="533323" if regioncode=="533323" & year==2001

replace regioncode="533324" if regioncode=="533324" & year==2001

replace regioncode="533325" if regioncode=="533325" & year==2001

replace regioncode="533421" if regioncode=="533421" & year==2001

replace regioncode="533422" if regioncode=="533422" & year==2001

replace regioncode="533423" if regioncode=="533423" & year==2001

replace regioncode="540102" if regioncode=="540102" & year==2001

replace regioncode="540121" if regioncode=="540121" & year==2001

replace regioncode="540122" if regioncode=="540122" & year==2001

replace regioncode="540123" if regioncode=="540123" & year==2001

replace regioncode="540124" if regioncode=="540124" & year==2001

replace regioncode="540125" if regioncode=="540125" & year==2001

replace regioncode="540126" if regioncode=="540126" & year==2001

replace regioncode="540127" if regioncode=="540127" & year==2001

replace regioncode="542121" if regioncode=="542121" & year==2001

replace regioncode="542122" if regioncode=="542122" & year==2001

replace regioncode="542123" if regioncode=="542123" & year==2001

replace regioncode="542124" if regioncode=="542124" & year==2001

replace regioncode="542125" if regioncode=="542125" & year==2001

replace regioncode="542126" if regioncode=="542126" & year==2001

replace regioncode="542127" if regioncode=="542127" & year==2001

replace regioncode="542128" if regioncode=="542128" & year==2001

replace regioncode="542129" if regioncode=="542129" & year==2001

replace regioncode="542132" if regioncode=="542132" & year==2001

replace regioncode="542133" if regioncode=="542133" & year==2001

replace regioncode="542221" if regioncode=="542221" & year==2001

replace regioncode="542223" if regioncode=="542223" & year==2001

replace regioncode="542224" if regioncode=="542224" & year==2001

replace regioncode="542225" if regioncode=="542225" & year==2001

replace regioncode="542227" if regioncode=="542227" & year==2001

replace regioncode="542228" if regioncode=="542228" & year==2001

replace regioncode="542229" if regioncode=="542229" & year==2001

replace regioncode="542231" if regioncode=="542231" & year==2001

replace regioncode="542232" if regioncode=="542232" & year==2001

replace regioncode="542233" if regioncode=="542233" & year==2001

replace regioncode="542301" if regioncode=="542301" & year==2001

replace regioncode="542322" if regioncode=="542322" & year==2001

replace regioncode="542323" if regioncode=="542323" & year==2001

replace regioncode="542324" if regioncode=="542324" & year==2001

replace regioncode="542325" if regioncode=="542325" & year==2001

replace regioncode="542326" if regioncode=="542326" & year==2001

replace regioncode="542327" if regioncode=="542327" & year==2001

replace regioncode="542328" if regioncode=="542328" & year==2001

replace regioncode="542329" if regioncode=="542329" & year==2001

replace regioncode="542330" if regioncode=="542330" & year==2001

replace regioncode="542331" if regioncode=="542331" & year==2001

replace regioncode="542332" if regioncode=="542332" & year==2001

replace regioncode="542334" if regioncode=="542334" & year==2001

replace regioncode="542336" if regioncode=="542336" & year==2001

replace regioncode="542338" if regioncode=="542338" & year==2001

replace regioncode="542421" if regioncode=="542421" & year==2001

replace regioncode="542422" if regioncode=="542422" & year==2001

replace regioncode="542423" if regioncode=="542423" & year==2001

replace regioncode="542424" if regioncode=="542424" & year==2001

replace regioncode="542425" if regioncode=="542425" & year==2001

replace regioncode="542426" if regioncode=="542426" & year==2001

replace regioncode="542427" if regioncode=="542427" & year==2001

replace regioncode="542429" if regioncode=="542429" & year==2001

replace regioncode="542430" if regioncode=="542430" & year==2001

replace regioncode="542523" if regioncode=="542523" & year==2001

replace regioncode="542621" if regioncode=="542621" & year==2001

replace regioncode="542622" if regioncode=="542622" & year==2001

replace regioncode="542623" if regioncode=="542623" & year==2001

replace regioncode="542625" if regioncode=="542625" & year==2001

replace regioncode="542626" if regioncode=="542626" & year==2001

replace regioncode="610102" if regioncode=="610102" & year==2001

replace regioncode="610103" if regioncode=="610103" & year==2001

replace regioncode="610104" if regioncode=="610104" & year==2001

replace regioncode="610111" if regioncode=="610111" & year==2001

replace regioncode="610112" if regioncode=="610112" & year==2001

replace regioncode="610113" if regioncode=="610113" & year==2001

replace regioncode="610114" if regioncode=="610114" & year==2001

replace regioncode="610115" if regioncode=="610115" & year==2001

replace regioncode="610116" if regioncode=="610121" & year==2001

replace regioncode="610122" if regioncode=="610122" & year==2001

replace regioncode="610124" if regioncode=="610124" & year==2001

replace regioncode="610125" if regioncode=="610125" & year==2001

replace regioncode="610126" if regioncode=="610126" & year==2001

replace regioncode="610202" if regioncode=="610202" & year==2001

replace regioncode="610203" if regioncode=="610203" & year==2001

replace regioncode="610204" if regioncode=="610221" & year==2001

replace regioncode="610222" if regioncode=="610222" & year==2001

replace regioncode="610302" if regioncode=="610302" & year==2001

replace regioncode="610303" if regioncode=="610303" & year==2001

replace regioncode="610304" if regioncode=="610321" & year==2001

replace regioncode="610322" if regioncode=="610322" & year==2001

replace regioncode="610323" if regioncode=="610323" & year==2001

replace regioncode="610324" if regioncode=="610324" & year==2001

replace regioncode="610326" if regioncode=="610326" & year==2001

replace regioncode="610327" if regioncode=="610327" & year==2001

replace regioncode="610328" if regioncode=="610328" & year==2001

replace regioncode="610329" if regioncode=="610329" & year==2001

replace regioncode="610330" if regioncode=="610330" & year==2001

replace regioncode="610331" if regioncode=="610331" & year==2001

replace regioncode="610402" if regioncode=="610402" & year==2001

replace regioncode="610403" if regioncode=="610403" & year==2001

replace regioncode="610404" if regioncode=="610404" & year==2001

replace regioncode="610422" if regioncode=="610422" & year==2001

replace regioncode="610423" if regioncode=="610423" & year==2001

replace regioncode="610424" if regioncode=="610424" & year==2001

replace regioncode="610425" if regioncode=="610425" & year==2001

replace regioncode="610426" if regioncode=="610426" & year==2001

replace regioncode="610427" if regioncode=="610427" & year==2001

replace regioncode="610428" if regioncode=="610428" & year==2001

replace regioncode="610429" if regioncode=="610429" & year==2001

replace regioncode="610430" if regioncode=="610430" & year==2001

replace regioncode="610431" if regioncode=="610431" & year==2001

replace regioncode="610481" if regioncode=="610481" & year==2001

replace regioncode="610502" if regioncode=="610502" & year==2001

replace regioncode="610521" if regioncode=="610521" & year==2001

replace regioncode="610522" if regioncode=="610522" & year==2001

replace regioncode="610523" if regioncode=="610523" & year==2001

replace regioncode="610524" if regioncode=="610524" & year==2001

replace regioncode="610525" if regioncode=="610525" & year==2001

replace regioncode="610526" if regioncode=="610526" & year==2001

replace regioncode="610527" if regioncode=="610527" & year==2001

replace regioncode="610528" if regioncode=="610528" & year==2001

replace regioncode="610581" if regioncode=="610581" & year==2001

replace regioncode="610582" if regioncode=="610582" & year==2001

replace regioncode="610602" if regioncode=="610602" & year==2001

replace regioncode="610621" if regioncode=="610621" & year==2001

replace regioncode="610622" if regioncode=="610622" & year==2001

replace regioncode="610623" if regioncode=="610623" & year==2001

replace regioncode="610624" if regioncode=="610624" & year==2001

replace regioncode="610625" if regioncode=="610625" & year==2001

replace regioncode="610626" if regioncode=="610626" & year==2001

replace regioncode="610627" if regioncode=="610627" & year==2001

replace regioncode="610628" if regioncode=="610628" & year==2001

replace regioncode="610629" if regioncode=="610629" & year==2001

replace regioncode="610630" if regioncode=="610630" & year==2001

replace regioncode="610631" if regioncode=="610631" & year==2001

replace regioncode="610632" if regioncode=="610632" & year==2001

replace regioncode="610702" if regioncode=="610702" & year==2001

replace regioncode="610721" if regioncode=="610721" & year==2001

replace regioncode="610722" if regioncode=="610722" & year==2001

replace regioncode="610723" if regioncode=="610723" & year==2001

replace regioncode="610724" if regioncode=="610724" & year==2001

replace regioncode="610725" if regioncode=="610725" & year==2001

replace regioncode="610726" if regioncode=="610726" & year==2001

replace regioncode="610727" if regioncode=="610727" & year==2001

replace regioncode="610728" if regioncode=="610728" & year==2001

replace regioncode="610729" if regioncode=="610729" & year==2001

replace regioncode="610730" if regioncode=="610730" & year==2001

replace regioncode="610802" if regioncode=="610802" & year==2001

replace regioncode="610821" if regioncode=="610821" & year==2001

replace regioncode="610822" if regioncode=="610822" & year==2001

replace regioncode="610823" if regioncode=="610823" & year==2001

replace regioncode="610824" if regioncode=="610824" & year==2001

replace regioncode="610825" if regioncode=="610825" & year==2001

replace regioncode="610826" if regioncode=="610826" & year==2001

replace regioncode="610827" if regioncode=="610827" & year==2001

replace regioncode="610828" if regioncode=="610828" & year==2001

replace regioncode="610829" if regioncode=="610829" & year==2001

replace regioncode="610830" if regioncode=="610830" & year==2001

replace regioncode="610831" if regioncode=="610831" & year==2001

replace regioncode="610902" if regioncode=="610902" & year==2001

replace regioncode="610921" if regioncode=="610921" & year==2001

replace regioncode="610922" if regioncode=="610922" & year==2001

replace regioncode="610923" if regioncode=="610923" & year==2001

replace regioncode="610924" if regioncode=="610924" & year==2001

replace regioncode="610925" if regioncode=="610925" & year==2001

replace regioncode="610926" if regioncode=="610926" & year==2001

replace regioncode="610927" if regioncode=="610927" & year==2001

replace regioncode="610928" if regioncode=="610928" & year==2001

replace regioncode="610929" if regioncode=="610929" & year==2001

replace regioncode="611002" if regioncode=="611002" & year==2001

replace regioncode="611021" if regioncode=="612522" & year==2001

replace regioncode="611022" if regioncode=="612523" & year==2001

replace regioncode="611023" if regioncode=="612524" & year==2001

replace regioncode="611024" if regioncode=="612525" & year==2001

replace regioncode="611025" if regioncode=="612526" & year==2001

replace regioncode="611026" if regioncode=="612527" & year==2001

replace regioncode="620102" if regioncode=="620102" & year==2001

replace regioncode="620103" if regioncode=="620103" & year==2001

replace regioncode="620104" if regioncode=="620104" & year==2001

replace regioncode="620105" if regioncode=="620105" & year==2001

replace regioncode="620111" if regioncode=="620111" & year==2001

replace regioncode="620121" if regioncode=="620121" & year==2001

replace regioncode="620122" if regioncode=="620122" & year==2001

replace regioncode="620123" if regioncode=="620123" & year==2001

replace regioncode="620201" if regioncode=="620201" & year==2001

replace regioncode="620302" if regioncode=="620302" & year==2001

replace regioncode="620321" if regioncode=="620321" & year==2001

replace regioncode="620402" if regioncode=="620402" & year==2001

replace regioncode="620403" if regioncode=="620403" & year==2001

replace regioncode="620421" if regioncode=="620421" & year==2001

replace regioncode="620422" if regioncode=="620422" & year==2001

replace regioncode="620423" if regioncode=="620423" & year==2001

replace regioncode="620502" if regioncode=="620502" & year==2001

replace regioncode="620503" if regioncode=="620503" & year==2001

replace regioncode="620521" if regioncode=="620521" & year==2001

replace regioncode="620522" if regioncode=="620522" & year==2001

replace regioncode="620523" if regioncode=="620523" & year==2001

replace regioncode="620524" if regioncode=="620524" & year==2001

replace regioncode="620525" if regioncode=="620525" & year==2001

replace regioncode="622301" if regioncode=="622301" & year==2001

replace regioncode="622322" if regioncode=="622322" & year==2001

replace regioncode="622323" if regioncode=="622323" & year==2001

replace regioncode="622326" if regioncode=="622326" & year==2001

replace regioncode="622201" if regioncode=="622201" & year==2001

replace regioncode="622222" if regioncode=="622222" & year==2001

replace regioncode="622223" if regioncode=="622223" & year==2001

replace regioncode="622224" if regioncode=="622224" & year==2001

replace regioncode="622225" if regioncode=="622225" & year==2001

replace regioncode="622226" if regioncode=="622226" & year==2001

replace regioncode="622701" if regioncode=="622701" & year==2001

replace regioncode="622722" if regioncode=="622722" & year==2001

replace regioncode="622723" if regioncode=="622723" & year==2001

replace regioncode="622724" if regioncode=="622724" & year==2001

replace regioncode="622725" if regioncode=="622725" & year==2001

replace regioncode="622726" if regioncode=="622726" & year==2001

replace regioncode="622727" if regioncode=="622727" & year==2001

replace regioncode="622102" if regioncode=="622102" & year==2001

replace regioncode="622123" if regioncode=="622123" & year==2001

replace regioncode="622126" if regioncode=="622126" & year==2001

replace regioncode="622124" if regioncode=="622124" & year==2001

replace regioncode="622125" if regioncode=="622125" & year==2001

replace regioncode="622101" if regioncode=="622101" & year==2001

replace regioncode="622103" if regioncode=="622103" & year==2001

replace regioncode="622801" if regioncode=="622801" & year==2001

replace regioncode="622821" if regioncode=="622821" & year==2001

replace regioncode="622822" if regioncode=="622822" & year==2001

replace regioncode="622823" if regioncode=="622823" & year==2001

replace regioncode="622824" if regioncode=="622824" & year==2001

replace regioncode="622825" if regioncode=="622825" & year==2001

replace regioncode="622826" if regioncode=="622826" & year==2001

replace regioncode="622827" if regioncode=="622827" & year==2001

replace regioncode="622421" if regioncode=="622421" & year==2001

replace regioncode="622424" if regioncode=="622424" & year==2001

replace regioncode="622425" if regioncode=="622425" & year==2001

replace regioncode="622426" if regioncode=="622426" & year==2001

replace regioncode="622427" if regioncode=="622427" & year==2001

replace regioncode="622428" if regioncode=="622428" & year==2001

replace regioncode="622429" if regioncode=="622429" & year==2001

replace regioncode="622621" if regioncode=="622621" & year==2001

replace regioncode="622624" if regioncode=="622624" & year==2001

replace regioncode="622626" if regioncode=="622626" & year==2001

replace regioncode="622623" if regioncode=="622623" & year==2001

replace regioncode="622625" if regioncode=="622625" & year==2001

replace regioncode="622627" if regioncode=="622627" & year==2001

replace regioncode="622628" if regioncode=="622628" & year==2001

replace regioncode="622630" if regioncode=="622630" & year==2001

replace regioncode="622629" if regioncode=="622629" & year==2001

replace regioncode="622901" if regioncode=="622901" & year==2001

replace regioncode="622921" if regioncode=="622921" & year==2001

replace regioncode="622922" if regioncode=="622922" & year==2001

replace regioncode="622923" if regioncode=="622923" & year==2001

replace regioncode="622924" if regioncode=="622924" & year==2001

replace regioncode="622925" if regioncode=="622925" & year==2001

replace regioncode="622926" if regioncode=="622926" & year==2001

replace regioncode="622927" if regioncode=="622927" & year==2001

replace regioncode="623001" if regioncode=="623001" & year==2001

replace regioncode="623021" if regioncode=="623021" & year==2001

replace regioncode="623022" if regioncode=="623022" & year==2001

replace regioncode="623023" if regioncode=="623023" & year==2001

replace regioncode="623024" if regioncode=="623024" & year==2001

replace regioncode="623025" if regioncode=="623025" & year==2001

replace regioncode="623026" if regioncode=="623026" & year==2001

replace regioncode="623027" if regioncode=="623027" & year==2001

replace regioncode="630102" if regioncode=="630102" & year==2001

replace regioncode="630103" if regioncode=="630103" & year==2001

replace regioncode="630104" if regioncode=="630104" & year==2001

replace regioncode="630105" if regioncode=="630105" & year==2001

replace regioncode="630121" if regioncode=="630121" & year==2001

replace regioncode="630122" if regioncode=="630122" & year==2001

replace regioncode="630123" if regioncode=="630123" & year==2001

replace regioncode="632121" if regioncode=="632121" & year==2001

replace regioncode="632122" if regioncode=="632122" & year==2001

replace regioncode="632123" if regioncode=="632123" & year==2001

replace regioncode="632126" if regioncode=="632126" & year==2001

replace regioncode="632127" if regioncode=="632127" & year==2001

replace regioncode="632128" if regioncode=="632128" & year==2001

replace regioncode="632221" if regioncode=="632221" & year==2001

replace regioncode="632222" if regioncode=="632222" & year==2001

replace regioncode="632223" if regioncode=="632223" & year==2001

replace regioncode="632224" if regioncode=="632224" & year==2001

replace regioncode="632321" if regioncode=="632321" & year==2001

replace regioncode="632322" if regioncode=="632322" & year==2001

replace regioncode="632521" if regioncode=="632521" & year==2001

replace regioncode="632522" if regioncode=="632522" & year==2001

replace regioncode="632523" if regioncode=="632523" & year==2001

replace regioncode="632524" if regioncode=="632524" & year==2001

replace regioncode="632525" if regioncode=="632525" & year==2001

replace regioncode="632621" if regioncode=="632621" & year==2001

replace regioncode="632622" if regioncode=="632622" & year==2001

replace regioncode="632623" if regioncode=="632623" & year==2001

replace regioncode="632624" if regioncode=="632624" & year==2001

replace regioncode="632625" if regioncode=="632625" & year==2001

replace regioncode="632626" if regioncode=="632626" & year==2001

replace regioncode="632721" if regioncode=="632721" & year==2001

replace regioncode="632723" if regioncode=="632723" & year==2001

replace regioncode="632724" if regioncode=="632724" & year==2001

replace regioncode="632725" if regioncode=="632725" & year==2001

replace regioncode="632726" if regioncode=="632726" & year==2001

replace regioncode="632801" if regioncode=="632801" & year==2001

replace regioncode="632802" if regioncode=="632802" & year==2001

replace regioncode="632821" if regioncode=="632821" & year==2001

replace regioncode="632822" if regioncode=="632822" & year==2001

replace regioncode="640104" if regioncode=="640102" & year==2001

replace regioncode="640105" if regioncode=="640103" & year==2001

replace regioncode="640106" if regioncode=="640111" & year==2001

replace regioncode="640121" if regioncode=="640121" & year==2001

replace regioncode="640122" if regioncode=="640122" & year==2001

replace regioncode="640181" if regioncode=="640382" & year==2001

replace regioncode="640202" if regioncode=="640202" & year==2001

replace regioncode="640203" if regioncode=="640203" & year==2001

replace regioncode="640221" if regioncode=="640221" & year==2001

replace regioncode="640302" if regioncode=="640302" & year==2001

replace regioncode="640321" if regioncode=="640321" & year==2001

replace regioncode="640322" if regioncode=="640322" & year==2001

replace regioncode="640323" if regioncode=="640323" & year==2001

replace regioncode="640324" if regioncode=="640324" & year==2001

replace regioncode="640381" if regioncode=="640381" & year==2001

replace regioncode="640402" if regioncode=="642221" & year==2001

replace regioncode="640421" if regioncode=="642222" & year==2001

replace regioncode="640422" if regioncode=="642223" & year==2001

replace regioncode="640423" if regioncode=="642224" & year==2001

replace regioncode="640424" if regioncode=="642225" & year==2001

replace regioncode="640425" if regioncode=="642226" & year==2001

replace regioncode="650102" if regioncode=="650102" & year==2001

replace regioncode="650103" if regioncode=="650103" & year==2001

replace regioncode="650104" if regioncode=="650104" & year==2001

replace regioncode="650105" if regioncode=="650105" & year==2001

replace regioncode="650106" if regioncode=="650106" & year==2001

replace regioncode="650107" if regioncode=="650107" & year==2001

replace regioncode="650108" if regioncode=="650108" & year==2001

replace regioncode="650121" if regioncode=="650121" & year==2001

replace regioncode="650202" if regioncode=="650202" & year==2001

replace regioncode="650203" if regioncode=="650203" & year==2001

replace regioncode="650204" if regioncode=="650204" & year==2001

replace regioncode="650205" if regioncode=="650205" & year==2001

replace regioncode="652101" if regioncode=="652101" & year==2001

replace regioncode="652122" if regioncode=="652122" & year==2001

replace regioncode="652123" if regioncode=="652123" & year==2001

replace regioncode="652201" if regioncode=="652201" & year==2001

replace regioncode="652222" if regioncode=="652222" & year==2001

replace regioncode="652223" if regioncode=="652223" & year==2001

replace regioncode="652301" if regioncode=="652301" & year==2001

replace regioncode="652302" if regioncode=="652302" & year==2001

replace regioncode="652303" if regioncode=="652303" & year==2001

replace regioncode="652323" if regioncode=="652323" & year==2001

replace regioncode="652324" if regioncode=="652324" & year==2001

replace regioncode="652325" if regioncode=="652325" & year==2001

replace regioncode="652327" if regioncode=="652327" & year==2001

replace regioncode="652328" if regioncode=="652328" & year==2001

replace regioncode="652701" if regioncode=="652701" & year==2001

replace regioncode="652722" if regioncode=="652722" & year==2001

replace regioncode="652723" if regioncode=="652723" & year==2001

replace regioncode="652801" if regioncode=="652801" & year==2001

replace regioncode="652822" if regioncode=="652822" & year==2001

replace regioncode="652823" if regioncode=="652823" & year==2001

replace regioncode="652824" if regioncode=="652824" & year==2001

replace regioncode="652825" if regioncode=="652825" & year==2001

replace regioncode="652826" if regioncode=="652826" & year==2001

replace regioncode="652827" if regioncode=="652827" & year==2001

replace regioncode="652828" if regioncode=="652828" & year==2001

replace regioncode="652829" if regioncode=="652829" & year==2001

replace regioncode="652901" if regioncode=="652901" & year==2001

replace regioncode="652922" if regioncode=="652922" & year==2001

replace regioncode="652923" if regioncode=="652923" & year==2001

replace regioncode="652924" if regioncode=="652924" & year==2001

replace regioncode="652925" if regioncode=="652925" & year==2001

replace regioncode="652926" if regioncode=="652926" & year==2001

replace regioncode="652928" if regioncode=="652928" & year==2001

replace regioncode="652929" if regioncode=="652929" & year==2001

replace regioncode="653001" if regioncode=="653001" & year==2001

replace regioncode="653022" if regioncode=="653022" & year==2001

replace regioncode="653023" if regioncode=="653023" & year==2001

replace regioncode="653024" if regioncode=="653024" & year==2001

replace regioncode="653101" if regioncode=="653101" & year==2001

replace regioncode="653121" if regioncode=="653121" & year==2001

replace regioncode="653122" if regioncode=="653122" & year==2001

replace regioncode="653123" if regioncode=="653123" & year==2001

replace regioncode="653124" if regioncode=="653124" & year==2001

replace regioncode="653125" if regioncode=="653125" & year==2001

replace regioncode="653126" if regioncode=="653126" & year==2001

replace regioncode="653127" if regioncode=="653127" & year==2001

replace regioncode="653128" if regioncode=="653128" & year==2001

replace regioncode="653129" if regioncode=="653129" & year==2001

replace regioncode="653130" if regioncode=="653130" & year==2001

replace regioncode="653131" if regioncode=="653131" & year==2001

replace regioncode="653201" if regioncode=="653201" & year==2001

replace regioncode="653221" if regioncode=="653221" & year==2001

replace regioncode="653222" if regioncode=="653222" & year==2001

replace regioncode="653223" if regioncode=="653223" & year==2001

replace regioncode="653224" if regioncode=="653224" & year==2001

replace regioncode="653225" if regioncode=="653225" & year==2001

replace regioncode="653226" if regioncode=="653226" & year==2001

replace regioncode="653227" if regioncode=="653227" & year==2001

replace regioncode="654002" if regioncode=="654002" & year==2001

replace regioncode="654003" if regioncode=="654003" & year==2001

replace regioncode="654021" if regioncode=="654021" & year==2001

replace regioncode="654022" if regioncode=="654022" & year==2001

replace regioncode="654023" if regioncode=="654023" & year==2001

replace regioncode="654024" if regioncode=="654024" & year==2001

replace regioncode="654025" if regioncode=="654025" & year==2001

replace regioncode="654026" if regioncode=="654026" & year==2001

replace regioncode="654027" if regioncode=="654027" & year==2001

replace regioncode="654028" if regioncode=="654028" & year==2001

replace regioncode="654201" if regioncode=="654201" & year==2001

replace regioncode="654202" if regioncode=="654202" & year==2001

replace regioncode="654221" if regioncode=="654221" & year==2001

replace regioncode="654223" if regioncode=="654223" & year==2001

replace regioncode="654224" if regioncode=="654224" & year==2001

replace regioncode="654225" if regioncode=="654225" & year==2001

replace regioncode="654226" if regioncode=="654226" & year==2001

replace regioncode="654301" if regioncode=="654301" & year==2001

replace regioncode="654321" if regioncode=="654321" & year==2001

replace regioncode="654322" if regioncode=="654322" & year==2001

replace regioncode="654323" if regioncode=="654323" & year==2001

replace regioncode="654324" if regioncode=="654324" & year==2001

replace regioncode="654325" if regioncode=="654325" & year==2001

replace regioncode="654326" if regioncode=="654326" & year==2001

replace regioncode="659001" if regioncode=="659001" & year==2001

replace regioncode="110101" if regioncode=="110101" & year==2002

replace regioncode="110102" if regioncode=="110102" & year==2002

replace regioncode="110103" if regioncode=="110103" & year==2002

replace regioncode="110104" if regioncode=="110104" & year==2002

replace regioncode="110105" if regioncode=="110105" & year==2002

replace regioncode="110106" if regioncode=="110106" & year==2002

replace regioncode="110107" if regioncode=="110107" & year==2002

replace regioncode="110108" if regioncode=="110108" & year==2002

replace regioncode="110109" if regioncode=="110109" & year==2002

replace regioncode="110111" if regioncode=="110111" & year==2002

replace regioncode="110112" if regioncode=="110112" & year==2002

replace regioncode="110113" if regioncode=="110113" & year==2002

replace regioncode="110114" if regioncode=="110114" & year==2002

replace regioncode="110115" if regioncode=="110115" & year==2002

replace regioncode="110117" if regioncode=="110117" & year==2002

replace regioncode="110116" if regioncode=="110116" & year==2002

replace regioncode="110228" if regioncode=="110228" & year==2002

replace regioncode="110229" if regioncode=="110229" & year==2002

replace regioncode="120101" if regioncode=="120101" & year==2002

replace regioncode="120102" if regioncode=="120102" & year==2002

replace regioncode="120103" if regioncode=="120103" & year==2002

replace regioncode="120104" if regioncode=="120104" & year==2002

replace regioncode="120105" if regioncode=="120105" & year==2002

replace regioncode="120106" if regioncode=="120106" & year==2002

replace regioncode="120107" if regioncode=="120107" & year==2002

replace regioncode="120108" if regioncode=="120108" & year==2002

replace regioncode="120109" if regioncode=="120109" & year==2002

replace regioncode="120110" if regioncode=="120110" & year==2002

replace regioncode="120111" if regioncode=="120111" & year==2002

replace regioncode="120112" if regioncode=="120112" & year==2002

replace regioncode="120113" if regioncode=="120113" & year==2002

replace regioncode="120114" if regioncode=="120114" & year==2002

replace regioncode="120115" if regioncode=="120115" & year==2002

replace regioncode="120221" if regioncode=="120221" & year==2002

replace regioncode="120223" if regioncode=="120223" & year==2002

replace regioncode="120225" if regioncode=="120225" & year==2002

replace regioncode="130102" if regioncode=="130102" & year==2002

replace regioncode="130103" if regioncode=="130103" & year==2002

replace regioncode="130104" if regioncode=="130104" & year==2002

replace regioncode="130105" if regioncode=="130105" & year==2002

replace regioncode="130107" if regioncode=="130107" & year==2002

replace regioncode="130108" if regioncode=="130108" & year==2002

replace regioncode="130121" if regioncode=="130121" & year==2002

replace regioncode="130123" if regioncode=="130123" & year==2002

replace regioncode="130124" if regioncode=="130124" & year==2002

replace regioncode="130125" if regioncode=="130125" & year==2002

replace regioncode="130126" if regioncode=="130126" & year==2002

replace regioncode="130127" if regioncode=="130127" & year==2002

replace regioncode="130128" if regioncode=="130128" & year==2002

replace regioncode="130129" if regioncode=="130129" & year==2002

replace regioncode="130130" if regioncode=="130130" & year==2002

replace regioncode="130131" if regioncode=="130131" & year==2002

replace regioncode="130132" if regioncode=="130132" & year==2002

replace regioncode="130133" if regioncode=="130133" & year==2002

replace regioncode="130181" if regioncode=="130181" & year==2002

replace regioncode="130182" if regioncode=="130182" & year==2002

replace regioncode="130183" if regioncode=="130183" & year==2002

replace regioncode="130184" if regioncode=="130184" & year==2002

replace regioncode="130185" if regioncode=="130185" & year==2002

replace regioncode="130202" if regioncode=="130202" & year==2002

replace regioncode="130203" if regioncode=="130203" & year==2002

replace regioncode="130204" if regioncode=="130204" & year==2002

replace regioncode="130205" if regioncode=="130205" & year==2002

replace regioncode="130208" if regioncode=="130208" & year==2002

replace regioncode="130223" if regioncode=="130223" & year==2002

replace regioncode="130224" if regioncode=="130224" & year==2002

replace regioncode="130225" if regioncode=="130225" & year==2002

replace regioncode="130227" if regioncode=="130227" & year==2002

replace regioncode="130229" if regioncode=="130229" & year==2002

replace regioncode="130230" if regioncode=="130230" & year==2002

replace regioncode="130281" if regioncode=="130281" & year==2002

replace regioncode="130207" if regioncode=="130282" & year==2002

replace regioncode="130283" if regioncode=="130283" & year==2002

replace regioncode="130302" if regioncode=="130302" & year==2002

replace regioncode="130303" if regioncode=="130303" & year==2002

replace regioncode="130304" if regioncode=="130304" & year==2002

replace regioncode="130321" if regioncode=="130321" & year==2002

replace regioncode="130322" if regioncode=="130322" & year==2002

replace regioncode="130323" if regioncode=="130323" & year==2002

replace regioncode="130324" if regioncode=="130324" & year==2002

replace regioncode="130402" if regioncode=="130402" & year==2002

replace regioncode="130403" if regioncode=="130403" & year==2002

replace regioncode="130404" if regioncode=="130404" & year==2002

replace regioncode="130406" if regioncode=="130406" & year==2002

replace regioncode="130421" if regioncode=="130421" & year==2002

replace regioncode="130423" if regioncode=="130423" & year==2002

replace regioncode="130424" if regioncode=="130424" & year==2002

replace regioncode="130425" if regioncode=="130425" & year==2002

replace regioncode="130426" if regioncode=="130426" & year==2002

replace regioncode="130427" if regioncode=="130427" & year==2002

replace regioncode="130428" if regioncode=="130428" & year==2002

replace regioncode="130429" if regioncode=="130429" & year==2002

replace regioncode="130430" if regioncode=="130430" & year==2002

replace regioncode="130431" if regioncode=="130431" & year==2002

replace regioncode="130432" if regioncode=="130432" & year==2002

replace regioncode="130433" if regioncode=="130433" & year==2002

replace regioncode="130434" if regioncode=="130434" & year==2002

replace regioncode="130435" if regioncode=="130435" & year==2002

replace regioncode="130481" if regioncode=="130481" & year==2002

replace regioncode="130502" if regioncode=="130502" & year==2002

replace regioncode="130503" if regioncode=="130503" & year==2002

replace regioncode="130521" if regioncode=="130521" & year==2002

replace regioncode="130522" if regioncode=="130522" & year==2002

replace regioncode="130523" if regioncode=="130523" & year==2002

replace regioncode="130524" if regioncode=="130524" & year==2002

replace regioncode="130525" if regioncode=="130525" & year==2002

replace regioncode="130526" if regioncode=="130526" & year==2002

replace regioncode="130527" if regioncode=="130527" & year==2002

replace regioncode="130528" if regioncode=="130528" & year==2002

replace regioncode="130529" if regioncode=="130529" & year==2002

replace regioncode="130530" if regioncode=="130530" & year==2002

replace regioncode="130531" if regioncode=="130531" & year==2002

replace regioncode="130532" if regioncode=="130532" & year==2002

replace regioncode="130533" if regioncode=="130533" & year==2002

replace regioncode="130534" if regioncode=="130534" & year==2002

replace regioncode="130535" if regioncode=="130535" & year==2002

replace regioncode="130581" if regioncode=="130581" & year==2002

replace regioncode="130582" if regioncode=="130582" & year==2002

replace regioncode="130602" if regioncode=="130602" & year==2002

replace regioncode="130603" if regioncode=="130603" & year==2002

replace regioncode="130604" if regioncode=="130604" & year==2002

replace regioncode="130621" if regioncode=="130621" & year==2002

replace regioncode="130622" if regioncode=="130622" & year==2002

replace regioncode="130623" if regioncode=="130623" & year==2002

replace regioncode="130624" if regioncode=="130624" & year==2002

replace regioncode="130625" if regioncode=="130625" & year==2002

replace regioncode="130626" if regioncode=="130626" & year==2002

replace regioncode="130627" if regioncode=="130627" & year==2002

replace regioncode="130628" if regioncode=="130628" & year==2002

replace regioncode="130629" if regioncode=="130629" & year==2002

replace regioncode="130630" if regioncode=="130630" & year==2002

replace regioncode="130631" if regioncode=="130631" & year==2002

replace regioncode="130632" if regioncode=="130632" & year==2002

replace regioncode="130633" if regioncode=="130633" & year==2002

replace regioncode="130634" if regioncode=="130634" & year==2002

replace regioncode="130635" if regioncode=="130635" & year==2002

replace regioncode="130636" if regioncode=="130636" & year==2002

replace regioncode="130637" if regioncode=="130637" & year==2002

replace regioncode="130638" if regioncode=="130638" & year==2002

replace regioncode="130681" if regioncode=="130681" & year==2002

replace regioncode="130682" if regioncode=="130682" & year==2002

replace regioncode="130683" if regioncode=="130683" & year==2002

replace regioncode="130684" if regioncode=="130684" & year==2002

replace regioncode="130702" if regioncode=="130702" & year==2002

replace regioncode="130703" if regioncode=="130703" & year==2002

replace regioncode="130705" if regioncode=="130705" & year==2002

replace regioncode="130706" if regioncode=="130706" & year==2002

replace regioncode="130721" if regioncode=="130721" & year==2002

replace regioncode="130722" if regioncode=="130722" & year==2002

replace regioncode="130723" if regioncode=="130723" & year==2002

replace regioncode="130724" if regioncode=="130724" & year==2002

replace regioncode="130725" if regioncode=="130725" & year==2002

replace regioncode="130726" if regioncode=="130726" & year==2002

replace regioncode="130727" if regioncode=="130727" & year==2002

replace regioncode="130728" if regioncode=="130728" & year==2002

replace regioncode="130729" if regioncode=="130729" & year==2002

replace regioncode="130730" if regioncode=="130730" & year==2002

replace regioncode="130731" if regioncode=="130731" & year==2002

replace regioncode="130732" if regioncode=="130732" & year==2002

replace regioncode="130733" if regioncode=="130733" & year==2002

replace regioncode="130802" if regioncode=="130802" & year==2002

replace regioncode="130803" if regioncode=="130803" & year==2002

replace regioncode="130804" if regioncode=="130804" & year==2002

replace regioncode="130821" if regioncode=="130821" & year==2002

replace regioncode="130822" if regioncode=="130822" & year==2002

replace regioncode="130823" if regioncode=="130823" & year==2002

replace regioncode="130824" if regioncode=="130824" & year==2002

replace regioncode="130825" if regioncode=="130825" & year==2002

replace regioncode="130826" if regioncode=="130826" & year==2002

replace regioncode="130827" if regioncode=="130827" & year==2002

replace regioncode="130828" if regioncode=="130828" & year==2002

replace regioncode="130902" if regioncode=="130902" & year==2002

replace regioncode="130903" if regioncode=="130903" & year==2002

replace regioncode="130921" if regioncode=="130921" & year==2002

replace regioncode="130922" if regioncode=="130922" & year==2002

replace regioncode="130923" if regioncode=="130923" & year==2002

replace regioncode="130924" if regioncode=="130924" & year==2002

replace regioncode="130925" if regioncode=="130925" & year==2002

replace regioncode="130926" if regioncode=="130926" & year==2002

replace regioncode="130927" if regioncode=="130927" & year==2002

replace regioncode="130928" if regioncode=="130928" & year==2002

replace regioncode="130929" if regioncode=="130929" & year==2002

replace regioncode="130930" if regioncode=="130930" & year==2002

replace regioncode="130981" if regioncode=="130981" & year==2002

replace regioncode="130982" if regioncode=="130982" & year==2002

replace regioncode="130983" if regioncode=="130983" & year==2002

replace regioncode="130984" if regioncode=="130984" & year==2002

replace regioncode="131002" if regioncode=="131002" & year==2002

replace regioncode="131003" if regioncode=="131003" & year==2002

replace regioncode="131022" if regioncode=="131022" & year==2002

replace regioncode="131023" if regioncode=="131023" & year==2002

replace regioncode="131024" if regioncode=="131024" & year==2002

replace regioncode="131025" if regioncode=="131025" & year==2002

replace regioncode="131026" if regioncode=="131026" & year==2002

replace regioncode="131028" if regioncode=="131028" & year==2002

replace regioncode="131081" if regioncode=="131081" & year==2002

replace regioncode="131082" if regioncode=="131082" & year==2002

replace regioncode="131102" if regioncode=="131102" & year==2002

replace regioncode="131121" if regioncode=="131121" & year==2002

replace regioncode="131122" if regioncode=="131122" & year==2002

replace regioncode="131123" if regioncode=="131123" & year==2002

replace regioncode="131124" if regioncode=="131124" & year==2002

replace regioncode="131125" if regioncode=="131125" & year==2002

replace regioncode="131126" if regioncode=="131126" & year==2002

replace regioncode="131127" if regioncode=="131127" & year==2002

replace regioncode="131128" if regioncode=="131128" & year==2002

replace regioncode="131181" if regioncode=="131181" & year==2002

replace regioncode="131182" if regioncode=="131182" & year==2002

replace regioncode="140105" if regioncode=="140105" & year==2002

replace regioncode="140106" if regioncode=="140106" & year==2002

replace regioncode="140107" if regioncode=="140107" & year==2002

replace regioncode="140108" if regioncode=="140108" & year==2002

replace regioncode="140109" if regioncode=="140109" & year==2002

replace regioncode="140110" if regioncode=="140110" & year==2002

replace regioncode="140121" if regioncode=="140121" & year==2002

replace regioncode="140122" if regioncode=="140122" & year==2002

replace regioncode="140123" if regioncode=="140123" & year==2002

replace regioncode="140181" if regioncode=="140181" & year==2002

replace regioncode="140202" if regioncode=="140202" & year==2002

replace regioncode="140203" if regioncode=="140203" & year==2002

replace regioncode="140211" if regioncode=="140211" & year==2002

replace regioncode="140212" if regioncode=="140212" & year==2002

replace regioncode="140221" if regioncode=="140221" & year==2002

replace regioncode="140222" if regioncode=="140222" & year==2002

replace regioncode="140223" if regioncode=="140223" & year==2002

replace regioncode="140224" if regioncode=="140224" & year==2002

replace regioncode="140225" if regioncode=="140225" & year==2002

replace regioncode="140226" if regioncode=="140226" & year==2002

replace regioncode="140227" if regioncode=="140227" & year==2002

replace regioncode="140302" if regioncode=="140301" & year==2002

replace regioncode="140303" if regioncode=="140303" & year==2002

replace regioncode="140311" if regioncode=="140311" & year==2002

replace regioncode="140321" if regioncode=="140321" & year==2002

replace regioncode="140322" if regioncode=="140322" & year==2002

replace regioncode="140402" if regioncode=="140402" & year==2002

replace regioncode="140411" if regioncode=="140411" & year==2002

replace regioncode="140421" if regioncode=="140421" & year==2002

replace regioncode="140423" if regioncode=="140423" & year==2002

replace regioncode="140424" if regioncode=="140424" & year==2002

replace regioncode="140425" if regioncode=="140425" & year==2002

replace regioncode="140426" if regioncode=="140426" & year==2002

replace regioncode="140427" if regioncode=="140427" & year==2002

replace regioncode="140428" if regioncode=="140428" & year==2002

replace regioncode="140429" if regioncode=="140429" & year==2002

replace regioncode="140430" if regioncode=="140430" & year==2002

replace regioncode="140431" if regioncode=="140431" & year==2002

replace regioncode="140481" if regioncode=="140481" & year==2002

replace regioncode="140502" if regioncode=="140502" & year==2002

replace regioncode="140521" if regioncode=="140521" & year==2002

replace regioncode="140522" if regioncode=="140522" & year==2002

replace regioncode="140524" if regioncode=="140524" & year==2002

replace regioncode="140525" if regioncode=="140525" & year==2002

replace regioncode="140581" if regioncode=="140581" & year==2002

replace regioncode="140602" if regioncode=="140602" & year==2002

replace regioncode="140603" if regioncode=="140603" & year==2002

replace regioncode="140621" if regioncode=="140621" & year==2002

replace regioncode="140622" if regioncode=="140622" & year==2002

replace regioncode="140623" if regioncode=="140623" & year==2002

replace regioncode="140624" if regioncode=="140624" & year==2002

replace regioncode="140902" if regioncode=="140902" & year==2002

replace regioncode="140981" if regioncode=="140981" & year==2002

replace regioncode="140921" if regioncode=="140921" & year==2002

replace regioncode="140922" if regioncode=="140922" & year==2002

replace regioncode="140923" if regioncode=="140923" & year==2002

replace regioncode="140924" if regioncode=="140924" & year==2002

replace regioncode="140925" if regioncode=="140925" & year==2002

replace regioncode="140926" if regioncode=="140926" & year==2002

replace regioncode="140927" if regioncode=="140927" & year==2002

replace regioncode="140928" if regioncode=="140928" & year==2002

replace regioncode="140929" if regioncode=="140929" & year==2002

replace regioncode="140930" if regioncode=="140930" & year==2002

replace regioncode="140931" if regioncode=="140931" & year==2002

replace regioncode="140932" if regioncode=="140932" & year==2002

replace regioncode="141181" if regioncode=="142301" & year==2002

replace regioncode="141102" if regioncode=="142302" & year==2002

replace regioncode="141182" if regioncode=="142303" & year==2002

replace regioncode="141121" if regioncode=="142322" & year==2002

replace regioncode="141122" if regioncode=="142323" & year==2002

replace regioncode="141123" if regioncode=="142325" & year==2002

replace regioncode="141124" if regioncode=="142326" & year==2002

replace regioncode="141125" if regioncode=="142327" & year==2002

replace regioncode="141126" if regioncode=="142328" & year==2002

replace regioncode="141127" if regioncode=="142329" & year==2002

replace regioncode="141128" if regioncode=="142330" & year==2002

replace regioncode="141129" if regioncode=="142332" & year==2002

replace regioncode="141130" if regioncode=="142333" & year==2002

replace regioncode="140702" if regioncode=="140702" & year==2002

replace regioncode="140781" if regioncode=="140781" & year==2002

replace regioncode="140721" if regioncode=="140721" & year==2002

replace regioncode="140722" if regioncode=="140722" & year==2002

replace regioncode="140723" if regioncode=="140723" & year==2002

replace regioncode="140724" if regioncode=="140724" & year==2002

replace regioncode="140725" if regioncode=="140725" & year==2002

replace regioncode="140726" if regioncode=="140726" & year==2002

replace regioncode="140727" if regioncode=="140727" & year==2002

replace regioncode="140728" if regioncode=="140728" & year==2002

replace regioncode="140729" if regioncode=="140729" & year==2002

replace regioncode="141002" if regioncode=="141002" & year==2002

replace regioncode="141081" if regioncode=="141081" & year==2002

replace regioncode="141082" if regioncode=="141082" & year==2002

replace regioncode="141021" if regioncode=="141021" & year==2002

replace regioncode="141022" if regioncode=="141022" & year==2002

replace regioncode="141023" if regioncode=="141023" & year==2002

replace regioncode="141024" if regioncode=="141024" & year==2002

replace regioncode="141025" if regioncode=="141025" & year==2002

replace regioncode="141026" if regioncode=="141026" & year==2002

replace regioncode="141027" if regioncode=="141027" & year==2002

replace regioncode="141028" if regioncode=="141028" & year==2002

replace regioncode="141029" if regioncode=="141029" & year==2002

replace regioncode="141033" if regioncode=="141033" & year==2002

replace regioncode="141030" if regioncode=="141030" & year==2002

replace regioncode="141032" if regioncode=="141032" & year==2002

replace regioncode="141031" if regioncode=="141031" & year==2002

replace regioncode="141034" if regioncode=="141034" & year==2002

replace regioncode="140802" if regioncode=="140802" & year==2002

replace regioncode="140881" if regioncode=="140881" & year==2002

replace regioncode="140882" if regioncode=="140882" & year==2002

replace regioncode="140830" if regioncode=="140830" & year==2002

replace regioncode="140821" if regioncode=="140821" & year==2002

replace regioncode="140822" if regioncode=="140822" & year==2002

replace regioncode="140825" if regioncode=="140825" & year==2002

replace regioncode="140824" if regioncode=="140824" & year==2002

replace regioncode="140823" if regioncode=="140823" & year==2002

replace regioncode="140828" if regioncode=="140828" & year==2002

replace regioncode="140826" if regioncode=="140826" & year==2002

replace regioncode="140829" if regioncode=="140829" & year==2002

replace regioncode="140827" if regioncode=="140827" & year==2002

replace regioncode="150102" if regioncode=="150102" & year==2002

replace regioncode="150103" if regioncode=="150103" & year==2002

replace regioncode="150104" if regioncode=="150104" & year==2002

replace regioncode="150105" if regioncode=="150105" & year==2002

replace regioncode="150121" if regioncode=="150121" & year==2002

replace regioncode="150122" if regioncode=="150122" & year==2002

replace regioncode="150123" if regioncode=="150123" & year==2002

replace regioncode="150124" if regioncode=="150124" & year==2002

replace regioncode="150125" if regioncode=="150125" & year==2002

replace regioncode="150202" if regioncode=="150202" & year==2002

replace regioncode="150203" if regioncode=="150203" & year==2002

replace regioncode="150204" if regioncode=="150204" & year==2002

replace regioncode="150205" if regioncode=="150205" & year==2002

replace regioncode="150206" if regioncode=="150206" & year==2002

replace regioncode="150207" if regioncode=="150207" & year==2002

replace regioncode="150221" if regioncode=="150221" & year==2002

replace regioncode="150222" if regioncode=="150222" & year==2002

replace regioncode="150223" if regioncode=="150223" & year==2002

replace regioncode="150302" if regioncode=="150302" & year==2002

replace regioncode="150303" if regioncode=="150303" & year==2002

replace regioncode="150304" if regioncode=="150304" & year==2002

replace regioncode="150402" if regioncode=="150402" & year==2002

replace regioncode="150403" if regioncode=="150403" & year==2002

replace regioncode="150404" if regioncode=="150404" & year==2002

replace regioncode="150421" if regioncode=="150421" & year==2002

replace regioncode="150422" if regioncode=="150422" & year==2002

replace regioncode="150423" if regioncode=="150423" & year==2002

replace regioncode="150424" if regioncode=="150424" & year==2002

replace regioncode="150425" if regioncode=="150425" & year==2002

replace regioncode="150426" if regioncode=="150426" & year==2002

replace regioncode="150428" if regioncode=="150428" & year==2002

replace regioncode="150429" if regioncode=="150429" & year==2002

replace regioncode="150430" if regioncode=="150430" & year==2002

replace regioncode="150702" if regioncode=="150702" & year==2002

replace regioncode="150781" if regioncode=="150781" & year==2002

replace regioncode="150783" if regioncode=="150783" & year==2002

replace regioncode="150782" if regioncode=="150782" & year==2002

replace regioncode="150785" if regioncode=="150785" & year==2002

replace regioncode="150784" if regioncode=="150784" & year==2002

replace regioncode="150721" if regioncode=="150721" & year==2002

replace regioncode="150722" if regioncode=="150722" & year==2002

replace regioncode="150723" if regioncode=="150723" & year==2002

replace regioncode="150724" if regioncode=="150724" & year==2002

replace regioncode="150727" if regioncode=="150727" & year==2002

replace regioncode="150726" if regioncode=="150726" & year==2002

replace regioncode="150725" if regioncode=="150725" & year==2002

replace regioncode="152201" if regioncode=="152201" & year==2002

replace regioncode="152202" if regioncode=="152202" & year==2002

replace regioncode="152221" if regioncode=="152221" & year==2002

replace regioncode="152222" if regioncode=="152222" & year==2002

replace regioncode="152223" if regioncode=="152223" & year==2002

replace regioncode="152224" if regioncode=="152224" & year==2002

replace regioncode="150502" if regioncode=="150502" & year==2002

replace regioncode="150581" if regioncode=="150581" & year==2002

replace regioncode="150521" if regioncode=="150521" & year==2002

replace regioncode="150522" if regioncode=="150522" & year==2002

replace regioncode="150523" if regioncode=="150523" & year==2002

replace regioncode="150524" if regioncode=="150524" & year==2002

replace regioncode="150525" if regioncode=="150525" & year==2002

replace regioncode="150526" if regioncode=="150526" & year==2002

replace regioncode="152501" if regioncode=="152501" & year==2002

replace regioncode="152502" if regioncode=="152502" & year==2002

replace regioncode="152522" if regioncode=="152522" & year==2002

replace regioncode="152523" if regioncode=="152523" & year==2002

replace regioncode="152524" if regioncode=="152524" & year==2002

replace regioncode="152525" if regioncode=="152525" & year==2002

replace regioncode="152526" if regioncode=="152526" & year==2002

replace regioncode="152527" if regioncode=="152527" & year==2002

replace regioncode="152528" if regioncode=="152528" & year==2002

replace regioncode="152529" if regioncode=="152529" & year==2002

replace regioncode="152530" if regioncode=="152530" & year==2002

replace regioncode="152531" if regioncode=="152531" & year==2002

replace regioncode="152601" if regioncode=="152601" & year==2002

replace regioncode="152602" if regioncode=="152602" & year==2002

replace regioncode="152624" if regioncode=="152624" & year==2002

replace regioncode="152625" if regioncode=="152625" & year==2002

replace regioncode="152626" if regioncode=="152626" & year==2002

replace regioncode="152627" if regioncode=="152627" & year==2002

replace regioncode="152629" if regioncode=="152629" & year==2002

replace regioncode="152630" if regioncode=="152630" & year==2002

replace regioncode="152631" if regioncode=="152631" & year==2002

replace regioncode="152632" if regioncode=="152632" & year==2002

replace regioncode="152634" if regioncode=="152634" & year==2002

replace regioncode="150602" if regioncode=="150602" & year==2002

replace regioncode="150621" if regioncode=="150621" & year==2002

replace regioncode="150622" if regioncode=="150622" & year==2002

replace regioncode="150623" if regioncode=="150623" & year==2002

replace regioncode="150624" if regioncode=="150624" & year==2002

replace regioncode="150625" if regioncode=="150625" & year==2002

replace regioncode="150626" if regioncode=="150626" & year==2002

replace regioncode="150627" if regioncode=="150627" & year==2002

replace regioncode="152801" if regioncode=="152801" & year==2002

replace regioncode="152822" if regioncode=="152822" & year==2002

replace regioncode="152823" if regioncode=="152823" & year==2002

replace regioncode="152824" if regioncode=="152824" & year==2002

replace regioncode="152825" if regioncode=="152825" & year==2002

replace regioncode="152826" if regioncode=="152826" & year==2002

replace regioncode="152827" if regioncode=="152827" & year==2002

replace regioncode="152921" if regioncode=="152921" & year==2002

replace regioncode="152922" if regioncode=="152922" & year==2002

replace regioncode="152923" if regioncode=="152923" & year==2002

replace regioncode="210102" if regioncode=="210102" & year==2002

replace regioncode="210103" if regioncode=="210103" & year==2002

replace regioncode="210104" if regioncode=="210104" & year==2002

replace regioncode="210105" if regioncode=="210105" & year==2002

replace regioncode="210106" if regioncode=="210106" & year==2002

replace regioncode="210111" if regioncode=="210111" & year==2002

replace regioncode="210112" if regioncode=="210112" & year==2002

replace regioncode="210113" if regioncode=="210113" & year==2002

replace regioncode="210114" if regioncode=="210114" & year==2002

replace regioncode="210122" if regioncode=="210122" & year==2002

replace regioncode="210123" if regioncode=="210123" & year==2002

replace regioncode="210124" if regioncode=="210124" & year==2002

replace regioncode="210181" if regioncode=="210181" & year==2002

replace regioncode="210202" if regioncode=="210202" & year==2002

replace regioncode="210203" if regioncode=="210203" & year==2002

replace regioncode="210204" if regioncode=="210204" & year==2002

replace regioncode="210211" if regioncode=="210211" & year==2002

replace regioncode="210212" if regioncode=="210212" & year==2002

replace regioncode="210213" if regioncode=="210213" & year==2002

replace regioncode="210224" if regioncode=="210224" & year==2002

replace regioncode="210281" if regioncode=="210281" & year==2002

replace regioncode="210282" if regioncode=="210282" & year==2002

replace regioncode="210283" if regioncode=="210283" & year==2002

replace regioncode="210302" if regioncode=="210302" & year==2002

replace regioncode="210303" if regioncode=="210303" & year==2002

replace regioncode="210304" if regioncode=="210304" & year==2002

replace regioncode="210311" if regioncode=="210311" & year==2002

replace regioncode="210321" if regioncode=="210321" & year==2002

replace regioncode="210323" if regioncode=="210323" & year==2002

replace regioncode="210381" if regioncode=="210381" & year==2002

replace regioncode="210402" if regioncode=="210402" & year==2002

replace regioncode="210403" if regioncode=="210403" & year==2002

replace regioncode="210404" if regioncode=="210404" & year==2002

replace regioncode="210411" if regioncode=="210411" & year==2002

replace regioncode="210421" if regioncode=="210421" & year==2002

replace regioncode="210422" if regioncode=="210422" & year==2002

replace regioncode="210423" if regioncode=="210423" & year==2002

replace regioncode="210502" if regioncode=="210502" & year==2002

replace regioncode="210503" if regioncode=="210503" & year==2002

replace regioncode="210504" if regioncode=="210504" & year==2002

replace regioncode="210505" if regioncode=="210505" & year==2002

replace regioncode="210521" if regioncode=="210521" & year==2002

replace regioncode="210522" if regioncode=="210522" & year==2002

replace regioncode="210602" if regioncode=="210602" & year==2002

replace regioncode="210603" if regioncode=="210603" & year==2002

replace regioncode="210604" if regioncode=="210604" & year==2002

replace regioncode="210624" if regioncode=="210624" & year==2002

replace regioncode="210681" if regioncode=="210681" & year==2002

replace regioncode="210682" if regioncode=="210682" & year==2002

replace regioncode="210702" if regioncode=="210702" & year==2002

replace regioncode="210703" if regioncode=="210703" & year==2002

replace regioncode="210711" if regioncode=="210711" & year==2002

replace regioncode="210726" if regioncode=="210726" & year==2002

replace regioncode="210727" if regioncode=="210727" & year==2002

replace regioncode="210781" if regioncode=="210781" & year==2002

replace regioncode="210782" if regioncode=="210782" & year==2002

replace regioncode="210802" if regioncode=="210802" & year==2002

replace regioncode="210803" if regioncode=="210803" & year==2002

replace regioncode="210804" if regioncode=="210804" & year==2002

replace regioncode="210811" if regioncode=="210811" & year==2002

replace regioncode="210881" if regioncode=="210881" & year==2002

replace regioncode="210882" if regioncode=="210882" & year==2002

replace regioncode="210902" if regioncode=="210902" & year==2002

replace regioncode="210903" if regioncode=="210903" & year==2002

replace regioncode="210904" if regioncode=="210904" & year==2002

replace regioncode="210905" if regioncode=="210905" & year==2002

replace regioncode="210911" if regioncode=="210911" & year==2002

replace regioncode="210921" if regioncode=="210921" & year==2002

replace regioncode="210922" if regioncode=="210922" & year==2002

replace regioncode="211002" if regioncode=="211002" & year==2002

replace regioncode="211003" if regioncode=="211003" & year==2002

replace regioncode="211004" if regioncode=="211004" & year==2002

replace regioncode="211005" if regioncode=="211005" & year==2002

replace regioncode="211011" if regioncode=="211011" & year==2002

replace regioncode="211021" if regioncode=="211021" & year==2002

replace regioncode="211081" if regioncode=="211081" & year==2002

replace regioncode="211102" if regioncode=="211102" & year==2002

replace regioncode="211103" if regioncode=="211103" & year==2002

replace regioncode="211121" if regioncode=="211121" & year==2002

replace regioncode="211122" if regioncode=="211122" & year==2002

replace regioncode="211202" if regioncode=="211202" & year==2002

replace regioncode="211204" if regioncode=="211204" & year==2002

replace regioncode="211221" if regioncode=="211221" & year==2002

replace regioncode="211223" if regioncode=="211223" & year==2002

replace regioncode="211224" if regioncode=="211224" & year==2002

replace regioncode="211281" if regioncode=="211281" & year==2002

replace regioncode="211282" if regioncode=="211282" & year==2002

replace regioncode="211302" if regioncode=="211302" & year==2002

replace regioncode="211303" if regioncode=="211303" & year==2002

replace regioncode="211321" if regioncode=="211321" & year==2002

replace regioncode="211322" if regioncode=="211322" & year==2002

replace regioncode="211324" if regioncode=="211324" & year==2002

replace regioncode="211381" if regioncode=="211381" & year==2002

replace regioncode="211382" if regioncode=="211382" & year==2002

replace regioncode="211402" if regioncode=="211402" & year==2002

replace regioncode="211403" if regioncode=="211403" & year==2002

replace regioncode="211404" if regioncode=="211404" & year==2002

replace regioncode="211421" if regioncode=="211421" & year==2002

replace regioncode="211422" if regioncode=="211422" & year==2002

replace regioncode="211481" if regioncode=="211481" & year==2002

replace regioncode="220102" if regioncode=="220102" & year==2002

replace regioncode="220103" if regioncode=="220103" & year==2002

replace regioncode="220104" if regioncode=="220104" & year==2002

replace regioncode="220105" if regioncode=="220105" & year==2002

replace regioncode="220106" if regioncode=="220106" & year==2002

replace regioncode="220112" if regioncode=="220112" & year==2002

replace regioncode="220122" if regioncode=="220122" & year==2002

replace regioncode="220181" if regioncode=="220181" & year==2002

replace regioncode="220182" if regioncode=="220182" & year==2002

replace regioncode="220183" if regioncode=="220183" & year==2002

replace regioncode="220202" if regioncode=="220202" & year==2002

replace regioncode="220203" if regioncode=="220203" & year==2002

replace regioncode="220204" if regioncode=="220204" & year==2002

replace regioncode="220211" if regioncode=="220211" & year==2002

replace regioncode="220221" if regioncode=="220221" & year==2002

replace regioncode="220281" if regioncode=="220281" & year==2002

replace regioncode="220282" if regioncode=="220282" & year==2002

replace regioncode="220283" if regioncode=="220283" & year==2002

replace regioncode="220284" if regioncode=="220284" & year==2002

replace regioncode="220302" if regioncode=="220302" & year==2002

replace regioncode="220303" if regioncode=="220303" & year==2002

replace regioncode="220322" if regioncode=="220322" & year==2002

replace regioncode="220323" if regioncode=="220323" & year==2002

replace regioncode="220381" if regioncode=="220381" & year==2002

replace regioncode="220382" if regioncode=="220382" & year==2002

replace regioncode="220402" if regioncode=="220402" & year==2002

replace regioncode="220403" if regioncode=="220403" & year==2002

replace regioncode="220421" if regioncode=="220421" & year==2002

replace regioncode="220422" if regioncode=="220422" & year==2002

replace regioncode="220502" if regioncode=="220502" & year==2002

replace regioncode="220503" if regioncode=="220503" & year==2002

replace regioncode="220521" if regioncode=="220521" & year==2002

replace regioncode="220523" if regioncode=="220523" & year==2002

replace regioncode="220524" if regioncode=="220524" & year==2002

replace regioncode="220581" if regioncode=="220581" & year==2002

replace regioncode="220582" if regioncode=="220582" & year==2002

replace regioncode="220602" if regioncode=="220602" & year==2002

replace regioncode="220621" if regioncode=="220621" & year==2002

replace regioncode="220622" if regioncode=="220622" & year==2002

replace regioncode="220623" if regioncode=="220623" & year==2002

replace regioncode="220625" if regioncode=="220625" & year==2002

replace regioncode="220681" if regioncode=="220681" & year==2002

replace regioncode="220702" if regioncode=="220702" & year==2002

replace regioncode="220721" if regioncode=="220721" & year==2002

replace regioncode="220722" if regioncode=="220722" & year==2002

replace regioncode="220723" if regioncode=="220723" & year==2002

replace regioncode="220724" if regioncode=="220724" & year==2002

replace regioncode="220802" if regioncode=="220802" & year==2002

replace regioncode="220821" if regioncode=="220821" & year==2002

replace regioncode="220822" if regioncode=="220822" & year==2002

replace regioncode="220881" if regioncode=="220881" & year==2002

replace regioncode="220882" if regioncode=="220882" & year==2002

replace regioncode="222401" if regioncode=="222401" & year==2002

replace regioncode="222402" if regioncode=="222402" & year==2002

replace regioncode="222403" if regioncode=="222403" & year==2002

replace regioncode="222404" if regioncode=="222404" & year==2002

replace regioncode="222405" if regioncode=="222405" & year==2002

replace regioncode="222406" if regioncode=="222406" & year==2002

replace regioncode="222424" if regioncode=="222424" & year==2002

replace regioncode="222426" if regioncode=="222426" & year==2002

replace regioncode="230102" if regioncode=="230102" & year==2002

replace regioncode="230103" if regioncode=="230103" & year==2002

replace regioncode="230104" if regioncode=="230104" & year==2002

replace regioncode="230105" if regioncode=="230105" & year==2002

replace regioncode="230107" if regioncode=="230107" & year==2002

replace regioncode="230108" if regioncode=="230108" & year==2002

replace regioncode="230121" if regioncode=="230121" & year==2002

replace regioncode="230123" if regioncode=="230123" & year==2002

replace regioncode="230124" if regioncode=="230124" & year==2002

replace regioncode="230125" if regioncode=="230125" & year==2002

replace regioncode="230126" if regioncode=="230126" & year==2002

replace regioncode="230127" if regioncode=="230127" & year==2002

replace regioncode="230128" if regioncode=="230128" & year==2002

replace regioncode="230129" if regioncode=="230129" & year==2002

replace regioncode="230181" if regioncode=="230181" & year==2002

replace regioncode="230182" if regioncode=="230182" & year==2002

replace regioncode="230183" if regioncode=="230183" & year==2002

replace regioncode="230184" if regioncode=="230184" & year==2002

replace regioncode="230202" if regioncode=="230202" & year==2002

replace regioncode="230203" if regioncode=="230203" & year==2002

replace regioncode="230204" if regioncode=="230204" & year==2002

replace regioncode="230205" if regioncode=="230205" & year==2002

replace regioncode="230206" if regioncode=="230206" & year==2002

replace regioncode="230207" if regioncode=="230207" & year==2002

replace regioncode="230208" if regioncode=="230208" & year==2002

replace regioncode="230221" if regioncode=="230221" & year==2002

replace regioncode="230223" if regioncode=="230223" & year==2002

replace regioncode="230224" if regioncode=="230224" & year==2002

replace regioncode="230225" if regioncode=="230225" & year==2002

replace regioncode="230227" if regioncode=="230227" & year==2002

replace regioncode="230229" if regioncode=="230229" & year==2002

replace regioncode="230230" if regioncode=="230230" & year==2002

replace regioncode="230231" if regioncode=="230231" & year==2002

replace regioncode="230281" if regioncode=="230281" & year==2002

replace regioncode="230302" if regioncode=="230302" & year==2002

replace regioncode="230303" if regioncode=="230303" & year==2002

replace regioncode="230304" if regioncode=="230304" & year==2002

replace regioncode="230305" if regioncode=="230305" & year==2002

replace regioncode="230306" if regioncode=="230306" & year==2002

replace regioncode="230307" if regioncode=="230307" & year==2002

replace regioncode="230321" if regioncode=="230321" & year==2002

replace regioncode="230381" if regioncode=="230381" & year==2002

replace regioncode="230382" if regioncode=="230382" & year==2002

replace regioncode="230402" if regioncode=="230402" & year==2002

replace regioncode="230403" if regioncode=="230403" & year==2002

replace regioncode="230404" if regioncode=="230404" & year==2002

replace regioncode="230405" if regioncode=="230405" & year==2002

replace regioncode="230406" if regioncode=="230406" & year==2002

replace regioncode="230407" if regioncode=="230407" & year==2002

replace regioncode="230421" if regioncode=="230421" & year==2002

replace regioncode="230422" if regioncode=="230422" & year==2002

replace regioncode="230502" if regioncode=="230502" & year==2002

replace regioncode="230503" if regioncode=="230503" & year==2002

replace regioncode="230505" if regioncode=="230505" & year==2002

replace regioncode="230506" if regioncode=="230506" & year==2002

replace regioncode="230521" if regioncode=="230521" & year==2002

replace regioncode="230522" if regioncode=="230522" & year==2002

replace regioncode="230523" if regioncode=="230523" & year==2002

replace regioncode="230524" if regioncode=="230524" & year==2002

replace regioncode="230602" if regioncode=="230602" & year==2002

replace regioncode="230603" if regioncode=="230603" & year==2002

replace regioncode="230604" if regioncode=="230604" & year==2002

replace regioncode="230605" if regioncode=="230605" & year==2002

replace regioncode="230606" if regioncode=="230606" & year==2002

replace regioncode="230621" if regioncode=="230621" & year==2002

replace regioncode="230622" if regioncode=="230622" & year==2002

replace regioncode="230623" if regioncode=="230623" & year==2002

replace regioncode="230624" if regioncode=="230624" & year==2002

replace regioncode="230702" if regioncode=="230702" & year==2002

replace regioncode="230703" if regioncode=="230703" & year==2002

replace regioncode="230704" if regioncode=="230704" & year==2002

replace regioncode="230705" if regioncode=="230705" & year==2002

replace regioncode="230706" if regioncode=="230706" & year==2002

replace regioncode="230707" if regioncode=="230707" & year==2002

replace regioncode="230708" if regioncode=="230708" & year==2002

replace regioncode="230709" if regioncode=="230709" & year==2002

replace regioncode="230710" if regioncode=="230710" & year==2002

replace regioncode="230711" if regioncode=="230711" & year==2002

replace regioncode="230712" if regioncode=="230712" & year==2002

replace regioncode="230713" if regioncode=="230713" & year==2002

replace regioncode="230714" if regioncode=="230714" & year==2002

replace regioncode="230715" if regioncode=="230715" & year==2002

replace regioncode="230716" if regioncode=="230716" & year==2002

replace regioncode="230722" if regioncode=="230722" & year==2002

replace regioncode="230781" if regioncode=="230781" & year==2002

replace regioncode="230802" if regioncode=="230802" & year==2002

replace regioncode="230803" if regioncode=="230803" & year==2002

replace regioncode="230804" if regioncode=="230804" & year==2002

replace regioncode="230805" if regioncode=="230805" & year==2002

replace regioncode="230811" if regioncode=="230811" & year==2002

replace regioncode="230822" if regioncode=="230822" & year==2002

replace regioncode="230826" if regioncode=="230826" & year==2002

replace regioncode="230828" if regioncode=="230828" & year==2002

replace regioncode="230833" if regioncode=="230833" & year==2002

replace regioncode="230881" if regioncode=="230881" & year==2002

replace regioncode="230882" if regioncode=="230882" & year==2002

replace regioncode="230902" if regioncode=="230902" & year==2002

replace regioncode="230903" if regioncode=="230903" & year==2002

replace regioncode="230904" if regioncode=="230904" & year==2002

replace regioncode="230921" if regioncode=="230921" & year==2002

replace regioncode="231002" if regioncode=="231002" & year==2002

replace regioncode="231003" if regioncode=="231003" & year==2002

replace regioncode="231004" if regioncode=="231004" & year==2002

replace regioncode="231005" if regioncode=="231005" & year==2002

replace regioncode="231024" if regioncode=="231024" & year==2002

replace regioncode="231025" if regioncode=="231025" & year==2002

replace regioncode="231081" if regioncode=="231081" & year==2002

replace regioncode="231083" if regioncode=="231083" & year==2002

replace regioncode="231084" if regioncode=="231084" & year==2002

replace regioncode="231085" if regioncode=="231085" & year==2002

replace regioncode="231102" if regioncode=="231102" & year==2002

replace regioncode="231121" if regioncode=="231121" & year==2002

replace regioncode="231123" if regioncode=="231123" & year==2002

replace regioncode="231124" if regioncode=="231124" & year==2002

replace regioncode="231181" if regioncode=="231181" & year==2002

replace regioncode="231182" if regioncode=="231182" & year==2002

replace regioncode="231202" if regioncode=="231202" & year==2002

replace regioncode="231221" if regioncode=="231221" & year==2002

replace regioncode="231222" if regioncode=="231222" & year==2002

replace regioncode="231223" if regioncode=="231223" & year==2002

replace regioncode="231224" if regioncode=="231224" & year==2002

replace regioncode="231225" if regioncode=="231225" & year==2002

replace regioncode="231226" if regioncode=="231226" & year==2002

replace regioncode="231281" if regioncode=="231281" & year==2002

replace regioncode="231282" if regioncode=="231282" & year==2002

replace regioncode="231283" if regioncode=="231283" & year==2002

replace regioncode="232701" if regioncode=="232701" & year==2002

replace regioncode="232702" if regioncode=="232702" & year==2002

replace regioncode="232703" if regioncode=="232703" & year==2002

replace regioncode="232704" if regioncode=="232704" & year==2002

replace regioncode="232721" if regioncode=="232721" & year==2002

replace regioncode="232722" if regioncode=="232722" & year==2002

replace regioncode="232723" if regioncode=="232723" & year==2002

replace regioncode="310101" if regioncode=="310101" & year==2002

replace regioncode="310103" if regioncode=="310103" & year==2002

replace regioncode="310104" if regioncode=="310104" & year==2002

replace regioncode="310105" if regioncode=="310105" & year==2002

replace regioncode="310106" if regioncode=="310106" & year==2002

replace regioncode="310107" if regioncode=="310107" & year==2002

replace regioncode="310108" if regioncode=="310108" & year==2002

replace regioncode="310109" if regioncode=="310109" & year==2002

replace regioncode="310110" if regioncode=="310110" & year==2002

replace regioncode="310112" if regioncode=="310112" & year==2002

replace regioncode="310113" if regioncode=="310113" & year==2002

replace regioncode="310114" if regioncode=="310114" & year==2002

replace regioncode="310115" if regioncode=="310115" & year==2002

replace regioncode="310116" if regioncode=="310116" & year==2002

replace regioncode="310117" if regioncode=="310117" & year==2002

replace regioncode="310118" if regioncode=="310118" & year==2002

replace regioncode="310119" if regioncode=="310119" & year==2002

replace regioncode="310120" if regioncode=="310120" & year==2002

replace regioncode="310230" if regioncode=="310230" & year==2002

replace regioncode="320102" if regioncode=="320102" & year==2002

replace regioncode="320103" if regioncode=="320103" & year==2002

replace regioncode="320104" if regioncode=="320104" & year==2002

replace regioncode="320105" if regioncode=="320105" & year==2002

replace regioncode="320106" if regioncode=="320106" & year==2002

replace regioncode="320107" if regioncode=="320107" & year==2002

replace regioncode="320111" if regioncode=="320111" & year==2002

replace regioncode="320113" if regioncode=="320113" & year==2002

replace regioncode="320114" if regioncode=="320114" & year==2002

replace regioncode="320115" if regioncode=="320115" & year==2002

replace regioncode="320116" if regioncode=="320116" & year==2002

replace regioncode="320124" if regioncode=="320124" & year==2002

replace regioncode="320125" if regioncode=="320125" & year==2002

replace regioncode="320202" if regioncode=="320202" & year==2002

replace regioncode="320203" if regioncode=="320203" & year==2002

replace regioncode="320204" if regioncode=="320204" & year==2002

replace regioncode="320205" if regioncode=="320205" & year==2002

replace regioncode="320211" if regioncode=="320211" & year==2002

replace regioncode="320281" if regioncode=="320281" & year==2002

replace regioncode="320282" if regioncode=="320282" & year==2002

replace regioncode="320302" if regioncode=="320302" & year==2002

replace regioncode="320303" if regioncode=="320303" & year==2002

replace regioncode="320304" if regioncode=="320304" & year==2002

replace regioncode="320305" if regioncode=="320305" & year==2002

replace regioncode="320311" if regioncode=="320311" & year==2002

replace regioncode="320321" if regioncode=="320321" & year==2002

replace regioncode="320322" if regioncode=="320322" & year==2002

replace regioncode="320323" if regioncode=="320323" & year==2002

replace regioncode="320324" if regioncode=="320324" & year==2002

replace regioncode="320381" if regioncode=="320381" & year==2002

replace regioncode="320382" if regioncode=="320382" & year==2002

replace regioncode="320402" if regioncode=="320402" & year==2002

replace regioncode="320404" if regioncode=="320404" & year==2002

replace regioncode="320405" if regioncode=="320405" & year==2002

replace regioncode="320411" if regioncode=="320411" & year==2002

replace regioncode="320412" if regioncode=="320412" & year==2002

replace regioncode="320481" if regioncode=="320481" & year==2002

replace regioncode="320482" if regioncode=="320482" & year==2002

replace regioncode="320502" if regioncode=="320502" & year==2002

replace regioncode="320503" if regioncode=="320503" & year==2002

replace regioncode="320504" if regioncode=="320504" & year==2002

replace regioncode="320505" if regioncode=="320505" & year==2002

replace regioncode="320581" if regioncode=="320581" & year==2002

replace regioncode="320582" if regioncode=="320582" & year==2002

replace regioncode="320583" if regioncode=="320583" & year==2002

replace regioncode="320584" if regioncode=="320584" & year==2002

replace regioncode="320585" if regioncode=="320585" & year==2002

replace regioncode="320586" if regioncode=="320586" & year==2002

replace regioncode="320602" if regioncode=="320602" & year==2002

replace regioncode="320611" if regioncode=="320611" & year==2002

replace regioncode="320621" if regioncode=="320621" & year==2002

replace regioncode="320623" if regioncode=="320623" & year==2002

replace regioncode="320681" if regioncode=="320681" & year==2002

replace regioncode="320682" if regioncode=="320682" & year==2002

replace regioncode="320683" if regioncode=="320683" & year==2002

replace regioncode="320684" if regioncode=="320684" & year==2002

replace regioncode="320703" if regioncode=="320703" & year==2002

replace regioncode="320705" if regioncode=="320705" & year==2002

replace regioncode="320706" if regioncode=="320706" & year==2002

replace regioncode="320721" if regioncode=="320721" & year==2002

replace regioncode="320722" if regioncode=="320722" & year==2002

replace regioncode="320723" if regioncode=="320723" & year==2002

replace regioncode="320724" if regioncode=="320724" & year==2002

replace regioncode="320802" if regioncode=="320802" & year==2002

replace regioncode="320803" if regioncode=="320803" & year==2002

replace regioncode="320804" if regioncode=="320804" & year==2002

replace regioncode="320811" if regioncode=="320811" & year==2002

replace regioncode="320826" if regioncode=="320826" & year==2002

replace regioncode="320829" if regioncode=="320829" & year==2002

replace regioncode="320830" if regioncode=="320830" & year==2002

replace regioncode="320831" if regioncode=="320831" & year==2002

replace regioncode="320902" if regioncode=="320902" & year==2002

replace regioncode="320903" if regioncode=="320928" & year==2002

replace regioncode="320921" if regioncode=="320921" & year==2002

replace regioncode="320922" if regioncode=="320922" & year==2002

replace regioncode="320923" if regioncode=="320923" & year==2002

replace regioncode="320924" if regioncode=="320924" & year==2002

replace regioncode="320925" if regioncode=="320925" & year==2002

replace regioncode="320981" if regioncode=="320981" & year==2002

replace regioncode="320982" if regioncode=="320982" & year==2002

replace regioncode="321002" if regioncode=="321002" & year==2002

replace regioncode="321003" if regioncode=="321003" & year==2002

replace regioncode="321011" if regioncode=="321011" & year==2002

replace regioncode="321023" if regioncode=="321023" & year==2002

replace regioncode="321081" if regioncode=="321081" & year==2002

replace regioncode="321084" if regioncode=="321084" & year==2002

replace regioncode="321088" if regioncode=="321088" & year==2002

replace regioncode="321102" if regioncode=="321102" & year==2002

replace regioncode="321111" if regioncode=="321111" & year==2002

replace regioncode="321112" if regioncode=="321112" & year==2002

replace regioncode="321181" if regioncode=="321181" & year==2002

replace regioncode="321182" if regioncode=="321182" & year==2002

replace regioncode="321183" if regioncode=="321183" & year==2002

replace regioncode="321202" if regioncode=="321202" & year==2002

replace regioncode="321203" if regioncode=="321203" & year==2002

replace regioncode="321281" if regioncode=="321281" & year==2002

replace regioncode="321282" if regioncode=="321282" & year==2002

replace regioncode="321283" if regioncode=="321283" & year==2002

replace regioncode="321284" if regioncode=="321284" & year==2002

replace regioncode="321302" if regioncode=="321302" & year==2002

replace regioncode="321321" if regioncode=="321321" & year==2002

replace regioncode="321322" if regioncode=="321322" & year==2002

replace regioncode="321323" if regioncode=="321323" & year==2002

replace regioncode="321324" if regioncode=="321324" & year==2002

replace regioncode="330102" if regioncode=="330102" & year==2002

replace regioncode="330103" if regioncode=="330103" & year==2002

replace regioncode="330104" if regioncode=="330104" & year==2002

replace regioncode="330105" if regioncode=="330105" & year==2002

replace regioncode="330106" if regioncode=="330106" & year==2002

replace regioncode="330108" if regioncode=="330108" & year==2002

replace regioncode="330109" if regioncode=="330109" & year==2002

replace regioncode="330110" if regioncode=="330110" & year==2002

replace regioncode="330122" if regioncode=="330122" & year==2002

replace regioncode="330127" if regioncode=="330127" & year==2002

replace regioncode="330182" if regioncode=="330182" & year==2002

replace regioncode="330183" if regioncode=="330183" & year==2002

replace regioncode="330185" if regioncode=="330185" & year==2002

replace regioncode="330203" if regioncode=="330203" & year==2002

replace regioncode="330204" if regioncode=="330204" & year==2002

replace regioncode="330205" if regioncode=="330205" & year==2002

replace regioncode="330206" if regioncode=="330206" & year==2002

replace regioncode="330211" if regioncode=="330211" & year==2002

replace regioncode="330212" if regioncode=="330212" & year==2002

replace regioncode="330225" if regioncode=="330225" & year==2002

replace regioncode="330226" if regioncode=="330226" & year==2002

replace regioncode="330281" if regioncode=="330281" & year==2002

replace regioncode="330282" if regioncode=="330282" & year==2002

replace regioncode="330283" if regioncode=="330283" & year==2002

replace regioncode="330302" if regioncode=="330302" & year==2002

replace regioncode="330303" if regioncode=="330303" & year==2002

replace regioncode="330304" if regioncode=="330304" & year==2002

replace regioncode="330322" if regioncode=="330322" & year==2002

replace regioncode="330324" if regioncode=="330324" & year==2002

replace regioncode="330326" if regioncode=="330326" & year==2002

replace regioncode="330327" if regioncode=="330327" & year==2002

replace regioncode="330328" if regioncode=="330328" & year==2002

replace regioncode="330329" if regioncode=="330329" & year==2002

replace regioncode="330381" if regioncode=="330381" & year==2002

replace regioncode="330382" if regioncode=="330382" & year==2002

replace regioncode="330402" if regioncode=="330402" & year==2002

replace regioncode="330411" if regioncode=="330411" & year==2002

replace regioncode="330421" if regioncode=="330421" & year==2002

replace regioncode="330424" if regioncode=="330424" & year==2002

replace regioncode="330481" if regioncode=="330481" & year==2002

replace regioncode="330482" if regioncode=="330482" & year==2002

replace regioncode="330483" if regioncode=="330483" & year==2002

replace regioncode="330501" if regioncode=="330501" & year==2002

replace regioncode="330521" if regioncode=="330521" & year==2002

replace regioncode="330522" if regioncode=="330522" & year==2002

replace regioncode="330523" if regioncode=="330523" & year==2002

replace regioncode="330602" if regioncode=="330602" & year==2002

replace regioncode="330621" if regioncode=="330621" & year==2002

replace regioncode="330624" if regioncode=="330624" & year==2002

replace regioncode="330681" if regioncode=="330681" & year==2002

replace regioncode="330682" if regioncode=="330682" & year==2002

replace regioncode="330683" if regioncode=="330683" & year==2002

replace regioncode="330702" if regioncode=="330702" & year==2002

replace regioncode="330703" if regioncode=="330703" & year==2002

replace regioncode="330723" if regioncode=="330723" & year==2002

replace regioncode="330726" if regioncode=="330726" & year==2002

replace regioncode="330727" if regioncode=="330727" & year==2002

replace regioncode="330781" if regioncode=="330781" & year==2002

replace regioncode="330782" if regioncode=="330782" & year==2002

replace regioncode="330783" if regioncode=="330783" & year==2002

replace regioncode="330784" if regioncode=="330784" & year==2002

replace regioncode="330802" if regioncode=="330802" & year==2002

replace regioncode="330803" if regioncode=="330803" & year==2002

replace regioncode="330822" if regioncode=="330822" & year==2002

replace regioncode="330824" if regioncode=="330824" & year==2002

replace regioncode="330825" if regioncode=="330825" & year==2002

replace regioncode="330881" if regioncode=="330881" & year==2002

replace regioncode="330902" if regioncode=="330902" & year==2002

replace regioncode="330903" if regioncode=="330903" & year==2002

replace regioncode="330921" if regioncode=="330921" & year==2002

replace regioncode="330922" if regioncode=="330922" & year==2002

replace regioncode="331002" if regioncode=="331002" & year==2002

replace regioncode="331003" if regioncode=="331003" & year==2002

replace regioncode="331004" if regioncode=="331004" & year==2002

replace regioncode="331021" if regioncode=="331021" & year==2002

replace regioncode="331022" if regioncode=="331022" & year==2002

replace regioncode="331023" if regioncode=="331023" & year==2002

replace regioncode="331024" if regioncode=="331024" & year==2002

replace regioncode="331081" if regioncode=="331081" & year==2002

replace regioncode="331082" if regioncode=="331082" & year==2002

replace regioncode="331102" if regioncode=="331102" & year==2002

replace regioncode="331121" if regioncode=="331121" & year==2002

replace regioncode="331122" if regioncode=="331122" & year==2002

replace regioncode="331123" if regioncode=="331123" & year==2002

replace regioncode="331124" if regioncode=="331124" & year==2002

replace regioncode="331125" if regioncode=="331125" & year==2002

replace regioncode="331126" if regioncode=="331126" & year==2002

replace regioncode="331127" if regioncode=="331127" & year==2002

replace regioncode="331181" if regioncode=="331181" & year==2002

replace regioncode="340102" if regioncode=="340102" & year==2002

replace regioncode="340103" if regioncode=="340103" & year==2002

replace regioncode="340104" if regioncode=="340104" & year==2002

replace regioncode="340111" if regioncode=="340111" & year==2002

replace regioncode="340121" if regioncode=="340121" & year==2002

replace regioncode="340122" if regioncode=="340122" & year==2002

replace regioncode="340123" if regioncode=="340123" & year==2002

replace regioncode="340202" if regioncode=="340202" & year==2002

replace regioncode="340203" if regioncode=="340203" & year==2002

replace regioncode="340204" if regioncode=="340204" & year==2002

replace regioncode="340207" if regioncode=="340207" & year==2002

replace regioncode="340221" if regioncode=="340221" & year==2002

replace regioncode="340222" if regioncode=="340222" & year==2002

replace regioncode="340223" if regioncode=="340223" & year==2002

replace regioncode="340302" if regioncode=="340302" & year==2002

replace regioncode="340303" if regioncode=="340303" & year==2002

replace regioncode="340304" if regioncode=="340304" & year==2002

replace regioncode="340311" if regioncode=="340311" & year==2002

replace regioncode="340321" if regioncode=="340321" & year==2002

replace regioncode="340322" if regioncode=="340322" & year==2002

replace regioncode="340323" if regioncode=="340323" & year==2002

replace regioncode="340402" if regioncode=="340402" & year==2002

replace regioncode="340403" if regioncode=="340403" & year==2002

replace regioncode="340404" if regioncode=="340404" & year==2002

replace regioncode="340405" if regioncode=="340405" & year==2002

replace regioncode="340406" if regioncode=="340406" & year==2002

replace regioncode="340421" if regioncode=="340421" & year==2002

replace regioncode="340502" if regioncode=="340502" & year==2002

replace regioncode="340503" if regioncode=="340503" & year==2002

replace regioncode="340504" if regioncode=="340504" & year==2002

replace regioncode="340521" if regioncode=="340521" & year==2002

replace regioncode="340602" if regioncode=="340602" & year==2002

replace regioncode="340603" if regioncode=="340603" & year==2002

replace regioncode="340604" if regioncode=="340604" & year==2002

replace regioncode="340621" if regioncode=="340621" & year==2002

replace regioncode="340702" if regioncode=="340702" & year==2002

replace regioncode="340703" if regioncode=="340703" & year==2002

replace regioncode="340711" if regioncode=="340711" & year==2002

replace regioncode="340721" if regioncode=="340721" & year==2002

replace regioncode="340802" if regioncode=="340802" & year==2002

replace regioncode="340803" if regioncode=="340803" & year==2002

replace regioncode="340811" if regioncode=="340811" & year==2002

replace regioncode="340822" if regioncode=="340822" & year==2002

replace regioncode="340823" if regioncode=="340823" & year==2002

replace regioncode="340824" if regioncode=="340824" & year==2002

replace regioncode="340825" if regioncode=="340825" & year==2002

replace regioncode="340826" if regioncode=="340826" & year==2002

replace regioncode="340827" if regioncode=="340827" & year==2002

replace regioncode="340828" if regioncode=="340828" & year==2002

replace regioncode="340881" if regioncode=="340881" & year==2002

replace regioncode="341002" if regioncode=="341002" & year==2002

replace regioncode="341003" if regioncode=="341003" & year==2002

replace regioncode="341004" if regioncode=="341004" & year==2002

replace regioncode="341021" if regioncode=="341021" & year==2002

replace regioncode="341022" if regioncode=="341022" & year==2002

replace regioncode="341023" if regioncode=="341023" & year==2002

replace regioncode="341024" if regioncode=="341024" & year==2002

replace regioncode="341102" if regioncode=="341102" & year==2002

replace regioncode="341103" if regioncode=="341103" & year==2002

replace regioncode="341122" if regioncode=="341122" & year==2002

replace regioncode="341124" if regioncode=="341124" & year==2002

replace regioncode="341125" if regioncode=="341125" & year==2002

replace regioncode="341126" if regioncode=="341126" & year==2002

replace regioncode="341181" if regioncode=="341181" & year==2002

replace regioncode="341182" if regioncode=="341182" & year==2002

replace regioncode="341202" if regioncode=="341202" & year==2002

replace regioncode="341203" if regioncode=="341203" & year==2002

replace regioncode="341204" if regioncode=="341204" & year==2002

replace regioncode="341221" if regioncode=="341221" & year==2002

replace regioncode="341222" if regioncode=="341222" & year==2002

replace regioncode="341621" if regioncode=="341621" & year==2002

replace regioncode="341622" if regioncode=="341622" & year==2002

replace regioncode="341225" if regioncode=="341225" & year==2002

replace regioncode="341226" if regioncode=="341226" & year==2002

replace regioncode="341623" if regioncode=="341623" & year==2002

replace regioncode="341282" if regioncode=="341282" & year==2002

replace regioncode="341302" if regioncode=="341302" & year==2002

replace regioncode="341321" if regioncode=="341321" & year==2002

replace regioncode="341322" if regioncode=="341322" & year==2002

replace regioncode="341323" if regioncode=="341323" & year==2002

replace regioncode="341324" if regioncode=="341324" & year==2002

replace regioncode="341402" if regioncode=="341402" & year==2002

replace regioncode="341421" if regioncode=="341421" & year==2002

replace regioncode="341422" if regioncode=="341422" & year==2002

replace regioncode="341423" if regioncode=="341423" & year==2002

replace regioncode="341424" if regioncode=="341424" & year==2002

replace regioncode="341521" if regioncode=="341521" & year==2002

replace regioncode="341522" if regioncode=="341522" & year==2002

replace regioncode="341523" if regioncode=="341523" & year==2002

replace regioncode="341524" if regioncode=="341524" & year==2002

replace regioncode="341525" if regioncode=="341525" & year==2002

replace regioncode="341802" if regioncode=="341802" & year==2002

replace regioncode="341881" if regioncode=="341881" & year==2002

replace regioncode="341821" if regioncode=="341821" & year==2002

replace regioncode="341822" if regioncode=="341822" & year==2002

replace regioncode="341823" if regioncode=="341823" & year==2002

replace regioncode="341825" if regioncode=="341825" & year==2002

replace regioncode="341824" if regioncode=="341824" & year==2002

replace regioncode="341702" if regioncode=="341702" & year==2002

replace regioncode="341721" if regioncode=="341721" & year==2002

replace regioncode="341722" if regioncode=="341722" & year==2002

replace regioncode="341723" if regioncode=="341723" & year==2002

replace regioncode="341502" if regioncode=="342401" & year==2002

replace regioncode="341602" if regioncode=="341602" & year==2002

replace regioncode="350102" if regioncode=="350102" & year==2002

replace regioncode="350103" if regioncode=="350103" & year==2002

replace regioncode="350104" if regioncode=="350104" & year==2002

replace regioncode="350105" if regioncode=="350105" & year==2002

replace regioncode="350111" if regioncode=="350111" & year==2002

replace regioncode="350121" if regioncode=="350121" & year==2002

replace regioncode="350122" if regioncode=="350122" & year==2002

replace regioncode="350123" if regioncode=="350123" & year==2002

replace regioncode="350124" if regioncode=="350124" & year==2002

replace regioncode="350125" if regioncode=="350125" & year==2002

replace regioncode="350128" if regioncode=="350128" & year==2002

replace regioncode="350181" if regioncode=="350181" & year==2002

replace regioncode="350182" if regioncode=="350182" & year==2002

replace regioncode="350203" if regioncode=="350203" & year==2002

replace regioncode="350205" if regioncode=="350205" & year==2002

replace regioncode="350206" if regioncode=="350206" & year==2002

replace regioncode="350211" if regioncode=="350211" & year==2002

replace regioncode="350212" if regioncode=="350212" & year==2002

replace regioncode="350213" if regioncode=="350213" & year==2002

replace regioncode="350302" if regioncode=="350302" & year==2002

replace regioncode="350303" if regioncode=="350303" & year==2002

replace regioncode="350321" if regioncode=="350321" & year==2002

replace regioncode="350322" if regioncode=="350322" & year==2002

replace regioncode="350402" if regioncode=="350402" & year==2002

replace regioncode="350403" if regioncode=="350403" & year==2002

replace regioncode="350421" if regioncode=="350421" & year==2002

replace regioncode="350423" if regioncode=="350423" & year==2002

replace regioncode="350424" if regioncode=="350424" & year==2002

replace regioncode="350425" if regioncode=="350425" & year==2002

replace regioncode="350426" if regioncode=="350426" & year==2002

replace regioncode="350427" if regioncode=="350427" & year==2002

replace regioncode="350428" if regioncode=="350428" & year==2002

replace regioncode="350429" if regioncode=="350429" & year==2002

replace regioncode="350430" if regioncode=="350430" & year==2002

replace regioncode="350481" if regioncode=="350481" & year==2002

replace regioncode="350502" if regioncode=="350502" & year==2002

replace regioncode="350503" if regioncode=="350503" & year==2002

replace regioncode="350504" if regioncode=="350504" & year==2002

replace regioncode="350505" if regioncode=="350505" & year==2002

replace regioncode="350521" if regioncode=="350521" & year==2002

replace regioncode="350524" if regioncode=="350524" & year==2002

replace regioncode="350525" if regioncode=="350525" & year==2002

replace regioncode="350526" if regioncode=="350526" & year==2002

replace regioncode="350581" if regioncode=="350581" & year==2002

replace regioncode="350582" if regioncode=="350582" & year==2002

replace regioncode="350583" if regioncode=="350583" & year==2002

replace regioncode="350602" if regioncode=="350602" & year==2002

replace regioncode="350603" if regioncode=="350603" & year==2002

replace regioncode="350622" if regioncode=="350622" & year==2002

replace regioncode="350623" if regioncode=="350623" & year==2002

replace regioncode="350624" if regioncode=="350624" & year==2002

replace regioncode="350625" if regioncode=="350625" & year==2002

replace regioncode="350626" if regioncode=="350626" & year==2002

replace regioncode="350627" if regioncode=="350627" & year==2002

replace regioncode="350628" if regioncode=="350628" & year==2002

replace regioncode="350629" if regioncode=="350629" & year==2002

replace regioncode="350681" if regioncode=="350681" & year==2002

replace regioncode="350702" if regioncode=="350702" & year==2002

replace regioncode="350721" if regioncode=="350721" & year==2002

replace regioncode="350722" if regioncode=="350722" & year==2002

replace regioncode="350723" if regioncode=="350723" & year==2002

replace regioncode="350724" if regioncode=="350724" & year==2002

replace regioncode="350725" if regioncode=="350725" & year==2002

replace regioncode="350781" if regioncode=="350781" & year==2002

replace regioncode="350782" if regioncode=="350782" & year==2002

replace regioncode="350783" if regioncode=="350783" & year==2002

replace regioncode="350784" if regioncode=="350784" & year==2002

replace regioncode="350802" if regioncode=="350802" & year==2002

replace regioncode="350821" if regioncode=="350821" & year==2002

replace regioncode="350822" if regioncode=="350822" & year==2002

replace regioncode="350823" if regioncode=="350823" & year==2002

replace regioncode="350824" if regioncode=="350824" & year==2002

replace regioncode="350825" if regioncode=="350825" & year==2002

replace regioncode="350881" if regioncode=="350881" & year==2002

replace regioncode="350902" if regioncode=="350902" & year==2002

replace regioncode="350981" if regioncode=="350981" & year==2002

replace regioncode="350982" if regioncode=="350982" & year==2002

replace regioncode="350921" if regioncode=="350921" & year==2002

replace regioncode="350922" if regioncode=="350922" & year==2002

replace regioncode="350923" if regioncode=="350923" & year==2002

replace regioncode="350924" if regioncode=="350924" & year==2002

replace regioncode="350925" if regioncode=="350925" & year==2002

replace regioncode="350926" if regioncode=="350926" & year==2002

replace regioncode="360102" if regioncode=="360102" & year==2002

replace regioncode="360103" if regioncode=="360103" & year==2002

replace regioncode="360104" if regioncode=="360104" & year==2002

replace regioncode="360105" if regioncode=="360105" & year==2002

replace regioncode="360111" if regioncode=="360111" & year==2002

replace regioncode="360121" if regioncode=="360121" & year==2002

replace regioncode="360122" if regioncode=="360122" & year==2002

replace regioncode="360123" if regioncode=="360123" & year==2002

replace regioncode="360124" if regioncode=="360124" & year==2002

replace regioncode="360202" if regioncode=="360202" & year==2002

replace regioncode="360203" if regioncode=="360203" & year==2002

replace regioncode="360222" if regioncode=="360222" & year==2002

replace regioncode="360281" if regioncode=="360281" & year==2002

replace regioncode="360302" if regioncode=="360302" & year==2002

replace regioncode="360313" if regioncode=="360313" & year==2002

replace regioncode="360321" if regioncode=="360321" & year==2002

replace regioncode="360322" if regioncode=="360322" & year==2002

replace regioncode="360323" if regioncode=="360323" & year==2002

replace regioncode="360402" if regioncode=="360402" & year==2002

replace regioncode="360403" if regioncode=="360403" & year==2002

replace regioncode="360421" if regioncode=="360421" & year==2002

replace regioncode="360423" if regioncode=="360423" & year==2002

replace regioncode="360424" if regioncode=="360424" & year==2002

replace regioncode="360425" if regioncode=="360425" & year==2002

replace regioncode="360426" if regioncode=="360426" & year==2002

replace regioncode="360427" if regioncode=="360427" & year==2002

replace regioncode="360428" if regioncode=="360428" & year==2002

replace regioncode="360429" if regioncode=="360429" & year==2002

replace regioncode="360430" if regioncode=="360430" & year==2002

replace regioncode="360481" if regioncode=="360481" & year==2002

replace regioncode="360502" if regioncode=="360502" & year==2002

replace regioncode="360521" if regioncode=="360521" & year==2002

replace regioncode="360602" if regioncode=="360602" & year==2002

replace regioncode="360622" if regioncode=="360622" & year==2002

replace regioncode="360681" if regioncode=="360681" & year==2002

replace regioncode="360702" if regioncode=="360702" & year==2002

replace regioncode="360721" if regioncode=="360721" & year==2002

replace regioncode="360722" if regioncode=="360722" & year==2002

replace regioncode="360723" if regioncode=="360723" & year==2002

replace regioncode="360724" if regioncode=="360724" & year==2002

replace regioncode="360725" if regioncode=="360725" & year==2002

replace regioncode="360726" if regioncode=="360726" & year==2002

replace regioncode="360727" if regioncode=="360727" & year==2002

replace regioncode="360728" if regioncode=="360728" & year==2002

replace regioncode="360729" if regioncode=="360729" & year==2002

replace regioncode="360730" if regioncode=="360730" & year==2002

replace regioncode="360731" if regioncode=="360731" & year==2002

replace regioncode="360732" if regioncode=="360732" & year==2002

replace regioncode="360733" if regioncode=="360733" & year==2002

replace regioncode="360734" if regioncode=="360734" & year==2002

replace regioncode="360735" if regioncode=="360735" & year==2002

replace regioncode="360781" if regioncode=="360781" & year==2002

replace regioncode="360782" if regioncode=="360782" & year==2002

replace regioncode="360802" if regioncode=="360802" & year==2002

replace regioncode="360821" if regioncode=="360821" & year==2002

replace regioncode="360822" if regioncode=="360822" & year==2002

replace regioncode="360823" if regioncode=="360823" & year==2002

replace regioncode="360824" if regioncode=="360824" & year==2002

replace regioncode="360825" if regioncode=="360825" & year==2002

replace regioncode="360826" if regioncode=="360826" & year==2002

replace regioncode="360827" if regioncode=="360827" & year==2002

replace regioncode="360828" if regioncode=="360828" & year==2002

replace regioncode="360829" if regioncode=="360829" & year==2002

replace regioncode="360830" if regioncode=="360830" & year==2002

replace regioncode="360881" if regioncode=="360881" & year==2002

replace regioncode="360902" if regioncode=="360902" & year==2002

replace regioncode="360921" if regioncode=="360921" & year==2002

replace regioncode="360922" if regioncode=="360922" & year==2002

replace regioncode="360923" if regioncode=="360923" & year==2002

replace regioncode="360924" if regioncode=="360924" & year==2002

replace regioncode="360925" if regioncode=="360925" & year==2002

replace regioncode="360926" if regioncode=="360926" & year==2002

replace regioncode="360981" if regioncode=="360981" & year==2002

replace regioncode="360982" if regioncode=="360982" & year==2002

replace regioncode="360983" if regioncode=="360983" & year==2002

replace regioncode="361002" if regioncode=="361002" & year==2002

replace regioncode="361021" if regioncode=="361021" & year==2002

replace regioncode="361022" if regioncode=="361022" & year==2002

replace regioncode="361023" if regioncode=="361023" & year==2002

replace regioncode="361024" if regioncode=="361024" & year==2002

replace regioncode="361025" if regioncode=="361025" & year==2002

replace regioncode="361026" if regioncode=="361026" & year==2002

replace regioncode="361027" if regioncode=="361027" & year==2002

replace regioncode="361028" if regioncode=="361028" & year==2002

replace regioncode="361029" if regioncode=="361029" & year==2002

replace regioncode="361030" if regioncode=="361030" & year==2002

replace regioncode="361102" if regioncode=="361102" & year==2002

replace regioncode="361121" if regioncode=="361121" & year==2002

replace regioncode="361122" if regioncode=="361122" & year==2002

replace regioncode="361123" if regioncode=="361123" & year==2002

replace regioncode="361124" if regioncode=="361124" & year==2002

replace regioncode="361125" if regioncode=="361125" & year==2002

replace regioncode="361126" if regioncode=="361126" & year==2002

replace regioncode="361127" if regioncode=="361127" & year==2002

replace regioncode="361128" if regioncode=="361128" & year==2002

replace regioncode="361129" if regioncode=="361129" & year==2002

replace regioncode="361130" if regioncode=="361130" & year==2002

replace regioncode="361181" if regioncode=="361181" & year==2002

replace regioncode="370102" if regioncode=="370102" & year==2002

replace regioncode="370103" if regioncode=="370103" & year==2002

replace regioncode="370104" if regioncode=="370104" & year==2002

replace regioncode="370105" if regioncode=="370105" & year==2002

replace regioncode="370112" if regioncode=="370112" & year==2002

replace regioncode="370113" if regioncode=="370113" & year==2002

replace regioncode="370124" if regioncode=="370124" & year==2002

replace regioncode="370125" if regioncode=="370125" & year==2002

replace regioncode="370126" if regioncode=="370126" & year==2002

replace regioncode="370181" if regioncode=="370181" & year==2002

replace regioncode="370202" if regioncode=="370202" & year==2002

replace regioncode="370203" if regioncode=="370203" & year==2002

replace regioncode="370205" if regioncode=="370205" & year==2002

replace regioncode="370211" if regioncode=="370211" & year==2002

replace regioncode="370212" if regioncode=="370212" & year==2002

replace regioncode="370213" if regioncode=="370213" & year==2002

replace regioncode="370214" if regioncode=="370214" & year==2002

replace regioncode="370281" if regioncode=="370281" & year==2002

replace regioncode="370282" if regioncode=="370282" & year==2002

replace regioncode="370283" if regioncode=="370283" & year==2002

replace regioncode="370284" if regioncode=="370284" & year==2002

replace regioncode="370285" if regioncode=="370285" & year==2002

replace regioncode="370302" if regioncode=="370302" & year==2002

replace regioncode="370303" if regioncode=="370303" & year==2002

replace regioncode="370304" if regioncode=="370304" & year==2002

replace regioncode="370305" if regioncode=="370305" & year==2002

replace regioncode="370306" if regioncode=="370306" & year==2002

replace regioncode="370321" if regioncode=="370321" & year==2002

replace regioncode="370322" if regioncode=="370322" & year==2002

replace regioncode="370323" if regioncode=="370323" & year==2002

replace regioncode="370402" if regioncode=="370402" & year==2002

replace regioncode="370403" if regioncode=="370403" & year==2002

replace regioncode="370404" if regioncode=="370404" & year==2002

replace regioncode="370405" if regioncode=="370405" & year==2002

replace regioncode="370406" if regioncode=="370406" & year==2002

replace regioncode="370481" if regioncode=="370481" & year==2002

replace regioncode="370502" if regioncode=="370502" & year==2002

replace regioncode="370503" if regioncode=="370503" & year==2002

replace regioncode="370521" if regioncode=="370521" & year==2002

replace regioncode="370522" if regioncode=="370522" & year==2002

replace regioncode="370523" if regioncode=="370523" & year==2002

replace regioncode="370602" if regioncode=="370602" & year==2002

replace regioncode="370611" if regioncode=="370611" & year==2002

replace regioncode="370612" if regioncode=="370612" & year==2002

replace regioncode="370613" if regioncode=="370613" & year==2002

replace regioncode="370634" if regioncode=="370634" & year==2002

replace regioncode="370681" if regioncode=="370681" & year==2002

replace regioncode="370682" if regioncode=="370682" & year==2002

replace regioncode="370683" if regioncode=="370683" & year==2002

replace regioncode="370684" if regioncode=="370684" & year==2002

replace regioncode="370685" if regioncode=="370685" & year==2002

replace regioncode="370686" if regioncode=="370686" & year==2002

replace regioncode="370687" if regioncode=="370687" & year==2002

replace regioncode="370702" if regioncode=="370702" & year==2002

replace regioncode="370703" if regioncode=="370703" & year==2002

replace regioncode="370704" if regioncode=="370704" & year==2002

replace regioncode="370705" if regioncode=="370705" & year==2002

replace regioncode="370724" if regioncode=="370724" & year==2002

replace regioncode="370725" if regioncode=="370725" & year==2002

replace regioncode="370781" if regioncode=="370781" & year==2002

replace regioncode="370782" if regioncode=="370782" & year==2002

replace regioncode="370783" if regioncode=="370783" & year==2002

replace regioncode="370784" if regioncode=="370784" & year==2002

replace regioncode="370785" if regioncode=="370785" & year==2002

replace regioncode="370786" if regioncode=="370786" & year==2002

replace regioncode="370802" if regioncode=="370802" & year==2002

replace regioncode="370811" if regioncode=="370811" & year==2002

replace regioncode="370826" if regioncode=="370826" & year==2002

replace regioncode="370827" if regioncode=="370827" & year==2002

replace regioncode="370828" if regioncode=="370828" & year==2002

replace regioncode="370829" if regioncode=="370829" & year==2002

replace regioncode="370830" if regioncode=="370830" & year==2002

replace regioncode="370831" if regioncode=="370831" & year==2002

replace regioncode="370832" if regioncode=="370832" & year==2002

replace regioncode="370881" if regioncode=="370881" & year==2002

replace regioncode="370882" if regioncode=="370882" & year==2002

replace regioncode="370883" if regioncode=="370883" & year==2002

replace regioncode="370902" if regioncode=="370902" & year==2002

replace regioncode="370903" if regioncode=="370903" & year==2002

replace regioncode="370921" if regioncode=="370921" & year==2002

replace regioncode="370923" if regioncode=="370923" & year==2002

replace regioncode="370982" if regioncode=="370982" & year==2002

replace regioncode="370983" if regioncode=="370983" & year==2002

replace regioncode="371002" if regioncode=="371002" & year==2002

replace regioncode="371081" if regioncode=="371081" & year==2002

replace regioncode="371082" if regioncode=="371082" & year==2002

replace regioncode="371083" if regioncode=="371083" & year==2002

replace regioncode="371102" if regioncode=="371102" & year==2002

replace regioncode="371121" if regioncode=="371121" & year==2002

replace regioncode="371122" if regioncode=="371122" & year==2002

replace regioncode="371202" if regioncode=="371202" & year==2002

replace regioncode="371203" if regioncode=="371203" & year==2002

replace regioncode="371302" if regioncode=="371302" & year==2002

replace regioncode="371311" if regioncode=="371311" & year==2002

replace regioncode="371312" if regioncode=="371312" & year==2002

replace regioncode="371321" if regioncode=="371321" & year==2002

replace regioncode="371322" if regioncode=="371322" & year==2002

replace regioncode="371323" if regioncode=="371323" & year==2002

replace regioncode="371324" if regioncode=="371324" & year==2002

replace regioncode="371325" if regioncode=="371325" & year==2002

replace regioncode="371326" if regioncode=="371326" & year==2002

replace regioncode="371327" if regioncode=="371327" & year==2002

replace regioncode="371328" if regioncode=="371328" & year==2002

replace regioncode="371329" if regioncode=="371329" & year==2002

replace regioncode="371402" if regioncode=="371402" & year==2002

replace regioncode="371421" if regioncode=="371421" & year==2002

replace regioncode="371422" if regioncode=="371422" & year==2002

replace regioncode="371423" if regioncode=="371423" & year==2002

replace regioncode="371424" if regioncode=="371424" & year==2002

replace regioncode="371425" if regioncode=="371425" & year==2002

replace regioncode="371426" if regioncode=="371426" & year==2002

replace regioncode="371427" if regioncode=="371427" & year==2002

replace regioncode="371428" if regioncode=="371428" & year==2002

replace regioncode="371481" if regioncode=="371481" & year==2002

replace regioncode="371482" if regioncode=="371482" & year==2002

replace regioncode="371502" if regioncode=="371502" & year==2002

replace regioncode="371521" if regioncode=="371521" & year==2002

replace regioncode="371522" if regioncode=="371522" & year==2002

replace regioncode="371523" if regioncode=="371523" & year==2002

replace regioncode="371524" if regioncode=="371524" & year==2002

replace regioncode="371525" if regioncode=="371525" & year==2002

replace regioncode="371526" if regioncode=="371526" & year==2002

replace regioncode="371581" if regioncode=="371581" & year==2002

replace regioncode="371602" if regioncode=="371602" & year==2002

replace regioncode="371621" if regioncode=="371621" & year==2002

replace regioncode="371622" if regioncode=="371622" & year==2002

replace regioncode="371623" if regioncode=="371623" & year==2002

replace regioncode="371624" if regioncode=="371624" & year==2002

replace regioncode="371625" if regioncode=="371625" & year==2002

replace regioncode="371626" if regioncode=="371626" & year==2002

replace regioncode="371702" if regioncode=="371702" & year==2002

replace regioncode="371721" if regioncode=="371721" & year==2002

replace regioncode="371722" if regioncode=="371722" & year==2002

replace regioncode="371723" if regioncode=="371723" & year==2002

replace regioncode="371724" if regioncode=="371724" & year==2002

replace regioncode="371725" if regioncode=="371725" & year==2002

replace regioncode="371726" if regioncode=="371726" & year==2002

replace regioncode="371727" if regioncode=="371727" & year==2002

replace regioncode="371728" if regioncode=="371728" & year==2002

replace regioncode="410102" if regioncode=="410102" & year==2002

replace regioncode="410103" if regioncode=="410103" & year==2002

replace regioncode="410104" if regioncode=="410104" & year==2002

replace regioncode="410105" if regioncode=="410105" & year==2002

replace regioncode="410106" if regioncode=="410106" & year==2002

replace regioncode="410108" if regioncode=="410108" & year==2002

replace regioncode="410122" if regioncode=="410122" & year==2002

replace regioncode="410181" if regioncode=="410181" & year==2002

replace regioncode="410182" if regioncode=="410182" & year==2002

replace regioncode="410183" if regioncode=="410183" & year==2002

replace regioncode="410184" if regioncode=="410184" & year==2002

replace regioncode="410185" if regioncode=="410185" & year==2002

replace regioncode="410202" if regioncode=="410202" & year==2002

replace regioncode="410203" if regioncode=="410203" & year==2002

replace regioncode="410204" if regioncode=="410204" & year==2002

replace regioncode="410205" if regioncode=="410205" & year==2002

replace regioncode="410211" if regioncode=="410211" & year==2002

replace regioncode="410221" if regioncode=="410221" & year==2002

replace regioncode="410222" if regioncode=="410222" & year==2002

replace regioncode="410223" if regioncode=="410223" & year==2002

replace regioncode="410224" if regioncode=="410224" & year==2002

replace regioncode="410225" if regioncode=="410225" & year==2002

replace regioncode="410302" if regioncode=="410302" & year==2002

replace regioncode="410303" if regioncode=="410303" & year==2002

replace regioncode="410304" if regioncode=="410304" & year==2002

replace regioncode="410305" if regioncode=="410305" & year==2002

replace regioncode="410306" if regioncode=="410306" & year==2002

replace regioncode="410307" if regioncode=="410307" & year==2002

replace regioncode="410322" if regioncode=="410322" & year==2002

replace regioncode="410323" if regioncode=="410323" & year==2002

replace regioncode="410324" if regioncode=="410324" & year==2002

replace regioncode="410325" if regioncode=="410325" & year==2002

replace regioncode="410326" if regioncode=="410326" & year==2002

replace regioncode="410327" if regioncode=="410327" & year==2002

replace regioncode="410328" if regioncode=="410328" & year==2002

replace regioncode="410329" if regioncode=="410329" & year==2002

replace regioncode="410381" if regioncode=="410381" & year==2002

replace regioncode="410402" if regioncode=="410402" & year==2002

replace regioncode="410403" if regioncode=="410403" & year==2002

replace regioncode="410404" if regioncode=="410404" & year==2002

replace regioncode="410411" if regioncode=="410411" & year==2002

replace regioncode="410421" if regioncode=="410421" & year==2002

replace regioncode="410422" if regioncode=="410422" & year==2002

replace regioncode="410423" if regioncode=="410423" & year==2002

replace regioncode="410425" if regioncode=="410425" & year==2002

replace regioncode="410481" if regioncode=="410481" & year==2002

replace regioncode="410482" if regioncode=="410482" & year==2002

replace regioncode="410502" if regioncode=="410502" & year==2002

replace regioncode="410503" if regioncode=="410503" & year==2002

replace regioncode="410504" if regioncode=="410504" & year==2002

replace regioncode="410511" if regioncode=="410511" & year==2002

replace regioncode="410522" if regioncode=="410522" & year==2002

replace regioncode="410523" if regioncode=="410523" & year==2002

replace regioncode="410526" if regioncode=="410526" & year==2002

replace regioncode="410527" if regioncode=="410527" & year==2002

replace regioncode="410581" if regioncode=="410581" & year==2002

replace regioncode="410602" if regioncode=="410602" & year==2002

replace regioncode="410603" if regioncode=="410603" & year==2002

replace regioncode="410611" if regioncode=="410611" & year==2002

replace regioncode="410621" if regioncode=="410621" & year==2002

replace regioncode="410622" if regioncode=="410622" & year==2002

replace regioncode="410702" if regioncode=="410702" & year==2002

replace regioncode="410703" if regioncode=="410703" & year==2002

replace regioncode="410704" if regioncode=="410704" & year==2002

replace regioncode="410711" if regioncode=="410711" & year==2002

replace regioncode="410721" if regioncode=="410721" & year==2002

replace regioncode="410724" if regioncode=="410724" & year==2002

replace regioncode="410725" if regioncode=="410725" & year==2002

replace regioncode="410726" if regioncode=="410726" & year==2002

replace regioncode="410727" if regioncode=="410727" & year==2002

replace regioncode="410728" if regioncode=="410728" & year==2002

replace regioncode="410781" if regioncode=="410781" & year==2002

replace regioncode="410782" if regioncode=="410782" & year==2002

replace regioncode="410802" if regioncode=="410802" & year==2002

replace regioncode="410803" if regioncode=="410803" & year==2002

replace regioncode="410804" if regioncode=="410804" & year==2002

replace regioncode="410811" if regioncode=="410811" & year==2002

replace regioncode="410821" if regioncode=="410821" & year==2002

replace regioncode="410822" if regioncode=="410822" & year==2002

replace regioncode="410823" if regioncode=="410823" & year==2002

replace regioncode="410825" if regioncode=="410825" & year==2002

replace regioncode="410881" if regioncode=="410881" & year==2002

replace regioncode="410882" if regioncode=="410882" & year==2002

replace regioncode="410883" if regioncode=="410883" & year==2002

replace regioncode="410902" if regioncode=="410902" & year==2002

replace regioncode="410922" if regioncode=="410922" & year==2002

replace regioncode="410923" if regioncode=="410923" & year==2002

replace regioncode="410926" if regioncode=="410926" & year==2002

replace regioncode="410927" if regioncode=="410927" & year==2002

replace regioncode="410928" if regioncode=="410928" & year==2002

replace regioncode="411002" if regioncode=="411002" & year==2002

replace regioncode="411023" if regioncode=="411023" & year==2002

replace regioncode="411024" if regioncode=="411024" & year==2002

replace regioncode="411025" if regioncode=="411025" & year==2002

replace regioncode="411081" if regioncode=="411081" & year==2002

replace regioncode="411082" if regioncode=="411082" & year==2002

replace regioncode="411102" if regioncode=="411102" & year==2002

replace regioncode="411123" if regioncode=="411123" & year==2002

replace regioncode="411121" if regioncode=="411121" & year==2002

replace regioncode="411122" if regioncode=="411122" & year==2002

replace regioncode="411202" if regioncode=="411202" & year==2002

replace regioncode="411221" if regioncode=="411221" & year==2002

replace regioncode="411222" if regioncode=="411222" & year==2002

replace regioncode="411224" if regioncode=="411224" & year==2002

replace regioncode="411281" if regioncode=="411281" & year==2002

replace regioncode="411282" if regioncode=="411282" & year==2002

replace regioncode="411302" if regioncode=="411302" & year==2002

replace regioncode="411303" if regioncode=="411303" & year==2002

replace regioncode="411321" if regioncode=="411321" & year==2002

replace regioncode="411322" if regioncode=="411322" & year==2002

replace regioncode="411323" if regioncode=="411323" & year==2002

replace regioncode="411324" if regioncode=="411324" & year==2002

replace regioncode="411325" if regioncode=="411325" & year==2002

replace regioncode="411326" if regioncode=="411326" & year==2002

replace regioncode="411327" if regioncode=="411327" & year==2002

replace regioncode="411328" if regioncode=="411328" & year==2002

replace regioncode="411329" if regioncode=="411329" & year==2002

replace regioncode="411330" if regioncode=="411330" & year==2002

replace regioncode="411381" if regioncode=="411381" & year==2002

replace regioncode="411402" if regioncode=="411402" & year==2002

replace regioncode="411403" if regioncode=="411403" & year==2002

replace regioncode="411421" if regioncode=="411421" & year==2002

replace regioncode="411422" if regioncode=="411422" & year==2002

replace regioncode="411423" if regioncode=="411423" & year==2002

replace regioncode="411424" if regioncode=="411424" & year==2002

replace regioncode="411425" if regioncode=="411425" & year==2002

replace regioncode="411426" if regioncode=="411426" & year==2002

replace regioncode="411481" if regioncode=="411481" & year==2002

replace regioncode="411502" if regioncode=="411502" & year==2002

replace regioncode="411503" if regioncode=="411503" & year==2002

replace regioncode="411521" if regioncode=="411521" & year==2002

replace regioncode="411522" if regioncode=="411522" & year==2002

replace regioncode="411523" if regioncode=="411523" & year==2002

replace regioncode="411524" if regioncode=="411524" & year==2002

replace regioncode="411525" if regioncode=="411525" & year==2002

replace regioncode="411526" if regioncode=="411526" & year==2002

replace regioncode="411527" if regioncode=="411527" & year==2002

replace regioncode="411528" if regioncode=="411528" & year==2002

replace regioncode="411602" if regioncode=="411602" & year==2002

replace regioncode="411621" if regioncode=="411621" & year==2002

replace regioncode="411622" if regioncode=="411622" & year==2002

replace regioncode="411623" if regioncode=="411623" & year==2002

replace regioncode="411624" if regioncode=="411624" & year==2002

replace regioncode="411625" if regioncode=="411625" & year==2002

replace regioncode="411626" if regioncode=="411626" & year==2002

replace regioncode="411627" if regioncode=="411627" & year==2002

replace regioncode="411628" if regioncode=="411628" & year==2002

replace regioncode="411681" if regioncode=="411681" & year==2002

replace regioncode="411702" if regioncode=="411702" & year==2002

replace regioncode="411721" if regioncode=="411721" & year==2002

replace regioncode="411722" if regioncode=="411722" & year==2002

replace regioncode="411723" if regioncode=="411723" & year==2002

replace regioncode="411724" if regioncode=="411724" & year==2002

replace regioncode="411725" if regioncode=="411725" & year==2002

replace regioncode="411726" if regioncode=="411726" & year==2002

replace regioncode="411727" if regioncode=="411727" & year==2002

replace regioncode="411728" if regioncode=="411728" & year==2002

replace regioncode="411729" if regioncode=="411729" & year==2002

replace regioncode="420102" if regioncode=="420102" & year==2002

replace regioncode="420103" if regioncode=="420103" & year==2002

replace regioncode="420104" if regioncode=="420104" & year==2002

replace regioncode="420105" if regioncode=="420105" & year==2002

replace regioncode="420106" if regioncode=="420106" & year==2002

replace regioncode="420107" if regioncode=="420107" & year==2002

replace regioncode="420111" if regioncode=="420111" & year==2002

replace regioncode="420112" if regioncode=="420112" & year==2002

replace regioncode="420113" if regioncode=="420113" & year==2002

replace regioncode="420114" if regioncode=="420114" & year==2002

replace regioncode="420115" if regioncode=="420115" & year==2002

replace regioncode="420116" if regioncode=="420116" & year==2002

replace regioncode="420117" if regioncode=="420117" & year==2002

replace regioncode="420202" if regioncode=="420202" & year==2002

replace regioncode="420203" if regioncode=="420203" & year==2002

replace regioncode="420204" if regioncode=="420204" & year==2002

replace regioncode="420205" if regioncode=="420205" & year==2002

replace regioncode="420222" if regioncode=="420222" & year==2002

replace regioncode="420281" if regioncode=="420281" & year==2002

replace regioncode="420302" if regioncode=="420302" & year==2002

replace regioncode="420303" if regioncode=="420303" & year==2002

replace regioncode="420321" if regioncode=="420321" & year==2002

replace regioncode="420322" if regioncode=="420322" & year==2002

replace regioncode="420323" if regioncode=="420323" & year==2002

replace regioncode="420324" if regioncode=="420324" & year==2002

replace regioncode="420325" if regioncode=="420325" & year==2002

replace regioncode="420381" if regioncode=="420381" & year==2002

replace regioncode="420502" if regioncode=="420502" & year==2002

replace regioncode="420503" if regioncode=="420503" & year==2002

replace regioncode="420504" if regioncode=="420504" & year==2002

replace regioncode="420505" if regioncode=="420505" & year==2002

replace regioncode="420506" if regioncode=="420506" & year==2002

replace regioncode="420525" if regioncode=="420525" & year==2002

replace regioncode="420526" if regioncode=="420526" & year==2002

replace regioncode="420527" if regioncode=="420527" & year==2002

replace regioncode="420528" if regioncode=="420528" & year==2002

replace regioncode="420529" if regioncode=="420529" & year==2002

replace regioncode="420581" if regioncode=="420581" & year==2002

replace regioncode="420582" if regioncode=="420582" & year==2002

replace regioncode="420583" if regioncode=="420583" & year==2002

replace regioncode="420602" if regioncode=="420602" & year==2002

replace regioncode="420606" if regioncode=="420606" & year==2002

replace regioncode="420607" if regioncode=="420607" & year==2002

replace regioncode="420624" if regioncode=="420624" & year==2002

replace regioncode="420625" if regioncode=="420625" & year==2002

replace regioncode="420626" if regioncode=="420626" & year==2002

replace regioncode="420682" if regioncode=="420682" & year==2002

replace regioncode="420683" if regioncode=="420683" & year==2002

replace regioncode="420684" if regioncode=="420684" & year==2002

replace regioncode="420702" if regioncode=="420702" & year==2002

replace regioncode="420703" if regioncode=="420703" & year==2002

replace regioncode="420704" if regioncode=="420704" & year==2002

replace regioncode="420802" if regioncode=="420802" & year==2002

replace regioncode="420804" if regioncode=="420804" & year==2002

replace regioncode="420821" if regioncode=="420821" & year==2002

replace regioncode="420822" if regioncode=="420822" & year==2002

replace regioncode="420881" if regioncode=="420881" & year==2002

replace regioncode="420902" if regioncode=="420902" & year==2002

replace regioncode="420921" if regioncode=="420921" & year==2002

replace regioncode="420922" if regioncode=="420922" & year==2002

replace regioncode="420923" if regioncode=="420923" & year==2002

replace regioncode="420981" if regioncode=="420981" & year==2002

replace regioncode="420982" if regioncode=="420982" & year==2002

replace regioncode="421381" if regioncode=="421381" & year==2002

replace regioncode="420984" if regioncode=="420984" & year==2002

replace regioncode="421002" if regioncode=="421002" & year==2002

replace regioncode="421003" if regioncode=="421003" & year==2002

replace regioncode="421022" if regioncode=="421022" & year==2002

replace regioncode="421023" if regioncode=="421023" & year==2002

replace regioncode="421024" if regioncode=="421024" & year==2002

replace regioncode="421081" if regioncode=="421081" & year==2002

replace regioncode="421083" if regioncode=="421083" & year==2002

replace regioncode="421087" if regioncode=="421087" & year==2002

replace regioncode="421102" if regioncode=="421102" & year==2002

replace regioncode="421121" if regioncode=="421121" & year==2002

replace regioncode="421122" if regioncode=="421122" & year==2002

replace regioncode="421123" if regioncode=="421123" & year==2002

replace regioncode="421124" if regioncode=="421124" & year==2002

replace regioncode="421125" if regioncode=="421125" & year==2002

replace regioncode="421126" if regioncode=="421126" & year==2002

replace regioncode="421127" if regioncode=="421127" & year==2002

replace regioncode="421181" if regioncode=="421181" & year==2002

replace regioncode="421182" if regioncode=="421182" & year==2002

replace regioncode="421202" if regioncode=="421202" & year==2002

replace regioncode="421221" if regioncode=="421221" & year==2002

replace regioncode="421222" if regioncode=="421222" & year==2002

replace regioncode="421223" if regioncode=="421223" & year==2002

replace regioncode="421224" if regioncode=="421224" & year==2002

replace regioncode="421281" if regioncode=="421281" & year==2002

replace regioncode="421302" if regioncode=="421302" & year==2002

replace regioncode="422801" if regioncode=="422801" & year==2002

replace regioncode="422802" if regioncode=="422802" & year==2002

replace regioncode="422822" if regioncode=="422822" & year==2002

replace regioncode="422823" if regioncode=="422823" & year==2002

replace regioncode="422825" if regioncode=="422825" & year==2002

replace regioncode="422826" if regioncode=="422826" & year==2002

replace regioncode="422827" if regioncode=="422827" & year==2002

replace regioncode="422828" if regioncode=="422828" & year==2002

replace regioncode="429004" if regioncode=="429004" & year==2002

replace regioncode="429005" if regioncode=="429005" & year==2002

replace regioncode="429006" if regioncode=="429006" & year==2002

replace regioncode="429021" if regioncode=="429021" & year==2002

replace regioncode="430102" if regioncode=="430102" & year==2002

replace regioncode="430103" if regioncode=="430103" & year==2002

replace regioncode="430104" if regioncode=="430104" & year==2002

replace regioncode="430105" if regioncode=="430105" & year==2002

replace regioncode="430111" if regioncode=="430111" & year==2002

replace regioncode="430121" if regioncode=="430121" & year==2002

replace regioncode="430122" if regioncode=="430122" & year==2002

replace regioncode="430124" if regioncode=="430124" & year==2002

replace regioncode="430181" if regioncode=="430181" & year==2002

replace regioncode="430202" if regioncode=="430202" & year==2002

replace regioncode="430203" if regioncode=="430203" & year==2002

replace regioncode="430204" if regioncode=="430204" & year==2002

replace regioncode="430211" if regioncode=="430211" & year==2002

replace regioncode="430221" if regioncode=="430221" & year==2002

replace regioncode="430223" if regioncode=="430223" & year==2002

replace regioncode="430224" if regioncode=="430224" & year==2002

replace regioncode="430225" if regioncode=="430225" & year==2002

replace regioncode="430281" if regioncode=="430281" & year==2002

replace regioncode="430302" if regioncode=="430302" & year==2002

replace regioncode="430304" if regioncode=="430304" & year==2002

replace regioncode="430321" if regioncode=="430321" & year==2002

replace regioncode="430381" if regioncode=="430381" & year==2002

replace regioncode="430382" if regioncode=="430382" & year==2002

replace regioncode="430405" if regioncode=="430405" & year==2002

replace regioncode="430406" if regioncode=="430406" & year==2002

replace regioncode="430407" if regioncode=="430407" & year==2002

replace regioncode="430408" if regioncode=="430408" & year==2002

replace regioncode="430412" if regioncode=="430412" & year==2002

replace regioncode="430421" if regioncode=="430421" & year==2002

replace regioncode="430422" if regioncode=="430422" & year==2002

replace regioncode="430423" if regioncode=="430423" & year==2002

replace regioncode="430424" if regioncode=="430424" & year==2002

replace regioncode="430426" if regioncode=="430426" & year==2002

replace regioncode="430481" if regioncode=="430481" & year==2002

replace regioncode="430482" if regioncode=="430482" & year==2002

replace regioncode="430502" if regioncode=="430502" & year==2002

replace regioncode="430503" if regioncode=="430503" & year==2002

replace regioncode="430511" if regioncode=="430511" & year==2002

replace regioncode="430521" if regioncode=="430521" & year==2002

replace regioncode="430522" if regioncode=="430522" & year==2002

replace regioncode="430523" if regioncode=="430523" & year==2002

replace regioncode="430524" if regioncode=="430524" & year==2002

replace regioncode="430525" if regioncode=="430525" & year==2002

replace regioncode="430527" if regioncode=="430527" & year==2002

replace regioncode="430528" if regioncode=="430528" & year==2002

replace regioncode="430529" if regioncode=="430529" & year==2002

replace regioncode="430581" if regioncode=="430581" & year==2002

replace regioncode="430602" if regioncode=="430602" & year==2002

replace regioncode="430603" if regioncode=="430603" & year==2002

replace regioncode="430611" if regioncode=="430611" & year==2002

replace regioncode="430621" if regioncode=="430621" & year==2002

replace regioncode="430623" if regioncode=="430623" & year==2002

replace regioncode="430624" if regioncode=="430624" & year==2002

replace regioncode="430626" if regioncode=="430626" & year==2002

replace regioncode="430681" if regioncode=="430681" & year==2002

replace regioncode="430682" if regioncode=="430682" & year==2002

replace regioncode="430702" if regioncode=="430702" & year==2002

replace regioncode="430703" if regioncode=="430703" & year==2002

replace regioncode="430721" if regioncode=="430721" & year==2002

replace regioncode="430722" if regioncode=="430722" & year==2002

replace regioncode="430723" if regioncode=="430723" & year==2002

replace regioncode="430724" if regioncode=="430724" & year==2002

replace regioncode="430725" if regioncode=="430725" & year==2002

replace regioncode="430726" if regioncode=="430726" & year==2002

replace regioncode="430781" if regioncode=="430781" & year==2002

replace regioncode="430802" if regioncode=="430802" & year==2002

replace regioncode="430811" if regioncode=="430811" & year==2002

replace regioncode="430821" if regioncode=="430821" & year==2002

replace regioncode="430822" if regioncode=="430822" & year==2002

replace regioncode="430902" if regioncode=="430902" & year==2002

replace regioncode="430903" if regioncode=="430903" & year==2002

replace regioncode="430921" if regioncode=="430921" & year==2002

replace regioncode="430922" if regioncode=="430922" & year==2002

replace regioncode="430923" if regioncode=="430923" & year==2002

replace regioncode="430981" if regioncode=="430981" & year==2002

replace regioncode="431002" if regioncode=="431002" & year==2002

replace regioncode="431003" if regioncode=="431003" & year==2002

replace regioncode="431021" if regioncode=="431021" & year==2002

replace regioncode="431022" if regioncode=="431022" & year==2002

replace regioncode="431023" if regioncode=="431023" & year==2002

replace regioncode="431024" if regioncode=="431024" & year==2002

replace regioncode="431025" if regioncode=="431025" & year==2002

replace regioncode="431026" if regioncode=="431026" & year==2002

replace regioncode="431027" if regioncode=="431027" & year==2002

replace regioncode="431028" if regioncode=="431028" & year==2002

replace regioncode="431081" if regioncode=="431081" & year==2002

replace regioncode="431102" if regioncode=="431102" & year==2002

replace regioncode="431103" if regioncode=="431103" & year==2002

replace regioncode="431121" if regioncode=="431121" & year==2002

replace regioncode="431122" if regioncode=="431122" & year==2002

replace regioncode="431123" if regioncode=="431123" & year==2002

replace regioncode="431124" if regioncode=="431124" & year==2002

replace regioncode="431125" if regioncode=="431125" & year==2002

replace regioncode="431126" if regioncode=="431126" & year==2002

replace regioncode="431127" if regioncode=="431127" & year==2002

replace regioncode="431128" if regioncode=="431128" & year==2002

replace regioncode="431129" if regioncode=="431129" & year==2002

replace regioncode="431202" if regioncode=="431202" & year==2002

replace regioncode="431221" if regioncode=="431221" & year==2002

replace regioncode="431222" if regioncode=="431222" & year==2002

replace regioncode="431223" if regioncode=="431223" & year==2002

replace regioncode="431224" if regioncode=="431224" & year==2002

replace regioncode="431225" if regioncode=="431225" & year==2002

replace regioncode="431226" if regioncode=="431226" & year==2002

replace regioncode="431227" if regioncode=="431227" & year==2002

replace regioncode="431228" if regioncode=="431228" & year==2002

replace regioncode="431229" if regioncode=="431229" & year==2002

replace regioncode="431230" if regioncode=="431230" & year==2002

replace regioncode="431281" if regioncode=="431281" & year==2002

replace regioncode="431302" if regioncode=="431302" & year==2002

replace regioncode="431321" if regioncode=="431321" & year==2002

replace regioncode="431322" if regioncode=="431322" & year==2002

replace regioncode="431381" if regioncode=="431381" & year==2002

replace regioncode="431382" if regioncode=="431382" & year==2002

replace regioncode="433101" if regioncode=="433101" & year==2002

replace regioncode="433122" if regioncode=="433122" & year==2002

replace regioncode="433123" if regioncode=="433123" & year==2002

replace regioncode="433124" if regioncode=="433124" & year==2002

replace regioncode="433125" if regioncode=="433125" & year==2002

replace regioncode="433126" if regioncode=="433126" & year==2002

replace regioncode="433127" if regioncode=="433127" & year==2002

replace regioncode="433130" if regioncode=="433130" & year==2002

replace regioncode="440102" if regioncode=="440102" & year==2002

replace regioncode="440103" if regioncode=="440103" & year==2002

replace regioncode="440104" if regioncode=="440104" & year==2002

replace regioncode="440105" if regioncode=="440105" & year==2002

replace regioncode="440106" if regioncode=="440106" & year==2002

replace regioncode="440107" if regioncode=="440107" & year==2002

replace regioncode="440111" if regioncode=="440111" & year==2002

replace regioncode="440112" if regioncode=="440112" & year==2002

replace regioncode="440113" if regioncode=="440113" & year==2002

replace regioncode="440114" if regioncode=="440114" & year==2002

replace regioncode="440183" if regioncode=="440183" & year==2002

replace regioncode="440184" if regioncode=="440184" & year==2002

replace regioncode="440203" if regioncode=="440203" & year==2002

replace regioncode="440204" if regioncode=="440204" & year==2002

replace regioncode="440221" if regioncode=="440221" & year==2002

replace regioncode="440222" if regioncode=="440222" & year==2002

replace regioncode="440224" if regioncode=="440224" & year==2002

replace regioncode="440229" if regioncode=="440229" & year==2002

replace regioncode="440232" if regioncode=="440232" & year==2002

replace regioncode="440233" if regioncode=="440233" & year==2002

replace regioncode="440281" if regioncode=="440281" & year==2002

replace regioncode="440282" if regioncode=="440282" & year==2002

replace regioncode="440303" if regioncode=="440303" & year==2002

replace regioncode="440304" if regioncode=="440304" & year==2002

replace regioncode="440305" if regioncode=="440305" & year==2002

replace regioncode="440306" if regioncode=="440306" & year==2002

replace regioncode="440307" if regioncode=="440307" & year==2002

replace regioncode="440308" if regioncode=="440308" & year==2002

replace regioncode="440402" if regioncode=="440402" & year==2002

replace regioncode="440403" if regioncode=="440403" & year==2002

replace regioncode="440404" if regioncode=="440404" & year==2002

replace regioncode="440507" if regioncode=="440507" & year==2002

replace regioncode="440511" if regioncode=="440509" & year==2002

replace regioncode="440512" if regioncode=="440510" & year==2002

replace regioncode="440514" if regioncode=="440582" & year==2002

replace regioncode="440515" if regioncode=="440583" & year==2002

replace regioncode="440523" if regioncode=="440523" & year==2002

replace regioncode="440604" if regioncode=="440603" & year==2002

replace regioncode="440605" if regioncode=="440682" & year==2002

replace regioncode="440606" if regioncode=="440681" & year==2002

replace regioncode="440607" if regioncode=="440683" & year==2002

replace regioncode="440608" if regioncode=="440684" & year==2002

replace regioncode="440703" if regioncode=="440703" & year==2002

replace regioncode="440704" if regioncode=="440704" & year==2002

replace regioncode="440781" if regioncode=="440781" & year==2002

replace regioncode="440705" if regioncode=="440782" & year==2002

replace regioncode="440783" if regioncode=="440783" & year==2002

replace regioncode="440784" if regioncode=="440784" & year==2002

replace regioncode="440785" if regioncode=="440785" & year==2002

replace regioncode="440802" if regioncode=="440802" & year==2002

replace regioncode="440803" if regioncode=="440803" & year==2002

replace regioncode="440804" if regioncode=="440804" & year==2002

replace regioncode="440811" if regioncode=="440811" & year==2002

replace regioncode="440823" if regioncode=="440823" & year==2002

replace regioncode="440825" if regioncode=="440825" & year==2002

replace regioncode="440881" if regioncode=="440881" & year==2002

replace regioncode="440882" if regioncode=="440882" & year==2002

replace regioncode="440883" if regioncode=="440883" & year==2002

replace regioncode="440902" if regioncode=="440902" & year==2002

replace regioncode="440903" if regioncode=="440903" & year==2002

replace regioncode="440923" if regioncode=="440923" & year==2002

replace regioncode="440981" if regioncode=="440981" & year==2002

replace regioncode="440982" if regioncode=="440982" & year==2002

replace regioncode="440983" if regioncode=="440983" & year==2002

replace regioncode="441202" if regioncode=="441202" & year==2002

replace regioncode="441203" if regioncode=="441203" & year==2002

replace regioncode="441223" if regioncode=="441223" & year==2002

replace regioncode="441224" if regioncode=="441224" & year==2002

replace regioncode="441225" if regioncode=="441225" & year==2002

replace regioncode="441226" if regioncode=="441226" & year==2002

replace regioncode="441283" if regioncode=="441283" & year==2002

replace regioncode="441284" if regioncode=="441284" & year==2002

replace regioncode="441302" if regioncode=="441302" & year==2002

replace regioncode="441303" if regioncode=="441381" & year==2002

replace regioncode="441322" if regioncode=="441322" & year==2002

replace regioncode="441323" if regioncode=="441323" & year==2002

replace regioncode="441324" if regioncode=="441324" & year==2002

replace regioncode="441402" if regioncode=="441402" & year==2002

replace regioncode="441421" if regioncode=="441421" & year==2002

replace regioncode="441422" if regioncode=="441422" & year==2002

replace regioncode="441423" if regioncode=="441423" & year==2002

replace regioncode="441424" if regioncode=="441424" & year==2002

replace regioncode="441426" if regioncode=="441426" & year==2002

replace regioncode="441427" if regioncode=="441427" & year==2002

replace regioncode="441481" if regioncode=="441481" & year==2002

replace regioncode="441502" if regioncode=="441502" & year==2002

replace regioncode="441521" if regioncode=="441521" & year==2002

replace regioncode="441523" if regioncode=="441523" & year==2002

replace regioncode="441581" if regioncode=="441581" & year==2002

replace regioncode="441602" if regioncode=="441602" & year==2002

replace regioncode="441621" if regioncode=="441621" & year==2002

replace regioncode="441622" if regioncode=="441622" & year==2002

replace regioncode="441623" if regioncode=="441623" & year==2002

replace regioncode="441624" if regioncode=="441624" & year==2002

replace regioncode="441625" if regioncode=="441625" & year==2002

replace regioncode="441702" if regioncode=="441702" & year==2002

replace regioncode="441721" if regioncode=="441721" & year==2002

replace regioncode="441723" if regioncode=="441723" & year==2002

replace regioncode="441781" if regioncode=="441781" & year==2002

replace regioncode="441802" if regioncode=="441802" & year==2002

replace regioncode="441821" if regioncode=="441821" & year==2002

replace regioncode="441823" if regioncode=="441823" & year==2002

replace regioncode="441825" if regioncode=="441825" & year==2002

replace regioncode="441826" if regioncode=="441826" & year==2002

replace regioncode="441827" if regioncode=="441827" & year==2002

replace regioncode="441881" if regioncode=="441881" & year==2002

replace regioncode="441882" if regioncode=="441882" & year==2002

replace regioncode="441900" if regioncode=="441900" & year==2002

replace regioncode="442000" if regioncode=="442000" & year==2002

replace regioncode="445102" if regioncode=="445102" & year==2002

replace regioncode="445121" if regioncode=="445121" & year==2002

replace regioncode="445122" if regioncode=="445122" & year==2002

replace regioncode="445202" if regioncode=="445202" & year==2002

replace regioncode="445221" if regioncode=="445221" & year==2002

replace regioncode="445222" if regioncode=="445222" & year==2002

replace regioncode="445224" if regioncode=="445224" & year==2002

replace regioncode="445281" if regioncode=="445281" & year==2002

replace regioncode="445302" if regioncode=="445302" & year==2002

replace regioncode="445321" if regioncode=="445321" & year==2002

replace regioncode="445322" if regioncode=="445322" & year==2002

replace regioncode="445323" if regioncode=="445323" & year==2002

replace regioncode="445381" if regioncode=="445381" & year==2002

replace regioncode="450102" if regioncode=="450102" & year==2002

replace regioncode="450103" if regioncode=="450103" & year==2002

replace regioncode="450104" if regioncode=="450104" & year==2002

replace regioncode="450105" if regioncode=="450105" & year==2002

replace regioncode="450106" if regioncode=="450106" & year==2002

replace regioncode="450121" if regioncode=="450121" & year==2002

replace regioncode="450122" if regioncode=="450122" & year==2002

replace regioncode="450123" if regioncode=="450123" & year==2002

replace regioncode="450124" if regioncode=="450124" & year==2002

replace regioncode="450125" if regioncode=="450125" & year==2002

replace regioncode="450126" if regioncode=="450126" & year==2002

replace regioncode="450127" if regioncode=="450127" & year==2002

replace regioncode="450202" if regioncode=="450202" & year==2002

replace regioncode="450203" if regioncode=="450203" & year==2002

replace regioncode="450204" if regioncode=="450204" & year==2002

replace regioncode="450205" if regioncode=="450205" & year==2002

replace regioncode="450221" if regioncode=="450221" & year==2002

replace regioncode="450222" if regioncode=="450222" & year==2002

replace regioncode="450223" if regioncode=="450223" & year==2002

replace regioncode="450224" if regioncode=="450224" & year==2002

replace regioncode="450225" if regioncode=="450225" & year==2002

replace regioncode="450226" if regioncode=="450226" & year==2002

replace regioncode="450302" if regioncode=="450302" & year==2002

replace regioncode="450303" if regioncode=="450303" & year==2002

replace regioncode="450304" if regioncode=="450304" & year==2002

replace regioncode="450305" if regioncode=="450305" & year==2002

replace regioncode="450311" if regioncode=="450311" & year==2002

replace regioncode="450321" if regioncode=="450321" & year==2002

replace regioncode="450322" if regioncode=="450322" & year==2002

replace regioncode="450323" if regioncode=="450323" & year==2002

replace regioncode="450324" if regioncode=="450324" & year==2002

replace regioncode="450325" if regioncode=="450325" & year==2002

replace regioncode="450326" if regioncode=="450326" & year==2002

replace regioncode="450327" if regioncode=="450327" & year==2002

replace regioncode="450328" if regioncode=="450328" & year==2002

replace regioncode="450329" if regioncode=="450329" & year==2002

replace regioncode="450330" if regioncode=="450330" & year==2002

replace regioncode="450331" if regioncode=="450331" & year==2002

replace regioncode="450332" if regioncode=="450332" & year==2002

replace regioncode="450403" if regioncode=="450403" & year==2002

replace regioncode="450404" if regioncode=="450404" & year==2002

replace regioncode="450421" if regioncode=="450421" & year==2002

replace regioncode="450422" if regioncode=="450422" & year==2002

replace regioncode="450423" if regioncode=="450423" & year==2002

replace regioncode="450481" if regioncode=="450481" & year==2002

replace regioncode="450502" if regioncode=="450502" & year==2002

replace regioncode="450503" if regioncode=="450503" & year==2002

replace regioncode="450512" if regioncode=="450512" & year==2002

replace regioncode="450521" if regioncode=="450521" & year==2002

replace regioncode="450602" if regioncode=="450602" & year==2002

replace regioncode="450603" if regioncode=="450603" & year==2002

replace regioncode="450621" if regioncode=="450621" & year==2002

replace regioncode="450681" if regioncode=="450681" & year==2002

replace regioncode="450702" if regioncode=="450702" & year==2002

replace regioncode="450703" if regioncode=="450703" & year==2002

replace regioncode="450721" if regioncode=="450721" & year==2002

replace regioncode="450722" if regioncode=="450722" & year==2002

replace regioncode="450802" if regioncode=="450802" & year==2002

replace regioncode="450803" if regioncode=="450803" & year==2002

replace regioncode="450804" if regioncode=="450804" & year==2002

replace regioncode="450821" if regioncode=="450821" & year==2002

replace regioncode="450881" if regioncode=="450881" & year==2002

replace regioncode="450902" if regioncode=="450902" & year==2002

replace regioncode="450921" if regioncode=="450921" & year==2002

replace regioncode="450922" if regioncode=="450922" & year==2002

replace regioncode="450923" if regioncode=="450923" & year==2002

replace regioncode="450924" if regioncode=="450924" & year==2002

replace regioncode="450981" if regioncode=="450981" & year==2002

replace regioncode="451002" if regioncode=="451002" & year==2002

replace regioncode="451021" if regioncode=="451021" & year==2002

replace regioncode="451022" if regioncode=="451022" & year==2002

replace regioncode="451023" if regioncode=="451023" & year==2002

replace regioncode="451024" if regioncode=="451024" & year==2002

replace regioncode="451025" if regioncode=="451025" & year==2002

replace regioncode="451026" if regioncode=="451026" & year==2002

replace regioncode="451027" if regioncode=="451027" & year==2002

replace regioncode="451028" if regioncode=="451028" & year==2002

replace regioncode="451029" if regioncode=="451029" & year==2002

replace regioncode="451030" if regioncode=="451030" & year==2002

replace regioncode="451031" if regioncode=="451031" & year==2002

replace regioncode="451102" if regioncode=="451102" & year==2002

replace regioncode="451121" if regioncode=="451121" & year==2002

replace regioncode="451122" if regioncode=="451122" & year==2002

replace regioncode="451123" if regioncode=="451123" & year==2002

replace regioncode="451202" if regioncode=="451202" & year==2002

replace regioncode="451221" if regioncode=="451221" & year==2002

replace regioncode="451222" if regioncode=="451222" & year==2002

replace regioncode="451223" if regioncode=="451223" & year==2002

replace regioncode="451224" if regioncode=="451224" & year==2002

replace regioncode="451225" if regioncode=="451225" & year==2002

replace regioncode="451226" if regioncode=="451226" & year==2002

replace regioncode="451227" if regioncode=="451227" & year==2002

replace regioncode="451228" if regioncode=="451228" & year==2002

replace regioncode="451229" if regioncode=="451229" & year==2002

replace regioncode="451281" if regioncode=="451281" & year==2002

replace regioncode="451302" if regioncode=="451302" & year==2002

replace regioncode="451321" if regioncode=="451321" & year==2002

replace regioncode="451322" if regioncode=="451322" & year==2002

replace regioncode="451323" if regioncode=="451323" & year==2002

replace regioncode="451324" if regioncode=="451324" & year==2002

replace regioncode="451381" if regioncode=="451381" & year==2002

replace regioncode="451402" if regioncode=="451401" & year==2002

replace regioncode="451421" if regioncode=="451421" & year==2002

replace regioncode="451422" if regioncode=="451422" & year==2002

replace regioncode="451423" if regioncode=="451423" & year==2002

replace regioncode="451424" if regioncode=="451424" & year==2002

replace regioncode="451425" if regioncode=="451425" & year==2002

replace regioncode="451481" if regioncode=="451481" & year==2002

replace regioncode="460105" if regioncode=="460105" & year==2002

replace regioncode="460106" if regioncode=="460106" & year==2002

replace regioncode="460107" if regioncode=="460104" & year==2002

replace regioncode="460108" if regioncode=="460108" & year==2002

replace regioncode="460001" if regioncode=="460001" & year==2002

replace regioncode="460002" if regioncode=="460002" & year==2002

replace regioncode="460003" if regioncode=="460003" & year==2002

replace regioncode="460005" if regioncode=="460005" & year==2002

replace regioncode="460006" if regioncode=="460006" & year==2002

replace regioncode="460007" if regioncode=="460007" & year==2002

replace regioncode="460030" if regioncode=="460030" & year==2002

replace regioncode="460031" if regioncode=="460031" & year==2002

replace regioncode="460033" if regioncode=="460033" & year==2002

replace regioncode="460034" if regioncode=="460034" & year==2002

replace regioncode="460036" if regioncode=="460036" & year==2002

replace regioncode="500101" if regioncode=="500101" & year==2002

replace regioncode="500102" if regioncode=="500102" & year==2002

replace regioncode="500103" if regioncode=="500103" & year==2002

replace regioncode="500104" if regioncode=="500104" & year==2002

replace regioncode="500105" if regioncode=="500105" & year==2002

replace regioncode="500106" if regioncode=="500106" & year==2002

replace regioncode="500107" if regioncode=="500107" & year==2002

replace regioncode="500108" if regioncode=="500108" & year==2002

replace regioncode="500109" if regioncode=="500109" & year==2002

replace regioncode="500110" if regioncode=="500110" & year==2002

replace regioncode="500111" if regioncode=="500111" & year==2002

replace regioncode="500112" if regioncode=="500112" & year==2002

replace regioncode="500113" if regioncode=="500113" & year==2002

replace regioncode="500114" if regioncode=="500114" & year==2002

replace regioncode="500115" if regioncode=="500115" & year==2002

replace regioncode="500381" if regioncode=="500381" & year==2002

replace regioncode="500382" if regioncode=="500382" & year==2002

replace regioncode="500383" if regioncode=="500383" & year==2002

replace regioncode="500384" if regioncode=="500384" & year==2002

replace regioncode="500222" if regioncode=="500222" & year==2002

replace regioncode="500223" if regioncode=="500223" & year==2002

replace regioncode="500224" if regioncode=="500224" & year==2002

replace regioncode="500225" if regioncode=="500225" & year==2002

replace regioncode="500226" if regioncode=="500226" & year==2002

replace regioncode="500227" if regioncode=="500227" & year==2002

replace regioncode="500228" if regioncode=="500228" & year==2002

replace regioncode="500229" if regioncode=="500229" & year==2002

replace regioncode="500230" if regioncode=="500230" & year==2002

replace regioncode="500231" if regioncode=="500231" & year==2002

replace regioncode="500232" if regioncode=="500232" & year==2002

replace regioncode="500233" if regioncode=="500233" & year==2002

replace regioncode="500234" if regioncode=="500234" & year==2002

replace regioncode="500235" if regioncode=="500235" & year==2002

replace regioncode="500236" if regioncode=="500236" & year==2002

replace regioncode="500237" if regioncode=="500237" & year==2002

replace regioncode="500238" if regioncode=="500238" & year==2002

replace regioncode="500240" if regioncode=="500240" & year==2002

replace regioncode="500241" if regioncode=="500241" & year==2002

replace regioncode="500242" if regioncode=="500242" & year==2002

replace regioncode="500243" if regioncode=="500243" & year==2002

replace regioncode="510104" if regioncode=="510104" & year==2002

replace regioncode="510105" if regioncode=="510105" & year==2002

replace regioncode="510106" if regioncode=="510106" & year==2002

replace regioncode="510107" if regioncode=="510107" & year==2002

replace regioncode="510108" if regioncode=="510108" & year==2002

replace regioncode="510112" if regioncode=="510112" & year==2002

replace regioncode="510113" if regioncode=="510113" & year==2002

replace regioncode="510114" if regioncode=="510114" & year==2002

replace regioncode="510115" if regioncode=="510123" & year==2002

replace regioncode="510121" if regioncode=="510121" & year==2002

replace regioncode="510122" if regioncode=="510122" & year==2002

replace regioncode="510124" if regioncode=="510124" & year==2002

replace regioncode="510129" if regioncode=="510129" & year==2002

replace regioncode="510131" if regioncode=="510131" & year==2002

replace regioncode="510132" if regioncode=="510132" & year==2002

replace regioncode="510181" if regioncode=="510181" & year==2002

replace regioncode="510182" if regioncode=="510182" & year==2002

replace regioncode="510183" if regioncode=="510183" & year==2002

replace regioncode="510184" if regioncode=="510184" & year==2002

replace regioncode="510302" if regioncode=="510302" & year==2002

replace regioncode="510303" if regioncode=="510303" & year==2002

replace regioncode="510304" if regioncode=="510304" & year==2002

replace regioncode="510311" if regioncode=="510311" & year==2002

replace regioncode="510321" if regioncode=="510321" & year==2002

replace regioncode="510322" if regioncode=="510322" & year==2002

replace regioncode="510402" if regioncode=="510402" & year==2002

replace regioncode="510403" if regioncode=="510403" & year==2002

replace regioncode="510411" if regioncode=="510411" & year==2002

replace regioncode="510421" if regioncode=="510421" & year==2002

replace regioncode="510422" if regioncode=="510422" & year==2002

replace regioncode="510502" if regioncode=="510502" & year==2002

replace regioncode="510503" if regioncode=="510503" & year==2002

replace regioncode="510504" if regioncode=="510504" & year==2002

replace regioncode="510521" if regioncode=="510521" & year==2002

replace regioncode="510522" if regioncode=="510522" & year==2002

replace regioncode="510524" if regioncode=="510524" & year==2002

replace regioncode="510525" if regioncode=="510525" & year==2002

replace regioncode="510603" if regioncode=="510603" & year==2002

replace regioncode="510623" if regioncode=="510623" & year==2002

replace regioncode="510626" if regioncode=="510626" & year==2002

replace regioncode="510681" if regioncode=="510681" & year==2002

replace regioncode="510682" if regioncode=="510682" & year==2002

replace regioncode="510683" if regioncode=="510683" & year==2002

replace regioncode="510703" if regioncode=="510703" & year==2002

replace regioncode="510704" if regioncode=="510704" & year==2002

replace regioncode="510722" if regioncode=="510722" & year==2002

replace regioncode="510723" if regioncode=="510723" & year==2002

replace regioncode="510724" if regioncode=="510724" & year==2002

replace regioncode="510725" if regioncode=="510725" & year==2002

replace regioncode="510726" if regioncode=="510726" & year==2002

replace regioncode="510727" if regioncode=="510727" & year==2002

replace regioncode="510781" if regioncode=="510781" & year==2002

replace regioncode="510802" if regioncode=="510802" & year==2002

replace regioncode="510811" if regioncode=="510811" & year==2002

replace regioncode="510812" if regioncode=="510812" & year==2002

replace regioncode="510821" if regioncode=="510821" & year==2002

replace regioncode="510822" if regioncode=="510822" & year==2002

replace regioncode="510823" if regioncode=="510823" & year==2002

replace regioncode="510824" if regioncode=="510824" & year==2002

replace regioncode="510902" if regioncode=="510902" & year==2002

replace regioncode="510921" if regioncode=="510921" & year==2002

replace regioncode="510922" if regioncode=="510922" & year==2002

replace regioncode="510923" if regioncode=="510923" & year==2002

replace regioncode="511002" if regioncode=="511002" & year==2002

replace regioncode="511011" if regioncode=="511011" & year==2002

replace regioncode="511024" if regioncode=="511024" & year==2002

replace regioncode="511025" if regioncode=="511025" & year==2002

replace regioncode="511028" if regioncode=="511028" & year==2002

replace regioncode="511102" if regioncode=="511102" & year==2002

replace regioncode="511111" if regioncode=="511111" & year==2002

replace regioncode="511112" if regioncode=="511112" & year==2002

replace regioncode="511113" if regioncode=="511113" & year==2002

replace regioncode="511123" if regioncode=="511123" & year==2002

replace regioncode="511124" if regioncode=="511124" & year==2002

replace regioncode="511126" if regioncode=="511126" & year==2002

replace regioncode="511129" if regioncode=="511129" & year==2002

replace regioncode="511132" if regioncode=="511132" & year==2002

replace regioncode="511133" if regioncode=="511133" & year==2002

replace regioncode="511181" if regioncode=="511181" & year==2002

replace regioncode="511302" if regioncode=="511302" & year==2002

replace regioncode="511303" if regioncode=="511303" & year==2002

replace regioncode="511304" if regioncode=="511304" & year==2002

replace regioncode="511321" if regioncode=="511321" & year==2002

replace regioncode="511322" if regioncode=="511322" & year==2002

replace regioncode="511323" if regioncode=="511323" & year==2002

replace regioncode="511324" if regioncode=="511324" & year==2002

replace regioncode="511325" if regioncode=="511325" & year==2002

replace regioncode="511381" if regioncode=="511381" & year==2002

replace regioncode="511402" if regioncode=="511402" & year==2002

replace regioncode="511421" if regioncode=="511421" & year==2002

replace regioncode="511422" if regioncode=="511422" & year==2002

replace regioncode="511423" if regioncode=="511423" & year==2002

replace regioncode="511424" if regioncode=="511424" & year==2002

replace regioncode="511425" if regioncode=="511425" & year==2002

replace regioncode="511502" if regioncode=="511502" & year==2002

replace regioncode="511521" if regioncode=="511521" & year==2002

replace regioncode="511522" if regioncode=="511522" & year==2002

replace regioncode="511523" if regioncode=="511523" & year==2002

replace regioncode="511524" if regioncode=="511524" & year==2002

replace regioncode="511525" if regioncode=="511525" & year==2002

replace regioncode="511526" if regioncode=="511526" & year==2002

replace regioncode="511527" if regioncode=="511527" & year==2002

replace regioncode="511528" if regioncode=="511528" & year==2002

replace regioncode="511529" if regioncode=="511529" & year==2002

replace regioncode="511602" if regioncode=="511602" & year==2002

replace regioncode="511621" if regioncode=="511621" & year==2002

replace regioncode="511622" if regioncode=="511622" & year==2002

replace regioncode="511623" if regioncode=="511623" & year==2002

replace regioncode="511681" if regioncode=="511681" & year==2002

replace regioncode="511702" if regioncode=="511702" & year==2002

replace regioncode="511721" if regioncode=="511721" & year==2002

replace regioncode="511722" if regioncode=="511722" & year==2002

replace regioncode="511723" if regioncode=="511723" & year==2002

replace regioncode="511724" if regioncode=="511724" & year==2002

replace regioncode="511725" if regioncode=="511725" & year==2002

replace regioncode="511781" if regioncode=="511781" & year==2002

replace regioncode="511802" if regioncode=="511802" & year==2002

replace regioncode="511821" if regioncode=="511821" & year==2002

replace regioncode="511822" if regioncode=="511822" & year==2002

replace regioncode="511823" if regioncode=="511823" & year==2002

replace regioncode="511824" if regioncode=="511824" & year==2002

replace regioncode="511825" if regioncode=="511825" & year==2002

replace regioncode="511826" if regioncode=="511826" & year==2002

replace regioncode="511827" if regioncode=="511827" & year==2002

replace regioncode="511902" if regioncode=="511902" & year==2002

replace regioncode="511921" if regioncode=="511921" & year==2002

replace regioncode="511922" if regioncode=="511922" & year==2002

replace regioncode="511923" if regioncode=="511923" & year==2002

replace regioncode="512002" if regioncode=="512002" & year==2002

replace regioncode="512021" if regioncode=="512021" & year==2002

replace regioncode="512022" if regioncode=="512022" & year==2002

replace regioncode="512081" if regioncode=="512081" & year==2002

replace regioncode="513221" if regioncode=="513221" & year==2002

replace regioncode="513222" if regioncode=="513222" & year==2002

replace regioncode="513223" if regioncode=="513223" & year==2002

replace regioncode="513224" if regioncode=="513224" & year==2002

replace regioncode="513225" if regioncode=="513225" & year==2002

replace regioncode="513226" if regioncode=="513226" & year==2002

replace regioncode="513227" if regioncode=="513227" & year==2002

replace regioncode="513228" if regioncode=="513228" & year==2002

replace regioncode="513229" if regioncode=="513229" & year==2002

replace regioncode="513230" if regioncode=="513230" & year==2002

replace regioncode="513231" if regioncode=="513231" & year==2002

replace regioncode="513232" if regioncode=="513232" & year==2002

replace regioncode="513233" if regioncode=="513233" & year==2002

replace regioncode="513321" if regioncode=="513321" & year==2002

replace regioncode="513322" if regioncode=="513322" & year==2002

replace regioncode="513323" if regioncode=="513323" & year==2002

replace regioncode="513324" if regioncode=="513324" & year==2002

replace regioncode="513325" if regioncode=="513325" & year==2002

replace regioncode="513326" if regioncode=="513326" & year==2002

replace regioncode="513327" if regioncode=="513327" & year==2002

replace regioncode="513328" if regioncode=="513328" & year==2002

replace regioncode="513329" if regioncode=="513329" & year==2002

replace regioncode="513330" if regioncode=="513330" & year==2002

replace regioncode="513331" if regioncode=="513331" & year==2002

replace regioncode="513332" if regioncode=="513332" & year==2002

replace regioncode="513333" if regioncode=="513333" & year==2002

replace regioncode="513334" if regioncode=="513334" & year==2002

replace regioncode="513335" if regioncode=="513335" & year==2002

replace regioncode="513336" if regioncode=="513336" & year==2002

replace regioncode="513337" if regioncode=="513337" & year==2002

replace regioncode="513338" if regioncode=="513338" & year==2002

replace regioncode="513401" if regioncode=="513401" & year==2002

replace regioncode="513422" if regioncode=="513422" & year==2002

replace regioncode="513423" if regioncode=="513423" & year==2002

replace regioncode="513424" if regioncode=="513424" & year==2002

replace regioncode="513425" if regioncode=="513425" & year==2002

replace regioncode="513426" if regioncode=="513426" & year==2002

replace regioncode="513427" if regioncode=="513427" & year==2002

replace regioncode="513428" if regioncode=="513428" & year==2002

replace regioncode="513429" if regioncode=="513429" & year==2002

replace regioncode="513430" if regioncode=="513430" & year==2002

replace regioncode="513431" if regioncode=="513431" & year==2002

replace regioncode="513432" if regioncode=="513432" & year==2002

replace regioncode="513433" if regioncode=="513433" & year==2002

replace regioncode="513434" if regioncode=="513434" & year==2002

replace regioncode="513435" if regioncode=="513435" & year==2002

replace regioncode="513436" if regioncode=="513436" & year==2002

replace regioncode="513437" if regioncode=="513437" & year==2002

replace regioncode="520102" if regioncode=="520102" & year==2002

replace regioncode="520103" if regioncode=="520103" & year==2002

replace regioncode="520111" if regioncode=="520111" & year==2002

replace regioncode="520112" if regioncode=="520112" & year==2002

replace regioncode="520113" if regioncode=="520113" & year==2002

replace regioncode="520114" if regioncode=="520114" & year==2002

replace regioncode="520121" if regioncode=="520121" & year==2002

replace regioncode="520122" if regioncode=="520122" & year==2002

replace regioncode="520123" if regioncode=="520123" & year==2002

replace regioncode="520181" if regioncode=="520181" & year==2002

replace regioncode="520201" if regioncode=="520201" & year==2002

replace regioncode="520222" if regioncode=="520222" & year==2002

replace regioncode="520203" if regioncode=="520203" & year==2002

replace regioncode="520221" if regioncode=="520221" & year==2002

replace regioncode="520302" if regioncode=="520302" & year==2002

replace regioncode="520321" if regioncode=="520321" & year==2002

replace regioncode="520322" if regioncode=="520322" & year==2002

replace regioncode="520323" if regioncode=="520323" & year==2002

replace regioncode="520324" if regioncode=="520324" & year==2002

replace regioncode="520325" if regioncode=="520325" & year==2002

replace regioncode="520326" if regioncode=="520326" & year==2002

replace regioncode="520327" if regioncode=="520327" & year==2002

replace regioncode="520328" if regioncode=="520328" & year==2002

replace regioncode="520329" if regioncode=="520329" & year==2002

replace regioncode="520330" if regioncode=="520330" & year==2002

replace regioncode="520381" if regioncode=="520381" & year==2002

replace regioncode="520382" if regioncode=="520382" & year==2002

replace regioncode="520402" if regioncode=="520402" & year==2002

replace regioncode="520421" if regioncode=="520421" & year==2002

replace regioncode="520422" if regioncode=="520422" & year==2002

replace regioncode="520423" if regioncode=="520423" & year==2002

replace regioncode="520424" if regioncode=="520424" & year==2002

replace regioncode="520425" if regioncode=="520425" & year==2002

replace regioncode="522201" if regioncode=="522201" & year==2002

replace regioncode="522222" if regioncode=="522222" & year==2002

replace regioncode="522223" if regioncode=="522223" & year==2002

replace regioncode="522224" if regioncode=="522224" & year==2002

replace regioncode="522225" if regioncode=="522225" & year==2002

replace regioncode="522226" if regioncode=="522226" & year==2002

replace regioncode="522227" if regioncode=="522227" & year==2002

replace regioncode="522228" if regioncode=="522228" & year==2002

replace regioncode="522229" if regioncode=="522229" & year==2002

replace regioncode="522230" if regioncode=="522230" & year==2002

replace regioncode="522301" if regioncode=="522301" & year==2002

replace regioncode="522322" if regioncode=="522322" & year==2002

replace regioncode="522323" if regioncode=="522323" & year==2002

replace regioncode="522324" if regioncode=="522324" & year==2002

replace regioncode="522325" if regioncode=="522325" & year==2002

replace regioncode="522326" if regioncode=="522326" & year==2002

replace regioncode="522327" if regioncode=="522327" & year==2002

replace regioncode="522328" if regioncode=="522328" & year==2002

replace regioncode="522401" if regioncode=="522401" & year==2002

replace regioncode="522422" if regioncode=="522422" & year==2002

replace regioncode="522423" if regioncode=="522423" & year==2002

replace regioncode="522424" if regioncode=="522424" & year==2002

replace regioncode="522425" if regioncode=="522425" & year==2002

replace regioncode="522426" if regioncode=="522426" & year==2002

replace regioncode="522427" if regioncode=="522427" & year==2002

replace regioncode="522428" if regioncode=="522428" & year==2002

replace regioncode="522601" if regioncode=="522601" & year==2002

replace regioncode="522622" if regioncode=="522622" & year==2002

replace regioncode="522623" if regioncode=="522623" & year==2002

replace regioncode="522624" if regioncode=="522624" & year==2002

replace regioncode="522625" if regioncode=="522625" & year==2002

replace regioncode="522626" if regioncode=="522626" & year==2002

replace regioncode="522627" if regioncode=="522627" & year==2002

replace regioncode="522628" if regioncode=="522628" & year==2002

replace regioncode="522629" if regioncode=="522629" & year==2002

replace regioncode="522630" if regioncode=="522630" & year==2002

replace regioncode="522631" if regioncode=="522631" & year==2002

replace regioncode="522632" if regioncode=="522632" & year==2002

replace regioncode="522633" if regioncode=="522633" & year==2002

replace regioncode="522634" if regioncode=="522634" & year==2002

replace regioncode="522635" if regioncode=="522635" & year==2002

replace regioncode="522636" if regioncode=="522636" & year==2002

replace regioncode="522701" if regioncode=="522701" & year==2002

replace regioncode="522702" if regioncode=="522702" & year==2002

replace regioncode="522722" if regioncode=="522722" & year==2002

replace regioncode="522723" if regioncode=="522723" & year==2002

replace regioncode="522725" if regioncode=="522725" & year==2002

replace regioncode="522726" if regioncode=="522726" & year==2002

replace regioncode="522727" if regioncode=="522727" & year==2002

replace regioncode="522728" if regioncode=="522728" & year==2002

replace regioncode="522729" if regioncode=="522729" & year==2002

replace regioncode="522730" if regioncode=="522730" & year==2002

replace regioncode="522731" if regioncode=="522731" & year==2002

replace regioncode="522732" if regioncode=="522732" & year==2002

replace regioncode="530102" if regioncode=="530102" & year==2002

replace regioncode="530103" if regioncode=="530103" & year==2002

replace regioncode="530111" if regioncode=="530111" & year==2002

replace regioncode="530112" if regioncode=="530112" & year==2002

replace regioncode="530113" if regioncode=="530113" & year==2002

replace regioncode="530121" if regioncode=="530121" & year==2002

replace regioncode="530122" if regioncode=="530122" & year==2002

replace regioncode="530124" if regioncode=="530124" & year==2002

replace regioncode="530125" if regioncode=="530125" & year==2002

replace regioncode="530126" if regioncode=="530126" & year==2002

replace regioncode="530127" if regioncode=="530127" & year==2002

replace regioncode="530128" if regioncode=="530128" & year==2002

replace regioncode="530129" if regioncode=="530129" & year==2002

replace regioncode="530181" if regioncode=="530181" & year==2002

replace regioncode="530302" if regioncode=="530302" & year==2002

replace regioncode="530321" if regioncode=="530321" & year==2002

replace regioncode="530322" if regioncode=="530322" & year==2002

replace regioncode="530323" if regioncode=="530323" & year==2002

replace regioncode="530324" if regioncode=="530324" & year==2002

replace regioncode="530325" if regioncode=="530325" & year==2002

replace regioncode="530326" if regioncode=="530326" & year==2002

replace regioncode="530328" if regioncode=="530328" & year==2002

replace regioncode="530381" if regioncode=="530381" & year==2002

replace regioncode="530402" if regioncode=="530402" & year==2002

replace regioncode="530421" if regioncode=="530421" & year==2002

replace regioncode="530422" if regioncode=="530422" & year==2002

replace regioncode="530423" if regioncode=="530423" & year==2002

replace regioncode="530424" if regioncode=="530424" & year==2002

replace regioncode="530425" if regioncode=="530425" & year==2002

replace regioncode="530426" if regioncode=="530426" & year==2002

replace regioncode="530427" if regioncode=="530427" & year==2002

replace regioncode="530428" if regioncode=="530428" & year==2002

replace regioncode="530502" if regioncode=="530502" & year==2002

replace regioncode="530521" if regioncode=="530521" & year==2002

replace regioncode="530522" if regioncode=="530522" & year==2002

replace regioncode="530523" if regioncode=="530523" & year==2002

replace regioncode="530524" if regioncode=="530524" & year==2002

replace regioncode="530602" if regioncode=="530602" & year==2002

replace regioncode="530621" if regioncode=="530621" & year==2002

replace regioncode="530622" if regioncode=="530622" & year==2002

replace regioncode="530623" if regioncode=="530623" & year==2002

replace regioncode="530624" if regioncode=="530624" & year==2002

replace regioncode="530625" if regioncode=="530625" & year==2002

replace regioncode="530626" if regioncode=="530626" & year==2002

replace regioncode="530627" if regioncode=="530627" & year==2002

replace regioncode="530628" if regioncode=="530628" & year==2002

replace regioncode="530629" if regioncode=="530629" & year==2002

replace regioncode="530630" if regioncode=="530630" & year==2002

replace regioncode="530721" if regioncode=="533221" & year==2002

replace regioncode="530722" if regioncode=="533222" & year==2002

replace regioncode="530723" if regioncode=="533223" & year==2002

replace regioncode="530724" if regioncode=="533224" & year==2002

replace regioncode="532701" if regioncode=="532701" & year==2002

replace regioncode="532722" if regioncode=="532722" & year==2002

replace regioncode="532723" if regioncode=="532723" & year==2002

replace regioncode="532724" if regioncode=="532724" & year==2002

replace regioncode="532725" if regioncode=="532725" & year==2002

replace regioncode="532726" if regioncode=="532726" & year==2002

replace regioncode="532727" if regioncode=="532727" & year==2002

replace regioncode="532728" if regioncode=="532728" & year==2002

replace regioncode="532729" if regioncode=="532729" & year==2002

replace regioncode="532730" if regioncode=="532730" & year==2002

replace regioncode="533521" if regioncode=="533521" & year==2002

replace regioncode="533522" if regioncode=="533522" & year==2002

replace regioncode="533523" if regioncode=="533523" & year==2002

replace regioncode="533524" if regioncode=="533524" & year==2002

replace regioncode="533525" if regioncode=="533525" & year==2002

replace regioncode="533526" if regioncode=="533526" & year==2002

replace regioncode="533527" if regioncode=="533527" & year==2002

replace regioncode="533528" if regioncode=="533528" & year==2002

replace regioncode="532301" if regioncode=="532301" & year==2002

replace regioncode="532322" if regioncode=="532322" & year==2002

replace regioncode="532323" if regioncode=="532323" & year==2002

replace regioncode="532324" if regioncode=="532324" & year==2002

replace regioncode="532325" if regioncode=="532325" & year==2002

replace regioncode="532326" if regioncode=="532326" & year==2002

replace regioncode="532327" if regioncode=="532327" & year==2002

replace regioncode="532328" if regioncode=="532328" & year==2002

replace regioncode="532329" if regioncode=="532329" & year==2002

replace regioncode="532331" if regioncode=="532331" & year==2002

replace regioncode="532501" if regioncode=="532501" & year==2002

replace regioncode="532502" if regioncode=="532502" & year==2002

replace regioncode="532522" if regioncode=="532522" & year==2002

replace regioncode="532523" if regioncode=="532523" & year==2002

replace regioncode="532524" if regioncode=="532524" & year==2002

replace regioncode="532525" if regioncode=="532525" & year==2002

replace regioncode="532526" if regioncode=="532526" & year==2002

replace regioncode="532527" if regioncode=="532527" & year==2002

replace regioncode="532528" if regioncode=="532528" & year==2002

replace regioncode="532529" if regioncode=="532529" & year==2002

replace regioncode="532530" if regioncode=="532530" & year==2002

replace regioncode="532531" if regioncode=="532531" & year==2002

replace regioncode="532532" if regioncode=="532532" & year==2002

replace regioncode="532621" if regioncode=="532621" & year==2002

replace regioncode="532622" if regioncode=="532622" & year==2002

replace regioncode="532623" if regioncode=="532623" & year==2002

replace regioncode="532624" if regioncode=="532624" & year==2002

replace regioncode="532625" if regioncode=="532625" & year==2002

replace regioncode="532626" if regioncode=="532626" & year==2002

replace regioncode="532627" if regioncode=="532627" & year==2002

replace regioncode="532628" if regioncode=="532628" & year==2002

replace regioncode="532801" if regioncode=="532801" & year==2002

replace regioncode="532822" if regioncode=="532822" & year==2002

replace regioncode="532823" if regioncode=="532823" & year==2002

replace regioncode="532901" if regioncode=="532901" & year==2002

replace regioncode="532922" if regioncode=="532922" & year==2002

replace regioncode="532923" if regioncode=="532923" & year==2002

replace regioncode="532924" if regioncode=="532924" & year==2002

replace regioncode="532925" if regioncode=="532925" & year==2002

replace regioncode="532926" if regioncode=="532926" & year==2002

replace regioncode="532927" if regioncode=="532927" & year==2002

replace regioncode="532928" if regioncode=="532928" & year==2002

replace regioncode="532929" if regioncode=="532929" & year==2002

replace regioncode="532930" if regioncode=="532930" & year==2002

replace regioncode="532931" if regioncode=="532931" & year==2002

replace regioncode="532932" if regioncode=="532932" & year==2002

replace regioncode="533102" if regioncode=="533102" & year==2002

replace regioncode="533103" if regioncode=="533103" & year==2002

replace regioncode="533122" if regioncode=="533122" & year==2002

replace regioncode="533123" if regioncode=="533123" & year==2002

replace regioncode="533124" if regioncode=="533124" & year==2002

replace regioncode="533321" if regioncode=="533321" & year==2002

replace regioncode="533323" if regioncode=="533323" & year==2002

replace regioncode="533324" if regioncode=="533324" & year==2002

replace regioncode="533325" if regioncode=="533325" & year==2002

replace regioncode="533421" if regioncode=="533421" & year==2002

replace regioncode="533422" if regioncode=="533422" & year==2002

replace regioncode="533423" if regioncode=="533423" & year==2002

replace regioncode="540102" if regioncode=="540102" & year==2002

replace regioncode="540121" if regioncode=="540121" & year==2002

replace regioncode="540122" if regioncode=="540122" & year==2002

replace regioncode="540123" if regioncode=="540123" & year==2002

replace regioncode="540124" if regioncode=="540124" & year==2002

replace regioncode="540125" if regioncode=="540125" & year==2002

replace regioncode="540126" if regioncode=="540126" & year==2002

replace regioncode="540127" if regioncode=="540127" & year==2002

replace regioncode="542121" if regioncode=="542121" & year==2002

replace regioncode="542122" if regioncode=="542122" & year==2002

replace regioncode="542123" if regioncode=="542123" & year==2002

replace regioncode="542124" if regioncode=="542124" & year==2002

replace regioncode="542125" if regioncode=="542125" & year==2002

replace regioncode="542126" if regioncode=="542126" & year==2002

replace regioncode="542127" if regioncode=="542127" & year==2002

replace regioncode="542128" if regioncode=="542128" & year==2002

replace regioncode="542129" if regioncode=="542129" & year==2002

replace regioncode="542132" if regioncode=="542132" & year==2002

replace regioncode="542133" if regioncode=="542133" & year==2002

replace regioncode="542221" if regioncode=="542221" & year==2002

replace regioncode="542223" if regioncode=="542223" & year==2002

replace regioncode="542224" if regioncode=="542224" & year==2002

replace regioncode="542225" if regioncode=="542225" & year==2002

replace regioncode="542227" if regioncode=="542227" & year==2002

replace regioncode="542228" if regioncode=="542228" & year==2002

replace regioncode="542229" if regioncode=="542229" & year==2002

replace regioncode="542231" if regioncode=="542231" & year==2002

replace regioncode="542232" if regioncode=="542232" & year==2002

replace regioncode="542233" if regioncode=="542233" & year==2002

replace regioncode="542301" if regioncode=="542301" & year==2002

replace regioncode="542322" if regioncode=="542322" & year==2002

replace regioncode="542323" if regioncode=="542323" & year==2002

replace regioncode="542324" if regioncode=="542324" & year==2002

replace regioncode="542325" if regioncode=="542325" & year==2002

replace regioncode="542326" if regioncode=="542326" & year==2002

replace regioncode="542327" if regioncode=="542327" & year==2002

replace regioncode="542328" if regioncode=="542328" & year==2002

replace regioncode="542329" if regioncode=="542329" & year==2002

replace regioncode="542330" if regioncode=="542330" & year==2002

replace regioncode="542331" if regioncode=="542331" & year==2002

replace regioncode="542332" if regioncode=="542332" & year==2002

replace regioncode="542334" if regioncode=="542334" & year==2002

replace regioncode="542336" if regioncode=="542336" & year==2002

replace regioncode="542338" if regioncode=="542338" & year==2002

replace regioncode="542421" if regioncode=="542421" & year==2002

replace regioncode="542422" if regioncode=="542422" & year==2002

replace regioncode="542423" if regioncode=="542423" & year==2002

replace regioncode="542424" if regioncode=="542424" & year==2002

replace regioncode="542425" if regioncode=="542425" & year==2002

replace regioncode="542426" if regioncode=="542426" & year==2002

replace regioncode="542427" if regioncode=="542427" & year==2002

replace regioncode="542429" if regioncode=="542429" & year==2002

replace regioncode="542430" if regioncode=="542430" & year==2002

replace regioncode="542523" if regioncode=="542523" & year==2002

replace regioncode="542621" if regioncode=="542621" & year==2002

replace regioncode="542622" if regioncode=="542622" & year==2002

replace regioncode="542623" if regioncode=="542623" & year==2002

replace regioncode="542625" if regioncode=="542625" & year==2002

replace regioncode="542626" if regioncode=="542626" & year==2002

replace regioncode="610102" if regioncode=="610102" & year==2002

replace regioncode="610103" if regioncode=="610103" & year==2002

replace regioncode="610104" if regioncode=="610104" & year==2002

replace regioncode="610111" if regioncode=="610111" & year==2002

replace regioncode="610112" if regioncode=="610112" & year==2002

replace regioncode="610113" if regioncode=="610113" & year==2002

replace regioncode="610114" if regioncode=="610114" & year==2002

replace regioncode="610115" if regioncode=="610115" & year==2002

replace regioncode="610116" if regioncode=="610116" & year==2002

replace regioncode="610122" if regioncode=="610122" & year==2002

replace regioncode="610124" if regioncode=="610124" & year==2002

replace regioncode="610125" if regioncode=="610125" & year==2002

replace regioncode="610126" if regioncode=="610126" & year==2002

replace regioncode="610202" if regioncode=="610202" & year==2002

replace regioncode="610203" if regioncode=="610203" & year==2002

replace regioncode="610204" if regioncode=="610204" & year==2002

replace regioncode="610222" if regioncode=="610222" & year==2002

replace regioncode="610302" if regioncode=="610302" & year==2002

replace regioncode="610303" if regioncode=="610303" & year==2002

replace regioncode="610304" if regioncode=="610321" & year==2002

replace regioncode="610322" if regioncode=="610322" & year==2002

replace regioncode="610323" if regioncode=="610323" & year==2002

replace regioncode="610324" if regioncode=="610324" & year==2002

replace regioncode="610326" if regioncode=="610326" & year==2002

replace regioncode="610327" if regioncode=="610327" & year==2002

replace regioncode="610328" if regioncode=="610328" & year==2002

replace regioncode="610329" if regioncode=="610329" & year==2002

replace regioncode="610330" if regioncode=="610330" & year==2002

replace regioncode="610331" if regioncode=="610331" & year==2002

replace regioncode="610402" if regioncode=="610402" & year==2002

replace regioncode="610403" if regioncode=="610403" & year==2002

replace regioncode="610404" if regioncode=="610404" & year==2002

replace regioncode="610422" if regioncode=="610422" & year==2002

replace regioncode="610423" if regioncode=="610423" & year==2002

replace regioncode="610424" if regioncode=="610424" & year==2002

replace regioncode="610425" if regioncode=="610425" & year==2002

replace regioncode="610426" if regioncode=="610426" & year==2002

replace regioncode="610427" if regioncode=="610427" & year==2002

replace regioncode="610428" if regioncode=="610428" & year==2002

replace regioncode="610429" if regioncode=="610429" & year==2002

replace regioncode="610430" if regioncode=="610430" & year==2002

replace regioncode="610431" if regioncode=="610431" & year==2002

replace regioncode="610481" if regioncode=="610481" & year==2002

replace regioncode="610502" if regioncode=="610502" & year==2002

replace regioncode="610521" if regioncode=="610521" & year==2002

replace regioncode="610522" if regioncode=="610522" & year==2002

replace regioncode="610523" if regioncode=="610523" & year==2002

replace regioncode="610524" if regioncode=="610524" & year==2002

replace regioncode="610525" if regioncode=="610525" & year==2002

replace regioncode="610526" if regioncode=="610526" & year==2002

replace regioncode="610527" if regioncode=="610527" & year==2002

replace regioncode="610528" if regioncode=="610528" & year==2002

replace regioncode="610581" if regioncode=="610581" & year==2002

replace regioncode="610582" if regioncode=="610582" & year==2002

replace regioncode="610602" if regioncode=="610602" & year==2002

replace regioncode="610621" if regioncode=="610621" & year==2002

replace regioncode="610622" if regioncode=="610622" & year==2002

replace regioncode="610623" if regioncode=="610623" & year==2002

replace regioncode="610624" if regioncode=="610624" & year==2002

replace regioncode="610625" if regioncode=="610625" & year==2002

replace regioncode="610626" if regioncode=="610626" & year==2002

replace regioncode="610627" if regioncode=="610627" & year==2002

replace regioncode="610628" if regioncode=="610628" & year==2002

replace regioncode="610629" if regioncode=="610629" & year==2002

replace regioncode="610630" if regioncode=="610630" & year==2002

replace regioncode="610631" if regioncode=="610631" & year==2002

replace regioncode="610632" if regioncode=="610632" & year==2002

replace regioncode="610702" if regioncode=="610702" & year==2002

replace regioncode="610721" if regioncode=="610721" & year==2002

replace regioncode="610722" if regioncode=="610722" & year==2002

replace regioncode="610723" if regioncode=="610723" & year==2002

replace regioncode="610724" if regioncode=="610724" & year==2002

replace regioncode="610725" if regioncode=="610725" & year==2002

replace regioncode="610726" if regioncode=="610726" & year==2002

replace regioncode="610727" if regioncode=="610727" & year==2002

replace regioncode="610728" if regioncode=="610728" & year==2002

replace regioncode="610729" if regioncode=="610729" & year==2002

replace regioncode="610730" if regioncode=="610730" & year==2002

replace regioncode="610802" if regioncode=="610802" & year==2002

replace regioncode="610821" if regioncode=="610821" & year==2002

replace regioncode="610822" if regioncode=="610822" & year==2002

replace regioncode="610823" if regioncode=="610823" & year==2002

replace regioncode="610824" if regioncode=="610824" & year==2002

replace regioncode="610825" if regioncode=="610825" & year==2002

replace regioncode="610826" if regioncode=="610826" & year==2002

replace regioncode="610827" if regioncode=="610827" & year==2002

replace regioncode="610828" if regioncode=="610828" & year==2002

replace regioncode="610829" if regioncode=="610829" & year==2002

replace regioncode="610830" if regioncode=="610830" & year==2002

replace regioncode="610831" if regioncode=="610831" & year==2002

replace regioncode="610902" if regioncode=="610902" & year==2002

replace regioncode="610921" if regioncode=="610921" & year==2002

replace regioncode="610922" if regioncode=="610922" & year==2002

replace regioncode="610923" if regioncode=="610923" & year==2002

replace regioncode="610924" if regioncode=="610924" & year==2002

replace regioncode="610925" if regioncode=="610925" & year==2002

replace regioncode="610926" if regioncode=="610926" & year==2002

replace regioncode="610927" if regioncode=="610927" & year==2002

replace regioncode="610928" if regioncode=="610928" & year==2002

replace regioncode="610929" if regioncode=="610929" & year==2002

replace regioncode="611002" if regioncode=="611002" & year==2002

replace regioncode="611021" if regioncode=="611021" & year==2002

replace regioncode="611022" if regioncode=="611022" & year==2002

replace regioncode="611023" if regioncode=="611023" & year==2002

replace regioncode="611024" if regioncode=="611024" & year==2002

replace regioncode="611025" if regioncode=="611025" & year==2002

replace regioncode="611026" if regioncode=="611026" & year==2002

replace regioncode="620102" if regioncode=="620102" & year==2002

replace regioncode="620103" if regioncode=="620103" & year==2002

replace regioncode="620104" if regioncode=="620104" & year==2002

replace regioncode="620105" if regioncode=="620105" & year==2002

replace regioncode="620111" if regioncode=="620111" & year==2002

replace regioncode="620121" if regioncode=="620121" & year==2002

replace regioncode="620122" if regioncode=="620122" & year==2002

replace regioncode="620123" if regioncode=="620123" & year==2002

replace regioncode="620201" if regioncode=="620201" & year==2002

replace regioncode="620302" if regioncode=="620302" & year==2002

replace regioncode="620321" if regioncode=="620321" & year==2002

replace regioncode="620402" if regioncode=="620402" & year==2002

replace regioncode="620403" if regioncode=="620403" & year==2002

replace regioncode="620421" if regioncode=="620421" & year==2002

replace regioncode="620422" if regioncode=="620422" & year==2002

replace regioncode="620423" if regioncode=="620423" & year==2002

replace regioncode="620502" if regioncode=="620502" & year==2002

replace regioncode="620503" if regioncode=="620503" & year==2002

replace regioncode="620521" if regioncode=="620521" & year==2002

replace regioncode="620522" if regioncode=="620522" & year==2002

replace regioncode="620523" if regioncode=="620523" & year==2002

replace regioncode="620524" if regioncode=="620524" & year==2002

replace regioncode="620525" if regioncode=="620525" & year==2002

replace regioncode="622301" if regioncode=="620601" & year==2002

replace regioncode="622322" if regioncode=="620621" & year==2002

replace regioncode="622323" if regioncode=="620622" & year==2002

replace regioncode="622326" if regioncode=="620623" & year==2002

replace regioncode="622201" if regioncode=="622201" & year==2002

replace regioncode="622222" if regioncode=="622222" & year==2002

replace regioncode="622223" if regioncode=="622223" & year==2002

replace regioncode="622224" if regioncode=="622224" & year==2002

replace regioncode="622225" if regioncode=="622225" & year==2002

replace regioncode="622226" if regioncode=="622226" & year==2002

replace regioncode="622701" if regioncode=="622701" & year==2002

replace regioncode="622722" if regioncode=="622722" & year==2002

replace regioncode="622723" if regioncode=="622723" & year==2002

replace regioncode="622724" if regioncode=="622724" & year==2002

replace regioncode="622725" if regioncode=="622725" & year==2002

replace regioncode="622726" if regioncode=="622726" & year==2002

replace regioncode="622727" if regioncode=="622727" & year==2002

replace regioncode="622102" if regioncode=="622102" & year==2002

replace regioncode="622123" if regioncode=="622123" & year==2002

replace regioncode="622126" if regioncode=="622126" & year==2002

replace regioncode="622124" if regioncode=="622124" & year==2002

replace regioncode="622125" if regioncode=="622125" & year==2002

replace regioncode="622101" if regioncode=="622101" & year==2002

replace regioncode="622103" if regioncode=="622103" & year==2002

replace regioncode="622801" if regioncode=="622801" & year==2002

replace regioncode="622821" if regioncode=="622821" & year==2002

replace regioncode="622822" if regioncode=="622822" & year==2002

replace regioncode="622823" if regioncode=="622823" & year==2002

replace regioncode="622824" if regioncode=="622824" & year==2002

replace regioncode="622825" if regioncode=="622825" & year==2002

replace regioncode="622826" if regioncode=="622826" & year==2002

replace regioncode="622827" if regioncode=="622827" & year==2002

replace regioncode="622421" if regioncode=="622421" & year==2002

replace regioncode="622424" if regioncode=="622424" & year==2002

replace regioncode="622425" if regioncode=="622425" & year==2002

replace regioncode="622426" if regioncode=="622426" & year==2002

replace regioncode="622427" if regioncode=="622427" & year==2002

replace regioncode="622428" if regioncode=="622428" & year==2002

replace regioncode="622429" if regioncode=="622429" & year==2002

replace regioncode="622621" if regioncode=="622621" & year==2002

replace regioncode="622624" if regioncode=="622624" & year==2002

replace regioncode="622626" if regioncode=="622626" & year==2002

replace regioncode="622623" if regioncode=="622623" & year==2002

replace regioncode="622625" if regioncode=="622625" & year==2002

replace regioncode="622627" if regioncode=="622627" & year==2002

replace regioncode="622628" if regioncode=="622628" & year==2002

replace regioncode="622630" if regioncode=="622630" & year==2002

replace regioncode="622629" if regioncode=="622629" & year==2002

replace regioncode="622901" if regioncode=="622901" & year==2002

replace regioncode="622921" if regioncode=="622921" & year==2002

replace regioncode="622922" if regioncode=="622922" & year==2002

replace regioncode="622923" if regioncode=="622923" & year==2002

replace regioncode="622924" if regioncode=="622924" & year==2002

replace regioncode="622925" if regioncode=="622925" & year==2002

replace regioncode="622926" if regioncode=="622926" & year==2002

replace regioncode="622927" if regioncode=="622927" & year==2002

replace regioncode="623001" if regioncode=="623001" & year==2002

replace regioncode="623021" if regioncode=="623021" & year==2002

replace regioncode="623022" if regioncode=="623022" & year==2002

replace regioncode="623023" if regioncode=="623023" & year==2002

replace regioncode="623024" if regioncode=="623024" & year==2002

replace regioncode="623025" if regioncode=="623025" & year==2002

replace regioncode="623026" if regioncode=="623026" & year==2002

replace regioncode="623027" if regioncode=="623027" & year==2002

replace regioncode="630102" if regioncode=="630102" & year==2002

replace regioncode="630103" if regioncode=="630103" & year==2002

replace regioncode="630104" if regioncode=="630104" & year==2002

replace regioncode="630105" if regioncode=="630105" & year==2002

replace regioncode="630121" if regioncode=="630121" & year==2002

replace regioncode="630122" if regioncode=="630122" & year==2002

replace regioncode="630123" if regioncode=="630123" & year==2002

replace regioncode="632121" if regioncode=="632121" & year==2002

replace regioncode="632122" if regioncode=="632122" & year==2002

replace regioncode="632123" if regioncode=="632123" & year==2002

replace regioncode="632126" if regioncode=="632126" & year==2002

replace regioncode="632127" if regioncode=="632127" & year==2002

replace regioncode="632128" if regioncode=="632128" & year==2002

replace regioncode="632221" if regioncode=="632221" & year==2002

replace regioncode="632222" if regioncode=="632222" & year==2002

replace regioncode="632223" if regioncode=="632223" & year==2002

replace regioncode="632224" if regioncode=="632224" & year==2002

replace regioncode="632321" if regioncode=="632321" & year==2002

replace regioncode="632322" if regioncode=="632322" & year==2002

replace regioncode="632521" if regioncode=="632521" & year==2002

replace regioncode="632522" if regioncode=="632522" & year==2002

replace regioncode="632523" if regioncode=="632523" & year==2002

replace regioncode="632524" if regioncode=="632524" & year==2002

replace regioncode="632525" if regioncode=="632525" & year==2002

replace regioncode="632621" if regioncode=="632621" & year==2002

replace regioncode="632622" if regioncode=="632622" & year==2002

replace regioncode="632623" if regioncode=="632623" & year==2002

replace regioncode="632624" if regioncode=="632624" & year==2002

replace regioncode="632625" if regioncode=="632625" & year==2002

replace regioncode="632626" if regioncode=="632626" & year==2002

replace regioncode="632721" if regioncode=="632721" & year==2002

replace regioncode="632723" if regioncode=="632723" & year==2002

replace regioncode="632724" if regioncode=="632724" & year==2002

replace regioncode="632725" if regioncode=="632725" & year==2002

replace regioncode="632726" if regioncode=="632726" & year==2002

replace regioncode="632801" if regioncode=="632801" & year==2002

replace regioncode="632802" if regioncode=="632802" & year==2002

replace regioncode="632821" if regioncode=="632821" & year==2002

replace regioncode="632822" if regioncode=="632822" & year==2002

replace regioncode="640104" if regioncode=="640102" & year==2002

replace regioncode="640105" if regioncode=="640103" & year==2002

replace regioncode="640106" if regioncode=="640111" & year==2002

replace regioncode="640121" if regioncode=="640121" & year==2002

replace regioncode="640122" if regioncode=="640122" & year==2002

replace regioncode="640181" if regioncode=="640382" & year==2002

replace regioncode="640202" if regioncode=="640202" & year==2002

replace regioncode="640203" if regioncode=="640203" & year==2002

replace regioncode="640221" if regioncode=="640221" & year==2002

replace regioncode="640302" if regioncode=="640302" & year==2002

replace regioncode="640321" if regioncode=="640321" & year==2002

replace regioncode="640322" if regioncode=="640322" & year==2002

replace regioncode="640323" if regioncode=="640323" & year==2002

replace regioncode="640324" if regioncode=="640324" & year==2002

replace regioncode="640381" if regioncode=="640381" & year==2002

replace regioncode="640402" if regioncode=="642221" & year==2002

replace regioncode="640421" if regioncode=="642222" & year==2002

replace regioncode="640422" if regioncode=="642223" & year==2002

replace regioncode="640423" if regioncode=="642224" & year==2002

replace regioncode="640424" if regioncode=="642225" & year==2002

replace regioncode="640425" if regioncode=="642226" & year==2002

replace regioncode="650102" if regioncode=="650102" & year==2002

replace regioncode="650103" if regioncode=="650103" & year==2002

replace regioncode="650104" if regioncode=="650104" & year==2002

replace regioncode="650105" if regioncode=="650105" & year==2002

replace regioncode="650106" if regioncode=="650106" & year==2002

replace regioncode="650107" if regioncode=="650107" & year==2002

replace regioncode="650108" if regioncode=="650108" & year==2002

replace regioncode="650121" if regioncode=="650121" & year==2002

replace regioncode="650202" if regioncode=="650202" & year==2002

replace regioncode="650203" if regioncode=="650203" & year==2002

replace regioncode="650204" if regioncode=="650204" & year==2002

replace regioncode="650205" if regioncode=="650205" & year==2002

replace regioncode="652101" if regioncode=="652101" & year==2002

replace regioncode="652122" if regioncode=="652122" & year==2002

replace regioncode="652123" if regioncode=="652123" & year==2002

replace regioncode="652201" if regioncode=="652201" & year==2002

replace regioncode="652222" if regioncode=="652222" & year==2002

replace regioncode="652223" if regioncode=="652223" & year==2002

replace regioncode="652301" if regioncode=="652301" & year==2002

replace regioncode="652302" if regioncode=="652302" & year==2002

replace regioncode="652303" if regioncode=="652303" & year==2002

replace regioncode="652323" if regioncode=="652323" & year==2002

replace regioncode="652324" if regioncode=="652324" & year==2002

replace regioncode="652325" if regioncode=="652325" & year==2002

replace regioncode="652327" if regioncode=="652327" & year==2002

replace regioncode="652328" if regioncode=="652328" & year==2002

replace regioncode="652701" if regioncode=="652701" & year==2002

replace regioncode="652722" if regioncode=="652722" & year==2002

replace regioncode="652723" if regioncode=="652723" & year==2002

replace regioncode="652801" if regioncode=="652801" & year==2002

replace regioncode="652822" if regioncode=="652822" & year==2002

replace regioncode="652823" if regioncode=="652823" & year==2002

replace regioncode="652824" if regioncode=="652824" & year==2002

replace regioncode="652825" if regioncode=="652825" & year==2002

replace regioncode="652826" if regioncode=="652826" & year==2002

replace regioncode="652827" if regioncode=="652827" & year==2002

replace regioncode="652828" if regioncode=="652828" & year==2002

replace regioncode="652829" if regioncode=="652829" & year==2002

replace regioncode="652901" if regioncode=="652901" & year==2002

replace regioncode="652922" if regioncode=="652922" & year==2002

replace regioncode="652923" if regioncode=="652923" & year==2002

replace regioncode="652924" if regioncode=="652924" & year==2002

replace regioncode="652925" if regioncode=="652925" & year==2002

replace regioncode="652926" if regioncode=="652926" & year==2002

replace regioncode="652928" if regioncode=="652928" & year==2002

replace regioncode="652929" if regioncode=="652929" & year==2002

replace regioncode="653001" if regioncode=="653001" & year==2002

replace regioncode="653022" if regioncode=="653022" & year==2002

replace regioncode="653023" if regioncode=="653023" & year==2002

replace regioncode="653024" if regioncode=="653024" & year==2002

replace regioncode="653101" if regioncode=="653101" & year==2002

replace regioncode="653121" if regioncode=="653121" & year==2002

replace regioncode="653122" if regioncode=="653122" & year==2002

replace regioncode="653123" if regioncode=="653123" & year==2002

replace regioncode="653124" if regioncode=="653124" & year==2002

replace regioncode="653125" if regioncode=="653125" & year==2002

replace regioncode="653126" if regioncode=="653126" & year==2002

replace regioncode="653127" if regioncode=="653127" & year==2002

replace regioncode="653128" if regioncode=="653128" & year==2002

replace regioncode="653129" if regioncode=="653129" & year==2002

replace regioncode="653130" if regioncode=="653130" & year==2002

replace regioncode="653131" if regioncode=="653131" & year==2002

replace regioncode="653201" if regioncode=="653201" & year==2002

replace regioncode="653221" if regioncode=="653221" & year==2002

replace regioncode="653222" if regioncode=="653222" & year==2002

replace regioncode="653223" if regioncode=="653223" & year==2002

replace regioncode="653224" if regioncode=="653224" & year==2002

replace regioncode="653225" if regioncode=="653225" & year==2002

replace regioncode="653226" if regioncode=="653226" & year==2002

replace regioncode="653227" if regioncode=="653227" & year==2002

replace regioncode="654002" if regioncode=="654002" & year==2002

replace regioncode="654003" if regioncode=="654003" & year==2002

replace regioncode="654021" if regioncode=="654021" & year==2002

replace regioncode="654022" if regioncode=="654022" & year==2002

replace regioncode="654023" if regioncode=="654023" & year==2002

replace regioncode="654024" if regioncode=="654024" & year==2002

replace regioncode="654025" if regioncode=="654025" & year==2002

replace regioncode="654026" if regioncode=="654026" & year==2002

replace regioncode="654027" if regioncode=="654027" & year==2002

replace regioncode="654028" if regioncode=="654028" & year==2002

replace regioncode="654201" if regioncode=="654201" & year==2002

replace regioncode="654202" if regioncode=="654202" & year==2002

replace regioncode="654221" if regioncode=="654221" & year==2002

replace regioncode="654223" if regioncode=="654223" & year==2002

replace regioncode="654224" if regioncode=="654224" & year==2002

replace regioncode="654225" if regioncode=="654225" & year==2002

replace regioncode="654226" if regioncode=="654226" & year==2002

replace regioncode="654301" if regioncode=="654301" & year==2002

replace regioncode="654321" if regioncode=="654321" & year==2002

replace regioncode="654322" if regioncode=="654322" & year==2002

replace regioncode="654323" if regioncode=="654323" & year==2002

replace regioncode="654324" if regioncode=="654324" & year==2002

replace regioncode="654325" if regioncode=="654325" & year==2002

replace regioncode="654326" if regioncode=="654326" & year==2002

replace regioncode="659001" if regioncode=="659001" & year==2002

replace regioncode="110101" if regioncode=="110101" & year==2004

replace regioncode="110102" if regioncode=="110102" & year==2004

replace regioncode="110103" if regioncode=="110103" & year==2004

replace regioncode="110104" if regioncode=="110104" & year==2004

replace regioncode="110105" if regioncode=="110105" & year==2004

replace regioncode="110106" if regioncode=="110106" & year==2004

replace regioncode="110107" if regioncode=="110107" & year==2004

replace regioncode="110108" if regioncode=="110108" & year==2004

replace regioncode="110109" if regioncode=="110109" & year==2004

replace regioncode="110111" if regioncode=="110111" & year==2004

replace regioncode="110112" if regioncode=="110112" & year==2004

replace regioncode="110113" if regioncode=="110113" & year==2004

replace regioncode="110114" if regioncode=="110114" & year==2004

replace regioncode="110115" if regioncode=="110115" & year==2004

replace regioncode="110117" if regioncode=="110117" & year==2004

replace regioncode="110116" if regioncode=="110116" & year==2004

replace regioncode="110228" if regioncode=="110228" & year==2004

replace regioncode="110229" if regioncode=="110229" & year==2004

replace regioncode="120101" if regioncode=="120101" & year==2004

replace regioncode="120102" if regioncode=="120102" & year==2004

replace regioncode="120103" if regioncode=="120103" & year==2004

replace regioncode="120104" if regioncode=="120104" & year==2004

replace regioncode="120105" if regioncode=="120105" & year==2004

replace regioncode="120106" if regioncode=="120106" & year==2004

replace regioncode="120107" if regioncode=="120107" & year==2004

replace regioncode="120108" if regioncode=="120108" & year==2004

replace regioncode="120109" if regioncode=="120109" & year==2004

replace regioncode="120110" if regioncode=="120110" & year==2004

replace regioncode="120111" if regioncode=="120111" & year==2004

replace regioncode="120112" if regioncode=="120112" & year==2004

replace regioncode="120113" if regioncode=="120113" & year==2004

replace regioncode="120114" if regioncode=="120114" & year==2004

replace regioncode="120115" if regioncode=="120115" & year==2004

replace regioncode="120221" if regioncode=="120221" & year==2004

replace regioncode="120223" if regioncode=="120223" & year==2004

replace regioncode="120225" if regioncode=="120225" & year==2004

replace regioncode="130102" if regioncode=="130102" & year==2004

replace regioncode="130103" if regioncode=="130103" & year==2004

replace regioncode="130104" if regioncode=="130104" & year==2004

replace regioncode="130105" if regioncode=="130105" & year==2004

replace regioncode="130107" if regioncode=="130107" & year==2004

replace regioncode="130108" if regioncode=="130108" & year==2004

replace regioncode="130121" if regioncode=="130121" & year==2004

replace regioncode="130123" if regioncode=="130123" & year==2004

replace regioncode="130124" if regioncode=="130124" & year==2004

replace regioncode="130125" if regioncode=="130125" & year==2004

replace regioncode="130126" if regioncode=="130126" & year==2004

replace regioncode="130127" if regioncode=="130127" & year==2004

replace regioncode="130128" if regioncode=="130128" & year==2004

replace regioncode="130129" if regioncode=="130129" & year==2004

replace regioncode="130130" if regioncode=="130130" & year==2004

replace regioncode="130131" if regioncode=="130131" & year==2004

replace regioncode="130132" if regioncode=="130132" & year==2004

replace regioncode="130133" if regioncode=="130133" & year==2004

replace regioncode="130181" if regioncode=="130181" & year==2004

replace regioncode="130182" if regioncode=="130182" & year==2004

replace regioncode="130183" if regioncode=="130183" & year==2004

replace regioncode="130184" if regioncode=="130184" & year==2004

replace regioncode="130185" if regioncode=="130185" & year==2004

replace regioncode="130202" if regioncode=="130202" & year==2004

replace regioncode="130203" if regioncode=="130203" & year==2004

replace regioncode="130204" if regioncode=="130204" & year==2004

replace regioncode="130205" if regioncode=="130205" & year==2004

replace regioncode="130208" if regioncode=="130208" & year==2004

replace regioncode="130223" if regioncode=="130223" & year==2004

replace regioncode="130224" if regioncode=="130224" & year==2004

replace regioncode="130225" if regioncode=="130225" & year==2004

replace regioncode="130227" if regioncode=="130227" & year==2004

replace regioncode="130229" if regioncode=="130229" & year==2004

replace regioncode="130230" if regioncode=="130230" & year==2004

replace regioncode="130281" if regioncode=="130281" & year==2004

replace regioncode="130207" if regioncode=="130207" & year==2004

replace regioncode="130283" if regioncode=="130283" & year==2004

replace regioncode="130302" if regioncode=="130302" & year==2004

replace regioncode="130303" if regioncode=="130303" & year==2004

replace regioncode="130304" if regioncode=="130304" & year==2004

replace regioncode="130321" if regioncode=="130321" & year==2004

replace regioncode="130322" if regioncode=="130322" & year==2004

replace regioncode="130323" if regioncode=="130323" & year==2004

replace regioncode="130324" if regioncode=="130324" & year==2004

replace regioncode="130402" if regioncode=="130402" & year==2004

replace regioncode="130403" if regioncode=="130403" & year==2004

replace regioncode="130404" if regioncode=="130404" & year==2004

replace regioncode="130406" if regioncode=="130406" & year==2004

replace regioncode="130421" if regioncode=="130421" & year==2004

replace regioncode="130423" if regioncode=="130423" & year==2004

replace regioncode="130424" if regioncode=="130424" & year==2004

replace regioncode="130425" if regioncode=="130425" & year==2004

replace regioncode="130426" if regioncode=="130426" & year==2004

replace regioncode="130427" if regioncode=="130427" & year==2004

replace regioncode="130428" if regioncode=="130428" & year==2004

replace regioncode="130429" if regioncode=="130429" & year==2004

replace regioncode="130430" if regioncode=="130430" & year==2004

replace regioncode="130431" if regioncode=="130431" & year==2004

replace regioncode="130432" if regioncode=="130432" & year==2004

replace regioncode="130433" if regioncode=="130433" & year==2004

replace regioncode="130434" if regioncode=="130434" & year==2004

replace regioncode="130435" if regioncode=="130435" & year==2004

replace regioncode="130481" if regioncode=="130481" & year==2004

replace regioncode="130502" if regioncode=="130502" & year==2004

replace regioncode="130503" if regioncode=="130503" & year==2004

replace regioncode="130521" if regioncode=="130521" & year==2004

replace regioncode="130522" if regioncode=="130522" & year==2004

replace regioncode="130523" if regioncode=="130523" & year==2004

replace regioncode="130524" if regioncode=="130524" & year==2004

replace regioncode="130525" if regioncode=="130525" & year==2004

replace regioncode="130526" if regioncode=="130526" & year==2004

replace regioncode="130527" if regioncode=="130527" & year==2004

replace regioncode="130528" if regioncode=="130528" & year==2004

replace regioncode="130529" if regioncode=="130529" & year==2004

replace regioncode="130530" if regioncode=="130530" & year==2004

replace regioncode="130531" if regioncode=="130531" & year==2004

replace regioncode="130532" if regioncode=="130532" & year==2004

replace regioncode="130533" if regioncode=="130533" & year==2004

replace regioncode="130534" if regioncode=="130534" & year==2004

replace regioncode="130535" if regioncode=="130535" & year==2004

replace regioncode="130581" if regioncode=="130581" & year==2004

replace regioncode="130582" if regioncode=="130582" & year==2004

replace regioncode="130602" if regioncode=="130602" & year==2004

replace regioncode="130603" if regioncode=="130603" & year==2004

replace regioncode="130604" if regioncode=="130604" & year==2004

replace regioncode="130621" if regioncode=="130621" & year==2004

replace regioncode="130622" if regioncode=="130622" & year==2004

replace regioncode="130623" if regioncode=="130623" & year==2004

replace regioncode="130624" if regioncode=="130624" & year==2004

replace regioncode="130625" if regioncode=="130625" & year==2004

replace regioncode="130626" if regioncode=="130626" & year==2004

replace regioncode="130627" if regioncode=="130627" & year==2004

replace regioncode="130628" if regioncode=="130628" & year==2004

replace regioncode="130629" if regioncode=="130629" & year==2004

replace regioncode="130630" if regioncode=="130630" & year==2004

replace regioncode="130631" if regioncode=="130631" & year==2004

replace regioncode="130632" if regioncode=="130632" & year==2004

replace regioncode="130633" if regioncode=="130633" & year==2004

replace regioncode="130634" if regioncode=="130634" & year==2004

replace regioncode="130635" if regioncode=="130635" & year==2004

replace regioncode="130636" if regioncode=="130636" & year==2004

replace regioncode="130637" if regioncode=="130637" & year==2004

replace regioncode="130638" if regioncode=="130638" & year==2004

replace regioncode="130681" if regioncode=="130681" & year==2004

replace regioncode="130682" if regioncode=="130682" & year==2004

replace regioncode="130683" if regioncode=="130683" & year==2004

replace regioncode="130684" if regioncode=="130684" & year==2004

replace regioncode="130702" if regioncode=="130702" & year==2004

replace regioncode="130703" if regioncode=="130703" & year==2004

replace regioncode="130705" if regioncode=="130705" & year==2004

replace regioncode="130706" if regioncode=="130706" & year==2004

replace regioncode="130721" if regioncode=="130721" & year==2004

replace regioncode="130722" if regioncode=="130722" & year==2004

replace regioncode="130723" if regioncode=="130723" & year==2004

replace regioncode="130724" if regioncode=="130724" & year==2004

replace regioncode="130725" if regioncode=="130725" & year==2004

replace regioncode="130726" if regioncode=="130726" & year==2004

replace regioncode="130727" if regioncode=="130727" & year==2004

replace regioncode="130728" if regioncode=="130728" & year==2004

replace regioncode="130729" if regioncode=="130729" & year==2004

replace regioncode="130730" if regioncode=="130730" & year==2004

replace regioncode="130731" if regioncode=="130731" & year==2004

replace regioncode="130732" if regioncode=="130732" & year==2004

replace regioncode="130733" if regioncode=="130733" & year==2004

replace regioncode="130802" if regioncode=="130802" & year==2004

replace regioncode="130803" if regioncode=="130803" & year==2004

replace regioncode="130804" if regioncode=="130804" & year==2004

replace regioncode="130821" if regioncode=="130821" & year==2004

replace regioncode="130822" if regioncode=="130822" & year==2004

replace regioncode="130823" if regioncode=="130823" & year==2004

replace regioncode="130824" if regioncode=="130824" & year==2004

replace regioncode="130825" if regioncode=="130825" & year==2004

replace regioncode="130826" if regioncode=="130826" & year==2004

replace regioncode="130827" if regioncode=="130827" & year==2004

replace regioncode="130828" if regioncode=="130828" & year==2004

replace regioncode="130902" if regioncode=="130902" & year==2004

replace regioncode="130903" if regioncode=="130903" & year==2004

replace regioncode="130921" if regioncode=="130921" & year==2004

replace regioncode="130922" if regioncode=="130922" & year==2004

replace regioncode="130923" if regioncode=="130923" & year==2004

replace regioncode="130924" if regioncode=="130924" & year==2004

replace regioncode="130925" if regioncode=="130925" & year==2004

replace regioncode="130926" if regioncode=="130926" & year==2004

replace regioncode="130927" if regioncode=="130927" & year==2004

replace regioncode="130928" if regioncode=="130928" & year==2004

replace regioncode="130929" if regioncode=="130929" & year==2004

replace regioncode="130930" if regioncode=="130930" & year==2004

replace regioncode="130981" if regioncode=="130981" & year==2004

replace regioncode="130982" if regioncode=="130982" & year==2004

replace regioncode="130983" if regioncode=="130983" & year==2004

replace regioncode="130984" if regioncode=="130984" & year==2004

replace regioncode="131002" if regioncode=="131002" & year==2004

replace regioncode="131003" if regioncode=="131003" & year==2004

replace regioncode="131022" if regioncode=="131022" & year==2004

replace regioncode="131023" if regioncode=="131023" & year==2004

replace regioncode="131024" if regioncode=="131024" & year==2004

replace regioncode="131025" if regioncode=="131025" & year==2004

replace regioncode="131026" if regioncode=="131026" & year==2004

replace regioncode="131028" if regioncode=="131028" & year==2004

replace regioncode="131081" if regioncode=="131081" & year==2004

replace regioncode="131082" if regioncode=="131082" & year==2004

replace regioncode="131102" if regioncode=="131102" & year==2004

replace regioncode="131121" if regioncode=="131121" & year==2004

replace regioncode="131122" if regioncode=="131122" & year==2004

replace regioncode="131123" if regioncode=="131123" & year==2004

replace regioncode="131124" if regioncode=="131124" & year==2004

replace regioncode="131125" if regioncode=="131125" & year==2004

replace regioncode="131126" if regioncode=="131126" & year==2004

replace regioncode="131127" if regioncode=="131127" & year==2004

replace regioncode="131128" if regioncode=="131128" & year==2004

replace regioncode="131181" if regioncode=="131181" & year==2004

replace regioncode="131182" if regioncode=="131182" & year==2004

replace regioncode="140105" if regioncode=="140105" & year==2004

replace regioncode="140106" if regioncode=="140106" & year==2004

replace regioncode="140107" if regioncode=="140107" & year==2004

replace regioncode="140108" if regioncode=="140108" & year==2004

replace regioncode="140109" if regioncode=="140109" & year==2004

replace regioncode="140110" if regioncode=="140110" & year==2004

replace regioncode="140121" if regioncode=="140121" & year==2004

replace regioncode="140122" if regioncode=="140122" & year==2004

replace regioncode="140123" if regioncode=="140123" & year==2004

replace regioncode="140181" if regioncode=="140181" & year==2004

replace regioncode="140202" if regioncode=="140202" & year==2004

replace regioncode="140203" if regioncode=="140203" & year==2004

replace regioncode="140211" if regioncode=="140211" & year==2004

replace regioncode="140212" if regioncode=="140212" & year==2004

replace regioncode="140221" if regioncode=="140221" & year==2004

replace regioncode="140222" if regioncode=="140222" & year==2004

replace regioncode="140223" if regioncode=="140223" & year==2004

replace regioncode="140224" if regioncode=="140224" & year==2004

replace regioncode="140225" if regioncode=="140225" & year==2004

replace regioncode="140226" if regioncode=="140226" & year==2004

replace regioncode="140227" if regioncode=="140227" & year==2004

replace regioncode="140302" if regioncode=="140302" & year==2004

replace regioncode="140303" if regioncode=="140303" & year==2004

replace regioncode="140311" if regioncode=="140311" & year==2004

replace regioncode="140321" if regioncode=="140321" & year==2004

replace regioncode="140322" if regioncode=="140322" & year==2004

replace regioncode="140402" if regioncode=="140402" & year==2004

replace regioncode="140411" if regioncode=="140411" & year==2004

replace regioncode="140421" if regioncode=="140421" & year==2004

replace regioncode="140423" if regioncode=="140423" & year==2004

replace regioncode="140424" if regioncode=="140424" & year==2004

replace regioncode="140425" if regioncode=="140425" & year==2004

replace regioncode="140426" if regioncode=="140426" & year==2004

replace regioncode="140427" if regioncode=="140427" & year==2004

replace regioncode="140428" if regioncode=="140428" & year==2004

replace regioncode="140429" if regioncode=="140429" & year==2004

replace regioncode="140430" if regioncode=="140430" & year==2004

replace regioncode="140431" if regioncode=="140431" & year==2004

replace regioncode="140481" if regioncode=="140481" & year==2004

replace regioncode="140502" if regioncode=="140502" & year==2004

replace regioncode="140521" if regioncode=="140521" & year==2004

replace regioncode="140522" if regioncode=="140522" & year==2004

replace regioncode="140524" if regioncode=="140524" & year==2004

replace regioncode="140525" if regioncode=="140525" & year==2004

replace regioncode="140581" if regioncode=="140581" & year==2004

replace regioncode="140602" if regioncode=="140602" & year==2004

replace regioncode="140603" if regioncode=="140603" & year==2004

replace regioncode="140621" if regioncode=="140621" & year==2004

replace regioncode="140622" if regioncode=="140622" & year==2004

replace regioncode="140623" if regioncode=="140623" & year==2004

replace regioncode="140624" if regioncode=="140624" & year==2004

replace regioncode="140902" if regioncode=="140902" & year==2004

replace regioncode="140981" if regioncode=="140981" & year==2004

replace regioncode="140921" if regioncode=="140921" & year==2004

replace regioncode="140922" if regioncode=="140922" & year==2004

replace regioncode="140923" if regioncode=="140923" & year==2004

replace regioncode="140924" if regioncode=="140924" & year==2004

replace regioncode="140925" if regioncode=="140925" & year==2004

replace regioncode="140926" if regioncode=="140926" & year==2004

replace regioncode="140927" if regioncode=="140927" & year==2004

replace regioncode="140928" if regioncode=="140928" & year==2004

replace regioncode="140929" if regioncode=="140929" & year==2004

replace regioncode="140930" if regioncode=="140930" & year==2004

replace regioncode="140931" if regioncode=="140931" & year==2004

replace regioncode="140932" if regioncode=="140932" & year==2004

replace regioncode="141181" if regioncode=="141181" & year==2004

replace regioncode="141102" if regioncode=="141102" & year==2004

replace regioncode="141182" if regioncode=="141182" & year==2004

replace regioncode="141121" if regioncode=="141121" & year==2004

replace regioncode="141122" if regioncode=="141122" & year==2004

replace regioncode="141123" if regioncode=="141123" & year==2004

replace regioncode="141124" if regioncode=="141124" & year==2004

replace regioncode="141125" if regioncode=="141125" & year==2004

replace regioncode="141126" if regioncode=="141126" & year==2004

replace regioncode="141127" if regioncode=="141127" & year==2004

replace regioncode="141128" if regioncode=="141128" & year==2004

replace regioncode="141129" if regioncode=="141129" & year==2004

replace regioncode="141130" if regioncode=="141130" & year==2004

replace regioncode="140702" if regioncode=="140702" & year==2004

replace regioncode="140781" if regioncode=="140781" & year==2004

replace regioncode="140721" if regioncode=="140721" & year==2004

replace regioncode="140722" if regioncode=="140722" & year==2004

replace regioncode="140723" if regioncode=="140723" & year==2004

replace regioncode="140724" if regioncode=="140724" & year==2004

replace regioncode="140725" if regioncode=="140725" & year==2004

replace regioncode="140726" if regioncode=="140726" & year==2004

replace regioncode="140727" if regioncode=="140727" & year==2004

replace regioncode="140728" if regioncode=="140728" & year==2004

replace regioncode="140729" if regioncode=="140729" & year==2004

replace regioncode="141002" if regioncode=="141002" & year==2004

replace regioncode="141081" if regioncode=="141081" & year==2004

replace regioncode="141082" if regioncode=="141082" & year==2004

replace regioncode="141021" if regioncode=="141021" & year==2004

replace regioncode="141022" if regioncode=="141022" & year==2004

replace regioncode="141023" if regioncode=="141023" & year==2004

replace regioncode="141024" if regioncode=="141024" & year==2004

replace regioncode="141025" if regioncode=="141025" & year==2004

replace regioncode="141026" if regioncode=="141026" & year==2004

replace regioncode="141027" if regioncode=="141027" & year==2004

replace regioncode="141028" if regioncode=="141028" & year==2004

replace regioncode="141029" if regioncode=="141029" & year==2004

replace regioncode="141033" if regioncode=="141033" & year==2004

replace regioncode="141030" if regioncode=="141030" & year==2004

replace regioncode="141032" if regioncode=="141032" & year==2004

replace regioncode="141031" if regioncode=="141031" & year==2004

replace regioncode="141034" if regioncode=="141034" & year==2004

replace regioncode="140802" if regioncode=="140802" & year==2004

replace regioncode="140881" if regioncode=="140881" & year==2004

replace regioncode="140882" if regioncode=="140882" & year==2004

replace regioncode="140830" if regioncode=="140830" & year==2004

replace regioncode="140821" if regioncode=="140821" & year==2004

replace regioncode="140822" if regioncode=="140822" & year==2004

replace regioncode="140825" if regioncode=="140825" & year==2004

replace regioncode="140824" if regioncode=="140824" & year==2004

replace regioncode="140823" if regioncode=="140823" & year==2004

replace regioncode="140828" if regioncode=="140828" & year==2004

replace regioncode="140826" if regioncode=="140826" & year==2004

replace regioncode="140829" if regioncode=="140829" & year==2004

replace regioncode="140827" if regioncode=="140827" & year==2004

replace regioncode="150102" if regioncode=="150102" & year==2004

replace regioncode="150103" if regioncode=="150103" & year==2004

replace regioncode="150104" if regioncode=="150104" & year==2004

replace regioncode="150105" if regioncode=="150105" & year==2004

replace regioncode="150121" if regioncode=="150121" & year==2004

replace regioncode="150122" if regioncode=="150122" & year==2004

replace regioncode="150123" if regioncode=="150123" & year==2004

replace regioncode="150124" if regioncode=="150124" & year==2004

replace regioncode="150125" if regioncode=="150125" & year==2004

replace regioncode="150202" if regioncode=="150202" & year==2004

replace regioncode="150203" if regioncode=="150203" & year==2004

replace regioncode="150204" if regioncode=="150204" & year==2004

replace regioncode="150205" if regioncode=="150205" & year==2004

replace regioncode="150206" if regioncode=="150206" & year==2004

replace regioncode="150207" if regioncode=="150207" & year==2004

replace regioncode="150221" if regioncode=="150221" & year==2004

replace regioncode="150222" if regioncode=="150222" & year==2004

replace regioncode="150223" if regioncode=="150223" & year==2004

replace regioncode="150302" if regioncode=="150302" & year==2004

replace regioncode="150303" if regioncode=="150303" & year==2004

replace regioncode="150304" if regioncode=="150304" & year==2004

replace regioncode="150402" if regioncode=="150402" & year==2004

replace regioncode="150403" if regioncode=="150403" & year==2004

replace regioncode="150404" if regioncode=="150404" & year==2004

replace regioncode="150421" if regioncode=="150421" & year==2004

replace regioncode="150422" if regioncode=="150422" & year==2004

replace regioncode="150423" if regioncode=="150423" & year==2004

replace regioncode="150424" if regioncode=="150424" & year==2004

replace regioncode="150425" if regioncode=="150425" & year==2004

replace regioncode="150426" if regioncode=="150426" & year==2004

replace regioncode="150428" if regioncode=="150428" & year==2004

replace regioncode="150429" if regioncode=="150429" & year==2004

replace regioncode="150430" if regioncode=="150430" & year==2004

replace regioncode="150702" if regioncode=="150702" & year==2004

replace regioncode="150781" if regioncode=="150781" & year==2004

replace regioncode="150783" if regioncode=="150783" & year==2004

replace regioncode="150782" if regioncode=="150782" & year==2004

replace regioncode="150785" if regioncode=="150785" & year==2004

replace regioncode="150784" if regioncode=="150784" & year==2004

replace regioncode="150721" if regioncode=="150721" & year==2004

replace regioncode="150722" if regioncode=="150722" & year==2004

replace regioncode="150723" if regioncode=="150723" & year==2004

replace regioncode="150724" if regioncode=="150724" & year==2004

replace regioncode="150727" if regioncode=="150727" & year==2004

replace regioncode="150726" if regioncode=="150726" & year==2004

replace regioncode="150725" if regioncode=="150725" & year==2004

replace regioncode="152201" if regioncode=="152201" & year==2004

replace regioncode="152202" if regioncode=="152202" & year==2004

replace regioncode="152221" if regioncode=="152221" & year==2004

replace regioncode="152222" if regioncode=="152222" & year==2004

replace regioncode="152223" if regioncode=="152223" & year==2004

replace regioncode="152224" if regioncode=="152224" & year==2004

replace regioncode="150502" if regioncode=="150502" & year==2004

replace regioncode="150581" if regioncode=="150581" & year==2004

replace regioncode="150521" if regioncode=="150521" & year==2004

replace regioncode="150522" if regioncode=="150522" & year==2004

replace regioncode="150523" if regioncode=="150523" & year==2004

replace regioncode="150524" if regioncode=="150524" & year==2004

replace regioncode="150525" if regioncode=="150525" & year==2004

replace regioncode="150526" if regioncode=="150526" & year==2004

replace regioncode="152501" if regioncode=="152501" & year==2004

replace regioncode="152502" if regioncode=="152502" & year==2004

replace regioncode="152522" if regioncode=="152522" & year==2004

replace regioncode="152523" if regioncode=="152523" & year==2004

replace regioncode="152524" if regioncode=="152524" & year==2004

replace regioncode="152525" if regioncode=="152525" & year==2004

replace regioncode="152526" if regioncode=="152526" & year==2004

replace regioncode="152527" if regioncode=="152527" & year==2004

replace regioncode="152528" if regioncode=="152528" & year==2004

replace regioncode="152529" if regioncode=="152529" & year==2004

replace regioncode="152530" if regioncode=="152530" & year==2004

replace regioncode="152531" if regioncode=="152531" & year==2004

replace regioncode="152601" if regioncode=="150902" & year==2004

replace regioncode="152602" if regioncode=="150981" & year==2004

replace regioncode="152624" if regioncode=="150921" & year==2004

replace regioncode="152625" if regioncode=="150922" & year==2004

replace regioncode="152626" if regioncode=="150923" & year==2004

replace regioncode="152627" if regioncode=="150924" & year==2004

replace regioncode="152629" if regioncode=="150925" & year==2004

replace regioncode="152630" if regioncode=="150926" & year==2004

replace regioncode="152631" if regioncode=="150927" & year==2004

replace regioncode="152632" if regioncode=="150928" & year==2004

replace regioncode="152634" if regioncode=="150929" & year==2004

replace regioncode="150602" if regioncode=="150602" & year==2004

replace regioncode="150621" if regioncode=="150621" & year==2004

replace regioncode="150622" if regioncode=="150622" & year==2004

replace regioncode="150623" if regioncode=="150623" & year==2004

replace regioncode="150624" if regioncode=="150624" & year==2004

replace regioncode="150625" if regioncode=="150625" & year==2004

replace regioncode="150626" if regioncode=="150626" & year==2004

replace regioncode="150627" if regioncode=="150627" & year==2004

replace regioncode="152801" if regioncode=="150802" & year==2004

replace regioncode="152822" if regioncode=="150821" & year==2004

replace regioncode="152823" if regioncode=="150822" & year==2004

replace regioncode="152824" if regioncode=="150823" & year==2004

replace regioncode="152825" if regioncode=="150824" & year==2004

replace regioncode="152826" if regioncode=="150825" & year==2004

replace regioncode="152827" if regioncode=="150826" & year==2004

replace regioncode="152921" if regioncode=="152921" & year==2004

replace regioncode="152922" if regioncode=="152922" & year==2004

replace regioncode="152923" if regioncode=="152923" & year==2004

replace regioncode="210102" if regioncode=="210102" & year==2004

replace regioncode="210103" if regioncode=="210103" & year==2004

replace regioncode="210104" if regioncode=="210104" & year==2004

replace regioncode="210105" if regioncode=="210105" & year==2004

replace regioncode="210106" if regioncode=="210106" & year==2004

replace regioncode="210111" if regioncode=="210111" & year==2004

replace regioncode="210112" if regioncode=="210112" & year==2004

replace regioncode="210113" if regioncode=="210113" & year==2004

replace regioncode="210114" if regioncode=="210114" & year==2004

replace regioncode="210122" if regioncode=="210122" & year==2004

replace regioncode="210123" if regioncode=="210123" & year==2004

replace regioncode="210124" if regioncode=="210124" & year==2004

replace regioncode="210181" if regioncode=="210181" & year==2004

replace regioncode="210202" if regioncode=="210202" & year==2004

replace regioncode="210203" if regioncode=="210203" & year==2004

replace regioncode="210204" if regioncode=="210204" & year==2004

replace regioncode="210211" if regioncode=="210211" & year==2004

replace regioncode="210212" if regioncode=="210212" & year==2004

replace regioncode="210213" if regioncode=="210213" & year==2004

replace regioncode="210224" if regioncode=="210224" & year==2004

replace regioncode="210281" if regioncode=="210281" & year==2004

replace regioncode="210282" if regioncode=="210282" & year==2004

replace regioncode="210283" if regioncode=="210283" & year==2004

replace regioncode="210302" if regioncode=="210302" & year==2004

replace regioncode="210303" if regioncode=="210303" & year==2004

replace regioncode="210304" if regioncode=="210304" & year==2004

replace regioncode="210311" if regioncode=="210311" & year==2004

replace regioncode="210321" if regioncode=="210321" & year==2004

replace regioncode="210323" if regioncode=="210323" & year==2004

replace regioncode="210381" if regioncode=="210381" & year==2004

replace regioncode="210402" if regioncode=="210402" & year==2004

replace regioncode="210403" if regioncode=="210403" & year==2004

replace regioncode="210404" if regioncode=="210404" & year==2004

replace regioncode="210411" if regioncode=="210411" & year==2004

replace regioncode="210421" if regioncode=="210421" & year==2004

replace regioncode="210422" if regioncode=="210422" & year==2004

replace regioncode="210423" if regioncode=="210423" & year==2004

replace regioncode="210502" if regioncode=="210502" & year==2004

replace regioncode="210503" if regioncode=="210503" & year==2004

replace regioncode="210504" if regioncode=="210504" & year==2004

replace regioncode="210505" if regioncode=="210505" & year==2004

replace regioncode="210521" if regioncode=="210521" & year==2004

replace regioncode="210522" if regioncode=="210522" & year==2004

replace regioncode="210602" if regioncode=="210602" & year==2004

replace regioncode="210603" if regioncode=="210603" & year==2004

replace regioncode="210604" if regioncode=="210604" & year==2004

replace regioncode="210624" if regioncode=="210624" & year==2004

replace regioncode="210681" if regioncode=="210681" & year==2004

replace regioncode="210682" if regioncode=="210682" & year==2004

replace regioncode="210702" if regioncode=="210702" & year==2004

replace regioncode="210703" if regioncode=="210703" & year==2004

replace regioncode="210711" if regioncode=="210711" & year==2004

replace regioncode="210726" if regioncode=="210726" & year==2004

replace regioncode="210727" if regioncode=="210727" & year==2004

replace regioncode="210781" if regioncode=="210781" & year==2004

replace regioncode="210782" if regioncode=="210782" & year==2004

replace regioncode="210802" if regioncode=="210802" & year==2004

replace regioncode="210803" if regioncode=="210803" & year==2004

replace regioncode="210804" if regioncode=="210804" & year==2004

replace regioncode="210811" if regioncode=="210811" & year==2004

replace regioncode="210881" if regioncode=="210881" & year==2004

replace regioncode="210882" if regioncode=="210882" & year==2004

replace regioncode="210902" if regioncode=="210902" & year==2004

replace regioncode="210903" if regioncode=="210903" & year==2004

replace regioncode="210904" if regioncode=="210904" & year==2004

replace regioncode="210905" if regioncode=="210905" & year==2004

replace regioncode="210911" if regioncode=="210911" & year==2004

replace regioncode="210921" if regioncode=="210921" & year==2004

replace regioncode="210922" if regioncode=="210922" & year==2004

replace regioncode="211002" if regioncode=="211002" & year==2004

replace regioncode="211003" if regioncode=="211003" & year==2004

replace regioncode="211004" if regioncode=="211004" & year==2004

replace regioncode="211005" if regioncode=="211005" & year==2004

replace regioncode="211011" if regioncode=="211011" & year==2004

replace regioncode="211021" if regioncode=="211021" & year==2004

replace regioncode="211081" if regioncode=="211081" & year==2004

replace regioncode="211102" if regioncode=="211102" & year==2004

replace regioncode="211103" if regioncode=="211103" & year==2004

replace regioncode="211121" if regioncode=="211121" & year==2004

replace regioncode="211122" if regioncode=="211122" & year==2004

replace regioncode="211202" if regioncode=="211202" & year==2004

replace regioncode="211204" if regioncode=="211204" & year==2004

replace regioncode="211221" if regioncode=="211221" & year==2004

replace regioncode="211223" if regioncode=="211223" & year==2004

replace regioncode="211224" if regioncode=="211224" & year==2004

replace regioncode="211281" if regioncode=="211281" & year==2004

replace regioncode="211282" if regioncode=="211282" & year==2004

replace regioncode="211302" if regioncode=="211302" & year==2004

replace regioncode="211303" if regioncode=="211303" & year==2004

replace regioncode="211321" if regioncode=="211321" & year==2004

replace regioncode="211322" if regioncode=="211322" & year==2004

replace regioncode="211324" if regioncode=="211324" & year==2004

replace regioncode="211381" if regioncode=="211381" & year==2004

replace regioncode="211382" if regioncode=="211382" & year==2004

replace regioncode="211402" if regioncode=="211402" & year==2004

replace regioncode="211403" if regioncode=="211403" & year==2004

replace regioncode="211404" if regioncode=="211404" & year==2004

replace regioncode="211421" if regioncode=="211421" & year==2004

replace regioncode="211422" if regioncode=="211422" & year==2004

replace regioncode="211481" if regioncode=="211481" & year==2004

replace regioncode="220102" if regioncode=="220102" & year==2004

replace regioncode="220103" if regioncode=="220103" & year==2004

replace regioncode="220104" if regioncode=="220104" & year==2004

replace regioncode="220105" if regioncode=="220105" & year==2004

replace regioncode="220106" if regioncode=="220106" & year==2004

replace regioncode="220112" if regioncode=="220112" & year==2004

replace regioncode="220122" if regioncode=="220122" & year==2004

replace regioncode="220181" if regioncode=="220181" & year==2004

replace regioncode="220182" if regioncode=="220182" & year==2004

replace regioncode="220183" if regioncode=="220183" & year==2004

replace regioncode="220202" if regioncode=="220202" & year==2004

replace regioncode="220203" if regioncode=="220203" & year==2004

replace regioncode="220204" if regioncode=="220204" & year==2004

replace regioncode="220211" if regioncode=="220211" & year==2004

replace regioncode="220221" if regioncode=="220221" & year==2004

replace regioncode="220281" if regioncode=="220281" & year==2004

replace regioncode="220282" if regioncode=="220282" & year==2004

replace regioncode="220283" if regioncode=="220283" & year==2004

replace regioncode="220284" if regioncode=="220284" & year==2004

replace regioncode="220302" if regioncode=="220302" & year==2004

replace regioncode="220303" if regioncode=="220303" & year==2004

replace regioncode="220322" if regioncode=="220322" & year==2004

replace regioncode="220323" if regioncode=="220323" & year==2004

replace regioncode="220381" if regioncode=="220381" & year==2004

replace regioncode="220382" if regioncode=="220382" & year==2004

replace regioncode="220402" if regioncode=="220402" & year==2004

replace regioncode="220403" if regioncode=="220403" & year==2004

replace regioncode="220421" if regioncode=="220421" & year==2004

replace regioncode="220422" if regioncode=="220422" & year==2004

replace regioncode="220502" if regioncode=="220502" & year==2004

replace regioncode="220503" if regioncode=="220503" & year==2004

replace regioncode="220521" if regioncode=="220521" & year==2004

replace regioncode="220523" if regioncode=="220523" & year==2004

replace regioncode="220524" if regioncode=="220524" & year==2004

replace regioncode="220581" if regioncode=="220581" & year==2004

replace regioncode="220582" if regioncode=="220582" & year==2004

replace regioncode="220602" if regioncode=="220602" & year==2004

replace regioncode="220621" if regioncode=="220621" & year==2004

replace regioncode="220622" if regioncode=="220622" & year==2004

replace regioncode="220623" if regioncode=="220623" & year==2004

replace regioncode="220625" if regioncode=="220625" & year==2004

replace regioncode="220681" if regioncode=="220681" & year==2004

replace regioncode="220702" if regioncode=="220702" & year==2004

replace regioncode="220721" if regioncode=="220721" & year==2004

replace regioncode="220722" if regioncode=="220722" & year==2004

replace regioncode="220723" if regioncode=="220723" & year==2004

replace regioncode="220724" if regioncode=="220724" & year==2004

replace regioncode="220802" if regioncode=="220802" & year==2004

replace regioncode="220821" if regioncode=="220821" & year==2004

replace regioncode="220822" if regioncode=="220822" & year==2004

replace regioncode="220881" if regioncode=="220881" & year==2004

replace regioncode="220882" if regioncode=="220882" & year==2004

replace regioncode="222401" if regioncode=="222401" & year==2004

replace regioncode="222402" if regioncode=="222402" & year==2004

replace regioncode="222403" if regioncode=="222403" & year==2004

replace regioncode="222404" if regioncode=="222404" & year==2004

replace regioncode="222405" if regioncode=="222405" & year==2004

replace regioncode="222406" if regioncode=="222406" & year==2004

replace regioncode="222424" if regioncode=="222424" & year==2004

replace regioncode="222426" if regioncode=="222426" & year==2004

replace regioncode="230102" if regioncode=="230102" & year==2004

replace regioncode="230103" if regioncode=="230103" & year==2004

replace regioncode="230104" if regioncode=="230104" & year==2004

replace regioncode="230105" if regioncode=="230105" & year==2004

replace regioncode="230107" if regioncode=="230107" & year==2004

replace regioncode="230108" if regioncode=="230108" & year==2004

replace regioncode="230121" if regioncode=="230111" & year==2004

replace regioncode="230123" if regioncode=="230123" & year==2004

replace regioncode="230124" if regioncode=="230124" & year==2004

replace regioncode="230125" if regioncode=="230125" & year==2004

replace regioncode="230126" if regioncode=="230126" & year==2004

replace regioncode="230127" if regioncode=="230127" & year==2004

replace regioncode="230128" if regioncode=="230128" & year==2004

replace regioncode="230129" if regioncode=="230129" & year==2004

replace regioncode="230181" if regioncode=="230181" & year==2004

replace regioncode="230182" if regioncode=="230182" & year==2004

replace regioncode="230183" if regioncode=="230183" & year==2004

replace regioncode="230184" if regioncode=="230184" & year==2004

replace regioncode="230202" if regioncode=="230202" & year==2004

replace regioncode="230203" if regioncode=="230203" & year==2004

replace regioncode="230204" if regioncode=="230204" & year==2004

replace regioncode="230205" if regioncode=="230205" & year==2004

replace regioncode="230206" if regioncode=="230206" & year==2004

replace regioncode="230207" if regioncode=="230207" & year==2004

replace regioncode="230208" if regioncode=="230208" & year==2004

replace regioncode="230221" if regioncode=="230221" & year==2004

replace regioncode="230223" if regioncode=="230223" & year==2004

replace regioncode="230224" if regioncode=="230224" & year==2004

replace regioncode="230225" if regioncode=="230225" & year==2004

replace regioncode="230227" if regioncode=="230227" & year==2004

replace regioncode="230229" if regioncode=="230229" & year==2004

replace regioncode="230230" if regioncode=="230230" & year==2004

replace regioncode="230231" if regioncode=="230231" & year==2004

replace regioncode="230281" if regioncode=="230281" & year==2004

replace regioncode="230302" if regioncode=="230302" & year==2004

replace regioncode="230303" if regioncode=="230303" & year==2004

replace regioncode="230304" if regioncode=="230304" & year==2004

replace regioncode="230305" if regioncode=="230305" & year==2004

replace regioncode="230306" if regioncode=="230306" & year==2004

replace regioncode="230307" if regioncode=="230307" & year==2004

replace regioncode="230321" if regioncode=="230321" & year==2004

replace regioncode="230381" if regioncode=="230381" & year==2004

replace regioncode="230382" if regioncode=="230382" & year==2004

replace regioncode="230402" if regioncode=="230402" & year==2004

replace regioncode="230403" if regioncode=="230403" & year==2004

replace regioncode="230404" if regioncode=="230404" & year==2004

replace regioncode="230405" if regioncode=="230405" & year==2004

replace regioncode="230406" if regioncode=="230406" & year==2004

replace regioncode="230407" if regioncode=="230407" & year==2004

replace regioncode="230421" if regioncode=="230421" & year==2004

replace regioncode="230422" if regioncode=="230422" & year==2004

replace regioncode="230502" if regioncode=="230502" & year==2004

replace regioncode="230503" if regioncode=="230503" & year==2004

replace regioncode="230505" if regioncode=="230505" & year==2004

replace regioncode="230506" if regioncode=="230506" & year==2004

replace regioncode="230521" if regioncode=="230521" & year==2004

replace regioncode="230522" if regioncode=="230522" & year==2004

replace regioncode="230523" if regioncode=="230523" & year==2004

replace regioncode="230524" if regioncode=="230524" & year==2004

replace regioncode="230602" if regioncode=="230602" & year==2004

replace regioncode="230603" if regioncode=="230603" & year==2004

replace regioncode="230604" if regioncode=="230604" & year==2004

replace regioncode="230605" if regioncode=="230605" & year==2004

replace regioncode="230606" if regioncode=="230606" & year==2004

replace regioncode="230621" if regioncode=="230621" & year==2004

replace regioncode="230622" if regioncode=="230622" & year==2004

replace regioncode="230623" if regioncode=="230623" & year==2004

replace regioncode="230624" if regioncode=="230624" & year==2004

replace regioncode="230702" if regioncode=="230702" & year==2004

replace regioncode="230703" if regioncode=="230703" & year==2004

replace regioncode="230704" if regioncode=="230704" & year==2004

replace regioncode="230705" if regioncode=="230705" & year==2004

replace regioncode="230706" if regioncode=="230706" & year==2004

replace regioncode="230707" if regioncode=="230707" & year==2004

replace regioncode="230708" if regioncode=="230708" & year==2004

replace regioncode="230709" if regioncode=="230709" & year==2004

replace regioncode="230710" if regioncode=="230710" & year==2004

replace regioncode="230711" if regioncode=="230711" & year==2004

replace regioncode="230712" if regioncode=="230712" & year==2004

replace regioncode="230713" if regioncode=="230713" & year==2004

replace regioncode="230714" if regioncode=="230714" & year==2004

replace regioncode="230715" if regioncode=="230715" & year==2004

replace regioncode="230716" if regioncode=="230716" & year==2004

replace regioncode="230722" if regioncode=="230722" & year==2004

replace regioncode="230781" if regioncode=="230781" & year==2004

replace regioncode="230802" if regioncode=="230802" & year==2004

replace regioncode="230803" if regioncode=="230803" & year==2004

replace regioncode="230804" if regioncode=="230804" & year==2004

replace regioncode="230805" if regioncode=="230805" & year==2004

replace regioncode="230811" if regioncode=="230811" & year==2004

replace regioncode="230822" if regioncode=="230822" & year==2004

replace regioncode="230826" if regioncode=="230826" & year==2004

replace regioncode="230828" if regioncode=="230828" & year==2004

replace regioncode="230833" if regioncode=="230833" & year==2004

replace regioncode="230881" if regioncode=="230881" & year==2004

replace regioncode="230882" if regioncode=="230882" & year==2004

replace regioncode="230902" if regioncode=="230902" & year==2004

replace regioncode="230903" if regioncode=="230903" & year==2004

replace regioncode="230904" if regioncode=="230904" & year==2004

replace regioncode="230921" if regioncode=="230921" & year==2004

replace regioncode="231002" if regioncode=="231002" & year==2004

replace regioncode="231003" if regioncode=="231003" & year==2004

replace regioncode="231004" if regioncode=="231004" & year==2004

replace regioncode="231005" if regioncode=="231005" & year==2004

replace regioncode="231024" if regioncode=="231024" & year==2004

replace regioncode="231025" if regioncode=="231025" & year==2004

replace regioncode="231081" if regioncode=="231081" & year==2004

replace regioncode="231083" if regioncode=="231083" & year==2004

replace regioncode="231084" if regioncode=="231084" & year==2004

replace regioncode="231085" if regioncode=="231085" & year==2004

replace regioncode="231102" if regioncode=="231102" & year==2004

replace regioncode="231121" if regioncode=="231121" & year==2004

replace regioncode="231123" if regioncode=="231123" & year==2004

replace regioncode="231124" if regioncode=="231124" & year==2004

replace regioncode="231181" if regioncode=="231181" & year==2004

replace regioncode="231182" if regioncode=="231182" & year==2004

replace regioncode="231202" if regioncode=="231202" & year==2004

replace regioncode="231221" if regioncode=="231221" & year==2004

replace regioncode="231222" if regioncode=="231222" & year==2004

replace regioncode="231223" if regioncode=="231223" & year==2004

replace regioncode="231224" if regioncode=="231224" & year==2004

replace regioncode="231225" if regioncode=="231225" & year==2004

replace regioncode="231226" if regioncode=="231226" & year==2004

replace regioncode="231281" if regioncode=="231281" & year==2004

replace regioncode="231282" if regioncode=="231282" & year==2004

replace regioncode="231283" if regioncode=="231283" & year==2004

replace regioncode="232701" if regioncode=="232701" & year==2004

replace regioncode="232702" if regioncode=="232702" & year==2004

replace regioncode="232703" if regioncode=="232703" & year==2004

replace regioncode="232704" if regioncode=="232704" & year==2004

replace regioncode="232721" if regioncode=="232721" & year==2004

replace regioncode="232722" if regioncode=="232722" & year==2004

replace regioncode="232723" if regioncode=="232723" & year==2004

replace regioncode="310101" if regioncode=="310101" & year==2004

replace regioncode="310103" if regioncode=="310103" & year==2004

replace regioncode="310104" if regioncode=="310104" & year==2004

replace regioncode="310105" if regioncode=="310105" & year==2004

replace regioncode="310106" if regioncode=="310106" & year==2004

replace regioncode="310107" if regioncode=="310107" & year==2004

replace regioncode="310108" if regioncode=="310108" & year==2004

replace regioncode="310109" if regioncode=="310109" & year==2004

replace regioncode="310110" if regioncode=="310110" & year==2004

replace regioncode="310112" if regioncode=="310112" & year==2004

replace regioncode="310113" if regioncode=="310113" & year==2004

replace regioncode="310114" if regioncode=="310114" & year==2004

replace regioncode="310115" if regioncode=="310115" & year==2004

replace regioncode="310116" if regioncode=="310116" & year==2004

replace regioncode="310117" if regioncode=="310117" & year==2004

replace regioncode="310118" if regioncode=="310118" & year==2004

replace regioncode="310119" if regioncode=="310119" & year==2004

replace regioncode="310120" if regioncode=="310120" & year==2004

replace regioncode="310230" if regioncode=="310230" & year==2004

replace regioncode="320102" if regioncode=="320102" & year==2004

replace regioncode="320103" if regioncode=="320103" & year==2004

replace regioncode="320104" if regioncode=="320104" & year==2004

replace regioncode="320105" if regioncode=="320105" & year==2004

replace regioncode="320106" if regioncode=="320106" & year==2004

replace regioncode="320107" if regioncode=="320107" & year==2004

replace regioncode="320111" if regioncode=="320111" & year==2004

replace regioncode="320113" if regioncode=="320113" & year==2004

replace regioncode="320114" if regioncode=="320114" & year==2004

replace regioncode="320115" if regioncode=="320115" & year==2004

replace regioncode="320116" if regioncode=="320116" & year==2004

replace regioncode="320124" if regioncode=="320124" & year==2004

replace regioncode="320125" if regioncode=="320125" & year==2004

replace regioncode="320202" if regioncode=="320202" & year==2004

replace regioncode="320203" if regioncode=="320203" & year==2004

replace regioncode="320204" if regioncode=="320204" & year==2004

replace regioncode="320205" if regioncode=="320205" & year==2004

replace regioncode="320211" if regioncode=="320211" & year==2004

replace regioncode="320281" if regioncode=="320281" & year==2004

replace regioncode="320282" if regioncode=="320282" & year==2004

replace regioncode="320302" if regioncode=="320302" & year==2004

replace regioncode="320303" if regioncode=="320303" & year==2004

replace regioncode="320304" if regioncode=="320304" & year==2004

replace regioncode="320305" if regioncode=="320305" & year==2004

replace regioncode="320311" if regioncode=="320311" & year==2004

replace regioncode="320321" if regioncode=="320321" & year==2004

replace regioncode="320322" if regioncode=="320322" & year==2004

replace regioncode="320323" if regioncode=="320323" & year==2004

replace regioncode="320324" if regioncode=="320324" & year==2004

replace regioncode="320381" if regioncode=="320381" & year==2004

replace regioncode="320382" if regioncode=="320382" & year==2004

replace regioncode="320402" if regioncode=="320402" & year==2004

replace regioncode="320404" if regioncode=="320404" & year==2004

replace regioncode="320405" if regioncode=="320405" & year==2004

replace regioncode="320411" if regioncode=="320411" & year==2004

replace regioncode="320412" if regioncode=="320412" & year==2004

replace regioncode="320481" if regioncode=="320481" & year==2004

replace regioncode="320482" if regioncode=="320482" & year==2004

replace regioncode="320502" if regioncode=="320502" & year==2004

replace regioncode="320503" if regioncode=="320503" & year==2004

replace regioncode="320504" if regioncode=="320504" & year==2004

replace regioncode="320505" if regioncode=="320505" & year==2004

replace regioncode="320581" if regioncode=="320581" & year==2004

replace regioncode="320582" if regioncode=="320582" & year==2004

replace regioncode="320583" if regioncode=="320583" & year==2004

replace regioncode="320584" if regioncode=="320584" & year==2004

replace regioncode="320585" if regioncode=="320585" & year==2004

replace regioncode="320586" if regioncode=="320586" & year==2004

replace regioncode="320602" if regioncode=="320602" & year==2004

replace regioncode="320611" if regioncode=="320611" & year==2004

replace regioncode="320621" if regioncode=="320621" & year==2004

replace regioncode="320623" if regioncode=="320623" & year==2004

replace regioncode="320681" if regioncode=="320681" & year==2004

replace regioncode="320682" if regioncode=="320682" & year==2004

replace regioncode="320683" if regioncode=="320683" & year==2004

replace regioncode="320684" if regioncode=="320684" & year==2004

replace regioncode="320703" if regioncode=="320703" & year==2004

replace regioncode="320705" if regioncode=="320705" & year==2004

replace regioncode="320706" if regioncode=="320706" & year==2004

replace regioncode="320721" if regioncode=="320721" & year==2004

replace regioncode="320722" if regioncode=="320722" & year==2004

replace regioncode="320723" if regioncode=="320723" & year==2004

replace regioncode="320724" if regioncode=="320724" & year==2004

replace regioncode="320802" if regioncode=="320802" & year==2004

replace regioncode="320803" if regioncode=="320803" & year==2004

replace regioncode="320804" if regioncode=="320804" & year==2004

replace regioncode="320811" if regioncode=="320811" & year==2004

replace regioncode="320826" if regioncode=="320826" & year==2004

replace regioncode="320829" if regioncode=="320829" & year==2004

replace regioncode="320830" if regioncode=="320830" & year==2004

replace regioncode="320831" if regioncode=="320831" & year==2004

replace regioncode="320902" if regioncode=="320902" & year==2004

replace regioncode="320903" if regioncode=="320903" & year==2004

replace regioncode="320921" if regioncode=="320921" & year==2004

replace regioncode="320922" if regioncode=="320922" & year==2004

replace regioncode="320923" if regioncode=="320923" & year==2004

replace regioncode="320924" if regioncode=="320924" & year==2004

replace regioncode="320925" if regioncode=="320925" & year==2004

replace regioncode="320981" if regioncode=="320981" & year==2004

replace regioncode="320982" if regioncode=="320982" & year==2004

replace regioncode="321002" if regioncode=="321002" & year==2004

replace regioncode="321003" if regioncode=="321003" & year==2004

replace regioncode="321011" if regioncode=="321011" & year==2004

replace regioncode="321023" if regioncode=="321023" & year==2004

replace regioncode="321081" if regioncode=="321081" & year==2004

replace regioncode="321084" if regioncode=="321084" & year==2004

replace regioncode="321088" if regioncode=="321088" & year==2004

replace regioncode="321102" if regioncode=="321102" & year==2004

replace regioncode="321111" if regioncode=="321111" & year==2004

replace regioncode="321112" if regioncode=="321112" & year==2004

replace regioncode="321181" if regioncode=="321181" & year==2004

replace regioncode="321182" if regioncode=="321182" & year==2004

replace regioncode="321183" if regioncode=="321183" & year==2004

replace regioncode="321202" if regioncode=="321202" & year==2004

replace regioncode="321203" if regioncode=="321203" & year==2004

replace regioncode="321281" if regioncode=="321281" & year==2004

replace regioncode="321282" if regioncode=="321282" & year==2004

replace regioncode="321283" if regioncode=="321283" & year==2004

replace regioncode="321284" if regioncode=="321284" & year==2004

replace regioncode="321302" if regioncode=="321302" & year==2004

replace regioncode="321321" if regioncode=="321311" & year==2004

replace regioncode="321322" if regioncode=="321322" & year==2004

replace regioncode="321323" if regioncode=="321323" & year==2004

replace regioncode="321324" if regioncode=="321324" & year==2004

replace regioncode="330102" if regioncode=="330102" & year==2004

replace regioncode="330103" if regioncode=="330103" & year==2004

replace regioncode="330104" if regioncode=="330104" & year==2004

replace regioncode="330105" if regioncode=="330105" & year==2004

replace regioncode="330106" if regioncode=="330106" & year==2004

replace regioncode="330108" if regioncode=="330108" & year==2004

replace regioncode="330109" if regioncode=="330109" & year==2004

replace regioncode="330110" if regioncode=="330110" & year==2004

replace regioncode="330122" if regioncode=="330122" & year==2004

replace regioncode="330127" if regioncode=="330127" & year==2004

replace regioncode="330182" if regioncode=="330182" & year==2004

replace regioncode="330183" if regioncode=="330183" & year==2004

replace regioncode="330185" if regioncode=="330185" & year==2004

replace regioncode="330203" if regioncode=="330203" & year==2004

replace regioncode="330204" if regioncode=="330204" & year==2004

replace regioncode="330205" if regioncode=="330205" & year==2004

replace regioncode="330206" if regioncode=="330206" & year==2004

replace regioncode="330211" if regioncode=="330211" & year==2004

replace regioncode="330212" if regioncode=="330212" & year==2004

replace regioncode="330225" if regioncode=="330225" & year==2004

replace regioncode="330226" if regioncode=="330226" & year==2004

replace regioncode="330281" if regioncode=="330281" & year==2004

replace regioncode="330282" if regioncode=="330282" & year==2004

replace regioncode="330283" if regioncode=="330283" & year==2004

replace regioncode="330302" if regioncode=="330302" & year==2004

replace regioncode="330303" if regioncode=="330303" & year==2004

replace regioncode="330304" if regioncode=="330304" & year==2004

replace regioncode="330322" if regioncode=="330322" & year==2004

replace regioncode="330324" if regioncode=="330324" & year==2004

replace regioncode="330326" if regioncode=="330326" & year==2004

replace regioncode="330327" if regioncode=="330327" & year==2004

replace regioncode="330328" if regioncode=="330328" & year==2004

replace regioncode="330329" if regioncode=="330329" & year==2004

replace regioncode="330381" if regioncode=="330381" & year==2004

replace regioncode="330382" if regioncode=="330382" & year==2004

replace regioncode="330402" if regioncode=="330402" & year==2004

replace regioncode="330411" if regioncode=="330411" & year==2004

replace regioncode="330421" if regioncode=="330421" & year==2004

replace regioncode="330424" if regioncode=="330424" & year==2004

replace regioncode="330481" if regioncode=="330481" & year==2004

replace regioncode="330482" if regioncode=="330482" & year==2004

replace regioncode="330483" if regioncode=="330483" & year==2004

replace regioncode="330501" if regioncode=="330501" & year==2004

replace regioncode="330521" if regioncode=="330521" & year==2004

replace regioncode="330522" if regioncode=="330522" & year==2004

replace regioncode="330523" if regioncode=="330523" & year==2004

replace regioncode="330602" if regioncode=="330602" & year==2004

replace regioncode="330621" if regioncode=="330621" & year==2004

replace regioncode="330624" if regioncode=="330624" & year==2004

replace regioncode="330681" if regioncode=="330681" & year==2004

replace regioncode="330682" if regioncode=="330682" & year==2004

replace regioncode="330683" if regioncode=="330683" & year==2004

replace regioncode="330702" if regioncode=="330702" & year==2004

replace regioncode="330703" if regioncode=="330703" & year==2004

replace regioncode="330723" if regioncode=="330723" & year==2004

replace regioncode="330726" if regioncode=="330726" & year==2004

replace regioncode="330727" if regioncode=="330727" & year==2004

replace regioncode="330781" if regioncode=="330781" & year==2004

replace regioncode="330782" if regioncode=="330782" & year==2004

replace regioncode="330783" if regioncode=="330783" & year==2004

replace regioncode="330784" if regioncode=="330784" & year==2004

replace regioncode="330802" if regioncode=="330802" & year==2004

replace regioncode="330803" if regioncode=="330803" & year==2004

replace regioncode="330822" if regioncode=="330822" & year==2004

replace regioncode="330824" if regioncode=="330824" & year==2004

replace regioncode="330825" if regioncode=="330825" & year==2004

replace regioncode="330881" if regioncode=="330881" & year==2004

replace regioncode="330902" if regioncode=="330902" & year==2004

replace regioncode="330903" if regioncode=="330903" & year==2004

replace regioncode="330921" if regioncode=="330921" & year==2004

replace regioncode="330922" if regioncode=="330922" & year==2004

replace regioncode="331002" if regioncode=="331002" & year==2004

replace regioncode="331003" if regioncode=="331003" & year==2004

replace regioncode="331004" if regioncode=="331004" & year==2004

replace regioncode="331021" if regioncode=="331021" & year==2004

replace regioncode="331022" if regioncode=="331022" & year==2004

replace regioncode="331023" if regioncode=="331023" & year==2004

replace regioncode="331024" if regioncode=="331024" & year==2004

replace regioncode="331081" if regioncode=="331081" & year==2004

replace regioncode="331082" if regioncode=="331082" & year==2004

replace regioncode="331102" if regioncode=="331102" & year==2004

replace regioncode="331121" if regioncode=="331121" & year==2004

replace regioncode="331122" if regioncode=="331122" & year==2004

replace regioncode="331123" if regioncode=="331123" & year==2004

replace regioncode="331124" if regioncode=="331124" & year==2004

replace regioncode="331125" if regioncode=="331125" & year==2004

replace regioncode="331126" if regioncode=="331126" & year==2004

replace regioncode="331127" if regioncode=="331127" & year==2004

replace regioncode="331181" if regioncode=="331181" & year==2004

replace regioncode="340102" if regioncode=="340102" & year==2004

replace regioncode="340103" if regioncode=="340103" & year==2004

replace regioncode="340104" if regioncode=="340104" & year==2004

replace regioncode="340111" if regioncode=="340111" & year==2004

replace regioncode="340121" if regioncode=="340121" & year==2004

replace regioncode="340122" if regioncode=="340122" & year==2004

replace regioncode="340123" if regioncode=="340123" & year==2004

replace regioncode="340202" if regioncode=="340202" & year==2004

replace regioncode="340203" if regioncode=="340203" & year==2004

replace regioncode="340204" if regioncode=="340204" & year==2004

replace regioncode="340207" if regioncode=="340207" & year==2004

replace regioncode="340221" if regioncode=="340221" & year==2004

replace regioncode="340222" if regioncode=="340222" & year==2004

replace regioncode="340223" if regioncode=="340223" & year==2004

replace regioncode="340302" if regioncode=="340302" & year==2004

replace regioncode="340303" if regioncode=="340303" & year==2004

replace regioncode="340304" if regioncode=="340304" & year==2004

replace regioncode="340311" if regioncode=="340311" & year==2004

replace regioncode="340321" if regioncode=="340321" & year==2004

replace regioncode="340322" if regioncode=="340322" & year==2004

replace regioncode="340323" if regioncode=="340323" & year==2004

replace regioncode="340402" if regioncode=="340402" & year==2004

replace regioncode="340403" if regioncode=="340403" & year==2004

replace regioncode="340404" if regioncode=="340404" & year==2004

replace regioncode="340405" if regioncode=="340405" & year==2004

replace regioncode="340406" if regioncode=="340406" & year==2004

replace regioncode="340421" if regioncode=="340421" & year==2004

replace regioncode="340502" if regioncode=="340502" & year==2004

replace regioncode="340503" if regioncode=="340503" & year==2004

replace regioncode="340504" if regioncode=="340504" & year==2004

replace regioncode="340521" if regioncode=="340521" & year==2004

replace regioncode="340602" if regioncode=="340602" & year==2004

replace regioncode="340603" if regioncode=="340603" & year==2004

replace regioncode="340604" if regioncode=="340604" & year==2004

replace regioncode="340621" if regioncode=="340621" & year==2004

replace regioncode="340702" if regioncode=="340702" & year==2004

replace regioncode="340703" if regioncode=="340703" & year==2004

replace regioncode="340711" if regioncode=="340711" & year==2004

replace regioncode="340721" if regioncode=="340721" & year==2004

replace regioncode="340802" if regioncode=="340802" & year==2004

replace regioncode="340803" if regioncode=="340803" & year==2004

replace regioncode="340811" if regioncode=="340811" & year==2004

replace regioncode="340822" if regioncode=="340822" & year==2004

replace regioncode="340823" if regioncode=="340823" & year==2004

replace regioncode="340824" if regioncode=="340824" & year==2004

replace regioncode="340825" if regioncode=="340825" & year==2004

replace regioncode="340826" if regioncode=="340826" & year==2004

replace regioncode="340827" if regioncode=="340827" & year==2004

replace regioncode="340828" if regioncode=="340828" & year==2004

replace regioncode="340881" if regioncode=="340881" & year==2004

replace regioncode="341002" if regioncode=="341002" & year==2004

replace regioncode="341003" if regioncode=="341003" & year==2004

replace regioncode="341004" if regioncode=="341004" & year==2004

replace regioncode="341021" if regioncode=="341021" & year==2004

replace regioncode="341022" if regioncode=="341022" & year==2004

replace regioncode="341023" if regioncode=="341023" & year==2004

replace regioncode="341024" if regioncode=="341024" & year==2004

replace regioncode="341102" if regioncode=="341102" & year==2004

replace regioncode="341103" if regioncode=="341103" & year==2004

replace regioncode="341122" if regioncode=="341122" & year==2004

replace regioncode="341124" if regioncode=="341124" & year==2004

replace regioncode="341125" if regioncode=="341125" & year==2004

replace regioncode="341126" if regioncode=="341126" & year==2004

replace regioncode="341181" if regioncode=="341181" & year==2004

replace regioncode="341182" if regioncode=="341182" & year==2004

replace regioncode="341202" if regioncode=="341202" & year==2004

replace regioncode="341203" if regioncode=="341203" & year==2004

replace regioncode="341204" if regioncode=="341204" & year==2004

replace regioncode="341221" if regioncode=="341221" & year==2004

replace regioncode="341222" if regioncode=="341222" & year==2004

replace regioncode="341621" if regioncode=="341621" & year==2004

replace regioncode="341622" if regioncode=="341622" & year==2004

replace regioncode="341225" if regioncode=="341225" & year==2004

replace regioncode="341226" if regioncode=="341226" & year==2004

replace regioncode="341623" if regioncode=="341623" & year==2004

replace regioncode="341282" if regioncode=="341282" & year==2004

replace regioncode="341302" if regioncode=="341302" & year==2004

replace regioncode="341321" if regioncode=="341321" & year==2004

replace regioncode="341322" if regioncode=="341322" & year==2004

replace regioncode="341323" if regioncode=="341323" & year==2004

replace regioncode="341324" if regioncode=="341324" & year==2004

replace regioncode="341402" if regioncode=="341402" & year==2004

replace regioncode="341421" if regioncode=="341421" & year==2004

replace regioncode="341422" if regioncode=="341422" & year==2004

replace regioncode="341423" if regioncode=="341423" & year==2004

replace regioncode="341424" if regioncode=="341424" & year==2004

replace regioncode="341521" if regioncode=="341521" & year==2004

replace regioncode="341522" if regioncode=="341522" & year==2004

replace regioncode="341523" if regioncode=="341523" & year==2004

replace regioncode="341524" if regioncode=="341524" & year==2004

replace regioncode="341525" if regioncode=="341525" & year==2004

replace regioncode="341802" if regioncode=="341802" & year==2004

replace regioncode="341881" if regioncode=="341881" & year==2004

replace regioncode="341821" if regioncode=="341821" & year==2004

replace regioncode="341822" if regioncode=="341822" & year==2004

replace regioncode="341823" if regioncode=="341823" & year==2004

replace regioncode="341825" if regioncode=="341825" & year==2004

replace regioncode="341824" if regioncode=="341824" & year==2004

replace regioncode="341702" if regioncode=="341702" & year==2004

replace regioncode="341721" if regioncode=="341721" & year==2004

replace regioncode="341722" if regioncode=="341722" & year==2004

replace regioncode="341723" if regioncode=="341723" & year==2004

replace regioncode="341502" if regioncode=="341502" & year==2004

replace regioncode="341602" if regioncode=="341602" & year==2004

replace regioncode="350102" if regioncode=="350102" & year==2004

replace regioncode="350103" if regioncode=="350103" & year==2004

replace regioncode="350104" if regioncode=="350104" & year==2004

replace regioncode="350105" if regioncode=="350105" & year==2004

replace regioncode="350111" if regioncode=="350111" & year==2004

replace regioncode="350121" if regioncode=="350121" & year==2004

replace regioncode="350122" if regioncode=="350122" & year==2004

replace regioncode="350123" if regioncode=="350123" & year==2004

replace regioncode="350124" if regioncode=="350124" & year==2004

replace regioncode="350125" if regioncode=="350125" & year==2004

replace regioncode="350128" if regioncode=="350128" & year==2004

replace regioncode="350181" if regioncode=="350181" & year==2004

replace regioncode="350182" if regioncode=="350182" & year==2004

replace regioncode="350203" if regioncode=="350203" & year==2004

replace regioncode="350205" if regioncode=="350205" & year==2004

replace regioncode="350206" if regioncode=="350206" & year==2004

replace regioncode="350211" if regioncode=="350211" & year==2004

replace regioncode="350212" if regioncode=="350212" & year==2004

replace regioncode="350213" if regioncode=="350213" & year==2004

replace regioncode="350302" if regioncode=="350302" & year==2004

replace regioncode="350303" if regioncode=="350303" & year==2004

replace regioncode="350321" if regioncode=="350321" & year==2004

replace regioncode="350322" if regioncode=="350322" & year==2004

replace regioncode="350402" if regioncode=="350402" & year==2004

replace regioncode="350403" if regioncode=="350403" & year==2004

replace regioncode="350421" if regioncode=="350421" & year==2004

replace regioncode="350423" if regioncode=="350423" & year==2004

replace regioncode="350424" if regioncode=="350424" & year==2004

replace regioncode="350425" if regioncode=="350425" & year==2004

replace regioncode="350426" if regioncode=="350426" & year==2004

replace regioncode="350427" if regioncode=="350427" & year==2004

replace regioncode="350428" if regioncode=="350428" & year==2004

replace regioncode="350429" if regioncode=="350429" & year==2004

replace regioncode="350430" if regioncode=="350430" & year==2004

replace regioncode="350481" if regioncode=="350481" & year==2004

replace regioncode="350502" if regioncode=="350502" & year==2004

replace regioncode="350503" if regioncode=="350503" & year==2004

replace regioncode="350504" if regioncode=="350504" & year==2004

replace regioncode="350505" if regioncode=="350505" & year==2004

replace regioncode="350521" if regioncode=="350521" & year==2004

replace regioncode="350524" if regioncode=="350524" & year==2004

replace regioncode="350525" if regioncode=="350525" & year==2004

replace regioncode="350526" if regioncode=="350526" & year==2004

replace regioncode="350581" if regioncode=="350581" & year==2004

replace regioncode="350582" if regioncode=="350582" & year==2004

replace regioncode="350583" if regioncode=="350583" & year==2004

replace regioncode="350602" if regioncode=="350602" & year==2004

replace regioncode="350603" if regioncode=="350603" & year==2004

replace regioncode="350622" if regioncode=="350622" & year==2004

replace regioncode="350623" if regioncode=="350623" & year==2004

replace regioncode="350624" if regioncode=="350624" & year==2004

replace regioncode="350625" if regioncode=="350625" & year==2004

replace regioncode="350626" if regioncode=="350626" & year==2004

replace regioncode="350627" if regioncode=="350627" & year==2004

replace regioncode="350628" if regioncode=="350628" & year==2004

replace regioncode="350629" if regioncode=="350629" & year==2004

replace regioncode="350681" if regioncode=="350681" & year==2004

replace regioncode="350702" if regioncode=="350702" & year==2004

replace regioncode="350721" if regioncode=="350721" & year==2004

replace regioncode="350722" if regioncode=="350722" & year==2004

replace regioncode="350723" if regioncode=="350723" & year==2004

replace regioncode="350724" if regioncode=="350724" & year==2004

replace regioncode="350725" if regioncode=="350725" & year==2004

replace regioncode="350781" if regioncode=="350781" & year==2004

replace regioncode="350782" if regioncode=="350782" & year==2004

replace regioncode="350783" if regioncode=="350783" & year==2004

replace regioncode="350784" if regioncode=="350784" & year==2004

replace regioncode="350802" if regioncode=="350802" & year==2004

replace regioncode="350821" if regioncode=="350821" & year==2004

replace regioncode="350822" if regioncode=="350822" & year==2004

replace regioncode="350823" if regioncode=="350823" & year==2004

replace regioncode="350824" if regioncode=="350824" & year==2004

replace regioncode="350825" if regioncode=="350825" & year==2004

replace regioncode="350881" if regioncode=="350881" & year==2004

replace regioncode="350902" if regioncode=="350902" & year==2004

replace regioncode="350981" if regioncode=="350981" & year==2004

replace regioncode="350982" if regioncode=="350982" & year==2004

replace regioncode="350921" if regioncode=="350921" & year==2004

replace regioncode="350922" if regioncode=="350922" & year==2004

replace regioncode="350923" if regioncode=="350923" & year==2004

replace regioncode="350924" if regioncode=="350924" & year==2004

replace regioncode="350925" if regioncode=="350925" & year==2004

replace regioncode="350926" if regioncode=="350926" & year==2004

replace regioncode="360102" if regioncode=="360102" & year==2004

replace regioncode="360103" if regioncode=="360103" & year==2004

replace regioncode="360104" if regioncode=="360104" & year==2004

replace regioncode="360105" if regioncode=="360105" & year==2004

replace regioncode="360111" if regioncode=="360111" & year==2004

replace regioncode="360121" if regioncode=="360121" & year==2004

replace regioncode="360122" if regioncode=="360122" & year==2004

replace regioncode="360123" if regioncode=="360123" & year==2004

replace regioncode="360124" if regioncode=="360124" & year==2004

replace regioncode="360202" if regioncode=="360202" & year==2004

replace regioncode="360203" if regioncode=="360203" & year==2004

replace regioncode="360222" if regioncode=="360222" & year==2004

replace regioncode="360281" if regioncode=="360281" & year==2004

replace regioncode="360302" if regioncode=="360302" & year==2004

replace regioncode="360313" if regioncode=="360313" & year==2004

replace regioncode="360321" if regioncode=="360321" & year==2004

replace regioncode="360322" if regioncode=="360322" & year==2004

replace regioncode="360323" if regioncode=="360323" & year==2004

replace regioncode="360402" if regioncode=="360402" & year==2004

replace regioncode="360403" if regioncode=="360403" & year==2004

replace regioncode="360421" if regioncode=="360421" & year==2004

replace regioncode="360423" if regioncode=="360423" & year==2004

replace regioncode="360424" if regioncode=="360424" & year==2004

replace regioncode="360425" if regioncode=="360425" & year==2004

replace regioncode="360426" if regioncode=="360426" & year==2004

replace regioncode="360427" if regioncode=="360427" & year==2004

replace regioncode="360428" if regioncode=="360428" & year==2004

replace regioncode="360429" if regioncode=="360429" & year==2004

replace regioncode="360430" if regioncode=="360430" & year==2004

replace regioncode="360481" if regioncode=="360481" & year==2004

replace regioncode="360502" if regioncode=="360502" & year==2004

replace regioncode="360521" if regioncode=="360521" & year==2004

replace regioncode="360602" if regioncode=="360602" & year==2004

replace regioncode="360622" if regioncode=="360622" & year==2004

replace regioncode="360681" if regioncode=="360681" & year==2004

replace regioncode="360702" if regioncode=="360702" & year==2004

replace regioncode="360721" if regioncode=="360721" & year==2004

replace regioncode="360722" if regioncode=="360722" & year==2004

replace regioncode="360723" if regioncode=="360723" & year==2004

replace regioncode="360724" if regioncode=="360724" & year==2004

replace regioncode="360725" if regioncode=="360725" & year==2004

replace regioncode="360726" if regioncode=="360726" & year==2004

replace regioncode="360727" if regioncode=="360727" & year==2004

replace regioncode="360728" if regioncode=="360728" & year==2004

replace regioncode="360729" if regioncode=="360729" & year==2004

replace regioncode="360730" if regioncode=="360730" & year==2004

replace regioncode="360731" if regioncode=="360731" & year==2004

replace regioncode="360732" if regioncode=="360732" & year==2004

replace regioncode="360733" if regioncode=="360733" & year==2004

replace regioncode="360734" if regioncode=="360734" & year==2004

replace regioncode="360735" if regioncode=="360735" & year==2004

replace regioncode="360781" if regioncode=="360781" & year==2004

replace regioncode="360782" if regioncode=="360782" & year==2004

replace regioncode="360802" if regioncode=="360802" & year==2004

replace regioncode="360821" if regioncode=="360821" & year==2004

replace regioncode="360822" if regioncode=="360822" & year==2004

replace regioncode="360823" if regioncode=="360823" & year==2004

replace regioncode="360824" if regioncode=="360824" & year==2004

replace regioncode="360825" if regioncode=="360825" & year==2004

replace regioncode="360826" if regioncode=="360826" & year==2004

replace regioncode="360827" if regioncode=="360827" & year==2004

replace regioncode="360828" if regioncode=="360828" & year==2004

replace regioncode="360829" if regioncode=="360829" & year==2004

replace regioncode="360830" if regioncode=="360830" & year==2004

replace regioncode="360881" if regioncode=="360881" & year==2004

replace regioncode="360902" if regioncode=="360902" & year==2004

replace regioncode="360921" if regioncode=="360921" & year==2004

replace regioncode="360922" if regioncode=="360922" & year==2004

replace regioncode="360923" if regioncode=="360923" & year==2004

replace regioncode="360924" if regioncode=="360924" & year==2004

replace regioncode="360925" if regioncode=="360925" & year==2004

replace regioncode="360926" if regioncode=="360926" & year==2004

replace regioncode="360981" if regioncode=="360981" & year==2004

replace regioncode="360982" if regioncode=="360982" & year==2004

replace regioncode="360983" if regioncode=="360983" & year==2004

replace regioncode="361002" if regioncode=="361002" & year==2004

replace regioncode="361021" if regioncode=="361021" & year==2004

replace regioncode="361022" if regioncode=="361022" & year==2004

replace regioncode="361023" if regioncode=="361023" & year==2004

replace regioncode="361024" if regioncode=="361024" & year==2004

replace regioncode="361025" if regioncode=="361025" & year==2004

replace regioncode="361026" if regioncode=="361026" & year==2004

replace regioncode="361027" if regioncode=="361027" & year==2004

replace regioncode="361028" if regioncode=="361028" & year==2004

replace regioncode="361029" if regioncode=="361029" & year==2004

replace regioncode="361030" if regioncode=="361030" & year==2004

replace regioncode="361102" if regioncode=="361102" & year==2004

replace regioncode="361121" if regioncode=="361121" & year==2004

replace regioncode="361122" if regioncode=="361122" & year==2004

replace regioncode="361123" if regioncode=="361123" & year==2004

replace regioncode="361124" if regioncode=="361124" & year==2004

replace regioncode="361125" if regioncode=="361125" & year==2004

replace regioncode="361126" if regioncode=="361126" & year==2004

replace regioncode="361127" if regioncode=="361127" & year==2004

replace regioncode="361128" if regioncode=="361128" & year==2004

replace regioncode="361129" if regioncode=="361129" & year==2004

replace regioncode="361130" if regioncode=="361130" & year==2004

replace regioncode="361181" if regioncode=="361181" & year==2004

replace regioncode="370102" if regioncode=="370102" & year==2004

replace regioncode="370103" if regioncode=="370103" & year==2004

replace regioncode="370104" if regioncode=="370104" & year==2004

replace regioncode="370105" if regioncode=="370105" & year==2004

replace regioncode="370112" if regioncode=="370112" & year==2004

replace regioncode="370113" if regioncode=="370113" & year==2004

replace regioncode="370124" if regioncode=="370124" & year==2004

replace regioncode="370125" if regioncode=="370125" & year==2004

replace regioncode="370126" if regioncode=="370126" & year==2004

replace regioncode="370181" if regioncode=="370181" & year==2004

replace regioncode="370202" if regioncode=="370202" & year==2004

replace regioncode="370203" if regioncode=="370203" & year==2004

replace regioncode="370205" if regioncode=="370205" & year==2004

replace regioncode="370211" if regioncode=="370211" & year==2004

replace regioncode="370212" if regioncode=="370212" & year==2004

replace regioncode="370213" if regioncode=="370213" & year==2004

replace regioncode="370214" if regioncode=="370214" & year==2004

replace regioncode="370281" if regioncode=="370281" & year==2004

replace regioncode="370282" if regioncode=="370282" & year==2004

replace regioncode="370283" if regioncode=="370283" & year==2004

replace regioncode="370284" if regioncode=="370284" & year==2004

replace regioncode="370285" if regioncode=="370285" & year==2004

replace regioncode="370302" if regioncode=="370302" & year==2004

replace regioncode="370303" if regioncode=="370303" & year==2004

replace regioncode="370304" if regioncode=="370304" & year==2004

replace regioncode="370305" if regioncode=="370305" & year==2004

replace regioncode="370306" if regioncode=="370306" & year==2004

replace regioncode="370321" if regioncode=="370321" & year==2004

replace regioncode="370322" if regioncode=="370322" & year==2004

replace regioncode="370323" if regioncode=="370323" & year==2004

replace regioncode="370402" if regioncode=="370402" & year==2004

replace regioncode="370403" if regioncode=="370403" & year==2004

replace regioncode="370404" if regioncode=="370404" & year==2004

replace regioncode="370405" if regioncode=="370405" & year==2004

replace regioncode="370406" if regioncode=="370406" & year==2004

replace regioncode="370481" if regioncode=="370481" & year==2004

replace regioncode="370502" if regioncode=="370502" & year==2004

replace regioncode="370503" if regioncode=="370503" & year==2004

replace regioncode="370521" if regioncode=="370521" & year==2004

replace regioncode="370522" if regioncode=="370522" & year==2004

replace regioncode="370523" if regioncode=="370523" & year==2004

replace regioncode="370602" if regioncode=="370602" & year==2004

replace regioncode="370611" if regioncode=="370611" & year==2004

replace regioncode="370612" if regioncode=="370612" & year==2004

replace regioncode="370613" if regioncode=="370613" & year==2004

replace regioncode="370634" if regioncode=="370634" & year==2004

replace regioncode="370681" if regioncode=="370681" & year==2004

replace regioncode="370682" if regioncode=="370682" & year==2004

replace regioncode="370683" if regioncode=="370683" & year==2004

replace regioncode="370684" if regioncode=="370684" & year==2004

replace regioncode="370685" if regioncode=="370685" & year==2004

replace regioncode="370686" if regioncode=="370686" & year==2004

replace regioncode="370687" if regioncode=="370687" & year==2004

replace regioncode="370702" if regioncode=="370702" & year==2004

replace regioncode="370703" if regioncode=="370703" & year==2004

replace regioncode="370704" if regioncode=="370704" & year==2004

replace regioncode="370705" if regioncode=="370705" & year==2004

replace regioncode="370724" if regioncode=="370724" & year==2004

replace regioncode="370725" if regioncode=="370725" & year==2004

replace regioncode="370781" if regioncode=="370781" & year==2004

replace regioncode="370782" if regioncode=="370782" & year==2004

replace regioncode="370783" if regioncode=="370783" & year==2004

replace regioncode="370784" if regioncode=="370784" & year==2004

replace regioncode="370785" if regioncode=="370785" & year==2004

replace regioncode="370786" if regioncode=="370786" & year==2004

replace regioncode="370802" if regioncode=="370802" & year==2004

replace regioncode="370811" if regioncode=="370811" & year==2004

replace regioncode="370826" if regioncode=="370826" & year==2004

replace regioncode="370827" if regioncode=="370827" & year==2004

replace regioncode="370828" if regioncode=="370828" & year==2004

replace regioncode="370829" if regioncode=="370829" & year==2004

replace regioncode="370830" if regioncode=="370830" & year==2004

replace regioncode="370831" if regioncode=="370831" & year==2004

replace regioncode="370832" if regioncode=="370832" & year==2004

replace regioncode="370881" if regioncode=="370881" & year==2004

replace regioncode="370882" if regioncode=="370882" & year==2004

replace regioncode="370883" if regioncode=="370883" & year==2004

replace regioncode="370902" if regioncode=="370902" & year==2004

replace regioncode="370903" if regioncode=="370903" & year==2004

replace regioncode="370921" if regioncode=="370921" & year==2004

replace regioncode="370923" if regioncode=="370923" & year==2004

replace regioncode="370982" if regioncode=="370982" & year==2004

replace regioncode="370983" if regioncode=="370983" & year==2004

replace regioncode="371002" if regioncode=="371002" & year==2004

replace regioncode="371081" if regioncode=="371081" & year==2004

replace regioncode="371082" if regioncode=="371082" & year==2004

replace regioncode="371083" if regioncode=="371083" & year==2004

replace regioncode="371102" if regioncode=="371102" & year==2004

replace regioncode="371121" if regioncode=="371121" & year==2004

replace regioncode="371122" if regioncode=="371122" & year==2004

replace regioncode="371202" if regioncode=="371202" & year==2004

replace regioncode="371203" if regioncode=="371203" & year==2004

replace regioncode="371302" if regioncode=="371302" & year==2004

replace regioncode="371311" if regioncode=="371311" & year==2004

replace regioncode="371312" if regioncode=="371312" & year==2004

replace regioncode="371321" if regioncode=="371321" & year==2004

replace regioncode="371322" if regioncode=="371322" & year==2004

replace regioncode="371323" if regioncode=="371323" & year==2004

replace regioncode="371324" if regioncode=="371324" & year==2004

replace regioncode="371325" if regioncode=="371325" & year==2004

replace regioncode="371326" if regioncode=="371326" & year==2004

replace regioncode="371327" if regioncode=="371327" & year==2004

replace regioncode="371328" if regioncode=="371328" & year==2004

replace regioncode="371329" if regioncode=="371329" & year==2004

replace regioncode="371402" if regioncode=="371402" & year==2004

replace regioncode="371421" if regioncode=="371421" & year==2004

replace regioncode="371422" if regioncode=="371422" & year==2004

replace regioncode="371423" if regioncode=="371423" & year==2004

replace regioncode="371424" if regioncode=="371424" & year==2004

replace regioncode="371425" if regioncode=="371425" & year==2004

replace regioncode="371426" if regioncode=="371426" & year==2004

replace regioncode="371427" if regioncode=="371427" & year==2004

replace regioncode="371428" if regioncode=="371428" & year==2004

replace regioncode="371481" if regioncode=="371481" & year==2004

replace regioncode="371482" if regioncode=="371482" & year==2004

replace regioncode="371502" if regioncode=="371502" & year==2004

replace regioncode="371521" if regioncode=="371521" & year==2004

replace regioncode="371522" if regioncode=="371522" & year==2004

replace regioncode="371523" if regioncode=="371523" & year==2004

replace regioncode="371524" if regioncode=="371524" & year==2004

replace regioncode="371525" if regioncode=="371525" & year==2004

replace regioncode="371526" if regioncode=="371526" & year==2004

replace regioncode="371581" if regioncode=="371581" & year==2004

replace regioncode="371602" if regioncode=="371602" & year==2004

replace regioncode="371621" if regioncode=="371621" & year==2004

replace regioncode="371622" if regioncode=="371622" & year==2004

replace regioncode="371623" if regioncode=="371623" & year==2004

replace regioncode="371624" if regioncode=="371624" & year==2004

replace regioncode="371625" if regioncode=="371625" & year==2004

replace regioncode="371626" if regioncode=="371626" & year==2004

replace regioncode="371702" if regioncode=="371702" & year==2004

replace regioncode="371721" if regioncode=="371721" & year==2004

replace regioncode="371722" if regioncode=="371722" & year==2004

replace regioncode="371723" if regioncode=="371723" & year==2004

replace regioncode="371724" if regioncode=="371724" & year==2004

replace regioncode="371725" if regioncode=="371725" & year==2004

replace regioncode="371726" if regioncode=="371726" & year==2004

replace regioncode="371727" if regioncode=="371727" & year==2004

replace regioncode="371728" if regioncode=="371728" & year==2004

replace regioncode="410102" if regioncode=="410102" & year==2004

replace regioncode="410103" if regioncode=="410103" & year==2004

replace regioncode="410104" if regioncode=="410104" & year==2004

replace regioncode="410105" if regioncode=="410105" & year==2004

replace regioncode="410106" if regioncode=="410106" & year==2004

replace regioncode="410108" if regioncode=="410108" & year==2004

replace regioncode="410122" if regioncode=="410122" & year==2004

replace regioncode="410181" if regioncode=="410181" & year==2004

replace regioncode="410182" if regioncode=="410182" & year==2004

replace regioncode="410183" if regioncode=="410183" & year==2004

replace regioncode="410184" if regioncode=="410184" & year==2004

replace regioncode="410185" if regioncode=="410185" & year==2004

replace regioncode="410202" if regioncode=="410202" & year==2004

replace regioncode="410203" if regioncode=="410203" & year==2004

replace regioncode="410204" if regioncode=="410204" & year==2004

replace regioncode="410205" if regioncode=="410205" & year==2004

replace regioncode="410211" if regioncode=="410211" & year==2004

replace regioncode="410221" if regioncode=="410221" & year==2004

replace regioncode="410222" if regioncode=="410222" & year==2004

replace regioncode="410223" if regioncode=="410223" & year==2004

replace regioncode="410224" if regioncode=="410224" & year==2004

replace regioncode="410225" if regioncode=="410225" & year==2004

replace regioncode="410302" if regioncode=="410302" & year==2004

replace regioncode="410303" if regioncode=="410303" & year==2004

replace regioncode="410304" if regioncode=="410304" & year==2004

replace regioncode="410305" if regioncode=="410305" & year==2004

replace regioncode="410306" if regioncode=="410306" & year==2004

replace regioncode="410307" if regioncode=="410307" & year==2004

replace regioncode="410322" if regioncode=="410322" & year==2004

replace regioncode="410323" if regioncode=="410323" & year==2004

replace regioncode="410324" if regioncode=="410324" & year==2004

replace regioncode="410325" if regioncode=="410325" & year==2004

replace regioncode="410326" if regioncode=="410326" & year==2004

replace regioncode="410327" if regioncode=="410327" & year==2004

replace regioncode="410328" if regioncode=="410328" & year==2004

replace regioncode="410329" if regioncode=="410329" & year==2004

replace regioncode="410381" if regioncode=="410381" & year==2004

replace regioncode="410402" if regioncode=="410402" & year==2004

replace regioncode="410403" if regioncode=="410403" & year==2004

replace regioncode="410404" if regioncode=="410404" & year==2004

replace regioncode="410411" if regioncode=="410411" & year==2004

replace regioncode="410421" if regioncode=="410421" & year==2004

replace regioncode="410422" if regioncode=="410422" & year==2004

replace regioncode="410423" if regioncode=="410423" & year==2004

replace regioncode="410425" if regioncode=="410425" & year==2004

replace regioncode="410481" if regioncode=="410481" & year==2004

replace regioncode="410482" if regioncode=="410482" & year==2004

replace regioncode="410502" if regioncode=="410502" & year==2004

replace regioncode="410503" if regioncode=="410503" & year==2004

replace regioncode="410504" if regioncode=="410505" & year==2004

replace regioncode="410511" if regioncode=="410506" & year==2004

replace regioncode="410522" if regioncode=="410522" & year==2004

replace regioncode="410523" if regioncode=="410523" & year==2004

replace regioncode="410526" if regioncode=="410526" & year==2004

replace regioncode="410527" if regioncode=="410527" & year==2004

replace regioncode="410581" if regioncode=="410581" & year==2004

replace regioncode="410602" if regioncode=="410602" & year==2004

replace regioncode="410603" if regioncode=="410603" & year==2004

replace regioncode="410611" if regioncode=="410611" & year==2004

replace regioncode="410621" if regioncode=="410621" & year==2004

replace regioncode="410622" if regioncode=="410622" & year==2004

replace regioncode="410702" if regioncode=="410702" & year==2004

replace regioncode="410703" if regioncode=="410703" & year==2004

replace regioncode="410704" if regioncode=="410704" & year==2004

replace regioncode="410711" if regioncode=="410711" & year==2004

replace regioncode="410721" if regioncode=="410721" & year==2004

replace regioncode="410724" if regioncode=="410724" & year==2004

replace regioncode="410725" if regioncode=="410725" & year==2004

replace regioncode="410726" if regioncode=="410726" & year==2004

replace regioncode="410727" if regioncode=="410727" & year==2004

replace regioncode="410728" if regioncode=="410728" & year==2004

replace regioncode="410781" if regioncode=="410781" & year==2004

replace regioncode="410782" if regioncode=="410782" & year==2004

replace regioncode="410802" if regioncode=="410802" & year==2004

replace regioncode="410803" if regioncode=="410803" & year==2004

replace regioncode="410804" if regioncode=="410804" & year==2004

replace regioncode="410811" if regioncode=="410811" & year==2004

replace regioncode="410821" if regioncode=="410821" & year==2004

replace regioncode="410822" if regioncode=="410822" & year==2004

replace regioncode="410823" if regioncode=="410823" & year==2004

replace regioncode="410825" if regioncode=="410825" & year==2004

replace regioncode="410881" if regioncode=="410881" & year==2004

replace regioncode="410882" if regioncode=="410882" & year==2004

replace regioncode="410883" if regioncode=="410883" & year==2004

replace regioncode="410902" if regioncode=="410902" & year==2004

replace regioncode="410922" if regioncode=="410922" & year==2004

replace regioncode="410923" if regioncode=="410923" & year==2004

replace regioncode="410926" if regioncode=="410926" & year==2004

replace regioncode="410927" if regioncode=="410927" & year==2004

replace regioncode="410928" if regioncode=="410928" & year==2004

replace regioncode="411002" if regioncode=="411002" & year==2004

replace regioncode="411023" if regioncode=="411023" & year==2004

replace regioncode="411024" if regioncode=="411024" & year==2004

replace regioncode="411025" if regioncode=="411025" & year==2004

replace regioncode="411081" if regioncode=="411081" & year==2004

replace regioncode="411082" if regioncode=="411082" & year==2004

replace regioncode="411102" if regioncode=="411102" & year==2004

replace regioncode="411123" if regioncode=="411103" & year==2004

replace regioncode="411121" if regioncode=="411121" & year==2004

replace regioncode="411122" if regioncode=="411122" & year==2004

replace regioncode="411202" if regioncode=="411202" & year==2004

replace regioncode="411221" if regioncode=="411221" & year==2004

replace regioncode="411222" if regioncode=="411222" & year==2004

replace regioncode="411224" if regioncode=="411224" & year==2004

replace regioncode="411281" if regioncode=="411281" & year==2004

replace regioncode="411282" if regioncode=="411282" & year==2004

replace regioncode="411302" if regioncode=="411302" & year==2004

replace regioncode="411303" if regioncode=="411303" & year==2004

replace regioncode="411321" if regioncode=="411321" & year==2004

replace regioncode="411322" if regioncode=="411322" & year==2004

replace regioncode="411323" if regioncode=="411323" & year==2004

replace regioncode="411324" if regioncode=="411324" & year==2004

replace regioncode="411325" if regioncode=="411325" & year==2004

replace regioncode="411326" if regioncode=="411326" & year==2004

replace regioncode="411327" if regioncode=="411327" & year==2004

replace regioncode="411328" if regioncode=="411328" & year==2004

replace regioncode="411329" if regioncode=="411329" & year==2004

replace regioncode="411330" if regioncode=="411330" & year==2004

replace regioncode="411381" if regioncode=="411381" & year==2004

replace regioncode="411402" if regioncode=="411402" & year==2004

replace regioncode="411403" if regioncode=="411403" & year==2004

replace regioncode="411421" if regioncode=="411421" & year==2004

replace regioncode="411422" if regioncode=="411422" & year==2004

replace regioncode="411423" if regioncode=="411423" & year==2004

replace regioncode="411424" if regioncode=="411424" & year==2004

replace regioncode="411425" if regioncode=="411425" & year==2004

replace regioncode="411426" if regioncode=="411426" & year==2004

replace regioncode="411481" if regioncode=="411481" & year==2004

replace regioncode="411502" if regioncode=="411502" & year==2004

replace regioncode="411503" if regioncode=="411503" & year==2004

replace regioncode="411521" if regioncode=="411521" & year==2004

replace regioncode="411522" if regioncode=="411522" & year==2004

replace regioncode="411523" if regioncode=="411523" & year==2004

replace regioncode="411524" if regioncode=="411524" & year==2004

replace regioncode="411525" if regioncode=="411525" & year==2004

replace regioncode="411526" if regioncode=="411526" & year==2004

replace regioncode="411527" if regioncode=="411527" & year==2004

replace regioncode="411528" if regioncode=="411528" & year==2004

replace regioncode="411602" if regioncode=="411602" & year==2004

replace regioncode="411621" if regioncode=="411621" & year==2004

replace regioncode="411622" if regioncode=="411622" & year==2004

replace regioncode="411623" if regioncode=="411623" & year==2004

replace regioncode="411624" if regioncode=="411624" & year==2004

replace regioncode="411625" if regioncode=="411625" & year==2004

replace regioncode="411626" if regioncode=="411626" & year==2004

replace regioncode="411627" if regioncode=="411627" & year==2004

replace regioncode="411628" if regioncode=="411628" & year==2004

replace regioncode="411681" if regioncode=="411681" & year==2004

replace regioncode="411702" if regioncode=="411702" & year==2004

replace regioncode="411721" if regioncode=="411721" & year==2004

replace regioncode="411722" if regioncode=="411722" & year==2004

replace regioncode="411723" if regioncode=="411723" & year==2004

replace regioncode="411724" if regioncode=="411724" & year==2004

replace regioncode="411725" if regioncode=="411725" & year==2004

replace regioncode="411726" if regioncode=="411726" & year==2004

replace regioncode="411727" if regioncode=="411727" & year==2004

replace regioncode="411728" if regioncode=="411728" & year==2004

replace regioncode="411729" if regioncode=="411729" & year==2004

replace regioncode="420102" if regioncode=="420102" & year==2004

replace regioncode="420103" if regioncode=="420103" & year==2004

replace regioncode="420104" if regioncode=="420104" & year==2004

replace regioncode="420105" if regioncode=="420105" & year==2004

replace regioncode="420106" if regioncode=="420106" & year==2004

replace regioncode="420107" if regioncode=="420107" & year==2004

replace regioncode="420111" if regioncode=="420111" & year==2004

replace regioncode="420112" if regioncode=="420112" & year==2004

replace regioncode="420113" if regioncode=="420113" & year==2004

replace regioncode="420114" if regioncode=="420114" & year==2004

replace regioncode="420115" if regioncode=="420115" & year==2004

replace regioncode="420116" if regioncode=="420116" & year==2004

replace regioncode="420117" if regioncode=="420117" & year==2004

replace regioncode="420202" if regioncode=="420202" & year==2004

replace regioncode="420203" if regioncode=="420203" & year==2004

replace regioncode="420204" if regioncode=="420204" & year==2004

replace regioncode="420205" if regioncode=="420205" & year==2004

replace regioncode="420222" if regioncode=="420222" & year==2004

replace regioncode="420281" if regioncode=="420281" & year==2004

replace regioncode="420302" if regioncode=="420302" & year==2004

replace regioncode="420303" if regioncode=="420303" & year==2004

replace regioncode="420321" if regioncode=="420321" & year==2004

replace regioncode="420322" if regioncode=="420322" & year==2004

replace regioncode="420323" if regioncode=="420323" & year==2004

replace regioncode="420324" if regioncode=="420324" & year==2004

replace regioncode="420325" if regioncode=="420325" & year==2004

replace regioncode="420381" if regioncode=="420381" & year==2004

replace regioncode="420502" if regioncode=="420502" & year==2004

replace regioncode="420503" if regioncode=="420503" & year==2004

replace regioncode="420504" if regioncode=="420504" & year==2004

replace regioncode="420505" if regioncode=="420505" & year==2004

replace regioncode="420506" if regioncode=="420506" & year==2004

replace regioncode="420525" if regioncode=="420525" & year==2004

replace regioncode="420526" if regioncode=="420526" & year==2004

replace regioncode="420527" if regioncode=="420527" & year==2004

replace regioncode="420528" if regioncode=="420528" & year==2004

replace regioncode="420529" if regioncode=="420529" & year==2004

replace regioncode="420581" if regioncode=="420581" & year==2004

replace regioncode="420582" if regioncode=="420582" & year==2004

replace regioncode="420583" if regioncode=="420583" & year==2004

replace regioncode="420602" if regioncode=="420602" & year==2004

replace regioncode="420606" if regioncode=="420606" & year==2004

replace regioncode="420607" if regioncode=="420607" & year==2004

replace regioncode="420624" if regioncode=="420624" & year==2004

replace regioncode="420625" if regioncode=="420625" & year==2004

replace regioncode="420626" if regioncode=="420626" & year==2004

replace regioncode="420682" if regioncode=="420682" & year==2004

replace regioncode="420683" if regioncode=="420683" & year==2004

replace regioncode="420684" if regioncode=="420684" & year==2004

replace regioncode="420702" if regioncode=="420702" & year==2004

replace regioncode="420703" if regioncode=="420703" & year==2004

replace regioncode="420704" if regioncode=="420704" & year==2004

replace regioncode="420802" if regioncode=="420802" & year==2004

replace regioncode="420804" if regioncode=="420804" & year==2004

replace regioncode="420821" if regioncode=="420821" & year==2004

replace regioncode="420822" if regioncode=="420822" & year==2004

replace regioncode="420881" if regioncode=="420881" & year==2004

replace regioncode="420902" if regioncode=="420902" & year==2004

replace regioncode="420921" if regioncode=="420921" & year==2004

replace regioncode="420922" if regioncode=="420922" & year==2004

replace regioncode="420923" if regioncode=="420923" & year==2004

replace regioncode="420981" if regioncode=="420981" & year==2004

replace regioncode="420982" if regioncode=="420982" & year==2004

replace regioncode="421381" if regioncode=="421381" & year==2004

replace regioncode="420984" if regioncode=="420984" & year==2004

replace regioncode="421002" if regioncode=="421002" & year==2004

replace regioncode="421003" if regioncode=="421003" & year==2004

replace regioncode="421022" if regioncode=="421022" & year==2004

replace regioncode="421023" if regioncode=="421023" & year==2004

replace regioncode="421024" if regioncode=="421024" & year==2004

replace regioncode="421081" if regioncode=="421081" & year==2004

replace regioncode="421083" if regioncode=="421083" & year==2004

replace regioncode="421087" if regioncode=="421087" & year==2004

replace regioncode="421102" if regioncode=="421102" & year==2004

replace regioncode="421121" if regioncode=="421121" & year==2004

replace regioncode="421122" if regioncode=="421122" & year==2004

replace regioncode="421123" if regioncode=="421123" & year==2004

replace regioncode="421124" if regioncode=="421124" & year==2004

replace regioncode="421125" if regioncode=="421125" & year==2004

replace regioncode="421126" if regioncode=="421126" & year==2004

replace regioncode="421127" if regioncode=="421127" & year==2004

replace regioncode="421181" if regioncode=="421181" & year==2004

replace regioncode="421182" if regioncode=="421182" & year==2004

replace regioncode="421202" if regioncode=="421202" & year==2004

replace regioncode="421221" if regioncode=="421221" & year==2004

replace regioncode="421222" if regioncode=="421222" & year==2004

replace regioncode="421223" if regioncode=="421223" & year==2004

replace regioncode="421224" if regioncode=="421224" & year==2004

replace regioncode="421281" if regioncode=="421281" & year==2004

replace regioncode="421302" if regioncode=="421302" & year==2004

replace regioncode="422801" if regioncode=="422801" & year==2004

replace regioncode="422802" if regioncode=="422802" & year==2004

replace regioncode="422822" if regioncode=="422822" & year==2004

replace regioncode="422823" if regioncode=="422823" & year==2004

replace regioncode="422825" if regioncode=="422825" & year==2004

replace regioncode="422826" if regioncode=="422826" & year==2004

replace regioncode="422827" if regioncode=="422827" & year==2004

replace regioncode="422828" if regioncode=="422828" & year==2004

replace regioncode="429004" if regioncode=="429004" & year==2004

replace regioncode="429005" if regioncode=="429005" & year==2004

replace regioncode="429006" if regioncode=="429006" & year==2004

replace regioncode="429021" if regioncode=="429021" & year==2004

replace regioncode="430102" if regioncode=="430102" & year==2004

replace regioncode="430103" if regioncode=="430103" & year==2004

replace regioncode="430104" if regioncode=="430104" & year==2004

replace regioncode="430105" if regioncode=="430105" & year==2004

replace regioncode="430111" if regioncode=="430111" & year==2004

replace regioncode="430121" if regioncode=="430121" & year==2004

replace regioncode="430122" if regioncode=="430122" & year==2004

replace regioncode="430124" if regioncode=="430124" & year==2004

replace regioncode="430181" if regioncode=="430181" & year==2004

replace regioncode="430202" if regioncode=="430202" & year==2004

replace regioncode="430203" if regioncode=="430203" & year==2004

replace regioncode="430204" if regioncode=="430204" & year==2004

replace regioncode="430211" if regioncode=="430211" & year==2004

replace regioncode="430221" if regioncode=="430221" & year==2004

replace regioncode="430223" if regioncode=="430223" & year==2004

replace regioncode="430224" if regioncode=="430224" & year==2004

replace regioncode="430225" if regioncode=="430225" & year==2004

replace regioncode="430281" if regioncode=="430281" & year==2004

replace regioncode="430302" if regioncode=="430302" & year==2004

replace regioncode="430304" if regioncode=="430304" & year==2004

replace regioncode="430321" if regioncode=="430321" & year==2004

replace regioncode="430381" if regioncode=="430381" & year==2004

replace regioncode="430382" if regioncode=="430382" & year==2004

replace regioncode="430405" if regioncode=="430405" & year==2004

replace regioncode="430406" if regioncode=="430406" & year==2004

replace regioncode="430407" if regioncode=="430407" & year==2004

replace regioncode="430408" if regioncode=="430408" & year==2004

replace regioncode="430412" if regioncode=="430412" & year==2004

replace regioncode="430421" if regioncode=="430421" & year==2004

replace regioncode="430422" if regioncode=="430422" & year==2004

replace regioncode="430423" if regioncode=="430423" & year==2004

replace regioncode="430424" if regioncode=="430424" & year==2004

replace regioncode="430426" if regioncode=="430426" & year==2004

replace regioncode="430481" if regioncode=="430481" & year==2004

replace regioncode="430482" if regioncode=="430482" & year==2004

replace regioncode="430502" if regioncode=="430502" & year==2004

replace regioncode="430503" if regioncode=="430503" & year==2004

replace regioncode="430511" if regioncode=="430511" & year==2004

replace regioncode="430521" if regioncode=="430521" & year==2004

replace regioncode="430522" if regioncode=="430522" & year==2004

replace regioncode="430523" if regioncode=="430523" & year==2004

replace regioncode="430524" if regioncode=="430524" & year==2004

replace regioncode="430525" if regioncode=="430525" & year==2004

replace regioncode="430527" if regioncode=="430527" & year==2004

replace regioncode="430528" if regioncode=="430528" & year==2004

replace regioncode="430529" if regioncode=="430529" & year==2004

replace regioncode="430581" if regioncode=="430581" & year==2004

replace regioncode="430602" if regioncode=="430602" & year==2004

replace regioncode="430603" if regioncode=="430603" & year==2004

replace regioncode="430611" if regioncode=="430611" & year==2004

replace regioncode="430621" if regioncode=="430621" & year==2004

replace regioncode="430623" if regioncode=="430623" & year==2004

replace regioncode="430624" if regioncode=="430624" & year==2004

replace regioncode="430626" if regioncode=="430626" & year==2004

replace regioncode="430681" if regioncode=="430681" & year==2004

replace regioncode="430682" if regioncode=="430682" & year==2004

replace regioncode="430702" if regioncode=="430702" & year==2004

replace regioncode="430703" if regioncode=="430703" & year==2004

replace regioncode="430721" if regioncode=="430721" & year==2004

replace regioncode="430722" if regioncode=="430722" & year==2004

replace regioncode="430723" if regioncode=="430723" & year==2004

replace regioncode="430724" if regioncode=="430724" & year==2004

replace regioncode="430725" if regioncode=="430725" & year==2004

replace regioncode="430726" if regioncode=="430726" & year==2004

replace regioncode="430781" if regioncode=="430781" & year==2004

replace regioncode="430802" if regioncode=="430802" & year==2004

replace regioncode="430811" if regioncode=="430811" & year==2004

replace regioncode="430821" if regioncode=="430821" & year==2004

replace regioncode="430822" if regioncode=="430822" & year==2004

replace regioncode="430902" if regioncode=="430902" & year==2004

replace regioncode="430903" if regioncode=="430903" & year==2004

replace regioncode="430921" if regioncode=="430921" & year==2004

replace regioncode="430922" if regioncode=="430922" & year==2004

replace regioncode="430923" if regioncode=="430923" & year==2004

replace regioncode="430981" if regioncode=="430981" & year==2004

replace regioncode="431002" if regioncode=="431002" & year==2004

replace regioncode="431003" if regioncode=="431003" & year==2004

replace regioncode="431021" if regioncode=="431021" & year==2004

replace regioncode="431022" if regioncode=="431022" & year==2004

replace regioncode="431023" if regioncode=="431023" & year==2004

replace regioncode="431024" if regioncode=="431024" & year==2004

replace regioncode="431025" if regioncode=="431025" & year==2004

replace regioncode="431026" if regioncode=="431026" & year==2004

replace regioncode="431027" if regioncode=="431027" & year==2004

replace regioncode="431028" if regioncode=="431028" & year==2004

replace regioncode="431081" if regioncode=="431081" & year==2004

replace regioncode="431102" if regioncode=="431102" & year==2004

replace regioncode="431103" if regioncode=="431103" & year==2004

replace regioncode="431121" if regioncode=="431121" & year==2004

replace regioncode="431122" if regioncode=="431122" & year==2004

replace regioncode="431123" if regioncode=="431123" & year==2004

replace regioncode="431124" if regioncode=="431124" & year==2004

replace regioncode="431125" if regioncode=="431125" & year==2004

replace regioncode="431126" if regioncode=="431126" & year==2004

replace regioncode="431127" if regioncode=="431127" & year==2004

replace regioncode="431128" if regioncode=="431128" & year==2004

replace regioncode="431129" if regioncode=="431129" & year==2004

replace regioncode="431202" if regioncode=="431202" & year==2004

replace regioncode="431221" if regioncode=="431221" & year==2004

replace regioncode="431222" if regioncode=="431222" & year==2004

replace regioncode="431223" if regioncode=="431223" & year==2004

replace regioncode="431224" if regioncode=="431224" & year==2004

replace regioncode="431225" if regioncode=="431225" & year==2004

replace regioncode="431226" if regioncode=="431226" & year==2004

replace regioncode="431227" if regioncode=="431227" & year==2004

replace regioncode="431228" if regioncode=="431228" & year==2004

replace regioncode="431229" if regioncode=="431229" & year==2004

replace regioncode="431230" if regioncode=="431230" & year==2004

replace regioncode="431281" if regioncode=="431281" & year==2004

replace regioncode="431302" if regioncode=="431302" & year==2004

replace regioncode="431321" if regioncode=="431321" & year==2004

replace regioncode="431322" if regioncode=="431322" & year==2004

replace regioncode="431381" if regioncode=="431381" & year==2004

replace regioncode="431382" if regioncode=="431382" & year==2004

replace regioncode="433101" if regioncode=="433101" & year==2004

replace regioncode="433122" if regioncode=="433122" & year==2004

replace regioncode="433123" if regioncode=="433123" & year==2004

replace regioncode="433124" if regioncode=="433124" & year==2004

replace regioncode="433125" if regioncode=="433125" & year==2004

replace regioncode="433126" if regioncode=="433126" & year==2004

replace regioncode="433127" if regioncode=="433127" & year==2004

replace regioncode="433130" if regioncode=="433130" & year==2004

replace regioncode="440102" if regioncode=="440102" & year==2004

replace regioncode="440103" if regioncode=="440103" & year==2004

replace regioncode="440104" if regioncode=="440104" & year==2004

replace regioncode="440105" if regioncode=="440105" & year==2004

replace regioncode="440106" if regioncode=="440106" & year==2004

replace regioncode="440107" if regioncode=="440107" & year==2004

replace regioncode="440111" if regioncode=="440111" & year==2004

replace regioncode="440112" if regioncode=="440112" & year==2004

replace regioncode="440113" if regioncode=="440113" & year==2004

replace regioncode="440114" if regioncode=="440114" & year==2004

replace regioncode="440183" if regioncode=="440183" & year==2004

replace regioncode="440184" if regioncode=="440184" & year==2004

replace regioncode="440203" if regioncode=="440203" & year==2004

replace regioncode="440204" if regioncode=="440204" & year==2004

replace regioncode="440221" if regioncode=="440205" & year==2004

replace regioncode="440222" if regioncode=="440222" & year==2004

replace regioncode="440224" if regioncode=="440224" & year==2004

replace regioncode="440229" if regioncode=="440229" & year==2004

replace regioncode="440232" if regioncode=="440232" & year==2004

replace regioncode="440233" if regioncode=="440233" & year==2004

replace regioncode="440281" if regioncode=="440281" & year==2004

replace regioncode="440282" if regioncode=="440282" & year==2004

replace regioncode="440303" if regioncode=="440303" & year==2004

replace regioncode="440304" if regioncode=="440304" & year==2004

replace regioncode="440305" if regioncode=="440305" & year==2004

replace regioncode="440306" if regioncode=="440306" & year==2004

replace regioncode="440307" if regioncode=="440307" & year==2004

replace regioncode="440308" if regioncode=="440308" & year==2004

replace regioncode="440402" if regioncode=="440402" & year==2004

replace regioncode="440403" if regioncode=="440403" & year==2004

replace regioncode="440404" if regioncode=="440404" & year==2004

replace regioncode="440507" if regioncode=="440507" & year==2004

replace regioncode="440511" if regioncode=="440511" & year==2004

replace regioncode="440512" if regioncode=="440512" & year==2004

replace regioncode="440514" if regioncode=="440514" & year==2004

replace regioncode="440515" if regioncode=="440515" & year==2004

replace regioncode="440523" if regioncode=="440523" & year==2004

replace regioncode="440604" if regioncode=="440604" & year==2004

replace regioncode="440605" if regioncode=="440605" & year==2004

replace regioncode="440606" if regioncode=="440606" & year==2004

replace regioncode="440607" if regioncode=="440607" & year==2004

replace regioncode="440608" if regioncode=="440608" & year==2004

replace regioncode="440703" if regioncode=="440703" & year==2004

replace regioncode="440704" if regioncode=="440704" & year==2004

replace regioncode="440781" if regioncode=="440781" & year==2004

replace regioncode="440705" if regioncode=="440705" & year==2004

replace regioncode="440783" if regioncode=="440783" & year==2004

replace regioncode="440784" if regioncode=="440784" & year==2004

replace regioncode="440785" if regioncode=="440785" & year==2004

replace regioncode="440802" if regioncode=="440802" & year==2004

replace regioncode="440803" if regioncode=="440803" & year==2004

replace regioncode="440804" if regioncode=="440804" & year==2004

replace regioncode="440811" if regioncode=="440811" & year==2004

replace regioncode="440823" if regioncode=="440823" & year==2004

replace regioncode="440825" if regioncode=="440825" & year==2004

replace regioncode="440881" if regioncode=="440881" & year==2004

replace regioncode="440882" if regioncode=="440882" & year==2004

replace regioncode="440883" if regioncode=="440883" & year==2004

replace regioncode="440902" if regioncode=="440902" & year==2004

replace regioncode="440903" if regioncode=="440903" & year==2004

replace regioncode="440923" if regioncode=="440923" & year==2004

replace regioncode="440981" if regioncode=="440981" & year==2004

replace regioncode="440982" if regioncode=="440982" & year==2004

replace regioncode="440983" if regioncode=="440983" & year==2004

replace regioncode="441202" if regioncode=="441202" & year==2004

replace regioncode="441203" if regioncode=="441203" & year==2004

replace regioncode="441223" if regioncode=="441223" & year==2004

replace regioncode="441224" if regioncode=="441224" & year==2004

replace regioncode="441225" if regioncode=="441225" & year==2004

replace regioncode="441226" if regioncode=="441226" & year==2004

replace regioncode="441283" if regioncode=="441283" & year==2004

replace regioncode="441284" if regioncode=="441284" & year==2004

replace regioncode="441302" if regioncode=="441302" & year==2004

replace regioncode="441303" if regioncode=="441303" & year==2004

replace regioncode="441322" if regioncode=="441322" & year==2004

replace regioncode="441323" if regioncode=="441323" & year==2004

replace regioncode="441324" if regioncode=="441324" & year==2004

replace regioncode="441402" if regioncode=="441402" & year==2004

replace regioncode="441421" if regioncode=="441421" & year==2004

replace regioncode="441422" if regioncode=="441422" & year==2004

replace regioncode="441423" if regioncode=="441423" & year==2004

replace regioncode="441424" if regioncode=="441424" & year==2004

replace regioncode="441426" if regioncode=="441426" & year==2004

replace regioncode="441427" if regioncode=="441427" & year==2004

replace regioncode="441481" if regioncode=="441481" & year==2004

replace regioncode="441502" if regioncode=="441502" & year==2004

replace regioncode="441521" if regioncode=="441521" & year==2004

replace regioncode="441523" if regioncode=="441523" & year==2004

replace regioncode="441581" if regioncode=="441581" & year==2004

replace regioncode="441602" if regioncode=="441602" & year==2004

replace regioncode="441621" if regioncode=="441621" & year==2004

replace regioncode="441622" if regioncode=="441622" & year==2004

replace regioncode="441623" if regioncode=="441623" & year==2004

replace regioncode="441624" if regioncode=="441624" & year==2004

replace regioncode="441625" if regioncode=="441625" & year==2004

replace regioncode="441702" if regioncode=="441702" & year==2004

replace regioncode="441721" if regioncode=="441721" & year==2004

replace regioncode="441723" if regioncode=="441723" & year==2004

replace regioncode="441781" if regioncode=="441781" & year==2004

replace regioncode="441802" if regioncode=="441802" & year==2004

replace regioncode="441821" if regioncode=="441821" & year==2004

replace regioncode="441823" if regioncode=="441823" & year==2004

replace regioncode="441825" if regioncode=="441825" & year==2004

replace regioncode="441826" if regioncode=="441826" & year==2004

replace regioncode="441827" if regioncode=="441827" & year==2004

replace regioncode="441881" if regioncode=="441881" & year==2004

replace regioncode="441882" if regioncode=="441882" & year==2004

replace regioncode="441900" if regioncode=="441900" & year==2004

replace regioncode="442000" if regioncode=="442000" & year==2004

replace regioncode="445102" if regioncode=="445102" & year==2004

replace regioncode="445121" if regioncode=="445121" & year==2004

replace regioncode="445122" if regioncode=="445122" & year==2004

replace regioncode="445202" if regioncode=="445202" & year==2004

replace regioncode="445221" if regioncode=="445221" & year==2004

replace regioncode="445222" if regioncode=="445222" & year==2004

replace regioncode="445224" if regioncode=="445224" & year==2004

replace regioncode="445281" if regioncode=="445281" & year==2004

replace regioncode="445302" if regioncode=="445302" & year==2004

replace regioncode="445321" if regioncode=="445321" & year==2004

replace regioncode="445322" if regioncode=="445322" & year==2004

replace regioncode="445323" if regioncode=="445323" & year==2004

replace regioncode="445381" if regioncode=="445381" & year==2004

replace regioncode="450102" if regioncode=="450102" & year==2004

replace regioncode="450103" if regioncode=="450103" & year==2004

replace regioncode="450104" if regioncode=="450107" & year==2004

replace regioncode="450105" if regioncode=="450105" & year==2004

replace regioncode="450106" if regioncode=="450108" & year==2004

replace regioncode="450121" if regioncode=="450109" & year==2004

replace regioncode="450122" if regioncode=="450122" & year==2004

replace regioncode="450123" if regioncode=="450123" & year==2004

replace regioncode="450124" if regioncode=="450124" & year==2004

replace regioncode="450125" if regioncode=="450125" & year==2004

replace regioncode="450126" if regioncode=="450126" & year==2004

replace regioncode="450127" if regioncode=="450127" & year==2004

replace regioncode="450202" if regioncode=="450202" & year==2004

replace regioncode="450203" if regioncode=="450203" & year==2004

replace regioncode="450204" if regioncode=="450204" & year==2004

replace regioncode="450205" if regioncode=="450205" & year==2004

replace regioncode="450221" if regioncode=="450221" & year==2004

replace regioncode="450222" if regioncode=="450222" & year==2004

replace regioncode="450223" if regioncode=="450223" & year==2004

replace regioncode="450224" if regioncode=="450224" & year==2004

replace regioncode="450225" if regioncode=="450225" & year==2004

replace regioncode="450226" if regioncode=="450226" & year==2004

replace regioncode="450302" if regioncode=="450302" & year==2004

replace regioncode="450303" if regioncode=="450303" & year==2004

replace regioncode="450304" if regioncode=="450304" & year==2004

replace regioncode="450305" if regioncode=="450305" & year==2004

replace regioncode="450311" if regioncode=="450311" & year==2004

replace regioncode="450321" if regioncode=="450321" & year==2004

replace regioncode="450322" if regioncode=="450322" & year==2004

replace regioncode="450323" if regioncode=="450323" & year==2004

replace regioncode="450324" if regioncode=="450324" & year==2004

replace regioncode="450325" if regioncode=="450325" & year==2004

replace regioncode="450326" if regioncode=="450326" & year==2004

replace regioncode="450327" if regioncode=="450327" & year==2004

replace regioncode="450328" if regioncode=="450328" & year==2004

replace regioncode="450329" if regioncode=="450329" & year==2004

replace regioncode="450330" if regioncode=="450330" & year==2004

replace regioncode="450331" if regioncode=="450331" & year==2004

replace regioncode="450332" if regioncode=="450332" & year==2004

replace regioncode="450403" if regioncode=="450403" & year==2004

replace regioncode="450404" if regioncode=="450404" & year==2004

replace regioncode="450421" if regioncode=="450421" & year==2004

replace regioncode="450422" if regioncode=="450422" & year==2004

replace regioncode="450423" if regioncode=="450423" & year==2004

replace regioncode="450481" if regioncode=="450481" & year==2004

replace regioncode="450502" if regioncode=="450502" & year==2004

replace regioncode="450503" if regioncode=="450503" & year==2004

replace regioncode="450512" if regioncode=="450512" & year==2004

replace regioncode="450521" if regioncode=="450521" & year==2004

replace regioncode="450602" if regioncode=="450602" & year==2004

replace regioncode="450603" if regioncode=="450603" & year==2004

replace regioncode="450621" if regioncode=="450621" & year==2004

replace regioncode="450681" if regioncode=="450681" & year==2004

replace regioncode="450702" if regioncode=="450702" & year==2004

replace regioncode="450703" if regioncode=="450703" & year==2004

replace regioncode="450721" if regioncode=="450721" & year==2004

replace regioncode="450722" if regioncode=="450722" & year==2004

replace regioncode="450802" if regioncode=="450802" & year==2004

replace regioncode="450803" if regioncode=="450803" & year==2004

replace regioncode="450804" if regioncode=="450804" & year==2004

replace regioncode="450821" if regioncode=="450821" & year==2004

replace regioncode="450881" if regioncode=="450881" & year==2004

replace regioncode="450902" if regioncode=="450902" & year==2004

replace regioncode="450921" if regioncode=="450921" & year==2004

replace regioncode="450922" if regioncode=="450922" & year==2004

replace regioncode="450923" if regioncode=="450923" & year==2004

replace regioncode="450924" if regioncode=="450924" & year==2004

replace regioncode="450981" if regioncode=="450981" & year==2004

replace regioncode="451002" if regioncode=="451002" & year==2004

replace regioncode="451021" if regioncode=="451021" & year==2004

replace regioncode="451022" if regioncode=="451022" & year==2004

replace regioncode="451023" if regioncode=="451023" & year==2004

replace regioncode="451024" if regioncode=="451024" & year==2004

replace regioncode="451025" if regioncode=="451025" & year==2004

replace regioncode="451026" if regioncode=="451026" & year==2004

replace regioncode="451027" if regioncode=="451027" & year==2004

replace regioncode="451028" if regioncode=="451028" & year==2004

replace regioncode="451029" if regioncode=="451029" & year==2004

replace regioncode="451030" if regioncode=="451030" & year==2004

replace regioncode="451031" if regioncode=="451031" & year==2004

replace regioncode="451102" if regioncode=="451102" & year==2004

replace regioncode="451121" if regioncode=="451121" & year==2004

replace regioncode="451122" if regioncode=="451122" & year==2004

replace regioncode="451123" if regioncode=="451123" & year==2004

replace regioncode="451202" if regioncode=="451202" & year==2004

replace regioncode="451221" if regioncode=="451221" & year==2004

replace regioncode="451222" if regioncode=="451222" & year==2004

replace regioncode="451223" if regioncode=="451223" & year==2004

replace regioncode="451224" if regioncode=="451224" & year==2004

replace regioncode="451225" if regioncode=="451225" & year==2004

replace regioncode="451226" if regioncode=="451226" & year==2004

replace regioncode="451227" if regioncode=="451227" & year==2004

replace regioncode="451228" if regioncode=="451228" & year==2004

replace regioncode="451229" if regioncode=="451229" & year==2004

replace regioncode="451281" if regioncode=="451281" & year==2004

replace regioncode="451302" if regioncode=="451302" & year==2004

replace regioncode="451321" if regioncode=="451321" & year==2004

replace regioncode="451322" if regioncode=="451322" & year==2004

replace regioncode="451323" if regioncode=="451323" & year==2004

replace regioncode="451324" if regioncode=="451324" & year==2004

replace regioncode="451381" if regioncode=="451381" & year==2004

replace regioncode="451402" if regioncode=="451402" & year==2004

replace regioncode="451421" if regioncode=="451421" & year==2004

replace regioncode="451422" if regioncode=="451422" & year==2004

replace regioncode="451423" if regioncode=="451423" & year==2004

replace regioncode="451424" if regioncode=="451424" & year==2004

replace regioncode="451425" if regioncode=="451425" & year==2004

replace regioncode="451481" if regioncode=="451481" & year==2004

replace regioncode="460105" if regioncode=="460105" & year==2004

replace regioncode="460106" if regioncode=="460106" & year==2004

replace regioncode="460107" if regioncode=="460107" & year==2004

replace regioncode="460108" if regioncode=="460108" & year==2004

replace regioncode="460001" if regioncode=="469001" & year==2004

replace regioncode="460002" if regioncode=="469002" & year==2004

replace regioncode="460003" if regioncode=="469003" & year==2004

replace regioncode="460005" if regioncode=="469005" & year==2004

replace regioncode="460006" if regioncode=="469006" & year==2004

replace regioncode="460007" if regioncode=="469007" & year==2004

replace regioncode="460030" if regioncode=="469025" & year==2004

replace regioncode="460031" if regioncode=="469026" & year==2004

replace regioncode="460033" if regioncode=="469027" & year==2004

replace regioncode="460034" if regioncode=="469028" & year==2004

replace regioncode="460036" if regioncode=="469030" & year==2004

replace regioncode="500101" if regioncode=="500101" & year==2004

replace regioncode="500102" if regioncode=="500102" & year==2004

replace regioncode="500103" if regioncode=="500103" & year==2004

replace regioncode="500104" if regioncode=="500104" & year==2004

replace regioncode="500105" if regioncode=="500105" & year==2004

replace regioncode="500106" if regioncode=="500106" & year==2004

replace regioncode="500107" if regioncode=="500107" & year==2004

replace regioncode="500108" if regioncode=="500108" & year==2004

replace regioncode="500109" if regioncode=="500109" & year==2004

replace regioncode="500110" if regioncode=="500110" & year==2004

replace regioncode="500111" if regioncode=="500111" & year==2004

replace regioncode="500112" if regioncode=="500112" & year==2004

replace regioncode="500113" if regioncode=="500113" & year==2004

replace regioncode="500114" if regioncode=="500114" & year==2004

replace regioncode="500115" if regioncode=="500115" & year==2004

replace regioncode="500381" if regioncode=="500381" & year==2004

replace regioncode="500382" if regioncode=="500382" & year==2004

replace regioncode="500383" if regioncode=="500383" & year==2004

replace regioncode="500384" if regioncode=="500384" & year==2004

replace regioncode="500222" if regioncode=="500222" & year==2004

replace regioncode="500223" if regioncode=="500223" & year==2004

replace regioncode="500224" if regioncode=="500224" & year==2004

replace regioncode="500225" if regioncode=="500225" & year==2004

replace regioncode="500226" if regioncode=="500226" & year==2004

replace regioncode="500227" if regioncode=="500227" & year==2004

replace regioncode="500228" if regioncode=="500228" & year==2004

replace regioncode="500229" if regioncode=="500229" & year==2004

replace regioncode="500230" if regioncode=="500230" & year==2004

replace regioncode="500231" if regioncode=="500231" & year==2004

replace regioncode="500232" if regioncode=="500232" & year==2004

replace regioncode="500233" if regioncode=="500233" & year==2004

replace regioncode="500234" if regioncode=="500234" & year==2004

replace regioncode="500235" if regioncode=="500235" & year==2004

replace regioncode="500236" if regioncode=="500236" & year==2004

replace regioncode="500237" if regioncode=="500237" & year==2004

replace regioncode="500238" if regioncode=="500238" & year==2004

replace regioncode="500240" if regioncode=="500240" & year==2004

replace regioncode="500241" if regioncode=="500241" & year==2004

replace regioncode="500242" if regioncode=="500242" & year==2004

replace regioncode="500243" if regioncode=="500243" & year==2004

replace regioncode="510104" if regioncode=="510104" & year==2004

replace regioncode="510105" if regioncode=="510105" & year==2004

replace regioncode="510106" if regioncode=="510106" & year==2004

replace regioncode="510107" if regioncode=="510107" & year==2004

replace regioncode="510108" if regioncode=="510108" & year==2004

replace regioncode="510112" if regioncode=="510112" & year==2004

replace regioncode="510113" if regioncode=="510113" & year==2004

replace regioncode="510114" if regioncode=="510114" & year==2004

replace regioncode="510115" if regioncode=="510115" & year==2004

replace regioncode="510121" if regioncode=="510121" & year==2004

replace regioncode="510122" if regioncode=="510122" & year==2004

replace regioncode="510124" if regioncode=="510124" & year==2004

replace regioncode="510129" if regioncode=="510129" & year==2004

replace regioncode="510131" if regioncode=="510131" & year==2004

replace regioncode="510132" if regioncode=="510132" & year==2004

replace regioncode="510181" if regioncode=="510181" & year==2004

replace regioncode="510182" if regioncode=="510182" & year==2004

replace regioncode="510183" if regioncode=="510183" & year==2004

replace regioncode="510184" if regioncode=="510184" & year==2004

replace regioncode="510302" if regioncode=="510302" & year==2004

replace regioncode="510303" if regioncode=="510303" & year==2004

replace regioncode="510304" if regioncode=="510304" & year==2004

replace regioncode="510311" if regioncode=="510311" & year==2004

replace regioncode="510321" if regioncode=="510321" & year==2004

replace regioncode="510322" if regioncode=="510322" & year==2004

replace regioncode="510402" if regioncode=="510402" & year==2004

replace regioncode="510403" if regioncode=="510403" & year==2004

replace regioncode="510411" if regioncode=="510411" & year==2004

replace regioncode="510421" if regioncode=="510421" & year==2004

replace regioncode="510422" if regioncode=="510422" & year==2004

replace regioncode="510502" if regioncode=="510502" & year==2004

replace regioncode="510503" if regioncode=="510503" & year==2004

replace regioncode="510504" if regioncode=="510504" & year==2004

replace regioncode="510521" if regioncode=="510521" & year==2004

replace regioncode="510522" if regioncode=="510522" & year==2004

replace regioncode="510524" if regioncode=="510524" & year==2004

replace regioncode="510525" if regioncode=="510525" & year==2004

replace regioncode="510603" if regioncode=="510603" & year==2004

replace regioncode="510623" if regioncode=="510623" & year==2004

replace regioncode="510626" if regioncode=="510626" & year==2004

replace regioncode="510681" if regioncode=="510681" & year==2004

replace regioncode="510682" if regioncode=="510682" & year==2004

replace regioncode="510683" if regioncode=="510683" & year==2004

replace regioncode="510703" if regioncode=="510703" & year==2004

replace regioncode="510704" if regioncode=="510704" & year==2004

replace regioncode="510722" if regioncode=="510722" & year==2004

replace regioncode="510723" if regioncode=="510723" & year==2004

replace regioncode="510724" if regioncode=="510724" & year==2004

replace regioncode="510725" if regioncode=="510725" & year==2004

replace regioncode="510726" if regioncode=="510726" & year==2004

replace regioncode="510727" if regioncode=="510727" & year==2004

replace regioncode="510781" if regioncode=="510781" & year==2004

replace regioncode="510802" if regioncode=="510802" & year==2004

replace regioncode="510811" if regioncode=="510811" & year==2004

replace regioncode="510812" if regioncode=="510812" & year==2004

replace regioncode="510821" if regioncode=="510821" & year==2004

replace regioncode="510822" if regioncode=="510822" & year==2004

replace regioncode="510823" if regioncode=="510823" & year==2004

replace regioncode="510824" if regioncode=="510824" & year==2004

replace regioncode="510902" if regioncode=="510903" & year==2004

replace regioncode="510921" if regioncode=="510921" & year==2004

replace regioncode="510922" if regioncode=="510922" & year==2004

replace regioncode="510923" if regioncode=="510923" & year==2004

replace regioncode="511002" if regioncode=="511002" & year==2004

replace regioncode="511011" if regioncode=="511011" & year==2004

replace regioncode="511024" if regioncode=="511024" & year==2004

replace regioncode="511025" if regioncode=="511025" & year==2004

replace regioncode="511028" if regioncode=="511028" & year==2004

replace regioncode="511102" if regioncode=="511102" & year==2004

replace regioncode="511111" if regioncode=="511111" & year==2004

replace regioncode="511112" if regioncode=="511112" & year==2004

replace regioncode="511113" if regioncode=="511113" & year==2004

replace regioncode="511123" if regioncode=="511123" & year==2004

replace regioncode="511124" if regioncode=="511124" & year==2004

replace regioncode="511126" if regioncode=="511126" & year==2004

replace regioncode="511129" if regioncode=="511129" & year==2004

replace regioncode="511132" if regioncode=="511132" & year==2004

replace regioncode="511133" if regioncode=="511133" & year==2004

replace regioncode="511181" if regioncode=="511181" & year==2004

replace regioncode="511302" if regioncode=="511302" & year==2004

replace regioncode="511303" if regioncode=="511303" & year==2004

replace regioncode="511304" if regioncode=="511304" & year==2004

replace regioncode="511321" if regioncode=="511321" & year==2004

replace regioncode="511322" if regioncode=="511322" & year==2004

replace regioncode="511323" if regioncode=="511323" & year==2004

replace regioncode="511324" if regioncode=="511324" & year==2004

replace regioncode="511325" if regioncode=="511325" & year==2004

replace regioncode="511381" if regioncode=="511381" & year==2004

replace regioncode="511402" if regioncode=="511402" & year==2004

replace regioncode="511421" if regioncode=="511421" & year==2004

replace regioncode="511422" if regioncode=="511422" & year==2004

replace regioncode="511423" if regioncode=="511423" & year==2004

replace regioncode="511424" if regioncode=="511424" & year==2004

replace regioncode="511425" if regioncode=="511425" & year==2004

replace regioncode="511502" if regioncode=="511502" & year==2004

replace regioncode="511521" if regioncode=="511521" & year==2004

replace regioncode="511522" if regioncode=="511522" & year==2004

replace regioncode="511523" if regioncode=="511523" & year==2004

replace regioncode="511524" if regioncode=="511524" & year==2004

replace regioncode="511525" if regioncode=="511525" & year==2004

replace regioncode="511526" if regioncode=="511526" & year==2004

replace regioncode="511527" if regioncode=="511527" & year==2004

replace regioncode="511528" if regioncode=="511528" & year==2004

replace regioncode="511529" if regioncode=="511529" & year==2004

replace regioncode="511602" if regioncode=="511602" & year==2004

replace regioncode="511621" if regioncode=="511621" & year==2004

replace regioncode="511622" if regioncode=="511622" & year==2004

replace regioncode="511623" if regioncode=="511623" & year==2004

replace regioncode="511681" if regioncode=="511681" & year==2004

replace regioncode="511702" if regioncode=="511702" & year==2004

replace regioncode="511721" if regioncode=="511721" & year==2004

replace regioncode="511722" if regioncode=="511722" & year==2004

replace regioncode="511723" if regioncode=="511723" & year==2004

replace regioncode="511724" if regioncode=="511724" & year==2004

replace regioncode="511725" if regioncode=="511725" & year==2004

replace regioncode="511781" if regioncode=="511781" & year==2004

replace regioncode="511802" if regioncode=="511802" & year==2004

replace regioncode="511821" if regioncode=="511821" & year==2004

replace regioncode="511822" if regioncode=="511822" & year==2004

replace regioncode="511823" if regioncode=="511823" & year==2004

replace regioncode="511824" if regioncode=="511824" & year==2004

replace regioncode="511825" if regioncode=="511825" & year==2004

replace regioncode="511826" if regioncode=="511826" & year==2004

replace regioncode="511827" if regioncode=="511827" & year==2004

replace regioncode="511902" if regioncode=="511902" & year==2004

replace regioncode="511921" if regioncode=="511921" & year==2004

replace regioncode="511922" if regioncode=="511922" & year==2004

replace regioncode="511923" if regioncode=="511923" & year==2004

replace regioncode="512002" if regioncode=="512002" & year==2004

replace regioncode="512021" if regioncode=="512021" & year==2004

replace regioncode="512022" if regioncode=="512022" & year==2004

replace regioncode="512081" if regioncode=="512081" & year==2004

replace regioncode="513221" if regioncode=="513221" & year==2004

replace regioncode="513222" if regioncode=="513222" & year==2004

replace regioncode="513223" if regioncode=="513223" & year==2004

replace regioncode="513224" if regioncode=="513224" & year==2004

replace regioncode="513225" if regioncode=="513225" & year==2004

replace regioncode="513226" if regioncode=="513226" & year==2004

replace regioncode="513227" if regioncode=="513227" & year==2004

replace regioncode="513228" if regioncode=="513228" & year==2004

replace regioncode="513229" if regioncode=="513229" & year==2004

replace regioncode="513230" if regioncode=="513230" & year==2004

replace regioncode="513231" if regioncode=="513231" & year==2004

replace regioncode="513232" if regioncode=="513232" & year==2004

replace regioncode="513233" if regioncode=="513233" & year==2004

replace regioncode="513321" if regioncode=="513321" & year==2004

replace regioncode="513322" if regioncode=="513322" & year==2004

replace regioncode="513323" if regioncode=="513323" & year==2004

replace regioncode="513324" if regioncode=="513324" & year==2004

replace regioncode="513325" if regioncode=="513325" & year==2004

replace regioncode="513326" if regioncode=="513326" & year==2004

replace regioncode="513327" if regioncode=="513327" & year==2004

replace regioncode="513328" if regioncode=="513328" & year==2004

replace regioncode="513329" if regioncode=="513329" & year==2004

replace regioncode="513330" if regioncode=="513330" & year==2004

replace regioncode="513331" if regioncode=="513331" & year==2004

replace regioncode="513332" if regioncode=="513332" & year==2004

replace regioncode="513333" if regioncode=="513333" & year==2004

replace regioncode="513334" if regioncode=="513334" & year==2004

replace regioncode="513335" if regioncode=="513335" & year==2004

replace regioncode="513336" if regioncode=="513336" & year==2004

replace regioncode="513337" if regioncode=="513337" & year==2004

replace regioncode="513338" if regioncode=="513338" & year==2004

replace regioncode="513401" if regioncode=="513401" & year==2004

replace regioncode="513422" if regioncode=="513422" & year==2004

replace regioncode="513423" if regioncode=="513423" & year==2004

replace regioncode="513424" if regioncode=="513424" & year==2004

replace regioncode="513425" if regioncode=="513425" & year==2004

replace regioncode="513426" if regioncode=="513426" & year==2004

replace regioncode="513427" if regioncode=="513427" & year==2004

replace regioncode="513428" if regioncode=="513428" & year==2004

replace regioncode="513429" if regioncode=="513429" & year==2004

replace regioncode="513430" if regioncode=="513430" & year==2004

replace regioncode="513431" if regioncode=="513431" & year==2004

replace regioncode="513432" if regioncode=="513432" & year==2004

replace regioncode="513433" if regioncode=="513433" & year==2004

replace regioncode="513434" if regioncode=="513434" & year==2004

replace regioncode="513435" if regioncode=="513435" & year==2004

replace regioncode="513436" if regioncode=="513436" & year==2004

replace regioncode="513437" if regioncode=="513437" & year==2004

replace regioncode="520102" if regioncode=="520102" & year==2004

replace regioncode="520103" if regioncode=="520103" & year==2004

replace regioncode="520111" if regioncode=="520111" & year==2004

replace regioncode="520112" if regioncode=="520112" & year==2004

replace regioncode="520113" if regioncode=="520113" & year==2004

replace regioncode="520114" if regioncode=="520114" & year==2004

replace regioncode="520121" if regioncode=="520121" & year==2004

replace regioncode="520122" if regioncode=="520122" & year==2004

replace regioncode="520123" if regioncode=="520123" & year==2004

replace regioncode="520181" if regioncode=="520181" & year==2004

replace regioncode="520201" if regioncode=="520201" & year==2004

replace regioncode="520222" if regioncode=="520222" & year==2004

replace regioncode="520203" if regioncode=="520203" & year==2004

replace regioncode="520221" if regioncode=="520221" & year==2004

replace regioncode="520302" if regioncode=="520302" & year==2004

replace regioncode="520321" if regioncode=="520321" & year==2004

replace regioncode="520322" if regioncode=="520322" & year==2004

replace regioncode="520323" if regioncode=="520323" & year==2004

replace regioncode="520324" if regioncode=="520324" & year==2004

replace regioncode="520325" if regioncode=="520325" & year==2004

replace regioncode="520326" if regioncode=="520326" & year==2004

replace regioncode="520327" if regioncode=="520327" & year==2004

replace regioncode="520328" if regioncode=="520328" & year==2004

replace regioncode="520329" if regioncode=="520329" & year==2004

replace regioncode="520330" if regioncode=="520330" & year==2004

replace regioncode="520381" if regioncode=="520381" & year==2004

replace regioncode="520382" if regioncode=="520382" & year==2004

replace regioncode="520402" if regioncode=="520402" & year==2004

replace regioncode="520421" if regioncode=="520421" & year==2004

replace regioncode="520422" if regioncode=="520422" & year==2004

replace regioncode="520423" if regioncode=="520423" & year==2004

replace regioncode="520424" if regioncode=="520424" & year==2004

replace regioncode="520425" if regioncode=="520425" & year==2004

replace regioncode="522201" if regioncode=="522201" & year==2004

replace regioncode="522222" if regioncode=="522222" & year==2004

replace regioncode="522223" if regioncode=="522223" & year==2004

replace regioncode="522224" if regioncode=="522224" & year==2004

replace regioncode="522225" if regioncode=="522225" & year==2004

replace regioncode="522226" if regioncode=="522226" & year==2004

replace regioncode="522227" if regioncode=="522227" & year==2004

replace regioncode="522228" if regioncode=="522228" & year==2004

replace regioncode="522229" if regioncode=="522229" & year==2004

replace regioncode="522230" if regioncode=="522230" & year==2004

replace regioncode="522301" if regioncode=="522301" & year==2004

replace regioncode="522322" if regioncode=="522322" & year==2004

replace regioncode="522323" if regioncode=="522323" & year==2004

replace regioncode="522324" if regioncode=="522324" & year==2004

replace regioncode="522325" if regioncode=="522325" & year==2004

replace regioncode="522326" if regioncode=="522326" & year==2004

replace regioncode="522327" if regioncode=="522327" & year==2004

replace regioncode="522328" if regioncode=="522328" & year==2004

replace regioncode="522401" if regioncode=="522401" & year==2004

replace regioncode="522422" if regioncode=="522422" & year==2004

replace regioncode="522423" if regioncode=="522423" & year==2004

replace regioncode="522424" if regioncode=="522424" & year==2004

replace regioncode="522425" if regioncode=="522425" & year==2004

replace regioncode="522426" if regioncode=="522426" & year==2004

replace regioncode="522427" if regioncode=="522427" & year==2004

replace regioncode="522428" if regioncode=="522428" & year==2004

replace regioncode="522601" if regioncode=="522601" & year==2004

replace regioncode="522622" if regioncode=="522622" & year==2004

replace regioncode="522623" if regioncode=="522623" & year==2004

replace regioncode="522624" if regioncode=="522624" & year==2004

replace regioncode="522625" if regioncode=="522625" & year==2004

replace regioncode="522626" if regioncode=="522626" & year==2004

replace regioncode="522627" if regioncode=="522627" & year==2004

replace regioncode="522628" if regioncode=="522628" & year==2004

replace regioncode="522629" if regioncode=="522629" & year==2004

replace regioncode="522630" if regioncode=="522630" & year==2004

replace regioncode="522631" if regioncode=="522631" & year==2004

replace regioncode="522632" if regioncode=="522632" & year==2004

replace regioncode="522633" if regioncode=="522633" & year==2004

replace regioncode="522634" if regioncode=="522634" & year==2004

replace regioncode="522635" if regioncode=="522635" & year==2004

replace regioncode="522636" if regioncode=="522636" & year==2004

replace regioncode="522701" if regioncode=="522701" & year==2004

replace regioncode="522702" if regioncode=="522702" & year==2004

replace regioncode="522722" if regioncode=="522722" & year==2004

replace regioncode="522723" if regioncode=="522723" & year==2004

replace regioncode="522725" if regioncode=="522725" & year==2004

replace regioncode="522726" if regioncode=="522726" & year==2004

replace regioncode="522727" if regioncode=="522727" & year==2004

replace regioncode="522728" if regioncode=="522728" & year==2004

replace regioncode="522729" if regioncode=="522729" & year==2004

replace regioncode="522730" if regioncode=="522730" & year==2004

replace regioncode="522731" if regioncode=="522731" & year==2004

replace regioncode="522732" if regioncode=="522732" & year==2004

replace regioncode="530102" if regioncode=="530102" & year==2004

replace regioncode="530103" if regioncode=="530103" & year==2004

replace regioncode="530111" if regioncode=="530111" & year==2004

replace regioncode="530112" if regioncode=="530112" & year==2004

replace regioncode="530113" if regioncode=="530113" & year==2004

replace regioncode="530121" if regioncode=="530121" & year==2004

replace regioncode="530122" if regioncode=="530122" & year==2004

replace regioncode="530124" if regioncode=="530124" & year==2004

replace regioncode="530125" if regioncode=="530125" & year==2004

replace regioncode="530126" if regioncode=="530126" & year==2004

replace regioncode="530127" if regioncode=="530127" & year==2004

replace regioncode="530128" if regioncode=="530128" & year==2004

replace regioncode="530129" if regioncode=="530129" & year==2004

replace regioncode="530181" if regioncode=="530181" & year==2004

replace regioncode="530302" if regioncode=="530302" & year==2004

replace regioncode="530321" if regioncode=="530321" & year==2004

replace regioncode="530322" if regioncode=="530322" & year==2004

replace regioncode="530323" if regioncode=="530323" & year==2004

replace regioncode="530324" if regioncode=="530324" & year==2004

replace regioncode="530325" if regioncode=="530325" & year==2004

replace regioncode="530326" if regioncode=="530326" & year==2004

replace regioncode="530328" if regioncode=="530328" & year==2004

replace regioncode="530381" if regioncode=="530381" & year==2004

replace regioncode="530402" if regioncode=="530402" & year==2004

replace regioncode="530421" if regioncode=="530421" & year==2004

replace regioncode="530422" if regioncode=="530422" & year==2004

replace regioncode="530423" if regioncode=="530423" & year==2004

replace regioncode="530424" if regioncode=="530424" & year==2004

replace regioncode="530425" if regioncode=="530425" & year==2004

replace regioncode="530426" if regioncode=="530426" & year==2004

replace regioncode="530427" if regioncode=="530427" & year==2004

replace regioncode="530428" if regioncode=="530428" & year==2004

replace regioncode="530502" if regioncode=="530502" & year==2004

replace regioncode="530521" if regioncode=="530521" & year==2004

replace regioncode="530522" if regioncode=="530522" & year==2004

replace regioncode="530523" if regioncode=="530523" & year==2004

replace regioncode="530524" if regioncode=="530524" & year==2004

replace regioncode="530602" if regioncode=="530602" & year==2004

replace regioncode="530621" if regioncode=="530621" & year==2004

replace regioncode="530622" if regioncode=="530622" & year==2004

replace regioncode="530623" if regioncode=="530623" & year==2004

replace regioncode="530624" if regioncode=="530624" & year==2004

replace regioncode="530625" if regioncode=="530625" & year==2004

replace regioncode="530626" if regioncode=="530626" & year==2004

replace regioncode="530627" if regioncode=="530627" & year==2004

replace regioncode="530628" if regioncode=="530628" & year==2004

replace regioncode="530629" if regioncode=="530629" & year==2004

replace regioncode="530630" if regioncode=="530630" & year==2004

replace regioncode="530721" if regioncode=="530721" & year==2004

replace regioncode="530722" if regioncode=="530722" & year==2004

replace regioncode="530723" if regioncode=="530723" & year==2004

replace regioncode="530724" if regioncode=="530724" & year==2004

replace regioncode="532701" if regioncode=="530802" & year==2004

replace regioncode="532722" if regioncode=="530821" & year==2004

replace regioncode="532723" if regioncode=="530822" & year==2004

replace regioncode="532724" if regioncode=="530823" & year==2004

replace regioncode="532725" if regioncode=="530824" & year==2004

replace regioncode="532726" if regioncode=="530825" & year==2004

replace regioncode="532727" if regioncode=="530826" & year==2004

replace regioncode="532728" if regioncode=="530827" & year==2004

replace regioncode="532729" if regioncode=="530828" & year==2004

replace regioncode="532730" if regioncode=="530829" & year==2004

replace regioncode="533521" if regioncode=="530902" & year==2004

replace regioncode="533522" if regioncode=="530921" & year==2004

replace regioncode="533523" if regioncode=="530922" & year==2004

replace regioncode="533524" if regioncode=="530923" & year==2004

replace regioncode="533525" if regioncode=="530924" & year==2004

replace regioncode="533526" if regioncode=="530925" & year==2004

replace regioncode="533527" if regioncode=="530926" & year==2004

replace regioncode="533528" if regioncode=="530927" & year==2004

replace regioncode="532301" if regioncode=="532301" & year==2004

replace regioncode="532322" if regioncode=="532322" & year==2004

replace regioncode="532323" if regioncode=="532323" & year==2004

replace regioncode="532324" if regioncode=="532324" & year==2004

replace regioncode="532325" if regioncode=="532325" & year==2004

replace regioncode="532326" if regioncode=="532326" & year==2004

replace regioncode="532327" if regioncode=="532327" & year==2004

replace regioncode="532328" if regioncode=="532328" & year==2004

replace regioncode="532329" if regioncode=="532329" & year==2004

replace regioncode="532331" if regioncode=="532331" & year==2004

replace regioncode="532501" if regioncode=="532501" & year==2004

replace regioncode="532502" if regioncode=="532502" & year==2004

replace regioncode="532522" if regioncode=="532522" & year==2004

replace regioncode="532523" if regioncode=="532523" & year==2004

replace regioncode="532524" if regioncode=="532524" & year==2004

replace regioncode="532525" if regioncode=="532525" & year==2004

replace regioncode="532526" if regioncode=="532526" & year==2004

replace regioncode="532527" if regioncode=="532527" & year==2004

replace regioncode="532528" if regioncode=="532528" & year==2004

replace regioncode="532529" if regioncode=="532529" & year==2004

replace regioncode="532530" if regioncode=="532530" & year==2004

replace regioncode="532531" if regioncode=="532531" & year==2004

replace regioncode="532532" if regioncode=="532532" & year==2004

replace regioncode="532621" if regioncode=="532621" & year==2004

replace regioncode="532622" if regioncode=="532622" & year==2004

replace regioncode="532623" if regioncode=="532623" & year==2004

replace regioncode="532624" if regioncode=="532624" & year==2004

replace regioncode="532625" if regioncode=="532625" & year==2004

replace regioncode="532626" if regioncode=="532626" & year==2004

replace regioncode="532627" if regioncode=="532627" & year==2004

replace regioncode="532628" if regioncode=="532628" & year==2004

replace regioncode="532801" if regioncode=="532801" & year==2004

replace regioncode="532822" if regioncode=="532822" & year==2004

replace regioncode="532823" if regioncode=="532823" & year==2004

replace regioncode="532901" if regioncode=="532901" & year==2004

replace regioncode="532922" if regioncode=="532922" & year==2004

replace regioncode="532923" if regioncode=="532923" & year==2004

replace regioncode="532924" if regioncode=="532924" & year==2004

replace regioncode="532925" if regioncode=="532925" & year==2004

replace regioncode="532926" if regioncode=="532926" & year==2004

replace regioncode="532927" if regioncode=="532927" & year==2004

replace regioncode="532928" if regioncode=="532928" & year==2004

replace regioncode="532929" if regioncode=="532929" & year==2004

replace regioncode="532930" if regioncode=="532930" & year==2004

replace regioncode="532931" if regioncode=="532931" & year==2004

replace regioncode="532932" if regioncode=="532932" & year==2004

replace regioncode="533102" if regioncode=="533102" & year==2004

replace regioncode="533103" if regioncode=="533103" & year==2004

replace regioncode="533122" if regioncode=="533122" & year==2004

replace regioncode="533123" if regioncode=="533123" & year==2004

replace regioncode="533124" if regioncode=="533124" & year==2004

replace regioncode="533321" if regioncode=="533321" & year==2004

replace regioncode="533323" if regioncode=="533323" & year==2004

replace regioncode="533324" if regioncode=="533324" & year==2004

replace regioncode="533325" if regioncode=="533325" & year==2004

replace regioncode="533421" if regioncode=="533421" & year==2004

replace regioncode="533422" if regioncode=="533422" & year==2004

replace regioncode="533423" if regioncode=="533423" & year==2004

replace regioncode="540102" if regioncode=="540102" & year==2004

replace regioncode="540121" if regioncode=="540121" & year==2004

replace regioncode="540122" if regioncode=="540122" & year==2004

replace regioncode="540123" if regioncode=="540123" & year==2004

replace regioncode="540124" if regioncode=="540124" & year==2004

replace regioncode="540125" if regioncode=="540125" & year==2004

replace regioncode="540126" if regioncode=="540126" & year==2004

replace regioncode="540127" if regioncode=="540127" & year==2004

replace regioncode="542121" if regioncode=="542121" & year==2004

replace regioncode="542122" if regioncode=="542122" & year==2004

replace regioncode="542123" if regioncode=="542123" & year==2004

replace regioncode="542124" if regioncode=="542124" & year==2004

replace regioncode="542125" if regioncode=="542125" & year==2004

replace regioncode="542126" if regioncode=="542126" & year==2004

replace regioncode="542127" if regioncode=="542127" & year==2004

replace regioncode="542128" if regioncode=="542128" & year==2004

replace regioncode="542129" if regioncode=="542129" & year==2004

replace regioncode="542132" if regioncode=="542132" & year==2004

replace regioncode="542133" if regioncode=="542133" & year==2004

replace regioncode="542221" if regioncode=="542221" & year==2004

replace regioncode="542223" if regioncode=="542223" & year==2004

replace regioncode="542224" if regioncode=="542224" & year==2004

replace regioncode="542225" if regioncode=="542225" & year==2004

replace regioncode="542227" if regioncode=="542227" & year==2004

replace regioncode="542228" if regioncode=="542228" & year==2004

replace regioncode="542229" if regioncode=="542229" & year==2004

replace regioncode="542231" if regioncode=="542231" & year==2004

replace regioncode="542232" if regioncode=="542232" & year==2004

replace regioncode="542233" if regioncode=="542233" & year==2004

replace regioncode="542301" if regioncode=="542301" & year==2004

replace regioncode="542322" if regioncode=="542322" & year==2004

replace regioncode="542323" if regioncode=="542323" & year==2004

replace regioncode="542324" if regioncode=="542324" & year==2004

replace regioncode="542325" if regioncode=="542325" & year==2004

replace regioncode="542326" if regioncode=="542326" & year==2004

replace regioncode="542327" if regioncode=="542327" & year==2004

replace regioncode="542328" if regioncode=="542328" & year==2004

replace regioncode="542329" if regioncode=="542329" & year==2004

replace regioncode="542330" if regioncode=="542330" & year==2004

replace regioncode="542331" if regioncode=="542331" & year==2004

replace regioncode="542332" if regioncode=="542332" & year==2004

replace regioncode="542334" if regioncode=="542334" & year==2004

replace regioncode="542336" if regioncode=="542336" & year==2004

replace regioncode="542338" if regioncode=="542338" & year==2004

replace regioncode="542421" if regioncode=="542421" & year==2004

replace regioncode="542422" if regioncode=="542422" & year==2004

replace regioncode="542423" if regioncode=="542423" & year==2004

replace regioncode="542424" if regioncode=="542424" & year==2004

replace regioncode="542425" if regioncode=="542425" & year==2004

replace regioncode="542426" if regioncode=="542426" & year==2004

replace regioncode="542427" if regioncode=="542427" & year==2004

replace regioncode="542429" if regioncode=="542429" & year==2004

replace regioncode="542430" if regioncode=="542430" & year==2004

replace regioncode="542523" if regioncode=="542523" & year==2004

replace regioncode="542621" if regioncode=="542621" & year==2004

replace regioncode="542622" if regioncode=="542622" & year==2004

replace regioncode="542623" if regioncode=="542623" & year==2004

replace regioncode="542625" if regioncode=="542625" & year==2004

replace regioncode="542626" if regioncode=="542626" & year==2004

replace regioncode="610102" if regioncode=="610102" & year==2004

replace regioncode="610103" if regioncode=="610103" & year==2004

replace regioncode="610104" if regioncode=="610104" & year==2004

replace regioncode="610111" if regioncode=="610111" & year==2004

replace regioncode="610112" if regioncode=="610112" & year==2004

replace regioncode="610113" if regioncode=="610113" & year==2004

replace regioncode="610114" if regioncode=="610114" & year==2004

replace regioncode="610115" if regioncode=="610115" & year==2004

replace regioncode="610116" if regioncode=="610116" & year==2004

replace regioncode="610122" if regioncode=="610122" & year==2004

replace regioncode="610124" if regioncode=="610124" & year==2004

replace regioncode="610125" if regioncode=="610125" & year==2004

replace regioncode="610126" if regioncode=="610126" & year==2004

replace regioncode="610202" if regioncode=="610202" & year==2004

replace regioncode="610203" if regioncode=="610203" & year==2004

replace regioncode="610204" if regioncode=="610204" & year==2004

replace regioncode="610222" if regioncode=="610222" & year==2004

replace regioncode="610302" if regioncode=="610302" & year==2004

replace regioncode="610303" if regioncode=="610303" & year==2004

replace regioncode="610304" if regioncode=="610304" & year==2004

replace regioncode="610322" if regioncode=="610322" & year==2004

replace regioncode="610323" if regioncode=="610323" & year==2004

replace regioncode="610324" if regioncode=="610324" & year==2004

replace regioncode="610326" if regioncode=="610326" & year==2004

replace regioncode="610327" if regioncode=="610327" & year==2004

replace regioncode="610328" if regioncode=="610328" & year==2004

replace regioncode="610329" if regioncode=="610329" & year==2004

replace regioncode="610330" if regioncode=="610330" & year==2004

replace regioncode="610331" if regioncode=="610331" & year==2004

replace regioncode="610402" if regioncode=="610402" & year==2004

replace regioncode="610403" if regioncode=="610403" & year==2004

replace regioncode="610404" if regioncode=="610404" & year==2004

replace regioncode="610422" if regioncode=="610422" & year==2004

replace regioncode="610423" if regioncode=="610423" & year==2004

replace regioncode="610424" if regioncode=="610424" & year==2004

replace regioncode="610425" if regioncode=="610425" & year==2004

replace regioncode="610426" if regioncode=="610426" & year==2004

replace regioncode="610427" if regioncode=="610427" & year==2004

replace regioncode="610428" if regioncode=="610428" & year==2004

replace regioncode="610429" if regioncode=="610429" & year==2004

replace regioncode="610430" if regioncode=="610430" & year==2004

replace regioncode="610431" if regioncode=="610431" & year==2004

replace regioncode="610481" if regioncode=="610481" & year==2004

replace regioncode="610502" if regioncode=="610502" & year==2004

replace regioncode="610521" if regioncode=="610521" & year==2004

replace regioncode="610522" if regioncode=="610522" & year==2004

replace regioncode="610523" if regioncode=="610523" & year==2004

replace regioncode="610524" if regioncode=="610524" & year==2004

replace regioncode="610525" if regioncode=="610525" & year==2004

replace regioncode="610526" if regioncode=="610526" & year==2004

replace regioncode="610527" if regioncode=="610527" & year==2004

replace regioncode="610528" if regioncode=="610528" & year==2004

replace regioncode="610581" if regioncode=="610581" & year==2004

replace regioncode="610582" if regioncode=="610582" & year==2004

replace regioncode="610602" if regioncode=="610602" & year==2004

replace regioncode="610621" if regioncode=="610621" & year==2004

replace regioncode="610622" if regioncode=="610622" & year==2004

replace regioncode="610623" if regioncode=="610623" & year==2004

replace regioncode="610624" if regioncode=="610624" & year==2004

replace regioncode="610625" if regioncode=="610625" & year==2004

replace regioncode="610626" if regioncode=="610626" & year==2004

replace regioncode="610627" if regioncode=="610627" & year==2004

replace regioncode="610628" if regioncode=="610628" & year==2004

replace regioncode="610629" if regioncode=="610629" & year==2004

replace regioncode="610630" if regioncode=="610630" & year==2004

replace regioncode="610631" if regioncode=="610631" & year==2004

replace regioncode="610632" if regioncode=="610632" & year==2004

replace regioncode="610702" if regioncode=="610702" & year==2004

replace regioncode="610721" if regioncode=="610721" & year==2004

replace regioncode="610722" if regioncode=="610722" & year==2004

replace regioncode="610723" if regioncode=="610723" & year==2004

replace regioncode="610724" if regioncode=="610724" & year==2004

replace regioncode="610725" if regioncode=="610725" & year==2004

replace regioncode="610726" if regioncode=="610726" & year==2004

replace regioncode="610727" if regioncode=="610727" & year==2004

replace regioncode="610728" if regioncode=="610728" & year==2004

replace regioncode="610729" if regioncode=="610729" & year==2004

replace regioncode="610730" if regioncode=="610730" & year==2004

replace regioncode="610802" if regioncode=="610802" & year==2004

replace regioncode="610821" if regioncode=="610821" & year==2004

replace regioncode="610822" if regioncode=="610822" & year==2004

replace regioncode="610823" if regioncode=="610823" & year==2004

replace regioncode="610824" if regioncode=="610824" & year==2004

replace regioncode="610825" if regioncode=="610825" & year==2004

replace regioncode="610826" if regioncode=="610826" & year==2004

replace regioncode="610827" if regioncode=="610827" & year==2004

replace regioncode="610828" if regioncode=="610828" & year==2004

replace regioncode="610829" if regioncode=="610829" & year==2004

replace regioncode="610830" if regioncode=="610830" & year==2004

replace regioncode="610831" if regioncode=="610831" & year==2004

replace regioncode="610902" if regioncode=="610902" & year==2004

replace regioncode="610921" if regioncode=="610921" & year==2004

replace regioncode="610922" if regioncode=="610922" & year==2004

replace regioncode="610923" if regioncode=="610923" & year==2004

replace regioncode="610924" if regioncode=="610924" & year==2004

replace regioncode="610925" if regioncode=="610925" & year==2004

replace regioncode="610926" if regioncode=="610926" & year==2004

replace regioncode="610927" if regioncode=="610927" & year==2004

replace regioncode="610928" if regioncode=="610928" & year==2004

replace regioncode="610929" if regioncode=="610929" & year==2004

replace regioncode="611002" if regioncode=="611002" & year==2004

replace regioncode="611021" if regioncode=="611021" & year==2004

replace regioncode="611022" if regioncode=="611022" & year==2004

replace regioncode="611023" if regioncode=="611023" & year==2004

replace regioncode="611024" if regioncode=="611024" & year==2004

replace regioncode="611025" if regioncode=="611025" & year==2004

replace regioncode="611026" if regioncode=="611026" & year==2004

replace regioncode="620102" if regioncode=="620102" & year==2004

replace regioncode="620103" if regioncode=="620103" & year==2004

replace regioncode="620104" if regioncode=="620104" & year==2004

replace regioncode="620105" if regioncode=="620105" & year==2004

replace regioncode="620111" if regioncode=="620111" & year==2004

replace regioncode="620121" if regioncode=="620121" & year==2004

replace regioncode="620122" if regioncode=="620122" & year==2004

replace regioncode="620123" if regioncode=="620123" & year==2004

replace regioncode="620201" if regioncode=="620201" & year==2004

replace regioncode="620302" if regioncode=="620302" & year==2004

replace regioncode="620321" if regioncode=="620321" & year==2004

replace regioncode="620402" if regioncode=="620402" & year==2004

replace regioncode="620403" if regioncode=="620403" & year==2004

replace regioncode="620421" if regioncode=="620421" & year==2004

replace regioncode="620422" if regioncode=="620422" & year==2004

replace regioncode="620423" if regioncode=="620423" & year==2004

replace regioncode="620502" if regioncode=="620502" & year==2004

replace regioncode="620503" if regioncode=="620503" & year==2004

replace regioncode="620521" if regioncode=="620521" & year==2004

replace regioncode="620522" if regioncode=="620522" & year==2004

replace regioncode="620523" if regioncode=="620523" & year==2004

replace regioncode="620524" if regioncode=="620524" & year==2004

replace regioncode="620525" if regioncode=="620525" & year==2004

replace regioncode="622301" if regioncode=="620602" & year==2004

replace regioncode="622322" if regioncode=="620621" & year==2004

replace regioncode="622323" if regioncode=="620622" & year==2004

replace regioncode="622326" if regioncode=="620623" & year==2004

replace regioncode="622201" if regioncode=="620702" & year==2004

replace regioncode="622222" if regioncode=="620721" & year==2004

replace regioncode="622223" if regioncode=="620722" & year==2004

replace regioncode="622224" if regioncode=="620723" & year==2004

replace regioncode="622225" if regioncode=="620724" & year==2004

replace regioncode="622226" if regioncode=="620725" & year==2004

replace regioncode="622701" if regioncode=="620802" & year==2004

replace regioncode="622722" if regioncode=="620821" & year==2004

replace regioncode="622723" if regioncode=="620822" & year==2004

replace regioncode="622724" if regioncode=="620823" & year==2004

replace regioncode="622725" if regioncode=="620824" & year==2004

replace regioncode="622726" if regioncode=="620825" & year==2004

replace regioncode="622727" if regioncode=="620826" & year==2004

replace regioncode="622102" if regioncode=="620902" & year==2004

replace regioncode="622123" if regioncode=="620921" & year==2004

replace regioncode="622126" if regioncode=="620922" & year==2004

replace regioncode="622124" if regioncode=="620923" & year==2004

replace regioncode="622125" if regioncode=="620924" & year==2004

replace regioncode="622101" if regioncode=="620981" & year==2004

replace regioncode="622103" if regioncode=="620982" & year==2004

replace regioncode="622801" if regioncode=="621002" & year==2004

replace regioncode="622821" if regioncode=="621021" & year==2004

replace regioncode="622822" if regioncode=="621022" & year==2004

replace regioncode="622823" if regioncode=="621023" & year==2004

replace regioncode="622824" if regioncode=="621024" & year==2004

replace regioncode="622825" if regioncode=="621025" & year==2004

replace regioncode="622826" if regioncode=="621026" & year==2004

replace regioncode="622827" if regioncode=="621027" & year==2004

replace regioncode="622421" if regioncode=="621102" & year==2004

replace regioncode="622424" if regioncode=="621121" & year==2004

replace regioncode="622425" if regioncode=="621122" & year==2004

replace regioncode="622426" if regioncode=="621123" & year==2004

replace regioncode="622427" if regioncode=="621124" & year==2004

replace regioncode="622428" if regioncode=="621125" & year==2004

replace regioncode="622429" if regioncode=="621126" & year==2004

replace regioncode="622621" if regioncode=="621202" & year==2004

replace regioncode="622624" if regioncode=="621221" & year==2004

replace regioncode="622626" if regioncode=="621222" & year==2004

replace regioncode="622623" if regioncode=="621223" & year==2004

replace regioncode="622625" if regioncode=="621224" & year==2004

replace regioncode="622627" if regioncode=="621225" & year==2004

replace regioncode="622628" if regioncode=="621226" & year==2004

replace regioncode="622630" if regioncode=="621227" & year==2004

replace regioncode="622629" if regioncode=="621228" & year==2004

replace regioncode="622901" if regioncode=="622901" & year==2004

replace regioncode="622921" if regioncode=="622921" & year==2004

replace regioncode="622922" if regioncode=="622922" & year==2004

replace regioncode="622923" if regioncode=="622923" & year==2004

replace regioncode="622924" if regioncode=="622924" & year==2004

replace regioncode="622925" if regioncode=="622925" & year==2004

replace regioncode="622926" if regioncode=="622926" & year==2004

replace regioncode="622927" if regioncode=="622927" & year==2004

replace regioncode="623001" if regioncode=="623001" & year==2004

replace regioncode="623021" if regioncode=="623021" & year==2004

replace regioncode="623022" if regioncode=="623022" & year==2004

replace regioncode="623023" if regioncode=="623023" & year==2004

replace regioncode="623024" if regioncode=="623024" & year==2004

replace regioncode="623025" if regioncode=="623025" & year==2004

replace regioncode="623026" if regioncode=="623026" & year==2004

replace regioncode="623027" if regioncode=="623027" & year==2004

replace regioncode="630102" if regioncode=="630102" & year==2004

replace regioncode="630103" if regioncode=="630103" & year==2004

replace regioncode="630104" if regioncode=="630104" & year==2004

replace regioncode="630105" if regioncode=="630105" & year==2004

replace regioncode="630121" if regioncode=="630121" & year==2004

replace regioncode="630122" if regioncode=="630122" & year==2004

replace regioncode="630123" if regioncode=="630123" & year==2004

replace regioncode="632121" if regioncode=="632121" & year==2004

replace regioncode="632122" if regioncode=="632122" & year==2004

replace regioncode="632123" if regioncode=="632123" & year==2004

replace regioncode="632126" if regioncode=="632126" & year==2004

replace regioncode="632127" if regioncode=="632127" & year==2004

replace regioncode="632128" if regioncode=="632128" & year==2004

replace regioncode="632221" if regioncode=="632221" & year==2004

replace regioncode="632222" if regioncode=="632222" & year==2004

replace regioncode="632223" if regioncode=="632223" & year==2004

replace regioncode="632224" if regioncode=="632224" & year==2004

replace regioncode="632321" if regioncode=="632321" & year==2004

replace regioncode="632322" if regioncode=="632322" & year==2004

replace regioncode="632521" if regioncode=="632521" & year==2004

replace regioncode="632522" if regioncode=="632522" & year==2004

replace regioncode="632523" if regioncode=="632523" & year==2004

replace regioncode="632524" if regioncode=="632524" & year==2004

replace regioncode="632525" if regioncode=="632525" & year==2004

replace regioncode="632621" if regioncode=="632621" & year==2004

replace regioncode="632622" if regioncode=="632622" & year==2004

replace regioncode="632623" if regioncode=="632623" & year==2004

replace regioncode="632624" if regioncode=="632624" & year==2004

replace regioncode="632625" if regioncode=="632625" & year==2004

replace regioncode="632626" if regioncode=="632626" & year==2004

replace regioncode="632721" if regioncode=="632721" & year==2004

replace regioncode="632723" if regioncode=="632723" & year==2004

replace regioncode="632724" if regioncode=="632724" & year==2004

replace regioncode="632725" if regioncode=="632725" & year==2004

replace regioncode="632726" if regioncode=="632726" & year==2004

replace regioncode="632801" if regioncode=="632801" & year==2004

replace regioncode="632802" if regioncode=="632802" & year==2004

replace regioncode="632821" if regioncode=="632821" & year==2004

replace regioncode="632822" if regioncode=="632822" & year==2004

replace regioncode="640104" if regioncode=="640104" & year==2004

replace regioncode="640105" if regioncode=="640105" & year==2004

replace regioncode="640106" if regioncode=="640106" & year==2004

replace regioncode="640121" if regioncode=="640121" & year==2004

replace regioncode="640122" if regioncode=="640122" & year==2004

replace regioncode="640181" if regioncode=="640181" & year==2004

replace regioncode="640202" if regioncode=="640202" & year==2004

replace regioncode="640203" if regioncode=="640205" & year==2004

replace regioncode="640221" if regioncode=="640221" & year==2004

replace regioncode="640302" if regioncode=="640302" & year==2004

replace regioncode="640321" if regioncode=="640501" & year==2004

replace regioncode="640322" if regioncode=="640521" & year==2004

replace regioncode="640323" if regioncode=="640323" & year==2004

replace regioncode="640324" if regioncode=="640324" & year==2004

replace regioncode="640381" if regioncode=="640381" & year==2004

replace regioncode="640402" if regioncode=="640402" & year==2004

replace regioncode="640421" if regioncode=="640522" & year==2004

replace regioncode="640422" if regioncode=="640422" & year==2004

replace regioncode="640423" if regioncode=="640423" & year==2004

replace regioncode="640424" if regioncode=="640424" & year==2004

replace regioncode="640425" if regioncode=="640425" & year==2004

replace regioncode="650102" if regioncode=="650102" & year==2004

replace regioncode="650103" if regioncode=="650103" & year==2004

replace regioncode="650104" if regioncode=="650104" & year==2004

replace regioncode="650105" if regioncode=="650105" & year==2004

replace regioncode="650106" if regioncode=="650106" & year==2004

replace regioncode="650107" if regioncode=="650107" & year==2004

replace regioncode="650108" if regioncode=="650108" & year==2004

replace regioncode="650121" if regioncode=="650121" & year==2004

replace regioncode="650202" if regioncode=="650202" & year==2004

replace regioncode="650203" if regioncode=="650203" & year==2004

replace regioncode="650204" if regioncode=="650204" & year==2004

replace regioncode="650205" if regioncode=="650205" & year==2004

replace regioncode="652101" if regioncode=="652101" & year==2004

replace regioncode="652122" if regioncode=="652122" & year==2004

replace regioncode="652123" if regioncode=="652123" & year==2004

replace regioncode="652201" if regioncode=="652201" & year==2004

replace regioncode="652222" if regioncode=="652222" & year==2004

replace regioncode="652223" if regioncode=="652223" & year==2004

replace regioncode="652301" if regioncode=="652301" & year==2004

replace regioncode="652302" if regioncode=="652302" & year==2004

replace regioncode="652303" if regioncode=="652303" & year==2004

replace regioncode="652323" if regioncode=="652323" & year==2004

replace regioncode="652324" if regioncode=="652324" & year==2004

replace regioncode="652325" if regioncode=="652325" & year==2004

replace regioncode="652327" if regioncode=="652327" & year==2004

replace regioncode="652328" if regioncode=="652328" & year==2004

replace regioncode="652701" if regioncode=="652701" & year==2004

replace regioncode="652722" if regioncode=="652722" & year==2004

replace regioncode="652723" if regioncode=="652723" & year==2004

replace regioncode="652801" if regioncode=="652801" & year==2004

replace regioncode="652822" if regioncode=="652822" & year==2004

replace regioncode="652823" if regioncode=="652823" & year==2004

replace regioncode="652824" if regioncode=="652824" & year==2004

replace regioncode="652825" if regioncode=="652825" & year==2004

replace regioncode="652826" if regioncode=="652826" & year==2004

replace regioncode="652827" if regioncode=="652827" & year==2004

replace regioncode="652828" if regioncode=="652828" & year==2004

replace regioncode="652829" if regioncode=="652829" & year==2004

replace regioncode="652901" if regioncode=="652901" & year==2004

replace regioncode="652922" if regioncode=="652922" & year==2004

replace regioncode="652923" if regioncode=="652923" & year==2004

replace regioncode="652924" if regioncode=="652924" & year==2004

replace regioncode="652925" if regioncode=="652925" & year==2004

replace regioncode="652926" if regioncode=="652926" & year==2004

replace regioncode="652928" if regioncode=="652928" & year==2004

replace regioncode="652929" if regioncode=="652929" & year==2004

replace regioncode="653001" if regioncode=="653001" & year==2004

replace regioncode="653022" if regioncode=="653022" & year==2004

replace regioncode="653023" if regioncode=="653023" & year==2004

replace regioncode="653024" if regioncode=="653024" & year==2004

replace regioncode="653101" if regioncode=="653101" & year==2004

replace regioncode="653121" if regioncode=="653121" & year==2004

replace regioncode="653122" if regioncode=="653122" & year==2004

replace regioncode="653123" if regioncode=="653123" & year==2004

replace regioncode="653124" if regioncode=="653124" & year==2004

replace regioncode="653125" if regioncode=="653125" & year==2004

replace regioncode="653126" if regioncode=="653126" & year==2004

replace regioncode="653127" if regioncode=="653127" & year==2004

replace regioncode="653128" if regioncode=="653128" & year==2004

replace regioncode="653129" if regioncode=="653129" & year==2004

replace regioncode="653130" if regioncode=="653130" & year==2004

replace regioncode="653131" if regioncode=="653131" & year==2004

replace regioncode="653201" if regioncode=="653201" & year==2004

replace regioncode="653221" if regioncode=="653221" & year==2004

replace regioncode="653222" if regioncode=="653222" & year==2004

replace regioncode="653223" if regioncode=="653223" & year==2004

replace regioncode="653224" if regioncode=="653224" & year==2004

replace regioncode="653225" if regioncode=="653225" & year==2004

replace regioncode="653226" if regioncode=="653226" & year==2004

replace regioncode="653227" if regioncode=="653227" & year==2004

replace regioncode="654002" if regioncode=="654002" & year==2004

replace regioncode="654003" if regioncode=="654003" & year==2004

replace regioncode="654021" if regioncode=="654021" & year==2004

replace regioncode="654022" if regioncode=="654022" & year==2004

replace regioncode="654023" if regioncode=="654023" & year==2004

replace regioncode="654024" if regioncode=="654024" & year==2004

replace regioncode="654025" if regioncode=="654025" & year==2004

replace regioncode="654026" if regioncode=="654026" & year==2004

replace regioncode="654027" if regioncode=="654027" & year==2004

replace regioncode="654028" if regioncode=="654028" & year==2004

replace regioncode="654201" if regioncode=="654201" & year==2004

replace regioncode="654202" if regioncode=="654202" & year==2004

replace regioncode="654221" if regioncode=="654221" & year==2004

replace regioncode="654223" if regioncode=="654223" & year==2004

replace regioncode="654224" if regioncode=="654224" & year==2004

replace regioncode="654225" if regioncode=="654225" & year==2004

replace regioncode="654226" if regioncode=="654226" & year==2004

replace regioncode="654301" if regioncode=="654301" & year==2004

replace regioncode="654321" if regioncode=="654321" & year==2004

replace regioncode="654322" if regioncode=="654322" & year==2004

replace regioncode="654323" if regioncode=="654323" & year==2004

replace regioncode="654324" if regioncode=="654324" & year==2004

replace regioncode="654325" if regioncode=="654325" & year==2004

replace regioncode="654326" if regioncode=="654326" & year==2004

replace regioncode="659001" if regioncode=="659001" & year==2004

replace regioncode="110101" if regioncode=="110101" & year==2005

replace regioncode="110102" if regioncode=="110102" & year==2005

replace regioncode="110103" if regioncode=="110103" & year==2005

replace regioncode="110104" if regioncode=="110104" & year==2005

replace regioncode="110105" if regioncode=="110105" & year==2005

replace regioncode="110106" if regioncode=="110106" & year==2005

replace regioncode="110107" if regioncode=="110107" & year==2005

replace regioncode="110108" if regioncode=="110108" & year==2005

replace regioncode="110109" if regioncode=="110109" & year==2005

replace regioncode="110111" if regioncode=="110111" & year==2005

replace regioncode="110112" if regioncode=="110112" & year==2005

replace regioncode="110113" if regioncode=="110113" & year==2005

replace regioncode="110114" if regioncode=="110114" & year==2005

replace regioncode="110115" if regioncode=="110115" & year==2005

replace regioncode="110117" if regioncode=="110117" & year==2005

replace regioncode="110116" if regioncode=="110116" & year==2005

replace regioncode="110228" if regioncode=="110228" & year==2005

replace regioncode="110229" if regioncode=="110229" & year==2005

replace regioncode="120101" if regioncode=="120101" & year==2005

replace regioncode="120102" if regioncode=="120102" & year==2005

replace regioncode="120103" if regioncode=="120103" & year==2005

replace regioncode="120104" if regioncode=="120104" & year==2005

replace regioncode="120105" if regioncode=="120105" & year==2005

replace regioncode="120106" if regioncode=="120106" & year==2005

replace regioncode="120107" if regioncode=="120107" & year==2005

replace regioncode="120108" if regioncode=="120108" & year==2005

replace regioncode="120109" if regioncode=="120109" & year==2005

replace regioncode="120110" if regioncode=="120110" & year==2005

replace regioncode="120111" if regioncode=="120111" & year==2005

replace regioncode="120112" if regioncode=="120112" & year==2005

replace regioncode="120113" if regioncode=="120113" & year==2005

replace regioncode="120114" if regioncode=="120114" & year==2005

replace regioncode="120115" if regioncode=="120115" & year==2005

replace regioncode="120221" if regioncode=="120221" & year==2005

replace regioncode="120223" if regioncode=="120223" & year==2005

replace regioncode="120225" if regioncode=="120225" & year==2005

replace regioncode="130102" if regioncode=="130102" & year==2005

replace regioncode="130103" if regioncode=="130103" & year==2005

replace regioncode="130104" if regioncode=="130104" & year==2005

replace regioncode="130105" if regioncode=="130105" & year==2005

replace regioncode="130107" if regioncode=="130107" & year==2005

replace regioncode="130108" if regioncode=="130108" & year==2005

replace regioncode="130121" if regioncode=="130121" & year==2005

replace regioncode="130123" if regioncode=="130123" & year==2005

replace regioncode="130124" if regioncode=="130124" & year==2005

replace regioncode="130125" if regioncode=="130125" & year==2005

replace regioncode="130126" if regioncode=="130126" & year==2005

replace regioncode="130127" if regioncode=="130127" & year==2005

replace regioncode="130128" if regioncode=="130128" & year==2005

replace regioncode="130129" if regioncode=="130129" & year==2005

replace regioncode="130130" if regioncode=="130130" & year==2005

replace regioncode="130131" if regioncode=="130131" & year==2005

replace regioncode="130132" if regioncode=="130132" & year==2005

replace regioncode="130133" if regioncode=="130133" & year==2005

replace regioncode="130181" if regioncode=="130181" & year==2005

replace regioncode="130182" if regioncode=="130182" & year==2005

replace regioncode="130183" if regioncode=="130183" & year==2005

replace regioncode="130184" if regioncode=="130184" & year==2005

replace regioncode="130185" if regioncode=="130185" & year==2005

replace regioncode="130202" if regioncode=="130202" & year==2005

replace regioncode="130203" if regioncode=="130203" & year==2005

replace regioncode="130204" if regioncode=="130204" & year==2005

replace regioncode="130205" if regioncode=="130205" & year==2005

replace regioncode="130208" if regioncode=="130208" & year==2005

replace regioncode="130223" if regioncode=="130223" & year==2005

replace regioncode="130224" if regioncode=="130224" & year==2005

replace regioncode="130225" if regioncode=="130225" & year==2005

replace regioncode="130227" if regioncode=="130227" & year==2005

replace regioncode="130229" if regioncode=="130229" & year==2005

replace regioncode="130230" if regioncode=="130230" & year==2005

replace regioncode="130281" if regioncode=="130281" & year==2005

replace regioncode="130207" if regioncode=="130207" & year==2005

replace regioncode="130283" if regioncode=="130283" & year==2005

replace regioncode="130302" if regioncode=="130302" & year==2005

replace regioncode="130303" if regioncode=="130303" & year==2005

replace regioncode="130304" if regioncode=="130304" & year==2005

replace regioncode="130321" if regioncode=="130321" & year==2005

replace regioncode="130322" if regioncode=="130322" & year==2005

replace regioncode="130323" if regioncode=="130323" & year==2005

replace regioncode="130324" if regioncode=="130324" & year==2005

replace regioncode="130402" if regioncode=="130402" & year==2005

replace regioncode="130403" if regioncode=="130403" & year==2005

replace regioncode="130404" if regioncode=="130404" & year==2005

replace regioncode="130406" if regioncode=="130406" & year==2005

replace regioncode="130421" if regioncode=="130421" & year==2005

replace regioncode="130423" if regioncode=="130423" & year==2005

replace regioncode="130424" if regioncode=="130424" & year==2005

replace regioncode="130425" if regioncode=="130425" & year==2005

replace regioncode="130426" if regioncode=="130426" & year==2005

replace regioncode="130427" if regioncode=="130427" & year==2005

replace regioncode="130428" if regioncode=="130428" & year==2005

replace regioncode="130429" if regioncode=="130429" & year==2005

replace regioncode="130430" if regioncode=="130430" & year==2005

replace regioncode="130431" if regioncode=="130431" & year==2005

replace regioncode="130432" if regioncode=="130432" & year==2005

replace regioncode="130433" if regioncode=="130433" & year==2005

replace regioncode="130434" if regioncode=="130434" & year==2005

replace regioncode="130435" if regioncode=="130435" & year==2005

replace regioncode="130481" if regioncode=="130481" & year==2005

replace regioncode="130502" if regioncode=="130502" & year==2005

replace regioncode="130503" if regioncode=="130503" & year==2005

replace regioncode="130521" if regioncode=="130521" & year==2005

replace regioncode="130522" if regioncode=="130522" & year==2005

replace regioncode="130523" if regioncode=="130523" & year==2005

replace regioncode="130524" if regioncode=="130524" & year==2005

replace regioncode="130525" if regioncode=="130525" & year==2005

replace regioncode="130526" if regioncode=="130526" & year==2005

replace regioncode="130527" if regioncode=="130527" & year==2005

replace regioncode="130528" if regioncode=="130528" & year==2005

replace regioncode="130529" if regioncode=="130529" & year==2005

replace regioncode="130530" if regioncode=="130530" & year==2005

replace regioncode="130531" if regioncode=="130531" & year==2005

replace regioncode="130532" if regioncode=="130532" & year==2005

replace regioncode="130533" if regioncode=="130533" & year==2005

replace regioncode="130534" if regioncode=="130534" & year==2005

replace regioncode="130535" if regioncode=="130535" & year==2005

replace regioncode="130581" if regioncode=="130581" & year==2005

replace regioncode="130582" if regioncode=="130582" & year==2005

replace regioncode="130602" if regioncode=="130602" & year==2005

replace regioncode="130603" if regioncode=="130603" & year==2005

replace regioncode="130604" if regioncode=="130604" & year==2005

replace regioncode="130621" if regioncode=="130621" & year==2005

replace regioncode="130622" if regioncode=="130622" & year==2005

replace regioncode="130623" if regioncode=="130623" & year==2005

replace regioncode="130624" if regioncode=="130624" & year==2005

replace regioncode="130625" if regioncode=="130625" & year==2005

replace regioncode="130626" if regioncode=="130626" & year==2005

replace regioncode="130627" if regioncode=="130627" & year==2005

replace regioncode="130628" if regioncode=="130628" & year==2005

replace regioncode="130629" if regioncode=="130629" & year==2005

replace regioncode="130630" if regioncode=="130630" & year==2005

replace regioncode="130631" if regioncode=="130631" & year==2005

replace regioncode="130632" if regioncode=="130632" & year==2005

replace regioncode="130633" if regioncode=="130633" & year==2005

replace regioncode="130634" if regioncode=="130634" & year==2005

replace regioncode="130635" if regioncode=="130635" & year==2005

replace regioncode="130636" if regioncode=="130636" & year==2005

replace regioncode="130637" if regioncode=="130637" & year==2005

replace regioncode="130638" if regioncode=="130638" & year==2005

replace regioncode="130681" if regioncode=="130681" & year==2005

replace regioncode="130682" if regioncode=="130682" & year==2005

replace regioncode="130683" if regioncode=="130683" & year==2005

replace regioncode="130684" if regioncode=="130684" & year==2005

replace regioncode="130702" if regioncode=="130702" & year==2005

replace regioncode="130703" if regioncode=="130703" & year==2005

replace regioncode="130705" if regioncode=="130705" & year==2005

replace regioncode="130706" if regioncode=="130706" & year==2005

replace regioncode="130721" if regioncode=="130721" & year==2005

replace regioncode="130722" if regioncode=="130722" & year==2005

replace regioncode="130723" if regioncode=="130723" & year==2005

replace regioncode="130724" if regioncode=="130724" & year==2005

replace regioncode="130725" if regioncode=="130725" & year==2005

replace regioncode="130726" if regioncode=="130726" & year==2005

replace regioncode="130727" if regioncode=="130727" & year==2005

replace regioncode="130728" if regioncode=="130728" & year==2005

replace regioncode="130729" if regioncode=="130729" & year==2005

replace regioncode="130730" if regioncode=="130730" & year==2005

replace regioncode="130731" if regioncode=="130731" & year==2005

replace regioncode="130732" if regioncode=="130732" & year==2005

replace regioncode="130733" if regioncode=="130733" & year==2005

replace regioncode="130802" if regioncode=="130802" & year==2005

replace regioncode="130803" if regioncode=="130803" & year==2005

replace regioncode="130804" if regioncode=="130804" & year==2005

replace regioncode="130821" if regioncode=="130821" & year==2005

replace regioncode="130822" if regioncode=="130822" & year==2005

replace regioncode="130823" if regioncode=="130823" & year==2005

replace regioncode="130824" if regioncode=="130824" & year==2005

replace regioncode="130825" if regioncode=="130825" & year==2005

replace regioncode="130826" if regioncode=="130826" & year==2005

replace regioncode="130827" if regioncode=="130827" & year==2005

replace regioncode="130828" if regioncode=="130828" & year==2005

replace regioncode="130902" if regioncode=="130902" & year==2005

replace regioncode="130903" if regioncode=="130903" & year==2005

replace regioncode="130921" if regioncode=="130921" & year==2005

replace regioncode="130922" if regioncode=="130922" & year==2005

replace regioncode="130923" if regioncode=="130923" & year==2005

replace regioncode="130924" if regioncode=="130924" & year==2005

replace regioncode="130925" if regioncode=="130925" & year==2005

replace regioncode="130926" if regioncode=="130926" & year==2005

replace regioncode="130927" if regioncode=="130927" & year==2005

replace regioncode="130928" if regioncode=="130928" & year==2005

replace regioncode="130929" if regioncode=="130929" & year==2005

replace regioncode="130930" if regioncode=="130930" & year==2005

replace regioncode="130981" if regioncode=="130981" & year==2005

replace regioncode="130982" if regioncode=="130982" & year==2005

replace regioncode="130983" if regioncode=="130983" & year==2005

replace regioncode="130984" if regioncode=="130984" & year==2005

replace regioncode="131002" if regioncode=="131002" & year==2005

replace regioncode="131003" if regioncode=="131003" & year==2005

replace regioncode="131022" if regioncode=="131022" & year==2005

replace regioncode="131023" if regioncode=="131023" & year==2005

replace regioncode="131024" if regioncode=="131024" & year==2005

replace regioncode="131025" if regioncode=="131025" & year==2005

replace regioncode="131026" if regioncode=="131026" & year==2005

replace regioncode="131028" if regioncode=="131028" & year==2005

replace regioncode="131081" if regioncode=="131081" & year==2005

replace regioncode="131082" if regioncode=="131082" & year==2005

replace regioncode="131102" if regioncode=="131102" & year==2005

replace regioncode="131121" if regioncode=="131121" & year==2005

replace regioncode="131122" if regioncode=="131122" & year==2005

replace regioncode="131123" if regioncode=="131123" & year==2005

replace regioncode="131124" if regioncode=="131124" & year==2005

replace regioncode="131125" if regioncode=="131125" & year==2005

replace regioncode="131126" if regioncode=="131126" & year==2005

replace regioncode="131127" if regioncode=="131127" & year==2005

replace regioncode="131128" if regioncode=="131128" & year==2005

replace regioncode="131181" if regioncode=="131181" & year==2005

replace regioncode="131182" if regioncode=="131182" & year==2005

replace regioncode="140105" if regioncode=="140105" & year==2005

replace regioncode="140106" if regioncode=="140106" & year==2005

replace regioncode="140107" if regioncode=="140107" & year==2005

replace regioncode="140108" if regioncode=="140108" & year==2005

replace regioncode="140109" if regioncode=="140109" & year==2005

replace regioncode="140110" if regioncode=="140110" & year==2005

replace regioncode="140121" if regioncode=="140121" & year==2005

replace regioncode="140122" if regioncode=="140122" & year==2005

replace regioncode="140123" if regioncode=="140123" & year==2005

replace regioncode="140181" if regioncode=="140181" & year==2005

replace regioncode="140202" if regioncode=="140202" & year==2005

replace regioncode="140203" if regioncode=="140203" & year==2005

replace regioncode="140211" if regioncode=="140211" & year==2005

replace regioncode="140212" if regioncode=="140212" & year==2005

replace regioncode="140221" if regioncode=="140221" & year==2005

replace regioncode="140222" if regioncode=="140222" & year==2005

replace regioncode="140223" if regioncode=="140223" & year==2005

replace regioncode="140224" if regioncode=="140224" & year==2005

replace regioncode="140225" if regioncode=="140225" & year==2005

replace regioncode="140226" if regioncode=="140226" & year==2005

replace regioncode="140227" if regioncode=="140227" & year==2005

replace regioncode="140302" if regioncode=="140302" & year==2005

replace regioncode="140303" if regioncode=="140303" & year==2005

replace regioncode="140311" if regioncode=="140311" & year==2005

replace regioncode="140321" if regioncode=="140321" & year==2005

replace regioncode="140322" if regioncode=="140322" & year==2005

replace regioncode="140402" if regioncode=="140402" & year==2005

replace regioncode="140411" if regioncode=="140411" & year==2005

replace regioncode="140421" if regioncode=="140421" & year==2005

replace regioncode="140423" if regioncode=="140423" & year==2005

replace regioncode="140424" if regioncode=="140424" & year==2005

replace regioncode="140425" if regioncode=="140425" & year==2005

replace regioncode="140426" if regioncode=="140426" & year==2005

replace regioncode="140427" if regioncode=="140427" & year==2005

replace regioncode="140428" if regioncode=="140428" & year==2005

replace regioncode="140429" if regioncode=="140429" & year==2005

replace regioncode="140430" if regioncode=="140430" & year==2005

replace regioncode="140431" if regioncode=="140431" & year==2005

replace regioncode="140481" if regioncode=="140481" & year==2005

replace regioncode="140502" if regioncode=="140502" & year==2005

replace regioncode="140521" if regioncode=="140521" & year==2005

replace regioncode="140522" if regioncode=="140522" & year==2005

replace regioncode="140524" if regioncode=="140524" & year==2005

replace regioncode="140525" if regioncode=="140525" & year==2005

replace regioncode="140581" if regioncode=="140581" & year==2005

replace regioncode="140602" if regioncode=="140602" & year==2005

replace regioncode="140603" if regioncode=="140603" & year==2005

replace regioncode="140621" if regioncode=="140621" & year==2005

replace regioncode="140622" if regioncode=="140622" & year==2005

replace regioncode="140623" if regioncode=="140623" & year==2005

replace regioncode="140624" if regioncode=="140624" & year==2005

replace regioncode="140902" if regioncode=="140902" & year==2005

replace regioncode="140981" if regioncode=="140981" & year==2005

replace regioncode="140921" if regioncode=="140921" & year==2005

replace regioncode="140922" if regioncode=="140922" & year==2005

replace regioncode="140923" if regioncode=="140923" & year==2005

replace regioncode="140924" if regioncode=="140924" & year==2005

replace regioncode="140925" if regioncode=="140925" & year==2005

replace regioncode="140926" if regioncode=="140926" & year==2005

replace regioncode="140927" if regioncode=="140927" & year==2005

replace regioncode="140928" if regioncode=="140928" & year==2005

replace regioncode="140929" if regioncode=="140929" & year==2005

replace regioncode="140930" if regioncode=="140930" & year==2005

replace regioncode="140931" if regioncode=="140931" & year==2005

replace regioncode="140932" if regioncode=="140932" & year==2005

replace regioncode="141181" if regioncode=="141181" & year==2005

replace regioncode="141102" if regioncode=="141102" & year==2005

replace regioncode="141182" if regioncode=="141182" & year==2005

replace regioncode="141121" if regioncode=="141121" & year==2005

replace regioncode="141122" if regioncode=="141122" & year==2005

replace regioncode="141123" if regioncode=="141123" & year==2005

replace regioncode="141124" if regioncode=="141124" & year==2005

replace regioncode="141125" if regioncode=="141125" & year==2005

replace regioncode="141126" if regioncode=="141126" & year==2005

replace regioncode="141127" if regioncode=="141127" & year==2005

replace regioncode="141128" if regioncode=="141128" & year==2005

replace regioncode="141129" if regioncode=="141129" & year==2005

replace regioncode="141130" if regioncode=="141130" & year==2005

replace regioncode="140702" if regioncode=="140702" & year==2005

replace regioncode="140781" if regioncode=="140781" & year==2005

replace regioncode="140721" if regioncode=="140721" & year==2005

replace regioncode="140722" if regioncode=="140722" & year==2005

replace regioncode="140723" if regioncode=="140723" & year==2005

replace regioncode="140724" if regioncode=="140724" & year==2005

replace regioncode="140725" if regioncode=="140725" & year==2005

replace regioncode="140726" if regioncode=="140726" & year==2005

replace regioncode="140727" if regioncode=="140727" & year==2005

replace regioncode="140728" if regioncode=="140728" & year==2005

replace regioncode="140729" if regioncode=="140729" & year==2005

replace regioncode="141002" if regioncode=="141002" & year==2005

replace regioncode="141081" if regioncode=="141081" & year==2005

replace regioncode="141082" if regioncode=="141082" & year==2005

replace regioncode="141021" if regioncode=="141021" & year==2005

replace regioncode="141022" if regioncode=="141022" & year==2005

replace regioncode="141023" if regioncode=="141023" & year==2005

replace regioncode="141024" if regioncode=="141024" & year==2005

replace regioncode="141025" if regioncode=="141025" & year==2005

replace regioncode="141026" if regioncode=="141026" & year==2005

replace regioncode="141027" if regioncode=="141027" & year==2005

replace regioncode="141028" if regioncode=="141028" & year==2005

replace regioncode="141029" if regioncode=="141029" & year==2005

replace regioncode="141033" if regioncode=="141033" & year==2005

replace regioncode="141030" if regioncode=="141030" & year==2005

replace regioncode="141032" if regioncode=="141032" & year==2005

replace regioncode="141031" if regioncode=="141031" & year==2005

replace regioncode="141034" if regioncode=="141034" & year==2005

replace regioncode="140802" if regioncode=="140802" & year==2005

replace regioncode="140881" if regioncode=="140881" & year==2005

replace regioncode="140882" if regioncode=="140882" & year==2005

replace regioncode="140830" if regioncode=="140830" & year==2005

replace regioncode="140821" if regioncode=="140821" & year==2005

replace regioncode="140822" if regioncode=="140822" & year==2005

replace regioncode="140825" if regioncode=="140825" & year==2005

replace regioncode="140824" if regioncode=="140824" & year==2005

replace regioncode="140823" if regioncode=="140823" & year==2005

replace regioncode="140828" if regioncode=="140828" & year==2005

replace regioncode="140826" if regioncode=="140826" & year==2005

replace regioncode="140829" if regioncode=="140829" & year==2005

replace regioncode="140827" if regioncode=="140827" & year==2005

replace regioncode="150102" if regioncode=="150102" & year==2005

replace regioncode="150103" if regioncode=="150103" & year==2005

replace regioncode="150104" if regioncode=="150104" & year==2005

replace regioncode="150105" if regioncode=="150105" & year==2005

replace regioncode="150121" if regioncode=="150121" & year==2005

replace regioncode="150122" if regioncode=="150122" & year==2005

replace regioncode="150123" if regioncode=="150123" & year==2005

replace regioncode="150124" if regioncode=="150124" & year==2005

replace regioncode="150125" if regioncode=="150125" & year==2005

replace regioncode="150202" if regioncode=="150202" & year==2005

replace regioncode="150203" if regioncode=="150203" & year==2005

replace regioncode="150204" if regioncode=="150204" & year==2005

replace regioncode="150205" if regioncode=="150205" & year==2005

replace regioncode="150206" if regioncode=="150206" & year==2005

replace regioncode="150207" if regioncode=="150207" & year==2005

replace regioncode="150221" if regioncode=="150221" & year==2005

replace regioncode="150222" if regioncode=="150222" & year==2005

replace regioncode="150223" if regioncode=="150223" & year==2005

replace regioncode="150302" if regioncode=="150302" & year==2005

replace regioncode="150303" if regioncode=="150303" & year==2005

replace regioncode="150304" if regioncode=="150304" & year==2005

replace regioncode="150402" if regioncode=="150402" & year==2005

replace regioncode="150403" if regioncode=="150403" & year==2005

replace regioncode="150404" if regioncode=="150404" & year==2005

replace regioncode="150421" if regioncode=="150421" & year==2005

replace regioncode="150422" if regioncode=="150422" & year==2005

replace regioncode="150423" if regioncode=="150423" & year==2005

replace regioncode="150424" if regioncode=="150424" & year==2005

replace regioncode="150425" if regioncode=="150425" & year==2005

replace regioncode="150426" if regioncode=="150426" & year==2005

replace regioncode="150428" if regioncode=="150428" & year==2005

replace regioncode="150429" if regioncode=="150429" & year==2005

replace regioncode="150430" if regioncode=="150430" & year==2005

replace regioncode="150702" if regioncode=="150702" & year==2005

replace regioncode="150781" if regioncode=="150781" & year==2005

replace regioncode="150783" if regioncode=="150783" & year==2005

replace regioncode="150782" if regioncode=="150782" & year==2005

replace regioncode="150785" if regioncode=="150785" & year==2005

replace regioncode="150784" if regioncode=="150784" & year==2005

replace regioncode="150721" if regioncode=="150721" & year==2005

replace regioncode="150722" if regioncode=="150722" & year==2005

replace regioncode="150723" if regioncode=="150723" & year==2005

replace regioncode="150724" if regioncode=="150724" & year==2005

replace regioncode="150727" if regioncode=="150727" & year==2005

replace regioncode="150726" if regioncode=="150726" & year==2005

replace regioncode="150725" if regioncode=="150725" & year==2005

replace regioncode="152201" if regioncode=="152201" & year==2005

replace regioncode="152202" if regioncode=="152202" & year==2005

replace regioncode="152221" if regioncode=="152221" & year==2005

replace regioncode="152222" if regioncode=="152222" & year==2005

replace regioncode="152223" if regioncode=="152223" & year==2005

replace regioncode="152224" if regioncode=="152224" & year==2005

replace regioncode="150502" if regioncode=="150502" & year==2005

replace regioncode="150581" if regioncode=="150581" & year==2005

replace regioncode="150521" if regioncode=="150521" & year==2005

replace regioncode="150522" if regioncode=="150522" & year==2005

replace regioncode="150523" if regioncode=="150523" & year==2005

replace regioncode="150524" if regioncode=="150524" & year==2005

replace regioncode="150525" if regioncode=="150525" & year==2005

replace regioncode="150526" if regioncode=="150526" & year==2005

replace regioncode="152501" if regioncode=="152501" & year==2005

replace regioncode="152502" if regioncode=="152502" & year==2005

replace regioncode="152522" if regioncode=="152522" & year==2005

replace regioncode="152523" if regioncode=="152523" & year==2005

replace regioncode="152524" if regioncode=="152524" & year==2005

replace regioncode="152525" if regioncode=="152525" & year==2005

replace regioncode="152526" if regioncode=="152526" & year==2005

replace regioncode="152527" if regioncode=="152527" & year==2005

replace regioncode="152528" if regioncode=="152528" & year==2005

replace regioncode="152529" if regioncode=="152529" & year==2005

replace regioncode="152530" if regioncode=="152530" & year==2005

replace regioncode="152531" if regioncode=="152531" & year==2005

replace regioncode="152601" if regioncode=="150902" & year==2005

replace regioncode="152602" if regioncode=="150981" & year==2005

replace regioncode="152624" if regioncode=="150921" & year==2005

replace regioncode="152625" if regioncode=="150922" & year==2005

replace regioncode="152626" if regioncode=="150923" & year==2005

replace regioncode="152627" if regioncode=="150924" & year==2005

replace regioncode="152629" if regioncode=="150925" & year==2005

replace regioncode="152630" if regioncode=="150926" & year==2005

replace regioncode="152631" if regioncode=="150927" & year==2005

replace regioncode="152632" if regioncode=="150928" & year==2005

replace regioncode="152634" if regioncode=="150929" & year==2005

replace regioncode="150602" if regioncode=="150602" & year==2005

replace regioncode="150621" if regioncode=="150621" & year==2005

replace regioncode="150622" if regioncode=="150622" & year==2005

replace regioncode="150623" if regioncode=="150623" & year==2005

replace regioncode="150624" if regioncode=="150624" & year==2005

replace regioncode="150625" if regioncode=="150625" & year==2005

replace regioncode="150626" if regioncode=="150626" & year==2005

replace regioncode="150627" if regioncode=="150627" & year==2005

replace regioncode="152801" if regioncode=="150802" & year==2005

replace regioncode="152822" if regioncode=="150821" & year==2005

replace regioncode="152823" if regioncode=="150822" & year==2005

replace regioncode="152824" if regioncode=="150823" & year==2005

replace regioncode="152825" if regioncode=="150824" & year==2005

replace regioncode="152826" if regioncode=="150825" & year==2005

replace regioncode="152827" if regioncode=="150826" & year==2005

replace regioncode="152921" if regioncode=="152921" & year==2005

replace regioncode="152922" if regioncode=="152922" & year==2005

replace regioncode="152923" if regioncode=="152923" & year==2005

replace regioncode="210102" if regioncode=="210102" & year==2005

replace regioncode="210103" if regioncode=="210103" & year==2005

replace regioncode="210104" if regioncode=="210104" & year==2005

replace regioncode="210105" if regioncode=="210105" & year==2005

replace regioncode="210106" if regioncode=="210106" & year==2005

replace regioncode="210111" if regioncode=="210111" & year==2005

replace regioncode="210112" if regioncode=="210112" & year==2005

replace regioncode="210113" if regioncode=="210113" & year==2005

replace regioncode="210114" if regioncode=="210114" & year==2005

replace regioncode="210122" if regioncode=="210122" & year==2005

replace regioncode="210123" if regioncode=="210123" & year==2005

replace regioncode="210124" if regioncode=="210124" & year==2005

replace regioncode="210181" if regioncode=="210181" & year==2005

replace regioncode="210202" if regioncode=="210202" & year==2005

replace regioncode="210203" if regioncode=="210203" & year==2005

replace regioncode="210204" if regioncode=="210204" & year==2005

replace regioncode="210211" if regioncode=="210211" & year==2005

replace regioncode="210212" if regioncode=="210212" & year==2005

replace regioncode="210213" if regioncode=="210213" & year==2005

replace regioncode="210224" if regioncode=="210224" & year==2005

replace regioncode="210281" if regioncode=="210281" & year==2005

replace regioncode="210282" if regioncode=="210282" & year==2005

replace regioncode="210283" if regioncode=="210283" & year==2005

replace regioncode="210302" if regioncode=="210302" & year==2005

replace regioncode="210303" if regioncode=="210303" & year==2005

replace regioncode="210304" if regioncode=="210304" & year==2005

replace regioncode="210311" if regioncode=="210311" & year==2005

replace regioncode="210321" if regioncode=="210321" & year==2005

replace regioncode="210323" if regioncode=="210323" & year==2005

replace regioncode="210381" if regioncode=="210381" & year==2005

replace regioncode="210402" if regioncode=="210402" & year==2005

replace regioncode="210403" if regioncode=="210403" & year==2005

replace regioncode="210404" if regioncode=="210404" & year==2005

replace regioncode="210411" if regioncode=="210411" & year==2005

replace regioncode="210421" if regioncode=="210421" & year==2005

replace regioncode="210422" if regioncode=="210422" & year==2005

replace regioncode="210423" if regioncode=="210423" & year==2005

replace regioncode="210502" if regioncode=="210502" & year==2005

replace regioncode="210503" if regioncode=="210503" & year==2005

replace regioncode="210504" if regioncode=="210504" & year==2005

replace regioncode="210505" if regioncode=="210505" & year==2005

replace regioncode="210521" if regioncode=="210521" & year==2005

replace regioncode="210522" if regioncode=="210522" & year==2005

replace regioncode="210602" if regioncode=="210602" & year==2005

replace regioncode="210603" if regioncode=="210603" & year==2005

replace regioncode="210604" if regioncode=="210604" & year==2005

replace regioncode="210624" if regioncode=="210624" & year==2005

replace regioncode="210681" if regioncode=="210681" & year==2005

replace regioncode="210682" if regioncode=="210682" & year==2005

replace regioncode="210702" if regioncode=="210702" & year==2005

replace regioncode="210703" if regioncode=="210703" & year==2005

replace regioncode="210711" if regioncode=="210711" & year==2005

replace regioncode="210726" if regioncode=="210726" & year==2005

replace regioncode="210727" if regioncode=="210727" & year==2005

replace regioncode="210781" if regioncode=="210781" & year==2005

replace regioncode="210782" if regioncode=="210782" & year==2005

replace regioncode="210802" if regioncode=="210802" & year==2005

replace regioncode="210803" if regioncode=="210803" & year==2005

replace regioncode="210804" if regioncode=="210804" & year==2005

replace regioncode="210811" if regioncode=="210811" & year==2005

replace regioncode="210881" if regioncode=="210881" & year==2005

replace regioncode="210882" if regioncode=="210882" & year==2005

replace regioncode="210902" if regioncode=="210902" & year==2005

replace regioncode="210903" if regioncode=="210903" & year==2005

replace regioncode="210904" if regioncode=="210904" & year==2005

replace regioncode="210905" if regioncode=="210905" & year==2005

replace regioncode="210911" if regioncode=="210911" & year==2005

replace regioncode="210921" if regioncode=="210921" & year==2005

replace regioncode="210922" if regioncode=="210922" & year==2005

replace regioncode="211002" if regioncode=="211002" & year==2005

replace regioncode="211003" if regioncode=="211003" & year==2005

replace regioncode="211004" if regioncode=="211004" & year==2005

replace regioncode="211005" if regioncode=="211005" & year==2005

replace regioncode="211011" if regioncode=="211011" & year==2005

replace regioncode="211021" if regioncode=="211021" & year==2005

replace regioncode="211081" if regioncode=="211081" & year==2005

replace regioncode="211102" if regioncode=="211102" & year==2005

replace regioncode="211103" if regioncode=="211103" & year==2005

replace regioncode="211121" if regioncode=="211121" & year==2005

replace regioncode="211122" if regioncode=="211122" & year==2005

replace regioncode="211202" if regioncode=="211202" & year==2005

replace regioncode="211204" if regioncode=="211204" & year==2005

replace regioncode="211221" if regioncode=="211221" & year==2005

replace regioncode="211223" if regioncode=="211223" & year==2005

replace regioncode="211224" if regioncode=="211224" & year==2005

replace regioncode="211281" if regioncode=="211281" & year==2005

replace regioncode="211282" if regioncode=="211282" & year==2005

replace regioncode="211302" if regioncode=="211302" & year==2005

replace regioncode="211303" if regioncode=="211303" & year==2005

replace regioncode="211321" if regioncode=="211321" & year==2005

replace regioncode="211322" if regioncode=="211322" & year==2005

replace regioncode="211324" if regioncode=="211324" & year==2005

replace regioncode="211381" if regioncode=="211381" & year==2005

replace regioncode="211382" if regioncode=="211382" & year==2005

replace regioncode="211402" if regioncode=="211402" & year==2005

replace regioncode="211403" if regioncode=="211403" & year==2005

replace regioncode="211404" if regioncode=="211404" & year==2005

replace regioncode="211421" if regioncode=="211421" & year==2005

replace regioncode="211422" if regioncode=="211422" & year==2005

replace regioncode="211481" if regioncode=="211481" & year==2005

replace regioncode="220102" if regioncode=="220102" & year==2005

replace regioncode="220103" if regioncode=="220103" & year==2005

replace regioncode="220104" if regioncode=="220104" & year==2005

replace regioncode="220105" if regioncode=="220105" & year==2005

replace regioncode="220106" if regioncode=="220106" & year==2005

replace regioncode="220112" if regioncode=="220112" & year==2005

replace regioncode="220122" if regioncode=="220122" & year==2005

replace regioncode="220181" if regioncode=="220181" & year==2005

replace regioncode="220182" if regioncode=="220182" & year==2005

replace regioncode="220183" if regioncode=="220183" & year==2005

replace regioncode="220202" if regioncode=="220202" & year==2005

replace regioncode="220203" if regioncode=="220203" & year==2005

replace regioncode="220204" if regioncode=="220204" & year==2005

replace regioncode="220211" if regioncode=="220211" & year==2005

replace regioncode="220221" if regioncode=="220221" & year==2005

replace regioncode="220281" if regioncode=="220281" & year==2005

replace regioncode="220282" if regioncode=="220282" & year==2005

replace regioncode="220283" if regioncode=="220283" & year==2005

replace regioncode="220284" if regioncode=="220284" & year==2005

replace regioncode="220302" if regioncode=="220302" & year==2005

replace regioncode="220303" if regioncode=="220303" & year==2005

replace regioncode="220322" if regioncode=="220322" & year==2005

replace regioncode="220323" if regioncode=="220323" & year==2005

replace regioncode="220381" if regioncode=="220381" & year==2005

replace regioncode="220382" if regioncode=="220382" & year==2005

replace regioncode="220402" if regioncode=="220402" & year==2005

replace regioncode="220403" if regioncode=="220403" & year==2005

replace regioncode="220421" if regioncode=="220421" & year==2005

replace regioncode="220422" if regioncode=="220422" & year==2005

replace regioncode="220502" if regioncode=="220502" & year==2005

replace regioncode="220503" if regioncode=="220503" & year==2005

replace regioncode="220521" if regioncode=="220521" & year==2005

replace regioncode="220523" if regioncode=="220523" & year==2005

replace regioncode="220524" if regioncode=="220524" & year==2005

replace regioncode="220581" if regioncode=="220581" & year==2005

replace regioncode="220582" if regioncode=="220582" & year==2005

replace regioncode="220602" if regioncode=="220602" & year==2005

replace regioncode="220621" if regioncode=="220621" & year==2005

replace regioncode="220622" if regioncode=="220622" & year==2005

replace regioncode="220623" if regioncode=="220623" & year==2005

replace regioncode="220625" if regioncode=="220625" & year==2005

replace regioncode="220681" if regioncode=="220681" & year==2005

replace regioncode="220702" if regioncode=="220702" & year==2005

replace regioncode="220721" if regioncode=="220721" & year==2005

replace regioncode="220722" if regioncode=="220722" & year==2005

replace regioncode="220723" if regioncode=="220723" & year==2005

replace regioncode="220724" if regioncode=="220724" & year==2005

replace regioncode="220802" if regioncode=="220802" & year==2005

replace regioncode="220821" if regioncode=="220821" & year==2005

replace regioncode="220822" if regioncode=="220822" & year==2005

replace regioncode="220881" if regioncode=="220881" & year==2005

replace regioncode="220882" if regioncode=="220882" & year==2005

replace regioncode="222401" if regioncode=="222401" & year==2005

replace regioncode="222402" if regioncode=="222402" & year==2005

replace regioncode="222403" if regioncode=="222403" & year==2005

replace regioncode="222404" if regioncode=="222404" & year==2005

replace regioncode="222405" if regioncode=="222405" & year==2005

replace regioncode="222406" if regioncode=="222406" & year==2005

replace regioncode="222424" if regioncode=="222424" & year==2005

replace regioncode="222426" if regioncode=="222426" & year==2005

replace regioncode="230102" if regioncode=="230102" & year==2005

replace regioncode="230103" if regioncode=="230103" & year==2005

replace regioncode="230104" if regioncode=="230104" & year==2005

replace regioncode="230105" if regioncode=="230105" & year==2005

replace regioncode="230107" if regioncode=="230107" & year==2005

replace regioncode="230108" if regioncode=="230108" & year==2005

replace regioncode="230121" if regioncode=="230111" & year==2005

replace regioncode="230123" if regioncode=="230123" & year==2005

replace regioncode="230124" if regioncode=="230124" & year==2005

replace regioncode="230125" if regioncode=="230125" & year==2005

replace regioncode="230126" if regioncode=="230126" & year==2005

replace regioncode="230127" if regioncode=="230127" & year==2005

replace regioncode="230128" if regioncode=="230128" & year==2005

replace regioncode="230129" if regioncode=="230129" & year==2005

replace regioncode="230181" if regioncode=="230181" & year==2005

replace regioncode="230182" if regioncode=="230182" & year==2005

replace regioncode="230183" if regioncode=="230183" & year==2005

replace regioncode="230184" if regioncode=="230184" & year==2005

replace regioncode="230202" if regioncode=="230202" & year==2005

replace regioncode="230203" if regioncode=="230203" & year==2005

replace regioncode="230204" if regioncode=="230204" & year==2005

replace regioncode="230205" if regioncode=="230205" & year==2005

replace regioncode="230206" if regioncode=="230206" & year==2005

replace regioncode="230207" if regioncode=="230207" & year==2005

replace regioncode="230208" if regioncode=="230208" & year==2005

replace regioncode="230221" if regioncode=="230221" & year==2005

replace regioncode="230223" if regioncode=="230223" & year==2005

replace regioncode="230224" if regioncode=="230224" & year==2005

replace regioncode="230225" if regioncode=="230225" & year==2005

replace regioncode="230227" if regioncode=="230227" & year==2005

replace regioncode="230229" if regioncode=="230229" & year==2005

replace regioncode="230230" if regioncode=="230230" & year==2005

replace regioncode="230231" if regioncode=="230231" & year==2005

replace regioncode="230281" if regioncode=="230281" & year==2005

replace regioncode="230302" if regioncode=="230302" & year==2005

replace regioncode="230303" if regioncode=="230303" & year==2005

replace regioncode="230304" if regioncode=="230304" & year==2005

replace regioncode="230305" if regioncode=="230305" & year==2005

replace regioncode="230306" if regioncode=="230306" & year==2005

replace regioncode="230307" if regioncode=="230307" & year==2005

replace regioncode="230321" if regioncode=="230321" & year==2005

replace regioncode="230381" if regioncode=="230381" & year==2005

replace regioncode="230382" if regioncode=="230382" & year==2005

replace regioncode="230402" if regioncode=="230402" & year==2005

replace regioncode="230403" if regioncode=="230403" & year==2005

replace regioncode="230404" if regioncode=="230404" & year==2005

replace regioncode="230405" if regioncode=="230405" & year==2005

replace regioncode="230406" if regioncode=="230406" & year==2005

replace regioncode="230407" if regioncode=="230407" & year==2005

replace regioncode="230421" if regioncode=="230421" & year==2005

replace regioncode="230422" if regioncode=="230422" & year==2005

replace regioncode="230502" if regioncode=="230502" & year==2005

replace regioncode="230503" if regioncode=="230503" & year==2005

replace regioncode="230505" if regioncode=="230505" & year==2005

replace regioncode="230506" if regioncode=="230506" & year==2005

replace regioncode="230521" if regioncode=="230521" & year==2005

replace regioncode="230522" if regioncode=="230522" & year==2005

replace regioncode="230523" if regioncode=="230523" & year==2005

replace regioncode="230524" if regioncode=="230524" & year==2005

replace regioncode="230602" if regioncode=="230602" & year==2005

replace regioncode="230603" if regioncode=="230603" & year==2005

replace regioncode="230604" if regioncode=="230604" & year==2005

replace regioncode="230605" if regioncode=="230605" & year==2005

replace regioncode="230606" if regioncode=="230606" & year==2005

replace regioncode="230621" if regioncode=="230621" & year==2005

replace regioncode="230622" if regioncode=="230622" & year==2005

replace regioncode="230623" if regioncode=="230623" & year==2005

replace regioncode="230624" if regioncode=="230624" & year==2005

replace regioncode="230702" if regioncode=="230702" & year==2005

replace regioncode="230703" if regioncode=="230703" & year==2005

replace regioncode="230704" if regioncode=="230704" & year==2005

replace regioncode="230705" if regioncode=="230705" & year==2005

replace regioncode="230706" if regioncode=="230706" & year==2005

replace regioncode="230707" if regioncode=="230707" & year==2005

replace regioncode="230708" if regioncode=="230708" & year==2005

replace regioncode="230709" if regioncode=="230709" & year==2005

replace regioncode="230710" if regioncode=="230710" & year==2005

replace regioncode="230711" if regioncode=="230711" & year==2005

replace regioncode="230712" if regioncode=="230712" & year==2005

replace regioncode="230713" if regioncode=="230713" & year==2005

replace regioncode="230714" if regioncode=="230714" & year==2005

replace regioncode="230715" if regioncode=="230715" & year==2005

replace regioncode="230716" if regioncode=="230716" & year==2005

replace regioncode="230722" if regioncode=="230722" & year==2005

replace regioncode="230781" if regioncode=="230781" & year==2005

replace regioncode="230802" if regioncode=="230802" & year==2005

replace regioncode="230803" if regioncode=="230803" & year==2005

replace regioncode="230804" if regioncode=="230804" & year==2005

replace regioncode="230805" if regioncode=="230805" & year==2005

replace regioncode="230811" if regioncode=="230811" & year==2005

replace regioncode="230822" if regioncode=="230822" & year==2005

replace regioncode="230826" if regioncode=="230826" & year==2005

replace regioncode="230828" if regioncode=="230828" & year==2005

replace regioncode="230833" if regioncode=="230833" & year==2005

replace regioncode="230881" if regioncode=="230881" & year==2005

replace regioncode="230882" if regioncode=="230882" & year==2005

replace regioncode="230902" if regioncode=="230902" & year==2005

replace regioncode="230903" if regioncode=="230903" & year==2005

replace regioncode="230904" if regioncode=="230904" & year==2005

replace regioncode="230921" if regioncode=="230921" & year==2005

replace regioncode="231002" if regioncode=="231002" & year==2005

replace regioncode="231003" if regioncode=="231003" & year==2005

replace regioncode="231004" if regioncode=="231004" & year==2005

replace regioncode="231005" if regioncode=="231005" & year==2005

replace regioncode="231024" if regioncode=="231024" & year==2005

replace regioncode="231025" if regioncode=="231025" & year==2005

replace regioncode="231081" if regioncode=="231081" & year==2005

replace regioncode="231083" if regioncode=="231083" & year==2005

replace regioncode="231084" if regioncode=="231084" & year==2005

replace regioncode="231085" if regioncode=="231085" & year==2005

replace regioncode="231102" if regioncode=="231102" & year==2005

replace regioncode="231121" if regioncode=="231121" & year==2005

replace regioncode="231123" if regioncode=="231123" & year==2005

replace regioncode="231124" if regioncode=="231124" & year==2005

replace regioncode="231181" if regioncode=="231181" & year==2005

replace regioncode="231182" if regioncode=="231182" & year==2005

replace regioncode="231202" if regioncode=="231202" & year==2005

replace regioncode="231221" if regioncode=="231221" & year==2005

replace regioncode="231222" if regioncode=="231222" & year==2005

replace regioncode="231223" if regioncode=="231223" & year==2005

replace regioncode="231224" if regioncode=="231224" & year==2005

replace regioncode="231225" if regioncode=="231225" & year==2005

replace regioncode="231226" if regioncode=="231226" & year==2005

replace regioncode="231281" if regioncode=="231281" & year==2005

replace regioncode="231282" if regioncode=="231282" & year==2005

replace regioncode="231283" if regioncode=="231283" & year==2005

replace regioncode="232701" if regioncode=="232701" & year==2005

replace regioncode="232702" if regioncode=="232702" & year==2005

replace regioncode="232703" if regioncode=="232703" & year==2005

replace regioncode="232704" if regioncode=="232704" & year==2005

replace regioncode="232721" if regioncode=="232721" & year==2005

replace regioncode="232722" if regioncode=="232722" & year==2005

replace regioncode="232723" if regioncode=="232723" & year==2005

replace regioncode="310101" if regioncode=="310101" & year==2005

replace regioncode="310103" if regioncode=="310103" & year==2005

replace regioncode="310104" if regioncode=="310104" & year==2005

replace regioncode="310105" if regioncode=="310105" & year==2005

replace regioncode="310106" if regioncode=="310106" & year==2005

replace regioncode="310107" if regioncode=="310107" & year==2005

replace regioncode="310108" if regioncode=="310108" & year==2005

replace regioncode="310109" if regioncode=="310109" & year==2005

replace regioncode="310110" if regioncode=="310110" & year==2005

replace regioncode="310112" if regioncode=="310112" & year==2005

replace regioncode="310113" if regioncode=="310113" & year==2005

replace regioncode="310114" if regioncode=="310114" & year==2005

replace regioncode="310115" if regioncode=="310115" & year==2005

replace regioncode="310116" if regioncode=="310116" & year==2005

replace regioncode="310117" if regioncode=="310117" & year==2005

replace regioncode="310118" if regioncode=="310118" & year==2005

replace regioncode="310119" if regioncode=="310119" & year==2005

replace regioncode="310120" if regioncode=="310120" & year==2005

replace regioncode="310230" if regioncode=="310230" & year==2005

replace regioncode="320102" if regioncode=="320102" & year==2005

replace regioncode="320103" if regioncode=="320103" & year==2005

replace regioncode="320104" if regioncode=="320104" & year==2005

replace regioncode="320105" if regioncode=="320105" & year==2005

replace regioncode="320106" if regioncode=="320106" & year==2005

replace regioncode="320107" if regioncode=="320107" & year==2005

replace regioncode="320111" if regioncode=="320111" & year==2005

replace regioncode="320113" if regioncode=="320113" & year==2005

replace regioncode="320114" if regioncode=="320114" & year==2005

replace regioncode="320115" if regioncode=="320115" & year==2005

replace regioncode="320116" if regioncode=="320116" & year==2005

replace regioncode="320124" if regioncode=="320124" & year==2005

replace regioncode="320125" if regioncode=="320125" & year==2005

replace regioncode="320202" if regioncode=="320202" & year==2005

replace regioncode="320203" if regioncode=="320203" & year==2005

replace regioncode="320204" if regioncode=="320204" & year==2005

replace regioncode="320205" if regioncode=="320205" & year==2005

replace regioncode="320211" if regioncode=="320211" & year==2005

replace regioncode="320281" if regioncode=="320281" & year==2005

replace regioncode="320282" if regioncode=="320282" & year==2005

replace regioncode="320302" if regioncode=="320302" & year==2005

replace regioncode="320303" if regioncode=="320303" & year==2005

replace regioncode="320304" if regioncode=="320304" & year==2005

replace regioncode="320305" if regioncode=="320305" & year==2005

replace regioncode="320311" if regioncode=="320311" & year==2005

replace regioncode="320321" if regioncode=="320321" & year==2005

replace regioncode="320322" if regioncode=="320322" & year==2005

replace regioncode="320323" if regioncode=="320323" & year==2005

replace regioncode="320324" if regioncode=="320324" & year==2005

replace regioncode="320381" if regioncode=="320381" & year==2005

replace regioncode="320382" if regioncode=="320382" & year==2005

replace regioncode="320402" if regioncode=="320402" & year==2005

replace regioncode="320404" if regioncode=="320404" & year==2005

replace regioncode="320405" if regioncode=="320405" & year==2005

replace regioncode="320411" if regioncode=="320411" & year==2005

replace regioncode="320412" if regioncode=="320412" & year==2005

replace regioncode="320481" if regioncode=="320481" & year==2005

replace regioncode="320482" if regioncode=="320482" & year==2005

replace regioncode="320502" if regioncode=="320502" & year==2005

replace regioncode="320503" if regioncode=="320503" & year==2005

replace regioncode="320504" if regioncode=="320504" & year==2005

replace regioncode="320505" if regioncode=="320505" & year==2005

replace regioncode="320581" if regioncode=="320581" & year==2005

replace regioncode="320582" if regioncode=="320582" & year==2005

replace regioncode="320583" if regioncode=="320583" & year==2005

replace regioncode="320584" if regioncode=="320584" & year==2005

replace regioncode="320585" if regioncode=="320585" & year==2005

replace regioncode="320586" if regioncode=="320586" & year==2005

replace regioncode="320602" if regioncode=="320602" & year==2005

replace regioncode="320611" if regioncode=="320611" & year==2005

replace regioncode="320621" if regioncode=="320621" & year==2005

replace regioncode="320623" if regioncode=="320623" & year==2005

replace regioncode="320681" if regioncode=="320681" & year==2005

replace regioncode="320682" if regioncode=="320682" & year==2005

replace regioncode="320683" if regioncode=="320683" & year==2005

replace regioncode="320684" if regioncode=="320684" & year==2005

replace regioncode="320703" if regioncode=="320703" & year==2005

replace regioncode="320705" if regioncode=="320705" & year==2005

replace regioncode="320706" if regioncode=="320706" & year==2005

replace regioncode="320721" if regioncode=="320721" & year==2005

replace regioncode="320722" if regioncode=="320722" & year==2005

replace regioncode="320723" if regioncode=="320723" & year==2005

replace regioncode="320724" if regioncode=="320724" & year==2005

replace regioncode="320802" if regioncode=="320802" & year==2005

replace regioncode="320803" if regioncode=="320803" & year==2005

replace regioncode="320804" if regioncode=="320804" & year==2005

replace regioncode="320811" if regioncode=="320811" & year==2005

replace regioncode="320826" if regioncode=="320826" & year==2005

replace regioncode="320829" if regioncode=="320829" & year==2005

replace regioncode="320830" if regioncode=="320830" & year==2005

replace regioncode="320831" if regioncode=="320831" & year==2005

replace regioncode="320902" if regioncode=="320902" & year==2005

replace regioncode="320903" if regioncode=="320903" & year==2005

replace regioncode="320921" if regioncode=="320921" & year==2005

replace regioncode="320922" if regioncode=="320922" & year==2005

replace regioncode="320923" if regioncode=="320923" & year==2005

replace regioncode="320924" if regioncode=="320924" & year==2005

replace regioncode="320925" if regioncode=="320925" & year==2005

replace regioncode="320981" if regioncode=="320981" & year==2005

replace regioncode="320982" if regioncode=="320982" & year==2005

replace regioncode="321002" if regioncode=="321002" & year==2005

replace regioncode="321003" if regioncode=="321003" & year==2005

replace regioncode="321011" if regioncode=="321011" & year==2005

replace regioncode="321023" if regioncode=="321023" & year==2005

replace regioncode="321081" if regioncode=="321081" & year==2005

replace regioncode="321084" if regioncode=="321084" & year==2005

replace regioncode="321088" if regioncode=="321088" & year==2005

replace regioncode="321102" if regioncode=="321102" & year==2005

replace regioncode="321111" if regioncode=="321111" & year==2005

replace regioncode="321112" if regioncode=="321112" & year==2005

replace regioncode="321181" if regioncode=="321181" & year==2005

replace regioncode="321182" if regioncode=="321182" & year==2005

replace regioncode="321183" if regioncode=="321183" & year==2005

replace regioncode="321202" if regioncode=="321202" & year==2005

replace regioncode="321203" if regioncode=="321203" & year==2005

replace regioncode="321281" if regioncode=="321281" & year==2005

replace regioncode="321282" if regioncode=="321282" & year==2005

replace regioncode="321283" if regioncode=="321283" & year==2005

replace regioncode="321284" if regioncode=="321284" & year==2005

replace regioncode="321302" if regioncode=="321302" & year==2005

replace regioncode="321321" if regioncode=="321311" & year==2005

replace regioncode="321322" if regioncode=="321322" & year==2005

replace regioncode="321323" if regioncode=="321323" & year==2005

replace regioncode="321324" if regioncode=="321324" & year==2005

replace regioncode="330102" if regioncode=="330102" & year==2005

replace regioncode="330103" if regioncode=="330103" & year==2005

replace regioncode="330104" if regioncode=="330104" & year==2005

replace regioncode="330105" if regioncode=="330105" & year==2005

replace regioncode="330106" if regioncode=="330106" & year==2005

replace regioncode="330108" if regioncode=="330108" & year==2005

replace regioncode="330109" if regioncode=="330109" & year==2005

replace regioncode="330110" if regioncode=="330110" & year==2005

replace regioncode="330122" if regioncode=="330122" & year==2005

replace regioncode="330127" if regioncode=="330127" & year==2005

replace regioncode="330182" if regioncode=="330182" & year==2005

replace regioncode="330183" if regioncode=="330183" & year==2005

replace regioncode="330185" if regioncode=="330185" & year==2005

replace regioncode="330203" if regioncode=="330203" & year==2005

replace regioncode="330204" if regioncode=="330204" & year==2005

replace regioncode="330205" if regioncode=="330205" & year==2005

replace regioncode="330206" if regioncode=="330206" & year==2005

replace regioncode="330211" if regioncode=="330211" & year==2005

replace regioncode="330212" if regioncode=="330212" & year==2005

replace regioncode="330225" if regioncode=="330225" & year==2005

replace regioncode="330226" if regioncode=="330226" & year==2005

replace regioncode="330281" if regioncode=="330281" & year==2005

replace regioncode="330282" if regioncode=="330282" & year==2005

replace regioncode="330283" if regioncode=="330283" & year==2005

replace regioncode="330302" if regioncode=="330302" & year==2005

replace regioncode="330303" if regioncode=="330303" & year==2005

replace regioncode="330304" if regioncode=="330304" & year==2005

replace regioncode="330322" if regioncode=="330322" & year==2005

replace regioncode="330324" if regioncode=="330324" & year==2005

replace regioncode="330326" if regioncode=="330326" & year==2005

replace regioncode="330327" if regioncode=="330327" & year==2005

replace regioncode="330328" if regioncode=="330328" & year==2005

replace regioncode="330329" if regioncode=="330329" & year==2005

replace regioncode="330381" if regioncode=="330381" & year==2005

replace regioncode="330382" if regioncode=="330382" & year==2005

replace regioncode="330402" if regioncode=="330402" & year==2005

replace regioncode="330411" if regioncode=="330411" & year==2005

replace regioncode="330421" if regioncode=="330421" & year==2005

replace regioncode="330424" if regioncode=="330424" & year==2005

replace regioncode="330481" if regioncode=="330481" & year==2005

replace regioncode="330482" if regioncode=="330482" & year==2005

replace regioncode="330483" if regioncode=="330483" & year==2005

replace regioncode="330501" if regioncode=="330501" & year==2005

replace regioncode="330521" if regioncode=="330521" & year==2005

replace regioncode="330522" if regioncode=="330522" & year==2005

replace regioncode="330523" if regioncode=="330523" & year==2005

replace regioncode="330602" if regioncode=="330602" & year==2005

replace regioncode="330621" if regioncode=="330621" & year==2005

replace regioncode="330624" if regioncode=="330624" & year==2005

replace regioncode="330681" if regioncode=="330681" & year==2005

replace regioncode="330682" if regioncode=="330682" & year==2005

replace regioncode="330683" if regioncode=="330683" & year==2005

replace regioncode="330702" if regioncode=="330702" & year==2005

replace regioncode="330703" if regioncode=="330703" & year==2005

replace regioncode="330723" if regioncode=="330723" & year==2005

replace regioncode="330726" if regioncode=="330726" & year==2005

replace regioncode="330727" if regioncode=="330727" & year==2005

replace regioncode="330781" if regioncode=="330781" & year==2005

replace regioncode="330782" if regioncode=="330782" & year==2005

replace regioncode="330783" if regioncode=="330783" & year==2005

replace regioncode="330784" if regioncode=="330784" & year==2005

replace regioncode="330802" if regioncode=="330802" & year==2005

replace regioncode="330803" if regioncode=="330803" & year==2005

replace regioncode="330822" if regioncode=="330822" & year==2005

replace regioncode="330824" if regioncode=="330824" & year==2005

replace regioncode="330825" if regioncode=="330825" & year==2005

replace regioncode="330881" if regioncode=="330881" & year==2005

replace regioncode="330902" if regioncode=="330902" & year==2005

replace regioncode="330903" if regioncode=="330903" & year==2005

replace regioncode="330921" if regioncode=="330921" & year==2005

replace regioncode="330922" if regioncode=="330922" & year==2005

replace regioncode="331002" if regioncode=="331002" & year==2005

replace regioncode="331003" if regioncode=="331003" & year==2005

replace regioncode="331004" if regioncode=="331004" & year==2005

replace regioncode="331021" if regioncode=="331021" & year==2005

replace regioncode="331022" if regioncode=="331022" & year==2005

replace regioncode="331023" if regioncode=="331023" & year==2005

replace regioncode="331024" if regioncode=="331024" & year==2005

replace regioncode="331081" if regioncode=="331081" & year==2005

replace regioncode="331082" if regioncode=="331082" & year==2005

replace regioncode="331102" if regioncode=="331102" & year==2005

replace regioncode="331121" if regioncode=="331121" & year==2005

replace regioncode="331122" if regioncode=="331122" & year==2005

replace regioncode="331123" if regioncode=="331123" & year==2005

replace regioncode="331124" if regioncode=="331124" & year==2005

replace regioncode="331125" if regioncode=="331125" & year==2005

replace regioncode="331126" if regioncode=="331126" & year==2005

replace regioncode="331127" if regioncode=="331127" & year==2005

replace regioncode="331181" if regioncode=="331181" & year==2005

replace regioncode="340102" if regioncode=="340102" & year==2005

replace regioncode="340103" if regioncode=="340103" & year==2005

replace regioncode="340104" if regioncode=="340104" & year==2005

replace regioncode="340111" if regioncode=="340111" & year==2005

replace regioncode="340121" if regioncode=="340121" & year==2005

replace regioncode="340122" if regioncode=="340122" & year==2005

replace regioncode="340123" if regioncode=="340123" & year==2005

replace regioncode="340202" if regioncode=="340202" & year==2005

replace regioncode="340203" if regioncode=="340203" & year==2005

replace regioncode="340204" if regioncode=="340204" & year==2005

replace regioncode="340207" if regioncode=="340207" & year==2005

replace regioncode="340221" if regioncode=="340221" & year==2005

replace regioncode="340222" if regioncode=="340222" & year==2005

replace regioncode="340223" if regioncode=="340223" & year==2005

replace regioncode="340302" if regioncode=="340302" & year==2005

replace regioncode="340303" if regioncode=="340303" & year==2005

replace regioncode="340304" if regioncode=="340304" & year==2005

replace regioncode="340311" if regioncode=="340311" & year==2005

replace regioncode="340321" if regioncode=="340321" & year==2005

replace regioncode="340322" if regioncode=="340322" & year==2005

replace regioncode="340323" if regioncode=="340323" & year==2005

replace regioncode="340402" if regioncode=="340402" & year==2005

replace regioncode="340403" if regioncode=="340403" & year==2005

replace regioncode="340404" if regioncode=="340404" & year==2005

replace regioncode="340405" if regioncode=="340405" & year==2005

replace regioncode="340406" if regioncode=="340406" & year==2005

replace regioncode="340421" if regioncode=="340421" & year==2005

replace regioncode="340502" if regioncode=="340502" & year==2005

replace regioncode="340503" if regioncode=="340503" & year==2005

replace regioncode="340504" if regioncode=="340504" & year==2005

replace regioncode="340521" if regioncode=="340521" & year==2005

replace regioncode="340602" if regioncode=="340602" & year==2005

replace regioncode="340603" if regioncode=="340603" & year==2005

replace regioncode="340604" if regioncode=="340604" & year==2005

replace regioncode="340621" if regioncode=="340621" & year==2005

replace regioncode="340702" if regioncode=="340702" & year==2005

replace regioncode="340703" if regioncode=="340703" & year==2005

replace regioncode="340711" if regioncode=="340711" & year==2005

replace regioncode="340721" if regioncode=="340721" & year==2005

replace regioncode="340802" if regioncode=="340802" & year==2005

replace regioncode="340803" if regioncode=="340803" & year==2005

replace regioncode="340811" if regioncode=="340811" & year==2005

replace regioncode="340822" if regioncode=="340822" & year==2005

replace regioncode="340823" if regioncode=="340823" & year==2005

replace regioncode="340824" if regioncode=="340824" & year==2005

replace regioncode="340825" if regioncode=="340825" & year==2005

replace regioncode="340826" if regioncode=="340826" & year==2005

replace regioncode="340827" if regioncode=="340827" & year==2005

replace regioncode="340828" if regioncode=="340828" & year==2005

replace regioncode="340881" if regioncode=="340881" & year==2005

replace regioncode="341002" if regioncode=="341002" & year==2005

replace regioncode="341003" if regioncode=="341003" & year==2005

replace regioncode="341004" if regioncode=="341004" & year==2005

replace regioncode="341021" if regioncode=="341021" & year==2005

replace regioncode="341022" if regioncode=="341022" & year==2005

replace regioncode="341023" if regioncode=="341023" & year==2005

replace regioncode="341024" if regioncode=="341024" & year==2005

replace regioncode="341102" if regioncode=="341102" & year==2005

replace regioncode="341103" if regioncode=="341103" & year==2005

replace regioncode="341122" if regioncode=="341122" & year==2005

replace regioncode="341124" if regioncode=="341124" & year==2005

replace regioncode="341125" if regioncode=="341125" & year==2005

replace regioncode="341126" if regioncode=="341126" & year==2005

replace regioncode="341181" if regioncode=="341181" & year==2005

replace regioncode="341182" if regioncode=="341182" & year==2005

replace regioncode="341202" if regioncode=="341202" & year==2005

replace regioncode="341203" if regioncode=="341203" & year==2005

replace regioncode="341204" if regioncode=="341204" & year==2005

replace regioncode="341221" if regioncode=="341221" & year==2005

replace regioncode="341222" if regioncode=="341222" & year==2005

replace regioncode="341621" if regioncode=="341621" & year==2005

replace regioncode="341622" if regioncode=="341622" & year==2005

replace regioncode="341225" if regioncode=="341225" & year==2005

replace regioncode="341226" if regioncode=="341226" & year==2005

replace regioncode="341623" if regioncode=="341623" & year==2005

replace regioncode="341282" if regioncode=="341282" & year==2005

replace regioncode="341302" if regioncode=="341302" & year==2005

replace regioncode="341321" if regioncode=="341321" & year==2005

replace regioncode="341322" if regioncode=="341322" & year==2005

replace regioncode="341323" if regioncode=="341323" & year==2005

replace regioncode="341324" if regioncode=="341324" & year==2005

replace regioncode="341402" if regioncode=="341402" & year==2005

replace regioncode="341421" if regioncode=="341421" & year==2005

replace regioncode="341422" if regioncode=="341422" & year==2005

replace regioncode="341423" if regioncode=="341423" & year==2005

replace regioncode="341424" if regioncode=="341424" & year==2005

replace regioncode="341521" if regioncode=="341521" & year==2005

replace regioncode="341522" if regioncode=="341522" & year==2005

replace regioncode="341523" if regioncode=="341523" & year==2005

replace regioncode="341524" if regioncode=="341524" & year==2005

replace regioncode="341525" if regioncode=="341525" & year==2005

replace regioncode="341802" if regioncode=="341802" & year==2005

replace regioncode="341881" if regioncode=="341881" & year==2005

replace regioncode="341821" if regioncode=="341821" & year==2005

replace regioncode="341822" if regioncode=="341822" & year==2005

replace regioncode="341823" if regioncode=="341823" & year==2005

replace regioncode="341825" if regioncode=="341825" & year==2005

replace regioncode="341824" if regioncode=="341824" & year==2005

replace regioncode="341702" if regioncode=="341702" & year==2005

replace regioncode="341721" if regioncode=="341721" & year==2005

replace regioncode="341722" if regioncode=="341722" & year==2005

replace regioncode="341723" if regioncode=="341723" & year==2005

replace regioncode="341502" if regioncode=="341502" & year==2005

replace regioncode="341602" if regioncode=="341602" & year==2005

replace regioncode="350102" if regioncode=="350102" & year==2005

replace regioncode="350103" if regioncode=="350103" & year==2005

replace regioncode="350104" if regioncode=="350104" & year==2005

replace regioncode="350105" if regioncode=="350105" & year==2005

replace regioncode="350111" if regioncode=="350111" & year==2005

replace regioncode="350121" if regioncode=="350121" & year==2005

replace regioncode="350122" if regioncode=="350122" & year==2005

replace regioncode="350123" if regioncode=="350123" & year==2005

replace regioncode="350124" if regioncode=="350124" & year==2005

replace regioncode="350125" if regioncode=="350125" & year==2005

replace regioncode="350128" if regioncode=="350128" & year==2005

replace regioncode="350181" if regioncode=="350181" & year==2005

replace regioncode="350182" if regioncode=="350182" & year==2005

replace regioncode="350203" if regioncode=="350203" & year==2005

replace regioncode="350205" if regioncode=="350205" & year==2005

replace regioncode="350206" if regioncode=="350206" & year==2005

replace regioncode="350211" if regioncode=="350211" & year==2005

replace regioncode="350212" if regioncode=="350212" & year==2005

replace regioncode="350213" if regioncode=="350213" & year==2005

replace regioncode="350302" if regioncode=="350302" & year==2005

replace regioncode="350303" if regioncode=="350303" & year==2005

replace regioncode="350321" if regioncode=="350321" & year==2005

replace regioncode="350322" if regioncode=="350322" & year==2005

replace regioncode="350402" if regioncode=="350402" & year==2005

replace regioncode="350403" if regioncode=="350403" & year==2005

replace regioncode="350421" if regioncode=="350421" & year==2005

replace regioncode="350423" if regioncode=="350423" & year==2005

replace regioncode="350424" if regioncode=="350424" & year==2005

replace regioncode="350425" if regioncode=="350425" & year==2005

replace regioncode="350426" if regioncode=="350426" & year==2005

replace regioncode="350427" if regioncode=="350427" & year==2005

replace regioncode="350428" if regioncode=="350428" & year==2005

replace regioncode="350429" if regioncode=="350429" & year==2005

replace regioncode="350430" if regioncode=="350430" & year==2005

replace regioncode="350481" if regioncode=="350481" & year==2005

replace regioncode="350502" if regioncode=="350502" & year==2005

replace regioncode="350503" if regioncode=="350503" & year==2005

replace regioncode="350504" if regioncode=="350504" & year==2005

replace regioncode="350505" if regioncode=="350505" & year==2005

replace regioncode="350521" if regioncode=="350521" & year==2005

replace regioncode="350524" if regioncode=="350524" & year==2005

replace regioncode="350525" if regioncode=="350525" & year==2005

replace regioncode="350526" if regioncode=="350526" & year==2005

replace regioncode="350581" if regioncode=="350581" & year==2005

replace regioncode="350582" if regioncode=="350582" & year==2005

replace regioncode="350583" if regioncode=="350583" & year==2005

replace regioncode="350602" if regioncode=="350602" & year==2005

replace regioncode="350603" if regioncode=="350603" & year==2005

replace regioncode="350622" if regioncode=="350622" & year==2005

replace regioncode="350623" if regioncode=="350623" & year==2005

replace regioncode="350624" if regioncode=="350624" & year==2005

replace regioncode="350625" if regioncode=="350625" & year==2005

replace regioncode="350626" if regioncode=="350626" & year==2005

replace regioncode="350627" if regioncode=="350627" & year==2005

replace regioncode="350628" if regioncode=="350628" & year==2005

replace regioncode="350629" if regioncode=="350629" & year==2005

replace regioncode="350681" if regioncode=="350681" & year==2005

replace regioncode="350702" if regioncode=="350702" & year==2005

replace regioncode="350721" if regioncode=="350721" & year==2005

replace regioncode="350722" if regioncode=="350722" & year==2005

replace regioncode="350723" if regioncode=="350723" & year==2005

replace regioncode="350724" if regioncode=="350724" & year==2005

replace regioncode="350725" if regioncode=="350725" & year==2005

replace regioncode="350781" if regioncode=="350781" & year==2005

replace regioncode="350782" if regioncode=="350782" & year==2005

replace regioncode="350783" if regioncode=="350783" & year==2005

replace regioncode="350784" if regioncode=="350784" & year==2005

replace regioncode="350802" if regioncode=="350802" & year==2005

replace regioncode="350821" if regioncode=="350821" & year==2005

replace regioncode="350822" if regioncode=="350822" & year==2005

replace regioncode="350823" if regioncode=="350823" & year==2005

replace regioncode="350824" if regioncode=="350824" & year==2005

replace regioncode="350825" if regioncode=="350825" & year==2005

replace regioncode="350881" if regioncode=="350881" & year==2005

replace regioncode="350902" if regioncode=="350902" & year==2005

replace regioncode="350981" if regioncode=="350981" & year==2005

replace regioncode="350982" if regioncode=="350982" & year==2005

replace regioncode="350921" if regioncode=="350921" & year==2005

replace regioncode="350922" if regioncode=="350922" & year==2005

replace regioncode="350923" if regioncode=="350923" & year==2005

replace regioncode="350924" if regioncode=="350924" & year==2005

replace regioncode="350925" if regioncode=="350925" & year==2005

replace regioncode="350926" if regioncode=="350926" & year==2005

replace regioncode="360102" if regioncode=="360102" & year==2005

replace regioncode="360103" if regioncode=="360103" & year==2005

replace regioncode="360104" if regioncode=="360104" & year==2005

replace regioncode="360105" if regioncode=="360105" & year==2005

replace regioncode="360111" if regioncode=="360111" & year==2005

replace regioncode="360121" if regioncode=="360121" & year==2005

replace regioncode="360122" if regioncode=="360122" & year==2005

replace regioncode="360123" if regioncode=="360123" & year==2005

replace regioncode="360124" if regioncode=="360124" & year==2005

replace regioncode="360202" if regioncode=="360202" & year==2005

replace regioncode="360203" if regioncode=="360203" & year==2005

replace regioncode="360222" if regioncode=="360222" & year==2005

replace regioncode="360281" if regioncode=="360281" & year==2005

replace regioncode="360302" if regioncode=="360302" & year==2005

replace regioncode="360313" if regioncode=="360313" & year==2005

replace regioncode="360321" if regioncode=="360321" & year==2005

replace regioncode="360322" if regioncode=="360322" & year==2005

replace regioncode="360323" if regioncode=="360323" & year==2005

replace regioncode="360402" if regioncode=="360402" & year==2005

replace regioncode="360403" if regioncode=="360403" & year==2005

replace regioncode="360421" if regioncode=="360421" & year==2005

replace regioncode="360423" if regioncode=="360423" & year==2005

replace regioncode="360424" if regioncode=="360424" & year==2005

replace regioncode="360425" if regioncode=="360425" & year==2005

replace regioncode="360426" if regioncode=="360426" & year==2005

replace regioncode="360427" if regioncode=="360427" & year==2005

replace regioncode="360428" if regioncode=="360428" & year==2005

replace regioncode="360429" if regioncode=="360429" & year==2005

replace regioncode="360430" if regioncode=="360430" & year==2005

replace regioncode="360481" if regioncode=="360481" & year==2005

replace regioncode="360502" if regioncode=="360502" & year==2005

replace regioncode="360521" if regioncode=="360521" & year==2005

replace regioncode="360602" if regioncode=="360602" & year==2005

replace regioncode="360622" if regioncode=="360622" & year==2005

replace regioncode="360681" if regioncode=="360681" & year==2005

replace regioncode="360702" if regioncode=="360702" & year==2005

replace regioncode="360721" if regioncode=="360721" & year==2005

replace regioncode="360722" if regioncode=="360722" & year==2005

replace regioncode="360723" if regioncode=="360723" & year==2005

replace regioncode="360724" if regioncode=="360724" & year==2005

replace regioncode="360725" if regioncode=="360725" & year==2005

replace regioncode="360726" if regioncode=="360726" & year==2005

replace regioncode="360727" if regioncode=="360727" & year==2005

replace regioncode="360728" if regioncode=="360728" & year==2005

replace regioncode="360729" if regioncode=="360729" & year==2005

replace regioncode="360730" if regioncode=="360730" & year==2005

replace regioncode="360731" if regioncode=="360731" & year==2005

replace regioncode="360732" if regioncode=="360732" & year==2005

replace regioncode="360733" if regioncode=="360733" & year==2005

replace regioncode="360734" if regioncode=="360734" & year==2005

replace regioncode="360735" if regioncode=="360735" & year==2005

replace regioncode="360781" if regioncode=="360781" & year==2005

replace regioncode="360782" if regioncode=="360782" & year==2005

replace regioncode="360802" if regioncode=="360802" & year==2005

replace regioncode="360821" if regioncode=="360821" & year==2005

replace regioncode="360822" if regioncode=="360822" & year==2005

replace regioncode="360823" if regioncode=="360823" & year==2005

replace regioncode="360824" if regioncode=="360824" & year==2005

replace regioncode="360825" if regioncode=="360825" & year==2005

replace regioncode="360826" if regioncode=="360826" & year==2005

replace regioncode="360827" if regioncode=="360827" & year==2005

replace regioncode="360828" if regioncode=="360828" & year==2005

replace regioncode="360829" if regioncode=="360829" & year==2005

replace regioncode="360830" if regioncode=="360830" & year==2005

replace regioncode="360881" if regioncode=="360881" & year==2005

replace regioncode="360902" if regioncode=="360902" & year==2005

replace regioncode="360921" if regioncode=="360921" & year==2005

replace regioncode="360922" if regioncode=="360922" & year==2005

replace regioncode="360923" if regioncode=="360923" & year==2005

replace regioncode="360924" if regioncode=="360924" & year==2005

replace regioncode="360925" if regioncode=="360925" & year==2005

replace regioncode="360926" if regioncode=="360926" & year==2005

replace regioncode="360981" if regioncode=="360981" & year==2005

replace regioncode="360982" if regioncode=="360982" & year==2005

replace regioncode="360983" if regioncode=="360983" & year==2005

replace regioncode="361002" if regioncode=="361002" & year==2005

replace regioncode="361021" if regioncode=="361021" & year==2005

replace regioncode="361022" if regioncode=="361022" & year==2005

replace regioncode="361023" if regioncode=="361023" & year==2005

replace regioncode="361024" if regioncode=="361024" & year==2005

replace regioncode="361025" if regioncode=="361025" & year==2005

replace regioncode="361026" if regioncode=="361026" & year==2005

replace regioncode="361027" if regioncode=="361027" & year==2005

replace regioncode="361028" if regioncode=="361028" & year==2005

replace regioncode="361029" if regioncode=="361029" & year==2005

replace regioncode="361030" if regioncode=="361030" & year==2005

replace regioncode="361102" if regioncode=="361102" & year==2005

replace regioncode="361121" if regioncode=="361121" & year==2005

replace regioncode="361122" if regioncode=="361122" & year==2005

replace regioncode="361123" if regioncode=="361123" & year==2005

replace regioncode="361124" if regioncode=="361124" & year==2005

replace regioncode="361125" if regioncode=="361125" & year==2005

replace regioncode="361126" if regioncode=="361126" & year==2005

replace regioncode="361127" if regioncode=="361127" & year==2005

replace regioncode="361128" if regioncode=="361128" & year==2005

replace regioncode="361129" if regioncode=="361129" & year==2005

replace regioncode="361130" if regioncode=="361130" & year==2005

replace regioncode="361181" if regioncode=="361181" & year==2005

replace regioncode="370102" if regioncode=="370102" & year==2005

replace regioncode="370103" if regioncode=="370103" & year==2005

replace regioncode="370104" if regioncode=="370104" & year==2005

replace regioncode="370105" if regioncode=="370105" & year==2005

replace regioncode="370112" if regioncode=="370112" & year==2005

replace regioncode="370113" if regioncode=="370113" & year==2005

replace regioncode="370124" if regioncode=="370124" & year==2005

replace regioncode="370125" if regioncode=="370125" & year==2005

replace regioncode="370126" if regioncode=="370126" & year==2005

replace regioncode="370181" if regioncode=="370181" & year==2005

replace regioncode="370202" if regioncode=="370202" & year==2005

replace regioncode="370203" if regioncode=="370203" & year==2005

replace regioncode="370205" if regioncode=="370205" & year==2005

replace regioncode="370211" if regioncode=="370211" & year==2005

replace regioncode="370212" if regioncode=="370212" & year==2005

replace regioncode="370213" if regioncode=="370213" & year==2005

replace regioncode="370214" if regioncode=="370214" & year==2005

replace regioncode="370281" if regioncode=="370281" & year==2005

replace regioncode="370282" if regioncode=="370282" & year==2005

replace regioncode="370283" if regioncode=="370283" & year==2005

replace regioncode="370284" if regioncode=="370284" & year==2005

replace regioncode="370285" if regioncode=="370285" & year==2005

replace regioncode="370302" if regioncode=="370302" & year==2005

replace regioncode="370303" if regioncode=="370303" & year==2005

replace regioncode="370304" if regioncode=="370304" & year==2005

replace regioncode="370305" if regioncode=="370305" & year==2005

replace regioncode="370306" if regioncode=="370306" & year==2005

replace regioncode="370321" if regioncode=="370321" & year==2005

replace regioncode="370322" if regioncode=="370322" & year==2005

replace regioncode="370323" if regioncode=="370323" & year==2005

replace regioncode="370402" if regioncode=="370402" & year==2005

replace regioncode="370403" if regioncode=="370403" & year==2005

replace regioncode="370404" if regioncode=="370404" & year==2005

replace regioncode="370405" if regioncode=="370405" & year==2005

replace regioncode="370406" if regioncode=="370406" & year==2005

replace regioncode="370481" if regioncode=="370481" & year==2005

replace regioncode="370502" if regioncode=="370502" & year==2005

replace regioncode="370503" if regioncode=="370503" & year==2005

replace regioncode="370521" if regioncode=="370521" & year==2005

replace regioncode="370522" if regioncode=="370522" & year==2005

replace regioncode="370523" if regioncode=="370523" & year==2005

replace regioncode="370602" if regioncode=="370602" & year==2005

replace regioncode="370611" if regioncode=="370611" & year==2005

replace regioncode="370612" if regioncode=="370612" & year==2005

replace regioncode="370613" if regioncode=="370613" & year==2005

replace regioncode="370634" if regioncode=="370634" & year==2005

replace regioncode="370681" if regioncode=="370681" & year==2005

replace regioncode="370682" if regioncode=="370682" & year==2005

replace regioncode="370683" if regioncode=="370683" & year==2005

replace regioncode="370684" if regioncode=="370684" & year==2005

replace regioncode="370685" if regioncode=="370685" & year==2005

replace regioncode="370686" if regioncode=="370686" & year==2005

replace regioncode="370687" if regioncode=="370687" & year==2005

replace regioncode="370702" if regioncode=="370702" & year==2005

replace regioncode="370703" if regioncode=="370703" & year==2005

replace regioncode="370704" if regioncode=="370704" & year==2005

replace regioncode="370705" if regioncode=="370705" & year==2005

replace regioncode="370724" if regioncode=="370724" & year==2005

replace regioncode="370725" if regioncode=="370725" & year==2005

replace regioncode="370781" if regioncode=="370781" & year==2005

replace regioncode="370782" if regioncode=="370782" & year==2005

replace regioncode="370783" if regioncode=="370783" & year==2005

replace regioncode="370784" if regioncode=="370784" & year==2005

replace regioncode="370785" if regioncode=="370785" & year==2005

replace regioncode="370786" if regioncode=="370786" & year==2005

replace regioncode="370802" if regioncode=="370802" & year==2005

replace regioncode="370811" if regioncode=="370811" & year==2005

replace regioncode="370826" if regioncode=="370826" & year==2005

replace regioncode="370827" if regioncode=="370827" & year==2005

replace regioncode="370828" if regioncode=="370828" & year==2005

replace regioncode="370829" if regioncode=="370829" & year==2005

replace regioncode="370830" if regioncode=="370830" & year==2005

replace regioncode="370831" if regioncode=="370831" & year==2005

replace regioncode="370832" if regioncode=="370832" & year==2005

replace regioncode="370881" if regioncode=="370881" & year==2005

replace regioncode="370882" if regioncode=="370882" & year==2005

replace regioncode="370883" if regioncode=="370883" & year==2005

replace regioncode="370902" if regioncode=="370902" & year==2005

replace regioncode="370903" if regioncode=="370903" & year==2005

replace regioncode="370921" if regioncode=="370921" & year==2005

replace regioncode="370923" if regioncode=="370923" & year==2005

replace regioncode="370982" if regioncode=="370982" & year==2005

replace regioncode="370983" if regioncode=="370983" & year==2005

replace regioncode="371002" if regioncode=="371002" & year==2005

replace regioncode="371081" if regioncode=="371081" & year==2005

replace regioncode="371082" if regioncode=="371082" & year==2005

replace regioncode="371083" if regioncode=="371083" & year==2005

replace regioncode="371102" if regioncode=="371102" & year==2005

replace regioncode="371121" if regioncode=="371121" & year==2005

replace regioncode="371122" if regioncode=="371122" & year==2005

replace regioncode="371202" if regioncode=="371202" & year==2005

replace regioncode="371203" if regioncode=="371203" & year==2005

replace regioncode="371302" if regioncode=="371302" & year==2005

replace regioncode="371311" if regioncode=="371311" & year==2005

replace regioncode="371312" if regioncode=="371312" & year==2005

replace regioncode="371321" if regioncode=="371321" & year==2005

replace regioncode="371322" if regioncode=="371322" & year==2005

replace regioncode="371323" if regioncode=="371323" & year==2005

replace regioncode="371324" if regioncode=="371324" & year==2005

replace regioncode="371325" if regioncode=="371325" & year==2005

replace regioncode="371326" if regioncode=="371326" & year==2005

replace regioncode="371327" if regioncode=="371327" & year==2005

replace regioncode="371328" if regioncode=="371328" & year==2005

replace regioncode="371329" if regioncode=="371329" & year==2005

replace regioncode="371402" if regioncode=="371402" & year==2005

replace regioncode="371421" if regioncode=="371421" & year==2005

replace regioncode="371422" if regioncode=="371422" & year==2005

replace regioncode="371423" if regioncode=="371423" & year==2005

replace regioncode="371424" if regioncode=="371424" & year==2005

replace regioncode="371425" if regioncode=="371425" & year==2005

replace regioncode="371426" if regioncode=="371426" & year==2005

replace regioncode="371427" if regioncode=="371427" & year==2005

replace regioncode="371428" if regioncode=="371428" & year==2005

replace regioncode="371481" if regioncode=="371481" & year==2005

replace regioncode="371482" if regioncode=="371482" & year==2005

replace regioncode="371502" if regioncode=="371502" & year==2005

replace regioncode="371521" if regioncode=="371521" & year==2005

replace regioncode="371522" if regioncode=="371522" & year==2005

replace regioncode="371523" if regioncode=="371523" & year==2005

replace regioncode="371524" if regioncode=="371524" & year==2005

replace regioncode="371525" if regioncode=="371525" & year==2005

replace regioncode="371526" if regioncode=="371526" & year==2005

replace regioncode="371581" if regioncode=="371581" & year==2005

replace regioncode="371602" if regioncode=="371602" & year==2005

replace regioncode="371621" if regioncode=="371621" & year==2005

replace regioncode="371622" if regioncode=="371622" & year==2005

replace regioncode="371623" if regioncode=="371623" & year==2005

replace regioncode="371624" if regioncode=="371624" & year==2005

replace regioncode="371625" if regioncode=="371625" & year==2005

replace regioncode="371626" if regioncode=="371626" & year==2005

replace regioncode="371702" if regioncode=="371702" & year==2005

replace regioncode="371721" if regioncode=="371721" & year==2005

replace regioncode="371722" if regioncode=="371722" & year==2005

replace regioncode="371723" if regioncode=="371723" & year==2005

replace regioncode="371724" if regioncode=="371724" & year==2005

replace regioncode="371725" if regioncode=="371725" & year==2005

replace regioncode="371726" if regioncode=="371726" & year==2005

replace regioncode="371727" if regioncode=="371727" & year==2005

replace regioncode="371728" if regioncode=="371728" & year==2005

replace regioncode="410102" if regioncode=="410102" & year==2005

replace regioncode="410103" if regioncode=="410103" & year==2005

replace regioncode="410104" if regioncode=="410104" & year==2005

replace regioncode="410105" if regioncode=="410105" & year==2005

replace regioncode="410106" if regioncode=="410106" & year==2005

replace regioncode="410108" if regioncode=="410108" & year==2005

replace regioncode="410122" if regioncode=="410122" & year==2005

replace regioncode="410181" if regioncode=="410181" & year==2005

replace regioncode="410182" if regioncode=="410182" & year==2005

replace regioncode="410183" if regioncode=="410183" & year==2005

replace regioncode="410184" if regioncode=="410184" & year==2005

replace regioncode="410185" if regioncode=="410185" & year==2005

replace regioncode="410202" if regioncode=="410202" & year==2005

replace regioncode="410203" if regioncode=="410203" & year==2005

replace regioncode="410204" if regioncode=="410204" & year==2005

replace regioncode="410205" if regioncode=="410205" & year==2005

replace regioncode="410211" if regioncode=="410211" & year==2005

replace regioncode="410221" if regioncode=="410221" & year==2005

replace regioncode="410222" if regioncode=="410222" & year==2005

replace regioncode="410223" if regioncode=="410223" & year==2005

replace regioncode="410224" if regioncode=="410224" & year==2005

replace regioncode="410225" if regioncode=="410225" & year==2005

replace regioncode="410302" if regioncode=="410302" & year==2005

replace regioncode="410303" if regioncode=="410303" & year==2005

replace regioncode="410304" if regioncode=="410304" & year==2005

replace regioncode="410305" if regioncode=="410305" & year==2005

replace regioncode="410306" if regioncode=="410306" & year==2005

replace regioncode="410307" if regioncode=="410307" & year==2005

replace regioncode="410322" if regioncode=="410322" & year==2005

replace regioncode="410323" if regioncode=="410323" & year==2005

replace regioncode="410324" if regioncode=="410324" & year==2005

replace regioncode="410325" if regioncode=="410325" & year==2005

replace regioncode="410326" if regioncode=="410326" & year==2005

replace regioncode="410327" if regioncode=="410327" & year==2005

replace regioncode="410328" if regioncode=="410328" & year==2005

replace regioncode="410329" if regioncode=="410329" & year==2005

replace regioncode="410381" if regioncode=="410381" & year==2005

replace regioncode="410402" if regioncode=="410402" & year==2005

replace regioncode="410403" if regioncode=="410403" & year==2005

replace regioncode="410404" if regioncode=="410404" & year==2005

replace regioncode="410411" if regioncode=="410411" & year==2005

replace regioncode="410421" if regioncode=="410421" & year==2005

replace regioncode="410422" if regioncode=="410422" & year==2005

replace regioncode="410423" if regioncode=="410423" & year==2005

replace regioncode="410425" if regioncode=="410425" & year==2005

replace regioncode="410481" if regioncode=="410481" & year==2005

replace regioncode="410482" if regioncode=="410482" & year==2005

replace regioncode="410502" if regioncode=="410502" & year==2005

replace regioncode="410503" if regioncode=="410503" & year==2005

replace regioncode="410504" if regioncode=="410505" & year==2005

replace regioncode="410511" if regioncode=="410506" & year==2005

replace regioncode="410522" if regioncode=="410522" & year==2005

replace regioncode="410523" if regioncode=="410523" & year==2005

replace regioncode="410526" if regioncode=="410526" & year==2005

replace regioncode="410527" if regioncode=="410527" & year==2005

replace regioncode="410581" if regioncode=="410581" & year==2005

replace regioncode="410602" if regioncode=="410602" & year==2005

replace regioncode="410603" if regioncode=="410603" & year==2005

replace regioncode="410611" if regioncode=="410611" & year==2005

replace regioncode="410621" if regioncode=="410621" & year==2005

replace regioncode="410622" if regioncode=="410622" & year==2005

replace regioncode="410702" if regioncode=="410702" & year==2005

replace regioncode="410703" if regioncode=="410703" & year==2005

replace regioncode="410704" if regioncode=="410704" & year==2005

replace regioncode="410711" if regioncode=="410711" & year==2005

replace regioncode="410721" if regioncode=="410721" & year==2005

replace regioncode="410724" if regioncode=="410724" & year==2005

replace regioncode="410725" if regioncode=="410725" & year==2005

replace regioncode="410726" if regioncode=="410726" & year==2005

replace regioncode="410727" if regioncode=="410727" & year==2005

replace regioncode="410728" if regioncode=="410728" & year==2005

replace regioncode="410781" if regioncode=="410781" & year==2005

replace regioncode="410782" if regioncode=="410782" & year==2005

replace regioncode="410802" if regioncode=="410802" & year==2005

replace regioncode="410803" if regioncode=="410803" & year==2005

replace regioncode="410804" if regioncode=="410804" & year==2005

replace regioncode="410811" if regioncode=="410811" & year==2005

replace regioncode="410821" if regioncode=="410821" & year==2005

replace regioncode="410822" if regioncode=="410822" & year==2005

replace regioncode="410823" if regioncode=="410823" & year==2005

replace regioncode="410825" if regioncode=="410825" & year==2005

replace regioncode="410881" if regioncode=="410881" & year==2005

replace regioncode="410882" if regioncode=="410882" & year==2005

replace regioncode="410883" if regioncode=="410883" & year==2005

replace regioncode="410902" if regioncode=="410902" & year==2005

replace regioncode="410922" if regioncode=="410922" & year==2005

replace regioncode="410923" if regioncode=="410923" & year==2005

replace regioncode="410926" if regioncode=="410926" & year==2005

replace regioncode="410927" if regioncode=="410927" & year==2005

replace regioncode="410928" if regioncode=="410928" & year==2005

replace regioncode="411002" if regioncode=="411002" & year==2005

replace regioncode="411023" if regioncode=="411023" & year==2005

replace regioncode="411024" if regioncode=="411024" & year==2005

replace regioncode="411025" if regioncode=="411025" & year==2005

replace regioncode="411081" if regioncode=="411081" & year==2005

replace regioncode="411082" if regioncode=="411082" & year==2005

replace regioncode="411102" if regioncode=="411102" & year==2005

replace regioncode="411123" if regioncode=="411103" & year==2005

replace regioncode="411121" if regioncode=="411121" & year==2005

replace regioncode="411122" if regioncode=="411122" & year==2005

replace regioncode="411202" if regioncode=="411202" & year==2005

replace regioncode="411221" if regioncode=="411221" & year==2005

replace regioncode="411222" if regioncode=="411222" & year==2005

replace regioncode="411224" if regioncode=="411224" & year==2005

replace regioncode="411281" if regioncode=="411281" & year==2005

replace regioncode="411282" if regioncode=="411282" & year==2005

replace regioncode="411302" if regioncode=="411302" & year==2005

replace regioncode="411303" if regioncode=="411303" & year==2005

replace regioncode="411321" if regioncode=="411321" & year==2005

replace regioncode="411322" if regioncode=="411322" & year==2005

replace regioncode="411323" if regioncode=="411323" & year==2005

replace regioncode="411324" if regioncode=="411324" & year==2005

replace regioncode="411325" if regioncode=="411325" & year==2005

replace regioncode="411326" if regioncode=="411326" & year==2005

replace regioncode="411327" if regioncode=="411327" & year==2005

replace regioncode="411328" if regioncode=="411328" & year==2005

replace regioncode="411329" if regioncode=="411329" & year==2005

replace regioncode="411330" if regioncode=="411330" & year==2005

replace regioncode="411381" if regioncode=="411381" & year==2005

replace regioncode="411402" if regioncode=="411402" & year==2005

replace regioncode="411403" if regioncode=="411403" & year==2005

replace regioncode="411421" if regioncode=="411421" & year==2005

replace regioncode="411422" if regioncode=="411422" & year==2005

replace regioncode="411423" if regioncode=="411423" & year==2005

replace regioncode="411424" if regioncode=="411424" & year==2005

replace regioncode="411425" if regioncode=="411425" & year==2005

replace regioncode="411426" if regioncode=="411426" & year==2005

replace regioncode="411481" if regioncode=="411481" & year==2005

replace regioncode="411502" if regioncode=="411502" & year==2005

replace regioncode="411503" if regioncode=="411503" & year==2005

replace regioncode="411521" if regioncode=="411521" & year==2005

replace regioncode="411522" if regioncode=="411522" & year==2005

replace regioncode="411523" if regioncode=="411523" & year==2005

replace regioncode="411524" if regioncode=="411524" & year==2005

replace regioncode="411525" if regioncode=="411525" & year==2005

replace regioncode="411526" if regioncode=="411526" & year==2005

replace regioncode="411527" if regioncode=="411527" & year==2005

replace regioncode="411528" if regioncode=="411528" & year==2005

replace regioncode="411602" if regioncode=="411602" & year==2005

replace regioncode="411621" if regioncode=="411621" & year==2005

replace regioncode="411622" if regioncode=="411622" & year==2005

replace regioncode="411623" if regioncode=="411623" & year==2005

replace regioncode="411624" if regioncode=="411624" & year==2005

replace regioncode="411625" if regioncode=="411625" & year==2005

replace regioncode="411626" if regioncode=="411626" & year==2005

replace regioncode="411627" if regioncode=="411627" & year==2005

replace regioncode="411628" if regioncode=="411628" & year==2005

replace regioncode="411681" if regioncode=="411681" & year==2005

replace regioncode="411702" if regioncode=="411702" & year==2005

replace regioncode="411721" if regioncode=="411721" & year==2005

replace regioncode="411722" if regioncode=="411722" & year==2005

replace regioncode="411723" if regioncode=="411723" & year==2005

replace regioncode="411724" if regioncode=="411724" & year==2005

replace regioncode="411725" if regioncode=="411725" & year==2005

replace regioncode="411726" if regioncode=="411726" & year==2005

replace regioncode="411727" if regioncode=="411727" & year==2005

replace regioncode="411728" if regioncode=="411728" & year==2005

replace regioncode="411729" if regioncode=="411729" & year==2005

replace regioncode="420102" if regioncode=="420102" & year==2005

replace regioncode="420103" if regioncode=="420103" & year==2005

replace regioncode="420104" if regioncode=="420104" & year==2005

replace regioncode="420105" if regioncode=="420105" & year==2005

replace regioncode="420106" if regioncode=="420106" & year==2005

replace regioncode="420107" if regioncode=="420107" & year==2005

replace regioncode="420111" if regioncode=="420111" & year==2005

replace regioncode="420112" if regioncode=="420112" & year==2005

replace regioncode="420113" if regioncode=="420113" & year==2005

replace regioncode="420114" if regioncode=="420114" & year==2005

replace regioncode="420115" if regioncode=="420115" & year==2005

replace regioncode="420116" if regioncode=="420116" & year==2005

replace regioncode="420117" if regioncode=="420117" & year==2005

replace regioncode="420202" if regioncode=="420202" & year==2005

replace regioncode="420203" if regioncode=="420203" & year==2005

replace regioncode="420204" if regioncode=="420204" & year==2005

replace regioncode="420205" if regioncode=="420205" & year==2005

replace regioncode="420222" if regioncode=="420222" & year==2005

replace regioncode="420281" if regioncode=="420281" & year==2005

replace regioncode="420302" if regioncode=="420302" & year==2005

replace regioncode="420303" if regioncode=="420303" & year==2005

replace regioncode="420321" if regioncode=="420321" & year==2005

replace regioncode="420322" if regioncode=="420322" & year==2005

replace regioncode="420323" if regioncode=="420323" & year==2005

replace regioncode="420324" if regioncode=="420324" & year==2005

replace regioncode="420325" if regioncode=="420325" & year==2005

replace regioncode="420381" if regioncode=="420381" & year==2005

replace regioncode="420502" if regioncode=="420502" & year==2005

replace regioncode="420503" if regioncode=="420503" & year==2005

replace regioncode="420504" if regioncode=="420504" & year==2005

replace regioncode="420505" if regioncode=="420505" & year==2005

replace regioncode="420506" if regioncode=="420506" & year==2005

replace regioncode="420525" if regioncode=="420525" & year==2005

replace regioncode="420526" if regioncode=="420526" & year==2005

replace regioncode="420527" if regioncode=="420527" & year==2005

replace regioncode="420528" if regioncode=="420528" & year==2005

replace regioncode="420529" if regioncode=="420529" & year==2005

replace regioncode="420581" if regioncode=="420581" & year==2005

replace regioncode="420582" if regioncode=="420582" & year==2005

replace regioncode="420583" if regioncode=="420583" & year==2005

replace regioncode="420602" if regioncode=="420602" & year==2005

replace regioncode="420606" if regioncode=="420606" & year==2005

replace regioncode="420607" if regioncode=="420607" & year==2005

replace regioncode="420624" if regioncode=="420624" & year==2005

replace regioncode="420625" if regioncode=="420625" & year==2005

replace regioncode="420626" if regioncode=="420626" & year==2005

replace regioncode="420682" if regioncode=="420682" & year==2005

replace regioncode="420683" if regioncode=="420683" & year==2005

replace regioncode="420684" if regioncode=="420684" & year==2005

replace regioncode="420702" if regioncode=="420702" & year==2005

replace regioncode="420703" if regioncode=="420703" & year==2005

replace regioncode="420704" if regioncode=="420704" & year==2005

replace regioncode="420802" if regioncode=="420802" & year==2005

replace regioncode="420804" if regioncode=="420804" & year==2005

replace regioncode="420821" if regioncode=="420821" & year==2005

replace regioncode="420822" if regioncode=="420822" & year==2005

replace regioncode="420881" if regioncode=="420881" & year==2005

replace regioncode="420902" if regioncode=="420902" & year==2005

replace regioncode="420921" if regioncode=="420921" & year==2005

replace regioncode="420922" if regioncode=="420922" & year==2005

replace regioncode="420923" if regioncode=="420923" & year==2005

replace regioncode="420981" if regioncode=="420981" & year==2005

replace regioncode="420982" if regioncode=="420982" & year==2005

replace regioncode="421381" if regioncode=="421381" & year==2005

replace regioncode="420984" if regioncode=="420984" & year==2005

replace regioncode="421002" if regioncode=="421002" & year==2005

replace regioncode="421003" if regioncode=="421003" & year==2005

replace regioncode="421022" if regioncode=="421022" & year==2005

replace regioncode="421023" if regioncode=="421023" & year==2005

replace regioncode="421024" if regioncode=="421024" & year==2005

replace regioncode="421081" if regioncode=="421081" & year==2005

replace regioncode="421083" if regioncode=="421083" & year==2005

replace regioncode="421087" if regioncode=="421087" & year==2005

replace regioncode="421102" if regioncode=="421102" & year==2005

replace regioncode="421121" if regioncode=="421121" & year==2005

replace regioncode="421122" if regioncode=="421122" & year==2005

replace regioncode="421123" if regioncode=="421123" & year==2005

replace regioncode="421124" if regioncode=="421124" & year==2005

replace regioncode="421125" if regioncode=="421125" & year==2005

replace regioncode="421126" if regioncode=="421126" & year==2005

replace regioncode="421127" if regioncode=="421127" & year==2005

replace regioncode="421181" if regioncode=="421181" & year==2005

replace regioncode="421182" if regioncode=="421182" & year==2005

replace regioncode="421202" if regioncode=="421202" & year==2005

replace regioncode="421221" if regioncode=="421221" & year==2005

replace regioncode="421222" if regioncode=="421222" & year==2005

replace regioncode="421223" if regioncode=="421223" & year==2005

replace regioncode="421224" if regioncode=="421224" & year==2005

replace regioncode="421281" if regioncode=="421281" & year==2005

replace regioncode="421302" if regioncode=="421302" & year==2005

replace regioncode="422801" if regioncode=="422801" & year==2005

replace regioncode="422802" if regioncode=="422802" & year==2005

replace regioncode="422822" if regioncode=="422822" & year==2005

replace regioncode="422823" if regioncode=="422823" & year==2005

replace regioncode="422825" if regioncode=="422825" & year==2005

replace regioncode="422826" if regioncode=="422826" & year==2005

replace regioncode="422827" if regioncode=="422827" & year==2005

replace regioncode="422828" if regioncode=="422828" & year==2005

replace regioncode="429004" if regioncode=="429004" & year==2005

replace regioncode="429005" if regioncode=="429005" & year==2005

replace regioncode="429006" if regioncode=="429006" & year==2005

replace regioncode="429021" if regioncode=="429021" & year==2005

replace regioncode="430102" if regioncode=="430102" & year==2005

replace regioncode="430103" if regioncode=="430103" & year==2005

replace regioncode="430104" if regioncode=="430104" & year==2005

replace regioncode="430105" if regioncode=="430105" & year==2005

replace regioncode="430111" if regioncode=="430111" & year==2005

replace regioncode="430121" if regioncode=="430121" & year==2005

replace regioncode="430122" if regioncode=="430122" & year==2005

replace regioncode="430124" if regioncode=="430124" & year==2005

replace regioncode="430181" if regioncode=="430181" & year==2005

replace regioncode="430202" if regioncode=="430202" & year==2005

replace regioncode="430203" if regioncode=="430203" & year==2005

replace regioncode="430204" if regioncode=="430204" & year==2005

replace regioncode="430211" if regioncode=="430211" & year==2005

replace regioncode="430221" if regioncode=="430221" & year==2005

replace regioncode="430223" if regioncode=="430223" & year==2005

replace regioncode="430224" if regioncode=="430224" & year==2005

replace regioncode="430225" if regioncode=="430225" & year==2005

replace regioncode="430281" if regioncode=="430281" & year==2005

replace regioncode="430302" if regioncode=="430302" & year==2005

replace regioncode="430304" if regioncode=="430304" & year==2005

replace regioncode="430321" if regioncode=="430321" & year==2005

replace regioncode="430381" if regioncode=="430381" & year==2005

replace regioncode="430382" if regioncode=="430382" & year==2005

replace regioncode="430405" if regioncode=="430405" & year==2005

replace regioncode="430406" if regioncode=="430406" & year==2005

replace regioncode="430407" if regioncode=="430407" & year==2005

replace regioncode="430408" if regioncode=="430408" & year==2005

replace regioncode="430412" if regioncode=="430412" & year==2005

replace regioncode="430421" if regioncode=="430421" & year==2005

replace regioncode="430422" if regioncode=="430422" & year==2005

replace regioncode="430423" if regioncode=="430423" & year==2005

replace regioncode="430424" if regioncode=="430424" & year==2005

replace regioncode="430426" if regioncode=="430426" & year==2005

replace regioncode="430481" if regioncode=="430481" & year==2005

replace regioncode="430482" if regioncode=="430482" & year==2005

replace regioncode="430502" if regioncode=="430502" & year==2005

replace regioncode="430503" if regioncode=="430503" & year==2005

replace regioncode="430511" if regioncode=="430511" & year==2005

replace regioncode="430521" if regioncode=="430521" & year==2005

replace regioncode="430522" if regioncode=="430522" & year==2005

replace regioncode="430523" if regioncode=="430523" & year==2005

replace regioncode="430524" if regioncode=="430524" & year==2005

replace regioncode="430525" if regioncode=="430525" & year==2005

replace regioncode="430527" if regioncode=="430527" & year==2005

replace regioncode="430528" if regioncode=="430528" & year==2005

replace regioncode="430529" if regioncode=="430529" & year==2005

replace regioncode="430581" if regioncode=="430581" & year==2005

replace regioncode="430602" if regioncode=="430602" & year==2005

replace regioncode="430603" if regioncode=="430603" & year==2005

replace regioncode="430611" if regioncode=="430611" & year==2005

replace regioncode="430621" if regioncode=="430621" & year==2005

replace regioncode="430623" if regioncode=="430623" & year==2005

replace regioncode="430624" if regioncode=="430624" & year==2005

replace regioncode="430626" if regioncode=="430626" & year==2005

replace regioncode="430681" if regioncode=="430681" & year==2005

replace regioncode="430682" if regioncode=="430682" & year==2005

replace regioncode="430702" if regioncode=="430702" & year==2005

replace regioncode="430703" if regioncode=="430703" & year==2005

replace regioncode="430721" if regioncode=="430721" & year==2005

replace regioncode="430722" if regioncode=="430722" & year==2005

replace regioncode="430723" if regioncode=="430723" & year==2005

replace regioncode="430724" if regioncode=="430724" & year==2005

replace regioncode="430725" if regioncode=="430725" & year==2005

replace regioncode="430726" if regioncode=="430726" & year==2005

replace regioncode="430781" if regioncode=="430781" & year==2005

replace regioncode="430802" if regioncode=="430802" & year==2005

replace regioncode="430811" if regioncode=="430811" & year==2005

replace regioncode="430821" if regioncode=="430821" & year==2005

replace regioncode="430822" if regioncode=="430822" & year==2005

replace regioncode="430902" if regioncode=="430902" & year==2005

replace regioncode="430903" if regioncode=="430903" & year==2005

replace regioncode="430921" if regioncode=="430921" & year==2005

replace regioncode="430922" if regioncode=="430922" & year==2005

replace regioncode="430923" if regioncode=="430923" & year==2005

replace regioncode="430981" if regioncode=="430981" & year==2005

replace regioncode="431002" if regioncode=="431002" & year==2005

replace regioncode="431003" if regioncode=="431003" & year==2005

replace regioncode="431021" if regioncode=="431021" & year==2005

replace regioncode="431022" if regioncode=="431022" & year==2005

replace regioncode="431023" if regioncode=="431023" & year==2005

replace regioncode="431024" if regioncode=="431024" & year==2005

replace regioncode="431025" if regioncode=="431025" & year==2005

replace regioncode="431026" if regioncode=="431026" & year==2005

replace regioncode="431027" if regioncode=="431027" & year==2005

replace regioncode="431028" if regioncode=="431028" & year==2005

replace regioncode="431081" if regioncode=="431081" & year==2005

replace regioncode="431102" if regioncode=="431102" & year==2005

replace regioncode="431103" if regioncode=="431103" & year==2005

replace regioncode="431121" if regioncode=="431121" & year==2005

replace regioncode="431122" if regioncode=="431122" & year==2005

replace regioncode="431123" if regioncode=="431123" & year==2005

replace regioncode="431124" if regioncode=="431124" & year==2005

replace regioncode="431125" if regioncode=="431125" & year==2005

replace regioncode="431126" if regioncode=="431126" & year==2005

replace regioncode="431127" if regioncode=="431127" & year==2005

replace regioncode="431128" if regioncode=="431128" & year==2005

replace regioncode="431129" if regioncode=="431129" & year==2005

replace regioncode="431202" if regioncode=="431202" & year==2005

replace regioncode="431221" if regioncode=="431221" & year==2005

replace regioncode="431222" if regioncode=="431222" & year==2005

replace regioncode="431223" if regioncode=="431223" & year==2005

replace regioncode="431224" if regioncode=="431224" & year==2005

replace regioncode="431225" if regioncode=="431225" & year==2005

replace regioncode="431226" if regioncode=="431226" & year==2005

replace regioncode="431227" if regioncode=="431227" & year==2005

replace regioncode="431228" if regioncode=="431228" & year==2005

replace regioncode="431229" if regioncode=="431229" & year==2005

replace regioncode="431230" if regioncode=="431230" & year==2005

replace regioncode="431281" if regioncode=="431281" & year==2005

replace regioncode="431302" if regioncode=="431302" & year==2005

replace regioncode="431321" if regioncode=="431321" & year==2005

replace regioncode="431322" if regioncode=="431322" & year==2005

replace regioncode="431381" if regioncode=="431381" & year==2005

replace regioncode="431382" if regioncode=="431382" & year==2005

replace regioncode="433101" if regioncode=="433101" & year==2005

replace regioncode="433122" if regioncode=="433122" & year==2005

replace regioncode="433123" if regioncode=="433123" & year==2005

replace regioncode="433124" if regioncode=="433124" & year==2005

replace regioncode="433125" if regioncode=="433125" & year==2005

replace regioncode="433126" if regioncode=="433126" & year==2005

replace regioncode="433127" if regioncode=="433127" & year==2005

replace regioncode="433130" if regioncode=="433130" & year==2005

replace regioncode="440102" if regioncode=="440102" & year==2005

replace regioncode="440103" if regioncode=="440103" & year==2005

replace regioncode="440104" if regioncode=="440104" & year==2005

replace regioncode="440105" if regioncode=="440105" & year==2005

replace regioncode="440106" if regioncode=="440106" & year==2005

replace regioncode="440107" if regioncode=="440116" & year==2005

replace regioncode="440111" if regioncode=="440111" & year==2005

replace regioncode="440112" if regioncode=="440112" & year==2005

replace regioncode="440113" if regioncode=="440113" & year==2005

replace regioncode="440114" if regioncode=="440114" & year==2005

replace regioncode="440183" if regioncode=="440183" & year==2005

replace regioncode="440184" if regioncode=="440184" & year==2005

replace regioncode="440203" if regioncode=="440203" & year==2005

replace regioncode="440204" if regioncode=="440204" & year==2005

replace regioncode="440221" if regioncode=="440205" & year==2005

replace regioncode="440222" if regioncode=="440222" & year==2005

replace regioncode="440224" if regioncode=="440224" & year==2005

replace regioncode="440229" if regioncode=="440229" & year==2005

replace regioncode="440232" if regioncode=="440232" & year==2005

replace regioncode="440233" if regioncode=="440233" & year==2005

replace regioncode="440281" if regioncode=="440281" & year==2005

replace regioncode="440282" if regioncode=="440282" & year==2005

replace regioncode="440303" if regioncode=="440303" & year==2005

replace regioncode="440304" if regioncode=="440304" & year==2005

replace regioncode="440305" if regioncode=="440305" & year==2005

replace regioncode="440306" if regioncode=="440306" & year==2005

replace regioncode="440307" if regioncode=="440307" & year==2005

replace regioncode="440308" if regioncode=="440308" & year==2005

replace regioncode="440402" if regioncode=="440402" & year==2005

replace regioncode="440403" if regioncode=="440403" & year==2005

replace regioncode="440404" if regioncode=="440404" & year==2005

replace regioncode="440507" if regioncode=="440507" & year==2005

replace regioncode="440511" if regioncode=="440511" & year==2005

replace regioncode="440512" if regioncode=="440512" & year==2005

replace regioncode="440514" if regioncode=="440514" & year==2005

replace regioncode="440515" if regioncode=="440515" & year==2005

replace regioncode="440523" if regioncode=="440523" & year==2005

replace regioncode="440604" if regioncode=="440604" & year==2005

replace regioncode="440605" if regioncode=="440605" & year==2005

replace regioncode="440606" if regioncode=="440606" & year==2005

replace regioncode="440607" if regioncode=="440607" & year==2005

replace regioncode="440608" if regioncode=="440608" & year==2005

replace regioncode="440703" if regioncode=="440703" & year==2005

replace regioncode="440704" if regioncode=="440704" & year==2005

replace regioncode="440781" if regioncode=="440781" & year==2005

replace regioncode="440705" if regioncode=="440705" & year==2005

replace regioncode="440783" if regioncode=="440783" & year==2005

replace regioncode="440784" if regioncode=="440784" & year==2005

replace regioncode="440785" if regioncode=="440785" & year==2005

replace regioncode="440802" if regioncode=="440802" & year==2005

replace regioncode="440803" if regioncode=="440803" & year==2005

replace regioncode="440804" if regioncode=="440804" & year==2005

replace regioncode="440811" if regioncode=="440811" & year==2005

replace regioncode="440823" if regioncode=="440823" & year==2005

replace regioncode="440825" if regioncode=="440825" & year==2005

replace regioncode="440881" if regioncode=="440881" & year==2005

replace regioncode="440882" if regioncode=="440882" & year==2005

replace regioncode="440883" if regioncode=="440883" & year==2005

replace regioncode="440902" if regioncode=="440902" & year==2005

replace regioncode="440903" if regioncode=="440903" & year==2005

replace regioncode="440923" if regioncode=="440923" & year==2005

replace regioncode="440981" if regioncode=="440981" & year==2005

replace regioncode="440982" if regioncode=="440982" & year==2005

replace regioncode="440983" if regioncode=="440983" & year==2005

replace regioncode="441202" if regioncode=="441202" & year==2005

replace regioncode="441203" if regioncode=="441203" & year==2005

replace regioncode="441223" if regioncode=="441223" & year==2005

replace regioncode="441224" if regioncode=="441224" & year==2005

replace regioncode="441225" if regioncode=="441225" & year==2005

replace regioncode="441226" if regioncode=="441226" & year==2005

replace regioncode="441283" if regioncode=="441283" & year==2005

replace regioncode="441284" if regioncode=="441284" & year==2005

replace regioncode="441302" if regioncode=="441302" & year==2005

replace regioncode="441303" if regioncode=="441303" & year==2005

replace regioncode="441322" if regioncode=="441322" & year==2005

replace regioncode="441323" if regioncode=="441323" & year==2005

replace regioncode="441324" if regioncode=="441324" & year==2005

replace regioncode="441402" if regioncode=="441402" & year==2005

replace regioncode="441421" if regioncode=="441421" & year==2005

replace regioncode="441422" if regioncode=="441422" & year==2005

replace regioncode="441423" if regioncode=="441423" & year==2005

replace regioncode="441424" if regioncode=="441424" & year==2005

replace regioncode="441426" if regioncode=="441426" & year==2005

replace regioncode="441427" if regioncode=="441427" & year==2005

replace regioncode="441481" if regioncode=="441481" & year==2005

replace regioncode="441502" if regioncode=="441502" & year==2005

replace regioncode="441521" if regioncode=="441521" & year==2005

replace regioncode="441523" if regioncode=="441523" & year==2005

replace regioncode="441581" if regioncode=="441581" & year==2005

replace regioncode="441602" if regioncode=="441602" & year==2005

replace regioncode="441621" if regioncode=="441621" & year==2005

replace regioncode="441622" if regioncode=="441622" & year==2005

replace regioncode="441623" if regioncode=="441623" & year==2005

replace regioncode="441624" if regioncode=="441624" & year==2005

replace regioncode="441625" if regioncode=="441625" & year==2005

replace regioncode="441702" if regioncode=="441702" & year==2005

replace regioncode="441721" if regioncode=="441721" & year==2005

replace regioncode="441723" if regioncode=="441723" & year==2005

replace regioncode="441781" if regioncode=="441781" & year==2005

replace regioncode="441802" if regioncode=="441802" & year==2005

replace regioncode="441821" if regioncode=="441821" & year==2005

replace regioncode="441823" if regioncode=="441823" & year==2005

replace regioncode="441825" if regioncode=="441825" & year==2005

replace regioncode="441826" if regioncode=="441826" & year==2005

replace regioncode="441827" if regioncode=="441827" & year==2005

replace regioncode="441881" if regioncode=="441881" & year==2005

replace regioncode="441882" if regioncode=="441882" & year==2005

replace regioncode="441900" if regioncode=="441900" & year==2005

replace regioncode="442000" if regioncode=="442000" & year==2005

replace regioncode="445102" if regioncode=="445102" & year==2005

replace regioncode="445121" if regioncode=="445121" & year==2005

replace regioncode="445122" if regioncode=="445122" & year==2005

replace regioncode="445202" if regioncode=="445202" & year==2005

replace regioncode="445221" if regioncode=="445221" & year==2005

replace regioncode="445222" if regioncode=="445222" & year==2005

replace regioncode="445224" if regioncode=="445224" & year==2005

replace regioncode="445281" if regioncode=="445281" & year==2005

replace regioncode="445302" if regioncode=="445302" & year==2005

replace regioncode="445321" if regioncode=="445321" & year==2005

replace regioncode="445322" if regioncode=="445322" & year==2005

replace regioncode="445323" if regioncode=="445323" & year==2005

replace regioncode="445381" if regioncode=="445381" & year==2005

replace regioncode="450102" if regioncode=="450102" & year==2005

replace regioncode="450103" if regioncode=="450103" & year==2005

replace regioncode="450104" if regioncode=="450107" & year==2005

replace regioncode="450105" if regioncode=="450105" & year==2005

replace regioncode="450106" if regioncode=="450108" & year==2005

replace regioncode="450121" if regioncode=="450109" & year==2005

replace regioncode="450122" if regioncode=="450122" & year==2005

replace regioncode="450123" if regioncode=="450123" & year==2005

replace regioncode="450124" if regioncode=="450124" & year==2005

replace regioncode="450125" if regioncode=="450125" & year==2005

replace regioncode="450126" if regioncode=="450126" & year==2005

replace regioncode="450127" if regioncode=="450127" & year==2005

replace regioncode="450202" if regioncode=="450202" & year==2005

replace regioncode="450203" if regioncode=="450203" & year==2005

replace regioncode="450204" if regioncode=="450204" & year==2005

replace regioncode="450205" if regioncode=="450205" & year==2005

replace regioncode="450221" if regioncode=="450221" & year==2005

replace regioncode="450222" if regioncode=="450222" & year==2005

replace regioncode="450223" if regioncode=="450223" & year==2005

replace regioncode="450224" if regioncode=="450224" & year==2005

replace regioncode="450225" if regioncode=="450225" & year==2005

replace regioncode="450226" if regioncode=="450226" & year==2005

replace regioncode="450302" if regioncode=="450302" & year==2005

replace regioncode="450303" if regioncode=="450303" & year==2005

replace regioncode="450304" if regioncode=="450304" & year==2005

replace regioncode="450305" if regioncode=="450305" & year==2005

replace regioncode="450311" if regioncode=="450311" & year==2005

replace regioncode="450321" if regioncode=="450321" & year==2005

replace regioncode="450322" if regioncode=="450322" & year==2005

replace regioncode="450323" if regioncode=="450323" & year==2005

replace regioncode="450324" if regioncode=="450324" & year==2005

replace regioncode="450325" if regioncode=="450325" & year==2005

replace regioncode="450326" if regioncode=="450326" & year==2005

replace regioncode="450327" if regioncode=="450327" & year==2005

replace regioncode="450328" if regioncode=="450328" & year==2005

replace regioncode="450329" if regioncode=="450329" & year==2005

replace regioncode="450330" if regioncode=="450330" & year==2005

replace regioncode="450331" if regioncode=="450331" & year==2005

replace regioncode="450332" if regioncode=="450332" & year==2005

replace regioncode="450403" if regioncode=="450403" & year==2005

replace regioncode="450404" if regioncode=="450404" & year==2005

replace regioncode="450421" if regioncode=="450421" & year==2005

replace regioncode="450422" if regioncode=="450422" & year==2005

replace regioncode="450423" if regioncode=="450423" & year==2005

replace regioncode="450481" if regioncode=="450481" & year==2005

replace regioncode="450502" if regioncode=="450502" & year==2005

replace regioncode="450503" if regioncode=="450503" & year==2005

replace regioncode="450512" if regioncode=="450512" & year==2005

replace regioncode="450521" if regioncode=="450521" & year==2005

replace regioncode="450602" if regioncode=="450602" & year==2005

replace regioncode="450603" if regioncode=="450603" & year==2005

replace regioncode="450621" if regioncode=="450621" & year==2005

replace regioncode="450681" if regioncode=="450681" & year==2005

replace regioncode="450702" if regioncode=="450702" & year==2005

replace regioncode="450703" if regioncode=="450703" & year==2005

replace regioncode="450721" if regioncode=="450721" & year==2005

replace regioncode="450722" if regioncode=="450722" & year==2005

replace regioncode="450802" if regioncode=="450802" & year==2005

replace regioncode="450803" if regioncode=="450803" & year==2005

replace regioncode="450804" if regioncode=="450804" & year==2005

replace regioncode="450821" if regioncode=="450821" & year==2005

replace regioncode="450881" if regioncode=="450881" & year==2005

replace regioncode="450902" if regioncode=="450902" & year==2005

replace regioncode="450921" if regioncode=="450921" & year==2005

replace regioncode="450922" if regioncode=="450922" & year==2005

replace regioncode="450923" if regioncode=="450923" & year==2005

replace regioncode="450924" if regioncode=="450924" & year==2005

replace regioncode="450981" if regioncode=="450981" & year==2005

replace regioncode="451002" if regioncode=="451002" & year==2005

replace regioncode="451021" if regioncode=="451021" & year==2005

replace regioncode="451022" if regioncode=="451022" & year==2005

replace regioncode="451023" if regioncode=="451023" & year==2005

replace regioncode="451024" if regioncode=="451024" & year==2005

replace regioncode="451025" if regioncode=="451025" & year==2005

replace regioncode="451026" if regioncode=="451026" & year==2005

replace regioncode="451027" if regioncode=="451027" & year==2005

replace regioncode="451028" if regioncode=="451028" & year==2005

replace regioncode="451029" if regioncode=="451029" & year==2005

replace regioncode="451030" if regioncode=="451030" & year==2005

replace regioncode="451031" if regioncode=="451031" & year==2005

replace regioncode="451102" if regioncode=="451102" & year==2005

replace regioncode="451121" if regioncode=="451121" & year==2005

replace regioncode="451122" if regioncode=="451122" & year==2005

replace regioncode="451123" if regioncode=="451123" & year==2005

replace regioncode="451202" if regioncode=="451202" & year==2005

replace regioncode="451221" if regioncode=="451221" & year==2005

replace regioncode="451222" if regioncode=="451222" & year==2005

replace regioncode="451223" if regioncode=="451223" & year==2005

replace regioncode="451224" if regioncode=="451224" & year==2005

replace regioncode="451225" if regioncode=="451225" & year==2005

replace regioncode="451226" if regioncode=="451226" & year==2005

replace regioncode="451227" if regioncode=="451227" & year==2005

replace regioncode="451228" if regioncode=="451228" & year==2005

replace regioncode="451229" if regioncode=="451229" & year==2005

replace regioncode="451281" if regioncode=="451281" & year==2005

replace regioncode="451302" if regioncode=="451302" & year==2005

replace regioncode="451321" if regioncode=="451321" & year==2005

replace regioncode="451322" if regioncode=="451322" & year==2005

replace regioncode="451323" if regioncode=="451323" & year==2005

replace regioncode="451324" if regioncode=="451324" & year==2005

replace regioncode="451381" if regioncode=="451381" & year==2005

replace regioncode="451402" if regioncode=="451402" & year==2005

replace regioncode="451421" if regioncode=="451421" & year==2005

replace regioncode="451422" if regioncode=="451422" & year==2005

replace regioncode="451423" if regioncode=="451423" & year==2005

replace regioncode="451424" if regioncode=="451424" & year==2005

replace regioncode="451425" if regioncode=="451425" & year==2005

replace regioncode="451481" if regioncode=="451481" & year==2005

replace regioncode="460105" if regioncode=="460105" & year==2005

replace regioncode="460106" if regioncode=="460106" & year==2005

replace regioncode="460107" if regioncode=="460107" & year==2005

replace regioncode="460108" if regioncode=="460108" & year==2005

replace regioncode="460001" if regioncode=="469001" & year==2005

replace regioncode="460002" if regioncode=="469002" & year==2005

replace regioncode="460003" if regioncode=="469003" & year==2005

replace regioncode="460005" if regioncode=="469005" & year==2005

replace regioncode="460006" if regioncode=="469006" & year==2005

replace regioncode="460007" if regioncode=="469007" & year==2005

replace regioncode="460030" if regioncode=="469025" & year==2005

replace regioncode="460031" if regioncode=="469026" & year==2005

replace regioncode="460033" if regioncode=="469027" & year==2005

replace regioncode="460034" if regioncode=="469028" & year==2005

replace regioncode="460036" if regioncode=="469030" & year==2005

replace regioncode="500101" if regioncode=="500101" & year==2005

replace regioncode="500102" if regioncode=="500102" & year==2005

replace regioncode="500103" if regioncode=="500103" & year==2005

replace regioncode="500104" if regioncode=="500104" & year==2005

replace regioncode="500105" if regioncode=="500105" & year==2005

replace regioncode="500106" if regioncode=="500106" & year==2005

replace regioncode="500107" if regioncode=="500107" & year==2005

replace regioncode="500108" if regioncode=="500108" & year==2005

replace regioncode="500109" if regioncode=="500109" & year==2005

replace regioncode="500110" if regioncode=="500110" & year==2005

replace regioncode="500111" if regioncode=="500111" & year==2005

replace regioncode="500112" if regioncode=="500112" & year==2005

replace regioncode="500113" if regioncode=="500113" & year==2005

replace regioncode="500114" if regioncode=="500114" & year==2005

replace regioncode="500115" if regioncode=="500115" & year==2005

replace regioncode="500381" if regioncode=="500381" & year==2005

replace regioncode="500382" if regioncode=="500382" & year==2005

replace regioncode="500383" if regioncode=="500383" & year==2005

replace regioncode="500384" if regioncode=="500384" & year==2005

replace regioncode="500222" if regioncode=="500222" & year==2005

replace regioncode="500223" if regioncode=="500223" & year==2005

replace regioncode="500224" if regioncode=="500224" & year==2005

replace regioncode="500225" if regioncode=="500225" & year==2005

replace regioncode="500226" if regioncode=="500226" & year==2005

replace regioncode="500227" if regioncode=="500227" & year==2005

replace regioncode="500228" if regioncode=="500228" & year==2005

replace regioncode="500229" if regioncode=="500229" & year==2005

replace regioncode="500230" if regioncode=="500230" & year==2005

replace regioncode="500231" if regioncode=="500231" & year==2005

replace regioncode="500232" if regioncode=="500232" & year==2005

replace regioncode="500233" if regioncode=="500233" & year==2005

replace regioncode="500234" if regioncode=="500234" & year==2005

replace regioncode="500235" if regioncode=="500235" & year==2005

replace regioncode="500236" if regioncode=="500236" & year==2005

replace regioncode="500237" if regioncode=="500237" & year==2005

replace regioncode="500238" if regioncode=="500238" & year==2005

replace regioncode="500240" if regioncode=="500240" & year==2005

replace regioncode="500241" if regioncode=="500241" & year==2005

replace regioncode="500242" if regioncode=="500242" & year==2005

replace regioncode="500243" if regioncode=="500243" & year==2005

replace regioncode="510104" if regioncode=="510104" & year==2005

replace regioncode="510105" if regioncode=="510105" & year==2005

replace regioncode="510106" if regioncode=="510106" & year==2005

replace regioncode="510107" if regioncode=="510107" & year==2005

replace regioncode="510108" if regioncode=="510108" & year==2005

replace regioncode="510112" if regioncode=="510112" & year==2005

replace regioncode="510113" if regioncode=="510113" & year==2005

replace regioncode="510114" if regioncode=="510114" & year==2005

replace regioncode="510115" if regioncode=="510115" & year==2005

replace regioncode="510121" if regioncode=="510121" & year==2005

replace regioncode="510122" if regioncode=="510122" & year==2005

replace regioncode="510124" if regioncode=="510124" & year==2005

replace regioncode="510129" if regioncode=="510129" & year==2005

replace regioncode="510131" if regioncode=="510131" & year==2005

replace regioncode="510132" if regioncode=="510132" & year==2005

replace regioncode="510181" if regioncode=="510181" & year==2005

replace regioncode="510182" if regioncode=="510182" & year==2005

replace regioncode="510183" if regioncode=="510183" & year==2005

replace regioncode="510184" if regioncode=="510184" & year==2005

replace regioncode="510302" if regioncode=="510302" & year==2005

replace regioncode="510303" if regioncode=="510303" & year==2005

replace regioncode="510304" if regioncode=="510304" & year==2005

replace regioncode="510311" if regioncode=="510311" & year==2005

replace regioncode="510321" if regioncode=="510321" & year==2005

replace regioncode="510322" if regioncode=="510322" & year==2005

replace regioncode="510402" if regioncode=="510402" & year==2005

replace regioncode="510403" if regioncode=="510403" & year==2005

replace regioncode="510411" if regioncode=="510411" & year==2005

replace regioncode="510421" if regioncode=="510421" & year==2005

replace regioncode="510422" if regioncode=="510422" & year==2005

replace regioncode="510502" if regioncode=="510502" & year==2005

replace regioncode="510503" if regioncode=="510503" & year==2005

replace regioncode="510504" if regioncode=="510504" & year==2005

replace regioncode="510521" if regioncode=="510521" & year==2005

replace regioncode="510522" if regioncode=="510522" & year==2005

replace regioncode="510524" if regioncode=="510524" & year==2005

replace regioncode="510525" if regioncode=="510525" & year==2005

replace regioncode="510603" if regioncode=="510603" & year==2005

replace regioncode="510623" if regioncode=="510623" & year==2005

replace regioncode="510626" if regioncode=="510626" & year==2005

replace regioncode="510681" if regioncode=="510681" & year==2005

replace regioncode="510682" if regioncode=="510682" & year==2005

replace regioncode="510683" if regioncode=="510683" & year==2005

replace regioncode="510703" if regioncode=="510703" & year==2005

replace regioncode="510704" if regioncode=="510704" & year==2005

replace regioncode="510722" if regioncode=="510722" & year==2005

replace regioncode="510723" if regioncode=="510723" & year==2005

replace regioncode="510724" if regioncode=="510724" & year==2005

replace regioncode="510725" if regioncode=="510725" & year==2005

replace regioncode="510726" if regioncode=="510726" & year==2005

replace regioncode="510727" if regioncode=="510727" & year==2005

replace regioncode="510781" if regioncode=="510781" & year==2005

replace regioncode="510802" if regioncode=="510802" & year==2005

replace regioncode="510811" if regioncode=="510811" & year==2005

replace regioncode="510812" if regioncode=="510812" & year==2005

replace regioncode="510821" if regioncode=="510821" & year==2005

replace regioncode="510822" if regioncode=="510822" & year==2005

replace regioncode="510823" if regioncode=="510823" & year==2005

replace regioncode="510824" if regioncode=="510824" & year==2005

replace regioncode="510902" if regioncode=="510903" & year==2005

replace regioncode="510921" if regioncode=="510921" & year==2005

replace regioncode="510922" if regioncode=="510922" & year==2005

replace regioncode="510923" if regioncode=="510923" & year==2005

replace regioncode="511002" if regioncode=="511002" & year==2005

replace regioncode="511011" if regioncode=="511011" & year==2005

replace regioncode="511024" if regioncode=="511024" & year==2005

replace regioncode="511025" if regioncode=="511025" & year==2005

replace regioncode="511028" if regioncode=="511028" & year==2005

replace regioncode="511102" if regioncode=="511102" & year==2005

replace regioncode="511111" if regioncode=="511111" & year==2005

replace regioncode="511112" if regioncode=="511112" & year==2005

replace regioncode="511113" if regioncode=="511113" & year==2005

replace regioncode="511123" if regioncode=="511123" & year==2005

replace regioncode="511124" if regioncode=="511124" & year==2005

replace regioncode="511126" if regioncode=="511126" & year==2005

replace regioncode="511129" if regioncode=="511129" & year==2005

replace regioncode="511132" if regioncode=="511132" & year==2005

replace regioncode="511133" if regioncode=="511133" & year==2005

replace regioncode="511181" if regioncode=="511181" & year==2005

replace regioncode="511302" if regioncode=="511302" & year==2005

replace regioncode="511303" if regioncode=="511303" & year==2005

replace regioncode="511304" if regioncode=="511304" & year==2005

replace regioncode="511321" if regioncode=="511321" & year==2005

replace regioncode="511322" if regioncode=="511322" & year==2005

replace regioncode="511323" if regioncode=="511323" & year==2005

replace regioncode="511324" if regioncode=="511324" & year==2005

replace regioncode="511325" if regioncode=="511325" & year==2005

replace regioncode="511381" if regioncode=="511381" & year==2005

replace regioncode="511402" if regioncode=="511402" & year==2005

replace regioncode="511421" if regioncode=="511421" & year==2005

replace regioncode="511422" if regioncode=="511422" & year==2005

replace regioncode="511423" if regioncode=="511423" & year==2005

replace regioncode="511424" if regioncode=="511424" & year==2005

replace regioncode="511425" if regioncode=="511425" & year==2005

replace regioncode="511502" if regioncode=="511502" & year==2005

replace regioncode="511521" if regioncode=="511521" & year==2005

replace regioncode="511522" if regioncode=="511522" & year==2005

replace regioncode="511523" if regioncode=="511523" & year==2005

replace regioncode="511524" if regioncode=="511524" & year==2005

replace regioncode="511525" if regioncode=="511525" & year==2005

replace regioncode="511526" if regioncode=="511526" & year==2005

replace regioncode="511527" if regioncode=="511527" & year==2005

replace regioncode="511528" if regioncode=="511528" & year==2005

replace regioncode="511529" if regioncode=="511529" & year==2005

replace regioncode="511602" if regioncode=="511602" & year==2005

replace regioncode="511621" if regioncode=="511621" & year==2005

replace regioncode="511622" if regioncode=="511622" & year==2005

replace regioncode="511623" if regioncode=="511623" & year==2005

replace regioncode="511681" if regioncode=="511681" & year==2005

replace regioncode="511702" if regioncode=="511702" & year==2005

replace regioncode="511721" if regioncode=="511721" & year==2005

replace regioncode="511722" if regioncode=="511722" & year==2005

replace regioncode="511723" if regioncode=="511723" & year==2005

replace regioncode="511724" if regioncode=="511724" & year==2005

replace regioncode="511725" if regioncode=="511725" & year==2005

replace regioncode="511781" if regioncode=="511781" & year==2005

replace regioncode="511802" if regioncode=="511802" & year==2005

replace regioncode="511821" if regioncode=="511821" & year==2005

replace regioncode="511822" if regioncode=="511822" & year==2005

replace regioncode="511823" if regioncode=="511823" & year==2005

replace regioncode="511824" if regioncode=="511824" & year==2005

replace regioncode="511825" if regioncode=="511825" & year==2005

replace regioncode="511826" if regioncode=="511826" & year==2005

replace regioncode="511827" if regioncode=="511827" & year==2005

replace regioncode="511902" if regioncode=="511902" & year==2005

replace regioncode="511921" if regioncode=="511921" & year==2005

replace regioncode="511922" if regioncode=="511922" & year==2005

replace regioncode="511923" if regioncode=="511923" & year==2005

replace regioncode="512002" if regioncode=="512002" & year==2005

replace regioncode="512021" if regioncode=="512021" & year==2005

replace regioncode="512022" if regioncode=="512022" & year==2005

replace regioncode="512081" if regioncode=="512081" & year==2005

replace regioncode="513221" if regioncode=="513221" & year==2005

replace regioncode="513222" if regioncode=="513222" & year==2005

replace regioncode="513223" if regioncode=="513223" & year==2005

replace regioncode="513224" if regioncode=="513224" & year==2005

replace regioncode="513225" if regioncode=="513225" & year==2005

replace regioncode="513226" if regioncode=="513226" & year==2005

replace regioncode="513227" if regioncode=="513227" & year==2005

replace regioncode="513228" if regioncode=="513228" & year==2005

replace regioncode="513229" if regioncode=="513229" & year==2005

replace regioncode="513230" if regioncode=="513230" & year==2005

replace regioncode="513231" if regioncode=="513231" & year==2005

replace regioncode="513232" if regioncode=="513232" & year==2005

replace regioncode="513233" if regioncode=="513233" & year==2005

replace regioncode="513321" if regioncode=="513321" & year==2005

replace regioncode="513322" if regioncode=="513322" & year==2005

replace regioncode="513323" if regioncode=="513323" & year==2005

replace regioncode="513324" if regioncode=="513324" & year==2005

replace regioncode="513325" if regioncode=="513325" & year==2005

replace regioncode="513326" if regioncode=="513326" & year==2005

replace regioncode="513327" if regioncode=="513327" & year==2005

replace regioncode="513328" if regioncode=="513328" & year==2005

replace regioncode="513329" if regioncode=="513329" & year==2005

replace regioncode="513330" if regioncode=="513330" & year==2005

replace regioncode="513331" if regioncode=="513331" & year==2005

replace regioncode="513332" if regioncode=="513332" & year==2005

replace regioncode="513333" if regioncode=="513333" & year==2005

replace regioncode="513334" if regioncode=="513334" & year==2005

replace regioncode="513335" if regioncode=="513335" & year==2005

replace regioncode="513336" if regioncode=="513336" & year==2005

replace regioncode="513337" if regioncode=="513337" & year==2005

replace regioncode="513338" if regioncode=="513338" & year==2005

replace regioncode="513401" if regioncode=="513401" & year==2005

replace regioncode="513422" if regioncode=="513422" & year==2005

replace regioncode="513423" if regioncode=="513423" & year==2005

replace regioncode="513424" if regioncode=="513424" & year==2005

replace regioncode="513425" if regioncode=="513425" & year==2005

replace regioncode="513426" if regioncode=="513426" & year==2005

replace regioncode="513427" if regioncode=="513427" & year==2005

replace regioncode="513428" if regioncode=="513428" & year==2005

replace regioncode="513429" if regioncode=="513429" & year==2005

replace regioncode="513430" if regioncode=="513430" & year==2005

replace regioncode="513431" if regioncode=="513431" & year==2005

replace regioncode="513432" if regioncode=="513432" & year==2005

replace regioncode="513433" if regioncode=="513433" & year==2005

replace regioncode="513434" if regioncode=="513434" & year==2005

replace regioncode="513435" if regioncode=="513435" & year==2005

replace regioncode="513436" if regioncode=="513436" & year==2005

replace regioncode="513437" if regioncode=="513437" & year==2005

replace regioncode="520102" if regioncode=="520102" & year==2005

replace regioncode="520103" if regioncode=="520103" & year==2005

replace regioncode="520111" if regioncode=="520111" & year==2005

replace regioncode="520112" if regioncode=="520112" & year==2005

replace regioncode="520113" if regioncode=="520113" & year==2005

replace regioncode="520114" if regioncode=="520114" & year==2005

replace regioncode="520121" if regioncode=="520121" & year==2005

replace regioncode="520122" if regioncode=="520122" & year==2005

replace regioncode="520123" if regioncode=="520123" & year==2005

replace regioncode="520181" if regioncode=="520181" & year==2005

replace regioncode="520201" if regioncode=="520201" & year==2005

replace regioncode="520222" if regioncode=="520222" & year==2005

replace regioncode="520203" if regioncode=="520203" & year==2005

replace regioncode="520221" if regioncode=="520221" & year==2005

replace regioncode="520302" if regioncode=="520302" & year==2005

replace regioncode="520321" if regioncode=="520321" & year==2005

replace regioncode="520322" if regioncode=="520322" & year==2005

replace regioncode="520323" if regioncode=="520323" & year==2005

replace regioncode="520324" if regioncode=="520324" & year==2005

replace regioncode="520325" if regioncode=="520325" & year==2005

replace regioncode="520326" if regioncode=="520326" & year==2005

replace regioncode="520327" if regioncode=="520327" & year==2005

replace regioncode="520328" if regioncode=="520328" & year==2005

replace regioncode="520329" if regioncode=="520329" & year==2005

replace regioncode="520330" if regioncode=="520330" & year==2005

replace regioncode="520381" if regioncode=="520381" & year==2005

replace regioncode="520382" if regioncode=="520382" & year==2005

replace regioncode="520402" if regioncode=="520402" & year==2005

replace regioncode="520421" if regioncode=="520421" & year==2005

replace regioncode="520422" if regioncode=="520422" & year==2005

replace regioncode="520423" if regioncode=="520423" & year==2005

replace regioncode="520424" if regioncode=="520424" & year==2005

replace regioncode="520425" if regioncode=="520425" & year==2005

replace regioncode="522201" if regioncode=="522201" & year==2005

replace regioncode="522222" if regioncode=="522222" & year==2005

replace regioncode="522223" if regioncode=="522223" & year==2005

replace regioncode="522224" if regioncode=="522224" & year==2005

replace regioncode="522225" if regioncode=="522225" & year==2005

replace regioncode="522226" if regioncode=="522226" & year==2005

replace regioncode="522227" if regioncode=="522227" & year==2005

replace regioncode="522228" if regioncode=="522228" & year==2005

replace regioncode="522229" if regioncode=="522229" & year==2005

replace regioncode="522230" if regioncode=="522230" & year==2005

replace regioncode="522301" if regioncode=="522301" & year==2005

replace regioncode="522322" if regioncode=="522322" & year==2005

replace regioncode="522323" if regioncode=="522323" & year==2005

replace regioncode="522324" if regioncode=="522324" & year==2005

replace regioncode="522325" if regioncode=="522325" & year==2005

replace regioncode="522326" if regioncode=="522326" & year==2005

replace regioncode="522327" if regioncode=="522327" & year==2005

replace regioncode="522328" if regioncode=="522328" & year==2005

replace regioncode="522401" if regioncode=="522401" & year==2005

replace regioncode="522422" if regioncode=="522422" & year==2005

replace regioncode="522423" if regioncode=="522423" & year==2005

replace regioncode="522424" if regioncode=="522424" & year==2005

replace regioncode="522425" if regioncode=="522425" & year==2005

replace regioncode="522426" if regioncode=="522426" & year==2005

replace regioncode="522427" if regioncode=="522427" & year==2005

replace regioncode="522428" if regioncode=="522428" & year==2005

replace regioncode="522601" if regioncode=="522601" & year==2005

replace regioncode="522622" if regioncode=="522622" & year==2005

replace regioncode="522623" if regioncode=="522623" & year==2005

replace regioncode="522624" if regioncode=="522624" & year==2005

replace regioncode="522625" if regioncode=="522625" & year==2005

replace regioncode="522626" if regioncode=="522626" & year==2005

replace regioncode="522627" if regioncode=="522627" & year==2005

replace regioncode="522628" if regioncode=="522628" & year==2005

replace regioncode="522629" if regioncode=="522629" & year==2005

replace regioncode="522630" if regioncode=="522630" & year==2005

replace regioncode="522631" if regioncode=="522631" & year==2005

replace regioncode="522632" if regioncode=="522632" & year==2005

replace regioncode="522633" if regioncode=="522633" & year==2005

replace regioncode="522634" if regioncode=="522634" & year==2005

replace regioncode="522635" if regioncode=="522635" & year==2005

replace regioncode="522636" if regioncode=="522636" & year==2005

replace regioncode="522701" if regioncode=="522701" & year==2005

replace regioncode="522702" if regioncode=="522702" & year==2005

replace regioncode="522722" if regioncode=="522722" & year==2005

replace regioncode="522723" if regioncode=="522723" & year==2005

replace regioncode="522725" if regioncode=="522725" & year==2005

replace regioncode="522726" if regioncode=="522726" & year==2005

replace regioncode="522727" if regioncode=="522727" & year==2005

replace regioncode="522728" if regioncode=="522728" & year==2005

replace regioncode="522729" if regioncode=="522729" & year==2005

replace regioncode="522730" if regioncode=="522730" & year==2005

replace regioncode="522731" if regioncode=="522731" & year==2005

replace regioncode="522732" if regioncode=="522732" & year==2005

replace regioncode="530102" if regioncode=="530102" & year==2005

replace regioncode="530103" if regioncode=="530103" & year==2005

replace regioncode="530111" if regioncode=="530111" & year==2005

replace regioncode="530112" if regioncode=="530112" & year==2005

replace regioncode="530113" if regioncode=="530113" & year==2005

replace regioncode="530121" if regioncode=="530121" & year==2005

replace regioncode="530122" if regioncode=="530122" & year==2005

replace regioncode="530124" if regioncode=="530124" & year==2005

replace regioncode="530125" if regioncode=="530125" & year==2005

replace regioncode="530126" if regioncode=="530126" & year==2005

replace regioncode="530127" if regioncode=="530127" & year==2005

replace regioncode="530128" if regioncode=="530128" & year==2005

replace regioncode="530129" if regioncode=="530129" & year==2005

replace regioncode="530181" if regioncode=="530181" & year==2005

replace regioncode="530302" if regioncode=="530302" & year==2005

replace regioncode="530321" if regioncode=="530321" & year==2005

replace regioncode="530322" if regioncode=="530322" & year==2005

replace regioncode="530323" if regioncode=="530323" & year==2005

replace regioncode="530324" if regioncode=="530324" & year==2005

replace regioncode="530325" if regioncode=="530325" & year==2005

replace regioncode="530326" if regioncode=="530326" & year==2005

replace regioncode="530328" if regioncode=="530328" & year==2005

replace regioncode="530381" if regioncode=="530381" & year==2005

replace regioncode="530402" if regioncode=="530402" & year==2005

replace regioncode="530421" if regioncode=="530421" & year==2005

replace regioncode="530422" if regioncode=="530422" & year==2005

replace regioncode="530423" if regioncode=="530423" & year==2005

replace regioncode="530424" if regioncode=="530424" & year==2005

replace regioncode="530425" if regioncode=="530425" & year==2005

replace regioncode="530426" if regioncode=="530426" & year==2005

replace regioncode="530427" if regioncode=="530427" & year==2005

replace regioncode="530428" if regioncode=="530428" & year==2005

replace regioncode="530502" if regioncode=="530502" & year==2005

replace regioncode="530521" if regioncode=="530521" & year==2005

replace regioncode="530522" if regioncode=="530522" & year==2005

replace regioncode="530523" if regioncode=="530523" & year==2005

replace regioncode="530524" if regioncode=="530524" & year==2005

replace regioncode="530602" if regioncode=="530602" & year==2005

replace regioncode="530621" if regioncode=="530621" & year==2005

replace regioncode="530622" if regioncode=="530622" & year==2005

replace regioncode="530623" if regioncode=="530623" & year==2005

replace regioncode="530624" if regioncode=="530624" & year==2005

replace regioncode="530625" if regioncode=="530625" & year==2005

replace regioncode="530626" if regioncode=="530626" & year==2005

replace regioncode="530627" if regioncode=="530627" & year==2005

replace regioncode="530628" if regioncode=="530628" & year==2005

replace regioncode="530629" if regioncode=="530629" & year==2005

replace regioncode="530630" if regioncode=="530630" & year==2005

replace regioncode="530721" if regioncode=="530721" & year==2005

replace regioncode="530722" if regioncode=="530722" & year==2005

replace regioncode="530723" if regioncode=="530723" & year==2005

replace regioncode="530724" if regioncode=="530724" & year==2005

replace regioncode="532701" if regioncode=="530802" & year==2005

replace regioncode="532722" if regioncode=="530821" & year==2005

replace regioncode="532723" if regioncode=="530822" & year==2005

replace regioncode="532724" if regioncode=="530823" & year==2005

replace regioncode="532725" if regioncode=="530824" & year==2005

replace regioncode="532726" if regioncode=="530825" & year==2005

replace regioncode="532727" if regioncode=="530826" & year==2005

replace regioncode="532728" if regioncode=="530827" & year==2005

replace regioncode="532729" if regioncode=="530828" & year==2005

replace regioncode="532730" if regioncode=="530829" & year==2005

replace regioncode="533521" if regioncode=="530902" & year==2005

replace regioncode="533522" if regioncode=="530921" & year==2005

replace regioncode="533523" if regioncode=="530922" & year==2005

replace regioncode="533524" if regioncode=="530923" & year==2005

replace regioncode="533525" if regioncode=="530924" & year==2005

replace regioncode="533526" if regioncode=="530925" & year==2005

replace regioncode="533527" if regioncode=="530926" & year==2005

replace regioncode="533528" if regioncode=="530927" & year==2005

replace regioncode="532301" if regioncode=="532301" & year==2005

replace regioncode="532322" if regioncode=="532322" & year==2005

replace regioncode="532323" if regioncode=="532323" & year==2005

replace regioncode="532324" if regioncode=="532324" & year==2005

replace regioncode="532325" if regioncode=="532325" & year==2005

replace regioncode="532326" if regioncode=="532326" & year==2005

replace regioncode="532327" if regioncode=="532327" & year==2005

replace regioncode="532328" if regioncode=="532328" & year==2005

replace regioncode="532329" if regioncode=="532329" & year==2005

replace regioncode="532331" if regioncode=="532331" & year==2005

replace regioncode="532501" if regioncode=="532501" & year==2005

replace regioncode="532502" if regioncode=="532502" & year==2005

replace regioncode="532522" if regioncode=="532522" & year==2005

replace regioncode="532523" if regioncode=="532523" & year==2005

replace regioncode="532524" if regioncode=="532524" & year==2005

replace regioncode="532525" if regioncode=="532525" & year==2005

replace regioncode="532526" if regioncode=="532526" & year==2005

replace regioncode="532527" if regioncode=="532527" & year==2005

replace regioncode="532528" if regioncode=="532528" & year==2005

replace regioncode="532529" if regioncode=="532529" & year==2005

replace regioncode="532530" if regioncode=="532530" & year==2005

replace regioncode="532531" if regioncode=="532531" & year==2005

replace regioncode="532532" if regioncode=="532532" & year==2005

replace regioncode="532621" if regioncode=="532621" & year==2005

replace regioncode="532622" if regioncode=="532622" & year==2005

replace regioncode="532623" if regioncode=="532623" & year==2005

replace regioncode="532624" if regioncode=="532624" & year==2005

replace regioncode="532625" if regioncode=="532625" & year==2005

replace regioncode="532626" if regioncode=="532626" & year==2005

replace regioncode="532627" if regioncode=="532627" & year==2005

replace regioncode="532628" if regioncode=="532628" & year==2005

replace regioncode="532801" if regioncode=="532801" & year==2005

replace regioncode="532822" if regioncode=="532822" & year==2005

replace regioncode="532823" if regioncode=="532823" & year==2005

replace regioncode="532901" if regioncode=="532901" & year==2005

replace regioncode="532922" if regioncode=="532922" & year==2005

replace regioncode="532923" if regioncode=="532923" & year==2005

replace regioncode="532924" if regioncode=="532924" & year==2005

replace regioncode="532925" if regioncode=="532925" & year==2005

replace regioncode="532926" if regioncode=="532926" & year==2005

replace regioncode="532927" if regioncode=="532927" & year==2005

replace regioncode="532928" if regioncode=="532928" & year==2005

replace regioncode="532929" if regioncode=="532929" & year==2005

replace regioncode="532930" if regioncode=="532930" & year==2005

replace regioncode="532931" if regioncode=="532931" & year==2005

replace regioncode="532932" if regioncode=="532932" & year==2005

replace regioncode="533102" if regioncode=="533102" & year==2005

replace regioncode="533103" if regioncode=="533103" & year==2005

replace regioncode="533122" if regioncode=="533122" & year==2005

replace regioncode="533123" if regioncode=="533123" & year==2005

replace regioncode="533124" if regioncode=="533124" & year==2005

replace regioncode="533321" if regioncode=="533321" & year==2005

replace regioncode="533323" if regioncode=="533323" & year==2005

replace regioncode="533324" if regioncode=="533324" & year==2005

replace regioncode="533325" if regioncode=="533325" & year==2005

replace regioncode="533421" if regioncode=="533421" & year==2005

replace regioncode="533422" if regioncode=="533422" & year==2005

replace regioncode="533423" if regioncode=="533423" & year==2005

replace regioncode="540102" if regioncode=="540102" & year==2005

replace regioncode="540121" if regioncode=="540121" & year==2005

replace regioncode="540122" if regioncode=="540122" & year==2005

replace regioncode="540123" if regioncode=="540123" & year==2005

replace regioncode="540124" if regioncode=="540124" & year==2005

replace regioncode="540125" if regioncode=="540125" & year==2005

replace regioncode="540126" if regioncode=="540126" & year==2005

replace regioncode="540127" if regioncode=="540127" & year==2005

replace regioncode="542121" if regioncode=="542121" & year==2005

replace regioncode="542122" if regioncode=="542122" & year==2005

replace regioncode="542123" if regioncode=="542123" & year==2005

replace regioncode="542124" if regioncode=="542124" & year==2005

replace regioncode="542125" if regioncode=="542125" & year==2005

replace regioncode="542126" if regioncode=="542126" & year==2005

replace regioncode="542127" if regioncode=="542127" & year==2005

replace regioncode="542128" if regioncode=="542128" & year==2005

replace regioncode="542129" if regioncode=="542129" & year==2005

replace regioncode="542132" if regioncode=="542132" & year==2005

replace regioncode="542133" if regioncode=="542133" & year==2005

replace regioncode="542221" if regioncode=="542221" & year==2005

replace regioncode="542223" if regioncode=="542223" & year==2005

replace regioncode="542224" if regioncode=="542224" & year==2005

replace regioncode="542225" if regioncode=="542225" & year==2005

replace regioncode="542227" if regioncode=="542227" & year==2005

replace regioncode="542228" if regioncode=="542228" & year==2005

replace regioncode="542229" if regioncode=="542229" & year==2005

replace regioncode="542231" if regioncode=="542231" & year==2005

replace regioncode="542232" if regioncode=="542232" & year==2005

replace regioncode="542233" if regioncode=="542233" & year==2005

replace regioncode="542301" if regioncode=="542301" & year==2005

replace regioncode="542322" if regioncode=="542322" & year==2005

replace regioncode="542323" if regioncode=="542323" & year==2005

replace regioncode="542324" if regioncode=="542324" & year==2005

replace regioncode="542325" if regioncode=="542325" & year==2005

replace regioncode="542326" if regioncode=="542326" & year==2005

replace regioncode="542327" if regioncode=="542327" & year==2005

replace regioncode="542328" if regioncode=="542328" & year==2005

replace regioncode="542329" if regioncode=="542329" & year==2005

replace regioncode="542330" if regioncode=="542330" & year==2005

replace regioncode="542331" if regioncode=="542331" & year==2005

replace regioncode="542332" if regioncode=="542332" & year==2005

replace regioncode="542334" if regioncode=="542334" & year==2005

replace regioncode="542336" if regioncode=="542336" & year==2005

replace regioncode="542338" if regioncode=="542338" & year==2005

replace regioncode="542421" if regioncode=="542421" & year==2005

replace regioncode="542422" if regioncode=="542422" & year==2005

replace regioncode="542423" if regioncode=="542423" & year==2005

replace regioncode="542424" if regioncode=="542424" & year==2005

replace regioncode="542425" if regioncode=="542425" & year==2005

replace regioncode="542426" if regioncode=="542426" & year==2005

replace regioncode="542427" if regioncode=="542427" & year==2005

replace regioncode="542429" if regioncode=="542429" & year==2005

replace regioncode="542430" if regioncode=="542430" & year==2005

replace regioncode="542523" if regioncode=="542523" & year==2005

replace regioncode="542621" if regioncode=="542621" & year==2005

replace regioncode="542622" if regioncode=="542622" & year==2005

replace regioncode="542623" if regioncode=="542623" & year==2005

replace regioncode="542625" if regioncode=="542625" & year==2005

replace regioncode="542626" if regioncode=="542626" & year==2005

replace regioncode="610102" if regioncode=="610102" & year==2005

replace regioncode="610103" if regioncode=="610103" & year==2005

replace regioncode="610104" if regioncode=="610104" & year==2005

replace regioncode="610111" if regioncode=="610111" & year==2005

replace regioncode="610112" if regioncode=="610112" & year==2005

replace regioncode="610113" if regioncode=="610113" & year==2005

replace regioncode="610114" if regioncode=="610114" & year==2005

replace regioncode="610115" if regioncode=="610115" & year==2005

replace regioncode="610116" if regioncode=="610116" & year==2005

replace regioncode="610122" if regioncode=="610122" & year==2005

replace regioncode="610124" if regioncode=="610124" & year==2005

replace regioncode="610125" if regioncode=="610125" & year==2005

replace regioncode="610126" if regioncode=="610126" & year==2005

replace regioncode="610202" if regioncode=="610202" & year==2005

replace regioncode="610203" if regioncode=="610203" & year==2005

replace regioncode="610204" if regioncode=="610204" & year==2005

replace regioncode="610222" if regioncode=="610222" & year==2005

replace regioncode="610302" if regioncode=="610302" & year==2005

replace regioncode="610303" if regioncode=="610303" & year==2005

replace regioncode="610304" if regioncode=="610304" & year==2005

replace regioncode="610322" if regioncode=="610322" & year==2005

replace regioncode="610323" if regioncode=="610323" & year==2005

replace regioncode="610324" if regioncode=="610324" & year==2005

replace regioncode="610326" if regioncode=="610326" & year==2005

replace regioncode="610327" if regioncode=="610327" & year==2005

replace regioncode="610328" if regioncode=="610328" & year==2005

replace regioncode="610329" if regioncode=="610329" & year==2005

replace regioncode="610330" if regioncode=="610330" & year==2005

replace regioncode="610331" if regioncode=="610331" & year==2005

replace regioncode="610402" if regioncode=="610402" & year==2005

replace regioncode="610403" if regioncode=="610403" & year==2005

replace regioncode="610404" if regioncode=="610404" & year==2005

replace regioncode="610422" if regioncode=="610422" & year==2005

replace regioncode="610423" if regioncode=="610423" & year==2005

replace regioncode="610424" if regioncode=="610424" & year==2005

replace regioncode="610425" if regioncode=="610425" & year==2005

replace regioncode="610426" if regioncode=="610426" & year==2005

replace regioncode="610427" if regioncode=="610427" & year==2005

replace regioncode="610428" if regioncode=="610428" & year==2005

replace regioncode="610429" if regioncode=="610429" & year==2005

replace regioncode="610430" if regioncode=="610430" & year==2005

replace regioncode="610431" if regioncode=="610431" & year==2005

replace regioncode="610481" if regioncode=="610481" & year==2005

replace regioncode="610502" if regioncode=="610502" & year==2005

replace regioncode="610521" if regioncode=="610521" & year==2005

replace regioncode="610522" if regioncode=="610522" & year==2005

replace regioncode="610523" if regioncode=="610523" & year==2005

replace regioncode="610524" if regioncode=="610524" & year==2005

replace regioncode="610525" if regioncode=="610525" & year==2005

replace regioncode="610526" if regioncode=="610526" & year==2005

replace regioncode="610527" if regioncode=="610527" & year==2005

replace regioncode="610528" if regioncode=="610528" & year==2005

replace regioncode="610581" if regioncode=="610581" & year==2005

replace regioncode="610582" if regioncode=="610582" & year==2005

replace regioncode="610602" if regioncode=="610602" & year==2005

replace regioncode="610621" if regioncode=="610621" & year==2005

replace regioncode="610622" if regioncode=="610622" & year==2005

replace regioncode="610623" if regioncode=="610623" & year==2005

replace regioncode="610624" if regioncode=="610624" & year==2005

replace regioncode="610625" if regioncode=="610625" & year==2005

replace regioncode="610626" if regioncode=="610626" & year==2005

replace regioncode="610627" if regioncode=="610627" & year==2005

replace regioncode="610628" if regioncode=="610628" & year==2005

replace regioncode="610629" if regioncode=="610629" & year==2005

replace regioncode="610630" if regioncode=="610630" & year==2005

replace regioncode="610631" if regioncode=="610631" & year==2005

replace regioncode="610632" if regioncode=="610632" & year==2005

replace regioncode="610702" if regioncode=="610702" & year==2005

replace regioncode="610721" if regioncode=="610721" & year==2005

replace regioncode="610722" if regioncode=="610722" & year==2005

replace regioncode="610723" if regioncode=="610723" & year==2005

replace regioncode="610724" if regioncode=="610724" & year==2005

replace regioncode="610725" if regioncode=="610725" & year==2005

replace regioncode="610726" if regioncode=="610726" & year==2005

replace regioncode="610727" if regioncode=="610727" & year==2005

replace regioncode="610728" if regioncode=="610728" & year==2005

replace regioncode="610729" if regioncode=="610729" & year==2005

replace regioncode="610730" if regioncode=="610730" & year==2005

replace regioncode="610802" if regioncode=="610802" & year==2005

replace regioncode="610821" if regioncode=="610821" & year==2005

replace regioncode="610822" if regioncode=="610822" & year==2005

replace regioncode="610823" if regioncode=="610823" & year==2005

replace regioncode="610824" if regioncode=="610824" & year==2005

replace regioncode="610825" if regioncode=="610825" & year==2005

replace regioncode="610826" if regioncode=="610826" & year==2005

replace regioncode="610827" if regioncode=="610827" & year==2005

replace regioncode="610828" if regioncode=="610828" & year==2005

replace regioncode="610829" if regioncode=="610829" & year==2005

replace regioncode="610830" if regioncode=="610830" & year==2005

replace regioncode="610831" if regioncode=="610831" & year==2005

replace regioncode="610902" if regioncode=="610902" & year==2005

replace regioncode="610921" if regioncode=="610921" & year==2005

replace regioncode="610922" if regioncode=="610922" & year==2005

replace regioncode="610923" if regioncode=="610923" & year==2005

replace regioncode="610924" if regioncode=="610924" & year==2005

replace regioncode="610925" if regioncode=="610925" & year==2005

replace regioncode="610926" if regioncode=="610926" & year==2005

replace regioncode="610927" if regioncode=="610927" & year==2005

replace regioncode="610928" if regioncode=="610928" & year==2005

replace regioncode="610929" if regioncode=="610929" & year==2005

replace regioncode="611002" if regioncode=="611002" & year==2005

replace regioncode="611021" if regioncode=="611021" & year==2005

replace regioncode="611022" if regioncode=="611022" & year==2005

replace regioncode="611023" if regioncode=="611023" & year==2005

replace regioncode="611024" if regioncode=="611024" & year==2005

replace regioncode="611025" if regioncode=="611025" & year==2005

replace regioncode="611026" if regioncode=="611026" & year==2005

replace regioncode="620102" if regioncode=="620102" & year==2005

replace regioncode="620103" if regioncode=="620103" & year==2005

replace regioncode="620104" if regioncode=="620104" & year==2005

replace regioncode="620105" if regioncode=="620105" & year==2005

replace regioncode="620111" if regioncode=="620111" & year==2005

replace regioncode="620121" if regioncode=="620121" & year==2005

replace regioncode="620122" if regioncode=="620122" & year==2005

replace regioncode="620123" if regioncode=="620123" & year==2005

replace regioncode="620201" if regioncode=="620201" & year==2005

replace regioncode="620302" if regioncode=="620302" & year==2005

replace regioncode="620321" if regioncode=="620321" & year==2005

replace regioncode="620402" if regioncode=="620402" & year==2005

replace regioncode="620403" if regioncode=="620403" & year==2005

replace regioncode="620421" if regioncode=="620421" & year==2005

replace regioncode="620422" if regioncode=="620422" & year==2005

replace regioncode="620423" if regioncode=="620423" & year==2005

replace regioncode="620502" if regioncode=="620502" & year==2005

replace regioncode="620503" if regioncode=="620503" & year==2005

replace regioncode="620521" if regioncode=="620521" & year==2005

replace regioncode="620522" if regioncode=="620522" & year==2005

replace regioncode="620523" if regioncode=="620523" & year==2005

replace regioncode="620524" if regioncode=="620524" & year==2005

replace regioncode="620525" if regioncode=="620525" & year==2005

replace regioncode="622301" if regioncode=="620602" & year==2005

replace regioncode="622322" if regioncode=="620621" & year==2005

replace regioncode="622323" if regioncode=="620622" & year==2005

replace regioncode="622326" if regioncode=="620623" & year==2005

replace regioncode="622201" if regioncode=="620702" & year==2005

replace regioncode="622222" if regioncode=="620721" & year==2005

replace regioncode="622223" if regioncode=="620722" & year==2005

replace regioncode="622224" if regioncode=="620723" & year==2005

replace regioncode="622225" if regioncode=="620724" & year==2005

replace regioncode="622226" if regioncode=="620725" & year==2005

replace regioncode="622701" if regioncode=="620802" & year==2005

replace regioncode="622722" if regioncode=="620821" & year==2005

replace regioncode="622723" if regioncode=="620822" & year==2005

replace regioncode="622724" if regioncode=="620823" & year==2005

replace regioncode="622725" if regioncode=="620824" & year==2005

replace regioncode="622726" if regioncode=="620825" & year==2005

replace regioncode="622727" if regioncode=="620826" & year==2005

replace regioncode="622102" if regioncode=="620902" & year==2005

replace regioncode="622123" if regioncode=="620921" & year==2005

replace regioncode="622126" if regioncode=="620922" & year==2005

replace regioncode="622124" if regioncode=="620923" & year==2005

replace regioncode="622125" if regioncode=="620924" & year==2005

replace regioncode="622101" if regioncode=="620981" & year==2005

replace regioncode="622103" if regioncode=="620982" & year==2005

replace regioncode="622801" if regioncode=="621002" & year==2005

replace regioncode="622821" if regioncode=="621021" & year==2005

replace regioncode="622822" if regioncode=="621022" & year==2005

replace regioncode="622823" if regioncode=="621023" & year==2005

replace regioncode="622824" if regioncode=="621024" & year==2005

replace regioncode="622825" if regioncode=="621025" & year==2005

replace regioncode="622826" if regioncode=="621026" & year==2005

replace regioncode="622827" if regioncode=="621027" & year==2005

replace regioncode="622421" if regioncode=="621102" & year==2005

replace regioncode="622424" if regioncode=="621121" & year==2005

replace regioncode="622425" if regioncode=="621122" & year==2005

replace regioncode="622426" if regioncode=="621123" & year==2005

replace regioncode="622427" if regioncode=="621124" & year==2005

replace regioncode="622428" if regioncode=="621125" & year==2005

replace regioncode="622429" if regioncode=="621126" & year==2005

replace regioncode="622621" if regioncode=="621202" & year==2005

replace regioncode="622624" if regioncode=="621221" & year==2005

replace regioncode="622626" if regioncode=="621222" & year==2005

replace regioncode="622623" if regioncode=="621223" & year==2005

replace regioncode="622625" if regioncode=="621224" & year==2005

replace regioncode="622627" if regioncode=="621225" & year==2005

replace regioncode="622628" if regioncode=="621226" & year==2005

replace regioncode="622630" if regioncode=="621227" & year==2005

replace regioncode="622629" if regioncode=="621228" & year==2005

replace regioncode="622901" if regioncode=="622901" & year==2005

replace regioncode="622921" if regioncode=="622921" & year==2005

replace regioncode="622922" if regioncode=="622922" & year==2005

replace regioncode="622923" if regioncode=="622923" & year==2005

replace regioncode="622924" if regioncode=="622924" & year==2005

replace regioncode="622925" if regioncode=="622925" & year==2005

replace regioncode="622926" if regioncode=="622926" & year==2005

replace regioncode="622927" if regioncode=="622927" & year==2005

replace regioncode="623001" if regioncode=="623001" & year==2005

replace regioncode="623021" if regioncode=="623021" & year==2005

replace regioncode="623022" if regioncode=="623022" & year==2005

replace regioncode="623023" if regioncode=="623023" & year==2005

replace regioncode="623024" if regioncode=="623024" & year==2005

replace regioncode="623025" if regioncode=="623025" & year==2005

replace regioncode="623026" if regioncode=="623026" & year==2005

replace regioncode="623027" if regioncode=="623027" & year==2005

replace regioncode="630102" if regioncode=="630102" & year==2005

replace regioncode="630103" if regioncode=="630103" & year==2005

replace regioncode="630104" if regioncode=="630104" & year==2005

replace regioncode="630105" if regioncode=="630105" & year==2005

replace regioncode="630121" if regioncode=="630121" & year==2005

replace regioncode="630122" if regioncode=="630122" & year==2005

replace regioncode="630123" if regioncode=="630123" & year==2005

replace regioncode="632121" if regioncode=="632121" & year==2005

replace regioncode="632122" if regioncode=="632122" & year==2005

replace regioncode="632123" if regioncode=="632123" & year==2005

replace regioncode="632126" if regioncode=="632126" & year==2005

replace regioncode="632127" if regioncode=="632127" & year==2005

replace regioncode="632128" if regioncode=="632128" & year==2005

replace regioncode="632221" if regioncode=="632221" & year==2005

replace regioncode="632222" if regioncode=="632222" & year==2005

replace regioncode="632223" if regioncode=="632223" & year==2005

replace regioncode="632224" if regioncode=="632224" & year==2005

replace regioncode="632321" if regioncode=="632321" & year==2005

replace regioncode="632322" if regioncode=="632322" & year==2005

replace regioncode="632521" if regioncode=="632521" & year==2005

replace regioncode="632522" if regioncode=="632522" & year==2005

replace regioncode="632523" if regioncode=="632523" & year==2005

replace regioncode="632524" if regioncode=="632524" & year==2005

replace regioncode="632525" if regioncode=="632525" & year==2005

replace regioncode="632621" if regioncode=="632621" & year==2005

replace regioncode="632622" if regioncode=="632622" & year==2005

replace regioncode="632623" if regioncode=="632623" & year==2005

replace regioncode="632624" if regioncode=="632624" & year==2005

replace regioncode="632625" if regioncode=="632625" & year==2005

replace regioncode="632626" if regioncode=="632626" & year==2005

replace regioncode="632721" if regioncode=="632721" & year==2005

replace regioncode="632723" if regioncode=="632723" & year==2005

replace regioncode="632724" if regioncode=="632724" & year==2005

replace regioncode="632725" if regioncode=="632725" & year==2005

replace regioncode="632726" if regioncode=="632726" & year==2005

replace regioncode="632801" if regioncode=="632801" & year==2005

replace regioncode="632802" if regioncode=="632802" & year==2005

replace regioncode="632821" if regioncode=="632821" & year==2005

replace regioncode="632822" if regioncode=="632822" & year==2005

replace regioncode="640104" if regioncode=="640104" & year==2005

replace regioncode="640105" if regioncode=="640105" & year==2005

replace regioncode="640106" if regioncode=="640106" & year==2005

replace regioncode="640121" if regioncode=="640121" & year==2005

replace regioncode="640122" if regioncode=="640122" & year==2005

replace regioncode="640181" if regioncode=="640181" & year==2005

replace regioncode="640202" if regioncode=="640202" & year==2005

replace regioncode="640203" if regioncode=="640205" & year==2005

replace regioncode="640221" if regioncode=="640221" & year==2005

replace regioncode="640302" if regioncode=="640302" & year==2005

replace regioncode="640321" if regioncode=="640502" & year==2005

replace regioncode="640322" if regioncode=="640521" & year==2005

replace regioncode="640323" if regioncode=="640323" & year==2005

replace regioncode="640324" if regioncode=="640324" & year==2005

replace regioncode="640381" if regioncode=="640381" & year==2005

replace regioncode="640402" if regioncode=="640402" & year==2005

replace regioncode="640421" if regioncode=="640522" & year==2005

replace regioncode="640422" if regioncode=="640422" & year==2005

replace regioncode="640423" if regioncode=="640423" & year==2005

replace regioncode="640424" if regioncode=="640424" & year==2005

replace regioncode="640425" if regioncode=="640425" & year==2005

replace regioncode="650102" if regioncode=="650102" & year==2005

replace regioncode="650103" if regioncode=="650103" & year==2005

replace regioncode="650104" if regioncode=="650104" & year==2005

replace regioncode="650105" if regioncode=="650105" & year==2005

replace regioncode="650106" if regioncode=="650106" & year==2005

replace regioncode="650107" if regioncode=="650107" & year==2005

replace regioncode="650108" if regioncode=="650108" & year==2005

replace regioncode="650121" if regioncode=="650121" & year==2005

replace regioncode="650202" if regioncode=="650202" & year==2005

replace regioncode="650203" if regioncode=="650203" & year==2005

replace regioncode="650204" if regioncode=="650204" & year==2005

replace regioncode="650205" if regioncode=="650205" & year==2005

replace regioncode="652101" if regioncode=="652101" & year==2005

replace regioncode="652122" if regioncode=="652122" & year==2005

replace regioncode="652123" if regioncode=="652123" & year==2005

replace regioncode="652201" if regioncode=="652201" & year==2005

replace regioncode="652222" if regioncode=="652222" & year==2005

replace regioncode="652223" if regioncode=="652223" & year==2005

replace regioncode="652301" if regioncode=="652301" & year==2005

replace regioncode="652302" if regioncode=="652302" & year==2005

replace regioncode="652303" if regioncode=="652303" & year==2005

replace regioncode="652323" if regioncode=="652323" & year==2005

replace regioncode="652324" if regioncode=="652324" & year==2005

replace regioncode="652325" if regioncode=="652325" & year==2005

replace regioncode="652327" if regioncode=="652327" & year==2005

replace regioncode="652328" if regioncode=="652328" & year==2005

replace regioncode="652701" if regioncode=="652701" & year==2005

replace regioncode="652722" if regioncode=="652722" & year==2005

replace regioncode="652723" if regioncode=="652723" & year==2005

replace regioncode="652801" if regioncode=="652801" & year==2005

replace regioncode="652822" if regioncode=="652822" & year==2005

replace regioncode="652823" if regioncode=="652823" & year==2005

replace regioncode="652824" if regioncode=="652824" & year==2005

replace regioncode="652825" if regioncode=="652825" & year==2005

replace regioncode="652826" if regioncode=="652826" & year==2005

replace regioncode="652827" if regioncode=="652827" & year==2005

replace regioncode="652828" if regioncode=="652828" & year==2005

replace regioncode="652829" if regioncode=="652829" & year==2005

replace regioncode="652901" if regioncode=="652901" & year==2005

replace regioncode="652922" if regioncode=="652922" & year==2005

replace regioncode="652923" if regioncode=="652923" & year==2005

replace regioncode="652924" if regioncode=="652924" & year==2005

replace regioncode="652925" if regioncode=="652925" & year==2005

replace regioncode="652926" if regioncode=="652926" & year==2005

replace regioncode="652928" if regioncode=="652928" & year==2005

replace regioncode="652929" if regioncode=="652929" & year==2005

replace regioncode="653001" if regioncode=="653001" & year==2005

replace regioncode="653022" if regioncode=="653022" & year==2005

replace regioncode="653023" if regioncode=="653023" & year==2005

replace regioncode="653024" if regioncode=="653024" & year==2005

replace regioncode="653101" if regioncode=="653101" & year==2005

replace regioncode="653121" if regioncode=="653121" & year==2005

replace regioncode="653122" if regioncode=="653122" & year==2005

replace regioncode="653123" if regioncode=="653123" & year==2005

replace regioncode="653124" if regioncode=="653124" & year==2005

replace regioncode="653125" if regioncode=="653125" & year==2005

replace regioncode="653126" if regioncode=="653126" & year==2005

replace regioncode="653127" if regioncode=="653127" & year==2005

replace regioncode="653128" if regioncode=="653128" & year==2005

replace regioncode="653129" if regioncode=="653129" & year==2005

replace regioncode="653130" if regioncode=="653130" & year==2005

replace regioncode="653131" if regioncode=="653131" & year==2005

replace regioncode="653201" if regioncode=="653201" & year==2005

replace regioncode="653221" if regioncode=="653221" & year==2005

replace regioncode="653222" if regioncode=="653222" & year==2005

replace regioncode="653223" if regioncode=="653223" & year==2005

replace regioncode="653224" if regioncode=="653224" & year==2005

replace regioncode="653225" if regioncode=="653225" & year==2005

replace regioncode="653226" if regioncode=="653226" & year==2005

replace regioncode="653227" if regioncode=="653227" & year==2005

replace regioncode="654002" if regioncode=="654002" & year==2005

replace regioncode="654003" if regioncode=="654003" & year==2005

replace regioncode="654021" if regioncode=="654021" & year==2005

replace regioncode="654022" if regioncode=="654022" & year==2005

replace regioncode="654023" if regioncode=="654023" & year==2005

replace regioncode="654024" if regioncode=="654024" & year==2005

replace regioncode="654025" if regioncode=="654025" & year==2005

replace regioncode="654026" if regioncode=="654026" & year==2005

replace regioncode="654027" if regioncode=="654027" & year==2005

replace regioncode="654028" if regioncode=="654028" & year==2005

replace regioncode="654201" if regioncode=="654201" & year==2005

replace regioncode="654202" if regioncode=="654202" & year==2005

replace regioncode="654221" if regioncode=="654221" & year==2005

replace regioncode="654223" if regioncode=="654223" & year==2005

replace regioncode="654224" if regioncode=="654224" & year==2005

replace regioncode="654225" if regioncode=="654225" & year==2005

replace regioncode="654226" if regioncode=="654226" & year==2005

replace regioncode="654301" if regioncode=="654301" & year==2005

replace regioncode="654321" if regioncode=="654321" & year==2005

replace regioncode="654322" if regioncode=="654322" & year==2005

replace regioncode="654323" if regioncode=="654323" & year==2005

replace regioncode="654324" if regioncode=="654324" & year==2005

replace regioncode="654325" if regioncode=="654325" & year==2005

replace regioncode="654326" if regioncode=="654326" & year==2005

replace regioncode="659001" if regioncode=="659001" & year==2005

replace regioncode="110101" if regioncode=="110101" & year==2006

replace regioncode="110102" if regioncode=="110102" & year==2006

replace regioncode="110103" if regioncode=="110103" & year==2006

replace regioncode="110104" if regioncode=="110104" & year==2006

replace regioncode="110105" if regioncode=="110105" & year==2006

replace regioncode="110106" if regioncode=="110106" & year==2006

replace regioncode="110107" if regioncode=="110107" & year==2006

replace regioncode="110108" if regioncode=="110108" & year==2006

replace regioncode="110109" if regioncode=="110109" & year==2006

replace regioncode="110111" if regioncode=="110111" & year==2006

replace regioncode="110112" if regioncode=="110112" & year==2006

replace regioncode="110113" if regioncode=="110113" & year==2006

replace regioncode="110114" if regioncode=="110114" & year==2006

replace regioncode="110115" if regioncode=="110115" & year==2006

replace regioncode="110117" if regioncode=="110117" & year==2006

replace regioncode="110116" if regioncode=="110116" & year==2006

replace regioncode="110228" if regioncode=="110228" & year==2006

replace regioncode="110229" if regioncode=="110229" & year==2006

replace regioncode="120101" if regioncode=="120101" & year==2006

replace regioncode="120102" if regioncode=="120102" & year==2006

replace regioncode="120103" if regioncode=="120103" & year==2006

replace regioncode="120104" if regioncode=="120104" & year==2006

replace regioncode="120105" if regioncode=="120105" & year==2006

replace regioncode="120106" if regioncode=="120106" & year==2006

replace regioncode="120107" if regioncode=="120107" & year==2006

replace regioncode="120108" if regioncode=="120108" & year==2006

replace regioncode="120109" if regioncode=="120109" & year==2006

replace regioncode="120110" if regioncode=="120110" & year==2006

replace regioncode="120111" if regioncode=="120111" & year==2006

replace regioncode="120112" if regioncode=="120112" & year==2006

replace regioncode="120113" if regioncode=="120113" & year==2006

replace regioncode="120114" if regioncode=="120114" & year==2006

replace regioncode="120115" if regioncode=="120115" & year==2006

replace regioncode="120221" if regioncode=="120221" & year==2006

replace regioncode="120223" if regioncode=="120223" & year==2006

replace regioncode="120225" if regioncode=="120225" & year==2006

replace regioncode="130102" if regioncode=="130102" & year==2006

replace regioncode="130103" if regioncode=="130103" & year==2006

replace regioncode="130104" if regioncode=="130104" & year==2006

replace regioncode="130105" if regioncode=="130105" & year==2006

replace regioncode="130107" if regioncode=="130107" & year==2006

replace regioncode="130108" if regioncode=="130108" & year==2006

replace regioncode="130121" if regioncode=="130121" & year==2006

replace regioncode="130123" if regioncode=="130123" & year==2006

replace regioncode="130124" if regioncode=="130124" & year==2006

replace regioncode="130125" if regioncode=="130125" & year==2006

replace regioncode="130126" if regioncode=="130126" & year==2006

replace regioncode="130127" if regioncode=="130127" & year==2006

replace regioncode="130128" if regioncode=="130128" & year==2006

replace regioncode="130129" if regioncode=="130129" & year==2006

replace regioncode="130130" if regioncode=="130130" & year==2006

replace regioncode="130131" if regioncode=="130131" & year==2006

replace regioncode="130132" if regioncode=="130132" & year==2006

replace regioncode="130133" if regioncode=="130133" & year==2006

replace regioncode="130181" if regioncode=="130181" & year==2006

replace regioncode="130182" if regioncode=="130182" & year==2006

replace regioncode="130183" if regioncode=="130183" & year==2006

replace regioncode="130184" if regioncode=="130184" & year==2006

replace regioncode="130185" if regioncode=="130185" & year==2006

replace regioncode="130202" if regioncode=="130202" & year==2006

replace regioncode="130203" if regioncode=="130203" & year==2006

replace regioncode="130204" if regioncode=="130204" & year==2006

replace regioncode="130205" if regioncode=="130205" & year==2006

replace regioncode="130208" if regioncode=="130208" & year==2006

replace regioncode="130223" if regioncode=="130223" & year==2006

replace regioncode="130224" if regioncode=="130224" & year==2006

replace regioncode="130225" if regioncode=="130225" & year==2006

replace regioncode="130227" if regioncode=="130227" & year==2006

replace regioncode="130229" if regioncode=="130229" & year==2006

replace regioncode="130230" if regioncode=="130230" & year==2006

replace regioncode="130281" if regioncode=="130281" & year==2006

replace regioncode="130207" if regioncode=="130207" & year==2006

replace regioncode="130283" if regioncode=="130283" & year==2006

replace regioncode="130302" if regioncode=="130302" & year==2006

replace regioncode="130303" if regioncode=="130303" & year==2006

replace regioncode="130304" if regioncode=="130304" & year==2006

replace regioncode="130321" if regioncode=="130321" & year==2006

replace regioncode="130322" if regioncode=="130322" & year==2006

replace regioncode="130323" if regioncode=="130323" & year==2006

replace regioncode="130324" if regioncode=="130324" & year==2006

replace regioncode="130402" if regioncode=="130402" & year==2006

replace regioncode="130403" if regioncode=="130403" & year==2006

replace regioncode="130404" if regioncode=="130404" & year==2006

replace regioncode="130406" if regioncode=="130406" & year==2006

replace regioncode="130421" if regioncode=="130421" & year==2006

replace regioncode="130423" if regioncode=="130423" & year==2006

replace regioncode="130424" if regioncode=="130424" & year==2006

replace regioncode="130425" if regioncode=="130425" & year==2006

replace regioncode="130426" if regioncode=="130426" & year==2006

replace regioncode="130427" if regioncode=="130427" & year==2006

replace regioncode="130428" if regioncode=="130428" & year==2006

replace regioncode="130429" if regioncode=="130429" & year==2006

replace regioncode="130430" if regioncode=="130430" & year==2006

replace regioncode="130431" if regioncode=="130431" & year==2006

replace regioncode="130432" if regioncode=="130432" & year==2006

replace regioncode="130433" if regioncode=="130433" & year==2006

replace regioncode="130434" if regioncode=="130434" & year==2006

replace regioncode="130435" if regioncode=="130435" & year==2006

replace regioncode="130481" if regioncode=="130481" & year==2006

replace regioncode="130502" if regioncode=="130502" & year==2006

replace regioncode="130503" if regioncode=="130503" & year==2006

replace regioncode="130521" if regioncode=="130521" & year==2006

replace regioncode="130522" if regioncode=="130522" & year==2006

replace regioncode="130523" if regioncode=="130523" & year==2006

replace regioncode="130524" if regioncode=="130524" & year==2006

replace regioncode="130525" if regioncode=="130525" & year==2006

replace regioncode="130526" if regioncode=="130526" & year==2006

replace regioncode="130527" if regioncode=="130527" & year==2006

replace regioncode="130528" if regioncode=="130528" & year==2006

replace regioncode="130529" if regioncode=="130529" & year==2006

replace regioncode="130530" if regioncode=="130530" & year==2006

replace regioncode="130531" if regioncode=="130531" & year==2006

replace regioncode="130532" if regioncode=="130532" & year==2006

replace regioncode="130533" if regioncode=="130533" & year==2006

replace regioncode="130534" if regioncode=="130534" & year==2006

replace regioncode="130535" if regioncode=="130535" & year==2006

replace regioncode="130581" if regioncode=="130581" & year==2006

replace regioncode="130582" if regioncode=="130582" & year==2006

replace regioncode="130602" if regioncode=="130602" & year==2006

replace regioncode="130603" if regioncode=="130603" & year==2006

replace regioncode="130604" if regioncode=="130604" & year==2006

replace regioncode="130621" if regioncode=="130621" & year==2006

replace regioncode="130622" if regioncode=="130622" & year==2006

replace regioncode="130623" if regioncode=="130623" & year==2006

replace regioncode="130624" if regioncode=="130624" & year==2006

replace regioncode="130625" if regioncode=="130625" & year==2006

replace regioncode="130626" if regioncode=="130626" & year==2006

replace regioncode="130627" if regioncode=="130627" & year==2006

replace regioncode="130628" if regioncode=="130628" & year==2006

replace regioncode="130629" if regioncode=="130629" & year==2006

replace regioncode="130630" if regioncode=="130630" & year==2006

replace regioncode="130631" if regioncode=="130631" & year==2006

replace regioncode="130632" if regioncode=="130632" & year==2006

replace regioncode="130633" if regioncode=="130633" & year==2006

replace regioncode="130634" if regioncode=="130634" & year==2006

replace regioncode="130635" if regioncode=="130635" & year==2006

replace regioncode="130636" if regioncode=="130636" & year==2006

replace regioncode="130637" if regioncode=="130637" & year==2006

replace regioncode="130638" if regioncode=="130638" & year==2006

replace regioncode="130681" if regioncode=="130681" & year==2006

replace regioncode="130682" if regioncode=="130682" & year==2006

replace regioncode="130683" if regioncode=="130683" & year==2006

replace regioncode="130684" if regioncode=="130684" & year==2006

replace regioncode="130702" if regioncode=="130702" & year==2006

replace regioncode="130703" if regioncode=="130703" & year==2006

replace regioncode="130705" if regioncode=="130705" & year==2006

replace regioncode="130706" if regioncode=="130706" & year==2006

replace regioncode="130721" if regioncode=="130721" & year==2006

replace regioncode="130722" if regioncode=="130722" & year==2006

replace regioncode="130723" if regioncode=="130723" & year==2006

replace regioncode="130724" if regioncode=="130724" & year==2006

replace regioncode="130725" if regioncode=="130725" & year==2006

replace regioncode="130726" if regioncode=="130726" & year==2006

replace regioncode="130727" if regioncode=="130727" & year==2006

replace regioncode="130728" if regioncode=="130728" & year==2006

replace regioncode="130729" if regioncode=="130729" & year==2006

replace regioncode="130730" if regioncode=="130730" & year==2006

replace regioncode="130731" if regioncode=="130731" & year==2006

replace regioncode="130732" if regioncode=="130732" & year==2006

replace regioncode="130733" if regioncode=="130733" & year==2006

replace regioncode="130802" if regioncode=="130802" & year==2006

replace regioncode="130803" if regioncode=="130803" & year==2006

replace regioncode="130804" if regioncode=="130804" & year==2006

replace regioncode="130821" if regioncode=="130821" & year==2006

replace regioncode="130822" if regioncode=="130822" & year==2006

replace regioncode="130823" if regioncode=="130823" & year==2006

replace regioncode="130824" if regioncode=="130824" & year==2006

replace regioncode="130825" if regioncode=="130825" & year==2006

replace regioncode="130826" if regioncode=="130826" & year==2006

replace regioncode="130827" if regioncode=="130827" & year==2006

replace regioncode="130828" if regioncode=="130828" & year==2006

replace regioncode="130902" if regioncode=="130902" & year==2006

replace regioncode="130903" if regioncode=="130903" & year==2006

replace regioncode="130921" if regioncode=="130921" & year==2006

replace regioncode="130922" if regioncode=="130922" & year==2006

replace regioncode="130923" if regioncode=="130923" & year==2006

replace regioncode="130924" if regioncode=="130924" & year==2006

replace regioncode="130925" if regioncode=="130925" & year==2006

replace regioncode="130926" if regioncode=="130926" & year==2006

replace regioncode="130927" if regioncode=="130927" & year==2006

replace regioncode="130928" if regioncode=="130928" & year==2006

replace regioncode="130929" if regioncode=="130929" & year==2006

replace regioncode="130930" if regioncode=="130930" & year==2006

replace regioncode="130981" if regioncode=="130981" & year==2006

replace regioncode="130982" if regioncode=="130982" & year==2006

replace regioncode="130983" if regioncode=="130983" & year==2006

replace regioncode="130984" if regioncode=="130984" & year==2006

replace regioncode="131002" if regioncode=="131002" & year==2006

replace regioncode="131003" if regioncode=="131003" & year==2006

replace regioncode="131022" if regioncode=="131022" & year==2006

replace regioncode="131023" if regioncode=="131023" & year==2006

replace regioncode="131024" if regioncode=="131024" & year==2006

replace regioncode="131025" if regioncode=="131025" & year==2006

replace regioncode="131026" if regioncode=="131026" & year==2006

replace regioncode="131028" if regioncode=="131028" & year==2006

replace regioncode="131081" if regioncode=="131081" & year==2006

replace regioncode="131082" if regioncode=="131082" & year==2006

replace regioncode="131102" if regioncode=="131102" & year==2006

replace regioncode="131121" if regioncode=="131121" & year==2006

replace regioncode="131122" if regioncode=="131122" & year==2006

replace regioncode="131123" if regioncode=="131123" & year==2006

replace regioncode="131124" if regioncode=="131124" & year==2006

replace regioncode="131125" if regioncode=="131125" & year==2006

replace regioncode="131126" if regioncode=="131126" & year==2006

replace regioncode="131127" if regioncode=="131127" & year==2006

replace regioncode="131128" if regioncode=="131128" & year==2006

replace regioncode="131181" if regioncode=="131181" & year==2006

replace regioncode="131182" if regioncode=="131182" & year==2006

replace regioncode="140105" if regioncode=="140105" & year==2006

replace regioncode="140106" if regioncode=="140106" & year==2006

replace regioncode="140107" if regioncode=="140107" & year==2006

replace regioncode="140108" if regioncode=="140108" & year==2006

replace regioncode="140109" if regioncode=="140109" & year==2006

replace regioncode="140110" if regioncode=="140110" & year==2006

replace regioncode="140121" if regioncode=="140121" & year==2006

replace regioncode="140122" if regioncode=="140122" & year==2006

replace regioncode="140123" if regioncode=="140123" & year==2006

replace regioncode="140181" if regioncode=="140181" & year==2006

replace regioncode="140202" if regioncode=="140202" & year==2006

replace regioncode="140203" if regioncode=="140203" & year==2006

replace regioncode="140211" if regioncode=="140211" & year==2006

replace regioncode="140212" if regioncode=="140212" & year==2006

replace regioncode="140221" if regioncode=="140221" & year==2006

replace regioncode="140222" if regioncode=="140222" & year==2006

replace regioncode="140223" if regioncode=="140223" & year==2006

replace regioncode="140224" if regioncode=="140224" & year==2006

replace regioncode="140225" if regioncode=="140225" & year==2006

replace regioncode="140226" if regioncode=="140226" & year==2006

replace regioncode="140227" if regioncode=="140227" & year==2006

replace regioncode="140302" if regioncode=="140302" & year==2006

replace regioncode="140303" if regioncode=="140303" & year==2006

replace regioncode="140311" if regioncode=="140311" & year==2006

replace regioncode="140321" if regioncode=="140321" & year==2006

replace regioncode="140322" if regioncode=="140322" & year==2006

replace regioncode="140402" if regioncode=="140402" & year==2006

replace regioncode="140411" if regioncode=="140411" & year==2006

replace regioncode="140421" if regioncode=="140421" & year==2006

replace regioncode="140423" if regioncode=="140423" & year==2006

replace regioncode="140424" if regioncode=="140424" & year==2006

replace regioncode="140425" if regioncode=="140425" & year==2006

replace regioncode="140426" if regioncode=="140426" & year==2006

replace regioncode="140427" if regioncode=="140427" & year==2006

replace regioncode="140428" if regioncode=="140428" & year==2006

replace regioncode="140429" if regioncode=="140429" & year==2006

replace regioncode="140430" if regioncode=="140430" & year==2006

replace regioncode="140431" if regioncode=="140431" & year==2006

replace regioncode="140481" if regioncode=="140481" & year==2006

replace regioncode="140502" if regioncode=="140502" & year==2006

replace regioncode="140521" if regioncode=="140521" & year==2006

replace regioncode="140522" if regioncode=="140522" & year==2006

replace regioncode="140524" if regioncode=="140524" & year==2006

replace regioncode="140525" if regioncode=="140525" & year==2006

replace regioncode="140581" if regioncode=="140581" & year==2006

replace regioncode="140602" if regioncode=="140602" & year==2006

replace regioncode="140603" if regioncode=="140603" & year==2006

replace regioncode="140621" if regioncode=="140621" & year==2006

replace regioncode="140622" if regioncode=="140622" & year==2006

replace regioncode="140623" if regioncode=="140623" & year==2006

replace regioncode="140624" if regioncode=="140624" & year==2006

replace regioncode="140902" if regioncode=="140902" & year==2006

replace regioncode="140981" if regioncode=="140981" & year==2006

replace regioncode="140921" if regioncode=="140921" & year==2006

replace regioncode="140922" if regioncode=="140922" & year==2006

replace regioncode="140923" if regioncode=="140923" & year==2006

replace regioncode="140924" if regioncode=="140924" & year==2006

replace regioncode="140925" if regioncode=="140925" & year==2006

replace regioncode="140926" if regioncode=="140926" & year==2006

replace regioncode="140927" if regioncode=="140927" & year==2006

replace regioncode="140928" if regioncode=="140928" & year==2006

replace regioncode="140929" if regioncode=="140929" & year==2006

replace regioncode="140930" if regioncode=="140930" & year==2006

replace regioncode="140931" if regioncode=="140931" & year==2006

replace regioncode="140932" if regioncode=="140932" & year==2006

replace regioncode="141181" if regioncode=="141181" & year==2006

replace regioncode="141102" if regioncode=="141102" & year==2006

replace regioncode="141182" if regioncode=="141182" & year==2006

replace regioncode="141121" if regioncode=="141121" & year==2006

replace regioncode="141122" if regioncode=="141122" & year==2006

replace regioncode="141123" if regioncode=="141123" & year==2006

replace regioncode="141124" if regioncode=="141124" & year==2006

replace regioncode="141125" if regioncode=="141125" & year==2006

replace regioncode="141126" if regioncode=="141126" & year==2006

replace regioncode="141127" if regioncode=="141127" & year==2006

replace regioncode="141128" if regioncode=="141128" & year==2006

replace regioncode="141129" if regioncode=="141129" & year==2006

replace regioncode="141130" if regioncode=="141130" & year==2006

replace regioncode="140702" if regioncode=="140702" & year==2006

replace regioncode="140781" if regioncode=="140781" & year==2006

replace regioncode="140721" if regioncode=="140721" & year==2006

replace regioncode="140722" if regioncode=="140722" & year==2006

replace regioncode="140723" if regioncode=="140723" & year==2006

replace regioncode="140724" if regioncode=="140724" & year==2006

replace regioncode="140725" if regioncode=="140725" & year==2006

replace regioncode="140726" if regioncode=="140726" & year==2006

replace regioncode="140727" if regioncode=="140727" & year==2006

replace regioncode="140728" if regioncode=="140728" & year==2006

replace regioncode="140729" if regioncode=="140729" & year==2006

replace regioncode="141002" if regioncode=="141002" & year==2006

replace regioncode="141081" if regioncode=="141081" & year==2006

replace regioncode="141082" if regioncode=="141082" & year==2006

replace regioncode="141021" if regioncode=="141021" & year==2006

replace regioncode="141022" if regioncode=="141022" & year==2006

replace regioncode="141023" if regioncode=="141023" & year==2006

replace regioncode="141024" if regioncode=="141024" & year==2006

replace regioncode="141025" if regioncode=="141025" & year==2006

replace regioncode="141026" if regioncode=="141026" & year==2006

replace regioncode="141027" if regioncode=="141027" & year==2006

replace regioncode="141028" if regioncode=="141028" & year==2006

replace regioncode="141029" if regioncode=="141029" & year==2006

replace regioncode="141033" if regioncode=="141033" & year==2006

replace regioncode="141030" if regioncode=="141030" & year==2006

replace regioncode="141032" if regioncode=="141032" & year==2006

replace regioncode="141031" if regioncode=="141031" & year==2006

replace regioncode="141034" if regioncode=="141034" & year==2006

replace regioncode="140802" if regioncode=="140802" & year==2006

replace regioncode="140881" if regioncode=="140881" & year==2006

replace regioncode="140882" if regioncode=="140882" & year==2006

replace regioncode="140830" if regioncode=="140830" & year==2006

replace regioncode="140821" if regioncode=="140821" & year==2006

replace regioncode="140822" if regioncode=="140822" & year==2006

replace regioncode="140825" if regioncode=="140825" & year==2006

replace regioncode="140824" if regioncode=="140824" & year==2006

replace regioncode="140823" if regioncode=="140823" & year==2006

replace regioncode="140828" if regioncode=="140828" & year==2006

replace regioncode="140826" if regioncode=="140826" & year==2006

replace regioncode="140829" if regioncode=="140829" & year==2006

replace regioncode="140827" if regioncode=="140827" & year==2006

replace regioncode="150102" if regioncode=="150102" & year==2006

replace regioncode="150103" if regioncode=="150103" & year==2006

replace regioncode="150104" if regioncode=="150104" & year==2006

replace regioncode="150105" if regioncode=="150105" & year==2006

replace regioncode="150121" if regioncode=="150121" & year==2006

replace regioncode="150122" if regioncode=="150122" & year==2006

replace regioncode="150123" if regioncode=="150123" & year==2006

replace regioncode="150124" if regioncode=="150124" & year==2006

replace regioncode="150125" if regioncode=="150125" & year==2006

replace regioncode="150202" if regioncode=="150202" & year==2006

replace regioncode="150203" if regioncode=="150203" & year==2006

replace regioncode="150204" if regioncode=="150204" & year==2006

replace regioncode="150205" if regioncode=="150205" & year==2006

replace regioncode="150206" if regioncode=="150206" & year==2006

replace regioncode="150207" if regioncode=="150207" & year==2006

replace regioncode="150221" if regioncode=="150221" & year==2006

replace regioncode="150222" if regioncode=="150222" & year==2006

replace regioncode="150223" if regioncode=="150223" & year==2006

replace regioncode="150302" if regioncode=="150302" & year==2006

replace regioncode="150303" if regioncode=="150303" & year==2006

replace regioncode="150304" if regioncode=="150304" & year==2006

replace regioncode="150402" if regioncode=="150402" & year==2006

replace regioncode="150403" if regioncode=="150403" & year==2006

replace regioncode="150404" if regioncode=="150404" & year==2006

replace regioncode="150421" if regioncode=="150421" & year==2006

replace regioncode="150422" if regioncode=="150422" & year==2006

replace regioncode="150423" if regioncode=="150423" & year==2006

replace regioncode="150424" if regioncode=="150424" & year==2006

replace regioncode="150425" if regioncode=="150425" & year==2006

replace regioncode="150426" if regioncode=="150426" & year==2006

replace regioncode="150428" if regioncode=="150428" & year==2006

replace regioncode="150429" if regioncode=="150429" & year==2006

replace regioncode="150430" if regioncode=="150430" & year==2006

replace regioncode="150702" if regioncode=="150702" & year==2006

replace regioncode="150781" if regioncode=="150781" & year==2006

replace regioncode="150783" if regioncode=="150783" & year==2006

replace regioncode="150782" if regioncode=="150782" & year==2006

replace regioncode="150785" if regioncode=="150785" & year==2006

replace regioncode="150784" if regioncode=="150784" & year==2006

replace regioncode="150721" if regioncode=="150721" & year==2006

replace regioncode="150722" if regioncode=="150722" & year==2006

replace regioncode="150723" if regioncode=="150723" & year==2006

replace regioncode="150724" if regioncode=="150724" & year==2006

replace regioncode="150727" if regioncode=="150727" & year==2006

replace regioncode="150726" if regioncode=="150726" & year==2006

replace regioncode="150725" if regioncode=="150725" & year==2006

replace regioncode="152201" if regioncode=="152201" & year==2006

replace regioncode="152202" if regioncode=="152202" & year==2006

replace regioncode="152221" if regioncode=="152221" & year==2006

replace regioncode="152222" if regioncode=="152222" & year==2006

replace regioncode="152223" if regioncode=="152223" & year==2006

replace regioncode="152224" if regioncode=="152224" & year==2006

replace regioncode="150502" if regioncode=="150502" & year==2006

replace regioncode="150581" if regioncode=="150581" & year==2006

replace regioncode="150521" if regioncode=="150521" & year==2006

replace regioncode="150522" if regioncode=="150522" & year==2006

replace regioncode="150523" if regioncode=="150523" & year==2006

replace regioncode="150524" if regioncode=="150524" & year==2006

replace regioncode="150525" if regioncode=="150525" & year==2006

replace regioncode="150526" if regioncode=="150526" & year==2006

replace regioncode="152501" if regioncode=="152501" & year==2006

replace regioncode="152502" if regioncode=="152502" & year==2006

replace regioncode="152522" if regioncode=="152522" & year==2006

replace regioncode="152523" if regioncode=="152523" & year==2006

replace regioncode="152524" if regioncode=="152524" & year==2006

replace regioncode="152525" if regioncode=="152525" & year==2006

replace regioncode="152526" if regioncode=="152526" & year==2006

replace regioncode="152527" if regioncode=="152527" & year==2006

replace regioncode="152528" if regioncode=="152528" & year==2006

replace regioncode="152529" if regioncode=="152529" & year==2006

replace regioncode="152530" if regioncode=="152530" & year==2006

replace regioncode="152531" if regioncode=="152531" & year==2006

replace regioncode="152601" if regioncode=="150902" & year==2006

replace regioncode="152602" if regioncode=="150981" & year==2006

replace regioncode="152624" if regioncode=="150921" & year==2006

replace regioncode="152625" if regioncode=="150922" & year==2006

replace regioncode="152626" if regioncode=="150923" & year==2006

replace regioncode="152627" if regioncode=="150924" & year==2006

replace regioncode="152629" if regioncode=="150925" & year==2006

replace regioncode="152630" if regioncode=="150926" & year==2006

replace regioncode="152631" if regioncode=="150927" & year==2006

replace regioncode="152632" if regioncode=="150928" & year==2006

replace regioncode="152634" if regioncode=="150929" & year==2006

replace regioncode="150602" if regioncode=="150602" & year==2006

replace regioncode="150621" if regioncode=="150621" & year==2006

replace regioncode="150622" if regioncode=="150622" & year==2006

replace regioncode="150623" if regioncode=="150623" & year==2006

replace regioncode="150624" if regioncode=="150624" & year==2006

replace regioncode="150625" if regioncode=="150625" & year==2006

replace regioncode="150626" if regioncode=="150626" & year==2006

replace regioncode="150627" if regioncode=="150627" & year==2006

replace regioncode="152801" if regioncode=="150802" & year==2006

replace regioncode="152822" if regioncode=="150821" & year==2006

replace regioncode="152823" if regioncode=="150822" & year==2006

replace regioncode="152824" if regioncode=="150823" & year==2006

replace regioncode="152825" if regioncode=="150824" & year==2006

replace regioncode="152826" if regioncode=="150825" & year==2006

replace regioncode="152827" if regioncode=="150826" & year==2006

replace regioncode="152921" if regioncode=="152921" & year==2006

replace regioncode="152922" if regioncode=="152922" & year==2006

replace regioncode="152923" if regioncode=="152923" & year==2006

replace regioncode="210102" if regioncode=="210102" & year==2006

replace regioncode="210103" if regioncode=="210103" & year==2006

replace regioncode="210104" if regioncode=="210104" & year==2006

replace regioncode="210105" if regioncode=="210105" & year==2006

replace regioncode="210106" if regioncode=="210106" & year==2006

replace regioncode="210111" if regioncode=="210111" & year==2006

replace regioncode="210112" if regioncode=="210112" & year==2006

replace regioncode="210113" if regioncode=="210113" & year==2006

replace regioncode="210114" if regioncode=="210114" & year==2006

replace regioncode="210122" if regioncode=="210122" & year==2006

replace regioncode="210123" if regioncode=="210123" & year==2006

replace regioncode="210124" if regioncode=="210124" & year==2006

replace regioncode="210181" if regioncode=="210181" & year==2006

replace regioncode="210202" if regioncode=="210202" & year==2006

replace regioncode="210203" if regioncode=="210203" & year==2006

replace regioncode="210204" if regioncode=="210204" & year==2006

replace regioncode="210211" if regioncode=="210211" & year==2006

replace regioncode="210212" if regioncode=="210212" & year==2006

replace regioncode="210213" if regioncode=="210213" & year==2006

replace regioncode="210224" if regioncode=="210224" & year==2006

replace regioncode="210281" if regioncode=="210281" & year==2006

replace regioncode="210282" if regioncode=="210282" & year==2006

replace regioncode="210283" if regioncode=="210283" & year==2006

replace regioncode="210302" if regioncode=="210302" & year==2006

replace regioncode="210303" if regioncode=="210303" & year==2006

replace regioncode="210304" if regioncode=="210304" & year==2006

replace regioncode="210311" if regioncode=="210311" & year==2006

replace regioncode="210321" if regioncode=="210321" & year==2006

replace regioncode="210323" if regioncode=="210323" & year==2006

replace regioncode="210381" if regioncode=="210381" & year==2006

replace regioncode="210402" if regioncode=="210402" & year==2006

replace regioncode="210403" if regioncode=="210403" & year==2006

replace regioncode="210404" if regioncode=="210404" & year==2006

replace regioncode="210411" if regioncode=="210411" & year==2006

replace regioncode="210421" if regioncode=="210421" & year==2006

replace regioncode="210422" if regioncode=="210422" & year==2006

replace regioncode="210423" if regioncode=="210423" & year==2006

replace regioncode="210502" if regioncode=="210502" & year==2006

replace regioncode="210503" if regioncode=="210503" & year==2006

replace regioncode="210504" if regioncode=="210504" & year==2006

replace regioncode="210505" if regioncode=="210505" & year==2006

replace regioncode="210521" if regioncode=="210521" & year==2006

replace regioncode="210522" if regioncode=="210522" & year==2006

replace regioncode="210602" if regioncode=="210602" & year==2006

replace regioncode="210603" if regioncode=="210603" & year==2006

replace regioncode="210604" if regioncode=="210604" & year==2006

replace regioncode="210624" if regioncode=="210624" & year==2006

replace regioncode="210681" if regioncode=="210681" & year==2006

replace regioncode="210682" if regioncode=="210682" & year==2006

replace regioncode="210702" if regioncode=="210702" & year==2006

replace regioncode="210703" if regioncode=="210703" & year==2006

replace regioncode="210711" if regioncode=="210711" & year==2006

replace regioncode="210726" if regioncode=="210726" & year==2006

replace regioncode="210727" if regioncode=="210727" & year==2006

replace regioncode="210781" if regioncode=="210781" & year==2006

replace regioncode="210782" if regioncode=="210782" & year==2006

replace regioncode="210802" if regioncode=="210802" & year==2006

replace regioncode="210803" if regioncode=="210803" & year==2006

replace regioncode="210804" if regioncode=="210804" & year==2006

replace regioncode="210811" if regioncode=="210811" & year==2006

replace regioncode="210881" if regioncode=="210881" & year==2006

replace regioncode="210882" if regioncode=="210882" & year==2006

replace regioncode="210902" if regioncode=="210902" & year==2006

replace regioncode="210903" if regioncode=="210903" & year==2006

replace regioncode="210904" if regioncode=="210904" & year==2006

replace regioncode="210905" if regioncode=="210905" & year==2006

replace regioncode="210911" if regioncode=="210911" & year==2006

replace regioncode="210921" if regioncode=="210921" & year==2006

replace regioncode="210922" if regioncode=="210922" & year==2006

replace regioncode="211002" if regioncode=="211002" & year==2006

replace regioncode="211003" if regioncode=="211003" & year==2006

replace regioncode="211004" if regioncode=="211004" & year==2006

replace regioncode="211005" if regioncode=="211005" & year==2006

replace regioncode="211011" if regioncode=="211011" & year==2006

replace regioncode="211021" if regioncode=="211021" & year==2006

replace regioncode="211081" if regioncode=="211081" & year==2006

replace regioncode="211102" if regioncode=="211102" & year==2006

replace regioncode="211103" if regioncode=="211103" & year==2006

replace regioncode="211121" if regioncode=="211121" & year==2006

replace regioncode="211122" if regioncode=="211122" & year==2006

replace regioncode="211202" if regioncode=="211202" & year==2006

replace regioncode="211204" if regioncode=="211204" & year==2006

replace regioncode="211221" if regioncode=="211221" & year==2006

replace regioncode="211223" if regioncode=="211223" & year==2006

replace regioncode="211224" if regioncode=="211224" & year==2006

replace regioncode="211281" if regioncode=="211281" & year==2006

replace regioncode="211282" if regioncode=="211282" & year==2006

replace regioncode="211302" if regioncode=="211302" & year==2006

replace regioncode="211303" if regioncode=="211303" & year==2006

replace regioncode="211321" if regioncode=="211321" & year==2006

replace regioncode="211322" if regioncode=="211322" & year==2006

replace regioncode="211324" if regioncode=="211324" & year==2006

replace regioncode="211381" if regioncode=="211381" & year==2006

replace regioncode="211382" if regioncode=="211382" & year==2006

replace regioncode="211402" if regioncode=="211402" & year==2006

replace regioncode="211403" if regioncode=="211403" & year==2006

replace regioncode="211404" if regioncode=="211404" & year==2006

replace regioncode="211421" if regioncode=="211421" & year==2006

replace regioncode="211422" if regioncode=="211422" & year==2006

replace regioncode="211481" if regioncode=="211481" & year==2006

replace regioncode="220102" if regioncode=="220102" & year==2006

replace regioncode="220103" if regioncode=="220103" & year==2006

replace regioncode="220104" if regioncode=="220104" & year==2006

replace regioncode="220105" if regioncode=="220105" & year==2006

replace regioncode="220106" if regioncode=="220106" & year==2006

replace regioncode="220112" if regioncode=="220112" & year==2006

replace regioncode="220122" if regioncode=="220122" & year==2006

replace regioncode="220181" if regioncode=="220181" & year==2006

replace regioncode="220182" if regioncode=="220182" & year==2006

replace regioncode="220183" if regioncode=="220183" & year==2006

replace regioncode="220202" if regioncode=="220202" & year==2006

replace regioncode="220203" if regioncode=="220203" & year==2006

replace regioncode="220204" if regioncode=="220204" & year==2006

replace regioncode="220211" if regioncode=="220211" & year==2006

replace regioncode="220221" if regioncode=="220221" & year==2006

replace regioncode="220281" if regioncode=="220281" & year==2006

replace regioncode="220282" if regioncode=="220282" & year==2006

replace regioncode="220283" if regioncode=="220283" & year==2006

replace regioncode="220284" if regioncode=="220284" & year==2006

replace regioncode="220302" if regioncode=="220302" & year==2006

replace regioncode="220303" if regioncode=="220303" & year==2006

replace regioncode="220322" if regioncode=="220322" & year==2006

replace regioncode="220323" if regioncode=="220323" & year==2006

replace regioncode="220381" if regioncode=="220381" & year==2006

replace regioncode="220382" if regioncode=="220382" & year==2006

replace regioncode="220402" if regioncode=="220402" & year==2006

replace regioncode="220403" if regioncode=="220403" & year==2006

replace regioncode="220421" if regioncode=="220421" & year==2006

replace regioncode="220422" if regioncode=="220422" & year==2006

replace regioncode="220502" if regioncode=="220502" & year==2006

replace regioncode="220503" if regioncode=="220503" & year==2006

replace regioncode="220521" if regioncode=="220521" & year==2006

replace regioncode="220523" if regioncode=="220523" & year==2006

replace regioncode="220524" if regioncode=="220524" & year==2006

replace regioncode="220581" if regioncode=="220581" & year==2006

replace regioncode="220582" if regioncode=="220582" & year==2006

replace regioncode="220602" if regioncode=="220602" & year==2006

replace regioncode="220621" if regioncode=="220621" & year==2006

replace regioncode="220622" if regioncode=="220622" & year==2006

replace regioncode="220623" if regioncode=="220623" & year==2006

replace regioncode="220625" if regioncode=="220625" & year==2006

replace regioncode="220681" if regioncode=="220681" & year==2006

replace regioncode="220702" if regioncode=="220702" & year==2006

replace regioncode="220721" if regioncode=="220721" & year==2006

replace regioncode="220722" if regioncode=="220722" & year==2006

replace regioncode="220723" if regioncode=="220723" & year==2006

replace regioncode="220724" if regioncode=="220724" & year==2006

replace regioncode="220802" if regioncode=="220802" & year==2006

replace regioncode="220821" if regioncode=="220821" & year==2006

replace regioncode="220822" if regioncode=="220822" & year==2006

replace regioncode="220881" if regioncode=="220881" & year==2006

replace regioncode="220882" if regioncode=="220882" & year==2006

replace regioncode="222401" if regioncode=="222401" & year==2006

replace regioncode="222402" if regioncode=="222402" & year==2006

replace regioncode="222403" if regioncode=="222403" & year==2006

replace regioncode="222404" if regioncode=="222404" & year==2006

replace regioncode="222405" if regioncode=="222405" & year==2006

replace regioncode="222406" if regioncode=="222406" & year==2006

replace regioncode="222424" if regioncode=="222424" & year==2006

replace regioncode="222426" if regioncode=="222426" & year==2006

replace regioncode="230102" if regioncode=="230102" & year==2006

replace regioncode="230103" if regioncode=="230103" & year==2006

replace regioncode="230104" if regioncode=="230104" & year==2006

replace regioncode="230105" if regioncode=="230105" & year==2006

replace regioncode="230107" if regioncode=="230107" & year==2006

replace regioncode="230108" if regioncode=="230108" & year==2006

replace regioncode="230121" if regioncode=="230111" & year==2006

replace regioncode="230123" if regioncode=="230123" & year==2006

replace regioncode="230124" if regioncode=="230124" & year==2006

replace regioncode="230125" if regioncode=="230125" & year==2006

replace regioncode="230126" if regioncode=="230126" & year==2006

replace regioncode="230127" if regioncode=="230127" & year==2006

replace regioncode="230128" if regioncode=="230128" & year==2006

replace regioncode="230129" if regioncode=="230129" & year==2006

replace regioncode="230181" if regioncode=="230181" & year==2006

replace regioncode="230182" if regioncode=="230182" & year==2006

replace regioncode="230183" if regioncode=="230183" & year==2006

replace regioncode="230184" if regioncode=="230184" & year==2006

replace regioncode="230202" if regioncode=="230202" & year==2006

replace regioncode="230203" if regioncode=="230203" & year==2006

replace regioncode="230204" if regioncode=="230204" & year==2006

replace regioncode="230205" if regioncode=="230205" & year==2006

replace regioncode="230206" if regioncode=="230206" & year==2006

replace regioncode="230207" if regioncode=="230207" & year==2006

replace regioncode="230208" if regioncode=="230208" & year==2006

replace regioncode="230221" if regioncode=="230221" & year==2006

replace regioncode="230223" if regioncode=="230223" & year==2006

replace regioncode="230224" if regioncode=="230224" & year==2006

replace regioncode="230225" if regioncode=="230225" & year==2006

replace regioncode="230227" if regioncode=="230227" & year==2006

replace regioncode="230229" if regioncode=="230229" & year==2006

replace regioncode="230230" if regioncode=="230230" & year==2006

replace regioncode="230231" if regioncode=="230231" & year==2006

replace regioncode="230281" if regioncode=="230281" & year==2006

replace regioncode="230302" if regioncode=="230302" & year==2006

replace regioncode="230303" if regioncode=="230303" & year==2006

replace regioncode="230304" if regioncode=="230304" & year==2006

replace regioncode="230305" if regioncode=="230305" & year==2006

replace regioncode="230306" if regioncode=="230306" & year==2006

replace regioncode="230307" if regioncode=="230307" & year==2006

replace regioncode="230321" if regioncode=="230321" & year==2006

replace regioncode="230381" if regioncode=="230381" & year==2006

replace regioncode="230382" if regioncode=="230382" & year==2006

replace regioncode="230402" if regioncode=="230402" & year==2006

replace regioncode="230403" if regioncode=="230403" & year==2006

replace regioncode="230404" if regioncode=="230404" & year==2006

replace regioncode="230405" if regioncode=="230405" & year==2006

replace regioncode="230406" if regioncode=="230406" & year==2006

replace regioncode="230407" if regioncode=="230407" & year==2006

replace regioncode="230421" if regioncode=="230421" & year==2006

replace regioncode="230422" if regioncode=="230422" & year==2006

replace regioncode="230502" if regioncode=="230502" & year==2006

replace regioncode="230503" if regioncode=="230503" & year==2006

replace regioncode="230505" if regioncode=="230505" & year==2006

replace regioncode="230506" if regioncode=="230506" & year==2006

replace regioncode="230521" if regioncode=="230521" & year==2006

replace regioncode="230522" if regioncode=="230522" & year==2006

replace regioncode="230523" if regioncode=="230523" & year==2006

replace regioncode="230524" if regioncode=="230524" & year==2006

replace regioncode="230602" if regioncode=="230602" & year==2006

replace regioncode="230603" if regioncode=="230603" & year==2006

replace regioncode="230604" if regioncode=="230604" & year==2006

replace regioncode="230605" if regioncode=="230605" & year==2006

replace regioncode="230606" if regioncode=="230606" & year==2006

replace regioncode="230621" if regioncode=="230621" & year==2006

replace regioncode="230622" if regioncode=="230622" & year==2006

replace regioncode="230623" if regioncode=="230623" & year==2006

replace regioncode="230624" if regioncode=="230624" & year==2006

replace regioncode="230702" if regioncode=="230702" & year==2006

replace regioncode="230703" if regioncode=="230703" & year==2006

replace regioncode="230704" if regioncode=="230704" & year==2006

replace regioncode="230705" if regioncode=="230705" & year==2006

replace regioncode="230706" if regioncode=="230706" & year==2006

replace regioncode="230707" if regioncode=="230707" & year==2006

replace regioncode="230708" if regioncode=="230708" & year==2006

replace regioncode="230709" if regioncode=="230709" & year==2006

replace regioncode="230710" if regioncode=="230710" & year==2006

replace regioncode="230711" if regioncode=="230711" & year==2006

replace regioncode="230712" if regioncode=="230712" & year==2006

replace regioncode="230713" if regioncode=="230713" & year==2006

replace regioncode="230714" if regioncode=="230714" & year==2006

replace regioncode="230715" if regioncode=="230715" & year==2006

replace regioncode="230716" if regioncode=="230716" & year==2006

replace regioncode="230722" if regioncode=="230722" & year==2006

replace regioncode="230781" if regioncode=="230781" & year==2006

replace regioncode="230802" if regioncode=="230802" & year==2006

replace regioncode="230803" if regioncode=="230803" & year==2006

replace regioncode="230804" if regioncode=="230804" & year==2006

replace regioncode="230805" if regioncode=="230805" & year==2006

replace regioncode="230811" if regioncode=="230811" & year==2006

replace regioncode="230822" if regioncode=="230822" & year==2006

replace regioncode="230826" if regioncode=="230826" & year==2006

replace regioncode="230828" if regioncode=="230828" & year==2006

replace regioncode="230833" if regioncode=="230833" & year==2006

replace regioncode="230881" if regioncode=="230881" & year==2006

replace regioncode="230882" if regioncode=="230882" & year==2006

replace regioncode="230902" if regioncode=="230902" & year==2006

replace regioncode="230903" if regioncode=="230903" & year==2006

replace regioncode="230904" if regioncode=="230904" & year==2006

replace regioncode="230921" if regioncode=="230921" & year==2006

replace regioncode="231002" if regioncode=="231002" & year==2006

replace regioncode="231003" if regioncode=="231003" & year==2006

replace regioncode="231004" if regioncode=="231004" & year==2006

replace regioncode="231005" if regioncode=="231005" & year==2006

replace regioncode="231024" if regioncode=="231024" & year==2006

replace regioncode="231025" if regioncode=="231025" & year==2006

replace regioncode="231081" if regioncode=="231081" & year==2006

replace regioncode="231083" if regioncode=="231083" & year==2006

replace regioncode="231084" if regioncode=="231084" & year==2006

replace regioncode="231085" if regioncode=="231085" & year==2006

replace regioncode="231102" if regioncode=="231102" & year==2006

replace regioncode="231121" if regioncode=="231121" & year==2006

replace regioncode="231123" if regioncode=="231123" & year==2006

replace regioncode="231124" if regioncode=="231124" & year==2006

replace regioncode="231181" if regioncode=="231181" & year==2006

replace regioncode="231182" if regioncode=="231182" & year==2006

replace regioncode="231202" if regioncode=="231202" & year==2006

replace regioncode="231221" if regioncode=="231221" & year==2006

replace regioncode="231222" if regioncode=="231222" & year==2006

replace regioncode="231223" if regioncode=="231223" & year==2006

replace regioncode="231224" if regioncode=="231224" & year==2006

replace regioncode="231225" if regioncode=="231225" & year==2006

replace regioncode="231226" if regioncode=="231226" & year==2006

replace regioncode="231281" if regioncode=="231281" & year==2006

replace regioncode="231282" if regioncode=="231282" & year==2006

replace regioncode="231283" if regioncode=="231283" & year==2006

replace regioncode="232701" if regioncode=="232701" & year==2006

replace regioncode="232702" if regioncode=="232702" & year==2006

replace regioncode="232703" if regioncode=="232703" & year==2006

replace regioncode="232704" if regioncode=="232704" & year==2006

replace regioncode="232721" if regioncode=="232721" & year==2006

replace regioncode="232722" if regioncode=="232722" & year==2006

replace regioncode="232723" if regioncode=="232723" & year==2006

replace regioncode="310101" if regioncode=="310101" & year==2006

replace regioncode="310103" if regioncode=="310103" & year==2006

replace regioncode="310104" if regioncode=="310104" & year==2006

replace regioncode="310105" if regioncode=="310105" & year==2006

replace regioncode="310106" if regioncode=="310106" & year==2006

replace regioncode="310107" if regioncode=="310107" & year==2006

replace regioncode="310108" if regioncode=="310108" & year==2006

replace regioncode="310109" if regioncode=="310109" & year==2006

replace regioncode="310110" if regioncode=="310110" & year==2006

replace regioncode="310112" if regioncode=="310112" & year==2006

replace regioncode="310113" if regioncode=="310113" & year==2006

replace regioncode="310114" if regioncode=="310114" & year==2006

replace regioncode="310115" if regioncode=="310115" & year==2006

replace regioncode="310116" if regioncode=="310116" & year==2006

replace regioncode="310117" if regioncode=="310117" & year==2006

replace regioncode="310118" if regioncode=="310118" & year==2006

replace regioncode="310119" if regioncode=="310119" & year==2006

replace regioncode="310120" if regioncode=="310120" & year==2006

replace regioncode="310230" if regioncode=="310230" & year==2006

replace regioncode="320102" if regioncode=="320102" & year==2006

replace regioncode="320103" if regioncode=="320103" & year==2006

replace regioncode="320104" if regioncode=="320104" & year==2006

replace regioncode="320105" if regioncode=="320105" & year==2006

replace regioncode="320106" if regioncode=="320106" & year==2006

replace regioncode="320107" if regioncode=="320107" & year==2006

replace regioncode="320111" if regioncode=="320111" & year==2006

replace regioncode="320113" if regioncode=="320113" & year==2006

replace regioncode="320114" if regioncode=="320114" & year==2006

replace regioncode="320115" if regioncode=="320115" & year==2006

replace regioncode="320116" if regioncode=="320116" & year==2006

replace regioncode="320124" if regioncode=="320124" & year==2006

replace regioncode="320125" if regioncode=="320125" & year==2006

replace regioncode="320202" if regioncode=="320202" & year==2006

replace regioncode="320203" if regioncode=="320203" & year==2006

replace regioncode="320204" if regioncode=="320204" & year==2006

replace regioncode="320205" if regioncode=="320205" & year==2006

replace regioncode="320211" if regioncode=="320211" & year==2006

replace regioncode="320281" if regioncode=="320281" & year==2006

replace regioncode="320282" if regioncode=="320282" & year==2006

replace regioncode="320302" if regioncode=="320302" & year==2006

replace regioncode="320303" if regioncode=="320303" & year==2006

replace regioncode="320304" if regioncode=="320304" & year==2006

replace regioncode="320305" if regioncode=="320305" & year==2006

replace regioncode="320311" if regioncode=="320311" & year==2006

replace regioncode="320321" if regioncode=="320321" & year==2006

replace regioncode="320322" if regioncode=="320322" & year==2006

replace regioncode="320323" if regioncode=="320323" & year==2006

replace regioncode="320324" if regioncode=="320324" & year==2006

replace regioncode="320381" if regioncode=="320381" & year==2006

replace regioncode="320382" if regioncode=="320382" & year==2006

replace regioncode="320402" if regioncode=="320402" & year==2006

replace regioncode="320404" if regioncode=="320404" & year==2006

replace regioncode="320405" if regioncode=="320405" & year==2006

replace regioncode="320411" if regioncode=="320411" & year==2006

replace regioncode="320412" if regioncode=="320412" & year==2006

replace regioncode="320481" if regioncode=="320481" & year==2006

replace regioncode="320482" if regioncode=="320482" & year==2006

replace regioncode="320502" if regioncode=="320502" & year==2006

replace regioncode="320503" if regioncode=="320503" & year==2006

replace regioncode="320504" if regioncode=="320504" & year==2006

replace regioncode="320505" if regioncode=="320505" & year==2006

replace regioncode="320581" if regioncode=="320581" & year==2006

replace regioncode="320582" if regioncode=="320582" & year==2006

replace regioncode="320583" if regioncode=="320583" & year==2006

replace regioncode="320584" if regioncode=="320584" & year==2006

replace regioncode="320585" if regioncode=="320585" & year==2006

replace regioncode="320586" if regioncode=="320586" & year==2006

replace regioncode="320602" if regioncode=="320602" & year==2006

replace regioncode="320611" if regioncode=="320611" & year==2006

replace regioncode="320621" if regioncode=="320621" & year==2006

replace regioncode="320623" if regioncode=="320623" & year==2006

replace regioncode="320681" if regioncode=="320681" & year==2006

replace regioncode="320682" if regioncode=="320682" & year==2006

replace regioncode="320683" if regioncode=="320683" & year==2006

replace regioncode="320684" if regioncode=="320684" & year==2006

replace regioncode="320703" if regioncode=="320703" & year==2006

replace regioncode="320705" if regioncode=="320705" & year==2006

replace regioncode="320706" if regioncode=="320706" & year==2006

replace regioncode="320721" if regioncode=="320721" & year==2006

replace regioncode="320722" if regioncode=="320722" & year==2006

replace regioncode="320723" if regioncode=="320723" & year==2006

replace regioncode="320724" if regioncode=="320724" & year==2006

replace regioncode="320802" if regioncode=="320802" & year==2006

replace regioncode="320803" if regioncode=="320803" & year==2006

replace regioncode="320804" if regioncode=="320804" & year==2006

replace regioncode="320811" if regioncode=="320811" & year==2006

replace regioncode="320826" if regioncode=="320826" & year==2006

replace regioncode="320829" if regioncode=="320829" & year==2006

replace regioncode="320830" if regioncode=="320830" & year==2006

replace regioncode="320831" if regioncode=="320831" & year==2006

replace regioncode="320902" if regioncode=="320902" & year==2006

replace regioncode="320903" if regioncode=="320903" & year==2006

replace regioncode="320921" if regioncode=="320921" & year==2006

replace regioncode="320922" if regioncode=="320922" & year==2006

replace regioncode="320923" if regioncode=="320923" & year==2006

replace regioncode="320924" if regioncode=="320924" & year==2006

replace regioncode="320925" if regioncode=="320925" & year==2006

replace regioncode="320981" if regioncode=="320981" & year==2006

replace regioncode="320982" if regioncode=="320982" & year==2006

replace regioncode="321002" if regioncode=="321002" & year==2006

replace regioncode="321003" if regioncode=="321003" & year==2006

replace regioncode="321011" if regioncode=="321011" & year==2006

replace regioncode="321023" if regioncode=="321023" & year==2006

replace regioncode="321081" if regioncode=="321081" & year==2006

replace regioncode="321084" if regioncode=="321084" & year==2006

replace regioncode="321088" if regioncode=="321088" & year==2006

replace regioncode="321102" if regioncode=="321102" & year==2006

replace regioncode="321111" if regioncode=="321111" & year==2006

replace regioncode="321112" if regioncode=="321112" & year==2006

replace regioncode="321181" if regioncode=="321181" & year==2006

replace regioncode="321182" if regioncode=="321182" & year==2006

replace regioncode="321183" if regioncode=="321183" & year==2006

replace regioncode="321202" if regioncode=="321202" & year==2006

replace regioncode="321203" if regioncode=="321203" & year==2006

replace regioncode="321281" if regioncode=="321281" & year==2006

replace regioncode="321282" if regioncode=="321282" & year==2006

replace regioncode="321283" if regioncode=="321283" & year==2006

replace regioncode="321284" if regioncode=="321284" & year==2006

replace regioncode="321302" if regioncode=="321302" & year==2006

replace regioncode="321321" if regioncode=="321311" & year==2006

replace regioncode="321322" if regioncode=="321322" & year==2006

replace regioncode="321323" if regioncode=="321323" & year==2006

replace regioncode="321324" if regioncode=="321324" & year==2006

replace regioncode="330102" if regioncode=="330102" & year==2006

replace regioncode="330103" if regioncode=="330103" & year==2006

replace regioncode="330104" if regioncode=="330104" & year==2006

replace regioncode="330105" if regioncode=="330105" & year==2006

replace regioncode="330106" if regioncode=="330106" & year==2006

replace regioncode="330108" if regioncode=="330108" & year==2006

replace regioncode="330109" if regioncode=="330109" & year==2006

replace regioncode="330110" if regioncode=="330110" & year==2006

replace regioncode="330122" if regioncode=="330122" & year==2006

replace regioncode="330127" if regioncode=="330127" & year==2006

replace regioncode="330182" if regioncode=="330182" & year==2006

replace regioncode="330183" if regioncode=="330183" & year==2006

replace regioncode="330185" if regioncode=="330185" & year==2006

replace regioncode="330203" if regioncode=="330203" & year==2006

replace regioncode="330204" if regioncode=="330204" & year==2006

replace regioncode="330205" if regioncode=="330205" & year==2006

replace regioncode="330206" if regioncode=="330206" & year==2006

replace regioncode="330211" if regioncode=="330211" & year==2006

replace regioncode="330212" if regioncode=="330212" & year==2006

replace regioncode="330225" if regioncode=="330225" & year==2006

replace regioncode="330226" if regioncode=="330226" & year==2006

replace regioncode="330281" if regioncode=="330281" & year==2006

replace regioncode="330282" if regioncode=="330282" & year==2006

replace regioncode="330283" if regioncode=="330283" & year==2006

replace regioncode="330302" if regioncode=="330302" & year==2006

replace regioncode="330303" if regioncode=="330303" & year==2006

replace regioncode="330304" if regioncode=="330304" & year==2006

replace regioncode="330322" if regioncode=="330322" & year==2006

replace regioncode="330324" if regioncode=="330324" & year==2006

replace regioncode="330326" if regioncode=="330326" & year==2006

replace regioncode="330327" if regioncode=="330327" & year==2006

replace regioncode="330328" if regioncode=="330328" & year==2006

replace regioncode="330329" if regioncode=="330329" & year==2006

replace regioncode="330381" if regioncode=="330381" & year==2006

replace regioncode="330382" if regioncode=="330382" & year==2006

replace regioncode="330402" if regioncode=="330402" & year==2006

replace regioncode="330411" if regioncode=="330411" & year==2006

replace regioncode="330421" if regioncode=="330421" & year==2006

replace regioncode="330424" if regioncode=="330424" & year==2006

replace regioncode="330481" if regioncode=="330481" & year==2006

replace regioncode="330482" if regioncode=="330482" & year==2006

replace regioncode="330483" if regioncode=="330483" & year==2006

replace regioncode="330501" if regioncode=="330501" & year==2006

replace regioncode="330521" if regioncode=="330521" & year==2006

replace regioncode="330522" if regioncode=="330522" & year==2006

replace regioncode="330523" if regioncode=="330523" & year==2006

replace regioncode="330602" if regioncode=="330602" & year==2006

replace regioncode="330621" if regioncode=="330621" & year==2006

replace regioncode="330624" if regioncode=="330624" & year==2006

replace regioncode="330681" if regioncode=="330681" & year==2006

replace regioncode="330682" if regioncode=="330682" & year==2006

replace regioncode="330683" if regioncode=="330683" & year==2006

replace regioncode="330702" if regioncode=="330702" & year==2006

replace regioncode="330703" if regioncode=="330703" & year==2006

replace regioncode="330723" if regioncode=="330723" & year==2006

replace regioncode="330726" if regioncode=="330726" & year==2006

replace regioncode="330727" if regioncode=="330727" & year==2006

replace regioncode="330781" if regioncode=="330781" & year==2006

replace regioncode="330782" if regioncode=="330782" & year==2006

replace regioncode="330783" if regioncode=="330783" & year==2006

replace regioncode="330784" if regioncode=="330784" & year==2006

replace regioncode="330802" if regioncode=="330802" & year==2006

replace regioncode="330803" if regioncode=="330803" & year==2006

replace regioncode="330822" if regioncode=="330822" & year==2006

replace regioncode="330824" if regioncode=="330824" & year==2006

replace regioncode="330825" if regioncode=="330825" & year==2006

replace regioncode="330881" if regioncode=="330881" & year==2006

replace regioncode="330902" if regioncode=="330902" & year==2006

replace regioncode="330903" if regioncode=="330903" & year==2006

replace regioncode="330921" if regioncode=="330921" & year==2006

replace regioncode="330922" if regioncode=="330922" & year==2006

replace regioncode="331002" if regioncode=="331002" & year==2006

replace regioncode="331003" if regioncode=="331003" & year==2006

replace regioncode="331004" if regioncode=="331004" & year==2006

replace regioncode="331021" if regioncode=="331021" & year==2006

replace regioncode="331022" if regioncode=="331022" & year==2006

replace regioncode="331023" if regioncode=="331023" & year==2006

replace regioncode="331024" if regioncode=="331024" & year==2006

replace regioncode="331081" if regioncode=="331081" & year==2006

replace regioncode="331082" if regioncode=="331082" & year==2006

replace regioncode="331102" if regioncode=="331102" & year==2006

replace regioncode="331121" if regioncode=="331121" & year==2006

replace regioncode="331122" if regioncode=="331122" & year==2006

replace regioncode="331123" if regioncode=="331123" & year==2006

replace regioncode="331124" if regioncode=="331124" & year==2006

replace regioncode="331125" if regioncode=="331125" & year==2006

replace regioncode="331126" if regioncode=="331126" & year==2006

replace regioncode="331127" if regioncode=="331127" & year==2006

replace regioncode="331181" if regioncode=="331181" & year==2006

replace regioncode="340102" if regioncode=="340102" & year==2006

replace regioncode="340103" if regioncode=="340103" & year==2006

replace regioncode="340104" if regioncode=="340104" & year==2006

replace regioncode="340111" if regioncode=="340111" & year==2006

replace regioncode="340121" if regioncode=="340121" & year==2006

replace regioncode="340122" if regioncode=="340122" & year==2006

replace regioncode="340123" if regioncode=="340123" & year==2006

replace regioncode="340202" if regioncode=="340202" & year==2006

replace regioncode="340203" if regioncode=="340203" & year==2006

replace regioncode="340204" if regioncode=="340204" & year==2006

replace regioncode="340207" if regioncode=="340207" & year==2006

replace regioncode="340221" if regioncode=="340221" & year==2006

replace regioncode="340222" if regioncode=="340222" & year==2006

replace regioncode="340223" if regioncode=="340223" & year==2006

replace regioncode="340302" if regioncode=="340302" & year==2006

replace regioncode="340303" if regioncode=="340303" & year==2006

replace regioncode="340304" if regioncode=="340304" & year==2006

replace regioncode="340311" if regioncode=="340311" & year==2006

replace regioncode="340321" if regioncode=="340321" & year==2006

replace regioncode="340322" if regioncode=="340322" & year==2006

replace regioncode="340323" if regioncode=="340323" & year==2006

replace regioncode="340402" if regioncode=="340402" & year==2006

replace regioncode="340403" if regioncode=="340403" & year==2006

replace regioncode="340404" if regioncode=="340404" & year==2006

replace regioncode="340405" if regioncode=="340405" & year==2006

replace regioncode="340406" if regioncode=="340406" & year==2006

replace regioncode="340421" if regioncode=="340421" & year==2006

replace regioncode="340502" if regioncode=="340502" & year==2006

replace regioncode="340503" if regioncode=="340503" & year==2006

replace regioncode="340504" if regioncode=="340504" & year==2006

replace regioncode="340521" if regioncode=="340521" & year==2006

replace regioncode="340602" if regioncode=="340602" & year==2006

replace regioncode="340603" if regioncode=="340603" & year==2006

replace regioncode="340604" if regioncode=="340604" & year==2006

replace regioncode="340621" if regioncode=="340621" & year==2006

replace regioncode="340702" if regioncode=="340702" & year==2006

replace regioncode="340703" if regioncode=="340703" & year==2006

replace regioncode="340711" if regioncode=="340711" & year==2006

replace regioncode="340721" if regioncode=="340721" & year==2006

replace regioncode="340802" if regioncode=="340802" & year==2006

replace regioncode="340803" if regioncode=="340803" & year==2006

replace regioncode="340811" if regioncode=="340811" & year==2006

replace regioncode="340822" if regioncode=="340822" & year==2006

replace regioncode="340823" if regioncode=="340823" & year==2006

replace regioncode="340824" if regioncode=="340824" & year==2006

replace regioncode="340825" if regioncode=="340825" & year==2006

replace regioncode="340826" if regioncode=="340826" & year==2006

replace regioncode="340827" if regioncode=="340827" & year==2006

replace regioncode="340828" if regioncode=="340828" & year==2006

replace regioncode="340881" if regioncode=="340881" & year==2006

replace regioncode="341002" if regioncode=="341002" & year==2006

replace regioncode="341003" if regioncode=="341003" & year==2006

replace regioncode="341004" if regioncode=="341004" & year==2006

replace regioncode="341021" if regioncode=="341021" & year==2006

replace regioncode="341022" if regioncode=="341022" & year==2006

replace regioncode="341023" if regioncode=="341023" & year==2006

replace regioncode="341024" if regioncode=="341024" & year==2006

replace regioncode="341102" if regioncode=="341102" & year==2006

replace regioncode="341103" if regioncode=="341103" & year==2006

replace regioncode="341122" if regioncode=="341122" & year==2006

replace regioncode="341124" if regioncode=="341124" & year==2006

replace regioncode="341125" if regioncode=="341125" & year==2006

replace regioncode="341126" if regioncode=="341126" & year==2006

replace regioncode="341181" if regioncode=="341181" & year==2006

replace regioncode="341182" if regioncode=="341182" & year==2006

replace regioncode="341202" if regioncode=="341202" & year==2006

replace regioncode="341203" if regioncode=="341203" & year==2006

replace regioncode="341204" if regioncode=="341204" & year==2006

replace regioncode="341221" if regioncode=="341221" & year==2006

replace regioncode="341222" if regioncode=="341222" & year==2006

replace regioncode="341621" if regioncode=="341621" & year==2006

replace regioncode="341622" if regioncode=="341622" & year==2006

replace regioncode="341225" if regioncode=="341225" & year==2006

replace regioncode="341226" if regioncode=="341226" & year==2006

replace regioncode="341623" if regioncode=="341623" & year==2006

replace regioncode="341282" if regioncode=="341282" & year==2006

replace regioncode="341302" if regioncode=="341302" & year==2006

replace regioncode="341321" if regioncode=="341321" & year==2006

replace regioncode="341322" if regioncode=="341322" & year==2006

replace regioncode="341323" if regioncode=="341323" & year==2006

replace regioncode="341324" if regioncode=="341324" & year==2006

replace regioncode="341402" if regioncode=="341402" & year==2006

replace regioncode="341421" if regioncode=="341421" & year==2006

replace regioncode="341422" if regioncode=="341422" & year==2006

replace regioncode="341423" if regioncode=="341423" & year==2006

replace regioncode="341424" if regioncode=="341424" & year==2006

replace regioncode="341521" if regioncode=="341521" & year==2006

replace regioncode="341522" if regioncode=="341522" & year==2006

replace regioncode="341523" if regioncode=="341523" & year==2006

replace regioncode="341524" if regioncode=="341524" & year==2006

replace regioncode="341525" if regioncode=="341525" & year==2006

replace regioncode="341802" if regioncode=="341802" & year==2006

replace regioncode="341881" if regioncode=="341881" & year==2006

replace regioncode="341821" if regioncode=="341821" & year==2006

replace regioncode="341822" if regioncode=="341822" & year==2006

replace regioncode="341823" if regioncode=="341823" & year==2006

replace regioncode="341825" if regioncode=="341825" & year==2006

replace regioncode="341824" if regioncode=="341824" & year==2006

replace regioncode="341702" if regioncode=="341702" & year==2006

replace regioncode="341721" if regioncode=="341721" & year==2006

replace regioncode="341722" if regioncode=="341722" & year==2006

replace regioncode="341723" if regioncode=="341723" & year==2006

replace regioncode="341502" if regioncode=="341502" & year==2006

replace regioncode="341602" if regioncode=="341602" & year==2006

replace regioncode="350102" if regioncode=="350102" & year==2006

replace regioncode="350103" if regioncode=="350103" & year==2006

replace regioncode="350104" if regioncode=="350104" & year==2006

replace regioncode="350105" if regioncode=="350105" & year==2006

replace regioncode="350111" if regioncode=="350111" & year==2006

replace regioncode="350121" if regioncode=="350121" & year==2006

replace regioncode="350122" if regioncode=="350122" & year==2006

replace regioncode="350123" if regioncode=="350123" & year==2006

replace regioncode="350124" if regioncode=="350124" & year==2006

replace regioncode="350125" if regioncode=="350125" & year==2006

replace regioncode="350128" if regioncode=="350128" & year==2006

replace regioncode="350181" if regioncode=="350181" & year==2006

replace regioncode="350182" if regioncode=="350182" & year==2006

replace regioncode="350203" if regioncode=="350203" & year==2006

replace regioncode="350205" if regioncode=="350205" & year==2006

replace regioncode="350206" if regioncode=="350206" & year==2006

replace regioncode="350211" if regioncode=="350211" & year==2006

replace regioncode="350212" if regioncode=="350212" & year==2006

replace regioncode="350213" if regioncode=="350213" & year==2006

replace regioncode="350302" if regioncode=="350302" & year==2006

replace regioncode="350303" if regioncode=="350303" & year==2006

replace regioncode="350321" if regioncode=="350321" & year==2006

replace regioncode="350322" if regioncode=="350322" & year==2006

replace regioncode="350402" if regioncode=="350402" & year==2006

replace regioncode="350403" if regioncode=="350403" & year==2006

replace regioncode="350421" if regioncode=="350421" & year==2006

replace regioncode="350423" if regioncode=="350423" & year==2006

replace regioncode="350424" if regioncode=="350424" & year==2006

replace regioncode="350425" if regioncode=="350425" & year==2006

replace regioncode="350426" if regioncode=="350426" & year==2006

replace regioncode="350427" if regioncode=="350427" & year==2006

replace regioncode="350428" if regioncode=="350428" & year==2006

replace regioncode="350429" if regioncode=="350429" & year==2006

replace regioncode="350430" if regioncode=="350430" & year==2006

replace regioncode="350481" if regioncode=="350481" & year==2006

replace regioncode="350502" if regioncode=="350502" & year==2006

replace regioncode="350503" if regioncode=="350503" & year==2006

replace regioncode="350504" if regioncode=="350504" & year==2006

replace regioncode="350505" if regioncode=="350505" & year==2006

replace regioncode="350521" if regioncode=="350521" & year==2006

replace regioncode="350524" if regioncode=="350524" & year==2006

replace regioncode="350525" if regioncode=="350525" & year==2006

replace regioncode="350526" if regioncode=="350526" & year==2006

replace regioncode="350581" if regioncode=="350581" & year==2006

replace regioncode="350582" if regioncode=="350582" & year==2006

replace regioncode="350583" if regioncode=="350583" & year==2006

replace regioncode="350602" if regioncode=="350602" & year==2006

replace regioncode="350603" if regioncode=="350603" & year==2006

replace regioncode="350622" if regioncode=="350622" & year==2006

replace regioncode="350623" if regioncode=="350623" & year==2006

replace regioncode="350624" if regioncode=="350624" & year==2006

replace regioncode="350625" if regioncode=="350625" & year==2006

replace regioncode="350626" if regioncode=="350626" & year==2006

replace regioncode="350627" if regioncode=="350627" & year==2006

replace regioncode="350628" if regioncode=="350628" & year==2006

replace regioncode="350629" if regioncode=="350629" & year==2006

replace regioncode="350681" if regioncode=="350681" & year==2006

replace regioncode="350702" if regioncode=="350702" & year==2006

replace regioncode="350721" if regioncode=="350721" & year==2006

replace regioncode="350722" if regioncode=="350722" & year==2006

replace regioncode="350723" if regioncode=="350723" & year==2006

replace regioncode="350724" if regioncode=="350724" & year==2006

replace regioncode="350725" if regioncode=="350725" & year==2006

replace regioncode="350781" if regioncode=="350781" & year==2006

replace regioncode="350782" if regioncode=="350782" & year==2006

replace regioncode="350783" if regioncode=="350783" & year==2006

replace regioncode="350784" if regioncode=="350784" & year==2006

replace regioncode="350802" if regioncode=="350802" & year==2006

replace regioncode="350821" if regioncode=="350821" & year==2006

replace regioncode="350822" if regioncode=="350822" & year==2006

replace regioncode="350823" if regioncode=="350823" & year==2006

replace regioncode="350824" if regioncode=="350824" & year==2006

replace regioncode="350825" if regioncode=="350825" & year==2006

replace regioncode="350881" if regioncode=="350881" & year==2006

replace regioncode="350902" if regioncode=="350902" & year==2006

replace regioncode="350981" if regioncode=="350981" & year==2006

replace regioncode="350982" if regioncode=="350982" & year==2006

replace regioncode="350921" if regioncode=="350921" & year==2006

replace regioncode="350922" if regioncode=="350922" & year==2006

replace regioncode="350923" if regioncode=="350923" & year==2006

replace regioncode="350924" if regioncode=="350924" & year==2006

replace regioncode="350925" if regioncode=="350925" & year==2006

replace regioncode="350926" if regioncode=="350926" & year==2006

replace regioncode="360102" if regioncode=="360102" & year==2006

replace regioncode="360103" if regioncode=="360103" & year==2006

replace regioncode="360104" if regioncode=="360104" & year==2006

replace regioncode="360105" if regioncode=="360105" & year==2006

replace regioncode="360111" if regioncode=="360111" & year==2006

replace regioncode="360121" if regioncode=="360121" & year==2006

replace regioncode="360122" if regioncode=="360122" & year==2006

replace regioncode="360123" if regioncode=="360123" & year==2006

replace regioncode="360124" if regioncode=="360124" & year==2006

replace regioncode="360202" if regioncode=="360202" & year==2006

replace regioncode="360203" if regioncode=="360203" & year==2006

replace regioncode="360222" if regioncode=="360222" & year==2006

replace regioncode="360281" if regioncode=="360281" & year==2006

replace regioncode="360302" if regioncode=="360302" & year==2006

replace regioncode="360313" if regioncode=="360313" & year==2006

replace regioncode="360321" if regioncode=="360321" & year==2006

replace regioncode="360322" if regioncode=="360322" & year==2006

replace regioncode="360323" if regioncode=="360323" & year==2006

replace regioncode="360402" if regioncode=="360402" & year==2006

replace regioncode="360403" if regioncode=="360403" & year==2006

replace regioncode="360421" if regioncode=="360421" & year==2006

replace regioncode="360423" if regioncode=="360423" & year==2006

replace regioncode="360424" if regioncode=="360424" & year==2006

replace regioncode="360425" if regioncode=="360425" & year==2006

replace regioncode="360426" if regioncode=="360426" & year==2006

replace regioncode="360427" if regioncode=="360427" & year==2006

replace regioncode="360428" if regioncode=="360428" & year==2006

replace regioncode="360429" if regioncode=="360429" & year==2006

replace regioncode="360430" if regioncode=="360430" & year==2006

replace regioncode="360481" if regioncode=="360481" & year==2006

replace regioncode="360502" if regioncode=="360502" & year==2006

replace regioncode="360521" if regioncode=="360521" & year==2006

replace regioncode="360602" if regioncode=="360602" & year==2006

replace regioncode="360622" if regioncode=="360622" & year==2006

replace regioncode="360681" if regioncode=="360681" & year==2006

replace regioncode="360702" if regioncode=="360702" & year==2006

replace regioncode="360721" if regioncode=="360721" & year==2006

replace regioncode="360722" if regioncode=="360722" & year==2006

replace regioncode="360723" if regioncode=="360723" & year==2006

replace regioncode="360724" if regioncode=="360724" & year==2006

replace regioncode="360725" if regioncode=="360725" & year==2006

replace regioncode="360726" if regioncode=="360726" & year==2006

replace regioncode="360727" if regioncode=="360727" & year==2006

replace regioncode="360728" if regioncode=="360728" & year==2006

replace regioncode="360729" if regioncode=="360729" & year==2006

replace regioncode="360730" if regioncode=="360730" & year==2006

replace regioncode="360731" if regioncode=="360731" & year==2006

replace regioncode="360732" if regioncode=="360732" & year==2006

replace regioncode="360733" if regioncode=="360733" & year==2006

replace regioncode="360734" if regioncode=="360734" & year==2006

replace regioncode="360735" if regioncode=="360735" & year==2006

replace regioncode="360781" if regioncode=="360781" & year==2006

replace regioncode="360782" if regioncode=="360782" & year==2006

replace regioncode="360802" if regioncode=="360802" & year==2006

replace regioncode="360821" if regioncode=="360821" & year==2006

replace regioncode="360822" if regioncode=="360822" & year==2006

replace regioncode="360823" if regioncode=="360823" & year==2006

replace regioncode="360824" if regioncode=="360824" & year==2006

replace regioncode="360825" if regioncode=="360825" & year==2006

replace regioncode="360826" if regioncode=="360826" & year==2006

replace regioncode="360827" if regioncode=="360827" & year==2006

replace regioncode="360828" if regioncode=="360828" & year==2006

replace regioncode="360829" if regioncode=="360829" & year==2006

replace regioncode="360830" if regioncode=="360830" & year==2006

replace regioncode="360881" if regioncode=="360881" & year==2006

replace regioncode="360902" if regioncode=="360902" & year==2006

replace regioncode="360921" if regioncode=="360921" & year==2006

replace regioncode="360922" if regioncode=="360922" & year==2006

replace regioncode="360923" if regioncode=="360923" & year==2006

replace regioncode="360924" if regioncode=="360924" & year==2006

replace regioncode="360925" if regioncode=="360925" & year==2006

replace regioncode="360926" if regioncode=="360926" & year==2006

replace regioncode="360981" if regioncode=="360981" & year==2006

replace regioncode="360982" if regioncode=="360982" & year==2006

replace regioncode="360983" if regioncode=="360983" & year==2006

replace regioncode="361002" if regioncode=="361002" & year==2006

replace regioncode="361021" if regioncode=="361021" & year==2006

replace regioncode="361022" if regioncode=="361022" & year==2006

replace regioncode="361023" if regioncode=="361023" & year==2006

replace regioncode="361024" if regioncode=="361024" & year==2006

replace regioncode="361025" if regioncode=="361025" & year==2006

replace regioncode="361026" if regioncode=="361026" & year==2006

replace regioncode="361027" if regioncode=="361027" & year==2006

replace regioncode="361028" if regioncode=="361028" & year==2006

replace regioncode="361029" if regioncode=="361029" & year==2006

replace regioncode="361030" if regioncode=="361030" & year==2006

replace regioncode="361102" if regioncode=="361102" & year==2006

replace regioncode="361121" if regioncode=="361121" & year==2006

replace regioncode="361122" if regioncode=="361122" & year==2006

replace regioncode="361123" if regioncode=="361123" & year==2006

replace regioncode="361124" if regioncode=="361124" & year==2006

replace regioncode="361125" if regioncode=="361125" & year==2006

replace regioncode="361126" if regioncode=="361126" & year==2006

replace regioncode="361127" if regioncode=="361127" & year==2006

replace regioncode="361128" if regioncode=="361128" & year==2006

replace regioncode="361129" if regioncode=="361129" & year==2006

replace regioncode="361130" if regioncode=="361130" & year==2006

replace regioncode="361181" if regioncode=="361181" & year==2006

replace regioncode="370102" if regioncode=="370102" & year==2006

replace regioncode="370103" if regioncode=="370103" & year==2006

replace regioncode="370104" if regioncode=="370104" & year==2006

replace regioncode="370105" if regioncode=="370105" & year==2006

replace regioncode="370112" if regioncode=="370112" & year==2006

replace regioncode="370113" if regioncode=="370113" & year==2006

replace regioncode="370124" if regioncode=="370124" & year==2006

replace regioncode="370125" if regioncode=="370125" & year==2006

replace regioncode="370126" if regioncode=="370126" & year==2006

replace regioncode="370181" if regioncode=="370181" & year==2006

replace regioncode="370202" if regioncode=="370202" & year==2006

replace regioncode="370203" if regioncode=="370203" & year==2006

replace regioncode="370205" if regioncode=="370205" & year==2006

replace regioncode="370211" if regioncode=="370211" & year==2006

replace regioncode="370212" if regioncode=="370212" & year==2006

replace regioncode="370213" if regioncode=="370213" & year==2006

replace regioncode="370214" if regioncode=="370214" & year==2006

replace regioncode="370281" if regioncode=="370281" & year==2006

replace regioncode="370282" if regioncode=="370282" & year==2006

replace regioncode="370283" if regioncode=="370283" & year==2006

replace regioncode="370284" if regioncode=="370284" & year==2006

replace regioncode="370285" if regioncode=="370285" & year==2006

replace regioncode="370302" if regioncode=="370302" & year==2006

replace regioncode="370303" if regioncode=="370303" & year==2006

replace regioncode="370304" if regioncode=="370304" & year==2006

replace regioncode="370305" if regioncode=="370305" & year==2006

replace regioncode="370306" if regioncode=="370306" & year==2006

replace regioncode="370321" if regioncode=="370321" & year==2006

replace regioncode="370322" if regioncode=="370322" & year==2006

replace regioncode="370323" if regioncode=="370323" & year==2006

replace regioncode="370402" if regioncode=="370402" & year==2006

replace regioncode="370403" if regioncode=="370403" & year==2006

replace regioncode="370404" if regioncode=="370404" & year==2006

replace regioncode="370405" if regioncode=="370405" & year==2006

replace regioncode="370406" if regioncode=="370406" & year==2006

replace regioncode="370481" if regioncode=="370481" & year==2006

replace regioncode="370502" if regioncode=="370502" & year==2006

replace regioncode="370503" if regioncode=="370503" & year==2006

replace regioncode="370521" if regioncode=="370521" & year==2006

replace regioncode="370522" if regioncode=="370522" & year==2006

replace regioncode="370523" if regioncode=="370523" & year==2006

replace regioncode="370602" if regioncode=="370602" & year==2006

replace regioncode="370611" if regioncode=="370611" & year==2006

replace regioncode="370612" if regioncode=="370612" & year==2006

replace regioncode="370613" if regioncode=="370613" & year==2006

replace regioncode="370634" if regioncode=="370634" & year==2006

replace regioncode="370681" if regioncode=="370681" & year==2006

replace regioncode="370682" if regioncode=="370682" & year==2006

replace regioncode="370683" if regioncode=="370683" & year==2006

replace regioncode="370684" if regioncode=="370684" & year==2006

replace regioncode="370685" if regioncode=="370685" & year==2006

replace regioncode="370686" if regioncode=="370686" & year==2006

replace regioncode="370687" if regioncode=="370687" & year==2006

replace regioncode="370702" if regioncode=="370702" & year==2006

replace regioncode="370703" if regioncode=="370703" & year==2006

replace regioncode="370704" if regioncode=="370704" & year==2006

replace regioncode="370705" if regioncode=="370705" & year==2006

replace regioncode="370724" if regioncode=="370724" & year==2006

replace regioncode="370725" if regioncode=="370725" & year==2006

replace regioncode="370781" if regioncode=="370781" & year==2006

replace regioncode="370782" if regioncode=="370782" & year==2006

replace regioncode="370783" if regioncode=="370783" & year==2006

replace regioncode="370784" if regioncode=="370784" & year==2006

replace regioncode="370785" if regioncode=="370785" & year==2006

replace regioncode="370786" if regioncode=="370786" & year==2006

replace regioncode="370802" if regioncode=="370802" & year==2006

replace regioncode="370811" if regioncode=="370811" & year==2006

replace regioncode="370826" if regioncode=="370826" & year==2006

replace regioncode="370827" if regioncode=="370827" & year==2006

replace regioncode="370828" if regioncode=="370828" & year==2006

replace regioncode="370829" if regioncode=="370829" & year==2006

replace regioncode="370830" if regioncode=="370830" & year==2006

replace regioncode="370831" if regioncode=="370831" & year==2006

replace regioncode="370832" if regioncode=="370832" & year==2006

replace regioncode="370881" if regioncode=="370881" & year==2006

replace regioncode="370882" if regioncode=="370882" & year==2006

replace regioncode="370883" if regioncode=="370883" & year==2006

replace regioncode="370902" if regioncode=="370902" & year==2006

replace regioncode="370903" if regioncode=="370903" & year==2006

replace regioncode="370921" if regioncode=="370921" & year==2006

replace regioncode="370923" if regioncode=="370923" & year==2006

replace regioncode="370982" if regioncode=="370982" & year==2006

replace regioncode="370983" if regioncode=="370983" & year==2006

replace regioncode="371002" if regioncode=="371002" & year==2006

replace regioncode="371081" if regioncode=="371081" & year==2006

replace regioncode="371082" if regioncode=="371082" & year==2006

replace regioncode="371083" if regioncode=="371083" & year==2006

replace regioncode="371102" if regioncode=="371102" & year==2006

replace regioncode="371121" if regioncode=="371121" & year==2006

replace regioncode="371122" if regioncode=="371122" & year==2006

replace regioncode="371202" if regioncode=="371202" & year==2006

replace regioncode="371203" if regioncode=="371203" & year==2006

replace regioncode="371302" if regioncode=="371302" & year==2006

replace regioncode="371311" if regioncode=="371311" & year==2006

replace regioncode="371312" if regioncode=="371312" & year==2006

replace regioncode="371321" if regioncode=="371321" & year==2006

replace regioncode="371322" if regioncode=="371322" & year==2006

replace regioncode="371323" if regioncode=="371323" & year==2006

replace regioncode="371324" if regioncode=="371324" & year==2006

replace regioncode="371325" if regioncode=="371325" & year==2006

replace regioncode="371326" if regioncode=="371326" & year==2006

replace regioncode="371327" if regioncode=="371327" & year==2006

replace regioncode="371328" if regioncode=="371328" & year==2006

replace regioncode="371329" if regioncode=="371329" & year==2006

replace regioncode="371402" if regioncode=="371402" & year==2006

replace regioncode="371421" if regioncode=="371421" & year==2006

replace regioncode="371422" if regioncode=="371422" & year==2006

replace regioncode="371423" if regioncode=="371423" & year==2006

replace regioncode="371424" if regioncode=="371424" & year==2006

replace regioncode="371425" if regioncode=="371425" & year==2006

replace regioncode="371426" if regioncode=="371426" & year==2006

replace regioncode="371427" if regioncode=="371427" & year==2006

replace regioncode="371428" if regioncode=="371428" & year==2006

replace regioncode="371481" if regioncode=="371481" & year==2006

replace regioncode="371482" if regioncode=="371482" & year==2006

replace regioncode="371502" if regioncode=="371502" & year==2006

replace regioncode="371521" if regioncode=="371521" & year==2006

replace regioncode="371522" if regioncode=="371522" & year==2006

replace regioncode="371523" if regioncode=="371523" & year==2006

replace regioncode="371524" if regioncode=="371524" & year==2006

replace regioncode="371525" if regioncode=="371525" & year==2006

replace regioncode="371526" if regioncode=="371526" & year==2006

replace regioncode="371581" if regioncode=="371581" & year==2006

replace regioncode="371602" if regioncode=="371602" & year==2006

replace regioncode="371621" if regioncode=="371621" & year==2006

replace regioncode="371622" if regioncode=="371622" & year==2006

replace regioncode="371623" if regioncode=="371623" & year==2006

replace regioncode="371624" if regioncode=="371624" & year==2006

replace regioncode="371625" if regioncode=="371625" & year==2006

replace regioncode="371626" if regioncode=="371626" & year==2006

replace regioncode="371702" if regioncode=="371702" & year==2006

replace regioncode="371721" if regioncode=="371721" & year==2006

replace regioncode="371722" if regioncode=="371722" & year==2006

replace regioncode="371723" if regioncode=="371723" & year==2006

replace regioncode="371724" if regioncode=="371724" & year==2006

replace regioncode="371725" if regioncode=="371725" & year==2006

replace regioncode="371726" if regioncode=="371726" & year==2006

replace regioncode="371727" if regioncode=="371727" & year==2006

replace regioncode="371728" if regioncode=="371728" & year==2006

replace regioncode="410102" if regioncode=="410102" & year==2006

replace regioncode="410103" if regioncode=="410103" & year==2006

replace regioncode="410104" if regioncode=="410104" & year==2006

replace regioncode="410105" if regioncode=="410105" & year==2006

replace regioncode="410106" if regioncode=="410106" & year==2006

replace regioncode="410108" if regioncode=="410108" & year==2006

replace regioncode="410122" if regioncode=="410122" & year==2006

replace regioncode="410181" if regioncode=="410181" & year==2006

replace regioncode="410182" if regioncode=="410182" & year==2006

replace regioncode="410183" if regioncode=="410183" & year==2006

replace regioncode="410184" if regioncode=="410184" & year==2006

replace regioncode="410185" if regioncode=="410185" & year==2006

replace regioncode="410202" if regioncode=="410202" & year==2006

replace regioncode="410203" if regioncode=="410203" & year==2006

replace regioncode="410204" if regioncode=="410204" & year==2006

replace regioncode="410205" if regioncode=="410205" & year==2006

replace regioncode="410211" if regioncode=="410211" & year==2006

replace regioncode="410221" if regioncode=="410221" & year==2006

replace regioncode="410222" if regioncode=="410222" & year==2006

replace regioncode="410223" if regioncode=="410223" & year==2006

replace regioncode="410224" if regioncode=="410224" & year==2006

replace regioncode="410225" if regioncode=="410225" & year==2006

replace regioncode="410302" if regioncode=="410302" & year==2006

replace regioncode="410303" if regioncode=="410303" & year==2006

replace regioncode="410304" if regioncode=="410304" & year==2006

replace regioncode="410305" if regioncode=="410305" & year==2006

replace regioncode="410306" if regioncode=="410306" & year==2006

replace regioncode="410307" if regioncode=="410307" & year==2006

replace regioncode="410322" if regioncode=="410322" & year==2006

replace regioncode="410323" if regioncode=="410323" & year==2006

replace regioncode="410324" if regioncode=="410324" & year==2006

replace regioncode="410325" if regioncode=="410325" & year==2006

replace regioncode="410326" if regioncode=="410326" & year==2006

replace regioncode="410327" if regioncode=="410327" & year==2006

replace regioncode="410328" if regioncode=="410328" & year==2006

replace regioncode="410329" if regioncode=="410329" & year==2006

replace regioncode="410381" if regioncode=="410381" & year==2006

replace regioncode="410402" if regioncode=="410402" & year==2006

replace regioncode="410403" if regioncode=="410403" & year==2006

replace regioncode="410404" if regioncode=="410404" & year==2006

replace regioncode="410411" if regioncode=="410411" & year==2006

replace regioncode="410421" if regioncode=="410421" & year==2006

replace regioncode="410422" if regioncode=="410422" & year==2006

replace regioncode="410423" if regioncode=="410423" & year==2006

replace regioncode="410425" if regioncode=="410425" & year==2006

replace regioncode="410481" if regioncode=="410481" & year==2006

replace regioncode="410482" if regioncode=="410482" & year==2006

replace regioncode="410502" if regioncode=="410502" & year==2006

replace regioncode="410503" if regioncode=="410503" & year==2006

replace regioncode="410504" if regioncode=="410505" & year==2006

replace regioncode="410511" if regioncode=="410506" & year==2006

replace regioncode="410522" if regioncode=="410522" & year==2006

replace regioncode="410523" if regioncode=="410523" & year==2006

replace regioncode="410526" if regioncode=="410526" & year==2006

replace regioncode="410527" if regioncode=="410527" & year==2006

replace regioncode="410581" if regioncode=="410581" & year==2006

replace regioncode="410602" if regioncode=="410602" & year==2006

replace regioncode="410603" if regioncode=="410603" & year==2006

replace regioncode="410611" if regioncode=="410611" & year==2006

replace regioncode="410621" if regioncode=="410621" & year==2006

replace regioncode="410622" if regioncode=="410622" & year==2006

replace regioncode="410702" if regioncode=="410702" & year==2006

replace regioncode="410703" if regioncode=="410703" & year==2006

replace regioncode="410704" if regioncode=="410704" & year==2006

replace regioncode="410711" if regioncode=="410711" & year==2006

replace regioncode="410721" if regioncode=="410721" & year==2006

replace regioncode="410724" if regioncode=="410724" & year==2006

replace regioncode="410725" if regioncode=="410725" & year==2006

replace regioncode="410726" if regioncode=="410726" & year==2006

replace regioncode="410727" if regioncode=="410727" & year==2006

replace regioncode="410728" if regioncode=="410728" & year==2006

replace regioncode="410781" if regioncode=="410781" & year==2006

replace regioncode="410782" if regioncode=="410782" & year==2006

replace regioncode="410802" if regioncode=="410802" & year==2006

replace regioncode="410803" if regioncode=="410803" & year==2006

replace regioncode="410804" if regioncode=="410804" & year==2006

replace regioncode="410811" if regioncode=="410811" & year==2006

replace regioncode="410821" if regioncode=="410821" & year==2006

replace regioncode="410822" if regioncode=="410822" & year==2006

replace regioncode="410823" if regioncode=="410823" & year==2006

replace regioncode="410825" if regioncode=="410825" & year==2006

replace regioncode="410881" if regioncode=="410881" & year==2006

replace regioncode="410882" if regioncode=="410882" & year==2006

replace regioncode="410883" if regioncode=="410883" & year==2006

replace regioncode="410902" if regioncode=="410902" & year==2006

replace regioncode="410922" if regioncode=="410922" & year==2006

replace regioncode="410923" if regioncode=="410923" & year==2006

replace regioncode="410926" if regioncode=="410926" & year==2006

replace regioncode="410927" if regioncode=="410927" & year==2006

replace regioncode="410928" if regioncode=="410928" & year==2006

replace regioncode="411002" if regioncode=="411002" & year==2006

replace regioncode="411023" if regioncode=="411023" & year==2006

replace regioncode="411024" if regioncode=="411024" & year==2006

replace regioncode="411025" if regioncode=="411025" & year==2006

replace regioncode="411081" if regioncode=="411081" & year==2006

replace regioncode="411082" if regioncode=="411082" & year==2006

replace regioncode="411102" if regioncode=="411102" & year==2006

replace regioncode="411123" if regioncode=="411103" & year==2006

replace regioncode="411121" if regioncode=="411121" & year==2006

replace regioncode="411122" if regioncode=="411122" & year==2006

replace regioncode="411202" if regioncode=="411202" & year==2006

replace regioncode="411221" if regioncode=="411221" & year==2006

replace regioncode="411222" if regioncode=="411222" & year==2006

replace regioncode="411224" if regioncode=="411224" & year==2006

replace regioncode="411281" if regioncode=="411281" & year==2006

replace regioncode="411282" if regioncode=="411282" & year==2006

replace regioncode="411302" if regioncode=="411302" & year==2006

replace regioncode="411303" if regioncode=="411303" & year==2006

replace regioncode="411321" if regioncode=="411321" & year==2006

replace regioncode="411322" if regioncode=="411322" & year==2006

replace regioncode="411323" if regioncode=="411323" & year==2006

replace regioncode="411324" if regioncode=="411324" & year==2006

replace regioncode="411325" if regioncode=="411325" & year==2006

replace regioncode="411326" if regioncode=="411326" & year==2006

replace regioncode="411327" if regioncode=="411327" & year==2006

replace regioncode="411328" if regioncode=="411328" & year==2006

replace regioncode="411329" if regioncode=="411329" & year==2006

replace regioncode="411330" if regioncode=="411330" & year==2006

replace regioncode="411381" if regioncode=="411381" & year==2006

replace regioncode="411402" if regioncode=="411402" & year==2006

replace regioncode="411403" if regioncode=="411403" & year==2006

replace regioncode="411421" if regioncode=="411421" & year==2006

replace regioncode="411422" if regioncode=="411422" & year==2006

replace regioncode="411423" if regioncode=="411423" & year==2006

replace regioncode="411424" if regioncode=="411424" & year==2006

replace regioncode="411425" if regioncode=="411425" & year==2006

replace regioncode="411426" if regioncode=="411426" & year==2006

replace regioncode="411481" if regioncode=="411481" & year==2006

replace regioncode="411502" if regioncode=="411502" & year==2006

replace regioncode="411503" if regioncode=="411503" & year==2006

replace regioncode="411521" if regioncode=="411521" & year==2006

replace regioncode="411522" if regioncode=="411522" & year==2006

replace regioncode="411523" if regioncode=="411523" & year==2006

replace regioncode="411524" if regioncode=="411524" & year==2006

replace regioncode="411525" if regioncode=="411525" & year==2006

replace regioncode="411526" if regioncode=="411526" & year==2006

replace regioncode="411527" if regioncode=="411527" & year==2006

replace regioncode="411528" if regioncode=="411528" & year==2006

replace regioncode="411602" if regioncode=="411602" & year==2006

replace regioncode="411621" if regioncode=="411621" & year==2006

replace regioncode="411622" if regioncode=="411622" & year==2006

replace regioncode="411623" if regioncode=="411623" & year==2006

replace regioncode="411624" if regioncode=="411624" & year==2006

replace regioncode="411625" if regioncode=="411625" & year==2006

replace regioncode="411626" if regioncode=="411626" & year==2006

replace regioncode="411627" if regioncode=="411627" & year==2006

replace regioncode="411628" if regioncode=="411628" & year==2006

replace regioncode="411681" if regioncode=="411681" & year==2006

replace regioncode="411702" if regioncode=="411702" & year==2006

replace regioncode="411721" if regioncode=="411721" & year==2006

replace regioncode="411722" if regioncode=="411722" & year==2006

replace regioncode="411723" if regioncode=="411723" & year==2006

replace regioncode="411724" if regioncode=="411724" & year==2006

replace regioncode="411725" if regioncode=="411725" & year==2006

replace regioncode="411726" if regioncode=="411726" & year==2006

replace regioncode="411727" if regioncode=="411727" & year==2006

replace regioncode="411728" if regioncode=="411728" & year==2006

replace regioncode="411729" if regioncode=="411729" & year==2006

replace regioncode="420102" if regioncode=="420102" & year==2006

replace regioncode="420103" if regioncode=="420103" & year==2006

replace regioncode="420104" if regioncode=="420104" & year==2006

replace regioncode="420105" if regioncode=="420105" & year==2006

replace regioncode="420106" if regioncode=="420106" & year==2006

replace regioncode="420107" if regioncode=="420107" & year==2006

replace regioncode="420111" if regioncode=="420111" & year==2006

replace regioncode="420112" if regioncode=="420112" & year==2006

replace regioncode="420113" if regioncode=="420113" & year==2006

replace regioncode="420114" if regioncode=="420114" & year==2006

replace regioncode="420115" if regioncode=="420115" & year==2006

replace regioncode="420116" if regioncode=="420116" & year==2006

replace regioncode="420117" if regioncode=="420117" & year==2006

replace regioncode="420202" if regioncode=="420202" & year==2006

replace regioncode="420203" if regioncode=="420203" & year==2006

replace regioncode="420204" if regioncode=="420204" & year==2006

replace regioncode="420205" if regioncode=="420205" & year==2006

replace regioncode="420222" if regioncode=="420222" & year==2006

replace regioncode="420281" if regioncode=="420281" & year==2006

replace regioncode="420302" if regioncode=="420302" & year==2006

replace regioncode="420303" if regioncode=="420303" & year==2006

replace regioncode="420321" if regioncode=="420321" & year==2006

replace regioncode="420322" if regioncode=="420322" & year==2006

replace regioncode="420323" if regioncode=="420323" & year==2006

replace regioncode="420324" if regioncode=="420324" & year==2006

replace regioncode="420325" if regioncode=="420325" & year==2006

replace regioncode="420381" if regioncode=="420381" & year==2006

replace regioncode="420502" if regioncode=="420502" & year==2006

replace regioncode="420503" if regioncode=="420503" & year==2006

replace regioncode="420504" if regioncode=="420504" & year==2006

replace regioncode="420505" if regioncode=="420505" & year==2006

replace regioncode="420506" if regioncode=="420506" & year==2006

replace regioncode="420525" if regioncode=="420525" & year==2006

replace regioncode="420526" if regioncode=="420526" & year==2006

replace regioncode="420527" if regioncode=="420527" & year==2006

replace regioncode="420528" if regioncode=="420528" & year==2006

replace regioncode="420529" if regioncode=="420529" & year==2006

replace regioncode="420581" if regioncode=="420581" & year==2006

replace regioncode="420582" if regioncode=="420582" & year==2006

replace regioncode="420583" if regioncode=="420583" & year==2006

replace regioncode="420602" if regioncode=="420602" & year==2006

replace regioncode="420606" if regioncode=="420606" & year==2006

replace regioncode="420607" if regioncode=="420607" & year==2006

replace regioncode="420624" if regioncode=="420624" & year==2006

replace regioncode="420625" if regioncode=="420625" & year==2006

replace regioncode="420626" if regioncode=="420626" & year==2006

replace regioncode="420682" if regioncode=="420682" & year==2006

replace regioncode="420683" if regioncode=="420683" & year==2006

replace regioncode="420684" if regioncode=="420684" & year==2006

replace regioncode="420702" if regioncode=="420702" & year==2006

replace regioncode="420703" if regioncode=="420703" & year==2006

replace regioncode="420704" if regioncode=="420704" & year==2006

replace regioncode="420802" if regioncode=="420802" & year==2006

replace regioncode="420804" if regioncode=="420804" & year==2006

replace regioncode="420821" if regioncode=="420821" & year==2006

replace regioncode="420822" if regioncode=="420822" & year==2006

replace regioncode="420881" if regioncode=="420881" & year==2006

replace regioncode="420902" if regioncode=="420902" & year==2006

replace regioncode="420921" if regioncode=="420921" & year==2006

replace regioncode="420922" if regioncode=="420922" & year==2006

replace regioncode="420923" if regioncode=="420923" & year==2006

replace regioncode="420981" if regioncode=="420981" & year==2006

replace regioncode="420982" if regioncode=="420982" & year==2006

replace regioncode="421381" if regioncode=="421381" & year==2006

replace regioncode="420984" if regioncode=="420984" & year==2006

replace regioncode="421002" if regioncode=="421002" & year==2006

replace regioncode="421003" if regioncode=="421003" & year==2006

replace regioncode="421022" if regioncode=="421022" & year==2006

replace regioncode="421023" if regioncode=="421023" & year==2006

replace regioncode="421024" if regioncode=="421024" & year==2006

replace regioncode="421081" if regioncode=="421081" & year==2006

replace regioncode="421083" if regioncode=="421083" & year==2006

replace regioncode="421087" if regioncode=="421087" & year==2006

replace regioncode="421102" if regioncode=="421102" & year==2006

replace regioncode="421121" if regioncode=="421121" & year==2006

replace regioncode="421122" if regioncode=="421122" & year==2006

replace regioncode="421123" if regioncode=="421123" & year==2006

replace regioncode="421124" if regioncode=="421124" & year==2006

replace regioncode="421125" if regioncode=="421125" & year==2006

replace regioncode="421126" if regioncode=="421126" & year==2006

replace regioncode="421127" if regioncode=="421127" & year==2006

replace regioncode="421181" if regioncode=="421181" & year==2006

replace regioncode="421182" if regioncode=="421182" & year==2006

replace regioncode="421202" if regioncode=="421202" & year==2006

replace regioncode="421221" if regioncode=="421221" & year==2006

replace regioncode="421222" if regioncode=="421222" & year==2006

replace regioncode="421223" if regioncode=="421223" & year==2006

replace regioncode="421224" if regioncode=="421224" & year==2006

replace regioncode="421281" if regioncode=="421281" & year==2006

replace regioncode="421302" if regioncode=="421302" & year==2006

replace regioncode="422801" if regioncode=="422801" & year==2006

replace regioncode="422802" if regioncode=="422802" & year==2006

replace regioncode="422822" if regioncode=="422822" & year==2006

replace regioncode="422823" if regioncode=="422823" & year==2006

replace regioncode="422825" if regioncode=="422825" & year==2006

replace regioncode="422826" if regioncode=="422826" & year==2006

replace regioncode="422827" if regioncode=="422827" & year==2006

replace regioncode="422828" if regioncode=="422828" & year==2006

replace regioncode="429004" if regioncode=="429004" & year==2006

replace regioncode="429005" if regioncode=="429005" & year==2006

replace regioncode="429006" if regioncode=="429006" & year==2006

replace regioncode="429021" if regioncode=="429021" & year==2006

replace regioncode="430102" if regioncode=="430102" & year==2006

replace regioncode="430103" if regioncode=="430103" & year==2006

replace regioncode="430104" if regioncode=="430104" & year==2006

replace regioncode="430105" if regioncode=="430105" & year==2006

replace regioncode="430111" if regioncode=="430111" & year==2006

replace regioncode="430121" if regioncode=="430121" & year==2006

replace regioncode="430122" if regioncode=="430122" & year==2006

replace regioncode="430124" if regioncode=="430124" & year==2006

replace regioncode="430181" if regioncode=="430181" & year==2006

replace regioncode="430202" if regioncode=="430202" & year==2006

replace regioncode="430203" if regioncode=="430203" & year==2006

replace regioncode="430204" if regioncode=="430204" & year==2006

replace regioncode="430211" if regioncode=="430211" & year==2006

replace regioncode="430221" if regioncode=="430221" & year==2006

replace regioncode="430223" if regioncode=="430223" & year==2006

replace regioncode="430224" if regioncode=="430224" & year==2006

replace regioncode="430225" if regioncode=="430225" & year==2006

replace regioncode="430281" if regioncode=="430281" & year==2006

replace regioncode="430302" if regioncode=="430302" & year==2006

replace regioncode="430304" if regioncode=="430304" & year==2006

replace regioncode="430321" if regioncode=="430321" & year==2006

replace regioncode="430381" if regioncode=="430381" & year==2006

replace regioncode="430382" if regioncode=="430382" & year==2006

replace regioncode="430405" if regioncode=="430405" & year==2006

replace regioncode="430406" if regioncode=="430406" & year==2006

replace regioncode="430407" if regioncode=="430407" & year==2006

replace regioncode="430408" if regioncode=="430408" & year==2006

replace regioncode="430412" if regioncode=="430412" & year==2006

replace regioncode="430421" if regioncode=="430421" & year==2006

replace regioncode="430422" if regioncode=="430422" & year==2006

replace regioncode="430423" if regioncode=="430423" & year==2006

replace regioncode="430424" if regioncode=="430424" & year==2006

replace regioncode="430426" if regioncode=="430426" & year==2006

replace regioncode="430481" if regioncode=="430481" & year==2006

replace regioncode="430482" if regioncode=="430482" & year==2006

replace regioncode="430502" if regioncode=="430502" & year==2006

replace regioncode="430503" if regioncode=="430503" & year==2006

replace regioncode="430511" if regioncode=="430511" & year==2006

replace regioncode="430521" if regioncode=="430521" & year==2006

replace regioncode="430522" if regioncode=="430522" & year==2006

replace regioncode="430523" if regioncode=="430523" & year==2006

replace regioncode="430524" if regioncode=="430524" & year==2006

replace regioncode="430525" if regioncode=="430525" & year==2006

replace regioncode="430527" if regioncode=="430527" & year==2006

replace regioncode="430528" if regioncode=="430528" & year==2006

replace regioncode="430529" if regioncode=="430529" & year==2006

replace regioncode="430581" if regioncode=="430581" & year==2006

replace regioncode="430602" if regioncode=="430602" & year==2006

replace regioncode="430603" if regioncode=="430603" & year==2006

replace regioncode="430611" if regioncode=="430611" & year==2006

replace regioncode="430621" if regioncode=="430621" & year==2006

replace regioncode="430623" if regioncode=="430623" & year==2006

replace regioncode="430624" if regioncode=="430624" & year==2006

replace regioncode="430626" if regioncode=="430626" & year==2006

replace regioncode="430681" if regioncode=="430681" & year==2006

replace regioncode="430682" if regioncode=="430682" & year==2006

replace regioncode="430702" if regioncode=="430702" & year==2006

replace regioncode="430703" if regioncode=="430703" & year==2006

replace regioncode="430721" if regioncode=="430721" & year==2006

replace regioncode="430722" if regioncode=="430722" & year==2006

replace regioncode="430723" if regioncode=="430723" & year==2006

replace regioncode="430724" if regioncode=="430724" & year==2006

replace regioncode="430725" if regioncode=="430725" & year==2006

replace regioncode="430726" if regioncode=="430726" & year==2006

replace regioncode="430781" if regioncode=="430781" & year==2006

replace regioncode="430802" if regioncode=="430802" & year==2006

replace regioncode="430811" if regioncode=="430811" & year==2006

replace regioncode="430821" if regioncode=="430821" & year==2006

replace regioncode="430822" if regioncode=="430822" & year==2006

replace regioncode="430902" if regioncode=="430902" & year==2006

replace regioncode="430903" if regioncode=="430903" & year==2006

replace regioncode="430921" if regioncode=="430921" & year==2006

replace regioncode="430922" if regioncode=="430922" & year==2006

replace regioncode="430923" if regioncode=="430923" & year==2006

replace regioncode="430981" if regioncode=="430981" & year==2006

replace regioncode="431002" if regioncode=="431002" & year==2006

replace regioncode="431003" if regioncode=="431003" & year==2006

replace regioncode="431021" if regioncode=="431021" & year==2006

replace regioncode="431022" if regioncode=="431022" & year==2006

replace regioncode="431023" if regioncode=="431023" & year==2006

replace regioncode="431024" if regioncode=="431024" & year==2006

replace regioncode="431025" if regioncode=="431025" & year==2006

replace regioncode="431026" if regioncode=="431026" & year==2006

replace regioncode="431027" if regioncode=="431027" & year==2006

replace regioncode="431028" if regioncode=="431028" & year==2006

replace regioncode="431081" if regioncode=="431081" & year==2006

replace regioncode="431102" if regioncode=="431102" & year==2006

replace regioncode="431103" if regioncode=="431103" & year==2006

replace regioncode="431121" if regioncode=="431121" & year==2006

replace regioncode="431122" if regioncode=="431122" & year==2006

replace regioncode="431123" if regioncode=="431123" & year==2006

replace regioncode="431124" if regioncode=="431124" & year==2006

replace regioncode="431125" if regioncode=="431125" & year==2006

replace regioncode="431126" if regioncode=="431126" & year==2006

replace regioncode="431127" if regioncode=="431127" & year==2006

replace regioncode="431128" if regioncode=="431128" & year==2006

replace regioncode="431129" if regioncode=="431129" & year==2006

replace regioncode="431202" if regioncode=="431202" & year==2006

replace regioncode="431221" if regioncode=="431221" & year==2006

replace regioncode="431222" if regioncode=="431222" & year==2006

replace regioncode="431223" if regioncode=="431223" & year==2006

replace regioncode="431224" if regioncode=="431224" & year==2006

replace regioncode="431225" if regioncode=="431225" & year==2006

replace regioncode="431226" if regioncode=="431226" & year==2006

replace regioncode="431227" if regioncode=="431227" & year==2006

replace regioncode="431228" if regioncode=="431228" & year==2006

replace regioncode="431229" if regioncode=="431229" & year==2006

replace regioncode="431230" if regioncode=="431230" & year==2006

replace regioncode="431281" if regioncode=="431281" & year==2006

replace regioncode="431302" if regioncode=="431302" & year==2006

replace regioncode="431321" if regioncode=="431321" & year==2006

replace regioncode="431322" if regioncode=="431322" & year==2006

replace regioncode="431381" if regioncode=="431381" & year==2006

replace regioncode="431382" if regioncode=="431382" & year==2006

replace regioncode="433101" if regioncode=="433101" & year==2006

replace regioncode="433122" if regioncode=="433122" & year==2006

replace regioncode="433123" if regioncode=="433123" & year==2006

replace regioncode="433124" if regioncode=="433124" & year==2006

replace regioncode="433125" if regioncode=="433125" & year==2006

replace regioncode="433126" if regioncode=="433126" & year==2006

replace regioncode="433127" if regioncode=="433127" & year==2006

replace regioncode="433130" if regioncode=="433130" & year==2006

replace regioncode="440102" if regioncode=="440102" & year==2006

replace regioncode="440103" if regioncode=="440103" & year==2006

replace regioncode="440104" if regioncode=="440104" & year==2006

replace regioncode="440105" if regioncode=="440105" & year==2006

replace regioncode="440106" if regioncode=="440106" & year==2006

replace regioncode="440107" if regioncode=="440116" & year==2006

replace regioncode="440111" if regioncode=="440111" & year==2006

replace regioncode="440112" if regioncode=="440112" & year==2006

replace regioncode="440113" if regioncode=="440113" & year==2006

replace regioncode="440114" if regioncode=="440114" & year==2006

replace regioncode="440183" if regioncode=="440183" & year==2006

replace regioncode="440184" if regioncode=="440184" & year==2006

replace regioncode="440203" if regioncode=="440203" & year==2006

replace regioncode="440204" if regioncode=="440204" & year==2006

replace regioncode="440221" if regioncode=="440205" & year==2006

replace regioncode="440222" if regioncode=="440222" & year==2006

replace regioncode="440224" if regioncode=="440224" & year==2006

replace regioncode="440229" if regioncode=="440229" & year==2006

replace regioncode="440232" if regioncode=="440232" & year==2006

replace regioncode="440233" if regioncode=="440233" & year==2006

replace regioncode="440281" if regioncode=="440281" & year==2006

replace regioncode="440282" if regioncode=="440282" & year==2006

replace regioncode="440303" if regioncode=="440303" & year==2006

replace regioncode="440304" if regioncode=="440304" & year==2006

replace regioncode="440305" if regioncode=="440305" & year==2006

replace regioncode="440306" if regioncode=="440306" & year==2006

replace regioncode="440307" if regioncode=="440307" & year==2006

replace regioncode="440308" if regioncode=="440308" & year==2006

replace regioncode="440402" if regioncode=="440402" & year==2006

replace regioncode="440403" if regioncode=="440403" & year==2006

replace regioncode="440404" if regioncode=="440404" & year==2006

replace regioncode="440507" if regioncode=="440507" & year==2006

replace regioncode="440511" if regioncode=="440511" & year==2006

replace regioncode="440512" if regioncode=="440512" & year==2006

replace regioncode="440514" if regioncode=="440514" & year==2006

replace regioncode="440515" if regioncode=="440515" & year==2006

replace regioncode="440523" if regioncode=="440523" & year==2006

replace regioncode="440604" if regioncode=="440604" & year==2006

replace regioncode="440605" if regioncode=="440605" & year==2006

replace regioncode="440606" if regioncode=="440606" & year==2006

replace regioncode="440607" if regioncode=="440607" & year==2006

replace regioncode="440608" if regioncode=="440608" & year==2006

replace regioncode="440703" if regioncode=="440703" & year==2006

replace regioncode="440704" if regioncode=="440704" & year==2006

replace regioncode="440781" if regioncode=="440781" & year==2006

replace regioncode="440705" if regioncode=="440705" & year==2006

replace regioncode="440783" if regioncode=="440783" & year==2006

replace regioncode="440784" if regioncode=="440784" & year==2006

replace regioncode="440785" if regioncode=="440785" & year==2006

replace regioncode="440802" if regioncode=="440802" & year==2006

replace regioncode="440803" if regioncode=="440803" & year==2006

replace regioncode="440804" if regioncode=="440804" & year==2006

replace regioncode="440811" if regioncode=="440811" & year==2006

replace regioncode="440823" if regioncode=="440823" & year==2006

replace regioncode="440825" if regioncode=="440825" & year==2006

replace regioncode="440881" if regioncode=="440881" & year==2006

replace regioncode="440882" if regioncode=="440882" & year==2006

replace regioncode="440883" if regioncode=="440883" & year==2006

replace regioncode="440902" if regioncode=="440902" & year==2006

replace regioncode="440903" if regioncode=="440903" & year==2006

replace regioncode="440923" if regioncode=="440923" & year==2006

replace regioncode="440981" if regioncode=="440981" & year==2006

replace regioncode="440982" if regioncode=="440982" & year==2006

replace regioncode="440983" if regioncode=="440983" & year==2006

replace regioncode="441202" if regioncode=="441202" & year==2006

replace regioncode="441203" if regioncode=="441203" & year==2006

replace regioncode="441223" if regioncode=="441223" & year==2006

replace regioncode="441224" if regioncode=="441224" & year==2006

replace regioncode="441225" if regioncode=="441225" & year==2006

replace regioncode="441226" if regioncode=="441226" & year==2006

replace regioncode="441283" if regioncode=="441283" & year==2006

replace regioncode="441284" if regioncode=="441284" & year==2006

replace regioncode="441302" if regioncode=="441302" & year==2006

replace regioncode="441303" if regioncode=="441303" & year==2006

replace regioncode="441322" if regioncode=="441322" & year==2006

replace regioncode="441323" if regioncode=="441323" & year==2006

replace regioncode="441324" if regioncode=="441324" & year==2006

replace regioncode="441402" if regioncode=="441402" & year==2006

replace regioncode="441421" if regioncode=="441421" & year==2006

replace regioncode="441422" if regioncode=="441422" & year==2006

replace regioncode="441423" if regioncode=="441423" & year==2006

replace regioncode="441424" if regioncode=="441424" & year==2006

replace regioncode="441426" if regioncode=="441426" & year==2006

replace regioncode="441427" if regioncode=="441427" & year==2006

replace regioncode="441481" if regioncode=="441481" & year==2006

replace regioncode="441502" if regioncode=="441502" & year==2006

replace regioncode="441521" if regioncode=="441521" & year==2006

replace regioncode="441523" if regioncode=="441523" & year==2006

replace regioncode="441581" if regioncode=="441581" & year==2006

replace regioncode="441602" if regioncode=="441602" & year==2006

replace regioncode="441621" if regioncode=="441621" & year==2006

replace regioncode="441622" if regioncode=="441622" & year==2006

replace regioncode="441623" if regioncode=="441623" & year==2006

replace regioncode="441624" if regioncode=="441624" & year==2006

replace regioncode="441625" if regioncode=="441625" & year==2006

replace regioncode="441702" if regioncode=="441702" & year==2006

replace regioncode="441721" if regioncode=="441721" & year==2006

replace regioncode="441723" if regioncode=="441723" & year==2006

replace regioncode="441781" if regioncode=="441781" & year==2006

replace regioncode="441802" if regioncode=="441802" & year==2006

replace regioncode="441821" if regioncode=="441821" & year==2006

replace regioncode="441823" if regioncode=="441823" & year==2006

replace regioncode="441825" if regioncode=="441825" & year==2006

replace regioncode="441826" if regioncode=="441826" & year==2006

replace regioncode="441827" if regioncode=="441827" & year==2006

replace regioncode="441881" if regioncode=="441881" & year==2006

replace regioncode="441882" if regioncode=="441882" & year==2006

replace regioncode="441900" if regioncode=="441900" & year==2006

replace regioncode="442000" if regioncode=="442000" & year==2006

replace regioncode="445102" if regioncode=="445102" & year==2006

replace regioncode="445121" if regioncode=="445121" & year==2006

replace regioncode="445122" if regioncode=="445122" & year==2006

replace regioncode="445202" if regioncode=="445202" & year==2006

replace regioncode="445221" if regioncode=="445221" & year==2006

replace regioncode="445222" if regioncode=="445222" & year==2006

replace regioncode="445224" if regioncode=="445224" & year==2006

replace regioncode="445281" if regioncode=="445281" & year==2006

replace regioncode="445302" if regioncode=="445302" & year==2006

replace regioncode="445321" if regioncode=="445321" & year==2006

replace regioncode="445322" if regioncode=="445322" & year==2006

replace regioncode="445323" if regioncode=="445323" & year==2006

replace regioncode="445381" if regioncode=="445381" & year==2006

replace regioncode="450102" if regioncode=="450102" & year==2006

replace regioncode="450103" if regioncode=="450103" & year==2006

replace regioncode="450104" if regioncode=="450107" & year==2006

replace regioncode="450105" if regioncode=="450105" & year==2006

replace regioncode="450106" if regioncode=="450108" & year==2006

replace regioncode="450121" if regioncode=="450109" & year==2006

replace regioncode="450122" if regioncode=="450122" & year==2006

replace regioncode="450123" if regioncode=="450123" & year==2006

replace regioncode="450124" if regioncode=="450124" & year==2006

replace regioncode="450125" if regioncode=="450125" & year==2006

replace regioncode="450126" if regioncode=="450126" & year==2006

replace regioncode="450127" if regioncode=="450127" & year==2006

replace regioncode="450202" if regioncode=="450202" & year==2006

replace regioncode="450203" if regioncode=="450203" & year==2006

replace regioncode="450204" if regioncode=="450204" & year==2006

replace regioncode="450205" if regioncode=="450205" & year==2006

replace regioncode="450221" if regioncode=="450221" & year==2006

replace regioncode="450222" if regioncode=="450222" & year==2006

replace regioncode="450223" if regioncode=="450223" & year==2006

replace regioncode="450224" if regioncode=="450224" & year==2006

replace regioncode="450225" if regioncode=="450225" & year==2006

replace regioncode="450226" if regioncode=="450226" & year==2006

replace regioncode="450302" if regioncode=="450302" & year==2006

replace regioncode="450303" if regioncode=="450303" & year==2006

replace regioncode="450304" if regioncode=="450304" & year==2006

replace regioncode="450305" if regioncode=="450305" & year==2006

replace regioncode="450311" if regioncode=="450311" & year==2006

replace regioncode="450321" if regioncode=="450321" & year==2006

replace regioncode="450322" if regioncode=="450322" & year==2006

replace regioncode="450323" if regioncode=="450323" & year==2006

replace regioncode="450324" if regioncode=="450324" & year==2006

replace regioncode="450325" if regioncode=="450325" & year==2006

replace regioncode="450326" if regioncode=="450326" & year==2006

replace regioncode="450327" if regioncode=="450327" & year==2006

replace regioncode="450328" if regioncode=="450328" & year==2006

replace regioncode="450329" if regioncode=="450329" & year==2006

replace regioncode="450330" if regioncode=="450330" & year==2006

replace regioncode="450331" if regioncode=="450331" & year==2006

replace regioncode="450332" if regioncode=="450332" & year==2006

replace regioncode="450403" if regioncode=="450403" & year==2006

replace regioncode="450404" if regioncode=="450404" & year==2006

replace regioncode="450421" if regioncode=="450421" & year==2006

replace regioncode="450422" if regioncode=="450422" & year==2006

replace regioncode="450423" if regioncode=="450423" & year==2006

replace regioncode="450481" if regioncode=="450481" & year==2006

replace regioncode="450502" if regioncode=="450502" & year==2006

replace regioncode="450503" if regioncode=="450503" & year==2006

replace regioncode="450512" if regioncode=="450512" & year==2006

replace regioncode="450521" if regioncode=="450521" & year==2006

replace regioncode="450602" if regioncode=="450602" & year==2006

replace regioncode="450603" if regioncode=="450603" & year==2006

replace regioncode="450621" if regioncode=="450621" & year==2006

replace regioncode="450681" if regioncode=="450681" & year==2006

replace regioncode="450702" if regioncode=="450702" & year==2006

replace regioncode="450703" if regioncode=="450703" & year==2006

replace regioncode="450721" if regioncode=="450721" & year==2006

replace regioncode="450722" if regioncode=="450722" & year==2006

replace regioncode="450802" if regioncode=="450802" & year==2006

replace regioncode="450803" if regioncode=="450803" & year==2006

replace regioncode="450804" if regioncode=="450804" & year==2006

replace regioncode="450821" if regioncode=="450821" & year==2006

replace regioncode="450881" if regioncode=="450881" & year==2006

replace regioncode="450902" if regioncode=="450902" & year==2006

replace regioncode="450921" if regioncode=="450921" & year==2006

replace regioncode="450922" if regioncode=="450922" & year==2006

replace regioncode="450923" if regioncode=="450923" & year==2006

replace regioncode="450924" if regioncode=="450924" & year==2006

replace regioncode="450981" if regioncode=="450981" & year==2006

replace regioncode="451002" if regioncode=="451002" & year==2006

replace regioncode="451021" if regioncode=="451021" & year==2006

replace regioncode="451022" if regioncode=="451022" & year==2006

replace regioncode="451023" if regioncode=="451023" & year==2006

replace regioncode="451024" if regioncode=="451024" & year==2006

replace regioncode="451025" if regioncode=="451025" & year==2006

replace regioncode="451026" if regioncode=="451026" & year==2006

replace regioncode="451027" if regioncode=="451027" & year==2006

replace regioncode="451028" if regioncode=="451028" & year==2006

replace regioncode="451029" if regioncode=="451029" & year==2006

replace regioncode="451030" if regioncode=="451030" & year==2006

replace regioncode="451031" if regioncode=="451031" & year==2006

replace regioncode="451102" if regioncode=="451102" & year==2006

replace regioncode="451121" if regioncode=="451121" & year==2006

replace regioncode="451122" if regioncode=="451122" & year==2006

replace regioncode="451123" if regioncode=="451123" & year==2006

replace regioncode="451202" if regioncode=="451202" & year==2006

replace regioncode="451221" if regioncode=="451221" & year==2006

replace regioncode="451222" if regioncode=="451222" & year==2006

replace regioncode="451223" if regioncode=="451223" & year==2006

replace regioncode="451224" if regioncode=="451224" & year==2006

replace regioncode="451225" if regioncode=="451225" & year==2006

replace regioncode="451226" if regioncode=="451226" & year==2006

replace regioncode="451227" if regioncode=="451227" & year==2006

replace regioncode="451228" if regioncode=="451228" & year==2006

replace regioncode="451229" if regioncode=="451229" & year==2006

replace regioncode="451281" if regioncode=="451281" & year==2006

replace regioncode="451302" if regioncode=="451302" & year==2006

replace regioncode="451321" if regioncode=="451321" & year==2006

replace regioncode="451322" if regioncode=="451322" & year==2006

replace regioncode="451323" if regioncode=="451323" & year==2006

replace regioncode="451324" if regioncode=="451324" & year==2006

replace regioncode="451381" if regioncode=="451381" & year==2006

replace regioncode="451402" if regioncode=="451402" & year==2006

replace regioncode="451421" if regioncode=="451421" & year==2006

replace regioncode="451422" if regioncode=="451422" & year==2006

replace regioncode="451423" if regioncode=="451423" & year==2006

replace regioncode="451424" if regioncode=="451424" & year==2006

replace regioncode="451425" if regioncode=="451425" & year==2006

replace regioncode="451481" if regioncode=="451481" & year==2006

replace regioncode="460105" if regioncode=="460105" & year==2006

replace regioncode="460106" if regioncode=="460106" & year==2006

replace regioncode="460107" if regioncode=="460107" & year==2006

replace regioncode="460108" if regioncode=="460108" & year==2006

replace regioncode="460001" if regioncode=="469001" & year==2006

replace regioncode="460002" if regioncode=="469002" & year==2006

replace regioncode="460003" if regioncode=="469003" & year==2006

replace regioncode="460005" if regioncode=="469005" & year==2006

replace regioncode="460006" if regioncode=="469006" & year==2006

replace regioncode="460007" if regioncode=="469007" & year==2006

replace regioncode="460030" if regioncode=="469025" & year==2006

replace regioncode="460031" if regioncode=="469026" & year==2006

replace regioncode="460033" if regioncode=="469027" & year==2006

replace regioncode="460034" if regioncode=="469028" & year==2006

replace regioncode="460036" if regioncode=="469030" & year==2006

replace regioncode="500101" if regioncode=="500101" & year==2006

replace regioncode="500102" if regioncode=="500102" & year==2006

replace regioncode="500103" if regioncode=="500103" & year==2006

replace regioncode="500104" if regioncode=="500104" & year==2006

replace regioncode="500105" if regioncode=="500105" & year==2006

replace regioncode="500106" if regioncode=="500106" & year==2006

replace regioncode="500107" if regioncode=="500107" & year==2006

replace regioncode="500108" if regioncode=="500108" & year==2006

replace regioncode="500109" if regioncode=="500109" & year==2006

replace regioncode="500110" if regioncode=="500110" & year==2006

replace regioncode="500111" if regioncode=="500111" & year==2006

replace regioncode="500112" if regioncode=="500112" & year==2006

replace regioncode="500113" if regioncode=="500113" & year==2006

replace regioncode="500114" if regioncode=="500114" & year==2006

replace regioncode="500115" if regioncode=="500115" & year==2006

replace regioncode="500381" if regioncode=="500381" & year==2006

replace regioncode="500382" if regioncode=="500382" & year==2006

replace regioncode="500383" if regioncode=="500383" & year==2006

replace regioncode="500384" if regioncode=="500384" & year==2006

replace regioncode="500222" if regioncode=="500222" & year==2006

replace regioncode="500223" if regioncode=="500223" & year==2006

replace regioncode="500224" if regioncode=="500224" & year==2006

replace regioncode="500225" if regioncode=="500225" & year==2006

replace regioncode="500226" if regioncode=="500226" & year==2006

replace regioncode="500227" if regioncode=="500227" & year==2006

replace regioncode="500228" if regioncode=="500228" & year==2006

replace regioncode="500229" if regioncode=="500229" & year==2006

replace regioncode="500230" if regioncode=="500230" & year==2006

replace regioncode="500231" if regioncode=="500231" & year==2006

replace regioncode="500232" if regioncode=="500232" & year==2006

replace regioncode="500233" if regioncode=="500233" & year==2006

replace regioncode="500234" if regioncode=="500234" & year==2006

replace regioncode="500235" if regioncode=="500235" & year==2006

replace regioncode="500236" if regioncode=="500236" & year==2006

replace regioncode="500237" if regioncode=="500237" & year==2006

replace regioncode="500238" if regioncode=="500238" & year==2006

replace regioncode="500240" if regioncode=="500240" & year==2006

replace regioncode="500241" if regioncode=="500241" & year==2006

replace regioncode="500242" if regioncode=="500242" & year==2006

replace regioncode="500243" if regioncode=="500243" & year==2006

replace regioncode="510104" if regioncode=="510104" & year==2006

replace regioncode="510105" if regioncode=="510105" & year==2006

replace regioncode="510106" if regioncode=="510106" & year==2006

replace regioncode="510107" if regioncode=="510107" & year==2006

replace regioncode="510108" if regioncode=="510108" & year==2006

replace regioncode="510112" if regioncode=="510112" & year==2006

replace regioncode="510113" if regioncode=="510113" & year==2006

replace regioncode="510114" if regioncode=="510114" & year==2006

replace regioncode="510115" if regioncode=="510115" & year==2006

replace regioncode="510121" if regioncode=="510121" & year==2006

replace regioncode="510122" if regioncode=="510122" & year==2006

replace regioncode="510124" if regioncode=="510124" & year==2006

replace regioncode="510129" if regioncode=="510129" & year==2006

replace regioncode="510131" if regioncode=="510131" & year==2006

replace regioncode="510132" if regioncode=="510132" & year==2006

replace regioncode="510181" if regioncode=="510181" & year==2006

replace regioncode="510182" if regioncode=="510182" & year==2006

replace regioncode="510183" if regioncode=="510183" & year==2006

replace regioncode="510184" if regioncode=="510184" & year==2006

replace regioncode="510302" if regioncode=="510302" & year==2006

replace regioncode="510303" if regioncode=="510303" & year==2006

replace regioncode="510304" if regioncode=="510304" & year==2006

replace regioncode="510311" if regioncode=="510311" & year==2006

replace regioncode="510321" if regioncode=="510321" & year==2006

replace regioncode="510322" if regioncode=="510322" & year==2006

replace regioncode="510402" if regioncode=="510402" & year==2006

replace regioncode="510403" if regioncode=="510403" & year==2006

replace regioncode="510411" if regioncode=="510411" & year==2006

replace regioncode="510421" if regioncode=="510421" & year==2006

replace regioncode="510422" if regioncode=="510422" & year==2006

replace regioncode="510502" if regioncode=="510502" & year==2006

replace regioncode="510503" if regioncode=="510503" & year==2006

replace regioncode="510504" if regioncode=="510504" & year==2006

replace regioncode="510521" if regioncode=="510521" & year==2006

replace regioncode="510522" if regioncode=="510522" & year==2006

replace regioncode="510524" if regioncode=="510524" & year==2006

replace regioncode="510525" if regioncode=="510525" & year==2006

replace regioncode="510603" if regioncode=="510603" & year==2006

replace regioncode="510623" if regioncode=="510623" & year==2006

replace regioncode="510626" if regioncode=="510626" & year==2006

replace regioncode="510681" if regioncode=="510681" & year==2006

replace regioncode="510682" if regioncode=="510682" & year==2006

replace regioncode="510683" if regioncode=="510683" & year==2006

replace regioncode="510703" if regioncode=="510703" & year==2006

replace regioncode="510704" if regioncode=="510704" & year==2006

replace regioncode="510722" if regioncode=="510722" & year==2006

replace regioncode="510723" if regioncode=="510723" & year==2006

replace regioncode="510724" if regioncode=="510724" & year==2006

replace regioncode="510725" if regioncode=="510725" & year==2006

replace regioncode="510726" if regioncode=="510726" & year==2006

replace regioncode="510727" if regioncode=="510727" & year==2006

replace regioncode="510781" if regioncode=="510781" & year==2006

replace regioncode="510802" if regioncode=="510802" & year==2006

replace regioncode="510811" if regioncode=="510811" & year==2006

replace regioncode="510812" if regioncode=="510812" & year==2006

replace regioncode="510821" if regioncode=="510821" & year==2006

replace regioncode="510822" if regioncode=="510822" & year==2006

replace regioncode="510823" if regioncode=="510823" & year==2006

replace regioncode="510824" if regioncode=="510824" & year==2006

replace regioncode="510902" if regioncode=="510903" & year==2006

replace regioncode="510921" if regioncode=="510921" & year==2006

replace regioncode="510922" if regioncode=="510922" & year==2006

replace regioncode="510923" if regioncode=="510923" & year==2006

replace regioncode="511002" if regioncode=="511002" & year==2006

replace regioncode="511011" if regioncode=="511011" & year==2006

replace regioncode="511024" if regioncode=="511024" & year==2006

replace regioncode="511025" if regioncode=="511025" & year==2006

replace regioncode="511028" if regioncode=="511028" & year==2006

replace regioncode="511102" if regioncode=="511102" & year==2006

replace regioncode="511111" if regioncode=="511111" & year==2006

replace regioncode="511112" if regioncode=="511112" & year==2006

replace regioncode="511113" if regioncode=="511113" & year==2006

replace regioncode="511123" if regioncode=="511123" & year==2006

replace regioncode="511124" if regioncode=="511124" & year==2006

replace regioncode="511126" if regioncode=="511126" & year==2006

replace regioncode="511129" if regioncode=="511129" & year==2006

replace regioncode="511132" if regioncode=="511132" & year==2006

replace regioncode="511133" if regioncode=="511133" & year==2006

replace regioncode="511181" if regioncode=="511181" & year==2006

replace regioncode="511302" if regioncode=="511302" & year==2006

replace regioncode="511303" if regioncode=="511303" & year==2006

replace regioncode="511304" if regioncode=="511304" & year==2006

replace regioncode="511321" if regioncode=="511321" & year==2006

replace regioncode="511322" if regioncode=="511322" & year==2006

replace regioncode="511323" if regioncode=="511323" & year==2006

replace regioncode="511324" if regioncode=="511324" & year==2006

replace regioncode="511325" if regioncode=="511325" & year==2006

replace regioncode="511381" if regioncode=="511381" & year==2006

replace regioncode="511402" if regioncode=="511402" & year==2006

replace regioncode="511421" if regioncode=="511421" & year==2006

replace regioncode="511422" if regioncode=="511422" & year==2006

replace regioncode="511423" if regioncode=="511423" & year==2006

replace regioncode="511424" if regioncode=="511424" & year==2006

replace regioncode="511425" if regioncode=="511425" & year==2006

replace regioncode="511502" if regioncode=="511502" & year==2006

replace regioncode="511521" if regioncode=="511521" & year==2006

replace regioncode="511522" if regioncode=="511522" & year==2006

replace regioncode="511523" if regioncode=="511523" & year==2006

replace regioncode="511524" if regioncode=="511524" & year==2006

replace regioncode="511525" if regioncode=="511525" & year==2006

replace regioncode="511526" if regioncode=="511526" & year==2006

replace regioncode="511527" if regioncode=="511527" & year==2006

replace regioncode="511528" if regioncode=="511528" & year==2006

replace regioncode="511529" if regioncode=="511529" & year==2006

replace regioncode="511602" if regioncode=="511602" & year==2006

replace regioncode="511621" if regioncode=="511621" & year==2006

replace regioncode="511622" if regioncode=="511622" & year==2006

replace regioncode="511623" if regioncode=="511623" & year==2006

replace regioncode="511681" if regioncode=="511681" & year==2006

replace regioncode="511702" if regioncode=="511702" & year==2006

replace regioncode="511721" if regioncode=="511721" & year==2006

replace regioncode="511722" if regioncode=="511722" & year==2006

replace regioncode="511723" if regioncode=="511723" & year==2006

replace regioncode="511724" if regioncode=="511724" & year==2006

replace regioncode="511725" if regioncode=="511725" & year==2006

replace regioncode="511781" if regioncode=="511781" & year==2006

replace regioncode="511802" if regioncode=="511802" & year==2006

replace regioncode="511821" if regioncode=="511821" & year==2006

replace regioncode="511822" if regioncode=="511822" & year==2006

replace regioncode="511823" if regioncode=="511823" & year==2006

replace regioncode="511824" if regioncode=="511824" & year==2006

replace regioncode="511825" if regioncode=="511825" & year==2006

replace regioncode="511826" if regioncode=="511826" & year==2006

replace regioncode="511827" if regioncode=="511827" & year==2006

replace regioncode="511902" if regioncode=="511902" & year==2006

replace regioncode="511921" if regioncode=="511921" & year==2006

replace regioncode="511922" if regioncode=="511922" & year==2006

replace regioncode="511923" if regioncode=="511923" & year==2006

replace regioncode="512002" if regioncode=="512002" & year==2006

replace regioncode="512021" if regioncode=="512021" & year==2006

replace regioncode="512022" if regioncode=="512022" & year==2006

replace regioncode="512081" if regioncode=="512081" & year==2006

replace regioncode="513221" if regioncode=="513221" & year==2006

replace regioncode="513222" if regioncode=="513222" & year==2006

replace regioncode="513223" if regioncode=="513223" & year==2006

replace regioncode="513224" if regioncode=="513224" & year==2006

replace regioncode="513225" if regioncode=="513225" & year==2006

replace regioncode="513226" if regioncode=="513226" & year==2006

replace regioncode="513227" if regioncode=="513227" & year==2006

replace regioncode="513228" if regioncode=="513228" & year==2006

replace regioncode="513229" if regioncode=="513229" & year==2006

replace regioncode="513230" if regioncode=="513230" & year==2006

replace regioncode="513231" if regioncode=="513231" & year==2006

replace regioncode="513232" if regioncode=="513232" & year==2006

replace regioncode="513233" if regioncode=="513233" & year==2006

replace regioncode="513321" if regioncode=="513321" & year==2006

replace regioncode="513322" if regioncode=="513322" & year==2006

replace regioncode="513323" if regioncode=="513323" & year==2006

replace regioncode="513324" if regioncode=="513324" & year==2006

replace regioncode="513325" if regioncode=="513325" & year==2006

replace regioncode="513326" if regioncode=="513326" & year==2006

replace regioncode="513327" if regioncode=="513327" & year==2006

replace regioncode="513328" if regioncode=="513328" & year==2006

replace regioncode="513329" if regioncode=="513329" & year==2006

replace regioncode="513330" if regioncode=="513330" & year==2006

replace regioncode="513331" if regioncode=="513331" & year==2006

replace regioncode="513332" if regioncode=="513332" & year==2006

replace regioncode="513333" if regioncode=="513333" & year==2006

replace regioncode="513334" if regioncode=="513334" & year==2006

replace regioncode="513335" if regioncode=="513335" & year==2006

replace regioncode="513336" if regioncode=="513336" & year==2006

replace regioncode="513337" if regioncode=="513337" & year==2006

replace regioncode="513338" if regioncode=="513338" & year==2006

replace regioncode="513401" if regioncode=="513401" & year==2006

replace regioncode="513422" if regioncode=="513422" & year==2006

replace regioncode="513423" if regioncode=="513423" & year==2006

replace regioncode="513424" if regioncode=="513424" & year==2006

replace regioncode="513425" if regioncode=="513425" & year==2006

replace regioncode="513426" if regioncode=="513426" & year==2006

replace regioncode="513427" if regioncode=="513427" & year==2006

replace regioncode="513428" if regioncode=="513428" & year==2006

replace regioncode="513429" if regioncode=="513429" & year==2006

replace regioncode="513430" if regioncode=="513430" & year==2006

replace regioncode="513431" if regioncode=="513431" & year==2006

replace regioncode="513432" if regioncode=="513432" & year==2006

replace regioncode="513433" if regioncode=="513433" & year==2006

replace regioncode="513434" if regioncode=="513434" & year==2006

replace regioncode="513435" if regioncode=="513435" & year==2006

replace regioncode="513436" if regioncode=="513436" & year==2006

replace regioncode="513437" if regioncode=="513437" & year==2006

replace regioncode="520102" if regioncode=="520102" & year==2006

replace regioncode="520103" if regioncode=="520103" & year==2006

replace regioncode="520111" if regioncode=="520111" & year==2006

replace regioncode="520112" if regioncode=="520112" & year==2006

replace regioncode="520113" if regioncode=="520113" & year==2006

replace regioncode="520114" if regioncode=="520114" & year==2006

replace regioncode="520121" if regioncode=="520121" & year==2006

replace regioncode="520122" if regioncode=="520122" & year==2006

replace regioncode="520123" if regioncode=="520123" & year==2006

replace regioncode="520181" if regioncode=="520181" & year==2006

replace regioncode="520201" if regioncode=="520201" & year==2006

replace regioncode="520222" if regioncode=="520222" & year==2006

replace regioncode="520203" if regioncode=="520203" & year==2006

replace regioncode="520221" if regioncode=="520221" & year==2006

replace regioncode="520302" if regioncode=="520302" & year==2006

replace regioncode="520321" if regioncode=="520321" & year==2006

replace regioncode="520322" if regioncode=="520322" & year==2006

replace regioncode="520323" if regioncode=="520323" & year==2006

replace regioncode="520324" if regioncode=="520324" & year==2006

replace regioncode="520325" if regioncode=="520325" & year==2006

replace regioncode="520326" if regioncode=="520326" & year==2006

replace regioncode="520327" if regioncode=="520327" & year==2006

replace regioncode="520328" if regioncode=="520328" & year==2006

replace regioncode="520329" if regioncode=="520329" & year==2006

replace regioncode="520330" if regioncode=="520330" & year==2006

replace regioncode="520381" if regioncode=="520381" & year==2006

replace regioncode="520382" if regioncode=="520382" & year==2006

replace regioncode="520402" if regioncode=="520402" & year==2006

replace regioncode="520421" if regioncode=="520421" & year==2006

replace regioncode="520422" if regioncode=="520422" & year==2006

replace regioncode="520423" if regioncode=="520423" & year==2006

replace regioncode="520424" if regioncode=="520424" & year==2006

replace regioncode="520425" if regioncode=="520425" & year==2006

replace regioncode="522201" if regioncode=="522201" & year==2006

replace regioncode="522222" if regioncode=="522222" & year==2006

replace regioncode="522223" if regioncode=="522223" & year==2006

replace regioncode="522224" if regioncode=="522224" & year==2006

replace regioncode="522225" if regioncode=="522225" & year==2006

replace regioncode="522226" if regioncode=="522226" & year==2006

replace regioncode="522227" if regioncode=="522227" & year==2006

replace regioncode="522228" if regioncode=="522228" & year==2006

replace regioncode="522229" if regioncode=="522229" & year==2006

replace regioncode="522230" if regioncode=="522230" & year==2006

replace regioncode="522301" if regioncode=="522301" & year==2006

replace regioncode="522322" if regioncode=="522322" & year==2006

replace regioncode="522323" if regioncode=="522323" & year==2006

replace regioncode="522324" if regioncode=="522324" & year==2006

replace regioncode="522325" if regioncode=="522325" & year==2006

replace regioncode="522326" if regioncode=="522326" & year==2006

replace regioncode="522327" if regioncode=="522327" & year==2006

replace regioncode="522328" if regioncode=="522328" & year==2006

replace regioncode="522401" if regioncode=="522401" & year==2006

replace regioncode="522422" if regioncode=="522422" & year==2006

replace regioncode="522423" if regioncode=="522423" & year==2006

replace regioncode="522424" if regioncode=="522424" & year==2006

replace regioncode="522425" if regioncode=="522425" & year==2006

replace regioncode="522426" if regioncode=="522426" & year==2006

replace regioncode="522427" if regioncode=="522427" & year==2006

replace regioncode="522428" if regioncode=="522428" & year==2006

replace regioncode="522601" if regioncode=="522601" & year==2006

replace regioncode="522622" if regioncode=="522622" & year==2006

replace regioncode="522623" if regioncode=="522623" & year==2006

replace regioncode="522624" if regioncode=="522624" & year==2006

replace regioncode="522625" if regioncode=="522625" & year==2006

replace regioncode="522626" if regioncode=="522626" & year==2006

replace regioncode="522627" if regioncode=="522627" & year==2006

replace regioncode="522628" if regioncode=="522628" & year==2006

replace regioncode="522629" if regioncode=="522629" & year==2006

replace regioncode="522630" if regioncode=="522630" & year==2006

replace regioncode="522631" if regioncode=="522631" & year==2006

replace regioncode="522632" if regioncode=="522632" & year==2006

replace regioncode="522633" if regioncode=="522633" & year==2006

replace regioncode="522634" if regioncode=="522634" & year==2006

replace regioncode="522635" if regioncode=="522635" & year==2006

replace regioncode="522636" if regioncode=="522636" & year==2006

replace regioncode="522701" if regioncode=="522701" & year==2006

replace regioncode="522702" if regioncode=="522702" & year==2006

replace regioncode="522722" if regioncode=="522722" & year==2006

replace regioncode="522723" if regioncode=="522723" & year==2006

replace regioncode="522725" if regioncode=="522725" & year==2006

replace regioncode="522726" if regioncode=="522726" & year==2006

replace regioncode="522727" if regioncode=="522727" & year==2006

replace regioncode="522728" if regioncode=="522728" & year==2006

replace regioncode="522729" if regioncode=="522729" & year==2006

replace regioncode="522730" if regioncode=="522730" & year==2006

replace regioncode="522731" if regioncode=="522731" & year==2006

replace regioncode="522732" if regioncode=="522732" & year==2006

replace regioncode="530102" if regioncode=="530102" & year==2006

replace regioncode="530103" if regioncode=="530103" & year==2006

replace regioncode="530111" if regioncode=="530111" & year==2006

replace regioncode="530112" if regioncode=="530112" & year==2006

replace regioncode="530113" if regioncode=="530113" & year==2006

replace regioncode="530121" if regioncode=="530121" & year==2006

replace regioncode="530122" if regioncode=="530122" & year==2006

replace regioncode="530124" if regioncode=="530124" & year==2006

replace regioncode="530125" if regioncode=="530125" & year==2006

replace regioncode="530126" if regioncode=="530126" & year==2006

replace regioncode="530127" if regioncode=="530127" & year==2006

replace regioncode="530128" if regioncode=="530128" & year==2006

replace regioncode="530129" if regioncode=="530129" & year==2006

replace regioncode="530181" if regioncode=="530181" & year==2006

replace regioncode="530302" if regioncode=="530302" & year==2006

replace regioncode="530321" if regioncode=="530321" & year==2006

replace regioncode="530322" if regioncode=="530322" & year==2006

replace regioncode="530323" if regioncode=="530323" & year==2006

replace regioncode="530324" if regioncode=="530324" & year==2006

replace regioncode="530325" if regioncode=="530325" & year==2006

replace regioncode="530326" if regioncode=="530326" & year==2006

replace regioncode="530328" if regioncode=="530328" & year==2006

replace regioncode="530381" if regioncode=="530381" & year==2006

replace regioncode="530402" if regioncode=="530402" & year==2006

replace regioncode="530421" if regioncode=="530421" & year==2006

replace regioncode="530422" if regioncode=="530422" & year==2006

replace regioncode="530423" if regioncode=="530423" & year==2006

replace regioncode="530424" if regioncode=="530424" & year==2006

replace regioncode="530425" if regioncode=="530425" & year==2006

replace regioncode="530426" if regioncode=="530426" & year==2006

replace regioncode="530427" if regioncode=="530427" & year==2006

replace regioncode="530428" if regioncode=="530428" & year==2006

replace regioncode="530502" if regioncode=="530502" & year==2006

replace regioncode="530521" if regioncode=="530521" & year==2006

replace regioncode="530522" if regioncode=="530522" & year==2006

replace regioncode="530523" if regioncode=="530523" & year==2006

replace regioncode="530524" if regioncode=="530524" & year==2006

replace regioncode="530602" if regioncode=="530602" & year==2006

replace regioncode="530621" if regioncode=="530621" & year==2006

replace regioncode="530622" if regioncode=="530622" & year==2006

replace regioncode="530623" if regioncode=="530623" & year==2006

replace regioncode="530624" if regioncode=="530624" & year==2006

replace regioncode="530625" if regioncode=="530625" & year==2006

replace regioncode="530626" if regioncode=="530626" & year==2006

replace regioncode="530627" if regioncode=="530627" & year==2006

replace regioncode="530628" if regioncode=="530628" & year==2006

replace regioncode="530629" if regioncode=="530629" & year==2006

replace regioncode="530630" if regioncode=="530630" & year==2006

replace regioncode="530721" if regioncode=="530721" & year==2006

replace regioncode="530722" if regioncode=="530722" & year==2006

replace regioncode="530723" if regioncode=="530723" & year==2006

replace regioncode="530724" if regioncode=="530724" & year==2006

replace regioncode="532701" if regioncode=="530802" & year==2006

replace regioncode="532722" if regioncode=="530821" & year==2006

replace regioncode="532723" if regioncode=="530822" & year==2006

replace regioncode="532724" if regioncode=="530823" & year==2006

replace regioncode="532725" if regioncode=="530824" & year==2006

replace regioncode="532726" if regioncode=="530825" & year==2006

replace regioncode="532727" if regioncode=="530826" & year==2006

replace regioncode="532728" if regioncode=="530827" & year==2006

replace regioncode="532729" if regioncode=="530828" & year==2006

replace regioncode="532730" if regioncode=="530829" & year==2006

replace regioncode="533521" if regioncode=="530902" & year==2006

replace regioncode="533522" if regioncode=="530921" & year==2006

replace regioncode="533523" if regioncode=="530922" & year==2006

replace regioncode="533524" if regioncode=="530923" & year==2006

replace regioncode="533525" if regioncode=="530924" & year==2006

replace regioncode="533526" if regioncode=="530925" & year==2006

replace regioncode="533527" if regioncode=="530926" & year==2006

replace regioncode="533528" if regioncode=="530927" & year==2006

replace regioncode="532301" if regioncode=="532301" & year==2006

replace regioncode="532322" if regioncode=="532322" & year==2006

replace regioncode="532323" if regioncode=="532323" & year==2006

replace regioncode="532324" if regioncode=="532324" & year==2006

replace regioncode="532325" if regioncode=="532325" & year==2006

replace regioncode="532326" if regioncode=="532326" & year==2006

replace regioncode="532327" if regioncode=="532327" & year==2006

replace regioncode="532328" if regioncode=="532328" & year==2006

replace regioncode="532329" if regioncode=="532329" & year==2006

replace regioncode="532331" if regioncode=="532331" & year==2006

replace regioncode="532501" if regioncode=="532501" & year==2006

replace regioncode="532502" if regioncode=="532502" & year==2006

replace regioncode="532522" if regioncode=="532522" & year==2006

replace regioncode="532523" if regioncode=="532523" & year==2006

replace regioncode="532524" if regioncode=="532524" & year==2006

replace regioncode="532525" if regioncode=="532525" & year==2006

replace regioncode="532526" if regioncode=="532526" & year==2006

replace regioncode="532527" if regioncode=="532527" & year==2006

replace regioncode="532528" if regioncode=="532528" & year==2006

replace regioncode="532529" if regioncode=="532529" & year==2006

replace regioncode="532530" if regioncode=="532530" & year==2006

replace regioncode="532531" if regioncode=="532531" & year==2006

replace regioncode="532532" if regioncode=="532532" & year==2006

replace regioncode="532621" if regioncode=="532621" & year==2006

replace regioncode="532622" if regioncode=="532622" & year==2006

replace regioncode="532623" if regioncode=="532623" & year==2006

replace regioncode="532624" if regioncode=="532624" & year==2006

replace regioncode="532625" if regioncode=="532625" & year==2006

replace regioncode="532626" if regioncode=="532626" & year==2006

replace regioncode="532627" if regioncode=="532627" & year==2006

replace regioncode="532628" if regioncode=="532628" & year==2006

replace regioncode="532801" if regioncode=="532801" & year==2006

replace regioncode="532822" if regioncode=="532822" & year==2006

replace regioncode="532823" if regioncode=="532823" & year==2006

replace regioncode="532901" if regioncode=="532901" & year==2006

replace regioncode="532922" if regioncode=="532922" & year==2006

replace regioncode="532923" if regioncode=="532923" & year==2006

replace regioncode="532924" if regioncode=="532924" & year==2006

replace regioncode="532925" if regioncode=="532925" & year==2006

replace regioncode="532926" if regioncode=="532926" & year==2006

replace regioncode="532927" if regioncode=="532927" & year==2006

replace regioncode="532928" if regioncode=="532928" & year==2006

replace regioncode="532929" if regioncode=="532929" & year==2006

replace regioncode="532930" if regioncode=="532930" & year==2006

replace regioncode="532931" if regioncode=="532931" & year==2006

replace regioncode="532932" if regioncode=="532932" & year==2006

replace regioncode="533102" if regioncode=="533102" & year==2006

replace regioncode="533103" if regioncode=="533103" & year==2006

replace regioncode="533122" if regioncode=="533122" & year==2006

replace regioncode="533123" if regioncode=="533123" & year==2006

replace regioncode="533124" if regioncode=="533124" & year==2006

replace regioncode="533321" if regioncode=="533321" & year==2006

replace regioncode="533323" if regioncode=="533323" & year==2006

replace regioncode="533324" if regioncode=="533324" & year==2006

replace regioncode="533325" if regioncode=="533325" & year==2006

replace regioncode="533421" if regioncode=="533421" & year==2006

replace regioncode="533422" if regioncode=="533422" & year==2006

replace regioncode="533423" if regioncode=="533423" & year==2006

replace regioncode="540102" if regioncode=="540102" & year==2006

replace regioncode="540121" if regioncode=="540121" & year==2006

replace regioncode="540122" if regioncode=="540122" & year==2006

replace regioncode="540123" if regioncode=="540123" & year==2006

replace regioncode="540124" if regioncode=="540124" & year==2006

replace regioncode="540125" if regioncode=="540125" & year==2006

replace regioncode="540126" if regioncode=="540126" & year==2006

replace regioncode="540127" if regioncode=="540127" & year==2006

replace regioncode="542121" if regioncode=="542121" & year==2006

replace regioncode="542122" if regioncode=="542122" & year==2006

replace regioncode="542123" if regioncode=="542123" & year==2006

replace regioncode="542124" if regioncode=="542124" & year==2006

replace regioncode="542125" if regioncode=="542125" & year==2006

replace regioncode="542126" if regioncode=="542126" & year==2006

replace regioncode="542127" if regioncode=="542127" & year==2006

replace regioncode="542128" if regioncode=="542128" & year==2006

replace regioncode="542129" if regioncode=="542129" & year==2006

replace regioncode="542132" if regioncode=="542132" & year==2006

replace regioncode="542133" if regioncode=="542133" & year==2006

replace regioncode="542221" if regioncode=="542221" & year==2006

replace regioncode="542223" if regioncode=="542223" & year==2006

replace regioncode="542224" if regioncode=="542224" & year==2006

replace regioncode="542225" if regioncode=="542225" & year==2006

replace regioncode="542227" if regioncode=="542227" & year==2006

replace regioncode="542228" if regioncode=="542228" & year==2006

replace regioncode="542229" if regioncode=="542229" & year==2006

replace regioncode="542231" if regioncode=="542231" & year==2006

replace regioncode="542232" if regioncode=="542232" & year==2006

replace regioncode="542233" if regioncode=="542233" & year==2006

replace regioncode="542301" if regioncode=="542301" & year==2006

replace regioncode="542322" if regioncode=="542322" & year==2006

replace regioncode="542323" if regioncode=="542323" & year==2006

replace regioncode="542324" if regioncode=="542324" & year==2006

replace regioncode="542325" if regioncode=="542325" & year==2006

replace regioncode="542326" if regioncode=="542326" & year==2006

replace regioncode="542327" if regioncode=="542327" & year==2006

replace regioncode="542328" if regioncode=="542328" & year==2006

replace regioncode="542329" if regioncode=="542329" & year==2006

replace regioncode="542330" if regioncode=="542330" & year==2006

replace regioncode="542331" if regioncode=="542331" & year==2006

replace regioncode="542332" if regioncode=="542332" & year==2006

replace regioncode="542334" if regioncode=="542334" & year==2006

replace regioncode="542336" if regioncode=="542336" & year==2006

replace regioncode="542338" if regioncode=="542338" & year==2006

replace regioncode="542421" if regioncode=="542421" & year==2006

replace regioncode="542422" if regioncode=="542422" & year==2006

replace regioncode="542423" if regioncode=="542423" & year==2006

replace regioncode="542424" if regioncode=="542424" & year==2006

replace regioncode="542425" if regioncode=="542425" & year==2006

replace regioncode="542426" if regioncode=="542426" & year==2006

replace regioncode="542427" if regioncode=="542427" & year==2006

replace regioncode="542429" if regioncode=="542429" & year==2006

replace regioncode="542430" if regioncode=="542430" & year==2006

replace regioncode="542523" if regioncode=="542523" & year==2006

replace regioncode="542621" if regioncode=="542621" & year==2006

replace regioncode="542622" if regioncode=="542622" & year==2006

replace regioncode="542623" if regioncode=="542623" & year==2006

replace regioncode="542625" if regioncode=="542625" & year==2006

replace regioncode="542626" if regioncode=="542626" & year==2006

replace regioncode="610102" if regioncode=="610102" & year==2006

replace regioncode="610103" if regioncode=="610103" & year==2006

replace regioncode="610104" if regioncode=="610104" & year==2006

replace regioncode="610111" if regioncode=="610111" & year==2006

replace regioncode="610112" if regioncode=="610112" & year==2006

replace regioncode="610113" if regioncode=="610113" & year==2006

replace regioncode="610114" if regioncode=="610114" & year==2006

replace regioncode="610115" if regioncode=="610115" & year==2006

replace regioncode="610116" if regioncode=="610116" & year==2006

replace regioncode="610122" if regioncode=="610122" & year==2006

replace regioncode="610124" if regioncode=="610124" & year==2006

replace regioncode="610125" if regioncode=="610125" & year==2006

replace regioncode="610126" if regioncode=="610126" & year==2006

replace regioncode="610202" if regioncode=="610202" & year==2006

replace regioncode="610203" if regioncode=="610203" & year==2006

replace regioncode="610204" if regioncode=="610204" & year==2006

replace regioncode="610222" if regioncode=="610222" & year==2006

replace regioncode="610302" if regioncode=="610302" & year==2006

replace regioncode="610303" if regioncode=="610303" & year==2006

replace regioncode="610304" if regioncode=="610304" & year==2006

replace regioncode="610322" if regioncode=="610322" & year==2006

replace regioncode="610323" if regioncode=="610323" & year==2006

replace regioncode="610324" if regioncode=="610324" & year==2006

replace regioncode="610326" if regioncode=="610326" & year==2006

replace regioncode="610327" if regioncode=="610327" & year==2006

replace regioncode="610328" if regioncode=="610328" & year==2006

replace regioncode="610329" if regioncode=="610329" & year==2006

replace regioncode="610330" if regioncode=="610330" & year==2006

replace regioncode="610331" if regioncode=="610331" & year==2006

replace regioncode="610402" if regioncode=="610402" & year==2006

replace regioncode="610403" if regioncode=="610403" & year==2006

replace regioncode="610404" if regioncode=="610404" & year==2006

replace regioncode="610422" if regioncode=="610422" & year==2006

replace regioncode="610423" if regioncode=="610423" & year==2006

replace regioncode="610424" if regioncode=="610424" & year==2006

replace regioncode="610425" if regioncode=="610425" & year==2006

replace regioncode="610426" if regioncode=="610426" & year==2006

replace regioncode="610427" if regioncode=="610427" & year==2006

replace regioncode="610428" if regioncode=="610428" & year==2006

replace regioncode="610429" if regioncode=="610429" & year==2006

replace regioncode="610430" if regioncode=="610430" & year==2006

replace regioncode="610431" if regioncode=="610431" & year==2006

replace regioncode="610481" if regioncode=="610481" & year==2006

replace regioncode="610502" if regioncode=="610502" & year==2006

replace regioncode="610521" if regioncode=="610521" & year==2006

replace regioncode="610522" if regioncode=="610522" & year==2006

replace regioncode="610523" if regioncode=="610523" & year==2006

replace regioncode="610524" if regioncode=="610524" & year==2006

replace regioncode="610525" if regioncode=="610525" & year==2006

replace regioncode="610526" if regioncode=="610526" & year==2006

replace regioncode="610527" if regioncode=="610527" & year==2006

replace regioncode="610528" if regioncode=="610528" & year==2006

replace regioncode="610581" if regioncode=="610581" & year==2006

replace regioncode="610582" if regioncode=="610582" & year==2006

replace regioncode="610602" if regioncode=="610602" & year==2006

replace regioncode="610621" if regioncode=="610621" & year==2006

replace regioncode="610622" if regioncode=="610622" & year==2006

replace regioncode="610623" if regioncode=="610623" & year==2006

replace regioncode="610624" if regioncode=="610624" & year==2006

replace regioncode="610625" if regioncode=="610625" & year==2006

replace regioncode="610626" if regioncode=="610626" & year==2006

replace regioncode="610627" if regioncode=="610627" & year==2006

replace regioncode="610628" if regioncode=="610628" & year==2006

replace regioncode="610629" if regioncode=="610629" & year==2006

replace regioncode="610630" if regioncode=="610630" & year==2006

replace regioncode="610631" if regioncode=="610631" & year==2006

replace regioncode="610632" if regioncode=="610632" & year==2006

replace regioncode="610702" if regioncode=="610702" & year==2006

replace regioncode="610721" if regioncode=="610721" & year==2006

replace regioncode="610722" if regioncode=="610722" & year==2006

replace regioncode="610723" if regioncode=="610723" & year==2006

replace regioncode="610724" if regioncode=="610724" & year==2006

replace regioncode="610725" if regioncode=="610725" & year==2006

replace regioncode="610726" if regioncode=="610726" & year==2006

replace regioncode="610727" if regioncode=="610727" & year==2006

replace regioncode="610728" if regioncode=="610728" & year==2006

replace regioncode="610729" if regioncode=="610729" & year==2006

replace regioncode="610730" if regioncode=="610730" & year==2006

replace regioncode="610802" if regioncode=="610802" & year==2006

replace regioncode="610821" if regioncode=="610821" & year==2006

replace regioncode="610822" if regioncode=="610822" & year==2006

replace regioncode="610823" if regioncode=="610823" & year==2006

replace regioncode="610824" if regioncode=="610824" & year==2006

replace regioncode="610825" if regioncode=="610825" & year==2006

replace regioncode="610826" if regioncode=="610826" & year==2006

replace regioncode="610827" if regioncode=="610827" & year==2006

replace regioncode="610828" if regioncode=="610828" & year==2006

replace regioncode="610829" if regioncode=="610829" & year==2006

replace regioncode="610830" if regioncode=="610830" & year==2006

replace regioncode="610831" if regioncode=="610831" & year==2006

replace regioncode="610902" if regioncode=="610902" & year==2006

replace regioncode="610921" if regioncode=="610921" & year==2006

replace regioncode="610922" if regioncode=="610922" & year==2006

replace regioncode="610923" if regioncode=="610923" & year==2006

replace regioncode="610924" if regioncode=="610924" & year==2006

replace regioncode="610925" if regioncode=="610925" & year==2006

replace regioncode="610926" if regioncode=="610926" & year==2006

replace regioncode="610927" if regioncode=="610927" & year==2006

replace regioncode="610928" if regioncode=="610928" & year==2006

replace regioncode="610929" if regioncode=="610929" & year==2006

replace regioncode="611002" if regioncode=="611002" & year==2006

replace regioncode="611021" if regioncode=="611021" & year==2006

replace regioncode="611022" if regioncode=="611022" & year==2006

replace regioncode="611023" if regioncode=="611023" & year==2006

replace regioncode="611024" if regioncode=="611024" & year==2006

replace regioncode="611025" if regioncode=="611025" & year==2006

replace regioncode="611026" if regioncode=="611026" & year==2006

replace regioncode="620102" if regioncode=="620102" & year==2006

replace regioncode="620103" if regioncode=="620103" & year==2006

replace regioncode="620104" if regioncode=="620104" & year==2006

replace regioncode="620105" if regioncode=="620105" & year==2006

replace regioncode="620111" if regioncode=="620111" & year==2006

replace regioncode="620121" if regioncode=="620121" & year==2006

replace regioncode="620122" if regioncode=="620122" & year==2006

replace regioncode="620123" if regioncode=="620123" & year==2006

replace regioncode="620201" if regioncode=="620201" & year==2006

replace regioncode="620302" if regioncode=="620302" & year==2006

replace regioncode="620321" if regioncode=="620321" & year==2006

replace regioncode="620402" if regioncode=="620402" & year==2006

replace regioncode="620403" if regioncode=="620403" & year==2006

replace regioncode="620421" if regioncode=="620421" & year==2006

replace regioncode="620422" if regioncode=="620422" & year==2006

replace regioncode="620423" if regioncode=="620423" & year==2006

replace regioncode="620502" if regioncode=="620502" & year==2006

replace regioncode="620503" if regioncode=="620503" & year==2006

replace regioncode="620521" if regioncode=="620521" & year==2006

replace regioncode="620522" if regioncode=="620522" & year==2006

replace regioncode="620523" if regioncode=="620523" & year==2006

replace regioncode="620524" if regioncode=="620524" & year==2006

replace regioncode="620525" if regioncode=="620525" & year==2006

replace regioncode="622301" if regioncode=="620602" & year==2006

replace regioncode="622322" if regioncode=="620621" & year==2006

replace regioncode="622323" if regioncode=="620622" & year==2006

replace regioncode="622326" if regioncode=="620623" & year==2006

replace regioncode="622201" if regioncode=="620702" & year==2006

replace regioncode="622222" if regioncode=="620721" & year==2006

replace regioncode="622223" if regioncode=="620722" & year==2006

replace regioncode="622224" if regioncode=="620723" & year==2006

replace regioncode="622225" if regioncode=="620724" & year==2006

replace regioncode="622226" if regioncode=="620725" & year==2006

replace regioncode="622701" if regioncode=="620802" & year==2006

replace regioncode="622722" if regioncode=="620821" & year==2006

replace regioncode="622723" if regioncode=="620822" & year==2006

replace regioncode="622724" if regioncode=="620823" & year==2006

replace regioncode="622725" if regioncode=="620824" & year==2006

replace regioncode="622726" if regioncode=="620825" & year==2006

replace regioncode="622727" if regioncode=="620826" & year==2006

replace regioncode="622102" if regioncode=="620902" & year==2006

replace regioncode="622123" if regioncode=="620921" & year==2006

replace regioncode="622126" if regioncode=="620922" & year==2006

replace regioncode="622124" if regioncode=="620923" & year==2006

replace regioncode="622125" if regioncode=="620924" & year==2006

replace regioncode="622101" if regioncode=="620981" & year==2006

replace regioncode="622103" if regioncode=="620982" & year==2006

replace regioncode="622801" if regioncode=="621002" & year==2006

replace regioncode="622821" if regioncode=="621021" & year==2006

replace regioncode="622822" if regioncode=="621022" & year==2006

replace regioncode="622823" if regioncode=="621023" & year==2006

replace regioncode="622824" if regioncode=="621024" & year==2006

replace regioncode="622825" if regioncode=="621025" & year==2006

replace regioncode="622826" if regioncode=="621026" & year==2006

replace regioncode="622827" if regioncode=="621027" & year==2006

replace regioncode="622421" if regioncode=="621102" & year==2006

replace regioncode="622424" if regioncode=="621121" & year==2006

replace regioncode="622425" if regioncode=="621122" & year==2006

replace regioncode="622426" if regioncode=="621123" & year==2006

replace regioncode="622427" if regioncode=="621124" & year==2006

replace regioncode="622428" if regioncode=="621125" & year==2006

replace regioncode="622429" if regioncode=="621126" & year==2006

replace regioncode="622621" if regioncode=="621202" & year==2006

replace regioncode="622624" if regioncode=="621221" & year==2006

replace regioncode="622626" if regioncode=="621222" & year==2006

replace regioncode="622623" if regioncode=="621223" & year==2006

replace regioncode="622625" if regioncode=="621224" & year==2006

replace regioncode="622627" if regioncode=="621225" & year==2006

replace regioncode="622628" if regioncode=="621226" & year==2006

replace regioncode="622630" if regioncode=="621227" & year==2006

replace regioncode="622629" if regioncode=="621228" & year==2006

replace regioncode="622901" if regioncode=="622901" & year==2006

replace regioncode="622921" if regioncode=="622921" & year==2006

replace regioncode="622922" if regioncode=="622922" & year==2006

replace regioncode="622923" if regioncode=="622923" & year==2006

replace regioncode="622924" if regioncode=="622924" & year==2006

replace regioncode="622925" if regioncode=="622925" & year==2006

replace regioncode="622926" if regioncode=="622926" & year==2006

replace regioncode="622927" if regioncode=="622927" & year==2006

replace regioncode="623001" if regioncode=="623001" & year==2006

replace regioncode="623021" if regioncode=="623021" & year==2006

replace regioncode="623022" if regioncode=="623022" & year==2006

replace regioncode="623023" if regioncode=="623023" & year==2006

replace regioncode="623024" if regioncode=="623024" & year==2006

replace regioncode="623025" if regioncode=="623025" & year==2006

replace regioncode="623026" if regioncode=="623026" & year==2006

replace regioncode="623027" if regioncode=="623027" & year==2006

replace regioncode="630102" if regioncode=="630102" & year==2006

replace regioncode="630103" if regioncode=="630103" & year==2006

replace regioncode="630104" if regioncode=="630104" & year==2006

replace regioncode="630105" if regioncode=="630105" & year==2006

replace regioncode="630121" if regioncode=="630121" & year==2006

replace regioncode="630122" if regioncode=="630122" & year==2006

replace regioncode="630123" if regioncode=="630123" & year==2006

replace regioncode="632121" if regioncode=="632121" & year==2006

replace regioncode="632122" if regioncode=="632122" & year==2006

replace regioncode="632123" if regioncode=="632123" & year==2006

replace regioncode="632126" if regioncode=="632126" & year==2006

replace regioncode="632127" if regioncode=="632127" & year==2006

replace regioncode="632128" if regioncode=="632128" & year==2006

replace regioncode="632221" if regioncode=="632221" & year==2006

replace regioncode="632222" if regioncode=="632222" & year==2006

replace regioncode="632223" if regioncode=="632223" & year==2006

replace regioncode="632224" if regioncode=="632224" & year==2006

replace regioncode="632321" if regioncode=="632321" & year==2006

replace regioncode="632322" if regioncode=="632322" & year==2006

replace regioncode="632521" if regioncode=="632521" & year==2006

replace regioncode="632522" if regioncode=="632522" & year==2006

replace regioncode="632523" if regioncode=="632523" & year==2006

replace regioncode="632524" if regioncode=="632524" & year==2006

replace regioncode="632525" if regioncode=="632525" & year==2006

replace regioncode="632621" if regioncode=="632621" & year==2006

replace regioncode="632622" if regioncode=="632622" & year==2006

replace regioncode="632623" if regioncode=="632623" & year==2006

replace regioncode="632624" if regioncode=="632624" & year==2006

replace regioncode="632625" if regioncode=="632625" & year==2006

replace regioncode="632626" if regioncode=="632626" & year==2006

replace regioncode="632721" if regioncode=="632721" & year==2006

replace regioncode="632723" if regioncode=="632723" & year==2006

replace regioncode="632724" if regioncode=="632724" & year==2006

replace regioncode="632725" if regioncode=="632725" & year==2006

replace regioncode="632726" if regioncode=="632726" & year==2006

replace regioncode="632801" if regioncode=="632801" & year==2006

replace regioncode="632802" if regioncode=="632802" & year==2006

replace regioncode="632821" if regioncode=="632821" & year==2006

replace regioncode="632822" if regioncode=="632822" & year==2006

replace regioncode="640104" if regioncode=="640104" & year==2006

replace regioncode="640105" if regioncode=="640105" & year==2006

replace regioncode="640106" if regioncode=="640106" & year==2006

replace regioncode="640121" if regioncode=="640121" & year==2006

replace regioncode="640122" if regioncode=="640122" & year==2006

replace regioncode="640181" if regioncode=="640181" & year==2006

replace regioncode="640202" if regioncode=="640202" & year==2006

replace regioncode="640203" if regioncode=="640205" & year==2006

replace regioncode="640221" if regioncode=="640221" & year==2006

replace regioncode="640302" if regioncode=="640302" & year==2006

replace regioncode="640321" if regioncode=="640502" & year==2006

replace regioncode="640322" if regioncode=="640521" & year==2006

replace regioncode="640323" if regioncode=="640323" & year==2006

replace regioncode="640324" if regioncode=="640324" & year==2006

replace regioncode="640381" if regioncode=="640381" & year==2006

replace regioncode="640402" if regioncode=="640402" & year==2006

replace regioncode="640421" if regioncode=="640522" & year==2006

replace regioncode="640422" if regioncode=="640422" & year==2006

replace regioncode="640423" if regioncode=="640423" & year==2006

replace regioncode="640424" if regioncode=="640424" & year==2006

replace regioncode="640425" if regioncode=="640425" & year==2006

replace regioncode="650102" if regioncode=="650102" & year==2006

replace regioncode="650103" if regioncode=="650103" & year==2006

replace regioncode="650104" if regioncode=="650104" & year==2006

replace regioncode="650105" if regioncode=="650105" & year==2006

replace regioncode="650106" if regioncode=="650106" & year==2006

replace regioncode="650107" if regioncode=="650107" & year==2006

replace regioncode="650108" if regioncode=="650108" & year==2006

replace regioncode="650121" if regioncode=="650121" & year==2006

replace regioncode="650202" if regioncode=="650202" & year==2006

replace regioncode="650203" if regioncode=="650203" & year==2006

replace regioncode="650204" if regioncode=="650204" & year==2006

replace regioncode="650205" if regioncode=="650205" & year==2006

replace regioncode="652101" if regioncode=="652101" & year==2006

replace regioncode="652122" if regioncode=="652122" & year==2006

replace regioncode="652123" if regioncode=="652123" & year==2006

replace regioncode="652201" if regioncode=="652201" & year==2006

replace regioncode="652222" if regioncode=="652222" & year==2006

replace regioncode="652223" if regioncode=="652223" & year==2006

replace regioncode="652301" if regioncode=="652301" & year==2006

replace regioncode="652302" if regioncode=="652302" & year==2006

replace regioncode="652303" if regioncode=="652303" & year==2006

replace regioncode="652323" if regioncode=="652323" & year==2006

replace regioncode="652324" if regioncode=="652324" & year==2006

replace regioncode="652325" if regioncode=="652325" & year==2006

replace regioncode="652327" if regioncode=="652327" & year==2006

replace regioncode="652328" if regioncode=="652328" & year==2006

replace regioncode="652701" if regioncode=="652701" & year==2006

replace regioncode="652722" if regioncode=="652722" & year==2006

replace regioncode="652723" if regioncode=="652723" & year==2006

replace regioncode="652801" if regioncode=="652801" & year==2006

replace regioncode="652822" if regioncode=="652822" & year==2006

replace regioncode="652823" if regioncode=="652823" & year==2006

replace regioncode="652824" if regioncode=="652824" & year==2006

replace regioncode="652825" if regioncode=="652825" & year==2006

replace regioncode="652826" if regioncode=="652826" & year==2006

replace regioncode="652827" if regioncode=="652827" & year==2006

replace regioncode="652828" if regioncode=="652828" & year==2006

replace regioncode="652829" if regioncode=="652829" & year==2006

replace regioncode="652901" if regioncode=="652901" & year==2006

replace regioncode="652922" if regioncode=="652922" & year==2006

replace regioncode="652923" if regioncode=="652923" & year==2006

replace regioncode="652924" if regioncode=="652924" & year==2006

replace regioncode="652925" if regioncode=="652925" & year==2006

replace regioncode="652926" if regioncode=="652926" & year==2006

replace regioncode="652928" if regioncode=="652928" & year==2006

replace regioncode="652929" if regioncode=="652929" & year==2006

replace regioncode="653001" if regioncode=="653001" & year==2006

replace regioncode="653022" if regioncode=="653022" & year==2006

replace regioncode="653023" if regioncode=="653023" & year==2006

replace regioncode="653024" if regioncode=="653024" & year==2006

replace regioncode="653101" if regioncode=="653101" & year==2006

replace regioncode="653121" if regioncode=="653121" & year==2006

replace regioncode="653122" if regioncode=="653122" & year==2006

replace regioncode="653123" if regioncode=="653123" & year==2006

replace regioncode="653124" if regioncode=="653124" & year==2006

replace regioncode="653125" if regioncode=="653125" & year==2006

replace regioncode="653126" if regioncode=="653126" & year==2006

replace regioncode="653127" if regioncode=="653127" & year==2006

replace regioncode="653128" if regioncode=="653128" & year==2006

replace regioncode="653129" if regioncode=="653129" & year==2006

replace regioncode="653130" if regioncode=="653130" & year==2006

replace regioncode="653131" if regioncode=="653131" & year==2006

replace regioncode="653201" if regioncode=="653201" & year==2006

replace regioncode="653221" if regioncode=="653221" & year==2006

replace regioncode="653222" if regioncode=="653222" & year==2006

replace regioncode="653223" if regioncode=="653223" & year==2006

replace regioncode="653224" if regioncode=="653224" & year==2006

replace regioncode="653225" if regioncode=="653225" & year==2006

replace regioncode="653226" if regioncode=="653226" & year==2006

replace regioncode="653227" if regioncode=="653227" & year==2006

replace regioncode="654002" if regioncode=="654002" & year==2006

replace regioncode="654003" if regioncode=="654003" & year==2006

replace regioncode="654021" if regioncode=="654021" & year==2006

replace regioncode="654022" if regioncode=="654022" & year==2006

replace regioncode="654023" if regioncode=="654023" & year==2006

replace regioncode="654024" if regioncode=="654024" & year==2006

replace regioncode="654025" if regioncode=="654025" & year==2006

replace regioncode="654026" if regioncode=="654026" & year==2006

replace regioncode="654027" if regioncode=="654027" & year==2006

replace regioncode="654028" if regioncode=="654028" & year==2006

replace regioncode="654201" if regioncode=="654201" & year==2006

replace regioncode="654202" if regioncode=="654202" & year==2006

replace regioncode="654221" if regioncode=="654221" & year==2006

replace regioncode="654223" if regioncode=="654223" & year==2006

replace regioncode="654224" if regioncode=="654224" & year==2006

replace regioncode="654225" if regioncode=="654225" & year==2006

replace regioncode="654226" if regioncode=="654226" & year==2006

replace regioncode="654301" if regioncode=="654301" & year==2006

replace regioncode="654321" if regioncode=="654321" & year==2006

replace regioncode="654322" if regioncode=="654322" & year==2006

replace regioncode="654323" if regioncode=="654323" & year==2006

replace regioncode="654324" if regioncode=="654324" & year==2006

replace regioncode="654325" if regioncode=="654325" & year==2006

replace regioncode="654326" if regioncode=="654326" & year==2006

replace regioncode="659001" if regioncode=="659001" & year==2006

replace regioncode="110101" if regioncode=="110101" & year==2007

replace regioncode="110102" if regioncode=="110102" & year==2007

replace regioncode="110103" if regioncode=="110103" & year==2007

replace regioncode="110104" if regioncode=="110104" & year==2007

replace regioncode="110105" if regioncode=="110105" & year==2007

replace regioncode="110106" if regioncode=="110106" & year==2007

replace regioncode="110107" if regioncode=="110107" & year==2007

replace regioncode="110108" if regioncode=="110108" & year==2007

replace regioncode="110109" if regioncode=="110109" & year==2007

replace regioncode="110111" if regioncode=="110111" & year==2007

replace regioncode="110112" if regioncode=="110112" & year==2007

replace regioncode="110113" if regioncode=="110113" & year==2007

replace regioncode="110114" if regioncode=="110114" & year==2007

replace regioncode="110115" if regioncode=="110115" & year==2007

replace regioncode="110117" if regioncode=="110117" & year==2007

replace regioncode="110116" if regioncode=="110116" & year==2007

replace regioncode="110228" if regioncode=="110228" & year==2007

replace regioncode="110229" if regioncode=="110229" & year==2007

replace regioncode="120101" if regioncode=="120101" & year==2007

replace regioncode="120102" if regioncode=="120102" & year==2007

replace regioncode="120103" if regioncode=="120103" & year==2007

replace regioncode="120104" if regioncode=="120104" & year==2007

replace regioncode="120105" if regioncode=="120105" & year==2007

replace regioncode="120106" if regioncode=="120106" & year==2007

replace regioncode="120107" if regioncode=="120107" & year==2007

replace regioncode="120108" if regioncode=="120108" & year==2007

replace regioncode="120109" if regioncode=="120109" & year==2007

replace regioncode="120110" if regioncode=="120110" & year==2007

replace regioncode="120111" if regioncode=="120111" & year==2007

replace regioncode="120112" if regioncode=="120112" & year==2007

replace regioncode="120113" if regioncode=="120113" & year==2007

replace regioncode="120114" if regioncode=="120114" & year==2007

replace regioncode="120115" if regioncode=="120115" & year==2007

replace regioncode="120221" if regioncode=="120221" & year==2007

replace regioncode="120223" if regioncode=="120223" & year==2007

replace regioncode="120225" if regioncode=="120225" & year==2007

replace regioncode="130102" if regioncode=="130102" & year==2007

replace regioncode="130103" if regioncode=="130103" & year==2007

replace regioncode="130104" if regioncode=="130104" & year==2007

replace regioncode="130105" if regioncode=="130105" & year==2007

replace regioncode="130107" if regioncode=="130107" & year==2007

replace regioncode="130108" if regioncode=="130108" & year==2007

replace regioncode="130121" if regioncode=="130121" & year==2007

replace regioncode="130123" if regioncode=="130123" & year==2007

replace regioncode="130124" if regioncode=="130124" & year==2007

replace regioncode="130125" if regioncode=="130125" & year==2007

replace regioncode="130126" if regioncode=="130126" & year==2007

replace regioncode="130127" if regioncode=="130127" & year==2007

replace regioncode="130128" if regioncode=="130128" & year==2007

replace regioncode="130129" if regioncode=="130129" & year==2007

replace regioncode="130130" if regioncode=="130130" & year==2007

replace regioncode="130131" if regioncode=="130131" & year==2007

replace regioncode="130132" if regioncode=="130132" & year==2007

replace regioncode="130133" if regioncode=="130133" & year==2007

replace regioncode="130181" if regioncode=="130181" & year==2007

replace regioncode="130182" if regioncode=="130182" & year==2007

replace regioncode="130183" if regioncode=="130183" & year==2007

replace regioncode="130184" if regioncode=="130184" & year==2007

replace regioncode="130185" if regioncode=="130185" & year==2007

replace regioncode="130202" if regioncode=="130202" & year==2007

replace regioncode="130203" if regioncode=="130203" & year==2007

replace regioncode="130204" if regioncode=="130204" & year==2007

replace regioncode="130205" if regioncode=="130205" & year==2007

replace regioncode="130208" if regioncode=="130208" & year==2007

replace regioncode="130223" if regioncode=="130223" & year==2007

replace regioncode="130224" if regioncode=="130224" & year==2007

replace regioncode="130225" if regioncode=="130225" & year==2007

replace regioncode="130227" if regioncode=="130227" & year==2007

replace regioncode="130229" if regioncode=="130229" & year==2007

replace regioncode="130230" if regioncode=="130230" & year==2007

replace regioncode="130281" if regioncode=="130281" & year==2007

replace regioncode="130207" if regioncode=="130207" & year==2007

replace regioncode="130283" if regioncode=="130283" & year==2007

replace regioncode="130302" if regioncode=="130302" & year==2007

replace regioncode="130303" if regioncode=="130303" & year==2007

replace regioncode="130304" if regioncode=="130304" & year==2007

replace regioncode="130321" if regioncode=="130321" & year==2007

replace regioncode="130322" if regioncode=="130322" & year==2007

replace regioncode="130323" if regioncode=="130323" & year==2007

replace regioncode="130324" if regioncode=="130324" & year==2007

replace regioncode="130402" if regioncode=="130402" & year==2007

replace regioncode="130403" if regioncode=="130403" & year==2007

replace regioncode="130404" if regioncode=="130404" & year==2007

replace regioncode="130406" if regioncode=="130406" & year==2007

replace regioncode="130421" if regioncode=="130421" & year==2007

replace regioncode="130423" if regioncode=="130423" & year==2007

replace regioncode="130424" if regioncode=="130424" & year==2007

replace regioncode="130425" if regioncode=="130425" & year==2007

replace regioncode="130426" if regioncode=="130426" & year==2007

replace regioncode="130427" if regioncode=="130427" & year==2007

replace regioncode="130428" if regioncode=="130428" & year==2007

replace regioncode="130429" if regioncode=="130429" & year==2007

replace regioncode="130430" if regioncode=="130430" & year==2007

replace regioncode="130431" if regioncode=="130431" & year==2007

replace regioncode="130432" if regioncode=="130432" & year==2007

replace regioncode="130433" if regioncode=="130433" & year==2007

replace regioncode="130434" if regioncode=="130434" & year==2007

replace regioncode="130435" if regioncode=="130435" & year==2007

replace regioncode="130481" if regioncode=="130481" & year==2007

replace regioncode="130502" if regioncode=="130502" & year==2007

replace regioncode="130503" if regioncode=="130503" & year==2007

replace regioncode="130521" if regioncode=="130521" & year==2007

replace regioncode="130522" if regioncode=="130522" & year==2007

replace regioncode="130523" if regioncode=="130523" & year==2007

replace regioncode="130524" if regioncode=="130524" & year==2007

replace regioncode="130525" if regioncode=="130525" & year==2007

replace regioncode="130526" if regioncode=="130526" & year==2007

replace regioncode="130527" if regioncode=="130527" & year==2007

replace regioncode="130528" if regioncode=="130528" & year==2007

replace regioncode="130529" if regioncode=="130529" & year==2007

replace regioncode="130530" if regioncode=="130530" & year==2007

replace regioncode="130531" if regioncode=="130531" & year==2007

replace regioncode="130532" if regioncode=="130532" & year==2007

replace regioncode="130533" if regioncode=="130533" & year==2007

replace regioncode="130534" if regioncode=="130534" & year==2007

replace regioncode="130535" if regioncode=="130535" & year==2007

replace regioncode="130581" if regioncode=="130581" & year==2007

replace regioncode="130582" if regioncode=="130582" & year==2007

replace regioncode="130602" if regioncode=="130602" & year==2007

replace regioncode="130603" if regioncode=="130603" & year==2007

replace regioncode="130604" if regioncode=="130604" & year==2007

replace regioncode="130621" if regioncode=="130621" & year==2007

replace regioncode="130622" if regioncode=="130622" & year==2007

replace regioncode="130623" if regioncode=="130623" & year==2007

replace regioncode="130624" if regioncode=="130624" & year==2007

replace regioncode="130625" if regioncode=="130625" & year==2007

replace regioncode="130626" if regioncode=="130626" & year==2007

replace regioncode="130627" if regioncode=="130627" & year==2007

replace regioncode="130628" if regioncode=="130628" & year==2007

replace regioncode="130629" if regioncode=="130629" & year==2007

replace regioncode="130630" if regioncode=="130630" & year==2007

replace regioncode="130631" if regioncode=="130631" & year==2007

replace regioncode="130632" if regioncode=="130632" & year==2007

replace regioncode="130633" if regioncode=="130633" & year==2007

replace regioncode="130634" if regioncode=="130634" & year==2007

replace regioncode="130635" if regioncode=="130635" & year==2007

replace regioncode="130636" if regioncode=="130636" & year==2007

replace regioncode="130637" if regioncode=="130637" & year==2007

replace regioncode="130638" if regioncode=="130638" & year==2007

replace regioncode="130681" if regioncode=="130681" & year==2007

replace regioncode="130682" if regioncode=="130682" & year==2007

replace regioncode="130683" if regioncode=="130683" & year==2007

replace regioncode="130684" if regioncode=="130684" & year==2007

replace regioncode="130702" if regioncode=="130702" & year==2007

replace regioncode="130703" if regioncode=="130703" & year==2007

replace regioncode="130705" if regioncode=="130705" & year==2007

replace regioncode="130706" if regioncode=="130706" & year==2007

replace regioncode="130721" if regioncode=="130721" & year==2007

replace regioncode="130722" if regioncode=="130722" & year==2007

replace regioncode="130723" if regioncode=="130723" & year==2007

replace regioncode="130724" if regioncode=="130724" & year==2007

replace regioncode="130725" if regioncode=="130725" & year==2007

replace regioncode="130726" if regioncode=="130726" & year==2007

replace regioncode="130727" if regioncode=="130727" & year==2007

replace regioncode="130728" if regioncode=="130728" & year==2007

replace regioncode="130729" if regioncode=="130729" & year==2007

replace regioncode="130730" if regioncode=="130730" & year==2007

replace regioncode="130731" if regioncode=="130731" & year==2007

replace regioncode="130732" if regioncode=="130732" & year==2007

replace regioncode="130733" if regioncode=="130733" & year==2007

replace regioncode="130802" if regioncode=="130802" & year==2007

replace regioncode="130803" if regioncode=="130803" & year==2007

replace regioncode="130804" if regioncode=="130804" & year==2007

replace regioncode="130821" if regioncode=="130821" & year==2007

replace regioncode="130822" if regioncode=="130822" & year==2007

replace regioncode="130823" if regioncode=="130823" & year==2007

replace regioncode="130824" if regioncode=="130824" & year==2007

replace regioncode="130825" if regioncode=="130825" & year==2007

replace regioncode="130826" if regioncode=="130826" & year==2007

replace regioncode="130827" if regioncode=="130827" & year==2007

replace regioncode="130828" if regioncode=="130828" & year==2007

replace regioncode="130902" if regioncode=="130902" & year==2007

replace regioncode="130903" if regioncode=="130903" & year==2007

replace regioncode="130921" if regioncode=="130921" & year==2007

replace regioncode="130922" if regioncode=="130922" & year==2007

replace regioncode="130923" if regioncode=="130923" & year==2007

replace regioncode="130924" if regioncode=="130924" & year==2007

replace regioncode="130925" if regioncode=="130925" & year==2007

replace regioncode="130926" if regioncode=="130926" & year==2007

replace regioncode="130927" if regioncode=="130927" & year==2007

replace regioncode="130928" if regioncode=="130928" & year==2007

replace regioncode="130929" if regioncode=="130929" & year==2007

replace regioncode="130930" if regioncode=="130930" & year==2007

replace regioncode="130981" if regioncode=="130981" & year==2007

replace regioncode="130982" if regioncode=="130982" & year==2007

replace regioncode="130983" if regioncode=="130983" & year==2007

replace regioncode="130984" if regioncode=="130984" & year==2007

replace regioncode="131002" if regioncode=="131002" & year==2007

replace regioncode="131003" if regioncode=="131003" & year==2007

replace regioncode="131022" if regioncode=="131022" & year==2007

replace regioncode="131023" if regioncode=="131023" & year==2007

replace regioncode="131024" if regioncode=="131024" & year==2007

replace regioncode="131025" if regioncode=="131025" & year==2007

replace regioncode="131026" if regioncode=="131026" & year==2007

replace regioncode="131028" if regioncode=="131028" & year==2007

replace regioncode="131081" if regioncode=="131081" & year==2007

replace regioncode="131082" if regioncode=="131082" & year==2007

replace regioncode="131102" if regioncode=="131102" & year==2007

replace regioncode="131121" if regioncode=="131121" & year==2007

replace regioncode="131122" if regioncode=="131122" & year==2007

replace regioncode="131123" if regioncode=="131123" & year==2007

replace regioncode="131124" if regioncode=="131124" & year==2007

replace regioncode="131125" if regioncode=="131125" & year==2007

replace regioncode="131126" if regioncode=="131126" & year==2007

replace regioncode="131127" if regioncode=="131127" & year==2007

replace regioncode="131128" if regioncode=="131128" & year==2007

replace regioncode="131181" if regioncode=="131181" & year==2007

replace regioncode="131182" if regioncode=="131182" & year==2007

replace regioncode="140105" if regioncode=="140105" & year==2007

replace regioncode="140106" if regioncode=="140106" & year==2007

replace regioncode="140107" if regioncode=="140107" & year==2007

replace regioncode="140108" if regioncode=="140108" & year==2007

replace regioncode="140109" if regioncode=="140109" & year==2007

replace regioncode="140110" if regioncode=="140110" & year==2007

replace regioncode="140121" if regioncode=="140121" & year==2007

replace regioncode="140122" if regioncode=="140122" & year==2007

replace regioncode="140123" if regioncode=="140123" & year==2007

replace regioncode="140181" if regioncode=="140181" & year==2007

replace regioncode="140202" if regioncode=="140202" & year==2007

replace regioncode="140203" if regioncode=="140203" & year==2007

replace regioncode="140211" if regioncode=="140211" & year==2007

replace regioncode="140212" if regioncode=="140212" & year==2007

replace regioncode="140221" if regioncode=="140221" & year==2007

replace regioncode="140222" if regioncode=="140222" & year==2007

replace regioncode="140223" if regioncode=="140223" & year==2007

replace regioncode="140224" if regioncode=="140224" & year==2007

replace regioncode="140225" if regioncode=="140225" & year==2007

replace regioncode="140226" if regioncode=="140226" & year==2007

replace regioncode="140227" if regioncode=="140227" & year==2007

replace regioncode="140302" if regioncode=="140302" & year==2007

replace regioncode="140303" if regioncode=="140303" & year==2007

replace regioncode="140311" if regioncode=="140311" & year==2007

replace regioncode="140321" if regioncode=="140321" & year==2007

replace regioncode="140322" if regioncode=="140322" & year==2007

replace regioncode="140402" if regioncode=="140402" & year==2007

replace regioncode="140411" if regioncode=="140411" & year==2007

replace regioncode="140421" if regioncode=="140421" & year==2007

replace regioncode="140423" if regioncode=="140423" & year==2007

replace regioncode="140424" if regioncode=="140424" & year==2007

replace regioncode="140425" if regioncode=="140425" & year==2007

replace regioncode="140426" if regioncode=="140426" & year==2007

replace regioncode="140427" if regioncode=="140427" & year==2007

replace regioncode="140428" if regioncode=="140428" & year==2007

replace regioncode="140429" if regioncode=="140429" & year==2007

replace regioncode="140430" if regioncode=="140430" & year==2007

replace regioncode="140431" if regioncode=="140431" & year==2007

replace regioncode="140481" if regioncode=="140481" & year==2007

replace regioncode="140502" if regioncode=="140502" & year==2007

replace regioncode="140521" if regioncode=="140521" & year==2007

replace regioncode="140522" if regioncode=="140522" & year==2007

replace regioncode="140524" if regioncode=="140524" & year==2007

replace regioncode="140525" if regioncode=="140525" & year==2007

replace regioncode="140581" if regioncode=="140581" & year==2007

replace regioncode="140602" if regioncode=="140602" & year==2007

replace regioncode="140603" if regioncode=="140603" & year==2007

replace regioncode="140621" if regioncode=="140621" & year==2007

replace regioncode="140622" if regioncode=="140622" & year==2007

replace regioncode="140623" if regioncode=="140623" & year==2007

replace regioncode="140624" if regioncode=="140624" & year==2007

replace regioncode="140902" if regioncode=="140902" & year==2007

replace regioncode="140981" if regioncode=="140981" & year==2007

replace regioncode="140921" if regioncode=="140921" & year==2007

replace regioncode="140922" if regioncode=="140922" & year==2007

replace regioncode="140923" if regioncode=="140923" & year==2007

replace regioncode="140924" if regioncode=="140924" & year==2007

replace regioncode="140925" if regioncode=="140925" & year==2007

replace regioncode="140926" if regioncode=="140926" & year==2007

replace regioncode="140927" if regioncode=="140927" & year==2007

replace regioncode="140928" if regioncode=="140928" & year==2007

replace regioncode="140929" if regioncode=="140929" & year==2007

replace regioncode="140930" if regioncode=="140930" & year==2007

replace regioncode="140931" if regioncode=="140931" & year==2007

replace regioncode="140932" if regioncode=="140932" & year==2007

replace regioncode="141181" if regioncode=="141181" & year==2007

replace regioncode="141102" if regioncode=="141102" & year==2007

replace regioncode="141182" if regioncode=="141182" & year==2007

replace regioncode="141121" if regioncode=="141121" & year==2007

replace regioncode="141122" if regioncode=="141122" & year==2007

replace regioncode="141123" if regioncode=="141123" & year==2007

replace regioncode="141124" if regioncode=="141124" & year==2007

replace regioncode="141125" if regioncode=="141125" & year==2007

replace regioncode="141126" if regioncode=="141126" & year==2007

replace regioncode="141127" if regioncode=="141127" & year==2007

replace regioncode="141128" if regioncode=="141128" & year==2007

replace regioncode="141129" if regioncode=="141129" & year==2007

replace regioncode="141130" if regioncode=="141130" & year==2007

replace regioncode="140702" if regioncode=="140702" & year==2007

replace regioncode="140781" if regioncode=="140781" & year==2007

replace regioncode="140721" if regioncode=="140721" & year==2007

replace regioncode="140722" if regioncode=="140722" & year==2007

replace regioncode="140723" if regioncode=="140723" & year==2007

replace regioncode="140724" if regioncode=="140724" & year==2007

replace regioncode="140725" if regioncode=="140725" & year==2007

replace regioncode="140726" if regioncode=="140726" & year==2007

replace regioncode="140727" if regioncode=="140727" & year==2007

replace regioncode="140728" if regioncode=="140728" & year==2007

replace regioncode="140729" if regioncode=="140729" & year==2007

replace regioncode="141002" if regioncode=="141002" & year==2007

replace regioncode="141081" if regioncode=="141081" & year==2007

replace regioncode="141082" if regioncode=="141082" & year==2007

replace regioncode="141021" if regioncode=="141021" & year==2007

replace regioncode="141022" if regioncode=="141022" & year==2007

replace regioncode="141023" if regioncode=="141023" & year==2007

replace regioncode="141024" if regioncode=="141024" & year==2007

replace regioncode="141025" if regioncode=="141025" & year==2007

replace regioncode="141026" if regioncode=="141026" & year==2007

replace regioncode="141027" if regioncode=="141027" & year==2007

replace regioncode="141028" if regioncode=="141028" & year==2007

replace regioncode="141029" if regioncode=="141029" & year==2007

replace regioncode="141033" if regioncode=="141033" & year==2007

replace regioncode="141030" if regioncode=="141030" & year==2007

replace regioncode="141032" if regioncode=="141032" & year==2007

replace regioncode="141031" if regioncode=="141031" & year==2007

replace regioncode="141034" if regioncode=="141034" & year==2007

replace regioncode="140802" if regioncode=="140802" & year==2007

replace regioncode="140881" if regioncode=="140881" & year==2007

replace regioncode="140882" if regioncode=="140882" & year==2007

replace regioncode="140830" if regioncode=="140830" & year==2007

replace regioncode="140821" if regioncode=="140821" & year==2007

replace regioncode="140822" if regioncode=="140822" & year==2007

replace regioncode="140825" if regioncode=="140825" & year==2007

replace regioncode="140824" if regioncode=="140824" & year==2007

replace regioncode="140823" if regioncode=="140823" & year==2007

replace regioncode="140828" if regioncode=="140828" & year==2007

replace regioncode="140826" if regioncode=="140826" & year==2007

replace regioncode="140829" if regioncode=="140829" & year==2007

replace regioncode="140827" if regioncode=="140827" & year==2007

replace regioncode="150102" if regioncode=="150102" & year==2007

replace regioncode="150103" if regioncode=="150103" & year==2007

replace regioncode="150104" if regioncode=="150104" & year==2007

replace regioncode="150105" if regioncode=="150105" & year==2007

replace regioncode="150121" if regioncode=="150121" & year==2007

replace regioncode="150122" if regioncode=="150122" & year==2007

replace regioncode="150123" if regioncode=="150123" & year==2007

replace regioncode="150124" if regioncode=="150124" & year==2007

replace regioncode="150125" if regioncode=="150125" & year==2007

replace regioncode="150202" if regioncode=="150202" & year==2007

replace regioncode="150203" if regioncode=="150203" & year==2007

replace regioncode="150204" if regioncode=="150204" & year==2007

replace regioncode="150205" if regioncode=="150205" & year==2007

replace regioncode="150206" if regioncode=="150206" & year==2007

replace regioncode="150207" if regioncode=="150207" & year==2007

replace regioncode="150221" if regioncode=="150221" & year==2007

replace regioncode="150222" if regioncode=="150222" & year==2007

replace regioncode="150223" if regioncode=="150223" & year==2007

replace regioncode="150302" if regioncode=="150302" & year==2007

replace regioncode="150303" if regioncode=="150303" & year==2007

replace regioncode="150304" if regioncode=="150304" & year==2007

replace regioncode="150402" if regioncode=="150402" & year==2007

replace regioncode="150403" if regioncode=="150403" & year==2007

replace regioncode="150404" if regioncode=="150404" & year==2007

replace regioncode="150421" if regioncode=="150421" & year==2007

replace regioncode="150422" if regioncode=="150422" & year==2007

replace regioncode="150423" if regioncode=="150423" & year==2007

replace regioncode="150424" if regioncode=="150424" & year==2007

replace regioncode="150425" if regioncode=="150425" & year==2007

replace regioncode="150426" if regioncode=="150426" & year==2007

replace regioncode="150428" if regioncode=="150428" & year==2007

replace regioncode="150429" if regioncode=="150429" & year==2007

replace regioncode="150430" if regioncode=="150430" & year==2007

replace regioncode="150702" if regioncode=="150702" & year==2007

replace regioncode="150781" if regioncode=="150781" & year==2007

replace regioncode="150783" if regioncode=="150783" & year==2007

replace regioncode="150782" if regioncode=="150782" & year==2007

replace regioncode="150785" if regioncode=="150785" & year==2007

replace regioncode="150784" if regioncode=="150784" & year==2007

replace regioncode="150721" if regioncode=="150721" & year==2007

replace regioncode="150722" if regioncode=="150722" & year==2007

replace regioncode="150723" if regioncode=="150723" & year==2007

replace regioncode="150724" if regioncode=="150724" & year==2007

replace regioncode="150727" if regioncode=="150727" & year==2007

replace regioncode="150726" if regioncode=="150726" & year==2007

replace regioncode="150725" if regioncode=="150725" & year==2007

replace regioncode="152201" if regioncode=="152201" & year==2007

replace regioncode="152202" if regioncode=="152202" & year==2007

replace regioncode="152221" if regioncode=="152221" & year==2007

replace regioncode="152222" if regioncode=="152222" & year==2007

replace regioncode="152223" if regioncode=="152223" & year==2007

replace regioncode="152224" if regioncode=="152224" & year==2007

replace regioncode="150502" if regioncode=="150502" & year==2007

replace regioncode="150581" if regioncode=="150581" & year==2007

replace regioncode="150521" if regioncode=="150521" & year==2007

replace regioncode="150522" if regioncode=="150522" & year==2007

replace regioncode="150523" if regioncode=="150523" & year==2007

replace regioncode="150524" if regioncode=="150524" & year==2007

replace regioncode="150525" if regioncode=="150525" & year==2007

replace regioncode="150526" if regioncode=="150526" & year==2007

replace regioncode="152501" if regioncode=="152501" & year==2007

replace regioncode="152502" if regioncode=="152502" & year==2007

replace regioncode="152522" if regioncode=="152522" & year==2007

replace regioncode="152523" if regioncode=="152523" & year==2007

replace regioncode="152524" if regioncode=="152524" & year==2007

replace regioncode="152525" if regioncode=="152525" & year==2007

replace regioncode="152526" if regioncode=="152526" & year==2007

replace regioncode="152527" if regioncode=="152527" & year==2007

replace regioncode="152528" if regioncode=="152528" & year==2007

replace regioncode="152529" if regioncode=="152529" & year==2007

replace regioncode="152530" if regioncode=="152530" & year==2007

replace regioncode="152531" if regioncode=="152531" & year==2007

replace regioncode="152601" if regioncode=="150902" & year==2007

replace regioncode="152602" if regioncode=="150981" & year==2007

replace regioncode="152624" if regioncode=="150921" & year==2007

replace regioncode="152625" if regioncode=="150922" & year==2007

replace regioncode="152626" if regioncode=="150923" & year==2007

replace regioncode="152627" if regioncode=="150924" & year==2007

replace regioncode="152629" if regioncode=="150925" & year==2007

replace regioncode="152630" if regioncode=="150926" & year==2007

replace regioncode="152631" if regioncode=="150927" & year==2007

replace regioncode="152632" if regioncode=="150928" & year==2007

replace regioncode="152634" if regioncode=="150929" & year==2007

replace regioncode="150602" if regioncode=="150602" & year==2007

replace regioncode="150621" if regioncode=="150621" & year==2007

replace regioncode="150622" if regioncode=="150622" & year==2007

replace regioncode="150623" if regioncode=="150623" & year==2007

replace regioncode="150624" if regioncode=="150624" & year==2007

replace regioncode="150625" if regioncode=="150625" & year==2007

replace regioncode="150626" if regioncode=="150626" & year==2007

replace regioncode="150627" if regioncode=="150627" & year==2007

replace regioncode="152801" if regioncode=="150802" & year==2007

replace regioncode="152822" if regioncode=="150821" & year==2007

replace regioncode="152823" if regioncode=="150822" & year==2007

replace regioncode="152824" if regioncode=="150823" & year==2007

replace regioncode="152825" if regioncode=="150824" & year==2007

replace regioncode="152826" if regioncode=="150825" & year==2007

replace regioncode="152827" if regioncode=="150826" & year==2007

replace regioncode="152921" if regioncode=="152921" & year==2007

replace regioncode="152922" if regioncode=="152922" & year==2007

replace regioncode="152923" if regioncode=="152923" & year==2007

replace regioncode="210102" if regioncode=="210102" & year==2007

replace regioncode="210103" if regioncode=="210103" & year==2007

replace regioncode="210104" if regioncode=="210104" & year==2007

replace regioncode="210105" if regioncode=="210105" & year==2007

replace regioncode="210106" if regioncode=="210106" & year==2007

replace regioncode="210111" if regioncode=="210111" & year==2007

replace regioncode="210112" if regioncode=="210112" & year==2007

replace regioncode="210113" if regioncode=="210113" & year==2007

replace regioncode="210114" if regioncode=="210114" & year==2007

replace regioncode="210122" if regioncode=="210122" & year==2007

replace regioncode="210123" if regioncode=="210123" & year==2007

replace regioncode="210124" if regioncode=="210124" & year==2007

replace regioncode="210181" if regioncode=="210181" & year==2007

replace regioncode="210202" if regioncode=="210202" & year==2007

replace regioncode="210203" if regioncode=="210203" & year==2007

replace regioncode="210204" if regioncode=="210204" & year==2007

replace regioncode="210211" if regioncode=="210211" & year==2007

replace regioncode="210212" if regioncode=="210212" & year==2007

replace regioncode="210213" if regioncode=="210213" & year==2007

replace regioncode="210224" if regioncode=="210224" & year==2007

replace regioncode="210281" if regioncode=="210281" & year==2007

replace regioncode="210282" if regioncode=="210282" & year==2007

replace regioncode="210283" if regioncode=="210283" & year==2007

replace regioncode="210302" if regioncode=="210302" & year==2007

replace regioncode="210303" if regioncode=="210303" & year==2007

replace regioncode="210304" if regioncode=="210304" & year==2007

replace regioncode="210311" if regioncode=="210311" & year==2007

replace regioncode="210321" if regioncode=="210321" & year==2007

replace regioncode="210323" if regioncode=="210323" & year==2007

replace regioncode="210381" if regioncode=="210381" & year==2007

replace regioncode="210402" if regioncode=="210402" & year==2007

replace regioncode="210403" if regioncode=="210403" & year==2007

replace regioncode="210404" if regioncode=="210404" & year==2007

replace regioncode="210411" if regioncode=="210411" & year==2007

replace regioncode="210421" if regioncode=="210421" & year==2007

replace regioncode="210422" if regioncode=="210422" & year==2007

replace regioncode="210423" if regioncode=="210423" & year==2007

replace regioncode="210502" if regioncode=="210502" & year==2007

replace regioncode="210503" if regioncode=="210503" & year==2007

replace regioncode="210504" if regioncode=="210504" & year==2007

replace regioncode="210505" if regioncode=="210505" & year==2007

replace regioncode="210521" if regioncode=="210521" & year==2007

replace regioncode="210522" if regioncode=="210522" & year==2007

replace regioncode="210602" if regioncode=="210602" & year==2007

replace regioncode="210603" if regioncode=="210603" & year==2007

replace regioncode="210604" if regioncode=="210604" & year==2007

replace regioncode="210624" if regioncode=="210624" & year==2007

replace regioncode="210681" if regioncode=="210681" & year==2007

replace regioncode="210682" if regioncode=="210682" & year==2007

replace regioncode="210702" if regioncode=="210702" & year==2007

replace regioncode="210703" if regioncode=="210703" & year==2007

replace regioncode="210711" if regioncode=="210711" & year==2007

replace regioncode="210726" if regioncode=="210726" & year==2007

replace regioncode="210727" if regioncode=="210727" & year==2007

replace regioncode="210781" if regioncode=="210781" & year==2007

replace regioncode="210782" if regioncode=="210782" & year==2007

replace regioncode="210802" if regioncode=="210802" & year==2007

replace regioncode="210803" if regioncode=="210803" & year==2007

replace regioncode="210804" if regioncode=="210804" & year==2007

replace regioncode="210811" if regioncode=="210811" & year==2007

replace regioncode="210881" if regioncode=="210881" & year==2007

replace regioncode="210882" if regioncode=="210882" & year==2007

replace regioncode="210902" if regioncode=="210902" & year==2007

replace regioncode="210903" if regioncode=="210903" & year==2007

replace regioncode="210904" if regioncode=="210904" & year==2007

replace regioncode="210905" if regioncode=="210905" & year==2007

replace regioncode="210911" if regioncode=="210911" & year==2007

replace regioncode="210921" if regioncode=="210921" & year==2007

replace regioncode="210922" if regioncode=="210922" & year==2007

replace regioncode="211002" if regioncode=="211002" & year==2007

replace regioncode="211003" if regioncode=="211003" & year==2007

replace regioncode="211004" if regioncode=="211004" & year==2007

replace regioncode="211005" if regioncode=="211005" & year==2007

replace regioncode="211011" if regioncode=="211011" & year==2007

replace regioncode="211021" if regioncode=="211021" & year==2007

replace regioncode="211081" if regioncode=="211081" & year==2007

replace regioncode="211102" if regioncode=="211102" & year==2007

replace regioncode="211103" if regioncode=="211103" & year==2007

replace regioncode="211121" if regioncode=="211121" & year==2007

replace regioncode="211122" if regioncode=="211122" & year==2007

replace regioncode="211202" if regioncode=="211202" & year==2007

replace regioncode="211204" if regioncode=="211204" & year==2007

replace regioncode="211221" if regioncode=="211221" & year==2007

replace regioncode="211223" if regioncode=="211223" & year==2007

replace regioncode="211224" if regioncode=="211224" & year==2007

replace regioncode="211281" if regioncode=="211281" & year==2007

replace regioncode="211282" if regioncode=="211282" & year==2007

replace regioncode="211302" if regioncode=="211302" & year==2007

replace regioncode="211303" if regioncode=="211303" & year==2007

replace regioncode="211321" if regioncode=="211321" & year==2007

replace regioncode="211322" if regioncode=="211322" & year==2007

replace regioncode="211324" if regioncode=="211324" & year==2007

replace regioncode="211381" if regioncode=="211381" & year==2007

replace regioncode="211382" if regioncode=="211382" & year==2007

replace regioncode="211402" if regioncode=="211402" & year==2007

replace regioncode="211403" if regioncode=="211403" & year==2007

replace regioncode="211404" if regioncode=="211404" & year==2007

replace regioncode="211421" if regioncode=="211421" & year==2007

replace regioncode="211422" if regioncode=="211422" & year==2007

replace regioncode="211481" if regioncode=="211481" & year==2007

replace regioncode="220102" if regioncode=="220102" & year==2007

replace regioncode="220103" if regioncode=="220103" & year==2007

replace regioncode="220104" if regioncode=="220104" & year==2007

replace regioncode="220105" if regioncode=="220105" & year==2007

replace regioncode="220106" if regioncode=="220106" & year==2007

replace regioncode="220112" if regioncode=="220112" & year==2007

replace regioncode="220122" if regioncode=="220122" & year==2007

replace regioncode="220181" if regioncode=="220181" & year==2007

replace regioncode="220182" if regioncode=="220182" & year==2007

replace regioncode="220183" if regioncode=="220183" & year==2007

replace regioncode="220202" if regioncode=="220202" & year==2007

replace regioncode="220203" if regioncode=="220203" & year==2007

replace regioncode="220204" if regioncode=="220204" & year==2007

replace regioncode="220211" if regioncode=="220211" & year==2007

replace regioncode="220221" if regioncode=="220221" & year==2007

replace regioncode="220281" if regioncode=="220281" & year==2007

replace regioncode="220282" if regioncode=="220282" & year==2007

replace regioncode="220283" if regioncode=="220283" & year==2007

replace regioncode="220284" if regioncode=="220284" & year==2007

replace regioncode="220302" if regioncode=="220302" & year==2007

replace regioncode="220303" if regioncode=="220303" & year==2007

replace regioncode="220322" if regioncode=="220322" & year==2007

replace regioncode="220323" if regioncode=="220323" & year==2007

replace regioncode="220381" if regioncode=="220381" & year==2007

replace regioncode="220382" if regioncode=="220382" & year==2007

replace regioncode="220402" if regioncode=="220402" & year==2007

replace regioncode="220403" if regioncode=="220403" & year==2007

replace regioncode="220421" if regioncode=="220421" & year==2007

replace regioncode="220422" if regioncode=="220422" & year==2007

replace regioncode="220502" if regioncode=="220502" & year==2007

replace regioncode="220503" if regioncode=="220503" & year==2007

replace regioncode="220521" if regioncode=="220521" & year==2007

replace regioncode="220523" if regioncode=="220523" & year==2007

replace regioncode="220524" if regioncode=="220524" & year==2007

replace regioncode="220581" if regioncode=="220581" & year==2007

replace regioncode="220582" if regioncode=="220582" & year==2007

replace regioncode="220602" if regioncode=="220602" & year==2007

replace regioncode="220621" if regioncode=="220621" & year==2007

replace regioncode="220622" if regioncode=="220622" & year==2007

replace regioncode="220623" if regioncode=="220623" & year==2007

replace regioncode="220625" if regioncode=="220625" & year==2007

replace regioncode="220681" if regioncode=="220681" & year==2007

replace regioncode="220702" if regioncode=="220702" & year==2007

replace regioncode="220721" if regioncode=="220721" & year==2007

replace regioncode="220722" if regioncode=="220722" & year==2007

replace regioncode="220723" if regioncode=="220723" & year==2007

replace regioncode="220724" if regioncode=="220724" & year==2007

replace regioncode="220802" if regioncode=="220802" & year==2007

replace regioncode="220821" if regioncode=="220821" & year==2007

replace regioncode="220822" if regioncode=="220822" & year==2007

replace regioncode="220881" if regioncode=="220881" & year==2007

replace regioncode="220882" if regioncode=="220882" & year==2007

replace regioncode="222401" if regioncode=="222401" & year==2007

replace regioncode="222402" if regioncode=="222402" & year==2007

replace regioncode="222403" if regioncode=="222403" & year==2007

replace regioncode="222404" if regioncode=="222404" & year==2007

replace regioncode="222405" if regioncode=="222405" & year==2007

replace regioncode="222406" if regioncode=="222406" & year==2007

replace regioncode="222424" if regioncode=="222424" & year==2007

replace regioncode="222426" if regioncode=="222426" & year==2007

replace regioncode="230102" if regioncode=="230102" & year==2007

replace regioncode="230103" if regioncode=="230103" & year==2007

replace regioncode="230104" if regioncode=="230104" & year==2007

replace regioncode="230105" if regioncode=="230105" & year==2007

replace regioncode="230107" if regioncode=="230110" & year==2007

replace regioncode="230108" if regioncode=="230108" & year==2007

replace regioncode="230121" if regioncode=="230111" & year==2007

replace regioncode="230123" if regioncode=="230123" & year==2007

replace regioncode="230124" if regioncode=="230124" & year==2007

replace regioncode="230125" if regioncode=="230125" & year==2007

replace regioncode="230126" if regioncode=="230126" & year==2007

replace regioncode="230127" if regioncode=="230127" & year==2007

replace regioncode="230128" if regioncode=="230128" & year==2007

replace regioncode="230129" if regioncode=="230129" & year==2007

replace regioncode="230181" if regioncode=="230112" & year==2007

replace regioncode="230182" if regioncode=="230182" & year==2007

replace regioncode="230183" if regioncode=="230183" & year==2007

replace regioncode="230184" if regioncode=="230184" & year==2007

replace regioncode="230202" if regioncode=="230202" & year==2007

replace regioncode="230203" if regioncode=="230203" & year==2007

replace regioncode="230204" if regioncode=="230204" & year==2007

replace regioncode="230205" if regioncode=="230205" & year==2007

replace regioncode="230206" if regioncode=="230206" & year==2007

replace regioncode="230207" if regioncode=="230207" & year==2007

replace regioncode="230208" if regioncode=="230208" & year==2007

replace regioncode="230221" if regioncode=="230221" & year==2007

replace regioncode="230223" if regioncode=="230223" & year==2007

replace regioncode="230224" if regioncode=="230224" & year==2007

replace regioncode="230225" if regioncode=="230225" & year==2007

replace regioncode="230227" if regioncode=="230227" & year==2007

replace regioncode="230229" if regioncode=="230229" & year==2007

replace regioncode="230230" if regioncode=="230230" & year==2007

replace regioncode="230231" if regioncode=="230231" & year==2007

replace regioncode="230281" if regioncode=="230281" & year==2007

replace regioncode="230302" if regioncode=="230302" & year==2007

replace regioncode="230303" if regioncode=="230303" & year==2007

replace regioncode="230304" if regioncode=="230304" & year==2007

replace regioncode="230305" if regioncode=="230305" & year==2007

replace regioncode="230306" if regioncode=="230306" & year==2007

replace regioncode="230307" if regioncode=="230307" & year==2007

replace regioncode="230321" if regioncode=="230321" & year==2007

replace regioncode="230381" if regioncode=="230381" & year==2007

replace regioncode="230382" if regioncode=="230382" & year==2007

replace regioncode="230402" if regioncode=="230402" & year==2007

replace regioncode="230403" if regioncode=="230403" & year==2007

replace regioncode="230404" if regioncode=="230404" & year==2007

replace regioncode="230405" if regioncode=="230405" & year==2007

replace regioncode="230406" if regioncode=="230406" & year==2007

replace regioncode="230407" if regioncode=="230407" & year==2007

replace regioncode="230421" if regioncode=="230421" & year==2007

replace regioncode="230422" if regioncode=="230422" & year==2007

replace regioncode="230502" if regioncode=="230502" & year==2007

replace regioncode="230503" if regioncode=="230503" & year==2007

replace regioncode="230505" if regioncode=="230505" & year==2007

replace regioncode="230506" if regioncode=="230506" & year==2007

replace regioncode="230521" if regioncode=="230521" & year==2007

replace regioncode="230522" if regioncode=="230522" & year==2007

replace regioncode="230523" if regioncode=="230523" & year==2007

replace regioncode="230524" if regioncode=="230524" & year==2007

replace regioncode="230602" if regioncode=="230602" & year==2007

replace regioncode="230603" if regioncode=="230603" & year==2007

replace regioncode="230604" if regioncode=="230604" & year==2007

replace regioncode="230605" if regioncode=="230605" & year==2007

replace regioncode="230606" if regioncode=="230606" & year==2007

replace regioncode="230621" if regioncode=="230621" & year==2007

replace regioncode="230622" if regioncode=="230622" & year==2007

replace regioncode="230623" if regioncode=="230623" & year==2007

replace regioncode="230624" if regioncode=="230624" & year==2007

replace regioncode="230702" if regioncode=="230702" & year==2007

replace regioncode="230703" if regioncode=="230703" & year==2007

replace regioncode="230704" if regioncode=="230704" & year==2007

replace regioncode="230705" if regioncode=="230705" & year==2007

replace regioncode="230706" if regioncode=="230706" & year==2007

replace regioncode="230707" if regioncode=="230707" & year==2007

replace regioncode="230708" if regioncode=="230708" & year==2007

replace regioncode="230709" if regioncode=="230709" & year==2007

replace regioncode="230710" if regioncode=="230710" & year==2007

replace regioncode="230711" if regioncode=="230711" & year==2007

replace regioncode="230712" if regioncode=="230712" & year==2007

replace regioncode="230713" if regioncode=="230713" & year==2007

replace regioncode="230714" if regioncode=="230714" & year==2007

replace regioncode="230715" if regioncode=="230715" & year==2007

replace regioncode="230716" if regioncode=="230716" & year==2007

replace regioncode="230722" if regioncode=="230722" & year==2007

replace regioncode="230781" if regioncode=="230781" & year==2007

replace regioncode="230802" if regioncode=="230802" & year==2007

replace regioncode="230803" if regioncode=="230803" & year==2007

replace regioncode="230804" if regioncode=="230804" & year==2007

replace regioncode="230805" if regioncode=="230805" & year==2007

replace regioncode="230811" if regioncode=="230811" & year==2007

replace regioncode="230822" if regioncode=="230822" & year==2007

replace regioncode="230826" if regioncode=="230826" & year==2007

replace regioncode="230828" if regioncode=="230828" & year==2007

replace regioncode="230833" if regioncode=="230833" & year==2007

replace regioncode="230881" if regioncode=="230881" & year==2007

replace regioncode="230882" if regioncode=="230882" & year==2007

replace regioncode="230902" if regioncode=="230902" & year==2007

replace regioncode="230903" if regioncode=="230903" & year==2007

replace regioncode="230904" if regioncode=="230904" & year==2007

replace regioncode="230921" if regioncode=="230921" & year==2007

replace regioncode="231002" if regioncode=="231002" & year==2007

replace regioncode="231003" if regioncode=="231003" & year==2007

replace regioncode="231004" if regioncode=="231004" & year==2007

replace regioncode="231005" if regioncode=="231005" & year==2007

replace regioncode="231024" if regioncode=="231024" & year==2007

replace regioncode="231025" if regioncode=="231025" & year==2007

replace regioncode="231081" if regioncode=="231081" & year==2007

replace regioncode="231083" if regioncode=="231083" & year==2007

replace regioncode="231084" if regioncode=="231084" & year==2007

replace regioncode="231085" if regioncode=="231085" & year==2007

replace regioncode="231102" if regioncode=="231102" & year==2007

replace regioncode="231121" if regioncode=="231121" & year==2007

replace regioncode="231123" if regioncode=="231123" & year==2007

replace regioncode="231124" if regioncode=="231124" & year==2007

replace regioncode="231181" if regioncode=="231181" & year==2007

replace regioncode="231182" if regioncode=="231182" & year==2007

replace regioncode="231202" if regioncode=="231202" & year==2007

replace regioncode="231221" if regioncode=="231221" & year==2007

replace regioncode="231222" if regioncode=="231222" & year==2007

replace regioncode="231223" if regioncode=="231223" & year==2007

replace regioncode="231224" if regioncode=="231224" & year==2007

replace regioncode="231225" if regioncode=="231225" & year==2007

replace regioncode="231226" if regioncode=="231226" & year==2007

replace regioncode="231281" if regioncode=="231281" & year==2007

replace regioncode="231282" if regioncode=="231282" & year==2007

replace regioncode="231283" if regioncode=="231283" & year==2007

replace regioncode="232701" if regioncode=="232701" & year==2007

replace regioncode="232702" if regioncode=="232702" & year==2007

replace regioncode="232703" if regioncode=="232703" & year==2007

replace regioncode="232704" if regioncode=="232704" & year==2007

replace regioncode="232721" if regioncode=="232721" & year==2007

replace regioncode="232722" if regioncode=="232722" & year==2007

replace regioncode="232723" if regioncode=="232723" & year==2007

replace regioncode="310101" if regioncode=="310101" & year==2007

replace regioncode="310103" if regioncode=="310103" & year==2007

replace regioncode="310104" if regioncode=="310104" & year==2007

replace regioncode="310105" if regioncode=="310105" & year==2007

replace regioncode="310106" if regioncode=="310106" & year==2007

replace regioncode="310107" if regioncode=="310107" & year==2007

replace regioncode="310108" if regioncode=="310108" & year==2007

replace regioncode="310109" if regioncode=="310109" & year==2007

replace regioncode="310110" if regioncode=="310110" & year==2007

replace regioncode="310112" if regioncode=="310112" & year==2007

replace regioncode="310113" if regioncode=="310113" & year==2007

replace regioncode="310114" if regioncode=="310114" & year==2007

replace regioncode="310115" if regioncode=="310115" & year==2007

replace regioncode="310116" if regioncode=="310116" & year==2007

replace regioncode="310117" if regioncode=="310117" & year==2007

replace regioncode="310118" if regioncode=="310118" & year==2007

replace regioncode="310119" if regioncode=="310119" & year==2007

replace regioncode="310120" if regioncode=="310120" & year==2007

replace regioncode="310230" if regioncode=="310230" & year==2007

replace regioncode="320102" if regioncode=="320102" & year==2007

replace regioncode="320103" if regioncode=="320103" & year==2007

replace regioncode="320104" if regioncode=="320104" & year==2007

replace regioncode="320105" if regioncode=="320105" & year==2007

replace regioncode="320106" if regioncode=="320106" & year==2007

replace regioncode="320107" if regioncode=="320107" & year==2007

replace regioncode="320111" if regioncode=="320111" & year==2007

replace regioncode="320113" if regioncode=="320113" & year==2007

replace regioncode="320114" if regioncode=="320114" & year==2007

replace regioncode="320115" if regioncode=="320115" & year==2007

replace regioncode="320116" if regioncode=="320116" & year==2007

replace regioncode="320124" if regioncode=="320124" & year==2007

replace regioncode="320125" if regioncode=="320125" & year==2007

replace regioncode="320202" if regioncode=="320202" & year==2007

replace regioncode="320203" if regioncode=="320203" & year==2007

replace regioncode="320204" if regioncode=="320204" & year==2007

replace regioncode="320205" if regioncode=="320205" & year==2007

replace regioncode="320211" if regioncode=="320211" & year==2007

replace regioncode="320281" if regioncode=="320281" & year==2007

replace regioncode="320282" if regioncode=="320282" & year==2007

replace regioncode="320302" if regioncode=="320302" & year==2007

replace regioncode="320303" if regioncode=="320303" & year==2007

replace regioncode="320304" if regioncode=="320304" & year==2007

replace regioncode="320305" if regioncode=="320305" & year==2007

replace regioncode="320311" if regioncode=="320311" & year==2007

replace regioncode="320321" if regioncode=="320321" & year==2007

replace regioncode="320322" if regioncode=="320322" & year==2007

replace regioncode="320323" if regioncode=="320323" & year==2007

replace regioncode="320324" if regioncode=="320324" & year==2007

replace regioncode="320381" if regioncode=="320381" & year==2007

replace regioncode="320382" if regioncode=="320382" & year==2007

replace regioncode="320402" if regioncode=="320402" & year==2007

replace regioncode="320404" if regioncode=="320404" & year==2007

replace regioncode="320405" if regioncode=="320405" & year==2007

replace regioncode="320411" if regioncode=="320411" & year==2007

replace regioncode="320412" if regioncode=="320412" & year==2007

replace regioncode="320481" if regioncode=="320481" & year==2007

replace regioncode="320482" if regioncode=="320482" & year==2007

replace regioncode="320502" if regioncode=="320502" & year==2007

replace regioncode="320503" if regioncode=="320503" & year==2007

replace regioncode="320504" if regioncode=="320504" & year==2007

replace regioncode="320505" if regioncode=="320505" & year==2007

replace regioncode="320581" if regioncode=="320581" & year==2007

replace regioncode="320582" if regioncode=="320582" & year==2007

replace regioncode="320583" if regioncode=="320583" & year==2007

replace regioncode="320584" if regioncode=="320584" & year==2007

replace regioncode="320585" if regioncode=="320585" & year==2007

replace regioncode="320586" if regioncode=="320586" & year==2007

replace regioncode="320602" if regioncode=="320602" & year==2007

replace regioncode="320611" if regioncode=="320611" & year==2007

replace regioncode="320621" if regioncode=="320621" & year==2007

replace regioncode="320623" if regioncode=="320623" & year==2007

replace regioncode="320681" if regioncode=="320681" & year==2007

replace regioncode="320682" if regioncode=="320682" & year==2007

replace regioncode="320683" if regioncode=="320683" & year==2007

replace regioncode="320684" if regioncode=="320684" & year==2007

replace regioncode="320703" if regioncode=="320703" & year==2007

replace regioncode="320705" if regioncode=="320705" & year==2007

replace regioncode="320706" if regioncode=="320706" & year==2007

replace regioncode="320721" if regioncode=="320721" & year==2007

replace regioncode="320722" if regioncode=="320722" & year==2007

replace regioncode="320723" if regioncode=="320723" & year==2007

replace regioncode="320724" if regioncode=="320724" & year==2007

replace regioncode="320802" if regioncode=="320802" & year==2007

replace regioncode="320803" if regioncode=="320803" & year==2007

replace regioncode="320804" if regioncode=="320804" & year==2007

replace regioncode="320811" if regioncode=="320811" & year==2007

replace regioncode="320826" if regioncode=="320826" & year==2007

replace regioncode="320829" if regioncode=="320829" & year==2007

replace regioncode="320830" if regioncode=="320830" & year==2007

replace regioncode="320831" if regioncode=="320831" & year==2007

replace regioncode="320902" if regioncode=="320902" & year==2007

replace regioncode="320903" if regioncode=="320903" & year==2007

replace regioncode="320921" if regioncode=="320921" & year==2007

replace regioncode="320922" if regioncode=="320922" & year==2007

replace regioncode="320923" if regioncode=="320923" & year==2007

replace regioncode="320924" if regioncode=="320924" & year==2007

replace regioncode="320925" if regioncode=="320925" & year==2007

replace regioncode="320981" if regioncode=="320981" & year==2007

replace regioncode="320982" if regioncode=="320982" & year==2007

replace regioncode="321002" if regioncode=="321002" & year==2007

replace regioncode="321003" if regioncode=="321003" & year==2007

replace regioncode="321011" if regioncode=="321011" & year==2007

replace regioncode="321023" if regioncode=="321023" & year==2007

replace regioncode="321081" if regioncode=="321081" & year==2007

replace regioncode="321084" if regioncode=="321084" & year==2007

replace regioncode="321088" if regioncode=="321088" & year==2007

replace regioncode="321102" if regioncode=="321102" & year==2007

replace regioncode="321111" if regioncode=="321111" & year==2007

replace regioncode="321112" if regioncode=="321112" & year==2007

replace regioncode="321181" if regioncode=="321181" & year==2007

replace regioncode="321182" if regioncode=="321182" & year==2007

replace regioncode="321183" if regioncode=="321183" & year==2007

replace regioncode="321202" if regioncode=="321202" & year==2007

replace regioncode="321203" if regioncode=="321203" & year==2007

replace regioncode="321281" if regioncode=="321281" & year==2007

replace regioncode="321282" if regioncode=="321282" & year==2007

replace regioncode="321283" if regioncode=="321283" & year==2007

replace regioncode="321284" if regioncode=="321284" & year==2007

replace regioncode="321302" if regioncode=="321302" & year==2007

replace regioncode="321321" if regioncode=="321311" & year==2007

replace regioncode="321322" if regioncode=="321322" & year==2007

replace regioncode="321323" if regioncode=="321323" & year==2007

replace regioncode="321324" if regioncode=="321324" & year==2007

replace regioncode="330102" if regioncode=="330102" & year==2007

replace regioncode="330103" if regioncode=="330103" & year==2007

replace regioncode="330104" if regioncode=="330104" & year==2007

replace regioncode="330105" if regioncode=="330105" & year==2007

replace regioncode="330106" if regioncode=="330106" & year==2007

replace regioncode="330108" if regioncode=="330108" & year==2007

replace regioncode="330109" if regioncode=="330109" & year==2007

replace regioncode="330110" if regioncode=="330110" & year==2007

replace regioncode="330122" if regioncode=="330122" & year==2007

replace regioncode="330127" if regioncode=="330127" & year==2007

replace regioncode="330182" if regioncode=="330182" & year==2007

replace regioncode="330183" if regioncode=="330183" & year==2007

replace regioncode="330185" if regioncode=="330185" & year==2007

replace regioncode="330203" if regioncode=="330203" & year==2007

replace regioncode="330204" if regioncode=="330204" & year==2007

replace regioncode="330205" if regioncode=="330205" & year==2007

replace regioncode="330206" if regioncode=="330206" & year==2007

replace regioncode="330211" if regioncode=="330211" & year==2007

replace regioncode="330212" if regioncode=="330212" & year==2007

replace regioncode="330225" if regioncode=="330225" & year==2007

replace regioncode="330226" if regioncode=="330226" & year==2007

replace regioncode="330281" if regioncode=="330281" & year==2007

replace regioncode="330282" if regioncode=="330282" & year==2007

replace regioncode="330283" if regioncode=="330283" & year==2007

replace regioncode="330302" if regioncode=="330302" & year==2007

replace regioncode="330303" if regioncode=="330303" & year==2007

replace regioncode="330304" if regioncode=="330304" & year==2007

replace regioncode="330322" if regioncode=="330322" & year==2007

replace regioncode="330324" if regioncode=="330324" & year==2007

replace regioncode="330326" if regioncode=="330326" & year==2007

replace regioncode="330327" if regioncode=="330327" & year==2007

replace regioncode="330328" if regioncode=="330328" & year==2007

replace regioncode="330329" if regioncode=="330329" & year==2007

replace regioncode="330381" if regioncode=="330381" & year==2007

replace regioncode="330382" if regioncode=="330382" & year==2007

replace regioncode="330402" if regioncode=="330402" & year==2007

replace regioncode="330411" if regioncode=="330411" & year==2007

replace regioncode="330421" if regioncode=="330421" & year==2007

replace regioncode="330424" if regioncode=="330424" & year==2007

replace regioncode="330481" if regioncode=="330481" & year==2007

replace regioncode="330482" if regioncode=="330482" & year==2007

replace regioncode="330483" if regioncode=="330483" & year==2007

replace regioncode="330501" if regioncode=="330501" & year==2007

replace regioncode="330521" if regioncode=="330521" & year==2007

replace regioncode="330522" if regioncode=="330522" & year==2007

replace regioncode="330523" if regioncode=="330523" & year==2007

replace regioncode="330602" if regioncode=="330602" & year==2007

replace regioncode="330621" if regioncode=="330621" & year==2007

replace regioncode="330624" if regioncode=="330624" & year==2007

replace regioncode="330681" if regioncode=="330681" & year==2007

replace regioncode="330682" if regioncode=="330682" & year==2007

replace regioncode="330683" if regioncode=="330683" & year==2007

replace regioncode="330702" if regioncode=="330702" & year==2007

replace regioncode="330703" if regioncode=="330703" & year==2007

replace regioncode="330723" if regioncode=="330723" & year==2007

replace regioncode="330726" if regioncode=="330726" & year==2007

replace regioncode="330727" if regioncode=="330727" & year==2007

replace regioncode="330781" if regioncode=="330781" & year==2007

replace regioncode="330782" if regioncode=="330782" & year==2007

replace regioncode="330783" if regioncode=="330783" & year==2007

replace regioncode="330784" if regioncode=="330784" & year==2007

replace regioncode="330802" if regioncode=="330802" & year==2007

replace regioncode="330803" if regioncode=="330803" & year==2007

replace regioncode="330822" if regioncode=="330822" & year==2007

replace regioncode="330824" if regioncode=="330824" & year==2007

replace regioncode="330825" if regioncode=="330825" & year==2007

replace regioncode="330881" if regioncode=="330881" & year==2007

replace regioncode="330902" if regioncode=="330902" & year==2007

replace regioncode="330903" if regioncode=="330903" & year==2007

replace regioncode="330921" if regioncode=="330921" & year==2007

replace regioncode="330922" if regioncode=="330922" & year==2007

replace regioncode="331002" if regioncode=="331002" & year==2007

replace regioncode="331003" if regioncode=="331003" & year==2007

replace regioncode="331004" if regioncode=="331004" & year==2007

replace regioncode="331021" if regioncode=="331021" & year==2007

replace regioncode="331022" if regioncode=="331022" & year==2007

replace regioncode="331023" if regioncode=="331023" & year==2007

replace regioncode="331024" if regioncode=="331024" & year==2007

replace regioncode="331081" if regioncode=="331081" & year==2007

replace regioncode="331082" if regioncode=="331082" & year==2007

replace regioncode="331102" if regioncode=="331102" & year==2007

replace regioncode="331121" if regioncode=="331121" & year==2007

replace regioncode="331122" if regioncode=="331122" & year==2007

replace regioncode="331123" if regioncode=="331123" & year==2007

replace regioncode="331124" if regioncode=="331124" & year==2007

replace regioncode="331125" if regioncode=="331125" & year==2007

replace regioncode="331126" if regioncode=="331126" & year==2007

replace regioncode="331127" if regioncode=="331127" & year==2007

replace regioncode="331181" if regioncode=="331181" & year==2007

replace regioncode="340102" if regioncode=="340102" & year==2007

replace regioncode="340103" if regioncode=="340103" & year==2007

replace regioncode="340104" if regioncode=="340104" & year==2007

replace regioncode="340111" if regioncode=="340111" & year==2007

replace regioncode="340121" if regioncode=="340121" & year==2007

replace regioncode="340122" if regioncode=="340122" & year==2007

replace regioncode="340123" if regioncode=="340123" & year==2007

replace regioncode="340202" if regioncode=="340202" & year==2007

replace regioncode="340203" if regioncode=="340203" & year==2007

replace regioncode="340204" if regioncode=="340204" & year==2007

replace regioncode="340207" if regioncode=="340207" & year==2007

replace regioncode="340221" if regioncode=="340221" & year==2007

replace regioncode="340222" if regioncode=="340222" & year==2007

replace regioncode="340223" if regioncode=="340223" & year==2007

replace regioncode="340302" if regioncode=="340302" & year==2007

replace regioncode="340303" if regioncode=="340303" & year==2007

replace regioncode="340304" if regioncode=="340304" & year==2007

replace regioncode="340311" if regioncode=="340311" & year==2007

replace regioncode="340321" if regioncode=="340321" & year==2007

replace regioncode="340322" if regioncode=="340322" & year==2007

replace regioncode="340323" if regioncode=="340323" & year==2007

replace regioncode="340402" if regioncode=="340402" & year==2007

replace regioncode="340403" if regioncode=="340403" & year==2007

replace regioncode="340404" if regioncode=="340404" & year==2007

replace regioncode="340405" if regioncode=="340405" & year==2007

replace regioncode="340406" if regioncode=="340406" & year==2007

replace regioncode="340421" if regioncode=="340421" & year==2007

replace regioncode="340502" if regioncode=="340502" & year==2007

replace regioncode="340503" if regioncode=="340503" & year==2007

replace regioncode="340504" if regioncode=="340504" & year==2007

replace regioncode="340521" if regioncode=="340521" & year==2007

replace regioncode="340602" if regioncode=="340602" & year==2007

replace regioncode="340603" if regioncode=="340603" & year==2007

replace regioncode="340604" if regioncode=="340604" & year==2007

replace regioncode="340621" if regioncode=="340621" & year==2007

replace regioncode="340702" if regioncode=="340702" & year==2007

replace regioncode="340703" if regioncode=="340703" & year==2007

replace regioncode="340711" if regioncode=="340711" & year==2007

replace regioncode="340721" if regioncode=="340721" & year==2007

replace regioncode="340802" if regioncode=="340802" & year==2007

replace regioncode="340803" if regioncode=="340803" & year==2007

replace regioncode="340811" if regioncode=="340811" & year==2007

replace regioncode="340822" if regioncode=="340822" & year==2007

replace regioncode="340823" if regioncode=="340823" & year==2007

replace regioncode="340824" if regioncode=="340824" & year==2007

replace regioncode="340825" if regioncode=="340825" & year==2007

replace regioncode="340826" if regioncode=="340826" & year==2007

replace regioncode="340827" if regioncode=="340827" & year==2007

replace regioncode="340828" if regioncode=="340828" & year==2007

replace regioncode="340881" if regioncode=="340881" & year==2007

replace regioncode="341002" if regioncode=="341002" & year==2007

replace regioncode="341003" if regioncode=="341003" & year==2007

replace regioncode="341004" if regioncode=="341004" & year==2007

replace regioncode="341021" if regioncode=="341021" & year==2007

replace regioncode="341022" if regioncode=="341022" & year==2007

replace regioncode="341023" if regioncode=="341023" & year==2007

replace regioncode="341024" if regioncode=="341024" & year==2007

replace regioncode="341102" if regioncode=="341102" & year==2007

replace regioncode="341103" if regioncode=="341103" & year==2007

replace regioncode="341122" if regioncode=="341122" & year==2007

replace regioncode="341124" if regioncode=="341124" & year==2007

replace regioncode="341125" if regioncode=="341125" & year==2007

replace regioncode="341126" if regioncode=="341126" & year==2007

replace regioncode="341181" if regioncode=="341181" & year==2007

replace regioncode="341182" if regioncode=="341182" & year==2007

replace regioncode="341202" if regioncode=="341202" & year==2007

replace regioncode="341203" if regioncode=="341203" & year==2007

replace regioncode="341204" if regioncode=="341204" & year==2007

replace regioncode="341221" if regioncode=="341221" & year==2007

replace regioncode="341222" if regioncode=="341222" & year==2007

replace regioncode="341621" if regioncode=="341621" & year==2007

replace regioncode="341622" if regioncode=="341622" & year==2007

replace regioncode="341225" if regioncode=="341225" & year==2007

replace regioncode="341226" if regioncode=="341226" & year==2007

replace regioncode="341623" if regioncode=="341623" & year==2007

replace regioncode="341282" if regioncode=="341282" & year==2007

replace regioncode="341302" if regioncode=="341302" & year==2007

replace regioncode="341321" if regioncode=="341321" & year==2007

replace regioncode="341322" if regioncode=="341322" & year==2007

replace regioncode="341323" if regioncode=="341323" & year==2007

replace regioncode="341324" if regioncode=="341324" & year==2007

replace regioncode="341402" if regioncode=="341402" & year==2007

replace regioncode="341421" if regioncode=="341421" & year==2007

replace regioncode="341422" if regioncode=="341422" & year==2007

replace regioncode="341423" if regioncode=="341423" & year==2007

replace regioncode="341424" if regioncode=="341424" & year==2007

replace regioncode="341521" if regioncode=="341521" & year==2007

replace regioncode="341522" if regioncode=="341522" & year==2007

replace regioncode="341523" if regioncode=="341523" & year==2007

replace regioncode="341524" if regioncode=="341524" & year==2007

replace regioncode="341525" if regioncode=="341525" & year==2007

replace regioncode="341802" if regioncode=="341802" & year==2007

replace regioncode="341881" if regioncode=="341881" & year==2007

replace regioncode="341821" if regioncode=="341821" & year==2007

replace regioncode="341822" if regioncode=="341822" & year==2007

replace regioncode="341823" if regioncode=="341823" & year==2007

replace regioncode="341825" if regioncode=="341825" & year==2007

replace regioncode="341824" if regioncode=="341824" & year==2007

replace regioncode="341702" if regioncode=="341702" & year==2007

replace regioncode="341721" if regioncode=="341721" & year==2007

replace regioncode="341722" if regioncode=="341722" & year==2007

replace regioncode="341723" if regioncode=="341723" & year==2007

replace regioncode="341502" if regioncode=="341502" & year==2007

replace regioncode="341602" if regioncode=="341602" & year==2007

replace regioncode="350102" if regioncode=="350102" & year==2007

replace regioncode="350103" if regioncode=="350103" & year==2007

replace regioncode="350104" if regioncode=="350104" & year==2007

replace regioncode="350105" if regioncode=="350105" & year==2007

replace regioncode="350111" if regioncode=="350111" & year==2007

replace regioncode="350121" if regioncode=="350121" & year==2007

replace regioncode="350122" if regioncode=="350122" & year==2007

replace regioncode="350123" if regioncode=="350123" & year==2007

replace regioncode="350124" if regioncode=="350124" & year==2007

replace regioncode="350125" if regioncode=="350125" & year==2007

replace regioncode="350128" if regioncode=="350128" & year==2007

replace regioncode="350181" if regioncode=="350181" & year==2007

replace regioncode="350182" if regioncode=="350182" & year==2007

replace regioncode="350203" if regioncode=="350203" & year==2007

replace regioncode="350205" if regioncode=="350205" & year==2007

replace regioncode="350206" if regioncode=="350206" & year==2007

replace regioncode="350211" if regioncode=="350211" & year==2007

replace regioncode="350212" if regioncode=="350212" & year==2007

replace regioncode="350213" if regioncode=="350213" & year==2007

replace regioncode="350302" if regioncode=="350302" & year==2007

replace regioncode="350303" if regioncode=="350303" & year==2007

replace regioncode="350321" if regioncode=="350321" & year==2007

replace regioncode="350322" if regioncode=="350322" & year==2007

replace regioncode="350402" if regioncode=="350402" & year==2007

replace regioncode="350403" if regioncode=="350403" & year==2007

replace regioncode="350421" if regioncode=="350421" & year==2007

replace regioncode="350423" if regioncode=="350423" & year==2007

replace regioncode="350424" if regioncode=="350424" & year==2007

replace regioncode="350425" if regioncode=="350425" & year==2007

replace regioncode="350426" if regioncode=="350426" & year==2007

replace regioncode="350427" if regioncode=="350427" & year==2007

replace regioncode="350428" if regioncode=="350428" & year==2007

replace regioncode="350429" if regioncode=="350429" & year==2007

replace regioncode="350430" if regioncode=="350430" & year==2007

replace regioncode="350481" if regioncode=="350481" & year==2007

replace regioncode="350502" if regioncode=="350502" & year==2007

replace regioncode="350503" if regioncode=="350503" & year==2007

replace regioncode="350504" if regioncode=="350504" & year==2007

replace regioncode="350505" if regioncode=="350505" & year==2007

replace regioncode="350521" if regioncode=="350521" & year==2007

replace regioncode="350524" if regioncode=="350524" & year==2007

replace regioncode="350525" if regioncode=="350525" & year==2007

replace regioncode="350526" if regioncode=="350526" & year==2007

replace regioncode="350581" if regioncode=="350581" & year==2007

replace regioncode="350582" if regioncode=="350582" & year==2007

replace regioncode="350583" if regioncode=="350583" & year==2007

replace regioncode="350602" if regioncode=="350602" & year==2007

replace regioncode="350603" if regioncode=="350603" & year==2007

replace regioncode="350622" if regioncode=="350622" & year==2007

replace regioncode="350623" if regioncode=="350623" & year==2007

replace regioncode="350624" if regioncode=="350624" & year==2007

replace regioncode="350625" if regioncode=="350625" & year==2007

replace regioncode="350626" if regioncode=="350626" & year==2007

replace regioncode="350627" if regioncode=="350627" & year==2007

replace regioncode="350628" if regioncode=="350628" & year==2007

replace regioncode="350629" if regioncode=="350629" & year==2007

replace regioncode="350681" if regioncode=="350681" & year==2007

replace regioncode="350702" if regioncode=="350702" & year==2007

replace regioncode="350721" if regioncode=="350721" & year==2007

replace regioncode="350722" if regioncode=="350722" & year==2007

replace regioncode="350723" if regioncode=="350723" & year==2007

replace regioncode="350724" if regioncode=="350724" & year==2007

replace regioncode="350725" if regioncode=="350725" & year==2007

replace regioncode="350781" if regioncode=="350781" & year==2007

replace regioncode="350782" if regioncode=="350782" & year==2007

replace regioncode="350783" if regioncode=="350783" & year==2007

replace regioncode="350784" if regioncode=="350784" & year==2007

replace regioncode="350802" if regioncode=="350802" & year==2007

replace regioncode="350821" if regioncode=="350821" & year==2007

replace regioncode="350822" if regioncode=="350822" & year==2007

replace regioncode="350823" if regioncode=="350823" & year==2007

replace regioncode="350824" if regioncode=="350824" & year==2007

replace regioncode="350825" if regioncode=="350825" & year==2007

replace regioncode="350881" if regioncode=="350881" & year==2007

replace regioncode="350902" if regioncode=="350902" & year==2007

replace regioncode="350981" if regioncode=="350981" & year==2007

replace regioncode="350982" if regioncode=="350982" & year==2007

replace regioncode="350921" if regioncode=="350921" & year==2007

replace regioncode="350922" if regioncode=="350922" & year==2007

replace regioncode="350923" if regioncode=="350923" & year==2007

replace regioncode="350924" if regioncode=="350924" & year==2007

replace regioncode="350925" if regioncode=="350925" & year==2007

replace regioncode="350926" if regioncode=="350926" & year==2007

replace regioncode="360102" if regioncode=="360102" & year==2007

replace regioncode="360103" if regioncode=="360103" & year==2007

replace regioncode="360104" if regioncode=="360104" & year==2007

replace regioncode="360105" if regioncode=="360105" & year==2007

replace regioncode="360111" if regioncode=="360111" & year==2007

replace regioncode="360121" if regioncode=="360121" & year==2007

replace regioncode="360122" if regioncode=="360122" & year==2007

replace regioncode="360123" if regioncode=="360123" & year==2007

replace regioncode="360124" if regioncode=="360124" & year==2007

replace regioncode="360202" if regioncode=="360202" & year==2007

replace regioncode="360203" if regioncode=="360203" & year==2007

replace regioncode="360222" if regioncode=="360222" & year==2007

replace regioncode="360281" if regioncode=="360281" & year==2007

replace regioncode="360302" if regioncode=="360302" & year==2007

replace regioncode="360313" if regioncode=="360313" & year==2007

replace regioncode="360321" if regioncode=="360321" & year==2007

replace regioncode="360322" if regioncode=="360322" & year==2007

replace regioncode="360323" if regioncode=="360323" & year==2007

replace regioncode="360402" if regioncode=="360402" & year==2007

replace regioncode="360403" if regioncode=="360403" & year==2007

replace regioncode="360421" if regioncode=="360421" & year==2007

replace regioncode="360423" if regioncode=="360423" & year==2007

replace regioncode="360424" if regioncode=="360424" & year==2007

replace regioncode="360425" if regioncode=="360425" & year==2007

replace regioncode="360426" if regioncode=="360426" & year==2007

replace regioncode="360427" if regioncode=="360427" & year==2007

replace regioncode="360428" if regioncode=="360428" & year==2007

replace regioncode="360429" if regioncode=="360429" & year==2007

replace regioncode="360430" if regioncode=="360430" & year==2007

replace regioncode="360481" if regioncode=="360481" & year==2007

replace regioncode="360502" if regioncode=="360502" & year==2007

replace regioncode="360521" if regioncode=="360521" & year==2007

replace regioncode="360602" if regioncode=="360602" & year==2007

replace regioncode="360622" if regioncode=="360622" & year==2007

replace regioncode="360681" if regioncode=="360681" & year==2007

replace regioncode="360702" if regioncode=="360702" & year==2007

replace regioncode="360721" if regioncode=="360721" & year==2007

replace regioncode="360722" if regioncode=="360722" & year==2007

replace regioncode="360723" if regioncode=="360723" & year==2007

replace regioncode="360724" if regioncode=="360724" & year==2007

replace regioncode="360725" if regioncode=="360725" & year==2007

replace regioncode="360726" if regioncode=="360726" & year==2007

replace regioncode="360727" if regioncode=="360727" & year==2007

replace regioncode="360728" if regioncode=="360728" & year==2007

replace regioncode="360729" if regioncode=="360729" & year==2007

replace regioncode="360730" if regioncode=="360730" & year==2007

replace regioncode="360731" if regioncode=="360731" & year==2007

replace regioncode="360732" if regioncode=="360732" & year==2007

replace regioncode="360733" if regioncode=="360733" & year==2007

replace regioncode="360734" if regioncode=="360734" & year==2007

replace regioncode="360735" if regioncode=="360735" & year==2007

replace regioncode="360781" if regioncode=="360781" & year==2007

replace regioncode="360782" if regioncode=="360782" & year==2007

replace regioncode="360802" if regioncode=="360802" & year==2007

replace regioncode="360821" if regioncode=="360821" & year==2007

replace regioncode="360822" if regioncode=="360822" & year==2007

replace regioncode="360823" if regioncode=="360823" & year==2007

replace regioncode="360824" if regioncode=="360824" & year==2007

replace regioncode="360825" if regioncode=="360825" & year==2007

replace regioncode="360826" if regioncode=="360826" & year==2007

replace regioncode="360827" if regioncode=="360827" & year==2007

replace regioncode="360828" if regioncode=="360828" & year==2007

replace regioncode="360829" if regioncode=="360829" & year==2007

replace regioncode="360830" if regioncode=="360830" & year==2007

replace regioncode="360881" if regioncode=="360881" & year==2007

replace regioncode="360902" if regioncode=="360902" & year==2007

replace regioncode="360921" if regioncode=="360921" & year==2007

replace regioncode="360922" if regioncode=="360922" & year==2007

replace regioncode="360923" if regioncode=="360923" & year==2007

replace regioncode="360924" if regioncode=="360924" & year==2007

replace regioncode="360925" if regioncode=="360925" & year==2007

replace regioncode="360926" if regioncode=="360926" & year==2007

replace regioncode="360981" if regioncode=="360981" & year==2007

replace regioncode="360982" if regioncode=="360982" & year==2007

replace regioncode="360983" if regioncode=="360983" & year==2007

replace regioncode="361002" if regioncode=="361002" & year==2007

replace regioncode="361021" if regioncode=="361021" & year==2007

replace regioncode="361022" if regioncode=="361022" & year==2007

replace regioncode="361023" if regioncode=="361023" & year==2007

replace regioncode="361024" if regioncode=="361024" & year==2007

replace regioncode="361025" if regioncode=="361025" & year==2007

replace regioncode="361026" if regioncode=="361026" & year==2007

replace regioncode="361027" if regioncode=="361027" & year==2007

replace regioncode="361028" if regioncode=="361028" & year==2007

replace regioncode="361029" if regioncode=="361029" & year==2007

replace regioncode="361030" if regioncode=="361030" & year==2007

replace regioncode="361102" if regioncode=="361102" & year==2007

replace regioncode="361121" if regioncode=="361121" & year==2007

replace regioncode="361122" if regioncode=="361122" & year==2007

replace regioncode="361123" if regioncode=="361123" & year==2007

replace regioncode="361124" if regioncode=="361124" & year==2007

replace regioncode="361125" if regioncode=="361125" & year==2007

replace regioncode="361126" if regioncode=="361126" & year==2007

replace regioncode="361127" if regioncode=="361127" & year==2007

replace regioncode="361128" if regioncode=="361128" & year==2007

replace regioncode="361129" if regioncode=="361129" & year==2007

replace regioncode="361130" if regioncode=="361130" & year==2007

replace regioncode="361181" if regioncode=="361181" & year==2007

replace regioncode="370102" if regioncode=="370102" & year==2007

replace regioncode="370103" if regioncode=="370103" & year==2007

replace regioncode="370104" if regioncode=="370104" & year==2007

replace regioncode="370105" if regioncode=="370105" & year==2007

replace regioncode="370112" if regioncode=="370112" & year==2007

replace regioncode="370113" if regioncode=="370113" & year==2007

replace regioncode="370124" if regioncode=="370124" & year==2007

replace regioncode="370125" if regioncode=="370125" & year==2007

replace regioncode="370126" if regioncode=="370126" & year==2007

replace regioncode="370181" if regioncode=="370181" & year==2007

replace regioncode="370202" if regioncode=="370202" & year==2007

replace regioncode="370203" if regioncode=="370203" & year==2007

replace regioncode="370205" if regioncode=="370205" & year==2007

replace regioncode="370211" if regioncode=="370211" & year==2007

replace regioncode="370212" if regioncode=="370212" & year==2007

replace regioncode="370213" if regioncode=="370213" & year==2007

replace regioncode="370214" if regioncode=="370214" & year==2007

replace regioncode="370281" if regioncode=="370281" & year==2007

replace regioncode="370282" if regioncode=="370282" & year==2007

replace regioncode="370283" if regioncode=="370283" & year==2007

replace regioncode="370284" if regioncode=="370284" & year==2007

replace regioncode="370285" if regioncode=="370285" & year==2007

replace regioncode="370302" if regioncode=="370302" & year==2007

replace regioncode="370303" if regioncode=="370303" & year==2007

replace regioncode="370304" if regioncode=="370304" & year==2007

replace regioncode="370305" if regioncode=="370305" & year==2007

replace regioncode="370306" if regioncode=="370306" & year==2007

replace regioncode="370321" if regioncode=="370321" & year==2007

replace regioncode="370322" if regioncode=="370322" & year==2007

replace regioncode="370323" if regioncode=="370323" & year==2007

replace regioncode="370402" if regioncode=="370402" & year==2007

replace regioncode="370403" if regioncode=="370403" & year==2007

replace regioncode="370404" if regioncode=="370404" & year==2007

replace regioncode="370405" if regioncode=="370405" & year==2007

replace regioncode="370406" if regioncode=="370406" & year==2007

replace regioncode="370481" if regioncode=="370481" & year==2007

replace regioncode="370502" if regioncode=="370502" & year==2007

replace regioncode="370503" if regioncode=="370503" & year==2007

replace regioncode="370521" if regioncode=="370521" & year==2007

replace regioncode="370522" if regioncode=="370522" & year==2007

replace regioncode="370523" if regioncode=="370523" & year==2007

replace regioncode="370602" if regioncode=="370602" & year==2007

replace regioncode="370611" if regioncode=="370611" & year==2007

replace regioncode="370612" if regioncode=="370612" & year==2007

replace regioncode="370613" if regioncode=="370613" & year==2007

replace regioncode="370634" if regioncode=="370634" & year==2007

replace regioncode="370681" if regioncode=="370681" & year==2007

replace regioncode="370682" if regioncode=="370682" & year==2007

replace regioncode="370683" if regioncode=="370683" & year==2007

replace regioncode="370684" if regioncode=="370684" & year==2007

replace regioncode="370685" if regioncode=="370685" & year==2007

replace regioncode="370686" if regioncode=="370686" & year==2007

replace regioncode="370687" if regioncode=="370687" & year==2007

replace regioncode="370702" if regioncode=="370702" & year==2007

replace regioncode="370703" if regioncode=="370703" & year==2007

replace regioncode="370704" if regioncode=="370704" & year==2007

replace regioncode="370705" if regioncode=="370705" & year==2007

replace regioncode="370724" if regioncode=="370724" & year==2007

replace regioncode="370725" if regioncode=="370725" & year==2007

replace regioncode="370781" if regioncode=="370781" & year==2007

replace regioncode="370782" if regioncode=="370782" & year==2007

replace regioncode="370783" if regioncode=="370783" & year==2007

replace regioncode="370784" if regioncode=="370784" & year==2007

replace regioncode="370785" if regioncode=="370785" & year==2007

replace regioncode="370786" if regioncode=="370786" & year==2007

replace regioncode="370802" if regioncode=="370802" & year==2007

replace regioncode="370811" if regioncode=="370811" & year==2007

replace regioncode="370826" if regioncode=="370826" & year==2007

replace regioncode="370827" if regioncode=="370827" & year==2007

replace regioncode="370828" if regioncode=="370828" & year==2007

replace regioncode="370829" if regioncode=="370829" & year==2007

replace regioncode="370830" if regioncode=="370830" & year==2007

replace regioncode="370831" if regioncode=="370831" & year==2007

replace regioncode="370832" if regioncode=="370832" & year==2007

replace regioncode="370881" if regioncode=="370881" & year==2007

replace regioncode="370882" if regioncode=="370882" & year==2007

replace regioncode="370883" if regioncode=="370883" & year==2007

replace regioncode="370902" if regioncode=="370902" & year==2007

replace regioncode="370903" if regioncode=="370911" & year==2007

replace regioncode="370921" if regioncode=="370921" & year==2007

replace regioncode="370923" if regioncode=="370923" & year==2007

replace regioncode="370982" if regioncode=="370982" & year==2007

replace regioncode="370983" if regioncode=="370983" & year==2007

replace regioncode="371002" if regioncode=="371002" & year==2007

replace regioncode="371081" if regioncode=="371081" & year==2007

replace regioncode="371082" if regioncode=="371082" & year==2007

replace regioncode="371083" if regioncode=="371083" & year==2007

replace regioncode="371102" if regioncode=="371102" & year==2007

replace regioncode="371121" if regioncode=="371121" & year==2007

replace regioncode="371122" if regioncode=="371122" & year==2007

replace regioncode="371202" if regioncode=="371202" & year==2007

replace regioncode="371203" if regioncode=="371203" & year==2007

replace regioncode="371302" if regioncode=="371302" & year==2007

replace regioncode="371311" if regioncode=="371311" & year==2007

replace regioncode="371312" if regioncode=="371312" & year==2007

replace regioncode="371321" if regioncode=="371321" & year==2007

replace regioncode="371322" if regioncode=="371322" & year==2007

replace regioncode="371323" if regioncode=="371323" & year==2007

replace regioncode="371324" if regioncode=="371324" & year==2007

replace regioncode="371325" if regioncode=="371325" & year==2007

replace regioncode="371326" if regioncode=="371326" & year==2007

replace regioncode="371327" if regioncode=="371327" & year==2007

replace regioncode="371328" if regioncode=="371328" & year==2007

replace regioncode="371329" if regioncode=="371329" & year==2007

replace regioncode="371402" if regioncode=="371402" & year==2007

replace regioncode="371421" if regioncode=="371421" & year==2007

replace regioncode="371422" if regioncode=="371422" & year==2007

replace regioncode="371423" if regioncode=="371423" & year==2007

replace regioncode="371424" if regioncode=="371424" & year==2007

replace regioncode="371425" if regioncode=="371425" & year==2007

replace regioncode="371426" if regioncode=="371426" & year==2007

replace regioncode="371427" if regioncode=="371427" & year==2007

replace regioncode="371428" if regioncode=="371428" & year==2007

replace regioncode="371481" if regioncode=="371481" & year==2007

replace regioncode="371482" if regioncode=="371482" & year==2007

replace regioncode="371502" if regioncode=="371502" & year==2007

replace regioncode="371521" if regioncode=="371521" & year==2007

replace regioncode="371522" if regioncode=="371522" & year==2007

replace regioncode="371523" if regioncode=="371523" & year==2007

replace regioncode="371524" if regioncode=="371524" & year==2007

replace regioncode="371525" if regioncode=="371525" & year==2007

replace regioncode="371526" if regioncode=="371526" & year==2007

replace regioncode="371581" if regioncode=="371581" & year==2007

replace regioncode="371602" if regioncode=="371602" & year==2007

replace regioncode="371621" if regioncode=="371621" & year==2007

replace regioncode="371622" if regioncode=="371622" & year==2007

replace regioncode="371623" if regioncode=="371623" & year==2007

replace regioncode="371624" if regioncode=="371624" & year==2007

replace regioncode="371625" if regioncode=="371625" & year==2007

replace regioncode="371626" if regioncode=="371626" & year==2007

replace regioncode="371702" if regioncode=="371702" & year==2007

replace regioncode="371721" if regioncode=="371721" & year==2007

replace regioncode="371722" if regioncode=="371722" & year==2007

replace regioncode="371723" if regioncode=="371723" & year==2007

replace regioncode="371724" if regioncode=="371724" & year==2007

replace regioncode="371725" if regioncode=="371725" & year==2007

replace regioncode="371726" if regioncode=="371726" & year==2007

replace regioncode="371727" if regioncode=="371727" & year==2007

replace regioncode="371728" if regioncode=="371728" & year==2007

replace regioncode="410102" if regioncode=="410102" & year==2007

replace regioncode="410103" if regioncode=="410103" & year==2007

replace regioncode="410104" if regioncode=="410104" & year==2007

replace regioncode="410105" if regioncode=="410105" & year==2007

replace regioncode="410106" if regioncode=="410106" & year==2007

replace regioncode="410108" if regioncode=="410108" & year==2007

replace regioncode="410122" if regioncode=="410122" & year==2007

replace regioncode="410181" if regioncode=="410181" & year==2007

replace regioncode="410182" if regioncode=="410182" & year==2007

replace regioncode="410183" if regioncode=="410183" & year==2007

replace regioncode="410184" if regioncode=="410184" & year==2007

replace regioncode="410185" if regioncode=="410185" & year==2007

replace regioncode="410202" if regioncode=="410202" & year==2007

replace regioncode="410203" if regioncode=="410203" & year==2007

replace regioncode="410204" if regioncode=="410204" & year==2007

replace regioncode="410205" if regioncode=="410205" & year==2007

replace regioncode="410211" if regioncode=="410211" & year==2007

replace regioncode="410221" if regioncode=="410221" & year==2007

replace regioncode="410222" if regioncode=="410222" & year==2007

replace regioncode="410223" if regioncode=="410223" & year==2007

replace regioncode="410224" if regioncode=="410224" & year==2007

replace regioncode="410225" if regioncode=="410225" & year==2007

replace regioncode="410302" if regioncode=="410302" & year==2007

replace regioncode="410303" if regioncode=="410303" & year==2007

replace regioncode="410304" if regioncode=="410304" & year==2007

replace regioncode="410305" if regioncode=="410305" & year==2007

replace regioncode="410306" if regioncode=="410306" & year==2007

replace regioncode="410307" if regioncode=="410307" & year==2007

replace regioncode="410322" if regioncode=="410322" & year==2007

replace regioncode="410323" if regioncode=="410323" & year==2007

replace regioncode="410324" if regioncode=="410324" & year==2007

replace regioncode="410325" if regioncode=="410325" & year==2007

replace regioncode="410326" if regioncode=="410326" & year==2007

replace regioncode="410327" if regioncode=="410327" & year==2007

replace regioncode="410328" if regioncode=="410328" & year==2007

replace regioncode="410329" if regioncode=="410329" & year==2007

replace regioncode="410381" if regioncode=="410381" & year==2007

replace regioncode="410402" if regioncode=="410402" & year==2007

replace regioncode="410403" if regioncode=="410403" & year==2007

replace regioncode="410404" if regioncode=="410404" & year==2007

replace regioncode="410411" if regioncode=="410411" & year==2007

replace regioncode="410421" if regioncode=="410421" & year==2007

replace regioncode="410422" if regioncode=="410422" & year==2007

replace regioncode="410423" if regioncode=="410423" & year==2007

replace regioncode="410425" if regioncode=="410425" & year==2007

replace regioncode="410481" if regioncode=="410481" & year==2007

replace regioncode="410482" if regioncode=="410482" & year==2007

replace regioncode="410502" if regioncode=="410502" & year==2007

replace regioncode="410503" if regioncode=="410503" & year==2007

replace regioncode="410504" if regioncode=="410505" & year==2007

replace regioncode="410511" if regioncode=="410506" & year==2007

replace regioncode="410522" if regioncode=="410522" & year==2007

replace regioncode="410523" if regioncode=="410523" & year==2007

replace regioncode="410526" if regioncode=="410526" & year==2007

replace regioncode="410527" if regioncode=="410527" & year==2007

replace regioncode="410581" if regioncode=="410581" & year==2007

replace regioncode="410602" if regioncode=="410602" & year==2007

replace regioncode="410603" if regioncode=="410603" & year==2007

replace regioncode="410611" if regioncode=="410611" & year==2007

replace regioncode="410621" if regioncode=="410621" & year==2007

replace regioncode="410622" if regioncode=="410622" & year==2007

replace regioncode="410702" if regioncode=="410702" & year==2007

replace regioncode="410703" if regioncode=="410703" & year==2007

replace regioncode="410704" if regioncode=="410704" & year==2007

replace regioncode="410711" if regioncode=="410711" & year==2007

replace regioncode="410721" if regioncode=="410721" & year==2007

replace regioncode="410724" if regioncode=="410724" & year==2007

replace regioncode="410725" if regioncode=="410725" & year==2007

replace regioncode="410726" if regioncode=="410726" & year==2007

replace regioncode="410727" if regioncode=="410727" & year==2007

replace regioncode="410728" if regioncode=="410728" & year==2007

replace regioncode="410781" if regioncode=="410781" & year==2007

replace regioncode="410782" if regioncode=="410782" & year==2007

replace regioncode="410802" if regioncode=="410802" & year==2007

replace regioncode="410803" if regioncode=="410803" & year==2007

replace regioncode="410804" if regioncode=="410804" & year==2007

replace regioncode="410811" if regioncode=="410811" & year==2007

replace regioncode="410821" if regioncode=="410821" & year==2007

replace regioncode="410822" if regioncode=="410822" & year==2007

replace regioncode="410823" if regioncode=="410823" & year==2007

replace regioncode="410825" if regioncode=="410825" & year==2007

replace regioncode="410881" if regioncode=="410881" & year==2007

replace regioncode="410882" if regioncode=="410882" & year==2007

replace regioncode="410883" if regioncode=="410883" & year==2007

replace regioncode="410902" if regioncode=="410902" & year==2007

replace regioncode="410922" if regioncode=="410922" & year==2007

replace regioncode="410923" if regioncode=="410923" & year==2007

replace regioncode="410926" if regioncode=="410926" & year==2007

replace regioncode="410927" if regioncode=="410927" & year==2007

replace regioncode="410928" if regioncode=="410928" & year==2007

replace regioncode="411002" if regioncode=="411002" & year==2007

replace regioncode="411023" if regioncode=="411023" & year==2007

replace regioncode="411024" if regioncode=="411024" & year==2007

replace regioncode="411025" if regioncode=="411025" & year==2007

replace regioncode="411081" if regioncode=="411081" & year==2007

replace regioncode="411082" if regioncode=="411082" & year==2007

replace regioncode="411102" if regioncode=="411102" & year==2007

replace regioncode="411123" if regioncode=="411103" & year==2007

replace regioncode="411121" if regioncode=="411121" & year==2007

replace regioncode="411122" if regioncode=="411122" & year==2007

replace regioncode="411202" if regioncode=="411202" & year==2007

replace regioncode="411221" if regioncode=="411221" & year==2007

replace regioncode="411222" if regioncode=="411222" & year==2007

replace regioncode="411224" if regioncode=="411224" & year==2007

replace regioncode="411281" if regioncode=="411281" & year==2007

replace regioncode="411282" if regioncode=="411282" & year==2007

replace regioncode="411302" if regioncode=="411302" & year==2007

replace regioncode="411303" if regioncode=="411303" & year==2007

replace regioncode="411321" if regioncode=="411321" & year==2007

replace regioncode="411322" if regioncode=="411322" & year==2007

replace regioncode="411323" if regioncode=="411323" & year==2007

replace regioncode="411324" if regioncode=="411324" & year==2007

replace regioncode="411325" if regioncode=="411325" & year==2007

replace regioncode="411326" if regioncode=="411326" & year==2007

replace regioncode="411327" if regioncode=="411327" & year==2007

replace regioncode="411328" if regioncode=="411328" & year==2007

replace regioncode="411329" if regioncode=="411329" & year==2007

replace regioncode="411330" if regioncode=="411330" & year==2007

replace regioncode="411381" if regioncode=="411381" & year==2007

replace regioncode="411402" if regioncode=="411402" & year==2007

replace regioncode="411403" if regioncode=="411403" & year==2007

replace regioncode="411421" if regioncode=="411421" & year==2007

replace regioncode="411422" if regioncode=="411422" & year==2007

replace regioncode="411423" if regioncode=="411423" & year==2007

replace regioncode="411424" if regioncode=="411424" & year==2007

replace regioncode="411425" if regioncode=="411425" & year==2007

replace regioncode="411426" if regioncode=="411426" & year==2007

replace regioncode="411481" if regioncode=="411481" & year==2007

replace regioncode="411502" if regioncode=="411502" & year==2007

replace regioncode="411503" if regioncode=="411503" & year==2007

replace regioncode="411521" if regioncode=="411521" & year==2007

replace regioncode="411522" if regioncode=="411522" & year==2007

replace regioncode="411523" if regioncode=="411523" & year==2007

replace regioncode="411524" if regioncode=="411524" & year==2007

replace regioncode="411525" if regioncode=="411525" & year==2007

replace regioncode="411526" if regioncode=="411526" & year==2007

replace regioncode="411527" if regioncode=="411527" & year==2007

replace regioncode="411528" if regioncode=="411528" & year==2007

replace regioncode="411602" if regioncode=="411602" & year==2007

replace regioncode="411621" if regioncode=="411621" & year==2007

replace regioncode="411622" if regioncode=="411622" & year==2007

replace regioncode="411623" if regioncode=="411623" & year==2007

replace regioncode="411624" if regioncode=="411624" & year==2007

replace regioncode="411625" if regioncode=="411625" & year==2007

replace regioncode="411626" if regioncode=="411626" & year==2007

replace regioncode="411627" if regioncode=="411627" & year==2007

replace regioncode="411628" if regioncode=="411628" & year==2007

replace regioncode="411681" if regioncode=="411681" & year==2007

replace regioncode="411702" if regioncode=="411702" & year==2007

replace regioncode="411721" if regioncode=="411721" & year==2007

replace regioncode="411722" if regioncode=="411722" & year==2007

replace regioncode="411723" if regioncode=="411723" & year==2007

replace regioncode="411724" if regioncode=="411724" & year==2007

replace regioncode="411725" if regioncode=="411725" & year==2007

replace regioncode="411726" if regioncode=="411726" & year==2007

replace regioncode="411727" if regioncode=="411727" & year==2007

replace regioncode="411728" if regioncode=="411728" & year==2007

replace regioncode="411729" if regioncode=="411729" & year==2007

replace regioncode="420102" if regioncode=="420102" & year==2007

replace regioncode="420103" if regioncode=="420103" & year==2007

replace regioncode="420104" if regioncode=="420104" & year==2007

replace regioncode="420105" if regioncode=="420105" & year==2007

replace regioncode="420106" if regioncode=="420106" & year==2007

replace regioncode="420107" if regioncode=="420107" & year==2007

replace regioncode="420111" if regioncode=="420111" & year==2007

replace regioncode="420112" if regioncode=="420112" & year==2007

replace regioncode="420113" if regioncode=="420113" & year==2007

replace regioncode="420114" if regioncode=="420114" & year==2007

replace regioncode="420115" if regioncode=="420115" & year==2007

replace regioncode="420116" if regioncode=="420116" & year==2007

replace regioncode="420117" if regioncode=="420117" & year==2007

replace regioncode="420202" if regioncode=="420202" & year==2007

replace regioncode="420203" if regioncode=="420203" & year==2007

replace regioncode="420204" if regioncode=="420204" & year==2007

replace regioncode="420205" if regioncode=="420205" & year==2007

replace regioncode="420222" if regioncode=="420222" & year==2007

replace regioncode="420281" if regioncode=="420281" & year==2007

replace regioncode="420302" if regioncode=="420302" & year==2007

replace regioncode="420303" if regioncode=="420303" & year==2007

replace regioncode="420321" if regioncode=="420321" & year==2007

replace regioncode="420322" if regioncode=="420322" & year==2007

replace regioncode="420323" if regioncode=="420323" & year==2007

replace regioncode="420324" if regioncode=="420324" & year==2007

replace regioncode="420325" if regioncode=="420325" & year==2007

replace regioncode="420381" if regioncode=="420381" & year==2007

replace regioncode="420502" if regioncode=="420502" & year==2007

replace regioncode="420503" if regioncode=="420503" & year==2007

replace regioncode="420504" if regioncode=="420504" & year==2007

replace regioncode="420505" if regioncode=="420505" & year==2007

replace regioncode="420506" if regioncode=="420506" & year==2007

replace regioncode="420525" if regioncode=="420525" & year==2007

replace regioncode="420526" if regioncode=="420526" & year==2007

replace regioncode="420527" if regioncode=="420527" & year==2007

replace regioncode="420528" if regioncode=="420528" & year==2007

replace regioncode="420529" if regioncode=="420529" & year==2007

replace regioncode="420581" if regioncode=="420581" & year==2007

replace regioncode="420582" if regioncode=="420582" & year==2007

replace regioncode="420583" if regioncode=="420583" & year==2007

replace regioncode="420602" if regioncode=="420602" & year==2007

replace regioncode="420606" if regioncode=="420606" & year==2007

replace regioncode="420607" if regioncode=="420607" & year==2007

replace regioncode="420624" if regioncode=="420624" & year==2007

replace regioncode="420625" if regioncode=="420625" & year==2007

replace regioncode="420626" if regioncode=="420626" & year==2007

replace regioncode="420682" if regioncode=="420682" & year==2007

replace regioncode="420683" if regioncode=="420683" & year==2007

replace regioncode="420684" if regioncode=="420684" & year==2007

replace regioncode="420702" if regioncode=="420702" & year==2007

replace regioncode="420703" if regioncode=="420703" & year==2007

replace regioncode="420704" if regioncode=="420704" & year==2007

replace regioncode="420802" if regioncode=="420802" & year==2007

replace regioncode="420804" if regioncode=="420804" & year==2007

replace regioncode="420821" if regioncode=="420821" & year==2007

replace regioncode="420822" if regioncode=="420822" & year==2007

replace regioncode="420881" if regioncode=="420881" & year==2007

replace regioncode="420902" if regioncode=="420902" & year==2007

replace regioncode="420921" if regioncode=="420921" & year==2007

replace regioncode="420922" if regioncode=="420922" & year==2007

replace regioncode="420923" if regioncode=="420923" & year==2007

replace regioncode="420981" if regioncode=="420981" & year==2007

replace regioncode="420982" if regioncode=="420982" & year==2007

replace regioncode="421381" if regioncode=="421381" & year==2007

replace regioncode="420984" if regioncode=="420984" & year==2007

replace regioncode="421002" if regioncode=="421002" & year==2007

replace regioncode="421003" if regioncode=="421003" & year==2007

replace regioncode="421022" if regioncode=="421022" & year==2007

replace regioncode="421023" if regioncode=="421023" & year==2007

replace regioncode="421024" if regioncode=="421024" & year==2007

replace regioncode="421081" if regioncode=="421081" & year==2007

replace regioncode="421083" if regioncode=="421083" & year==2007

replace regioncode="421087" if regioncode=="421087" & year==2007

replace regioncode="421102" if regioncode=="421102" & year==2007

replace regioncode="421121" if regioncode=="421121" & year==2007

replace regioncode="421122" if regioncode=="421122" & year==2007

replace regioncode="421123" if regioncode=="421123" & year==2007

replace regioncode="421124" if regioncode=="421124" & year==2007

replace regioncode="421125" if regioncode=="421125" & year==2007

replace regioncode="421126" if regioncode=="421126" & year==2007

replace regioncode="421127" if regioncode=="421127" & year==2007

replace regioncode="421181" if regioncode=="421181" & year==2007

replace regioncode="421182" if regioncode=="421182" & year==2007

replace regioncode="421202" if regioncode=="421202" & year==2007

replace regioncode="421221" if regioncode=="421221" & year==2007

replace regioncode="421222" if regioncode=="421222" & year==2007

replace regioncode="421223" if regioncode=="421223" & year==2007

replace regioncode="421224" if regioncode=="421224" & year==2007

replace regioncode="421281" if regioncode=="421281" & year==2007

replace regioncode="421302" if regioncode=="421302" & year==2007

replace regioncode="422801" if regioncode=="422801" & year==2007

replace regioncode="422802" if regioncode=="422802" & year==2007

replace regioncode="422822" if regioncode=="422822" & year==2007

replace regioncode="422823" if regioncode=="422823" & year==2007

replace regioncode="422825" if regioncode=="422825" & year==2007

replace regioncode="422826" if regioncode=="422826" & year==2007

replace regioncode="422827" if regioncode=="422827" & year==2007

replace regioncode="422828" if regioncode=="422828" & year==2007

replace regioncode="429004" if regioncode=="429004" & year==2007

replace regioncode="429005" if regioncode=="429005" & year==2007

replace regioncode="429006" if regioncode=="429006" & year==2007

replace regioncode="429021" if regioncode=="429021" & year==2007

replace regioncode="430102" if regioncode=="430102" & year==2007

replace regioncode="430103" if regioncode=="430103" & year==2007

replace regioncode="430104" if regioncode=="430104" & year==2007

replace regioncode="430105" if regioncode=="430105" & year==2007

replace regioncode="430111" if regioncode=="430111" & year==2007

replace regioncode="430121" if regioncode=="430121" & year==2007

replace regioncode="430122" if regioncode=="430122" & year==2007

replace regioncode="430124" if regioncode=="430124" & year==2007

replace regioncode="430181" if regioncode=="430181" & year==2007

replace regioncode="430202" if regioncode=="430202" & year==2007

replace regioncode="430203" if regioncode=="430203" & year==2007

replace regioncode="430204" if regioncode=="430204" & year==2007

replace regioncode="430211" if regioncode=="430211" & year==2007

replace regioncode="430221" if regioncode=="430221" & year==2007

replace regioncode="430223" if regioncode=="430223" & year==2007

replace regioncode="430224" if regioncode=="430224" & year==2007

replace regioncode="430225" if regioncode=="430225" & year==2007

replace regioncode="430281" if regioncode=="430281" & year==2007

replace regioncode="430302" if regioncode=="430302" & year==2007

replace regioncode="430304" if regioncode=="430304" & year==2007

replace regioncode="430321" if regioncode=="430321" & year==2007

replace regioncode="430381" if regioncode=="430381" & year==2007

replace regioncode="430382" if regioncode=="430382" & year==2007

replace regioncode="430405" if regioncode=="430405" & year==2007

replace regioncode="430406" if regioncode=="430406" & year==2007

replace regioncode="430407" if regioncode=="430407" & year==2007

replace regioncode="430408" if regioncode=="430408" & year==2007

replace regioncode="430412" if regioncode=="430412" & year==2007

replace regioncode="430421" if regioncode=="430421" & year==2007

replace regioncode="430422" if regioncode=="430422" & year==2007

replace regioncode="430423" if regioncode=="430423" & year==2007

replace regioncode="430424" if regioncode=="430424" & year==2007

replace regioncode="430426" if regioncode=="430426" & year==2007

replace regioncode="430481" if regioncode=="430481" & year==2007

replace regioncode="430482" if regioncode=="430482" & year==2007

replace regioncode="430502" if regioncode=="430502" & year==2007

replace regioncode="430503" if regioncode=="430503" & year==2007

replace regioncode="430511" if regioncode=="430511" & year==2007

replace regioncode="430521" if regioncode=="430521" & year==2007

replace regioncode="430522" if regioncode=="430522" & year==2007

replace regioncode="430523" if regioncode=="430523" & year==2007

replace regioncode="430524" if regioncode=="430524" & year==2007

replace regioncode="430525" if regioncode=="430525" & year==2007

replace regioncode="430527" if regioncode=="430527" & year==2007

replace regioncode="430528" if regioncode=="430528" & year==2007

replace regioncode="430529" if regioncode=="430529" & year==2007

replace regioncode="430581" if regioncode=="430581" & year==2007

replace regioncode="430602" if regioncode=="430602" & year==2007

replace regioncode="430603" if regioncode=="430603" & year==2007

replace regioncode="430611" if regioncode=="430611" & year==2007

replace regioncode="430621" if regioncode=="430621" & year==2007

replace regioncode="430623" if regioncode=="430623" & year==2007

replace regioncode="430624" if regioncode=="430624" & year==2007

replace regioncode="430626" if regioncode=="430626" & year==2007

replace regioncode="430681" if regioncode=="430681" & year==2007

replace regioncode="430682" if regioncode=="430682" & year==2007

replace regioncode="430702" if regioncode=="430702" & year==2007

replace regioncode="430703" if regioncode=="430703" & year==2007

replace regioncode="430721" if regioncode=="430721" & year==2007

replace regioncode="430722" if regioncode=="430722" & year==2007

replace regioncode="430723" if regioncode=="430723" & year==2007

replace regioncode="430724" if regioncode=="430724" & year==2007

replace regioncode="430725" if regioncode=="430725" & year==2007

replace regioncode="430726" if regioncode=="430726" & year==2007

replace regioncode="430781" if regioncode=="430781" & year==2007

replace regioncode="430802" if regioncode=="430802" & year==2007

replace regioncode="430811" if regioncode=="430811" & year==2007

replace regioncode="430821" if regioncode=="430821" & year==2007

replace regioncode="430822" if regioncode=="430822" & year==2007

replace regioncode="430902" if regioncode=="430902" & year==2007

replace regioncode="430903" if regioncode=="430903" & year==2007

replace regioncode="430921" if regioncode=="430921" & year==2007

replace regioncode="430922" if regioncode=="430922" & year==2007

replace regioncode="430923" if regioncode=="430923" & year==2007

replace regioncode="430981" if regioncode=="430981" & year==2007

replace regioncode="431002" if regioncode=="431002" & year==2007

replace regioncode="431003" if regioncode=="431003" & year==2007

replace regioncode="431021" if regioncode=="431021" & year==2007

replace regioncode="431022" if regioncode=="431022" & year==2007

replace regioncode="431023" if regioncode=="431023" & year==2007

replace regioncode="431024" if regioncode=="431024" & year==2007

replace regioncode="431025" if regioncode=="431025" & year==2007

replace regioncode="431026" if regioncode=="431026" & year==2007

replace regioncode="431027" if regioncode=="431027" & year==2007

replace regioncode="431028" if regioncode=="431028" & year==2007

replace regioncode="431081" if regioncode=="431081" & year==2007

replace regioncode="431102" if regioncode=="431102" & year==2007

replace regioncode="431103" if regioncode=="431103" & year==2007

replace regioncode="431121" if regioncode=="431121" & year==2007

replace regioncode="431122" if regioncode=="431122" & year==2007

replace regioncode="431123" if regioncode=="431123" & year==2007

replace regioncode="431124" if regioncode=="431124" & year==2007

replace regioncode="431125" if regioncode=="431125" & year==2007

replace regioncode="431126" if regioncode=="431126" & year==2007

replace regioncode="431127" if regioncode=="431127" & year==2007

replace regioncode="431128" if regioncode=="431128" & year==2007

replace regioncode="431129" if regioncode=="431129" & year==2007

replace regioncode="431202" if regioncode=="431202" & year==2007

replace regioncode="431221" if regioncode=="431221" & year==2007

replace regioncode="431222" if regioncode=="431222" & year==2007

replace regioncode="431223" if regioncode=="431223" & year==2007

replace regioncode="431224" if regioncode=="431224" & year==2007

replace regioncode="431225" if regioncode=="431225" & year==2007

replace regioncode="431226" if regioncode=="431226" & year==2007

replace regioncode="431227" if regioncode=="431227" & year==2007

replace regioncode="431228" if regioncode=="431228" & year==2007

replace regioncode="431229" if regioncode=="431229" & year==2007

replace regioncode="431230" if regioncode=="431230" & year==2007

replace regioncode="431281" if regioncode=="431281" & year==2007

replace regioncode="431302" if regioncode=="431302" & year==2007

replace regioncode="431321" if regioncode=="431321" & year==2007

replace regioncode="431322" if regioncode=="431322" & year==2007

replace regioncode="431381" if regioncode=="431381" & year==2007

replace regioncode="431382" if regioncode=="431382" & year==2007

replace regioncode="433101" if regioncode=="433101" & year==2007

replace regioncode="433122" if regioncode=="433122" & year==2007

replace regioncode="433123" if regioncode=="433123" & year==2007

replace regioncode="433124" if regioncode=="433124" & year==2007

replace regioncode="433125" if regioncode=="433125" & year==2007

replace regioncode="433126" if regioncode=="433126" & year==2007

replace regioncode="433127" if regioncode=="433127" & year==2007

replace regioncode="433130" if regioncode=="433130" & year==2007

replace regioncode="440102" if regioncode=="440115" & year==2007

replace regioncode="440103" if regioncode=="440103" & year==2007

replace regioncode="440104" if regioncode=="440104" & year==2007

replace regioncode="440105" if regioncode=="440105" & year==2007

replace regioncode="440106" if regioncode=="440106" & year==2007

replace regioncode="440107" if regioncode=="440116" & year==2007

replace regioncode="440111" if regioncode=="440111" & year==2007

replace regioncode="440112" if regioncode=="440112" & year==2007

replace regioncode="440113" if regioncode=="440113" & year==2007

replace regioncode="440114" if regioncode=="440114" & year==2007

replace regioncode="440183" if regioncode=="440183" & year==2007

replace regioncode="440184" if regioncode=="440184" & year==2007

replace regioncode="440203" if regioncode=="440203" & year==2007

replace regioncode="440204" if regioncode=="440204" & year==2007

replace regioncode="440221" if regioncode=="440205" & year==2007

replace regioncode="440222" if regioncode=="440222" & year==2007

replace regioncode="440224" if regioncode=="440224" & year==2007

replace regioncode="440229" if regioncode=="440229" & year==2007

replace regioncode="440232" if regioncode=="440232" & year==2007

replace regioncode="440233" if regioncode=="440233" & year==2007

replace regioncode="440281" if regioncode=="440281" & year==2007

replace regioncode="440282" if regioncode=="440282" & year==2007

replace regioncode="440303" if regioncode=="440303" & year==2007

replace regioncode="440304" if regioncode=="440304" & year==2007

replace regioncode="440305" if regioncode=="440305" & year==2007

replace regioncode="440306" if regioncode=="440306" & year==2007

replace regioncode="440307" if regioncode=="440307" & year==2007

replace regioncode="440308" if regioncode=="440308" & year==2007

replace regioncode="440402" if regioncode=="440402" & year==2007

replace regioncode="440403" if regioncode=="440403" & year==2007

replace regioncode="440404" if regioncode=="440404" & year==2007

replace regioncode="440507" if regioncode=="440507" & year==2007

replace regioncode="440511" if regioncode=="440511" & year==2007

replace regioncode="440512" if regioncode=="440512" & year==2007

replace regioncode="440514" if regioncode=="440514" & year==2007

replace regioncode="440515" if regioncode=="440515" & year==2007

replace regioncode="440523" if regioncode=="440523" & year==2007

replace regioncode="440604" if regioncode=="440604" & year==2007

replace regioncode="440605" if regioncode=="440605" & year==2007

replace regioncode="440606" if regioncode=="440606" & year==2007

replace regioncode="440607" if regioncode=="440607" & year==2007

replace regioncode="440608" if regioncode=="440608" & year==2007

replace regioncode="440703" if regioncode=="440703" & year==2007

replace regioncode="440704" if regioncode=="440704" & year==2007

replace regioncode="440781" if regioncode=="440781" & year==2007

replace regioncode="440705" if regioncode=="440705" & year==2007

replace regioncode="440783" if regioncode=="440783" & year==2007

replace regioncode="440784" if regioncode=="440784" & year==2007

replace regioncode="440785" if regioncode=="440785" & year==2007

replace regioncode="440802" if regioncode=="440802" & year==2007

replace regioncode="440803" if regioncode=="440803" & year==2007

replace regioncode="440804" if regioncode=="440804" & year==2007

replace regioncode="440811" if regioncode=="440811" & year==2007

replace regioncode="440823" if regioncode=="440823" & year==2007

replace regioncode="440825" if regioncode=="440825" & year==2007

replace regioncode="440881" if regioncode=="440881" & year==2007

replace regioncode="440882" if regioncode=="440882" & year==2007

replace regioncode="440883" if regioncode=="440883" & year==2007

replace regioncode="440902" if regioncode=="440902" & year==2007

replace regioncode="440903" if regioncode=="440903" & year==2007

replace regioncode="440923" if regioncode=="440923" & year==2007

replace regioncode="440981" if regioncode=="440981" & year==2007

replace regioncode="440982" if regioncode=="440982" & year==2007

replace regioncode="440983" if regioncode=="440983" & year==2007

replace regioncode="441202" if regioncode=="441202" & year==2007

replace regioncode="441203" if regioncode=="441203" & year==2007

replace regioncode="441223" if regioncode=="441223" & year==2007

replace regioncode="441224" if regioncode=="441224" & year==2007

replace regioncode="441225" if regioncode=="441225" & year==2007

replace regioncode="441226" if regioncode=="441226" & year==2007

replace regioncode="441283" if regioncode=="441283" & year==2007

replace regioncode="441284" if regioncode=="441284" & year==2007

replace regioncode="441302" if regioncode=="441302" & year==2007

replace regioncode="441303" if regioncode=="441303" & year==2007

replace regioncode="441322" if regioncode=="441322" & year==2007

replace regioncode="441323" if regioncode=="441323" & year==2007

replace regioncode="441324" if regioncode=="441324" & year==2007

replace regioncode="441402" if regioncode=="441402" & year==2007

replace regioncode="441421" if regioncode=="441421" & year==2007

replace regioncode="441422" if regioncode=="441422" & year==2007

replace regioncode="441423" if regioncode=="441423" & year==2007

replace regioncode="441424" if regioncode=="441424" & year==2007

replace regioncode="441426" if regioncode=="441426" & year==2007

replace regioncode="441427" if regioncode=="441427" & year==2007

replace regioncode="441481" if regioncode=="441481" & year==2007

replace regioncode="441502" if regioncode=="441502" & year==2007

replace regioncode="441521" if regioncode=="441521" & year==2007

replace regioncode="441523" if regioncode=="441523" & year==2007

replace regioncode="441581" if regioncode=="441581" & year==2007

replace regioncode="441602" if regioncode=="441602" & year==2007

replace regioncode="441621" if regioncode=="441621" & year==2007

replace regioncode="441622" if regioncode=="441622" & year==2007

replace regioncode="441623" if regioncode=="441623" & year==2007

replace regioncode="441624" if regioncode=="441624" & year==2007

replace regioncode="441625" if regioncode=="441625" & year==2007

replace regioncode="441702" if regioncode=="441702" & year==2007

replace regioncode="441721" if regioncode=="441721" & year==2007

replace regioncode="441723" if regioncode=="441723" & year==2007

replace regioncode="441781" if regioncode=="441781" & year==2007

replace regioncode="441802" if regioncode=="441802" & year==2007

replace regioncode="441821" if regioncode=="441821" & year==2007

replace regioncode="441823" if regioncode=="441823" & year==2007

replace regioncode="441825" if regioncode=="441825" & year==2007

replace regioncode="441826" if regioncode=="441826" & year==2007

replace regioncode="441827" if regioncode=="441827" & year==2007

replace regioncode="441881" if regioncode=="441881" & year==2007

replace regioncode="441882" if regioncode=="441882" & year==2007

replace regioncode="441900" if regioncode=="441900" & year==2007

replace regioncode="442000" if regioncode=="442000" & year==2007

replace regioncode="445102" if regioncode=="445102" & year==2007

replace regioncode="445121" if regioncode=="445121" & year==2007

replace regioncode="445122" if regioncode=="445122" & year==2007

replace regioncode="445202" if regioncode=="445202" & year==2007

replace regioncode="445221" if regioncode=="445221" & year==2007

replace regioncode="445222" if regioncode=="445222" & year==2007

replace regioncode="445224" if regioncode=="445224" & year==2007

replace regioncode="445281" if regioncode=="445281" & year==2007

replace regioncode="445302" if regioncode=="445302" & year==2007

replace regioncode="445321" if regioncode=="445321" & year==2007

replace regioncode="445322" if regioncode=="445322" & year==2007

replace regioncode="445323" if regioncode=="445323" & year==2007

replace regioncode="445381" if regioncode=="445381" & year==2007

replace regioncode="450102" if regioncode=="450102" & year==2007

replace regioncode="450103" if regioncode=="450103" & year==2007

replace regioncode="450104" if regioncode=="450107" & year==2007

replace regioncode="450105" if regioncode=="450105" & year==2007

replace regioncode="450106" if regioncode=="450108" & year==2007

replace regioncode="450121" if regioncode=="450109" & year==2007

replace regioncode="450122" if regioncode=="450122" & year==2007

replace regioncode="450123" if regioncode=="450123" & year==2007

replace regioncode="450124" if regioncode=="450124" & year==2007

replace regioncode="450125" if regioncode=="450125" & year==2007

replace regioncode="450126" if regioncode=="450126" & year==2007

replace regioncode="450127" if regioncode=="450127" & year==2007

replace regioncode="450202" if regioncode=="450202" & year==2007

replace regioncode="450203" if regioncode=="450203" & year==2007

replace regioncode="450204" if regioncode=="450204" & year==2007

replace regioncode="450205" if regioncode=="450205" & year==2007

replace regioncode="450221" if regioncode=="450221" & year==2007

replace regioncode="450222" if regioncode=="450222" & year==2007

replace regioncode="450223" if regioncode=="450223" & year==2007

replace regioncode="450224" if regioncode=="450224" & year==2007

replace regioncode="450225" if regioncode=="450225" & year==2007

replace regioncode="450226" if regioncode=="450226" & year==2007

replace regioncode="450302" if regioncode=="450302" & year==2007

replace regioncode="450303" if regioncode=="450303" & year==2007

replace regioncode="450304" if regioncode=="450304" & year==2007

replace regioncode="450305" if regioncode=="450305" & year==2007

replace regioncode="450311" if regioncode=="450311" & year==2007

replace regioncode="450321" if regioncode=="450321" & year==2007

replace regioncode="450322" if regioncode=="450322" & year==2007

replace regioncode="450323" if regioncode=="450323" & year==2007

replace regioncode="450324" if regioncode=="450324" & year==2007

replace regioncode="450325" if regioncode=="450325" & year==2007

replace regioncode="450326" if regioncode=="450326" & year==2007

replace regioncode="450327" if regioncode=="450327" & year==2007

replace regioncode="450328" if regioncode=="450328" & year==2007

replace regioncode="450329" if regioncode=="450329" & year==2007

replace regioncode="450330" if regioncode=="450330" & year==2007

replace regioncode="450331" if regioncode=="450331" & year==2007

replace regioncode="450332" if regioncode=="450332" & year==2007

replace regioncode="450403" if regioncode=="450403" & year==2007

replace regioncode="450404" if regioncode=="450404" & year==2007

replace regioncode="450421" if regioncode=="450421" & year==2007

replace regioncode="450422" if regioncode=="450422" & year==2007

replace regioncode="450423" if regioncode=="450423" & year==2007

replace regioncode="450481" if regioncode=="450481" & year==2007

replace regioncode="450502" if regioncode=="450502" & year==2007

replace regioncode="450503" if regioncode=="450503" & year==2007

replace regioncode="450512" if regioncode=="450512" & year==2007

replace regioncode="450521" if regioncode=="450521" & year==2007

replace regioncode="450602" if regioncode=="450602" & year==2007

replace regioncode="450603" if regioncode=="450603" & year==2007

replace regioncode="450621" if regioncode=="450621" & year==2007

replace regioncode="450681" if regioncode=="450681" & year==2007

replace regioncode="450702" if regioncode=="450702" & year==2007

replace regioncode="450703" if regioncode=="450703" & year==2007

replace regioncode="450721" if regioncode=="450721" & year==2007

replace regioncode="450722" if regioncode=="450722" & year==2007

replace regioncode="450802" if regioncode=="450802" & year==2007

replace regioncode="450803" if regioncode=="450803" & year==2007

replace regioncode="450804" if regioncode=="450804" & year==2007

replace regioncode="450821" if regioncode=="450821" & year==2007

replace regioncode="450881" if regioncode=="450881" & year==2007

replace regioncode="450902" if regioncode=="450902" & year==2007

replace regioncode="450921" if regioncode=="450921" & year==2007

replace regioncode="450922" if regioncode=="450922" & year==2007

replace regioncode="450923" if regioncode=="450923" & year==2007

replace regioncode="450924" if regioncode=="450924" & year==2007

replace regioncode="450981" if regioncode=="450981" & year==2007

replace regioncode="451002" if regioncode=="451002" & year==2007

replace regioncode="451021" if regioncode=="451021" & year==2007

replace regioncode="451022" if regioncode=="451022" & year==2007

replace regioncode="451023" if regioncode=="451023" & year==2007

replace regioncode="451024" if regioncode=="451024" & year==2007

replace regioncode="451025" if regioncode=="451025" & year==2007

replace regioncode="451026" if regioncode=="451026" & year==2007

replace regioncode="451027" if regioncode=="451027" & year==2007

replace regioncode="451028" if regioncode=="451028" & year==2007

replace regioncode="451029" if regioncode=="451029" & year==2007

replace regioncode="451030" if regioncode=="451030" & year==2007

replace regioncode="451031" if regioncode=="451031" & year==2007

replace regioncode="451102" if regioncode=="451102" & year==2007

replace regioncode="451121" if regioncode=="451121" & year==2007

replace regioncode="451122" if regioncode=="451122" & year==2007

replace regioncode="451123" if regioncode=="451123" & year==2007

replace regioncode="451202" if regioncode=="451202" & year==2007

replace regioncode="451221" if regioncode=="451221" & year==2007

replace regioncode="451222" if regioncode=="451222" & year==2007

replace regioncode="451223" if regioncode=="451223" & year==2007

replace regioncode="451224" if regioncode=="451224" & year==2007

replace regioncode="451225" if regioncode=="451225" & year==2007

replace regioncode="451226" if regioncode=="451226" & year==2007

replace regioncode="451227" if regioncode=="451227" & year==2007

replace regioncode="451228" if regioncode=="451228" & year==2007

replace regioncode="451229" if regioncode=="451229" & year==2007

replace regioncode="451281" if regioncode=="451281" & year==2007

replace regioncode="451302" if regioncode=="451302" & year==2007

replace regioncode="451321" if regioncode=="451321" & year==2007

replace regioncode="451322" if regioncode=="451322" & year==2007

replace regioncode="451323" if regioncode=="451323" & year==2007

replace regioncode="451324" if regioncode=="451324" & year==2007

replace regioncode="451381" if regioncode=="451381" & year==2007

replace regioncode="451402" if regioncode=="451402" & year==2007

replace regioncode="451421" if regioncode=="451421" & year==2007

replace regioncode="451422" if regioncode=="451422" & year==2007

replace regioncode="451423" if regioncode=="451423" & year==2007

replace regioncode="451424" if regioncode=="451424" & year==2007

replace regioncode="451425" if regioncode=="451425" & year==2007

replace regioncode="451481" if regioncode=="451481" & year==2007

replace regioncode="460105" if regioncode=="460105" & year==2007

replace regioncode="460106" if regioncode=="460106" & year==2007

replace regioncode="460107" if regioncode=="460107" & year==2007

replace regioncode="460108" if regioncode=="460108" & year==2007

replace regioncode="460001" if regioncode=="469001" & year==2007

replace regioncode="460002" if regioncode=="469002" & year==2007

replace regioncode="460003" if regioncode=="469003" & year==2007

replace regioncode="460005" if regioncode=="469005" & year==2007

replace regioncode="460006" if regioncode=="469006" & year==2007

replace regioncode="460007" if regioncode=="469007" & year==2007

replace regioncode="460030" if regioncode=="469025" & year==2007

replace regioncode="460031" if regioncode=="469026" & year==2007

replace regioncode="460033" if regioncode=="469027" & year==2007

replace regioncode="460034" if regioncode=="469028" & year==2007

replace regioncode="460036" if regioncode=="469030" & year==2007

replace regioncode="500101" if regioncode=="500101" & year==2007

replace regioncode="500102" if regioncode=="500102" & year==2007

replace regioncode="500103" if regioncode=="500103" & year==2007

replace regioncode="500104" if regioncode=="500104" & year==2007

replace regioncode="500105" if regioncode=="500105" & year==2007

replace regioncode="500106" if regioncode=="500106" & year==2007

replace regioncode="500107" if regioncode=="500107" & year==2007

replace regioncode="500108" if regioncode=="500108" & year==2007

replace regioncode="500109" if regioncode=="500109" & year==2007

replace regioncode="500110" if regioncode=="500110" & year==2007

replace regioncode="500111" if regioncode=="500111" & year==2007

replace regioncode="500112" if regioncode=="500112" & year==2007

replace regioncode="500113" if regioncode=="500113" & year==2007

replace regioncode="500114" if regioncode=="500114" & year==2007

replace regioncode="500115" if regioncode=="500115" & year==2007

replace regioncode="500381" if regioncode=="500116" & year==2007

replace regioncode="500382" if regioncode=="500117" & year==2007

replace regioncode="500383" if regioncode=="500118" & year==2007

replace regioncode="500384" if regioncode=="500119" & year==2007

replace regioncode="500222" if regioncode=="500222" & year==2007

replace regioncode="500223" if regioncode=="500223" & year==2007

replace regioncode="500224" if regioncode=="500224" & year==2007

replace regioncode="500225" if regioncode=="500225" & year==2007

replace regioncode="500226" if regioncode=="500226" & year==2007

replace regioncode="500227" if regioncode=="500227" & year==2007

replace regioncode="500228" if regioncode=="500228" & year==2007

replace regioncode="500229" if regioncode=="500229" & year==2007

replace regioncode="500230" if regioncode=="500230" & year==2007

replace regioncode="500231" if regioncode=="500231" & year==2007

replace regioncode="500232" if regioncode=="500232" & year==2007

replace regioncode="500233" if regioncode=="500233" & year==2007

replace regioncode="500234" if regioncode=="500234" & year==2007

replace regioncode="500235" if regioncode=="500235" & year==2007

replace regioncode="500236" if regioncode=="500236" & year==2007

replace regioncode="500237" if regioncode=="500237" & year==2007

replace regioncode="500238" if regioncode=="500238" & year==2007

replace regioncode="500240" if regioncode=="500240" & year==2007

replace regioncode="500241" if regioncode=="500241" & year==2007

replace regioncode="500242" if regioncode=="500242" & year==2007

replace regioncode="500243" if regioncode=="500243" & year==2007

replace regioncode="510104" if regioncode=="510104" & year==2007

replace regioncode="510105" if regioncode=="510105" & year==2007

replace regioncode="510106" if regioncode=="510106" & year==2007

replace regioncode="510107" if regioncode=="510107" & year==2007

replace regioncode="510108" if regioncode=="510108" & year==2007

replace regioncode="510112" if regioncode=="510112" & year==2007

replace regioncode="510113" if regioncode=="510113" & year==2007

replace regioncode="510114" if regioncode=="510114" & year==2007

replace regioncode="510115" if regioncode=="510115" & year==2007

replace regioncode="510121" if regioncode=="510121" & year==2007

replace regioncode="510122" if regioncode=="510122" & year==2007

replace regioncode="510124" if regioncode=="510124" & year==2007

replace regioncode="510129" if regioncode=="510129" & year==2007

replace regioncode="510131" if regioncode=="510131" & year==2007

replace regioncode="510132" if regioncode=="510132" & year==2007

replace regioncode="510181" if regioncode=="510181" & year==2007

replace regioncode="510182" if regioncode=="510182" & year==2007

replace regioncode="510183" if regioncode=="510183" & year==2007

replace regioncode="510184" if regioncode=="510184" & year==2007

replace regioncode="510302" if regioncode=="510302" & year==2007

replace regioncode="510303" if regioncode=="510303" & year==2007

replace regioncode="510304" if regioncode=="510304" & year==2007

replace regioncode="510311" if regioncode=="510311" & year==2007

replace regioncode="510321" if regioncode=="510321" & year==2007

replace regioncode="510322" if regioncode=="510322" & year==2007

replace regioncode="510402" if regioncode=="510402" & year==2007

replace regioncode="510403" if regioncode=="510403" & year==2007

replace regioncode="510411" if regioncode=="510411" & year==2007

replace regioncode="510421" if regioncode=="510421" & year==2007

replace regioncode="510422" if regioncode=="510422" & year==2007

replace regioncode="510502" if regioncode=="510502" & year==2007

replace regioncode="510503" if regioncode=="510503" & year==2007

replace regioncode="510504" if regioncode=="510504" & year==2007

replace regioncode="510521" if regioncode=="510521" & year==2007

replace regioncode="510522" if regioncode=="510522" & year==2007

replace regioncode="510524" if regioncode=="510524" & year==2007

replace regioncode="510525" if regioncode=="510525" & year==2007

replace regioncode="510603" if regioncode=="510603" & year==2007

replace regioncode="510623" if regioncode=="510623" & year==2007

replace regioncode="510626" if regioncode=="510626" & year==2007

replace regioncode="510681" if regioncode=="510681" & year==2007

replace regioncode="510682" if regioncode=="510682" & year==2007

replace regioncode="510683" if regioncode=="510683" & year==2007

replace regioncode="510703" if regioncode=="510703" & year==2007

replace regioncode="510704" if regioncode=="510704" & year==2007

replace regioncode="510722" if regioncode=="510722" & year==2007

replace regioncode="510723" if regioncode=="510723" & year==2007

replace regioncode="510724" if regioncode=="510724" & year==2007

replace regioncode="510725" if regioncode=="510725" & year==2007

replace regioncode="510726" if regioncode=="510726" & year==2007

replace regioncode="510727" if regioncode=="510727" & year==2007

replace regioncode="510781" if regioncode=="510781" & year==2007

replace regioncode="510802" if regioncode=="510802" & year==2007

replace regioncode="510811" if regioncode=="510811" & year==2007

replace regioncode="510812" if regioncode=="510812" & year==2007

replace regioncode="510821" if regioncode=="510821" & year==2007

replace regioncode="510822" if regioncode=="510822" & year==2007

replace regioncode="510823" if regioncode=="510823" & year==2007

replace regioncode="510824" if regioncode=="510824" & year==2007

replace regioncode="510902" if regioncode=="510903" & year==2007

replace regioncode="510921" if regioncode=="510921" & year==2007

replace regioncode="510922" if regioncode=="510922" & year==2007

replace regioncode="510923" if regioncode=="510923" & year==2007

replace regioncode="511002" if regioncode=="511002" & year==2007

replace regioncode="511011" if regioncode=="511011" & year==2007

replace regioncode="511024" if regioncode=="511024" & year==2007

replace regioncode="511025" if regioncode=="511025" & year==2007

replace regioncode="511028" if regioncode=="511028" & year==2007

replace regioncode="511102" if regioncode=="511102" & year==2007

replace regioncode="511111" if regioncode=="511111" & year==2007

replace regioncode="511112" if regioncode=="511112" & year==2007

replace regioncode="511113" if regioncode=="511113" & year==2007

replace regioncode="511123" if regioncode=="511123" & year==2007

replace regioncode="511124" if regioncode=="511124" & year==2007

replace regioncode="511126" if regioncode=="511126" & year==2007

replace regioncode="511129" if regioncode=="511129" & year==2007

replace regioncode="511132" if regioncode=="511132" & year==2007

replace regioncode="511133" if regioncode=="511133" & year==2007

replace regioncode="511181" if regioncode=="511181" & year==2007

replace regioncode="511302" if regioncode=="511302" & year==2007

replace regioncode="511303" if regioncode=="511303" & year==2007

replace regioncode="511304" if regioncode=="511304" & year==2007

replace regioncode="511321" if regioncode=="511321" & year==2007

replace regioncode="511322" if regioncode=="511322" & year==2007

replace regioncode="511323" if regioncode=="511323" & year==2007

replace regioncode="511324" if regioncode=="511324" & year==2007

replace regioncode="511325" if regioncode=="511325" & year==2007

replace regioncode="511381" if regioncode=="511381" & year==2007

replace regioncode="511402" if regioncode=="511402" & year==2007

replace regioncode="511421" if regioncode=="511421" & year==2007

replace regioncode="511422" if regioncode=="511422" & year==2007

replace regioncode="511423" if regioncode=="511423" & year==2007

replace regioncode="511424" if regioncode=="511424" & year==2007

replace regioncode="511425" if regioncode=="511425" & year==2007

replace regioncode="511502" if regioncode=="511502" & year==2007

replace regioncode="511521" if regioncode=="511521" & year==2007

replace regioncode="511522" if regioncode=="511522" & year==2007

replace regioncode="511523" if regioncode=="511523" & year==2007

replace regioncode="511524" if regioncode=="511524" & year==2007

replace regioncode="511525" if regioncode=="511525" & year==2007

replace regioncode="511526" if regioncode=="511526" & year==2007

replace regioncode="511527" if regioncode=="511527" & year==2007

replace regioncode="511528" if regioncode=="511528" & year==2007

replace regioncode="511529" if regioncode=="511529" & year==2007

replace regioncode="511602" if regioncode=="511602" & year==2007

replace regioncode="511621" if regioncode=="511621" & year==2007

replace regioncode="511622" if regioncode=="511622" & year==2007

replace regioncode="511623" if regioncode=="511623" & year==2007

replace regioncode="511681" if regioncode=="511681" & year==2007

replace regioncode="511702" if regioncode=="511702" & year==2007

replace regioncode="511721" if regioncode=="511721" & year==2007

replace regioncode="511722" if regioncode=="511722" & year==2007

replace regioncode="511723" if regioncode=="511723" & year==2007

replace regioncode="511724" if regioncode=="511724" & year==2007

replace regioncode="511725" if regioncode=="511725" & year==2007

replace regioncode="511781" if regioncode=="511781" & year==2007

replace regioncode="511802" if regioncode=="511802" & year==2007

replace regioncode="511821" if regioncode=="511821" & year==2007

replace regioncode="511822" if regioncode=="511822" & year==2007

replace regioncode="511823" if regioncode=="511823" & year==2007

replace regioncode="511824" if regioncode=="511824" & year==2007

replace regioncode="511825" if regioncode=="511825" & year==2007

replace regioncode="511826" if regioncode=="511826" & year==2007

replace regioncode="511827" if regioncode=="511827" & year==2007

replace regioncode="511902" if regioncode=="511902" & year==2007

replace regioncode="511921" if regioncode=="511921" & year==2007

replace regioncode="511922" if regioncode=="511922" & year==2007

replace regioncode="511923" if regioncode=="511923" & year==2007

replace regioncode="512002" if regioncode=="512002" & year==2007

replace regioncode="512021" if regioncode=="512021" & year==2007

replace regioncode="512022" if regioncode=="512022" & year==2007

replace regioncode="512081" if regioncode=="512081" & year==2007

replace regioncode="513221" if regioncode=="513221" & year==2007

replace regioncode="513222" if regioncode=="513222" & year==2007

replace regioncode="513223" if regioncode=="513223" & year==2007

replace regioncode="513224" if regioncode=="513224" & year==2007

replace regioncode="513225" if regioncode=="513225" & year==2007

replace regioncode="513226" if regioncode=="513226" & year==2007

replace regioncode="513227" if regioncode=="513227" & year==2007

replace regioncode="513228" if regioncode=="513228" & year==2007

replace regioncode="513229" if regioncode=="513229" & year==2007

replace regioncode="513230" if regioncode=="513230" & year==2007

replace regioncode="513231" if regioncode=="513231" & year==2007

replace regioncode="513232" if regioncode=="513232" & year==2007

replace regioncode="513233" if regioncode=="513233" & year==2007

replace regioncode="513321" if regioncode=="513321" & year==2007

replace regioncode="513322" if regioncode=="513322" & year==2007

replace regioncode="513323" if regioncode=="513323" & year==2007

replace regioncode="513324" if regioncode=="513324" & year==2007

replace regioncode="513325" if regioncode=="513325" & year==2007

replace regioncode="513326" if regioncode=="513326" & year==2007

replace regioncode="513327" if regioncode=="513327" & year==2007

replace regioncode="513328" if regioncode=="513328" & year==2007

replace regioncode="513329" if regioncode=="513329" & year==2007

replace regioncode="513330" if regioncode=="513330" & year==2007

replace regioncode="513331" if regioncode=="513331" & year==2007

replace regioncode="513332" if regioncode=="513332" & year==2007

replace regioncode="513333" if regioncode=="513333" & year==2007

replace regioncode="513334" if regioncode=="513334" & year==2007

replace regioncode="513335" if regioncode=="513335" & year==2007

replace regioncode="513336" if regioncode=="513336" & year==2007

replace regioncode="513337" if regioncode=="513337" & year==2007

replace regioncode="513338" if regioncode=="513338" & year==2007

replace regioncode="513401" if regioncode=="513401" & year==2007

replace regioncode="513422" if regioncode=="513422" & year==2007

replace regioncode="513423" if regioncode=="513423" & year==2007

replace regioncode="513424" if regioncode=="513424" & year==2007

replace regioncode="513425" if regioncode=="513425" & year==2007

replace regioncode="513426" if regioncode=="513426" & year==2007

replace regioncode="513427" if regioncode=="513427" & year==2007

replace regioncode="513428" if regioncode=="513428" & year==2007

replace regioncode="513429" if regioncode=="513429" & year==2007

replace regioncode="513430" if regioncode=="513430" & year==2007

replace regioncode="513431" if regioncode=="513431" & year==2007

replace regioncode="513432" if regioncode=="513432" & year==2007

replace regioncode="513433" if regioncode=="513433" & year==2007

replace regioncode="513434" if regioncode=="513434" & year==2007

replace regioncode="513435" if regioncode=="513435" & year==2007

replace regioncode="513436" if regioncode=="513436" & year==2007

replace regioncode="513437" if regioncode=="513437" & year==2007

replace regioncode="520102" if regioncode=="520102" & year==2007

replace regioncode="520103" if regioncode=="520103" & year==2007

replace regioncode="520111" if regioncode=="520111" & year==2007

replace regioncode="520112" if regioncode=="520112" & year==2007

replace regioncode="520113" if regioncode=="520113" & year==2007

replace regioncode="520114" if regioncode=="520114" & year==2007

replace regioncode="520121" if regioncode=="520121" & year==2007

replace regioncode="520122" if regioncode=="520122" & year==2007

replace regioncode="520123" if regioncode=="520123" & year==2007

replace regioncode="520181" if regioncode=="520181" & year==2007

replace regioncode="520201" if regioncode=="520201" & year==2007

replace regioncode="520222" if regioncode=="520222" & year==2007

replace regioncode="520203" if regioncode=="520203" & year==2007

replace regioncode="520221" if regioncode=="520221" & year==2007

replace regioncode="520302" if regioncode=="520302" & year==2007

replace regioncode="520321" if regioncode=="520321" & year==2007

replace regioncode="520322" if regioncode=="520322" & year==2007

replace regioncode="520323" if regioncode=="520323" & year==2007

replace regioncode="520324" if regioncode=="520324" & year==2007

replace regioncode="520325" if regioncode=="520325" & year==2007

replace regioncode="520326" if regioncode=="520326" & year==2007

replace regioncode="520327" if regioncode=="520327" & year==2007

replace regioncode="520328" if regioncode=="520328" & year==2007

replace regioncode="520329" if regioncode=="520329" & year==2007

replace regioncode="520330" if regioncode=="520330" & year==2007

replace regioncode="520381" if regioncode=="520381" & year==2007

replace regioncode="520382" if regioncode=="520382" & year==2007

replace regioncode="520402" if regioncode=="520402" & year==2007

replace regioncode="520421" if regioncode=="520421" & year==2007

replace regioncode="520422" if regioncode=="520422" & year==2007

replace regioncode="520423" if regioncode=="520423" & year==2007

replace regioncode="520424" if regioncode=="520424" & year==2007

replace regioncode="520425" if regioncode=="520425" & year==2007

replace regioncode="522201" if regioncode=="522201" & year==2007

replace regioncode="522222" if regioncode=="522222" & year==2007

replace regioncode="522223" if regioncode=="522223" & year==2007

replace regioncode="522224" if regioncode=="522224" & year==2007

replace regioncode="522225" if regioncode=="522225" & year==2007

replace regioncode="522226" if regioncode=="522226" & year==2007

replace regioncode="522227" if regioncode=="522227" & year==2007

replace regioncode="522228" if regioncode=="522228" & year==2007

replace regioncode="522229" if regioncode=="522229" & year==2007

replace regioncode="522230" if regioncode=="522230" & year==2007

replace regioncode="522301" if regioncode=="522301" & year==2007

replace regioncode="522322" if regioncode=="522322" & year==2007

replace regioncode="522323" if regioncode=="522323" & year==2007

replace regioncode="522324" if regioncode=="522324" & year==2007

replace regioncode="522325" if regioncode=="522325" & year==2007

replace regioncode="522326" if regioncode=="522326" & year==2007

replace regioncode="522327" if regioncode=="522327" & year==2007

replace regioncode="522328" if regioncode=="522328" & year==2007

replace regioncode="522401" if regioncode=="522401" & year==2007

replace regioncode="522422" if regioncode=="522422" & year==2007

replace regioncode="522423" if regioncode=="522423" & year==2007

replace regioncode="522424" if regioncode=="522424" & year==2007

replace regioncode="522425" if regioncode=="522425" & year==2007

replace regioncode="522426" if regioncode=="522426" & year==2007

replace regioncode="522427" if regioncode=="522427" & year==2007

replace regioncode="522428" if regioncode=="522428" & year==2007

replace regioncode="522601" if regioncode=="522601" & year==2007

replace regioncode="522622" if regioncode=="522622" & year==2007

replace regioncode="522623" if regioncode=="522623" & year==2007

replace regioncode="522624" if regioncode=="522624" & year==2007

replace regioncode="522625" if regioncode=="522625" & year==2007

replace regioncode="522626" if regioncode=="522626" & year==2007

replace regioncode="522627" if regioncode=="522627" & year==2007

replace regioncode="522628" if regioncode=="522628" & year==2007

replace regioncode="522629" if regioncode=="522629" & year==2007

replace regioncode="522630" if regioncode=="522630" & year==2007

replace regioncode="522631" if regioncode=="522631" & year==2007

replace regioncode="522632" if regioncode=="522632" & year==2007

replace regioncode="522633" if regioncode=="522633" & year==2007

replace regioncode="522634" if regioncode=="522634" & year==2007

replace regioncode="522635" if regioncode=="522635" & year==2007

replace regioncode="522636" if regioncode=="522636" & year==2007

replace regioncode="522701" if regioncode=="522701" & year==2007

replace regioncode="522702" if regioncode=="522702" & year==2007

replace regioncode="522722" if regioncode=="522722" & year==2007

replace regioncode="522723" if regioncode=="522723" & year==2007

replace regioncode="522725" if regioncode=="522725" & year==2007

replace regioncode="522726" if regioncode=="522726" & year==2007

replace regioncode="522727" if regioncode=="522727" & year==2007

replace regioncode="522728" if regioncode=="522728" & year==2007

replace regioncode="522729" if regioncode=="522729" & year==2007

replace regioncode="522730" if regioncode=="522730" & year==2007

replace regioncode="522731" if regioncode=="522731" & year==2007

replace regioncode="522732" if regioncode=="522732" & year==2007

replace regioncode="530102" if regioncode=="530102" & year==2007

replace regioncode="530103" if regioncode=="530103" & year==2007

replace regioncode="530111" if regioncode=="530111" & year==2007

replace regioncode="530112" if regioncode=="530112" & year==2007

replace regioncode="530113" if regioncode=="530113" & year==2007

replace regioncode="530121" if regioncode=="530121" & year==2007

replace regioncode="530122" if regioncode=="530122" & year==2007

replace regioncode="530124" if regioncode=="530124" & year==2007

replace regioncode="530125" if regioncode=="530125" & year==2007

replace regioncode="530126" if regioncode=="530126" & year==2007

replace regioncode="530127" if regioncode=="530127" & year==2007

replace regioncode="530128" if regioncode=="530128" & year==2007

replace regioncode="530129" if regioncode=="530129" & year==2007

replace regioncode="530181" if regioncode=="530181" & year==2007

replace regioncode="530302" if regioncode=="530302" & year==2007

replace regioncode="530321" if regioncode=="530321" & year==2007

replace regioncode="530322" if regioncode=="530322" & year==2007

replace regioncode="530323" if regioncode=="530323" & year==2007

replace regioncode="530324" if regioncode=="530324" & year==2007

replace regioncode="530325" if regioncode=="530325" & year==2007

replace regioncode="530326" if regioncode=="530326" & year==2007

replace regioncode="530328" if regioncode=="530328" & year==2007

replace regioncode="530381" if regioncode=="530381" & year==2007

replace regioncode="530402" if regioncode=="530402" & year==2007

replace regioncode="530421" if regioncode=="530421" & year==2007

replace regioncode="530422" if regioncode=="530422" & year==2007

replace regioncode="530423" if regioncode=="530423" & year==2007

replace regioncode="530424" if regioncode=="530424" & year==2007

replace regioncode="530425" if regioncode=="530425" & year==2007

replace regioncode="530426" if regioncode=="530426" & year==2007

replace regioncode="530427" if regioncode=="530427" & year==2007

replace regioncode="530428" if regioncode=="530428" & year==2007

replace regioncode="530502" if regioncode=="530502" & year==2007

replace regioncode="530521" if regioncode=="530521" & year==2007

replace regioncode="530522" if regioncode=="530522" & year==2007

replace regioncode="530523" if regioncode=="530523" & year==2007

replace regioncode="530524" if regioncode=="530524" & year==2007

replace regioncode="530602" if regioncode=="530602" & year==2007

replace regioncode="530621" if regioncode=="530621" & year==2007

replace regioncode="530622" if regioncode=="530622" & year==2007

replace regioncode="530623" if regioncode=="530623" & year==2007

replace regioncode="530624" if regioncode=="530624" & year==2007

replace regioncode="530625" if regioncode=="530625" & year==2007

replace regioncode="530626" if regioncode=="530626" & year==2007

replace regioncode="530627" if regioncode=="530627" & year==2007

replace regioncode="530628" if regioncode=="530628" & year==2007

replace regioncode="530629" if regioncode=="530629" & year==2007

replace regioncode="530630" if regioncode=="530630" & year==2007

replace regioncode="530721" if regioncode=="530721" & year==2007

replace regioncode="530722" if regioncode=="530722" & year==2007

replace regioncode="530723" if regioncode=="530723" & year==2007

replace regioncode="530724" if regioncode=="530724" & year==2007

replace regioncode="532701" if regioncode=="530802" & year==2007

replace regioncode="532722" if regioncode=="530821" & year==2007

replace regioncode="532723" if regioncode=="530822" & year==2007

replace regioncode="532724" if regioncode=="530823" & year==2007

replace regioncode="532725" if regioncode=="530824" & year==2007

replace regioncode="532726" if regioncode=="530825" & year==2007

replace regioncode="532727" if regioncode=="530826" & year==2007

replace regioncode="532728" if regioncode=="530827" & year==2007

replace regioncode="532729" if regioncode=="530828" & year==2007

replace regioncode="532730" if regioncode=="530829" & year==2007

replace regioncode="533521" if regioncode=="530902" & year==2007

replace regioncode="533522" if regioncode=="530921" & year==2007

replace regioncode="533523" if regioncode=="530922" & year==2007

replace regioncode="533524" if regioncode=="530923" & year==2007

replace regioncode="533525" if regioncode=="530924" & year==2007

replace regioncode="533526" if regioncode=="530925" & year==2007

replace regioncode="533527" if regioncode=="530926" & year==2007

replace regioncode="533528" if regioncode=="530927" & year==2007

replace regioncode="532301" if regioncode=="532301" & year==2007

replace regioncode="532322" if regioncode=="532322" & year==2007

replace regioncode="532323" if regioncode=="532323" & year==2007

replace regioncode="532324" if regioncode=="532324" & year==2007

replace regioncode="532325" if regioncode=="532325" & year==2007

replace regioncode="532326" if regioncode=="532326" & year==2007

replace regioncode="532327" if regioncode=="532327" & year==2007

replace regioncode="532328" if regioncode=="532328" & year==2007

replace regioncode="532329" if regioncode=="532329" & year==2007

replace regioncode="532331" if regioncode=="532331" & year==2007

replace regioncode="532501" if regioncode=="532501" & year==2007

replace regioncode="532502" if regioncode=="532502" & year==2007

replace regioncode="532522" if regioncode=="532522" & year==2007

replace regioncode="532523" if regioncode=="532523" & year==2007

replace regioncode="532524" if regioncode=="532524" & year==2007

replace regioncode="532525" if regioncode=="532525" & year==2007

replace regioncode="532526" if regioncode=="532526" & year==2007

replace regioncode="532527" if regioncode=="532527" & year==2007

replace regioncode="532528" if regioncode=="532528" & year==2007

replace regioncode="532529" if regioncode=="532529" & year==2007

replace regioncode="532530" if regioncode=="532530" & year==2007

replace regioncode="532531" if regioncode=="532531" & year==2007

replace regioncode="532532" if regioncode=="532532" & year==2007

replace regioncode="532621" if regioncode=="532621" & year==2007

replace regioncode="532622" if regioncode=="532622" & year==2007

replace regioncode="532623" if regioncode=="532623" & year==2007

replace regioncode="532624" if regioncode=="532624" & year==2007

replace regioncode="532625" if regioncode=="532625" & year==2007

replace regioncode="532626" if regioncode=="532626" & year==2007

replace regioncode="532627" if regioncode=="532627" & year==2007

replace regioncode="532628" if regioncode=="532628" & year==2007

replace regioncode="532801" if regioncode=="532801" & year==2007

replace regioncode="532822" if regioncode=="532822" & year==2007

replace regioncode="532823" if regioncode=="532823" & year==2007

replace regioncode="532901" if regioncode=="532901" & year==2007

replace regioncode="532922" if regioncode=="532922" & year==2007

replace regioncode="532923" if regioncode=="532923" & year==2007

replace regioncode="532924" if regioncode=="532924" & year==2007

replace regioncode="532925" if regioncode=="532925" & year==2007

replace regioncode="532926" if regioncode=="532926" & year==2007

replace regioncode="532927" if regioncode=="532927" & year==2007

replace regioncode="532928" if regioncode=="532928" & year==2007

replace regioncode="532929" if regioncode=="532929" & year==2007

replace regioncode="532930" if regioncode=="532930" & year==2007

replace regioncode="532931" if regioncode=="532931" & year==2007

replace regioncode="532932" if regioncode=="532932" & year==2007

replace regioncode="533102" if regioncode=="533102" & year==2007

replace regioncode="533103" if regioncode=="533103" & year==2007

replace regioncode="533122" if regioncode=="533122" & year==2007

replace regioncode="533123" if regioncode=="533123" & year==2007

replace regioncode="533124" if regioncode=="533124" & year==2007

replace regioncode="533321" if regioncode=="533321" & year==2007

replace regioncode="533323" if regioncode=="533323" & year==2007

replace regioncode="533324" if regioncode=="533324" & year==2007

replace regioncode="533325" if regioncode=="533325" & year==2007

replace regioncode="533421" if regioncode=="533421" & year==2007

replace regioncode="533422" if regioncode=="533422" & year==2007

replace regioncode="533423" if regioncode=="533423" & year==2007

replace regioncode="540102" if regioncode=="540102" & year==2007

replace regioncode="540121" if regioncode=="540121" & year==2007

replace regioncode="540122" if regioncode=="540122" & year==2007

replace regioncode="540123" if regioncode=="540123" & year==2007

replace regioncode="540124" if regioncode=="540124" & year==2007

replace regioncode="540125" if regioncode=="540125" & year==2007

replace regioncode="540126" if regioncode=="540126" & year==2007

replace regioncode="540127" if regioncode=="540127" & year==2007

replace regioncode="542121" if regioncode=="542121" & year==2007

replace regioncode="542122" if regioncode=="542122" & year==2007

replace regioncode="542123" if regioncode=="542123" & year==2007

replace regioncode="542124" if regioncode=="542124" & year==2007

replace regioncode="542125" if regioncode=="542125" & year==2007

replace regioncode="542126" if regioncode=="542126" & year==2007

replace regioncode="542127" if regioncode=="542127" & year==2007

replace regioncode="542128" if regioncode=="542128" & year==2007

replace regioncode="542129" if regioncode=="542129" & year==2007

replace regioncode="542132" if regioncode=="542132" & year==2007

replace regioncode="542133" if regioncode=="542133" & year==2007

replace regioncode="542221" if regioncode=="542221" & year==2007

replace regioncode="542223" if regioncode=="542223" & year==2007

replace regioncode="542224" if regioncode=="542224" & year==2007

replace regioncode="542225" if regioncode=="542225" & year==2007

replace regioncode="542227" if regioncode=="542227" & year==2007

replace regioncode="542228" if regioncode=="542228" & year==2007

replace regioncode="542229" if regioncode=="542229" & year==2007

replace regioncode="542231" if regioncode=="542231" & year==2007

replace regioncode="542232" if regioncode=="542232" & year==2007

replace regioncode="542233" if regioncode=="542233" & year==2007

replace regioncode="542301" if regioncode=="542301" & year==2007

replace regioncode="542322" if regioncode=="542322" & year==2007

replace regioncode="542323" if regioncode=="542323" & year==2007

replace regioncode="542324" if regioncode=="542324" & year==2007

replace regioncode="542325" if regioncode=="542325" & year==2007

replace regioncode="542326" if regioncode=="542326" & year==2007

replace regioncode="542327" if regioncode=="542327" & year==2007

replace regioncode="542328" if regioncode=="542328" & year==2007

replace regioncode="542329" if regioncode=="542329" & year==2007

replace regioncode="542330" if regioncode=="542330" & year==2007

replace regioncode="542331" if regioncode=="542331" & year==2007

replace regioncode="542332" if regioncode=="542332" & year==2007

replace regioncode="542334" if regioncode=="542334" & year==2007

replace regioncode="542336" if regioncode=="542336" & year==2007

replace regioncode="542338" if regioncode=="542338" & year==2007

replace regioncode="542421" if regioncode=="542421" & year==2007

replace regioncode="542422" if regioncode=="542422" & year==2007

replace regioncode="542423" if regioncode=="542423" & year==2007

replace regioncode="542424" if regioncode=="542424" & year==2007

replace regioncode="542425" if regioncode=="542425" & year==2007

replace regioncode="542426" if regioncode=="542426" & year==2007

replace regioncode="542427" if regioncode=="542427" & year==2007

replace regioncode="542429" if regioncode=="542429" & year==2007

replace regioncode="542430" if regioncode=="542430" & year==2007

replace regioncode="542523" if regioncode=="542523" & year==2007

replace regioncode="542621" if regioncode=="542621" & year==2007

replace regioncode="542622" if regioncode=="542622" & year==2007

replace regioncode="542623" if regioncode=="542623" & year==2007

replace regioncode="542625" if regioncode=="542625" & year==2007

replace regioncode="542626" if regioncode=="542626" & year==2007

replace regioncode="610102" if regioncode=="610102" & year==2007

replace regioncode="610103" if regioncode=="610103" & year==2007

replace regioncode="610104" if regioncode=="610104" & year==2007

replace regioncode="610111" if regioncode=="610111" & year==2007

replace regioncode="610112" if regioncode=="610112" & year==2007

replace regioncode="610113" if regioncode=="610113" & year==2007

replace regioncode="610114" if regioncode=="610114" & year==2007

replace regioncode="610115" if regioncode=="610115" & year==2007

replace regioncode="610116" if regioncode=="610116" & year==2007

replace regioncode="610122" if regioncode=="610122" & year==2007

replace regioncode="610124" if regioncode=="610124" & year==2007

replace regioncode="610125" if regioncode=="610125" & year==2007

replace regioncode="610126" if regioncode=="610126" & year==2007

replace regioncode="610202" if regioncode=="610202" & year==2007

replace regioncode="610203" if regioncode=="610203" & year==2007

replace regioncode="610204" if regioncode=="610204" & year==2007

replace regioncode="610222" if regioncode=="610222" & year==2007

replace regioncode="610302" if regioncode=="610302" & year==2007

replace regioncode="610303" if regioncode=="610303" & year==2007

replace regioncode="610304" if regioncode=="610304" & year==2007

replace regioncode="610322" if regioncode=="610322" & year==2007

replace regioncode="610323" if regioncode=="610323" & year==2007

replace regioncode="610324" if regioncode=="610324" & year==2007

replace regioncode="610326" if regioncode=="610326" & year==2007

replace regioncode="610327" if regioncode=="610327" & year==2007

replace regioncode="610328" if regioncode=="610328" & year==2007

replace regioncode="610329" if regioncode=="610329" & year==2007

replace regioncode="610330" if regioncode=="610330" & year==2007

replace regioncode="610331" if regioncode=="610331" & year==2007

replace regioncode="610402" if regioncode=="610402" & year==2007

replace regioncode="610403" if regioncode=="610403" & year==2007

replace regioncode="610404" if regioncode=="610404" & year==2007

replace regioncode="610422" if regioncode=="610422" & year==2007

replace regioncode="610423" if regioncode=="610423" & year==2007

replace regioncode="610424" if regioncode=="610424" & year==2007

replace regioncode="610425" if regioncode=="610425" & year==2007

replace regioncode="610426" if regioncode=="610426" & year==2007

replace regioncode="610427" if regioncode=="610427" & year==2007

replace regioncode="610428" if regioncode=="610428" & year==2007

replace regioncode="610429" if regioncode=="610429" & year==2007

replace regioncode="610430" if regioncode=="610430" & year==2007

replace regioncode="610431" if regioncode=="610431" & year==2007

replace regioncode="610481" if regioncode=="610481" & year==2007

replace regioncode="610502" if regioncode=="610502" & year==2007

replace regioncode="610521" if regioncode=="610521" & year==2007

replace regioncode="610522" if regioncode=="610522" & year==2007

replace regioncode="610523" if regioncode=="610523" & year==2007

replace regioncode="610524" if regioncode=="610524" & year==2007

replace regioncode="610525" if regioncode=="610525" & year==2007

replace regioncode="610526" if regioncode=="610526" & year==2007

replace regioncode="610527" if regioncode=="610527" & year==2007

replace regioncode="610528" if regioncode=="610528" & year==2007

replace regioncode="610581" if regioncode=="610581" & year==2007

replace regioncode="610582" if regioncode=="610582" & year==2007

replace regioncode="610602" if regioncode=="610602" & year==2007

replace regioncode="610621" if regioncode=="610621" & year==2007

replace regioncode="610622" if regioncode=="610622" & year==2007

replace regioncode="610623" if regioncode=="610623" & year==2007

replace regioncode="610624" if regioncode=="610624" & year==2007

replace regioncode="610625" if regioncode=="610625" & year==2007

replace regioncode="610626" if regioncode=="610626" & year==2007

replace regioncode="610627" if regioncode=="610627" & year==2007

replace regioncode="610628" if regioncode=="610628" & year==2007

replace regioncode="610629" if regioncode=="610629" & year==2007

replace regioncode="610630" if regioncode=="610630" & year==2007

replace regioncode="610631" if regioncode=="610631" & year==2007

replace regioncode="610632" if regioncode=="610632" & year==2007

replace regioncode="610702" if regioncode=="610702" & year==2007

replace regioncode="610721" if regioncode=="610721" & year==2007

replace regioncode="610722" if regioncode=="610722" & year==2007

replace regioncode="610723" if regioncode=="610723" & year==2007

replace regioncode="610724" if regioncode=="610724" & year==2007

replace regioncode="610725" if regioncode=="610725" & year==2007

replace regioncode="610726" if regioncode=="610726" & year==2007

replace regioncode="610727" if regioncode=="610727" & year==2007

replace regioncode="610728" if regioncode=="610728" & year==2007

replace regioncode="610729" if regioncode=="610729" & year==2007

replace regioncode="610730" if regioncode=="610730" & year==2007

replace regioncode="610802" if regioncode=="610802" & year==2007

replace regioncode="610821" if regioncode=="610821" & year==2007

replace regioncode="610822" if regioncode=="610822" & year==2007

replace regioncode="610823" if regioncode=="610823" & year==2007

replace regioncode="610824" if regioncode=="610824" & year==2007

replace regioncode="610825" if regioncode=="610825" & year==2007

replace regioncode="610826" if regioncode=="610826" & year==2007

replace regioncode="610827" if regioncode=="610827" & year==2007

replace regioncode="610828" if regioncode=="610828" & year==2007

replace regioncode="610829" if regioncode=="610829" & year==2007

replace regioncode="610830" if regioncode=="610830" & year==2007

replace regioncode="610831" if regioncode=="610831" & year==2007

replace regioncode="610902" if regioncode=="610902" & year==2007

replace regioncode="610921" if regioncode=="610921" & year==2007

replace regioncode="610922" if regioncode=="610922" & year==2007

replace regioncode="610923" if regioncode=="610923" & year==2007

replace regioncode="610924" if regioncode=="610924" & year==2007

replace regioncode="610925" if regioncode=="610925" & year==2007

replace regioncode="610926" if regioncode=="610926" & year==2007

replace regioncode="610927" if regioncode=="610927" & year==2007

replace regioncode="610928" if regioncode=="610928" & year==2007

replace regioncode="610929" if regioncode=="610929" & year==2007

replace regioncode="611002" if regioncode=="611002" & year==2007

replace regioncode="611021" if regioncode=="611021" & year==2007

replace regioncode="611022" if regioncode=="611022" & year==2007

replace regioncode="611023" if regioncode=="611023" & year==2007

replace regioncode="611024" if regioncode=="611024" & year==2007

replace regioncode="611025" if regioncode=="611025" & year==2007

replace regioncode="611026" if regioncode=="611026" & year==2007

replace regioncode="620102" if regioncode=="620102" & year==2007

replace regioncode="620103" if regioncode=="620103" & year==2007

replace regioncode="620104" if regioncode=="620104" & year==2007

replace regioncode="620105" if regioncode=="620105" & year==2007

replace regioncode="620111" if regioncode=="620111" & year==2007

replace regioncode="620121" if regioncode=="620121" & year==2007

replace regioncode="620122" if regioncode=="620122" & year==2007

replace regioncode="620123" if regioncode=="620123" & year==2007

replace regioncode="620201" if regioncode=="620201" & year==2007

replace regioncode="620302" if regioncode=="620302" & year==2007

replace regioncode="620321" if regioncode=="620321" & year==2007

replace regioncode="620402" if regioncode=="620402" & year==2007

replace regioncode="620403" if regioncode=="620403" & year==2007

replace regioncode="620421" if regioncode=="620421" & year==2007

replace regioncode="620422" if regioncode=="620422" & year==2007

replace regioncode="620423" if regioncode=="620423" & year==2007

replace regioncode="620502" if regioncode=="620502" & year==2007

replace regioncode="620503" if regioncode=="620503" & year==2007

replace regioncode="620521" if regioncode=="620521" & year==2007

replace regioncode="620522" if regioncode=="620522" & year==2007

replace regioncode="620523" if regioncode=="620523" & year==2007

replace regioncode="620524" if regioncode=="620524" & year==2007

replace regioncode="620525" if regioncode=="620525" & year==2007

replace regioncode="622301" if regioncode=="620602" & year==2007

replace regioncode="622322" if regioncode=="620621" & year==2007

replace regioncode="622323" if regioncode=="620622" & year==2007

replace regioncode="622326" if regioncode=="620623" & year==2007

replace regioncode="622201" if regioncode=="620702" & year==2007

replace regioncode="622222" if regioncode=="620721" & year==2007

replace regioncode="622223" if regioncode=="620722" & year==2007

replace regioncode="622224" if regioncode=="620723" & year==2007

replace regioncode="622225" if regioncode=="620724" & year==2007

replace regioncode="622226" if regioncode=="620725" & year==2007

replace regioncode="622701" if regioncode=="620802" & year==2007

replace regioncode="622722" if regioncode=="620821" & year==2007

replace regioncode="622723" if regioncode=="620822" & year==2007

replace regioncode="622724" if regioncode=="620823" & year==2007

replace regioncode="622725" if regioncode=="620824" & year==2007

replace regioncode="622726" if regioncode=="620825" & year==2007

replace regioncode="622727" if regioncode=="620826" & year==2007

replace regioncode="622102" if regioncode=="620902" & year==2007

replace regioncode="622123" if regioncode=="620921" & year==2007

replace regioncode="622126" if regioncode=="620922" & year==2007

replace regioncode="622124" if regioncode=="620923" & year==2007

replace regioncode="622125" if regioncode=="620924" & year==2007

replace regioncode="622101" if regioncode=="620981" & year==2007

replace regioncode="622103" if regioncode=="620982" & year==2007

replace regioncode="622801" if regioncode=="621002" & year==2007

replace regioncode="622821" if regioncode=="621021" & year==2007

replace regioncode="622822" if regioncode=="621022" & year==2007

replace regioncode="622823" if regioncode=="621023" & year==2007

replace regioncode="622824" if regioncode=="621024" & year==2007

replace regioncode="622825" if regioncode=="621025" & year==2007

replace regioncode="622826" if regioncode=="621026" & year==2007

replace regioncode="622827" if regioncode=="621027" & year==2007

replace regioncode="622421" if regioncode=="621102" & year==2007

replace regioncode="622424" if regioncode=="621121" & year==2007

replace regioncode="622425" if regioncode=="621122" & year==2007

replace regioncode="622426" if regioncode=="621123" & year==2007

replace regioncode="622427" if regioncode=="621124" & year==2007

replace regioncode="622428" if regioncode=="621125" & year==2007

replace regioncode="622429" if regioncode=="621126" & year==2007

replace regioncode="622621" if regioncode=="621202" & year==2007

replace regioncode="622624" if regioncode=="621221" & year==2007

replace regioncode="622626" if regioncode=="621222" & year==2007

replace regioncode="622623" if regioncode=="621223" & year==2007

replace regioncode="622625" if regioncode=="621224" & year==2007

replace regioncode="622627" if regioncode=="621225" & year==2007

replace regioncode="622628" if regioncode=="621226" & year==2007

replace regioncode="622630" if regioncode=="621227" & year==2007

replace regioncode="622629" if regioncode=="621228" & year==2007

replace regioncode="622901" if regioncode=="622901" & year==2007

replace regioncode="622921" if regioncode=="622921" & year==2007

replace regioncode="622922" if regioncode=="622922" & year==2007

replace regioncode="622923" if regioncode=="622923" & year==2007

replace regioncode="622924" if regioncode=="622924" & year==2007

replace regioncode="622925" if regioncode=="622925" & year==2007

replace regioncode="622926" if regioncode=="622926" & year==2007

replace regioncode="622927" if regioncode=="622927" & year==2007

replace regioncode="623001" if regioncode=="623001" & year==2007

replace regioncode="623021" if regioncode=="623021" & year==2007

replace regioncode="623022" if regioncode=="623022" & year==2007

replace regioncode="623023" if regioncode=="623023" & year==2007

replace regioncode="623024" if regioncode=="623024" & year==2007

replace regioncode="623025" if regioncode=="623025" & year==2007

replace regioncode="623026" if regioncode=="623026" & year==2007

replace regioncode="623027" if regioncode=="623027" & year==2007

replace regioncode="630102" if regioncode=="630102" & year==2007

replace regioncode="630103" if regioncode=="630103" & year==2007

replace regioncode="630104" if regioncode=="630104" & year==2007

replace regioncode="630105" if regioncode=="630105" & year==2007

replace regioncode="630121" if regioncode=="630121" & year==2007

replace regioncode="630122" if regioncode=="630122" & year==2007

replace regioncode="630123" if regioncode=="630123" & year==2007

replace regioncode="632121" if regioncode=="632121" & year==2007

replace regioncode="632122" if regioncode=="632122" & year==2007

replace regioncode="632123" if regioncode=="632123" & year==2007

replace regioncode="632126" if regioncode=="632126" & year==2007

replace regioncode="632127" if regioncode=="632127" & year==2007

replace regioncode="632128" if regioncode=="632128" & year==2007

replace regioncode="632221" if regioncode=="632221" & year==2007

replace regioncode="632222" if regioncode=="632222" & year==2007

replace regioncode="632223" if regioncode=="632223" & year==2007

replace regioncode="632224" if regioncode=="632224" & year==2007

replace regioncode="632321" if regioncode=="632321" & year==2007

replace regioncode="632322" if regioncode=="632322" & year==2007

replace regioncode="632521" if regioncode=="632521" & year==2007

replace regioncode="632522" if regioncode=="632522" & year==2007

replace regioncode="632523" if regioncode=="632523" & year==2007

replace regioncode="632524" if regioncode=="632524" & year==2007

replace regioncode="632525" if regioncode=="632525" & year==2007

replace regioncode="632621" if regioncode=="632621" & year==2007

replace regioncode="632622" if regioncode=="632622" & year==2007

replace regioncode="632623" if regioncode=="632623" & year==2007

replace regioncode="632624" if regioncode=="632624" & year==2007

replace regioncode="632625" if regioncode=="632625" & year==2007

replace regioncode="632626" if regioncode=="632626" & year==2007

replace regioncode="632721" if regioncode=="632721" & year==2007

replace regioncode="632723" if regioncode=="632723" & year==2007

replace regioncode="632724" if regioncode=="632724" & year==2007

replace regioncode="632725" if regioncode=="632725" & year==2007

replace regioncode="632726" if regioncode=="632726" & year==2007

replace regioncode="632801" if regioncode=="632801" & year==2007

replace regioncode="632802" if regioncode=="632802" & year==2007

replace regioncode="632821" if regioncode=="632821" & year==2007

replace regioncode="632822" if regioncode=="632822" & year==2007

replace regioncode="640104" if regioncode=="640104" & year==2007

replace regioncode="640105" if regioncode=="640105" & year==2007

replace regioncode="640106" if regioncode=="640106" & year==2007

replace regioncode="640121" if regioncode=="640121" & year==2007

replace regioncode="640122" if regioncode=="640122" & year==2007

replace regioncode="640181" if regioncode=="640181" & year==2007

replace regioncode="640202" if regioncode=="640202" & year==2007

replace regioncode="640203" if regioncode=="640205" & year==2007

replace regioncode="640221" if regioncode=="640221" & year==2007

replace regioncode="640302" if regioncode=="640302" & year==2007

replace regioncode="640321" if regioncode=="640501" & year==2007

replace regioncode="640322" if regioncode=="640521" & year==2007

replace regioncode="640323" if regioncode=="640323" & year==2007

replace regioncode="640324" if regioncode=="640324" & year==2007

replace regioncode="640381" if regioncode=="640381" & year==2007

replace regioncode="640402" if regioncode=="640402" & year==2007

replace regioncode="640421" if regioncode=="640522" & year==2007

replace regioncode="640422" if regioncode=="640422" & year==2007

replace regioncode="640423" if regioncode=="640423" & year==2007

replace regioncode="640424" if regioncode=="640424" & year==2007

replace regioncode="640425" if regioncode=="640425" & year==2007

replace regioncode="650102" if regioncode=="650102" & year==2007

replace regioncode="650103" if regioncode=="650103" & year==2007

replace regioncode="650104" if regioncode=="650104" & year==2007

replace regioncode="650105" if regioncode=="650105" & year==2007

replace regioncode="650106" if regioncode=="650106" & year==2007

replace regioncode="650107" if regioncode=="650107" & year==2007

replace regioncode="650108" if regioncode=="650108" & year==2007

replace regioncode="650121" if regioncode=="650121" & year==2007

replace regioncode="650202" if regioncode=="650202" & year==2007

replace regioncode="650203" if regioncode=="650203" & year==2007

replace regioncode="650204" if regioncode=="650204" & year==2007

replace regioncode="650205" if regioncode=="650205" & year==2007

replace regioncode="652101" if regioncode=="652101" & year==2007

replace regioncode="652122" if regioncode=="652122" & year==2007

replace regioncode="652123" if regioncode=="652123" & year==2007

replace regioncode="652201" if regioncode=="652201" & year==2007

replace regioncode="652222" if regioncode=="652222" & year==2007

replace regioncode="652223" if regioncode=="652223" & year==2007

replace regioncode="652301" if regioncode=="652301" & year==2007

replace regioncode="652302" if regioncode=="652302" & year==2007

replace regioncode="652303" if regioncode=="652303" & year==2007

replace regioncode="652323" if regioncode=="652323" & year==2007

replace regioncode="652324" if regioncode=="652324" & year==2007

replace regioncode="652325" if regioncode=="652325" & year==2007

replace regioncode="652327" if regioncode=="652327" & year==2007

replace regioncode="652328" if regioncode=="652328" & year==2007

replace regioncode="652701" if regioncode=="652701" & year==2007

replace regioncode="652722" if regioncode=="652722" & year==2007

replace regioncode="652723" if regioncode=="652723" & year==2007

replace regioncode="652801" if regioncode=="652801" & year==2007

replace regioncode="652822" if regioncode=="652822" & year==2007

replace regioncode="652823" if regioncode=="652823" & year==2007

replace regioncode="652824" if regioncode=="652824" & year==2007

replace regioncode="652825" if regioncode=="652825" & year==2007

replace regioncode="652826" if regioncode=="652826" & year==2007

replace regioncode="652827" if regioncode=="652827" & year==2007

replace regioncode="652828" if regioncode=="652828" & year==2007

replace regioncode="652829" if regioncode=="652829" & year==2007

replace regioncode="652901" if regioncode=="652901" & year==2007

replace regioncode="652922" if regioncode=="652922" & year==2007

replace regioncode="652923" if regioncode=="652923" & year==2007

replace regioncode="652924" if regioncode=="652924" & year==2007

replace regioncode="652925" if regioncode=="652925" & year==2007

replace regioncode="652926" if regioncode=="652926" & year==2007

replace regioncode="652928" if regioncode=="652928" & year==2007

replace regioncode="652929" if regioncode=="652929" & year==2007

replace regioncode="653001" if regioncode=="653001" & year==2007

replace regioncode="653022" if regioncode=="653022" & year==2007

replace regioncode="653023" if regioncode=="653023" & year==2007

replace regioncode="653024" if regioncode=="653024" & year==2007

replace regioncode="653101" if regioncode=="653101" & year==2007

replace regioncode="653121" if regioncode=="653121" & year==2007

replace regioncode="653122" if regioncode=="653122" & year==2007

replace regioncode="653123" if regioncode=="653123" & year==2007

replace regioncode="653124" if regioncode=="653124" & year==2007

replace regioncode="653125" if regioncode=="653125" & year==2007

replace regioncode="653126" if regioncode=="653126" & year==2007

replace regioncode="653127" if regioncode=="653127" & year==2007

replace regioncode="653128" if regioncode=="653128" & year==2007

replace regioncode="653129" if regioncode=="653129" & year==2007

replace regioncode="653130" if regioncode=="653130" & year==2007

replace regioncode="653131" if regioncode=="653131" & year==2007

replace regioncode="653201" if regioncode=="653201" & year==2007

replace regioncode="653221" if regioncode=="653221" & year==2007

replace regioncode="653222" if regioncode=="653222" & year==2007

replace regioncode="653223" if regioncode=="653223" & year==2007

replace regioncode="653224" if regioncode=="653224" & year==2007

replace regioncode="653225" if regioncode=="653225" & year==2007

replace regioncode="653226" if regioncode=="653226" & year==2007

replace regioncode="653227" if regioncode=="653227" & year==2007

replace regioncode="654002" if regioncode=="654002" & year==2007

replace regioncode="654003" if regioncode=="654003" & year==2007

replace regioncode="654021" if regioncode=="654021" & year==2007

replace regioncode="654022" if regioncode=="654022" & year==2007

replace regioncode="654023" if regioncode=="654023" & year==2007

replace regioncode="654024" if regioncode=="654024" & year==2007

replace regioncode="654025" if regioncode=="654025" & year==2007

replace regioncode="654026" if regioncode=="654026" & year==2007

replace regioncode="654027" if regioncode=="654027" & year==2007

replace regioncode="654028" if regioncode=="654028" & year==2007

replace regioncode="654201" if regioncode=="654201" & year==2007

replace regioncode="654202" if regioncode=="654202" & year==2007

replace regioncode="654221" if regioncode=="654221" & year==2007

replace regioncode="654223" if regioncode=="654223" & year==2007

replace regioncode="654224" if regioncode=="654224" & year==2007

replace regioncode="654225" if regioncode=="654225" & year==2007

replace regioncode="654226" if regioncode=="654226" & year==2007

replace regioncode="654301" if regioncode=="654301" & year==2007

replace regioncode="654321" if regioncode=="654321" & year==2007

replace regioncode="654322" if regioncode=="654322" & year==2007

replace regioncode="654323" if regioncode=="654323" & year==2007

replace regioncode="654324" if regioncode=="654324" & year==2007

replace regioncode="654325" if regioncode=="654325" & year==2007

replace regioncode="654326" if regioncode=="654326" & year==2007

replace regioncode="659001" if regioncode=="659001" & year==2007

replace regioncode="110101" if regioncode=="110101" & year==2008

replace regioncode="110102" if regioncode=="110102" & year==2008

replace regioncode="110103" if regioncode=="110103" & year==2008

replace regioncode="110104" if regioncode=="110104" & year==2008

replace regioncode="110105" if regioncode=="110105" & year==2008

replace regioncode="110106" if regioncode=="110106" & year==2008

replace regioncode="110107" if regioncode=="110107" & year==2008

replace regioncode="110108" if regioncode=="110108" & year==2008

replace regioncode="110109" if regioncode=="110109" & year==2008

replace regioncode="110111" if regioncode=="110111" & year==2008

replace regioncode="110112" if regioncode=="110112" & year==2008

replace regioncode="110113" if regioncode=="110113" & year==2008

replace regioncode="110114" if regioncode=="110114" & year==2008

replace regioncode="110115" if regioncode=="110115" & year==2008

replace regioncode="110117" if regioncode=="110117" & year==2008

replace regioncode="110116" if regioncode=="110116" & year==2008

replace regioncode="110228" if regioncode=="110228" & year==2008

replace regioncode="110229" if regioncode=="110229" & year==2008

replace regioncode="120101" if regioncode=="120101" & year==2008

replace regioncode="120102" if regioncode=="120102" & year==2008

replace regioncode="120103" if regioncode=="120103" & year==2008

replace regioncode="120104" if regioncode=="120104" & year==2008

replace regioncode="120105" if regioncode=="120105" & year==2008

replace regioncode="120106" if regioncode=="120106" & year==2008

replace regioncode="120107" if regioncode=="120107" & year==2008

replace regioncode="120108" if regioncode=="120108" & year==2008

replace regioncode="120109" if regioncode=="120109" & year==2008

replace regioncode="120110" if regioncode=="120110" & year==2008

replace regioncode="120111" if regioncode=="120111" & year==2008

replace regioncode="120112" if regioncode=="120112" & year==2008

replace regioncode="120113" if regioncode=="120113" & year==2008

replace regioncode="120114" if regioncode=="120114" & year==2008

replace regioncode="120115" if regioncode=="120115" & year==2008

replace regioncode="120221" if regioncode=="120221" & year==2008

replace regioncode="120223" if regioncode=="120223" & year==2008

replace regioncode="120225" if regioncode=="120225" & year==2008

replace regioncode="130102" if regioncode=="130102" & year==2008

replace regioncode="130103" if regioncode=="130103" & year==2008

replace regioncode="130104" if regioncode=="130104" & year==2008

replace regioncode="130105" if regioncode=="130105" & year==2008

replace regioncode="130107" if regioncode=="130107" & year==2008

replace regioncode="130108" if regioncode=="130108" & year==2008

replace regioncode="130121" if regioncode=="130121" & year==2008

replace regioncode="130123" if regioncode=="130123" & year==2008

replace regioncode="130124" if regioncode=="130124" & year==2008

replace regioncode="130125" if regioncode=="130125" & year==2008

replace regioncode="130126" if regioncode=="130126" & year==2008

replace regioncode="130127" if regioncode=="130127" & year==2008

replace regioncode="130128" if regioncode=="130128" & year==2008

replace regioncode="130129" if regioncode=="130129" & year==2008

replace regioncode="130130" if regioncode=="130130" & year==2008

replace regioncode="130131" if regioncode=="130131" & year==2008

replace regioncode="130132" if regioncode=="130132" & year==2008

replace regioncode="130133" if regioncode=="130133" & year==2008

replace regioncode="130181" if regioncode=="130181" & year==2008

replace regioncode="130182" if regioncode=="130182" & year==2008

replace regioncode="130183" if regioncode=="130183" & year==2008

replace regioncode="130184" if regioncode=="130184" & year==2008

replace regioncode="130185" if regioncode=="130185" & year==2008

replace regioncode="130202" if regioncode=="130202" & year==2008

replace regioncode="130203" if regioncode=="130203" & year==2008

replace regioncode="130204" if regioncode=="130204" & year==2008

replace regioncode="130205" if regioncode=="130205" & year==2008

replace regioncode="130208" if regioncode=="130208" & year==2008

replace regioncode="130223" if regioncode=="130223" & year==2008

replace regioncode="130224" if regioncode=="130224" & year==2008

replace regioncode="130225" if regioncode=="130225" & year==2008

replace regioncode="130227" if regioncode=="130227" & year==2008

replace regioncode="130229" if regioncode=="130229" & year==2008

replace regioncode="130230" if regioncode=="130230" & year==2008

replace regioncode="130281" if regioncode=="130281" & year==2008

replace regioncode="130207" if regioncode=="130207" & year==2008

replace regioncode="130283" if regioncode=="130283" & year==2008

replace regioncode="130302" if regioncode=="130302" & year==2008

replace regioncode="130303" if regioncode=="130303" & year==2008

replace regioncode="130304" if regioncode=="130304" & year==2008

replace regioncode="130321" if regioncode=="130321" & year==2008

replace regioncode="130322" if regioncode=="130322" & year==2008

replace regioncode="130323" if regioncode=="130323" & year==2008

replace regioncode="130324" if regioncode=="130324" & year==2008

replace regioncode="130402" if regioncode=="130402" & year==2008

replace regioncode="130403" if regioncode=="130403" & year==2008

replace regioncode="130404" if regioncode=="130404" & year==2008

replace regioncode="130406" if regioncode=="130406" & year==2008

replace regioncode="130421" if regioncode=="130421" & year==2008

replace regioncode="130423" if regioncode=="130423" & year==2008

replace regioncode="130424" if regioncode=="130424" & year==2008

replace regioncode="130425" if regioncode=="130425" & year==2008

replace regioncode="130426" if regioncode=="130426" & year==2008

replace regioncode="130427" if regioncode=="130427" & year==2008

replace regioncode="130428" if regioncode=="130428" & year==2008

replace regioncode="130429" if regioncode=="130429" & year==2008

replace regioncode="130430" if regioncode=="130430" & year==2008

replace regioncode="130431" if regioncode=="130431" & year==2008

replace regioncode="130432" if regioncode=="130432" & year==2008

replace regioncode="130433" if regioncode=="130433" & year==2008

replace regioncode="130434" if regioncode=="130434" & year==2008

replace regioncode="130435" if regioncode=="130435" & year==2008

replace regioncode="130481" if regioncode=="130481" & year==2008

replace regioncode="130502" if regioncode=="130502" & year==2008

replace regioncode="130503" if regioncode=="130503" & year==2008

replace regioncode="130521" if regioncode=="130521" & year==2008

replace regioncode="130522" if regioncode=="130522" & year==2008

replace regioncode="130523" if regioncode=="130523" & year==2008

replace regioncode="130524" if regioncode=="130524" & year==2008

replace regioncode="130525" if regioncode=="130525" & year==2008

replace regioncode="130526" if regioncode=="130526" & year==2008

replace regioncode="130527" if regioncode=="130527" & year==2008

replace regioncode="130528" if regioncode=="130528" & year==2008

replace regioncode="130529" if regioncode=="130529" & year==2008

replace regioncode="130530" if regioncode=="130530" & year==2008

replace regioncode="130531" if regioncode=="130531" & year==2008

replace regioncode="130532" if regioncode=="130532" & year==2008

replace regioncode="130533" if regioncode=="130533" & year==2008

replace regioncode="130534" if regioncode=="130534" & year==2008

replace regioncode="130535" if regioncode=="130535" & year==2008

replace regioncode="130581" if regioncode=="130581" & year==2008

replace regioncode="130582" if regioncode=="130582" & year==2008

replace regioncode="130602" if regioncode=="130602" & year==2008

replace regioncode="130603" if regioncode=="130603" & year==2008

replace regioncode="130604" if regioncode=="130604" & year==2008

replace regioncode="130621" if regioncode=="130621" & year==2008

replace regioncode="130622" if regioncode=="130622" & year==2008

replace regioncode="130623" if regioncode=="130623" & year==2008

replace regioncode="130624" if regioncode=="130624" & year==2008

replace regioncode="130625" if regioncode=="130625" & year==2008

replace regioncode="130626" if regioncode=="130626" & year==2008

replace regioncode="130627" if regioncode=="130627" & year==2008

replace regioncode="130628" if regioncode=="130628" & year==2008

replace regioncode="130629" if regioncode=="130629" & year==2008

replace regioncode="130630" if regioncode=="130630" & year==2008

replace regioncode="130631" if regioncode=="130631" & year==2008

replace regioncode="130632" if regioncode=="130632" & year==2008

replace regioncode="130633" if regioncode=="130633" & year==2008

replace regioncode="130634" if regioncode=="130634" & year==2008

replace regioncode="130635" if regioncode=="130635" & year==2008

replace regioncode="130636" if regioncode=="130636" & year==2008

replace regioncode="130637" if regioncode=="130637" & year==2008

replace regioncode="130638" if regioncode=="130638" & year==2008

replace regioncode="130681" if regioncode=="130681" & year==2008

replace regioncode="130682" if regioncode=="130682" & year==2008

replace regioncode="130683" if regioncode=="130683" & year==2008

replace regioncode="130684" if regioncode=="130684" & year==2008

replace regioncode="130702" if regioncode=="130702" & year==2008

replace regioncode="130703" if regioncode=="130703" & year==2008

replace regioncode="130705" if regioncode=="130705" & year==2008

replace regioncode="130706" if regioncode=="130706" & year==2008

replace regioncode="130721" if regioncode=="130721" & year==2008

replace regioncode="130722" if regioncode=="130722" & year==2008

replace regioncode="130723" if regioncode=="130723" & year==2008

replace regioncode="130724" if regioncode=="130724" & year==2008

replace regioncode="130725" if regioncode=="130725" & year==2008

replace regioncode="130726" if regioncode=="130726" & year==2008

replace regioncode="130727" if regioncode=="130727" & year==2008

replace regioncode="130728" if regioncode=="130728" & year==2008

replace regioncode="130729" if regioncode=="130729" & year==2008

replace regioncode="130730" if regioncode=="130730" & year==2008

replace regioncode="130731" if regioncode=="130731" & year==2008

replace regioncode="130732" if regioncode=="130732" & year==2008

replace regioncode="130733" if regioncode=="130733" & year==2008

replace regioncode="130802" if regioncode=="130802" & year==2008

replace regioncode="130803" if regioncode=="130803" & year==2008

replace regioncode="130804" if regioncode=="130804" & year==2008

replace regioncode="130821" if regioncode=="130821" & year==2008

replace regioncode="130822" if regioncode=="130822" & year==2008

replace regioncode="130823" if regioncode=="130823" & year==2008

replace regioncode="130824" if regioncode=="130824" & year==2008

replace regioncode="130825" if regioncode=="130825" & year==2008

replace regioncode="130826" if regioncode=="130826" & year==2008

replace regioncode="130827" if regioncode=="130827" & year==2008

replace regioncode="130828" if regioncode=="130828" & year==2008

replace regioncode="130902" if regioncode=="130902" & year==2008

replace regioncode="130903" if regioncode=="130903" & year==2008

replace regioncode="130921" if regioncode=="130921" & year==2008

replace regioncode="130922" if regioncode=="130922" & year==2008

replace regioncode="130923" if regioncode=="130923" & year==2008

replace regioncode="130924" if regioncode=="130924" & year==2008

replace regioncode="130925" if regioncode=="130925" & year==2008

replace regioncode="130926" if regioncode=="130926" & year==2008

replace regioncode="130927" if regioncode=="130927" & year==2008

replace regioncode="130928" if regioncode=="130928" & year==2008

replace regioncode="130929" if regioncode=="130929" & year==2008

replace regioncode="130930" if regioncode=="130930" & year==2008

replace regioncode="130981" if regioncode=="130981" & year==2008

replace regioncode="130982" if regioncode=="130982" & year==2008

replace regioncode="130983" if regioncode=="130983" & year==2008

replace regioncode="130984" if regioncode=="130984" & year==2008

replace regioncode="131002" if regioncode=="131002" & year==2008

replace regioncode="131003" if regioncode=="131003" & year==2008

replace regioncode="131022" if regioncode=="131022" & year==2008

replace regioncode="131023" if regioncode=="131023" & year==2008

replace regioncode="131024" if regioncode=="131024" & year==2008

replace regioncode="131025" if regioncode=="131025" & year==2008

replace regioncode="131026" if regioncode=="131026" & year==2008

replace regioncode="131028" if regioncode=="131028" & year==2008

replace regioncode="131081" if regioncode=="131081" & year==2008

replace regioncode="131082" if regioncode=="131082" & year==2008

replace regioncode="131102" if regioncode=="131102" & year==2008

replace regioncode="131121" if regioncode=="131121" & year==2008

replace regioncode="131122" if regioncode=="131122" & year==2008

replace regioncode="131123" if regioncode=="131123" & year==2008

replace regioncode="131124" if regioncode=="131124" & year==2008

replace regioncode="131125" if regioncode=="131125" & year==2008

replace regioncode="131126" if regioncode=="131126" & year==2008

replace regioncode="131127" if regioncode=="131127" & year==2008

replace regioncode="131128" if regioncode=="131128" & year==2008

replace regioncode="131181" if regioncode=="131181" & year==2008

replace regioncode="131182" if regioncode=="131182" & year==2008

replace regioncode="140105" if regioncode=="140105" & year==2008

replace regioncode="140106" if regioncode=="140106" & year==2008

replace regioncode="140107" if regioncode=="140107" & year==2008

replace regioncode="140108" if regioncode=="140108" & year==2008

replace regioncode="140109" if regioncode=="140109" & year==2008

replace regioncode="140110" if regioncode=="140110" & year==2008

replace regioncode="140121" if regioncode=="140121" & year==2008

replace regioncode="140122" if regioncode=="140122" & year==2008

replace regioncode="140123" if regioncode=="140123" & year==2008

replace regioncode="140181" if regioncode=="140181" & year==2008

replace regioncode="140202" if regioncode=="140202" & year==2008

replace regioncode="140203" if regioncode=="140203" & year==2008

replace regioncode="140211" if regioncode=="140211" & year==2008

replace regioncode="140212" if regioncode=="140212" & year==2008

replace regioncode="140221" if regioncode=="140221" & year==2008

replace regioncode="140222" if regioncode=="140222" & year==2008

replace regioncode="140223" if regioncode=="140223" & year==2008

replace regioncode="140224" if regioncode=="140224" & year==2008

replace regioncode="140225" if regioncode=="140225" & year==2008

replace regioncode="140226" if regioncode=="140226" & year==2008

replace regioncode="140227" if regioncode=="140227" & year==2008

replace regioncode="140302" if regioncode=="140302" & year==2008

replace regioncode="140303" if regioncode=="140303" & year==2008

replace regioncode="140311" if regioncode=="140311" & year==2008

replace regioncode="140321" if regioncode=="140321" & year==2008

replace regioncode="140322" if regioncode=="140322" & year==2008

replace regioncode="140402" if regioncode=="140402" & year==2008

replace regioncode="140411" if regioncode=="140411" & year==2008

replace regioncode="140421" if regioncode=="140421" & year==2008

replace regioncode="140423" if regioncode=="140423" & year==2008

replace regioncode="140424" if regioncode=="140424" & year==2008

replace regioncode="140425" if regioncode=="140425" & year==2008

replace regioncode="140426" if regioncode=="140426" & year==2008

replace regioncode="140427" if regioncode=="140427" & year==2008

replace regioncode="140428" if regioncode=="140428" & year==2008

replace regioncode="140429" if regioncode=="140429" & year==2008

replace regioncode="140430" if regioncode=="140430" & year==2008

replace regioncode="140431" if regioncode=="140431" & year==2008

replace regioncode="140481" if regioncode=="140481" & year==2008

replace regioncode="140502" if regioncode=="140502" & year==2008

replace regioncode="140521" if regioncode=="140521" & year==2008

replace regioncode="140522" if regioncode=="140522" & year==2008

replace regioncode="140524" if regioncode=="140524" & year==2008

replace regioncode="140525" if regioncode=="140525" & year==2008

replace regioncode="140581" if regioncode=="140581" & year==2008

replace regioncode="140602" if regioncode=="140602" & year==2008

replace regioncode="140603" if regioncode=="140603" & year==2008

replace regioncode="140621" if regioncode=="140621" & year==2008

replace regioncode="140622" if regioncode=="140622" & year==2008

replace regioncode="140623" if regioncode=="140623" & year==2008

replace regioncode="140624" if regioncode=="140624" & year==2008

replace regioncode="140902" if regioncode=="140902" & year==2008

replace regioncode="140981" if regioncode=="140981" & year==2008

replace regioncode="140921" if regioncode=="140921" & year==2008

replace regioncode="140922" if regioncode=="140922" & year==2008

replace regioncode="140923" if regioncode=="140923" & year==2008

replace regioncode="140924" if regioncode=="140924" & year==2008

replace regioncode="140925" if regioncode=="140925" & year==2008

replace regioncode="140926" if regioncode=="140926" & year==2008

replace regioncode="140927" if regioncode=="140927" & year==2008

replace regioncode="140928" if regioncode=="140928" & year==2008

replace regioncode="140929" if regioncode=="140929" & year==2008

replace regioncode="140930" if regioncode=="140930" & year==2008

replace regioncode="140931" if regioncode=="140931" & year==2008

replace regioncode="140932" if regioncode=="140932" & year==2008

replace regioncode="141181" if regioncode=="141181" & year==2008

replace regioncode="141102" if regioncode=="141102" & year==2008

replace regioncode="141182" if regioncode=="141182" & year==2008

replace regioncode="141121" if regioncode=="141121" & year==2008

replace regioncode="141122" if regioncode=="141122" & year==2008

replace regioncode="141123" if regioncode=="141123" & year==2008

replace regioncode="141124" if regioncode=="141124" & year==2008

replace regioncode="141125" if regioncode=="141125" & year==2008

replace regioncode="141126" if regioncode=="141126" & year==2008

replace regioncode="141127" if regioncode=="141127" & year==2008

replace regioncode="141128" if regioncode=="141128" & year==2008

replace regioncode="141129" if regioncode=="141129" & year==2008

replace regioncode="141130" if regioncode=="141130" & year==2008

replace regioncode="140702" if regioncode=="140702" & year==2008

replace regioncode="140781" if regioncode=="140781" & year==2008

replace regioncode="140721" if regioncode=="140721" & year==2008

replace regioncode="140722" if regioncode=="140722" & year==2008

replace regioncode="140723" if regioncode=="140723" & year==2008

replace regioncode="140724" if regioncode=="140724" & year==2008

replace regioncode="140725" if regioncode=="140725" & year==2008

replace regioncode="140726" if regioncode=="140726" & year==2008

replace regioncode="140727" if regioncode=="140727" & year==2008

replace regioncode="140728" if regioncode=="140728" & year==2008

replace regioncode="140729" if regioncode=="140729" & year==2008

replace regioncode="141002" if regioncode=="141002" & year==2008

replace regioncode="141081" if regioncode=="141081" & year==2008

replace regioncode="141082" if regioncode=="141082" & year==2008

replace regioncode="141021" if regioncode=="141021" & year==2008

replace regioncode="141022" if regioncode=="141022" & year==2008

replace regioncode="141023" if regioncode=="141023" & year==2008

replace regioncode="141024" if regioncode=="141024" & year==2008

replace regioncode="141025" if regioncode=="141025" & year==2008

replace regioncode="141026" if regioncode=="141026" & year==2008

replace regioncode="141027" if regioncode=="141027" & year==2008

replace regioncode="141028" if regioncode=="141028" & year==2008

replace regioncode="141029" if regioncode=="141029" & year==2008

replace regioncode="141033" if regioncode=="141033" & year==2008

replace regioncode="141030" if regioncode=="141030" & year==2008

replace regioncode="141032" if regioncode=="141032" & year==2008

replace regioncode="141031" if regioncode=="141031" & year==2008

replace regioncode="141034" if regioncode=="141034" & year==2008

replace regioncode="140802" if regioncode=="140802" & year==2008

replace regioncode="140881" if regioncode=="140881" & year==2008

replace regioncode="140882" if regioncode=="140882" & year==2008

replace regioncode="140830" if regioncode=="140830" & year==2008

replace regioncode="140821" if regioncode=="140821" & year==2008

replace regioncode="140822" if regioncode=="140822" & year==2008

replace regioncode="140825" if regioncode=="140825" & year==2008

replace regioncode="140824" if regioncode=="140824" & year==2008

replace regioncode="140823" if regioncode=="140823" & year==2008

replace regioncode="140828" if regioncode=="140828" & year==2008

replace regioncode="140826" if regioncode=="140826" & year==2008

replace regioncode="140829" if regioncode=="140829" & year==2008

replace regioncode="140827" if regioncode=="140827" & year==2008

replace regioncode="150102" if regioncode=="150102" & year==2008

replace regioncode="150103" if regioncode=="150103" & year==2008

replace regioncode="150104" if regioncode=="150104" & year==2008

replace regioncode="150105" if regioncode=="150105" & year==2008

replace regioncode="150121" if regioncode=="150121" & year==2008

replace regioncode="150122" if regioncode=="150122" & year==2008

replace regioncode="150123" if regioncode=="150123" & year==2008

replace regioncode="150124" if regioncode=="150124" & year==2008

replace regioncode="150125" if regioncode=="150125" & year==2008

replace regioncode="150202" if regioncode=="150202" & year==2008

replace regioncode="150203" if regioncode=="150203" & year==2008

replace regioncode="150204" if regioncode=="150204" & year==2008

replace regioncode="150205" if regioncode=="150205" & year==2008

replace regioncode="150206" if regioncode=="150206" & year==2008

replace regioncode="150207" if regioncode=="150207" & year==2008

replace regioncode="150221" if regioncode=="150221" & year==2008

replace regioncode="150222" if regioncode=="150222" & year==2008

replace regioncode="150223" if regioncode=="150223" & year==2008

replace regioncode="150302" if regioncode=="150302" & year==2008

replace regioncode="150303" if regioncode=="150303" & year==2008

replace regioncode="150304" if regioncode=="150304" & year==2008

replace regioncode="150402" if regioncode=="150402" & year==2008

replace regioncode="150403" if regioncode=="150403" & year==2008

replace regioncode="150404" if regioncode=="150404" & year==2008

replace regioncode="150421" if regioncode=="150421" & year==2008

replace regioncode="150422" if regioncode=="150422" & year==2008

replace regioncode="150423" if regioncode=="150423" & year==2008

replace regioncode="150424" if regioncode=="150424" & year==2008

replace regioncode="150425" if regioncode=="150425" & year==2008

replace regioncode="150426" if regioncode=="150426" & year==2008

replace regioncode="150428" if regioncode=="150428" & year==2008

replace regioncode="150429" if regioncode=="150429" & year==2008

replace regioncode="150430" if regioncode=="150430" & year==2008

replace regioncode="150702" if regioncode=="150702" & year==2008

replace regioncode="150781" if regioncode=="150781" & year==2008

replace regioncode="150783" if regioncode=="150783" & year==2008

replace regioncode="150782" if regioncode=="150782" & year==2008

replace regioncode="150785" if regioncode=="150785" & year==2008

replace regioncode="150784" if regioncode=="150784" & year==2008

replace regioncode="150721" if regioncode=="150721" & year==2008

replace regioncode="150722" if regioncode=="150722" & year==2008

replace regioncode="150723" if regioncode=="150723" & year==2008

replace regioncode="150724" if regioncode=="150724" & year==2008

replace regioncode="150727" if regioncode=="150727" & year==2008

replace regioncode="150726" if regioncode=="150726" & year==2008

replace regioncode="150725" if regioncode=="150725" & year==2008

replace regioncode="152201" if regioncode=="152201" & year==2008

replace regioncode="152202" if regioncode=="152202" & year==2008

replace regioncode="152221" if regioncode=="152221" & year==2008

replace regioncode="152222" if regioncode=="152222" & year==2008

replace regioncode="152223" if regioncode=="152223" & year==2008

replace regioncode="152224" if regioncode=="152224" & year==2008

replace regioncode="150502" if regioncode=="150502" & year==2008

replace regioncode="150581" if regioncode=="150581" & year==2008

replace regioncode="150521" if regioncode=="150521" & year==2008

replace regioncode="150522" if regioncode=="150522" & year==2008

replace regioncode="150523" if regioncode=="150523" & year==2008

replace regioncode="150524" if regioncode=="150524" & year==2008

replace regioncode="150525" if regioncode=="150525" & year==2008

replace regioncode="150526" if regioncode=="150526" & year==2008

replace regioncode="152501" if regioncode=="152501" & year==2008

replace regioncode="152502" if regioncode=="152502" & year==2008

replace regioncode="152522" if regioncode=="152522" & year==2008

replace regioncode="152523" if regioncode=="152523" & year==2008

replace regioncode="152524" if regioncode=="152524" & year==2008

replace regioncode="152525" if regioncode=="152525" & year==2008

replace regioncode="152526" if regioncode=="152526" & year==2008

replace regioncode="152527" if regioncode=="152527" & year==2008

replace regioncode="152528" if regioncode=="152528" & year==2008

replace regioncode="152529" if regioncode=="152529" & year==2008

replace regioncode="152530" if regioncode=="152530" & year==2008

replace regioncode="152531" if regioncode=="152531" & year==2008

replace regioncode="152601" if regioncode=="150902" & year==2008

replace regioncode="152602" if regioncode=="150981" & year==2008

replace regioncode="152624" if regioncode=="150921" & year==2008

replace regioncode="152625" if regioncode=="150922" & year==2008

replace regioncode="152626" if regioncode=="150923" & year==2008

replace regioncode="152627" if regioncode=="150924" & year==2008

replace regioncode="152629" if regioncode=="150925" & year==2008

replace regioncode="152630" if regioncode=="150926" & year==2008

replace regioncode="152631" if regioncode=="150927" & year==2008

replace regioncode="152632" if regioncode=="150928" & year==2008

replace regioncode="152634" if regioncode=="150929" & year==2008

replace regioncode="150602" if regioncode=="150602" & year==2008

replace regioncode="150621" if regioncode=="150621" & year==2008

replace regioncode="150622" if regioncode=="150622" & year==2008

replace regioncode="150623" if regioncode=="150623" & year==2008

replace regioncode="150624" if regioncode=="150624" & year==2008

replace regioncode="150625" if regioncode=="150625" & year==2008

replace regioncode="150626" if regioncode=="150626" & year==2008

replace regioncode="150627" if regioncode=="150627" & year==2008

replace regioncode="152801" if regioncode=="150802" & year==2008

replace regioncode="152822" if regioncode=="150821" & year==2008

replace regioncode="152823" if regioncode=="150822" & year==2008

replace regioncode="152824" if regioncode=="150823" & year==2008

replace regioncode="152825" if regioncode=="150824" & year==2008

replace regioncode="152826" if regioncode=="150825" & year==2008

replace regioncode="152827" if regioncode=="150826" & year==2008

replace regioncode="152921" if regioncode=="152921" & year==2008

replace regioncode="152922" if regioncode=="152922" & year==2008

replace regioncode="152923" if regioncode=="152923" & year==2008

replace regioncode="210102" if regioncode=="210102" & year==2008

replace regioncode="210103" if regioncode=="210103" & year==2008

replace regioncode="210104" if regioncode=="210104" & year==2008

replace regioncode="210105" if regioncode=="210105" & year==2008

replace regioncode="210106" if regioncode=="210106" & year==2008

replace regioncode="210111" if regioncode=="210111" & year==2008

replace regioncode="210112" if regioncode=="210112" & year==2008

replace regioncode="210113" if regioncode=="210113" & year==2008

replace regioncode="210114" if regioncode=="210114" & year==2008

replace regioncode="210122" if regioncode=="210122" & year==2008

replace regioncode="210123" if regioncode=="210123" & year==2008

replace regioncode="210124" if regioncode=="210124" & year==2008

replace regioncode="210181" if regioncode=="210181" & year==2008

replace regioncode="210202" if regioncode=="210202" & year==2008

replace regioncode="210203" if regioncode=="210203" & year==2008

replace regioncode="210204" if regioncode=="210204" & year==2008

replace regioncode="210211" if regioncode=="210211" & year==2008

replace regioncode="210212" if regioncode=="210212" & year==2008

replace regioncode="210213" if regioncode=="210213" & year==2008

replace regioncode="210224" if regioncode=="210224" & year==2008

replace regioncode="210281" if regioncode=="210281" & year==2008

replace regioncode="210282" if regioncode=="210282" & year==2008

replace regioncode="210283" if regioncode=="210283" & year==2008

replace regioncode="210302" if regioncode=="210302" & year==2008

replace regioncode="210303" if regioncode=="210303" & year==2008

replace regioncode="210304" if regioncode=="210304" & year==2008

replace regioncode="210311" if regioncode=="210311" & year==2008

replace regioncode="210321" if regioncode=="210321" & year==2008

replace regioncode="210323" if regioncode=="210323" & year==2008

replace regioncode="210381" if regioncode=="210381" & year==2008

replace regioncode="210402" if regioncode=="210402" & year==2008

replace regioncode="210403" if regioncode=="210403" & year==2008

replace regioncode="210404" if regioncode=="210404" & year==2008

replace regioncode="210411" if regioncode=="210411" & year==2008

replace regioncode="210421" if regioncode=="210421" & year==2008

replace regioncode="210422" if regioncode=="210422" & year==2008

replace regioncode="210423" if regioncode=="210423" & year==2008

replace regioncode="210502" if regioncode=="210502" & year==2008

replace regioncode="210503" if regioncode=="210503" & year==2008

replace regioncode="210504" if regioncode=="210504" & year==2008

replace regioncode="210505" if regioncode=="210505" & year==2008

replace regioncode="210521" if regioncode=="210521" & year==2008

replace regioncode="210522" if regioncode=="210522" & year==2008

replace regioncode="210602" if regioncode=="210602" & year==2008

replace regioncode="210603" if regioncode=="210603" & year==2008

replace regioncode="210604" if regioncode=="210604" & year==2008

replace regioncode="210624" if regioncode=="210624" & year==2008

replace regioncode="210681" if regioncode=="210681" & year==2008

replace regioncode="210682" if regioncode=="210682" & year==2008

replace regioncode="210702" if regioncode=="210702" & year==2008

replace regioncode="210703" if regioncode=="210703" & year==2008

replace regioncode="210711" if regioncode=="210711" & year==2008

replace regioncode="210726" if regioncode=="210726" & year==2008

replace regioncode="210727" if regioncode=="210727" & year==2008

replace regioncode="210781" if regioncode=="210781" & year==2008

replace regioncode="210782" if regioncode=="210782" & year==2008

replace regioncode="210802" if regioncode=="210802" & year==2008

replace regioncode="210803" if regioncode=="210803" & year==2008

replace regioncode="210804" if regioncode=="210804" & year==2008

replace regioncode="210811" if regioncode=="210811" & year==2008

replace regioncode="210881" if regioncode=="210881" & year==2008

replace regioncode="210882" if regioncode=="210882" & year==2008

replace regioncode="210902" if regioncode=="210902" & year==2008

replace regioncode="210903" if regioncode=="210903" & year==2008

replace regioncode="210904" if regioncode=="210904" & year==2008

replace regioncode="210905" if regioncode=="210905" & year==2008

replace regioncode="210911" if regioncode=="210911" & year==2008

replace regioncode="210921" if regioncode=="210921" & year==2008

replace regioncode="210922" if regioncode=="210922" & year==2008

replace regioncode="211002" if regioncode=="211002" & year==2008

replace regioncode="211003" if regioncode=="211003" & year==2008

replace regioncode="211004" if regioncode=="211004" & year==2008

replace regioncode="211005" if regioncode=="211005" & year==2008

replace regioncode="211011" if regioncode=="211011" & year==2008

replace regioncode="211021" if regioncode=="211021" & year==2008

replace regioncode="211081" if regioncode=="211081" & year==2008

replace regioncode="211102" if regioncode=="211102" & year==2008

replace regioncode="211103" if regioncode=="211103" & year==2008

replace regioncode="211121" if regioncode=="211121" & year==2008

replace regioncode="211122" if regioncode=="211122" & year==2008

replace regioncode="211202" if regioncode=="211202" & year==2008

replace regioncode="211204" if regioncode=="211204" & year==2008

replace regioncode="211221" if regioncode=="211221" & year==2008

replace regioncode="211223" if regioncode=="211223" & year==2008

replace regioncode="211224" if regioncode=="211224" & year==2008

replace regioncode="211281" if regioncode=="211281" & year==2008

replace regioncode="211282" if regioncode=="211282" & year==2008

replace regioncode="211302" if regioncode=="211302" & year==2008

replace regioncode="211303" if regioncode=="211303" & year==2008

replace regioncode="211321" if regioncode=="211321" & year==2008

replace regioncode="211322" if regioncode=="211322" & year==2008

replace regioncode="211324" if regioncode=="211324" & year==2008

replace regioncode="211381" if regioncode=="211381" & year==2008

replace regioncode="211382" if regioncode=="211382" & year==2008

replace regioncode="211402" if regioncode=="211402" & year==2008

replace regioncode="211403" if regioncode=="211403" & year==2008

replace regioncode="211404" if regioncode=="211404" & year==2008

replace regioncode="211421" if regioncode=="211421" & year==2008

replace regioncode="211422" if regioncode=="211422" & year==2008

replace regioncode="211481" if regioncode=="211481" & year==2008

replace regioncode="220102" if regioncode=="220102" & year==2008

replace regioncode="220103" if regioncode=="220103" & year==2008

replace regioncode="220104" if regioncode=="220104" & year==2008

replace regioncode="220105" if regioncode=="220105" & year==2008

replace regioncode="220106" if regioncode=="220106" & year==2008

replace regioncode="220112" if regioncode=="220112" & year==2008

replace regioncode="220122" if regioncode=="220122" & year==2008

replace regioncode="220181" if regioncode=="220181" & year==2008

replace regioncode="220182" if regioncode=="220182" & year==2008

replace regioncode="220183" if regioncode=="220183" & year==2008

replace regioncode="220202" if regioncode=="220202" & year==2008

replace regioncode="220203" if regioncode=="220203" & year==2008

replace regioncode="220204" if regioncode=="220204" & year==2008

replace regioncode="220211" if regioncode=="220211" & year==2008

replace regioncode="220221" if regioncode=="220221" & year==2008

replace regioncode="220281" if regioncode=="220281" & year==2008

replace regioncode="220282" if regioncode=="220282" & year==2008

replace regioncode="220283" if regioncode=="220283" & year==2008

replace regioncode="220284" if regioncode=="220284" & year==2008

replace regioncode="220302" if regioncode=="220302" & year==2008

replace regioncode="220303" if regioncode=="220303" & year==2008

replace regioncode="220322" if regioncode=="220322" & year==2008

replace regioncode="220323" if regioncode=="220323" & year==2008

replace regioncode="220381" if regioncode=="220381" & year==2008

replace regioncode="220382" if regioncode=="220382" & year==2008

replace regioncode="220402" if regioncode=="220402" & year==2008

replace regioncode="220403" if regioncode=="220403" & year==2008

replace regioncode="220421" if regioncode=="220421" & year==2008

replace regioncode="220422" if regioncode=="220422" & year==2008

replace regioncode="220502" if regioncode=="220502" & year==2008

replace regioncode="220503" if regioncode=="220503" & year==2008

replace regioncode="220521" if regioncode=="220521" & year==2008

replace regioncode="220523" if regioncode=="220523" & year==2008

replace regioncode="220524" if regioncode=="220524" & year==2008

replace regioncode="220581" if regioncode=="220581" & year==2008

replace regioncode="220582" if regioncode=="220582" & year==2008

replace regioncode="220602" if regioncode=="220602" & year==2008

replace regioncode="220621" if regioncode=="220621" & year==2008

replace regioncode="220622" if regioncode=="220622" & year==2008

replace regioncode="220623" if regioncode=="220623" & year==2008

replace regioncode="220625" if regioncode=="220625" & year==2008

replace regioncode="220681" if regioncode=="220681" & year==2008

replace regioncode="220702" if regioncode=="220702" & year==2008

replace regioncode="220721" if regioncode=="220721" & year==2008

replace regioncode="220722" if regioncode=="220722" & year==2008

replace regioncode="220723" if regioncode=="220723" & year==2008

replace regioncode="220724" if regioncode=="220724" & year==2008

replace regioncode="220802" if regioncode=="220802" & year==2008

replace regioncode="220821" if regioncode=="220821" & year==2008

replace regioncode="220822" if regioncode=="220822" & year==2008

replace regioncode="220881" if regioncode=="220881" & year==2008

replace regioncode="220882" if regioncode=="220882" & year==2008

replace regioncode="222401" if regioncode=="222401" & year==2008

replace regioncode="222402" if regioncode=="222402" & year==2008

replace regioncode="222403" if regioncode=="222403" & year==2008

replace regioncode="222404" if regioncode=="222404" & year==2008

replace regioncode="222405" if regioncode=="222405" & year==2008

replace regioncode="222406" if regioncode=="222406" & year==2008

replace regioncode="222424" if regioncode=="222424" & year==2008

replace regioncode="222426" if regioncode=="222426" & year==2008

replace regioncode="230102" if regioncode=="230102" & year==2008

replace regioncode="230103" if regioncode=="230103" & year==2008

replace regioncode="230104" if regioncode=="230104" & year==2008

replace regioncode="230105" if regioncode=="230105" & year==2008

replace regioncode="230107" if regioncode=="230110" & year==2008

replace regioncode="230108" if regioncode=="230108" & year==2008

replace regioncode="230121" if regioncode=="230111" & year==2008

replace regioncode="230123" if regioncode=="230123" & year==2008

replace regioncode="230124" if regioncode=="230124" & year==2008

replace regioncode="230125" if regioncode=="230125" & year==2008

replace regioncode="230126" if regioncode=="230126" & year==2008

replace regioncode="230127" if regioncode=="230127" & year==2008

replace regioncode="230128" if regioncode=="230128" & year==2008

replace regioncode="230129" if regioncode=="230129" & year==2008

replace regioncode="230181" if regioncode=="230112" & year==2008

replace regioncode="230182" if regioncode=="230182" & year==2008

replace regioncode="230183" if regioncode=="230183" & year==2008

replace regioncode="230184" if regioncode=="230184" & year==2008

replace regioncode="230202" if regioncode=="230202" & year==2008

replace regioncode="230203" if regioncode=="230203" & year==2008

replace regioncode="230204" if regioncode=="230204" & year==2008

replace regioncode="230205" if regioncode=="230205" & year==2008

replace regioncode="230206" if regioncode=="230206" & year==2008

replace regioncode="230207" if regioncode=="230207" & year==2008

replace regioncode="230208" if regioncode=="230208" & year==2008

replace regioncode="230221" if regioncode=="230221" & year==2008

replace regioncode="230223" if regioncode=="230223" & year==2008

replace regioncode="230224" if regioncode=="230224" & year==2008

replace regioncode="230225" if regioncode=="230225" & year==2008

replace regioncode="230227" if regioncode=="230227" & year==2008

replace regioncode="230229" if regioncode=="230229" & year==2008

replace regioncode="230230" if regioncode=="230230" & year==2008

replace regioncode="230231" if regioncode=="230231" & year==2008

replace regioncode="230281" if regioncode=="230281" & year==2008

replace regioncode="230302" if regioncode=="230302" & year==2008

replace regioncode="230303" if regioncode=="230303" & year==2008

replace regioncode="230304" if regioncode=="230304" & year==2008

replace regioncode="230305" if regioncode=="230305" & year==2008

replace regioncode="230306" if regioncode=="230306" & year==2008

replace regioncode="230307" if regioncode=="230307" & year==2008

replace regioncode="230321" if regioncode=="230321" & year==2008

replace regioncode="230381" if regioncode=="230381" & year==2008

replace regioncode="230382" if regioncode=="230382" & year==2008

replace regioncode="230402" if regioncode=="230402" & year==2008

replace regioncode="230403" if regioncode=="230403" & year==2008

replace regioncode="230404" if regioncode=="230404" & year==2008

replace regioncode="230405" if regioncode=="230405" & year==2008

replace regioncode="230406" if regioncode=="230406" & year==2008

replace regioncode="230407" if regioncode=="230407" & year==2008

replace regioncode="230421" if regioncode=="230421" & year==2008

replace regioncode="230422" if regioncode=="230422" & year==2008

replace regioncode="230502" if regioncode=="230502" & year==2008

replace regioncode="230503" if regioncode=="230503" & year==2008

replace regioncode="230505" if regioncode=="230505" & year==2008

replace regioncode="230506" if regioncode=="230506" & year==2008

replace regioncode="230521" if regioncode=="230521" & year==2008

replace regioncode="230522" if regioncode=="230522" & year==2008

replace regioncode="230523" if regioncode=="230523" & year==2008

replace regioncode="230524" if regioncode=="230524" & year==2008

replace regioncode="230602" if regioncode=="230602" & year==2008

replace regioncode="230603" if regioncode=="230603" & year==2008

replace regioncode="230604" if regioncode=="230604" & year==2008

replace regioncode="230605" if regioncode=="230605" & year==2008

replace regioncode="230606" if regioncode=="230606" & year==2008

replace regioncode="230621" if regioncode=="230621" & year==2008

replace regioncode="230622" if regioncode=="230622" & year==2008

replace regioncode="230623" if regioncode=="230623" & year==2008

replace regioncode="230624" if regioncode=="230624" & year==2008

replace regioncode="230702" if regioncode=="230702" & year==2008

replace regioncode="230703" if regioncode=="230703" & year==2008

replace regioncode="230704" if regioncode=="230704" & year==2008

replace regioncode="230705" if regioncode=="230705" & year==2008

replace regioncode="230706" if regioncode=="230706" & year==2008

replace regioncode="230707" if regioncode=="230707" & year==2008

replace regioncode="230708" if regioncode=="230708" & year==2008

replace regioncode="230709" if regioncode=="230709" & year==2008

replace regioncode="230710" if regioncode=="230710" & year==2008

replace regioncode="230711" if regioncode=="230711" & year==2008

replace regioncode="230712" if regioncode=="230712" & year==2008

replace regioncode="230713" if regioncode=="230713" & year==2008

replace regioncode="230714" if regioncode=="230714" & year==2008

replace regioncode="230715" if regioncode=="230715" & year==2008

replace regioncode="230716" if regioncode=="230716" & year==2008

replace regioncode="230722" if regioncode=="230722" & year==2008

replace regioncode="230781" if regioncode=="230781" & year==2008

replace regioncode="230802" if regioncode=="230802" & year==2008

replace regioncode="230803" if regioncode=="230803" & year==2008

replace regioncode="230804" if regioncode=="230804" & year==2008

replace regioncode="230805" if regioncode=="230805" & year==2008

replace regioncode="230811" if regioncode=="230811" & year==2008

replace regioncode="230822" if regioncode=="230822" & year==2008

replace regioncode="230826" if regioncode=="230826" & year==2008

replace regioncode="230828" if regioncode=="230828" & year==2008

replace regioncode="230833" if regioncode=="230833" & year==2008

replace regioncode="230881" if regioncode=="230881" & year==2008

replace regioncode="230882" if regioncode=="230882" & year==2008

replace regioncode="230902" if regioncode=="230902" & year==2008

replace regioncode="230903" if regioncode=="230903" & year==2008

replace regioncode="230904" if regioncode=="230904" & year==2008

replace regioncode="230921" if regioncode=="230921" & year==2008

replace regioncode="231002" if regioncode=="231002" & year==2008

replace regioncode="231003" if regioncode=="231003" & year==2008

replace regioncode="231004" if regioncode=="231004" & year==2008

replace regioncode="231005" if regioncode=="231005" & year==2008

replace regioncode="231024" if regioncode=="231024" & year==2008

replace regioncode="231025" if regioncode=="231025" & year==2008

replace regioncode="231081" if regioncode=="231081" & year==2008

replace regioncode="231083" if regioncode=="231083" & year==2008

replace regioncode="231084" if regioncode=="231084" & year==2008

replace regioncode="231085" if regioncode=="231085" & year==2008

replace regioncode="231102" if regioncode=="231102" & year==2008

replace regioncode="231121" if regioncode=="231121" & year==2008

replace regioncode="231123" if regioncode=="231123" & year==2008

replace regioncode="231124" if regioncode=="231124" & year==2008

replace regioncode="231181" if regioncode=="231181" & year==2008

replace regioncode="231182" if regioncode=="231182" & year==2008

replace regioncode="231202" if regioncode=="231202" & year==2008

replace regioncode="231221" if regioncode=="231221" & year==2008

replace regioncode="231222" if regioncode=="231222" & year==2008

replace regioncode="231223" if regioncode=="231223" & year==2008

replace regioncode="231224" if regioncode=="231224" & year==2008

replace regioncode="231225" if regioncode=="231225" & year==2008

replace regioncode="231226" if regioncode=="231226" & year==2008

replace regioncode="231281" if regioncode=="231281" & year==2008

replace regioncode="231282" if regioncode=="231282" & year==2008

replace regioncode="231283" if regioncode=="231283" & year==2008

replace regioncode="232701" if regioncode=="232701" & year==2008

replace regioncode="232702" if regioncode=="232702" & year==2008

replace regioncode="232703" if regioncode=="232703" & year==2008

replace regioncode="232704" if regioncode=="232704" & year==2008

replace regioncode="232721" if regioncode=="232721" & year==2008

replace regioncode="232722" if regioncode=="232722" & year==2008

replace regioncode="232723" if regioncode=="232723" & year==2008

replace regioncode="310101" if regioncode=="310101" & year==2008

replace regioncode="310103" if regioncode=="310103" & year==2008

replace regioncode="310104" if regioncode=="310104" & year==2008

replace regioncode="310105" if regioncode=="310105" & year==2008

replace regioncode="310106" if regioncode=="310106" & year==2008

replace regioncode="310107" if regioncode=="310107" & year==2008

replace regioncode="310108" if regioncode=="310108" & year==2008

replace regioncode="310109" if regioncode=="310109" & year==2008

replace regioncode="310110" if regioncode=="310110" & year==2008

replace regioncode="310112" if regioncode=="310112" & year==2008

replace regioncode="310113" if regioncode=="310113" & year==2008

replace regioncode="310114" if regioncode=="310114" & year==2008

replace regioncode="310115" if regioncode=="310115" & year==2008

replace regioncode="310116" if regioncode=="310116" & year==2008

replace regioncode="310117" if regioncode=="310117" & year==2008

replace regioncode="310118" if regioncode=="310118" & year==2008

replace regioncode="310119" if regioncode=="310119" & year==2008

replace regioncode="310120" if regioncode=="310120" & year==2008

replace regioncode="310230" if regioncode=="310230" & year==2008

replace regioncode="320102" if regioncode=="320102" & year==2008

replace regioncode="320103" if regioncode=="320103" & year==2008

replace regioncode="320104" if regioncode=="320104" & year==2008

replace regioncode="320105" if regioncode=="320105" & year==2008

replace regioncode="320106" if regioncode=="320106" & year==2008

replace regioncode="320107" if regioncode=="320107" & year==2008

replace regioncode="320111" if regioncode=="320111" & year==2008

replace regioncode="320113" if regioncode=="320113" & year==2008

replace regioncode="320114" if regioncode=="320114" & year==2008

replace regioncode="320115" if regioncode=="320115" & year==2008

replace regioncode="320116" if regioncode=="320116" & year==2008

replace regioncode="320124" if regioncode=="320124" & year==2008

replace regioncode="320125" if regioncode=="320125" & year==2008

replace regioncode="320202" if regioncode=="320202" & year==2008

replace regioncode="320203" if regioncode=="320203" & year==2008

replace regioncode="320204" if regioncode=="320204" & year==2008

replace regioncode="320205" if regioncode=="320205" & year==2008

replace regioncode="320211" if regioncode=="320211" & year==2008

replace regioncode="320281" if regioncode=="320281" & year==2008

replace regioncode="320282" if regioncode=="320282" & year==2008

replace regioncode="320302" if regioncode=="320302" & year==2008

replace regioncode="320303" if regioncode=="320303" & year==2008

replace regioncode="320304" if regioncode=="320304" & year==2008

replace regioncode="320305" if regioncode=="320305" & year==2008

replace regioncode="320311" if regioncode=="320311" & year==2008

replace regioncode="320321" if regioncode=="320321" & year==2008

replace regioncode="320322" if regioncode=="320322" & year==2008

replace regioncode="320323" if regioncode=="320323" & year==2008

replace regioncode="320324" if regioncode=="320324" & year==2008

replace regioncode="320381" if regioncode=="320381" & year==2008

replace regioncode="320382" if regioncode=="320382" & year==2008

replace regioncode="320402" if regioncode=="320402" & year==2008

replace regioncode="320404" if regioncode=="320404" & year==2008

replace regioncode="320405" if regioncode=="320405" & year==2008

replace regioncode="320411" if regioncode=="320411" & year==2008

replace regioncode="320412" if regioncode=="320412" & year==2008

replace regioncode="320481" if regioncode=="320481" & year==2008

replace regioncode="320482" if regioncode=="320482" & year==2008

replace regioncode="320502" if regioncode=="320502" & year==2008

replace regioncode="320503" if regioncode=="320503" & year==2008

replace regioncode="320504" if regioncode=="320504" & year==2008

replace regioncode="320505" if regioncode=="320505" & year==2008

replace regioncode="320581" if regioncode=="320581" & year==2008

replace regioncode="320582" if regioncode=="320582" & year==2008

replace regioncode="320583" if regioncode=="320583" & year==2008

replace regioncode="320584" if regioncode=="320584" & year==2008

replace regioncode="320585" if regioncode=="320585" & year==2008

replace regioncode="320586" if regioncode=="320586" & year==2008

replace regioncode="320602" if regioncode=="320602" & year==2008

replace regioncode="320611" if regioncode=="320611" & year==2008

replace regioncode="320621" if regioncode=="320621" & year==2008

replace regioncode="320623" if regioncode=="320623" & year==2008

replace regioncode="320681" if regioncode=="320681" & year==2008

replace regioncode="320682" if regioncode=="320682" & year==2008

replace regioncode="320683" if regioncode=="320683" & year==2008

replace regioncode="320684" if regioncode=="320684" & year==2008

replace regioncode="320703" if regioncode=="320703" & year==2008

replace regioncode="320705" if regioncode=="320705" & year==2008

replace regioncode="320706" if regioncode=="320706" & year==2008

replace regioncode="320721" if regioncode=="320721" & year==2008

replace regioncode="320722" if regioncode=="320722" & year==2008

replace regioncode="320723" if regioncode=="320723" & year==2008

replace regioncode="320724" if regioncode=="320724" & year==2008

replace regioncode="320802" if regioncode=="320802" & year==2008

replace regioncode="320803" if regioncode=="320803" & year==2008

replace regioncode="320804" if regioncode=="320804" & year==2008

replace regioncode="320811" if regioncode=="320811" & year==2008

replace regioncode="320826" if regioncode=="320826" & year==2008

replace regioncode="320829" if regioncode=="320829" & year==2008

replace regioncode="320830" if regioncode=="320830" & year==2008

replace regioncode="320831" if regioncode=="320831" & year==2008

replace regioncode="320902" if regioncode=="320902" & year==2008

replace regioncode="320903" if regioncode=="320903" & year==2008

replace regioncode="320921" if regioncode=="320921" & year==2008

replace regioncode="320922" if regioncode=="320922" & year==2008

replace regioncode="320923" if regioncode=="320923" & year==2008

replace regioncode="320924" if regioncode=="320924" & year==2008

replace regioncode="320925" if regioncode=="320925" & year==2008

replace regioncode="320981" if regioncode=="320981" & year==2008

replace regioncode="320982" if regioncode=="320982" & year==2008

replace regioncode="321002" if regioncode=="321002" & year==2008

replace regioncode="321003" if regioncode=="321003" & year==2008

replace regioncode="321011" if regioncode=="321011" & year==2008

replace regioncode="321023" if regioncode=="321023" & year==2008

replace regioncode="321081" if regioncode=="321081" & year==2008

replace regioncode="321084" if regioncode=="321084" & year==2008

replace regioncode="321088" if regioncode=="321088" & year==2008

replace regioncode="321102" if regioncode=="321102" & year==2008

replace regioncode="321111" if regioncode=="321111" & year==2008

replace regioncode="321112" if regioncode=="321112" & year==2008

replace regioncode="321181" if regioncode=="321181" & year==2008

replace regioncode="321182" if regioncode=="321182" & year==2008

replace regioncode="321183" if regioncode=="321183" & year==2008

replace regioncode="321202" if regioncode=="321202" & year==2008

replace regioncode="321203" if regioncode=="321203" & year==2008

replace regioncode="321281" if regioncode=="321281" & year==2008

replace regioncode="321282" if regioncode=="321282" & year==2008

replace regioncode="321283" if regioncode=="321283" & year==2008

replace regioncode="321284" if regioncode=="321284" & year==2008

replace regioncode="321302" if regioncode=="321302" & year==2008

replace regioncode="321321" if regioncode=="321311" & year==2008

replace regioncode="321322" if regioncode=="321322" & year==2008

replace regioncode="321323" if regioncode=="321323" & year==2008

replace regioncode="321324" if regioncode=="321324" & year==2008

replace regioncode="330102" if regioncode=="330102" & year==2008

replace regioncode="330103" if regioncode=="330103" & year==2008

replace regioncode="330104" if regioncode=="330104" & year==2008

replace regioncode="330105" if regioncode=="330105" & year==2008

replace regioncode="330106" if regioncode=="330106" & year==2008

replace regioncode="330108" if regioncode=="330108" & year==2008

replace regioncode="330109" if regioncode=="330109" & year==2008

replace regioncode="330110" if regioncode=="330110" & year==2008

replace regioncode="330122" if regioncode=="330122" & year==2008

replace regioncode="330127" if regioncode=="330127" & year==2008

replace regioncode="330182" if regioncode=="330182" & year==2008

replace regioncode="330183" if regioncode=="330183" & year==2008

replace regioncode="330185" if regioncode=="330185" & year==2008

replace regioncode="330203" if regioncode=="330203" & year==2008

replace regioncode="330204" if regioncode=="330204" & year==2008

replace regioncode="330205" if regioncode=="330205" & year==2008

replace regioncode="330206" if regioncode=="330206" & year==2008

replace regioncode="330211" if regioncode=="330211" & year==2008

replace regioncode="330212" if regioncode=="330212" & year==2008

replace regioncode="330225" if regioncode=="330225" & year==2008

replace regioncode="330226" if regioncode=="330226" & year==2008

replace regioncode="330281" if regioncode=="330281" & year==2008

replace regioncode="330282" if regioncode=="330282" & year==2008

replace regioncode="330283" if regioncode=="330283" & year==2008

replace regioncode="330302" if regioncode=="330302" & year==2008

replace regioncode="330303" if regioncode=="330303" & year==2008

replace regioncode="330304" if regioncode=="330304" & year==2008

replace regioncode="330322" if regioncode=="330322" & year==2008

replace regioncode="330324" if regioncode=="330324" & year==2008

replace regioncode="330326" if regioncode=="330326" & year==2008

replace regioncode="330327" if regioncode=="330327" & year==2008

replace regioncode="330328" if regioncode=="330328" & year==2008

replace regioncode="330329" if regioncode=="330329" & year==2008

replace regioncode="330381" if regioncode=="330381" & year==2008

replace regioncode="330382" if regioncode=="330382" & year==2008

replace regioncode="330402" if regioncode=="330402" & year==2008

replace regioncode="330411" if regioncode=="330411" & year==2008

replace regioncode="330421" if regioncode=="330421" & year==2008

replace regioncode="330424" if regioncode=="330424" & year==2008

replace regioncode="330481" if regioncode=="330481" & year==2008

replace regioncode="330482" if regioncode=="330482" & year==2008

replace regioncode="330483" if regioncode=="330483" & year==2008

replace regioncode="330501" if regioncode=="330501" & year==2008

replace regioncode="330521" if regioncode=="330521" & year==2008

replace regioncode="330522" if regioncode=="330522" & year==2008

replace regioncode="330523" if regioncode=="330523" & year==2008

replace regioncode="330602" if regioncode=="330602" & year==2008

replace regioncode="330621" if regioncode=="330621" & year==2008

replace regioncode="330624" if regioncode=="330624" & year==2008

replace regioncode="330681" if regioncode=="330681" & year==2008

replace regioncode="330682" if regioncode=="330682" & year==2008

replace regioncode="330683" if regioncode=="330683" & year==2008

replace regioncode="330702" if regioncode=="330702" & year==2008

replace regioncode="330703" if regioncode=="330703" & year==2008

replace regioncode="330723" if regioncode=="330723" & year==2008

replace regioncode="330726" if regioncode=="330726" & year==2008

replace regioncode="330727" if regioncode=="330727" & year==2008

replace regioncode="330781" if regioncode=="330781" & year==2008

replace regioncode="330782" if regioncode=="330782" & year==2008

replace regioncode="330783" if regioncode=="330783" & year==2008

replace regioncode="330784" if regioncode=="330784" & year==2008

replace regioncode="330802" if regioncode=="330802" & year==2008

replace regioncode="330803" if regioncode=="330803" & year==2008

replace regioncode="330822" if regioncode=="330822" & year==2008

replace regioncode="330824" if regioncode=="330824" & year==2008

replace regioncode="330825" if regioncode=="330825" & year==2008

replace regioncode="330881" if regioncode=="330881" & year==2008

replace regioncode="330902" if regioncode=="330902" & year==2008

replace regioncode="330903" if regioncode=="330903" & year==2008

replace regioncode="330921" if regioncode=="330921" & year==2008

replace regioncode="330922" if regioncode=="330922" & year==2008

replace regioncode="331002" if regioncode=="331002" & year==2008

replace regioncode="331003" if regioncode=="331003" & year==2008

replace regioncode="331004" if regioncode=="331004" & year==2008

replace regioncode="331021" if regioncode=="331021" & year==2008

replace regioncode="331022" if regioncode=="331022" & year==2008

replace regioncode="331023" if regioncode=="331023" & year==2008

replace regioncode="331024" if regioncode=="331024" & year==2008

replace regioncode="331081" if regioncode=="331081" & year==2008

replace regioncode="331082" if regioncode=="331082" & year==2008

replace regioncode="331102" if regioncode=="331102" & year==2008

replace regioncode="331121" if regioncode=="331121" & year==2008

replace regioncode="331122" if regioncode=="331122" & year==2008

replace regioncode="331123" if regioncode=="331123" & year==2008

replace regioncode="331124" if regioncode=="331124" & year==2008

replace regioncode="331125" if regioncode=="331125" & year==2008

replace regioncode="331126" if regioncode=="331126" & year==2008

replace regioncode="331127" if regioncode=="331127" & year==2008

replace regioncode="331181" if regioncode=="331181" & year==2008

replace regioncode="340102" if regioncode=="340102" & year==2008

replace regioncode="340103" if regioncode=="340103" & year==2008

replace regioncode="340104" if regioncode=="340104" & year==2008

replace regioncode="340111" if regioncode=="340111" & year==2008

replace regioncode="340121" if regioncode=="340121" & year==2008

replace regioncode="340122" if regioncode=="340122" & year==2008

replace regioncode="340123" if regioncode=="340123" & year==2008

replace regioncode="340202" if regioncode=="340202" & year==2008

replace regioncode="340203" if regioncode=="340203" & year==2008

replace regioncode="340204" if regioncode=="340204" & year==2008

replace regioncode="340207" if regioncode=="340207" & year==2008

replace regioncode="340221" if regioncode=="340221" & year==2008

replace regioncode="340222" if regioncode=="340222" & year==2008

replace regioncode="340223" if regioncode=="340223" & year==2008

replace regioncode="340302" if regioncode=="340302" & year==2008

replace regioncode="340303" if regioncode=="340303" & year==2008

replace regioncode="340304" if regioncode=="340304" & year==2008

replace regioncode="340311" if regioncode=="340311" & year==2008

replace regioncode="340321" if regioncode=="340321" & year==2008

replace regioncode="340322" if regioncode=="340322" & year==2008

replace regioncode="340323" if regioncode=="340323" & year==2008

replace regioncode="340402" if regioncode=="340402" & year==2008

replace regioncode="340403" if regioncode=="340403" & year==2008

replace regioncode="340404" if regioncode=="340404" & year==2008

replace regioncode="340405" if regioncode=="340405" & year==2008

replace regioncode="340406" if regioncode=="340406" & year==2008

replace regioncode="340421" if regioncode=="340421" & year==2008

replace regioncode="340502" if regioncode=="340502" & year==2008

replace regioncode="340503" if regioncode=="340503" & year==2008

replace regioncode="340504" if regioncode=="340504" & year==2008

replace regioncode="340521" if regioncode=="340521" & year==2008

replace regioncode="340602" if regioncode=="340602" & year==2008

replace regioncode="340603" if regioncode=="340603" & year==2008

replace regioncode="340604" if regioncode=="340604" & year==2008

replace regioncode="340621" if regioncode=="340621" & year==2008

replace regioncode="340702" if regioncode=="340702" & year==2008

replace regioncode="340703" if regioncode=="340703" & year==2008

replace regioncode="340711" if regioncode=="340711" & year==2008

replace regioncode="340721" if regioncode=="340721" & year==2008

replace regioncode="340802" if regioncode=="340802" & year==2008

replace regioncode="340803" if regioncode=="340803" & year==2008

replace regioncode="340811" if regioncode=="340811" & year==2008

replace regioncode="340822" if regioncode=="340822" & year==2008

replace regioncode="340823" if regioncode=="340823" & year==2008

replace regioncode="340824" if regioncode=="340824" & year==2008

replace regioncode="340825" if regioncode=="340825" & year==2008

replace regioncode="340826" if regioncode=="340826" & year==2008

replace regioncode="340827" if regioncode=="340827" & year==2008

replace regioncode="340828" if regioncode=="340828" & year==2008

replace regioncode="340881" if regioncode=="340881" & year==2008

replace regioncode="341002" if regioncode=="341002" & year==2008

replace regioncode="341003" if regioncode=="341003" & year==2008

replace regioncode="341004" if regioncode=="341004" & year==2008

replace regioncode="341021" if regioncode=="341021" & year==2008

replace regioncode="341022" if regioncode=="341022" & year==2008

replace regioncode="341023" if regioncode=="341023" & year==2008

replace regioncode="341024" if regioncode=="341024" & year==2008

replace regioncode="341102" if regioncode=="341102" & year==2008

replace regioncode="341103" if regioncode=="341103" & year==2008

replace regioncode="341122" if regioncode=="341122" & year==2008

replace regioncode="341124" if regioncode=="341124" & year==2008

replace regioncode="341125" if regioncode=="341125" & year==2008

replace regioncode="341126" if regioncode=="341126" & year==2008

replace regioncode="341181" if regioncode=="341181" & year==2008

replace regioncode="341182" if regioncode=="341182" & year==2008

replace regioncode="341202" if regioncode=="341202" & year==2008

replace regioncode="341203" if regioncode=="341203" & year==2008

replace regioncode="341204" if regioncode=="341204" & year==2008

replace regioncode="341221" if regioncode=="341221" & year==2008

replace regioncode="341222" if regioncode=="341222" & year==2008

replace regioncode="341621" if regioncode=="341621" & year==2008

replace regioncode="341622" if regioncode=="341622" & year==2008

replace regioncode="341225" if regioncode=="341225" & year==2008

replace regioncode="341226" if regioncode=="341226" & year==2008

replace regioncode="341623" if regioncode=="341623" & year==2008

replace regioncode="341282" if regioncode=="341282" & year==2008

replace regioncode="341302" if regioncode=="341302" & year==2008

replace regioncode="341321" if regioncode=="341321" & year==2008

replace regioncode="341322" if regioncode=="341322" & year==2008

replace regioncode="341323" if regioncode=="341323" & year==2008

replace regioncode="341324" if regioncode=="341324" & year==2008

replace regioncode="341402" if regioncode=="341402" & year==2008

replace regioncode="341421" if regioncode=="341421" & year==2008

replace regioncode="341422" if regioncode=="341422" & year==2008

replace regioncode="341423" if regioncode=="341423" & year==2008

replace regioncode="341424" if regioncode=="341424" & year==2008

replace regioncode="341521" if regioncode=="341521" & year==2008

replace regioncode="341522" if regioncode=="341522" & year==2008

replace regioncode="341523" if regioncode=="341523" & year==2008

replace regioncode="341524" if regioncode=="341524" & year==2008

replace regioncode="341525" if regioncode=="341525" & year==2008

replace regioncode="341802" if regioncode=="341802" & year==2008

replace regioncode="341881" if regioncode=="341881" & year==2008

replace regioncode="341821" if regioncode=="341821" & year==2008

replace regioncode="341822" if regioncode=="341822" & year==2008

replace regioncode="341823" if regioncode=="341823" & year==2008

replace regioncode="341825" if regioncode=="341825" & year==2008

replace regioncode="341824" if regioncode=="341824" & year==2008

replace regioncode="341702" if regioncode=="341702" & year==2008

replace regioncode="341721" if regioncode=="341721" & year==2008

replace regioncode="341722" if regioncode=="341722" & year==2008

replace regioncode="341723" if regioncode=="341723" & year==2008

replace regioncode="341502" if regioncode=="341502" & year==2008

replace regioncode="341602" if regioncode=="341602" & year==2008

replace regioncode="350102" if regioncode=="350102" & year==2008

replace regioncode="350103" if regioncode=="350103" & year==2008

replace regioncode="350104" if regioncode=="350104" & year==2008

replace regioncode="350105" if regioncode=="350105" & year==2008

replace regioncode="350111" if regioncode=="350111" & year==2008

replace regioncode="350121" if regioncode=="350121" & year==2008

replace regioncode="350122" if regioncode=="350122" & year==2008

replace regioncode="350123" if regioncode=="350123" & year==2008

replace regioncode="350124" if regioncode=="350124" & year==2008

replace regioncode="350125" if regioncode=="350125" & year==2008

replace regioncode="350128" if regioncode=="350128" & year==2008

replace regioncode="350181" if regioncode=="350181" & year==2008

replace regioncode="350182" if regioncode=="350182" & year==2008

replace regioncode="350203" if regioncode=="350203" & year==2008

replace regioncode="350205" if regioncode=="350205" & year==2008

replace regioncode="350206" if regioncode=="350206" & year==2008

replace regioncode="350211" if regioncode=="350211" & year==2008

replace regioncode="350212" if regioncode=="350212" & year==2008

replace regioncode="350213" if regioncode=="350213" & year==2008

replace regioncode="350302" if regioncode=="350302" & year==2008

replace regioncode="350303" if regioncode=="350303" & year==2008

replace regioncode="350321" if regioncode=="350321" & year==2008

replace regioncode="350322" if regioncode=="350322" & year==2008

replace regioncode="350402" if regioncode=="350402" & year==2008

replace regioncode="350403" if regioncode=="350403" & year==2008

replace regioncode="350421" if regioncode=="350421" & year==2008

replace regioncode="350423" if regioncode=="350423" & year==2008

replace regioncode="350424" if regioncode=="350424" & year==2008

replace regioncode="350425" if regioncode=="350425" & year==2008

replace regioncode="350426" if regioncode=="350426" & year==2008

replace regioncode="350427" if regioncode=="350427" & year==2008

replace regioncode="350428" if regioncode=="350428" & year==2008

replace regioncode="350429" if regioncode=="350429" & year==2008

replace regioncode="350430" if regioncode=="350430" & year==2008

replace regioncode="350481" if regioncode=="350481" & year==2008

replace regioncode="350502" if regioncode=="350502" & year==2008

replace regioncode="350503" if regioncode=="350503" & year==2008

replace regioncode="350504" if regioncode=="350504" & year==2008

replace regioncode="350505" if regioncode=="350505" & year==2008

replace regioncode="350521" if regioncode=="350521" & year==2008

replace regioncode="350524" if regioncode=="350524" & year==2008

replace regioncode="350525" if regioncode=="350525" & year==2008

replace regioncode="350526" if regioncode=="350526" & year==2008

replace regioncode="350581" if regioncode=="350581" & year==2008

replace regioncode="350582" if regioncode=="350582" & year==2008

replace regioncode="350583" if regioncode=="350583" & year==2008

replace regioncode="350602" if regioncode=="350602" & year==2008

replace regioncode="350603" if regioncode=="350603" & year==2008

replace regioncode="350622" if regioncode=="350622" & year==2008

replace regioncode="350623" if regioncode=="350623" & year==2008

replace regioncode="350624" if regioncode=="350624" & year==2008

replace regioncode="350625" if regioncode=="350625" & year==2008

replace regioncode="350626" if regioncode=="350626" & year==2008

replace regioncode="350627" if regioncode=="350627" & year==2008

replace regioncode="350628" if regioncode=="350628" & year==2008

replace regioncode="350629" if regioncode=="350629" & year==2008

replace regioncode="350681" if regioncode=="350681" & year==2008

replace regioncode="350702" if regioncode=="350702" & year==2008

replace regioncode="350721" if regioncode=="350721" & year==2008

replace regioncode="350722" if regioncode=="350722" & year==2008

replace regioncode="350723" if regioncode=="350723" & year==2008

replace regioncode="350724" if regioncode=="350724" & year==2008

replace regioncode="350725" if regioncode=="350725" & year==2008

replace regioncode="350781" if regioncode=="350781" & year==2008

replace regioncode="350782" if regioncode=="350782" & year==2008

replace regioncode="350783" if regioncode=="350783" & year==2008

replace regioncode="350784" if regioncode=="350784" & year==2008

replace regioncode="350802" if regioncode=="350802" & year==2008

replace regioncode="350821" if regioncode=="350821" & year==2008

replace regioncode="350822" if regioncode=="350822" & year==2008

replace regioncode="350823" if regioncode=="350823" & year==2008

replace regioncode="350824" if regioncode=="350824" & year==2008

replace regioncode="350825" if regioncode=="350825" & year==2008

replace regioncode="350881" if regioncode=="350881" & year==2008

replace regioncode="350902" if regioncode=="350902" & year==2008

replace regioncode="350981" if regioncode=="350981" & year==2008

replace regioncode="350982" if regioncode=="350982" & year==2008

replace regioncode="350921" if regioncode=="350921" & year==2008

replace regioncode="350922" if regioncode=="350922" & year==2008

replace regioncode="350923" if regioncode=="350923" & year==2008

replace regioncode="350924" if regioncode=="350924" & year==2008

replace regioncode="350925" if regioncode=="350925" & year==2008

replace regioncode="350926" if regioncode=="350926" & year==2008

replace regioncode="360102" if regioncode=="360102" & year==2008

replace regioncode="360103" if regioncode=="360103" & year==2008

replace regioncode="360104" if regioncode=="360104" & year==2008

replace regioncode="360105" if regioncode=="360105" & year==2008

replace regioncode="360111" if regioncode=="360111" & year==2008

replace regioncode="360121" if regioncode=="360121" & year==2008

replace regioncode="360122" if regioncode=="360122" & year==2008

replace regioncode="360123" if regioncode=="360123" & year==2008

replace regioncode="360124" if regioncode=="360124" & year==2008

replace regioncode="360202" if regioncode=="360202" & year==2008

replace regioncode="360203" if regioncode=="360203" & year==2008

replace regioncode="360222" if regioncode=="360222" & year==2008

replace regioncode="360281" if regioncode=="360281" & year==2008

replace regioncode="360302" if regioncode=="360302" & year==2008

replace regioncode="360313" if regioncode=="360313" & year==2008

replace regioncode="360321" if regioncode=="360321" & year==2008

replace regioncode="360322" if regioncode=="360322" & year==2008

replace regioncode="360323" if regioncode=="360323" & year==2008

replace regioncode="360402" if regioncode=="360402" & year==2008

replace regioncode="360403" if regioncode=="360403" & year==2008

replace regioncode="360421" if regioncode=="360421" & year==2008

replace regioncode="360423" if regioncode=="360423" & year==2008

replace regioncode="360424" if regioncode=="360424" & year==2008

replace regioncode="360425" if regioncode=="360425" & year==2008

replace regioncode="360426" if regioncode=="360426" & year==2008

replace regioncode="360427" if regioncode=="360427" & year==2008

replace regioncode="360428" if regioncode=="360428" & year==2008

replace regioncode="360429" if regioncode=="360429" & year==2008

replace regioncode="360430" if regioncode=="360430" & year==2008

replace regioncode="360481" if regioncode=="360481" & year==2008

replace regioncode="360502" if regioncode=="360502" & year==2008

replace regioncode="360521" if regioncode=="360521" & year==2008

replace regioncode="360602" if regioncode=="360602" & year==2008

replace regioncode="360622" if regioncode=="360622" & year==2008

replace regioncode="360681" if regioncode=="360681" & year==2008

replace regioncode="360702" if regioncode=="360702" & year==2008

replace regioncode="360721" if regioncode=="360721" & year==2008

replace regioncode="360722" if regioncode=="360722" & year==2008

replace regioncode="360723" if regioncode=="360723" & year==2008

replace regioncode="360724" if regioncode=="360724" & year==2008

replace regioncode="360725" if regioncode=="360725" & year==2008

replace regioncode="360726" if regioncode=="360726" & year==2008

replace regioncode="360727" if regioncode=="360727" & year==2008

replace regioncode="360728" if regioncode=="360728" & year==2008

replace regioncode="360729" if regioncode=="360729" & year==2008

replace regioncode="360730" if regioncode=="360730" & year==2008

replace regioncode="360731" if regioncode=="360731" & year==2008

replace regioncode="360732" if regioncode=="360732" & year==2008

replace regioncode="360733" if regioncode=="360733" & year==2008

replace regioncode="360734" if regioncode=="360734" & year==2008

replace regioncode="360735" if regioncode=="360735" & year==2008

replace regioncode="360781" if regioncode=="360781" & year==2008

replace regioncode="360782" if regioncode=="360782" & year==2008

replace regioncode="360802" if regioncode=="360802" & year==2008

replace regioncode="360821" if regioncode=="360821" & year==2008

replace regioncode="360822" if regioncode=="360822" & year==2008

replace regioncode="360823" if regioncode=="360823" & year==2008

replace regioncode="360824" if regioncode=="360824" & year==2008

replace regioncode="360825" if regioncode=="360825" & year==2008

replace regioncode="360826" if regioncode=="360826" & year==2008

replace regioncode="360827" if regioncode=="360827" & year==2008

replace regioncode="360828" if regioncode=="360828" & year==2008

replace regioncode="360829" if regioncode=="360829" & year==2008

replace regioncode="360830" if regioncode=="360830" & year==2008

replace regioncode="360881" if regioncode=="360881" & year==2008

replace regioncode="360902" if regioncode=="360902" & year==2008

replace regioncode="360921" if regioncode=="360921" & year==2008

replace regioncode="360922" if regioncode=="360922" & year==2008

replace regioncode="360923" if regioncode=="360923" & year==2008

replace regioncode="360924" if regioncode=="360924" & year==2008

replace regioncode="360925" if regioncode=="360925" & year==2008

replace regioncode="360926" if regioncode=="360926" & year==2008

replace regioncode="360981" if regioncode=="360981" & year==2008

replace regioncode="360982" if regioncode=="360982" & year==2008

replace regioncode="360983" if regioncode=="360983" & year==2008

replace regioncode="361002" if regioncode=="361002" & year==2008

replace regioncode="361021" if regioncode=="361021" & year==2008

replace regioncode="361022" if regioncode=="361022" & year==2008

replace regioncode="361023" if regioncode=="361023" & year==2008

replace regioncode="361024" if regioncode=="361024" & year==2008

replace regioncode="361025" if regioncode=="361025" & year==2008

replace regioncode="361026" if regioncode=="361026" & year==2008

replace regioncode="361027" if regioncode=="361027" & year==2008

replace regioncode="361028" if regioncode=="361028" & year==2008

replace regioncode="361029" if regioncode=="361029" & year==2008

replace regioncode="361030" if regioncode=="361030" & year==2008

replace regioncode="361102" if regioncode=="361102" & year==2008

replace regioncode="361121" if regioncode=="361121" & year==2008

replace regioncode="361122" if regioncode=="361122" & year==2008

replace regioncode="361123" if regioncode=="361123" & year==2008

replace regioncode="361124" if regioncode=="361124" & year==2008

replace regioncode="361125" if regioncode=="361125" & year==2008

replace regioncode="361126" if regioncode=="361126" & year==2008

replace regioncode="361127" if regioncode=="361127" & year==2008

replace regioncode="361128" if regioncode=="361128" & year==2008

replace regioncode="361129" if regioncode=="361129" & year==2008

replace regioncode="361130" if regioncode=="361130" & year==2008

replace regioncode="361181" if regioncode=="361181" & year==2008

replace regioncode="370102" if regioncode=="370102" & year==2008

replace regioncode="370103" if regioncode=="370103" & year==2008

replace regioncode="370104" if regioncode=="370104" & year==2008

replace regioncode="370105" if regioncode=="370105" & year==2008

replace regioncode="370112" if regioncode=="370112" & year==2008

replace regioncode="370113" if regioncode=="370113" & year==2008

replace regioncode="370124" if regioncode=="370124" & year==2008

replace regioncode="370125" if regioncode=="370125" & year==2008

replace regioncode="370126" if regioncode=="370126" & year==2008

replace regioncode="370181" if regioncode=="370181" & year==2008

replace regioncode="370202" if regioncode=="370202" & year==2008

replace regioncode="370203" if regioncode=="370203" & year==2008

replace regioncode="370205" if regioncode=="370205" & year==2008

replace regioncode="370211" if regioncode=="370211" & year==2008

replace regioncode="370212" if regioncode=="370212" & year==2008

replace regioncode="370213" if regioncode=="370213" & year==2008

replace regioncode="370214" if regioncode=="370214" & year==2008

replace regioncode="370281" if regioncode=="370281" & year==2008

replace regioncode="370282" if regioncode=="370282" & year==2008

replace regioncode="370283" if regioncode=="370283" & year==2008

replace regioncode="370284" if regioncode=="370284" & year==2008

replace regioncode="370285" if regioncode=="370285" & year==2008

replace regioncode="370302" if regioncode=="370302" & year==2008

replace regioncode="370303" if regioncode=="370303" & year==2008

replace regioncode="370304" if regioncode=="370304" & year==2008

replace regioncode="370305" if regioncode=="370305" & year==2008

replace regioncode="370306" if regioncode=="370306" & year==2008

replace regioncode="370321" if regioncode=="370321" & year==2008

replace regioncode="370322" if regioncode=="370322" & year==2008

replace regioncode="370323" if regioncode=="370323" & year==2008

replace regioncode="370402" if regioncode=="370402" & year==2008

replace regioncode="370403" if regioncode=="370403" & year==2008

replace regioncode="370404" if regioncode=="370404" & year==2008

replace regioncode="370405" if regioncode=="370405" & year==2008

replace regioncode="370406" if regioncode=="370406" & year==2008

replace regioncode="370481" if regioncode=="370481" & year==2008

replace regioncode="370502" if regioncode=="370502" & year==2008

replace regioncode="370503" if regioncode=="370503" & year==2008

replace regioncode="370521" if regioncode=="370521" & year==2008

replace regioncode="370522" if regioncode=="370522" & year==2008

replace regioncode="370523" if regioncode=="370523" & year==2008

replace regioncode="370602" if regioncode=="370602" & year==2008

replace regioncode="370611" if regioncode=="370611" & year==2008

replace regioncode="370612" if regioncode=="370612" & year==2008

replace regioncode="370613" if regioncode=="370613" & year==2008

replace regioncode="370634" if regioncode=="370634" & year==2008

replace regioncode="370681" if regioncode=="370681" & year==2008

replace regioncode="370682" if regioncode=="370682" & year==2008

replace regioncode="370683" if regioncode=="370683" & year==2008

replace regioncode="370684" if regioncode=="370684" & year==2008

replace regioncode="370685" if regioncode=="370685" & year==2008

replace regioncode="370686" if regioncode=="370686" & year==2008

replace regioncode="370687" if regioncode=="370687" & year==2008

replace regioncode="370702" if regioncode=="370702" & year==2008

replace regioncode="370703" if regioncode=="370703" & year==2008

replace regioncode="370704" if regioncode=="370704" & year==2008

replace regioncode="370705" if regioncode=="370705" & year==2008

replace regioncode="370724" if regioncode=="370724" & year==2008

replace regioncode="370725" if regioncode=="370725" & year==2008

replace regioncode="370781" if regioncode=="370781" & year==2008

replace regioncode="370782" if regioncode=="370782" & year==2008

replace regioncode="370783" if regioncode=="370783" & year==2008

replace regioncode="370784" if regioncode=="370784" & year==2008

replace regioncode="370785" if regioncode=="370785" & year==2008

replace regioncode="370786" if regioncode=="370786" & year==2008

replace regioncode="370802" if regioncode=="370802" & year==2008

replace regioncode="370811" if regioncode=="370811" & year==2008

replace regioncode="370826" if regioncode=="370826" & year==2008

replace regioncode="370827" if regioncode=="370827" & year==2008

replace regioncode="370828" if regioncode=="370828" & year==2008

replace regioncode="370829" if regioncode=="370829" & year==2008

replace regioncode="370830" if regioncode=="370830" & year==2008

replace regioncode="370831" if regioncode=="370831" & year==2008

replace regioncode="370832" if regioncode=="370832" & year==2008

replace regioncode="370881" if regioncode=="370881" & year==2008

replace regioncode="370882" if regioncode=="370882" & year==2008

replace regioncode="370883" if regioncode=="370883" & year==2008

replace regioncode="370902" if regioncode=="370902" & year==2008

replace regioncode="370903" if regioncode=="370911" & year==2008

replace regioncode="370921" if regioncode=="370921" & year==2008

replace regioncode="370923" if regioncode=="370923" & year==2008

replace regioncode="370982" if regioncode=="370982" & year==2008

replace regioncode="370983" if regioncode=="370983" & year==2008

replace regioncode="371002" if regioncode=="371002" & year==2008

replace regioncode="371081" if regioncode=="371081" & year==2008

replace regioncode="371082" if regioncode=="371082" & year==2008

replace regioncode="371083" if regioncode=="371083" & year==2008

replace regioncode="371102" if regioncode=="371102" & year==2008

replace regioncode="371121" if regioncode=="371121" & year==2008

replace regioncode="371122" if regioncode=="371122" & year==2008

replace regioncode="371202" if regioncode=="371202" & year==2008

replace regioncode="371203" if regioncode=="371203" & year==2008

replace regioncode="371302" if regioncode=="371302" & year==2008

replace regioncode="371311" if regioncode=="371311" & year==2008

replace regioncode="371312" if regioncode=="371312" & year==2008

replace regioncode="371321" if regioncode=="371321" & year==2008

replace regioncode="371322" if regioncode=="371322" & year==2008

replace regioncode="371323" if regioncode=="371323" & year==2008

replace regioncode="371324" if regioncode=="371324" & year==2008

replace regioncode="371325" if regioncode=="371325" & year==2008

replace regioncode="371326" if regioncode=="371326" & year==2008

replace regioncode="371327" if regioncode=="371327" & year==2008

replace regioncode="371328" if regioncode=="371328" & year==2008

replace regioncode="371329" if regioncode=="371329" & year==2008

replace regioncode="371402" if regioncode=="371402" & year==2008

replace regioncode="371421" if regioncode=="371421" & year==2008

replace regioncode="371422" if regioncode=="371422" & year==2008

replace regioncode="371423" if regioncode=="371423" & year==2008

replace regioncode="371424" if regioncode=="371424" & year==2008

replace regioncode="371425" if regioncode=="371425" & year==2008

replace regioncode="371426" if regioncode=="371426" & year==2008

replace regioncode="371427" if regioncode=="371427" & year==2008

replace regioncode="371428" if regioncode=="371428" & year==2008

replace regioncode="371481" if regioncode=="371481" & year==2008

replace regioncode="371482" if regioncode=="371482" & year==2008

replace regioncode="371502" if regioncode=="371502" & year==2008

replace regioncode="371521" if regioncode=="371521" & year==2008

replace regioncode="371522" if regioncode=="371522" & year==2008

replace regioncode="371523" if regioncode=="371523" & year==2008

replace regioncode="371524" if regioncode=="371524" & year==2008

replace regioncode="371525" if regioncode=="371525" & year==2008

replace regioncode="371526" if regioncode=="371526" & year==2008

replace regioncode="371581" if regioncode=="371581" & year==2008

replace regioncode="371602" if regioncode=="371602" & year==2008

replace regioncode="371621" if regioncode=="371621" & year==2008

replace regioncode="371622" if regioncode=="371622" & year==2008

replace regioncode="371623" if regioncode=="371623" & year==2008

replace regioncode="371624" if regioncode=="371624" & year==2008

replace regioncode="371625" if regioncode=="371625" & year==2008

replace regioncode="371626" if regioncode=="371626" & year==2008

replace regioncode="371702" if regioncode=="371702" & year==2008

replace regioncode="371721" if regioncode=="371721" & year==2008

replace regioncode="371722" if regioncode=="371722" & year==2008

replace regioncode="371723" if regioncode=="371723" & year==2008

replace regioncode="371724" if regioncode=="371724" & year==2008

replace regioncode="371725" if regioncode=="371725" & year==2008

replace regioncode="371726" if regioncode=="371726" & year==2008

replace regioncode="371727" if regioncode=="371727" & year==2008

replace regioncode="371728" if regioncode=="371728" & year==2008

replace regioncode="410102" if regioncode=="410102" & year==2008

replace regioncode="410103" if regioncode=="410103" & year==2008

replace regioncode="410104" if regioncode=="410104" & year==2008

replace regioncode="410105" if regioncode=="410105" & year==2008

replace regioncode="410106" if regioncode=="410106" & year==2008

replace regioncode="410108" if regioncode=="410108" & year==2008

replace regioncode="410122" if regioncode=="410122" & year==2008

replace regioncode="410181" if regioncode=="410181" & year==2008

replace regioncode="410182" if regioncode=="410182" & year==2008

replace regioncode="410183" if regioncode=="410183" & year==2008

replace regioncode="410184" if regioncode=="410184" & year==2008

replace regioncode="410185" if regioncode=="410185" & year==2008

replace regioncode="410202" if regioncode=="410202" & year==2008

replace regioncode="410203" if regioncode=="410203" & year==2008

replace regioncode="410204" if regioncode=="410204" & year==2008

replace regioncode="410205" if regioncode=="410205" & year==2008

replace regioncode="410211" if regioncode=="410211" & year==2008

replace regioncode="410221" if regioncode=="410221" & year==2008

replace regioncode="410222" if regioncode=="410222" & year==2008

replace regioncode="410223" if regioncode=="410223" & year==2008

replace regioncode="410224" if regioncode=="410224" & year==2008

replace regioncode="410225" if regioncode=="410225" & year==2008

replace regioncode="410302" if regioncode=="410302" & year==2008

replace regioncode="410303" if regioncode=="410303" & year==2008

replace regioncode="410304" if regioncode=="410304" & year==2008

replace regioncode="410305" if regioncode=="410305" & year==2008

replace regioncode="410306" if regioncode=="410306" & year==2008

replace regioncode="410307" if regioncode=="410311" & year==2008

replace regioncode="410322" if regioncode=="410322" & year==2008

replace regioncode="410323" if regioncode=="410323" & year==2008

replace regioncode="410324" if regioncode=="410324" & year==2008

replace regioncode="410325" if regioncode=="410325" & year==2008

replace regioncode="410326" if regioncode=="410326" & year==2008

replace regioncode="410327" if regioncode=="410327" & year==2008

replace regioncode="410328" if regioncode=="410328" & year==2008

replace regioncode="410329" if regioncode=="410329" & year==2008

replace regioncode="410381" if regioncode=="410381" & year==2008

replace regioncode="410402" if regioncode=="410402" & year==2008

replace regioncode="410403" if regioncode=="410403" & year==2008

replace regioncode="410404" if regioncode=="410404" & year==2008

replace regioncode="410411" if regioncode=="410411" & year==2008

replace regioncode="410421" if regioncode=="410421" & year==2008

replace regioncode="410422" if regioncode=="410422" & year==2008

replace regioncode="410423" if regioncode=="410423" & year==2008

replace regioncode="410425" if regioncode=="410425" & year==2008

replace regioncode="410481" if regioncode=="410481" & year==2008

replace regioncode="410482" if regioncode=="410482" & year==2008

replace regioncode="410502" if regioncode=="410502" & year==2008

replace regioncode="410503" if regioncode=="410503" & year==2008

replace regioncode="410504" if regioncode=="410505" & year==2008

replace regioncode="410511" if regioncode=="410506" & year==2008

replace regioncode="410522" if regioncode=="410522" & year==2008

replace regioncode="410523" if regioncode=="410523" & year==2008

replace regioncode="410526" if regioncode=="410526" & year==2008

replace regioncode="410527" if regioncode=="410527" & year==2008

replace regioncode="410581" if regioncode=="410581" & year==2008

replace regioncode="410602" if regioncode=="410602" & year==2008

replace regioncode="410603" if regioncode=="410603" & year==2008

replace regioncode="410611" if regioncode=="410611" & year==2008

replace regioncode="410621" if regioncode=="410621" & year==2008

replace regioncode="410622" if regioncode=="410622" & year==2008

replace regioncode="410702" if regioncode=="410702" & year==2008

replace regioncode="410703" if regioncode=="410703" & year==2008

replace regioncode="410704" if regioncode=="410704" & year==2008

replace regioncode="410711" if regioncode=="410711" & year==2008

replace regioncode="410721" if regioncode=="410721" & year==2008

replace regioncode="410724" if regioncode=="410724" & year==2008

replace regioncode="410725" if regioncode=="410725" & year==2008

replace regioncode="410726" if regioncode=="410726" & year==2008

replace regioncode="410727" if regioncode=="410727" & year==2008

replace regioncode="410728" if regioncode=="410728" & year==2008

replace regioncode="410781" if regioncode=="410781" & year==2008

replace regioncode="410782" if regioncode=="410782" & year==2008

replace regioncode="410802" if regioncode=="410802" & year==2008

replace regioncode="410803" if regioncode=="410803" & year==2008

replace regioncode="410804" if regioncode=="410804" & year==2008

replace regioncode="410811" if regioncode=="410811" & year==2008

replace regioncode="410821" if regioncode=="410821" & year==2008

replace regioncode="410822" if regioncode=="410822" & year==2008

replace regioncode="410823" if regioncode=="410823" & year==2008

replace regioncode="410825" if regioncode=="410825" & year==2008

replace regioncode="410881" if regioncode=="410881" & year==2008

replace regioncode="410882" if regioncode=="410882" & year==2008

replace regioncode="410883" if regioncode=="410883" & year==2008

replace regioncode="410902" if regioncode=="410902" & year==2008

replace regioncode="410922" if regioncode=="410922" & year==2008

replace regioncode="410923" if regioncode=="410923" & year==2008

replace regioncode="410926" if regioncode=="410926" & year==2008

replace regioncode="410927" if regioncode=="410927" & year==2008

replace regioncode="410928" if regioncode=="410928" & year==2008

replace regioncode="411002" if regioncode=="411002" & year==2008

replace regioncode="411023" if regioncode=="411023" & year==2008

replace regioncode="411024" if regioncode=="411024" & year==2008

replace regioncode="411025" if regioncode=="411025" & year==2008

replace regioncode="411081" if regioncode=="411081" & year==2008

replace regioncode="411082" if regioncode=="411082" & year==2008

replace regioncode="411102" if regioncode=="411102" & year==2008

replace regioncode="411123" if regioncode=="411103" & year==2008

replace regioncode="411121" if regioncode=="411121" & year==2008

replace regioncode="411122" if regioncode=="411122" & year==2008

replace regioncode="411202" if regioncode=="411202" & year==2008

replace regioncode="411221" if regioncode=="411221" & year==2008

replace regioncode="411222" if regioncode=="411222" & year==2008

replace regioncode="411224" if regioncode=="411224" & year==2008

replace regioncode="411281" if regioncode=="411281" & year==2008

replace regioncode="411282" if regioncode=="411282" & year==2008

replace regioncode="411302" if regioncode=="411302" & year==2008

replace regioncode="411303" if regioncode=="411303" & year==2008

replace regioncode="411321" if regioncode=="411321" & year==2008

replace regioncode="411322" if regioncode=="411322" & year==2008

replace regioncode="411323" if regioncode=="411323" & year==2008

replace regioncode="411324" if regioncode=="411324" & year==2008

replace regioncode="411325" if regioncode=="411325" & year==2008

replace regioncode="411326" if regioncode=="411326" & year==2008

replace regioncode="411327" if regioncode=="411327" & year==2008

replace regioncode="411328" if regioncode=="411328" & year==2008

replace regioncode="411329" if regioncode=="411329" & year==2008

replace regioncode="411330" if regioncode=="411330" & year==2008

replace regioncode="411381" if regioncode=="411381" & year==2008

replace regioncode="411402" if regioncode=="411402" & year==2008

replace regioncode="411403" if regioncode=="411403" & year==2008

replace regioncode="411421" if regioncode=="411421" & year==2008

replace regioncode="411422" if regioncode=="411422" & year==2008

replace regioncode="411423" if regioncode=="411423" & year==2008

replace regioncode="411424" if regioncode=="411424" & year==2008

replace regioncode="411425" if regioncode=="411425" & year==2008

replace regioncode="411426" if regioncode=="411426" & year==2008

replace regioncode="411481" if regioncode=="411481" & year==2008

replace regioncode="411502" if regioncode=="411502" & year==2008

replace regioncode="411503" if regioncode=="411503" & year==2008

replace regioncode="411521" if regioncode=="411521" & year==2008

replace regioncode="411522" if regioncode=="411522" & year==2008

replace regioncode="411523" if regioncode=="411523" & year==2008

replace regioncode="411524" if regioncode=="411524" & year==2008

replace regioncode="411525" if regioncode=="411525" & year==2008

replace regioncode="411526" if regioncode=="411526" & year==2008

replace regioncode="411527" if regioncode=="411527" & year==2008

replace regioncode="411528" if regioncode=="411528" & year==2008

replace regioncode="411602" if regioncode=="411602" & year==2008

replace regioncode="411621" if regioncode=="411621" & year==2008

replace regioncode="411622" if regioncode=="411622" & year==2008

replace regioncode="411623" if regioncode=="411623" & year==2008

replace regioncode="411624" if regioncode=="411624" & year==2008

replace regioncode="411625" if regioncode=="411625" & year==2008

replace regioncode="411626" if regioncode=="411626" & year==2008

replace regioncode="411627" if regioncode=="411627" & year==2008

replace regioncode="411628" if regioncode=="411628" & year==2008

replace regioncode="411681" if regioncode=="411681" & year==2008

replace regioncode="411702" if regioncode=="411702" & year==2008

replace regioncode="411721" if regioncode=="411721" & year==2008

replace regioncode="411722" if regioncode=="411722" & year==2008

replace regioncode="411723" if regioncode=="411723" & year==2008

replace regioncode="411724" if regioncode=="411724" & year==2008

replace regioncode="411725" if regioncode=="411725" & year==2008

replace regioncode="411726" if regioncode=="411726" & year==2008

replace regioncode="411727" if regioncode=="411727" & year==2008

replace regioncode="411728" if regioncode=="411728" & year==2008

replace regioncode="411729" if regioncode=="411729" & year==2008

replace regioncode="420102" if regioncode=="420102" & year==2008

replace regioncode="420103" if regioncode=="420103" & year==2008

replace regioncode="420104" if regioncode=="420104" & year==2008

replace regioncode="420105" if regioncode=="420105" & year==2008

replace regioncode="420106" if regioncode=="420106" & year==2008

replace regioncode="420107" if regioncode=="420107" & year==2008

replace regioncode="420111" if regioncode=="420111" & year==2008

replace regioncode="420112" if regioncode=="420112" & year==2008

replace regioncode="420113" if regioncode=="420113" & year==2008

replace regioncode="420114" if regioncode=="420114" & year==2008

replace regioncode="420115" if regioncode=="420115" & year==2008

replace regioncode="420116" if regioncode=="420116" & year==2008

replace regioncode="420117" if regioncode=="420117" & year==2008

replace regioncode="420202" if regioncode=="420202" & year==2008

replace regioncode="420203" if regioncode=="420203" & year==2008

replace regioncode="420204" if regioncode=="420204" & year==2008

replace regioncode="420205" if regioncode=="420205" & year==2008

replace regioncode="420222" if regioncode=="420222" & year==2008

replace regioncode="420281" if regioncode=="420281" & year==2008

replace regioncode="420302" if regioncode=="420302" & year==2008

replace regioncode="420303" if regioncode=="420303" & year==2008

replace regioncode="420321" if regioncode=="420321" & year==2008

replace regioncode="420322" if regioncode=="420322" & year==2008

replace regioncode="420323" if regioncode=="420323" & year==2008

replace regioncode="420324" if regioncode=="420324" & year==2008

replace regioncode="420325" if regioncode=="420325" & year==2008

replace regioncode="420381" if regioncode=="420381" & year==2008

replace regioncode="420502" if regioncode=="420502" & year==2008

replace regioncode="420503" if regioncode=="420503" & year==2008

replace regioncode="420504" if regioncode=="420504" & year==2008

replace regioncode="420505" if regioncode=="420505" & year==2008

replace regioncode="420506" if regioncode=="420506" & year==2008

replace regioncode="420525" if regioncode=="420525" & year==2008

replace regioncode="420526" if regioncode=="420526" & year==2008

replace regioncode="420527" if regioncode=="420527" & year==2008

replace regioncode="420528" if regioncode=="420528" & year==2008

replace regioncode="420529" if regioncode=="420529" & year==2008

replace regioncode="420581" if regioncode=="420581" & year==2008

replace regioncode="420582" if regioncode=="420582" & year==2008

replace regioncode="420583" if regioncode=="420583" & year==2008

replace regioncode="420602" if regioncode=="420602" & year==2008

replace regioncode="420606" if regioncode=="420606" & year==2008

replace regioncode="420607" if regioncode=="420607" & year==2008

replace regioncode="420624" if regioncode=="420624" & year==2008

replace regioncode="420625" if regioncode=="420625" & year==2008

replace regioncode="420626" if regioncode=="420626" & year==2008

replace regioncode="420682" if regioncode=="420682" & year==2008

replace regioncode="420683" if regioncode=="420683" & year==2008

replace regioncode="420684" if regioncode=="420684" & year==2008

replace regioncode="420702" if regioncode=="420702" & year==2008

replace regioncode="420703" if regioncode=="420703" & year==2008

replace regioncode="420704" if regioncode=="420704" & year==2008

replace regioncode="420802" if regioncode=="420802" & year==2008

replace regioncode="420804" if regioncode=="420804" & year==2008

replace regioncode="420821" if regioncode=="420821" & year==2008

replace regioncode="420822" if regioncode=="420822" & year==2008

replace regioncode="420881" if regioncode=="420881" & year==2008

replace regioncode="420902" if regioncode=="420902" & year==2008

replace regioncode="420921" if regioncode=="420921" & year==2008

replace regioncode="420922" if regioncode=="420922" & year==2008

replace regioncode="420923" if regioncode=="420923" & year==2008

replace regioncode="420981" if regioncode=="420981" & year==2008

replace regioncode="420982" if regioncode=="420982" & year==2008

replace regioncode="421381" if regioncode=="421381" & year==2008

replace regioncode="420984" if regioncode=="420984" & year==2008

replace regioncode="421002" if regioncode=="421002" & year==2008

replace regioncode="421003" if regioncode=="421003" & year==2008

replace regioncode="421022" if regioncode=="421022" & year==2008

replace regioncode="421023" if regioncode=="421023" & year==2008

replace regioncode="421024" if regioncode=="421024" & year==2008

replace regioncode="421081" if regioncode=="421081" & year==2008

replace regioncode="421083" if regioncode=="421083" & year==2008

replace regioncode="421087" if regioncode=="421087" & year==2008

replace regioncode="421102" if regioncode=="421102" & year==2008

replace regioncode="421121" if regioncode=="421121" & year==2008

replace regioncode="421122" if regioncode=="421122" & year==2008

replace regioncode="421123" if regioncode=="421123" & year==2008

replace regioncode="421124" if regioncode=="421124" & year==2008

replace regioncode="421125" if regioncode=="421125" & year==2008

replace regioncode="421126" if regioncode=="421126" & year==2008

replace regioncode="421127" if regioncode=="421127" & year==2008

replace regioncode="421181" if regioncode=="421181" & year==2008

replace regioncode="421182" if regioncode=="421182" & year==2008

replace regioncode="421202" if regioncode=="421202" & year==2008

replace regioncode="421221" if regioncode=="421221" & year==2008

replace regioncode="421222" if regioncode=="421222" & year==2008

replace regioncode="421223" if regioncode=="421223" & year==2008

replace regioncode="421224" if regioncode=="421224" & year==2008

replace regioncode="421281" if regioncode=="421281" & year==2008

replace regioncode="421302" if regioncode=="421302" & year==2008

replace regioncode="422801" if regioncode=="422801" & year==2008

replace regioncode="422802" if regioncode=="422802" & year==2008

replace regioncode="422822" if regioncode=="422822" & year==2008

replace regioncode="422823" if regioncode=="422823" & year==2008

replace regioncode="422825" if regioncode=="422825" & year==2008

replace regioncode="422826" if regioncode=="422826" & year==2008

replace regioncode="422827" if regioncode=="422827" & year==2008

replace regioncode="422828" if regioncode=="422828" & year==2008

replace regioncode="429004" if regioncode=="429004" & year==2008

replace regioncode="429005" if regioncode=="429005" & year==2008

replace regioncode="429006" if regioncode=="429006" & year==2008

replace regioncode="429021" if regioncode=="429021" & year==2008

replace regioncode="430102" if regioncode=="430102" & year==2008

replace regioncode="430103" if regioncode=="430103" & year==2008

replace regioncode="430104" if regioncode=="430104" & year==2008

replace regioncode="430105" if regioncode=="430105" & year==2008

replace regioncode="430111" if regioncode=="430111" & year==2008

replace regioncode="430121" if regioncode=="430121" & year==2008

replace regioncode="430122" if regioncode=="430122" & year==2008

replace regioncode="430124" if regioncode=="430124" & year==2008

replace regioncode="430181" if regioncode=="430181" & year==2008

replace regioncode="430202" if regioncode=="430202" & year==2008

replace regioncode="430203" if regioncode=="430203" & year==2008

replace regioncode="430204" if regioncode=="430204" & year==2008

replace regioncode="430211" if regioncode=="430211" & year==2008

replace regioncode="430221" if regioncode=="430221" & year==2008

replace regioncode="430223" if regioncode=="430223" & year==2008

replace regioncode="430224" if regioncode=="430224" & year==2008

replace regioncode="430225" if regioncode=="430225" & year==2008

replace regioncode="430281" if regioncode=="430281" & year==2008

replace regioncode="430302" if regioncode=="430302" & year==2008

replace regioncode="430304" if regioncode=="430304" & year==2008

replace regioncode="430321" if regioncode=="430321" & year==2008

replace regioncode="430381" if regioncode=="430381" & year==2008

replace regioncode="430382" if regioncode=="430382" & year==2008

replace regioncode="430405" if regioncode=="430405" & year==2008

replace regioncode="430406" if regioncode=="430406" & year==2008

replace regioncode="430407" if regioncode=="430407" & year==2008

replace regioncode="430408" if regioncode=="430408" & year==2008

replace regioncode="430412" if regioncode=="430412" & year==2008

replace regioncode="430421" if regioncode=="430421" & year==2008

replace regioncode="430422" if regioncode=="430422" & year==2008

replace regioncode="430423" if regioncode=="430423" & year==2008

replace regioncode="430424" if regioncode=="430424" & year==2008

replace regioncode="430426" if regioncode=="430426" & year==2008

replace regioncode="430481" if regioncode=="430481" & year==2008

replace regioncode="430482" if regioncode=="430482" & year==2008

replace regioncode="430502" if regioncode=="430502" & year==2008

replace regioncode="430503" if regioncode=="430503" & year==2008

replace regioncode="430511" if regioncode=="430511" & year==2008

replace regioncode="430521" if regioncode=="430521" & year==2008

replace regioncode="430522" if regioncode=="430522" & year==2008

replace regioncode="430523" if regioncode=="430523" & year==2008

replace regioncode="430524" if regioncode=="430524" & year==2008

replace regioncode="430525" if regioncode=="430525" & year==2008

replace regioncode="430527" if regioncode=="430527" & year==2008

replace regioncode="430528" if regioncode=="430528" & year==2008

replace regioncode="430529" if regioncode=="430529" & year==2008

replace regioncode="430581" if regioncode=="430581" & year==2008

replace regioncode="430602" if regioncode=="430602" & year==2008

replace regioncode="430603" if regioncode=="430603" & year==2008

replace regioncode="430611" if regioncode=="430611" & year==2008

replace regioncode="430621" if regioncode=="430621" & year==2008

replace regioncode="430623" if regioncode=="430623" & year==2008

replace regioncode="430624" if regioncode=="430624" & year==2008

replace regioncode="430626" if regioncode=="430626" & year==2008

replace regioncode="430681" if regioncode=="430681" & year==2008

replace regioncode="430682" if regioncode=="430682" & year==2008

replace regioncode="430702" if regioncode=="430702" & year==2008

replace regioncode="430703" if regioncode=="430703" & year==2008

replace regioncode="430721" if regioncode=="430721" & year==2008

replace regioncode="430722" if regioncode=="430722" & year==2008

replace regioncode="430723" if regioncode=="430723" & year==2008

replace regioncode="430724" if regioncode=="430724" & year==2008

replace regioncode="430725" if regioncode=="430725" & year==2008

replace regioncode="430726" if regioncode=="430726" & year==2008

replace regioncode="430781" if regioncode=="430781" & year==2008

replace regioncode="430802" if regioncode=="430802" & year==2008

replace regioncode="430811" if regioncode=="430811" & year==2008

replace regioncode="430821" if regioncode=="430821" & year==2008

replace regioncode="430822" if regioncode=="430822" & year==2008

replace regioncode="430902" if regioncode=="430902" & year==2008

replace regioncode="430903" if regioncode=="430903" & year==2008

replace regioncode="430921" if regioncode=="430921" & year==2008

replace regioncode="430922" if regioncode=="430922" & year==2008

replace regioncode="430923" if regioncode=="430923" & year==2008

replace regioncode="430981" if regioncode=="430981" & year==2008

replace regioncode="431002" if regioncode=="431002" & year==2008

replace regioncode="431003" if regioncode=="431003" & year==2008

replace regioncode="431021" if regioncode=="431021" & year==2008

replace regioncode="431022" if regioncode=="431022" & year==2008

replace regioncode="431023" if regioncode=="431023" & year==2008

replace regioncode="431024" if regioncode=="431024" & year==2008

replace regioncode="431025" if regioncode=="431025" & year==2008

replace regioncode="431026" if regioncode=="431026" & year==2008

replace regioncode="431027" if regioncode=="431027" & year==2008

replace regioncode="431028" if regioncode=="431028" & year==2008

replace regioncode="431081" if regioncode=="431081" & year==2008

replace regioncode="431102" if regioncode=="431102" & year==2008

replace regioncode="431103" if regioncode=="431103" & year==2008

replace regioncode="431121" if regioncode=="431121" & year==2008

replace regioncode="431122" if regioncode=="431122" & year==2008

replace regioncode="431123" if regioncode=="431123" & year==2008

replace regioncode="431124" if regioncode=="431124" & year==2008

replace regioncode="431125" if regioncode=="431125" & year==2008

replace regioncode="431126" if regioncode=="431126" & year==2008

replace regioncode="431127" if regioncode=="431127" & year==2008

replace regioncode="431128" if regioncode=="431128" & year==2008

replace regioncode="431129" if regioncode=="431129" & year==2008

replace regioncode="431202" if regioncode=="431202" & year==2008

replace regioncode="431221" if regioncode=="431221" & year==2008

replace regioncode="431222" if regioncode=="431222" & year==2008

replace regioncode="431223" if regioncode=="431223" & year==2008

replace regioncode="431224" if regioncode=="431224" & year==2008

replace regioncode="431225" if regioncode=="431225" & year==2008

replace regioncode="431226" if regioncode=="431226" & year==2008

replace regioncode="431227" if regioncode=="431227" & year==2008

replace regioncode="431228" if regioncode=="431228" & year==2008

replace regioncode="431229" if regioncode=="431229" & year==2008

replace regioncode="431230" if regioncode=="431230" & year==2008

replace regioncode="431281" if regioncode=="431281" & year==2008

replace regioncode="431302" if regioncode=="431302" & year==2008

replace regioncode="431321" if regioncode=="431321" & year==2008

replace regioncode="431322" if regioncode=="431322" & year==2008

replace regioncode="431381" if regioncode=="431381" & year==2008

replace regioncode="431382" if regioncode=="431382" & year==2008

replace regioncode="433101" if regioncode=="433101" & year==2008

replace regioncode="433122" if regioncode=="433122" & year==2008

replace regioncode="433123" if regioncode=="433123" & year==2008

replace regioncode="433124" if regioncode=="433124" & year==2008

replace regioncode="433125" if regioncode=="433125" & year==2008

replace regioncode="433126" if regioncode=="433126" & year==2008

replace regioncode="433127" if regioncode=="433127" & year==2008

replace regioncode="433130" if regioncode=="433130" & year==2008

replace regioncode="440102" if regioncode=="440115" & year==2008

replace regioncode="440103" if regioncode=="440103" & year==2008

replace regioncode="440104" if regioncode=="440104" & year==2008

replace regioncode="440105" if regioncode=="440105" & year==2008

replace regioncode="440106" if regioncode=="440106" & year==2008

replace regioncode="440107" if regioncode=="440116" & year==2008

replace regioncode="440111" if regioncode=="440111" & year==2008

replace regioncode="440112" if regioncode=="440112" & year==2008

replace regioncode="440113" if regioncode=="440113" & year==2008

replace regioncode="440114" if regioncode=="440114" & year==2008

replace regioncode="440183" if regioncode=="440183" & year==2008

replace regioncode="440184" if regioncode=="440184" & year==2008

replace regioncode="440203" if regioncode=="440203" & year==2008

replace regioncode="440204" if regioncode=="440204" & year==2008

replace regioncode="440221" if regioncode=="440205" & year==2008

replace regioncode="440222" if regioncode=="440222" & year==2008

replace regioncode="440224" if regioncode=="440224" & year==2008

replace regioncode="440229" if regioncode=="440229" & year==2008

replace regioncode="440232" if regioncode=="440232" & year==2008

replace regioncode="440233" if regioncode=="440233" & year==2008

replace regioncode="440281" if regioncode=="440281" & year==2008

replace regioncode="440282" if regioncode=="440282" & year==2008

replace regioncode="440303" if regioncode=="440303" & year==2008

replace regioncode="440304" if regioncode=="440304" & year==2008

replace regioncode="440305" if regioncode=="440305" & year==2008

replace regioncode="440306" if regioncode=="440306" & year==2008

replace regioncode="440307" if regioncode=="440307" & year==2008

replace regioncode="440308" if regioncode=="440308" & year==2008

replace regioncode="440402" if regioncode=="440402" & year==2008

replace regioncode="440403" if regioncode=="440403" & year==2008

replace regioncode="440404" if regioncode=="440404" & year==2008

replace regioncode="440507" if regioncode=="440507" & year==2008

replace regioncode="440511" if regioncode=="440511" & year==2008

replace regioncode="440512" if regioncode=="440512" & year==2008

replace regioncode="440514" if regioncode=="440514" & year==2008

replace regioncode="440515" if regioncode=="440515" & year==2008

replace regioncode="440523" if regioncode=="440523" & year==2008

replace regioncode="440604" if regioncode=="440604" & year==2008

replace regioncode="440605" if regioncode=="440605" & year==2008

replace regioncode="440606" if regioncode=="440606" & year==2008

replace regioncode="440607" if regioncode=="440607" & year==2008

replace regioncode="440608" if regioncode=="440608" & year==2008

replace regioncode="440703" if regioncode=="440703" & year==2008

replace regioncode="440704" if regioncode=="440704" & year==2008

replace regioncode="440781" if regioncode=="440781" & year==2008

replace regioncode="440705" if regioncode=="440705" & year==2008

replace regioncode="440783" if regioncode=="440783" & year==2008

replace regioncode="440784" if regioncode=="440784" & year==2008

replace regioncode="440785" if regioncode=="440785" & year==2008

replace regioncode="440802" if regioncode=="440802" & year==2008

replace regioncode="440803" if regioncode=="440803" & year==2008

replace regioncode="440804" if regioncode=="440804" & year==2008

replace regioncode="440811" if regioncode=="440811" & year==2008

replace regioncode="440823" if regioncode=="440823" & year==2008

replace regioncode="440825" if regioncode=="440825" & year==2008

replace regioncode="440881" if regioncode=="440881" & year==2008

replace regioncode="440882" if regioncode=="440882" & year==2008

replace regioncode="440883" if regioncode=="440883" & year==2008

replace regioncode="440902" if regioncode=="440902" & year==2008

replace regioncode="440903" if regioncode=="440903" & year==2008

replace regioncode="440923" if regioncode=="440923" & year==2008

replace regioncode="440981" if regioncode=="440981" & year==2008

replace regioncode="440982" if regioncode=="440982" & year==2008

replace regioncode="440983" if regioncode=="440983" & year==2008

replace regioncode="441202" if regioncode=="441202" & year==2008

replace regioncode="441203" if regioncode=="441203" & year==2008

replace regioncode="441223" if regioncode=="441223" & year==2008

replace regioncode="441224" if regioncode=="441224" & year==2008

replace regioncode="441225" if regioncode=="441225" & year==2008

replace regioncode="441226" if regioncode=="441226" & year==2008

replace regioncode="441283" if regioncode=="441283" & year==2008

replace regioncode="441284" if regioncode=="441284" & year==2008

replace regioncode="441302" if regioncode=="441302" & year==2008

replace regioncode="441303" if regioncode=="441303" & year==2008

replace regioncode="441322" if regioncode=="441322" & year==2008

replace regioncode="441323" if regioncode=="441323" & year==2008

replace regioncode="441324" if regioncode=="441324" & year==2008

replace regioncode="441402" if regioncode=="441402" & year==2008

replace regioncode="441421" if regioncode=="441421" & year==2008

replace regioncode="441422" if regioncode=="441422" & year==2008

replace regioncode="441423" if regioncode=="441423" & year==2008

replace regioncode="441424" if regioncode=="441424" & year==2008

replace regioncode="441426" if regioncode=="441426" & year==2008

replace regioncode="441427" if regioncode=="441427" & year==2008

replace regioncode="441481" if regioncode=="441481" & year==2008

replace regioncode="441502" if regioncode=="441502" & year==2008

replace regioncode="441521" if regioncode=="441521" & year==2008

replace regioncode="441523" if regioncode=="441523" & year==2008

replace regioncode="441581" if regioncode=="441581" & year==2008

replace regioncode="441602" if regioncode=="441602" & year==2008

replace regioncode="441621" if regioncode=="441621" & year==2008

replace regioncode="441622" if regioncode=="441622" & year==2008

replace regioncode="441623" if regioncode=="441623" & year==2008

replace regioncode="441624" if regioncode=="441624" & year==2008

replace regioncode="441625" if regioncode=="441625" & year==2008

replace regioncode="441702" if regioncode=="441702" & year==2008

replace regioncode="441721" if regioncode=="441721" & year==2008

replace regioncode="441723" if regioncode=="441723" & year==2008

replace regioncode="441781" if regioncode=="441781" & year==2008

replace regioncode="441802" if regioncode=="441802" & year==2008

replace regioncode="441821" if regioncode=="441821" & year==2008

replace regioncode="441823" if regioncode=="441823" & year==2008

replace regioncode="441825" if regioncode=="441825" & year==2008

replace regioncode="441826" if regioncode=="441826" & year==2008

replace regioncode="441827" if regioncode=="441827" & year==2008

replace regioncode="441881" if regioncode=="441881" & year==2008

replace regioncode="441882" if regioncode=="441882" & year==2008

replace regioncode="441900" if regioncode=="441901" & year==2008

replace regioncode="442000" if regioncode=="442001" & year==2008

replace regioncode="445102" if regioncode=="445102" & year==2008

replace regioncode="445121" if regioncode=="445121" & year==2008

replace regioncode="445122" if regioncode=="445122" & year==2008

replace regioncode="445202" if regioncode=="445202" & year==2008

replace regioncode="445221" if regioncode=="445221" & year==2008

replace regioncode="445222" if regioncode=="445222" & year==2008

replace regioncode="445224" if regioncode=="445224" & year==2008

replace regioncode="445281" if regioncode=="445281" & year==2008

replace regioncode="445302" if regioncode=="445302" & year==2008

replace regioncode="445321" if regioncode=="445321" & year==2008

replace regioncode="445322" if regioncode=="445322" & year==2008

replace regioncode="445323" if regioncode=="445323" & year==2008

replace regioncode="445381" if regioncode=="445381" & year==2008

replace regioncode="450102" if regioncode=="450102" & year==2008

replace regioncode="450103" if regioncode=="450103" & year==2008

replace regioncode="450104" if regioncode=="450107" & year==2008

replace regioncode="450105" if regioncode=="450105" & year==2008

replace regioncode="450106" if regioncode=="450108" & year==2008

replace regioncode="450121" if regioncode=="450109" & year==2008

replace regioncode="450122" if regioncode=="450122" & year==2008

replace regioncode="450123" if regioncode=="450123" & year==2008

replace regioncode="450124" if regioncode=="450124" & year==2008

replace regioncode="450125" if regioncode=="450125" & year==2008

replace regioncode="450126" if regioncode=="450126" & year==2008

replace regioncode="450127" if regioncode=="450127" & year==2008

replace regioncode="450202" if regioncode=="450202" & year==2008

replace regioncode="450203" if regioncode=="450203" & year==2008

replace regioncode="450204" if regioncode=="450204" & year==2008

replace regioncode="450205" if regioncode=="450205" & year==2008

replace regioncode="450221" if regioncode=="450221" & year==2008

replace regioncode="450222" if regioncode=="450222" & year==2008

replace regioncode="450223" if regioncode=="450223" & year==2008

replace regioncode="450224" if regioncode=="450224" & year==2008

replace regioncode="450225" if regioncode=="450225" & year==2008

replace regioncode="450226" if regioncode=="450226" & year==2008

replace regioncode="450302" if regioncode=="450302" & year==2008

replace regioncode="450303" if regioncode=="450303" & year==2008

replace regioncode="450304" if regioncode=="450304" & year==2008

replace regioncode="450305" if regioncode=="450305" & year==2008

replace regioncode="450311" if regioncode=="450311" & year==2008

replace regioncode="450321" if regioncode=="450321" & year==2008

replace regioncode="450322" if regioncode=="450322" & year==2008

replace regioncode="450323" if regioncode=="450323" & year==2008

replace regioncode="450324" if regioncode=="450324" & year==2008

replace regioncode="450325" if regioncode=="450325" & year==2008

replace regioncode="450326" if regioncode=="450326" & year==2008

replace regioncode="450327" if regioncode=="450327" & year==2008

replace regioncode="450328" if regioncode=="450328" & year==2008

replace regioncode="450329" if regioncode=="450329" & year==2008

replace regioncode="450330" if regioncode=="450330" & year==2008

replace regioncode="450331" if regioncode=="450331" & year==2008

replace regioncode="450332" if regioncode=="450332" & year==2008

replace regioncode="450403" if regioncode=="450403" & year==2008

replace regioncode="450404" if regioncode=="450404" & year==2008

replace regioncode="450421" if regioncode=="450421" & year==2008

replace regioncode="450422" if regioncode=="450422" & year==2008

replace regioncode="450423" if regioncode=="450423" & year==2008

replace regioncode="450481" if regioncode=="450481" & year==2008

replace regioncode="450502" if regioncode=="450502" & year==2008

replace regioncode="450503" if regioncode=="450503" & year==2008

replace regioncode="450512" if regioncode=="450512" & year==2008

replace regioncode="450521" if regioncode=="450521" & year==2008

replace regioncode="450602" if regioncode=="450602" & year==2008

replace regioncode="450603" if regioncode=="450603" & year==2008

replace regioncode="450621" if regioncode=="450621" & year==2008

replace regioncode="450681" if regioncode=="450681" & year==2008

replace regioncode="450702" if regioncode=="450702" & year==2008

replace regioncode="450703" if regioncode=="450703" & year==2008

replace regioncode="450721" if regioncode=="450721" & year==2008

replace regioncode="450722" if regioncode=="450722" & year==2008

replace regioncode="450802" if regioncode=="450802" & year==2008

replace regioncode="450803" if regioncode=="450803" & year==2008

replace regioncode="450804" if regioncode=="450804" & year==2008

replace regioncode="450821" if regioncode=="450821" & year==2008

replace regioncode="450881" if regioncode=="450881" & year==2008

replace regioncode="450902" if regioncode=="450902" & year==2008

replace regioncode="450921" if regioncode=="450921" & year==2008

replace regioncode="450922" if regioncode=="450922" & year==2008

replace regioncode="450923" if regioncode=="450923" & year==2008

replace regioncode="450924" if regioncode=="450924" & year==2008

replace regioncode="450981" if regioncode=="450981" & year==2008

replace regioncode="451002" if regioncode=="451002" & year==2008

replace regioncode="451021" if regioncode=="451021" & year==2008

replace regioncode="451022" if regioncode=="451022" & year==2008

replace regioncode="451023" if regioncode=="451023" & year==2008

replace regioncode="451024" if regioncode=="451024" & year==2008

replace regioncode="451025" if regioncode=="451025" & year==2008

replace regioncode="451026" if regioncode=="451026" & year==2008

replace regioncode="451027" if regioncode=="451027" & year==2008

replace regioncode="451028" if regioncode=="451028" & year==2008

replace regioncode="451029" if regioncode=="451029" & year==2008

replace regioncode="451030" if regioncode=="451030" & year==2008

replace regioncode="451031" if regioncode=="451031" & year==2008

replace regioncode="451102" if regioncode=="451102" & year==2008

replace regioncode="451121" if regioncode=="451121" & year==2008

replace regioncode="451122" if regioncode=="451122" & year==2008

replace regioncode="451123" if regioncode=="451123" & year==2008

replace regioncode="451202" if regioncode=="451202" & year==2008

replace regioncode="451221" if regioncode=="451221" & year==2008

replace regioncode="451222" if regioncode=="451222" & year==2008

replace regioncode="451223" if regioncode=="451223" & year==2008

replace regioncode="451224" if regioncode=="451224" & year==2008

replace regioncode="451225" if regioncode=="451225" & year==2008

replace regioncode="451226" if regioncode=="451226" & year==2008

replace regioncode="451227" if regioncode=="451227" & year==2008

replace regioncode="451228" if regioncode=="451228" & year==2008

replace regioncode="451229" if regioncode=="451229" & year==2008

replace regioncode="451281" if regioncode=="451281" & year==2008

replace regioncode="451302" if regioncode=="451302" & year==2008

replace regioncode="451321" if regioncode=="451321" & year==2008

replace regioncode="451322" if regioncode=="451322" & year==2008

replace regioncode="451323" if regioncode=="451323" & year==2008

replace regioncode="451324" if regioncode=="451324" & year==2008

replace regioncode="451381" if regioncode=="451381" & year==2008

replace regioncode="451402" if regioncode=="451402" & year==2008

replace regioncode="451421" if regioncode=="451421" & year==2008

replace regioncode="451422" if regioncode=="451422" & year==2008

replace regioncode="451423" if regioncode=="451423" & year==2008

replace regioncode="451424" if regioncode=="451424" & year==2008

replace regioncode="451425" if regioncode=="451425" & year==2008

replace regioncode="451481" if regioncode=="451481" & year==2008

replace regioncode="460105" if regioncode=="460105" & year==2008

replace regioncode="460106" if regioncode=="460106" & year==2008

replace regioncode="460107" if regioncode=="460107" & year==2008

replace regioncode="460108" if regioncode=="460108" & year==2008

replace regioncode="460001" if regioncode=="469001" & year==2008

replace regioncode="460002" if regioncode=="469002" & year==2008

replace regioncode="460003" if regioncode=="469003" & year==2008

replace regioncode="460005" if regioncode=="469005" & year==2008

replace regioncode="460006" if regioncode=="469006" & year==2008

replace regioncode="460007" if regioncode=="469007" & year==2008

replace regioncode="460030" if regioncode=="469025" & year==2008

replace regioncode="460031" if regioncode=="469026" & year==2008

replace regioncode="460033" if regioncode=="469027" & year==2008

replace regioncode="460034" if regioncode=="469028" & year==2008

replace regioncode="460036" if regioncode=="469030" & year==2008

replace regioncode="500101" if regioncode=="500101" & year==2008

replace regioncode="500102" if regioncode=="500102" & year==2008

replace regioncode="500103" if regioncode=="500103" & year==2008

replace regioncode="500104" if regioncode=="500104" & year==2008

replace regioncode="500105" if regioncode=="500105" & year==2008

replace regioncode="500106" if regioncode=="500106" & year==2008

replace regioncode="500107" if regioncode=="500107" & year==2008

replace regioncode="500108" if regioncode=="500108" & year==2008

replace regioncode="500109" if regioncode=="500109" & year==2008

replace regioncode="500110" if regioncode=="500110" & year==2008

replace regioncode="500111" if regioncode=="500111" & year==2008

replace regioncode="500112" if regioncode=="500112" & year==2008

replace regioncode="500113" if regioncode=="500113" & year==2008

replace regioncode="500114" if regioncode=="500114" & year==2008

replace regioncode="500115" if regioncode=="500115" & year==2008

replace regioncode="500381" if regioncode=="500116" & year==2008

replace regioncode="500382" if regioncode=="500117" & year==2008

replace regioncode="500383" if regioncode=="500118" & year==2008

replace regioncode="500384" if regioncode=="500119" & year==2008

replace regioncode="500222" if regioncode=="500222" & year==2008

replace regioncode="500223" if regioncode=="500223" & year==2008

replace regioncode="500224" if regioncode=="500224" & year==2008

replace regioncode="500225" if regioncode=="500225" & year==2008

replace regioncode="500226" if regioncode=="500226" & year==2008

replace regioncode="500227" if regioncode=="500227" & year==2008

replace regioncode="500228" if regioncode=="500228" & year==2008

replace regioncode="500229" if regioncode=="500229" & year==2008

replace regioncode="500230" if regioncode=="500230" & year==2008

replace regioncode="500231" if regioncode=="500231" & year==2008

replace regioncode="500232" if regioncode=="500232" & year==2008

replace regioncode="500233" if regioncode=="500233" & year==2008

replace regioncode="500234" if regioncode=="500234" & year==2008

replace regioncode="500235" if regioncode=="500235" & year==2008

replace regioncode="500236" if regioncode=="500236" & year==2008

replace regioncode="500237" if regioncode=="500237" & year==2008

replace regioncode="500238" if regioncode=="500238" & year==2008

replace regioncode="500240" if regioncode=="500240" & year==2008

replace regioncode="500241" if regioncode=="500241" & year==2008

replace regioncode="500242" if regioncode=="500242" & year==2008

replace regioncode="500243" if regioncode=="500243" & year==2008

replace regioncode="510104" if regioncode=="510104" & year==2008

replace regioncode="510105" if regioncode=="510105" & year==2008

replace regioncode="510106" if regioncode=="510106" & year==2008

replace regioncode="510107" if regioncode=="510107" & year==2008

replace regioncode="510108" if regioncode=="510108" & year==2008

replace regioncode="510112" if regioncode=="510112" & year==2008

replace regioncode="510113" if regioncode=="510113" & year==2008

replace regioncode="510114" if regioncode=="510114" & year==2008

replace regioncode="510115" if regioncode=="510115" & year==2008

replace regioncode="510121" if regioncode=="510121" & year==2008

replace regioncode="510122" if regioncode=="510122" & year==2008

replace regioncode="510124" if regioncode=="510124" & year==2008

replace regioncode="510129" if regioncode=="510129" & year==2008

replace regioncode="510131" if regioncode=="510131" & year==2008

replace regioncode="510132" if regioncode=="510132" & year==2008

replace regioncode="510181" if regioncode=="510181" & year==2008

replace regioncode="510182" if regioncode=="510182" & year==2008

replace regioncode="510183" if regioncode=="510183" & year==2008

replace regioncode="510184" if regioncode=="510184" & year==2008

replace regioncode="510302" if regioncode=="510302" & year==2008

replace regioncode="510303" if regioncode=="510303" & year==2008

replace regioncode="510304" if regioncode=="510304" & year==2008

replace regioncode="510311" if regioncode=="510311" & year==2008

replace regioncode="510321" if regioncode=="510321" & year==2008

replace regioncode="510322" if regioncode=="510322" & year==2008

replace regioncode="510402" if regioncode=="510402" & year==2008

replace regioncode="510403" if regioncode=="510403" & year==2008

replace regioncode="510411" if regioncode=="510411" & year==2008

replace regioncode="510421" if regioncode=="510421" & year==2008

replace regioncode="510422" if regioncode=="510422" & year==2008

replace regioncode="510502" if regioncode=="510502" & year==2008

replace regioncode="510503" if regioncode=="510503" & year==2008

replace regioncode="510504" if regioncode=="510504" & year==2008

replace regioncode="510521" if regioncode=="510521" & year==2008

replace regioncode="510522" if regioncode=="510522" & year==2008

replace regioncode="510524" if regioncode=="510524" & year==2008

replace regioncode="510525" if regioncode=="510525" & year==2008

replace regioncode="510603" if regioncode=="510603" & year==2008

replace regioncode="510623" if regioncode=="510623" & year==2008

replace regioncode="510626" if regioncode=="510626" & year==2008

replace regioncode="510681" if regioncode=="510681" & year==2008

replace regioncode="510682" if regioncode=="510682" & year==2008

replace regioncode="510683" if regioncode=="510683" & year==2008

replace regioncode="510703" if regioncode=="510703" & year==2008

replace regioncode="510704" if regioncode=="510704" & year==2008

replace regioncode="510722" if regioncode=="510722" & year==2008

replace regioncode="510723" if regioncode=="510723" & year==2008

replace regioncode="510724" if regioncode=="510724" & year==2008

replace regioncode="510725" if regioncode=="510725" & year==2008

replace regioncode="510726" if regioncode=="510726" & year==2008

replace regioncode="510727" if regioncode=="510727" & year==2008

replace regioncode="510781" if regioncode=="510781" & year==2008

replace regioncode="510802" if regioncode=="510802" & year==2008

replace regioncode="510811" if regioncode=="510811" & year==2008

replace regioncode="510812" if regioncode=="510812" & year==2008

replace regioncode="510821" if regioncode=="510821" & year==2008

replace regioncode="510822" if regioncode=="510822" & year==2008

replace regioncode="510823" if regioncode=="510823" & year==2008

replace regioncode="510824" if regioncode=="510824" & year==2008

replace regioncode="510902" if regioncode=="510903" & year==2008

replace regioncode="510921" if regioncode=="510921" & year==2008

replace regioncode="510922" if regioncode=="510922" & year==2008

replace regioncode="510923" if regioncode=="510923" & year==2008

replace regioncode="511002" if regioncode=="511002" & year==2008

replace regioncode="511011" if regioncode=="511011" & year==2008

replace regioncode="511024" if regioncode=="511024" & year==2008

replace regioncode="511025" if regioncode=="511025" & year==2008

replace regioncode="511028" if regioncode=="511028" & year==2008

replace regioncode="511102" if regioncode=="511102" & year==2008

replace regioncode="511111" if regioncode=="511111" & year==2008

replace regioncode="511112" if regioncode=="511112" & year==2008

replace regioncode="511113" if regioncode=="511113" & year==2008

replace regioncode="511123" if regioncode=="511123" & year==2008

replace regioncode="511124" if regioncode=="511124" & year==2008

replace regioncode="511126" if regioncode=="511126" & year==2008

replace regioncode="511129" if regioncode=="511129" & year==2008

replace regioncode="511132" if regioncode=="511132" & year==2008

replace regioncode="511133" if regioncode=="511133" & year==2008

replace regioncode="511181" if regioncode=="511181" & year==2008

replace regioncode="511302" if regioncode=="511302" & year==2008

replace regioncode="511303" if regioncode=="511303" & year==2008

replace regioncode="511304" if regioncode=="511304" & year==2008

replace regioncode="511321" if regioncode=="511321" & year==2008

replace regioncode="511322" if regioncode=="511322" & year==2008

replace regioncode="511323" if regioncode=="511323" & year==2008

replace regioncode="511324" if regioncode=="511324" & year==2008

replace regioncode="511325" if regioncode=="511325" & year==2008

replace regioncode="511381" if regioncode=="511381" & year==2008

replace regioncode="511402" if regioncode=="511402" & year==2008

replace regioncode="511421" if regioncode=="511421" & year==2008

replace regioncode="511422" if regioncode=="511422" & year==2008

replace regioncode="511423" if regioncode=="511423" & year==2008

replace regioncode="511424" if regioncode=="511424" & year==2008

replace regioncode="511425" if regioncode=="511425" & year==2008

replace regioncode="511502" if regioncode=="511502" & year==2008

replace regioncode="511521" if regioncode=="511521" & year==2008

replace regioncode="511522" if regioncode=="511522" & year==2008

replace regioncode="511523" if regioncode=="511523" & year==2008

replace regioncode="511524" if regioncode=="511524" & year==2008

replace regioncode="511525" if regioncode=="511525" & year==2008

replace regioncode="511526" if regioncode=="511526" & year==2008

replace regioncode="511527" if regioncode=="511527" & year==2008

replace regioncode="511528" if regioncode=="511528" & year==2008

replace regioncode="511529" if regioncode=="511529" & year==2008

replace regioncode="511602" if regioncode=="511602" & year==2008

replace regioncode="511621" if regioncode=="511621" & year==2008

replace regioncode="511622" if regioncode=="511622" & year==2008

replace regioncode="511623" if regioncode=="511623" & year==2008

replace regioncode="511681" if regioncode=="511681" & year==2008

replace regioncode="511702" if regioncode=="511702" & year==2008

replace regioncode="511721" if regioncode=="511721" & year==2008

replace regioncode="511722" if regioncode=="511722" & year==2008

replace regioncode="511723" if regioncode=="511723" & year==2008

replace regioncode="511724" if regioncode=="511724" & year==2008

replace regioncode="511725" if regioncode=="511725" & year==2008

replace regioncode="511781" if regioncode=="511781" & year==2008

replace regioncode="511802" if regioncode=="511802" & year==2008

replace regioncode="511821" if regioncode=="511821" & year==2008

replace regioncode="511822" if regioncode=="511822" & year==2008

replace regioncode="511823" if regioncode=="511823" & year==2008

replace regioncode="511824" if regioncode=="511824" & year==2008

replace regioncode="511825" if regioncode=="511825" & year==2008

replace regioncode="511826" if regioncode=="511826" & year==2008

replace regioncode="511827" if regioncode=="511827" & year==2008

replace regioncode="511902" if regioncode=="511902" & year==2008

replace regioncode="511921" if regioncode=="511921" & year==2008

replace regioncode="511922" if regioncode=="511922" & year==2008

replace regioncode="511923" if regioncode=="511923" & year==2008

replace regioncode="512002" if regioncode=="512002" & year==2008

replace regioncode="512021" if regioncode=="512021" & year==2008

replace regioncode="512022" if regioncode=="512022" & year==2008

replace regioncode="512081" if regioncode=="512081" & year==2008

replace regioncode="513221" if regioncode=="513221" & year==2008

replace regioncode="513222" if regioncode=="513222" & year==2008

replace regioncode="513223" if regioncode=="513223" & year==2008

replace regioncode="513224" if regioncode=="513224" & year==2008

replace regioncode="513225" if regioncode=="513225" & year==2008

replace regioncode="513226" if regioncode=="513226" & year==2008

replace regioncode="513227" if regioncode=="513227" & year==2008

replace regioncode="513228" if regioncode=="513228" & year==2008

replace regioncode="513229" if regioncode=="513229" & year==2008

replace regioncode="513230" if regioncode=="513230" & year==2008

replace regioncode="513231" if regioncode=="513231" & year==2008

replace regioncode="513232" if regioncode=="513232" & year==2008

replace regioncode="513233" if regioncode=="513233" & year==2008

replace regioncode="513321" if regioncode=="513321" & year==2008

replace regioncode="513322" if regioncode=="513322" & year==2008

replace regioncode="513323" if regioncode=="513323" & year==2008

replace regioncode="513324" if regioncode=="513324" & year==2008

replace regioncode="513325" if regioncode=="513325" & year==2008

replace regioncode="513326" if regioncode=="513326" & year==2008

replace regioncode="513327" if regioncode=="513327" & year==2008

replace regioncode="513328" if regioncode=="513328" & year==2008

replace regioncode="513329" if regioncode=="513329" & year==2008

replace regioncode="513330" if regioncode=="513330" & year==2008

replace regioncode="513331" if regioncode=="513331" & year==2008

replace regioncode="513332" if regioncode=="513332" & year==2008

replace regioncode="513333" if regioncode=="513333" & year==2008

replace regioncode="513334" if regioncode=="513334" & year==2008

replace regioncode="513335" if regioncode=="513335" & year==2008

replace regioncode="513336" if regioncode=="513336" & year==2008

replace regioncode="513337" if regioncode=="513337" & year==2008

replace regioncode="513338" if regioncode=="513338" & year==2008

replace regioncode="513401" if regioncode=="513401" & year==2008

replace regioncode="513422" if regioncode=="513422" & year==2008

replace regioncode="513423" if regioncode=="513423" & year==2008

replace regioncode="513424" if regioncode=="513424" & year==2008

replace regioncode="513425" if regioncode=="513425" & year==2008

replace regioncode="513426" if regioncode=="513426" & year==2008

replace regioncode="513427" if regioncode=="513427" & year==2008

replace regioncode="513428" if regioncode=="513428" & year==2008

replace regioncode="513429" if regioncode=="513429" & year==2008

replace regioncode="513430" if regioncode=="513430" & year==2008

replace regioncode="513431" if regioncode=="513431" & year==2008

replace regioncode="513432" if regioncode=="513432" & year==2008

replace regioncode="513433" if regioncode=="513433" & year==2008

replace regioncode="513434" if regioncode=="513434" & year==2008

replace regioncode="513435" if regioncode=="513435" & year==2008

replace regioncode="513436" if regioncode=="513436" & year==2008

replace regioncode="513437" if regioncode=="513437" & year==2008

replace regioncode="520102" if regioncode=="520102" & year==2008

replace regioncode="520103" if regioncode=="520103" & year==2008

replace regioncode="520111" if regioncode=="520111" & year==2008

replace regioncode="520112" if regioncode=="520112" & year==2008

replace regioncode="520113" if regioncode=="520113" & year==2008

replace regioncode="520114" if regioncode=="520114" & year==2008

replace regioncode="520121" if regioncode=="520121" & year==2008

replace regioncode="520122" if regioncode=="520122" & year==2008

replace regioncode="520123" if regioncode=="520123" & year==2008

replace regioncode="520181" if regioncode=="520181" & year==2008

replace regioncode="520201" if regioncode=="520201" & year==2008

replace regioncode="520222" if regioncode=="520222" & year==2008

replace regioncode="520203" if regioncode=="520203" & year==2008

replace regioncode="520221" if regioncode=="520221" & year==2008

replace regioncode="520302" if regioncode=="520302" & year==2008

replace regioncode="520321" if regioncode=="520321" & year==2008

replace regioncode="520322" if regioncode=="520322" & year==2008

replace regioncode="520323" if regioncode=="520323" & year==2008

replace regioncode="520324" if regioncode=="520324" & year==2008

replace regioncode="520325" if regioncode=="520325" & year==2008

replace regioncode="520326" if regioncode=="520326" & year==2008

replace regioncode="520327" if regioncode=="520327" & year==2008

replace regioncode="520328" if regioncode=="520328" & year==2008

replace regioncode="520329" if regioncode=="520329" & year==2008

replace regioncode="520330" if regioncode=="520330" & year==2008

replace regioncode="520381" if regioncode=="520381" & year==2008

replace regioncode="520382" if regioncode=="520382" & year==2008

replace regioncode="520402" if regioncode=="520402" & year==2008

replace regioncode="520421" if regioncode=="520421" & year==2008

replace regioncode="520422" if regioncode=="520422" & year==2008

replace regioncode="520423" if regioncode=="520423" & year==2008

replace regioncode="520424" if regioncode=="520424" & year==2008

replace regioncode="520425" if regioncode=="520425" & year==2008

replace regioncode="522201" if regioncode=="522201" & year==2008

replace regioncode="522222" if regioncode=="522222" & year==2008

replace regioncode="522223" if regioncode=="522223" & year==2008

replace regioncode="522224" if regioncode=="522224" & year==2008

replace regioncode="522225" if regioncode=="522225" & year==2008

replace regioncode="522226" if regioncode=="522226" & year==2008

replace regioncode="522227" if regioncode=="522227" & year==2008

replace regioncode="522228" if regioncode=="522228" & year==2008

replace regioncode="522229" if regioncode=="522229" & year==2008

replace regioncode="522230" if regioncode=="522230" & year==2008

replace regioncode="522301" if regioncode=="522301" & year==2008

replace regioncode="522322" if regioncode=="522322" & year==2008

replace regioncode="522323" if regioncode=="522323" & year==2008

replace regioncode="522324" if regioncode=="522324" & year==2008

replace regioncode="522325" if regioncode=="522325" & year==2008

replace regioncode="522326" if regioncode=="522326" & year==2008

replace regioncode="522327" if regioncode=="522327" & year==2008

replace regioncode="522328" if regioncode=="522328" & year==2008

replace regioncode="522401" if regioncode=="522401" & year==2008

replace regioncode="522422" if regioncode=="522422" & year==2008

replace regioncode="522423" if regioncode=="522423" & year==2008

replace regioncode="522424" if regioncode=="522424" & year==2008

replace regioncode="522425" if regioncode=="522425" & year==2008

replace regioncode="522426" if regioncode=="522426" & year==2008

replace regioncode="522427" if regioncode=="522427" & year==2008

replace regioncode="522428" if regioncode=="522428" & year==2008

replace regioncode="522601" if regioncode=="522601" & year==2008

replace regioncode="522622" if regioncode=="522622" & year==2008

replace regioncode="522623" if regioncode=="522623" & year==2008

replace regioncode="522624" if regioncode=="522624" & year==2008

replace regioncode="522625" if regioncode=="522625" & year==2008

replace regioncode="522626" if regioncode=="522626" & year==2008

replace regioncode="522627" if regioncode=="522627" & year==2008

replace regioncode="522628" if regioncode=="522628" & year==2008

replace regioncode="522629" if regioncode=="522629" & year==2008

replace regioncode="522630" if regioncode=="522630" & year==2008

replace regioncode="522631" if regioncode=="522631" & year==2008

replace regioncode="522632" if regioncode=="522632" & year==2008

replace regioncode="522633" if regioncode=="522633" & year==2008

replace regioncode="522634" if regioncode=="522634" & year==2008

replace regioncode="522635" if regioncode=="522635" & year==2008

replace regioncode="522636" if regioncode=="522636" & year==2008

replace regioncode="522701" if regioncode=="522701" & year==2008

replace regioncode="522702" if regioncode=="522702" & year==2008

replace regioncode="522722" if regioncode=="522722" & year==2008

replace regioncode="522723" if regioncode=="522723" & year==2008

replace regioncode="522725" if regioncode=="522725" & year==2008

replace regioncode="522726" if regioncode=="522726" & year==2008

replace regioncode="522727" if regioncode=="522727" & year==2008

replace regioncode="522728" if regioncode=="522728" & year==2008

replace regioncode="522729" if regioncode=="522729" & year==2008

replace regioncode="522730" if regioncode=="522730" & year==2008

replace regioncode="522731" if regioncode=="522731" & year==2008

replace regioncode="522732" if regioncode=="522732" & year==2008

replace regioncode="530102" if regioncode=="530102" & year==2008

replace regioncode="530103" if regioncode=="530103" & year==2008

replace regioncode="530111" if regioncode=="530111" & year==2008

replace regioncode="530112" if regioncode=="530112" & year==2008

replace regioncode="530113" if regioncode=="530113" & year==2008

replace regioncode="530121" if regioncode=="530121" & year==2008

replace regioncode="530122" if regioncode=="530122" & year==2008

replace regioncode="530124" if regioncode=="530124" & year==2008

replace regioncode="530125" if regioncode=="530125" & year==2008

replace regioncode="530126" if regioncode=="530126" & year==2008

replace regioncode="530127" if regioncode=="530127" & year==2008

replace regioncode="530128" if regioncode=="530128" & year==2008

replace regioncode="530129" if regioncode=="530129" & year==2008

replace regioncode="530181" if regioncode=="530181" & year==2008

replace regioncode="530302" if regioncode=="530302" & year==2008

replace regioncode="530321" if regioncode=="530321" & year==2008

replace regioncode="530322" if regioncode=="530322" & year==2008

replace regioncode="530323" if regioncode=="530323" & year==2008

replace regioncode="530324" if regioncode=="530324" & year==2008

replace regioncode="530325" if regioncode=="530325" & year==2008

replace regioncode="530326" if regioncode=="530326" & year==2008

replace regioncode="530328" if regioncode=="530328" & year==2008

replace regioncode="530381" if regioncode=="530381" & year==2008

replace regioncode="530402" if regioncode=="530402" & year==2008

replace regioncode="530421" if regioncode=="530421" & year==2008

replace regioncode="530422" if regioncode=="530422" & year==2008

replace regioncode="530423" if regioncode=="530423" & year==2008

replace regioncode="530424" if regioncode=="530424" & year==2008

replace regioncode="530425" if regioncode=="530425" & year==2008

replace regioncode="530426" if regioncode=="530426" & year==2008

replace regioncode="530427" if regioncode=="530427" & year==2008

replace regioncode="530428" if regioncode=="530428" & year==2008

replace regioncode="530502" if regioncode=="530502" & year==2008

replace regioncode="530521" if regioncode=="530521" & year==2008

replace regioncode="530522" if regioncode=="530522" & year==2008

replace regioncode="530523" if regioncode=="530523" & year==2008

replace regioncode="530524" if regioncode=="530524" & year==2008

replace regioncode="530602" if regioncode=="530602" & year==2008

replace regioncode="530621" if regioncode=="530621" & year==2008

replace regioncode="530622" if regioncode=="530622" & year==2008

replace regioncode="530623" if regioncode=="530623" & year==2008

replace regioncode="530624" if regioncode=="530624" & year==2008

replace regioncode="530625" if regioncode=="530625" & year==2008

replace regioncode="530626" if regioncode=="530626" & year==2008

replace regioncode="530627" if regioncode=="530627" & year==2008

replace regioncode="530628" if regioncode=="530628" & year==2008

replace regioncode="530629" if regioncode=="530629" & year==2008

replace regioncode="530630" if regioncode=="530630" & year==2008

replace regioncode="530721" if regioncode=="530721" & year==2008

replace regioncode="530722" if regioncode=="530722" & year==2008

replace regioncode="530723" if regioncode=="530723" & year==2008

replace regioncode="530724" if regioncode=="530724" & year==2008

replace regioncode="532701" if regioncode=="530802" & year==2008

replace regioncode="532722" if regioncode=="530821" & year==2008

replace regioncode="532723" if regioncode=="530822" & year==2008

replace regioncode="532724" if regioncode=="530823" & year==2008

replace regioncode="532725" if regioncode=="530824" & year==2008

replace regioncode="532726" if regioncode=="530825" & year==2008

replace regioncode="532727" if regioncode=="530826" & year==2008

replace regioncode="532728" if regioncode=="530827" & year==2008

replace regioncode="532729" if regioncode=="530828" & year==2008

replace regioncode="532730" if regioncode=="530829" & year==2008

replace regioncode="533521" if regioncode=="530902" & year==2008

replace regioncode="533522" if regioncode=="530921" & year==2008

replace regioncode="533523" if regioncode=="530922" & year==2008

replace regioncode="533524" if regioncode=="530923" & year==2008

replace regioncode="533525" if regioncode=="530924" & year==2008

replace regioncode="533526" if regioncode=="530925" & year==2008

replace regioncode="533527" if regioncode=="530926" & year==2008

replace regioncode="533528" if regioncode=="530927" & year==2008

replace regioncode="532301" if regioncode=="532301" & year==2008

replace regioncode="532322" if regioncode=="532322" & year==2008

replace regioncode="532323" if regioncode=="532323" & year==2008

replace regioncode="532324" if regioncode=="532324" & year==2008

replace regioncode="532325" if regioncode=="532325" & year==2008

replace regioncode="532326" if regioncode=="532326" & year==2008

replace regioncode="532327" if regioncode=="532327" & year==2008

replace regioncode="532328" if regioncode=="532328" & year==2008

replace regioncode="532329" if regioncode=="532329" & year==2008

replace regioncode="532331" if regioncode=="532331" & year==2008

replace regioncode="532501" if regioncode=="532501" & year==2008

replace regioncode="532502" if regioncode=="532502" & year==2008

replace regioncode="532522" if regioncode=="532522" & year==2008

replace regioncode="532523" if regioncode=="532523" & year==2008

replace regioncode="532524" if regioncode=="532524" & year==2008

replace regioncode="532525" if regioncode=="532525" & year==2008

replace regioncode="532526" if regioncode=="532526" & year==2008

replace regioncode="532527" if regioncode=="532527" & year==2008

replace regioncode="532528" if regioncode=="532528" & year==2008

replace regioncode="532529" if regioncode=="532529" & year==2008

replace regioncode="532530" if regioncode=="532530" & year==2008

replace regioncode="532531" if regioncode=="532531" & year==2008

replace regioncode="532532" if regioncode=="532532" & year==2008

replace regioncode="532621" if regioncode=="532621" & year==2008

replace regioncode="532622" if regioncode=="532622" & year==2008

replace regioncode="532623" if regioncode=="532623" & year==2008

replace regioncode="532624" if regioncode=="532624" & year==2008

replace regioncode="532625" if regioncode=="532625" & year==2008

replace regioncode="532626" if regioncode=="532626" & year==2008

replace regioncode="532627" if regioncode=="532627" & year==2008

replace regioncode="532628" if regioncode=="532628" & year==2008

replace regioncode="532801" if regioncode=="532801" & year==2008

replace regioncode="532822" if regioncode=="532822" & year==2008

replace regioncode="532823" if regioncode=="532823" & year==2008

replace regioncode="532901" if regioncode=="532901" & year==2008

replace regioncode="532922" if regioncode=="532922" & year==2008

replace regioncode="532923" if regioncode=="532923" & year==2008

replace regioncode="532924" if regioncode=="532924" & year==2008

replace regioncode="532925" if regioncode=="532925" & year==2008

replace regioncode="532926" if regioncode=="532926" & year==2008

replace regioncode="532927" if regioncode=="532927" & year==2008

replace regioncode="532928" if regioncode=="532928" & year==2008

replace regioncode="532929" if regioncode=="532929" & year==2008

replace regioncode="532930" if regioncode=="532930" & year==2008

replace regioncode="532931" if regioncode=="532931" & year==2008

replace regioncode="532932" if regioncode=="532932" & year==2008

replace regioncode="533102" if regioncode=="533102" & year==2008

replace regioncode="533103" if regioncode=="533103" & year==2008

replace regioncode="533122" if regioncode=="533122" & year==2008

replace regioncode="533123" if regioncode=="533123" & year==2008

replace regioncode="533124" if regioncode=="533124" & year==2008

replace regioncode="533321" if regioncode=="533321" & year==2008

replace regioncode="533323" if regioncode=="533323" & year==2008

replace regioncode="533324" if regioncode=="533324" & year==2008

replace regioncode="533325" if regioncode=="533325" & year==2008

replace regioncode="533421" if regioncode=="533421" & year==2008

replace regioncode="533422" if regioncode=="533422" & year==2008

replace regioncode="533423" if regioncode=="533423" & year==2008

replace regioncode="540102" if regioncode=="540102" & year==2008

replace regioncode="540121" if regioncode=="540121" & year==2008

replace regioncode="540122" if regioncode=="540122" & year==2008

replace regioncode="540123" if regioncode=="540123" & year==2008

replace regioncode="540124" if regioncode=="540124" & year==2008

replace regioncode="540125" if regioncode=="540125" & year==2008

replace regioncode="540126" if regioncode=="540126" & year==2008

replace regioncode="540127" if regioncode=="540127" & year==2008

replace regioncode="542121" if regioncode=="542121" & year==2008

replace regioncode="542122" if regioncode=="542122" & year==2008

replace regioncode="542123" if regioncode=="542123" & year==2008

replace regioncode="542124" if regioncode=="542124" & year==2008

replace regioncode="542125" if regioncode=="542125" & year==2008

replace regioncode="542126" if regioncode=="542126" & year==2008

replace regioncode="542127" if regioncode=="542127" & year==2008

replace regioncode="542128" if regioncode=="542128" & year==2008

replace regioncode="542129" if regioncode=="542129" & year==2008

replace regioncode="542132" if regioncode=="542132" & year==2008

replace regioncode="542133" if regioncode=="542133" & year==2008

replace regioncode="542221" if regioncode=="542221" & year==2008

replace regioncode="542223" if regioncode=="542223" & year==2008

replace regioncode="542224" if regioncode=="542224" & year==2008

replace regioncode="542225" if regioncode=="542225" & year==2008

replace regioncode="542227" if regioncode=="542227" & year==2008

replace regioncode="542228" if regioncode=="542228" & year==2008

replace regioncode="542229" if regioncode=="542229" & year==2008

replace regioncode="542231" if regioncode=="542231" & year==2008

replace regioncode="542232" if regioncode=="542232" & year==2008

replace regioncode="542233" if regioncode=="542233" & year==2008

replace regioncode="542301" if regioncode=="542301" & year==2008

replace regioncode="542322" if regioncode=="542322" & year==2008

replace regioncode="542323" if regioncode=="542323" & year==2008

replace regioncode="542324" if regioncode=="542324" & year==2008

replace regioncode="542325" if regioncode=="542325" & year==2008

replace regioncode="542326" if regioncode=="542326" & year==2008

replace regioncode="542327" if regioncode=="542327" & year==2008

replace regioncode="542328" if regioncode=="542328" & year==2008

replace regioncode="542329" if regioncode=="542329" & year==2008

replace regioncode="542330" if regioncode=="542330" & year==2008

replace regioncode="542331" if regioncode=="542331" & year==2008

replace regioncode="542332" if regioncode=="542332" & year==2008

replace regioncode="542334" if regioncode=="542334" & year==2008

replace regioncode="542336" if regioncode=="542336" & year==2008

replace regioncode="542338" if regioncode=="542338" & year==2008

replace regioncode="542421" if regioncode=="542421" & year==2008

replace regioncode="542422" if regioncode=="542422" & year==2008

replace regioncode="542423" if regioncode=="542423" & year==2008

replace regioncode="542424" if regioncode=="542424" & year==2008

replace regioncode="542425" if regioncode=="542425" & year==2008

replace regioncode="542426" if regioncode=="542426" & year==2008

replace regioncode="542427" if regioncode=="542427" & year==2008

replace regioncode="542429" if regioncode=="542429" & year==2008

replace regioncode="542430" if regioncode=="542430" & year==2008

replace regioncode="542523" if regioncode=="542523" & year==2008

replace regioncode="542621" if regioncode=="542621" & year==2008

replace regioncode="542622" if regioncode=="542622" & year==2008

replace regioncode="542623" if regioncode=="542623" & year==2008

replace regioncode="542625" if regioncode=="542625" & year==2008

replace regioncode="542626" if regioncode=="542626" & year==2008

replace regioncode="610102" if regioncode=="610102" & year==2008

replace regioncode="610103" if regioncode=="610103" & year==2008

replace regioncode="610104" if regioncode=="610104" & year==2008

replace regioncode="610111" if regioncode=="610111" & year==2008

replace regioncode="610112" if regioncode=="610112" & year==2008

replace regioncode="610113" if regioncode=="610113" & year==2008

replace regioncode="610114" if regioncode=="610114" & year==2008

replace regioncode="610115" if regioncode=="610115" & year==2008

replace regioncode="610116" if regioncode=="610116" & year==2008

replace regioncode="610122" if regioncode=="610122" & year==2008

replace regioncode="610124" if regioncode=="610124" & year==2008

replace regioncode="610125" if regioncode=="610125" & year==2008

replace regioncode="610126" if regioncode=="610126" & year==2008

replace regioncode="610202" if regioncode=="610202" & year==2008

replace regioncode="610203" if regioncode=="610203" & year==2008

replace regioncode="610204" if regioncode=="610204" & year==2008

replace regioncode="610222" if regioncode=="610222" & year==2008

replace regioncode="610302" if regioncode=="610302" & year==2008

replace regioncode="610303" if regioncode=="610303" & year==2008

replace regioncode="610304" if regioncode=="610304" & year==2008

replace regioncode="610322" if regioncode=="610322" & year==2008

replace regioncode="610323" if regioncode=="610323" & year==2008

replace regioncode="610324" if regioncode=="610324" & year==2008

replace regioncode="610326" if regioncode=="610326" & year==2008

replace regioncode="610327" if regioncode=="610327" & year==2008

replace regioncode="610328" if regioncode=="610328" & year==2008

replace regioncode="610329" if regioncode=="610329" & year==2008

replace regioncode="610330" if regioncode=="610330" & year==2008

replace regioncode="610331" if regioncode=="610331" & year==2008

replace regioncode="610402" if regioncode=="610402" & year==2008

replace regioncode="610403" if regioncode=="610403" & year==2008

replace regioncode="610404" if regioncode=="610404" & year==2008

replace regioncode="610422" if regioncode=="610422" & year==2008

replace regioncode="610423" if regioncode=="610423" & year==2008

replace regioncode="610424" if regioncode=="610424" & year==2008

replace regioncode="610425" if regioncode=="610425" & year==2008

replace regioncode="610426" if regioncode=="610426" & year==2008

replace regioncode="610427" if regioncode=="610427" & year==2008

replace regioncode="610428" if regioncode=="610428" & year==2008

replace regioncode="610429" if regioncode=="610429" & year==2008

replace regioncode="610430" if regioncode=="610430" & year==2008

replace regioncode="610431" if regioncode=="610431" & year==2008

replace regioncode="610481" if regioncode=="610481" & year==2008

replace regioncode="610502" if regioncode=="610502" & year==2008

replace regioncode="610521" if regioncode=="610521" & year==2008

replace regioncode="610522" if regioncode=="610522" & year==2008

replace regioncode="610523" if regioncode=="610523" & year==2008

replace regioncode="610524" if regioncode=="610524" & year==2008

replace regioncode="610525" if regioncode=="610525" & year==2008

replace regioncode="610526" if regioncode=="610526" & year==2008

replace regioncode="610527" if regioncode=="610527" & year==2008

replace regioncode="610528" if regioncode=="610528" & year==2008

replace regioncode="610581" if regioncode=="610581" & year==2008

replace regioncode="610582" if regioncode=="610582" & year==2008

replace regioncode="610602" if regioncode=="610602" & year==2008

replace regioncode="610621" if regioncode=="610621" & year==2008

replace regioncode="610622" if regioncode=="610622" & year==2008

replace regioncode="610623" if regioncode=="610623" & year==2008

replace regioncode="610624" if regioncode=="610624" & year==2008

replace regioncode="610625" if regioncode=="610625" & year==2008

replace regioncode="610626" if regioncode=="610626" & year==2008

replace regioncode="610627" if regioncode=="610627" & year==2008

replace regioncode="610628" if regioncode=="610628" & year==2008

replace regioncode="610629" if regioncode=="610629" & year==2008

replace regioncode="610630" if regioncode=="610630" & year==2008

replace regioncode="610631" if regioncode=="610631" & year==2008

replace regioncode="610632" if regioncode=="610632" & year==2008

replace regioncode="610702" if regioncode=="610702" & year==2008

replace regioncode="610721" if regioncode=="610721" & year==2008

replace regioncode="610722" if regioncode=="610722" & year==2008

replace regioncode="610723" if regioncode=="610723" & year==2008

replace regioncode="610724" if regioncode=="610724" & year==2008

replace regioncode="610725" if regioncode=="610725" & year==2008

replace regioncode="610726" if regioncode=="610726" & year==2008

replace regioncode="610727" if regioncode=="610727" & year==2008

replace regioncode="610728" if regioncode=="610728" & year==2008

replace regioncode="610729" if regioncode=="610729" & year==2008

replace regioncode="610730" if regioncode=="610730" & year==2008

replace regioncode="610802" if regioncode=="610802" & year==2008

replace regioncode="610821" if regioncode=="610821" & year==2008

replace regioncode="610822" if regioncode=="610822" & year==2008

replace regioncode="610823" if regioncode=="610823" & year==2008

replace regioncode="610824" if regioncode=="610824" & year==2008

replace regioncode="610825" if regioncode=="610825" & year==2008

replace regioncode="610826" if regioncode=="610826" & year==2008

replace regioncode="610827" if regioncode=="610827" & year==2008

replace regioncode="610828" if regioncode=="610828" & year==2008

replace regioncode="610829" if regioncode=="610829" & year==2008

replace regioncode="610830" if regioncode=="610830" & year==2008

replace regioncode="610831" if regioncode=="610831" & year==2008

replace regioncode="610902" if regioncode=="610902" & year==2008

replace regioncode="610921" if regioncode=="610921" & year==2008

replace regioncode="610922" if regioncode=="610922" & year==2008

replace regioncode="610923" if regioncode=="610923" & year==2008

replace regioncode="610924" if regioncode=="610924" & year==2008

replace regioncode="610925" if regioncode=="610925" & year==2008

replace regioncode="610926" if regioncode=="610926" & year==2008

replace regioncode="610927" if regioncode=="610927" & year==2008

replace regioncode="610928" if regioncode=="610928" & year==2008

replace regioncode="610929" if regioncode=="610929" & year==2008

replace regioncode="611002" if regioncode=="611002" & year==2008

replace regioncode="611021" if regioncode=="611021" & year==2008

replace regioncode="611022" if regioncode=="611022" & year==2008

replace regioncode="611023" if regioncode=="611023" & year==2008

replace regioncode="611024" if regioncode=="611024" & year==2008

replace regioncode="611025" if regioncode=="611025" & year==2008

replace regioncode="611026" if regioncode=="611026" & year==2008

replace regioncode="620102" if regioncode=="620102" & year==2008

replace regioncode="620103" if regioncode=="620103" & year==2008

replace regioncode="620104" if regioncode=="620104" & year==2008

replace regioncode="620105" if regioncode=="620105" & year==2008

replace regioncode="620111" if regioncode=="620111" & year==2008

replace regioncode="620121" if regioncode=="620121" & year==2008

replace regioncode="620122" if regioncode=="620122" & year==2008

replace regioncode="620123" if regioncode=="620123" & year==2008

replace regioncode="620201" if regioncode=="620201" & year==2008

replace regioncode="620302" if regioncode=="620302" & year==2008

replace regioncode="620321" if regioncode=="620321" & year==2008

replace regioncode="620402" if regioncode=="620402" & year==2008

replace regioncode="620403" if regioncode=="620403" & year==2008

replace regioncode="620421" if regioncode=="620421" & year==2008

replace regioncode="620422" if regioncode=="620422" & year==2008

replace regioncode="620423" if regioncode=="620423" & year==2008

replace regioncode="620502" if regioncode=="620502" & year==2008

replace regioncode="620503" if regioncode=="620503" & year==2008

replace regioncode="620521" if regioncode=="620521" & year==2008

replace regioncode="620522" if regioncode=="620522" & year==2008

replace regioncode="620523" if regioncode=="620523" & year==2008

replace regioncode="620524" if regioncode=="620524" & year==2008

replace regioncode="620525" if regioncode=="620525" & year==2008

replace regioncode="622301" if regioncode=="620602" & year==2008

replace regioncode="622322" if regioncode=="620621" & year==2008

replace regioncode="622323" if regioncode=="620622" & year==2008

replace regioncode="622326" if regioncode=="620623" & year==2008

replace regioncode="622201" if regioncode=="620702" & year==2008

replace regioncode="622222" if regioncode=="620721" & year==2008

replace regioncode="622223" if regioncode=="620722" & year==2008

replace regioncode="622224" if regioncode=="620723" & year==2008

replace regioncode="622225" if regioncode=="620724" & year==2008

replace regioncode="622226" if regioncode=="620725" & year==2008

replace regioncode="622701" if regioncode=="620802" & year==2008

replace regioncode="622722" if regioncode=="620821" & year==2008

replace regioncode="622723" if regioncode=="620822" & year==2008

replace regioncode="622724" if regioncode=="620823" & year==2008

replace regioncode="622725" if regioncode=="620824" & year==2008

replace regioncode="622726" if regioncode=="620825" & year==2008

replace regioncode="622727" if regioncode=="620826" & year==2008

replace regioncode="622102" if regioncode=="620902" & year==2008

replace regioncode="622123" if regioncode=="620921" & year==2008

replace regioncode="622126" if regioncode=="620922" & year==2008

replace regioncode="622124" if regioncode=="620923" & year==2008

replace regioncode="622125" if regioncode=="620924" & year==2008

replace regioncode="622101" if regioncode=="620981" & year==2008

replace regioncode="622103" if regioncode=="620982" & year==2008

replace regioncode="622801" if regioncode=="621002" & year==2008

replace regioncode="622821" if regioncode=="621021" & year==2008

replace regioncode="622822" if regioncode=="621022" & year==2008

replace regioncode="622823" if regioncode=="621023" & year==2008

replace regioncode="622824" if regioncode=="621024" & year==2008

replace regioncode="622825" if regioncode=="621025" & year==2008

replace regioncode="622826" if regioncode=="621026" & year==2008

replace regioncode="622827" if regioncode=="621027" & year==2008

replace regioncode="622421" if regioncode=="621102" & year==2008

replace regioncode="622424" if regioncode=="621121" & year==2008

replace regioncode="622425" if regioncode=="621122" & year==2008

replace regioncode="622426" if regioncode=="621123" & year==2008

replace regioncode="622427" if regioncode=="621124" & year==2008

replace regioncode="622428" if regioncode=="621125" & year==2008

replace regioncode="622429" if regioncode=="621126" & year==2008

replace regioncode="622621" if regioncode=="621202" & year==2008

replace regioncode="622624" if regioncode=="621221" & year==2008

replace regioncode="622626" if regioncode=="621222" & year==2008

replace regioncode="622623" if regioncode=="621223" & year==2008

replace regioncode="622625" if regioncode=="621224" & year==2008

replace regioncode="622627" if regioncode=="621225" & year==2008

replace regioncode="622628" if regioncode=="621226" & year==2008

replace regioncode="622630" if regioncode=="621227" & year==2008

replace regioncode="622629" if regioncode=="621228" & year==2008

replace regioncode="622901" if regioncode=="622901" & year==2008

replace regioncode="622921" if regioncode=="622921" & year==2008

replace regioncode="622922" if regioncode=="622922" & year==2008

replace regioncode="622923" if regioncode=="622923" & year==2008

replace regioncode="622924" if regioncode=="622924" & year==2008

replace regioncode="622925" if regioncode=="622925" & year==2008

replace regioncode="622926" if regioncode=="622926" & year==2008

replace regioncode="622927" if regioncode=="622927" & year==2008

replace regioncode="623001" if regioncode=="623001" & year==2008

replace regioncode="623021" if regioncode=="623021" & year==2008

replace regioncode="623022" if regioncode=="623022" & year==2008

replace regioncode="623023" if regioncode=="623023" & year==2008

replace regioncode="623024" if regioncode=="623024" & year==2008

replace regioncode="623025" if regioncode=="623025" & year==2008

replace regioncode="623026" if regioncode=="623026" & year==2008

replace regioncode="623027" if regioncode=="623027" & year==2008

replace regioncode="630102" if regioncode=="630102" & year==2008

replace regioncode="630103" if regioncode=="630103" & year==2008

replace regioncode="630104" if regioncode=="630104" & year==2008

replace regioncode="630105" if regioncode=="630105" & year==2008

replace regioncode="630121" if regioncode=="630121" & year==2008

replace regioncode="630122" if regioncode=="630122" & year==2008

replace regioncode="630123" if regioncode=="630123" & year==2008

replace regioncode="632121" if regioncode=="632121" & year==2008

replace regioncode="632122" if regioncode=="632122" & year==2008

replace regioncode="632123" if regioncode=="632123" & year==2008

replace regioncode="632126" if regioncode=="632126" & year==2008

replace regioncode="632127" if regioncode=="632127" & year==2008

replace regioncode="632128" if regioncode=="632128" & year==2008

replace regioncode="632221" if regioncode=="632221" & year==2008

replace regioncode="632222" if regioncode=="632222" & year==2008

replace regioncode="632223" if regioncode=="632223" & year==2008

replace regioncode="632224" if regioncode=="632224" & year==2008

replace regioncode="632321" if regioncode=="632321" & year==2008

replace regioncode="632322" if regioncode=="632322" & year==2008

replace regioncode="632521" if regioncode=="632521" & year==2008

replace regioncode="632522" if regioncode=="632522" & year==2008

replace regioncode="632523" if regioncode=="632523" & year==2008

replace regioncode="632524" if regioncode=="632524" & year==2008

replace regioncode="632525" if regioncode=="632525" & year==2008

replace regioncode="632621" if regioncode=="632621" & year==2008

replace regioncode="632622" if regioncode=="632622" & year==2008

replace regioncode="632623" if regioncode=="632623" & year==2008

replace regioncode="632624" if regioncode=="632624" & year==2008

replace regioncode="632625" if regioncode=="632625" & year==2008

replace regioncode="632626" if regioncode=="632626" & year==2008

replace regioncode="632721" if regioncode=="632721" & year==2008

replace regioncode="632723" if regioncode=="632723" & year==2008

replace regioncode="632724" if regioncode=="632724" & year==2008

replace regioncode="632725" if regioncode=="632725" & year==2008

replace regioncode="632726" if regioncode=="632726" & year==2008

replace regioncode="632801" if regioncode=="632801" & year==2008

replace regioncode="632802" if regioncode=="632802" & year==2008

replace regioncode="632821" if regioncode=="632821" & year==2008

replace regioncode="632822" if regioncode=="632822" & year==2008

replace regioncode="640104" if regioncode=="640104" & year==2008

replace regioncode="640105" if regioncode=="640105" & year==2008

replace regioncode="640106" if regioncode=="640106" & year==2008

replace regioncode="640121" if regioncode=="640121" & year==2008

replace regioncode="640122" if regioncode=="640122" & year==2008

replace regioncode="640181" if regioncode=="640181" & year==2008

replace regioncode="640202" if regioncode=="640202" & year==2008

replace regioncode="640203" if regioncode=="640205" & year==2008

replace regioncode="640221" if regioncode=="640221" & year==2008

replace regioncode="640302" if regioncode=="640302" & year==2008

replace regioncode="640321" if regioncode=="640502" & year==2008

replace regioncode="640322" if regioncode=="640521" & year==2008

replace regioncode="640323" if regioncode=="640323" & year==2008

replace regioncode="640324" if regioncode=="640324" & year==2008

replace regioncode="640381" if regioncode=="640381" & year==2008

replace regioncode="640402" if regioncode=="640402" & year==2008

replace regioncode="640421" if regioncode=="640522" & year==2008

replace regioncode="640422" if regioncode=="640422" & year==2008

replace regioncode="640423" if regioncode=="640423" & year==2008

replace regioncode="640424" if regioncode=="640424" & year==2008

replace regioncode="640425" if regioncode=="640425" & year==2008

replace regioncode="650102" if regioncode=="650102" & year==2008

replace regioncode="650103" if regioncode=="650103" & year==2008

replace regioncode="650104" if regioncode=="650104" & year==2008

replace regioncode="650105" if regioncode=="650105" & year==2008

replace regioncode="650106" if regioncode=="650106" & year==2008

replace regioncode="650107" if regioncode=="650107" & year==2008

replace regioncode="650108" if regioncode=="650109" & year==2008

replace regioncode="650121" if regioncode=="650121" & year==2008

replace regioncode="650202" if regioncode=="650202" & year==2008

replace regioncode="650203" if regioncode=="650203" & year==2008

replace regioncode="650204" if regioncode=="650204" & year==2008

replace regioncode="650205" if regioncode=="650205" & year==2008

replace regioncode="652101" if regioncode=="652101" & year==2008

replace regioncode="652122" if regioncode=="652122" & year==2008

replace regioncode="652123" if regioncode=="652123" & year==2008

replace regioncode="652201" if regioncode=="652201" & year==2008

replace regioncode="652222" if regioncode=="652222" & year==2008

replace regioncode="652223" if regioncode=="652223" & year==2008

replace regioncode="652301" if regioncode=="652301" & year==2008

replace regioncode="652302" if regioncode=="652302" & year==2008

replace regioncode="652303" if regioncode=="652303" & year==2008

replace regioncode="652323" if regioncode=="652323" & year==2008

replace regioncode="652324" if regioncode=="652324" & year==2008

replace regioncode="652325" if regioncode=="652325" & year==2008

replace regioncode="652327" if regioncode=="652327" & year==2008

replace regioncode="652328" if regioncode=="652328" & year==2008

replace regioncode="652701" if regioncode=="652701" & year==2008

replace regioncode="652722" if regioncode=="652722" & year==2008

replace regioncode="652723" if regioncode=="652723" & year==2008

replace regioncode="652801" if regioncode=="652801" & year==2008

replace regioncode="652822" if regioncode=="652822" & year==2008

replace regioncode="652823" if regioncode=="652823" & year==2008

replace regioncode="652824" if regioncode=="652824" & year==2008

replace regioncode="652825" if regioncode=="652825" & year==2008

replace regioncode="652826" if regioncode=="652826" & year==2008

replace regioncode="652827" if regioncode=="652827" & year==2008

replace regioncode="652828" if regioncode=="652828" & year==2008

replace regioncode="652829" if regioncode=="652829" & year==2008

replace regioncode="652901" if regioncode=="652901" & year==2008

replace regioncode="652922" if regioncode=="652922" & year==2008

replace regioncode="652923" if regioncode=="652923" & year==2008

replace regioncode="652924" if regioncode=="652924" & year==2008

replace regioncode="652925" if regioncode=="652925" & year==2008

replace regioncode="652926" if regioncode=="652926" & year==2008

replace regioncode="652928" if regioncode=="652928" & year==2008

replace regioncode="652929" if regioncode=="652929" & year==2008

replace regioncode="653001" if regioncode=="653001" & year==2008

replace regioncode="653022" if regioncode=="653022" & year==2008

replace regioncode="653023" if regioncode=="653023" & year==2008

replace regioncode="653024" if regioncode=="653024" & year==2008

replace regioncode="653101" if regioncode=="653101" & year==2008

replace regioncode="653121" if regioncode=="653121" & year==2008

replace regioncode="653122" if regioncode=="653122" & year==2008

replace regioncode="653123" if regioncode=="653123" & year==2008

replace regioncode="653124" if regioncode=="653124" & year==2008

replace regioncode="653125" if regioncode=="653125" & year==2008

replace regioncode="653126" if regioncode=="653126" & year==2008

replace regioncode="653127" if regioncode=="653127" & year==2008

replace regioncode="653128" if regioncode=="653128" & year==2008

replace regioncode="653129" if regioncode=="653129" & year==2008

replace regioncode="653130" if regioncode=="653130" & year==2008

replace regioncode="653131" if regioncode=="653131" & year==2008

replace regioncode="653201" if regioncode=="653201" & year==2008

replace regioncode="653221" if regioncode=="653221" & year==2008

replace regioncode="653222" if regioncode=="653222" & year==2008

replace regioncode="653223" if regioncode=="653223" & year==2008

replace regioncode="653224" if regioncode=="653224" & year==2008

replace regioncode="653225" if regioncode=="653225" & year==2008

replace regioncode="653226" if regioncode=="653226" & year==2008

replace regioncode="653227" if regioncode=="653227" & year==2008

replace regioncode="654002" if regioncode=="654002" & year==2008

replace regioncode="654003" if regioncode=="654003" & year==2008

replace regioncode="654021" if regioncode=="654021" & year==2008

replace regioncode="654022" if regioncode=="654022" & year==2008

replace regioncode="654023" if regioncode=="654023" & year==2008

replace regioncode="654024" if regioncode=="654024" & year==2008

replace regioncode="654025" if regioncode=="654025" & year==2008

replace regioncode="654026" if regioncode=="654026" & year==2008

replace regioncode="654027" if regioncode=="654027" & year==2008

replace regioncode="654028" if regioncode=="654028" & year==2008

replace regioncode="654201" if regioncode=="654201" & year==2008

replace regioncode="654202" if regioncode=="654202" & year==2008

replace regioncode="654221" if regioncode=="654221" & year==2008

replace regioncode="654223" if regioncode=="654223" & year==2008

replace regioncode="654224" if regioncode=="654224" & year==2008

replace regioncode="654225" if regioncode=="654225" & year==2008

replace regioncode="654226" if regioncode=="654226" & year==2008

replace regioncode="654301" if regioncode=="654301" & year==2008

replace regioncode="654321" if regioncode=="654321" & year==2008

replace regioncode="654322" if regioncode=="654322" & year==2008

replace regioncode="654323" if regioncode=="654323" & year==2008

replace regioncode="654324" if regioncode=="654324" & year==2008

replace regioncode="654325" if regioncode=="654325" & year==2008

replace regioncode="654326" if regioncode=="654326" & year==2008

replace regioncode="659001" if regioncode=="659001" & year==2008

replace regioncode="110101" if regioncode=="110101" & year==2009

replace regioncode="110102" if regioncode=="110102" & year==2009

replace regioncode="110103" if regioncode=="110103" & year==2009

replace regioncode="110104" if regioncode=="110104" & year==2009

replace regioncode="110105" if regioncode=="110105" & year==2009

replace regioncode="110106" if regioncode=="110106" & year==2009

replace regioncode="110107" if regioncode=="110107" & year==2009

replace regioncode="110108" if regioncode=="110108" & year==2009

replace regioncode="110109" if regioncode=="110109" & year==2009

replace regioncode="110111" if regioncode=="110111" & year==2009

replace regioncode="110112" if regioncode=="110112" & year==2009

replace regioncode="110113" if regioncode=="110113" & year==2009

replace regioncode="110114" if regioncode=="110114" & year==2009

replace regioncode="110115" if regioncode=="110115" & year==2009

replace regioncode="110117" if regioncode=="110117" & year==2009

replace regioncode="110116" if regioncode=="110116" & year==2009

replace regioncode="110228" if regioncode=="110228" & year==2009

replace regioncode="110229" if regioncode=="110229" & year==2009

replace regioncode="120101" if regioncode=="120101" & year==2009

replace regioncode="120102" if regioncode=="120102" & year==2009

replace regioncode="120103" if regioncode=="120103" & year==2009

replace regioncode="120104" if regioncode=="120104" & year==2009

replace regioncode="120105" if regioncode=="120105" & year==2009

replace regioncode="120106" if regioncode=="120106" & year==2009

replace regioncode="120107" if regioncode=="120107" & year==2009

replace regioncode="120108" if regioncode=="120108" & year==2009

replace regioncode="120109" if regioncode=="120109" & year==2009

replace regioncode="120110" if regioncode=="120110" & year==2009

replace regioncode="120111" if regioncode=="120111" & year==2009

replace regioncode="120112" if regioncode=="120112" & year==2009

replace regioncode="120113" if regioncode=="120113" & year==2009

replace regioncode="120114" if regioncode=="120114" & year==2009

replace regioncode="120115" if regioncode=="120115" & year==2009

replace regioncode="120221" if regioncode=="120221" & year==2009

replace regioncode="120223" if regioncode=="120223" & year==2009

replace regioncode="120225" if regioncode=="120225" & year==2009

replace regioncode="130102" if regioncode=="130102" & year==2009

replace regioncode="130103" if regioncode=="130103" & year==2009

replace regioncode="130104" if regioncode=="130104" & year==2009

replace regioncode="130105" if regioncode=="130105" & year==2009

replace regioncode="130107" if regioncode=="130107" & year==2009

replace regioncode="130108" if regioncode=="130108" & year==2009

replace regioncode="130121" if regioncode=="130121" & year==2009

replace regioncode="130123" if regioncode=="130123" & year==2009

replace regioncode="130124" if regioncode=="130124" & year==2009

replace regioncode="130125" if regioncode=="130125" & year==2009

replace regioncode="130126" if regioncode=="130126" & year==2009

replace regioncode="130127" if regioncode=="130127" & year==2009

replace regioncode="130128" if regioncode=="130128" & year==2009

replace regioncode="130129" if regioncode=="130129" & year==2009

replace regioncode="130130" if regioncode=="130130" & year==2009

replace regioncode="130131" if regioncode=="130131" & year==2009

replace regioncode="130132" if regioncode=="130132" & year==2009

replace regioncode="130133" if regioncode=="130133" & year==2009

replace regioncode="130181" if regioncode=="130181" & year==2009

replace regioncode="130182" if regioncode=="130182" & year==2009

replace regioncode="130183" if regioncode=="130183" & year==2009

replace regioncode="130184" if regioncode=="130184" & year==2009

replace regioncode="130185" if regioncode=="130185" & year==2009

replace regioncode="130202" if regioncode=="130202" & year==2009

replace regioncode="130203" if regioncode=="130203" & year==2009

replace regioncode="130204" if regioncode=="130204" & year==2009

replace regioncode="130205" if regioncode=="130205" & year==2009

replace regioncode="130208" if regioncode=="130208" & year==2009

replace regioncode="130223" if regioncode=="130223" & year==2009

replace regioncode="130224" if regioncode=="130224" & year==2009

replace regioncode="130225" if regioncode=="130225" & year==2009

replace regioncode="130227" if regioncode=="130227" & year==2009

replace regioncode="130229" if regioncode=="130229" & year==2009

replace regioncode="130230" if regioncode=="130230" & year==2009

replace regioncode="130281" if regioncode=="130281" & year==2009

replace regioncode="130207" if regioncode=="130207" & year==2009

replace regioncode="130283" if regioncode=="130283" & year==2009

replace regioncode="130302" if regioncode=="130302" & year==2009

replace regioncode="130303" if regioncode=="130303" & year==2009

replace regioncode="130304" if regioncode=="130304" & year==2009

replace regioncode="130321" if regioncode=="130321" & year==2009

replace regioncode="130322" if regioncode=="130322" & year==2009

replace regioncode="130323" if regioncode=="130323" & year==2009

replace regioncode="130324" if regioncode=="130324" & year==2009

replace regioncode="130402" if regioncode=="130402" & year==2009

replace regioncode="130403" if regioncode=="130403" & year==2009

replace regioncode="130404" if regioncode=="130404" & year==2009

replace regioncode="130406" if regioncode=="130406" & year==2009

replace regioncode="130421" if regioncode=="130421" & year==2009

replace regioncode="130423" if regioncode=="130423" & year==2009

replace regioncode="130424" if regioncode=="130424" & year==2009

replace regioncode="130425" if regioncode=="130425" & year==2009

replace regioncode="130426" if regioncode=="130426" & year==2009

replace regioncode="130427" if regioncode=="130427" & year==2009

replace regioncode="130428" if regioncode=="130428" & year==2009

replace regioncode="130429" if regioncode=="130429" & year==2009

replace regioncode="130430" if regioncode=="130430" & year==2009

replace regioncode="130431" if regioncode=="130431" & year==2009

replace regioncode="130432" if regioncode=="130432" & year==2009

replace regioncode="130433" if regioncode=="130433" & year==2009

replace regioncode="130434" if regioncode=="130434" & year==2009

replace regioncode="130435" if regioncode=="130435" & year==2009

replace regioncode="130481" if regioncode=="130481" & year==2009

replace regioncode="130502" if regioncode=="130502" & year==2009

replace regioncode="130503" if regioncode=="130503" & year==2009

replace regioncode="130521" if regioncode=="130521" & year==2009

replace regioncode="130522" if regioncode=="130522" & year==2009

replace regioncode="130523" if regioncode=="130523" & year==2009

replace regioncode="130524" if regioncode=="130524" & year==2009

replace regioncode="130525" if regioncode=="130525" & year==2009

replace regioncode="130526" if regioncode=="130526" & year==2009

replace regioncode="130527" if regioncode=="130527" & year==2009

replace regioncode="130528" if regioncode=="130528" & year==2009

replace regioncode="130529" if regioncode=="130529" & year==2009

replace regioncode="130530" if regioncode=="130530" & year==2009

replace regioncode="130531" if regioncode=="130531" & year==2009

replace regioncode="130532" if regioncode=="130532" & year==2009

replace regioncode="130533" if regioncode=="130533" & year==2009

replace regioncode="130534" if regioncode=="130534" & year==2009

replace regioncode="130535" if regioncode=="130535" & year==2009

replace regioncode="130581" if regioncode=="130581" & year==2009

replace regioncode="130582" if regioncode=="130582" & year==2009

replace regioncode="130602" if regioncode=="130602" & year==2009

replace regioncode="130603" if regioncode=="130603" & year==2009

replace regioncode="130604" if regioncode=="130604" & year==2009

replace regioncode="130621" if regioncode=="130621" & year==2009

replace regioncode="130622" if regioncode=="130622" & year==2009

replace regioncode="130623" if regioncode=="130623" & year==2009

replace regioncode="130624" if regioncode=="130624" & year==2009

replace regioncode="130625" if regioncode=="130625" & year==2009

replace regioncode="130626" if regioncode=="130626" & year==2009

replace regioncode="130627" if regioncode=="130627" & year==2009

replace regioncode="130628" if regioncode=="130628" & year==2009

replace regioncode="130629" if regioncode=="130629" & year==2009

replace regioncode="130630" if regioncode=="130630" & year==2009

replace regioncode="130631" if regioncode=="130631" & year==2009

replace regioncode="130632" if regioncode=="130632" & year==2009

replace regioncode="130633" if regioncode=="130633" & year==2009

replace regioncode="130634" if regioncode=="130634" & year==2009

replace regioncode="130635" if regioncode=="130635" & year==2009

replace regioncode="130636" if regioncode=="130636" & year==2009

replace regioncode="130637" if regioncode=="130637" & year==2009

replace regioncode="130638" if regioncode=="130638" & year==2009

replace regioncode="130681" if regioncode=="130681" & year==2009

replace regioncode="130682" if regioncode=="130682" & year==2009

replace regioncode="130683" if regioncode=="130683" & year==2009

replace regioncode="130684" if regioncode=="130684" & year==2009

replace regioncode="130702" if regioncode=="130702" & year==2009

replace regioncode="130703" if regioncode=="130703" & year==2009

replace regioncode="130705" if regioncode=="130705" & year==2009

replace regioncode="130706" if regioncode=="130706" & year==2009

replace regioncode="130721" if regioncode=="130721" & year==2009

replace regioncode="130722" if regioncode=="130722" & year==2009

replace regioncode="130723" if regioncode=="130723" & year==2009

replace regioncode="130724" if regioncode=="130724" & year==2009

replace regioncode="130725" if regioncode=="130725" & year==2009

replace regioncode="130726" if regioncode=="130726" & year==2009

replace regioncode="130727" if regioncode=="130727" & year==2009

replace regioncode="130728" if regioncode=="130728" & year==2009

replace regioncode="130729" if regioncode=="130729" & year==2009

replace regioncode="130730" if regioncode=="130730" & year==2009

replace regioncode="130731" if regioncode=="130731" & year==2009

replace regioncode="130732" if regioncode=="130732" & year==2009

replace regioncode="130733" if regioncode=="130733" & year==2009

replace regioncode="130802" if regioncode=="130802" & year==2009

replace regioncode="130803" if regioncode=="130803" & year==2009

replace regioncode="130804" if regioncode=="130804" & year==2009

replace regioncode="130821" if regioncode=="130821" & year==2009

replace regioncode="130822" if regioncode=="130822" & year==2009

replace regioncode="130823" if regioncode=="130823" & year==2009

replace regioncode="130824" if regioncode=="130824" & year==2009

replace regioncode="130825" if regioncode=="130825" & year==2009

replace regioncode="130826" if regioncode=="130826" & year==2009

replace regioncode="130827" if regioncode=="130827" & year==2009

replace regioncode="130828" if regioncode=="130828" & year==2009

replace regioncode="130902" if regioncode=="130902" & year==2009

replace regioncode="130903" if regioncode=="130903" & year==2009

replace regioncode="130921" if regioncode=="130921" & year==2009

replace regioncode="130922" if regioncode=="130922" & year==2009

replace regioncode="130923" if regioncode=="130923" & year==2009

replace regioncode="130924" if regioncode=="130924" & year==2009

replace regioncode="130925" if regioncode=="130925" & year==2009

replace regioncode="130926" if regioncode=="130926" & year==2009

replace regioncode="130927" if regioncode=="130927" & year==2009

replace regioncode="130928" if regioncode=="130928" & year==2009

replace regioncode="130929" if regioncode=="130929" & year==2009

replace regioncode="130930" if regioncode=="130930" & year==2009

replace regioncode="130981" if regioncode=="130981" & year==2009

replace regioncode="130982" if regioncode=="130982" & year==2009

replace regioncode="130983" if regioncode=="130983" & year==2009

replace regioncode="130984" if regioncode=="130984" & year==2009

replace regioncode="131002" if regioncode=="131002" & year==2009

replace regioncode="131003" if regioncode=="131003" & year==2009

replace regioncode="131022" if regioncode=="131022" & year==2009

replace regioncode="131023" if regioncode=="131023" & year==2009

replace regioncode="131024" if regioncode=="131024" & year==2009

replace regioncode="131025" if regioncode=="131025" & year==2009

replace regioncode="131026" if regioncode=="131026" & year==2009

replace regioncode="131028" if regioncode=="131028" & year==2009

replace regioncode="131081" if regioncode=="131081" & year==2009

replace regioncode="131082" if regioncode=="131082" & year==2009

replace regioncode="131102" if regioncode=="131102" & year==2009

replace regioncode="131121" if regioncode=="131121" & year==2009

replace regioncode="131122" if regioncode=="131122" & year==2009

replace regioncode="131123" if regioncode=="131123" & year==2009

replace regioncode="131124" if regioncode=="131124" & year==2009

replace regioncode="131125" if regioncode=="131125" & year==2009

replace regioncode="131126" if regioncode=="131126" & year==2009

replace regioncode="131127" if regioncode=="131127" & year==2009

replace regioncode="131128" if regioncode=="131128" & year==2009

replace regioncode="131181" if regioncode=="131181" & year==2009

replace regioncode="131182" if regioncode=="131182" & year==2009

replace regioncode="140105" if regioncode=="140105" & year==2009

replace regioncode="140106" if regioncode=="140106" & year==2009

replace regioncode="140107" if regioncode=="140107" & year==2009

replace regioncode="140108" if regioncode=="140108" & year==2009

replace regioncode="140109" if regioncode=="140109" & year==2009

replace regioncode="140110" if regioncode=="140110" & year==2009

replace regioncode="140121" if regioncode=="140121" & year==2009

replace regioncode="140122" if regioncode=="140122" & year==2009

replace regioncode="140123" if regioncode=="140123" & year==2009

replace regioncode="140181" if regioncode=="140181" & year==2009

replace regioncode="140202" if regioncode=="140202" & year==2009

replace regioncode="140203" if regioncode=="140203" & year==2009

replace regioncode="140211" if regioncode=="140211" & year==2009

replace regioncode="140212" if regioncode=="140212" & year==2009

replace regioncode="140221" if regioncode=="140221" & year==2009

replace regioncode="140222" if regioncode=="140222" & year==2009

replace regioncode="140223" if regioncode=="140223" & year==2009

replace regioncode="140224" if regioncode=="140224" & year==2009

replace regioncode="140225" if regioncode=="140225" & year==2009

replace regioncode="140226" if regioncode=="140226" & year==2009

replace regioncode="140227" if regioncode=="140227" & year==2009

replace regioncode="140302" if regioncode=="140302" & year==2009

replace regioncode="140303" if regioncode=="140303" & year==2009

replace regioncode="140311" if regioncode=="140311" & year==2009

replace regioncode="140321" if regioncode=="140321" & year==2009

replace regioncode="140322" if regioncode=="140322" & year==2009

replace regioncode="140402" if regioncode=="140402" & year==2009

replace regioncode="140411" if regioncode=="140411" & year==2009

replace regioncode="140421" if regioncode=="140421" & year==2009

replace regioncode="140423" if regioncode=="140423" & year==2009

replace regioncode="140424" if regioncode=="140424" & year==2009

replace regioncode="140425" if regioncode=="140425" & year==2009

replace regioncode="140426" if regioncode=="140426" & year==2009

replace regioncode="140427" if regioncode=="140427" & year==2009

replace regioncode="140428" if regioncode=="140428" & year==2009

replace regioncode="140429" if regioncode=="140429" & year==2009

replace regioncode="140430" if regioncode=="140430" & year==2009

replace regioncode="140431" if regioncode=="140431" & year==2009

replace regioncode="140481" if regioncode=="140481" & year==2009

replace regioncode="140502" if regioncode=="140502" & year==2009

replace regioncode="140521" if regioncode=="140521" & year==2009

replace regioncode="140522" if regioncode=="140522" & year==2009

replace regioncode="140524" if regioncode=="140524" & year==2009

replace regioncode="140525" if regioncode=="140525" & year==2009

replace regioncode="140581" if regioncode=="140581" & year==2009

replace regioncode="140602" if regioncode=="140602" & year==2009

replace regioncode="140603" if regioncode=="140603" & year==2009

replace regioncode="140621" if regioncode=="140621" & year==2009

replace regioncode="140622" if regioncode=="140622" & year==2009

replace regioncode="140623" if regioncode=="140623" & year==2009

replace regioncode="140624" if regioncode=="140624" & year==2009

replace regioncode="140902" if regioncode=="140902" & year==2009

replace regioncode="140981" if regioncode=="140981" & year==2009

replace regioncode="140921" if regioncode=="140921" & year==2009

replace regioncode="140922" if regioncode=="140922" & year==2009

replace regioncode="140923" if regioncode=="140923" & year==2009

replace regioncode="140924" if regioncode=="140924" & year==2009

replace regioncode="140925" if regioncode=="140925" & year==2009

replace regioncode="140926" if regioncode=="140926" & year==2009

replace regioncode="140927" if regioncode=="140927" & year==2009

replace regioncode="140928" if regioncode=="140928" & year==2009

replace regioncode="140929" if regioncode=="140929" & year==2009

replace regioncode="140930" if regioncode=="140930" & year==2009

replace regioncode="140931" if regioncode=="140931" & year==2009

replace regioncode="140932" if regioncode=="140932" & year==2009

replace regioncode="141181" if regioncode=="141181" & year==2009

replace regioncode="141102" if regioncode=="141102" & year==2009

replace regioncode="141182" if regioncode=="141182" & year==2009

replace regioncode="141121" if regioncode=="141121" & year==2009

replace regioncode="141122" if regioncode=="141122" & year==2009

replace regioncode="141123" if regioncode=="141123" & year==2009

replace regioncode="141124" if regioncode=="141124" & year==2009

replace regioncode="141125" if regioncode=="141125" & year==2009

replace regioncode="141126" if regioncode=="141126" & year==2009

replace regioncode="141127" if regioncode=="141127" & year==2009

replace regioncode="141128" if regioncode=="141128" & year==2009

replace regioncode="141129" if regioncode=="141129" & year==2009

replace regioncode="141130" if regioncode=="141130" & year==2009

replace regioncode="140702" if regioncode=="140702" & year==2009

replace regioncode="140781" if regioncode=="140781" & year==2009

replace regioncode="140721" if regioncode=="140721" & year==2009

replace regioncode="140722" if regioncode=="140722" & year==2009

replace regioncode="140723" if regioncode=="140723" & year==2009

replace regioncode="140724" if regioncode=="140724" & year==2009

replace regioncode="140725" if regioncode=="140725" & year==2009

replace regioncode="140726" if regioncode=="140726" & year==2009

replace regioncode="140727" if regioncode=="140727" & year==2009

replace regioncode="140728" if regioncode=="140728" & year==2009

replace regioncode="140729" if regioncode=="140729" & year==2009

replace regioncode="141002" if regioncode=="141002" & year==2009

replace regioncode="141081" if regioncode=="141081" & year==2009

replace regioncode="141082" if regioncode=="141082" & year==2009

replace regioncode="141021" if regioncode=="141021" & year==2009

replace regioncode="141022" if regioncode=="141022" & year==2009

replace regioncode="141023" if regioncode=="141023" & year==2009

replace regioncode="141024" if regioncode=="141024" & year==2009

replace regioncode="141025" if regioncode=="141025" & year==2009

replace regioncode="141026" if regioncode=="141026" & year==2009

replace regioncode="141027" if regioncode=="141027" & year==2009

replace regioncode="141028" if regioncode=="141028" & year==2009

replace regioncode="141029" if regioncode=="141029" & year==2009

replace regioncode="141033" if regioncode=="141033" & year==2009

replace regioncode="141030" if regioncode=="141030" & year==2009

replace regioncode="141032" if regioncode=="141032" & year==2009

replace regioncode="141031" if regioncode=="141031" & year==2009

replace regioncode="141034" if regioncode=="141034" & year==2009

replace regioncode="140802" if regioncode=="140802" & year==2009

replace regioncode="140881" if regioncode=="140881" & year==2009

replace regioncode="140882" if regioncode=="140882" & year==2009

replace regioncode="140830" if regioncode=="140830" & year==2009

replace regioncode="140821" if regioncode=="140821" & year==2009

replace regioncode="140822" if regioncode=="140822" & year==2009

replace regioncode="140825" if regioncode=="140825" & year==2009

replace regioncode="140824" if regioncode=="140824" & year==2009

replace regioncode="140823" if regioncode=="140823" & year==2009

replace regioncode="140828" if regioncode=="140828" & year==2009

replace regioncode="140826" if regioncode=="140826" & year==2009

replace regioncode="140829" if regioncode=="140829" & year==2009

replace regioncode="140827" if regioncode=="140827" & year==2009

replace regioncode="150102" if regioncode=="150102" & year==2009

replace regioncode="150103" if regioncode=="150103" & year==2009

replace regioncode="150104" if regioncode=="150104" & year==2009

replace regioncode="150105" if regioncode=="150105" & year==2009

replace regioncode="150121" if regioncode=="150121" & year==2009

replace regioncode="150122" if regioncode=="150122" & year==2009

replace regioncode="150123" if regioncode=="150123" & year==2009

replace regioncode="150124" if regioncode=="150124" & year==2009

replace regioncode="150125" if regioncode=="150125" & year==2009

replace regioncode="150202" if regioncode=="150202" & year==2009

replace regioncode="150203" if regioncode=="150203" & year==2009

replace regioncode="150204" if regioncode=="150204" & year==2009

replace regioncode="150205" if regioncode=="150205" & year==2009

replace regioncode="150206" if regioncode=="150206" & year==2009

replace regioncode="150207" if regioncode=="150207" & year==2009

replace regioncode="150221" if regioncode=="150221" & year==2009

replace regioncode="150222" if regioncode=="150222" & year==2009

replace regioncode="150223" if regioncode=="150223" & year==2009

replace regioncode="150302" if regioncode=="150302" & year==2009

replace regioncode="150303" if regioncode=="150303" & year==2009

replace regioncode="150304" if regioncode=="150304" & year==2009

replace regioncode="150402" if regioncode=="150402" & year==2009

replace regioncode="150403" if regioncode=="150403" & year==2009

replace regioncode="150404" if regioncode=="150404" & year==2009

replace regioncode="150421" if regioncode=="150421" & year==2009

replace regioncode="150422" if regioncode=="150422" & year==2009

replace regioncode="150423" if regioncode=="150423" & year==2009

replace regioncode="150424" if regioncode=="150424" & year==2009

replace regioncode="150425" if regioncode=="150425" & year==2009

replace regioncode="150426" if regioncode=="150426" & year==2009

replace regioncode="150428" if regioncode=="150428" & year==2009

replace regioncode="150429" if regioncode=="150429" & year==2009

replace regioncode="150430" if regioncode=="150430" & year==2009

replace regioncode="150702" if regioncode=="150702" & year==2009

replace regioncode="150781" if regioncode=="150781" & year==2009

replace regioncode="150783" if regioncode=="150783" & year==2009

replace regioncode="150782" if regioncode=="150782" & year==2009

replace regioncode="150785" if regioncode=="150785" & year==2009

replace regioncode="150784" if regioncode=="150784" & year==2009

replace regioncode="150721" if regioncode=="150721" & year==2009

replace regioncode="150722" if regioncode=="150722" & year==2009

replace regioncode="150723" if regioncode=="150723" & year==2009

replace regioncode="150724" if regioncode=="150724" & year==2009

replace regioncode="150727" if regioncode=="150727" & year==2009

replace regioncode="150726" if regioncode=="150726" & year==2009

replace regioncode="150725" if regioncode=="150725" & year==2009

replace regioncode="152201" if regioncode=="152201" & year==2009

replace regioncode="152202" if regioncode=="152202" & year==2009

replace regioncode="152221" if regioncode=="152221" & year==2009

replace regioncode="152222" if regioncode=="152222" & year==2009

replace regioncode="152223" if regioncode=="152223" & year==2009

replace regioncode="152224" if regioncode=="152224" & year==2009

replace regioncode="150502" if regioncode=="150502" & year==2009

replace regioncode="150581" if regioncode=="150581" & year==2009

replace regioncode="150521" if regioncode=="150521" & year==2009

replace regioncode="150522" if regioncode=="150522" & year==2009

replace regioncode="150523" if regioncode=="150523" & year==2009

replace regioncode="150524" if regioncode=="150524" & year==2009

replace regioncode="150525" if regioncode=="150525" & year==2009

replace regioncode="150526" if regioncode=="150526" & year==2009

replace regioncode="152501" if regioncode=="152501" & year==2009

replace regioncode="152502" if regioncode=="152502" & year==2009

replace regioncode="152522" if regioncode=="152522" & year==2009

replace regioncode="152523" if regioncode=="152523" & year==2009

replace regioncode="152524" if regioncode=="152524" & year==2009

replace regioncode="152525" if regioncode=="152525" & year==2009

replace regioncode="152526" if regioncode=="152526" & year==2009

replace regioncode="152527" if regioncode=="152527" & year==2009

replace regioncode="152528" if regioncode=="152528" & year==2009

replace regioncode="152529" if regioncode=="152529" & year==2009

replace regioncode="152530" if regioncode=="152530" & year==2009

replace regioncode="152531" if regioncode=="152531" & year==2009

replace regioncode="152601" if regioncode=="150902" & year==2009

replace regioncode="152602" if regioncode=="150981" & year==2009

replace regioncode="152624" if regioncode=="150921" & year==2009

replace regioncode="152625" if regioncode=="150922" & year==2009

replace regioncode="152626" if regioncode=="150923" & year==2009

replace regioncode="152627" if regioncode=="150924" & year==2009

replace regioncode="152629" if regioncode=="150925" & year==2009

replace regioncode="152630" if regioncode=="150926" & year==2009

replace regioncode="152631" if regioncode=="150927" & year==2009

replace regioncode="152632" if regioncode=="150928" & year==2009

replace regioncode="152634" if regioncode=="150929" & year==2009

replace regioncode="150602" if regioncode=="150602" & year==2009

replace regioncode="150621" if regioncode=="150621" & year==2009

replace regioncode="150622" if regioncode=="150622" & year==2009

replace regioncode="150623" if regioncode=="150623" & year==2009

replace regioncode="150624" if regioncode=="150624" & year==2009

replace regioncode="150625" if regioncode=="150625" & year==2009

replace regioncode="150626" if regioncode=="150626" & year==2009

replace regioncode="150627" if regioncode=="150627" & year==2009

replace regioncode="152801" if regioncode=="150802" & year==2009

replace regioncode="152822" if regioncode=="150821" & year==2009

replace regioncode="152823" if regioncode=="150822" & year==2009

replace regioncode="152824" if regioncode=="150823" & year==2009

replace regioncode="152825" if regioncode=="150824" & year==2009

replace regioncode="152826" if regioncode=="150825" & year==2009

replace regioncode="152827" if regioncode=="150826" & year==2009

replace regioncode="152921" if regioncode=="152921" & year==2009

replace regioncode="152922" if regioncode=="152922" & year==2009

replace regioncode="152923" if regioncode=="152923" & year==2009

replace regioncode="210102" if regioncode=="210102" & year==2009

replace regioncode="210103" if regioncode=="210103" & year==2009

replace regioncode="210104" if regioncode=="210104" & year==2009

replace regioncode="210105" if regioncode=="210105" & year==2009

replace regioncode="210106" if regioncode=="210106" & year==2009

replace regioncode="210111" if regioncode=="210111" & year==2009

replace regioncode="210112" if regioncode=="210112" & year==2009

replace regioncode="210113" if regioncode=="210113" & year==2009

replace regioncode="210114" if regioncode=="210114" & year==2009

replace regioncode="210122" if regioncode=="210122" & year==2009

replace regioncode="210123" if regioncode=="210123" & year==2009

replace regioncode="210124" if regioncode=="210124" & year==2009

replace regioncode="210181" if regioncode=="210181" & year==2009

replace regioncode="210202" if regioncode=="210202" & year==2009

replace regioncode="210203" if regioncode=="210203" & year==2009

replace regioncode="210204" if regioncode=="210204" & year==2009

replace regioncode="210211" if regioncode=="210211" & year==2009

replace regioncode="210212" if regioncode=="210212" & year==2009

replace regioncode="210213" if regioncode=="210213" & year==2009

replace regioncode="210224" if regioncode=="210224" & year==2009

replace regioncode="210281" if regioncode=="210281" & year==2009

replace regioncode="210282" if regioncode=="210282" & year==2009

replace regioncode="210283" if regioncode=="210283" & year==2009

replace regioncode="210302" if regioncode=="210302" & year==2009

replace regioncode="210303" if regioncode=="210303" & year==2009

replace regioncode="210304" if regioncode=="210304" & year==2009

replace regioncode="210311" if regioncode=="210311" & year==2009

replace regioncode="210321" if regioncode=="210321" & year==2009

replace regioncode="210323" if regioncode=="210323" & year==2009

replace regioncode="210381" if regioncode=="210381" & year==2009

replace regioncode="210402" if regioncode=="210402" & year==2009

replace regioncode="210403" if regioncode=="210403" & year==2009

replace regioncode="210404" if regioncode=="210404" & year==2009

replace regioncode="210411" if regioncode=="210411" & year==2009

replace regioncode="210421" if regioncode=="210421" & year==2009

replace regioncode="210422" if regioncode=="210422" & year==2009

replace regioncode="210423" if regioncode=="210423" & year==2009

replace regioncode="210502" if regioncode=="210502" & year==2009

replace regioncode="210503" if regioncode=="210503" & year==2009

replace regioncode="210504" if regioncode=="210504" & year==2009

replace regioncode="210505" if regioncode=="210505" & year==2009

replace regioncode="210521" if regioncode=="210521" & year==2009

replace regioncode="210522" if regioncode=="210522" & year==2009

replace regioncode="210602" if regioncode=="210602" & year==2009

replace regioncode="210603" if regioncode=="210603" & year==2009

replace regioncode="210604" if regioncode=="210604" & year==2009

replace regioncode="210624" if regioncode=="210624" & year==2009

replace regioncode="210681" if regioncode=="210681" & year==2009

replace regioncode="210682" if regioncode=="210682" & year==2009

replace regioncode="210702" if regioncode=="210702" & year==2009

replace regioncode="210703" if regioncode=="210703" & year==2009

replace regioncode="210711" if regioncode=="210711" & year==2009

replace regioncode="210726" if regioncode=="210726" & year==2009

replace regioncode="210727" if regioncode=="210727" & year==2009

replace regioncode="210781" if regioncode=="210781" & year==2009

replace regioncode="210782" if regioncode=="210782" & year==2009

replace regioncode="210802" if regioncode=="210802" & year==2009

replace regioncode="210803" if regioncode=="210803" & year==2009

replace regioncode="210804" if regioncode=="210804" & year==2009

replace regioncode="210811" if regioncode=="210811" & year==2009

replace regioncode="210881" if regioncode=="210881" & year==2009

replace regioncode="210882" if regioncode=="210882" & year==2009

replace regioncode="210902" if regioncode=="210902" & year==2009

replace regioncode="210903" if regioncode=="210903" & year==2009

replace regioncode="210904" if regioncode=="210904" & year==2009

replace regioncode="210905" if regioncode=="210905" & year==2009

replace regioncode="210911" if regioncode=="210911" & year==2009

replace regioncode="210921" if regioncode=="210921" & year==2009

replace regioncode="210922" if regioncode=="210922" & year==2009

replace regioncode="211002" if regioncode=="211002" & year==2009

replace regioncode="211003" if regioncode=="211003" & year==2009

replace regioncode="211004" if regioncode=="211004" & year==2009

replace regioncode="211005" if regioncode=="211005" & year==2009

replace regioncode="211011" if regioncode=="211011" & year==2009

replace regioncode="211021" if regioncode=="211021" & year==2009

replace regioncode="211081" if regioncode=="211081" & year==2009

replace regioncode="211102" if regioncode=="211102" & year==2009

replace regioncode="211103" if regioncode=="211103" & year==2009

replace regioncode="211121" if regioncode=="211121" & year==2009

replace regioncode="211122" if regioncode=="211122" & year==2009

replace regioncode="211202" if regioncode=="211202" & year==2009

replace regioncode="211204" if regioncode=="211204" & year==2009

replace regioncode="211221" if regioncode=="211221" & year==2009

replace regioncode="211223" if regioncode=="211223" & year==2009

replace regioncode="211224" if regioncode=="211224" & year==2009

replace regioncode="211281" if regioncode=="211281" & year==2009

replace regioncode="211282" if regioncode=="211282" & year==2009

replace regioncode="211302" if regioncode=="211302" & year==2009

replace regioncode="211303" if regioncode=="211303" & year==2009

replace regioncode="211321" if regioncode=="211321" & year==2009

replace regioncode="211322" if regioncode=="211322" & year==2009

replace regioncode="211324" if regioncode=="211324" & year==2009

replace regioncode="211381" if regioncode=="211381" & year==2009

replace regioncode="211382" if regioncode=="211382" & year==2009

replace regioncode="211402" if regioncode=="211402" & year==2009

replace regioncode="211403" if regioncode=="211403" & year==2009

replace regioncode="211404" if regioncode=="211404" & year==2009

replace regioncode="211421" if regioncode=="211421" & year==2009

replace regioncode="211422" if regioncode=="211422" & year==2009

replace regioncode="211481" if regioncode=="211481" & year==2009

replace regioncode="220102" if regioncode=="220102" & year==2009

replace regioncode="220103" if regioncode=="220103" & year==2009

replace regioncode="220104" if regioncode=="220104" & year==2009

replace regioncode="220105" if regioncode=="220105" & year==2009

replace regioncode="220106" if regioncode=="220106" & year==2009

replace regioncode="220112" if regioncode=="220112" & year==2009

replace regioncode="220122" if regioncode=="220122" & year==2009

replace regioncode="220181" if regioncode=="220181" & year==2009

replace regioncode="220182" if regioncode=="220182" & year==2009

replace regioncode="220183" if regioncode=="220183" & year==2009

replace regioncode="220202" if regioncode=="220202" & year==2009

replace regioncode="220203" if regioncode=="220203" & year==2009

replace regioncode="220204" if regioncode=="220204" & year==2009

replace regioncode="220211" if regioncode=="220211" & year==2009

replace regioncode="220221" if regioncode=="220221" & year==2009

replace regioncode="220281" if regioncode=="220281" & year==2009

replace regioncode="220282" if regioncode=="220282" & year==2009

replace regioncode="220283" if regioncode=="220283" & year==2009

replace regioncode="220284" if regioncode=="220284" & year==2009

replace regioncode="220302" if regioncode=="220302" & year==2009

replace regioncode="220303" if regioncode=="220303" & year==2009

replace regioncode="220322" if regioncode=="220322" & year==2009

replace regioncode="220323" if regioncode=="220323" & year==2009

replace regioncode="220381" if regioncode=="220381" & year==2009

replace regioncode="220382" if regioncode=="220382" & year==2009

replace regioncode="220402" if regioncode=="220402" & year==2009

replace regioncode="220403" if regioncode=="220403" & year==2009

replace regioncode="220421" if regioncode=="220421" & year==2009

replace regioncode="220422" if regioncode=="220422" & year==2009

replace regioncode="220502" if regioncode=="220502" & year==2009

replace regioncode="220503" if regioncode=="220503" & year==2009

replace regioncode="220521" if regioncode=="220521" & year==2009

replace regioncode="220523" if regioncode=="220523" & year==2009

replace regioncode="220524" if regioncode=="220524" & year==2009

replace regioncode="220581" if regioncode=="220581" & year==2009

replace regioncode="220582" if regioncode=="220582" & year==2009

replace regioncode="220602" if regioncode=="220602" & year==2009

replace regioncode="220621" if regioncode=="220621" & year==2009

replace regioncode="220622" if regioncode=="220622" & year==2009

replace regioncode="220623" if regioncode=="220623" & year==2009

replace regioncode="220625" if regioncode=="220625" & year==2009

replace regioncode="220681" if regioncode=="220681" & year==2009

replace regioncode="220702" if regioncode=="220702" & year==2009

replace regioncode="220721" if regioncode=="220721" & year==2009

replace regioncode="220722" if regioncode=="220722" & year==2009

replace regioncode="220723" if regioncode=="220723" & year==2009

replace regioncode="220724" if regioncode=="220724" & year==2009

replace regioncode="220802" if regioncode=="220802" & year==2009

replace regioncode="220821" if regioncode=="220821" & year==2009

replace regioncode="220822" if regioncode=="220822" & year==2009

replace regioncode="220881" if regioncode=="220881" & year==2009

replace regioncode="220882" if regioncode=="220882" & year==2009

replace regioncode="222401" if regioncode=="222401" & year==2009

replace regioncode="222402" if regioncode=="222402" & year==2009

replace regioncode="222403" if regioncode=="222403" & year==2009

replace regioncode="222404" if regioncode=="222404" & year==2009

replace regioncode="222405" if regioncode=="222405" & year==2009

replace regioncode="222406" if regioncode=="222406" & year==2009

replace regioncode="222424" if regioncode=="222424" & year==2009

replace regioncode="222426" if regioncode=="222426" & year==2009

replace regioncode="230102" if regioncode=="230102" & year==2009

replace regioncode="230103" if regioncode=="230103" & year==2009

replace regioncode="230104" if regioncode=="230104" & year==2009

replace regioncode="230105" if regioncode=="230105" & year==2009

replace regioncode="230107" if regioncode=="230110" & year==2009

replace regioncode="230108" if regioncode=="230108" & year==2009

replace regioncode="230121" if regioncode=="230111" & year==2009

replace regioncode="230123" if regioncode=="230123" & year==2009

replace regioncode="230124" if regioncode=="230124" & year==2009

replace regioncode="230125" if regioncode=="230125" & year==2009

replace regioncode="230126" if regioncode=="230126" & year==2009

replace regioncode="230127" if regioncode=="230127" & year==2009

replace regioncode="230128" if regioncode=="230128" & year==2009

replace regioncode="230129" if regioncode=="230129" & year==2009

replace regioncode="230181" if regioncode=="230112" & year==2009

replace regioncode="230182" if regioncode=="230182" & year==2009

replace regioncode="230183" if regioncode=="230183" & year==2009

replace regioncode="230184" if regioncode=="230184" & year==2009

replace regioncode="230202" if regioncode=="230202" & year==2009

replace regioncode="230203" if regioncode=="230203" & year==2009

replace regioncode="230204" if regioncode=="230204" & year==2009

replace regioncode="230205" if regioncode=="230205" & year==2009

replace regioncode="230206" if regioncode=="230206" & year==2009

replace regioncode="230207" if regioncode=="230207" & year==2009

replace regioncode="230208" if regioncode=="230208" & year==2009

replace regioncode="230221" if regioncode=="230221" & year==2009

replace regioncode="230223" if regioncode=="230223" & year==2009

replace regioncode="230224" if regioncode=="230224" & year==2009

replace regioncode="230225" if regioncode=="230225" & year==2009

replace regioncode="230227" if regioncode=="230227" & year==2009

replace regioncode="230229" if regioncode=="230229" & year==2009

replace regioncode="230230" if regioncode=="230230" & year==2009

replace regioncode="230231" if regioncode=="230231" & year==2009

replace regioncode="230281" if regioncode=="230281" & year==2009

replace regioncode="230302" if regioncode=="230302" & year==2009

replace regioncode="230303" if regioncode=="230303" & year==2009

replace regioncode="230304" if regioncode=="230304" & year==2009

replace regioncode="230305" if regioncode=="230305" & year==2009

replace regioncode="230306" if regioncode=="230306" & year==2009

replace regioncode="230307" if regioncode=="230307" & year==2009

replace regioncode="230321" if regioncode=="230321" & year==2009

replace regioncode="230381" if regioncode=="230381" & year==2009

replace regioncode="230382" if regioncode=="230382" & year==2009

replace regioncode="230402" if regioncode=="230402" & year==2009

replace regioncode="230403" if regioncode=="230403" & year==2009

replace regioncode="230404" if regioncode=="230404" & year==2009

replace regioncode="230405" if regioncode=="230405" & year==2009

replace regioncode="230406" if regioncode=="230406" & year==2009

replace regioncode="230407" if regioncode=="230407" & year==2009

replace regioncode="230421" if regioncode=="230421" & year==2009

replace regioncode="230422" if regioncode=="230422" & year==2009

replace regioncode="230502" if regioncode=="230502" & year==2009

replace regioncode="230503" if regioncode=="230503" & year==2009

replace regioncode="230505" if regioncode=="230505" & year==2009

replace regioncode="230506" if regioncode=="230506" & year==2009

replace regioncode="230521" if regioncode=="230521" & year==2009

replace regioncode="230522" if regioncode=="230522" & year==2009

replace regioncode="230523" if regioncode=="230523" & year==2009

replace regioncode="230524" if regioncode=="230524" & year==2009

replace regioncode="230602" if regioncode=="230602" & year==2009

replace regioncode="230603" if regioncode=="230603" & year==2009

replace regioncode="230604" if regioncode=="230604" & year==2009

replace regioncode="230605" if regioncode=="230605" & year==2009

replace regioncode="230606" if regioncode=="230606" & year==2009

replace regioncode="230621" if regioncode=="230621" & year==2009

replace regioncode="230622" if regioncode=="230622" & year==2009

replace regioncode="230623" if regioncode=="230623" & year==2009

replace regioncode="230624" if regioncode=="230624" & year==2009

replace regioncode="230702" if regioncode=="230702" & year==2009

replace regioncode="230703" if regioncode=="230703" & year==2009

replace regioncode="230704" if regioncode=="230704" & year==2009

replace regioncode="230705" if regioncode=="230705" & year==2009

replace regioncode="230706" if regioncode=="230706" & year==2009

replace regioncode="230707" if regioncode=="230707" & year==2009

replace regioncode="230708" if regioncode=="230708" & year==2009

replace regioncode="230709" if regioncode=="230709" & year==2009

replace regioncode="230710" if regioncode=="230710" & year==2009

replace regioncode="230711" if regioncode=="230711" & year==2009

replace regioncode="230712" if regioncode=="230712" & year==2009

replace regioncode="230713" if regioncode=="230713" & year==2009

replace regioncode="230714" if regioncode=="230714" & year==2009

replace regioncode="230715" if regioncode=="230715" & year==2009

replace regioncode="230716" if regioncode=="230716" & year==2009

replace regioncode="230722" if regioncode=="230722" & year==2009

replace regioncode="230781" if regioncode=="230781" & year==2009

replace regioncode="230802" if regioncode=="230802" & year==2009

replace regioncode="230803" if regioncode=="230803" & year==2009

replace regioncode="230804" if regioncode=="230804" & year==2009

replace regioncode="230805" if regioncode=="230805" & year==2009

replace regioncode="230811" if regioncode=="230811" & year==2009

replace regioncode="230822" if regioncode=="230822" & year==2009

replace regioncode="230826" if regioncode=="230826" & year==2009

replace regioncode="230828" if regioncode=="230828" & year==2009

replace regioncode="230833" if regioncode=="230833" & year==2009

replace regioncode="230881" if regioncode=="230881" & year==2009

replace regioncode="230882" if regioncode=="230882" & year==2009

replace regioncode="230902" if regioncode=="230902" & year==2009

replace regioncode="230903" if regioncode=="230903" & year==2009

replace regioncode="230904" if regioncode=="230904" & year==2009

replace regioncode="230921" if regioncode=="230921" & year==2009

replace regioncode="231002" if regioncode=="231002" & year==2009

replace regioncode="231003" if regioncode=="231003" & year==2009

replace regioncode="231004" if regioncode=="231004" & year==2009

replace regioncode="231005" if regioncode=="231005" & year==2009

replace regioncode="231024" if regioncode=="231024" & year==2009

replace regioncode="231025" if regioncode=="231025" & year==2009

replace regioncode="231081" if regioncode=="231081" & year==2009

replace regioncode="231083" if regioncode=="231083" & year==2009

replace regioncode="231084" if regioncode=="231084" & year==2009

replace regioncode="231085" if regioncode=="231085" & year==2009

replace regioncode="231102" if regioncode=="231102" & year==2009

replace regioncode="231121" if regioncode=="231121" & year==2009

replace regioncode="231123" if regioncode=="231123" & year==2009

replace regioncode="231124" if regioncode=="231124" & year==2009

replace regioncode="231181" if regioncode=="231181" & year==2009

replace regioncode="231182" if regioncode=="231182" & year==2009

replace regioncode="231202" if regioncode=="231202" & year==2009

replace regioncode="231221" if regioncode=="231221" & year==2009

replace regioncode="231222" if regioncode=="231222" & year==2009

replace regioncode="231223" if regioncode=="231223" & year==2009

replace regioncode="231224" if regioncode=="231224" & year==2009

replace regioncode="231225" if regioncode=="231225" & year==2009

replace regioncode="231226" if regioncode=="231226" & year==2009

replace regioncode="231281" if regioncode=="231281" & year==2009

replace regioncode="231282" if regioncode=="231282" & year==2009

replace regioncode="231283" if regioncode=="231283" & year==2009

replace regioncode="232701" if regioncode=="232701" & year==2009

replace regioncode="232702" if regioncode=="232702" & year==2009

replace regioncode="232703" if regioncode=="232703" & year==2009

replace regioncode="232704" if regioncode=="232704" & year==2009

replace regioncode="232721" if regioncode=="232721" & year==2009

replace regioncode="232722" if regioncode=="232722" & year==2009

replace regioncode="232723" if regioncode=="232723" & year==2009

replace regioncode="310101" if regioncode=="310101" & year==2009

replace regioncode="310103" if regioncode=="310103" & year==2009

replace regioncode="310104" if regioncode=="310104" & year==2009

replace regioncode="310105" if regioncode=="310105" & year==2009

replace regioncode="310106" if regioncode=="310106" & year==2009

replace regioncode="310107" if regioncode=="310107" & year==2009

replace regioncode="310108" if regioncode=="310108" & year==2009

replace regioncode="310109" if regioncode=="310109" & year==2009

replace regioncode="310110" if regioncode=="310110" & year==2009

replace regioncode="310112" if regioncode=="310112" & year==2009

replace regioncode="310113" if regioncode=="310113" & year==2009

replace regioncode="310114" if regioncode=="310114" & year==2009

replace regioncode="310115" if regioncode=="310115" & year==2009

replace regioncode="310116" if regioncode=="310116" & year==2009

replace regioncode="310117" if regioncode=="310117" & year==2009

replace regioncode="310118" if regioncode=="310118" & year==2009

replace regioncode="310119" if regioncode=="310119" & year==2009

replace regioncode="310120" if regioncode=="310120" & year==2009

replace regioncode="310230" if regioncode=="310230" & year==2009

replace regioncode="320102" if regioncode=="320102" & year==2009

replace regioncode="320103" if regioncode=="320103" & year==2009

replace regioncode="320104" if regioncode=="320104" & year==2009

replace regioncode="320105" if regioncode=="320105" & year==2009

replace regioncode="320106" if regioncode=="320106" & year==2009

replace regioncode="320107" if regioncode=="320107" & year==2009

replace regioncode="320111" if regioncode=="320111" & year==2009

replace regioncode="320113" if regioncode=="320113" & year==2009

replace regioncode="320114" if regioncode=="320114" & year==2009

replace regioncode="320115" if regioncode=="320115" & year==2009

replace regioncode="320116" if regioncode=="320116" & year==2009

replace regioncode="320124" if regioncode=="320124" & year==2009

replace regioncode="320125" if regioncode=="320125" & year==2009

replace regioncode="320202" if regioncode=="320202" & year==2009

replace regioncode="320203" if regioncode=="320203" & year==2009

replace regioncode="320204" if regioncode=="320204" & year==2009

replace regioncode="320205" if regioncode=="320205" & year==2009

replace regioncode="320211" if regioncode=="320211" & year==2009

replace regioncode="320281" if regioncode=="320281" & year==2009

replace regioncode="320282" if regioncode=="320282" & year==2009

replace regioncode="320302" if regioncode=="320302" & year==2009

replace regioncode="320303" if regioncode=="320303" & year==2009

replace regioncode="320304" if regioncode=="320304" & year==2009

replace regioncode="320305" if regioncode=="320305" & year==2009

replace regioncode="320311" if regioncode=="320311" & year==2009

replace regioncode="320321" if regioncode=="320321" & year==2009

replace regioncode="320322" if regioncode=="320322" & year==2009

replace regioncode="320323" if regioncode=="320323" & year==2009

replace regioncode="320324" if regioncode=="320324" & year==2009

replace regioncode="320381" if regioncode=="320381" & year==2009

replace regioncode="320382" if regioncode=="320382" & year==2009

replace regioncode="320402" if regioncode=="320402" & year==2009

replace regioncode="320404" if regioncode=="320404" & year==2009

replace regioncode="320405" if regioncode=="320405" & year==2009

replace regioncode="320411" if regioncode=="320411" & year==2009

replace regioncode="320412" if regioncode=="320412" & year==2009

replace regioncode="320481" if regioncode=="320481" & year==2009

replace regioncode="320482" if regioncode=="320482" & year==2009

replace regioncode="320502" if regioncode=="320502" & year==2009

replace regioncode="320503" if regioncode=="320503" & year==2009

replace regioncode="320504" if regioncode=="320504" & year==2009

replace regioncode="320505" if regioncode=="320505" & year==2009

replace regioncode="320581" if regioncode=="320581" & year==2009

replace regioncode="320582" if regioncode=="320582" & year==2009

replace regioncode="320583" if regioncode=="320583" & year==2009

replace regioncode="320584" if regioncode=="320584" & year==2009

replace regioncode="320585" if regioncode=="320585" & year==2009

replace regioncode="320586" if regioncode=="320586" & year==2009

replace regioncode="320602" if regioncode=="320602" & year==2009

replace regioncode="320611" if regioncode=="320611" & year==2009

replace regioncode="320621" if regioncode=="320621" & year==2009

replace regioncode="320623" if regioncode=="320623" & year==2009

replace regioncode="320681" if regioncode=="320681" & year==2009

replace regioncode="320682" if regioncode=="320682" & year==2009

replace regioncode="320683" if regioncode=="320683" & year==2009

replace regioncode="320684" if regioncode=="320684" & year==2009

replace regioncode="320703" if regioncode=="320703" & year==2009

replace regioncode="320705" if regioncode=="320705" & year==2009

replace regioncode="320706" if regioncode=="320706" & year==2009

replace regioncode="320721" if regioncode=="320721" & year==2009

replace regioncode="320722" if regioncode=="320722" & year==2009

replace regioncode="320723" if regioncode=="320723" & year==2009

replace regioncode="320724" if regioncode=="320724" & year==2009

replace regioncode="320802" if regioncode=="320802" & year==2009

replace regioncode="320803" if regioncode=="320803" & year==2009

replace regioncode="320804" if regioncode=="320804" & year==2009

replace regioncode="320811" if regioncode=="320811" & year==2009

replace regioncode="320826" if regioncode=="320826" & year==2009

replace regioncode="320829" if regioncode=="320829" & year==2009

replace regioncode="320830" if regioncode=="320830" & year==2009

replace regioncode="320831" if regioncode=="320831" & year==2009

replace regioncode="320902" if regioncode=="320902" & year==2009

replace regioncode="320903" if regioncode=="320903" & year==2009

replace regioncode="320921" if regioncode=="320921" & year==2009

replace regioncode="320922" if regioncode=="320922" & year==2009

replace regioncode="320923" if regioncode=="320923" & year==2009

replace regioncode="320924" if regioncode=="320924" & year==2009

replace regioncode="320925" if regioncode=="320925" & year==2009

replace regioncode="320981" if regioncode=="320981" & year==2009

replace regioncode="320982" if regioncode=="320982" & year==2009

replace regioncode="321002" if regioncode=="321002" & year==2009

replace regioncode="321003" if regioncode=="321003" & year==2009

replace regioncode="321011" if regioncode=="321011" & year==2009

replace regioncode="321023" if regioncode=="321023" & year==2009

replace regioncode="321081" if regioncode=="321081" & year==2009

replace regioncode="321084" if regioncode=="321084" & year==2009

replace regioncode="321088" if regioncode=="321088" & year==2009

replace regioncode="321102" if regioncode=="321102" & year==2009

replace regioncode="321111" if regioncode=="321111" & year==2009

replace regioncode="321112" if regioncode=="321112" & year==2009

replace regioncode="321181" if regioncode=="321181" & year==2009

replace regioncode="321182" if regioncode=="321182" & year==2009

replace regioncode="321183" if regioncode=="321183" & year==2009

replace regioncode="321202" if regioncode=="321202" & year==2009

replace regioncode="321203" if regioncode=="321203" & year==2009

replace regioncode="321281" if regioncode=="321281" & year==2009

replace regioncode="321282" if regioncode=="321282" & year==2009

replace regioncode="321283" if regioncode=="321283" & year==2009

replace regioncode="321284" if regioncode=="321284" & year==2009

replace regioncode="321302" if regioncode=="321302" & year==2009

replace regioncode="321321" if regioncode=="321311" & year==2009

replace regioncode="321322" if regioncode=="321322" & year==2009

replace regioncode="321323" if regioncode=="321323" & year==2009

replace regioncode="321324" if regioncode=="321324" & year==2009

replace regioncode="330102" if regioncode=="330102" & year==2009

replace regioncode="330103" if regioncode=="330103" & year==2009

replace regioncode="330104" if regioncode=="330104" & year==2009

replace regioncode="330105" if regioncode=="330105" & year==2009

replace regioncode="330106" if regioncode=="330106" & year==2009

replace regioncode="330108" if regioncode=="330108" & year==2009

replace regioncode="330109" if regioncode=="330109" & year==2009

replace regioncode="330110" if regioncode=="330110" & year==2009

replace regioncode="330122" if regioncode=="330122" & year==2009

replace regioncode="330127" if regioncode=="330127" & year==2009

replace regioncode="330182" if regioncode=="330182" & year==2009

replace regioncode="330183" if regioncode=="330183" & year==2009

replace regioncode="330185" if regioncode=="330185" & year==2009

replace regioncode="330203" if regioncode=="330203" & year==2009

replace regioncode="330204" if regioncode=="330204" & year==2009

replace regioncode="330205" if regioncode=="330205" & year==2009

replace regioncode="330206" if regioncode=="330206" & year==2009

replace regioncode="330211" if regioncode=="330211" & year==2009

replace regioncode="330212" if regioncode=="330212" & year==2009

replace regioncode="330225" if regioncode=="330225" & year==2009

replace regioncode="330226" if regioncode=="330226" & year==2009

replace regioncode="330281" if regioncode=="330281" & year==2009

replace regioncode="330282" if regioncode=="330282" & year==2009

replace regioncode="330283" if regioncode=="330283" & year==2009

replace regioncode="330302" if regioncode=="330302" & year==2009

replace regioncode="330303" if regioncode=="330303" & year==2009

replace regioncode="330304" if regioncode=="330304" & year==2009

replace regioncode="330322" if regioncode=="330322" & year==2009

replace regioncode="330324" if regioncode=="330324" & year==2009

replace regioncode="330326" if regioncode=="330326" & year==2009

replace regioncode="330327" if regioncode=="330327" & year==2009

replace regioncode="330328" if regioncode=="330328" & year==2009

replace regioncode="330329" if regioncode=="330329" & year==2009

replace regioncode="330381" if regioncode=="330381" & year==2009

replace regioncode="330382" if regioncode=="330382" & year==2009

replace regioncode="330402" if regioncode=="330402" & year==2009

replace regioncode="330411" if regioncode=="330411" & year==2009

replace regioncode="330421" if regioncode=="330421" & year==2009

replace regioncode="330424" if regioncode=="330424" & year==2009

replace regioncode="330481" if regioncode=="330481" & year==2009

replace regioncode="330482" if regioncode=="330482" & year==2009

replace regioncode="330483" if regioncode=="330483" & year==2009

replace regioncode="330501" if regioncode=="330501" & year==2009

replace regioncode="330521" if regioncode=="330521" & year==2009

replace regioncode="330522" if regioncode=="330522" & year==2009

replace regioncode="330523" if regioncode=="330523" & year==2009

replace regioncode="330602" if regioncode=="330602" & year==2009

replace regioncode="330621" if regioncode=="330621" & year==2009

replace regioncode="330624" if regioncode=="330624" & year==2009

replace regioncode="330681" if regioncode=="330681" & year==2009

replace regioncode="330682" if regioncode=="330682" & year==2009

replace regioncode="330683" if regioncode=="330683" & year==2009

replace regioncode="330702" if regioncode=="330702" & year==2009

replace regioncode="330703" if regioncode=="330703" & year==2009

replace regioncode="330723" if regioncode=="330723" & year==2009

replace regioncode="330726" if regioncode=="330726" & year==2009

replace regioncode="330727" if regioncode=="330727" & year==2009

replace regioncode="330781" if regioncode=="330781" & year==2009

replace regioncode="330782" if regioncode=="330782" & year==2009

replace regioncode="330783" if regioncode=="330783" & year==2009

replace regioncode="330784" if regioncode=="330784" & year==2009

replace regioncode="330802" if regioncode=="330802" & year==2009

replace regioncode="330803" if regioncode=="330803" & year==2009

replace regioncode="330822" if regioncode=="330822" & year==2009

replace regioncode="330824" if regioncode=="330824" & year==2009

replace regioncode="330825" if regioncode=="330825" & year==2009

replace regioncode="330881" if regioncode=="330881" & year==2009

replace regioncode="330902" if regioncode=="330902" & year==2009

replace regioncode="330903" if regioncode=="330903" & year==2009

replace regioncode="330921" if regioncode=="330921" & year==2009

replace regioncode="330922" if regioncode=="330922" & year==2009

replace regioncode="331002" if regioncode=="331002" & year==2009

replace regioncode="331003" if regioncode=="331003" & year==2009

replace regioncode="331004" if regioncode=="331004" & year==2009

replace regioncode="331021" if regioncode=="331021" & year==2009

replace regioncode="331022" if regioncode=="331022" & year==2009

replace regioncode="331023" if regioncode=="331023" & year==2009

replace regioncode="331024" if regioncode=="331024" & year==2009

replace regioncode="331081" if regioncode=="331081" & year==2009

replace regioncode="331082" if regioncode=="331082" & year==2009

replace regioncode="331102" if regioncode=="331102" & year==2009

replace regioncode="331121" if regioncode=="331121" & year==2009

replace regioncode="331122" if regioncode=="331122" & year==2009

replace regioncode="331123" if regioncode=="331123" & year==2009

replace regioncode="331124" if regioncode=="331124" & year==2009

replace regioncode="331125" if regioncode=="331125" & year==2009

replace regioncode="331126" if regioncode=="331126" & year==2009

replace regioncode="331127" if regioncode=="331127" & year==2009

replace regioncode="331181" if regioncode=="331181" & year==2009

replace regioncode="340102" if regioncode=="340102" & year==2009

replace regioncode="340103" if regioncode=="340103" & year==2009

replace regioncode="340104" if regioncode=="340104" & year==2009

replace regioncode="340111" if regioncode=="340111" & year==2009

replace regioncode="340121" if regioncode=="340121" & year==2009

replace regioncode="340122" if regioncode=="340122" & year==2009

replace regioncode="340123" if regioncode=="340123" & year==2009

replace regioncode="340202" if regioncode=="340202" & year==2009

replace regioncode="340203" if regioncode=="340203" & year==2009

replace regioncode="340204" if regioncode=="340204" & year==2009

replace regioncode="340207" if regioncode=="340207" & year==2009

replace regioncode="340221" if regioncode=="340221" & year==2009

replace regioncode="340222" if regioncode=="340222" & year==2009

replace regioncode="340223" if regioncode=="340223" & year==2009

replace regioncode="340302" if regioncode=="340302" & year==2009

replace regioncode="340303" if regioncode=="340303" & year==2009

replace regioncode="340304" if regioncode=="340304" & year==2009

replace regioncode="340311" if regioncode=="340311" & year==2009

replace regioncode="340321" if regioncode=="340321" & year==2009

replace regioncode="340322" if regioncode=="340322" & year==2009

replace regioncode="340323" if regioncode=="340323" & year==2009

replace regioncode="340402" if regioncode=="340402" & year==2009

replace regioncode="340403" if regioncode=="340403" & year==2009

replace regioncode="340404" if regioncode=="340404" & year==2009

replace regioncode="340405" if regioncode=="340405" & year==2009

replace regioncode="340406" if regioncode=="340406" & year==2009

replace regioncode="340421" if regioncode=="340421" & year==2009

replace regioncode="340502" if regioncode=="340502" & year==2009

replace regioncode="340503" if regioncode=="340503" & year==2009

replace regioncode="340504" if regioncode=="340504" & year==2009

replace regioncode="340521" if regioncode=="340521" & year==2009

replace regioncode="340602" if regioncode=="340602" & year==2009

replace regioncode="340603" if regioncode=="340603" & year==2009

replace regioncode="340604" if regioncode=="340604" & year==2009

replace regioncode="340621" if regioncode=="340621" & year==2009

replace regioncode="340702" if regioncode=="340702" & year==2009

replace regioncode="340703" if regioncode=="340703" & year==2009

replace regioncode="340711" if regioncode=="340711" & year==2009

replace regioncode="340721" if regioncode=="340721" & year==2009

replace regioncode="340802" if regioncode=="340802" & year==2009

replace regioncode="340803" if regioncode=="340803" & year==2009

replace regioncode="340811" if regioncode=="340811" & year==2009

replace regioncode="340822" if regioncode=="340822" & year==2009

replace regioncode="340823" if regioncode=="340823" & year==2009

replace regioncode="340824" if regioncode=="340824" & year==2009

replace regioncode="340825" if regioncode=="340825" & year==2009

replace regioncode="340826" if regioncode=="340826" & year==2009

replace regioncode="340827" if regioncode=="340827" & year==2009

replace regioncode="340828" if regioncode=="340828" & year==2009

replace regioncode="340881" if regioncode=="340881" & year==2009

replace regioncode="341002" if regioncode=="341002" & year==2009

replace regioncode="341003" if regioncode=="341003" & year==2009

replace regioncode="341004" if regioncode=="341004" & year==2009

replace regioncode="341021" if regioncode=="341021" & year==2009

replace regioncode="341022" if regioncode=="341022" & year==2009

replace regioncode="341023" if regioncode=="341023" & year==2009

replace regioncode="341024" if regioncode=="341024" & year==2009

replace regioncode="341102" if regioncode=="341102" & year==2009

replace regioncode="341103" if regioncode=="341103" & year==2009

replace regioncode="341122" if regioncode=="341122" & year==2009

replace regioncode="341124" if regioncode=="341124" & year==2009

replace regioncode="341125" if regioncode=="341125" & year==2009

replace regioncode="341126" if regioncode=="341126" & year==2009

replace regioncode="341181" if regioncode=="341181" & year==2009

replace regioncode="341182" if regioncode=="341182" & year==2009

replace regioncode="341202" if regioncode=="341202" & year==2009

replace regioncode="341203" if regioncode=="341203" & year==2009

replace regioncode="341204" if regioncode=="341204" & year==2009

replace regioncode="341221" if regioncode=="341221" & year==2009

replace regioncode="341222" if regioncode=="341222" & year==2009

replace regioncode="341621" if regioncode=="341621" & year==2009

replace regioncode="341622" if regioncode=="341622" & year==2009

replace regioncode="341225" if regioncode=="341225" & year==2009

replace regioncode="341226" if regioncode=="341226" & year==2009

replace regioncode="341623" if regioncode=="341623" & year==2009

replace regioncode="341282" if regioncode=="341282" & year==2009

replace regioncode="341302" if regioncode=="341302" & year==2009

replace regioncode="341321" if regioncode=="341321" & year==2009

replace regioncode="341322" if regioncode=="341322" & year==2009

replace regioncode="341323" if regioncode=="341323" & year==2009

replace regioncode="341324" if regioncode=="341324" & year==2009

replace regioncode="341402" if regioncode=="341402" & year==2009

replace regioncode="341421" if regioncode=="341421" & year==2009

replace regioncode="341422" if regioncode=="341422" & year==2009

replace regioncode="341423" if regioncode=="341423" & year==2009

replace regioncode="341424" if regioncode=="341424" & year==2009

replace regioncode="341521" if regioncode=="341521" & year==2009

replace regioncode="341522" if regioncode=="341522" & year==2009

replace regioncode="341523" if regioncode=="341523" & year==2009

replace regioncode="341524" if regioncode=="341524" & year==2009

replace regioncode="341525" if regioncode=="341525" & year==2009

replace regioncode="341802" if regioncode=="341802" & year==2009

replace regioncode="341881" if regioncode=="341881" & year==2009

replace regioncode="341821" if regioncode=="341821" & year==2009

replace regioncode="341822" if regioncode=="341822" & year==2009

replace regioncode="341823" if regioncode=="341823" & year==2009

replace regioncode="341825" if regioncode=="341825" & year==2009

replace regioncode="341824" if regioncode=="341824" & year==2009

replace regioncode="341702" if regioncode=="341702" & year==2009

replace regioncode="341721" if regioncode=="341721" & year==2009

replace regioncode="341722" if regioncode=="341722" & year==2009

replace regioncode="341723" if regioncode=="341723" & year==2009

replace regioncode="341502" if regioncode=="341502" & year==2009

replace regioncode="341602" if regioncode=="341602" & year==2009

replace regioncode="350102" if regioncode=="350102" & year==2009

replace regioncode="350103" if regioncode=="350103" & year==2009

replace regioncode="350104" if regioncode=="350104" & year==2009

replace regioncode="350105" if regioncode=="350105" & year==2009

replace regioncode="350111" if regioncode=="350111" & year==2009

replace regioncode="350121" if regioncode=="350121" & year==2009

replace regioncode="350122" if regioncode=="350122" & year==2009

replace regioncode="350123" if regioncode=="350123" & year==2009

replace regioncode="350124" if regioncode=="350124" & year==2009

replace regioncode="350125" if regioncode=="350125" & year==2009

replace regioncode="350128" if regioncode=="350128" & year==2009

replace regioncode="350181" if regioncode=="350181" & year==2009

replace regioncode="350182" if regioncode=="350182" & year==2009

replace regioncode="350203" if regioncode=="350203" & year==2009

replace regioncode="350205" if regioncode=="350205" & year==2009

replace regioncode="350206" if regioncode=="350206" & year==2009

replace regioncode="350211" if regioncode=="350211" & year==2009

replace regioncode="350212" if regioncode=="350212" & year==2009

replace regioncode="350213" if regioncode=="350213" & year==2009

replace regioncode="350302" if regioncode=="350302" & year==2009

replace regioncode="350303" if regioncode=="350303" & year==2009

replace regioncode="350321" if regioncode=="350321" & year==2009

replace regioncode="350322" if regioncode=="350322" & year==2009

replace regioncode="350402" if regioncode=="350402" & year==2009

replace regioncode="350403" if regioncode=="350403" & year==2009

replace regioncode="350421" if regioncode=="350421" & year==2009

replace regioncode="350423" if regioncode=="350423" & year==2009

replace regioncode="350424" if regioncode=="350424" & year==2009

replace regioncode="350425" if regioncode=="350425" & year==2009

replace regioncode="350426" if regioncode=="350426" & year==2009

replace regioncode="350427" if regioncode=="350427" & year==2009

replace regioncode="350428" if regioncode=="350428" & year==2009

replace regioncode="350429" if regioncode=="350429" & year==2009

replace regioncode="350430" if regioncode=="350430" & year==2009

replace regioncode="350481" if regioncode=="350481" & year==2009

replace regioncode="350502" if regioncode=="350502" & year==2009

replace regioncode="350503" if regioncode=="350503" & year==2009

replace regioncode="350504" if regioncode=="350504" & year==2009

replace regioncode="350505" if regioncode=="350505" & year==2009

replace regioncode="350521" if regioncode=="350521" & year==2009

replace regioncode="350524" if regioncode=="350524" & year==2009

replace regioncode="350525" if regioncode=="350525" & year==2009

replace regioncode="350526" if regioncode=="350526" & year==2009

replace regioncode="350581" if regioncode=="350581" & year==2009

replace regioncode="350582" if regioncode=="350582" & year==2009

replace regioncode="350583" if regioncode=="350583" & year==2009

replace regioncode="350602" if regioncode=="350602" & year==2009

replace regioncode="350603" if regioncode=="350603" & year==2009

replace regioncode="350622" if regioncode=="350622" & year==2009

replace regioncode="350623" if regioncode=="350623" & year==2009

replace regioncode="350624" if regioncode=="350624" & year==2009

replace regioncode="350625" if regioncode=="350625" & year==2009

replace regioncode="350626" if regioncode=="350626" & year==2009

replace regioncode="350627" if regioncode=="350627" & year==2009

replace regioncode="350628" if regioncode=="350628" & year==2009

replace regioncode="350629" if regioncode=="350629" & year==2009

replace regioncode="350681" if regioncode=="350681" & year==2009

replace regioncode="350702" if regioncode=="350702" & year==2009

replace regioncode="350721" if regioncode=="350721" & year==2009

replace regioncode="350722" if regioncode=="350722" & year==2009

replace regioncode="350723" if regioncode=="350723" & year==2009

replace regioncode="350724" if regioncode=="350724" & year==2009

replace regioncode="350725" if regioncode=="350725" & year==2009

replace regioncode="350781" if regioncode=="350781" & year==2009

replace regioncode="350782" if regioncode=="350782" & year==2009

replace regioncode="350783" if regioncode=="350783" & year==2009

replace regioncode="350784" if regioncode=="350784" & year==2009

replace regioncode="350802" if regioncode=="350802" & year==2009

replace regioncode="350821" if regioncode=="350821" & year==2009

replace regioncode="350822" if regioncode=="350822" & year==2009

replace regioncode="350823" if regioncode=="350823" & year==2009

replace regioncode="350824" if regioncode=="350824" & year==2009

replace regioncode="350825" if regioncode=="350825" & year==2009

replace regioncode="350881" if regioncode=="350881" & year==2009

replace regioncode="350902" if regioncode=="350902" & year==2009

replace regioncode="350981" if regioncode=="350981" & year==2009

replace regioncode="350982" if regioncode=="350982" & year==2009

replace regioncode="350921" if regioncode=="350921" & year==2009

replace regioncode="350922" if regioncode=="350922" & year==2009

replace regioncode="350923" if regioncode=="350923" & year==2009

replace regioncode="350924" if regioncode=="350924" & year==2009

replace regioncode="350925" if regioncode=="350925" & year==2009

replace regioncode="350926" if regioncode=="350926" & year==2009

replace regioncode="360102" if regioncode=="360102" & year==2009

replace regioncode="360103" if regioncode=="360103" & year==2009

replace regioncode="360104" if regioncode=="360104" & year==2009

replace regioncode="360105" if regioncode=="360105" & year==2009

replace regioncode="360111" if regioncode=="360111" & year==2009

replace regioncode="360121" if regioncode=="360121" & year==2009

replace regioncode="360122" if regioncode=="360122" & year==2009

replace regioncode="360123" if regioncode=="360123" & year==2009

replace regioncode="360124" if regioncode=="360124" & year==2009

replace regioncode="360202" if regioncode=="360202" & year==2009

replace regioncode="360203" if regioncode=="360203" & year==2009

replace regioncode="360222" if regioncode=="360222" & year==2009

replace regioncode="360281" if regioncode=="360281" & year==2009

replace regioncode="360302" if regioncode=="360302" & year==2009

replace regioncode="360313" if regioncode=="360313" & year==2009

replace regioncode="360321" if regioncode=="360321" & year==2009

replace regioncode="360322" if regioncode=="360322" & year==2009

replace regioncode="360323" if regioncode=="360323" & year==2009

replace regioncode="360402" if regioncode=="360402" & year==2009

replace regioncode="360403" if regioncode=="360403" & year==2009

replace regioncode="360421" if regioncode=="360421" & year==2009

replace regioncode="360423" if regioncode=="360423" & year==2009

replace regioncode="360424" if regioncode=="360424" & year==2009

replace regioncode="360425" if regioncode=="360425" & year==2009

replace regioncode="360426" if regioncode=="360426" & year==2009

replace regioncode="360427" if regioncode=="360427" & year==2009

replace regioncode="360428" if regioncode=="360428" & year==2009

replace regioncode="360429" if regioncode=="360429" & year==2009

replace regioncode="360430" if regioncode=="360430" & year==2009

replace regioncode="360481" if regioncode=="360481" & year==2009

replace regioncode="360502" if regioncode=="360502" & year==2009

replace regioncode="360521" if regioncode=="360521" & year==2009

replace regioncode="360602" if regioncode=="360602" & year==2009

replace regioncode="360622" if regioncode=="360622" & year==2009

replace regioncode="360681" if regioncode=="360681" & year==2009

replace regioncode="360702" if regioncode=="360702" & year==2009

replace regioncode="360721" if regioncode=="360721" & year==2009

replace regioncode="360722" if regioncode=="360722" & year==2009

replace regioncode="360723" if regioncode=="360723" & year==2009

replace regioncode="360724" if regioncode=="360724" & year==2009

replace regioncode="360725" if regioncode=="360725" & year==2009

replace regioncode="360726" if regioncode=="360726" & year==2009

replace regioncode="360727" if regioncode=="360727" & year==2009

replace regioncode="360728" if regioncode=="360728" & year==2009

replace regioncode="360729" if regioncode=="360729" & year==2009

replace regioncode="360730" if regioncode=="360730" & year==2009

replace regioncode="360731" if regioncode=="360731" & year==2009

replace regioncode="360732" if regioncode=="360732" & year==2009

replace regioncode="360733" if regioncode=="360733" & year==2009

replace regioncode="360734" if regioncode=="360734" & year==2009

replace regioncode="360735" if regioncode=="360735" & year==2009

replace regioncode="360781" if regioncode=="360781" & year==2009

replace regioncode="360782" if regioncode=="360782" & year==2009

replace regioncode="360802" if regioncode=="360802" & year==2009

replace regioncode="360821" if regioncode=="360821" & year==2009

replace regioncode="360822" if regioncode=="360822" & year==2009

replace regioncode="360823" if regioncode=="360823" & year==2009

replace regioncode="360824" if regioncode=="360824" & year==2009

replace regioncode="360825" if regioncode=="360825" & year==2009

replace regioncode="360826" if regioncode=="360826" & year==2009

replace regioncode="360827" if regioncode=="360827" & year==2009

replace regioncode="360828" if regioncode=="360828" & year==2009

replace regioncode="360829" if regioncode=="360829" & year==2009

replace regioncode="360830" if regioncode=="360830" & year==2009

replace regioncode="360881" if regioncode=="360881" & year==2009

replace regioncode="360902" if regioncode=="360902" & year==2009

replace regioncode="360921" if regioncode=="360921" & year==2009

replace regioncode="360922" if regioncode=="360922" & year==2009

replace regioncode="360923" if regioncode=="360923" & year==2009

replace regioncode="360924" if regioncode=="360924" & year==2009

replace regioncode="360925" if regioncode=="360925" & year==2009

replace regioncode="360926" if regioncode=="360926" & year==2009

replace regioncode="360981" if regioncode=="360981" & year==2009

replace regioncode="360982" if regioncode=="360982" & year==2009

replace regioncode="360983" if regioncode=="360983" & year==2009

replace regioncode="361002" if regioncode=="361002" & year==2009

replace regioncode="361021" if regioncode=="361021" & year==2009

replace regioncode="361022" if regioncode=="361022" & year==2009

replace regioncode="361023" if regioncode=="361023" & year==2009

replace regioncode="361024" if regioncode=="361024" & year==2009

replace regioncode="361025" if regioncode=="361025" & year==2009

replace regioncode="361026" if regioncode=="361026" & year==2009

replace regioncode="361027" if regioncode=="361027" & year==2009

replace regioncode="361028" if regioncode=="361028" & year==2009

replace regioncode="361029" if regioncode=="361029" & year==2009

replace regioncode="361030" if regioncode=="361030" & year==2009

replace regioncode="361102" if regioncode=="361102" & year==2009

replace regioncode="361121" if regioncode=="361121" & year==2009

replace regioncode="361122" if regioncode=="361122" & year==2009

replace regioncode="361123" if regioncode=="361123" & year==2009

replace regioncode="361124" if regioncode=="361124" & year==2009

replace regioncode="361125" if regioncode=="361125" & year==2009

replace regioncode="361126" if regioncode=="361126" & year==2009

replace regioncode="361127" if regioncode=="361127" & year==2009

replace regioncode="361128" if regioncode=="361128" & year==2009

replace regioncode="361129" if regioncode=="361129" & year==2009

replace regioncode="361130" if regioncode=="361130" & year==2009

replace regioncode="361181" if regioncode=="361181" & year==2009

replace regioncode="370102" if regioncode=="370102" & year==2009

replace regioncode="370103" if regioncode=="370103" & year==2009

replace regioncode="370104" if regioncode=="370104" & year==2009

replace regioncode="370105" if regioncode=="370105" & year==2009

replace regioncode="370112" if regioncode=="370112" & year==2009

replace regioncode="370113" if regioncode=="370113" & year==2009

replace regioncode="370124" if regioncode=="370124" & year==2009

replace regioncode="370125" if regioncode=="370125" & year==2009

replace regioncode="370126" if regioncode=="370126" & year==2009

replace regioncode="370181" if regioncode=="370181" & year==2009

replace regioncode="370202" if regioncode=="370202" & year==2009

replace regioncode="370203" if regioncode=="370203" & year==2009

replace regioncode="370205" if regioncode=="370205" & year==2009

replace regioncode="370211" if regioncode=="370211" & year==2009

replace regioncode="370212" if regioncode=="370212" & year==2009

replace regioncode="370213" if regioncode=="370213" & year==2009

replace regioncode="370214" if regioncode=="370214" & year==2009

replace regioncode="370281" if regioncode=="370281" & year==2009

replace regioncode="370282" if regioncode=="370282" & year==2009

replace regioncode="370283" if regioncode=="370283" & year==2009

replace regioncode="370284" if regioncode=="370284" & year==2009

replace regioncode="370285" if regioncode=="370285" & year==2009

replace regioncode="370302" if regioncode=="370302" & year==2009

replace regioncode="370303" if regioncode=="370303" & year==2009

replace regioncode="370304" if regioncode=="370304" & year==2009

replace regioncode="370305" if regioncode=="370305" & year==2009

replace regioncode="370306" if regioncode=="370306" & year==2009

replace regioncode="370321" if regioncode=="370321" & year==2009

replace regioncode="370322" if regioncode=="370322" & year==2009

replace regioncode="370323" if regioncode=="370323" & year==2009

replace regioncode="370402" if regioncode=="370402" & year==2009

replace regioncode="370403" if regioncode=="370403" & year==2009

replace regioncode="370404" if regioncode=="370404" & year==2009

replace regioncode="370405" if regioncode=="370405" & year==2009

replace regioncode="370406" if regioncode=="370406" & year==2009

replace regioncode="370481" if regioncode=="370481" & year==2009

replace regioncode="370502" if regioncode=="370502" & year==2009

replace regioncode="370503" if regioncode=="370503" & year==2009

replace regioncode="370521" if regioncode=="370521" & year==2009

replace regioncode="370522" if regioncode=="370522" & year==2009

replace regioncode="370523" if regioncode=="370523" & year==2009

replace regioncode="370602" if regioncode=="370602" & year==2009

replace regioncode="370611" if regioncode=="370611" & year==2009

replace regioncode="370612" if regioncode=="370612" & year==2009

replace regioncode="370613" if regioncode=="370613" & year==2009

replace regioncode="370634" if regioncode=="370634" & year==2009

replace regioncode="370681" if regioncode=="370681" & year==2009

replace regioncode="370682" if regioncode=="370682" & year==2009

replace regioncode="370683" if regioncode=="370683" & year==2009

replace regioncode="370684" if regioncode=="370684" & year==2009

replace regioncode="370685" if regioncode=="370685" & year==2009

replace regioncode="370686" if regioncode=="370686" & year==2009

replace regioncode="370687" if regioncode=="370687" & year==2009

replace regioncode="370702" if regioncode=="370702" & year==2009

replace regioncode="370703" if regioncode=="370703" & year==2009

replace regioncode="370704" if regioncode=="370704" & year==2009

replace regioncode="370705" if regioncode=="370705" & year==2009

replace regioncode="370724" if regioncode=="370724" & year==2009

replace regioncode="370725" if regioncode=="370725" & year==2009

replace regioncode="370781" if regioncode=="370781" & year==2009

replace regioncode="370782" if regioncode=="370782" & year==2009

replace regioncode="370783" if regioncode=="370783" & year==2009

replace regioncode="370784" if regioncode=="370784" & year==2009

replace regioncode="370785" if regioncode=="370785" & year==2009

replace regioncode="370786" if regioncode=="370786" & year==2009

replace regioncode="370802" if regioncode=="370802" & year==2009

replace regioncode="370811" if regioncode=="370811" & year==2009

replace regioncode="370826" if regioncode=="370826" & year==2009

replace regioncode="370827" if regioncode=="370827" & year==2009

replace regioncode="370828" if regioncode=="370828" & year==2009

replace regioncode="370829" if regioncode=="370829" & year==2009

replace regioncode="370830" if regioncode=="370830" & year==2009

replace regioncode="370831" if regioncode=="370831" & year==2009

replace regioncode="370832" if regioncode=="370832" & year==2009

replace regioncode="370881" if regioncode=="370881" & year==2009

replace regioncode="370882" if regioncode=="370882" & year==2009

replace regioncode="370883" if regioncode=="370883" & year==2009

replace regioncode="370902" if regioncode=="370902" & year==2009

replace regioncode="370903" if regioncode=="370911" & year==2009

replace regioncode="370921" if regioncode=="370921" & year==2009

replace regioncode="370923" if regioncode=="370923" & year==2009

replace regioncode="370982" if regioncode=="370982" & year==2009

replace regioncode="370983" if regioncode=="370983" & year==2009

replace regioncode="371002" if regioncode=="371002" & year==2009

replace regioncode="371081" if regioncode=="371081" & year==2009

replace regioncode="371082" if regioncode=="371082" & year==2009

replace regioncode="371083" if regioncode=="371083" & year==2009

replace regioncode="371102" if regioncode=="371102" & year==2009

replace regioncode="371121" if regioncode=="371121" & year==2009

replace regioncode="371122" if regioncode=="371122" & year==2009

replace regioncode="371202" if regioncode=="371202" & year==2009

replace regioncode="371203" if regioncode=="371203" & year==2009

replace regioncode="371302" if regioncode=="371302" & year==2009

replace regioncode="371311" if regioncode=="371311" & year==2009

replace regioncode="371312" if regioncode=="371312" & year==2009

replace regioncode="371321" if regioncode=="371321" & year==2009

replace regioncode="371322" if regioncode=="371322" & year==2009

replace regioncode="371323" if regioncode=="371323" & year==2009

replace regioncode="371324" if regioncode=="371324" & year==2009

replace regioncode="371325" if regioncode=="371325" & year==2009

replace regioncode="371326" if regioncode=="371326" & year==2009

replace regioncode="371327" if regioncode=="371327" & year==2009

replace regioncode="371328" if regioncode=="371328" & year==2009

replace regioncode="371329" if regioncode=="371329" & year==2009

replace regioncode="371402" if regioncode=="371402" & year==2009

replace regioncode="371421" if regioncode=="371421" & year==2009

replace regioncode="371422" if regioncode=="371422" & year==2009

replace regioncode="371423" if regioncode=="371423" & year==2009

replace regioncode="371424" if regioncode=="371424" & year==2009

replace regioncode="371425" if regioncode=="371425" & year==2009

replace regioncode="371426" if regioncode=="371426" & year==2009

replace regioncode="371427" if regioncode=="371427" & year==2009

replace regioncode="371428" if regioncode=="371428" & year==2009

replace regioncode="371481" if regioncode=="371481" & year==2009

replace regioncode="371482" if regioncode=="371482" & year==2009

replace regioncode="371502" if regioncode=="371502" & year==2009

replace regioncode="371521" if regioncode=="371521" & year==2009

replace regioncode="371522" if regioncode=="371522" & year==2009

replace regioncode="371523" if regioncode=="371523" & year==2009

replace regioncode="371524" if regioncode=="371524" & year==2009

replace regioncode="371525" if regioncode=="371525" & year==2009

replace regioncode="371526" if regioncode=="371526" & year==2009

replace regioncode="371581" if regioncode=="371581" & year==2009

replace regioncode="371602" if regioncode=="371602" & year==2009

replace regioncode="371621" if regioncode=="371621" & year==2009

replace regioncode="371622" if regioncode=="371622" & year==2009

replace regioncode="371623" if regioncode=="371623" & year==2009

replace regioncode="371624" if regioncode=="371624" & year==2009

replace regioncode="371625" if regioncode=="371625" & year==2009

replace regioncode="371626" if regioncode=="371626" & year==2009

replace regioncode="371702" if regioncode=="371702" & year==2009

replace regioncode="371721" if regioncode=="371721" & year==2009

replace regioncode="371722" if regioncode=="371722" & year==2009

replace regioncode="371723" if regioncode=="371723" & year==2009

replace regioncode="371724" if regioncode=="371724" & year==2009

replace regioncode="371725" if regioncode=="371725" & year==2009

replace regioncode="371726" if regioncode=="371726" & year==2009

replace regioncode="371727" if regioncode=="371727" & year==2009

replace regioncode="371728" if regioncode=="371728" & year==2009

replace regioncode="410102" if regioncode=="410102" & year==2009

replace regioncode="410103" if regioncode=="410103" & year==2009

replace regioncode="410104" if regioncode=="410104" & year==2009

replace regioncode="410105" if regioncode=="410105" & year==2009

replace regioncode="410106" if regioncode=="410106" & year==2009

replace regioncode="410108" if regioncode=="410108" & year==2009

replace regioncode="410122" if regioncode=="410122" & year==2009

replace regioncode="410181" if regioncode=="410181" & year==2009

replace regioncode="410182" if regioncode=="410182" & year==2009

replace regioncode="410183" if regioncode=="410183" & year==2009

replace regioncode="410184" if regioncode=="410184" & year==2009

replace regioncode="410185" if regioncode=="410185" & year==2009

replace regioncode="410202" if regioncode=="410202" & year==2009

replace regioncode="410203" if regioncode=="410203" & year==2009

replace regioncode="410204" if regioncode=="410204" & year==2009

replace regioncode="410205" if regioncode=="410205" & year==2009

replace regioncode="410211" if regioncode=="410211" & year==2009

replace regioncode="410221" if regioncode=="410221" & year==2009

replace regioncode="410222" if regioncode=="410222" & year==2009

replace regioncode="410223" if regioncode=="410223" & year==2009

replace regioncode="410224" if regioncode=="410224" & year==2009

replace regioncode="410225" if regioncode=="410225" & year==2009

replace regioncode="410302" if regioncode=="410302" & year==2009

replace regioncode="410303" if regioncode=="410303" & year==2009

replace regioncode="410304" if regioncode=="410304" & year==2009

replace regioncode="410305" if regioncode=="410305" & year==2009

replace regioncode="410306" if regioncode=="410306" & year==2009

replace regioncode="410307" if regioncode=="410311" & year==2009

replace regioncode="410322" if regioncode=="410322" & year==2009

replace regioncode="410323" if regioncode=="410323" & year==2009

replace regioncode="410324" if regioncode=="410324" & year==2009

replace regioncode="410325" if regioncode=="410325" & year==2009

replace regioncode="410326" if regioncode=="410326" & year==2009

replace regioncode="410327" if regioncode=="410327" & year==2009

replace regioncode="410328" if regioncode=="410328" & year==2009

replace regioncode="410329" if regioncode=="410329" & year==2009

replace regioncode="410381" if regioncode=="410381" & year==2009

replace regioncode="410402" if regioncode=="410402" & year==2009

replace regioncode="410403" if regioncode=="410403" & year==2009

replace regioncode="410404" if regioncode=="410404" & year==2009

replace regioncode="410411" if regioncode=="410411" & year==2009

replace regioncode="410421" if regioncode=="410421" & year==2009

replace regioncode="410422" if regioncode=="410422" & year==2009

replace regioncode="410423" if regioncode=="410423" & year==2009

replace regioncode="410425" if regioncode=="410425" & year==2009

replace regioncode="410481" if regioncode=="410481" & year==2009

replace regioncode="410482" if regioncode=="410482" & year==2009

replace regioncode="410502" if regioncode=="410502" & year==2009

replace regioncode="410503" if regioncode=="410503" & year==2009

replace regioncode="410504" if regioncode=="410505" & year==2009

replace regioncode="410511" if regioncode=="410506" & year==2009

replace regioncode="410522" if regioncode=="410522" & year==2009

replace regioncode="410523" if regioncode=="410523" & year==2009

replace regioncode="410526" if regioncode=="410526" & year==2009

replace regioncode="410527" if regioncode=="410527" & year==2009

replace regioncode="410581" if regioncode=="410581" & year==2009

replace regioncode="410602" if regioncode=="410602" & year==2009

replace regioncode="410603" if regioncode=="410603" & year==2009

replace regioncode="410611" if regioncode=="410611" & year==2009

replace regioncode="410621" if regioncode=="410621" & year==2009

replace regioncode="410622" if regioncode=="410622" & year==2009

replace regioncode="410702" if regioncode=="410702" & year==2009

replace regioncode="410703" if regioncode=="410703" & year==2009

replace regioncode="410704" if regioncode=="410704" & year==2009

replace regioncode="410711" if regioncode=="410711" & year==2009

replace regioncode="410721" if regioncode=="410721" & year==2009

replace regioncode="410724" if regioncode=="410724" & year==2009

replace regioncode="410725" if regioncode=="410725" & year==2009

replace regioncode="410726" if regioncode=="410726" & year==2009

replace regioncode="410727" if regioncode=="410727" & year==2009

replace regioncode="410728" if regioncode=="410728" & year==2009

replace regioncode="410781" if regioncode=="410781" & year==2009

replace regioncode="410782" if regioncode=="410782" & year==2009

replace regioncode="410802" if regioncode=="410802" & year==2009

replace regioncode="410803" if regioncode=="410803" & year==2009

replace regioncode="410804" if regioncode=="410804" & year==2009

replace regioncode="410811" if regioncode=="410811" & year==2009

replace regioncode="410821" if regioncode=="410821" & year==2009

replace regioncode="410822" if regioncode=="410822" & year==2009

replace regioncode="410823" if regioncode=="410823" & year==2009

replace regioncode="410825" if regioncode=="410825" & year==2009

replace regioncode="410881" if regioncode=="410881" & year==2009

replace regioncode="410882" if regioncode=="410882" & year==2009

replace regioncode="410883" if regioncode=="410883" & year==2009

replace regioncode="410902" if regioncode=="410902" & year==2009

replace regioncode="410922" if regioncode=="410922" & year==2009

replace regioncode="410923" if regioncode=="410923" & year==2009

replace regioncode="410926" if regioncode=="410926" & year==2009

replace regioncode="410927" if regioncode=="410927" & year==2009

replace regioncode="410928" if regioncode=="410928" & year==2009

replace regioncode="411002" if regioncode=="411002" & year==2009

replace regioncode="411023" if regioncode=="411023" & year==2009

replace regioncode="411024" if regioncode=="411024" & year==2009

replace regioncode="411025" if regioncode=="411025" & year==2009

replace regioncode="411081" if regioncode=="411081" & year==2009

replace regioncode="411082" if regioncode=="411082" & year==2009

replace regioncode="411102" if regioncode=="411102" & year==2009

replace regioncode="411123" if regioncode=="411103" & year==2009

replace regioncode="411121" if regioncode=="411121" & year==2009

replace regioncode="411122" if regioncode=="411122" & year==2009

replace regioncode="411202" if regioncode=="411202" & year==2009

replace regioncode="411221" if regioncode=="411221" & year==2009

replace regioncode="411222" if regioncode=="411222" & year==2009

replace regioncode="411224" if regioncode=="411224" & year==2009

replace regioncode="411281" if regioncode=="411281" & year==2009

replace regioncode="411282" if regioncode=="411282" & year==2009

replace regioncode="411302" if regioncode=="411302" & year==2009

replace regioncode="411303" if regioncode=="411303" & year==2009

replace regioncode="411321" if regioncode=="411321" & year==2009

replace regioncode="411322" if regioncode=="411322" & year==2009

replace regioncode="411323" if regioncode=="411323" & year==2009

replace regioncode="411324" if regioncode=="411324" & year==2009

replace regioncode="411325" if regioncode=="411325" & year==2009

replace regioncode="411326" if regioncode=="411326" & year==2009

replace regioncode="411327" if regioncode=="411327" & year==2009

replace regioncode="411328" if regioncode=="411328" & year==2009

replace regioncode="411329" if regioncode=="411329" & year==2009

replace regioncode="411330" if regioncode=="411330" & year==2009

replace regioncode="411381" if regioncode=="411381" & year==2009

replace regioncode="411402" if regioncode=="411402" & year==2009

replace regioncode="411403" if regioncode=="411403" & year==2009

replace regioncode="411421" if regioncode=="411421" & year==2009

replace regioncode="411422" if regioncode=="411422" & year==2009

replace regioncode="411423" if regioncode=="411423" & year==2009

replace regioncode="411424" if regioncode=="411424" & year==2009

replace regioncode="411425" if regioncode=="411425" & year==2009

replace regioncode="411426" if regioncode=="411426" & year==2009

replace regioncode="411481" if regioncode=="411481" & year==2009

replace regioncode="411502" if regioncode=="411502" & year==2009

replace regioncode="411503" if regioncode=="411503" & year==2009

replace regioncode="411521" if regioncode=="411521" & year==2009

replace regioncode="411522" if regioncode=="411522" & year==2009

replace regioncode="411523" if regioncode=="411523" & year==2009

replace regioncode="411524" if regioncode=="411524" & year==2009

replace regioncode="411525" if regioncode=="411525" & year==2009

replace regioncode="411526" if regioncode=="411526" & year==2009

replace regioncode="411527" if regioncode=="411527" & year==2009

replace regioncode="411528" if regioncode=="411528" & year==2009

replace regioncode="411602" if regioncode=="411602" & year==2009

replace regioncode="411621" if regioncode=="411621" & year==2009

replace regioncode="411622" if regioncode=="411622" & year==2009

replace regioncode="411623" if regioncode=="411623" & year==2009

replace regioncode="411624" if regioncode=="411624" & year==2009

replace regioncode="411625" if regioncode=="411625" & year==2009

replace regioncode="411626" if regioncode=="411626" & year==2009

replace regioncode="411627" if regioncode=="411627" & year==2009

replace regioncode="411628" if regioncode=="411628" & year==2009

replace regioncode="411681" if regioncode=="411681" & year==2009

replace regioncode="411702" if regioncode=="411702" & year==2009

replace regioncode="411721" if regioncode=="411721" & year==2009

replace regioncode="411722" if regioncode=="411722" & year==2009

replace regioncode="411723" if regioncode=="411723" & year==2009

replace regioncode="411724" if regioncode=="411724" & year==2009

replace regioncode="411725" if regioncode=="411725" & year==2009

replace regioncode="411726" if regioncode=="411726" & year==2009

replace regioncode="411727" if regioncode=="411727" & year==2009

replace regioncode="411728" if regioncode=="411728" & year==2009

replace regioncode="411729" if regioncode=="411729" & year==2009

replace regioncode="420102" if regioncode=="420102" & year==2009

replace regioncode="420103" if regioncode=="420103" & year==2009

replace regioncode="420104" if regioncode=="420104" & year==2009

replace regioncode="420105" if regioncode=="420105" & year==2009

replace regioncode="420106" if regioncode=="420106" & year==2009

replace regioncode="420107" if regioncode=="420107" & year==2009

replace regioncode="420111" if regioncode=="420111" & year==2009

replace regioncode="420112" if regioncode=="420112" & year==2009

replace regioncode="420113" if regioncode=="420113" & year==2009

replace regioncode="420114" if regioncode=="420114" & year==2009

replace regioncode="420115" if regioncode=="420115" & year==2009

replace regioncode="420116" if regioncode=="420116" & year==2009

replace regioncode="420117" if regioncode=="420117" & year==2009

replace regioncode="420202" if regioncode=="420202" & year==2009

replace regioncode="420203" if regioncode=="420203" & year==2009

replace regioncode="420204" if regioncode=="420204" & year==2009

replace regioncode="420205" if regioncode=="420205" & year==2009

replace regioncode="420222" if regioncode=="420222" & year==2009

replace regioncode="420281" if regioncode=="420281" & year==2009

replace regioncode="420302" if regioncode=="420302" & year==2009

replace regioncode="420303" if regioncode=="420303" & year==2009

replace regioncode="420321" if regioncode=="420321" & year==2009

replace regioncode="420322" if regioncode=="420322" & year==2009

replace regioncode="420323" if regioncode=="420323" & year==2009

replace regioncode="420324" if regioncode=="420324" & year==2009

replace regioncode="420325" if regioncode=="420325" & year==2009

replace regioncode="420381" if regioncode=="420381" & year==2009

replace regioncode="420502" if regioncode=="420502" & year==2009

replace regioncode="420503" if regioncode=="420503" & year==2009

replace regioncode="420504" if regioncode=="420504" & year==2009

replace regioncode="420505" if regioncode=="420505" & year==2009

replace regioncode="420506" if regioncode=="420506" & year==2009

replace regioncode="420525" if regioncode=="420525" & year==2009

replace regioncode="420526" if regioncode=="420526" & year==2009

replace regioncode="420527" if regioncode=="420527" & year==2009

replace regioncode="420528" if regioncode=="420528" & year==2009

replace regioncode="420529" if regioncode=="420529" & year==2009

replace regioncode="420581" if regioncode=="420581" & year==2009

replace regioncode="420582" if regioncode=="420582" & year==2009

replace regioncode="420583" if regioncode=="420583" & year==2009

replace regioncode="420602" if regioncode=="420602" & year==2009

replace regioncode="420606" if regioncode=="420606" & year==2009

replace regioncode="420607" if regioncode=="420607" & year==2009

replace regioncode="420624" if regioncode=="420624" & year==2009

replace regioncode="420625" if regioncode=="420625" & year==2009

replace regioncode="420626" if regioncode=="420626" & year==2009

replace regioncode="420682" if regioncode=="420682" & year==2009

replace regioncode="420683" if regioncode=="420683" & year==2009

replace regioncode="420684" if regioncode=="420684" & year==2009

replace regioncode="420702" if regioncode=="420702" & year==2009

replace regioncode="420703" if regioncode=="420703" & year==2009

replace regioncode="420704" if regioncode=="420704" & year==2009

replace regioncode="420802" if regioncode=="420802" & year==2009

replace regioncode="420804" if regioncode=="420804" & year==2009

replace regioncode="420821" if regioncode=="420821" & year==2009

replace regioncode="420822" if regioncode=="420822" & year==2009

replace regioncode="420881" if regioncode=="420881" & year==2009

replace regioncode="420902" if regioncode=="420902" & year==2009

replace regioncode="420921" if regioncode=="420921" & year==2009

replace regioncode="420922" if regioncode=="420922" & year==2009

replace regioncode="420923" if regioncode=="420923" & year==2009

replace regioncode="420981" if regioncode=="420981" & year==2009

replace regioncode="420982" if regioncode=="420982" & year==2009

replace regioncode="421381" if regioncode=="421381" & year==2009

replace regioncode="420984" if regioncode=="420984" & year==2009

replace regioncode="421002" if regioncode=="421002" & year==2009

replace regioncode="421003" if regioncode=="421003" & year==2009

replace regioncode="421022" if regioncode=="421022" & year==2009

replace regioncode="421023" if regioncode=="421023" & year==2009

replace regioncode="421024" if regioncode=="421024" & year==2009

replace regioncode="421081" if regioncode=="421081" & year==2009

replace regioncode="421083" if regioncode=="421083" & year==2009

replace regioncode="421087" if regioncode=="421087" & year==2009

replace regioncode="421102" if regioncode=="421102" & year==2009

replace regioncode="421121" if regioncode=="421121" & year==2009

replace regioncode="421122" if regioncode=="421122" & year==2009

replace regioncode="421123" if regioncode=="421123" & year==2009

replace regioncode="421124" if regioncode=="421124" & year==2009

replace regioncode="421125" if regioncode=="421125" & year==2009

replace regioncode="421126" if regioncode=="421126" & year==2009

replace regioncode="421127" if regioncode=="421127" & year==2009

replace regioncode="421181" if regioncode=="421181" & year==2009

replace regioncode="421182" if regioncode=="421182" & year==2009

replace regioncode="421202" if regioncode=="421202" & year==2009

replace regioncode="421221" if regioncode=="421221" & year==2009

replace regioncode="421222" if regioncode=="421222" & year==2009

replace regioncode="421223" if regioncode=="421223" & year==2009

replace regioncode="421224" if regioncode=="421224" & year==2009

replace regioncode="421281" if regioncode=="421281" & year==2009

replace regioncode="421302" if regioncode=="421302" & year==2009

replace regioncode="422801" if regioncode=="422801" & year==2009

replace regioncode="422802" if regioncode=="422802" & year==2009

replace regioncode="422822" if regioncode=="422822" & year==2009

replace regioncode="422823" if regioncode=="422823" & year==2009

replace regioncode="422825" if regioncode=="422825" & year==2009

replace regioncode="422826" if regioncode=="422826" & year==2009

replace regioncode="422827" if regioncode=="422827" & year==2009

replace regioncode="422828" if regioncode=="422828" & year==2009

replace regioncode="429004" if regioncode=="429004" & year==2009

replace regioncode="429005" if regioncode=="429005" & year==2009

replace regioncode="429006" if regioncode=="429006" & year==2009

replace regioncode="429021" if regioncode=="429021" & year==2009

replace regioncode="430102" if regioncode=="430102" & year==2009

replace regioncode="430103" if regioncode=="430103" & year==2009

replace regioncode="430104" if regioncode=="430104" & year==2009

replace regioncode="430105" if regioncode=="430105" & year==2009

replace regioncode="430111" if regioncode=="430111" & year==2009

replace regioncode="430121" if regioncode=="430121" & year==2009

replace regioncode="430122" if regioncode=="430122" & year==2009

replace regioncode="430124" if regioncode=="430124" & year==2009

replace regioncode="430181" if regioncode=="430181" & year==2009

replace regioncode="430202" if regioncode=="430202" & year==2009

replace regioncode="430203" if regioncode=="430203" & year==2009

replace regioncode="430204" if regioncode=="430204" & year==2009

replace regioncode="430211" if regioncode=="430211" & year==2009

replace regioncode="430221" if regioncode=="430221" & year==2009

replace regioncode="430223" if regioncode=="430223" & year==2009

replace regioncode="430224" if regioncode=="430224" & year==2009

replace regioncode="430225" if regioncode=="430225" & year==2009

replace regioncode="430281" if regioncode=="430281" & year==2009

replace regioncode="430302" if regioncode=="430302" & year==2009

replace regioncode="430304" if regioncode=="430304" & year==2009

replace regioncode="430321" if regioncode=="430321" & year==2009

replace regioncode="430381" if regioncode=="430381" & year==2009

replace regioncode="430382" if regioncode=="430382" & year==2009

replace regioncode="430405" if regioncode=="430405" & year==2009

replace regioncode="430406" if regioncode=="430406" & year==2009

replace regioncode="430407" if regioncode=="430407" & year==2009

replace regioncode="430408" if regioncode=="430408" & year==2009

replace regioncode="430412" if regioncode=="430412" & year==2009

replace regioncode="430421" if regioncode=="430421" & year==2009

replace regioncode="430422" if regioncode=="430422" & year==2009

replace regioncode="430423" if regioncode=="430423" & year==2009

replace regioncode="430424" if regioncode=="430424" & year==2009

replace regioncode="430426" if regioncode=="430426" & year==2009

replace regioncode="430481" if regioncode=="430481" & year==2009

replace regioncode="430482" if regioncode=="430482" & year==2009

replace regioncode="430502" if regioncode=="430502" & year==2009

replace regioncode="430503" if regioncode=="430503" & year==2009

replace regioncode="430511" if regioncode=="430511" & year==2009

replace regioncode="430521" if regioncode=="430521" & year==2009

replace regioncode="430522" if regioncode=="430522" & year==2009

replace regioncode="430523" if regioncode=="430523" & year==2009

replace regioncode="430524" if regioncode=="430524" & year==2009

replace regioncode="430525" if regioncode=="430525" & year==2009

replace regioncode="430527" if regioncode=="430527" & year==2009

replace regioncode="430528" if regioncode=="430528" & year==2009

replace regioncode="430529" if regioncode=="430529" & year==2009

replace regioncode="430581" if regioncode=="430581" & year==2009

replace regioncode="430602" if regioncode=="430602" & year==2009

replace regioncode="430603" if regioncode=="430603" & year==2009

replace regioncode="430611" if regioncode=="430611" & year==2009

replace regioncode="430621" if regioncode=="430621" & year==2009

replace regioncode="430623" if regioncode=="430623" & year==2009

replace regioncode="430624" if regioncode=="430624" & year==2009

replace regioncode="430626" if regioncode=="430626" & year==2009

replace regioncode="430681" if regioncode=="430681" & year==2009

replace regioncode="430682" if regioncode=="430682" & year==2009

replace regioncode="430702" if regioncode=="430702" & year==2009

replace regioncode="430703" if regioncode=="430703" & year==2009

replace regioncode="430721" if regioncode=="430721" & year==2009

replace regioncode="430722" if regioncode=="430722" & year==2009

replace regioncode="430723" if regioncode=="430723" & year==2009

replace regioncode="430724" if regioncode=="430724" & year==2009

replace regioncode="430725" if regioncode=="430725" & year==2009

replace regioncode="430726" if regioncode=="430726" & year==2009

replace regioncode="430781" if regioncode=="430781" & year==2009

replace regioncode="430802" if regioncode=="430802" & year==2009

replace regioncode="430811" if regioncode=="430811" & year==2009

replace regioncode="430821" if regioncode=="430821" & year==2009

replace regioncode="430822" if regioncode=="430822" & year==2009

replace regioncode="430902" if regioncode=="430902" & year==2009

replace regioncode="430903" if regioncode=="430903" & year==2009

replace regioncode="430921" if regioncode=="430921" & year==2009

replace regioncode="430922" if regioncode=="430922" & year==2009

replace regioncode="430923" if regioncode=="430923" & year==2009

replace regioncode="430981" if regioncode=="430981" & year==2009

replace regioncode="431002" if regioncode=="431002" & year==2009

replace regioncode="431003" if regioncode=="431003" & year==2009

replace regioncode="431021" if regioncode=="431021" & year==2009

replace regioncode="431022" if regioncode=="431022" & year==2009

replace regioncode="431023" if regioncode=="431023" & year==2009

replace regioncode="431024" if regioncode=="431024" & year==2009

replace regioncode="431025" if regioncode=="431025" & year==2009

replace regioncode="431026" if regioncode=="431026" & year==2009

replace regioncode="431027" if regioncode=="431027" & year==2009

replace regioncode="431028" if regioncode=="431028" & year==2009

replace regioncode="431081" if regioncode=="431081" & year==2009

replace regioncode="431102" if regioncode=="431102" & year==2009

replace regioncode="431103" if regioncode=="431103" & year==2009

replace regioncode="431121" if regioncode=="431121" & year==2009

replace regioncode="431122" if regioncode=="431122" & year==2009

replace regioncode="431123" if regioncode=="431123" & year==2009

replace regioncode="431124" if regioncode=="431124" & year==2009

replace regioncode="431125" if regioncode=="431125" & year==2009

replace regioncode="431126" if regioncode=="431126" & year==2009

replace regioncode="431127" if regioncode=="431127" & year==2009

replace regioncode="431128" if regioncode=="431128" & year==2009

replace regioncode="431129" if regioncode=="431129" & year==2009

replace regioncode="431202" if regioncode=="431202" & year==2009

replace regioncode="431221" if regioncode=="431221" & year==2009

replace regioncode="431222" if regioncode=="431222" & year==2009

replace regioncode="431223" if regioncode=="431223" & year==2009

replace regioncode="431224" if regioncode=="431224" & year==2009

replace regioncode="431225" if regioncode=="431225" & year==2009

replace regioncode="431226" if regioncode=="431226" & year==2009

replace regioncode="431227" if regioncode=="431227" & year==2009

replace regioncode="431228" if regioncode=="431228" & year==2009

replace regioncode="431229" if regioncode=="431229" & year==2009

replace regioncode="431230" if regioncode=="431230" & year==2009

replace regioncode="431281" if regioncode=="431281" & year==2009

replace regioncode="431302" if regioncode=="431302" & year==2009

replace regioncode="431321" if regioncode=="431321" & year==2009

replace regioncode="431322" if regioncode=="431322" & year==2009

replace regioncode="431381" if regioncode=="431381" & year==2009

replace regioncode="431382" if regioncode=="431382" & year==2009

replace regioncode="433101" if regioncode=="433101" & year==2009

replace regioncode="433122" if regioncode=="433122" & year==2009

replace regioncode="433123" if regioncode=="433123" & year==2009

replace regioncode="433124" if regioncode=="433124" & year==2009

replace regioncode="433125" if regioncode=="433125" & year==2009

replace regioncode="433126" if regioncode=="433126" & year==2009

replace regioncode="433127" if regioncode=="433127" & year==2009

replace regioncode="433130" if regioncode=="433130" & year==2009

replace regioncode="440102" if regioncode=="440115" & year==2009

replace regioncode="440103" if regioncode=="440103" & year==2009

replace regioncode="440104" if regioncode=="440104" & year==2009

replace regioncode="440105" if regioncode=="440105" & year==2009

replace regioncode="440106" if regioncode=="440106" & year==2009

replace regioncode="440107" if regioncode=="440116" & year==2009

replace regioncode="440111" if regioncode=="440111" & year==2009

replace regioncode="440112" if regioncode=="440112" & year==2009

replace regioncode="440113" if regioncode=="440113" & year==2009

replace regioncode="440114" if regioncode=="440114" & year==2009

replace regioncode="440183" if regioncode=="440183" & year==2009

replace regioncode="440184" if regioncode=="440184" & year==2009

replace regioncode="440203" if regioncode=="440203" & year==2009

replace regioncode="440204" if regioncode=="440204" & year==2009

replace regioncode="440221" if regioncode=="440205" & year==2009

replace regioncode="440222" if regioncode=="440222" & year==2009

replace regioncode="440224" if regioncode=="440224" & year==2009

replace regioncode="440229" if regioncode=="440229" & year==2009

replace regioncode="440232" if regioncode=="440232" & year==2009

replace regioncode="440233" if regioncode=="440233" & year==2009

replace regioncode="440281" if regioncode=="440281" & year==2009

replace regioncode="440282" if regioncode=="440282" & year==2009

replace regioncode="440303" if regioncode=="440303" & year==2009

replace regioncode="440304" if regioncode=="440304" & year==2009

replace regioncode="440305" if regioncode=="440305" & year==2009

replace regioncode="440306" if regioncode=="440306" & year==2009

replace regioncode="440307" if regioncode=="440307" & year==2009

replace regioncode="440308" if regioncode=="440308" & year==2009

replace regioncode="440402" if regioncode=="440402" & year==2009

replace regioncode="440403" if regioncode=="440403" & year==2009

replace regioncode="440404" if regioncode=="440404" & year==2009

replace regioncode="440507" if regioncode=="440507" & year==2009

replace regioncode="440511" if regioncode=="440511" & year==2009

replace regioncode="440512" if regioncode=="440512" & year==2009

replace regioncode="440514" if regioncode=="440514" & year==2009

replace regioncode="440515" if regioncode=="440515" & year==2009

replace regioncode="440523" if regioncode=="440523" & year==2009

replace regioncode="440604" if regioncode=="440604" & year==2009

replace regioncode="440605" if regioncode=="440605" & year==2009

replace regioncode="440606" if regioncode=="440606" & year==2009

replace regioncode="440607" if regioncode=="440607" & year==2009

replace regioncode="440608" if regioncode=="440608" & year==2009

replace regioncode="440703" if regioncode=="440703" & year==2009

replace regioncode="440704" if regioncode=="440704" & year==2009

replace regioncode="440781" if regioncode=="440781" & year==2009

replace regioncode="440705" if regioncode=="440705" & year==2009

replace regioncode="440783" if regioncode=="440783" & year==2009

replace regioncode="440784" if regioncode=="440784" & year==2009

replace regioncode="440785" if regioncode=="440785" & year==2009

replace regioncode="440802" if regioncode=="440802" & year==2009

replace regioncode="440803" if regioncode=="440803" & year==2009

replace regioncode="440804" if regioncode=="440804" & year==2009

replace regioncode="440811" if regioncode=="440811" & year==2009

replace regioncode="440823" if regioncode=="440823" & year==2009

replace regioncode="440825" if regioncode=="440825" & year==2009

replace regioncode="440881" if regioncode=="440881" & year==2009

replace regioncode="440882" if regioncode=="440882" & year==2009

replace regioncode="440883" if regioncode=="440883" & year==2009

replace regioncode="440902" if regioncode=="440902" & year==2009

replace regioncode="440903" if regioncode=="440903" & year==2009

replace regioncode="440923" if regioncode=="440923" & year==2009

replace regioncode="440981" if regioncode=="440981" & year==2009

replace regioncode="440982" if regioncode=="440982" & year==2009

replace regioncode="440983" if regioncode=="440983" & year==2009

replace regioncode="441202" if regioncode=="441202" & year==2009

replace regioncode="441203" if regioncode=="441203" & year==2009

replace regioncode="441223" if regioncode=="441223" & year==2009

replace regioncode="441224" if regioncode=="441224" & year==2009

replace regioncode="441225" if regioncode=="441225" & year==2009

replace regioncode="441226" if regioncode=="441226" & year==2009

replace regioncode="441283" if regioncode=="441283" & year==2009

replace regioncode="441284" if regioncode=="441284" & year==2009

replace regioncode="441302" if regioncode=="441302" & year==2009

replace regioncode="441303" if regioncode=="441303" & year==2009

replace regioncode="441322" if regioncode=="441322" & year==2009

replace regioncode="441323" if regioncode=="441323" & year==2009

replace regioncode="441324" if regioncode=="441324" & year==2009

replace regioncode="441402" if regioncode=="441402" & year==2009

replace regioncode="441421" if regioncode=="441421" & year==2009

replace regioncode="441422" if regioncode=="441422" & year==2009

replace regioncode="441423" if regioncode=="441423" & year==2009

replace regioncode="441424" if regioncode=="441424" & year==2009

replace regioncode="441426" if regioncode=="441426" & year==2009

replace regioncode="441427" if regioncode=="441427" & year==2009

replace regioncode="441481" if regioncode=="441481" & year==2009

replace regioncode="441502" if regioncode=="441502" & year==2009

replace regioncode="441521" if regioncode=="441521" & year==2009

replace regioncode="441523" if regioncode=="441523" & year==2009

replace regioncode="441581" if regioncode=="441581" & year==2009

replace regioncode="441602" if regioncode=="441602" & year==2009

replace regioncode="441621" if regioncode=="441621" & year==2009

replace regioncode="441622" if regioncode=="441622" & year==2009

replace regioncode="441623" if regioncode=="441623" & year==2009

replace regioncode="441624" if regioncode=="441624" & year==2009

replace regioncode="441625" if regioncode=="441625" & year==2009

replace regioncode="441702" if regioncode=="441702" & year==2009

replace regioncode="441721" if regioncode=="441721" & year==2009

replace regioncode="441723" if regioncode=="441723" & year==2009

replace regioncode="441781" if regioncode=="441781" & year==2009

replace regioncode="441802" if regioncode=="441802" & year==2009

replace regioncode="441821" if regioncode=="441821" & year==2009

replace regioncode="441823" if regioncode=="441823" & year==2009

replace regioncode="441825" if regioncode=="441825" & year==2009

replace regioncode="441826" if regioncode=="441826" & year==2009

replace regioncode="441827" if regioncode=="441827" & year==2009

replace regioncode="441881" if regioncode=="441881" & year==2009

replace regioncode="441882" if regioncode=="441882" & year==2009

replace regioncode="441900" if regioncode=="441901" & year==2009

replace regioncode="442000" if regioncode=="442001" & year==2009

replace regioncode="445102" if regioncode=="445102" & year==2009

replace regioncode="445121" if regioncode=="445121" & year==2009

replace regioncode="445122" if regioncode=="445122" & year==2009

replace regioncode="445202" if regioncode=="445202" & year==2009

replace regioncode="445221" if regioncode=="445221" & year==2009

replace regioncode="445222" if regioncode=="445222" & year==2009

replace regioncode="445224" if regioncode=="445224" & year==2009

replace regioncode="445281" if regioncode=="445281" & year==2009

replace regioncode="445302" if regioncode=="445302" & year==2009

replace regioncode="445321" if regioncode=="445321" & year==2009

replace regioncode="445322" if regioncode=="445322" & year==2009

replace regioncode="445323" if regioncode=="445323" & year==2009

replace regioncode="445381" if regioncode=="445381" & year==2009

replace regioncode="450102" if regioncode=="450102" & year==2009

replace regioncode="450103" if regioncode=="450103" & year==2009

replace regioncode="450104" if regioncode=="450107" & year==2009

replace regioncode="450105" if regioncode=="450105" & year==2009

replace regioncode="450106" if regioncode=="450108" & year==2009

replace regioncode="450121" if regioncode=="450109" & year==2009

replace regioncode="450122" if regioncode=="450122" & year==2009

replace regioncode="450123" if regioncode=="450123" & year==2009

replace regioncode="450124" if regioncode=="450124" & year==2009

replace regioncode="450125" if regioncode=="450125" & year==2009

replace regioncode="450126" if regioncode=="450126" & year==2009

replace regioncode="450127" if regioncode=="450127" & year==2009

replace regioncode="450202" if regioncode=="450202" & year==2009

replace regioncode="450203" if regioncode=="450203" & year==2009

replace regioncode="450204" if regioncode=="450204" & year==2009

replace regioncode="450205" if regioncode=="450205" & year==2009

replace regioncode="450221" if regioncode=="450221" & year==2009

replace regioncode="450222" if regioncode=="450222" & year==2009

replace regioncode="450223" if regioncode=="450223" & year==2009

replace regioncode="450224" if regioncode=="450224" & year==2009

replace regioncode="450225" if regioncode=="450225" & year==2009

replace regioncode="450226" if regioncode=="450226" & year==2009

replace regioncode="450302" if regioncode=="450302" & year==2009

replace regioncode="450303" if regioncode=="450303" & year==2009

replace regioncode="450304" if regioncode=="450304" & year==2009

replace regioncode="450305" if regioncode=="450305" & year==2009

replace regioncode="450311" if regioncode=="450311" & year==2009

replace regioncode="450321" if regioncode=="450321" & year==2009

replace regioncode="450322" if regioncode=="450322" & year==2009

replace regioncode="450323" if regioncode=="450323" & year==2009

replace regioncode="450324" if regioncode=="450324" & year==2009

replace regioncode="450325" if regioncode=="450325" & year==2009

replace regioncode="450326" if regioncode=="450326" & year==2009

replace regioncode="450327" if regioncode=="450327" & year==2009

replace regioncode="450328" if regioncode=="450328" & year==2009

replace regioncode="450329" if regioncode=="450329" & year==2009

replace regioncode="450330" if regioncode=="450330" & year==2009

replace regioncode="450331" if regioncode=="450331" & year==2009

replace regioncode="450332" if regioncode=="450332" & year==2009

replace regioncode="450403" if regioncode=="450403" & year==2009

replace regioncode="450404" if regioncode=="450404" & year==2009

replace regioncode="450421" if regioncode=="450421" & year==2009

replace regioncode="450422" if regioncode=="450422" & year==2009

replace regioncode="450423" if regioncode=="450423" & year==2009

replace regioncode="450481" if regioncode=="450481" & year==2009

replace regioncode="450502" if regioncode=="450502" & year==2009

replace regioncode="450503" if regioncode=="450503" & year==2009

replace regioncode="450512" if regioncode=="450512" & year==2009

replace regioncode="450521" if regioncode=="450521" & year==2009

replace regioncode="450602" if regioncode=="450602" & year==2009

replace regioncode="450603" if regioncode=="450603" & year==2009

replace regioncode="450621" if regioncode=="450621" & year==2009

replace regioncode="450681" if regioncode=="450681" & year==2009

replace regioncode="450702" if regioncode=="450702" & year==2009

replace regioncode="450703" if regioncode=="450703" & year==2009

replace regioncode="450721" if regioncode=="450721" & year==2009

replace regioncode="450722" if regioncode=="450722" & year==2009

replace regioncode="450802" if regioncode=="450802" & year==2009

replace regioncode="450803" if regioncode=="450803" & year==2009

replace regioncode="450804" if regioncode=="450804" & year==2009

replace regioncode="450821" if regioncode=="450821" & year==2009

replace regioncode="450881" if regioncode=="450881" & year==2009

replace regioncode="450902" if regioncode=="450902" & year==2009

replace regioncode="450921" if regioncode=="450921" & year==2009

replace regioncode="450922" if regioncode=="450922" & year==2009

replace regioncode="450923" if regioncode=="450923" & year==2009

replace regioncode="450924" if regioncode=="450924" & year==2009

replace regioncode="450981" if regioncode=="450981" & year==2009

replace regioncode="451002" if regioncode=="451002" & year==2009

replace regioncode="451021" if regioncode=="451021" & year==2009

replace regioncode="451022" if regioncode=="451022" & year==2009

replace regioncode="451023" if regioncode=="451023" & year==2009

replace regioncode="451024" if regioncode=="451024" & year==2009

replace regioncode="451025" if regioncode=="451025" & year==2009

replace regioncode="451026" if regioncode=="451026" & year==2009

replace regioncode="451027" if regioncode=="451027" & year==2009

replace regioncode="451028" if regioncode=="451028" & year==2009

replace regioncode="451029" if regioncode=="451029" & year==2009

replace regioncode="451030" if regioncode=="451030" & year==2009

replace regioncode="451031" if regioncode=="451031" & year==2009

replace regioncode="451102" if regioncode=="451102" & year==2009

replace regioncode="451121" if regioncode=="451121" & year==2009

replace regioncode="451122" if regioncode=="451122" & year==2009

replace regioncode="451123" if regioncode=="451123" & year==2009

replace regioncode="451202" if regioncode=="451202" & year==2009

replace regioncode="451221" if regioncode=="451221" & year==2009

replace regioncode="451222" if regioncode=="451222" & year==2009

replace regioncode="451223" if regioncode=="451223" & year==2009

replace regioncode="451224" if regioncode=="451224" & year==2009

replace regioncode="451225" if regioncode=="451225" & year==2009

replace regioncode="451226" if regioncode=="451226" & year==2009

replace regioncode="451227" if regioncode=="451227" & year==2009

replace regioncode="451228" if regioncode=="451228" & year==2009

replace regioncode="451229" if regioncode=="451229" & year==2009

replace regioncode="451281" if regioncode=="451281" & year==2009

replace regioncode="451302" if regioncode=="451302" & year==2009

replace regioncode="451321" if regioncode=="451321" & year==2009

replace regioncode="451322" if regioncode=="451322" & year==2009

replace regioncode="451323" if regioncode=="451323" & year==2009

replace regioncode="451324" if regioncode=="451324" & year==2009

replace regioncode="451381" if regioncode=="451381" & year==2009

replace regioncode="451402" if regioncode=="451402" & year==2009

replace regioncode="451421" if regioncode=="451421" & year==2009

replace regioncode="451422" if regioncode=="451422" & year==2009

replace regioncode="451423" if regioncode=="451423" & year==2009

replace regioncode="451424" if regioncode=="451424" & year==2009

replace regioncode="451425" if regioncode=="451425" & year==2009

replace regioncode="451481" if regioncode=="451481" & year==2009

replace regioncode="460105" if regioncode=="460105" & year==2009

replace regioncode="460106" if regioncode=="460106" & year==2009

replace regioncode="460107" if regioncode=="460107" & year==2009

replace regioncode="460108" if regioncode=="460108" & year==2009

replace regioncode="460001" if regioncode=="469001" & year==2009

replace regioncode="460002" if regioncode=="469002" & year==2009

replace regioncode="460003" if regioncode=="469003" & year==2009

replace regioncode="460005" if regioncode=="469005" & year==2009

replace regioncode="460006" if regioncode=="469006" & year==2009

replace regioncode="460007" if regioncode=="469007" & year==2009

replace regioncode="460030" if regioncode=="469025" & year==2009

replace regioncode="460031" if regioncode=="469026" & year==2009

replace regioncode="460033" if regioncode=="469027" & year==2009

replace regioncode="460034" if regioncode=="469028" & year==2009

replace regioncode="460036" if regioncode=="469030" & year==2009

replace regioncode="500101" if regioncode=="500101" & year==2009

replace regioncode="500102" if regioncode=="500102" & year==2009

replace regioncode="500103" if regioncode=="500103" & year==2009

replace regioncode="500104" if regioncode=="500104" & year==2009

replace regioncode="500105" if regioncode=="500105" & year==2009

replace regioncode="500106" if regioncode=="500106" & year==2009

replace regioncode="500107" if regioncode=="500107" & year==2009

replace regioncode="500108" if regioncode=="500108" & year==2009

replace regioncode="500109" if regioncode=="500109" & year==2009

replace regioncode="500110" if regioncode=="500110" & year==2009

replace regioncode="500111" if regioncode=="500111" & year==2009

replace regioncode="500112" if regioncode=="500112" & year==2009

replace regioncode="500113" if regioncode=="500113" & year==2009

replace regioncode="500114" if regioncode=="500114" & year==2009

replace regioncode="500115" if regioncode=="500115" & year==2009

replace regioncode="500381" if regioncode=="500116" & year==2009

replace regioncode="500382" if regioncode=="500117" & year==2009

replace regioncode="500383" if regioncode=="500118" & year==2009

replace regioncode="500384" if regioncode=="500119" & year==2009

replace regioncode="500222" if regioncode=="500222" & year==2009

replace regioncode="500223" if regioncode=="500223" & year==2009

replace regioncode="500224" if regioncode=="500224" & year==2009

replace regioncode="500225" if regioncode=="500225" & year==2009

replace regioncode="500226" if regioncode=="500226" & year==2009

replace regioncode="500227" if regioncode=="500227" & year==2009

replace regioncode="500228" if regioncode=="500228" & year==2009

replace regioncode="500229" if regioncode=="500229" & year==2009

replace regioncode="500230" if regioncode=="500230" & year==2009

replace regioncode="500231" if regioncode=="500231" & year==2009

replace regioncode="500232" if regioncode=="500232" & year==2009

replace regioncode="500233" if regioncode=="500233" & year==2009

replace regioncode="500234" if regioncode=="500234" & year==2009

replace regioncode="500235" if regioncode=="500235" & year==2009

replace regioncode="500236" if regioncode=="500236" & year==2009

replace regioncode="500237" if regioncode=="500237" & year==2009

replace regioncode="500238" if regioncode=="500238" & year==2009

replace regioncode="500240" if regioncode=="500240" & year==2009

replace regioncode="500241" if regioncode=="500241" & year==2009

replace regioncode="500242" if regioncode=="500242" & year==2009

replace regioncode="500243" if regioncode=="500243" & year==2009

replace regioncode="510104" if regioncode=="510104" & year==2009

replace regioncode="510105" if regioncode=="510105" & year==2009

replace regioncode="510106" if regioncode=="510106" & year==2009

replace regioncode="510107" if regioncode=="510107" & year==2009

replace regioncode="510108" if regioncode=="510108" & year==2009

replace regioncode="510112" if regioncode=="510112" & year==2009

replace regioncode="510113" if regioncode=="510113" & year==2009

replace regioncode="510114" if regioncode=="510114" & year==2009

replace regioncode="510115" if regioncode=="510115" & year==2009

replace regioncode="510121" if regioncode=="510121" & year==2009

replace regioncode="510122" if regioncode=="510122" & year==2009

replace regioncode="510124" if regioncode=="510124" & year==2009

replace regioncode="510129" if regioncode=="510129" & year==2009

replace regioncode="510131" if regioncode=="510131" & year==2009

replace regioncode="510132" if regioncode=="510132" & year==2009

replace regioncode="510181" if regioncode=="510181" & year==2009

replace regioncode="510182" if regioncode=="510182" & year==2009

replace regioncode="510183" if regioncode=="510183" & year==2009

replace regioncode="510184" if regioncode=="510184" & year==2009

replace regioncode="510302" if regioncode=="510302" & year==2009

replace regioncode="510303" if regioncode=="510303" & year==2009

replace regioncode="510304" if regioncode=="510304" & year==2009

replace regioncode="510311" if regioncode=="510311" & year==2009

replace regioncode="510321" if regioncode=="510321" & year==2009

replace regioncode="510322" if regioncode=="510322" & year==2009

replace regioncode="510402" if regioncode=="510402" & year==2009

replace regioncode="510403" if regioncode=="510403" & year==2009

replace regioncode="510411" if regioncode=="510411" & year==2009

replace regioncode="510421" if regioncode=="510421" & year==2009

replace regioncode="510422" if regioncode=="510422" & year==2009

replace regioncode="510502" if regioncode=="510502" & year==2009

replace regioncode="510503" if regioncode=="510503" & year==2009

replace regioncode="510504" if regioncode=="510504" & year==2009

replace regioncode="510521" if regioncode=="510521" & year==2009

replace regioncode="510522" if regioncode=="510522" & year==2009

replace regioncode="510524" if regioncode=="510524" & year==2009

replace regioncode="510525" if regioncode=="510525" & year==2009

replace regioncode="510603" if regioncode=="510603" & year==2009

replace regioncode="510623" if regioncode=="510623" & year==2009

replace regioncode="510626" if regioncode=="510626" & year==2009

replace regioncode="510681" if regioncode=="510681" & year==2009

replace regioncode="510682" if regioncode=="510682" & year==2009

replace regioncode="510683" if regioncode=="510683" & year==2009

replace regioncode="510703" if regioncode=="510703" & year==2009

replace regioncode="510704" if regioncode=="510704" & year==2009

replace regioncode="510722" if regioncode=="510722" & year==2009

replace regioncode="510723" if regioncode=="510723" & year==2009

replace regioncode="510724" if regioncode=="510724" & year==2009

replace regioncode="510725" if regioncode=="510725" & year==2009

replace regioncode="510726" if regioncode=="510726" & year==2009

replace regioncode="510727" if regioncode=="510727" & year==2009

replace regioncode="510781" if regioncode=="510781" & year==2009

replace regioncode="510802" if regioncode=="510802" & year==2009

replace regioncode="510811" if regioncode=="510811" & year==2009

replace regioncode="510812" if regioncode=="510812" & year==2009

replace regioncode="510821" if regioncode=="510821" & year==2009

replace regioncode="510822" if regioncode=="510822" & year==2009

replace regioncode="510823" if regioncode=="510823" & year==2009

replace regioncode="510824" if regioncode=="510824" & year==2009

replace regioncode="510902" if regioncode=="510903" & year==2009

replace regioncode="510921" if regioncode=="510921" & year==2009

replace regioncode="510922" if regioncode=="510922" & year==2009

replace regioncode="510923" if regioncode=="510923" & year==2009

replace regioncode="511002" if regioncode=="511002" & year==2009

replace regioncode="511011" if regioncode=="511011" & year==2009

replace regioncode="511024" if regioncode=="511024" & year==2009

replace regioncode="511025" if regioncode=="511025" & year==2009

replace regioncode="511028" if regioncode=="511028" & year==2009

replace regioncode="511102" if regioncode=="511102" & year==2009

replace regioncode="511111" if regioncode=="511111" & year==2009

replace regioncode="511112" if regioncode=="511112" & year==2009

replace regioncode="511113" if regioncode=="511113" & year==2009

replace regioncode="511123" if regioncode=="511123" & year==2009

replace regioncode="511124" if regioncode=="511124" & year==2009

replace regioncode="511126" if regioncode=="511126" & year==2009

replace regioncode="511129" if regioncode=="511129" & year==2009

replace regioncode="511132" if regioncode=="511132" & year==2009

replace regioncode="511133" if regioncode=="511133" & year==2009

replace regioncode="511181" if regioncode=="511181" & year==2009

replace regioncode="511302" if regioncode=="511302" & year==2009

replace regioncode="511303" if regioncode=="511303" & year==2009

replace regioncode="511304" if regioncode=="511304" & year==2009

replace regioncode="511321" if regioncode=="511321" & year==2009

replace regioncode="511322" if regioncode=="511322" & year==2009

replace regioncode="511323" if regioncode=="511323" & year==2009

replace regioncode="511324" if regioncode=="511324" & year==2009

replace regioncode="511325" if regioncode=="511325" & year==2009

replace regioncode="511381" if regioncode=="511381" & year==2009

replace regioncode="511402" if regioncode=="511402" & year==2009

replace regioncode="511421" if regioncode=="511421" & year==2009

replace regioncode="511422" if regioncode=="511422" & year==2009

replace regioncode="511423" if regioncode=="511423" & year==2009

replace regioncode="511424" if regioncode=="511424" & year==2009

replace regioncode="511425" if regioncode=="511425" & year==2009

replace regioncode="511502" if regioncode=="511502" & year==2009

replace regioncode="511521" if regioncode=="511521" & year==2009

replace regioncode="511522" if regioncode=="511522" & year==2009

replace regioncode="511523" if regioncode=="511523" & year==2009

replace regioncode="511524" if regioncode=="511524" & year==2009

replace regioncode="511525" if regioncode=="511525" & year==2009

replace regioncode="511526" if regioncode=="511526" & year==2009

replace regioncode="511527" if regioncode=="511527" & year==2009

replace regioncode="511528" if regioncode=="511528" & year==2009

replace regioncode="511529" if regioncode=="511529" & year==2009

replace regioncode="511602" if regioncode=="511602" & year==2009

replace regioncode="511621" if regioncode=="511621" & year==2009

replace regioncode="511622" if regioncode=="511622" & year==2009

replace regioncode="511623" if regioncode=="511623" & year==2009

replace regioncode="511681" if regioncode=="511681" & year==2009

replace regioncode="511702" if regioncode=="511702" & year==2009

replace regioncode="511721" if regioncode=="511721" & year==2009

replace regioncode="511722" if regioncode=="511722" & year==2009

replace regioncode="511723" if regioncode=="511723" & year==2009

replace regioncode="511724" if regioncode=="511724" & year==2009

replace regioncode="511725" if regioncode=="511725" & year==2009

replace regioncode="511781" if regioncode=="511781" & year==2009

replace regioncode="511802" if regioncode=="511802" & year==2009

replace regioncode="511821" if regioncode=="511821" & year==2009

replace regioncode="511822" if regioncode=="511822" & year==2009

replace regioncode="511823" if regioncode=="511823" & year==2009

replace regioncode="511824" if regioncode=="511824" & year==2009

replace regioncode="511825" if regioncode=="511825" & year==2009

replace regioncode="511826" if regioncode=="511826" & year==2009

replace regioncode="511827" if regioncode=="511827" & year==2009

replace regioncode="511902" if regioncode=="511902" & year==2009

replace regioncode="511921" if regioncode=="511921" & year==2009

replace regioncode="511922" if regioncode=="511922" & year==2009

replace regioncode="511923" if regioncode=="511923" & year==2009

replace regioncode="512002" if regioncode=="512002" & year==2009

replace regioncode="512021" if regioncode=="512021" & year==2009

replace regioncode="512022" if regioncode=="512022" & year==2009

replace regioncode="512081" if regioncode=="512081" & year==2009

replace regioncode="513221" if regioncode=="513221" & year==2009

replace regioncode="513222" if regioncode=="513222" & year==2009

replace regioncode="513223" if regioncode=="513223" & year==2009

replace regioncode="513224" if regioncode=="513224" & year==2009

replace regioncode="513225" if regioncode=="513225" & year==2009

replace regioncode="513226" if regioncode=="513226" & year==2009

replace regioncode="513227" if regioncode=="513227" & year==2009

replace regioncode="513228" if regioncode=="513228" & year==2009

replace regioncode="513229" if regioncode=="513229" & year==2009

replace regioncode="513230" if regioncode=="513230" & year==2009

replace regioncode="513231" if regioncode=="513231" & year==2009

replace regioncode="513232" if regioncode=="513232" & year==2009

replace regioncode="513233" if regioncode=="513233" & year==2009

replace regioncode="513321" if regioncode=="513321" & year==2009

replace regioncode="513322" if regioncode=="513322" & year==2009

replace regioncode="513323" if regioncode=="513323" & year==2009

replace regioncode="513324" if regioncode=="513324" & year==2009

replace regioncode="513325" if regioncode=="513325" & year==2009

replace regioncode="513326" if regioncode=="513326" & year==2009

replace regioncode="513327" if regioncode=="513327" & year==2009

replace regioncode="513328" if regioncode=="513328" & year==2009

replace regioncode="513329" if regioncode=="513329" & year==2009

replace regioncode="513330" if regioncode=="513330" & year==2009

replace regioncode="513331" if regioncode=="513331" & year==2009

replace regioncode="513332" if regioncode=="513332" & year==2009

replace regioncode="513333" if regioncode=="513333" & year==2009

replace regioncode="513334" if regioncode=="513334" & year==2009

replace regioncode="513335" if regioncode=="513335" & year==2009

replace regioncode="513336" if regioncode=="513336" & year==2009

replace regioncode="513337" if regioncode=="513337" & year==2009

replace regioncode="513338" if regioncode=="513338" & year==2009

replace regioncode="513401" if regioncode=="513401" & year==2009

replace regioncode="513422" if regioncode=="513422" & year==2009

replace regioncode="513423" if regioncode=="513423" & year==2009

replace regioncode="513424" if regioncode=="513424" & year==2009

replace regioncode="513425" if regioncode=="513425" & year==2009

replace regioncode="513426" if regioncode=="513426" & year==2009

replace regioncode="513427" if regioncode=="513427" & year==2009

replace regioncode="513428" if regioncode=="513428" & year==2009

replace regioncode="513429" if regioncode=="513429" & year==2009

replace regioncode="513430" if regioncode=="513430" & year==2009

replace regioncode="513431" if regioncode=="513431" & year==2009

replace regioncode="513432" if regioncode=="513432" & year==2009

replace regioncode="513433" if regioncode=="513433" & year==2009

replace regioncode="513434" if regioncode=="513434" & year==2009

replace regioncode="513435" if regioncode=="513435" & year==2009

replace regioncode="513436" if regioncode=="513436" & year==2009

replace regioncode="513437" if regioncode=="513437" & year==2009

replace regioncode="520102" if regioncode=="520102" & year==2009

replace regioncode="520103" if regioncode=="520103" & year==2009

replace regioncode="520111" if regioncode=="520111" & year==2009

replace regioncode="520112" if regioncode=="520112" & year==2009

replace regioncode="520113" if regioncode=="520113" & year==2009

replace regioncode="520114" if regioncode=="520114" & year==2009

replace regioncode="520121" if regioncode=="520121" & year==2009

replace regioncode="520122" if regioncode=="520122" & year==2009

replace regioncode="520123" if regioncode=="520123" & year==2009

replace regioncode="520181" if regioncode=="520181" & year==2009

replace regioncode="520201" if regioncode=="520201" & year==2009

replace regioncode="520222" if regioncode=="520222" & year==2009

replace regioncode="520203" if regioncode=="520203" & year==2009

replace regioncode="520221" if regioncode=="520221" & year==2009

replace regioncode="520302" if regioncode=="520302" & year==2009

replace regioncode="520321" if regioncode=="520321" & year==2009

replace regioncode="520322" if regioncode=="520322" & year==2009

replace regioncode="520323" if regioncode=="520323" & year==2009

replace regioncode="520324" if regioncode=="520324" & year==2009

replace regioncode="520325" if regioncode=="520325" & year==2009

replace regioncode="520326" if regioncode=="520326" & year==2009

replace regioncode="520327" if regioncode=="520327" & year==2009

replace regioncode="520328" if regioncode=="520328" & year==2009

replace regioncode="520329" if regioncode=="520329" & year==2009

replace regioncode="520330" if regioncode=="520330" & year==2009

replace regioncode="520381" if regioncode=="520381" & year==2009

replace regioncode="520382" if regioncode=="520382" & year==2009

replace regioncode="520402" if regioncode=="520402" & year==2009

replace regioncode="520421" if regioncode=="520421" & year==2009

replace regioncode="520422" if regioncode=="520422" & year==2009

replace regioncode="520423" if regioncode=="520423" & year==2009

replace regioncode="520424" if regioncode=="520424" & year==2009

replace regioncode="520425" if regioncode=="520425" & year==2009

replace regioncode="522201" if regioncode=="522201" & year==2009

replace regioncode="522222" if regioncode=="522222" & year==2009

replace regioncode="522223" if regioncode=="522223" & year==2009

replace regioncode="522224" if regioncode=="522224" & year==2009

replace regioncode="522225" if regioncode=="522225" & year==2009

replace regioncode="522226" if regioncode=="522226" & year==2009

replace regioncode="522227" if regioncode=="522227" & year==2009

replace regioncode="522228" if regioncode=="522228" & year==2009

replace regioncode="522229" if regioncode=="522229" & year==2009

replace regioncode="522230" if regioncode=="522230" & year==2009

replace regioncode="522301" if regioncode=="522301" & year==2009

replace regioncode="522322" if regioncode=="522322" & year==2009

replace regioncode="522323" if regioncode=="522323" & year==2009

replace regioncode="522324" if regioncode=="522324" & year==2009

replace regioncode="522325" if regioncode=="522325" & year==2009

replace regioncode="522326" if regioncode=="522326" & year==2009

replace regioncode="522327" if regioncode=="522327" & year==2009

replace regioncode="522328" if regioncode=="522328" & year==2009

replace regioncode="522401" if regioncode=="522401" & year==2009

replace regioncode="522422" if regioncode=="522422" & year==2009

replace regioncode="522423" if regioncode=="522423" & year==2009

replace regioncode="522424" if regioncode=="522424" & year==2009

replace regioncode="522425" if regioncode=="522425" & year==2009

replace regioncode="522426" if regioncode=="522426" & year==2009

replace regioncode="522427" if regioncode=="522427" & year==2009

replace regioncode="522428" if regioncode=="522428" & year==2009

replace regioncode="522601" if regioncode=="522601" & year==2009

replace regioncode="522622" if regioncode=="522622" & year==2009

replace regioncode="522623" if regioncode=="522623" & year==2009

replace regioncode="522624" if regioncode=="522624" & year==2009

replace regioncode="522625" if regioncode=="522625" & year==2009

replace regioncode="522626" if regioncode=="522626" & year==2009

replace regioncode="522627" if regioncode=="522627" & year==2009

replace regioncode="522628" if regioncode=="522628" & year==2009

replace regioncode="522629" if regioncode=="522629" & year==2009

replace regioncode="522630" if regioncode=="522630" & year==2009

replace regioncode="522631" if regioncode=="522631" & year==2009

replace regioncode="522632" if regioncode=="522632" & year==2009

replace regioncode="522633" if regioncode=="522633" & year==2009

replace regioncode="522634" if regioncode=="522634" & year==2009

replace regioncode="522635" if regioncode=="522635" & year==2009

replace regioncode="522636" if regioncode=="522636" & year==2009

replace regioncode="522701" if regioncode=="522701" & year==2009

replace regioncode="522702" if regioncode=="522702" & year==2009

replace regioncode="522722" if regioncode=="522722" & year==2009

replace regioncode="522723" if regioncode=="522723" & year==2009

replace regioncode="522725" if regioncode=="522725" & year==2009

replace regioncode="522726" if regioncode=="522726" & year==2009

replace regioncode="522727" if regioncode=="522727" & year==2009

replace regioncode="522728" if regioncode=="522728" & year==2009

replace regioncode="522729" if regioncode=="522729" & year==2009

replace regioncode="522730" if regioncode=="522730" & year==2009

replace regioncode="522731" if regioncode=="522731" & year==2009

replace regioncode="522732" if regioncode=="522732" & year==2009

replace regioncode="530102" if regioncode=="530102" & year==2009

replace regioncode="530103" if regioncode=="530103" & year==2009

replace regioncode="530111" if regioncode=="530111" & year==2009

replace regioncode="530112" if regioncode=="530112" & year==2009

replace regioncode="530113" if regioncode=="530113" & year==2009

replace regioncode="530121" if regioncode=="530121" & year==2009

replace regioncode="530122" if regioncode=="530122" & year==2009

replace regioncode="530124" if regioncode=="530124" & year==2009

replace regioncode="530125" if regioncode=="530125" & year==2009

replace regioncode="530126" if regioncode=="530126" & year==2009

replace regioncode="530127" if regioncode=="530127" & year==2009

replace regioncode="530128" if regioncode=="530128" & year==2009

replace regioncode="530129" if regioncode=="530129" & year==2009

replace regioncode="530181" if regioncode=="530181" & year==2009

replace regioncode="530302" if regioncode=="530302" & year==2009

replace regioncode="530321" if regioncode=="530321" & year==2009

replace regioncode="530322" if regioncode=="530322" & year==2009

replace regioncode="530323" if regioncode=="530323" & year==2009

replace regioncode="530324" if regioncode=="530324" & year==2009

replace regioncode="530325" if regioncode=="530325" & year==2009

replace regioncode="530326" if regioncode=="530326" & year==2009

replace regioncode="530328" if regioncode=="530328" & year==2009

replace regioncode="530381" if regioncode=="530381" & year==2009

replace regioncode="530402" if regioncode=="530402" & year==2009

replace regioncode="530421" if regioncode=="530421" & year==2009

replace regioncode="530422" if regioncode=="530422" & year==2009

replace regioncode="530423" if regioncode=="530423" & year==2009

replace regioncode="530424" if regioncode=="530424" & year==2009

replace regioncode="530425" if regioncode=="530425" & year==2009

replace regioncode="530426" if regioncode=="530426" & year==2009

replace regioncode="530427" if regioncode=="530427" & year==2009

replace regioncode="530428" if regioncode=="530428" & year==2009

replace regioncode="530502" if regioncode=="530502" & year==2009

replace regioncode="530521" if regioncode=="530521" & year==2009

replace regioncode="530522" if regioncode=="530522" & year==2009

replace regioncode="530523" if regioncode=="530523" & year==2009

replace regioncode="530524" if regioncode=="530524" & year==2009

replace regioncode="530602" if regioncode=="530602" & year==2009

replace regioncode="530621" if regioncode=="530621" & year==2009

replace regioncode="530622" if regioncode=="530622" & year==2009

replace regioncode="530623" if regioncode=="530623" & year==2009

replace regioncode="530624" if regioncode=="530624" & year==2009

replace regioncode="530625" if regioncode=="530625" & year==2009

replace regioncode="530626" if regioncode=="530626" & year==2009

replace regioncode="530627" if regioncode=="530627" & year==2009

replace regioncode="530628" if regioncode=="530628" & year==2009

replace regioncode="530629" if regioncode=="530629" & year==2009

replace regioncode="530630" if regioncode=="530630" & year==2009

replace regioncode="530721" if regioncode=="530721" & year==2009

replace regioncode="530722" if regioncode=="530722" & year==2009

replace regioncode="530723" if regioncode=="530723" & year==2009

replace regioncode="530724" if regioncode=="530724" & year==2009

replace regioncode="532701" if regioncode=="530802" & year==2009

replace regioncode="532722" if regioncode=="530821" & year==2009

replace regioncode="532723" if regioncode=="530822" & year==2009

replace regioncode="532724" if regioncode=="530823" & year==2009

replace regioncode="532725" if regioncode=="530824" & year==2009

replace regioncode="532726" if regioncode=="530825" & year==2009

replace regioncode="532727" if regioncode=="530826" & year==2009

replace regioncode="532728" if regioncode=="530827" & year==2009

replace regioncode="532729" if regioncode=="530828" & year==2009

replace regioncode="532730" if regioncode=="530829" & year==2009

replace regioncode="533521" if regioncode=="530902" & year==2009

replace regioncode="533522" if regioncode=="530921" & year==2009

replace regioncode="533523" if regioncode=="530922" & year==2009

replace regioncode="533524" if regioncode=="530923" & year==2009

replace regioncode="533525" if regioncode=="530924" & year==2009

replace regioncode="533526" if regioncode=="530925" & year==2009

replace regioncode="533527" if regioncode=="530926" & year==2009

replace regioncode="533528" if regioncode=="530927" & year==2009

replace regioncode="532301" if regioncode=="532301" & year==2009

replace regioncode="532322" if regioncode=="532322" & year==2009

replace regioncode="532323" if regioncode=="532323" & year==2009

replace regioncode="532324" if regioncode=="532324" & year==2009

replace regioncode="532325" if regioncode=="532325" & year==2009

replace regioncode="532326" if regioncode=="532326" & year==2009

replace regioncode="532327" if regioncode=="532327" & year==2009

replace regioncode="532328" if regioncode=="532328" & year==2009

replace regioncode="532329" if regioncode=="532329" & year==2009

replace regioncode="532331" if regioncode=="532331" & year==2009

replace regioncode="532501" if regioncode=="532501" & year==2009

replace regioncode="532502" if regioncode=="532502" & year==2009

replace regioncode="532522" if regioncode=="532522" & year==2009

replace regioncode="532523" if regioncode=="532523" & year==2009

replace regioncode="532524" if regioncode=="532524" & year==2009

replace regioncode="532525" if regioncode=="532525" & year==2009

replace regioncode="532526" if regioncode=="532526" & year==2009

replace regioncode="532527" if regioncode=="532527" & year==2009

replace regioncode="532528" if regioncode=="532528" & year==2009

replace regioncode="532529" if regioncode=="532529" & year==2009

replace regioncode="532530" if regioncode=="532530" & year==2009

replace regioncode="532531" if regioncode=="532531" & year==2009

replace regioncode="532532" if regioncode=="532532" & year==2009

replace regioncode="532621" if regioncode=="532621" & year==2009

replace regioncode="532622" if regioncode=="532622" & year==2009

replace regioncode="532623" if regioncode=="532623" & year==2009

replace regioncode="532624" if regioncode=="532624" & year==2009

replace regioncode="532625" if regioncode=="532625" & year==2009

replace regioncode="532626" if regioncode=="532626" & year==2009

replace regioncode="532627" if regioncode=="532627" & year==2009

replace regioncode="532628" if regioncode=="532628" & year==2009

replace regioncode="532801" if regioncode=="532801" & year==2009

replace regioncode="532822" if regioncode=="532822" & year==2009

replace regioncode="532823" if regioncode=="532823" & year==2009

replace regioncode="532901" if regioncode=="532901" & year==2009

replace regioncode="532922" if regioncode=="532922" & year==2009

replace regioncode="532923" if regioncode=="532923" & year==2009

replace regioncode="532924" if regioncode=="532924" & year==2009

replace regioncode="532925" if regioncode=="532925" & year==2009

replace regioncode="532926" if regioncode=="532926" & year==2009

replace regioncode="532927" if regioncode=="532927" & year==2009

replace regioncode="532928" if regioncode=="532928" & year==2009

replace regioncode="532929" if regioncode=="532929" & year==2009

replace regioncode="532930" if regioncode=="532930" & year==2009

replace regioncode="532931" if regioncode=="532931" & year==2009

replace regioncode="532932" if regioncode=="532932" & year==2009

replace regioncode="533102" if regioncode=="533102" & year==2009

replace regioncode="533103" if regioncode=="533103" & year==2009

replace regioncode="533122" if regioncode=="533122" & year==2009

replace regioncode="533123" if regioncode=="533123" & year==2009

replace regioncode="533124" if regioncode=="533124" & year==2009

replace regioncode="533321" if regioncode=="533321" & year==2009

replace regioncode="533323" if regioncode=="533323" & year==2009

replace regioncode="533324" if regioncode=="533324" & year==2009

replace regioncode="533325" if regioncode=="533325" & year==2009

replace regioncode="533421" if regioncode=="533421" & year==2009

replace regioncode="533422" if regioncode=="533422" & year==2009

replace regioncode="533423" if regioncode=="533423" & year==2009

replace regioncode="540102" if regioncode=="540102" & year==2009

replace regioncode="540121" if regioncode=="540121" & year==2009

replace regioncode="540122" if regioncode=="540122" & year==2009

replace regioncode="540123" if regioncode=="540123" & year==2009

replace regioncode="540124" if regioncode=="540124" & year==2009

replace regioncode="540125" if regioncode=="540125" & year==2009

replace regioncode="540126" if regioncode=="540126" & year==2009

replace regioncode="540127" if regioncode=="540127" & year==2009

replace regioncode="542121" if regioncode=="542121" & year==2009

replace regioncode="542122" if regioncode=="542122" & year==2009

replace regioncode="542123" if regioncode=="542123" & year==2009

replace regioncode="542124" if regioncode=="542124" & year==2009

replace regioncode="542125" if regioncode=="542125" & year==2009

replace regioncode="542126" if regioncode=="542126" & year==2009

replace regioncode="542127" if regioncode=="542127" & year==2009

replace regioncode="542128" if regioncode=="542128" & year==2009

replace regioncode="542129" if regioncode=="542129" & year==2009

replace regioncode="542132" if regioncode=="542132" & year==2009

replace regioncode="542133" if regioncode=="542133" & year==2009

replace regioncode="542221" if regioncode=="542221" & year==2009

replace regioncode="542223" if regioncode=="542223" & year==2009

replace regioncode="542224" if regioncode=="542224" & year==2009

replace regioncode="542225" if regioncode=="542225" & year==2009

replace regioncode="542227" if regioncode=="542227" & year==2009

replace regioncode="542228" if regioncode=="542228" & year==2009

replace regioncode="542229" if regioncode=="542229" & year==2009

replace regioncode="542231" if regioncode=="542231" & year==2009

replace regioncode="542232" if regioncode=="542232" & year==2009

replace regioncode="542233" if regioncode=="542233" & year==2009

replace regioncode="542301" if regioncode=="542301" & year==2009

replace regioncode="542322" if regioncode=="542322" & year==2009

replace regioncode="542323" if regioncode=="542323" & year==2009

replace regioncode="542324" if regioncode=="542324" & year==2009

replace regioncode="542325" if regioncode=="542325" & year==2009

replace regioncode="542326" if regioncode=="542326" & year==2009

replace regioncode="542327" if regioncode=="542327" & year==2009

replace regioncode="542328" if regioncode=="542328" & year==2009

replace regioncode="542329" if regioncode=="542329" & year==2009

replace regioncode="542330" if regioncode=="542330" & year==2009

replace regioncode="542331" if regioncode=="542331" & year==2009

replace regioncode="542332" if regioncode=="542332" & year==2009

replace regioncode="542334" if regioncode=="542334" & year==2009

replace regioncode="542336" if regioncode=="542336" & year==2009

replace regioncode="542338" if regioncode=="542338" & year==2009

replace regioncode="542421" if regioncode=="542421" & year==2009

replace regioncode="542422" if regioncode=="542422" & year==2009

replace regioncode="542423" if regioncode=="542423" & year==2009

replace regioncode="542424" if regioncode=="542424" & year==2009

replace regioncode="542425" if regioncode=="542425" & year==2009

replace regioncode="542426" if regioncode=="542426" & year==2009

replace regioncode="542427" if regioncode=="542427" & year==2009

replace regioncode="542429" if regioncode=="542429" & year==2009

replace regioncode="542430" if regioncode=="542430" & year==2009

replace regioncode="542523" if regioncode=="542523" & year==2009

replace regioncode="542621" if regioncode=="542621" & year==2009

replace regioncode="542622" if regioncode=="542622" & year==2009

replace regioncode="542623" if regioncode=="542623" & year==2009

replace regioncode="542625" if regioncode=="542625" & year==2009

replace regioncode="542626" if regioncode=="542626" & year==2009

replace regioncode="610102" if regioncode=="610102" & year==2009

replace regioncode="610103" if regioncode=="610103" & year==2009

replace regioncode="610104" if regioncode=="610104" & year==2009

replace regioncode="610111" if regioncode=="610111" & year==2009

replace regioncode="610112" if regioncode=="610112" & year==2009

replace regioncode="610113" if regioncode=="610113" & year==2009

replace regioncode="610114" if regioncode=="610114" & year==2009

replace regioncode="610115" if regioncode=="610115" & year==2009

replace regioncode="610116" if regioncode=="610116" & year==2009

replace regioncode="610122" if regioncode=="610122" & year==2009

replace regioncode="610124" if regioncode=="610124" & year==2009

replace regioncode="610125" if regioncode=="610125" & year==2009

replace regioncode="610126" if regioncode=="610126" & year==2009

replace regioncode="610202" if regioncode=="610202" & year==2009

replace regioncode="610203" if regioncode=="610203" & year==2009

replace regioncode="610204" if regioncode=="610204" & year==2009

replace regioncode="610222" if regioncode=="610222" & year==2009

replace regioncode="610302" if regioncode=="610302" & year==2009

replace regioncode="610303" if regioncode=="610303" & year==2009

replace regioncode="610304" if regioncode=="610304" & year==2009

replace regioncode="610322" if regioncode=="610322" & year==2009

replace regioncode="610323" if regioncode=="610323" & year==2009

replace regioncode="610324" if regioncode=="610324" & year==2009

replace regioncode="610326" if regioncode=="610326" & year==2009

replace regioncode="610327" if regioncode=="610327" & year==2009

replace regioncode="610328" if regioncode=="610328" & year==2009

replace regioncode="610329" if regioncode=="610329" & year==2009

replace regioncode="610330" if regioncode=="610330" & year==2009

replace regioncode="610331" if regioncode=="610331" & year==2009

replace regioncode="610402" if regioncode=="610402" & year==2009

replace regioncode="610403" if regioncode=="610403" & year==2009

replace regioncode="610404" if regioncode=="610404" & year==2009

replace regioncode="610422" if regioncode=="610422" & year==2009

replace regioncode="610423" if regioncode=="610423" & year==2009

replace regioncode="610424" if regioncode=="610424" & year==2009

replace regioncode="610425" if regioncode=="610425" & year==2009

replace regioncode="610426" if regioncode=="610426" & year==2009

replace regioncode="610427" if regioncode=="610427" & year==2009

replace regioncode="610428" if regioncode=="610428" & year==2009

replace regioncode="610429" if regioncode=="610429" & year==2009

replace regioncode="610430" if regioncode=="610430" & year==2009

replace regioncode="610431" if regioncode=="610431" & year==2009

replace regioncode="610481" if regioncode=="610481" & year==2009

replace regioncode="610502" if regioncode=="610502" & year==2009

replace regioncode="610521" if regioncode=="610521" & year==2009

replace regioncode="610522" if regioncode=="610522" & year==2009

replace regioncode="610523" if regioncode=="610523" & year==2009

replace regioncode="610524" if regioncode=="610524" & year==2009

replace regioncode="610525" if regioncode=="610525" & year==2009

replace regioncode="610526" if regioncode=="610526" & year==2009

replace regioncode="610527" if regioncode=="610527" & year==2009

replace regioncode="610528" if regioncode=="610528" & year==2009

replace regioncode="610581" if regioncode=="610581" & year==2009

replace regioncode="610582" if regioncode=="610582" & year==2009

replace regioncode="610602" if regioncode=="610602" & year==2009

replace regioncode="610621" if regioncode=="610621" & year==2009

replace regioncode="610622" if regioncode=="610622" & year==2009

replace regioncode="610623" if regioncode=="610623" & year==2009

replace regioncode="610624" if regioncode=="610624" & year==2009

replace regioncode="610625" if regioncode=="610625" & year==2009

replace regioncode="610626" if regioncode=="610626" & year==2009

replace regioncode="610627" if regioncode=="610627" & year==2009

replace regioncode="610628" if regioncode=="610628" & year==2009

replace regioncode="610629" if regioncode=="610629" & year==2009

replace regioncode="610630" if regioncode=="610630" & year==2009

replace regioncode="610631" if regioncode=="610631" & year==2009

replace regioncode="610632" if regioncode=="610632" & year==2009

replace regioncode="610702" if regioncode=="610702" & year==2009

replace regioncode="610721" if regioncode=="610721" & year==2009

replace regioncode="610722" if regioncode=="610722" & year==2009

replace regioncode="610723" if regioncode=="610723" & year==2009

replace regioncode="610724" if regioncode=="610724" & year==2009

replace regioncode="610725" if regioncode=="610725" & year==2009

replace regioncode="610726" if regioncode=="610726" & year==2009

replace regioncode="610727" if regioncode=="610727" & year==2009

replace regioncode="610728" if regioncode=="610728" & year==2009

replace regioncode="610729" if regioncode=="610729" & year==2009

replace regioncode="610730" if regioncode=="610730" & year==2009

replace regioncode="610802" if regioncode=="610802" & year==2009

replace regioncode="610821" if regioncode=="610821" & year==2009

replace regioncode="610822" if regioncode=="610822" & year==2009

replace regioncode="610823" if regioncode=="610823" & year==2009

replace regioncode="610824" if regioncode=="610824" & year==2009

replace regioncode="610825" if regioncode=="610825" & year==2009

replace regioncode="610826" if regioncode=="610826" & year==2009

replace regioncode="610827" if regioncode=="610827" & year==2009

replace regioncode="610828" if regioncode=="610828" & year==2009

replace regioncode="610829" if regioncode=="610829" & year==2009

replace regioncode="610830" if regioncode=="610830" & year==2009

replace regioncode="610831" if regioncode=="610831" & year==2009

replace regioncode="610902" if regioncode=="610902" & year==2009

replace regioncode="610921" if regioncode=="610921" & year==2009

replace regioncode="610922" if regioncode=="610922" & year==2009

replace regioncode="610923" if regioncode=="610923" & year==2009

replace regioncode="610924" if regioncode=="610924" & year==2009

replace regioncode="610925" if regioncode=="610925" & year==2009

replace regioncode="610926" if regioncode=="610926" & year==2009

replace regioncode="610927" if regioncode=="610927" & year==2009

replace regioncode="610928" if regioncode=="610928" & year==2009

replace regioncode="610929" if regioncode=="610929" & year==2009

replace regioncode="611002" if regioncode=="611002" & year==2009

replace regioncode="611021" if regioncode=="611021" & year==2009

replace regioncode="611022" if regioncode=="611022" & year==2009

replace regioncode="611023" if regioncode=="611023" & year==2009

replace regioncode="611024" if regioncode=="611024" & year==2009

replace regioncode="611025" if regioncode=="611025" & year==2009

replace regioncode="611026" if regioncode=="611026" & year==2009

replace regioncode="620102" if regioncode=="620102" & year==2009

replace regioncode="620103" if regioncode=="620103" & year==2009

replace regioncode="620104" if regioncode=="620104" & year==2009

replace regioncode="620105" if regioncode=="620105" & year==2009

replace regioncode="620111" if regioncode=="620111" & year==2009

replace regioncode="620121" if regioncode=="620121" & year==2009

replace regioncode="620122" if regioncode=="620122" & year==2009

replace regioncode="620123" if regioncode=="620123" & year==2009

replace regioncode="620201" if regioncode=="620201" & year==2009

replace regioncode="620302" if regioncode=="620302" & year==2009

replace regioncode="620321" if regioncode=="620321" & year==2009

replace regioncode="620402" if regioncode=="620402" & year==2009

replace regioncode="620403" if regioncode=="620403" & year==2009

replace regioncode="620421" if regioncode=="620421" & year==2009

replace regioncode="620422" if regioncode=="620422" & year==2009

replace regioncode="620423" if regioncode=="620423" & year==2009

replace regioncode="620502" if regioncode=="620502" & year==2009

replace regioncode="620503" if regioncode=="620503" & year==2009

replace regioncode="620521" if regioncode=="620521" & year==2009

replace regioncode="620522" if regioncode=="620522" & year==2009

replace regioncode="620523" if regioncode=="620523" & year==2009

replace regioncode="620524" if regioncode=="620524" & year==2009

replace regioncode="620525" if regioncode=="620525" & year==2009

replace regioncode="622301" if regioncode=="620602" & year==2009

replace regioncode="622322" if regioncode=="620621" & year==2009

replace regioncode="622323" if regioncode=="620622" & year==2009

replace regioncode="622326" if regioncode=="620623" & year==2009

replace regioncode="622201" if regioncode=="620702" & year==2009

replace regioncode="622222" if regioncode=="620721" & year==2009

replace regioncode="622223" if regioncode=="620722" & year==2009

replace regioncode="622224" if regioncode=="620723" & year==2009

replace regioncode="622225" if regioncode=="620724" & year==2009

replace regioncode="622226" if regioncode=="620725" & year==2009

replace regioncode="622701" if regioncode=="620802" & year==2009

replace regioncode="622722" if regioncode=="620821" & year==2009

replace regioncode="622723" if regioncode=="620822" & year==2009

replace regioncode="622724" if regioncode=="620823" & year==2009

replace regioncode="622725" if regioncode=="620824" & year==2009

replace regioncode="622726" if regioncode=="620825" & year==2009

replace regioncode="622727" if regioncode=="620826" & year==2009

replace regioncode="622102" if regioncode=="620902" & year==2009

replace regioncode="622123" if regioncode=="620921" & year==2009

replace regioncode="622126" if regioncode=="620922" & year==2009

replace regioncode="622124" if regioncode=="620923" & year==2009

replace regioncode="622125" if regioncode=="620924" & year==2009

replace regioncode="622101" if regioncode=="620981" & year==2009

replace regioncode="622103" if regioncode=="620982" & year==2009

replace regioncode="622801" if regioncode=="621002" & year==2009

replace regioncode="622821" if regioncode=="621021" & year==2009

replace regioncode="622822" if regioncode=="621022" & year==2009

replace regioncode="622823" if regioncode=="621023" & year==2009

replace regioncode="622824" if regioncode=="621024" & year==2009

replace regioncode="622825" if regioncode=="621025" & year==2009

replace regioncode="622826" if regioncode=="621026" & year==2009

replace regioncode="622827" if regioncode=="621027" & year==2009

replace regioncode="622421" if regioncode=="621102" & year==2009

replace regioncode="622424" if regioncode=="621121" & year==2009

replace regioncode="622425" if regioncode=="621122" & year==2009

replace regioncode="622426" if regioncode=="621123" & year==2009

replace regioncode="622427" if regioncode=="621124" & year==2009

replace regioncode="622428" if regioncode=="621125" & year==2009

replace regioncode="622429" if regioncode=="621126" & year==2009

replace regioncode="622621" if regioncode=="621202" & year==2009

replace regioncode="622624" if regioncode=="621221" & year==2009

replace regioncode="622626" if regioncode=="621222" & year==2009

replace regioncode="622623" if regioncode=="621223" & year==2009

replace regioncode="622625" if regioncode=="621224" & year==2009

replace regioncode="622627" if regioncode=="621225" & year==2009

replace regioncode="622628" if regioncode=="621226" & year==2009

replace regioncode="622630" if regioncode=="621227" & year==2009

replace regioncode="622629" if regioncode=="621228" & year==2009

replace regioncode="622901" if regioncode=="622901" & year==2009

replace regioncode="622921" if regioncode=="622921" & year==2009

replace regioncode="622922" if regioncode=="622922" & year==2009

replace regioncode="622923" if regioncode=="622923" & year==2009

replace regioncode="622924" if regioncode=="622924" & year==2009

replace regioncode="622925" if regioncode=="622925" & year==2009

replace regioncode="622926" if regioncode=="622926" & year==2009

replace regioncode="622927" if regioncode=="622927" & year==2009

replace regioncode="623001" if regioncode=="623001" & year==2009

replace regioncode="623021" if regioncode=="623021" & year==2009

replace regioncode="623022" if regioncode=="623022" & year==2009

replace regioncode="623023" if regioncode=="623023" & year==2009

replace regioncode="623024" if regioncode=="623024" & year==2009

replace regioncode="623025" if regioncode=="623025" & year==2009

replace regioncode="623026" if regioncode=="623026" & year==2009

replace regioncode="623027" if regioncode=="623027" & year==2009

replace regioncode="630102" if regioncode=="630102" & year==2009

replace regioncode="630103" if regioncode=="630103" & year==2009

replace regioncode="630104" if regioncode=="630104" & year==2009

replace regioncode="630105" if regioncode=="630105" & year==2009

replace regioncode="630121" if regioncode=="630121" & year==2009

replace regioncode="630122" if regioncode=="630122" & year==2009

replace regioncode="630123" if regioncode=="630123" & year==2009

replace regioncode="632121" if regioncode=="632121" & year==2009

replace regioncode="632122" if regioncode=="632122" & year==2009

replace regioncode="632123" if regioncode=="632123" & year==2009

replace regioncode="632126" if regioncode=="632126" & year==2009

replace regioncode="632127" if regioncode=="632127" & year==2009

replace regioncode="632128" if regioncode=="632128" & year==2009

replace regioncode="632221" if regioncode=="632221" & year==2009

replace regioncode="632222" if regioncode=="632222" & year==2009

replace regioncode="632223" if regioncode=="632223" & year==2009

replace regioncode="632224" if regioncode=="632224" & year==2009

replace regioncode="632321" if regioncode=="632321" & year==2009

replace regioncode="632322" if regioncode=="632322" & year==2009

replace regioncode="632521" if regioncode=="632521" & year==2009

replace regioncode="632522" if regioncode=="632522" & year==2009

replace regioncode="632523" if regioncode=="632523" & year==2009

replace regioncode="632524" if regioncode=="632524" & year==2009

replace regioncode="632525" if regioncode=="632525" & year==2009

replace regioncode="632621" if regioncode=="632621" & year==2009

replace regioncode="632622" if regioncode=="632622" & year==2009

replace regioncode="632623" if regioncode=="632623" & year==2009

replace regioncode="632624" if regioncode=="632624" & year==2009

replace regioncode="632625" if regioncode=="632625" & year==2009

replace regioncode="632626" if regioncode=="632626" & year==2009

replace regioncode="632721" if regioncode=="632721" & year==2009

replace regioncode="632723" if regioncode=="632723" & year==2009

replace regioncode="632724" if regioncode=="632724" & year==2009

replace regioncode="632725" if regioncode=="632725" & year==2009

replace regioncode="632726" if regioncode=="632726" & year==2009

replace regioncode="632801" if regioncode=="632801" & year==2009

replace regioncode="632802" if regioncode=="632802" & year==2009

replace regioncode="632821" if regioncode=="632821" & year==2009

replace regioncode="632822" if regioncode=="632822" & year==2009

replace regioncode="640104" if regioncode=="640104" & year==2009

replace regioncode="640105" if regioncode=="640105" & year==2009

replace regioncode="640106" if regioncode=="640106" & year==2009

replace regioncode="640121" if regioncode=="640121" & year==2009

replace regioncode="640122" if regioncode=="640122" & year==2009

replace regioncode="640181" if regioncode=="640181" & year==2009

replace regioncode="640202" if regioncode=="640202" & year==2009

replace regioncode="640203" if regioncode=="640205" & year==2009

replace regioncode="640221" if regioncode=="640221" & year==2009

replace regioncode="640302" if regioncode=="640302" & year==2009

replace regioncode="640321" if regioncode=="640502" & year==2009

replace regioncode="640322" if regioncode=="640521" & year==2009

replace regioncode="640323" if regioncode=="640323" & year==2009

replace regioncode="640324" if regioncode=="640324" & year==2009

replace regioncode="640381" if regioncode=="640381" & year==2009

replace regioncode="640402" if regioncode=="640402" & year==2009

replace regioncode="640421" if regioncode=="640522" & year==2009

replace regioncode="640422" if regioncode=="640422" & year==2009

replace regioncode="640423" if regioncode=="640423" & year==2009

replace regioncode="640424" if regioncode=="640424" & year==2009

replace regioncode="640425" if regioncode=="640425" & year==2009

replace regioncode="650102" if regioncode=="650102" & year==2009

replace regioncode="650103" if regioncode=="650103" & year==2009

replace regioncode="650104" if regioncode=="650104" & year==2009

replace regioncode="650105" if regioncode=="650105" & year==2009

replace regioncode="650106" if regioncode=="650106" & year==2009

replace regioncode="650107" if regioncode=="650107" & year==2009

replace regioncode="650108" if regioncode=="650109" & year==2009

replace regioncode="650121" if regioncode=="650121" & year==2009

replace regioncode="650202" if regioncode=="650202" & year==2009

replace regioncode="650203" if regioncode=="650203" & year==2009

replace regioncode="650204" if regioncode=="650204" & year==2009

replace regioncode="650205" if regioncode=="650205" & year==2009

replace regioncode="652101" if regioncode=="652101" & year==2009

replace regioncode="652122" if regioncode=="652122" & year==2009

replace regioncode="652123" if regioncode=="652123" & year==2009

replace regioncode="652201" if regioncode=="652201" & year==2009

replace regioncode="652222" if regioncode=="652222" & year==2009

replace regioncode="652223" if regioncode=="652223" & year==2009

replace regioncode="652301" if regioncode=="652301" & year==2009

replace regioncode="652302" if regioncode=="652302" & year==2009

replace regioncode="652303" if regioncode=="652303" & year==2009

replace regioncode="652323" if regioncode=="652323" & year==2009

replace regioncode="652324" if regioncode=="652324" & year==2009

replace regioncode="652325" if regioncode=="652325" & year==2009

replace regioncode="652327" if regioncode=="652327" & year==2009

replace regioncode="652328" if regioncode=="652328" & year==2009

replace regioncode="652701" if regioncode=="652701" & year==2009

replace regioncode="652722" if regioncode=="652722" & year==2009

replace regioncode="652723" if regioncode=="652723" & year==2009

replace regioncode="652801" if regioncode=="652801" & year==2009

replace regioncode="652822" if regioncode=="652822" & year==2009

replace regioncode="652823" if regioncode=="652823" & year==2009

replace regioncode="652824" if regioncode=="652824" & year==2009

replace regioncode="652825" if regioncode=="652825" & year==2009

replace regioncode="652826" if regioncode=="652826" & year==2009

replace regioncode="652827" if regioncode=="652827" & year==2009

replace regioncode="652828" if regioncode=="652828" & year==2009

replace regioncode="652829" if regioncode=="652829" & year==2009

replace regioncode="652901" if regioncode=="652901" & year==2009

replace regioncode="652922" if regioncode=="652922" & year==2009

replace regioncode="652923" if regioncode=="652923" & year==2009

replace regioncode="652924" if regioncode=="652924" & year==2009

replace regioncode="652925" if regioncode=="652925" & year==2009

replace regioncode="652926" if regioncode=="652926" & year==2009

replace regioncode="652928" if regioncode=="652928" & year==2009

replace regioncode="652929" if regioncode=="652929" & year==2009

replace regioncode="653001" if regioncode=="653001" & year==2009

replace regioncode="653022" if regioncode=="653022" & year==2009

replace regioncode="653023" if regioncode=="653023" & year==2009

replace regioncode="653024" if regioncode=="653024" & year==2009

replace regioncode="653101" if regioncode=="653101" & year==2009

replace regioncode="653121" if regioncode=="653121" & year==2009

replace regioncode="653122" if regioncode=="653122" & year==2009

replace regioncode="653123" if regioncode=="653123" & year==2009

replace regioncode="653124" if regioncode=="653124" & year==2009

replace regioncode="653125" if regioncode=="653125" & year==2009

replace regioncode="653126" if regioncode=="653126" & year==2009

replace regioncode="653127" if regioncode=="653127" & year==2009

replace regioncode="653128" if regioncode=="653128" & year==2009

replace regioncode="653129" if regioncode=="653129" & year==2009

replace regioncode="653130" if regioncode=="653130" & year==2009

replace regioncode="653131" if regioncode=="653131" & year==2009

replace regioncode="653201" if regioncode=="653201" & year==2009

replace regioncode="653221" if regioncode=="653221" & year==2009

replace regioncode="653222" if regioncode=="653222" & year==2009

replace regioncode="653223" if regioncode=="653223" & year==2009

replace regioncode="653224" if regioncode=="653224" & year==2009

replace regioncode="653225" if regioncode=="653225" & year==2009

replace regioncode="653226" if regioncode=="653226" & year==2009

replace regioncode="653227" if regioncode=="653227" & year==2009

replace regioncode="654002" if regioncode=="654002" & year==2009

replace regioncode="654003" if regioncode=="654003" & year==2009

replace regioncode="654021" if regioncode=="654021" & year==2009

replace regioncode="654022" if regioncode=="654022" & year==2009

replace regioncode="654023" if regioncode=="654023" & year==2009

replace regioncode="654024" if regioncode=="654024" & year==2009

replace regioncode="654025" if regioncode=="654025" & year==2009

replace regioncode="654026" if regioncode=="654026" & year==2009

replace regioncode="654027" if regioncode=="654027" & year==2009

replace regioncode="654028" if regioncode=="654028" & year==2009

replace regioncode="654201" if regioncode=="654201" & year==2009

replace regioncode="654202" if regioncode=="654202" & year==2009

replace regioncode="654221" if regioncode=="654221" & year==2009

replace regioncode="654223" if regioncode=="654223" & year==2009

replace regioncode="654224" if regioncode=="654224" & year==2009

replace regioncode="654225" if regioncode=="654225" & year==2009

replace regioncode="654226" if regioncode=="654226" & year==2009

replace regioncode="654301" if regioncode=="654301" & year==2009

replace regioncode="654321" if regioncode=="654321" & year==2009

replace regioncode="654322" if regioncode=="654322" & year==2009

replace regioncode="654323" if regioncode=="654323" & year==2009

replace regioncode="654324" if regioncode=="654324" & year==2009

replace regioncode="654325" if regioncode=="654325" & year==2009

replace regioncode="654326" if regioncode=="654326" & year==2009

replace regioncode="659001" if regioncode=="659001" & year==2009

replace regioncode="110101" if regioncode=="110101" & year==2010

replace regioncode="110102" if regioncode=="110102" & year==2010

replace regioncode="110103" if regioncode=="110103" & year==2010

replace regioncode="110104" if regioncode=="110104" & year==2010

replace regioncode="110105" if regioncode=="110105" & year==2010

replace regioncode="110106" if regioncode=="110106" & year==2010

replace regioncode="110107" if regioncode=="110107" & year==2010

replace regioncode="110108" if regioncode=="110108" & year==2010

replace regioncode="110109" if regioncode=="110109" & year==2010

replace regioncode="110111" if regioncode=="110111" & year==2010

replace regioncode="110112" if regioncode=="110112" & year==2010

replace regioncode="110113" if regioncode=="110113" & year==2010

replace regioncode="110114" if regioncode=="110114" & year==2010

replace regioncode="110115" if regioncode=="110115" & year==2010

replace regioncode="110117" if regioncode=="110117" & year==2010

replace regioncode="110116" if regioncode=="110116" & year==2010

replace regioncode="110228" if regioncode=="110228" & year==2010

replace regioncode="110229" if regioncode=="110229" & year==2010

replace regioncode="120101" if regioncode=="120101" & year==2010

replace regioncode="120102" if regioncode=="120102" & year==2010

replace regioncode="120103" if regioncode=="120103" & year==2010

replace regioncode="120104" if regioncode=="120104" & year==2010

replace regioncode="120105" if regioncode=="120105" & year==2010

replace regioncode="120106" if regioncode=="120106" & year==2010

replace regioncode="120107" if regioncode=="120107" & year==2010

replace regioncode="120108" if regioncode=="120108" & year==2010

replace regioncode="120109" if regioncode=="120109" & year==2010

replace regioncode="120110" if regioncode=="120110" & year==2010

replace regioncode="120111" if regioncode=="120111" & year==2010

replace regioncode="120112" if regioncode=="120112" & year==2010

replace regioncode="120113" if regioncode=="120113" & year==2010

replace regioncode="120114" if regioncode=="120114" & year==2010

replace regioncode="120115" if regioncode=="120115" & year==2010

replace regioncode="120221" if regioncode=="120221" & year==2010

replace regioncode="120223" if regioncode=="120223" & year==2010

replace regioncode="120225" if regioncode=="120225" & year==2010

replace regioncode="130102" if regioncode=="130102" & year==2010

replace regioncode="130103" if regioncode=="130103" & year==2010

replace regioncode="130104" if regioncode=="130104" & year==2010

replace regioncode="130105" if regioncode=="130105" & year==2010

replace regioncode="130107" if regioncode=="130107" & year==2010

replace regioncode="130108" if regioncode=="130108" & year==2010

replace regioncode="130121" if regioncode=="130121" & year==2010

replace regioncode="130123" if regioncode=="130123" & year==2010

replace regioncode="130124" if regioncode=="130124" & year==2010

replace regioncode="130125" if regioncode=="130125" & year==2010

replace regioncode="130126" if regioncode=="130126" & year==2010

replace regioncode="130127" if regioncode=="130127" & year==2010

replace regioncode="130128" if regioncode=="130128" & year==2010

replace regioncode="130129" if regioncode=="130129" & year==2010

replace regioncode="130130" if regioncode=="130130" & year==2010

replace regioncode="130131" if regioncode=="130131" & year==2010

replace regioncode="130132" if regioncode=="130132" & year==2010

replace regioncode="130133" if regioncode=="130133" & year==2010

replace regioncode="130181" if regioncode=="130181" & year==2010

replace regioncode="130182" if regioncode=="130182" & year==2010

replace regioncode="130183" if regioncode=="130183" & year==2010

replace regioncode="130184" if regioncode=="130184" & year==2010

replace regioncode="130185" if regioncode=="130185" & year==2010

replace regioncode="130202" if regioncode=="130202" & year==2010

replace regioncode="130203" if regioncode=="130203" & year==2010

replace regioncode="130204" if regioncode=="130204" & year==2010

replace regioncode="130205" if regioncode=="130205" & year==2010

replace regioncode="130208" if regioncode=="130208" & year==2010

replace regioncode="130223" if regioncode=="130223" & year==2010

replace regioncode="130224" if regioncode=="130224" & year==2010

replace regioncode="130225" if regioncode=="130225" & year==2010

replace regioncode="130227" if regioncode=="130227" & year==2010

replace regioncode="130229" if regioncode=="130229" & year==2010

replace regioncode="130230" if regioncode=="130230" & year==2010

replace regioncode="130281" if regioncode=="130281" & year==2010

replace regioncode="130207" if regioncode=="130207" & year==2010

replace regioncode="130283" if regioncode=="130283" & year==2010

replace regioncode="130302" if regioncode=="130302" & year==2010

replace regioncode="130303" if regioncode=="130303" & year==2010

replace regioncode="130304" if regioncode=="130304" & year==2010

replace regioncode="130321" if regioncode=="130321" & year==2010

replace regioncode="130322" if regioncode=="130322" & year==2010

replace regioncode="130323" if regioncode=="130323" & year==2010

replace regioncode="130324" if regioncode=="130324" & year==2010

replace regioncode="130402" if regioncode=="130402" & year==2010

replace regioncode="130403" if regioncode=="130403" & year==2010

replace regioncode="130404" if regioncode=="130404" & year==2010

replace regioncode="130406" if regioncode=="130406" & year==2010

replace regioncode="130421" if regioncode=="130421" & year==2010

replace regioncode="130423" if regioncode=="130423" & year==2010

replace regioncode="130424" if regioncode=="130424" & year==2010

replace regioncode="130425" if regioncode=="130425" & year==2010

replace regioncode="130426" if regioncode=="130426" & year==2010

replace regioncode="130427" if regioncode=="130427" & year==2010

replace regioncode="130428" if regioncode=="130428" & year==2010

replace regioncode="130429" if regioncode=="130429" & year==2010

replace regioncode="130430" if regioncode=="130430" & year==2010

replace regioncode="130431" if regioncode=="130431" & year==2010

replace regioncode="130432" if regioncode=="130432" & year==2010

replace regioncode="130433" if regioncode=="130433" & year==2010

replace regioncode="130434" if regioncode=="130434" & year==2010

replace regioncode="130435" if regioncode=="130435" & year==2010

replace regioncode="130481" if regioncode=="130481" & year==2010

replace regioncode="130502" if regioncode=="130502" & year==2010

replace regioncode="130503" if regioncode=="130503" & year==2010

replace regioncode="130521" if regioncode=="130521" & year==2010

replace regioncode="130522" if regioncode=="130522" & year==2010

replace regioncode="130523" if regioncode=="130523" & year==2010

replace regioncode="130524" if regioncode=="130524" & year==2010

replace regioncode="130525" if regioncode=="130525" & year==2010

replace regioncode="130526" if regioncode=="130526" & year==2010

replace regioncode="130527" if regioncode=="130527" & year==2010

replace regioncode="130528" if regioncode=="130528" & year==2010

replace regioncode="130529" if regioncode=="130529" & year==2010

replace regioncode="130530" if regioncode=="130530" & year==2010

replace regioncode="130531" if regioncode=="130531" & year==2010

replace regioncode="130532" if regioncode=="130532" & year==2010

replace regioncode="130533" if regioncode=="130533" & year==2010

replace regioncode="130534" if regioncode=="130534" & year==2010

replace regioncode="130535" if regioncode=="130535" & year==2010

replace regioncode="130581" if regioncode=="130581" & year==2010

replace regioncode="130582" if regioncode=="130582" & year==2010

replace regioncode="130602" if regioncode=="130602" & year==2010

replace regioncode="130603" if regioncode=="130603" & year==2010

replace regioncode="130604" if regioncode=="130604" & year==2010

replace regioncode="130621" if regioncode=="130621" & year==2010

replace regioncode="130622" if regioncode=="130622" & year==2010

replace regioncode="130623" if regioncode=="130623" & year==2010

replace regioncode="130624" if regioncode=="130624" & year==2010

replace regioncode="130625" if regioncode=="130625" & year==2010

replace regioncode="130626" if regioncode=="130626" & year==2010

replace regioncode="130627" if regioncode=="130627" & year==2010

replace regioncode="130628" if regioncode=="130628" & year==2010

replace regioncode="130629" if regioncode=="130629" & year==2010

replace regioncode="130630" if regioncode=="130630" & year==2010

replace regioncode="130631" if regioncode=="130631" & year==2010

replace regioncode="130632" if regioncode=="130632" & year==2010

replace regioncode="130633" if regioncode=="130633" & year==2010

replace regioncode="130634" if regioncode=="130634" & year==2010

replace regioncode="130635" if regioncode=="130635" & year==2010

replace regioncode="130636" if regioncode=="130636" & year==2010

replace regioncode="130637" if regioncode=="130637" & year==2010

replace regioncode="130638" if regioncode=="130638" & year==2010

replace regioncode="130681" if regioncode=="130681" & year==2010

replace regioncode="130682" if regioncode=="130682" & year==2010

replace regioncode="130683" if regioncode=="130683" & year==2010

replace regioncode="130684" if regioncode=="130684" & year==2010

replace regioncode="130702" if regioncode=="130702" & year==2010

replace regioncode="130703" if regioncode=="130703" & year==2010

replace regioncode="130705" if regioncode=="130705" & year==2010

replace regioncode="130706" if regioncode=="130706" & year==2010

replace regioncode="130721" if regioncode=="130721" & year==2010

replace regioncode="130722" if regioncode=="130722" & year==2010

replace regioncode="130723" if regioncode=="130723" & year==2010

replace regioncode="130724" if regioncode=="130724" & year==2010

replace regioncode="130725" if regioncode=="130725" & year==2010

replace regioncode="130726" if regioncode=="130726" & year==2010

replace regioncode="130727" if regioncode=="130727" & year==2010

replace regioncode="130728" if regioncode=="130728" & year==2010

replace regioncode="130729" if regioncode=="130729" & year==2010

replace regioncode="130730" if regioncode=="130730" & year==2010

replace regioncode="130731" if regioncode=="130731" & year==2010

replace regioncode="130732" if regioncode=="130732" & year==2010

replace regioncode="130733" if regioncode=="130733" & year==2010

replace regioncode="130802" if regioncode=="130802" & year==2010

replace regioncode="130803" if regioncode=="130803" & year==2010

replace regioncode="130804" if regioncode=="130804" & year==2010

replace regioncode="130821" if regioncode=="130821" & year==2010

replace regioncode="130822" if regioncode=="130822" & year==2010

replace regioncode="130823" if regioncode=="130823" & year==2010

replace regioncode="130824" if regioncode=="130824" & year==2010

replace regioncode="130825" if regioncode=="130825" & year==2010

replace regioncode="130826" if regioncode=="130826" & year==2010

replace regioncode="130827" if regioncode=="130827" & year==2010

replace regioncode="130828" if regioncode=="130828" & year==2010

replace regioncode="130902" if regioncode=="130902" & year==2010

replace regioncode="130903" if regioncode=="130903" & year==2010

replace regioncode="130921" if regioncode=="130921" & year==2010

replace regioncode="130922" if regioncode=="130922" & year==2010

replace regioncode="130923" if regioncode=="130923" & year==2010

replace regioncode="130924" if regioncode=="130924" & year==2010

replace regioncode="130925" if regioncode=="130925" & year==2010

replace regioncode="130926" if regioncode=="130926" & year==2010

replace regioncode="130927" if regioncode=="130927" & year==2010

replace regioncode="130928" if regioncode=="130928" & year==2010

replace regioncode="130929" if regioncode=="130929" & year==2010

replace regioncode="130930" if regioncode=="130930" & year==2010

replace regioncode="130981" if regioncode=="130981" & year==2010

replace regioncode="130982" if regioncode=="130982" & year==2010

replace regioncode="130983" if regioncode=="130983" & year==2010

replace regioncode="130984" if regioncode=="130984" & year==2010

replace regioncode="131002" if regioncode=="131002" & year==2010

replace regioncode="131003" if regioncode=="131003" & year==2010

replace regioncode="131022" if regioncode=="131022" & year==2010

replace regioncode="131023" if regioncode=="131023" & year==2010

replace regioncode="131024" if regioncode=="131024" & year==2010

replace regioncode="131025" if regioncode=="131025" & year==2010

replace regioncode="131026" if regioncode=="131026" & year==2010

replace regioncode="131028" if regioncode=="131028" & year==2010

replace regioncode="131081" if regioncode=="131081" & year==2010

replace regioncode="131082" if regioncode=="131082" & year==2010

replace regioncode="131102" if regioncode=="131102" & year==2010

replace regioncode="131121" if regioncode=="131121" & year==2010

replace regioncode="131122" if regioncode=="131122" & year==2010

replace regioncode="131123" if regioncode=="131123" & year==2010

replace regioncode="131124" if regioncode=="131124" & year==2010

replace regioncode="131125" if regioncode=="131125" & year==2010

replace regioncode="131126" if regioncode=="131126" & year==2010

replace regioncode="131127" if regioncode=="131127" & year==2010

replace regioncode="131128" if regioncode=="131128" & year==2010

replace regioncode="131181" if regioncode=="131181" & year==2010

replace regioncode="131182" if regioncode=="131182" & year==2010

replace regioncode="140105" if regioncode=="140105" & year==2010

replace regioncode="140106" if regioncode=="140106" & year==2010

replace regioncode="140107" if regioncode=="140107" & year==2010

replace regioncode="140108" if regioncode=="140108" & year==2010

replace regioncode="140109" if regioncode=="140109" & year==2010

replace regioncode="140110" if regioncode=="140110" & year==2010

replace regioncode="140121" if regioncode=="140121" & year==2010

replace regioncode="140122" if regioncode=="140122" & year==2010

replace regioncode="140123" if regioncode=="140123" & year==2010

replace regioncode="140181" if regioncode=="140181" & year==2010

replace regioncode="140202" if regioncode=="140202" & year==2010

replace regioncode="140203" if regioncode=="140203" & year==2010

replace regioncode="140211" if regioncode=="140211" & year==2010

replace regioncode="140212" if regioncode=="140212" & year==2010

replace regioncode="140221" if regioncode=="140221" & year==2010

replace regioncode="140222" if regioncode=="140222" & year==2010

replace regioncode="140223" if regioncode=="140223" & year==2010

replace regioncode="140224" if regioncode=="140224" & year==2010

replace regioncode="140225" if regioncode=="140225" & year==2010

replace regioncode="140226" if regioncode=="140226" & year==2010

replace regioncode="140227" if regioncode=="140227" & year==2010

replace regioncode="140302" if regioncode=="140302" & year==2010

replace regioncode="140303" if regioncode=="140303" & year==2010

replace regioncode="140311" if regioncode=="140311" & year==2010

replace regioncode="140321" if regioncode=="140321" & year==2010

replace regioncode="140322" if regioncode=="140322" & year==2010

replace regioncode="140402" if regioncode=="140402" & year==2010

replace regioncode="140411" if regioncode=="140411" & year==2010

replace regioncode="140421" if regioncode=="140421" & year==2010

replace regioncode="140423" if regioncode=="140423" & year==2010

replace regioncode="140424" if regioncode=="140424" & year==2010

replace regioncode="140425" if regioncode=="140425" & year==2010

replace regioncode="140426" if regioncode=="140426" & year==2010

replace regioncode="140427" if regioncode=="140427" & year==2010

replace regioncode="140428" if regioncode=="140428" & year==2010

replace regioncode="140429" if regioncode=="140429" & year==2010

replace regioncode="140430" if regioncode=="140430" & year==2010

replace regioncode="140431" if regioncode=="140431" & year==2010

replace regioncode="140481" if regioncode=="140481" & year==2010

replace regioncode="140502" if regioncode=="140502" & year==2010

replace regioncode="140521" if regioncode=="140521" & year==2010

replace regioncode="140522" if regioncode=="140522" & year==2010

replace regioncode="140524" if regioncode=="140524" & year==2010

replace regioncode="140525" if regioncode=="140525" & year==2010

replace regioncode="140581" if regioncode=="140581" & year==2010

replace regioncode="140602" if regioncode=="140602" & year==2010

replace regioncode="140603" if regioncode=="140603" & year==2010

replace regioncode="140621" if regioncode=="140621" & year==2010

replace regioncode="140622" if regioncode=="140622" & year==2010

replace regioncode="140623" if regioncode=="140623" & year==2010

replace regioncode="140624" if regioncode=="140624" & year==2010

replace regioncode="140902" if regioncode=="140902" & year==2010

replace regioncode="140981" if regioncode=="140981" & year==2010

replace regioncode="140921" if regioncode=="140921" & year==2010

replace regioncode="140922" if regioncode=="140922" & year==2010

replace regioncode="140923" if regioncode=="140923" & year==2010

replace regioncode="140924" if regioncode=="140924" & year==2010

replace regioncode="140925" if regioncode=="140925" & year==2010

replace regioncode="140926" if regioncode=="140926" & year==2010

replace regioncode="140927" if regioncode=="140927" & year==2010

replace regioncode="140928" if regioncode=="140928" & year==2010

replace regioncode="140929" if regioncode=="140929" & year==2010

replace regioncode="140930" if regioncode=="140930" & year==2010

replace regioncode="140931" if regioncode=="140931" & year==2010

replace regioncode="140932" if regioncode=="140932" & year==2010

replace regioncode="141181" if regioncode=="141181" & year==2010

replace regioncode="141102" if regioncode=="141102" & year==2010

replace regioncode="141182" if regioncode=="141182" & year==2010

replace regioncode="141121" if regioncode=="141121" & year==2010

replace regioncode="141122" if regioncode=="141122" & year==2010

replace regioncode="141123" if regioncode=="141123" & year==2010

replace regioncode="141124" if regioncode=="141124" & year==2010

replace regioncode="141125" if regioncode=="141125" & year==2010

replace regioncode="141126" if regioncode=="141126" & year==2010

replace regioncode="141127" if regioncode=="141127" & year==2010

replace regioncode="141128" if regioncode=="141128" & year==2010

replace regioncode="141129" if regioncode=="141129" & year==2010

replace regioncode="141130" if regioncode=="141130" & year==2010

replace regioncode="140702" if regioncode=="140702" & year==2010

replace regioncode="140781" if regioncode=="140781" & year==2010

replace regioncode="140721" if regioncode=="140721" & year==2010

replace regioncode="140722" if regioncode=="140722" & year==2010

replace regioncode="140723" if regioncode=="140723" & year==2010

replace regioncode="140724" if regioncode=="140724" & year==2010

replace regioncode="140725" if regioncode=="140725" & year==2010

replace regioncode="140726" if regioncode=="140726" & year==2010

replace regioncode="140727" if regioncode=="140727" & year==2010

replace regioncode="140728" if regioncode=="140728" & year==2010

replace regioncode="140729" if regioncode=="140729" & year==2010

replace regioncode="141002" if regioncode=="141002" & year==2010

replace regioncode="141081" if regioncode=="141081" & year==2010

replace regioncode="141082" if regioncode=="141082" & year==2010

replace regioncode="141021" if regioncode=="141021" & year==2010

replace regioncode="141022" if regioncode=="141022" & year==2010

replace regioncode="141023" if regioncode=="141023" & year==2010

replace regioncode="141024" if regioncode=="141024" & year==2010

replace regioncode="141025" if regioncode=="141025" & year==2010

replace regioncode="141026" if regioncode=="141026" & year==2010

replace regioncode="141027" if regioncode=="141027" & year==2010

replace regioncode="141028" if regioncode=="141028" & year==2010

replace regioncode="141029" if regioncode=="141029" & year==2010

replace regioncode="141033" if regioncode=="141033" & year==2010

replace regioncode="141030" if regioncode=="141030" & year==2010

replace regioncode="141032" if regioncode=="141032" & year==2010

replace regioncode="141031" if regioncode=="141031" & year==2010

replace regioncode="141034" if regioncode=="141034" & year==2010

replace regioncode="140802" if regioncode=="140802" & year==2010

replace regioncode="140881" if regioncode=="140881" & year==2010

replace regioncode="140882" if regioncode=="140882" & year==2010

replace regioncode="140830" if regioncode=="140830" & year==2010

replace regioncode="140821" if regioncode=="140821" & year==2010

replace regioncode="140822" if regioncode=="140822" & year==2010

replace regioncode="140825" if regioncode=="140825" & year==2010

replace regioncode="140824" if regioncode=="140824" & year==2010

replace regioncode="140823" if regioncode=="140823" & year==2010

replace regioncode="140828" if regioncode=="140828" & year==2010

replace regioncode="140826" if regioncode=="140826" & year==2010

replace regioncode="140829" if regioncode=="140829" & year==2010

replace regioncode="140827" if regioncode=="140827" & year==2010

replace regioncode="150102" if regioncode=="150102" & year==2010

replace regioncode="150103" if regioncode=="150103" & year==2010

replace regioncode="150104" if regioncode=="150104" & year==2010

replace regioncode="150105" if regioncode=="150105" & year==2010

replace regioncode="150121" if regioncode=="150121" & year==2010

replace regioncode="150122" if regioncode=="150122" & year==2010

replace regioncode="150123" if regioncode=="150123" & year==2010

replace regioncode="150124" if regioncode=="150124" & year==2010

replace regioncode="150125" if regioncode=="150125" & year==2010

replace regioncode="150202" if regioncode=="150202" & year==2010

replace regioncode="150203" if regioncode=="150203" & year==2010

replace regioncode="150204" if regioncode=="150204" & year==2010

replace regioncode="150205" if regioncode=="150205" & year==2010

replace regioncode="150206" if regioncode=="150206" & year==2010

replace regioncode="150207" if regioncode=="150207" & year==2010

replace regioncode="150221" if regioncode=="150221" & year==2010

replace regioncode="150222" if regioncode=="150222" & year==2010

replace regioncode="150223" if regioncode=="150223" & year==2010

replace regioncode="150302" if regioncode=="150302" & year==2010

replace regioncode="150303" if regioncode=="150303" & year==2010

replace regioncode="150304" if regioncode=="150304" & year==2010

replace regioncode="150402" if regioncode=="150402" & year==2010

replace regioncode="150403" if regioncode=="150403" & year==2010

replace regioncode="150404" if regioncode=="150404" & year==2010

replace regioncode="150421" if regioncode=="150421" & year==2010

replace regioncode="150422" if regioncode=="150422" & year==2010

replace regioncode="150423" if regioncode=="150423" & year==2010

replace regioncode="150424" if regioncode=="150424" & year==2010

replace regioncode="150425" if regioncode=="150425" & year==2010

replace regioncode="150426" if regioncode=="150426" & year==2010

replace regioncode="150428" if regioncode=="150428" & year==2010

replace regioncode="150429" if regioncode=="150429" & year==2010

replace regioncode="150430" if regioncode=="150430" & year==2010

replace regioncode="150702" if regioncode=="150702" & year==2010

replace regioncode="150781" if regioncode=="150781" & year==2010

replace regioncode="150783" if regioncode=="150783" & year==2010

replace regioncode="150782" if regioncode=="150782" & year==2010

replace regioncode="150785" if regioncode=="150785" & year==2010

replace regioncode="150784" if regioncode=="150784" & year==2010

replace regioncode="150721" if regioncode=="150721" & year==2010

replace regioncode="150722" if regioncode=="150722" & year==2010

replace regioncode="150723" if regioncode=="150723" & year==2010

replace regioncode="150724" if regioncode=="150724" & year==2010

replace regioncode="150727" if regioncode=="150727" & year==2010

replace regioncode="150726" if regioncode=="150726" & year==2010

replace regioncode="150725" if regioncode=="150725" & year==2010

replace regioncode="152201" if regioncode=="152201" & year==2010

replace regioncode="152202" if regioncode=="152202" & year==2010

replace regioncode="152221" if regioncode=="152221" & year==2010

replace regioncode="152222" if regioncode=="152222" & year==2010

replace regioncode="152223" if regioncode=="152223" & year==2010

replace regioncode="152224" if regioncode=="152224" & year==2010

replace regioncode="150502" if regioncode=="150502" & year==2010

replace regioncode="150581" if regioncode=="150581" & year==2010

replace regioncode="150521" if regioncode=="150521" & year==2010

replace regioncode="150522" if regioncode=="150522" & year==2010

replace regioncode="150523" if regioncode=="150523" & year==2010

replace regioncode="150524" if regioncode=="150524" & year==2010

replace regioncode="150525" if regioncode=="150525" & year==2010

replace regioncode="150526" if regioncode=="150526" & year==2010

replace regioncode="152501" if regioncode=="152501" & year==2010

replace regioncode="152502" if regioncode=="152502" & year==2010

replace regioncode="152522" if regioncode=="152522" & year==2010

replace regioncode="152523" if regioncode=="152523" & year==2010

replace regioncode="152524" if regioncode=="152524" & year==2010

replace regioncode="152525" if regioncode=="152525" & year==2010

replace regioncode="152526" if regioncode=="152526" & year==2010

replace regioncode="152527" if regioncode=="152527" & year==2010

replace regioncode="152528" if regioncode=="152528" & year==2010

replace regioncode="152529" if regioncode=="152529" & year==2010

replace regioncode="152530" if regioncode=="152530" & year==2010

replace regioncode="152531" if regioncode=="152531" & year==2010

replace regioncode="152601" if regioncode=="150902" & year==2010

replace regioncode="152602" if regioncode=="150981" & year==2010

replace regioncode="152624" if regioncode=="150921" & year==2010

replace regioncode="152625" if regioncode=="150922" & year==2010

replace regioncode="152626" if regioncode=="150923" & year==2010

replace regioncode="152627" if regioncode=="150924" & year==2010

replace regioncode="152629" if regioncode=="150925" & year==2010

replace regioncode="152630" if regioncode=="150926" & year==2010

replace regioncode="152631" if regioncode=="150927" & year==2010

replace regioncode="152632" if regioncode=="150928" & year==2010

replace regioncode="152634" if regioncode=="150929" & year==2010

replace regioncode="150602" if regioncode=="150602" & year==2010

replace regioncode="150621" if regioncode=="150621" & year==2010

replace regioncode="150622" if regioncode=="150622" & year==2010

replace regioncode="150623" if regioncode=="150623" & year==2010

replace regioncode="150624" if regioncode=="150624" & year==2010

replace regioncode="150625" if regioncode=="150625" & year==2010

replace regioncode="150626" if regioncode=="150626" & year==2010

replace regioncode="150627" if regioncode=="150627" & year==2010

replace regioncode="152801" if regioncode=="150802" & year==2010

replace regioncode="152822" if regioncode=="150821" & year==2010

replace regioncode="152823" if regioncode=="150822" & year==2010

replace regioncode="152824" if regioncode=="150823" & year==2010

replace regioncode="152825" if regioncode=="150824" & year==2010

replace regioncode="152826" if regioncode=="150825" & year==2010

replace regioncode="152827" if regioncode=="150826" & year==2010

replace regioncode="152921" if regioncode=="152921" & year==2010

replace regioncode="152922" if regioncode=="152922" & year==2010

replace regioncode="152923" if regioncode=="152923" & year==2010

replace regioncode="210102" if regioncode=="210102" & year==2010

replace regioncode="210103" if regioncode=="210103" & year==2010

replace regioncode="210104" if regioncode=="210104" & year==2010

replace regioncode="210105" if regioncode=="210105" & year==2010

replace regioncode="210106" if regioncode=="210106" & year==2010

replace regioncode="210111" if regioncode=="210111" & year==2010

replace regioncode="210112" if regioncode=="210112" & year==2010

replace regioncode="210113" if regioncode=="210113" & year==2010

replace regioncode="210114" if regioncode=="210114" & year==2010

replace regioncode="210122" if regioncode=="210122" & year==2010

replace regioncode="210123" if regioncode=="210123" & year==2010

replace regioncode="210124" if regioncode=="210124" & year==2010

replace regioncode="210181" if regioncode=="210181" & year==2010

replace regioncode="210202" if regioncode=="210202" & year==2010

replace regioncode="210203" if regioncode=="210203" & year==2010

replace regioncode="210204" if regioncode=="210204" & year==2010

replace regioncode="210211" if regioncode=="210211" & year==2010

replace regioncode="210212" if regioncode=="210212" & year==2010

replace regioncode="210213" if regioncode=="210213" & year==2010

replace regioncode="210224" if regioncode=="210224" & year==2010

replace regioncode="210281" if regioncode=="210281" & year==2010

replace regioncode="210282" if regioncode=="210282" & year==2010

replace regioncode="210283" if regioncode=="210283" & year==2010

replace regioncode="210302" if regioncode=="210302" & year==2010

replace regioncode="210303" if regioncode=="210303" & year==2010

replace regioncode="210304" if regioncode=="210304" & year==2010

replace regioncode="210311" if regioncode=="210311" & year==2010

replace regioncode="210321" if regioncode=="210321" & year==2010

replace regioncode="210323" if regioncode=="210323" & year==2010

replace regioncode="210381" if regioncode=="210381" & year==2010

replace regioncode="210402" if regioncode=="210402" & year==2010

replace regioncode="210403" if regioncode=="210403" & year==2010

replace regioncode="210404" if regioncode=="210404" & year==2010

replace regioncode="210411" if regioncode=="210411" & year==2010

replace regioncode="210421" if regioncode=="210421" & year==2010

replace regioncode="210422" if regioncode=="210422" & year==2010

replace regioncode="210423" if regioncode=="210423" & year==2010

replace regioncode="210502" if regioncode=="210502" & year==2010

replace regioncode="210503" if regioncode=="210503" & year==2010

replace regioncode="210504" if regioncode=="210504" & year==2010

replace regioncode="210505" if regioncode=="210505" & year==2010

replace regioncode="210521" if regioncode=="210521" & year==2010

replace regioncode="210522" if regioncode=="210522" & year==2010

replace regioncode="210602" if regioncode=="210602" & year==2010

replace regioncode="210603" if regioncode=="210603" & year==2010

replace regioncode="210604" if regioncode=="210604" & year==2010

replace regioncode="210624" if regioncode=="210624" & year==2010

replace regioncode="210681" if regioncode=="210681" & year==2010

replace regioncode="210682" if regioncode=="210682" & year==2010

replace regioncode="210702" if regioncode=="210702" & year==2010

replace regioncode="210703" if regioncode=="210703" & year==2010

replace regioncode="210711" if regioncode=="210711" & year==2010

replace regioncode="210726" if regioncode=="210726" & year==2010

replace regioncode="210727" if regioncode=="210727" & year==2010

replace regioncode="210781" if regioncode=="210781" & year==2010

replace regioncode="210782" if regioncode=="210782" & year==2010

replace regioncode="210802" if regioncode=="210802" & year==2010

replace regioncode="210803" if regioncode=="210803" & year==2010

replace regioncode="210804" if regioncode=="210804" & year==2010

replace regioncode="210811" if regioncode=="210811" & year==2010

replace regioncode="210881" if regioncode=="210881" & year==2010

replace regioncode="210882" if regioncode=="210882" & year==2010

replace regioncode="210902" if regioncode=="210902" & year==2010

replace regioncode="210903" if regioncode=="210903" & year==2010

replace regioncode="210904" if regioncode=="210904" & year==2010

replace regioncode="210905" if regioncode=="210905" & year==2010

replace regioncode="210911" if regioncode=="210911" & year==2010

replace regioncode="210921" if regioncode=="210921" & year==2010

replace regioncode="210922" if regioncode=="210922" & year==2010

replace regioncode="211002" if regioncode=="211002" & year==2010

replace regioncode="211003" if regioncode=="211003" & year==2010

replace regioncode="211004" if regioncode=="211004" & year==2010

replace regioncode="211005" if regioncode=="211005" & year==2010

replace regioncode="211011" if regioncode=="211011" & year==2010

replace regioncode="211021" if regioncode=="211021" & year==2010

replace regioncode="211081" if regioncode=="211081" & year==2010

replace regioncode="211102" if regioncode=="211102" & year==2010

replace regioncode="211103" if regioncode=="211103" & year==2010

replace regioncode="211121" if regioncode=="211121" & year==2010

replace regioncode="211122" if regioncode=="211122" & year==2010

replace regioncode="211202" if regioncode=="211202" & year==2010

replace regioncode="211204" if regioncode=="211204" & year==2010

replace regioncode="211221" if regioncode=="211221" & year==2010

replace regioncode="211223" if regioncode=="211223" & year==2010

replace regioncode="211224" if regioncode=="211224" & year==2010

replace regioncode="211281" if regioncode=="211281" & year==2010

replace regioncode="211282" if regioncode=="211282" & year==2010

replace regioncode="211302" if regioncode=="211302" & year==2010

replace regioncode="211303" if regioncode=="211303" & year==2010

replace regioncode="211321" if regioncode=="211321" & year==2010

replace regioncode="211322" if regioncode=="211322" & year==2010

replace regioncode="211324" if regioncode=="211324" & year==2010

replace regioncode="211381" if regioncode=="211381" & year==2010

replace regioncode="211382" if regioncode=="211382" & year==2010

replace regioncode="211402" if regioncode=="211402" & year==2010

replace regioncode="211403" if regioncode=="211403" & year==2010

replace regioncode="211404" if regioncode=="211404" & year==2010

replace regioncode="211421" if regioncode=="211421" & year==2010

replace regioncode="211422" if regioncode=="211422" & year==2010

replace regioncode="211481" if regioncode=="211481" & year==2010

replace regioncode="220102" if regioncode=="220102" & year==2010

replace regioncode="220103" if regioncode=="220103" & year==2010

replace regioncode="220104" if regioncode=="220104" & year==2010

replace regioncode="220105" if regioncode=="220105" & year==2010

replace regioncode="220106" if regioncode=="220106" & year==2010

replace regioncode="220112" if regioncode=="220112" & year==2010

replace regioncode="220122" if regioncode=="220122" & year==2010

replace regioncode="220181" if regioncode=="220181" & year==2010

replace regioncode="220182" if regioncode=="220182" & year==2010

replace regioncode="220183" if regioncode=="220183" & year==2010

replace regioncode="220202" if regioncode=="220202" & year==2010

replace regioncode="220203" if regioncode=="220203" & year==2010

replace regioncode="220204" if regioncode=="220204" & year==2010

replace regioncode="220211" if regioncode=="220211" & year==2010

replace regioncode="220221" if regioncode=="220221" & year==2010

replace regioncode="220281" if regioncode=="220281" & year==2010

replace regioncode="220282" if regioncode=="220282" & year==2010

replace regioncode="220283" if regioncode=="220283" & year==2010

replace regioncode="220284" if regioncode=="220284" & year==2010

replace regioncode="220302" if regioncode=="220302" & year==2010

replace regioncode="220303" if regioncode=="220303" & year==2010

replace regioncode="220322" if regioncode=="220322" & year==2010

replace regioncode="220323" if regioncode=="220323" & year==2010

replace regioncode="220381" if regioncode=="220381" & year==2010

replace regioncode="220382" if regioncode=="220382" & year==2010

replace regioncode="220402" if regioncode=="220402" & year==2010

replace regioncode="220403" if regioncode=="220403" & year==2010

replace regioncode="220421" if regioncode=="220421" & year==2010

replace regioncode="220422" if regioncode=="220422" & year==2010

replace regioncode="220502" if regioncode=="220502" & year==2010

replace regioncode="220503" if regioncode=="220503" & year==2010

replace regioncode="220521" if regioncode=="220521" & year==2010

replace regioncode="220523" if regioncode=="220523" & year==2010

replace regioncode="220524" if regioncode=="220524" & year==2010

replace regioncode="220581" if regioncode=="220581" & year==2010

replace regioncode="220582" if regioncode=="220582" & year==2010

replace regioncode="220602" if regioncode=="220602" & year==2010

replace regioncode="220621" if regioncode=="220621" & year==2010

replace regioncode="220622" if regioncode=="220622" & year==2010

replace regioncode="220623" if regioncode=="220623" & year==2010

replace regioncode="220625" if regioncode=="220625" & year==2010

replace regioncode="220681" if regioncode=="220681" & year==2010

replace regioncode="220702" if regioncode=="220702" & year==2010

replace regioncode="220721" if regioncode=="220721" & year==2010

replace regioncode="220722" if regioncode=="220722" & year==2010

replace regioncode="220723" if regioncode=="220723" & year==2010

replace regioncode="220724" if regioncode=="220724" & year==2010

replace regioncode="220802" if regioncode=="220802" & year==2010

replace regioncode="220821" if regioncode=="220821" & year==2010

replace regioncode="220822" if regioncode=="220822" & year==2010

replace regioncode="220881" if regioncode=="220881" & year==2010

replace regioncode="220882" if regioncode=="220882" & year==2010

replace regioncode="222401" if regioncode=="222401" & year==2010

replace regioncode="222402" if regioncode=="222402" & year==2010

replace regioncode="222403" if regioncode=="222403" & year==2010

replace regioncode="222404" if regioncode=="222404" & year==2010

replace regioncode="222405" if regioncode=="222405" & year==2010

replace regioncode="222406" if regioncode=="222406" & year==2010

replace regioncode="222424" if regioncode=="222424" & year==2010

replace regioncode="222426" if regioncode=="222426" & year==2010

replace regioncode="230102" if regioncode=="230102" & year==2010

replace regioncode="230103" if regioncode=="230103" & year==2010

replace regioncode="230104" if regioncode=="230104" & year==2010

replace regioncode="230105" if regioncode=="230105" & year==2010

replace regioncode="230107" if regioncode=="230110" & year==2010

replace regioncode="230108" if regioncode=="230108" & year==2010

replace regioncode="230121" if regioncode=="230111" & year==2010

replace regioncode="230123" if regioncode=="230123" & year==2010

replace regioncode="230124" if regioncode=="230124" & year==2010

replace regioncode="230125" if regioncode=="230125" & year==2010

replace regioncode="230126" if regioncode=="230126" & year==2010

replace regioncode="230127" if regioncode=="230127" & year==2010

replace regioncode="230128" if regioncode=="230128" & year==2010

replace regioncode="230129" if regioncode=="230129" & year==2010

replace regioncode="230181" if regioncode=="230112" & year==2010

replace regioncode="230182" if regioncode=="230182" & year==2010

replace regioncode="230183" if regioncode=="230183" & year==2010

replace regioncode="230184" if regioncode=="230184" & year==2010

replace regioncode="230202" if regioncode=="230202" & year==2010

replace regioncode="230203" if regioncode=="230203" & year==2010

replace regioncode="230204" if regioncode=="230204" & year==2010

replace regioncode="230205" if regioncode=="230205" & year==2010

replace regioncode="230206" if regioncode=="230206" & year==2010

replace regioncode="230207" if regioncode=="230207" & year==2010

replace regioncode="230208" if regioncode=="230208" & year==2010

replace regioncode="230221" if regioncode=="230221" & year==2010

replace regioncode="230223" if regioncode=="230223" & year==2010

replace regioncode="230224" if regioncode=="230224" & year==2010

replace regioncode="230225" if regioncode=="230225" & year==2010

replace regioncode="230227" if regioncode=="230227" & year==2010

replace regioncode="230229" if regioncode=="230229" & year==2010

replace regioncode="230230" if regioncode=="230230" & year==2010

replace regioncode="230231" if regioncode=="230231" & year==2010

replace regioncode="230281" if regioncode=="230281" & year==2010

replace regioncode="230302" if regioncode=="230302" & year==2010

replace regioncode="230303" if regioncode=="230303" & year==2010

replace regioncode="230304" if regioncode=="230304" & year==2010

replace regioncode="230305" if regioncode=="230305" & year==2010

replace regioncode="230306" if regioncode=="230306" & year==2010

replace regioncode="230307" if regioncode=="230307" & year==2010

replace regioncode="230321" if regioncode=="230321" & year==2010

replace regioncode="230381" if regioncode=="230381" & year==2010

replace regioncode="230382" if regioncode=="230382" & year==2010

replace regioncode="230402" if regioncode=="230402" & year==2010

replace regioncode="230403" if regioncode=="230403" & year==2010

replace regioncode="230404" if regioncode=="230404" & year==2010

replace regioncode="230405" if regioncode=="230405" & year==2010

replace regioncode="230406" if regioncode=="230406" & year==2010

replace regioncode="230407" if regioncode=="230407" & year==2010

replace regioncode="230421" if regioncode=="230421" & year==2010

replace regioncode="230422" if regioncode=="230422" & year==2010

replace regioncode="230502" if regioncode=="230502" & year==2010

replace regioncode="230503" if regioncode=="230503" & year==2010

replace regioncode="230505" if regioncode=="230505" & year==2010

replace regioncode="230506" if regioncode=="230506" & year==2010

replace regioncode="230521" if regioncode=="230521" & year==2010

replace regioncode="230522" if regioncode=="230522" & year==2010

replace regioncode="230523" if regioncode=="230523" & year==2010

replace regioncode="230524" if regioncode=="230524" & year==2010

replace regioncode="230602" if regioncode=="230602" & year==2010

replace regioncode="230603" if regioncode=="230603" & year==2010

replace regioncode="230604" if regioncode=="230604" & year==2010

replace regioncode="230605" if regioncode=="230605" & year==2010

replace regioncode="230606" if regioncode=="230606" & year==2010

replace regioncode="230621" if regioncode=="230621" & year==2010

replace regioncode="230622" if regioncode=="230622" & year==2010

replace regioncode="230623" if regioncode=="230623" & year==2010

replace regioncode="230624" if regioncode=="230624" & year==2010

replace regioncode="230702" if regioncode=="230702" & year==2010

replace regioncode="230703" if regioncode=="230703" & year==2010

replace regioncode="230704" if regioncode=="230704" & year==2010

replace regioncode="230705" if regioncode=="230705" & year==2010

replace regioncode="230706" if regioncode=="230706" & year==2010

replace regioncode="230707" if regioncode=="230707" & year==2010

replace regioncode="230708" if regioncode=="230708" & year==2010

replace regioncode="230709" if regioncode=="230709" & year==2010

replace regioncode="230710" if regioncode=="230710" & year==2010

replace regioncode="230711" if regioncode=="230711" & year==2010

replace regioncode="230712" if regioncode=="230712" & year==2010

replace regioncode="230713" if regioncode=="230713" & year==2010

replace regioncode="230714" if regioncode=="230714" & year==2010

replace regioncode="230715" if regioncode=="230715" & year==2010

replace regioncode="230716" if regioncode=="230716" & year==2010

replace regioncode="230722" if regioncode=="230722" & year==2010

replace regioncode="230781" if regioncode=="230781" & year==2010

replace regioncode="230802" if regioncode=="230802" & year==2010

replace regioncode="230803" if regioncode=="230803" & year==2010

replace regioncode="230804" if regioncode=="230804" & year==2010

replace regioncode="230805" if regioncode=="230805" & year==2010

replace regioncode="230811" if regioncode=="230811" & year==2010

replace regioncode="230822" if regioncode=="230822" & year==2010

replace regioncode="230826" if regioncode=="230826" & year==2010

replace regioncode="230828" if regioncode=="230828" & year==2010

replace regioncode="230833" if regioncode=="230833" & year==2010

replace regioncode="230881" if regioncode=="230881" & year==2010

replace regioncode="230882" if regioncode=="230882" & year==2010

replace regioncode="230902" if regioncode=="230902" & year==2010

replace regioncode="230903" if regioncode=="230903" & year==2010

replace regioncode="230904" if regioncode=="230904" & year==2010

replace regioncode="230921" if regioncode=="230921" & year==2010

replace regioncode="231002" if regioncode=="231002" & year==2010

replace regioncode="231003" if regioncode=="231003" & year==2010

replace regioncode="231004" if regioncode=="231004" & year==2010

replace regioncode="231005" if regioncode=="231005" & year==2010

replace regioncode="231024" if regioncode=="231024" & year==2010

replace regioncode="231025" if regioncode=="231025" & year==2010

replace regioncode="231081" if regioncode=="231081" & year==2010

replace regioncode="231083" if regioncode=="231083" & year==2010

replace regioncode="231084" if regioncode=="231084" & year==2010

replace regioncode="231085" if regioncode=="231085" & year==2010

replace regioncode="231102" if regioncode=="231102" & year==2010

replace regioncode="231121" if regioncode=="231121" & year==2010

replace regioncode="231123" if regioncode=="231123" & year==2010

replace regioncode="231124" if regioncode=="231124" & year==2010

replace regioncode="231181" if regioncode=="231181" & year==2010

replace regioncode="231182" if regioncode=="231182" & year==2010

replace regioncode="231202" if regioncode=="231202" & year==2010

replace regioncode="231221" if regioncode=="231221" & year==2010

replace regioncode="231222" if regioncode=="231222" & year==2010

replace regioncode="231223" if regioncode=="231223" & year==2010

replace regioncode="231224" if regioncode=="231224" & year==2010

replace regioncode="231225" if regioncode=="231225" & year==2010

replace regioncode="231226" if regioncode=="231226" & year==2010

replace regioncode="231281" if regioncode=="231281" & year==2010

replace regioncode="231282" if regioncode=="231282" & year==2010

replace regioncode="231283" if regioncode=="231283" & year==2010

replace regioncode="232701" if regioncode=="232701" & year==2010

replace regioncode="232702" if regioncode=="232702" & year==2010

replace regioncode="232703" if regioncode=="232703" & year==2010

replace regioncode="232704" if regioncode=="232704" & year==2010

replace regioncode="232721" if regioncode=="232721" & year==2010

replace regioncode="232722" if regioncode=="232722" & year==2010

replace regioncode="232723" if regioncode=="232723" & year==2010

replace regioncode="310101" if regioncode=="310101" & year==2010

replace regioncode="310103" if regioncode=="310103" & year==2010

replace regioncode="310104" if regioncode=="310104" & year==2010

replace regioncode="310105" if regioncode=="310105" & year==2010

replace regioncode="310106" if regioncode=="310106" & year==2010

replace regioncode="310107" if regioncode=="310107" & year==2010

replace regioncode="310108" if regioncode=="310108" & year==2010

replace regioncode="310109" if regioncode=="310109" & year==2010

replace regioncode="310110" if regioncode=="310110" & year==2010

replace regioncode="310112" if regioncode=="310112" & year==2010

replace regioncode="310113" if regioncode=="310113" & year==2010

replace regioncode="310114" if regioncode=="310114" & year==2010

replace regioncode="310115" if regioncode=="310115" & year==2010

replace regioncode="310116" if regioncode=="310116" & year==2010

replace regioncode="310117" if regioncode=="310117" & year==2010

replace regioncode="310118" if regioncode=="310118" & year==2010

replace regioncode="310119" if regioncode=="310119" & year==2010

replace regioncode="310120" if regioncode=="310120" & year==2010

replace regioncode="310230" if regioncode=="310230" & year==2010

replace regioncode="320102" if regioncode=="320102" & year==2010

replace regioncode="320103" if regioncode=="320103" & year==2010

replace regioncode="320104" if regioncode=="320104" & year==2010

replace regioncode="320105" if regioncode=="320105" & year==2010

replace regioncode="320106" if regioncode=="320106" & year==2010

replace regioncode="320107" if regioncode=="320107" & year==2010

replace regioncode="320111" if regioncode=="320111" & year==2010

replace regioncode="320113" if regioncode=="320113" & year==2010

replace regioncode="320114" if regioncode=="320114" & year==2010

replace regioncode="320115" if regioncode=="320115" & year==2010

replace regioncode="320116" if regioncode=="320116" & year==2010

replace regioncode="320124" if regioncode=="320124" & year==2010

replace regioncode="320125" if regioncode=="320125" & year==2010

replace regioncode="320202" if regioncode=="320202" & year==2010

replace regioncode="320203" if regioncode=="320203" & year==2010

replace regioncode="320204" if regioncode=="320204" & year==2010

replace regioncode="320205" if regioncode=="320205" & year==2010

replace regioncode="320211" if regioncode=="320211" & year==2010

replace regioncode="320281" if regioncode=="320281" & year==2010

replace regioncode="320282" if regioncode=="320282" & year==2010

replace regioncode="320302" if regioncode=="320302" & year==2010

replace regioncode="320303" if regioncode=="320303" & year==2010

replace regioncode="320304" if regioncode=="320304" & year==2010

replace regioncode="320305" if regioncode=="320305" & year==2010

replace regioncode="320311" if regioncode=="320311" & year==2010

replace regioncode="320321" if regioncode=="320321" & year==2010

replace regioncode="320322" if regioncode=="320322" & year==2010

replace regioncode="320323" if regioncode=="320323" & year==2010

replace regioncode="320324" if regioncode=="320324" & year==2010

replace regioncode="320381" if regioncode=="320381" & year==2010

replace regioncode="320382" if regioncode=="320382" & year==2010

replace regioncode="320402" if regioncode=="320402" & year==2010

replace regioncode="320404" if regioncode=="320404" & year==2010

replace regioncode="320405" if regioncode=="320405" & year==2010

replace regioncode="320411" if regioncode=="320411" & year==2010

replace regioncode="320412" if regioncode=="320412" & year==2010

replace regioncode="320481" if regioncode=="320481" & year==2010

replace regioncode="320482" if regioncode=="320482" & year==2010

replace regioncode="320502" if regioncode=="320502" & year==2010

replace regioncode="320503" if regioncode=="320503" & year==2010

replace regioncode="320504" if regioncode=="320504" & year==2010

replace regioncode="320505" if regioncode=="320505" & year==2010

replace regioncode="320581" if regioncode=="320581" & year==2010

replace regioncode="320582" if regioncode=="320582" & year==2010

replace regioncode="320583" if regioncode=="320583" & year==2010

replace regioncode="320584" if regioncode=="320584" & year==2010

replace regioncode="320585" if regioncode=="320585" & year==2010

replace regioncode="320586" if regioncode=="320586" & year==2010

replace regioncode="320602" if regioncode=="320602" & year==2010

replace regioncode="320611" if regioncode=="320611" & year==2010

replace regioncode="320621" if regioncode=="320621" & year==2010

replace regioncode="320623" if regioncode=="320623" & year==2010

replace regioncode="320681" if regioncode=="320681" & year==2010

replace regioncode="320682" if regioncode=="320682" & year==2010

replace regioncode="320683" if regioncode=="320683" & year==2010

replace regioncode="320684" if regioncode=="320684" & year==2010

replace regioncode="320703" if regioncode=="320703" & year==2010

replace regioncode="320705" if regioncode=="320705" & year==2010

replace regioncode="320706" if regioncode=="320706" & year==2010

replace regioncode="320721" if regioncode=="320721" & year==2010

replace regioncode="320722" if regioncode=="320722" & year==2010

replace regioncode="320723" if regioncode=="320723" & year==2010

replace regioncode="320724" if regioncode=="320724" & year==2010

replace regioncode="320802" if regioncode=="320802" & year==2010

replace regioncode="320803" if regioncode=="320803" & year==2010

replace regioncode="320804" if regioncode=="320804" & year==2010

replace regioncode="320811" if regioncode=="320811" & year==2010

replace regioncode="320826" if regioncode=="320826" & year==2010

replace regioncode="320829" if regioncode=="320829" & year==2010

replace regioncode="320830" if regioncode=="320830" & year==2010

replace regioncode="320831" if regioncode=="320831" & year==2010

replace regioncode="320902" if regioncode=="320902" & year==2010

replace regioncode="320903" if regioncode=="320903" & year==2010

replace regioncode="320921" if regioncode=="320921" & year==2010

replace regioncode="320922" if regioncode=="320922" & year==2010

replace regioncode="320923" if regioncode=="320923" & year==2010

replace regioncode="320924" if regioncode=="320924" & year==2010

replace regioncode="320925" if regioncode=="320925" & year==2010

replace regioncode="320981" if regioncode=="320981" & year==2010

replace regioncode="320982" if regioncode=="320982" & year==2010

replace regioncode="321002" if regioncode=="321002" & year==2010

replace regioncode="321003" if regioncode=="321003" & year==2010

replace regioncode="321011" if regioncode=="321011" & year==2010

replace regioncode="321023" if regioncode=="321023" & year==2010

replace regioncode="321081" if regioncode=="321081" & year==2010

replace regioncode="321084" if regioncode=="321084" & year==2010

replace regioncode="321088" if regioncode=="321088" & year==2010

replace regioncode="321102" if regioncode=="321102" & year==2010

replace regioncode="321111" if regioncode=="321111" & year==2010

replace regioncode="321112" if regioncode=="321112" & year==2010

replace regioncode="321181" if regioncode=="321181" & year==2010

replace regioncode="321182" if regioncode=="321182" & year==2010

replace regioncode="321183" if regioncode=="321183" & year==2010

replace regioncode="321202" if regioncode=="321202" & year==2010

replace regioncode="321203" if regioncode=="321203" & year==2010

replace regioncode="321281" if regioncode=="321281" & year==2010

replace regioncode="321282" if regioncode=="321282" & year==2010

replace regioncode="321283" if regioncode=="321283" & year==2010

replace regioncode="321284" if regioncode=="321284" & year==2010

replace regioncode="321302" if regioncode=="321302" & year==2010

replace regioncode="321321" if regioncode=="321311" & year==2010

replace regioncode="321322" if regioncode=="321322" & year==2010

replace regioncode="321323" if regioncode=="321323" & year==2010

replace regioncode="321324" if regioncode=="321324" & year==2010

replace regioncode="330102" if regioncode=="330102" & year==2010

replace regioncode="330103" if regioncode=="330103" & year==2010

replace regioncode="330104" if regioncode=="330104" & year==2010

replace regioncode="330105" if regioncode=="330105" & year==2010

replace regioncode="330106" if regioncode=="330106" & year==2010

replace regioncode="330108" if regioncode=="330108" & year==2010

replace regioncode="330109" if regioncode=="330109" & year==2010

replace regioncode="330110" if regioncode=="330110" & year==2010

replace regioncode="330122" if regioncode=="330122" & year==2010

replace regioncode="330127" if regioncode=="330127" & year==2010

replace regioncode="330182" if regioncode=="330182" & year==2010

replace regioncode="330183" if regioncode=="330183" & year==2010

replace regioncode="330185" if regioncode=="330185" & year==2010

replace regioncode="330203" if regioncode=="330203" & year==2010

replace regioncode="330204" if regioncode=="330204" & year==2010

replace regioncode="330205" if regioncode=="330205" & year==2010

replace regioncode="330206" if regioncode=="330206" & year==2010

replace regioncode="330211" if regioncode=="330211" & year==2010

replace regioncode="330212" if regioncode=="330212" & year==2010

replace regioncode="330225" if regioncode=="330225" & year==2010

replace regioncode="330226" if regioncode=="330226" & year==2010

replace regioncode="330281" if regioncode=="330281" & year==2010

replace regioncode="330282" if regioncode=="330282" & year==2010

replace regioncode="330283" if regioncode=="330283" & year==2010

replace regioncode="330302" if regioncode=="330302" & year==2010

replace regioncode="330303" if regioncode=="330303" & year==2010

replace regioncode="330304" if regioncode=="330304" & year==2010

replace regioncode="330322" if regioncode=="330322" & year==2010

replace regioncode="330324" if regioncode=="330324" & year==2010

replace regioncode="330326" if regioncode=="330326" & year==2010

replace regioncode="330327" if regioncode=="330327" & year==2010

replace regioncode="330328" if regioncode=="330328" & year==2010

replace regioncode="330329" if regioncode=="330329" & year==2010

replace regioncode="330381" if regioncode=="330381" & year==2010

replace regioncode="330382" if regioncode=="330382" & year==2010

replace regioncode="330402" if regioncode=="330402" & year==2010

replace regioncode="330411" if regioncode=="330411" & year==2010

replace regioncode="330421" if regioncode=="330421" & year==2010

replace regioncode="330424" if regioncode=="330424" & year==2010

replace regioncode="330481" if regioncode=="330481" & year==2010

replace regioncode="330482" if regioncode=="330482" & year==2010

replace regioncode="330483" if regioncode=="330483" & year==2010

replace regioncode="330501" if regioncode=="330501" & year==2010

replace regioncode="330521" if regioncode=="330521" & year==2010

replace regioncode="330522" if regioncode=="330522" & year==2010

replace regioncode="330523" if regioncode=="330523" & year==2010

replace regioncode="330602" if regioncode=="330602" & year==2010

replace regioncode="330621" if regioncode=="330621" & year==2010

replace regioncode="330624" if regioncode=="330624" & year==2010

replace regioncode="330681" if regioncode=="330681" & year==2010

replace regioncode="330682" if regioncode=="330682" & year==2010

replace regioncode="330683" if regioncode=="330683" & year==2010

replace regioncode="330702" if regioncode=="330702" & year==2010

replace regioncode="330703" if regioncode=="330703" & year==2010

replace regioncode="330723" if regioncode=="330723" & year==2010

replace regioncode="330726" if regioncode=="330726" & year==2010

replace regioncode="330727" if regioncode=="330727" & year==2010

replace regioncode="330781" if regioncode=="330781" & year==2010

replace regioncode="330782" if regioncode=="330782" & year==2010

replace regioncode="330783" if regioncode=="330783" & year==2010

replace regioncode="330784" if regioncode=="330784" & year==2010

replace regioncode="330802" if regioncode=="330802" & year==2010

replace regioncode="330803" if regioncode=="330803" & year==2010

replace regioncode="330822" if regioncode=="330822" & year==2010

replace regioncode="330824" if regioncode=="330824" & year==2010

replace regioncode="330825" if regioncode=="330825" & year==2010

replace regioncode="330881" if regioncode=="330881" & year==2010

replace regioncode="330902" if regioncode=="330902" & year==2010

replace regioncode="330903" if regioncode=="330903" & year==2010

replace regioncode="330921" if regioncode=="330921" & year==2010

replace regioncode="330922" if regioncode=="330922" & year==2010

replace regioncode="331002" if regioncode=="331002" & year==2010

replace regioncode="331003" if regioncode=="331003" & year==2010

replace regioncode="331004" if regioncode=="331004" & year==2010

replace regioncode="331021" if regioncode=="331021" & year==2010

replace regioncode="331022" if regioncode=="331022" & year==2010

replace regioncode="331023" if regioncode=="331023" & year==2010

replace regioncode="331024" if regioncode=="331024" & year==2010

replace regioncode="331081" if regioncode=="331081" & year==2010

replace regioncode="331082" if regioncode=="331082" & year==2010

replace regioncode="331102" if regioncode=="331102" & year==2010

replace regioncode="331121" if regioncode=="331121" & year==2010

replace regioncode="331122" if regioncode=="331122" & year==2010

replace regioncode="331123" if regioncode=="331123" & year==2010

replace regioncode="331124" if regioncode=="331124" & year==2010

replace regioncode="331125" if regioncode=="331125" & year==2010

replace regioncode="331126" if regioncode=="331126" & year==2010

replace regioncode="331127" if regioncode=="331127" & year==2010

replace regioncode="331181" if regioncode=="331181" & year==2010

replace regioncode="340102" if regioncode=="340102" & year==2010

replace regioncode="340103" if regioncode=="340103" & year==2010

replace regioncode="340104" if regioncode=="340104" & year==2010

replace regioncode="340111" if regioncode=="340111" & year==2010

replace regioncode="340121" if regioncode=="340121" & year==2010

replace regioncode="340122" if regioncode=="340122" & year==2010

replace regioncode="340123" if regioncode=="340123" & year==2010

replace regioncode="340202" if regioncode=="340202" & year==2010

replace regioncode="340203" if regioncode=="340203" & year==2010

replace regioncode="340204" if regioncode=="340204" & year==2010

replace regioncode="340207" if regioncode=="340207" & year==2010

replace regioncode="340221" if regioncode=="340221" & year==2010

replace regioncode="340222" if regioncode=="340222" & year==2010

replace regioncode="340223" if regioncode=="340223" & year==2010

replace regioncode="340302" if regioncode=="340302" & year==2010

replace regioncode="340303" if regioncode=="340303" & year==2010

replace regioncode="340304" if regioncode=="340304" & year==2010

replace regioncode="340311" if regioncode=="340311" & year==2010

replace regioncode="340321" if regioncode=="340321" & year==2010

replace regioncode="340322" if regioncode=="340322" & year==2010

replace regioncode="340323" if regioncode=="340323" & year==2010

replace regioncode="340402" if regioncode=="340402" & year==2010

replace regioncode="340403" if regioncode=="340403" & year==2010

replace regioncode="340404" if regioncode=="340404" & year==2010

replace regioncode="340405" if regioncode=="340405" & year==2010

replace regioncode="340406" if regioncode=="340406" & year==2010

replace regioncode="340421" if regioncode=="340421" & year==2010

replace regioncode="340502" if regioncode=="340502" & year==2010

replace regioncode="340503" if regioncode=="340503" & year==2010

replace regioncode="340504" if regioncode=="340504" & year==2010

replace regioncode="340521" if regioncode=="340521" & year==2010

replace regioncode="340602" if regioncode=="340602" & year==2010

replace regioncode="340603" if regioncode=="340603" & year==2010

replace regioncode="340604" if regioncode=="340604" & year==2010

replace regioncode="340621" if regioncode=="340621" & year==2010

replace regioncode="340702" if regioncode=="340702" & year==2010

replace regioncode="340703" if regioncode=="340703" & year==2010

replace regioncode="340711" if regioncode=="340711" & year==2010

replace regioncode="340721" if regioncode=="340721" & year==2010

replace regioncode="340802" if regioncode=="340802" & year==2010

replace regioncode="340803" if regioncode=="340803" & year==2010

replace regioncode="340811" if regioncode=="340811" & year==2010

replace regioncode="340822" if regioncode=="340822" & year==2010

replace regioncode="340823" if regioncode=="340823" & year==2010

replace regioncode="340824" if regioncode=="340824" & year==2010

replace regioncode="340825" if regioncode=="340825" & year==2010

replace regioncode="340826" if regioncode=="340826" & year==2010

replace regioncode="340827" if regioncode=="340827" & year==2010

replace regioncode="340828" if regioncode=="340828" & year==2010

replace regioncode="340881" if regioncode=="340881" & year==2010

replace regioncode="341002" if regioncode=="341002" & year==2010

replace regioncode="341003" if regioncode=="341003" & year==2010

replace regioncode="341004" if regioncode=="341004" & year==2010

replace regioncode="341021" if regioncode=="341021" & year==2010

replace regioncode="341022" if regioncode=="341022" & year==2010

replace regioncode="341023" if regioncode=="341023" & year==2010

replace regioncode="341024" if regioncode=="341024" & year==2010

replace regioncode="341102" if regioncode=="341102" & year==2010

replace regioncode="341103" if regioncode=="341103" & year==2010

replace regioncode="341122" if regioncode=="341122" & year==2010

replace regioncode="341124" if regioncode=="341124" & year==2010

replace regioncode="341125" if regioncode=="341125" & year==2010

replace regioncode="341126" if regioncode=="341126" & year==2010

replace regioncode="341181" if regioncode=="341181" & year==2010

replace regioncode="341182" if regioncode=="341182" & year==2010

replace regioncode="341202" if regioncode=="341202" & year==2010

replace regioncode="341203" if regioncode=="341203" & year==2010

replace regioncode="341204" if regioncode=="341204" & year==2010

replace regioncode="341221" if regioncode=="341221" & year==2010

replace regioncode="341222" if regioncode=="341222" & year==2010

replace regioncode="341621" if regioncode=="341621" & year==2010

replace regioncode="341622" if regioncode=="341622" & year==2010

replace regioncode="341225" if regioncode=="341225" & year==2010

replace regioncode="341226" if regioncode=="341226" & year==2010

replace regioncode="341623" if regioncode=="341623" & year==2010

replace regioncode="341282" if regioncode=="341282" & year==2010

replace regioncode="341302" if regioncode=="341302" & year==2010

replace regioncode="341321" if regioncode=="341321" & year==2010

replace regioncode="341322" if regioncode=="341322" & year==2010

replace regioncode="341323" if regioncode=="341323" & year==2010

replace regioncode="341324" if regioncode=="341324" & year==2010

replace regioncode="341402" if regioncode=="341402" & year==2010

replace regioncode="341421" if regioncode=="341421" & year==2010

replace regioncode="341422" if regioncode=="341422" & year==2010

replace regioncode="341423" if regioncode=="341423" & year==2010

replace regioncode="341424" if regioncode=="341424" & year==2010

replace regioncode="341521" if regioncode=="341521" & year==2010

replace regioncode="341522" if regioncode=="341522" & year==2010

replace regioncode="341523" if regioncode=="341523" & year==2010

replace regioncode="341524" if regioncode=="341524" & year==2010

replace regioncode="341525" if regioncode=="341525" & year==2010

replace regioncode="341802" if regioncode=="341802" & year==2010

replace regioncode="341881" if regioncode=="341881" & year==2010

replace regioncode="341821" if regioncode=="341821" & year==2010

replace regioncode="341822" if regioncode=="341822" & year==2010

replace regioncode="341823" if regioncode=="341823" & year==2010

replace regioncode="341825" if regioncode=="341825" & year==2010

replace regioncode="341824" if regioncode=="341824" & year==2010

replace regioncode="341702" if regioncode=="341702" & year==2010

replace regioncode="341721" if regioncode=="341721" & year==2010

replace regioncode="341722" if regioncode=="341722" & year==2010

replace regioncode="341723" if regioncode=="341723" & year==2010

replace regioncode="341502" if regioncode=="341502" & year==2010

replace regioncode="341602" if regioncode=="341602" & year==2010

replace regioncode="350102" if regioncode=="350102" & year==2010

replace regioncode="350103" if regioncode=="350103" & year==2010

replace regioncode="350104" if regioncode=="350104" & year==2010

replace regioncode="350105" if regioncode=="350105" & year==2010

replace regioncode="350111" if regioncode=="350111" & year==2010

replace regioncode="350121" if regioncode=="350121" & year==2010

replace regioncode="350122" if regioncode=="350122" & year==2010

replace regioncode="350123" if regioncode=="350123" & year==2010

replace regioncode="350124" if regioncode=="350124" & year==2010

replace regioncode="350125" if regioncode=="350125" & year==2010

replace regioncode="350128" if regioncode=="350128" & year==2010

replace regioncode="350181" if regioncode=="350181" & year==2010

replace regioncode="350182" if regioncode=="350182" & year==2010

replace regioncode="350203" if regioncode=="350203" & year==2010

replace regioncode="350205" if regioncode=="350205" & year==2010

replace regioncode="350206" if regioncode=="350206" & year==2010

replace regioncode="350211" if regioncode=="350211" & year==2010

replace regioncode="350212" if regioncode=="350212" & year==2010

replace regioncode="350213" if regioncode=="350213" & year==2010

replace regioncode="350302" if regioncode=="350302" & year==2010

replace regioncode="350303" if regioncode=="350303" & year==2010

replace regioncode="350321" if regioncode=="350321" & year==2010

replace regioncode="350322" if regioncode=="350322" & year==2010

replace regioncode="350402" if regioncode=="350402" & year==2010

replace regioncode="350403" if regioncode=="350403" & year==2010

replace regioncode="350421" if regioncode=="350421" & year==2010

replace regioncode="350423" if regioncode=="350423" & year==2010

replace regioncode="350424" if regioncode=="350424" & year==2010

replace regioncode="350425" if regioncode=="350425" & year==2010

replace regioncode="350426" if regioncode=="350426" & year==2010

replace regioncode="350427" if regioncode=="350427" & year==2010

replace regioncode="350428" if regioncode=="350428" & year==2010

replace regioncode="350429" if regioncode=="350429" & year==2010

replace regioncode="350430" if regioncode=="350430" & year==2010

replace regioncode="350481" if regioncode=="350481" & year==2010

replace regioncode="350502" if regioncode=="350502" & year==2010

replace regioncode="350503" if regioncode=="350503" & year==2010

replace regioncode="350504" if regioncode=="350504" & year==2010

replace regioncode="350505" if regioncode=="350505" & year==2010

replace regioncode="350521" if regioncode=="350521" & year==2010

replace regioncode="350524" if regioncode=="350524" & year==2010

replace regioncode="350525" if regioncode=="350525" & year==2010

replace regioncode="350526" if regioncode=="350526" & year==2010

replace regioncode="350581" if regioncode=="350581" & year==2010

replace regioncode="350582" if regioncode=="350582" & year==2010

replace regioncode="350583" if regioncode=="350583" & year==2010

replace regioncode="350602" if regioncode=="350602" & year==2010

replace regioncode="350603" if regioncode=="350603" & year==2010

replace regioncode="350622" if regioncode=="350622" & year==2010

replace regioncode="350623" if regioncode=="350623" & year==2010

replace regioncode="350624" if regioncode=="350624" & year==2010

replace regioncode="350625" if regioncode=="350625" & year==2010

replace regioncode="350626" if regioncode=="350626" & year==2010

replace regioncode="350627" if regioncode=="350627" & year==2010

replace regioncode="350628" if regioncode=="350628" & year==2010

replace regioncode="350629" if regioncode=="350629" & year==2010

replace regioncode="350681" if regioncode=="350681" & year==2010

replace regioncode="350702" if regioncode=="350702" & year==2010

replace regioncode="350721" if regioncode=="350721" & year==2010

replace regioncode="350722" if regioncode=="350722" & year==2010

replace regioncode="350723" if regioncode=="350723" & year==2010

replace regioncode="350724" if regioncode=="350724" & year==2010

replace regioncode="350725" if regioncode=="350725" & year==2010

replace regioncode="350781" if regioncode=="350781" & year==2010

replace regioncode="350782" if regioncode=="350782" & year==2010

replace regioncode="350783" if regioncode=="350783" & year==2010

replace regioncode="350784" if regioncode=="350784" & year==2010

replace regioncode="350802" if regioncode=="350802" & year==2010

replace regioncode="350821" if regioncode=="350821" & year==2010

replace regioncode="350822" if regioncode=="350822" & year==2010

replace regioncode="350823" if regioncode=="350823" & year==2010

replace regioncode="350824" if regioncode=="350824" & year==2010

replace regioncode="350825" if regioncode=="350825" & year==2010

replace regioncode="350881" if regioncode=="350881" & year==2010

replace regioncode="350902" if regioncode=="350902" & year==2010

replace regioncode="350981" if regioncode=="350981" & year==2010

replace regioncode="350982" if regioncode=="350982" & year==2010

replace regioncode="350921" if regioncode=="350921" & year==2010

replace regioncode="350922" if regioncode=="350922" & year==2010

replace regioncode="350923" if regioncode=="350923" & year==2010

replace regioncode="350924" if regioncode=="350924" & year==2010

replace regioncode="350925" if regioncode=="350925" & year==2010

replace regioncode="350926" if regioncode=="350926" & year==2010

replace regioncode="360102" if regioncode=="360102" & year==2010

replace regioncode="360103" if regioncode=="360103" & year==2010

replace regioncode="360104" if regioncode=="360104" & year==2010

replace regioncode="360105" if regioncode=="360105" & year==2010

replace regioncode="360111" if regioncode=="360111" & year==2010

replace regioncode="360121" if regioncode=="360121" & year==2010

replace regioncode="360122" if regioncode=="360122" & year==2010

replace regioncode="360123" if regioncode=="360123" & year==2010

replace regioncode="360124" if regioncode=="360124" & year==2010

replace regioncode="360202" if regioncode=="360202" & year==2010

replace regioncode="360203" if regioncode=="360203" & year==2010

replace regioncode="360222" if regioncode=="360222" & year==2010

replace regioncode="360281" if regioncode=="360281" & year==2010

replace regioncode="360302" if regioncode=="360302" & year==2010

replace regioncode="360313" if regioncode=="360313" & year==2010

replace regioncode="360321" if regioncode=="360321" & year==2010

replace regioncode="360322" if regioncode=="360322" & year==2010

replace regioncode="360323" if regioncode=="360323" & year==2010

replace regioncode="360402" if regioncode=="360402" & year==2010

replace regioncode="360403" if regioncode=="360403" & year==2010

replace regioncode="360421" if regioncode=="360421" & year==2010

replace regioncode="360423" if regioncode=="360423" & year==2010

replace regioncode="360424" if regioncode=="360424" & year==2010

replace regioncode="360425" if regioncode=="360425" & year==2010

replace regioncode="360426" if regioncode=="360426" & year==2010

replace regioncode="360427" if regioncode=="360427" & year==2010

replace regioncode="360428" if regioncode=="360428" & year==2010

replace regioncode="360429" if regioncode=="360429" & year==2010

replace regioncode="360430" if regioncode=="360430" & year==2010

replace regioncode="360481" if regioncode=="360481" & year==2010

replace regioncode="360502" if regioncode=="360502" & year==2010

replace regioncode="360521" if regioncode=="360521" & year==2010

replace regioncode="360602" if regioncode=="360602" & year==2010

replace regioncode="360622" if regioncode=="360622" & year==2010

replace regioncode="360681" if regioncode=="360681" & year==2010

replace regioncode="360702" if regioncode=="360702" & year==2010

replace regioncode="360721" if regioncode=="360721" & year==2010

replace regioncode="360722" if regioncode=="360722" & year==2010

replace regioncode="360723" if regioncode=="360723" & year==2010

replace regioncode="360724" if regioncode=="360724" & year==2010

replace regioncode="360725" if regioncode=="360725" & year==2010

replace regioncode="360726" if regioncode=="360726" & year==2010

replace regioncode="360727" if regioncode=="360727" & year==2010

replace regioncode="360728" if regioncode=="360728" & year==2010

replace regioncode="360729" if regioncode=="360729" & year==2010

replace regioncode="360730" if regioncode=="360730" & year==2010

replace regioncode="360731" if regioncode=="360731" & year==2010

replace regioncode="360732" if regioncode=="360732" & year==2010

replace regioncode="360733" if regioncode=="360733" & year==2010

replace regioncode="360734" if regioncode=="360734" & year==2010

replace regioncode="360735" if regioncode=="360735" & year==2010

replace regioncode="360781" if regioncode=="360781" & year==2010

replace regioncode="360782" if regioncode=="360782" & year==2010

replace regioncode="360802" if regioncode=="360802" & year==2010

replace regioncode="360821" if regioncode=="360821" & year==2010

replace regioncode="360822" if regioncode=="360822" & year==2010

replace regioncode="360823" if regioncode=="360823" & year==2010

replace regioncode="360824" if regioncode=="360824" & year==2010

replace regioncode="360825" if regioncode=="360825" & year==2010

replace regioncode="360826" if regioncode=="360826" & year==2010

replace regioncode="360827" if regioncode=="360827" & year==2010

replace regioncode="360828" if regioncode=="360828" & year==2010

replace regioncode="360829" if regioncode=="360829" & year==2010

replace regioncode="360830" if regioncode=="360830" & year==2010

replace regioncode="360881" if regioncode=="360881" & year==2010

replace regioncode="360902" if regioncode=="360902" & year==2010

replace regioncode="360921" if regioncode=="360921" & year==2010

replace regioncode="360922" if regioncode=="360922" & year==2010

replace regioncode="360923" if regioncode=="360923" & year==2010

replace regioncode="360924" if regioncode=="360924" & year==2010

replace regioncode="360925" if regioncode=="360925" & year==2010

replace regioncode="360926" if regioncode=="360926" & year==2010

replace regioncode="360981" if regioncode=="360981" & year==2010

replace regioncode="360982" if regioncode=="360982" & year==2010

replace regioncode="360983" if regioncode=="360983" & year==2010

replace regioncode="361002" if regioncode=="361002" & year==2010

replace regioncode="361021" if regioncode=="361021" & year==2010

replace regioncode="361022" if regioncode=="361022" & year==2010

replace regioncode="361023" if regioncode=="361023" & year==2010

replace regioncode="361024" if regioncode=="361024" & year==2010

replace regioncode="361025" if regioncode=="361025" & year==2010

replace regioncode="361026" if regioncode=="361026" & year==2010

replace regioncode="361027" if regioncode=="361027" & year==2010

replace regioncode="361028" if regioncode=="361028" & year==2010

replace regioncode="361029" if regioncode=="361029" & year==2010

replace regioncode="361030" if regioncode=="361030" & year==2010

replace regioncode="361102" if regioncode=="361102" & year==2010

replace regioncode="361121" if regioncode=="361121" & year==2010

replace regioncode="361122" if regioncode=="361122" & year==2010

replace regioncode="361123" if regioncode=="361123" & year==2010

replace regioncode="361124" if regioncode=="361124" & year==2010

replace regioncode="361125" if regioncode=="361125" & year==2010

replace regioncode="361126" if regioncode=="361126" & year==2010

replace regioncode="361127" if regioncode=="361127" & year==2010

replace regioncode="361128" if regioncode=="361128" & year==2010

replace regioncode="361129" if regioncode=="361129" & year==2010

replace regioncode="361130" if regioncode=="361130" & year==2010

replace regioncode="361181" if regioncode=="361181" & year==2010

replace regioncode="370102" if regioncode=="370102" & year==2010

replace regioncode="370103" if regioncode=="370103" & year==2010

replace regioncode="370104" if regioncode=="370104" & year==2010

replace regioncode="370105" if regioncode=="370105" & year==2010

replace regioncode="370112" if regioncode=="370112" & year==2010

replace regioncode="370113" if regioncode=="370113" & year==2010

replace regioncode="370124" if regioncode=="370124" & year==2010

replace regioncode="370125" if regioncode=="370125" & year==2010

replace regioncode="370126" if regioncode=="370126" & year==2010

replace regioncode="370181" if regioncode=="370181" & year==2010

replace regioncode="370202" if regioncode=="370202" & year==2010

replace regioncode="370203" if regioncode=="370203" & year==2010

replace regioncode="370205" if regioncode=="370205" & year==2010

replace regioncode="370211" if regioncode=="370211" & year==2010

replace regioncode="370212" if regioncode=="370212" & year==2010

replace regioncode="370213" if regioncode=="370213" & year==2010

replace regioncode="370214" if regioncode=="370214" & year==2010

replace regioncode="370281" if regioncode=="370281" & year==2010

replace regioncode="370282" if regioncode=="370282" & year==2010

replace regioncode="370283" if regioncode=="370283" & year==2010

replace regioncode="370284" if regioncode=="370284" & year==2010

replace regioncode="370285" if regioncode=="370285" & year==2010

replace regioncode="370302" if regioncode=="370302" & year==2010

replace regioncode="370303" if regioncode=="370303" & year==2010

replace regioncode="370304" if regioncode=="370304" & year==2010

replace regioncode="370305" if regioncode=="370305" & year==2010

replace regioncode="370306" if regioncode=="370306" & year==2010

replace regioncode="370321" if regioncode=="370321" & year==2010

replace regioncode="370322" if regioncode=="370322" & year==2010

replace regioncode="370323" if regioncode=="370323" & year==2010

replace regioncode="370402" if regioncode=="370402" & year==2010

replace regioncode="370403" if regioncode=="370403" & year==2010

replace regioncode="370404" if regioncode=="370404" & year==2010

replace regioncode="370405" if regioncode=="370405" & year==2010

replace regioncode="370406" if regioncode=="370406" & year==2010

replace regioncode="370481" if regioncode=="370481" & year==2010

replace regioncode="370502" if regioncode=="370502" & year==2010

replace regioncode="370503" if regioncode=="370503" & year==2010

replace regioncode="370521" if regioncode=="370521" & year==2010

replace regioncode="370522" if regioncode=="370522" & year==2010

replace regioncode="370523" if regioncode=="370523" & year==2010

replace regioncode="370602" if regioncode=="370602" & year==2010

replace regioncode="370611" if regioncode=="370611" & year==2010

replace regioncode="370612" if regioncode=="370612" & year==2010

replace regioncode="370613" if regioncode=="370613" & year==2010

replace regioncode="370634" if regioncode=="370634" & year==2010

replace regioncode="370681" if regioncode=="370681" & year==2010

replace regioncode="370682" if regioncode=="370682" & year==2010

replace regioncode="370683" if regioncode=="370683" & year==2010

replace regioncode="370684" if regioncode=="370684" & year==2010

replace regioncode="370685" if regioncode=="370685" & year==2010

replace regioncode="370686" if regioncode=="370686" & year==2010

replace regioncode="370687" if regioncode=="370687" & year==2010

replace regioncode="370702" if regioncode=="370702" & year==2010

replace regioncode="370703" if regioncode=="370703" & year==2010

replace regioncode="370704" if regioncode=="370704" & year==2010

replace regioncode="370705" if regioncode=="370705" & year==2010

replace regioncode="370724" if regioncode=="370724" & year==2010

replace regioncode="370725" if regioncode=="370725" & year==2010

replace regioncode="370781" if regioncode=="370781" & year==2010

replace regioncode="370782" if regioncode=="370782" & year==2010

replace regioncode="370783" if regioncode=="370783" & year==2010

replace regioncode="370784" if regioncode=="370784" & year==2010

replace regioncode="370785" if regioncode=="370785" & year==2010

replace regioncode="370786" if regioncode=="370786" & year==2010

replace regioncode="370802" if regioncode=="370802" & year==2010

replace regioncode="370811" if regioncode=="370811" & year==2010

replace regioncode="370826" if regioncode=="370826" & year==2010

replace regioncode="370827" if regioncode=="370827" & year==2010

replace regioncode="370828" if regioncode=="370828" & year==2010

replace regioncode="370829" if regioncode=="370829" & year==2010

replace regioncode="370830" if regioncode=="370830" & year==2010

replace regioncode="370831" if regioncode=="370831" & year==2010

replace regioncode="370832" if regioncode=="370832" & year==2010

replace regioncode="370881" if regioncode=="370881" & year==2010

replace regioncode="370882" if regioncode=="370882" & year==2010

replace regioncode="370883" if regioncode=="370883" & year==2010

replace regioncode="370902" if regioncode=="370902" & year==2010

replace regioncode="370903" if regioncode=="370911" & year==2010

replace regioncode="370921" if regioncode=="370921" & year==2010

replace regioncode="370923" if regioncode=="370923" & year==2010

replace regioncode="370982" if regioncode=="370982" & year==2010

replace regioncode="370983" if regioncode=="370983" & year==2010

replace regioncode="371002" if regioncode=="371002" & year==2010

replace regioncode="371081" if regioncode=="371081" & year==2010

replace regioncode="371082" if regioncode=="371082" & year==2010

replace regioncode="371083" if regioncode=="371083" & year==2010

replace regioncode="371102" if regioncode=="371102" & year==2010

replace regioncode="371121" if regioncode=="371121" & year==2010

replace regioncode="371122" if regioncode=="371122" & year==2010

replace regioncode="371202" if regioncode=="371202" & year==2010

replace regioncode="371203" if regioncode=="371203" & year==2010

replace regioncode="371302" if regioncode=="371302" & year==2010

replace regioncode="371311" if regioncode=="371311" & year==2010

replace regioncode="371312" if regioncode=="371312" & year==2010

replace regioncode="371321" if regioncode=="371321" & year==2010

replace regioncode="371322" if regioncode=="371322" & year==2010

replace regioncode="371323" if regioncode=="371323" & year==2010

replace regioncode="371324" if regioncode=="371324" & year==2010

replace regioncode="371325" if regioncode=="371325" & year==2010

replace regioncode="371326" if regioncode=="371326" & year==2010

replace regioncode="371327" if regioncode=="371327" & year==2010

replace regioncode="371328" if regioncode=="371328" & year==2010

replace regioncode="371329" if regioncode=="371329" & year==2010

replace regioncode="371402" if regioncode=="371402" & year==2010

replace regioncode="371421" if regioncode=="371421" & year==2010

replace regioncode="371422" if regioncode=="371422" & year==2010

replace regioncode="371423" if regioncode=="371423" & year==2010

replace regioncode="371424" if regioncode=="371424" & year==2010

replace regioncode="371425" if regioncode=="371425" & year==2010

replace regioncode="371426" if regioncode=="371426" & year==2010

replace regioncode="371427" if regioncode=="371427" & year==2010

replace regioncode="371428" if regioncode=="371428" & year==2010

replace regioncode="371481" if regioncode=="371481" & year==2010

replace regioncode="371482" if regioncode=="371482" & year==2010

replace regioncode="371502" if regioncode=="371502" & year==2010

replace regioncode="371521" if regioncode=="371521" & year==2010

replace regioncode="371522" if regioncode=="371522" & year==2010

replace regioncode="371523" if regioncode=="371523" & year==2010

replace regioncode="371524" if regioncode=="371524" & year==2010

replace regioncode="371525" if regioncode=="371525" & year==2010

replace regioncode="371526" if regioncode=="371526" & year==2010

replace regioncode="371581" if regioncode=="371581" & year==2010

replace regioncode="371602" if regioncode=="371602" & year==2010

replace regioncode="371621" if regioncode=="371621" & year==2010

replace regioncode="371622" if regioncode=="371622" & year==2010

replace regioncode="371623" if regioncode=="371623" & year==2010

replace regioncode="371624" if regioncode=="371624" & year==2010

replace regioncode="371625" if regioncode=="371625" & year==2010

replace regioncode="371626" if regioncode=="371626" & year==2010

replace regioncode="371702" if regioncode=="371702" & year==2010

replace regioncode="371721" if regioncode=="371721" & year==2010

replace regioncode="371722" if regioncode=="371722" & year==2010

replace regioncode="371723" if regioncode=="371723" & year==2010

replace regioncode="371724" if regioncode=="371724" & year==2010

replace regioncode="371725" if regioncode=="371725" & year==2010

replace regioncode="371726" if regioncode=="371726" & year==2010

replace regioncode="371727" if regioncode=="371727" & year==2010

replace regioncode="371728" if regioncode=="371728" & year==2010

replace regioncode="410102" if regioncode=="410102" & year==2010

replace regioncode="410103" if regioncode=="410103" & year==2010

replace regioncode="410104" if regioncode=="410104" & year==2010

replace regioncode="410105" if regioncode=="410105" & year==2010

replace regioncode="410106" if regioncode=="410106" & year==2010

replace regioncode="410108" if regioncode=="410108" & year==2010

replace regioncode="410122" if regioncode=="410122" & year==2010

replace regioncode="410181" if regioncode=="410181" & year==2010

replace regioncode="410182" if regioncode=="410182" & year==2010

replace regioncode="410183" if regioncode=="410183" & year==2010

replace regioncode="410184" if regioncode=="410184" & year==2010

replace regioncode="410185" if regioncode=="410185" & year==2010

replace regioncode="410202" if regioncode=="410202" & year==2010

replace regioncode="410203" if regioncode=="410203" & year==2010

replace regioncode="410204" if regioncode=="410204" & year==2010

replace regioncode="410205" if regioncode=="410205" & year==2010

replace regioncode="410211" if regioncode=="410211" & year==2010

replace regioncode="410221" if regioncode=="410221" & year==2010

replace regioncode="410222" if regioncode=="410222" & year==2010

replace regioncode="410223" if regioncode=="410223" & year==2010

replace regioncode="410224" if regioncode=="410224" & year==2010

replace regioncode="410225" if regioncode=="410225" & year==2010

replace regioncode="410302" if regioncode=="410302" & year==2010

replace regioncode="410303" if regioncode=="410303" & year==2010

replace regioncode="410304" if regioncode=="410304" & year==2010

replace regioncode="410305" if regioncode=="410305" & year==2010

replace regioncode="410306" if regioncode=="410306" & year==2010

replace regioncode="410307" if regioncode=="410311" & year==2010

replace regioncode="410322" if regioncode=="410322" & year==2010

replace regioncode="410323" if regioncode=="410323" & year==2010

replace regioncode="410324" if regioncode=="410324" & year==2010

replace regioncode="410325" if regioncode=="410325" & year==2010

replace regioncode="410326" if regioncode=="410326" & year==2010

replace regioncode="410327" if regioncode=="410327" & year==2010

replace regioncode="410328" if regioncode=="410328" & year==2010

replace regioncode="410329" if regioncode=="410329" & year==2010

replace regioncode="410381" if regioncode=="410381" & year==2010

replace regioncode="410402" if regioncode=="410402" & year==2010

replace regioncode="410403" if regioncode=="410403" & year==2010

replace regioncode="410404" if regioncode=="410404" & year==2010

replace regioncode="410411" if regioncode=="410411" & year==2010

replace regioncode="410421" if regioncode=="410421" & year==2010

replace regioncode="410422" if regioncode=="410422" & year==2010

replace regioncode="410423" if regioncode=="410423" & year==2010

replace regioncode="410425" if regioncode=="410425" & year==2010

replace regioncode="410481" if regioncode=="410481" & year==2010

replace regioncode="410482" if regioncode=="410482" & year==2010

replace regioncode="410502" if regioncode=="410502" & year==2010

replace regioncode="410503" if regioncode=="410503" & year==2010

replace regioncode="410504" if regioncode=="410505" & year==2010

replace regioncode="410511" if regioncode=="410506" & year==2010

replace regioncode="410522" if regioncode=="410522" & year==2010

replace regioncode="410523" if regioncode=="410523" & year==2010

replace regioncode="410526" if regioncode=="410526" & year==2010

replace regioncode="410527" if regioncode=="410527" & year==2010

replace regioncode="410581" if regioncode=="410581" & year==2010

replace regioncode="410602" if regioncode=="410602" & year==2010

replace regioncode="410603" if regioncode=="410603" & year==2010

replace regioncode="410611" if regioncode=="410611" & year==2010

replace regioncode="410621" if regioncode=="410621" & year==2010

replace regioncode="410622" if regioncode=="410622" & year==2010

replace regioncode="410702" if regioncode=="410702" & year==2010

replace regioncode="410703" if regioncode=="410703" & year==2010

replace regioncode="410704" if regioncode=="410704" & year==2010

replace regioncode="410711" if regioncode=="410711" & year==2010

replace regioncode="410721" if regioncode=="410721" & year==2010

replace regioncode="410724" if regioncode=="410724" & year==2010

replace regioncode="410725" if regioncode=="410725" & year==2010

replace regioncode="410726" if regioncode=="410726" & year==2010

replace regioncode="410727" if regioncode=="410727" & year==2010

replace regioncode="410728" if regioncode=="410728" & year==2010

replace regioncode="410781" if regioncode=="410781" & year==2010

replace regioncode="410782" if regioncode=="410782" & year==2010

replace regioncode="410802" if regioncode=="410802" & year==2010

replace regioncode="410803" if regioncode=="410803" & year==2010

replace regioncode="410804" if regioncode=="410804" & year==2010

replace regioncode="410811" if regioncode=="410811" & year==2010

replace regioncode="410821" if regioncode=="410821" & year==2010

replace regioncode="410822" if regioncode=="410822" & year==2010

replace regioncode="410823" if regioncode=="410823" & year==2010

replace regioncode="410825" if regioncode=="410825" & year==2010

replace regioncode="410881" if regioncode=="410881" & year==2010

replace regioncode="410882" if regioncode=="410882" & year==2010

replace regioncode="410883" if regioncode=="410883" & year==2010

replace regioncode="410902" if regioncode=="410902" & year==2010

replace regioncode="410922" if regioncode=="410922" & year==2010

replace regioncode="410923" if regioncode=="410923" & year==2010

replace regioncode="410926" if regioncode=="410926" & year==2010

replace regioncode="410927" if regioncode=="410927" & year==2010

replace regioncode="410928" if regioncode=="410928" & year==2010

replace regioncode="411002" if regioncode=="411002" & year==2010

replace regioncode="411023" if regioncode=="411023" & year==2010

replace regioncode="411024" if regioncode=="411024" & year==2010

replace regioncode="411025" if regioncode=="411025" & year==2010

replace regioncode="411081" if regioncode=="411081" & year==2010

replace regioncode="411082" if regioncode=="411082" & year==2010

replace regioncode="411102" if regioncode=="411102" & year==2010

replace regioncode="411123" if regioncode=="411103" & year==2010

replace regioncode="411121" if regioncode=="411121" & year==2010

replace regioncode="411122" if regioncode=="411122" & year==2010

replace regioncode="411202" if regioncode=="411202" & year==2010

replace regioncode="411221" if regioncode=="411221" & year==2010

replace regioncode="411222" if regioncode=="411222" & year==2010

replace regioncode="411224" if regioncode=="411224" & year==2010

replace regioncode="411281" if regioncode=="411281" & year==2010

replace regioncode="411282" if regioncode=="411282" & year==2010

replace regioncode="411302" if regioncode=="411302" & year==2010

replace regioncode="411303" if regioncode=="411303" & year==2010

replace regioncode="411321" if regioncode=="411321" & year==2010

replace regioncode="411322" if regioncode=="411322" & year==2010

replace regioncode="411323" if regioncode=="411323" & year==2010

replace regioncode="411324" if regioncode=="411324" & year==2010

replace regioncode="411325" if regioncode=="411325" & year==2010

replace regioncode="411326" if regioncode=="411326" & year==2010

replace regioncode="411327" if regioncode=="411327" & year==2010

replace regioncode="411328" if regioncode=="411328" & year==2010

replace regioncode="411329" if regioncode=="411329" & year==2010

replace regioncode="411330" if regioncode=="411330" & year==2010

replace regioncode="411381" if regioncode=="411381" & year==2010

replace regioncode="411402" if regioncode=="411402" & year==2010

replace regioncode="411403" if regioncode=="411403" & year==2010

replace regioncode="411421" if regioncode=="411421" & year==2010

replace regioncode="411422" if regioncode=="411422" & year==2010

replace regioncode="411423" if regioncode=="411423" & year==2010

replace regioncode="411424" if regioncode=="411424" & year==2010

replace regioncode="411425" if regioncode=="411425" & year==2010

replace regioncode="411426" if regioncode=="411426" & year==2010

replace regioncode="411481" if regioncode=="411481" & year==2010

replace regioncode="411502" if regioncode=="411502" & year==2010

replace regioncode="411503" if regioncode=="411503" & year==2010

replace regioncode="411521" if regioncode=="411521" & year==2010

replace regioncode="411522" if regioncode=="411522" & year==2010

replace regioncode="411523" if regioncode=="411523" & year==2010

replace regioncode="411524" if regioncode=="411524" & year==2010

replace regioncode="411525" if regioncode=="411525" & year==2010

replace regioncode="411526" if regioncode=="411526" & year==2010

replace regioncode="411527" if regioncode=="411527" & year==2010

replace regioncode="411528" if regioncode=="411528" & year==2010

replace regioncode="411602" if regioncode=="411602" & year==2010

replace regioncode="411621" if regioncode=="411621" & year==2010

replace regioncode="411622" if regioncode=="411622" & year==2010

replace regioncode="411623" if regioncode=="411623" & year==2010

replace regioncode="411624" if regioncode=="411624" & year==2010

replace regioncode="411625" if regioncode=="411625" & year==2010

replace regioncode="411626" if regioncode=="411626" & year==2010

replace regioncode="411627" if regioncode=="411627" & year==2010

replace regioncode="411628" if regioncode=="411628" & year==2010

replace regioncode="411681" if regioncode=="411681" & year==2010

replace regioncode="411702" if regioncode=="411702" & year==2010

replace regioncode="411721" if regioncode=="411721" & year==2010

replace regioncode="411722" if regioncode=="411722" & year==2010

replace regioncode="411723" if regioncode=="411723" & year==2010

replace regioncode="411724" if regioncode=="411724" & year==2010

replace regioncode="411725" if regioncode=="411725" & year==2010

replace regioncode="411726" if regioncode=="411726" & year==2010

replace regioncode="411727" if regioncode=="411727" & year==2010

replace regioncode="411728" if regioncode=="411728" & year==2010

replace regioncode="411729" if regioncode=="411729" & year==2010

replace regioncode="420102" if regioncode=="420102" & year==2010

replace regioncode="420103" if regioncode=="420103" & year==2010

replace regioncode="420104" if regioncode=="420104" & year==2010

replace regioncode="420105" if regioncode=="420105" & year==2010

replace regioncode="420106" if regioncode=="420106" & year==2010

replace regioncode="420107" if regioncode=="420107" & year==2010

replace regioncode="420111" if regioncode=="420111" & year==2010

replace regioncode="420112" if regioncode=="420112" & year==2010

replace regioncode="420113" if regioncode=="420113" & year==2010

replace regioncode="420114" if regioncode=="420114" & year==2010

replace regioncode="420115" if regioncode=="420115" & year==2010

replace regioncode="420116" if regioncode=="420116" & year==2010

replace regioncode="420117" if regioncode=="420117" & year==2010

replace regioncode="420202" if regioncode=="420202" & year==2010

replace regioncode="420203" if regioncode=="420203" & year==2010

replace regioncode="420204" if regioncode=="420204" & year==2010

replace regioncode="420205" if regioncode=="420205" & year==2010

replace regioncode="420222" if regioncode=="420222" & year==2010

replace regioncode="420281" if regioncode=="420281" & year==2010

replace regioncode="420302" if regioncode=="420302" & year==2010

replace regioncode="420303" if regioncode=="420303" & year==2010

replace regioncode="420321" if regioncode=="420321" & year==2010

replace regioncode="420322" if regioncode=="420322" & year==2010

replace regioncode="420323" if regioncode=="420323" & year==2010

replace regioncode="420324" if regioncode=="420324" & year==2010

replace regioncode="420325" if regioncode=="420325" & year==2010

replace regioncode="420381" if regioncode=="420381" & year==2010

replace regioncode="420502" if regioncode=="420502" & year==2010

replace regioncode="420503" if regioncode=="420503" & year==2010

replace regioncode="420504" if regioncode=="420504" & year==2010

replace regioncode="420505" if regioncode=="420505" & year==2010

replace regioncode="420506" if regioncode=="420506" & year==2010

replace regioncode="420525" if regioncode=="420525" & year==2010

replace regioncode="420526" if regioncode=="420526" & year==2010

replace regioncode="420527" if regioncode=="420527" & year==2010

replace regioncode="420528" if regioncode=="420528" & year==2010

replace regioncode="420529" if regioncode=="420529" & year==2010

replace regioncode="420581" if regioncode=="420581" & year==2010

replace regioncode="420582" if regioncode=="420582" & year==2010

replace regioncode="420583" if regioncode=="420583" & year==2010

replace regioncode="420602" if regioncode=="420602" & year==2010

replace regioncode="420606" if regioncode=="420606" & year==2010

replace regioncode="420607" if regioncode=="420607" & year==2010

replace regioncode="420624" if regioncode=="420624" & year==2010

replace regioncode="420625" if regioncode=="420625" & year==2010

replace regioncode="420626" if regioncode=="420626" & year==2010

replace regioncode="420682" if regioncode=="420682" & year==2010

replace regioncode="420683" if regioncode=="420683" & year==2010

replace regioncode="420684" if regioncode=="420684" & year==2010

replace regioncode="420702" if regioncode=="420702" & year==2010

replace regioncode="420703" if regioncode=="420703" & year==2010

replace regioncode="420704" if regioncode=="420704" & year==2010

replace regioncode="420802" if regioncode=="420802" & year==2010

replace regioncode="420804" if regioncode=="420804" & year==2010

replace regioncode="420821" if regioncode=="420821" & year==2010

replace regioncode="420822" if regioncode=="420822" & year==2010

replace regioncode="420881" if regioncode=="420881" & year==2010

replace regioncode="420902" if regioncode=="420902" & year==2010

replace regioncode="420921" if regioncode=="420921" & year==2010

replace regioncode="420922" if regioncode=="420922" & year==2010

replace regioncode="420923" if regioncode=="420923" & year==2010

replace regioncode="420981" if regioncode=="420981" & year==2010

replace regioncode="420982" if regioncode=="420982" & year==2010

replace regioncode="421381" if regioncode=="421381" & year==2010

replace regioncode="420984" if regioncode=="420984" & year==2010

replace regioncode="421002" if regioncode=="421002" & year==2010

replace regioncode="421003" if regioncode=="421003" & year==2010

replace regioncode="421022" if regioncode=="421022" & year==2010

replace regioncode="421023" if regioncode=="421023" & year==2010

replace regioncode="421024" if regioncode=="421024" & year==2010

replace regioncode="421081" if regioncode=="421081" & year==2010

replace regioncode="421083" if regioncode=="421083" & year==2010

replace regioncode="421087" if regioncode=="421087" & year==2010

replace regioncode="421102" if regioncode=="421102" & year==2010

replace regioncode="421121" if regioncode=="421121" & year==2010

replace regioncode="421122" if regioncode=="421122" & year==2010

replace regioncode="421123" if regioncode=="421123" & year==2010

replace regioncode="421124" if regioncode=="421124" & year==2010

replace regioncode="421125" if regioncode=="421125" & year==2010

replace regioncode="421126" if regioncode=="421126" & year==2010

replace regioncode="421127" if regioncode=="421127" & year==2010

replace regioncode="421181" if regioncode=="421181" & year==2010

replace regioncode="421182" if regioncode=="421182" & year==2010

replace regioncode="421202" if regioncode=="421202" & year==2010

replace regioncode="421221" if regioncode=="421221" & year==2010

replace regioncode="421222" if regioncode=="421222" & year==2010

replace regioncode="421223" if regioncode=="421223" & year==2010

replace regioncode="421224" if regioncode=="421224" & year==2010

replace regioncode="421281" if regioncode=="421281" & year==2010

replace regioncode="421302" if regioncode=="421302" & year==2010

replace regioncode="422801" if regioncode=="422801" & year==2010

replace regioncode="422802" if regioncode=="422802" & year==2010

replace regioncode="422822" if regioncode=="422822" & year==2010

replace regioncode="422823" if regioncode=="422823" & year==2010

replace regioncode="422825" if regioncode=="422825" & year==2010

replace regioncode="422826" if regioncode=="422826" & year==2010

replace regioncode="422827" if regioncode=="422827" & year==2010

replace regioncode="422828" if regioncode=="422828" & year==2010

replace regioncode="429004" if regioncode=="429004" & year==2010

replace regioncode="429005" if regioncode=="429005" & year==2010

replace regioncode="429006" if regioncode=="429006" & year==2010

replace regioncode="429021" if regioncode=="429021" & year==2010

replace regioncode="430102" if regioncode=="430102" & year==2010

replace regioncode="430103" if regioncode=="430103" & year==2010

replace regioncode="430104" if regioncode=="430104" & year==2010

replace regioncode="430105" if regioncode=="430105" & year==2010

replace regioncode="430111" if regioncode=="430111" & year==2010

replace regioncode="430121" if regioncode=="430121" & year==2010

replace regioncode="430122" if regioncode=="430122" & year==2010

replace regioncode="430124" if regioncode=="430124" & year==2010

replace regioncode="430181" if regioncode=="430181" & year==2010

replace regioncode="430202" if regioncode=="430202" & year==2010

replace regioncode="430203" if regioncode=="430203" & year==2010

replace regioncode="430204" if regioncode=="430204" & year==2010

replace regioncode="430211" if regioncode=="430211" & year==2010

replace regioncode="430221" if regioncode=="430221" & year==2010

replace regioncode="430223" if regioncode=="430223" & year==2010

replace regioncode="430224" if regioncode=="430224" & year==2010

replace regioncode="430225" if regioncode=="430225" & year==2010

replace regioncode="430281" if regioncode=="430281" & year==2010

replace regioncode="430302" if regioncode=="430302" & year==2010

replace regioncode="430304" if regioncode=="430304" & year==2010

replace regioncode="430321" if regioncode=="430321" & year==2010

replace regioncode="430381" if regioncode=="430381" & year==2010

replace regioncode="430382" if regioncode=="430382" & year==2010

replace regioncode="430405" if regioncode=="430405" & year==2010

replace regioncode="430406" if regioncode=="430406" & year==2010

replace regioncode="430407" if regioncode=="430407" & year==2010

replace regioncode="430408" if regioncode=="430408" & year==2010

replace regioncode="430412" if regioncode=="430412" & year==2010

replace regioncode="430421" if regioncode=="430421" & year==2010

replace regioncode="430422" if regioncode=="430422" & year==2010

replace regioncode="430423" if regioncode=="430423" & year==2010

replace regioncode="430424" if regioncode=="430424" & year==2010

replace regioncode="430426" if regioncode=="430426" & year==2010

replace regioncode="430481" if regioncode=="430481" & year==2010

replace regioncode="430482" if regioncode=="430482" & year==2010

replace regioncode="430502" if regioncode=="430502" & year==2010

replace regioncode="430503" if regioncode=="430503" & year==2010

replace regioncode="430511" if regioncode=="430511" & year==2010

replace regioncode="430521" if regioncode=="430521" & year==2010

replace regioncode="430522" if regioncode=="430522" & year==2010

replace regioncode="430523" if regioncode=="430523" & year==2010

replace regioncode="430524" if regioncode=="430524" & year==2010

replace regioncode="430525" if regioncode=="430525" & year==2010

replace regioncode="430527" if regioncode=="430527" & year==2010

replace regioncode="430528" if regioncode=="430528" & year==2010

replace regioncode="430529" if regioncode=="430529" & year==2010

replace regioncode="430581" if regioncode=="430581" & year==2010

replace regioncode="430602" if regioncode=="430602" & year==2010

replace regioncode="430603" if regioncode=="430603" & year==2010

replace regioncode="430611" if regioncode=="430611" & year==2010

replace regioncode="430621" if regioncode=="430621" & year==2010

replace regioncode="430623" if regioncode=="430623" & year==2010

replace regioncode="430624" if regioncode=="430624" & year==2010

replace regioncode="430626" if regioncode=="430626" & year==2010

replace regioncode="430681" if regioncode=="430681" & year==2010

replace regioncode="430682" if regioncode=="430682" & year==2010

replace regioncode="430702" if regioncode=="430702" & year==2010

replace regioncode="430703" if regioncode=="430703" & year==2010

replace regioncode="430721" if regioncode=="430721" & year==2010

replace regioncode="430722" if regioncode=="430722" & year==2010

replace regioncode="430723" if regioncode=="430723" & year==2010

replace regioncode="430724" if regioncode=="430724" & year==2010

replace regioncode="430725" if regioncode=="430725" & year==2010

replace regioncode="430726" if regioncode=="430726" & year==2010

replace regioncode="430781" if regioncode=="430781" & year==2010

replace regioncode="430802" if regioncode=="430802" & year==2010

replace regioncode="430811" if regioncode=="430811" & year==2010

replace regioncode="430821" if regioncode=="430821" & year==2010

replace regioncode="430822" if regioncode=="430822" & year==2010

replace regioncode="430902" if regioncode=="430902" & year==2010

replace regioncode="430903" if regioncode=="430903" & year==2010

replace regioncode="430921" if regioncode=="430921" & year==2010

replace regioncode="430922" if regioncode=="430922" & year==2010

replace regioncode="430923" if regioncode=="430923" & year==2010

replace regioncode="430981" if regioncode=="430981" & year==2010

replace regioncode="431002" if regioncode=="431002" & year==2010

replace regioncode="431003" if regioncode=="431003" & year==2010

replace regioncode="431021" if regioncode=="431021" & year==2010

replace regioncode="431022" if regioncode=="431022" & year==2010

replace regioncode="431023" if regioncode=="431023" & year==2010

replace regioncode="431024" if regioncode=="431024" & year==2010

replace regioncode="431025" if regioncode=="431025" & year==2010

replace regioncode="431026" if regioncode=="431026" & year==2010

replace regioncode="431027" if regioncode=="431027" & year==2010

replace regioncode="431028" if regioncode=="431028" & year==2010

replace regioncode="431081" if regioncode=="431081" & year==2010

replace regioncode="431102" if regioncode=="431102" & year==2010

replace regioncode="431103" if regioncode=="431103" & year==2010

replace regioncode="431121" if regioncode=="431121" & year==2010

replace regioncode="431122" if regioncode=="431122" & year==2010

replace regioncode="431123" if regioncode=="431123" & year==2010

replace regioncode="431124" if regioncode=="431124" & year==2010

replace regioncode="431125" if regioncode=="431125" & year==2010

replace regioncode="431126" if regioncode=="431126" & year==2010

replace regioncode="431127" if regioncode=="431127" & year==2010

replace regioncode="431128" if regioncode=="431128" & year==2010

replace regioncode="431129" if regioncode=="431129" & year==2010

replace regioncode="431202" if regioncode=="431202" & year==2010

replace regioncode="431221" if regioncode=="431221" & year==2010

replace regioncode="431222" if regioncode=="431222" & year==2010

replace regioncode="431223" if regioncode=="431223" & year==2010

replace regioncode="431224" if regioncode=="431224" & year==2010

replace regioncode="431225" if regioncode=="431225" & year==2010

replace regioncode="431226" if regioncode=="431226" & year==2010

replace regioncode="431227" if regioncode=="431227" & year==2010

replace regioncode="431228" if regioncode=="431228" & year==2010

replace regioncode="431229" if regioncode=="431229" & year==2010

replace regioncode="431230" if regioncode=="431230" & year==2010

replace regioncode="431281" if regioncode=="431281" & year==2010

replace regioncode="431302" if regioncode=="431302" & year==2010

replace regioncode="431321" if regioncode=="431321" & year==2010

replace regioncode="431322" if regioncode=="431322" & year==2010

replace regioncode="431381" if regioncode=="431381" & year==2010

replace regioncode="431382" if regioncode=="431382" & year==2010

replace regioncode="433101" if regioncode=="433101" & year==2010

replace regioncode="433122" if regioncode=="433122" & year==2010

replace regioncode="433123" if regioncode=="433123" & year==2010

replace regioncode="433124" if regioncode=="433124" & year==2010

replace regioncode="433125" if regioncode=="433125" & year==2010

replace regioncode="433126" if regioncode=="433126" & year==2010

replace regioncode="433127" if regioncode=="433127" & year==2010

replace regioncode="433130" if regioncode=="433130" & year==2010

replace regioncode="440102" if regioncode=="440115" & year==2010

replace regioncode="440103" if regioncode=="440103" & year==2010

replace regioncode="440104" if regioncode=="440104" & year==2010

replace regioncode="440105" if regioncode=="440105" & year==2010

replace regioncode="440106" if regioncode=="440106" & year==2010

replace regioncode="440107" if regioncode=="440116" & year==2010

replace regioncode="440111" if regioncode=="440111" & year==2010

replace regioncode="440112" if regioncode=="440112" & year==2010

replace regioncode="440113" if regioncode=="440113" & year==2010

replace regioncode="440114" if regioncode=="440114" & year==2010

replace regioncode="440183" if regioncode=="440183" & year==2010

replace regioncode="440184" if regioncode=="440184" & year==2010

replace regioncode="440203" if regioncode=="440203" & year==2010

replace regioncode="440204" if regioncode=="440204" & year==2010

replace regioncode="440221" if regioncode=="440205" & year==2010

replace regioncode="440222" if regioncode=="440222" & year==2010

replace regioncode="440224" if regioncode=="440224" & year==2010

replace regioncode="440229" if regioncode=="440229" & year==2010

replace regioncode="440232" if regioncode=="440232" & year==2010

replace regioncode="440233" if regioncode=="440233" & year==2010

replace regioncode="440281" if regioncode=="440281" & year==2010

replace regioncode="440282" if regioncode=="440282" & year==2010

replace regioncode="440303" if regioncode=="440303" & year==2010

replace regioncode="440304" if regioncode=="440304" & year==2010

replace regioncode="440305" if regioncode=="440305" & year==2010

replace regioncode="440306" if regioncode=="440306" & year==2010

replace regioncode="440307" if regioncode=="440307" & year==2010

replace regioncode="440308" if regioncode=="440308" & year==2010

replace regioncode="440402" if regioncode=="440402" & year==2010

replace regioncode="440403" if regioncode=="440403" & year==2010

replace regioncode="440404" if regioncode=="440404" & year==2010

replace regioncode="440507" if regioncode=="440507" & year==2010

replace regioncode="440511" if regioncode=="440511" & year==2010

replace regioncode="440512" if regioncode=="440512" & year==2010

replace regioncode="440514" if regioncode=="440514" & year==2010

replace regioncode="440515" if regioncode=="440515" & year==2010

replace regioncode="440523" if regioncode=="440523" & year==2010

replace regioncode="440604" if regioncode=="440604" & year==2010

replace regioncode="440605" if regioncode=="440605" & year==2010

replace regioncode="440606" if regioncode=="440606" & year==2010

replace regioncode="440607" if regioncode=="440607" & year==2010

replace regioncode="440608" if regioncode=="440608" & year==2010

replace regioncode="440703" if regioncode=="440703" & year==2010

replace regioncode="440704" if regioncode=="440704" & year==2010

replace regioncode="440781" if regioncode=="440781" & year==2010

replace regioncode="440705" if regioncode=="440705" & year==2010

replace regioncode="440783" if regioncode=="440783" & year==2010

replace regioncode="440784" if regioncode=="440784" & year==2010

replace regioncode="440785" if regioncode=="440785" & year==2010

replace regioncode="440802" if regioncode=="440802" & year==2010

replace regioncode="440803" if regioncode=="440803" & year==2010

replace regioncode="440804" if regioncode=="440804" & year==2010

replace regioncode="440811" if regioncode=="440811" & year==2010

replace regioncode="440823" if regioncode=="440823" & year==2010

replace regioncode="440825" if regioncode=="440825" & year==2010

replace regioncode="440881" if regioncode=="440881" & year==2010

replace regioncode="440882" if regioncode=="440882" & year==2010

replace regioncode="440883" if regioncode=="440883" & year==2010

replace regioncode="440902" if regioncode=="440902" & year==2010

replace regioncode="440903" if regioncode=="440903" & year==2010

replace regioncode="440923" if regioncode=="440923" & year==2010

replace regioncode="440981" if regioncode=="440981" & year==2010

replace regioncode="440982" if regioncode=="440982" & year==2010

replace regioncode="440983" if regioncode=="440983" & year==2010

replace regioncode="441202" if regioncode=="441202" & year==2010

replace regioncode="441203" if regioncode=="441203" & year==2010

replace regioncode="441223" if regioncode=="441223" & year==2010

replace regioncode="441224" if regioncode=="441224" & year==2010

replace regioncode="441225" if regioncode=="441225" & year==2010

replace regioncode="441226" if regioncode=="441226" & year==2010

replace regioncode="441283" if regioncode=="441283" & year==2010

replace regioncode="441284" if regioncode=="441284" & year==2010

replace regioncode="441302" if regioncode=="441302" & year==2010

replace regioncode="441303" if regioncode=="441303" & year==2010

replace regioncode="441322" if regioncode=="441322" & year==2010

replace regioncode="441323" if regioncode=="441323" & year==2010

replace regioncode="441324" if regioncode=="441324" & year==2010

replace regioncode="441402" if regioncode=="441402" & year==2010

replace regioncode="441421" if regioncode=="441421" & year==2010

replace regioncode="441422" if regioncode=="441422" & year==2010

replace regioncode="441423" if regioncode=="441423" & year==2010

replace regioncode="441424" if regioncode=="441424" & year==2010

replace regioncode="441426" if regioncode=="441426" & year==2010

replace regioncode="441427" if regioncode=="441427" & year==2010

replace regioncode="441481" if regioncode=="441481" & year==2010

replace regioncode="441502" if regioncode=="441502" & year==2010

replace regioncode="441521" if regioncode=="441521" & year==2010

replace regioncode="441523" if regioncode=="441523" & year==2010

replace regioncode="441581" if regioncode=="441581" & year==2010

replace regioncode="441602" if regioncode=="441602" & year==2010

replace regioncode="441621" if regioncode=="441621" & year==2010

replace regioncode="441622" if regioncode=="441622" & year==2010

replace regioncode="441623" if regioncode=="441623" & year==2010

replace regioncode="441624" if regioncode=="441624" & year==2010

replace regioncode="441625" if regioncode=="441625" & year==2010

replace regioncode="441702" if regioncode=="441702" & year==2010

replace regioncode="441721" if regioncode=="441721" & year==2010

replace regioncode="441723" if regioncode=="441723" & year==2010

replace regioncode="441781" if regioncode=="441781" & year==2010

replace regioncode="441802" if regioncode=="441802" & year==2010

replace regioncode="441821" if regioncode=="441821" & year==2010

replace regioncode="441823" if regioncode=="441823" & year==2010

replace regioncode="441825" if regioncode=="441825" & year==2010

replace regioncode="441826" if regioncode=="441826" & year==2010

replace regioncode="441827" if regioncode=="441827" & year==2010

replace regioncode="441881" if regioncode=="441881" & year==2010

replace regioncode="441882" if regioncode=="441882" & year==2010

replace regioncode="441900" if regioncode=="441901" & year==2010

replace regioncode="442000" if regioncode=="442001" & year==2010

replace regioncode="445102" if regioncode=="445102" & year==2010

replace regioncode="445121" if regioncode=="445121" & year==2010

replace regioncode="445122" if regioncode=="445122" & year==2010

replace regioncode="445202" if regioncode=="445202" & year==2010

replace regioncode="445221" if regioncode=="445221" & year==2010

replace regioncode="445222" if regioncode=="445222" & year==2010

replace regioncode="445224" if regioncode=="445224" & year==2010

replace regioncode="445281" if regioncode=="445281" & year==2010

replace regioncode="445302" if regioncode=="445302" & year==2010

replace regioncode="445321" if regioncode=="445321" & year==2010

replace regioncode="445322" if regioncode=="445322" & year==2010

replace regioncode="445323" if regioncode=="445323" & year==2010

replace regioncode="445381" if regioncode=="445381" & year==2010

replace regioncode="450102" if regioncode=="450102" & year==2010

replace regioncode="450103" if regioncode=="450103" & year==2010

replace regioncode="450104" if regioncode=="450107" & year==2010

replace regioncode="450105" if regioncode=="450105" & year==2010

replace regioncode="450106" if regioncode=="450108" & year==2010

replace regioncode="450121" if regioncode=="450109" & year==2010

replace regioncode="450122" if regioncode=="450122" & year==2010

replace regioncode="450123" if regioncode=="450123" & year==2010

replace regioncode="450124" if regioncode=="450124" & year==2010

replace regioncode="450125" if regioncode=="450125" & year==2010

replace regioncode="450126" if regioncode=="450126" & year==2010

replace regioncode="450127" if regioncode=="450127" & year==2010

replace regioncode="450202" if regioncode=="450202" & year==2010

replace regioncode="450203" if regioncode=="450203" & year==2010

replace regioncode="450204" if regioncode=="450204" & year==2010

replace regioncode="450205" if regioncode=="450205" & year==2010

replace regioncode="450221" if regioncode=="450221" & year==2010

replace regioncode="450222" if regioncode=="450222" & year==2010

replace regioncode="450223" if regioncode=="450223" & year==2010

replace regioncode="450224" if regioncode=="450224" & year==2010

replace regioncode="450225" if regioncode=="450225" & year==2010

replace regioncode="450226" if regioncode=="450226" & year==2010

replace regioncode="450302" if regioncode=="450302" & year==2010

replace regioncode="450303" if regioncode=="450303" & year==2010

replace regioncode="450304" if regioncode=="450304" & year==2010

replace regioncode="450305" if regioncode=="450305" & year==2010

replace regioncode="450311" if regioncode=="450311" & year==2010

replace regioncode="450321" if regioncode=="450321" & year==2010

replace regioncode="450322" if regioncode=="450322" & year==2010

replace regioncode="450323" if regioncode=="450323" & year==2010

replace regioncode="450324" if regioncode=="450324" & year==2010

replace regioncode="450325" if regioncode=="450325" & year==2010

replace regioncode="450326" if regioncode=="450326" & year==2010

replace regioncode="450327" if regioncode=="450327" & year==2010

replace regioncode="450328" if regioncode=="450328" & year==2010

replace regioncode="450329" if regioncode=="450329" & year==2010

replace regioncode="450330" if regioncode=="450330" & year==2010

replace regioncode="450331" if regioncode=="450331" & year==2010

replace regioncode="450332" if regioncode=="450332" & year==2010

replace regioncode="450403" if regioncode=="450403" & year==2010

replace regioncode="450404" if regioncode=="450404" & year==2010

replace regioncode="450421" if regioncode=="450421" & year==2010

replace regioncode="450422" if regioncode=="450422" & year==2010

replace regioncode="450423" if regioncode=="450423" & year==2010

replace regioncode="450481" if regioncode=="450481" & year==2010

replace regioncode="450502" if regioncode=="450502" & year==2010

replace regioncode="450503" if regioncode=="450503" & year==2010

replace regioncode="450512" if regioncode=="450512" & year==2010

replace regioncode="450521" if regioncode=="450521" & year==2010

replace regioncode="450602" if regioncode=="450602" & year==2010

replace regioncode="450603" if regioncode=="450603" & year==2010

replace regioncode="450621" if regioncode=="450621" & year==2010

replace regioncode="450681" if regioncode=="450681" & year==2010

replace regioncode="450702" if regioncode=="450702" & year==2010

replace regioncode="450703" if regioncode=="450703" & year==2010

replace regioncode="450721" if regioncode=="450721" & year==2010

replace regioncode="450722" if regioncode=="450722" & year==2010

replace regioncode="450802" if regioncode=="450802" & year==2010

replace regioncode="450803" if regioncode=="450803" & year==2010

replace regioncode="450804" if regioncode=="450804" & year==2010

replace regioncode="450821" if regioncode=="450821" & year==2010

replace regioncode="450881" if regioncode=="450881" & year==2010

replace regioncode="450902" if regioncode=="450902" & year==2010

replace regioncode="450921" if regioncode=="450921" & year==2010

replace regioncode="450922" if regioncode=="450922" & year==2010

replace regioncode="450923" if regioncode=="450923" & year==2010

replace regioncode="450924" if regioncode=="450924" & year==2010

replace regioncode="450981" if regioncode=="450981" & year==2010

replace regioncode="451002" if regioncode=="451002" & year==2010

replace regioncode="451021" if regioncode=="451021" & year==2010

replace regioncode="451022" if regioncode=="451022" & year==2010

replace regioncode="451023" if regioncode=="451023" & year==2010

replace regioncode="451024" if regioncode=="451024" & year==2010

replace regioncode="451025" if regioncode=="451025" & year==2010

replace regioncode="451026" if regioncode=="451026" & year==2010

replace regioncode="451027" if regioncode=="451027" & year==2010

replace regioncode="451028" if regioncode=="451028" & year==2010

replace regioncode="451029" if regioncode=="451029" & year==2010

replace regioncode="451030" if regioncode=="451030" & year==2010

replace regioncode="451031" if regioncode=="451031" & year==2010

replace regioncode="451102" if regioncode=="451102" & year==2010

replace regioncode="451121" if regioncode=="451121" & year==2010

replace regioncode="451122" if regioncode=="451122" & year==2010

replace regioncode="451123" if regioncode=="451123" & year==2010

replace regioncode="451202" if regioncode=="451202" & year==2010

replace regioncode="451221" if regioncode=="451221" & year==2010

replace regioncode="451222" if regioncode=="451222" & year==2010

replace regioncode="451223" if regioncode=="451223" & year==2010

replace regioncode="451224" if regioncode=="451224" & year==2010

replace regioncode="451225" if regioncode=="451225" & year==2010

replace regioncode="451226" if regioncode=="451226" & year==2010

replace regioncode="451227" if regioncode=="451227" & year==2010

replace regioncode="451228" if regioncode=="451228" & year==2010

replace regioncode="451229" if regioncode=="451229" & year==2010

replace regioncode="451281" if regioncode=="451281" & year==2010

replace regioncode="451302" if regioncode=="451302" & year==2010

replace regioncode="451321" if regioncode=="451321" & year==2010

replace regioncode="451322" if regioncode=="451322" & year==2010

replace regioncode="451323" if regioncode=="451323" & year==2010

replace regioncode="451324" if regioncode=="451324" & year==2010

replace regioncode="451381" if regioncode=="451381" & year==2010

replace regioncode="451402" if regioncode=="451402" & year==2010

replace regioncode="451421" if regioncode=="451421" & year==2010

replace regioncode="451422" if regioncode=="451422" & year==2010

replace regioncode="451423" if regioncode=="451423" & year==2010

replace regioncode="451424" if regioncode=="451424" & year==2010

replace regioncode="451425" if regioncode=="451425" & year==2010

replace regioncode="451481" if regioncode=="451481" & year==2010

replace regioncode="460105" if regioncode=="460105" & year==2010

replace regioncode="460106" if regioncode=="460106" & year==2010

replace regioncode="460107" if regioncode=="460107" & year==2010

replace regioncode="460108" if regioncode=="460108" & year==2010

replace regioncode="460001" if regioncode=="469001" & year==2010

replace regioncode="460002" if regioncode=="469002" & year==2010

replace regioncode="460003" if regioncode=="469003" & year==2010

replace regioncode="460005" if regioncode=="469005" & year==2010

replace regioncode="460006" if regioncode=="469006" & year==2010

replace regioncode="460007" if regioncode=="469007" & year==2010

replace regioncode="460030" if regioncode=="469025" & year==2010

replace regioncode="460031" if regioncode=="469026" & year==2010

replace regioncode="460033" if regioncode=="469027" & year==2010

replace regioncode="460034" if regioncode=="469028" & year==2010

replace regioncode="460036" if regioncode=="469030" & year==2010

replace regioncode="500101" if regioncode=="500101" & year==2010

replace regioncode="500102" if regioncode=="500102" & year==2010

replace regioncode="500103" if regioncode=="500103" & year==2010

replace regioncode="500104" if regioncode=="500104" & year==2010

replace regioncode="500105" if regioncode=="500105" & year==2010

replace regioncode="500106" if regioncode=="500106" & year==2010

replace regioncode="500107" if regioncode=="500107" & year==2010

replace regioncode="500108" if regioncode=="500108" & year==2010

replace regioncode="500109" if regioncode=="500109" & year==2010

replace regioncode="500110" if regioncode=="500110" & year==2010

replace regioncode="500111" if regioncode=="500111" & year==2010

replace regioncode="500112" if regioncode=="500112" & year==2010

replace regioncode="500113" if regioncode=="500113" & year==2010

replace regioncode="500114" if regioncode=="500114" & year==2010

replace regioncode="500115" if regioncode=="500115" & year==2010

replace regioncode="500381" if regioncode=="500116" & year==2010

replace regioncode="500382" if regioncode=="500117" & year==2010

replace regioncode="500383" if regioncode=="500118" & year==2010

replace regioncode="500384" if regioncode=="500119" & year==2010

replace regioncode="500222" if regioncode=="500222" & year==2010

replace regioncode="500223" if regioncode=="500223" & year==2010

replace regioncode="500224" if regioncode=="500224" & year==2010

replace regioncode="500225" if regioncode=="500225" & year==2010

replace regioncode="500226" if regioncode=="500226" & year==2010

replace regioncode="500227" if regioncode=="500227" & year==2010

replace regioncode="500228" if regioncode=="500228" & year==2010

replace regioncode="500229" if regioncode=="500229" & year==2010

replace regioncode="500230" if regioncode=="500230" & year==2010

replace regioncode="500231" if regioncode=="500231" & year==2010

replace regioncode="500232" if regioncode=="500232" & year==2010

replace regioncode="500233" if regioncode=="500233" & year==2010

replace regioncode="500234" if regioncode=="500234" & year==2010

replace regioncode="500235" if regioncode=="500235" & year==2010

replace regioncode="500236" if regioncode=="500236" & year==2010

replace regioncode="500237" if regioncode=="500237" & year==2010

replace regioncode="500238" if regioncode=="500238" & year==2010

replace regioncode="500240" if regioncode=="500240" & year==2010

replace regioncode="500241" if regioncode=="500241" & year==2010

replace regioncode="500242" if regioncode=="500242" & year==2010

replace regioncode="500243" if regioncode=="500243" & year==2010

replace regioncode="510104" if regioncode=="510104" & year==2010

replace regioncode="510105" if regioncode=="510105" & year==2010

replace regioncode="510106" if regioncode=="510106" & year==2010

replace regioncode="510107" if regioncode=="510107" & year==2010

replace regioncode="510108" if regioncode=="510108" & year==2010

replace regioncode="510112" if regioncode=="510112" & year==2010

replace regioncode="510113" if regioncode=="510113" & year==2010

replace regioncode="510114" if regioncode=="510114" & year==2010

replace regioncode="510115" if regioncode=="510115" & year==2010

replace regioncode="510121" if regioncode=="510121" & year==2010

replace regioncode="510122" if regioncode=="510122" & year==2010

replace regioncode="510124" if regioncode=="510124" & year==2010

replace regioncode="510129" if regioncode=="510129" & year==2010

replace regioncode="510131" if regioncode=="510131" & year==2010

replace regioncode="510132" if regioncode=="510132" & year==2010

replace regioncode="510181" if regioncode=="510181" & year==2010

replace regioncode="510182" if regioncode=="510182" & year==2010

replace regioncode="510183" if regioncode=="510183" & year==2010

replace regioncode="510184" if regioncode=="510184" & year==2010

replace regioncode="510302" if regioncode=="510302" & year==2010

replace regioncode="510303" if regioncode=="510303" & year==2010

replace regioncode="510304" if regioncode=="510304" & year==2010

replace regioncode="510311" if regioncode=="510311" & year==2010

replace regioncode="510321" if regioncode=="510321" & year==2010

replace regioncode="510322" if regioncode=="510322" & year==2010

replace regioncode="510402" if regioncode=="510402" & year==2010

replace regioncode="510403" if regioncode=="510403" & year==2010

replace regioncode="510411" if regioncode=="510411" & year==2010

replace regioncode="510421" if regioncode=="510421" & year==2010

replace regioncode="510422" if regioncode=="510422" & year==2010

replace regioncode="510502" if regioncode=="510502" & year==2010

replace regioncode="510503" if regioncode=="510503" & year==2010

replace regioncode="510504" if regioncode=="510504" & year==2010

replace regioncode="510521" if regioncode=="510521" & year==2010

replace regioncode="510522" if regioncode=="510522" & year==2010

replace regioncode="510524" if regioncode=="510524" & year==2010

replace regioncode="510525" if regioncode=="510525" & year==2010

replace regioncode="510603" if regioncode=="510603" & year==2010

replace regioncode="510623" if regioncode=="510623" & year==2010

replace regioncode="510626" if regioncode=="510626" & year==2010

replace regioncode="510681" if regioncode=="510681" & year==2010

replace regioncode="510682" if regioncode=="510682" & year==2010

replace regioncode="510683" if regioncode=="510683" & year==2010

replace regioncode="510703" if regioncode=="510703" & year==2010

replace regioncode="510704" if regioncode=="510704" & year==2010

replace regioncode="510722" if regioncode=="510722" & year==2010

replace regioncode="510723" if regioncode=="510723" & year==2010

replace regioncode="510724" if regioncode=="510724" & year==2010

replace regioncode="510725" if regioncode=="510725" & year==2010

replace regioncode="510726" if regioncode=="510726" & year==2010

replace regioncode="510727" if regioncode=="510727" & year==2010

replace regioncode="510781" if regioncode=="510781" & year==2010

replace regioncode="510802" if regioncode=="510802" & year==2010

replace regioncode="510811" if regioncode=="510811" & year==2010

replace regioncode="510812" if regioncode=="510812" & year==2010

replace regioncode="510821" if regioncode=="510821" & year==2010

replace regioncode="510822" if regioncode=="510822" & year==2010

replace regioncode="510823" if regioncode=="510823" & year==2010

replace regioncode="510824" if regioncode=="510824" & year==2010

replace regioncode="510902" if regioncode=="510903" & year==2010

replace regioncode="510921" if regioncode=="510921" & year==2010

replace regioncode="510922" if regioncode=="510922" & year==2010

replace regioncode="510923" if regioncode=="510923" & year==2010

replace regioncode="511002" if regioncode=="511002" & year==2010

replace regioncode="511011" if regioncode=="511011" & year==2010

replace regioncode="511024" if regioncode=="511024" & year==2010

replace regioncode="511025" if regioncode=="511025" & year==2010

replace regioncode="511028" if regioncode=="511028" & year==2010

replace regioncode="511102" if regioncode=="511102" & year==2010

replace regioncode="511111" if regioncode=="511111" & year==2010

replace regioncode="511112" if regioncode=="511112" & year==2010

replace regioncode="511113" if regioncode=="511113" & year==2010

replace regioncode="511123" if regioncode=="511123" & year==2010

replace regioncode="511124" if regioncode=="511124" & year==2010

replace regioncode="511126" if regioncode=="511126" & year==2010

replace regioncode="511129" if regioncode=="511129" & year==2010

replace regioncode="511132" if regioncode=="511132" & year==2010

replace regioncode="511133" if regioncode=="511133" & year==2010

replace regioncode="511181" if regioncode=="511181" & year==2010

replace regioncode="511302" if regioncode=="511302" & year==2010

replace regioncode="511303" if regioncode=="511303" & year==2010

replace regioncode="511304" if regioncode=="511304" & year==2010

replace regioncode="511321" if regioncode=="511321" & year==2010

replace regioncode="511322" if regioncode=="511322" & year==2010

replace regioncode="511323" if regioncode=="511323" & year==2010

replace regioncode="511324" if regioncode=="511324" & year==2010

replace regioncode="511325" if regioncode=="511325" & year==2010

replace regioncode="511381" if regioncode=="511381" & year==2010

replace regioncode="511402" if regioncode=="511402" & year==2010

replace regioncode="511421" if regioncode=="511421" & year==2010

replace regioncode="511422" if regioncode=="511422" & year==2010

replace regioncode="511423" if regioncode=="511423" & year==2010

replace regioncode="511424" if regioncode=="511424" & year==2010

replace regioncode="511425" if regioncode=="511425" & year==2010

replace regioncode="511502" if regioncode=="511502" & year==2010

replace regioncode="511521" if regioncode=="511521" & year==2010

replace regioncode="511522" if regioncode=="511522" & year==2010

replace regioncode="511523" if regioncode=="511523" & year==2010

replace regioncode="511524" if regioncode=="511524" & year==2010

replace regioncode="511525" if regioncode=="511525" & year==2010

replace regioncode="511526" if regioncode=="511526" & year==2010

replace regioncode="511527" if regioncode=="511527" & year==2010

replace regioncode="511528" if regioncode=="511528" & year==2010

replace regioncode="511529" if regioncode=="511529" & year==2010

replace regioncode="511602" if regioncode=="511602" & year==2010

replace regioncode="511621" if regioncode=="511621" & year==2010

replace regioncode="511622" if regioncode=="511622" & year==2010

replace regioncode="511623" if regioncode=="511623" & year==2010

replace regioncode="511681" if regioncode=="511681" & year==2010

replace regioncode="511702" if regioncode=="511702" & year==2010

replace regioncode="511721" if regioncode=="511721" & year==2010

replace regioncode="511722" if regioncode=="511722" & year==2010

replace regioncode="511723" if regioncode=="511723" & year==2010

replace regioncode="511724" if regioncode=="511724" & year==2010

replace regioncode="511725" if regioncode=="511725" & year==2010

replace regioncode="511781" if regioncode=="511781" & year==2010

replace regioncode="511802" if regioncode=="511802" & year==2010

replace regioncode="511821" if regioncode=="511821" & year==2010

replace regioncode="511822" if regioncode=="511822" & year==2010

replace regioncode="511823" if regioncode=="511823" & year==2010

replace regioncode="511824" if regioncode=="511824" & year==2010

replace regioncode="511825" if regioncode=="511825" & year==2010

replace regioncode="511826" if regioncode=="511826" & year==2010

replace regioncode="511827" if regioncode=="511827" & year==2010

replace regioncode="511902" if regioncode=="511902" & year==2010

replace regioncode="511921" if regioncode=="511921" & year==2010

replace regioncode="511922" if regioncode=="511922" & year==2010

replace regioncode="511923" if regioncode=="511923" & year==2010

replace regioncode="512002" if regioncode=="512002" & year==2010

replace regioncode="512021" if regioncode=="512021" & year==2010

replace regioncode="512022" if regioncode=="512022" & year==2010

replace regioncode="512081" if regioncode=="512081" & year==2010

replace regioncode="513221" if regioncode=="513221" & year==2010

replace regioncode="513222" if regioncode=="513222" & year==2010

replace regioncode="513223" if regioncode=="513223" & year==2010

replace regioncode="513224" if regioncode=="513224" & year==2010

replace regioncode="513225" if regioncode=="513225" & year==2010

replace regioncode="513226" if regioncode=="513226" & year==2010

replace regioncode="513227" if regioncode=="513227" & year==2010

replace regioncode="513228" if regioncode=="513228" & year==2010

replace regioncode="513229" if regioncode=="513229" & year==2010

replace regioncode="513230" if regioncode=="513230" & year==2010

replace regioncode="513231" if regioncode=="513231" & year==2010

replace regioncode="513232" if regioncode=="513232" & year==2010

replace regioncode="513233" if regioncode=="513233" & year==2010

replace regioncode="513321" if regioncode=="513321" & year==2010

replace regioncode="513322" if regioncode=="513322" & year==2010

replace regioncode="513323" if regioncode=="513323" & year==2010

replace regioncode="513324" if regioncode=="513324" & year==2010

replace regioncode="513325" if regioncode=="513325" & year==2010

replace regioncode="513326" if regioncode=="513326" & year==2010

replace regioncode="513327" if regioncode=="513327" & year==2010

replace regioncode="513328" if regioncode=="513328" & year==2010

replace regioncode="513329" if regioncode=="513329" & year==2010

replace regioncode="513330" if regioncode=="513330" & year==2010

replace regioncode="513331" if regioncode=="513331" & year==2010

replace regioncode="513332" if regioncode=="513332" & year==2010

replace regioncode="513333" if regioncode=="513333" & year==2010

replace regioncode="513334" if regioncode=="513334" & year==2010

replace regioncode="513335" if regioncode=="513335" & year==2010

replace regioncode="513336" if regioncode=="513336" & year==2010

replace regioncode="513337" if regioncode=="513337" & year==2010

replace regioncode="513338" if regioncode=="513338" & year==2010

replace regioncode="513401" if regioncode=="513401" & year==2010

replace regioncode="513422" if regioncode=="513422" & year==2010

replace regioncode="513423" if regioncode=="513423" & year==2010

replace regioncode="513424" if regioncode=="513424" & year==2010

replace regioncode="513425" if regioncode=="513425" & year==2010

replace regioncode="513426" if regioncode=="513426" & year==2010

replace regioncode="513427" if regioncode=="513427" & year==2010

replace regioncode="513428" if regioncode=="513428" & year==2010

replace regioncode="513429" if regioncode=="513429" & year==2010

replace regioncode="513430" if regioncode=="513430" & year==2010

replace regioncode="513431" if regioncode=="513431" & year==2010

replace regioncode="513432" if regioncode=="513432" & year==2010

replace regioncode="513433" if regioncode=="513433" & year==2010

replace regioncode="513434" if regioncode=="513434" & year==2010

replace regioncode="513435" if regioncode=="513435" & year==2010

replace regioncode="513436" if regioncode=="513436" & year==2010

replace regioncode="513437" if regioncode=="513437" & year==2010

replace regioncode="520102" if regioncode=="520102" & year==2010

replace regioncode="520103" if regioncode=="520103" & year==2010

replace regioncode="520111" if regioncode=="520111" & year==2010

replace regioncode="520112" if regioncode=="520112" & year==2010

replace regioncode="520113" if regioncode=="520113" & year==2010

replace regioncode="520114" if regioncode=="520114" & year==2010

replace regioncode="520121" if regioncode=="520121" & year==2010

replace regioncode="520122" if regioncode=="520122" & year==2010

replace regioncode="520123" if regioncode=="520123" & year==2010

replace regioncode="520181" if regioncode=="520181" & year==2010

replace regioncode="520201" if regioncode=="520201" & year==2010

replace regioncode="520222" if regioncode=="520222" & year==2010

replace regioncode="520203" if regioncode=="520203" & year==2010

replace regioncode="520221" if regioncode=="520221" & year==2010

replace regioncode="520302" if regioncode=="520302" & year==2010

replace regioncode="520321" if regioncode=="520321" & year==2010

replace regioncode="520322" if regioncode=="520322" & year==2010

replace regioncode="520323" if regioncode=="520323" & year==2010

replace regioncode="520324" if regioncode=="520324" & year==2010

replace regioncode="520325" if regioncode=="520325" & year==2010

replace regioncode="520326" if regioncode=="520326" & year==2010

replace regioncode="520327" if regioncode=="520327" & year==2010

replace regioncode="520328" if regioncode=="520328" & year==2010

replace regioncode="520329" if regioncode=="520329" & year==2010

replace regioncode="520330" if regioncode=="520330" & year==2010

replace regioncode="520381" if regioncode=="520381" & year==2010

replace regioncode="520382" if regioncode=="520382" & year==2010

replace regioncode="520402" if regioncode=="520402" & year==2010

replace regioncode="520421" if regioncode=="520421" & year==2010

replace regioncode="520422" if regioncode=="520422" & year==2010

replace regioncode="520423" if regioncode=="520423" & year==2010

replace regioncode="520424" if regioncode=="520424" & year==2010

replace regioncode="520425" if regioncode=="520425" & year==2010

replace regioncode="522201" if regioncode=="522201" & year==2010

replace regioncode="522222" if regioncode=="522222" & year==2010

replace regioncode="522223" if regioncode=="522223" & year==2010

replace regioncode="522224" if regioncode=="522224" & year==2010

replace regioncode="522225" if regioncode=="522225" & year==2010

replace regioncode="522226" if regioncode=="522226" & year==2010

replace regioncode="522227" if regioncode=="522227" & year==2010

replace regioncode="522228" if regioncode=="522228" & year==2010

replace regioncode="522229" if regioncode=="522229" & year==2010

replace regioncode="522230" if regioncode=="522230" & year==2010

replace regioncode="522301" if regioncode=="522301" & year==2010

replace regioncode="522322" if regioncode=="522322" & year==2010

replace regioncode="522323" if regioncode=="522323" & year==2010

replace regioncode="522324" if regioncode=="522324" & year==2010

replace regioncode="522325" if regioncode=="522325" & year==2010

replace regioncode="522326" if regioncode=="522326" & year==2010

replace regioncode="522327" if regioncode=="522327" & year==2010

replace regioncode="522328" if regioncode=="522328" & year==2010

replace regioncode="522401" if regioncode=="522401" & year==2010

replace regioncode="522422" if regioncode=="522422" & year==2010

replace regioncode="522423" if regioncode=="522423" & year==2010

replace regioncode="522424" if regioncode=="522424" & year==2010

replace regioncode="522425" if regioncode=="522425" & year==2010

replace regioncode="522426" if regioncode=="522426" & year==2010

replace regioncode="522427" if regioncode=="522427" & year==2010

replace regioncode="522428" if regioncode=="522428" & year==2010

replace regioncode="522601" if regioncode=="522601" & year==2010

replace regioncode="522622" if regioncode=="522622" & year==2010

replace regioncode="522623" if regioncode=="522623" & year==2010

replace regioncode="522624" if regioncode=="522624" & year==2010

replace regioncode="522625" if regioncode=="522625" & year==2010

replace regioncode="522626" if regioncode=="522626" & year==2010

replace regioncode="522627" if regioncode=="522627" & year==2010

replace regioncode="522628" if regioncode=="522628" & year==2010

replace regioncode="522629" if regioncode=="522629" & year==2010

replace regioncode="522630" if regioncode=="522630" & year==2010

replace regioncode="522631" if regioncode=="522631" & year==2010

replace regioncode="522632" if regioncode=="522632" & year==2010

replace regioncode="522633" if regioncode=="522633" & year==2010

replace regioncode="522634" if regioncode=="522634" & year==2010

replace regioncode="522635" if regioncode=="522635" & year==2010

replace regioncode="522636" if regioncode=="522636" & year==2010

replace regioncode="522701" if regioncode=="522701" & year==2010

replace regioncode="522702" if regioncode=="522702" & year==2010

replace regioncode="522722" if regioncode=="522722" & year==2010

replace regioncode="522723" if regioncode=="522723" & year==2010

replace regioncode="522725" if regioncode=="522725" & year==2010

replace regioncode="522726" if regioncode=="522726" & year==2010

replace regioncode="522727" if regioncode=="522727" & year==2010

replace regioncode="522728" if regioncode=="522728" & year==2010

replace regioncode="522729" if regioncode=="522729" & year==2010

replace regioncode="522730" if regioncode=="522730" & year==2010

replace regioncode="522731" if regioncode=="522731" & year==2010

replace regioncode="522732" if regioncode=="522732" & year==2010

replace regioncode="530102" if regioncode=="530102" & year==2010

replace regioncode="530103" if regioncode=="530103" & year==2010

replace regioncode="530111" if regioncode=="530111" & year==2010

replace regioncode="530112" if regioncode=="530112" & year==2010

replace regioncode="530113" if regioncode=="530113" & year==2010

replace regioncode="530121" if regioncode=="530121" & year==2010

replace regioncode="530122" if regioncode=="530122" & year==2010

replace regioncode="530124" if regioncode=="530124" & year==2010

replace regioncode="530125" if regioncode=="530125" & year==2010

replace regioncode="530126" if regioncode=="530126" & year==2010

replace regioncode="530127" if regioncode=="530127" & year==2010

replace regioncode="530128" if regioncode=="530128" & year==2010

replace regioncode="530129" if regioncode=="530129" & year==2010

replace regioncode="530181" if regioncode=="530181" & year==2010

replace regioncode="530302" if regioncode=="530302" & year==2010

replace regioncode="530321" if regioncode=="530321" & year==2010

replace regioncode="530322" if regioncode=="530322" & year==2010

replace regioncode="530323" if regioncode=="530323" & year==2010

replace regioncode="530324" if regioncode=="530324" & year==2010

replace regioncode="530325" if regioncode=="530325" & year==2010

replace regioncode="530326" if regioncode=="530326" & year==2010

replace regioncode="530328" if regioncode=="530328" & year==2010

replace regioncode="530381" if regioncode=="530381" & year==2010

replace regioncode="530402" if regioncode=="530402" & year==2010

replace regioncode="530421" if regioncode=="530421" & year==2010

replace regioncode="530422" if regioncode=="530422" & year==2010

replace regioncode="530423" if regioncode=="530423" & year==2010

replace regioncode="530424" if regioncode=="530424" & year==2010

replace regioncode="530425" if regioncode=="530425" & year==2010

replace regioncode="530426" if regioncode=="530426" & year==2010

replace regioncode="530427" if regioncode=="530427" & year==2010

replace regioncode="530428" if regioncode=="530428" & year==2010

replace regioncode="530502" if regioncode=="530502" & year==2010

replace regioncode="530521" if regioncode=="530521" & year==2010

replace regioncode="530522" if regioncode=="530522" & year==2010

replace regioncode="530523" if regioncode=="530523" & year==2010

replace regioncode="530524" if regioncode=="530524" & year==2010

replace regioncode="530602" if regioncode=="530602" & year==2010

replace regioncode="530621" if regioncode=="530621" & year==2010

replace regioncode="530622" if regioncode=="530622" & year==2010

replace regioncode="530623" if regioncode=="530623" & year==2010

replace regioncode="530624" if regioncode=="530624" & year==2010

replace regioncode="530625" if regioncode=="530625" & year==2010

replace regioncode="530626" if regioncode=="530626" & year==2010

replace regioncode="530627" if regioncode=="530627" & year==2010

replace regioncode="530628" if regioncode=="530628" & year==2010

replace regioncode="530629" if regioncode=="530629" & year==2010

replace regioncode="530630" if regioncode=="530630" & year==2010

replace regioncode="530721" if regioncode=="530721" & year==2010

replace regioncode="530722" if regioncode=="530722" & year==2010

replace regioncode="530723" if regioncode=="530723" & year==2010

replace regioncode="530724" if regioncode=="530724" & year==2010

replace regioncode="532701" if regioncode=="530802" & year==2010

replace regioncode="532722" if regioncode=="530821" & year==2010

replace regioncode="532723" if regioncode=="530822" & year==2010

replace regioncode="532724" if regioncode=="530823" & year==2010

replace regioncode="532725" if regioncode=="530824" & year==2010

replace regioncode="532726" if regioncode=="530825" & year==2010

replace regioncode="532727" if regioncode=="530826" & year==2010

replace regioncode="532728" if regioncode=="530827" & year==2010

replace regioncode="532729" if regioncode=="530828" & year==2010

replace regioncode="532730" if regioncode=="530829" & year==2010

replace regioncode="533521" if regioncode=="530902" & year==2010

replace regioncode="533522" if regioncode=="530921" & year==2010

replace regioncode="533523" if regioncode=="530922" & year==2010

replace regioncode="533524" if regioncode=="530923" & year==2010

replace regioncode="533525" if regioncode=="530924" & year==2010

replace regioncode="533526" if regioncode=="530925" & year==2010

replace regioncode="533527" if regioncode=="530926" & year==2010

replace regioncode="533528" if regioncode=="530927" & year==2010

replace regioncode="532301" if regioncode=="532301" & year==2010

replace regioncode="532322" if regioncode=="532322" & year==2010

replace regioncode="532323" if regioncode=="532323" & year==2010

replace regioncode="532324" if regioncode=="532324" & year==2010

replace regioncode="532325" if regioncode=="532325" & year==2010

replace regioncode="532326" if regioncode=="532326" & year==2010

replace regioncode="532327" if regioncode=="532327" & year==2010

replace regioncode="532328" if regioncode=="532328" & year==2010

replace regioncode="532329" if regioncode=="532329" & year==2010

replace regioncode="532331" if regioncode=="532331" & year==2010

replace regioncode="532501" if regioncode=="532501" & year==2010

replace regioncode="532502" if regioncode=="532502" & year==2010

replace regioncode="532522" if regioncode=="532522" & year==2010

replace regioncode="532523" if regioncode=="532523" & year==2010

replace regioncode="532524" if regioncode=="532524" & year==2010

replace regioncode="532525" if regioncode=="532525" & year==2010

replace regioncode="532526" if regioncode=="532526" & year==2010

replace regioncode="532527" if regioncode=="532527" & year==2010

replace regioncode="532528" if regioncode=="532528" & year==2010

replace regioncode="532529" if regioncode=="532529" & year==2010

replace regioncode="532530" if regioncode=="532530" & year==2010

replace regioncode="532531" if regioncode=="532531" & year==2010

replace regioncode="532532" if regioncode=="532532" & year==2010

replace regioncode="532621" if regioncode=="532621" & year==2010

replace regioncode="532622" if regioncode=="532622" & year==2010

replace regioncode="532623" if regioncode=="532623" & year==2010

replace regioncode="532624" if regioncode=="532624" & year==2010

replace regioncode="532625" if regioncode=="532625" & year==2010

replace regioncode="532626" if regioncode=="532626" & year==2010

replace regioncode="532627" if regioncode=="532627" & year==2010

replace regioncode="532628" if regioncode=="532628" & year==2010

replace regioncode="532801" if regioncode=="532801" & year==2010

replace regioncode="532822" if regioncode=="532822" & year==2010

replace regioncode="532823" if regioncode=="532823" & year==2010

replace regioncode="532901" if regioncode=="532901" & year==2010

replace regioncode="532922" if regioncode=="532922" & year==2010

replace regioncode="532923" if regioncode=="532923" & year==2010

replace regioncode="532924" if regioncode=="532924" & year==2010

replace regioncode="532925" if regioncode=="532925" & year==2010

replace regioncode="532926" if regioncode=="532926" & year==2010

replace regioncode="532927" if regioncode=="532927" & year==2010

replace regioncode="532928" if regioncode=="532928" & year==2010

replace regioncode="532929" if regioncode=="532929" & year==2010

replace regioncode="532930" if regioncode=="532930" & year==2010

replace regioncode="532931" if regioncode=="532931" & year==2010

replace regioncode="532932" if regioncode=="532932" & year==2010

replace regioncode="533102" if regioncode=="533102" & year==2010

replace regioncode="533103" if regioncode=="533103" & year==2010

replace regioncode="533122" if regioncode=="533122" & year==2010

replace regioncode="533123" if regioncode=="533123" & year==2010

replace regioncode="533124" if regioncode=="533124" & year==2010

replace regioncode="533321" if regioncode=="533321" & year==2010

replace regioncode="533323" if regioncode=="533323" & year==2010

replace regioncode="533324" if regioncode=="533324" & year==2010

replace regioncode="533325" if regioncode=="533325" & year==2010

replace regioncode="533421" if regioncode=="533421" & year==2010

replace regioncode="533422" if regioncode=="533422" & year==2010

replace regioncode="533423" if regioncode=="533423" & year==2010

replace regioncode="540102" if regioncode=="540102" & year==2010

replace regioncode="540121" if regioncode=="540121" & year==2010

replace regioncode="540122" if regioncode=="540122" & year==2010

replace regioncode="540123" if regioncode=="540123" & year==2010

replace regioncode="540124" if regioncode=="540124" & year==2010

replace regioncode="540125" if regioncode=="540125" & year==2010

replace regioncode="540126" if regioncode=="540126" & year==2010

replace regioncode="540127" if regioncode=="540127" & year==2010

replace regioncode="542121" if regioncode=="542121" & year==2010

replace regioncode="542122" if regioncode=="542122" & year==2010

replace regioncode="542123" if regioncode=="542123" & year==2010

replace regioncode="542124" if regioncode=="542124" & year==2010

replace regioncode="542125" if regioncode=="542125" & year==2010

replace regioncode="542126" if regioncode=="542126" & year==2010

replace regioncode="542127" if regioncode=="542127" & year==2010

replace regioncode="542128" if regioncode=="542128" & year==2010

replace regioncode="542129" if regioncode=="542129" & year==2010

replace regioncode="542132" if regioncode=="542132" & year==2010

replace regioncode="542133" if regioncode=="542133" & year==2010

replace regioncode="542221" if regioncode=="542221" & year==2010

replace regioncode="542223" if regioncode=="542223" & year==2010

replace regioncode="542224" if regioncode=="542224" & year==2010

replace regioncode="542225" if regioncode=="542225" & year==2010

replace regioncode="542227" if regioncode=="542227" & year==2010

replace regioncode="542228" if regioncode=="542228" & year==2010

replace regioncode="542229" if regioncode=="542229" & year==2010

replace regioncode="542231" if regioncode=="542231" & year==2010

replace regioncode="542232" if regioncode=="542232" & year==2010

replace regioncode="542233" if regioncode=="542233" & year==2010

replace regioncode="542301" if regioncode=="542301" & year==2010

replace regioncode="542322" if regioncode=="542322" & year==2010

replace regioncode="542323" if regioncode=="542323" & year==2010

replace regioncode="542324" if regioncode=="542324" & year==2010

replace regioncode="542325" if regioncode=="542325" & year==2010

replace regioncode="542326" if regioncode=="542326" & year==2010

replace regioncode="542327" if regioncode=="542327" & year==2010

replace regioncode="542328" if regioncode=="542328" & year==2010

replace regioncode="542329" if regioncode=="542329" & year==2010

replace regioncode="542330" if regioncode=="542330" & year==2010

replace regioncode="542331" if regioncode=="542331" & year==2010

replace regioncode="542332" if regioncode=="542332" & year==2010

replace regioncode="542334" if regioncode=="542334" & year==2010

replace regioncode="542336" if regioncode=="542336" & year==2010

replace regioncode="542338" if regioncode=="542338" & year==2010

replace regioncode="542421" if regioncode=="542421" & year==2010

replace regioncode="542422" if regioncode=="542422" & year==2010

replace regioncode="542423" if regioncode=="542423" & year==2010

replace regioncode="542424" if regioncode=="542424" & year==2010

replace regioncode="542425" if regioncode=="542425" & year==2010

replace regioncode="542426" if regioncode=="542426" & year==2010

replace regioncode="542427" if regioncode=="542427" & year==2010

replace regioncode="542429" if regioncode=="542429" & year==2010

replace regioncode="542430" if regioncode=="542430" & year==2010

replace regioncode="542523" if regioncode=="542523" & year==2010

replace regioncode="542621" if regioncode=="542621" & year==2010

replace regioncode="542622" if regioncode=="542622" & year==2010

replace regioncode="542623" if regioncode=="542623" & year==2010

replace regioncode="542625" if regioncode=="542625" & year==2010

replace regioncode="542626" if regioncode=="542626" & year==2010

replace regioncode="610102" if regioncode=="610102" & year==2010

replace regioncode="610103" if regioncode=="610103" & year==2010

replace regioncode="610104" if regioncode=="610104" & year==2010

replace regioncode="610111" if regioncode=="610111" & year==2010

replace regioncode="610112" if regioncode=="610112" & year==2010

replace regioncode="610113" if regioncode=="610113" & year==2010

replace regioncode="610114" if regioncode=="610114" & year==2010

replace regioncode="610115" if regioncode=="610115" & year==2010

replace regioncode="610116" if regioncode=="610116" & year==2010

replace regioncode="610122" if regioncode=="610122" & year==2010

replace regioncode="610124" if regioncode=="610124" & year==2010

replace regioncode="610125" if regioncode=="610125" & year==2010

replace regioncode="610126" if regioncode=="610126" & year==2010

replace regioncode="610202" if regioncode=="610202" & year==2010

replace regioncode="610203" if regioncode=="610203" & year==2010

replace regioncode="610204" if regioncode=="610204" & year==2010

replace regioncode="610222" if regioncode=="610222" & year==2010

replace regioncode="610302" if regioncode=="610302" & year==2010

replace regioncode="610303" if regioncode=="610303" & year==2010

replace regioncode="610304" if regioncode=="610304" & year==2010

replace regioncode="610322" if regioncode=="610322" & year==2010

replace regioncode="610323" if regioncode=="610323" & year==2010

replace regioncode="610324" if regioncode=="610324" & year==2010

replace regioncode="610326" if regioncode=="610326" & year==2010

replace regioncode="610327" if regioncode=="610327" & year==2010

replace regioncode="610328" if regioncode=="610328" & year==2010

replace regioncode="610329" if regioncode=="610329" & year==2010

replace regioncode="610330" if regioncode=="610330" & year==2010

replace regioncode="610331" if regioncode=="610331" & year==2010

replace regioncode="610402" if regioncode=="610402" & year==2010

replace regioncode="610403" if regioncode=="610403" & year==2010

replace regioncode="610404" if regioncode=="610404" & year==2010

replace regioncode="610422" if regioncode=="610422" & year==2010

replace regioncode="610423" if regioncode=="610423" & year==2010

replace regioncode="610424" if regioncode=="610424" & year==2010

replace regioncode="610425" if regioncode=="610425" & year==2010

replace regioncode="610426" if regioncode=="610426" & year==2010

replace regioncode="610427" if regioncode=="610427" & year==2010

replace regioncode="610428" if regioncode=="610428" & year==2010

replace regioncode="610429" if regioncode=="610429" & year==2010

replace regioncode="610430" if regioncode=="610430" & year==2010

replace regioncode="610431" if regioncode=="610431" & year==2010

replace regioncode="610481" if regioncode=="610481" & year==2010

replace regioncode="610502" if regioncode=="610502" & year==2010

replace regioncode="610521" if regioncode=="610521" & year==2010

replace regioncode="610522" if regioncode=="610522" & year==2010

replace regioncode="610523" if regioncode=="610523" & year==2010

replace regioncode="610524" if regioncode=="610524" & year==2010

replace regioncode="610525" if regioncode=="610525" & year==2010

replace regioncode="610526" if regioncode=="610526" & year==2010

replace regioncode="610527" if regioncode=="610527" & year==2010

replace regioncode="610528" if regioncode=="610528" & year==2010

replace regioncode="610581" if regioncode=="610581" & year==2010

replace regioncode="610582" if regioncode=="610582" & year==2010

replace regioncode="610602" if regioncode=="610602" & year==2010

replace regioncode="610621" if regioncode=="610621" & year==2010

replace regioncode="610622" if regioncode=="610622" & year==2010

replace regioncode="610623" if regioncode=="610623" & year==2010

replace regioncode="610624" if regioncode=="610624" & year==2010

replace regioncode="610625" if regioncode=="610625" & year==2010

replace regioncode="610626" if regioncode=="610626" & year==2010

replace regioncode="610627" if regioncode=="610627" & year==2010

replace regioncode="610628" if regioncode=="610628" & year==2010

replace regioncode="610629" if regioncode=="610629" & year==2010

replace regioncode="610630" if regioncode=="610630" & year==2010

replace regioncode="610631" if regioncode=="610631" & year==2010

replace regioncode="610632" if regioncode=="610632" & year==2010

replace regioncode="610702" if regioncode=="610702" & year==2010

replace regioncode="610721" if regioncode=="610721" & year==2010

replace regioncode="610722" if regioncode=="610722" & year==2010

replace regioncode="610723" if regioncode=="610723" & year==2010

replace regioncode="610724" if regioncode=="610724" & year==2010

replace regioncode="610725" if regioncode=="610725" & year==2010

replace regioncode="610726" if regioncode=="610726" & year==2010

replace regioncode="610727" if regioncode=="610727" & year==2010

replace regioncode="610728" if regioncode=="610728" & year==2010

replace regioncode="610729" if regioncode=="610729" & year==2010

replace regioncode="610730" if regioncode=="610730" & year==2010

replace regioncode="610802" if regioncode=="610802" & year==2010

replace regioncode="610821" if regioncode=="610821" & year==2010

replace regioncode="610822" if regioncode=="610822" & year==2010

replace regioncode="610823" if regioncode=="610823" & year==2010

replace regioncode="610824" if regioncode=="610824" & year==2010

replace regioncode="610825" if regioncode=="610825" & year==2010

replace regioncode="610826" if regioncode=="610826" & year==2010

replace regioncode="610827" if regioncode=="610827" & year==2010

replace regioncode="610828" if regioncode=="610828" & year==2010

replace regioncode="610829" if regioncode=="610829" & year==2010

replace regioncode="610830" if regioncode=="610830" & year==2010

replace regioncode="610831" if regioncode=="610831" & year==2010

replace regioncode="610902" if regioncode=="610902" & year==2010

replace regioncode="610921" if regioncode=="610921" & year==2010

replace regioncode="610922" if regioncode=="610922" & year==2010

replace regioncode="610923" if regioncode=="610923" & year==2010

replace regioncode="610924" if regioncode=="610924" & year==2010

replace regioncode="610925" if regioncode=="610925" & year==2010

replace regioncode="610926" if regioncode=="610926" & year==2010

replace regioncode="610927" if regioncode=="610927" & year==2010

replace regioncode="610928" if regioncode=="610928" & year==2010

replace regioncode="610929" if regioncode=="610929" & year==2010

replace regioncode="611002" if regioncode=="611002" & year==2010

replace regioncode="611021" if regioncode=="611021" & year==2010

replace regioncode="611022" if regioncode=="611022" & year==2010

replace regioncode="611023" if regioncode=="611023" & year==2010

replace regioncode="611024" if regioncode=="611024" & year==2010

replace regioncode="611025" if regioncode=="611025" & year==2010

replace regioncode="611026" if regioncode=="611026" & year==2010

replace regioncode="620102" if regioncode=="620102" & year==2010

replace regioncode="620103" if regioncode=="620103" & year==2010

replace regioncode="620104" if regioncode=="620104" & year==2010

replace regioncode="620105" if regioncode=="620105" & year==2010

replace regioncode="620111" if regioncode=="620111" & year==2010

replace regioncode="620121" if regioncode=="620121" & year==2010

replace regioncode="620122" if regioncode=="620122" & year==2010

replace regioncode="620123" if regioncode=="620123" & year==2010

replace regioncode="620201" if regioncode=="620201" & year==2010

replace regioncode="620302" if regioncode=="620302" & year==2010

replace regioncode="620321" if regioncode=="620321" & year==2010

replace regioncode="620402" if regioncode=="620402" & year==2010

replace regioncode="620403" if regioncode=="620403" & year==2010

replace regioncode="620421" if regioncode=="620421" & year==2010

replace regioncode="620422" if regioncode=="620422" & year==2010

replace regioncode="620423" if regioncode=="620423" & year==2010

replace regioncode="620502" if regioncode=="620502" & year==2010

replace regioncode="620503" if regioncode=="620503" & year==2010

replace regioncode="620521" if regioncode=="620521" & year==2010

replace regioncode="620522" if regioncode=="620522" & year==2010

replace regioncode="620523" if regioncode=="620523" & year==2010

replace regioncode="620524" if regioncode=="620524" & year==2010

replace regioncode="620525" if regioncode=="620525" & year==2010

replace regioncode="622301" if regioncode=="620602" & year==2010

replace regioncode="622322" if regioncode=="620621" & year==2010

replace regioncode="622323" if regioncode=="620622" & year==2010

replace regioncode="622326" if regioncode=="620623" & year==2010

replace regioncode="622201" if regioncode=="620702" & year==2010

replace regioncode="622222" if regioncode=="620721" & year==2010

replace regioncode="622223" if regioncode=="620722" & year==2010

replace regioncode="622224" if regioncode=="620723" & year==2010

replace regioncode="622225" if regioncode=="620724" & year==2010

replace regioncode="622226" if regioncode=="620725" & year==2010

replace regioncode="622701" if regioncode=="620802" & year==2010

replace regioncode="622722" if regioncode=="620821" & year==2010

replace regioncode="622723" if regioncode=="620822" & year==2010

replace regioncode="622724" if regioncode=="620823" & year==2010

replace regioncode="622725" if regioncode=="620824" & year==2010

replace regioncode="622726" if regioncode=="620825" & year==2010

replace regioncode="622727" if regioncode=="620826" & year==2010

replace regioncode="622102" if regioncode=="620902" & year==2010

replace regioncode="622123" if regioncode=="620921" & year==2010

replace regioncode="622126" if regioncode=="620922" & year==2010

replace regioncode="622124" if regioncode=="620923" & year==2010

replace regioncode="622125" if regioncode=="620924" & year==2010

replace regioncode="622101" if regioncode=="620981" & year==2010

replace regioncode="622103" if regioncode=="620982" & year==2010

replace regioncode="622801" if regioncode=="621002" & year==2010

replace regioncode="622821" if regioncode=="621021" & year==2010

replace regioncode="622822" if regioncode=="621022" & year==2010

replace regioncode="622823" if regioncode=="621023" & year==2010

replace regioncode="622824" if regioncode=="621024" & year==2010

replace regioncode="622825" if regioncode=="621025" & year==2010

replace regioncode="622826" if regioncode=="621026" & year==2010

replace regioncode="622827" if regioncode=="621027" & year==2010

replace regioncode="622421" if regioncode=="621102" & year==2010

replace regioncode="622424" if regioncode=="621121" & year==2010

replace regioncode="622425" if regioncode=="621122" & year==2010

replace regioncode="622426" if regioncode=="621123" & year==2010

replace regioncode="622427" if regioncode=="621124" & year==2010

replace regioncode="622428" if regioncode=="621125" & year==2010

replace regioncode="622429" if regioncode=="621126" & year==2010

replace regioncode="622621" if regioncode=="621202" & year==2010

replace regioncode="622624" if regioncode=="621221" & year==2010

replace regioncode="622626" if regioncode=="621222" & year==2010

replace regioncode="622623" if regioncode=="621223" & year==2010

replace regioncode="622625" if regioncode=="621224" & year==2010

replace regioncode="622627" if regioncode=="621225" & year==2010

replace regioncode="622628" if regioncode=="621226" & year==2010

replace regioncode="622630" if regioncode=="621227" & year==2010

replace regioncode="622629" if regioncode=="621228" & year==2010

replace regioncode="622901" if regioncode=="622901" & year==2010

replace regioncode="622921" if regioncode=="622921" & year==2010

replace regioncode="622922" if regioncode=="622922" & year==2010

replace regioncode="622923" if regioncode=="622923" & year==2010

replace regioncode="622924" if regioncode=="622924" & year==2010

replace regioncode="622925" if regioncode=="622925" & year==2010

replace regioncode="622926" if regioncode=="622926" & year==2010

replace regioncode="622927" if regioncode=="622927" & year==2010

replace regioncode="623001" if regioncode=="623001" & year==2010

replace regioncode="623021" if regioncode=="623021" & year==2010

replace regioncode="623022" if regioncode=="623022" & year==2010

replace regioncode="623023" if regioncode=="623023" & year==2010

replace regioncode="623024" if regioncode=="623024" & year==2010

replace regioncode="623025" if regioncode=="623025" & year==2010

replace regioncode="623026" if regioncode=="623026" & year==2010

replace regioncode="623027" if regioncode=="623027" & year==2010

replace regioncode="630102" if regioncode=="630102" & year==2010

replace regioncode="630103" if regioncode=="630103" & year==2010

replace regioncode="630104" if regioncode=="630104" & year==2010

replace regioncode="630105" if regioncode=="630105" & year==2010

replace regioncode="630121" if regioncode=="630121" & year==2010

replace regioncode="630122" if regioncode=="630122" & year==2010

replace regioncode="630123" if regioncode=="630123" & year==2010

replace regioncode="632121" if regioncode=="632121" & year==2010

replace regioncode="632122" if regioncode=="632122" & year==2010

replace regioncode="632123" if regioncode=="632123" & year==2010

replace regioncode="632126" if regioncode=="632126" & year==2010

replace regioncode="632127" if regioncode=="632127" & year==2010

replace regioncode="632128" if regioncode=="632128" & year==2010

replace regioncode="632221" if regioncode=="632221" & year==2010

replace regioncode="632222" if regioncode=="632222" & year==2010

replace regioncode="632223" if regioncode=="632223" & year==2010

replace regioncode="632224" if regioncode=="632224" & year==2010

replace regioncode="632321" if regioncode=="632321" & year==2010

replace regioncode="632322" if regioncode=="632322" & year==2010

replace regioncode="632521" if regioncode=="632521" & year==2010

replace regioncode="632522" if regioncode=="632522" & year==2010

replace regioncode="632523" if regioncode=="632523" & year==2010

replace regioncode="632524" if regioncode=="632524" & year==2010

replace regioncode="632525" if regioncode=="632525" & year==2010

replace regioncode="632621" if regioncode=="632621" & year==2010

replace regioncode="632622" if regioncode=="632622" & year==2010

replace regioncode="632623" if regioncode=="632623" & year==2010

replace regioncode="632624" if regioncode=="632624" & year==2010

replace regioncode="632625" if regioncode=="632625" & year==2010

replace regioncode="632626" if regioncode=="632626" & year==2010

replace regioncode="632721" if regioncode=="632721" & year==2010

replace regioncode="632723" if regioncode=="632723" & year==2010

replace regioncode="632724" if regioncode=="632724" & year==2010

replace regioncode="632725" if regioncode=="632725" & year==2010

replace regioncode="632726" if regioncode=="632726" & year==2010

replace regioncode="632801" if regioncode=="632801" & year==2010

replace regioncode="632802" if regioncode=="632802" & year==2010

replace regioncode="632821" if regioncode=="632821" & year==2010

replace regioncode="632822" if regioncode=="632822" & year==2010

replace regioncode="640104" if regioncode=="640104" & year==2010

replace regioncode="640105" if regioncode=="640105" & year==2010

replace regioncode="640106" if regioncode=="640106" & year==2010

replace regioncode="640121" if regioncode=="640121" & year==2010

replace regioncode="640122" if regioncode=="640122" & year==2010

replace regioncode="640181" if regioncode=="640181" & year==2010

replace regioncode="640202" if regioncode=="640202" & year==2010

replace regioncode="640203" if regioncode=="640205" & year==2010

replace regioncode="640221" if regioncode=="640221" & year==2010

replace regioncode="640302" if regioncode=="640302" & year==2010

replace regioncode="640321" if regioncode=="640502" & year==2010

replace regioncode="640322" if regioncode=="640521" & year==2010

replace regioncode="640323" if regioncode=="640323" & year==2010

replace regioncode="640324" if regioncode=="640324" & year==2010

replace regioncode="640381" if regioncode=="640381" & year==2010

replace regioncode="640402" if regioncode=="640402" & year==2010

replace regioncode="640421" if regioncode=="640522" & year==2010

replace regioncode="640422" if regioncode=="640422" & year==2010

replace regioncode="640423" if regioncode=="640423" & year==2010

replace regioncode="640424" if regioncode=="640424" & year==2010

replace regioncode="640425" if regioncode=="640425" & year==2010

replace regioncode="650102" if regioncode=="650102" & year==2010

replace regioncode="650103" if regioncode=="650103" & year==2010

replace regioncode="650104" if regioncode=="650104" & year==2010

replace regioncode="650105" if regioncode=="650105" & year==2010

replace regioncode="650106" if regioncode=="650106" & year==2010

replace regioncode="650107" if regioncode=="650107" & year==2010

replace regioncode="650108" if regioncode=="650109" & year==2010

replace regioncode="650121" if regioncode=="650121" & year==2010

replace regioncode="650202" if regioncode=="650202" & year==2010

replace regioncode="650203" if regioncode=="650203" & year==2010

replace regioncode="650204" if regioncode=="650204" & year==2010

replace regioncode="650205" if regioncode=="650205" & year==2010

replace regioncode="652101" if regioncode=="652101" & year==2010

replace regioncode="652122" if regioncode=="652122" & year==2010

replace regioncode="652123" if regioncode=="652123" & year==2010

replace regioncode="652201" if regioncode=="652201" & year==2010

replace regioncode="652222" if regioncode=="652222" & year==2010

replace regioncode="652223" if regioncode=="652223" & year==2010

replace regioncode="652301" if regioncode=="652301" & year==2010

replace regioncode="652302" if regioncode=="652302" & year==2010

replace regioncode="652303" if regioncode=="652303" & year==2010

replace regioncode="652323" if regioncode=="652323" & year==2010

replace regioncode="652324" if regioncode=="652324" & year==2010

replace regioncode="652325" if regioncode=="652325" & year==2010

replace regioncode="652327" if regioncode=="652327" & year==2010

replace regioncode="652328" if regioncode=="652328" & year==2010

replace regioncode="652701" if regioncode=="652701" & year==2010

replace regioncode="652722" if regioncode=="652722" & year==2010

replace regioncode="652723" if regioncode=="652723" & year==2010

replace regioncode="652801" if regioncode=="652801" & year==2010

replace regioncode="652822" if regioncode=="652822" & year==2010

replace regioncode="652823" if regioncode=="652823" & year==2010

replace regioncode="652824" if regioncode=="652824" & year==2010

replace regioncode="652825" if regioncode=="652825" & year==2010

replace regioncode="652826" if regioncode=="652826" & year==2010

replace regioncode="652827" if regioncode=="652827" & year==2010

replace regioncode="652828" if regioncode=="652828" & year==2010

replace regioncode="652829" if regioncode=="652829" & year==2010

replace regioncode="652901" if regioncode=="652901" & year==2010

replace regioncode="652922" if regioncode=="652922" & year==2010

replace regioncode="652923" if regioncode=="652923" & year==2010

replace regioncode="652924" if regioncode=="652924" & year==2010

replace regioncode="652925" if regioncode=="652925" & year==2010

replace regioncode="652926" if regioncode=="652926" & year==2010

replace regioncode="652928" if regioncode=="652928" & year==2010

replace regioncode="652929" if regioncode=="652929" & year==2010

replace regioncode="653001" if regioncode=="653001" & year==2010

replace regioncode="653022" if regioncode=="653022" & year==2010

replace regioncode="653023" if regioncode=="653023" & year==2010

replace regioncode="653024" if regioncode=="653024" & year==2010

replace regioncode="653101" if regioncode=="653101" & year==2010

replace regioncode="653121" if regioncode=="653121" & year==2010

replace regioncode="653122" if regioncode=="653122" & year==2010

replace regioncode="653123" if regioncode=="653123" & year==2010

replace regioncode="653124" if regioncode=="653124" & year==2010

replace regioncode="653125" if regioncode=="653125" & year==2010

replace regioncode="653126" if regioncode=="653126" & year==2010

replace regioncode="653127" if regioncode=="653127" & year==2010

replace regioncode="653128" if regioncode=="653128" & year==2010

replace regioncode="653129" if regioncode=="653129" & year==2010

replace regioncode="653130" if regioncode=="653130" & year==2010

replace regioncode="653131" if regioncode=="653131" & year==2010

replace regioncode="653201" if regioncode=="653201" & year==2010

replace regioncode="653221" if regioncode=="653221" & year==2010

replace regioncode="653222" if regioncode=="653222" & year==2010

replace regioncode="653223" if regioncode=="653223" & year==2010

replace regioncode="653224" if regioncode=="653224" & year==2010

replace regioncode="653225" if regioncode=="653225" & year==2010

replace regioncode="653226" if regioncode=="653226" & year==2010

replace regioncode="653227" if regioncode=="653227" & year==2010

replace regioncode="654002" if regioncode=="654002" & year==2010

replace regioncode="654003" if regioncode=="654003" & year==2010

replace regioncode="654021" if regioncode=="654021" & year==2010

replace regioncode="654022" if regioncode=="654022" & year==2010

replace regioncode="654023" if regioncode=="654023" & year==2010

replace regioncode="654024" if regioncode=="654024" & year==2010

replace regioncode="654025" if regioncode=="654025" & year==2010

replace regioncode="654026" if regioncode=="654026" & year==2010

replace regioncode="654027" if regioncode=="654027" & year==2010

replace regioncode="654028" if regioncode=="654028" & year==2010

replace regioncode="654201" if regioncode=="654201" & year==2010

replace regioncode="654202" if regioncode=="654202" & year==2010

replace regioncode="654221" if regioncode=="654221" & year==2010

replace regioncode="654223" if regioncode=="654223" & year==2010

replace regioncode="654224" if regioncode=="654224" & year==2010

replace regioncode="654225" if regioncode=="654225" & year==2010

replace regioncode="654226" if regioncode=="654226" & year==2010

replace regioncode="654301" if regioncode=="654301" & year==2010

replace regioncode="654321" if regioncode=="654321" & year==2010

replace regioncode="654322" if regioncode=="654322" & year==2010

replace regioncode="654323" if regioncode=="654323" & year==2010

replace regioncode="654324" if regioncode=="654324" & year==2010

replace regioncode="654325" if regioncode=="654325" & year==2010

replace regioncode="654326" if regioncode=="654326" & year==2010

replace regioncode="659001" if regioncode=="659001" & year==2010

replace regioncode="110101" if regioncode=="110101" & year==2011

replace regioncode="110102" if regioncode=="110102" & year==2011

replace regioncode="110103" if regioncode=="110103" & year==2011

replace regioncode="110104" if regioncode=="110104" & year==2011

replace regioncode="110105" if regioncode=="110105" & year==2011

replace regioncode="110106" if regioncode=="110106" & year==2011

replace regioncode="110107" if regioncode=="110107" & year==2011

replace regioncode="110108" if regioncode=="110108" & year==2011

replace regioncode="110109" if regioncode=="110109" & year==2011

replace regioncode="110111" if regioncode=="110111" & year==2011

replace regioncode="110112" if regioncode=="110112" & year==2011

replace regioncode="110113" if regioncode=="110113" & year==2011

replace regioncode="110114" if regioncode=="110114" & year==2011

replace regioncode="110115" if regioncode=="110115" & year==2011

replace regioncode="110117" if regioncode=="110117" & year==2011

replace regioncode="110116" if regioncode=="110116" & year==2011

replace regioncode="110228" if regioncode=="110228" & year==2011

replace regioncode="110229" if regioncode=="110229" & year==2011

replace regioncode="120101" if regioncode=="120101" & year==2011

replace regioncode="120102" if regioncode=="120102" & year==2011

replace regioncode="120103" if regioncode=="120103" & year==2011

replace regioncode="120104" if regioncode=="120104" & year==2011

replace regioncode="120105" if regioncode=="120105" & year==2011

replace regioncode="120106" if regioncode=="120106" & year==2011

replace regioncode="120107" if regioncode=="120107" & year==2011

replace regioncode="120108" if regioncode=="120108" & year==2011

replace regioncode="120109" if regioncode=="120109" & year==2011

replace regioncode="120110" if regioncode=="120110" & year==2011

replace regioncode="120111" if regioncode=="120111" & year==2011

replace regioncode="120112" if regioncode=="120112" & year==2011

replace regioncode="120113" if regioncode=="120113" & year==2011

replace regioncode="120114" if regioncode=="120114" & year==2011

replace regioncode="120115" if regioncode=="120115" & year==2011

replace regioncode="120221" if regioncode=="120221" & year==2011

replace regioncode="120223" if regioncode=="120223" & year==2011

replace regioncode="120225" if regioncode=="120225" & year==2011

replace regioncode="130102" if regioncode=="130102" & year==2011

replace regioncode="130103" if regioncode=="130103" & year==2011

replace regioncode="130104" if regioncode=="130104" & year==2011

replace regioncode="130105" if regioncode=="130105" & year==2011

replace regioncode="130107" if regioncode=="130107" & year==2011

replace regioncode="130108" if regioncode=="130108" & year==2011

replace regioncode="130121" if regioncode=="130121" & year==2011

replace regioncode="130123" if regioncode=="130123" & year==2011

replace regioncode="130124" if regioncode=="130124" & year==2011

replace regioncode="130125" if regioncode=="130125" & year==2011

replace regioncode="130126" if regioncode=="130126" & year==2011

replace regioncode="130127" if regioncode=="130127" & year==2011

replace regioncode="130128" if regioncode=="130128" & year==2011

replace regioncode="130129" if regioncode=="130129" & year==2011

replace regioncode="130130" if regioncode=="130130" & year==2011

replace regioncode="130131" if regioncode=="130131" & year==2011

replace regioncode="130132" if regioncode=="130132" & year==2011

replace regioncode="130133" if regioncode=="130133" & year==2011

replace regioncode="130181" if regioncode=="130181" & year==2011

replace regioncode="130182" if regioncode=="130182" & year==2011

replace regioncode="130183" if regioncode=="130183" & year==2011

replace regioncode="130184" if regioncode=="130184" & year==2011

replace regioncode="130185" if regioncode=="130185" & year==2011

replace regioncode="130202" if regioncode=="130202" & year==2011

replace regioncode="130203" if regioncode=="130203" & year==2011

replace regioncode="130204" if regioncode=="130204" & year==2011

replace regioncode="130205" if regioncode=="130205" & year==2011

replace regioncode="130208" if regioncode=="130208" & year==2011

replace regioncode="130223" if regioncode=="130223" & year==2011

replace regioncode="130224" if regioncode=="130224" & year==2011

replace regioncode="130225" if regioncode=="130225" & year==2011

replace regioncode="130227" if regioncode=="130227" & year==2011

replace regioncode="130229" if regioncode=="130229" & year==2011

replace regioncode="130230" if regioncode=="130230" & year==2011

replace regioncode="130281" if regioncode=="130281" & year==2011

replace regioncode="130207" if regioncode=="130207" & year==2011

replace regioncode="130283" if regioncode=="130283" & year==2011

replace regioncode="130302" if regioncode=="130302" & year==2011

replace regioncode="130303" if regioncode=="130303" & year==2011

replace regioncode="130304" if regioncode=="130304" & year==2011

replace regioncode="130321" if regioncode=="130321" & year==2011

replace regioncode="130322" if regioncode=="130322" & year==2011

replace regioncode="130323" if regioncode=="130323" & year==2011

replace regioncode="130324" if regioncode=="130324" & year==2011

replace regioncode="130402" if regioncode=="130402" & year==2011

replace regioncode="130403" if regioncode=="130403" & year==2011

replace regioncode="130404" if regioncode=="130404" & year==2011

replace regioncode="130406" if regioncode=="130406" & year==2011

replace regioncode="130421" if regioncode=="130421" & year==2011

replace regioncode="130423" if regioncode=="130423" & year==2011

replace regioncode="130424" if regioncode=="130424" & year==2011

replace regioncode="130425" if regioncode=="130425" & year==2011

replace regioncode="130426" if regioncode=="130426" & year==2011

replace regioncode="130427" if regioncode=="130427" & year==2011

replace regioncode="130428" if regioncode=="130428" & year==2011

replace regioncode="130429" if regioncode=="130429" & year==2011

replace regioncode="130430" if regioncode=="130430" & year==2011

replace regioncode="130431" if regioncode=="130431" & year==2011

replace regioncode="130432" if regioncode=="130432" & year==2011

replace regioncode="130433" if regioncode=="130433" & year==2011

replace regioncode="130434" if regioncode=="130434" & year==2011

replace regioncode="130435" if regioncode=="130435" & year==2011

replace regioncode="130481" if regioncode=="130481" & year==2011

replace regioncode="130502" if regioncode=="130502" & year==2011

replace regioncode="130503" if regioncode=="130503" & year==2011

replace regioncode="130521" if regioncode=="130521" & year==2011

replace regioncode="130522" if regioncode=="130522" & year==2011

replace regioncode="130523" if regioncode=="130523" & year==2011

replace regioncode="130524" if regioncode=="130524" & year==2011

replace regioncode="130525" if regioncode=="130525" & year==2011

replace regioncode="130526" if regioncode=="130526" & year==2011

replace regioncode="130527" if regioncode=="130527" & year==2011

replace regioncode="130528" if regioncode=="130528" & year==2011

replace regioncode="130529" if regioncode=="130529" & year==2011

replace regioncode="130530" if regioncode=="130530" & year==2011

replace regioncode="130531" if regioncode=="130531" & year==2011

replace regioncode="130532" if regioncode=="130532" & year==2011

replace regioncode="130533" if regioncode=="130533" & year==2011

replace regioncode="130534" if regioncode=="130534" & year==2011

replace regioncode="130535" if regioncode=="130535" & year==2011

replace regioncode="130581" if regioncode=="130581" & year==2011

replace regioncode="130582" if regioncode=="130582" & year==2011

replace regioncode="130602" if regioncode=="130602" & year==2011

replace regioncode="130603" if regioncode=="130603" & year==2011

replace regioncode="130604" if regioncode=="130604" & year==2011

replace regioncode="130621" if regioncode=="130621" & year==2011

replace regioncode="130622" if regioncode=="130622" & year==2011

replace regioncode="130623" if regioncode=="130623" & year==2011

replace regioncode="130624" if regioncode=="130624" & year==2011

replace regioncode="130625" if regioncode=="130625" & year==2011

replace regioncode="130626" if regioncode=="130626" & year==2011

replace regioncode="130627" if regioncode=="130627" & year==2011

replace regioncode="130628" if regioncode=="130628" & year==2011

replace regioncode="130629" if regioncode=="130629" & year==2011

replace regioncode="130630" if regioncode=="130630" & year==2011

replace regioncode="130631" if regioncode=="130631" & year==2011

replace regioncode="130632" if regioncode=="130632" & year==2011

replace regioncode="130633" if regioncode=="130633" & year==2011

replace regioncode="130634" if regioncode=="130634" & year==2011

replace regioncode="130635" if regioncode=="130635" & year==2011

replace regioncode="130636" if regioncode=="130636" & year==2011

replace regioncode="130637" if regioncode=="130637" & year==2011

replace regioncode="130638" if regioncode=="130638" & year==2011

replace regioncode="130681" if regioncode=="130681" & year==2011

replace regioncode="130682" if regioncode=="130682" & year==2011

replace regioncode="130683" if regioncode=="130683" & year==2011

replace regioncode="130684" if regioncode=="130684" & year==2011

replace regioncode="130702" if regioncode=="130702" & year==2011

replace regioncode="130703" if regioncode=="130703" & year==2011

replace regioncode="130705" if regioncode=="130705" & year==2011

replace regioncode="130706" if regioncode=="130706" & year==2011

replace regioncode="130721" if regioncode=="130721" & year==2011

replace regioncode="130722" if regioncode=="130722" & year==2011

replace regioncode="130723" if regioncode=="130723" & year==2011

replace regioncode="130724" if regioncode=="130724" & year==2011

replace regioncode="130725" if regioncode=="130725" & year==2011

replace regioncode="130726" if regioncode=="130726" & year==2011

replace regioncode="130727" if regioncode=="130727" & year==2011

replace regioncode="130728" if regioncode=="130728" & year==2011

replace regioncode="130729" if regioncode=="130729" & year==2011

replace regioncode="130730" if regioncode=="130730" & year==2011

replace regioncode="130731" if regioncode=="130731" & year==2011

replace regioncode="130732" if regioncode=="130732" & year==2011

replace regioncode="130733" if regioncode=="130733" & year==2011

replace regioncode="130802" if regioncode=="130802" & year==2011

replace regioncode="130803" if regioncode=="130803" & year==2011

replace regioncode="130804" if regioncode=="130804" & year==2011

replace regioncode="130821" if regioncode=="130821" & year==2011

replace regioncode="130822" if regioncode=="130822" & year==2011

replace regioncode="130823" if regioncode=="130823" & year==2011

replace regioncode="130824" if regioncode=="130824" & year==2011

replace regioncode="130825" if regioncode=="130825" & year==2011

replace regioncode="130826" if regioncode=="130826" & year==2011

replace regioncode="130827" if regioncode=="130827" & year==2011

replace regioncode="130828" if regioncode=="130828" & year==2011

replace regioncode="130902" if regioncode=="130902" & year==2011

replace regioncode="130903" if regioncode=="130903" & year==2011

replace regioncode="130921" if regioncode=="130921" & year==2011

replace regioncode="130922" if regioncode=="130922" & year==2011

replace regioncode="130923" if regioncode=="130923" & year==2011

replace regioncode="130924" if regioncode=="130924" & year==2011

replace regioncode="130925" if regioncode=="130925" & year==2011

replace regioncode="130926" if regioncode=="130926" & year==2011

replace regioncode="130927" if regioncode=="130927" & year==2011

replace regioncode="130928" if regioncode=="130928" & year==2011

replace regioncode="130929" if regioncode=="130929" & year==2011

replace regioncode="130930" if regioncode=="130930" & year==2011

replace regioncode="130981" if regioncode=="130981" & year==2011

replace regioncode="130982" if regioncode=="130982" & year==2011

replace regioncode="130983" if regioncode=="130983" & year==2011

replace regioncode="130984" if regioncode=="130984" & year==2011

replace regioncode="131002" if regioncode=="131002" & year==2011

replace regioncode="131003" if regioncode=="131003" & year==2011

replace regioncode="131022" if regioncode=="131022" & year==2011

replace regioncode="131023" if regioncode=="131023" & year==2011

replace regioncode="131024" if regioncode=="131024" & year==2011

replace regioncode="131025" if regioncode=="131025" & year==2011

replace regioncode="131026" if regioncode=="131026" & year==2011

replace regioncode="131028" if regioncode=="131028" & year==2011

replace regioncode="131081" if regioncode=="131081" & year==2011

replace regioncode="131082" if regioncode=="131082" & year==2011

replace regioncode="131102" if regioncode=="131102" & year==2011

replace regioncode="131121" if regioncode=="131121" & year==2011

replace regioncode="131122" if regioncode=="131122" & year==2011

replace regioncode="131123" if regioncode=="131123" & year==2011

replace regioncode="131124" if regioncode=="131124" & year==2011

replace regioncode="131125" if regioncode=="131125" & year==2011

replace regioncode="131126" if regioncode=="131126" & year==2011

replace regioncode="131127" if regioncode=="131127" & year==2011

replace regioncode="131128" if regioncode=="131128" & year==2011

replace regioncode="131181" if regioncode=="131181" & year==2011

replace regioncode="131182" if regioncode=="131182" & year==2011

replace regioncode="140105" if regioncode=="140105" & year==2011

replace regioncode="140106" if regioncode=="140106" & year==2011

replace regioncode="140107" if regioncode=="140107" & year==2011

replace regioncode="140108" if regioncode=="140108" & year==2011

replace regioncode="140109" if regioncode=="140109" & year==2011

replace regioncode="140110" if regioncode=="140110" & year==2011

replace regioncode="140121" if regioncode=="140121" & year==2011

replace regioncode="140122" if regioncode=="140122" & year==2011

replace regioncode="140123" if regioncode=="140123" & year==2011

replace regioncode="140181" if regioncode=="140181" & year==2011

replace regioncode="140202" if regioncode=="140202" & year==2011

replace regioncode="140203" if regioncode=="140203" & year==2011

replace regioncode="140211" if regioncode=="140211" & year==2011

replace regioncode="140212" if regioncode=="140212" & year==2011

replace regioncode="140221" if regioncode=="140221" & year==2011

replace regioncode="140222" if regioncode=="140222" & year==2011

replace regioncode="140223" if regioncode=="140223" & year==2011

replace regioncode="140224" if regioncode=="140224" & year==2011

replace regioncode="140225" if regioncode=="140225" & year==2011

replace regioncode="140226" if regioncode=="140226" & year==2011

replace regioncode="140227" if regioncode=="140227" & year==2011

replace regioncode="140302" if regioncode=="140302" & year==2011

replace regioncode="140303" if regioncode=="140303" & year==2011

replace regioncode="140311" if regioncode=="140311" & year==2011

replace regioncode="140321" if regioncode=="140321" & year==2011

replace regioncode="140322" if regioncode=="140322" & year==2011

replace regioncode="140402" if regioncode=="140402" & year==2011

replace regioncode="140411" if regioncode=="140411" & year==2011

replace regioncode="140421" if regioncode=="140421" & year==2011

replace regioncode="140423" if regioncode=="140423" & year==2011

replace regioncode="140424" if regioncode=="140424" & year==2011

replace regioncode="140425" if regioncode=="140425" & year==2011

replace regioncode="140426" if regioncode=="140426" & year==2011

replace regioncode="140427" if regioncode=="140427" & year==2011

replace regioncode="140428" if regioncode=="140428" & year==2011

replace regioncode="140429" if regioncode=="140429" & year==2011

replace regioncode="140430" if regioncode=="140430" & year==2011

replace regioncode="140431" if regioncode=="140431" & year==2011

replace regioncode="140481" if regioncode=="140481" & year==2011

replace regioncode="140502" if regioncode=="140502" & year==2011

replace regioncode="140521" if regioncode=="140521" & year==2011

replace regioncode="140522" if regioncode=="140522" & year==2011

replace regioncode="140524" if regioncode=="140524" & year==2011

replace regioncode="140525" if regioncode=="140525" & year==2011

replace regioncode="140581" if regioncode=="140581" & year==2011

replace regioncode="140602" if regioncode=="140602" & year==2011

replace regioncode="140603" if regioncode=="140603" & year==2011

replace regioncode="140621" if regioncode=="140621" & year==2011

replace regioncode="140622" if regioncode=="140622" & year==2011

replace regioncode="140623" if regioncode=="140623" & year==2011

replace regioncode="140624" if regioncode=="140624" & year==2011

replace regioncode="140902" if regioncode=="140902" & year==2011

replace regioncode="140981" if regioncode=="140981" & year==2011

replace regioncode="140921" if regioncode=="140921" & year==2011

replace regioncode="140922" if regioncode=="140922" & year==2011

replace regioncode="140923" if regioncode=="140923" & year==2011

replace regioncode="140924" if regioncode=="140924" & year==2011

replace regioncode="140925" if regioncode=="140925" & year==2011

replace regioncode="140926" if regioncode=="140926" & year==2011

replace regioncode="140927" if regioncode=="140927" & year==2011

replace regioncode="140928" if regioncode=="140928" & year==2011

replace regioncode="140929" if regioncode=="140929" & year==2011

replace regioncode="140930" if regioncode=="140930" & year==2011

replace regioncode="140931" if regioncode=="140931" & year==2011

replace regioncode="140932" if regioncode=="140932" & year==2011

replace regioncode="141181" if regioncode=="141181" & year==2011

replace regioncode="141102" if regioncode=="141102" & year==2011

replace regioncode="141182" if regioncode=="141182" & year==2011

replace regioncode="141121" if regioncode=="141121" & year==2011

replace regioncode="141122" if regioncode=="141122" & year==2011

replace regioncode="141123" if regioncode=="141123" & year==2011

replace regioncode="141124" if regioncode=="141124" & year==2011

replace regioncode="141125" if regioncode=="141125" & year==2011

replace regioncode="141126" if regioncode=="141126" & year==2011

replace regioncode="141127" if regioncode=="141127" & year==2011

replace regioncode="141128" if regioncode=="141128" & year==2011

replace regioncode="141129" if regioncode=="141129" & year==2011

replace regioncode="141130" if regioncode=="141130" & year==2011

replace regioncode="140702" if regioncode=="140702" & year==2011

replace regioncode="140781" if regioncode=="140781" & year==2011

replace regioncode="140721" if regioncode=="140721" & year==2011

replace regioncode="140722" if regioncode=="140722" & year==2011

replace regioncode="140723" if regioncode=="140723" & year==2011

replace regioncode="140724" if regioncode=="140724" & year==2011

replace regioncode="140725" if regioncode=="140725" & year==2011

replace regioncode="140726" if regioncode=="140726" & year==2011

replace regioncode="140727" if regioncode=="140727" & year==2011

replace regioncode="140728" if regioncode=="140728" & year==2011

replace regioncode="140729" if regioncode=="140729" & year==2011

replace regioncode="141002" if regioncode=="141002" & year==2011

replace regioncode="141081" if regioncode=="141081" & year==2011

replace regioncode="141082" if regioncode=="141082" & year==2011

replace regioncode="141021" if regioncode=="141021" & year==2011

replace regioncode="141022" if regioncode=="141022" & year==2011

replace regioncode="141023" if regioncode=="141023" & year==2011

replace regioncode="141024" if regioncode=="141024" & year==2011

replace regioncode="141025" if regioncode=="141025" & year==2011

replace regioncode="141026" if regioncode=="141026" & year==2011

replace regioncode="141027" if regioncode=="141027" & year==2011

replace regioncode="141028" if regioncode=="141028" & year==2011

replace regioncode="141029" if regioncode=="141029" & year==2011

replace regioncode="141033" if regioncode=="141033" & year==2011

replace regioncode="141030" if regioncode=="141030" & year==2011

replace regioncode="141032" if regioncode=="141032" & year==2011

replace regioncode="141031" if regioncode=="141031" & year==2011

replace regioncode="141034" if regioncode=="141034" & year==2011

replace regioncode="140802" if regioncode=="140802" & year==2011

replace regioncode="140881" if regioncode=="140881" & year==2011

replace regioncode="140882" if regioncode=="140882" & year==2011

replace regioncode="140830" if regioncode=="140830" & year==2011

replace regioncode="140821" if regioncode=="140821" & year==2011

replace regioncode="140822" if regioncode=="140822" & year==2011

replace regioncode="140825" if regioncode=="140825" & year==2011

replace regioncode="140824" if regioncode=="140824" & year==2011

replace regioncode="140823" if regioncode=="140823" & year==2011

replace regioncode="140828" if regioncode=="140828" & year==2011

replace regioncode="140826" if regioncode=="140826" & year==2011

replace regioncode="140829" if regioncode=="140829" & year==2011

replace regioncode="140827" if regioncode=="140827" & year==2011

replace regioncode="150102" if regioncode=="150102" & year==2011

replace regioncode="150103" if regioncode=="150103" & year==2011

replace regioncode="150104" if regioncode=="150104" & year==2011

replace regioncode="150105" if regioncode=="150105" & year==2011

replace regioncode="150121" if regioncode=="150121" & year==2011

replace regioncode="150122" if regioncode=="150122" & year==2011

replace regioncode="150123" if regioncode=="150123" & year==2011

replace regioncode="150124" if regioncode=="150124" & year==2011

replace regioncode="150125" if regioncode=="150125" & year==2011

replace regioncode="150202" if regioncode=="150202" & year==2011

replace regioncode="150203" if regioncode=="150203" & year==2011

replace regioncode="150204" if regioncode=="150204" & year==2011

replace regioncode="150205" if regioncode=="150205" & year==2011

replace regioncode="150206" if regioncode=="150206" & year==2011

replace regioncode="150207" if regioncode=="150207" & year==2011

replace regioncode="150221" if regioncode=="150221" & year==2011

replace regioncode="150222" if regioncode=="150222" & year==2011

replace regioncode="150223" if regioncode=="150223" & year==2011

replace regioncode="150302" if regioncode=="150302" & year==2011

replace regioncode="150303" if regioncode=="150303" & year==2011

replace regioncode="150304" if regioncode=="150304" & year==2011

replace regioncode="150402" if regioncode=="150402" & year==2011

replace regioncode="150403" if regioncode=="150403" & year==2011

replace regioncode="150404" if regioncode=="150404" & year==2011

replace regioncode="150421" if regioncode=="150421" & year==2011

replace regioncode="150422" if regioncode=="150422" & year==2011

replace regioncode="150423" if regioncode=="150423" & year==2011

replace regioncode="150424" if regioncode=="150424" & year==2011

replace regioncode="150425" if regioncode=="150425" & year==2011

replace regioncode="150426" if regioncode=="150426" & year==2011

replace regioncode="150428" if regioncode=="150428" & year==2011

replace regioncode="150429" if regioncode=="150429" & year==2011

replace regioncode="150430" if regioncode=="150430" & year==2011

replace regioncode="150702" if regioncode=="150702" & year==2011

replace regioncode="150781" if regioncode=="150781" & year==2011

replace regioncode="150783" if regioncode=="150783" & year==2011

replace regioncode="150782" if regioncode=="150782" & year==2011

replace regioncode="150785" if regioncode=="150785" & year==2011

replace regioncode="150784" if regioncode=="150784" & year==2011

replace regioncode="150721" if regioncode=="150721" & year==2011

replace regioncode="150722" if regioncode=="150722" & year==2011

replace regioncode="150723" if regioncode=="150723" & year==2011

replace regioncode="150724" if regioncode=="150724" & year==2011

replace regioncode="150727" if regioncode=="150727" & year==2011

replace regioncode="150726" if regioncode=="150726" & year==2011

replace regioncode="150725" if regioncode=="150725" & year==2011

replace regioncode="152201" if regioncode=="152201" & year==2011

replace regioncode="152202" if regioncode=="152202" & year==2011

replace regioncode="152221" if regioncode=="152221" & year==2011

replace regioncode="152222" if regioncode=="152222" & year==2011

replace regioncode="152223" if regioncode=="152223" & year==2011

replace regioncode="152224" if regioncode=="152224" & year==2011

replace regioncode="150502" if regioncode=="150502" & year==2011

replace regioncode="150581" if regioncode=="150581" & year==2011

replace regioncode="150521" if regioncode=="150521" & year==2011

replace regioncode="150522" if regioncode=="150522" & year==2011

replace regioncode="150523" if regioncode=="150523" & year==2011

replace regioncode="150524" if regioncode=="150524" & year==2011

replace regioncode="150525" if regioncode=="150525" & year==2011

replace regioncode="150526" if regioncode=="150526" & year==2011

replace regioncode="152501" if regioncode=="152501" & year==2011

replace regioncode="152502" if regioncode=="152502" & year==2011

replace regioncode="152522" if regioncode=="152522" & year==2011

replace regioncode="152523" if regioncode=="152523" & year==2011

replace regioncode="152524" if regioncode=="152524" & year==2011

replace regioncode="152525" if regioncode=="152525" & year==2011

replace regioncode="152526" if regioncode=="152526" & year==2011

replace regioncode="152527" if regioncode=="152527" & year==2011

replace regioncode="152528" if regioncode=="152528" & year==2011

replace regioncode="152529" if regioncode=="152529" & year==2011

replace regioncode="152530" if regioncode=="152530" & year==2011

replace regioncode="152531" if regioncode=="152531" & year==2011

replace regioncode="152601" if regioncode=="150902" & year==2011

replace regioncode="152602" if regioncode=="150981" & year==2011

replace regioncode="152624" if regioncode=="150921" & year==2011

replace regioncode="152625" if regioncode=="150922" & year==2011

replace regioncode="152626" if regioncode=="150923" & year==2011

replace regioncode="152627" if regioncode=="150924" & year==2011

replace regioncode="152629" if regioncode=="150925" & year==2011

replace regioncode="152630" if regioncode=="150926" & year==2011

replace regioncode="152631" if regioncode=="150927" & year==2011

replace regioncode="152632" if regioncode=="150928" & year==2011

replace regioncode="152634" if regioncode=="150929" & year==2011

replace regioncode="150602" if regioncode=="150602" & year==2011

replace regioncode="150621" if regioncode=="150621" & year==2011

replace regioncode="150622" if regioncode=="150622" & year==2011

replace regioncode="150623" if regioncode=="150623" & year==2011

replace regioncode="150624" if regioncode=="150624" & year==2011

replace regioncode="150625" if regioncode=="150625" & year==2011

replace regioncode="150626" if regioncode=="150626" & year==2011

replace regioncode="150627" if regioncode=="150627" & year==2011

replace regioncode="152801" if regioncode=="150802" & year==2011

replace regioncode="152822" if regioncode=="150821" & year==2011

replace regioncode="152823" if regioncode=="150822" & year==2011

replace regioncode="152824" if regioncode=="150823" & year==2011

replace regioncode="152825" if regioncode=="150824" & year==2011

replace regioncode="152826" if regioncode=="150825" & year==2011

replace regioncode="152827" if regioncode=="150826" & year==2011

replace regioncode="152921" if regioncode=="152921" & year==2011

replace regioncode="152922" if regioncode=="152922" & year==2011

replace regioncode="152923" if regioncode=="152923" & year==2011

replace regioncode="210102" if regioncode=="210102" & year==2011

replace regioncode="210103" if regioncode=="210103" & year==2011

replace regioncode="210104" if regioncode=="210104" & year==2011

replace regioncode="210105" if regioncode=="210105" & year==2011

replace regioncode="210106" if regioncode=="210106" & year==2011

replace regioncode="210111" if regioncode=="210111" & year==2011

replace regioncode="210112" if regioncode=="210112" & year==2011

replace regioncode="210113" if regioncode=="210113" & year==2011

replace regioncode="210114" if regioncode=="210114" & year==2011

replace regioncode="210122" if regioncode=="210122" & year==2011

replace regioncode="210123" if regioncode=="210123" & year==2011

replace regioncode="210124" if regioncode=="210124" & year==2011

replace regioncode="210181" if regioncode=="210181" & year==2011

replace regioncode="210202" if regioncode=="210202" & year==2011

replace regioncode="210203" if regioncode=="210203" & year==2011

replace regioncode="210204" if regioncode=="210204" & year==2011

replace regioncode="210211" if regioncode=="210211" & year==2011

replace regioncode="210212" if regioncode=="210212" & year==2011

replace regioncode="210213" if regioncode=="210213" & year==2011

replace regioncode="210224" if regioncode=="210224" & year==2011

replace regioncode="210281" if regioncode=="210281" & year==2011

replace regioncode="210282" if regioncode=="210282" & year==2011

replace regioncode="210283" if regioncode=="210283" & year==2011

replace regioncode="210302" if regioncode=="210302" & year==2011

replace regioncode="210303" if regioncode=="210303" & year==2011

replace regioncode="210304" if regioncode=="210304" & year==2011

replace regioncode="210311" if regioncode=="210311" & year==2011

replace regioncode="210321" if regioncode=="210321" & year==2011

replace regioncode="210323" if regioncode=="210323" & year==2011

replace regioncode="210381" if regioncode=="210381" & year==2011

replace regioncode="210402" if regioncode=="210402" & year==2011

replace regioncode="210403" if regioncode=="210403" & year==2011

replace regioncode="210404" if regioncode=="210404" & year==2011

replace regioncode="210411" if regioncode=="210411" & year==2011

replace regioncode="210421" if regioncode=="210421" & year==2011

replace regioncode="210422" if regioncode=="210422" & year==2011

replace regioncode="210423" if regioncode=="210423" & year==2011

replace regioncode="210502" if regioncode=="210502" & year==2011

replace regioncode="210503" if regioncode=="210503" & year==2011

replace regioncode="210504" if regioncode=="210504" & year==2011

replace regioncode="210505" if regioncode=="210505" & year==2011

replace regioncode="210521" if regioncode=="210521" & year==2011

replace regioncode="210522" if regioncode=="210522" & year==2011

replace regioncode="210602" if regioncode=="210602" & year==2011

replace regioncode="210603" if regioncode=="210603" & year==2011

replace regioncode="210604" if regioncode=="210604" & year==2011

replace regioncode="210624" if regioncode=="210624" & year==2011

replace regioncode="210681" if regioncode=="210681" & year==2011

replace regioncode="210682" if regioncode=="210682" & year==2011

replace regioncode="210702" if regioncode=="210702" & year==2011

replace regioncode="210703" if regioncode=="210703" & year==2011

replace regioncode="210711" if regioncode=="210711" & year==2011

replace regioncode="210726" if regioncode=="210726" & year==2011

replace regioncode="210727" if regioncode=="210727" & year==2011

replace regioncode="210781" if regioncode=="210781" & year==2011

replace regioncode="210782" if regioncode=="210782" & year==2011

replace regioncode="210802" if regioncode=="210802" & year==2011

replace regioncode="210803" if regioncode=="210803" & year==2011

replace regioncode="210804" if regioncode=="210804" & year==2011

replace regioncode="210811" if regioncode=="210811" & year==2011

replace regioncode="210881" if regioncode=="210881" & year==2011

replace regioncode="210882" if regioncode=="210882" & year==2011

replace regioncode="210902" if regioncode=="210902" & year==2011

replace regioncode="210903" if regioncode=="210903" & year==2011

replace regioncode="210904" if regioncode=="210904" & year==2011

replace regioncode="210905" if regioncode=="210905" & year==2011

replace regioncode="210911" if regioncode=="210911" & year==2011

replace regioncode="210921" if regioncode=="210921" & year==2011

replace regioncode="210922" if regioncode=="210922" & year==2011

replace regioncode="211002" if regioncode=="211002" & year==2011

replace regioncode="211003" if regioncode=="211003" & year==2011

replace regioncode="211004" if regioncode=="211004" & year==2011

replace regioncode="211005" if regioncode=="211005" & year==2011

replace regioncode="211011" if regioncode=="211011" & year==2011

replace regioncode="211021" if regioncode=="211021" & year==2011

replace regioncode="211081" if regioncode=="211081" & year==2011

replace regioncode="211102" if regioncode=="211102" & year==2011

replace regioncode="211103" if regioncode=="211103" & year==2011

replace regioncode="211121" if regioncode=="211121" & year==2011

replace regioncode="211122" if regioncode=="211122" & year==2011

replace regioncode="211202" if regioncode=="211202" & year==2011

replace regioncode="211204" if regioncode=="211204" & year==2011

replace regioncode="211221" if regioncode=="211221" & year==2011

replace regioncode="211223" if regioncode=="211223" & year==2011

replace regioncode="211224" if regioncode=="211224" & year==2011

replace regioncode="211281" if regioncode=="211281" & year==2011

replace regioncode="211282" if regioncode=="211282" & year==2011

replace regioncode="211302" if regioncode=="211302" & year==2011

replace regioncode="211303" if regioncode=="211303" & year==2011

replace regioncode="211321" if regioncode=="211321" & year==2011

replace regioncode="211322" if regioncode=="211322" & year==2011

replace regioncode="211324" if regioncode=="211324" & year==2011

replace regioncode="211381" if regioncode=="211381" & year==2011

replace regioncode="211382" if regioncode=="211382" & year==2011

replace regioncode="211402" if regioncode=="211402" & year==2011

replace regioncode="211403" if regioncode=="211403" & year==2011

replace regioncode="211404" if regioncode=="211404" & year==2011

replace regioncode="211421" if regioncode=="211421" & year==2011

replace regioncode="211422" if regioncode=="211422" & year==2011

replace regioncode="211481" if regioncode=="211481" & year==2011

replace regioncode="220102" if regioncode=="220102" & year==2011

replace regioncode="220103" if regioncode=="220103" & year==2011

replace regioncode="220104" if regioncode=="220104" & year==2011

replace regioncode="220105" if regioncode=="220105" & year==2011

replace regioncode="220106" if regioncode=="220106" & year==2011

replace regioncode="220112" if regioncode=="220112" & year==2011

replace regioncode="220122" if regioncode=="220122" & year==2011

replace regioncode="220181" if regioncode=="220181" & year==2011

replace regioncode="220182" if regioncode=="220182" & year==2011

replace regioncode="220183" if regioncode=="220183" & year==2011

replace regioncode="220202" if regioncode=="220202" & year==2011

replace regioncode="220203" if regioncode=="220203" & year==2011

replace regioncode="220204" if regioncode=="220204" & year==2011

replace regioncode="220211" if regioncode=="220211" & year==2011

replace regioncode="220221" if regioncode=="220221" & year==2011

replace regioncode="220281" if regioncode=="220281" & year==2011

replace regioncode="220282" if regioncode=="220282" & year==2011

replace regioncode="220283" if regioncode=="220283" & year==2011

replace regioncode="220284" if regioncode=="220284" & year==2011

replace regioncode="220302" if regioncode=="220302" & year==2011

replace regioncode="220303" if regioncode=="220303" & year==2011

replace regioncode="220322" if regioncode=="220322" & year==2011

replace regioncode="220323" if regioncode=="220323" & year==2011

replace regioncode="220381" if regioncode=="220381" & year==2011

replace regioncode="220382" if regioncode=="220382" & year==2011

replace regioncode="220402" if regioncode=="220402" & year==2011

replace regioncode="220403" if regioncode=="220403" & year==2011

replace regioncode="220421" if regioncode=="220421" & year==2011

replace regioncode="220422" if regioncode=="220422" & year==2011

replace regioncode="220502" if regioncode=="220502" & year==2011

replace regioncode="220503" if regioncode=="220503" & year==2011

replace regioncode="220521" if regioncode=="220521" & year==2011

replace regioncode="220523" if regioncode=="220523" & year==2011

replace regioncode="220524" if regioncode=="220524" & year==2011

replace regioncode="220581" if regioncode=="220581" & year==2011

replace regioncode="220582" if regioncode=="220582" & year==2011

replace regioncode="220602" if regioncode=="220602" & year==2011

replace regioncode="220621" if regioncode=="220621" & year==2011

replace regioncode="220622" if regioncode=="220622" & year==2011

replace regioncode="220623" if regioncode=="220623" & year==2011

replace regioncode="220625" if regioncode=="220625" & year==2011

replace regioncode="220681" if regioncode=="220681" & year==2011

replace regioncode="220702" if regioncode=="220702" & year==2011

replace regioncode="220721" if regioncode=="220721" & year==2011

replace regioncode="220722" if regioncode=="220722" & year==2011

replace regioncode="220723" if regioncode=="220723" & year==2011

replace regioncode="220724" if regioncode=="220724" & year==2011

replace regioncode="220802" if regioncode=="220802" & year==2011

replace regioncode="220821" if regioncode=="220821" & year==2011

replace regioncode="220822" if regioncode=="220822" & year==2011

replace regioncode="220881" if regioncode=="220881" & year==2011

replace regioncode="220882" if regioncode=="220882" & year==2011

replace regioncode="222401" if regioncode=="222401" & year==2011

replace regioncode="222402" if regioncode=="222402" & year==2011

replace regioncode="222403" if regioncode=="222403" & year==2011

replace regioncode="222404" if regioncode=="222404" & year==2011

replace regioncode="222405" if regioncode=="222405" & year==2011

replace regioncode="222406" if regioncode=="222406" & year==2011

replace regioncode="222424" if regioncode=="222424" & year==2011

replace regioncode="222426" if regioncode=="222426" & year==2011

replace regioncode="230102" if regioncode=="230102" & year==2011

replace regioncode="230103" if regioncode=="230103" & year==2011

replace regioncode="230104" if regioncode=="230104" & year==2011

replace regioncode="230105" if regioncode=="230105" & year==2011

replace regioncode="230107" if regioncode=="230110" & year==2011

replace regioncode="230108" if regioncode=="230108" & year==2011

replace regioncode="230121" if regioncode=="230111" & year==2011

replace regioncode="230123" if regioncode=="230123" & year==2011

replace regioncode="230124" if regioncode=="230124" & year==2011

replace regioncode="230125" if regioncode=="230125" & year==2011

replace regioncode="230126" if regioncode=="230126" & year==2011

replace regioncode="230127" if regioncode=="230127" & year==2011

replace regioncode="230128" if regioncode=="230128" & year==2011

replace regioncode="230129" if regioncode=="230129" & year==2011

replace regioncode="230181" if regioncode=="230112" & year==2011

replace regioncode="230182" if regioncode=="230182" & year==2011

replace regioncode="230183" if regioncode=="230183" & year==2011

replace regioncode="230184" if regioncode=="230184" & year==2011

replace regioncode="230202" if regioncode=="230202" & year==2011

replace regioncode="230203" if regioncode=="230203" & year==2011

replace regioncode="230204" if regioncode=="230204" & year==2011

replace regioncode="230205" if regioncode=="230205" & year==2011

replace regioncode="230206" if regioncode=="230206" & year==2011

replace regioncode="230207" if regioncode=="230207" & year==2011

replace regioncode="230208" if regioncode=="230208" & year==2011

replace regioncode="230221" if regioncode=="230221" & year==2011

replace regioncode="230223" if regioncode=="230223" & year==2011

replace regioncode="230224" if regioncode=="230224" & year==2011

replace regioncode="230225" if regioncode=="230225" & year==2011

replace regioncode="230227" if regioncode=="230227" & year==2011

replace regioncode="230229" if regioncode=="230229" & year==2011

replace regioncode="230230" if regioncode=="230230" & year==2011

replace regioncode="230231" if regioncode=="230231" & year==2011

replace regioncode="230281" if regioncode=="230281" & year==2011

replace regioncode="230302" if regioncode=="230302" & year==2011

replace regioncode="230303" if regioncode=="230303" & year==2011

replace regioncode="230304" if regioncode=="230304" & year==2011

replace regioncode="230305" if regioncode=="230305" & year==2011

replace regioncode="230306" if regioncode=="230306" & year==2011

replace regioncode="230307" if regioncode=="230307" & year==2011

replace regioncode="230321" if regioncode=="230321" & year==2011

replace regioncode="230381" if regioncode=="230381" & year==2011

replace regioncode="230382" if regioncode=="230382" & year==2011

replace regioncode="230402" if regioncode=="230402" & year==2011

replace regioncode="230403" if regioncode=="230403" & year==2011

replace regioncode="230404" if regioncode=="230404" & year==2011

replace regioncode="230405" if regioncode=="230405" & year==2011

replace regioncode="230406" if regioncode=="230406" & year==2011

replace regioncode="230407" if regioncode=="230407" & year==2011

replace regioncode="230421" if regioncode=="230421" & year==2011

replace regioncode="230422" if regioncode=="230422" & year==2011

replace regioncode="230502" if regioncode=="230502" & year==2011

replace regioncode="230503" if regioncode=="230503" & year==2011

replace regioncode="230505" if regioncode=="230505" & year==2011

replace regioncode="230506" if regioncode=="230506" & year==2011

replace regioncode="230521" if regioncode=="230521" & year==2011

replace regioncode="230522" if regioncode=="230522" & year==2011

replace regioncode="230523" if regioncode=="230523" & year==2011

replace regioncode="230524" if regioncode=="230524" & year==2011

replace regioncode="230602" if regioncode=="230602" & year==2011

replace regioncode="230603" if regioncode=="230603" & year==2011

replace regioncode="230604" if regioncode=="230604" & year==2011

replace regioncode="230605" if regioncode=="230605" & year==2011

replace regioncode="230606" if regioncode=="230606" & year==2011

replace regioncode="230621" if regioncode=="230621" & year==2011

replace regioncode="230622" if regioncode=="230622" & year==2011

replace regioncode="230623" if regioncode=="230623" & year==2011

replace regioncode="230624" if regioncode=="230624" & year==2011

replace regioncode="230702" if regioncode=="230702" & year==2011

replace regioncode="230703" if regioncode=="230703" & year==2011

replace regioncode="230704" if regioncode=="230704" & year==2011

replace regioncode="230705" if regioncode=="230705" & year==2011

replace regioncode="230706" if regioncode=="230706" & year==2011

replace regioncode="230707" if regioncode=="230707" & year==2011

replace regioncode="230708" if regioncode=="230708" & year==2011

replace regioncode="230709" if regioncode=="230709" & year==2011

replace regioncode="230710" if regioncode=="230710" & year==2011

replace regioncode="230711" if regioncode=="230711" & year==2011

replace regioncode="230712" if regioncode=="230712" & year==2011

replace regioncode="230713" if regioncode=="230713" & year==2011

replace regioncode="230714" if regioncode=="230714" & year==2011

replace regioncode="230715" if regioncode=="230715" & year==2011

replace regioncode="230716" if regioncode=="230716" & year==2011

replace regioncode="230722" if regioncode=="230722" & year==2011

replace regioncode="230781" if regioncode=="230781" & year==2011

replace regioncode="230802" if regioncode=="230802" & year==2011

replace regioncode="230803" if regioncode=="230803" & year==2011

replace regioncode="230804" if regioncode=="230804" & year==2011

replace regioncode="230805" if regioncode=="230805" & year==2011

replace regioncode="230811" if regioncode=="230811" & year==2011

replace regioncode="230822" if regioncode=="230822" & year==2011

replace regioncode="230826" if regioncode=="230826" & year==2011

replace regioncode="230828" if regioncode=="230828" & year==2011

replace regioncode="230833" if regioncode=="230833" & year==2011

replace regioncode="230881" if regioncode=="230881" & year==2011

replace regioncode="230882" if regioncode=="230882" & year==2011

replace regioncode="230902" if regioncode=="230902" & year==2011

replace regioncode="230903" if regioncode=="230903" & year==2011

replace regioncode="230904" if regioncode=="230904" & year==2011

replace regioncode="230921" if regioncode=="230921" & year==2011

replace regioncode="231002" if regioncode=="231002" & year==2011

replace regioncode="231003" if regioncode=="231003" & year==2011

replace regioncode="231004" if regioncode=="231004" & year==2011

replace regioncode="231005" if regioncode=="231005" & year==2011

replace regioncode="231024" if regioncode=="231024" & year==2011

replace regioncode="231025" if regioncode=="231025" & year==2011

replace regioncode="231081" if regioncode=="231081" & year==2011

replace regioncode="231083" if regioncode=="231083" & year==2011

replace regioncode="231084" if regioncode=="231084" & year==2011

replace regioncode="231085" if regioncode=="231085" & year==2011

replace regioncode="231102" if regioncode=="231102" & year==2011

replace regioncode="231121" if regioncode=="231121" & year==2011

replace regioncode="231123" if regioncode=="231123" & year==2011

replace regioncode="231124" if regioncode=="231124" & year==2011

replace regioncode="231181" if regioncode=="231181" & year==2011

replace regioncode="231182" if regioncode=="231182" & year==2011

replace regioncode="231202" if regioncode=="231202" & year==2011

replace regioncode="231221" if regioncode=="231221" & year==2011

replace regioncode="231222" if regioncode=="231222" & year==2011

replace regioncode="231223" if regioncode=="231223" & year==2011

replace regioncode="231224" if regioncode=="231224" & year==2011

replace regioncode="231225" if regioncode=="231225" & year==2011

replace regioncode="231226" if regioncode=="231226" & year==2011

replace regioncode="231281" if regioncode=="231281" & year==2011

replace regioncode="231282" if regioncode=="231282" & year==2011

replace regioncode="231283" if regioncode=="231283" & year==2011

replace regioncode="232701" if regioncode=="232701" & year==2011

replace regioncode="232702" if regioncode=="232702" & year==2011

replace regioncode="232703" if regioncode=="232703" & year==2011

replace regioncode="232704" if regioncode=="232704" & year==2011

replace regioncode="232721" if regioncode=="232721" & year==2011

replace regioncode="232722" if regioncode=="232722" & year==2011

replace regioncode="232723" if regioncode=="232723" & year==2011

replace regioncode="310101" if regioncode=="310101" & year==2011

replace regioncode="310103" if regioncode=="310103" & year==2011

replace regioncode="310104" if regioncode=="310104" & year==2011

replace regioncode="310105" if regioncode=="310105" & year==2011

replace regioncode="310106" if regioncode=="310106" & year==2011

replace regioncode="310107" if regioncode=="310107" & year==2011

replace regioncode="310108" if regioncode=="310108" & year==2011

replace regioncode="310109" if regioncode=="310109" & year==2011

replace regioncode="310110" if regioncode=="310110" & year==2011

replace regioncode="310112" if regioncode=="310112" & year==2011

replace regioncode="310113" if regioncode=="310113" & year==2011

replace regioncode="310114" if regioncode=="310114" & year==2011

replace regioncode="310115" if regioncode=="310115" & year==2011

replace regioncode="310116" if regioncode=="310116" & year==2011

replace regioncode="310117" if regioncode=="310117" & year==2011

replace regioncode="310118" if regioncode=="310118" & year==2011

replace regioncode="310119" if regioncode=="310119" & year==2011

replace regioncode="310120" if regioncode=="310120" & year==2011

replace regioncode="310230" if regioncode=="310230" & year==2011

replace regioncode="320102" if regioncode=="320102" & year==2011

replace regioncode="320103" if regioncode=="320103" & year==2011

replace regioncode="320104" if regioncode=="320104" & year==2011

replace regioncode="320105" if regioncode=="320105" & year==2011

replace regioncode="320106" if regioncode=="320106" & year==2011

replace regioncode="320107" if regioncode=="320107" & year==2011

replace regioncode="320111" if regioncode=="320111" & year==2011

replace regioncode="320113" if regioncode=="320113" & year==2011

replace regioncode="320114" if regioncode=="320114" & year==2011

replace regioncode="320115" if regioncode=="320115" & year==2011

replace regioncode="320116" if regioncode=="320116" & year==2011

replace regioncode="320124" if regioncode=="320124" & year==2011

replace regioncode="320125" if regioncode=="320125" & year==2011

replace regioncode="320202" if regioncode=="320202" & year==2011

replace regioncode="320203" if regioncode=="320203" & year==2011

replace regioncode="320204" if regioncode=="320204" & year==2011

replace regioncode="320205" if regioncode=="320205" & year==2011

replace regioncode="320211" if regioncode=="320211" & year==2011

replace regioncode="320281" if regioncode=="320281" & year==2011

replace regioncode="320282" if regioncode=="320282" & year==2011

replace regioncode="320302" if regioncode=="320302" & year==2011

replace regioncode="320303" if regioncode=="320303" & year==2011

replace regioncode="320304" if regioncode=="320304" & year==2011

replace regioncode="320305" if regioncode=="320305" & year==2011

replace regioncode="320311" if regioncode=="320311" & year==2011

replace regioncode="320321" if regioncode=="320321" & year==2011

replace regioncode="320322" if regioncode=="320322" & year==2011

replace regioncode="320323" if regioncode=="320323" & year==2011

replace regioncode="320324" if regioncode=="320324" & year==2011

replace regioncode="320381" if regioncode=="320381" & year==2011

replace regioncode="320382" if regioncode=="320382" & year==2011

replace regioncode="320402" if regioncode=="320402" & year==2011

replace regioncode="320404" if regioncode=="320404" & year==2011

replace regioncode="320405" if regioncode=="320405" & year==2011

replace regioncode="320411" if regioncode=="320411" & year==2011

replace regioncode="320412" if regioncode=="320412" & year==2011

replace regioncode="320481" if regioncode=="320481" & year==2011

replace regioncode="320482" if regioncode=="320482" & year==2011

replace regioncode="320502" if regioncode=="320502" & year==2011

replace regioncode="320503" if regioncode=="320503" & year==2011

replace regioncode="320504" if regioncode=="320504" & year==2011

replace regioncode="320505" if regioncode=="320505" & year==2011

replace regioncode="320581" if regioncode=="320581" & year==2011

replace regioncode="320582" if regioncode=="320582" & year==2011

replace regioncode="320583" if regioncode=="320583" & year==2011

replace regioncode="320584" if regioncode=="320584" & year==2011

replace regioncode="320585" if regioncode=="320585" & year==2011

replace regioncode="320586" if regioncode=="320586" & year==2011

replace regioncode="320602" if regioncode=="320602" & year==2011

replace regioncode="320611" if regioncode=="320611" & year==2011

replace regioncode="320621" if regioncode=="320621" & year==2011

replace regioncode="320623" if regioncode=="320623" & year==2011

replace regioncode="320681" if regioncode=="320681" & year==2011

replace regioncode="320682" if regioncode=="320682" & year==2011

replace regioncode="320683" if regioncode=="320683" & year==2011

replace regioncode="320684" if regioncode=="320684" & year==2011

replace regioncode="320703" if regioncode=="320703" & year==2011

replace regioncode="320705" if regioncode=="320705" & year==2011

replace regioncode="320706" if regioncode=="320706" & year==2011

replace regioncode="320721" if regioncode=="320721" & year==2011

replace regioncode="320722" if regioncode=="320722" & year==2011

replace regioncode="320723" if regioncode=="320723" & year==2011

replace regioncode="320724" if regioncode=="320724" & year==2011

replace regioncode="320802" if regioncode=="320802" & year==2011

replace regioncode="320803" if regioncode=="320803" & year==2011

replace regioncode="320804" if regioncode=="320804" & year==2011

replace regioncode="320811" if regioncode=="320811" & year==2011

replace regioncode="320826" if regioncode=="320826" & year==2011

replace regioncode="320829" if regioncode=="320829" & year==2011

replace regioncode="320830" if regioncode=="320830" & year==2011

replace regioncode="320831" if regioncode=="320831" & year==2011

replace regioncode="320902" if regioncode=="320902" & year==2011

replace regioncode="320903" if regioncode=="320903" & year==2011

replace regioncode="320921" if regioncode=="320921" & year==2011

replace regioncode="320922" if regioncode=="320922" & year==2011

replace regioncode="320923" if regioncode=="320923" & year==2011

replace regioncode="320924" if regioncode=="320924" & year==2011

replace regioncode="320925" if regioncode=="320925" & year==2011

replace regioncode="320981" if regioncode=="320981" & year==2011

replace regioncode="320982" if regioncode=="320982" & year==2011

replace regioncode="321002" if regioncode=="321002" & year==2011

replace regioncode="321003" if regioncode=="321003" & year==2011

replace regioncode="321011" if regioncode=="321011" & year==2011

replace regioncode="321023" if regioncode=="321023" & year==2011

replace regioncode="321081" if regioncode=="321081" & year==2011

replace regioncode="321084" if regioncode=="321084" & year==2011

replace regioncode="321088" if regioncode=="321088" & year==2011

replace regioncode="321102" if regioncode=="321102" & year==2011

replace regioncode="321111" if regioncode=="321111" & year==2011

replace regioncode="321112" if regioncode=="321112" & year==2011

replace regioncode="321181" if regioncode=="321181" & year==2011

replace regioncode="321182" if regioncode=="321182" & year==2011

replace regioncode="321183" if regioncode=="321183" & year==2011

replace regioncode="321202" if regioncode=="321202" & year==2011

replace regioncode="321203" if regioncode=="321203" & year==2011

replace regioncode="321281" if regioncode=="321281" & year==2011

replace regioncode="321282" if regioncode=="321282" & year==2011

replace regioncode="321283" if regioncode=="321283" & year==2011

replace regioncode="321284" if regioncode=="321284" & year==2011

replace regioncode="321302" if regioncode=="321302" & year==2011

replace regioncode="321321" if regioncode=="321311" & year==2011

replace regioncode="321322" if regioncode=="321322" & year==2011

replace regioncode="321323" if regioncode=="321323" & year==2011

replace regioncode="321324" if regioncode=="321324" & year==2011

replace regioncode="330102" if regioncode=="330102" & year==2011

replace regioncode="330103" if regioncode=="330103" & year==2011

replace regioncode="330104" if regioncode=="330104" & year==2011

replace regioncode="330105" if regioncode=="330105" & year==2011

replace regioncode="330106" if regioncode=="330106" & year==2011

replace regioncode="330108" if regioncode=="330108" & year==2011

replace regioncode="330109" if regioncode=="330109" & year==2011

replace regioncode="330110" if regioncode=="330110" & year==2011

replace regioncode="330122" if regioncode=="330122" & year==2011

replace regioncode="330127" if regioncode=="330127" & year==2011

replace regioncode="330182" if regioncode=="330182" & year==2011

replace regioncode="330183" if regioncode=="330183" & year==2011

replace regioncode="330185" if regioncode=="330185" & year==2011

replace regioncode="330203" if regioncode=="330203" & year==2011

replace regioncode="330204" if regioncode=="330204" & year==2011

replace regioncode="330205" if regioncode=="330205" & year==2011

replace regioncode="330206" if regioncode=="330206" & year==2011

replace regioncode="330211" if regioncode=="330211" & year==2011

replace regioncode="330212" if regioncode=="330212" & year==2011

replace regioncode="330225" if regioncode=="330225" & year==2011

replace regioncode="330226" if regioncode=="330226" & year==2011

replace regioncode="330281" if regioncode=="330281" & year==2011

replace regioncode="330282" if regioncode=="330282" & year==2011

replace regioncode="330283" if regioncode=="330283" & year==2011

replace regioncode="330302" if regioncode=="330302" & year==2011

replace regioncode="330303" if regioncode=="330303" & year==2011

replace regioncode="330304" if regioncode=="330304" & year==2011

replace regioncode="330322" if regioncode=="330322" & year==2011

replace regioncode="330324" if regioncode=="330324" & year==2011

replace regioncode="330326" if regioncode=="330326" & year==2011

replace regioncode="330327" if regioncode=="330327" & year==2011

replace regioncode="330328" if regioncode=="330328" & year==2011

replace regioncode="330329" if regioncode=="330329" & year==2011

replace regioncode="330381" if regioncode=="330381" & year==2011

replace regioncode="330382" if regioncode=="330382" & year==2011

replace regioncode="330402" if regioncode=="330402" & year==2011

replace regioncode="330411" if regioncode=="330411" & year==2011

replace regioncode="330421" if regioncode=="330421" & year==2011

replace regioncode="330424" if regioncode=="330424" & year==2011

replace regioncode="330481" if regioncode=="330481" & year==2011

replace regioncode="330482" if regioncode=="330482" & year==2011

replace regioncode="330483" if regioncode=="330483" & year==2011

replace regioncode="330501" if regioncode=="330501" & year==2011

replace regioncode="330521" if regioncode=="330521" & year==2011

replace regioncode="330522" if regioncode=="330522" & year==2011

replace regioncode="330523" if regioncode=="330523" & year==2011

replace regioncode="330602" if regioncode=="330602" & year==2011

replace regioncode="330621" if regioncode=="330621" & year==2011

replace regioncode="330624" if regioncode=="330624" & year==2011

replace regioncode="330681" if regioncode=="330681" & year==2011

replace regioncode="330682" if regioncode=="330682" & year==2011

replace regioncode="330683" if regioncode=="330683" & year==2011

replace regioncode="330702" if regioncode=="330702" & year==2011

replace regioncode="330703" if regioncode=="330703" & year==2011

replace regioncode="330723" if regioncode=="330723" & year==2011

replace regioncode="330726" if regioncode=="330726" & year==2011

replace regioncode="330727" if regioncode=="330727" & year==2011

replace regioncode="330781" if regioncode=="330781" & year==2011

replace regioncode="330782" if regioncode=="330782" & year==2011

replace regioncode="330783" if regioncode=="330783" & year==2011

replace regioncode="330784" if regioncode=="330784" & year==2011

replace regioncode="330802" if regioncode=="330802" & year==2011

replace regioncode="330803" if regioncode=="330803" & year==2011

replace regioncode="330822" if regioncode=="330822" & year==2011

replace regioncode="330824" if regioncode=="330824" & year==2011

replace regioncode="330825" if regioncode=="330825" & year==2011

replace regioncode="330881" if regioncode=="330881" & year==2011

replace regioncode="330902" if regioncode=="330902" & year==2011

replace regioncode="330903" if regioncode=="330903" & year==2011

replace regioncode="330921" if regioncode=="330921" & year==2011

replace regioncode="330922" if regioncode=="330922" & year==2011

replace regioncode="331002" if regioncode=="331002" & year==2011

replace regioncode="331003" if regioncode=="331003" & year==2011

replace regioncode="331004" if regioncode=="331004" & year==2011

replace regioncode="331021" if regioncode=="331021" & year==2011

replace regioncode="331022" if regioncode=="331022" & year==2011

replace regioncode="331023" if regioncode=="331023" & year==2011

replace regioncode="331024" if regioncode=="331024" & year==2011

replace regioncode="331081" if regioncode=="331081" & year==2011

replace regioncode="331082" if regioncode=="331082" & year==2011

replace regioncode="331102" if regioncode=="331102" & year==2011

replace regioncode="331121" if regioncode=="331121" & year==2011

replace regioncode="331122" if regioncode=="331122" & year==2011

replace regioncode="331123" if regioncode=="331123" & year==2011

replace regioncode="331124" if regioncode=="331124" & year==2011

replace regioncode="331125" if regioncode=="331125" & year==2011

replace regioncode="331126" if regioncode=="331126" & year==2011

replace regioncode="331127" if regioncode=="331127" & year==2011

replace regioncode="331181" if regioncode=="331181" & year==2011

replace regioncode="340102" if regioncode=="340102" & year==2011

replace regioncode="340103" if regioncode=="340103" & year==2011

replace regioncode="340104" if regioncode=="340104" & year==2011

replace regioncode="340111" if regioncode=="340111" & year==2011

replace regioncode="340121" if regioncode=="340121" & year==2011

replace regioncode="340122" if regioncode=="340122" & year==2011

replace regioncode="340123" if regioncode=="340123" & year==2011

replace regioncode="340202" if regioncode=="340202" & year==2011

replace regioncode="340203" if regioncode=="340203" & year==2011

replace regioncode="340204" if regioncode=="340204" & year==2011

replace regioncode="340207" if regioncode=="340207" & year==2011

replace regioncode="340221" if regioncode=="340221" & year==2011

replace regioncode="340222" if regioncode=="340222" & year==2011

replace regioncode="340223" if regioncode=="340223" & year==2011

replace regioncode="340302" if regioncode=="340302" & year==2011

replace regioncode="340303" if regioncode=="340303" & year==2011

replace regioncode="340304" if regioncode=="340304" & year==2011

replace regioncode="340311" if regioncode=="340311" & year==2011

replace regioncode="340321" if regioncode=="340321" & year==2011

replace regioncode="340322" if regioncode=="340322" & year==2011

replace regioncode="340323" if regioncode=="340323" & year==2011

replace regioncode="340402" if regioncode=="340402" & year==2011

replace regioncode="340403" if regioncode=="340403" & year==2011

replace regioncode="340404" if regioncode=="340404" & year==2011

replace regioncode="340405" if regioncode=="340405" & year==2011

replace regioncode="340406" if regioncode=="340406" & year==2011

replace regioncode="340421" if regioncode=="340421" & year==2011

replace regioncode="340502" if regioncode=="340502" & year==2011

replace regioncode="340503" if regioncode=="340503" & year==2011

replace regioncode="340504" if regioncode=="340504" & year==2011

replace regioncode="340521" if regioncode=="340521" & year==2011

replace regioncode="340602" if regioncode=="340602" & year==2011

replace regioncode="340603" if regioncode=="340603" & year==2011

replace regioncode="340604" if regioncode=="340604" & year==2011

replace regioncode="340621" if regioncode=="340621" & year==2011

replace regioncode="340702" if regioncode=="340702" & year==2011

replace regioncode="340703" if regioncode=="340703" & year==2011

replace regioncode="340711" if regioncode=="340711" & year==2011

replace regioncode="340721" if regioncode=="340721" & year==2011

replace regioncode="340802" if regioncode=="340802" & year==2011

replace regioncode="340803" if regioncode=="340803" & year==2011

replace regioncode="340811" if regioncode=="340811" & year==2011

replace regioncode="340822" if regioncode=="340822" & year==2011

replace regioncode="340823" if regioncode=="340823" & year==2011

replace regioncode="340824" if regioncode=="340824" & year==2011

replace regioncode="340825" if regioncode=="340825" & year==2011

replace regioncode="340826" if regioncode=="340826" & year==2011

replace regioncode="340827" if regioncode=="340827" & year==2011

replace regioncode="340828" if regioncode=="340828" & year==2011

replace regioncode="340881" if regioncode=="340881" & year==2011

replace regioncode="341002" if regioncode=="341002" & year==2011

replace regioncode="341003" if regioncode=="341003" & year==2011

replace regioncode="341004" if regioncode=="341004" & year==2011

replace regioncode="341021" if regioncode=="341021" & year==2011

replace regioncode="341022" if regioncode=="341022" & year==2011

replace regioncode="341023" if regioncode=="341023" & year==2011

replace regioncode="341024" if regioncode=="341024" & year==2011

replace regioncode="341102" if regioncode=="341102" & year==2011

replace regioncode="341103" if regioncode=="341103" & year==2011

replace regioncode="341122" if regioncode=="341122" & year==2011

replace regioncode="341124" if regioncode=="341124" & year==2011

replace regioncode="341125" if regioncode=="341125" & year==2011

replace regioncode="341126" if regioncode=="341126" & year==2011

replace regioncode="341181" if regioncode=="341181" & year==2011

replace regioncode="341182" if regioncode=="341182" & year==2011

replace regioncode="341202" if regioncode=="341202" & year==2011

replace regioncode="341203" if regioncode=="341203" & year==2011

replace regioncode="341204" if regioncode=="341204" & year==2011

replace regioncode="341221" if regioncode=="341221" & year==2011

replace regioncode="341222" if regioncode=="341222" & year==2011

replace regioncode="341621" if regioncode=="341621" & year==2011

replace regioncode="341622" if regioncode=="341622" & year==2011

replace regioncode="341225" if regioncode=="341225" & year==2011

replace regioncode="341226" if regioncode=="341226" & year==2011

replace regioncode="341623" if regioncode=="341623" & year==2011

replace regioncode="341282" if regioncode=="341282" & year==2011

replace regioncode="341302" if regioncode=="341302" & year==2011

replace regioncode="341321" if regioncode=="341321" & year==2011

replace regioncode="341322" if regioncode=="341322" & year==2011

replace regioncode="341323" if regioncode=="341323" & year==2011

replace regioncode="341324" if regioncode=="341324" & year==2011

replace regioncode="341402" if regioncode=="341402" & year==2011

replace regioncode="341421" if regioncode=="341421" & year==2011

replace regioncode="341422" if regioncode=="341422" & year==2011

replace regioncode="341423" if regioncode=="341423" & year==2011

replace regioncode="341424" if regioncode=="341424" & year==2011

replace regioncode="341521" if regioncode=="341521" & year==2011

replace regioncode="341522" if regioncode=="341522" & year==2011

replace regioncode="341523" if regioncode=="341523" & year==2011

replace regioncode="341524" if regioncode=="341524" & year==2011

replace regioncode="341525" if regioncode=="341525" & year==2011

replace regioncode="341802" if regioncode=="341802" & year==2011

replace regioncode="341881" if regioncode=="341881" & year==2011

replace regioncode="341821" if regioncode=="341821" & year==2011

replace regioncode="341822" if regioncode=="341822" & year==2011

replace regioncode="341823" if regioncode=="341823" & year==2011

replace regioncode="341825" if regioncode=="341825" & year==2011

replace regioncode="341824" if regioncode=="341824" & year==2011

replace regioncode="341702" if regioncode=="341702" & year==2011

replace regioncode="341721" if regioncode=="341721" & year==2011

replace regioncode="341722" if regioncode=="341722" & year==2011

replace regioncode="341723" if regioncode=="341723" & year==2011

replace regioncode="341502" if regioncode=="341502" & year==2011

replace regioncode="341602" if regioncode=="341602" & year==2011

replace regioncode="350102" if regioncode=="350102" & year==2011

replace regioncode="350103" if regioncode=="350103" & year==2011

replace regioncode="350104" if regioncode=="350104" & year==2011

replace regioncode="350105" if regioncode=="350105" & year==2011

replace regioncode="350111" if regioncode=="350111" & year==2011

replace regioncode="350121" if regioncode=="350121" & year==2011

replace regioncode="350122" if regioncode=="350122" & year==2011

replace regioncode="350123" if regioncode=="350123" & year==2011

replace regioncode="350124" if regioncode=="350124" & year==2011

replace regioncode="350125" if regioncode=="350125" & year==2011

replace regioncode="350128" if regioncode=="350128" & year==2011

replace regioncode="350181" if regioncode=="350181" & year==2011

replace regioncode="350182" if regioncode=="350182" & year==2011

replace regioncode="350203" if regioncode=="350203" & year==2011

replace regioncode="350205" if regioncode=="350205" & year==2011

replace regioncode="350206" if regioncode=="350206" & year==2011

replace regioncode="350211" if regioncode=="350211" & year==2011

replace regioncode="350212" if regioncode=="350212" & year==2011

replace regioncode="350213" if regioncode=="350213" & year==2011

replace regioncode="350302" if regioncode=="350302" & year==2011

replace regioncode="350303" if regioncode=="350303" & year==2011

replace regioncode="350321" if regioncode=="350321" & year==2011

replace regioncode="350322" if regioncode=="350322" & year==2011

replace regioncode="350402" if regioncode=="350402" & year==2011

replace regioncode="350403" if regioncode=="350403" & year==2011

replace regioncode="350421" if regioncode=="350421" & year==2011

replace regioncode="350423" if regioncode=="350423" & year==2011

replace regioncode="350424" if regioncode=="350424" & year==2011

replace regioncode="350425" if regioncode=="350425" & year==2011

replace regioncode="350426" if regioncode=="350426" & year==2011

replace regioncode="350427" if regioncode=="350427" & year==2011

replace regioncode="350428" if regioncode=="350428" & year==2011

replace regioncode="350429" if regioncode=="350429" & year==2011

replace regioncode="350430" if regioncode=="350430" & year==2011

replace regioncode="350481" if regioncode=="350481" & year==2011

replace regioncode="350502" if regioncode=="350502" & year==2011

replace regioncode="350503" if regioncode=="350503" & year==2011

replace regioncode="350504" if regioncode=="350504" & year==2011

replace regioncode="350505" if regioncode=="350505" & year==2011

replace regioncode="350521" if regioncode=="350521" & year==2011

replace regioncode="350524" if regioncode=="350524" & year==2011

replace regioncode="350525" if regioncode=="350525" & year==2011

replace regioncode="350526" if regioncode=="350526" & year==2011

replace regioncode="350581" if regioncode=="350581" & year==2011

replace regioncode="350582" if regioncode=="350582" & year==2011

replace regioncode="350583" if regioncode=="350583" & year==2011

replace regioncode="350602" if regioncode=="350602" & year==2011

replace regioncode="350603" if regioncode=="350603" & year==2011

replace regioncode="350622" if regioncode=="350622" & year==2011

replace regioncode="350623" if regioncode=="350623" & year==2011

replace regioncode="350624" if regioncode=="350624" & year==2011

replace regioncode="350625" if regioncode=="350625" & year==2011

replace regioncode="350626" if regioncode=="350626" & year==2011

replace regioncode="350627" if regioncode=="350627" & year==2011

replace regioncode="350628" if regioncode=="350628" & year==2011

replace regioncode="350629" if regioncode=="350629" & year==2011

replace regioncode="350681" if regioncode=="350681" & year==2011

replace regioncode="350702" if regioncode=="350702" & year==2011

replace regioncode="350721" if regioncode=="350721" & year==2011

replace regioncode="350722" if regioncode=="350722" & year==2011

replace regioncode="350723" if regioncode=="350723" & year==2011

replace regioncode="350724" if regioncode=="350724" & year==2011

replace regioncode="350725" if regioncode=="350725" & year==2011

replace regioncode="350781" if regioncode=="350781" & year==2011

replace regioncode="350782" if regioncode=="350782" & year==2011

replace regioncode="350783" if regioncode=="350783" & year==2011

replace regioncode="350784" if regioncode=="350784" & year==2011

replace regioncode="350802" if regioncode=="350802" & year==2011

replace regioncode="350821" if regioncode=="350821" & year==2011

replace regioncode="350822" if regioncode=="350822" & year==2011

replace regioncode="350823" if regioncode=="350823" & year==2011

replace regioncode="350824" if regioncode=="350824" & year==2011

replace regioncode="350825" if regioncode=="350825" & year==2011

replace regioncode="350881" if regioncode=="350881" & year==2011

replace regioncode="350902" if regioncode=="350902" & year==2011

replace regioncode="350981" if regioncode=="350981" & year==2011

replace regioncode="350982" if regioncode=="350982" & year==2011

replace regioncode="350921" if regioncode=="350921" & year==2011

replace regioncode="350922" if regioncode=="350922" & year==2011

replace regioncode="350923" if regioncode=="350923" & year==2011

replace regioncode="350924" if regioncode=="350924" & year==2011

replace regioncode="350925" if regioncode=="350925" & year==2011

replace regioncode="350926" if regioncode=="350926" & year==2011

replace regioncode="360102" if regioncode=="360102" & year==2011

replace regioncode="360103" if regioncode=="360103" & year==2011

replace regioncode="360104" if regioncode=="360104" & year==2011

replace regioncode="360105" if regioncode=="360105" & year==2011

replace regioncode="360111" if regioncode=="360111" & year==2011

replace regioncode="360121" if regioncode=="360121" & year==2011

replace regioncode="360122" if regioncode=="360122" & year==2011

replace regioncode="360123" if regioncode=="360123" & year==2011

replace regioncode="360124" if regioncode=="360124" & year==2011

replace regioncode="360202" if regioncode=="360202" & year==2011

replace regioncode="360203" if regioncode=="360203" & year==2011

replace regioncode="360222" if regioncode=="360222" & year==2011

replace regioncode="360281" if regioncode=="360281" & year==2011

replace regioncode="360302" if regioncode=="360302" & year==2011

replace regioncode="360313" if regioncode=="360313" & year==2011

replace regioncode="360321" if regioncode=="360321" & year==2011

replace regioncode="360322" if regioncode=="360322" & year==2011

replace regioncode="360323" if regioncode=="360323" & year==2011

replace regioncode="360402" if regioncode=="360402" & year==2011

replace regioncode="360403" if regioncode=="360403" & year==2011

replace regioncode="360421" if regioncode=="360421" & year==2011

replace regioncode="360423" if regioncode=="360423" & year==2011

replace regioncode="360424" if regioncode=="360424" & year==2011

replace regioncode="360425" if regioncode=="360425" & year==2011

replace regioncode="360426" if regioncode=="360426" & year==2011

replace regioncode="360427" if regioncode=="360427" & year==2011

replace regioncode="360428" if regioncode=="360428" & year==2011

replace regioncode="360429" if regioncode=="360429" & year==2011

replace regioncode="360430" if regioncode=="360430" & year==2011

replace regioncode="360481" if regioncode=="360481" & year==2011

replace regioncode="360502" if regioncode=="360502" & year==2011

replace regioncode="360521" if regioncode=="360521" & year==2011

replace regioncode="360602" if regioncode=="360602" & year==2011

replace regioncode="360622" if regioncode=="360622" & year==2011

replace regioncode="360681" if regioncode=="360681" & year==2011

replace regioncode="360702" if regioncode=="360702" & year==2011

replace regioncode="360721" if regioncode=="360721" & year==2011

replace regioncode="360722" if regioncode=="360722" & year==2011

replace regioncode="360723" if regioncode=="360723" & year==2011

replace regioncode="360724" if regioncode=="360724" & year==2011

replace regioncode="360725" if regioncode=="360725" & year==2011

replace regioncode="360726" if regioncode=="360726" & year==2011

replace regioncode="360727" if regioncode=="360727" & year==2011

replace regioncode="360728" if regioncode=="360728" & year==2011

replace regioncode="360729" if regioncode=="360729" & year==2011

replace regioncode="360730" if regioncode=="360730" & year==2011

replace regioncode="360731" if regioncode=="360731" & year==2011

replace regioncode="360732" if regioncode=="360732" & year==2011

replace regioncode="360733" if regioncode=="360733" & year==2011

replace regioncode="360734" if regioncode=="360734" & year==2011

replace regioncode="360735" if regioncode=="360735" & year==2011

replace regioncode="360781" if regioncode=="360781" & year==2011

replace regioncode="360782" if regioncode=="360782" & year==2011

replace regioncode="360802" if regioncode=="360802" & year==2011

replace regioncode="360821" if regioncode=="360821" & year==2011

replace regioncode="360822" if regioncode=="360822" & year==2011

replace regioncode="360823" if regioncode=="360823" & year==2011

replace regioncode="360824" if regioncode=="360824" & year==2011

replace regioncode="360825" if regioncode=="360825" & year==2011

replace regioncode="360826" if regioncode=="360826" & year==2011

replace regioncode="360827" if regioncode=="360827" & year==2011

replace regioncode="360828" if regioncode=="360828" & year==2011

replace regioncode="360829" if regioncode=="360829" & year==2011

replace regioncode="360830" if regioncode=="360830" & year==2011

replace regioncode="360881" if regioncode=="360881" & year==2011

replace regioncode="360902" if regioncode=="360902" & year==2011

replace regioncode="360921" if regioncode=="360921" & year==2011

replace regioncode="360922" if regioncode=="360922" & year==2011

replace regioncode="360923" if regioncode=="360923" & year==2011

replace regioncode="360924" if regioncode=="360924" & year==2011

replace regioncode="360925" if regioncode=="360925" & year==2011

replace regioncode="360926" if regioncode=="360926" & year==2011

replace regioncode="360981" if regioncode=="360981" & year==2011

replace regioncode="360982" if regioncode=="360982" & year==2011

replace regioncode="360983" if regioncode=="360983" & year==2011

replace regioncode="361002" if regioncode=="361002" & year==2011

replace regioncode="361021" if regioncode=="361021" & year==2011

replace regioncode="361022" if regioncode=="361022" & year==2011

replace regioncode="361023" if regioncode=="361023" & year==2011

replace regioncode="361024" if regioncode=="361024" & year==2011

replace regioncode="361025" if regioncode=="361025" & year==2011

replace regioncode="361026" if regioncode=="361026" & year==2011

replace regioncode="361027" if regioncode=="361027" & year==2011

replace regioncode="361028" if regioncode=="361028" & year==2011

replace regioncode="361029" if regioncode=="361029" & year==2011

replace regioncode="361030" if regioncode=="361030" & year==2011

replace regioncode="361102" if regioncode=="361102" & year==2011

replace regioncode="361121" if regioncode=="361121" & year==2011

replace regioncode="361122" if regioncode=="361122" & year==2011

replace regioncode="361123" if regioncode=="361123" & year==2011

replace regioncode="361124" if regioncode=="361124" & year==2011

replace regioncode="361125" if regioncode=="361125" & year==2011

replace regioncode="361126" if regioncode=="361126" & year==2011

replace regioncode="361127" if regioncode=="361127" & year==2011

replace regioncode="361128" if regioncode=="361128" & year==2011

replace regioncode="361129" if regioncode=="361129" & year==2011

replace regioncode="361130" if regioncode=="361130" & year==2011

replace regioncode="361181" if regioncode=="361181" & year==2011

replace regioncode="370102" if regioncode=="370102" & year==2011

replace regioncode="370103" if regioncode=="370103" & year==2011

replace regioncode="370104" if regioncode=="370104" & year==2011

replace regioncode="370105" if regioncode=="370105" & year==2011

replace regioncode="370112" if regioncode=="370112" & year==2011

replace regioncode="370113" if regioncode=="370113" & year==2011

replace regioncode="370124" if regioncode=="370124" & year==2011

replace regioncode="370125" if regioncode=="370125" & year==2011

replace regioncode="370126" if regioncode=="370126" & year==2011

replace regioncode="370181" if regioncode=="370181" & year==2011

replace regioncode="370202" if regioncode=="370202" & year==2011

replace regioncode="370203" if regioncode=="370203" & year==2011

replace regioncode="370205" if regioncode=="370205" & year==2011

replace regioncode="370211" if regioncode=="370211" & year==2011

replace regioncode="370212" if regioncode=="370212" & year==2011

replace regioncode="370213" if regioncode=="370213" & year==2011

replace regioncode="370214" if regioncode=="370214" & year==2011

replace regioncode="370281" if regioncode=="370281" & year==2011

replace regioncode="370282" if regioncode=="370282" & year==2011

replace regioncode="370283" if regioncode=="370283" & year==2011

replace regioncode="370284" if regioncode=="370284" & year==2011

replace regioncode="370285" if regioncode=="370285" & year==2011

replace regioncode="370302" if regioncode=="370302" & year==2011

replace regioncode="370303" if regioncode=="370303" & year==2011

replace regioncode="370304" if regioncode=="370304" & year==2011

replace regioncode="370305" if regioncode=="370305" & year==2011

replace regioncode="370306" if regioncode=="370306" & year==2011

replace regioncode="370321" if regioncode=="370321" & year==2011

replace regioncode="370322" if regioncode=="370322" & year==2011

replace regioncode="370323" if regioncode=="370323" & year==2011

replace regioncode="370402" if regioncode=="370402" & year==2011

replace regioncode="370403" if regioncode=="370403" & year==2011

replace regioncode="370404" if regioncode=="370404" & year==2011

replace regioncode="370405" if regioncode=="370405" & year==2011

replace regioncode="370406" if regioncode=="370406" & year==2011

replace regioncode="370481" if regioncode=="370481" & year==2011

replace regioncode="370502" if regioncode=="370502" & year==2011

replace regioncode="370503" if regioncode=="370503" & year==2011

replace regioncode="370521" if regioncode=="370521" & year==2011

replace regioncode="370522" if regioncode=="370522" & year==2011

replace regioncode="370523" if regioncode=="370523" & year==2011

replace regioncode="370602" if regioncode=="370602" & year==2011

replace regioncode="370611" if regioncode=="370611" & year==2011

replace regioncode="370612" if regioncode=="370612" & year==2011

replace regioncode="370613" if regioncode=="370613" & year==2011

replace regioncode="370634" if regioncode=="370634" & year==2011

replace regioncode="370681" if regioncode=="370681" & year==2011

replace regioncode="370682" if regioncode=="370682" & year==2011

replace regioncode="370683" if regioncode=="370683" & year==2011

replace regioncode="370684" if regioncode=="370684" & year==2011

replace regioncode="370685" if regioncode=="370685" & year==2011

replace regioncode="370686" if regioncode=="370686" & year==2011

replace regioncode="370687" if regioncode=="370687" & year==2011

replace regioncode="370702" if regioncode=="370702" & year==2011

replace regioncode="370703" if regioncode=="370703" & year==2011

replace regioncode="370704" if regioncode=="370704" & year==2011

replace regioncode="370705" if regioncode=="370705" & year==2011

replace regioncode="370724" if regioncode=="370724" & year==2011

replace regioncode="370725" if regioncode=="370725" & year==2011

replace regioncode="370781" if regioncode=="370781" & year==2011

replace regioncode="370782" if regioncode=="370782" & year==2011

replace regioncode="370783" if regioncode=="370783" & year==2011

replace regioncode="370784" if regioncode=="370784" & year==2011

replace regioncode="370785" if regioncode=="370785" & year==2011

replace regioncode="370786" if regioncode=="370786" & year==2011

replace regioncode="370802" if regioncode=="370802" & year==2011

replace regioncode="370811" if regioncode=="370811" & year==2011

replace regioncode="370826" if regioncode=="370826" & year==2011

replace regioncode="370827" if regioncode=="370827" & year==2011

replace regioncode="370828" if regioncode=="370828" & year==2011

replace regioncode="370829" if regioncode=="370829" & year==2011

replace regioncode="370830" if regioncode=="370830" & year==2011

replace regioncode="370831" if regioncode=="370831" & year==2011

replace regioncode="370832" if regioncode=="370832" & year==2011

replace regioncode="370881" if regioncode=="370881" & year==2011

replace regioncode="370882" if regioncode=="370882" & year==2011

replace regioncode="370883" if regioncode=="370883" & year==2011

replace regioncode="370902" if regioncode=="370902" & year==2011

replace regioncode="370903" if regioncode=="370911" & year==2011

replace regioncode="370921" if regioncode=="370921" & year==2011

replace regioncode="370923" if regioncode=="370923" & year==2011

replace regioncode="370982" if regioncode=="370982" & year==2011

replace regioncode="370983" if regioncode=="370983" & year==2011

replace regioncode="371002" if regioncode=="371002" & year==2011

replace regioncode="371081" if regioncode=="371081" & year==2011

replace regioncode="371082" if regioncode=="371082" & year==2011

replace regioncode="371083" if regioncode=="371083" & year==2011

replace regioncode="371102" if regioncode=="371102" & year==2011

replace regioncode="371121" if regioncode=="371121" & year==2011

replace regioncode="371122" if regioncode=="371122" & year==2011

replace regioncode="371202" if regioncode=="371202" & year==2011

replace regioncode="371203" if regioncode=="371203" & year==2011

replace regioncode="371302" if regioncode=="371302" & year==2011

replace regioncode="371311" if regioncode=="371311" & year==2011

replace regioncode="371312" if regioncode=="371312" & year==2011

replace regioncode="371321" if regioncode=="371321" & year==2011

replace regioncode="371322" if regioncode=="371322" & year==2011

replace regioncode="371323" if regioncode=="371323" & year==2011

replace regioncode="371324" if regioncode=="371324" & year==2011

replace regioncode="371325" if regioncode=="371325" & year==2011

replace regioncode="371326" if regioncode=="371326" & year==2011

replace regioncode="371327" if regioncode=="371327" & year==2011

replace regioncode="371328" if regioncode=="371328" & year==2011

replace regioncode="371329" if regioncode=="371329" & year==2011

replace regioncode="371402" if regioncode=="371402" & year==2011

replace regioncode="371421" if regioncode=="371421" & year==2011

replace regioncode="371422" if regioncode=="371422" & year==2011

replace regioncode="371423" if regioncode=="371423" & year==2011

replace regioncode="371424" if regioncode=="371424" & year==2011

replace regioncode="371425" if regioncode=="371425" & year==2011

replace regioncode="371426" if regioncode=="371426" & year==2011

replace regioncode="371427" if regioncode=="371427" & year==2011

replace regioncode="371428" if regioncode=="371428" & year==2011

replace regioncode="371481" if regioncode=="371481" & year==2011

replace regioncode="371482" if regioncode=="371482" & year==2011

replace regioncode="371502" if regioncode=="371502" & year==2011

replace regioncode="371521" if regioncode=="371521" & year==2011

replace regioncode="371522" if regioncode=="371522" & year==2011

replace regioncode="371523" if regioncode=="371523" & year==2011

replace regioncode="371524" if regioncode=="371524" & year==2011

replace regioncode="371525" if regioncode=="371525" & year==2011

replace regioncode="371526" if regioncode=="371526" & year==2011

replace regioncode="371581" if regioncode=="371581" & year==2011

replace regioncode="371602" if regioncode=="371602" & year==2011

replace regioncode="371621" if regioncode=="371621" & year==2011

replace regioncode="371622" if regioncode=="371622" & year==2011

replace regioncode="371623" if regioncode=="371623" & year==2011

replace regioncode="371624" if regioncode=="371624" & year==2011

replace regioncode="371625" if regioncode=="371625" & year==2011

replace regioncode="371626" if regioncode=="371626" & year==2011

replace regioncode="371702" if regioncode=="371702" & year==2011

replace regioncode="371721" if regioncode=="371721" & year==2011

replace regioncode="371722" if regioncode=="371722" & year==2011

replace regioncode="371723" if regioncode=="371723" & year==2011

replace regioncode="371724" if regioncode=="371724" & year==2011

replace regioncode="371725" if regioncode=="371725" & year==2011

replace regioncode="371726" if regioncode=="371726" & year==2011

replace regioncode="371727" if regioncode=="371727" & year==2011

replace regioncode="371728" if regioncode=="371728" & year==2011

replace regioncode="410102" if regioncode=="410102" & year==2011

replace regioncode="410103" if regioncode=="410103" & year==2011

replace regioncode="410104" if regioncode=="410104" & year==2011

replace regioncode="410105" if regioncode=="410105" & year==2011

replace regioncode="410106" if regioncode=="410106" & year==2011

replace regioncode="410108" if regioncode=="410108" & year==2011

replace regioncode="410122" if regioncode=="410122" & year==2011

replace regioncode="410181" if regioncode=="410181" & year==2011

replace regioncode="410182" if regioncode=="410182" & year==2011

replace regioncode="410183" if regioncode=="410183" & year==2011

replace regioncode="410184" if regioncode=="410184" & year==2011

replace regioncode="410185" if regioncode=="410185" & year==2011

replace regioncode="410202" if regioncode=="410202" & year==2011

replace regioncode="410203" if regioncode=="410203" & year==2011

replace regioncode="410204" if regioncode=="410204" & year==2011

replace regioncode="410205" if regioncode=="410205" & year==2011

replace regioncode="410211" if regioncode=="410211" & year==2011

replace regioncode="410221" if regioncode=="410221" & year==2011

replace regioncode="410222" if regioncode=="410222" & year==2011

replace regioncode="410223" if regioncode=="410223" & year==2011

replace regioncode="410224" if regioncode=="410224" & year==2011

replace regioncode="410225" if regioncode=="410225" & year==2011

replace regioncode="410302" if regioncode=="410302" & year==2011

replace regioncode="410303" if regioncode=="410303" & year==2011

replace regioncode="410304" if regioncode=="410304" & year==2011

replace regioncode="410305" if regioncode=="410305" & year==2011

replace regioncode="410306" if regioncode=="410306" & year==2011

replace regioncode="410307" if regioncode=="410311" & year==2011

replace regioncode="410322" if regioncode=="410322" & year==2011

replace regioncode="410323" if regioncode=="410323" & year==2011

replace regioncode="410324" if regioncode=="410324" & year==2011

replace regioncode="410325" if regioncode=="410325" & year==2011

replace regioncode="410326" if regioncode=="410326" & year==2011

replace regioncode="410327" if regioncode=="410327" & year==2011

replace regioncode="410328" if regioncode=="410328" & year==2011

replace regioncode="410329" if regioncode=="410329" & year==2011

replace regioncode="410381" if regioncode=="410381" & year==2011

replace regioncode="410402" if regioncode=="410402" & year==2011

replace regioncode="410403" if regioncode=="410403" & year==2011

replace regioncode="410404" if regioncode=="410404" & year==2011

replace regioncode="410411" if regioncode=="410411" & year==2011

replace regioncode="410421" if regioncode=="410421" & year==2011

replace regioncode="410422" if regioncode=="410422" & year==2011

replace regioncode="410423" if regioncode=="410423" & year==2011

replace regioncode="410425" if regioncode=="410425" & year==2011

replace regioncode="410481" if regioncode=="410481" & year==2011

replace regioncode="410482" if regioncode=="410482" & year==2011

replace regioncode="410502" if regioncode=="410502" & year==2011

replace regioncode="410503" if regioncode=="410503" & year==2011

replace regioncode="410504" if regioncode=="410505" & year==2011

replace regioncode="410511" if regioncode=="410506" & year==2011

replace regioncode="410522" if regioncode=="410522" & year==2011

replace regioncode="410523" if regioncode=="410523" & year==2011

replace regioncode="410526" if regioncode=="410526" & year==2011

replace regioncode="410527" if regioncode=="410527" & year==2011

replace regioncode="410581" if regioncode=="410581" & year==2011

replace regioncode="410602" if regioncode=="410602" & year==2011

replace regioncode="410603" if regioncode=="410603" & year==2011

replace regioncode="410611" if regioncode=="410611" & year==2011

replace regioncode="410621" if regioncode=="410621" & year==2011

replace regioncode="410622" if regioncode=="410622" & year==2011

replace regioncode="410702" if regioncode=="410702" & year==2011

replace regioncode="410703" if regioncode=="410703" & year==2011

replace regioncode="410704" if regioncode=="410704" & year==2011

replace regioncode="410711" if regioncode=="410711" & year==2011

replace regioncode="410721" if regioncode=="410721" & year==2011

replace regioncode="410724" if regioncode=="410724" & year==2011

replace regioncode="410725" if regioncode=="410725" & year==2011

replace regioncode="410726" if regioncode=="410726" & year==2011

replace regioncode="410727" if regioncode=="410727" & year==2011

replace regioncode="410728" if regioncode=="410728" & year==2011

replace regioncode="410781" if regioncode=="410781" & year==2011

replace regioncode="410782" if regioncode=="410782" & year==2011

replace regioncode="410802" if regioncode=="410802" & year==2011

replace regioncode="410803" if regioncode=="410803" & year==2011

replace regioncode="410804" if regioncode=="410804" & year==2011

replace regioncode="410811" if regioncode=="410811" & year==2011

replace regioncode="410821" if regioncode=="410821" & year==2011

replace regioncode="410822" if regioncode=="410822" & year==2011

replace regioncode="410823" if regioncode=="410823" & year==2011

replace regioncode="410825" if regioncode=="410825" & year==2011

replace regioncode="410881" if regioncode=="410881" & year==2011

replace regioncode="410882" if regioncode=="410882" & year==2011

replace regioncode="410883" if regioncode=="410883" & year==2011

replace regioncode="410902" if regioncode=="410902" & year==2011

replace regioncode="410922" if regioncode=="410922" & year==2011

replace regioncode="410923" if regioncode=="410923" & year==2011

replace regioncode="410926" if regioncode=="410926" & year==2011

replace regioncode="410927" if regioncode=="410927" & year==2011

replace regioncode="410928" if regioncode=="410928" & year==2011

replace regioncode="411002" if regioncode=="411002" & year==2011

replace regioncode="411023" if regioncode=="411023" & year==2011

replace regioncode="411024" if regioncode=="411024" & year==2011

replace regioncode="411025" if regioncode=="411025" & year==2011

replace regioncode="411081" if regioncode=="411081" & year==2011

replace regioncode="411082" if regioncode=="411082" & year==2011

replace regioncode="411102" if regioncode=="411102" & year==2011

replace regioncode="411123" if regioncode=="411103" & year==2011

replace regioncode="411121" if regioncode=="411121" & year==2011

replace regioncode="411122" if regioncode=="411122" & year==2011

replace regioncode="411202" if regioncode=="411202" & year==2011

replace regioncode="411221" if regioncode=="411221" & year==2011

replace regioncode="411222" if regioncode=="411222" & year==2011

replace regioncode="411224" if regioncode=="411224" & year==2011

replace regioncode="411281" if regioncode=="411281" & year==2011

replace regioncode="411282" if regioncode=="411282" & year==2011

replace regioncode="411302" if regioncode=="411302" & year==2011

replace regioncode="411303" if regioncode=="411303" & year==2011

replace regioncode="411321" if regioncode=="411321" & year==2011

replace regioncode="411322" if regioncode=="411322" & year==2011

replace regioncode="411323" if regioncode=="411323" & year==2011

replace regioncode="411324" if regioncode=="411324" & year==2011

replace regioncode="411325" if regioncode=="411325" & year==2011

replace regioncode="411326" if regioncode=="411326" & year==2011

replace regioncode="411327" if regioncode=="411327" & year==2011

replace regioncode="411328" if regioncode=="411328" & year==2011

replace regioncode="411329" if regioncode=="411329" & year==2011

replace regioncode="411330" if regioncode=="411330" & year==2011

replace regioncode="411381" if regioncode=="411381" & year==2011

replace regioncode="411402" if regioncode=="411402" & year==2011

replace regioncode="411403" if regioncode=="411403" & year==2011

replace regioncode="411421" if regioncode=="411421" & year==2011

replace regioncode="411422" if regioncode=="411422" & year==2011

replace regioncode="411423" if regioncode=="411423" & year==2011

replace regioncode="411424" if regioncode=="411424" & year==2011

replace regioncode="411425" if regioncode=="411425" & year==2011

replace regioncode="411426" if regioncode=="411426" & year==2011

replace regioncode="411481" if regioncode=="411481" & year==2011

replace regioncode="411502" if regioncode=="411502" & year==2011

replace regioncode="411503" if regioncode=="411503" & year==2011

replace regioncode="411521" if regioncode=="411521" & year==2011

replace regioncode="411522" if regioncode=="411522" & year==2011

replace regioncode="411523" if regioncode=="411523" & year==2011

replace regioncode="411524" if regioncode=="411524" & year==2011

replace regioncode="411525" if regioncode=="411525" & year==2011

replace regioncode="411526" if regioncode=="411526" & year==2011

replace regioncode="411527" if regioncode=="411527" & year==2011

replace regioncode="411528" if regioncode=="411528" & year==2011

replace regioncode="411602" if regioncode=="411602" & year==2011

replace regioncode="411621" if regioncode=="411621" & year==2011

replace regioncode="411622" if regioncode=="411622" & year==2011

replace regioncode="411623" if regioncode=="411623" & year==2011

replace regioncode="411624" if regioncode=="411624" & year==2011

replace regioncode="411625" if regioncode=="411625" & year==2011

replace regioncode="411626" if regioncode=="411626" & year==2011

replace regioncode="411627" if regioncode=="411627" & year==2011

replace regioncode="411628" if regioncode=="411628" & year==2011

replace regioncode="411681" if regioncode=="411681" & year==2011

replace regioncode="411702" if regioncode=="411702" & year==2011

replace regioncode="411721" if regioncode=="411721" & year==2011

replace regioncode="411722" if regioncode=="411722" & year==2011

replace regioncode="411723" if regioncode=="411723" & year==2011

replace regioncode="411724" if regioncode=="411724" & year==2011

replace regioncode="411725" if regioncode=="411725" & year==2011

replace regioncode="411726" if regioncode=="411726" & year==2011

replace regioncode="411727" if regioncode=="411727" & year==2011

replace regioncode="411728" if regioncode=="411728" & year==2011

replace regioncode="411729" if regioncode=="411729" & year==2011

replace regioncode="420102" if regioncode=="420102" & year==2011

replace regioncode="420103" if regioncode=="420103" & year==2011

replace regioncode="420104" if regioncode=="420104" & year==2011

replace regioncode="420105" if regioncode=="420105" & year==2011

replace regioncode="420106" if regioncode=="420106" & year==2011

replace regioncode="420107" if regioncode=="420107" & year==2011

replace regioncode="420111" if regioncode=="420111" & year==2011

replace regioncode="420112" if regioncode=="420112" & year==2011

replace regioncode="420113" if regioncode=="420113" & year==2011

replace regioncode="420114" if regioncode=="420114" & year==2011

replace regioncode="420115" if regioncode=="420115" & year==2011

replace regioncode="420116" if regioncode=="420116" & year==2011

replace regioncode="420117" if regioncode=="420117" & year==2011

replace regioncode="420202" if regioncode=="420202" & year==2011

replace regioncode="420203" if regioncode=="420203" & year==2011

replace regioncode="420204" if regioncode=="420204" & year==2011

replace regioncode="420205" if regioncode=="420205" & year==2011

replace regioncode="420222" if regioncode=="420222" & year==2011

replace regioncode="420281" if regioncode=="420281" & year==2011

replace regioncode="420302" if regioncode=="420302" & year==2011

replace regioncode="420303" if regioncode=="420303" & year==2011

replace regioncode="420321" if regioncode=="420321" & year==2011

replace regioncode="420322" if regioncode=="420322" & year==2011

replace regioncode="420323" if regioncode=="420323" & year==2011

replace regioncode="420324" if regioncode=="420324" & year==2011

replace regioncode="420325" if regioncode=="420325" & year==2011

replace regioncode="420381" if regioncode=="420381" & year==2011

replace regioncode="420502" if regioncode=="420502" & year==2011

replace regioncode="420503" if regioncode=="420503" & year==2011

replace regioncode="420504" if regioncode=="420504" & year==2011

replace regioncode="420505" if regioncode=="420505" & year==2011

replace regioncode="420506" if regioncode=="420506" & year==2011

replace regioncode="420525" if regioncode=="420525" & year==2011

replace regioncode="420526" if regioncode=="420526" & year==2011

replace regioncode="420527" if regioncode=="420527" & year==2011

replace regioncode="420528" if regioncode=="420528" & year==2011

replace regioncode="420529" if regioncode=="420529" & year==2011

replace regioncode="420581" if regioncode=="420581" & year==2011

replace regioncode="420582" if regioncode=="420582" & year==2011

replace regioncode="420583" if regioncode=="420583" & year==2011

replace regioncode="420602" if regioncode=="420602" & year==2011

replace regioncode="420606" if regioncode=="420606" & year==2011

replace regioncode="420607" if regioncode=="420607" & year==2011

replace regioncode="420624" if regioncode=="420624" & year==2011

replace regioncode="420625" if regioncode=="420625" & year==2011

replace regioncode="420626" if regioncode=="420626" & year==2011

replace regioncode="420682" if regioncode=="420682" & year==2011

replace regioncode="420683" if regioncode=="420683" & year==2011

replace regioncode="420684" if regioncode=="420684" & year==2011

replace regioncode="420702" if regioncode=="420702" & year==2011

replace regioncode="420703" if regioncode=="420703" & year==2011

replace regioncode="420704" if regioncode=="420704" & year==2011

replace regioncode="420802" if regioncode=="420802" & year==2011

replace regioncode="420804" if regioncode=="420804" & year==2011

replace regioncode="420821" if regioncode=="420821" & year==2011

replace regioncode="420822" if regioncode=="420822" & year==2011

replace regioncode="420881" if regioncode=="420881" & year==2011

replace regioncode="420902" if regioncode=="420902" & year==2011

replace regioncode="420921" if regioncode=="420921" & year==2011

replace regioncode="420922" if regioncode=="420922" & year==2011

replace regioncode="420923" if regioncode=="420923" & year==2011

replace regioncode="420981" if regioncode=="420981" & year==2011

replace regioncode="420982" if regioncode=="420982" & year==2011

replace regioncode="421381" if regioncode=="421381" & year==2011

replace regioncode="420984" if regioncode=="420984" & year==2011

replace regioncode="421002" if regioncode=="421002" & year==2011

replace regioncode="421003" if regioncode=="421003" & year==2011

replace regioncode="421022" if regioncode=="421022" & year==2011

replace regioncode="421023" if regioncode=="421023" & year==2011

replace regioncode="421024" if regioncode=="421024" & year==2011

replace regioncode="421081" if regioncode=="421081" & year==2011

replace regioncode="421083" if regioncode=="421083" & year==2011

replace regioncode="421087" if regioncode=="421087" & year==2011

replace regioncode="421102" if regioncode=="421102" & year==2011

replace regioncode="421121" if regioncode=="421121" & year==2011

replace regioncode="421122" if regioncode=="421122" & year==2011

replace regioncode="421123" if regioncode=="421123" & year==2011

replace regioncode="421124" if regioncode=="421124" & year==2011

replace regioncode="421125" if regioncode=="421125" & year==2011

replace regioncode="421126" if regioncode=="421126" & year==2011

replace regioncode="421127" if regioncode=="421127" & year==2011

replace regioncode="421181" if regioncode=="421181" & year==2011

replace regioncode="421182" if regioncode=="421182" & year==2011

replace regioncode="421202" if regioncode=="421202" & year==2011

replace regioncode="421221" if regioncode=="421221" & year==2011

replace regioncode="421222" if regioncode=="421222" & year==2011

replace regioncode="421223" if regioncode=="421223" & year==2011

replace regioncode="421224" if regioncode=="421224" & year==2011

replace regioncode="421281" if regioncode=="421281" & year==2011

replace regioncode="421302" if regioncode=="421302" & year==2011

replace regioncode="422801" if regioncode=="422801" & year==2011

replace regioncode="422802" if regioncode=="422802" & year==2011

replace regioncode="422822" if regioncode=="422822" & year==2011

replace regioncode="422823" if regioncode=="422823" & year==2011

replace regioncode="422825" if regioncode=="422825" & year==2011

replace regioncode="422826" if regioncode=="422826" & year==2011

replace regioncode="422827" if regioncode=="422827" & year==2011

replace regioncode="422828" if regioncode=="422828" & year==2011

replace regioncode="429004" if regioncode=="429004" & year==2011

replace regioncode="429005" if regioncode=="429005" & year==2011

replace regioncode="429006" if regioncode=="429006" & year==2011

replace regioncode="429021" if regioncode=="429021" & year==2011

replace regioncode="430102" if regioncode=="430102" & year==2011

replace regioncode="430103" if regioncode=="430103" & year==2011

replace regioncode="430104" if regioncode=="430104" & year==2011

replace regioncode="430105" if regioncode=="430105" & year==2011

replace regioncode="430111" if regioncode=="430111" & year==2011

replace regioncode="430121" if regioncode=="430121" & year==2011

replace regioncode="430122" if regioncode=="430122" & year==2011

replace regioncode="430124" if regioncode=="430124" & year==2011

replace regioncode="430181" if regioncode=="430181" & year==2011

replace regioncode="430202" if regioncode=="430202" & year==2011

replace regioncode="430203" if regioncode=="430203" & year==2011

replace regioncode="430204" if regioncode=="430204" & year==2011

replace regioncode="430211" if regioncode=="430211" & year==2011

replace regioncode="430221" if regioncode=="430221" & year==2011

replace regioncode="430223" if regioncode=="430223" & year==2011

replace regioncode="430224" if regioncode=="430224" & year==2011

replace regioncode="430225" if regioncode=="430225" & year==2011

replace regioncode="430281" if regioncode=="430281" & year==2011

replace regioncode="430302" if regioncode=="430302" & year==2011

replace regioncode="430304" if regioncode=="430304" & year==2011

replace regioncode="430321" if regioncode=="430321" & year==2011

replace regioncode="430381" if regioncode=="430381" & year==2011

replace regioncode="430382" if regioncode=="430382" & year==2011

replace regioncode="430405" if regioncode=="430405" & year==2011

replace regioncode="430406" if regioncode=="430406" & year==2011

replace regioncode="430407" if regioncode=="430407" & year==2011

replace regioncode="430408" if regioncode=="430408" & year==2011

replace regioncode="430412" if regioncode=="430412" & year==2011

replace regioncode="430421" if regioncode=="430421" & year==2011

replace regioncode="430422" if regioncode=="430422" & year==2011

replace regioncode="430423" if regioncode=="430423" & year==2011

replace regioncode="430424" if regioncode=="430424" & year==2011

replace regioncode="430426" if regioncode=="430426" & year==2011

replace regioncode="430481" if regioncode=="430481" & year==2011

replace regioncode="430482" if regioncode=="430482" & year==2011

replace regioncode="430502" if regioncode=="430502" & year==2011

replace regioncode="430503" if regioncode=="430503" & year==2011

replace regioncode="430511" if regioncode=="430511" & year==2011

replace regioncode="430521" if regioncode=="430521" & year==2011

replace regioncode="430522" if regioncode=="430522" & year==2011

replace regioncode="430523" if regioncode=="430523" & year==2011

replace regioncode="430524" if regioncode=="430524" & year==2011

replace regioncode="430525" if regioncode=="430525" & year==2011

replace regioncode="430527" if regioncode=="430527" & year==2011

replace regioncode="430528" if regioncode=="430528" & year==2011

replace regioncode="430529" if regioncode=="430529" & year==2011

replace regioncode="430581" if regioncode=="430581" & year==2011

replace regioncode="430602" if regioncode=="430602" & year==2011

replace regioncode="430603" if regioncode=="430603" & year==2011

replace regioncode="430611" if regioncode=="430611" & year==2011

replace regioncode="430621" if regioncode=="430621" & year==2011

replace regioncode="430623" if regioncode=="430623" & year==2011

replace regioncode="430624" if regioncode=="430624" & year==2011

replace regioncode="430626" if regioncode=="430626" & year==2011

replace regioncode="430681" if regioncode=="430681" & year==2011

replace regioncode="430682" if regioncode=="430682" & year==2011

replace regioncode="430702" if regioncode=="430702" & year==2011

replace regioncode="430703" if regioncode=="430703" & year==2011

replace regioncode="430721" if regioncode=="430721" & year==2011

replace regioncode="430722" if regioncode=="430722" & year==2011

replace regioncode="430723" if regioncode=="430723" & year==2011

replace regioncode="430724" if regioncode=="430724" & year==2011

replace regioncode="430725" if regioncode=="430725" & year==2011

replace regioncode="430726" if regioncode=="430726" & year==2011

replace regioncode="430781" if regioncode=="430781" & year==2011

replace regioncode="430802" if regioncode=="430802" & year==2011

replace regioncode="430811" if regioncode=="430811" & year==2011

replace regioncode="430821" if regioncode=="430821" & year==2011

replace regioncode="430822" if regioncode=="430822" & year==2011

replace regioncode="430902" if regioncode=="430902" & year==2011

replace regioncode="430903" if regioncode=="430903" & year==2011

replace regioncode="430921" if regioncode=="430921" & year==2011

replace regioncode="430922" if regioncode=="430922" & year==2011

replace regioncode="430923" if regioncode=="430923" & year==2011

replace regioncode="430981" if regioncode=="430981" & year==2011

replace regioncode="431002" if regioncode=="431002" & year==2011

replace regioncode="431003" if regioncode=="431003" & year==2011

replace regioncode="431021" if regioncode=="431021" & year==2011

replace regioncode="431022" if regioncode=="431022" & year==2011

replace regioncode="431023" if regioncode=="431023" & year==2011

replace regioncode="431024" if regioncode=="431024" & year==2011

replace regioncode="431025" if regioncode=="431025" & year==2011

replace regioncode="431026" if regioncode=="431026" & year==2011

replace regioncode="431027" if regioncode=="431027" & year==2011

replace regioncode="431028" if regioncode=="431028" & year==2011

replace regioncode="431081" if regioncode=="431081" & year==2011

replace regioncode="431102" if regioncode=="431102" & year==2011

replace regioncode="431103" if regioncode=="431103" & year==2011

replace regioncode="431121" if regioncode=="431121" & year==2011

replace regioncode="431122" if regioncode=="431122" & year==2011

replace regioncode="431123" if regioncode=="431123" & year==2011

replace regioncode="431124" if regioncode=="431124" & year==2011

replace regioncode="431125" if regioncode=="431125" & year==2011

replace regioncode="431126" if regioncode=="431126" & year==2011

replace regioncode="431127" if regioncode=="431127" & year==2011

replace regioncode="431128" if regioncode=="431128" & year==2011

replace regioncode="431129" if regioncode=="431129" & year==2011

replace regioncode="431202" if regioncode=="431202" & year==2011

replace regioncode="431221" if regioncode=="431221" & year==2011

replace regioncode="431222" if regioncode=="431222" & year==2011

replace regioncode="431223" if regioncode=="431223" & year==2011

replace regioncode="431224" if regioncode=="431224" & year==2011

replace regioncode="431225" if regioncode=="431225" & year==2011

replace regioncode="431226" if regioncode=="431226" & year==2011

replace regioncode="431227" if regioncode=="431227" & year==2011

replace regioncode="431228" if regioncode=="431228" & year==2011

replace regioncode="431229" if regioncode=="431229" & year==2011

replace regioncode="431230" if regioncode=="431230" & year==2011

replace regioncode="431281" if regioncode=="431281" & year==2011

replace regioncode="431302" if regioncode=="431302" & year==2011

replace regioncode="431321" if regioncode=="431321" & year==2011

replace regioncode="431322" if regioncode=="431322" & year==2011

replace regioncode="431381" if regioncode=="431381" & year==2011

replace regioncode="431382" if regioncode=="431382" & year==2011

replace regioncode="433101" if regioncode=="433101" & year==2011

replace regioncode="433122" if regioncode=="433122" & year==2011

replace regioncode="433123" if regioncode=="433123" & year==2011

replace regioncode="433124" if regioncode=="433124" & year==2011

replace regioncode="433125" if regioncode=="433125" & year==2011

replace regioncode="433126" if regioncode=="433126" & year==2011

replace regioncode="433127" if regioncode=="433127" & year==2011

replace regioncode="433130" if regioncode=="433130" & year==2011

replace regioncode="440102" if regioncode=="440115" & year==2011

replace regioncode="440103" if regioncode=="440103" & year==2011

replace regioncode="440104" if regioncode=="440104" & year==2011

replace regioncode="440105" if regioncode=="440105" & year==2011

replace regioncode="440106" if regioncode=="440106" & year==2011

replace regioncode="440107" if regioncode=="440116" & year==2011

replace regioncode="440111" if regioncode=="440111" & year==2011

replace regioncode="440112" if regioncode=="440112" & year==2011

replace regioncode="440113" if regioncode=="440113" & year==2011

replace regioncode="440114" if regioncode=="440114" & year==2011

replace regioncode="440183" if regioncode=="440183" & year==2011

replace regioncode="440184" if regioncode=="440184" & year==2011

replace regioncode="440203" if regioncode=="440203" & year==2011

replace regioncode="440204" if regioncode=="440204" & year==2011

replace regioncode="440221" if regioncode=="440205" & year==2011

replace regioncode="440222" if regioncode=="440222" & year==2011

replace regioncode="440224" if regioncode=="440224" & year==2011

replace regioncode="440229" if regioncode=="440229" & year==2011

replace regioncode="440232" if regioncode=="440232" & year==2011

replace regioncode="440233" if regioncode=="440233" & year==2011

replace regioncode="440281" if regioncode=="440281" & year==2011

replace regioncode="440282" if regioncode=="440282" & year==2011

replace regioncode="440303" if regioncode=="440303" & year==2011

replace regioncode="440304" if regioncode=="440304" & year==2011

replace regioncode="440305" if regioncode=="440305" & year==2011

replace regioncode="440306" if regioncode=="440306" & year==2011

replace regioncode="440307" if regioncode=="440307" & year==2011

replace regioncode="440308" if regioncode=="440308" & year==2011

replace regioncode="440402" if regioncode=="440402" & year==2011

replace regioncode="440403" if regioncode=="440403" & year==2011

replace regioncode="440404" if regioncode=="440404" & year==2011

replace regioncode="440507" if regioncode=="440507" & year==2011

replace regioncode="440511" if regioncode=="440511" & year==2011

replace regioncode="440512" if regioncode=="440512" & year==2011

replace regioncode="440514" if regioncode=="440514" & year==2011

replace regioncode="440515" if regioncode=="440515" & year==2011

replace regioncode="440523" if regioncode=="440523" & year==2011

replace regioncode="440604" if regioncode=="440604" & year==2011

replace regioncode="440605" if regioncode=="440605" & year==2011

replace regioncode="440606" if regioncode=="440606" & year==2011

replace regioncode="440607" if regioncode=="440607" & year==2011

replace regioncode="440608" if regioncode=="440608" & year==2011

replace regioncode="440703" if regioncode=="440703" & year==2011

replace regioncode="440704" if regioncode=="440704" & year==2011

replace regioncode="440781" if regioncode=="440781" & year==2011

replace regioncode="440705" if regioncode=="440705" & year==2011

replace regioncode="440783" if regioncode=="440783" & year==2011

replace regioncode="440784" if regioncode=="440784" & year==2011

replace regioncode="440785" if regioncode=="440785" & year==2011

replace regioncode="440802" if regioncode=="440802" & year==2011

replace regioncode="440803" if regioncode=="440803" & year==2011

replace regioncode="440804" if regioncode=="440804" & year==2011

replace regioncode="440811" if regioncode=="440811" & year==2011

replace regioncode="440823" if regioncode=="440823" & year==2011

replace regioncode="440825" if regioncode=="440825" & year==2011

replace regioncode="440881" if regioncode=="440881" & year==2011

replace regioncode="440882" if regioncode=="440882" & year==2011

replace regioncode="440883" if regioncode=="440883" & year==2011

replace regioncode="440902" if regioncode=="440902" & year==2011

replace regioncode="440903" if regioncode=="440903" & year==2011

replace regioncode="440923" if regioncode=="440923" & year==2011

replace regioncode="440981" if regioncode=="440981" & year==2011

replace regioncode="440982" if regioncode=="440982" & year==2011

replace regioncode="440983" if regioncode=="440983" & year==2011

replace regioncode="441202" if regioncode=="441202" & year==2011

replace regioncode="441203" if regioncode=="441203" & year==2011

replace regioncode="441223" if regioncode=="441223" & year==2011

replace regioncode="441224" if regioncode=="441224" & year==2011

replace regioncode="441225" if regioncode=="441225" & year==2011

replace regioncode="441226" if regioncode=="441226" & year==2011

replace regioncode="441283" if regioncode=="441283" & year==2011

replace regioncode="441284" if regioncode=="441284" & year==2011

replace regioncode="441302" if regioncode=="441302" & year==2011

replace regioncode="441303" if regioncode=="441303" & year==2011

replace regioncode="441322" if regioncode=="441322" & year==2011

replace regioncode="441323" if regioncode=="441323" & year==2011

replace regioncode="441324" if regioncode=="441324" & year==2011

replace regioncode="441402" if regioncode=="441402" & year==2011

replace regioncode="441421" if regioncode=="441421" & year==2011

replace regioncode="441422" if regioncode=="441422" & year==2011

replace regioncode="441423" if regioncode=="441423" & year==2011

replace regioncode="441424" if regioncode=="441424" & year==2011

replace regioncode="441426" if regioncode=="441426" & year==2011

replace regioncode="441427" if regioncode=="441427" & year==2011

replace regioncode="441481" if regioncode=="441481" & year==2011

replace regioncode="441502" if regioncode=="441502" & year==2011

replace regioncode="441521" if regioncode=="441521" & year==2011

replace regioncode="441523" if regioncode=="441523" & year==2011

replace regioncode="441581" if regioncode=="441581" & year==2011

replace regioncode="441602" if regioncode=="441602" & year==2011

replace regioncode="441621" if regioncode=="441621" & year==2011

replace regioncode="441622" if regioncode=="441622" & year==2011

replace regioncode="441623" if regioncode=="441623" & year==2011

replace regioncode="441624" if regioncode=="441624" & year==2011

replace regioncode="441625" if regioncode=="441625" & year==2011

replace regioncode="441702" if regioncode=="441702" & year==2011

replace regioncode="441721" if regioncode=="441721" & year==2011

replace regioncode="441723" if regioncode=="441723" & year==2011

replace regioncode="441781" if regioncode=="441781" & year==2011

replace regioncode="441802" if regioncode=="441802" & year==2011

replace regioncode="441821" if regioncode=="441821" & year==2011

replace regioncode="441823" if regioncode=="441823" & year==2011

replace regioncode="441825" if regioncode=="441825" & year==2011

replace regioncode="441826" if regioncode=="441826" & year==2011

replace regioncode="441827" if regioncode=="441827" & year==2011

replace regioncode="441881" if regioncode=="441881" & year==2011

replace regioncode="441882" if regioncode=="441882" & year==2011

replace regioncode="441900" if regioncode=="441901" & year==2011

replace regioncode="442000" if regioncode=="442001" & year==2011

replace regioncode="445102" if regioncode=="445102" & year==2011

replace regioncode="445121" if regioncode=="445121" & year==2011

replace regioncode="445122" if regioncode=="445122" & year==2011

replace regioncode="445202" if regioncode=="445202" & year==2011

replace regioncode="445221" if regioncode=="445221" & year==2011

replace regioncode="445222" if regioncode=="445222" & year==2011

replace regioncode="445224" if regioncode=="445224" & year==2011

replace regioncode="445281" if regioncode=="445281" & year==2011

replace regioncode="445302" if regioncode=="445302" & year==2011

replace regioncode="445321" if regioncode=="445321" & year==2011

replace regioncode="445322" if regioncode=="445322" & year==2011

replace regioncode="445323" if regioncode=="445323" & year==2011

replace regioncode="445381" if regioncode=="445381" & year==2011

replace regioncode="450102" if regioncode=="450102" & year==2011

replace regioncode="450103" if regioncode=="450103" & year==2011

replace regioncode="450104" if regioncode=="450107" & year==2011

replace regioncode="450105" if regioncode=="450105" & year==2011

replace regioncode="450106" if regioncode=="450108" & year==2011

replace regioncode="450121" if regioncode=="450109" & year==2011

replace regioncode="450122" if regioncode=="450122" & year==2011

replace regioncode="450123" if regioncode=="450123" & year==2011

replace regioncode="450124" if regioncode=="450124" & year==2011

replace regioncode="450125" if regioncode=="450125" & year==2011

replace regioncode="450126" if regioncode=="450126" & year==2011

replace regioncode="450127" if regioncode=="450127" & year==2011

replace regioncode="450202" if regioncode=="450202" & year==2011

replace regioncode="450203" if regioncode=="450203" & year==2011

replace regioncode="450204" if regioncode=="450204" & year==2011

replace regioncode="450205" if regioncode=="450205" & year==2011

replace regioncode="450221" if regioncode=="450221" & year==2011

replace regioncode="450222" if regioncode=="450222" & year==2011

replace regioncode="450223" if regioncode=="450223" & year==2011

replace regioncode="450224" if regioncode=="450224" & year==2011

replace regioncode="450225" if regioncode=="450225" & year==2011

replace regioncode="450226" if regioncode=="450226" & year==2011

replace regioncode="450302" if regioncode=="450302" & year==2011

replace regioncode="450303" if regioncode=="450303" & year==2011

replace regioncode="450304" if regioncode=="450304" & year==2011

replace regioncode="450305" if regioncode=="450305" & year==2011

replace regioncode="450311" if regioncode=="450311" & year==2011

replace regioncode="450321" if regioncode=="450321" & year==2011

replace regioncode="450322" if regioncode=="450322" & year==2011

replace regioncode="450323" if regioncode=="450323" & year==2011

replace regioncode="450324" if regioncode=="450324" & year==2011

replace regioncode="450325" if regioncode=="450325" & year==2011

replace regioncode="450326" if regioncode=="450326" & year==2011

replace regioncode="450327" if regioncode=="450327" & year==2011

replace regioncode="450328" if regioncode=="450328" & year==2011

replace regioncode="450329" if regioncode=="450329" & year==2011

replace regioncode="450330" if regioncode=="450330" & year==2011

replace regioncode="450331" if regioncode=="450331" & year==2011

replace regioncode="450332" if regioncode=="450332" & year==2011

replace regioncode="450403" if regioncode=="450403" & year==2011

replace regioncode="450404" if regioncode=="450404" & year==2011

replace regioncode="450421" if regioncode=="450421" & year==2011

replace regioncode="450422" if regioncode=="450422" & year==2011

replace regioncode="450423" if regioncode=="450423" & year==2011

replace regioncode="450481" if regioncode=="450481" & year==2011

replace regioncode="450502" if regioncode=="450502" & year==2011

replace regioncode="450503" if regioncode=="450503" & year==2011

replace regioncode="450512" if regioncode=="450512" & year==2011

replace regioncode="450521" if regioncode=="450521" & year==2011

replace regioncode="450602" if regioncode=="450602" & year==2011

replace regioncode="450603" if regioncode=="450603" & year==2011

replace regioncode="450621" if regioncode=="450621" & year==2011

replace regioncode="450681" if regioncode=="450681" & year==2011

replace regioncode="450702" if regioncode=="450702" & year==2011

replace regioncode="450703" if regioncode=="450703" & year==2011

replace regioncode="450721" if regioncode=="450721" & year==2011

replace regioncode="450722" if regioncode=="450722" & year==2011

replace regioncode="450802" if regioncode=="450802" & year==2011

replace regioncode="450803" if regioncode=="450803" & year==2011

replace regioncode="450804" if regioncode=="450804" & year==2011

replace regioncode="450821" if regioncode=="450821" & year==2011

replace regioncode="450881" if regioncode=="450881" & year==2011

replace regioncode="450902" if regioncode=="450902" & year==2011

replace regioncode="450921" if regioncode=="450921" & year==2011

replace regioncode="450922" if regioncode=="450922" & year==2011

replace regioncode="450923" if regioncode=="450923" & year==2011

replace regioncode="450924" if regioncode=="450924" & year==2011

replace regioncode="450981" if regioncode=="450981" & year==2011

replace regioncode="451002" if regioncode=="451002" & year==2011

replace regioncode="451021" if regioncode=="451021" & year==2011

replace regioncode="451022" if regioncode=="451022" & year==2011

replace regioncode="451023" if regioncode=="451023" & year==2011

replace regioncode="451024" if regioncode=="451024" & year==2011

replace regioncode="451025" if regioncode=="451025" & year==2011

replace regioncode="451026" if regioncode=="451026" & year==2011

replace regioncode="451027" if regioncode=="451027" & year==2011

replace regioncode="451028" if regioncode=="451028" & year==2011

replace regioncode="451029" if regioncode=="451029" & year==2011

replace regioncode="451030" if regioncode=="451030" & year==2011

replace regioncode="451031" if regioncode=="451031" & year==2011

replace regioncode="451102" if regioncode=="451102" & year==2011

replace regioncode="451121" if regioncode=="451121" & year==2011

replace regioncode="451122" if regioncode=="451122" & year==2011

replace regioncode="451123" if regioncode=="451123" & year==2011

replace regioncode="451202" if regioncode=="451202" & year==2011

replace regioncode="451221" if regioncode=="451221" & year==2011

replace regioncode="451222" if regioncode=="451222" & year==2011

replace regioncode="451223" if regioncode=="451223" & year==2011

replace regioncode="451224" if regioncode=="451224" & year==2011

replace regioncode="451225" if regioncode=="451225" & year==2011

replace regioncode="451226" if regioncode=="451226" & year==2011

replace regioncode="451227" if regioncode=="451227" & year==2011

replace regioncode="451228" if regioncode=="451228" & year==2011

replace regioncode="451229" if regioncode=="451229" & year==2011

replace regioncode="451281" if regioncode=="451281" & year==2011

replace regioncode="451302" if regioncode=="451302" & year==2011

replace regioncode="451321" if regioncode=="451321" & year==2011

replace regioncode="451322" if regioncode=="451322" & year==2011

replace regioncode="451323" if regioncode=="451323" & year==2011

replace regioncode="451324" if regioncode=="451324" & year==2011

replace regioncode="451381" if regioncode=="451381" & year==2011

replace regioncode="451402" if regioncode=="451402" & year==2011

replace regioncode="451421" if regioncode=="451421" & year==2011

replace regioncode="451422" if regioncode=="451422" & year==2011

replace regioncode="451423" if regioncode=="451423" & year==2011

replace regioncode="451424" if regioncode=="451424" & year==2011

replace regioncode="451425" if regioncode=="451425" & year==2011

replace regioncode="451481" if regioncode=="451481" & year==2011

replace regioncode="460105" if regioncode=="460105" & year==2011

replace regioncode="460106" if regioncode=="460106" & year==2011

replace regioncode="460107" if regioncode=="460107" & year==2011

replace regioncode="460108" if regioncode=="460108" & year==2011

replace regioncode="460001" if regioncode=="469001" & year==2011

replace regioncode="460002" if regioncode=="469002" & year==2011

replace regioncode="460003" if regioncode=="469003" & year==2011

replace regioncode="460005" if regioncode=="469005" & year==2011

replace regioncode="460006" if regioncode=="469006" & year==2011

replace regioncode="460007" if regioncode=="469007" & year==2011

replace regioncode="460030" if regioncode=="469025" & year==2011

replace regioncode="460031" if regioncode=="469026" & year==2011

replace regioncode="460033" if regioncode=="469027" & year==2011

replace regioncode="460034" if regioncode=="469028" & year==2011

replace regioncode="460036" if regioncode=="469030" & year==2011

replace regioncode="500101" if regioncode=="500101" & year==2011

replace regioncode="500102" if regioncode=="500102" & year==2011

replace regioncode="500103" if regioncode=="500103" & year==2011

replace regioncode="500104" if regioncode=="500104" & year==2011

replace regioncode="500105" if regioncode=="500105" & year==2011

replace regioncode="500106" if regioncode=="500106" & year==2011

replace regioncode="500107" if regioncode=="500107" & year==2011

replace regioncode="500108" if regioncode=="500108" & year==2011

replace regioncode="500109" if regioncode=="500109" & year==2011

replace regioncode="500110" if regioncode=="500110" & year==2011

replace regioncode="500111" if regioncode=="500111" & year==2011

replace regioncode="500112" if regioncode=="500112" & year==2011

replace regioncode="500113" if regioncode=="500113" & year==2011

replace regioncode="500114" if regioncode=="500114" & year==2011

replace regioncode="500115" if regioncode=="500115" & year==2011

replace regioncode="500381" if regioncode=="500116" & year==2011

replace regioncode="500382" if regioncode=="500117" & year==2011

replace regioncode="500383" if regioncode=="500118" & year==2011

replace regioncode="500384" if regioncode=="500119" & year==2011

replace regioncode="500222" if regioncode=="500222" & year==2011

replace regioncode="500223" if regioncode=="500223" & year==2011

replace regioncode="500224" if regioncode=="500224" & year==2011

replace regioncode="500225" if regioncode=="500225" & year==2011

replace regioncode="500226" if regioncode=="500226" & year==2011

replace regioncode="500227" if regioncode=="500227" & year==2011

replace regioncode="500228" if regioncode=="500228" & year==2011

replace regioncode="500229" if regioncode=="500229" & year==2011

replace regioncode="500230" if regioncode=="500230" & year==2011

replace regioncode="500231" if regioncode=="500231" & year==2011

replace regioncode="500232" if regioncode=="500232" & year==2011

replace regioncode="500233" if regioncode=="500233" & year==2011

replace regioncode="500234" if regioncode=="500234" & year==2011

replace regioncode="500235" if regioncode=="500235" & year==2011

replace regioncode="500236" if regioncode=="500236" & year==2011

replace regioncode="500237" if regioncode=="500237" & year==2011

replace regioncode="500238" if regioncode=="500238" & year==2011

replace regioncode="500240" if regioncode=="500240" & year==2011

replace regioncode="500241" if regioncode=="500241" & year==2011

replace regioncode="500242" if regioncode=="500242" & year==2011

replace regioncode="500243" if regioncode=="500243" & year==2011

replace regioncode="510104" if regioncode=="510104" & year==2011

replace regioncode="510105" if regioncode=="510105" & year==2011

replace regioncode="510106" if regioncode=="510106" & year==2011

replace regioncode="510107" if regioncode=="510107" & year==2011

replace regioncode="510108" if regioncode=="510108" & year==2011

replace regioncode="510112" if regioncode=="510112" & year==2011

replace regioncode="510113" if regioncode=="510113" & year==2011

replace regioncode="510114" if regioncode=="510114" & year==2011

replace regioncode="510115" if regioncode=="510115" & year==2011

replace regioncode="510121" if regioncode=="510121" & year==2011

replace regioncode="510122" if regioncode=="510122" & year==2011

replace regioncode="510124" if regioncode=="510124" & year==2011

replace regioncode="510129" if regioncode=="510129" & year==2011

replace regioncode="510131" if regioncode=="510131" & year==2011

replace regioncode="510132" if regioncode=="510132" & year==2011

replace regioncode="510181" if regioncode=="510181" & year==2011

replace regioncode="510182" if regioncode=="510182" & year==2011

replace regioncode="510183" if regioncode=="510183" & year==2011

replace regioncode="510184" if regioncode=="510184" & year==2011

replace regioncode="510302" if regioncode=="510302" & year==2011

replace regioncode="510303" if regioncode=="510303" & year==2011

replace regioncode="510304" if regioncode=="510304" & year==2011

replace regioncode="510311" if regioncode=="510311" & year==2011

replace regioncode="510321" if regioncode=="510321" & year==2011

replace regioncode="510322" if regioncode=="510322" & year==2011

replace regioncode="510402" if regioncode=="510402" & year==2011

replace regioncode="510403" if regioncode=="510403" & year==2011

replace regioncode="510411" if regioncode=="510411" & year==2011

replace regioncode="510421" if regioncode=="510421" & year==2011

replace regioncode="510422" if regioncode=="510422" & year==2011

replace regioncode="510502" if regioncode=="510502" & year==2011

replace regioncode="510503" if regioncode=="510503" & year==2011

replace regioncode="510504" if regioncode=="510504" & year==2011

replace regioncode="510521" if regioncode=="510521" & year==2011

replace regioncode="510522" if regioncode=="510522" & year==2011

replace regioncode="510524" if regioncode=="510524" & year==2011

replace regioncode="510525" if regioncode=="510525" & year==2011

replace regioncode="510603" if regioncode=="510603" & year==2011

replace regioncode="510623" if regioncode=="510623" & year==2011

replace regioncode="510626" if regioncode=="510626" & year==2011

replace regioncode="510681" if regioncode=="510681" & year==2011

replace regioncode="510682" if regioncode=="510682" & year==2011

replace regioncode="510683" if regioncode=="510683" & year==2011

replace regioncode="510703" if regioncode=="510703" & year==2011

replace regioncode="510704" if regioncode=="510704" & year==2011

replace regioncode="510722" if regioncode=="510722" & year==2011

replace regioncode="510723" if regioncode=="510723" & year==2011

replace regioncode="510724" if regioncode=="510724" & year==2011

replace regioncode="510725" if regioncode=="510725" & year==2011

replace regioncode="510726" if regioncode=="510726" & year==2011

replace regioncode="510727" if regioncode=="510727" & year==2011

replace regioncode="510781" if regioncode=="510781" & year==2011

replace regioncode="510802" if regioncode=="510802" & year==2011

replace regioncode="510811" if regioncode=="510811" & year==2011

replace regioncode="510812" if regioncode=="510812" & year==2011

replace regioncode="510821" if regioncode=="510821" & year==2011

replace regioncode="510822" if regioncode=="510822" & year==2011

replace regioncode="510823" if regioncode=="510823" & year==2011

replace regioncode="510824" if regioncode=="510824" & year==2011

replace regioncode="510902" if regioncode=="510903" & year==2011

replace regioncode="510921" if regioncode=="510921" & year==2011

replace regioncode="510922" if regioncode=="510922" & year==2011

replace regioncode="510923" if regioncode=="510923" & year==2011

replace regioncode="511002" if regioncode=="511002" & year==2011

replace regioncode="511011" if regioncode=="511011" & year==2011

replace regioncode="511024" if regioncode=="511024" & year==2011

replace regioncode="511025" if regioncode=="511025" & year==2011

replace regioncode="511028" if regioncode=="511028" & year==2011

replace regioncode="511102" if regioncode=="511102" & year==2011

replace regioncode="511111" if regioncode=="511111" & year==2011

replace regioncode="511112" if regioncode=="511112" & year==2011

replace regioncode="511113" if regioncode=="511113" & year==2011

replace regioncode="511123" if regioncode=="511123" & year==2011

replace regioncode="511124" if regioncode=="511124" & year==2011

replace regioncode="511126" if regioncode=="511126" & year==2011

replace regioncode="511129" if regioncode=="511129" & year==2011

replace regioncode="511132" if regioncode=="511132" & year==2011

replace regioncode="511133" if regioncode=="511133" & year==2011

replace regioncode="511181" if regioncode=="511181" & year==2011

replace regioncode="511302" if regioncode=="511302" & year==2011

replace regioncode="511303" if regioncode=="511303" & year==2011

replace regioncode="511304" if regioncode=="511304" & year==2011

replace regioncode="511321" if regioncode=="511321" & year==2011

replace regioncode="511322" if regioncode=="511322" & year==2011

replace regioncode="511323" if regioncode=="511323" & year==2011

replace regioncode="511324" if regioncode=="511324" & year==2011

replace regioncode="511325" if regioncode=="511325" & year==2011

replace regioncode="511381" if regioncode=="511381" & year==2011

replace regioncode="511402" if regioncode=="511402" & year==2011

replace regioncode="511421" if regioncode=="511421" & year==2011

replace regioncode="511422" if regioncode=="511422" & year==2011

replace regioncode="511423" if regioncode=="511423" & year==2011

replace regioncode="511424" if regioncode=="511424" & year==2011

replace regioncode="511425" if regioncode=="511425" & year==2011

replace regioncode="511502" if regioncode=="511502" & year==2011

replace regioncode="511521" if regioncode=="511521" & year==2011

replace regioncode="511522" if regioncode=="511522" & year==2011

replace regioncode="511523" if regioncode=="511523" & year==2011

replace regioncode="511524" if regioncode=="511524" & year==2011

replace regioncode="511525" if regioncode=="511525" & year==2011

replace regioncode="511526" if regioncode=="511526" & year==2011

replace regioncode="511527" if regioncode=="511527" & year==2011

replace regioncode="511528" if regioncode=="511528" & year==2011

replace regioncode="511529" if regioncode=="511529" & year==2011

replace regioncode="511602" if regioncode=="511602" & year==2011

replace regioncode="511621" if regioncode=="511621" & year==2011

replace regioncode="511622" if regioncode=="511622" & year==2011

replace regioncode="511623" if regioncode=="511623" & year==2011

replace regioncode="511681" if regioncode=="511681" & year==2011

replace regioncode="511702" if regioncode=="511702" & year==2011

replace regioncode="511721" if regioncode=="511721" & year==2011

replace regioncode="511722" if regioncode=="511722" & year==2011

replace regioncode="511723" if regioncode=="511723" & year==2011

replace regioncode="511724" if regioncode=="511724" & year==2011

replace regioncode="511725" if regioncode=="511725" & year==2011

replace regioncode="511781" if regioncode=="511781" & year==2011

replace regioncode="511802" if regioncode=="511802" & year==2011

replace regioncode="511821" if regioncode=="511821" & year==2011

replace regioncode="511822" if regioncode=="511822" & year==2011

replace regioncode="511823" if regioncode=="511823" & year==2011

replace regioncode="511824" if regioncode=="511824" & year==2011

replace regioncode="511825" if regioncode=="511825" & year==2011

replace regioncode="511826" if regioncode=="511826" & year==2011

replace regioncode="511827" if regioncode=="511827" & year==2011

replace regioncode="511902" if regioncode=="511902" & year==2011

replace regioncode="511921" if regioncode=="511921" & year==2011

replace regioncode="511922" if regioncode=="511922" & year==2011

replace regioncode="511923" if regioncode=="511923" & year==2011

replace regioncode="512002" if regioncode=="512002" & year==2011

replace regioncode="512021" if regioncode=="512021" & year==2011

replace regioncode="512022" if regioncode=="512022" & year==2011

replace regioncode="512081" if regioncode=="512081" & year==2011

replace regioncode="513221" if regioncode=="513221" & year==2011

replace regioncode="513222" if regioncode=="513222" & year==2011

replace regioncode="513223" if regioncode=="513223" & year==2011

replace regioncode="513224" if regioncode=="513224" & year==2011

replace regioncode="513225" if regioncode=="513225" & year==2011

replace regioncode="513226" if regioncode=="513226" & year==2011

replace regioncode="513227" if regioncode=="513227" & year==2011

replace regioncode="513228" if regioncode=="513228" & year==2011

replace regioncode="513229" if regioncode=="513229" & year==2011

replace regioncode="513230" if regioncode=="513230" & year==2011

replace regioncode="513231" if regioncode=="513231" & year==2011

replace regioncode="513232" if regioncode=="513232" & year==2011

replace regioncode="513233" if regioncode=="513233" & year==2011

replace regioncode="513321" if regioncode=="513321" & year==2011

replace regioncode="513322" if regioncode=="513322" & year==2011

replace regioncode="513323" if regioncode=="513323" & year==2011

replace regioncode="513324" if regioncode=="513324" & year==2011

replace regioncode="513325" if regioncode=="513325" & year==2011

replace regioncode="513326" if regioncode=="513326" & year==2011

replace regioncode="513327" if regioncode=="513327" & year==2011

replace regioncode="513328" if regioncode=="513328" & year==2011

replace regioncode="513329" if regioncode=="513329" & year==2011

replace regioncode="513330" if regioncode=="513330" & year==2011

replace regioncode="513331" if regioncode=="513331" & year==2011

replace regioncode="513332" if regioncode=="513332" & year==2011

replace regioncode="513333" if regioncode=="513333" & year==2011

replace regioncode="513334" if regioncode=="513334" & year==2011

replace regioncode="513335" if regioncode=="513335" & year==2011

replace regioncode="513336" if regioncode=="513336" & year==2011

replace regioncode="513337" if regioncode=="513337" & year==2011

replace regioncode="513338" if regioncode=="513338" & year==2011

replace regioncode="513401" if regioncode=="513401" & year==2011

replace regioncode="513422" if regioncode=="513422" & year==2011

replace regioncode="513423" if regioncode=="513423" & year==2011

replace regioncode="513424" if regioncode=="513424" & year==2011

replace regioncode="513425" if regioncode=="513425" & year==2011

replace regioncode="513426" if regioncode=="513426" & year==2011

replace regioncode="513427" if regioncode=="513427" & year==2011

replace regioncode="513428" if regioncode=="513428" & year==2011

replace regioncode="513429" if regioncode=="513429" & year==2011

replace regioncode="513430" if regioncode=="513430" & year==2011

replace regioncode="513431" if regioncode=="513431" & year==2011

replace regioncode="513432" if regioncode=="513432" & year==2011

replace regioncode="513433" if regioncode=="513433" & year==2011

replace regioncode="513434" if regioncode=="513434" & year==2011

replace regioncode="513435" if regioncode=="513435" & year==2011

replace regioncode="513436" if regioncode=="513436" & year==2011

replace regioncode="513437" if regioncode=="513437" & year==2011

replace regioncode="520102" if regioncode=="520102" & year==2011

replace regioncode="520103" if regioncode=="520103" & year==2011

replace regioncode="520111" if regioncode=="520111" & year==2011

replace regioncode="520112" if regioncode=="520112" & year==2011

replace regioncode="520113" if regioncode=="520113" & year==2011

replace regioncode="520114" if regioncode=="520114" & year==2011

replace regioncode="520121" if regioncode=="520121" & year==2011

replace regioncode="520122" if regioncode=="520122" & year==2011

replace regioncode="520123" if regioncode=="520123" & year==2011

replace regioncode="520181" if regioncode=="520181" & year==2011

replace regioncode="520201" if regioncode=="520201" & year==2011

replace regioncode="520222" if regioncode=="520222" & year==2011

replace regioncode="520203" if regioncode=="520203" & year==2011

replace regioncode="520221" if regioncode=="520221" & year==2011

replace regioncode="520302" if regioncode=="520302" & year==2011

replace regioncode="520321" if regioncode=="520321" & year==2011

replace regioncode="520322" if regioncode=="520322" & year==2011

replace regioncode="520323" if regioncode=="520323" & year==2011

replace regioncode="520324" if regioncode=="520324" & year==2011

replace regioncode="520325" if regioncode=="520325" & year==2011

replace regioncode="520326" if regioncode=="520326" & year==2011

replace regioncode="520327" if regioncode=="520327" & year==2011

replace regioncode="520328" if regioncode=="520328" & year==2011

replace regioncode="520329" if regioncode=="520329" & year==2011

replace regioncode="520330" if regioncode=="520330" & year==2011

replace regioncode="520381" if regioncode=="520381" & year==2011

replace regioncode="520382" if regioncode=="520382" & year==2011

replace regioncode="520402" if regioncode=="520402" & year==2011

replace regioncode="520421" if regioncode=="520421" & year==2011

replace regioncode="520422" if regioncode=="520422" & year==2011

replace regioncode="520423" if regioncode=="520423" & year==2011

replace regioncode="520424" if regioncode=="520424" & year==2011

replace regioncode="520425" if regioncode=="520425" & year==2011

replace regioncode="522201" if regioncode=="522201" & year==2011

replace regioncode="522222" if regioncode=="522222" & year==2011

replace regioncode="522223" if regioncode=="522223" & year==2011

replace regioncode="522224" if regioncode=="522224" & year==2011

replace regioncode="522225" if regioncode=="522225" & year==2011

replace regioncode="522226" if regioncode=="522226" & year==2011

replace regioncode="522227" if regioncode=="522227" & year==2011

replace regioncode="522228" if regioncode=="522228" & year==2011

replace regioncode="522229" if regioncode=="522229" & year==2011

replace regioncode="522230" if regioncode=="522230" & year==2011

replace regioncode="522301" if regioncode=="522301" & year==2011

replace regioncode="522322" if regioncode=="522322" & year==2011

replace regioncode="522323" if regioncode=="522323" & year==2011

replace regioncode="522324" if regioncode=="522324" & year==2011

replace regioncode="522325" if regioncode=="522325" & year==2011

replace regioncode="522326" if regioncode=="522326" & year==2011

replace regioncode="522327" if regioncode=="522327" & year==2011

replace regioncode="522328" if regioncode=="522328" & year==2011

replace regioncode="522401" if regioncode=="522401" & year==2011

replace regioncode="522422" if regioncode=="522422" & year==2011

replace regioncode="522423" if regioncode=="522423" & year==2011

replace regioncode="522424" if regioncode=="522424" & year==2011

replace regioncode="522425" if regioncode=="522425" & year==2011

replace regioncode="522426" if regioncode=="522426" & year==2011

replace regioncode="522427" if regioncode=="522427" & year==2011

replace regioncode="522428" if regioncode=="522428" & year==2011

replace regioncode="522601" if regioncode=="522601" & year==2011

replace regioncode="522622" if regioncode=="522622" & year==2011

replace regioncode="522623" if regioncode=="522623" & year==2011

replace regioncode="522624" if regioncode=="522624" & year==2011

replace regioncode="522625" if regioncode=="522625" & year==2011

replace regioncode="522626" if regioncode=="522626" & year==2011

replace regioncode="522627" if regioncode=="522627" & year==2011

replace regioncode="522628" if regioncode=="522628" & year==2011

replace regioncode="522629" if regioncode=="522629" & year==2011

replace regioncode="522630" if regioncode=="522630" & year==2011

replace regioncode="522631" if regioncode=="522631" & year==2011

replace regioncode="522632" if regioncode=="522632" & year==2011

replace regioncode="522633" if regioncode=="522633" & year==2011

replace regioncode="522634" if regioncode=="522634" & year==2011

replace regioncode="522635" if regioncode=="522635" & year==2011

replace regioncode="522636" if regioncode=="522636" & year==2011

replace regioncode="522701" if regioncode=="522701" & year==2011

replace regioncode="522702" if regioncode=="522702" & year==2011

replace regioncode="522722" if regioncode=="522722" & year==2011

replace regioncode="522723" if regioncode=="522723" & year==2011

replace regioncode="522725" if regioncode=="522725" & year==2011

replace regioncode="522726" if regioncode=="522726" & year==2011

replace regioncode="522727" if regioncode=="522727" & year==2011

replace regioncode="522728" if regioncode=="522728" & year==2011

replace regioncode="522729" if regioncode=="522729" & year==2011

replace regioncode="522730" if regioncode=="522730" & year==2011

replace regioncode="522731" if regioncode=="522731" & year==2011

replace regioncode="522732" if regioncode=="522732" & year==2011

replace regioncode="530102" if regioncode=="530102" & year==2011

replace regioncode="530103" if regioncode=="530103" & year==2011

replace regioncode="530111" if regioncode=="530111" & year==2011

replace regioncode="530112" if regioncode=="530112" & year==2011

replace regioncode="530113" if regioncode=="530113" & year==2011

replace regioncode="530121" if regioncode=="530121" & year==2011

replace regioncode="530122" if regioncode=="530122" & year==2011

replace regioncode="530124" if regioncode=="530124" & year==2011

replace regioncode="530125" if regioncode=="530125" & year==2011

replace regioncode="530126" if regioncode=="530126" & year==2011

replace regioncode="530127" if regioncode=="530127" & year==2011

replace regioncode="530128" if regioncode=="530128" & year==2011

replace regioncode="530129" if regioncode=="530129" & year==2011

replace regioncode="530181" if regioncode=="530181" & year==2011

replace regioncode="530302" if regioncode=="530302" & year==2011

replace regioncode="530321" if regioncode=="530321" & year==2011

replace regioncode="530322" if regioncode=="530322" & year==2011

replace regioncode="530323" if regioncode=="530323" & year==2011

replace regioncode="530324" if regioncode=="530324" & year==2011

replace regioncode="530325" if regioncode=="530325" & year==2011

replace regioncode="530326" if regioncode=="530326" & year==2011

replace regioncode="530328" if regioncode=="530328" & year==2011

replace regioncode="530381" if regioncode=="530381" & year==2011

replace regioncode="530402" if regioncode=="530402" & year==2011

replace regioncode="530421" if regioncode=="530421" & year==2011

replace regioncode="530422" if regioncode=="530422" & year==2011

replace regioncode="530423" if regioncode=="530423" & year==2011

replace regioncode="530424" if regioncode=="530424" & year==2011

replace regioncode="530425" if regioncode=="530425" & year==2011

replace regioncode="530426" if regioncode=="530426" & year==2011

replace regioncode="530427" if regioncode=="530427" & year==2011

replace regioncode="530428" if regioncode=="530428" & year==2011

replace regioncode="530502" if regioncode=="530502" & year==2011

replace regioncode="530521" if regioncode=="530521" & year==2011

replace regioncode="530522" if regioncode=="530522" & year==2011

replace regioncode="530523" if regioncode=="530523" & year==2011

replace regioncode="530524" if regioncode=="530524" & year==2011

replace regioncode="530602" if regioncode=="530602" & year==2011

replace regioncode="530621" if regioncode=="530621" & year==2011

replace regioncode="530622" if regioncode=="530622" & year==2011

replace regioncode="530623" if regioncode=="530623" & year==2011

replace regioncode="530624" if regioncode=="530624" & year==2011

replace regioncode="530625" if regioncode=="530625" & year==2011

replace regioncode="530626" if regioncode=="530626" & year==2011

replace regioncode="530627" if regioncode=="530627" & year==2011

replace regioncode="530628" if regioncode=="530628" & year==2011

replace regioncode="530629" if regioncode=="530629" & year==2011

replace regioncode="530630" if regioncode=="530630" & year==2011

replace regioncode="530721" if regioncode=="530721" & year==2011

replace regioncode="530722" if regioncode=="530722" & year==2011

replace regioncode="530723" if regioncode=="530723" & year==2011

replace regioncode="530724" if regioncode=="530724" & year==2011

replace regioncode="532701" if regioncode=="530802" & year==2011

replace regioncode="532722" if regioncode=="530821" & year==2011

replace regioncode="532723" if regioncode=="530822" & year==2011

replace regioncode="532724" if regioncode=="530823" & year==2011

replace regioncode="532725" if regioncode=="530824" & year==2011

replace regioncode="532726" if regioncode=="530825" & year==2011

replace regioncode="532727" if regioncode=="530826" & year==2011

replace regioncode="532728" if regioncode=="530827" & year==2011

replace regioncode="532729" if regioncode=="530828" & year==2011

replace regioncode="532730" if regioncode=="530829" & year==2011

replace regioncode="533521" if regioncode=="530902" & year==2011

replace regioncode="533522" if regioncode=="530921" & year==2011

replace regioncode="533523" if regioncode=="530922" & year==2011

replace regioncode="533524" if regioncode=="530923" & year==2011

replace regioncode="533525" if regioncode=="530924" & year==2011

replace regioncode="533526" if regioncode=="530925" & year==2011

replace regioncode="533527" if regioncode=="530926" & year==2011

replace regioncode="533528" if regioncode=="530927" & year==2011

replace regioncode="532301" if regioncode=="532301" & year==2011

replace regioncode="532322" if regioncode=="532322" & year==2011

replace regioncode="532323" if regioncode=="532323" & year==2011

replace regioncode="532324" if regioncode=="532324" & year==2011

replace regioncode="532325" if regioncode=="532325" & year==2011

replace regioncode="532326" if regioncode=="532326" & year==2011

replace regioncode="532327" if regioncode=="532327" & year==2011

replace regioncode="532328" if regioncode=="532328" & year==2011

replace regioncode="532329" if regioncode=="532329" & year==2011

replace regioncode="532331" if regioncode=="532331" & year==2011

replace regioncode="532501" if regioncode=="532501" & year==2011

replace regioncode="532502" if regioncode=="532502" & year==2011

replace regioncode="532522" if regioncode=="532522" & year==2011

replace regioncode="532523" if regioncode=="532523" & year==2011

replace regioncode="532524" if regioncode=="532524" & year==2011

replace regioncode="532525" if regioncode=="532525" & year==2011

replace regioncode="532526" if regioncode=="532526" & year==2011

replace regioncode="532527" if regioncode=="532527" & year==2011

replace regioncode="532528" if regioncode=="532528" & year==2011

replace regioncode="532529" if regioncode=="532529" & year==2011

replace regioncode="532530" if regioncode=="532530" & year==2011

replace regioncode="532531" if regioncode=="532531" & year==2011

replace regioncode="532532" if regioncode=="532532" & year==2011

replace regioncode="532621" if regioncode=="532621" & year==2011

replace regioncode="532622" if regioncode=="532622" & year==2011

replace regioncode="532623" if regioncode=="532623" & year==2011

replace regioncode="532624" if regioncode=="532624" & year==2011

replace regioncode="532625" if regioncode=="532625" & year==2011

replace regioncode="532626" if regioncode=="532626" & year==2011

replace regioncode="532627" if regioncode=="532627" & year==2011

replace regioncode="532628" if regioncode=="532628" & year==2011

replace regioncode="532801" if regioncode=="532801" & year==2011

replace regioncode="532822" if regioncode=="532822" & year==2011

replace regioncode="532823" if regioncode=="532823" & year==2011

replace regioncode="532901" if regioncode=="532901" & year==2011

replace regioncode="532922" if regioncode=="532922" & year==2011

replace regioncode="532923" if regioncode=="532923" & year==2011

replace regioncode="532924" if regioncode=="532924" & year==2011

replace regioncode="532925" if regioncode=="532925" & year==2011

replace regioncode="532926" if regioncode=="532926" & year==2011

replace regioncode="532927" if regioncode=="532927" & year==2011

replace regioncode="532928" if regioncode=="532928" & year==2011

replace regioncode="532929" if regioncode=="532929" & year==2011

replace regioncode="532930" if regioncode=="532930" & year==2011

replace regioncode="532931" if regioncode=="532931" & year==2011

replace regioncode="532932" if regioncode=="532932" & year==2011

replace regioncode="533102" if regioncode=="533102" & year==2011

replace regioncode="533103" if regioncode=="533103" & year==2011

replace regioncode="533122" if regioncode=="533122" & year==2011

replace regioncode="533123" if regioncode=="533123" & year==2011

replace regioncode="533124" if regioncode=="533124" & year==2011

replace regioncode="533321" if regioncode=="533321" & year==2011

replace regioncode="533323" if regioncode=="533323" & year==2011

replace regioncode="533324" if regioncode=="533324" & year==2011

replace regioncode="533325" if regioncode=="533325" & year==2011

replace regioncode="533421" if regioncode=="533421" & year==2011

replace regioncode="533422" if regioncode=="533422" & year==2011

replace regioncode="533423" if regioncode=="533423" & year==2011

replace regioncode="540102" if regioncode=="540102" & year==2011

replace regioncode="540121" if regioncode=="540121" & year==2011

replace regioncode="540122" if regioncode=="540122" & year==2011

replace regioncode="540123" if regioncode=="540123" & year==2011

replace regioncode="540124" if regioncode=="540124" & year==2011

replace regioncode="540125" if regioncode=="540125" & year==2011

replace regioncode="540126" if regioncode=="540126" & year==2011

replace regioncode="540127" if regioncode=="540127" & year==2011

replace regioncode="542121" if regioncode=="542121" & year==2011

replace regioncode="542122" if regioncode=="542122" & year==2011

replace regioncode="542123" if regioncode=="542123" & year==2011

replace regioncode="542124" if regioncode=="542124" & year==2011

replace regioncode="542125" if regioncode=="542125" & year==2011

replace regioncode="542126" if regioncode=="542126" & year==2011

replace regioncode="542127" if regioncode=="542127" & year==2011

replace regioncode="542128" if regioncode=="542128" & year==2011

replace regioncode="542129" if regioncode=="542129" & year==2011

replace regioncode="542132" if regioncode=="542132" & year==2011

replace regioncode="542133" if regioncode=="542133" & year==2011

replace regioncode="542221" if regioncode=="542221" & year==2011

replace regioncode="542223" if regioncode=="542223" & year==2011

replace regioncode="542224" if regioncode=="542224" & year==2011

replace regioncode="542225" if regioncode=="542225" & year==2011

replace regioncode="542227" if regioncode=="542227" & year==2011

replace regioncode="542228" if regioncode=="542228" & year==2011

replace regioncode="542229" if regioncode=="542229" & year==2011

replace regioncode="542231" if regioncode=="542231" & year==2011

replace regioncode="542232" if regioncode=="542232" & year==2011

replace regioncode="542233" if regioncode=="542233" & year==2011

replace regioncode="542301" if regioncode=="542301" & year==2011

replace regioncode="542322" if regioncode=="542322" & year==2011

replace regioncode="542323" if regioncode=="542323" & year==2011

replace regioncode="542324" if regioncode=="542324" & year==2011

replace regioncode="542325" if regioncode=="542325" & year==2011

replace regioncode="542326" if regioncode=="542326" & year==2011

replace regioncode="542327" if regioncode=="542327" & year==2011

replace regioncode="542328" if regioncode=="542328" & year==2011

replace regioncode="542329" if regioncode=="542329" & year==2011

replace regioncode="542330" if regioncode=="542330" & year==2011

replace regioncode="542331" if regioncode=="542331" & year==2011

replace regioncode="542332" if regioncode=="542332" & year==2011

replace regioncode="542334" if regioncode=="542334" & year==2011

replace regioncode="542336" if regioncode=="542336" & year==2011

replace regioncode="542338" if regioncode=="542338" & year==2011

replace regioncode="542421" if regioncode=="542421" & year==2011

replace regioncode="542422" if regioncode=="542422" & year==2011

replace regioncode="542423" if regioncode=="542423" & year==2011

replace regioncode="542424" if regioncode=="542424" & year==2011

replace regioncode="542425" if regioncode=="542425" & year==2011

replace regioncode="542426" if regioncode=="542426" & year==2011

replace regioncode="542427" if regioncode=="542427" & year==2011

replace regioncode="542429" if regioncode=="542429" & year==2011

replace regioncode="542430" if regioncode=="542430" & year==2011

replace regioncode="542523" if regioncode=="542523" & year==2011

replace regioncode="542621" if regioncode=="542621" & year==2011

replace regioncode="542622" if regioncode=="542622" & year==2011

replace regioncode="542623" if regioncode=="542623" & year==2011

replace regioncode="542625" if regioncode=="542625" & year==2011

replace regioncode="542626" if regioncode=="542626" & year==2011

replace regioncode="610102" if regioncode=="610102" & year==2011

replace regioncode="610103" if regioncode=="610103" & year==2011

replace regioncode="610104" if regioncode=="610104" & year==2011

replace regioncode="610111" if regioncode=="610111" & year==2011

replace regioncode="610112" if regioncode=="610112" & year==2011

replace regioncode="610113" if regioncode=="610113" & year==2011

replace regioncode="610114" if regioncode=="610114" & year==2011

replace regioncode="610115" if regioncode=="610115" & year==2011

replace regioncode="610116" if regioncode=="610116" & year==2011

replace regioncode="610122" if regioncode=="610122" & year==2011

replace regioncode="610124" if regioncode=="610124" & year==2011

replace regioncode="610125" if regioncode=="610125" & year==2011

replace regioncode="610126" if regioncode=="610126" & year==2011

replace regioncode="610202" if regioncode=="610202" & year==2011

replace regioncode="610203" if regioncode=="610203" & year==2011

replace regioncode="610204" if regioncode=="610204" & year==2011

replace regioncode="610222" if regioncode=="610222" & year==2011

replace regioncode="610302" if regioncode=="610302" & year==2011

replace regioncode="610303" if regioncode=="610303" & year==2011

replace regioncode="610304" if regioncode=="610304" & year==2011

replace regioncode="610322" if regioncode=="610322" & year==2011

replace regioncode="610323" if regioncode=="610323" & year==2011

replace regioncode="610324" if regioncode=="610324" & year==2011

replace regioncode="610326" if regioncode=="610326" & year==2011

replace regioncode="610327" if regioncode=="610327" & year==2011

replace regioncode="610328" if regioncode=="610328" & year==2011

replace regioncode="610329" if regioncode=="610329" & year==2011

replace regioncode="610330" if regioncode=="610330" & year==2011

replace regioncode="610331" if regioncode=="610331" & year==2011

replace regioncode="610402" if regioncode=="610402" & year==2011

replace regioncode="610403" if regioncode=="610403" & year==2011

replace regioncode="610404" if regioncode=="610404" & year==2011

replace regioncode="610422" if regioncode=="610422" & year==2011

replace regioncode="610423" if regioncode=="610423" & year==2011

replace regioncode="610424" if regioncode=="610424" & year==2011

replace regioncode="610425" if regioncode=="610425" & year==2011

replace regioncode="610426" if regioncode=="610426" & year==2011

replace regioncode="610427" if regioncode=="610427" & year==2011

replace regioncode="610428" if regioncode=="610428" & year==2011

replace regioncode="610429" if regioncode=="610429" & year==2011

replace regioncode="610430" if regioncode=="610430" & year==2011

replace regioncode="610431" if regioncode=="610431" & year==2011

replace regioncode="610481" if regioncode=="610481" & year==2011

replace regioncode="610502" if regioncode=="610502" & year==2011

replace regioncode="610521" if regioncode=="610521" & year==2011

replace regioncode="610522" if regioncode=="610522" & year==2011

replace regioncode="610523" if regioncode=="610523" & year==2011

replace regioncode="610524" if regioncode=="610524" & year==2011

replace regioncode="610525" if regioncode=="610525" & year==2011

replace regioncode="610526" if regioncode=="610526" & year==2011

replace regioncode="610527" if regioncode=="610527" & year==2011

replace regioncode="610528" if regioncode=="610528" & year==2011

replace regioncode="610581" if regioncode=="610581" & year==2011

replace regioncode="610582" if regioncode=="610582" & year==2011

replace regioncode="610602" if regioncode=="610602" & year==2011

replace regioncode="610621" if regioncode=="610621" & year==2011

replace regioncode="610622" if regioncode=="610622" & year==2011

replace regioncode="610623" if regioncode=="610623" & year==2011

replace regioncode="610624" if regioncode=="610624" & year==2011

replace regioncode="610625" if regioncode=="610625" & year==2011

replace regioncode="610626" if regioncode=="610626" & year==2011

replace regioncode="610627" if regioncode=="610627" & year==2011

replace regioncode="610628" if regioncode=="610628" & year==2011

replace regioncode="610629" if regioncode=="610629" & year==2011

replace regioncode="610630" if regioncode=="610630" & year==2011

replace regioncode="610631" if regioncode=="610631" & year==2011

replace regioncode="610632" if regioncode=="610632" & year==2011

replace regioncode="610702" if regioncode=="610702" & year==2011

replace regioncode="610721" if regioncode=="610721" & year==2011

replace regioncode="610722" if regioncode=="610722" & year==2011

replace regioncode="610723" if regioncode=="610723" & year==2011

replace regioncode="610724" if regioncode=="610724" & year==2011

replace regioncode="610725" if regioncode=="610725" & year==2011

replace regioncode="610726" if regioncode=="610726" & year==2011

replace regioncode="610727" if regioncode=="610727" & year==2011

replace regioncode="610728" if regioncode=="610728" & year==2011

replace regioncode="610729" if regioncode=="610729" & year==2011

replace regioncode="610730" if regioncode=="610730" & year==2011

replace regioncode="610802" if regioncode=="610802" & year==2011

replace regioncode="610821" if regioncode=="610821" & year==2011

replace regioncode="610822" if regioncode=="610822" & year==2011

replace regioncode="610823" if regioncode=="610823" & year==2011

replace regioncode="610824" if regioncode=="610824" & year==2011

replace regioncode="610825" if regioncode=="610825" & year==2011

replace regioncode="610826" if regioncode=="610826" & year==2011

replace regioncode="610827" if regioncode=="610827" & year==2011

replace regioncode="610828" if regioncode=="610828" & year==2011

replace regioncode="610829" if regioncode=="610829" & year==2011

replace regioncode="610830" if regioncode=="610830" & year==2011

replace regioncode="610831" if regioncode=="610831" & year==2011

replace regioncode="610902" if regioncode=="610902" & year==2011

replace regioncode="610921" if regioncode=="610921" & year==2011

replace regioncode="610922" if regioncode=="610922" & year==2011

replace regioncode="610923" if regioncode=="610923" & year==2011

replace regioncode="610924" if regioncode=="610924" & year==2011

replace regioncode="610925" if regioncode=="610925" & year==2011

replace regioncode="610926" if regioncode=="610926" & year==2011

replace regioncode="610927" if regioncode=="610927" & year==2011

replace regioncode="610928" if regioncode=="610928" & year==2011

replace regioncode="610929" if regioncode=="610929" & year==2011

replace regioncode="611002" if regioncode=="611002" & year==2011

replace regioncode="611021" if regioncode=="611021" & year==2011

replace regioncode="611022" if regioncode=="611022" & year==2011

replace regioncode="611023" if regioncode=="611023" & year==2011

replace regioncode="611024" if regioncode=="611024" & year==2011

replace regioncode="611025" if regioncode=="611025" & year==2011

replace regioncode="611026" if regioncode=="611026" & year==2011

replace regioncode="620102" if regioncode=="620102" & year==2011

replace regioncode="620103" if regioncode=="620103" & year==2011

replace regioncode="620104" if regioncode=="620104" & year==2011

replace regioncode="620105" if regioncode=="620105" & year==2011

replace regioncode="620111" if regioncode=="620111" & year==2011

replace regioncode="620121" if regioncode=="620121" & year==2011

replace regioncode="620122" if regioncode=="620122" & year==2011

replace regioncode="620123" if regioncode=="620123" & year==2011

replace regioncode="620201" if regioncode=="620201" & year==2011

replace regioncode="620302" if regioncode=="620302" & year==2011

replace regioncode="620321" if regioncode=="620321" & year==2011

replace regioncode="620402" if regioncode=="620402" & year==2011

replace regioncode="620403" if regioncode=="620403" & year==2011

replace regioncode="620421" if regioncode=="620421" & year==2011

replace regioncode="620422" if regioncode=="620422" & year==2011

replace regioncode="620423" if regioncode=="620423" & year==2011

replace regioncode="620502" if regioncode=="620502" & year==2011

replace regioncode="620503" if regioncode=="620503" & year==2011

replace regioncode="620521" if regioncode=="620521" & year==2011

replace regioncode="620522" if regioncode=="620522" & year==2011

replace regioncode="620523" if regioncode=="620523" & year==2011

replace regioncode="620524" if regioncode=="620524" & year==2011

replace regioncode="620525" if regioncode=="620525" & year==2011

replace regioncode="622301" if regioncode=="620602" & year==2011

replace regioncode="622322" if regioncode=="620621" & year==2011

replace regioncode="622323" if regioncode=="620622" & year==2011

replace regioncode="622326" if regioncode=="620623" & year==2011

replace regioncode="622201" if regioncode=="620702" & year==2011

replace regioncode="622222" if regioncode=="620721" & year==2011

replace regioncode="622223" if regioncode=="620722" & year==2011

replace regioncode="622224" if regioncode=="620723" & year==2011

replace regioncode="622225" if regioncode=="620724" & year==2011

replace regioncode="622226" if regioncode=="620725" & year==2011

replace regioncode="622701" if regioncode=="620802" & year==2011

replace regioncode="622722" if regioncode=="620821" & year==2011

replace regioncode="622723" if regioncode=="620822" & year==2011

replace regioncode="622724" if regioncode=="620823" & year==2011

replace regioncode="622725" if regioncode=="620824" & year==2011

replace regioncode="622726" if regioncode=="620825" & year==2011

replace regioncode="622727" if regioncode=="620826" & year==2011

replace regioncode="622102" if regioncode=="620902" & year==2011

replace regioncode="622123" if regioncode=="620921" & year==2011

replace regioncode="622126" if regioncode=="620922" & year==2011

replace regioncode="622124" if regioncode=="620923" & year==2011

replace regioncode="622125" if regioncode=="620924" & year==2011

replace regioncode="622101" if regioncode=="620981" & year==2011

replace regioncode="622103" if regioncode=="620982" & year==2011

replace regioncode="622801" if regioncode=="621002" & year==2011

replace regioncode="622821" if regioncode=="621021" & year==2011

replace regioncode="622822" if regioncode=="621022" & year==2011

replace regioncode="622823" if regioncode=="621023" & year==2011

replace regioncode="622824" if regioncode=="621024" & year==2011

replace regioncode="622825" if regioncode=="621025" & year==2011

replace regioncode="622826" if regioncode=="621026" & year==2011

replace regioncode="622827" if regioncode=="621027" & year==2011

replace regioncode="622421" if regioncode=="621102" & year==2011

replace regioncode="622424" if regioncode=="621121" & year==2011

replace regioncode="622425" if regioncode=="621122" & year==2011

replace regioncode="622426" if regioncode=="621123" & year==2011

replace regioncode="622427" if regioncode=="621124" & year==2011

replace regioncode="622428" if regioncode=="621125" & year==2011

replace regioncode="622429" if regioncode=="621126" & year==2011

replace regioncode="622621" if regioncode=="621202" & year==2011

replace regioncode="622624" if regioncode=="621221" & year==2011

replace regioncode="622626" if regioncode=="621222" & year==2011

replace regioncode="622623" if regioncode=="621223" & year==2011

replace regioncode="622625" if regioncode=="621224" & year==2011

replace regioncode="622627" if regioncode=="621225" & year==2011

replace regioncode="622628" if regioncode=="621226" & year==2011

replace regioncode="622630" if regioncode=="621227" & year==2011

replace regioncode="622629" if regioncode=="621228" & year==2011

replace regioncode="622901" if regioncode=="622901" & year==2011

replace regioncode="622921" if regioncode=="622921" & year==2011

replace regioncode="622922" if regioncode=="622922" & year==2011

replace regioncode="622923" if regioncode=="622923" & year==2011

replace regioncode="622924" if regioncode=="622924" & year==2011

replace regioncode="622925" if regioncode=="622925" & year==2011

replace regioncode="622926" if regioncode=="622926" & year==2011

replace regioncode="622927" if regioncode=="622927" & year==2011

replace regioncode="623001" if regioncode=="623001" & year==2011

replace regioncode="623021" if regioncode=="623021" & year==2011

replace regioncode="623022" if regioncode=="623022" & year==2011

replace regioncode="623023" if regioncode=="623023" & year==2011

replace regioncode="623024" if regioncode=="623024" & year==2011

replace regioncode="623025" if regioncode=="623025" & year==2011

replace regioncode="623026" if regioncode=="623026" & year==2011

replace regioncode="623027" if regioncode=="623027" & year==2011

replace regioncode="630102" if regioncode=="630102" & year==2011

replace regioncode="630103" if regioncode=="630103" & year==2011

replace regioncode="630104" if regioncode=="630104" & year==2011

replace regioncode="630105" if regioncode=="630105" & year==2011

replace regioncode="630121" if regioncode=="630121" & year==2011

replace regioncode="630122" if regioncode=="630122" & year==2011

replace regioncode="630123" if regioncode=="630123" & year==2011

replace regioncode="632121" if regioncode=="632121" & year==2011

replace regioncode="632122" if regioncode=="632122" & year==2011

replace regioncode="632123" if regioncode=="632123" & year==2011

replace regioncode="632126" if regioncode=="632126" & year==2011

replace regioncode="632127" if regioncode=="632127" & year==2011

replace regioncode="632128" if regioncode=="632128" & year==2011

replace regioncode="632221" if regioncode=="632221" & year==2011

replace regioncode="632222" if regioncode=="632222" & year==2011

replace regioncode="632223" if regioncode=="632223" & year==2011

replace regioncode="632224" if regioncode=="632224" & year==2011

replace regioncode="632321" if regioncode=="632321" & year==2011

replace regioncode="632322" if regioncode=="632322" & year==2011

replace regioncode="632521" if regioncode=="632521" & year==2011

replace regioncode="632522" if regioncode=="632522" & year==2011

replace regioncode="632523" if regioncode=="632523" & year==2011

replace regioncode="632524" if regioncode=="632524" & year==2011

replace regioncode="632525" if regioncode=="632525" & year==2011

replace regioncode="632621" if regioncode=="632621" & year==2011

replace regioncode="632622" if regioncode=="632622" & year==2011

replace regioncode="632623" if regioncode=="632623" & year==2011

replace regioncode="632624" if regioncode=="632624" & year==2011

replace regioncode="632625" if regioncode=="632625" & year==2011

replace regioncode="632626" if regioncode=="632626" & year==2011

replace regioncode="632721" if regioncode=="632721" & year==2011

replace regioncode="632723" if regioncode=="632723" & year==2011

replace regioncode="632724" if regioncode=="632724" & year==2011

replace regioncode="632725" if regioncode=="632725" & year==2011

replace regioncode="632726" if regioncode=="632726" & year==2011

replace regioncode="632801" if regioncode=="632801" & year==2011

replace regioncode="632802" if regioncode=="632802" & year==2011

replace regioncode="632821" if regioncode=="632821" & year==2011

replace regioncode="632822" if regioncode=="632822" & year==2011

replace regioncode="640104" if regioncode=="640104" & year==2011

replace regioncode="640105" if regioncode=="640105" & year==2011

replace regioncode="640106" if regioncode=="640106" & year==2011

replace regioncode="640121" if regioncode=="640121" & year==2011

replace regioncode="640122" if regioncode=="640122" & year==2011

replace regioncode="640181" if regioncode=="640181" & year==2011

replace regioncode="640202" if regioncode=="640202" & year==2011

replace regioncode="640203" if regioncode=="640205" & year==2011

replace regioncode="640221" if regioncode=="640221" & year==2011

replace regioncode="640302" if regioncode=="640302" & year==2011

replace regioncode="640321" if regioncode=="640502" & year==2011

replace regioncode="640322" if regioncode=="640521" & year==2011

replace regioncode="640323" if regioncode=="640323" & year==2011

replace regioncode="640324" if regioncode=="640324" & year==2011

replace regioncode="640381" if regioncode=="640381" & year==2011

replace regioncode="640402" if regioncode=="640402" & year==2011

replace regioncode="640421" if regioncode=="640522" & year==2011

replace regioncode="640422" if regioncode=="640422" & year==2011

replace regioncode="640423" if regioncode=="640423" & year==2011

replace regioncode="640424" if regioncode=="640424" & year==2011

replace regioncode="640425" if regioncode=="640425" & year==2011

replace regioncode="650102" if regioncode=="650102" & year==2011

replace regioncode="650103" if regioncode=="650103" & year==2011

replace regioncode="650104" if regioncode=="650104" & year==2011

replace regioncode="650105" if regioncode=="650105" & year==2011

replace regioncode="650106" if regioncode=="650106" & year==2011

replace regioncode="650107" if regioncode=="650107" & year==2011

replace regioncode="650108" if regioncode=="650109" & year==2011

replace regioncode="650121" if regioncode=="650121" & year==2011

replace regioncode="650202" if regioncode=="650202" & year==2011

replace regioncode="650203" if regioncode=="650203" & year==2011

replace regioncode="650204" if regioncode=="650204" & year==2011

replace regioncode="650205" if regioncode=="650205" & year==2011

replace regioncode="652101" if regioncode=="652101" & year==2011

replace regioncode="652122" if regioncode=="652122" & year==2011

replace regioncode="652123" if regioncode=="652123" & year==2011

replace regioncode="652201" if regioncode=="652201" & year==2011

replace regioncode="652222" if regioncode=="652222" & year==2011

replace regioncode="652223" if regioncode=="652223" & year==2011

replace regioncode="652301" if regioncode=="652301" & year==2011

replace regioncode="652302" if regioncode=="652302" & year==2011

replace regioncode="652303" if regioncode=="652303" & year==2011

replace regioncode="652323" if regioncode=="652323" & year==2011

replace regioncode="652324" if regioncode=="652324" & year==2011

replace regioncode="652325" if regioncode=="652325" & year==2011

replace regioncode="652327" if regioncode=="652327" & year==2011

replace regioncode="652328" if regioncode=="652328" & year==2011

replace regioncode="652701" if regioncode=="652701" & year==2011

replace regioncode="652722" if regioncode=="652722" & year==2011

replace regioncode="652723" if regioncode=="652723" & year==2011

replace regioncode="652801" if regioncode=="652801" & year==2011

replace regioncode="652822" if regioncode=="652822" & year==2011

replace regioncode="652823" if regioncode=="652823" & year==2011

replace regioncode="652824" if regioncode=="652824" & year==2011

replace regioncode="652825" if regioncode=="652825" & year==2011

replace regioncode="652826" if regioncode=="652826" & year==2011

replace regioncode="652827" if regioncode=="652827" & year==2011

replace regioncode="652828" if regioncode=="652828" & year==2011

replace regioncode="652829" if regioncode=="652829" & year==2011

replace regioncode="652901" if regioncode=="652901" & year==2011

replace regioncode="652922" if regioncode=="652922" & year==2011

replace regioncode="652923" if regioncode=="652923" & year==2011

replace regioncode="652924" if regioncode=="652924" & year==2011

replace regioncode="652925" if regioncode=="652925" & year==2011

replace regioncode="652926" if regioncode=="652926" & year==2011

replace regioncode="652928" if regioncode=="652928" & year==2011

replace regioncode="652929" if regioncode=="652929" & year==2011

replace regioncode="653001" if regioncode=="653001" & year==2011

replace regioncode="653022" if regioncode=="653022" & year==2011

replace regioncode="653023" if regioncode=="653023" & year==2011

replace regioncode="653024" if regioncode=="653024" & year==2011

replace regioncode="653101" if regioncode=="653101" & year==2011

replace regioncode="653121" if regioncode=="653121" & year==2011

replace regioncode="653122" if regioncode=="653122" & year==2011

replace regioncode="653123" if regioncode=="653123" & year==2011

replace regioncode="653124" if regioncode=="653124" & year==2011

replace regioncode="653125" if regioncode=="653125" & year==2011

replace regioncode="653126" if regioncode=="653126" & year==2011

replace regioncode="653127" if regioncode=="653127" & year==2011

replace regioncode="653128" if regioncode=="653128" & year==2011

replace regioncode="653129" if regioncode=="653129" & year==2011

replace regioncode="653130" if regioncode=="653130" & year==2011

replace regioncode="653131" if regioncode=="653131" & year==2011

replace regioncode="653201" if regioncode=="653201" & year==2011

replace regioncode="653221" if regioncode=="653221" & year==2011

replace regioncode="653222" if regioncode=="653222" & year==2011

replace regioncode="653223" if regioncode=="653223" & year==2011

replace regioncode="653224" if regioncode=="653224" & year==2011

replace regioncode="653225" if regioncode=="653225" & year==2011

replace regioncode="653226" if regioncode=="653226" & year==2011

replace regioncode="653227" if regioncode=="653227" & year==2011

replace regioncode="654002" if regioncode=="654002" & year==2011

replace regioncode="654003" if regioncode=="654003" & year==2011

replace regioncode="654021" if regioncode=="654021" & year==2011

replace regioncode="654022" if regioncode=="654022" & year==2011

replace regioncode="654023" if regioncode=="654023" & year==2011

replace regioncode="654024" if regioncode=="654024" & year==2011

replace regioncode="654025" if regioncode=="654025" & year==2011

replace regioncode="654026" if regioncode=="654026" & year==2011

replace regioncode="654027" if regioncode=="654027" & year==2011

replace regioncode="654028" if regioncode=="654028" & year==2011

replace regioncode="654201" if regioncode=="654201" & year==2011

replace regioncode="654202" if regioncode=="654202" & year==2011

replace regioncode="654221" if regioncode=="654221" & year==2011

replace regioncode="654223" if regioncode=="654223" & year==2011

replace regioncode="654224" if regioncode=="654224" & year==2011

replace regioncode="654225" if regioncode=="654225" & year==2011

replace regioncode="654226" if regioncode=="654226" & year==2011

replace regioncode="654301" if regioncode=="654301" & year==2011

replace regioncode="654321" if regioncode=="654321" & year==2011

replace regioncode="654322" if regioncode=="654322" & year==2011

replace regioncode="654323" if regioncode=="654323" & year==2011

replace regioncode="654324" if regioncode=="654324" & year==2011

replace regioncode="654325" if regioncode=="654325" & year==2011

replace regioncode="654326" if regioncode=="654326" & year==2011

replace regioncode="659001" if regioncode=="659001" & year==2011

replace regioncode="110101" if regioncode=="110101" & year==2012

replace regioncode="110102" if regioncode=="110102" & year==2012

replace regioncode="110103" if regioncode=="110103" & year==2012

replace regioncode="110104" if regioncode=="110104" & year==2012

replace regioncode="110105" if regioncode=="110105" & year==2012

replace regioncode="110106" if regioncode=="110106" & year==2012

replace regioncode="110107" if regioncode=="110107" & year==2012

replace regioncode="110108" if regioncode=="110108" & year==2012

replace regioncode="110109" if regioncode=="110109" & year==2012

replace regioncode="110111" if regioncode=="110111" & year==2012

replace regioncode="110112" if regioncode=="110112" & year==2012

replace regioncode="110113" if regioncode=="110113" & year==2012

replace regioncode="110114" if regioncode=="110114" & year==2012

replace regioncode="110115" if regioncode=="110115" & year==2012

replace regioncode="110117" if regioncode=="110117" & year==2012

replace regioncode="110116" if regioncode=="110116" & year==2012

replace regioncode="110228" if regioncode=="110228" & year==2012

replace regioncode="110229" if regioncode=="110229" & year==2012

replace regioncode="120101" if regioncode=="120101" & year==2012

replace regioncode="120102" if regioncode=="120102" & year==2012

replace regioncode="120103" if regioncode=="120103" & year==2012

replace regioncode="120104" if regioncode=="120104" & year==2012

replace regioncode="120105" if regioncode=="120105" & year==2012

replace regioncode="120106" if regioncode=="120106" & year==2012

replace regioncode="120107" if regioncode=="120107" & year==2012

replace regioncode="120108" if regioncode=="120108" & year==2012

replace regioncode="120109" if regioncode=="120109" & year==2012

replace regioncode="120110" if regioncode=="120110" & year==2012

replace regioncode="120111" if regioncode=="120111" & year==2012

replace regioncode="120112" if regioncode=="120112" & year==2012

replace regioncode="120113" if regioncode=="120113" & year==2012

replace regioncode="120114" if regioncode=="120114" & year==2012

replace regioncode="120115" if regioncode=="120115" & year==2012

replace regioncode="120221" if regioncode=="120221" & year==2012

replace regioncode="120223" if regioncode=="120223" & year==2012

replace regioncode="120225" if regioncode=="120225" & year==2012

replace regioncode="130102" if regioncode=="130102" & year==2012

replace regioncode="130103" if regioncode=="130103" & year==2012

replace regioncode="130104" if regioncode=="130104" & year==2012

replace regioncode="130105" if regioncode=="130105" & year==2012

replace regioncode="130107" if regioncode=="130107" & year==2012

replace regioncode="130108" if regioncode=="130108" & year==2012

replace regioncode="130121" if regioncode=="130121" & year==2012

replace regioncode="130123" if regioncode=="130123" & year==2012

replace regioncode="130124" if regioncode=="130124" & year==2012

replace regioncode="130125" if regioncode=="130125" & year==2012

replace regioncode="130126" if regioncode=="130126" & year==2012

replace regioncode="130127" if regioncode=="130127" & year==2012

replace regioncode="130128" if regioncode=="130128" & year==2012

replace regioncode="130129" if regioncode=="130129" & year==2012

replace regioncode="130130" if regioncode=="130130" & year==2012

replace regioncode="130131" if regioncode=="130131" & year==2012

replace regioncode="130132" if regioncode=="130132" & year==2012

replace regioncode="130133" if regioncode=="130133" & year==2012

replace regioncode="130181" if regioncode=="130181" & year==2012

replace regioncode="130182" if regioncode=="130182" & year==2012

replace regioncode="130183" if regioncode=="130183" & year==2012

replace regioncode="130184" if regioncode=="130184" & year==2012

replace regioncode="130185" if regioncode=="130185" & year==2012

replace regioncode="130202" if regioncode=="130202" & year==2012

replace regioncode="130203" if regioncode=="130203" & year==2012

replace regioncode="130204" if regioncode=="130204" & year==2012

replace regioncode="130205" if regioncode=="130205" & year==2012

replace regioncode="130208" if regioncode=="130208" & year==2012

replace regioncode="130223" if regioncode=="130223" & year==2012

replace regioncode="130224" if regioncode=="130224" & year==2012

replace regioncode="130225" if regioncode=="130225" & year==2012

replace regioncode="130227" if regioncode=="130227" & year==2012

replace regioncode="130229" if regioncode=="130229" & year==2012

replace regioncode="130230" if regioncode=="130230" & year==2012

replace regioncode="130281" if regioncode=="130281" & year==2012

replace regioncode="130207" if regioncode=="130207" & year==2012

replace regioncode="130283" if regioncode=="130283" & year==2012

replace regioncode="130302" if regioncode=="130302" & year==2012

replace regioncode="130303" if regioncode=="130303" & year==2012

replace regioncode="130304" if regioncode=="130304" & year==2012

replace regioncode="130321" if regioncode=="130321" & year==2012

replace regioncode="130322" if regioncode=="130322" & year==2012

replace regioncode="130323" if regioncode=="130323" & year==2012

replace regioncode="130324" if regioncode=="130324" & year==2012

replace regioncode="130402" if regioncode=="130402" & year==2012

replace regioncode="130403" if regioncode=="130403" & year==2012

replace regioncode="130404" if regioncode=="130404" & year==2012

replace regioncode="130406" if regioncode=="130406" & year==2012

replace regioncode="130421" if regioncode=="130421" & year==2012

replace regioncode="130423" if regioncode=="130423" & year==2012

replace regioncode="130424" if regioncode=="130424" & year==2012

replace regioncode="130425" if regioncode=="130425" & year==2012

replace regioncode="130426" if regioncode=="130426" & year==2012

replace regioncode="130427" if regioncode=="130427" & year==2012

replace regioncode="130428" if regioncode=="130428" & year==2012

replace regioncode="130429" if regioncode=="130429" & year==2012

replace regioncode="130430" if regioncode=="130430" & year==2012

replace regioncode="130431" if regioncode=="130431" & year==2012

replace regioncode="130432" if regioncode=="130432" & year==2012

replace regioncode="130433" if regioncode=="130433" & year==2012

replace regioncode="130434" if regioncode=="130434" & year==2012

replace regioncode="130435" if regioncode=="130435" & year==2012

replace regioncode="130481" if regioncode=="130481" & year==2012

replace regioncode="130502" if regioncode=="130502" & year==2012

replace regioncode="130503" if regioncode=="130503" & year==2012

replace regioncode="130521" if regioncode=="130521" & year==2012

replace regioncode="130522" if regioncode=="130522" & year==2012

replace regioncode="130523" if regioncode=="130523" & year==2012

replace regioncode="130524" if regioncode=="130524" & year==2012

replace regioncode="130525" if regioncode=="130525" & year==2012

replace regioncode="130526" if regioncode=="130526" & year==2012

replace regioncode="130527" if regioncode=="130527" & year==2012

replace regioncode="130528" if regioncode=="130528" & year==2012

replace regioncode="130529" if regioncode=="130529" & year==2012

replace regioncode="130530" if regioncode=="130530" & year==2012

replace regioncode="130531" if regioncode=="130531" & year==2012

replace regioncode="130532" if regioncode=="130532" & year==2012

replace regioncode="130533" if regioncode=="130533" & year==2012

replace regioncode="130534" if regioncode=="130534" & year==2012

replace regioncode="130535" if regioncode=="130535" & year==2012

replace regioncode="130581" if regioncode=="130581" & year==2012

replace regioncode="130582" if regioncode=="130582" & year==2012

replace regioncode="130602" if regioncode=="130602" & year==2012

replace regioncode="130603" if regioncode=="130603" & year==2012

replace regioncode="130604" if regioncode=="130604" & year==2012

replace regioncode="130621" if regioncode=="130621" & year==2012

replace regioncode="130622" if regioncode=="130622" & year==2012

replace regioncode="130623" if regioncode=="130623" & year==2012

replace regioncode="130624" if regioncode=="130624" & year==2012

replace regioncode="130625" if regioncode=="130625" & year==2012

replace regioncode="130626" if regioncode=="130626" & year==2012

replace regioncode="130627" if regioncode=="130627" & year==2012

replace regioncode="130628" if regioncode=="130628" & year==2012

replace regioncode="130629" if regioncode=="130629" & year==2012

replace regioncode="130630" if regioncode=="130630" & year==2012

replace regioncode="130631" if regioncode=="130631" & year==2012

replace regioncode="130632" if regioncode=="130632" & year==2012

replace regioncode="130633" if regioncode=="130633" & year==2012

replace regioncode="130634" if regioncode=="130634" & year==2012

replace regioncode="130635" if regioncode=="130635" & year==2012

replace regioncode="130636" if regioncode=="130636" & year==2012

replace regioncode="130637" if regioncode=="130637" & year==2012

replace regioncode="130638" if regioncode=="130638" & year==2012

replace regioncode="130681" if regioncode=="130681" & year==2012

replace regioncode="130682" if regioncode=="130682" & year==2012

replace regioncode="130683" if regioncode=="130683" & year==2012

replace regioncode="130684" if regioncode=="130684" & year==2012

replace regioncode="130702" if regioncode=="130702" & year==2012

replace regioncode="130703" if regioncode=="130703" & year==2012

replace regioncode="130705" if regioncode=="130705" & year==2012

replace regioncode="130706" if regioncode=="130706" & year==2012

replace regioncode="130721" if regioncode=="130721" & year==2012

replace regioncode="130722" if regioncode=="130722" & year==2012

replace regioncode="130723" if regioncode=="130723" & year==2012

replace regioncode="130724" if regioncode=="130724" & year==2012

replace regioncode="130725" if regioncode=="130725" & year==2012

replace regioncode="130726" if regioncode=="130726" & year==2012

replace regioncode="130727" if regioncode=="130727" & year==2012

replace regioncode="130728" if regioncode=="130728" & year==2012

replace regioncode="130729" if regioncode=="130729" & year==2012

replace regioncode="130730" if regioncode=="130730" & year==2012

replace regioncode="130731" if regioncode=="130731" & year==2012

replace regioncode="130732" if regioncode=="130732" & year==2012

replace regioncode="130733" if regioncode=="130733" & year==2012

replace regioncode="130802" if regioncode=="130802" & year==2012

replace regioncode="130803" if regioncode=="130803" & year==2012

replace regioncode="130804" if regioncode=="130804" & year==2012

replace regioncode="130821" if regioncode=="130821" & year==2012

replace regioncode="130822" if regioncode=="130822" & year==2012

replace regioncode="130823" if regioncode=="130823" & year==2012

replace regioncode="130824" if regioncode=="130824" & year==2012

replace regioncode="130825" if regioncode=="130825" & year==2012

replace regioncode="130826" if regioncode=="130826" & year==2012

replace regioncode="130827" if regioncode=="130827" & year==2012

replace regioncode="130828" if regioncode=="130828" & year==2012

replace regioncode="130902" if regioncode=="130902" & year==2012

replace regioncode="130903" if regioncode=="130903" & year==2012

replace regioncode="130921" if regioncode=="130921" & year==2012

replace regioncode="130922" if regioncode=="130922" & year==2012

replace regioncode="130923" if regioncode=="130923" & year==2012

replace regioncode="130924" if regioncode=="130924" & year==2012

replace regioncode="130925" if regioncode=="130925" & year==2012

replace regioncode="130926" if regioncode=="130926" & year==2012

replace regioncode="130927" if regioncode=="130927" & year==2012

replace regioncode="130928" if regioncode=="130928" & year==2012

replace regioncode="130929" if regioncode=="130929" & year==2012

replace regioncode="130930" if regioncode=="130930" & year==2012

replace regioncode="130981" if regioncode=="130981" & year==2012

replace regioncode="130982" if regioncode=="130982" & year==2012

replace regioncode="130983" if regioncode=="130983" & year==2012

replace regioncode="130984" if regioncode=="130984" & year==2012

replace regioncode="131002" if regioncode=="131002" & year==2012

replace regioncode="131003" if regioncode=="131003" & year==2012

replace regioncode="131022" if regioncode=="131022" & year==2012

replace regioncode="131023" if regioncode=="131023" & year==2012

replace regioncode="131024" if regioncode=="131024" & year==2012

replace regioncode="131025" if regioncode=="131025" & year==2012

replace regioncode="131026" if regioncode=="131026" & year==2012

replace regioncode="131028" if regioncode=="131028" & year==2012

replace regioncode="131081" if regioncode=="131081" & year==2012

replace regioncode="131082" if regioncode=="131082" & year==2012

replace regioncode="131102" if regioncode=="131102" & year==2012

replace regioncode="131121" if regioncode=="131121" & year==2012

replace regioncode="131122" if regioncode=="131122" & year==2012

replace regioncode="131123" if regioncode=="131123" & year==2012

replace regioncode="131124" if regioncode=="131124" & year==2012

replace regioncode="131125" if regioncode=="131125" & year==2012

replace regioncode="131126" if regioncode=="131126" & year==2012

replace regioncode="131127" if regioncode=="131127" & year==2012

replace regioncode="131128" if regioncode=="131128" & year==2012

replace regioncode="131181" if regioncode=="131181" & year==2012

replace regioncode="131182" if regioncode=="131182" & year==2012

replace regioncode="140105" if regioncode=="140105" & year==2012

replace regioncode="140106" if regioncode=="140106" & year==2012

replace regioncode="140107" if regioncode=="140107" & year==2012

replace regioncode="140108" if regioncode=="140108" & year==2012

replace regioncode="140109" if regioncode=="140109" & year==2012

replace regioncode="140110" if regioncode=="140110" & year==2012

replace regioncode="140121" if regioncode=="140121" & year==2012

replace regioncode="140122" if regioncode=="140122" & year==2012

replace regioncode="140123" if regioncode=="140123" & year==2012

replace regioncode="140181" if regioncode=="140181" & year==2012

replace regioncode="140202" if regioncode=="140202" & year==2012

replace regioncode="140203" if regioncode=="140203" & year==2012

replace regioncode="140211" if regioncode=="140211" & year==2012

replace regioncode="140212" if regioncode=="140212" & year==2012

replace regioncode="140221" if regioncode=="140221" & year==2012

replace regioncode="140222" if regioncode=="140222" & year==2012

replace regioncode="140223" if regioncode=="140223" & year==2012

replace regioncode="140224" if regioncode=="140224" & year==2012

replace regioncode="140225" if regioncode=="140225" & year==2012

replace regioncode="140226" if regioncode=="140226" & year==2012

replace regioncode="140227" if regioncode=="140227" & year==2012

replace regioncode="140302" if regioncode=="140302" & year==2012

replace regioncode="140303" if regioncode=="140303" & year==2012

replace regioncode="140311" if regioncode=="140311" & year==2012

replace regioncode="140321" if regioncode=="140321" & year==2012

replace regioncode="140322" if regioncode=="140322" & year==2012

replace regioncode="140402" if regioncode=="140402" & year==2012

replace regioncode="140411" if regioncode=="140411" & year==2012

replace regioncode="140421" if regioncode=="140421" & year==2012

replace regioncode="140423" if regioncode=="140423" & year==2012

replace regioncode="140424" if regioncode=="140424" & year==2012

replace regioncode="140425" if regioncode=="140425" & year==2012

replace regioncode="140426" if regioncode=="140426" & year==2012

replace regioncode="140427" if regioncode=="140427" & year==2012

replace regioncode="140428" if regioncode=="140428" & year==2012

replace regioncode="140429" if regioncode=="140429" & year==2012

replace regioncode="140430" if regioncode=="140430" & year==2012

replace regioncode="140431" if regioncode=="140431" & year==2012

replace regioncode="140481" if regioncode=="140481" & year==2012

replace regioncode="140502" if regioncode=="140502" & year==2012

replace regioncode="140521" if regioncode=="140521" & year==2012

replace regioncode="140522" if regioncode=="140522" & year==2012

replace regioncode="140524" if regioncode=="140524" & year==2012

replace regioncode="140525" if regioncode=="140525" & year==2012

replace regioncode="140581" if regioncode=="140581" & year==2012

replace regioncode="140602" if regioncode=="140602" & year==2012

replace regioncode="140603" if regioncode=="140603" & year==2012

replace regioncode="140621" if regioncode=="140621" & year==2012

replace regioncode="140622" if regioncode=="140622" & year==2012

replace regioncode="140623" if regioncode=="140623" & year==2012

replace regioncode="140624" if regioncode=="140624" & year==2012

replace regioncode="140902" if regioncode=="140902" & year==2012

replace regioncode="140981" if regioncode=="140981" & year==2012

replace regioncode="140921" if regioncode=="140921" & year==2012

replace regioncode="140922" if regioncode=="140922" & year==2012

replace regioncode="140923" if regioncode=="140923" & year==2012

replace regioncode="140924" if regioncode=="140924" & year==2012

replace regioncode="140925" if regioncode=="140925" & year==2012

replace regioncode="140926" if regioncode=="140926" & year==2012

replace regioncode="140927" if regioncode=="140927" & year==2012

replace regioncode="140928" if regioncode=="140928" & year==2012

replace regioncode="140929" if regioncode=="140929" & year==2012

replace regioncode="140930" if regioncode=="140930" & year==2012

replace regioncode="140931" if regioncode=="140931" & year==2012

replace regioncode="140932" if regioncode=="140932" & year==2012

replace regioncode="141181" if regioncode=="141181" & year==2012

replace regioncode="141102" if regioncode=="141102" & year==2012

replace regioncode="141182" if regioncode=="141182" & year==2012

replace regioncode="141121" if regioncode=="141121" & year==2012

replace regioncode="141122" if regioncode=="141122" & year==2012

replace regioncode="141123" if regioncode=="141123" & year==2012

replace regioncode="141124" if regioncode=="141124" & year==2012

replace regioncode="141125" if regioncode=="141125" & year==2012

replace regioncode="141126" if regioncode=="141126" & year==2012

replace regioncode="141127" if regioncode=="141127" & year==2012

replace regioncode="141128" if regioncode=="141128" & year==2012

replace regioncode="141129" if regioncode=="141129" & year==2012

replace regioncode="141130" if regioncode=="141130" & year==2012

replace regioncode="140702" if regioncode=="140702" & year==2012

replace regioncode="140781" if regioncode=="140781" & year==2012

replace regioncode="140721" if regioncode=="140721" & year==2012

replace regioncode="140722" if regioncode=="140722" & year==2012

replace regioncode="140723" if regioncode=="140723" & year==2012

replace regioncode="140724" if regioncode=="140724" & year==2012

replace regioncode="140725" if regioncode=="140725" & year==2012

replace regioncode="140726" if regioncode=="140726" & year==2012

replace regioncode="140727" if regioncode=="140727" & year==2012

replace regioncode="140728" if regioncode=="140728" & year==2012

replace regioncode="140729" if regioncode=="140729" & year==2012

replace regioncode="141002" if regioncode=="141002" & year==2012

replace regioncode="141081" if regioncode=="141081" & year==2012

replace regioncode="141082" if regioncode=="141082" & year==2012

replace regioncode="141021" if regioncode=="141021" & year==2012

replace regioncode="141022" if regioncode=="141022" & year==2012

replace regioncode="141023" if regioncode=="141023" & year==2012

replace regioncode="141024" if regioncode=="141024" & year==2012

replace regioncode="141025" if regioncode=="141025" & year==2012

replace regioncode="141026" if regioncode=="141026" & year==2012

replace regioncode="141027" if regioncode=="141027" & year==2012

replace regioncode="141028" if regioncode=="141028" & year==2012

replace regioncode="141029" if regioncode=="141029" & year==2012

replace regioncode="141033" if regioncode=="141033" & year==2012

replace regioncode="141030" if regioncode=="141030" & year==2012

replace regioncode="141032" if regioncode=="141032" & year==2012

replace regioncode="141031" if regioncode=="141031" & year==2012

replace regioncode="141034" if regioncode=="141034" & year==2012

replace regioncode="140802" if regioncode=="140802" & year==2012

replace regioncode="140881" if regioncode=="140881" & year==2012

replace regioncode="140882" if regioncode=="140882" & year==2012

replace regioncode="140830" if regioncode=="140830" & year==2012

replace regioncode="140821" if regioncode=="140821" & year==2012

replace regioncode="140822" if regioncode=="140822" & year==2012

replace regioncode="140825" if regioncode=="140825" & year==2012

replace regioncode="140824" if regioncode=="140824" & year==2012

replace regioncode="140823" if regioncode=="140823" & year==2012

replace regioncode="140828" if regioncode=="140828" & year==2012

replace regioncode="140826" if regioncode=="140826" & year==2012

replace regioncode="140829" if regioncode=="140829" & year==2012

replace regioncode="140827" if regioncode=="140827" & year==2012

replace regioncode="150102" if regioncode=="150102" & year==2012

replace regioncode="150103" if regioncode=="150103" & year==2012

replace regioncode="150104" if regioncode=="150104" & year==2012

replace regioncode="150105" if regioncode=="150105" & year==2012

replace regioncode="150121" if regioncode=="150121" & year==2012

replace regioncode="150122" if regioncode=="150122" & year==2012

replace regioncode="150123" if regioncode=="150123" & year==2012

replace regioncode="150124" if regioncode=="150124" & year==2012

replace regioncode="150125" if regioncode=="150125" & year==2012

replace regioncode="150202" if regioncode=="150202" & year==2012

replace regioncode="150203" if regioncode=="150203" & year==2012

replace regioncode="150204" if regioncode=="150204" & year==2012

replace regioncode="150205" if regioncode=="150205" & year==2012

replace regioncode="150206" if regioncode=="150206" & year==2012

replace regioncode="150207" if regioncode=="150207" & year==2012

replace regioncode="150221" if regioncode=="150221" & year==2012

replace regioncode="150222" if regioncode=="150222" & year==2012

replace regioncode="150223" if regioncode=="150223" & year==2012

replace regioncode="150302" if regioncode=="150302" & year==2012

replace regioncode="150303" if regioncode=="150303" & year==2012

replace regioncode="150304" if regioncode=="150304" & year==2012

replace regioncode="150402" if regioncode=="150402" & year==2012

replace regioncode="150403" if regioncode=="150403" & year==2012

replace regioncode="150404" if regioncode=="150404" & year==2012

replace regioncode="150421" if regioncode=="150421" & year==2012

replace regioncode="150422" if regioncode=="150422" & year==2012

replace regioncode="150423" if regioncode=="150423" & year==2012

replace regioncode="150424" if regioncode=="150424" & year==2012

replace regioncode="150425" if regioncode=="150425" & year==2012

replace regioncode="150426" if regioncode=="150426" & year==2012

replace regioncode="150428" if regioncode=="150428" & year==2012

replace regioncode="150429" if regioncode=="150429" & year==2012

replace regioncode="150430" if regioncode=="150430" & year==2012

replace regioncode="150702" if regioncode=="150702" & year==2012

replace regioncode="150781" if regioncode=="150781" & year==2012

replace regioncode="150783" if regioncode=="150783" & year==2012

replace regioncode="150782" if regioncode=="150782" & year==2012

replace regioncode="150785" if regioncode=="150785" & year==2012

replace regioncode="150784" if regioncode=="150784" & year==2012

replace regioncode="150721" if regioncode=="150721" & year==2012

replace regioncode="150722" if regioncode=="150722" & year==2012

replace regioncode="150723" if regioncode=="150723" & year==2012

replace regioncode="150724" if regioncode=="150724" & year==2012

replace regioncode="150727" if regioncode=="150727" & year==2012

replace regioncode="150726" if regioncode=="150726" & year==2012

replace regioncode="150725" if regioncode=="150725" & year==2012

replace regioncode="152201" if regioncode=="152201" & year==2012

replace regioncode="152202" if regioncode=="152202" & year==2012

replace regioncode="152221" if regioncode=="152221" & year==2012

replace regioncode="152222" if regioncode=="152222" & year==2012

replace regioncode="152223" if regioncode=="152223" & year==2012

replace regioncode="152224" if regioncode=="152224" & year==2012

replace regioncode="150502" if regioncode=="150502" & year==2012

replace regioncode="150581" if regioncode=="150581" & year==2012

replace regioncode="150521" if regioncode=="150521" & year==2012

replace regioncode="150522" if regioncode=="150522" & year==2012

replace regioncode="150523" if regioncode=="150523" & year==2012

replace regioncode="150524" if regioncode=="150524" & year==2012

replace regioncode="150525" if regioncode=="150525" & year==2012

replace regioncode="150526" if regioncode=="150526" & year==2012

replace regioncode="152501" if regioncode=="152501" & year==2012

replace regioncode="152502" if regioncode=="152502" & year==2012

replace regioncode="152522" if regioncode=="152522" & year==2012

replace regioncode="152523" if regioncode=="152523" & year==2012

replace regioncode="152524" if regioncode=="152524" & year==2012

replace regioncode="152525" if regioncode=="152525" & year==2012

replace regioncode="152526" if regioncode=="152526" & year==2012

replace regioncode="152527" if regioncode=="152527" & year==2012

replace regioncode="152528" if regioncode=="152528" & year==2012

replace regioncode="152529" if regioncode=="152529" & year==2012

replace regioncode="152530" if regioncode=="152530" & year==2012

replace regioncode="152531" if regioncode=="152531" & year==2012

replace regioncode="152601" if regioncode=="150902" & year==2012

replace regioncode="152602" if regioncode=="150981" & year==2012

replace regioncode="152624" if regioncode=="150921" & year==2012

replace regioncode="152625" if regioncode=="150922" & year==2012

replace regioncode="152626" if regioncode=="150923" & year==2012

replace regioncode="152627" if regioncode=="150924" & year==2012

replace regioncode="152629" if regioncode=="150925" & year==2012

replace regioncode="152630" if regioncode=="150926" & year==2012

replace regioncode="152631" if regioncode=="150927" & year==2012

replace regioncode="152632" if regioncode=="150928" & year==2012

replace regioncode="152634" if regioncode=="150929" & year==2012

replace regioncode="150602" if regioncode=="150602" & year==2012

replace regioncode="150621" if regioncode=="150621" & year==2012

replace regioncode="150622" if regioncode=="150622" & year==2012

replace regioncode="150623" if regioncode=="150623" & year==2012

replace regioncode="150624" if regioncode=="150624" & year==2012

replace regioncode="150625" if regioncode=="150625" & year==2012

replace regioncode="150626" if regioncode=="150626" & year==2012

replace regioncode="150627" if regioncode=="150627" & year==2012

replace regioncode="152801" if regioncode=="150802" & year==2012

replace regioncode="152822" if regioncode=="150821" & year==2012

replace regioncode="152823" if regioncode=="150822" & year==2012

replace regioncode="152824" if regioncode=="150823" & year==2012

replace regioncode="152825" if regioncode=="150824" & year==2012

replace regioncode="152826" if regioncode=="150825" & year==2012

replace regioncode="152827" if regioncode=="150826" & year==2012

replace regioncode="152921" if regioncode=="152921" & year==2012

replace regioncode="152922" if regioncode=="152922" & year==2012

replace regioncode="152923" if regioncode=="152923" & year==2012

replace regioncode="210102" if regioncode=="210102" & year==2012

replace regioncode="210103" if regioncode=="210103" & year==2012

replace regioncode="210104" if regioncode=="210104" & year==2012

replace regioncode="210105" if regioncode=="210105" & year==2012

replace regioncode="210106" if regioncode=="210106" & year==2012

replace regioncode="210111" if regioncode=="210111" & year==2012

replace regioncode="210112" if regioncode=="210112" & year==2012

replace regioncode="210113" if regioncode=="210113" & year==2012

replace regioncode="210114" if regioncode=="210114" & year==2012

replace regioncode="210122" if regioncode=="210122" & year==2012

replace regioncode="210123" if regioncode=="210123" & year==2012

replace regioncode="210124" if regioncode=="210124" & year==2012

replace regioncode="210181" if regioncode=="210181" & year==2012

replace regioncode="210202" if regioncode=="210202" & year==2012

replace regioncode="210203" if regioncode=="210203" & year==2012

replace regioncode="210204" if regioncode=="210204" & year==2012

replace regioncode="210211" if regioncode=="210211" & year==2012

replace regioncode="210212" if regioncode=="210212" & year==2012

replace regioncode="210213" if regioncode=="210213" & year==2012

replace regioncode="210224" if regioncode=="210224" & year==2012

replace regioncode="210281" if regioncode=="210281" & year==2012

replace regioncode="210282" if regioncode=="210282" & year==2012

replace regioncode="210283" if regioncode=="210283" & year==2012

replace regioncode="210302" if regioncode=="210302" & year==2012

replace regioncode="210303" if regioncode=="210303" & year==2012

replace regioncode="210304" if regioncode=="210304" & year==2012

replace regioncode="210311" if regioncode=="210311" & year==2012

replace regioncode="210321" if regioncode=="210321" & year==2012

replace regioncode="210323" if regioncode=="210323" & year==2012

replace regioncode="210381" if regioncode=="210381" & year==2012

replace regioncode="210402" if regioncode=="210402" & year==2012

replace regioncode="210403" if regioncode=="210403" & year==2012

replace regioncode="210404" if regioncode=="210404" & year==2012

replace regioncode="210411" if regioncode=="210411" & year==2012

replace regioncode="210421" if regioncode=="210421" & year==2012

replace regioncode="210422" if regioncode=="210422" & year==2012

replace regioncode="210423" if regioncode=="210423" & year==2012

replace regioncode="210502" if regioncode=="210502" & year==2012

replace regioncode="210503" if regioncode=="210503" & year==2012

replace regioncode="210504" if regioncode=="210504" & year==2012

replace regioncode="210505" if regioncode=="210505" & year==2012

replace regioncode="210521" if regioncode=="210521" & year==2012

replace regioncode="210522" if regioncode=="210522" & year==2012

replace regioncode="210602" if regioncode=="210602" & year==2012

replace regioncode="210603" if regioncode=="210603" & year==2012

replace regioncode="210604" if regioncode=="210604" & year==2012

replace regioncode="210624" if regioncode=="210624" & year==2012

replace regioncode="210681" if regioncode=="210681" & year==2012

replace regioncode="210682" if regioncode=="210682" & year==2012

replace regioncode="210702" if regioncode=="210702" & year==2012

replace regioncode="210703" if regioncode=="210703" & year==2012

replace regioncode="210711" if regioncode=="210711" & year==2012

replace regioncode="210726" if regioncode=="210726" & year==2012

replace regioncode="210727" if regioncode=="210727" & year==2012

replace regioncode="210781" if regioncode=="210781" & year==2012

replace regioncode="210782" if regioncode=="210782" & year==2012

replace regioncode="210802" if regioncode=="210802" & year==2012

replace regioncode="210803" if regioncode=="210803" & year==2012

replace regioncode="210804" if regioncode=="210804" & year==2012

replace regioncode="210811" if regioncode=="210811" & year==2012

replace regioncode="210881" if regioncode=="210881" & year==2012

replace regioncode="210882" if regioncode=="210882" & year==2012

replace regioncode="210902" if regioncode=="210902" & year==2012

replace regioncode="210903" if regioncode=="210903" & year==2012

replace regioncode="210904" if regioncode=="210904" & year==2012

replace regioncode="210905" if regioncode=="210905" & year==2012

replace regioncode="210911" if regioncode=="210911" & year==2012

replace regioncode="210921" if regioncode=="210921" & year==2012

replace regioncode="210922" if regioncode=="210922" & year==2012

replace regioncode="211002" if regioncode=="211002" & year==2012

replace regioncode="211003" if regioncode=="211003" & year==2012

replace regioncode="211004" if regioncode=="211004" & year==2012

replace regioncode="211005" if regioncode=="211005" & year==2012

replace regioncode="211011" if regioncode=="211011" & year==2012

replace regioncode="211021" if regioncode=="211021" & year==2012

replace regioncode="211081" if regioncode=="211081" & year==2012

replace regioncode="211102" if regioncode=="211102" & year==2012

replace regioncode="211103" if regioncode=="211103" & year==2012

replace regioncode="211121" if regioncode=="211121" & year==2012

replace regioncode="211122" if regioncode=="211122" & year==2012

replace regioncode="211202" if regioncode=="211202" & year==2012

replace regioncode="211204" if regioncode=="211204" & year==2012

replace regioncode="211221" if regioncode=="211221" & year==2012

replace regioncode="211223" if regioncode=="211223" & year==2012

replace regioncode="211224" if regioncode=="211224" & year==2012

replace regioncode="211281" if regioncode=="211281" & year==2012

replace regioncode="211282" if regioncode=="211282" & year==2012

replace regioncode="211302" if regioncode=="211302" & year==2012

replace regioncode="211303" if regioncode=="211303" & year==2012

replace regioncode="211321" if regioncode=="211321" & year==2012

replace regioncode="211322" if regioncode=="211322" & year==2012

replace regioncode="211324" if regioncode=="211324" & year==2012

replace regioncode="211381" if regioncode=="211381" & year==2012

replace regioncode="211382" if regioncode=="211382" & year==2012

replace regioncode="211402" if regioncode=="211402" & year==2012

replace regioncode="211403" if regioncode=="211403" & year==2012

replace regioncode="211404" if regioncode=="211404" & year==2012

replace regioncode="211421" if regioncode=="211421" & year==2012

replace regioncode="211422" if regioncode=="211422" & year==2012

replace regioncode="211481" if regioncode=="211481" & year==2012

replace regioncode="220102" if regioncode=="220102" & year==2012

replace regioncode="220103" if regioncode=="220103" & year==2012

replace regioncode="220104" if regioncode=="220104" & year==2012

replace regioncode="220105" if regioncode=="220105" & year==2012

replace regioncode="220106" if regioncode=="220106" & year==2012

replace regioncode="220112" if regioncode=="220112" & year==2012

replace regioncode="220122" if regioncode=="220122" & year==2012

replace regioncode="220181" if regioncode=="220181" & year==2012

replace regioncode="220182" if regioncode=="220182" & year==2012

replace regioncode="220183" if regioncode=="220183" & year==2012

replace regioncode="220202" if regioncode=="220202" & year==2012

replace regioncode="220203" if regioncode=="220203" & year==2012

replace regioncode="220204" if regioncode=="220204" & year==2012

replace regioncode="220211" if regioncode=="220211" & year==2012

replace regioncode="220221" if regioncode=="220221" & year==2012

replace regioncode="220281" if regioncode=="220281" & year==2012

replace regioncode="220282" if regioncode=="220282" & year==2012

replace regioncode="220283" if regioncode=="220283" & year==2012

replace regioncode="220284" if regioncode=="220284" & year==2012

replace regioncode="220302" if regioncode=="220302" & year==2012

replace regioncode="220303" if regioncode=="220303" & year==2012

replace regioncode="220322" if regioncode=="220322" & year==2012

replace regioncode="220323" if regioncode=="220323" & year==2012

replace regioncode="220381" if regioncode=="220381" & year==2012

replace regioncode="220382" if regioncode=="220382" & year==2012

replace regioncode="220402" if regioncode=="220402" & year==2012

replace regioncode="220403" if regioncode=="220403" & year==2012

replace regioncode="220421" if regioncode=="220421" & year==2012

replace regioncode="220422" if regioncode=="220422" & year==2012

replace regioncode="220502" if regioncode=="220502" & year==2012

replace regioncode="220503" if regioncode=="220503" & year==2012

replace regioncode="220521" if regioncode=="220521" & year==2012

replace regioncode="220523" if regioncode=="220523" & year==2012

replace regioncode="220524" if regioncode=="220524" & year==2012

replace regioncode="220581" if regioncode=="220581" & year==2012

replace regioncode="220582" if regioncode=="220582" & year==2012

replace regioncode="220602" if regioncode=="220602" & year==2012

replace regioncode="220621" if regioncode=="220621" & year==2012

replace regioncode="220622" if regioncode=="220622" & year==2012

replace regioncode="220623" if regioncode=="220623" & year==2012

replace regioncode="220625" if regioncode=="220625" & year==2012

replace regioncode="220681" if regioncode=="220681" & year==2012

replace regioncode="220702" if regioncode=="220702" & year==2012

replace regioncode="220721" if regioncode=="220721" & year==2012

replace regioncode="220722" if regioncode=="220722" & year==2012

replace regioncode="220723" if regioncode=="220723" & year==2012

replace regioncode="220724" if regioncode=="220724" & year==2012

replace regioncode="220802" if regioncode=="220802" & year==2012

replace regioncode="220821" if regioncode=="220821" & year==2012

replace regioncode="220822" if regioncode=="220822" & year==2012

replace regioncode="220881" if regioncode=="220881" & year==2012

replace regioncode="220882" if regioncode=="220882" & year==2012

replace regioncode="222401" if regioncode=="222401" & year==2012

replace regioncode="222402" if regioncode=="222402" & year==2012

replace regioncode="222403" if regioncode=="222403" & year==2012

replace regioncode="222404" if regioncode=="222404" & year==2012

replace regioncode="222405" if regioncode=="222405" & year==2012

replace regioncode="222406" if regioncode=="222406" & year==2012

replace regioncode="222424" if regioncode=="222424" & year==2012

replace regioncode="222426" if regioncode=="222426" & year==2012

replace regioncode="230102" if regioncode=="230102" & year==2012

replace regioncode="230103" if regioncode=="230103" & year==2012

replace regioncode="230104" if regioncode=="230104" & year==2012

replace regioncode="230105" if regioncode=="230105" & year==2012

replace regioncode="230107" if regioncode=="230110" & year==2012

replace regioncode="230108" if regioncode=="230108" & year==2012

replace regioncode="230121" if regioncode=="230111" & year==2012

replace regioncode="230123" if regioncode=="230123" & year==2012

replace regioncode="230124" if regioncode=="230124" & year==2012

replace regioncode="230125" if regioncode=="230125" & year==2012

replace regioncode="230126" if regioncode=="230126" & year==2012

replace regioncode="230127" if regioncode=="230127" & year==2012

replace regioncode="230128" if regioncode=="230128" & year==2012

replace regioncode="230129" if regioncode=="230129" & year==2012

replace regioncode="230181" if regioncode=="230112" & year==2012

replace regioncode="230182" if regioncode=="230182" & year==2012

replace regioncode="230183" if regioncode=="230183" & year==2012

replace regioncode="230184" if regioncode=="230184" & year==2012

replace regioncode="230202" if regioncode=="230202" & year==2012

replace regioncode="230203" if regioncode=="230203" & year==2012

replace regioncode="230204" if regioncode=="230204" & year==2012

replace regioncode="230205" if regioncode=="230205" & year==2012

replace regioncode="230206" if regioncode=="230206" & year==2012

replace regioncode="230207" if regioncode=="230207" & year==2012

replace regioncode="230208" if regioncode=="230208" & year==2012

replace regioncode="230221" if regioncode=="230221" & year==2012

replace regioncode="230223" if regioncode=="230223" & year==2012

replace regioncode="230224" if regioncode=="230224" & year==2012

replace regioncode="230225" if regioncode=="230225" & year==2012

replace regioncode="230227" if regioncode=="230227" & year==2012

replace regioncode="230229" if regioncode=="230229" & year==2012

replace regioncode="230230" if regioncode=="230230" & year==2012

replace regioncode="230231" if regioncode=="230231" & year==2012

replace regioncode="230281" if regioncode=="230281" & year==2012

replace regioncode="230302" if regioncode=="230302" & year==2012

replace regioncode="230303" if regioncode=="230303" & year==2012

replace regioncode="230304" if regioncode=="230304" & year==2012

replace regioncode="230305" if regioncode=="230305" & year==2012

replace regioncode="230306" if regioncode=="230306" & year==2012

replace regioncode="230307" if regioncode=="230307" & year==2012

replace regioncode="230321" if regioncode=="230321" & year==2012

replace regioncode="230381" if regioncode=="230381" & year==2012

replace regioncode="230382" if regioncode=="230382" & year==2012

replace regioncode="230402" if regioncode=="230402" & year==2012

replace regioncode="230403" if regioncode=="230403" & year==2012

replace regioncode="230404" if regioncode=="230404" & year==2012

replace regioncode="230405" if regioncode=="230405" & year==2012

replace regioncode="230406" if regioncode=="230406" & year==2012

replace regioncode="230407" if regioncode=="230407" & year==2012

replace regioncode="230421" if regioncode=="230421" & year==2012

replace regioncode="230422" if regioncode=="230422" & year==2012

replace regioncode="230502" if regioncode=="230502" & year==2012

replace regioncode="230503" if regioncode=="230503" & year==2012

replace regioncode="230505" if regioncode=="230505" & year==2012

replace regioncode="230506" if regioncode=="230506" & year==2012

replace regioncode="230521" if regioncode=="230521" & year==2012

replace regioncode="230522" if regioncode=="230522" & year==2012

replace regioncode="230523" if regioncode=="230523" & year==2012

replace regioncode="230524" if regioncode=="230524" & year==2012

replace regioncode="230602" if regioncode=="230602" & year==2012

replace regioncode="230603" if regioncode=="230603" & year==2012

replace regioncode="230604" if regioncode=="230604" & year==2012

replace regioncode="230605" if regioncode=="230605" & year==2012

replace regioncode="230606" if regioncode=="230606" & year==2012

replace regioncode="230621" if regioncode=="230621" & year==2012

replace regioncode="230622" if regioncode=="230622" & year==2012

replace regioncode="230623" if regioncode=="230623" & year==2012

replace regioncode="230624" if regioncode=="230624" & year==2012

replace regioncode="230702" if regioncode=="230702" & year==2012

replace regioncode="230703" if regioncode=="230703" & year==2012

replace regioncode="230704" if regioncode=="230704" & year==2012

replace regioncode="230705" if regioncode=="230705" & year==2012

replace regioncode="230706" if regioncode=="230706" & year==2012

replace regioncode="230707" if regioncode=="230707" & year==2012

replace regioncode="230708" if regioncode=="230708" & year==2012

replace regioncode="230709" if regioncode=="230709" & year==2012

replace regioncode="230710" if regioncode=="230710" & year==2012

replace regioncode="230711" if regioncode=="230711" & year==2012

replace regioncode="230712" if regioncode=="230712" & year==2012

replace regioncode="230713" if regioncode=="230713" & year==2012

replace regioncode="230714" if regioncode=="230714" & year==2012

replace regioncode="230715" if regioncode=="230715" & year==2012

replace regioncode="230716" if regioncode=="230716" & year==2012

replace regioncode="230722" if regioncode=="230722" & year==2012

replace regioncode="230781" if regioncode=="230781" & year==2012

replace regioncode="230802" if regioncode=="230802" & year==2012

replace regioncode="230803" if regioncode=="230803" & year==2012

replace regioncode="230804" if regioncode=="230804" & year==2012

replace regioncode="230805" if regioncode=="230805" & year==2012

replace regioncode="230811" if regioncode=="230811" & year==2012

replace regioncode="230822" if regioncode=="230822" & year==2012

replace regioncode="230826" if regioncode=="230826" & year==2012

replace regioncode="230828" if regioncode=="230828" & year==2012

replace regioncode="230833" if regioncode=="230833" & year==2012

replace regioncode="230881" if regioncode=="230881" & year==2012

replace regioncode="230882" if regioncode=="230882" & year==2012

replace regioncode="230902" if regioncode=="230902" & year==2012

replace regioncode="230903" if regioncode=="230903" & year==2012

replace regioncode="230904" if regioncode=="230904" & year==2012

replace regioncode="230921" if regioncode=="230921" & year==2012

replace regioncode="231002" if regioncode=="231002" & year==2012

replace regioncode="231003" if regioncode=="231003" & year==2012

replace regioncode="231004" if regioncode=="231004" & year==2012

replace regioncode="231005" if regioncode=="231005" & year==2012

replace regioncode="231024" if regioncode=="231024" & year==2012

replace regioncode="231025" if regioncode=="231025" & year==2012

replace regioncode="231081" if regioncode=="231081" & year==2012

replace regioncode="231083" if regioncode=="231083" & year==2012

replace regioncode="231084" if regioncode=="231084" & year==2012

replace regioncode="231085" if regioncode=="231085" & year==2012

replace regioncode="231102" if regioncode=="231102" & year==2012

replace regioncode="231121" if regioncode=="231121" & year==2012

replace regioncode="231123" if regioncode=="231123" & year==2012

replace regioncode="231124" if regioncode=="231124" & year==2012

replace regioncode="231181" if regioncode=="231181" & year==2012

replace regioncode="231182" if regioncode=="231182" & year==2012

replace regioncode="231202" if regioncode=="231202" & year==2012

replace regioncode="231221" if regioncode=="231221" & year==2012

replace regioncode="231222" if regioncode=="231222" & year==2012

replace regioncode="231223" if regioncode=="231223" & year==2012

replace regioncode="231224" if regioncode=="231224" & year==2012

replace regioncode="231225" if regioncode=="231225" & year==2012

replace regioncode="231226" if regioncode=="231226" & year==2012

replace regioncode="231281" if regioncode=="231281" & year==2012

replace regioncode="231282" if regioncode=="231282" & year==2012

replace regioncode="231283" if regioncode=="231283" & year==2012

replace regioncode="232701" if regioncode=="232701" & year==2012

replace regioncode="232702" if regioncode=="232702" & year==2012

replace regioncode="232703" if regioncode=="232703" & year==2012

replace regioncode="232704" if regioncode=="232704" & year==2012

replace regioncode="232721" if regioncode=="232721" & year==2012

replace regioncode="232722" if regioncode=="232722" & year==2012

replace regioncode="232723" if regioncode=="232723" & year==2012

replace regioncode="310101" if regioncode=="310101" & year==2012

replace regioncode="310103" if regioncode=="310103" & year==2012

replace regioncode="310104" if regioncode=="310104" & year==2012

replace regioncode="310105" if regioncode=="310105" & year==2012

replace regioncode="310106" if regioncode=="310106" & year==2012

replace regioncode="310107" if regioncode=="310107" & year==2012

replace regioncode="310108" if regioncode=="310108" & year==2012

replace regioncode="310109" if regioncode=="310109" & year==2012

replace regioncode="310110" if regioncode=="310110" & year==2012

replace regioncode="310112" if regioncode=="310112" & year==2012

replace regioncode="310113" if regioncode=="310113" & year==2012

replace regioncode="310114" if regioncode=="310114" & year==2012

replace regioncode="310115" if regioncode=="310115" & year==2012

replace regioncode="310116" if regioncode=="310116" & year==2012

replace regioncode="310117" if regioncode=="310117" & year==2012

replace regioncode="310118" if regioncode=="310118" & year==2012

replace regioncode="310119" if regioncode=="310119" & year==2012

replace regioncode="310120" if regioncode=="310120" & year==2012

replace regioncode="310230" if regioncode=="310230" & year==2012

replace regioncode="320102" if regioncode=="320102" & year==2012

replace regioncode="320103" if regioncode=="320103" & year==2012

replace regioncode="320104" if regioncode=="320104" & year==2012

replace regioncode="320105" if regioncode=="320105" & year==2012

replace regioncode="320106" if regioncode=="320106" & year==2012

replace regioncode="320107" if regioncode=="320107" & year==2012

replace regioncode="320111" if regioncode=="320111" & year==2012

replace regioncode="320113" if regioncode=="320113" & year==2012

replace regioncode="320114" if regioncode=="320114" & year==2012

replace regioncode="320115" if regioncode=="320115" & year==2012

replace regioncode="320116" if regioncode=="320116" & year==2012

replace regioncode="320124" if regioncode=="320124" & year==2012

replace regioncode="320125" if regioncode=="320125" & year==2012

replace regioncode="320202" if regioncode=="320202" & year==2012

replace regioncode="320203" if regioncode=="320203" & year==2012

replace regioncode="320204" if regioncode=="320204" & year==2012

replace regioncode="320205" if regioncode=="320205" & year==2012

replace regioncode="320211" if regioncode=="320211" & year==2012

replace regioncode="320281" if regioncode=="320281" & year==2012

replace regioncode="320282" if regioncode=="320282" & year==2012

replace regioncode="320302" if regioncode=="320302" & year==2012

replace regioncode="320303" if regioncode=="320303" & year==2012

replace regioncode="320304" if regioncode=="320304" & year==2012

replace regioncode="320305" if regioncode=="320305" & year==2012

replace regioncode="320311" if regioncode=="320311" & year==2012

replace regioncode="320321" if regioncode=="320321" & year==2012

replace regioncode="320322" if regioncode=="320322" & year==2012

replace regioncode="320323" if regioncode=="320323" & year==2012

replace regioncode="320324" if regioncode=="320324" & year==2012

replace regioncode="320381" if regioncode=="320381" & year==2012

replace regioncode="320382" if regioncode=="320382" & year==2012

replace regioncode="320402" if regioncode=="320402" & year==2012

replace regioncode="320404" if regioncode=="320404" & year==2012

replace regioncode="320405" if regioncode=="320405" & year==2012

replace regioncode="320411" if regioncode=="320411" & year==2012

replace regioncode="320412" if regioncode=="320412" & year==2012

replace regioncode="320481" if regioncode=="320481" & year==2012

replace regioncode="320482" if regioncode=="320482" & year==2012

replace regioncode="320502" if regioncode=="320502" & year==2012

replace regioncode="320503" if regioncode=="320503" & year==2012

replace regioncode="320504" if regioncode=="320504" & year==2012

replace regioncode="320505" if regioncode=="320505" & year==2012

replace regioncode="320581" if regioncode=="320581" & year==2012

replace regioncode="320582" if regioncode=="320582" & year==2012

replace regioncode="320583" if regioncode=="320583" & year==2012

replace regioncode="320584" if regioncode=="320584" & year==2012

replace regioncode="320585" if regioncode=="320585" & year==2012

replace regioncode="320586" if regioncode=="320586" & year==2012

replace regioncode="320602" if regioncode=="320602" & year==2012

replace regioncode="320611" if regioncode=="320611" & year==2012

replace regioncode="320621" if regioncode=="320621" & year==2012

replace regioncode="320623" if regioncode=="320623" & year==2012

replace regioncode="320681" if regioncode=="320681" & year==2012

replace regioncode="320682" if regioncode=="320682" & year==2012

replace regioncode="320683" if regioncode=="320683" & year==2012

replace regioncode="320684" if regioncode=="320684" & year==2012

replace regioncode="320703" if regioncode=="320703" & year==2012

replace regioncode="320705" if regioncode=="320705" & year==2012

replace regioncode="320706" if regioncode=="320706" & year==2012

replace regioncode="320721" if regioncode=="320721" & year==2012

replace regioncode="320722" if regioncode=="320722" & year==2012

replace regioncode="320723" if regioncode=="320723" & year==2012

replace regioncode="320724" if regioncode=="320724" & year==2012

replace regioncode="320802" if regioncode=="320802" & year==2012

replace regioncode="320803" if regioncode=="320803" & year==2012

replace regioncode="320804" if regioncode=="320804" & year==2012

replace regioncode="320811" if regioncode=="320811" & year==2012

replace regioncode="320826" if regioncode=="320826" & year==2012

replace regioncode="320829" if regioncode=="320829" & year==2012

replace regioncode="320830" if regioncode=="320830" & year==2012

replace regioncode="320831" if regioncode=="320831" & year==2012

replace regioncode="320902" if regioncode=="320902" & year==2012

replace regioncode="320903" if regioncode=="320903" & year==2012

replace regioncode="320921" if regioncode=="320921" & year==2012

replace regioncode="320922" if regioncode=="320922" & year==2012

replace regioncode="320923" if regioncode=="320923" & year==2012

replace regioncode="320924" if regioncode=="320924" & year==2012

replace regioncode="320925" if regioncode=="320925" & year==2012

replace regioncode="320981" if regioncode=="320981" & year==2012

replace regioncode="320982" if regioncode=="320982" & year==2012

replace regioncode="321002" if regioncode=="321002" & year==2012

replace regioncode="321003" if regioncode=="321003" & year==2012

replace regioncode="321011" if regioncode=="321011" & year==2012

replace regioncode="321023" if regioncode=="321023" & year==2012

replace regioncode="321081" if regioncode=="321081" & year==2012

replace regioncode="321084" if regioncode=="321084" & year==2012

replace regioncode="321088" if regioncode=="321088" & year==2012

replace regioncode="321102" if regioncode=="321102" & year==2012

replace regioncode="321111" if regioncode=="321111" & year==2012

replace regioncode="321112" if regioncode=="321112" & year==2012

replace regioncode="321181" if regioncode=="321181" & year==2012

replace regioncode="321182" if regioncode=="321182" & year==2012

replace regioncode="321183" if regioncode=="321183" & year==2012

replace regioncode="321202" if regioncode=="321202" & year==2012

replace regioncode="321203" if regioncode=="321203" & year==2012

replace regioncode="321281" if regioncode=="321281" & year==2012

replace regioncode="321282" if regioncode=="321282" & year==2012

replace regioncode="321283" if regioncode=="321283" & year==2012

replace regioncode="321284" if regioncode=="321284" & year==2012

replace regioncode="321302" if regioncode=="321302" & year==2012

replace regioncode="321321" if regioncode=="321311" & year==2012

replace regioncode="321322" if regioncode=="321322" & year==2012

replace regioncode="321323" if regioncode=="321323" & year==2012

replace regioncode="321324" if regioncode=="321324" & year==2012

replace regioncode="330102" if regioncode=="330102" & year==2012

replace regioncode="330103" if regioncode=="330103" & year==2012

replace regioncode="330104" if regioncode=="330104" & year==2012

replace regioncode="330105" if regioncode=="330105" & year==2012

replace regioncode="330106" if regioncode=="330106" & year==2012

replace regioncode="330108" if regioncode=="330108" & year==2012

replace regioncode="330109" if regioncode=="330109" & year==2012

replace regioncode="330110" if regioncode=="330110" & year==2012

replace regioncode="330122" if regioncode=="330122" & year==2012

replace regioncode="330127" if regioncode=="330127" & year==2012

replace regioncode="330182" if regioncode=="330182" & year==2012

replace regioncode="330183" if regioncode=="330183" & year==2012

replace regioncode="330185" if regioncode=="330185" & year==2012

replace regioncode="330203" if regioncode=="330203" & year==2012

replace regioncode="330204" if regioncode=="330204" & year==2012

replace regioncode="330205" if regioncode=="330205" & year==2012

replace regioncode="330206" if regioncode=="330206" & year==2012

replace regioncode="330211" if regioncode=="330211" & year==2012

replace regioncode="330212" if regioncode=="330212" & year==2012

replace regioncode="330225" if regioncode=="330225" & year==2012

replace regioncode="330226" if regioncode=="330226" & year==2012

replace regioncode="330281" if regioncode=="330281" & year==2012

replace regioncode="330282" if regioncode=="330282" & year==2012

replace regioncode="330283" if regioncode=="330283" & year==2012

replace regioncode="330302" if regioncode=="330302" & year==2012

replace regioncode="330303" if regioncode=="330303" & year==2012

replace regioncode="330304" if regioncode=="330304" & year==2012

replace regioncode="330322" if regioncode=="330322" & year==2012

replace regioncode="330324" if regioncode=="330324" & year==2012

replace regioncode="330326" if regioncode=="330326" & year==2012

replace regioncode="330327" if regioncode=="330327" & year==2012

replace regioncode="330328" if regioncode=="330328" & year==2012

replace regioncode="330329" if regioncode=="330329" & year==2012

replace regioncode="330381" if regioncode=="330381" & year==2012

replace regioncode="330382" if regioncode=="330382" & year==2012

replace regioncode="330402" if regioncode=="330402" & year==2012

replace regioncode="330411" if regioncode=="330411" & year==2012

replace regioncode="330421" if regioncode=="330421" & year==2012

replace regioncode="330424" if regioncode=="330424" & year==2012

replace regioncode="330481" if regioncode=="330481" & year==2012

replace regioncode="330482" if regioncode=="330482" & year==2012

replace regioncode="330483" if regioncode=="330483" & year==2012

replace regioncode="330501" if regioncode=="330501" & year==2012

replace regioncode="330521" if regioncode=="330521" & year==2012

replace regioncode="330522" if regioncode=="330522" & year==2012

replace regioncode="330523" if regioncode=="330523" & year==2012

replace regioncode="330602" if regioncode=="330602" & year==2012

replace regioncode="330621" if regioncode=="330621" & year==2012

replace regioncode="330624" if regioncode=="330624" & year==2012

replace regioncode="330681" if regioncode=="330681" & year==2012

replace regioncode="330682" if regioncode=="330682" & year==2012

replace regioncode="330683" if regioncode=="330683" & year==2012

replace regioncode="330702" if regioncode=="330702" & year==2012

replace regioncode="330703" if regioncode=="330703" & year==2012

replace regioncode="330723" if regioncode=="330723" & year==2012

replace regioncode="330726" if regioncode=="330726" & year==2012

replace regioncode="330727" if regioncode=="330727" & year==2012

replace regioncode="330781" if regioncode=="330781" & year==2012

replace regioncode="330782" if regioncode=="330782" & year==2012

replace regioncode="330783" if regioncode=="330783" & year==2012

replace regioncode="330784" if regioncode=="330784" & year==2012

replace regioncode="330802" if regioncode=="330802" & year==2012

replace regioncode="330803" if regioncode=="330803" & year==2012

replace regioncode="330822" if regioncode=="330822" & year==2012

replace regioncode="330824" if regioncode=="330824" & year==2012

replace regioncode="330825" if regioncode=="330825" & year==2012

replace regioncode="330881" if regioncode=="330881" & year==2012

replace regioncode="330902" if regioncode=="330902" & year==2012

replace regioncode="330903" if regioncode=="330903" & year==2012

replace regioncode="330921" if regioncode=="330921" & year==2012

replace regioncode="330922" if regioncode=="330922" & year==2012

replace regioncode="331002" if regioncode=="331002" & year==2012

replace regioncode="331003" if regioncode=="331003" & year==2012

replace regioncode="331004" if regioncode=="331004" & year==2012

replace regioncode="331021" if regioncode=="331021" & year==2012

replace regioncode="331022" if regioncode=="331022" & year==2012

replace regioncode="331023" if regioncode=="331023" & year==2012

replace regioncode="331024" if regioncode=="331024" & year==2012

replace regioncode="331081" if regioncode=="331081" & year==2012

replace regioncode="331082" if regioncode=="331082" & year==2012

replace regioncode="331102" if regioncode=="331102" & year==2012

replace regioncode="331121" if regioncode=="331121" & year==2012

replace regioncode="331122" if regioncode=="331122" & year==2012

replace regioncode="331123" if regioncode=="331123" & year==2012

replace regioncode="331124" if regioncode=="331124" & year==2012

replace regioncode="331125" if regioncode=="331125" & year==2012

replace regioncode="331126" if regioncode=="331126" & year==2012

replace regioncode="331127" if regioncode=="331127" & year==2012

replace regioncode="331181" if regioncode=="331181" & year==2012

replace regioncode="340102" if regioncode=="340102" & year==2012

replace regioncode="340103" if regioncode=="340103" & year==2012

replace regioncode="340104" if regioncode=="340104" & year==2012

replace regioncode="340111" if regioncode=="340111" & year==2012

replace regioncode="340121" if regioncode=="340121" & year==2012

replace regioncode="340122" if regioncode=="340122" & year==2012

replace regioncode="340123" if regioncode=="340123" & year==2012

replace regioncode="340202" if regioncode=="340202" & year==2012

replace regioncode="340203" if regioncode=="340203" & year==2012

replace regioncode="340204" if regioncode=="340204" & year==2012

replace regioncode="340207" if regioncode=="340207" & year==2012

replace regioncode="340221" if regioncode=="340221" & year==2012

replace regioncode="340222" if regioncode=="340222" & year==2012

replace regioncode="340223" if regioncode=="340223" & year==2012

replace regioncode="340302" if regioncode=="340302" & year==2012

replace regioncode="340303" if regioncode=="340303" & year==2012

replace regioncode="340304" if regioncode=="340304" & year==2012

replace regioncode="340311" if regioncode=="340311" & year==2012

replace regioncode="340321" if regioncode=="340321" & year==2012

replace regioncode="340322" if regioncode=="340322" & year==2012

replace regioncode="340323" if regioncode=="340323" & year==2012

replace regioncode="340402" if regioncode=="340402" & year==2012

replace regioncode="340403" if regioncode=="340403" & year==2012

replace regioncode="340404" if regioncode=="340404" & year==2012

replace regioncode="340405" if regioncode=="340405" & year==2012

replace regioncode="340406" if regioncode=="340406" & year==2012

replace regioncode="340421" if regioncode=="340421" & year==2012

replace regioncode="340502" if regioncode=="340502" & year==2012

replace regioncode="340503" if regioncode=="340503" & year==2012

replace regioncode="340504" if regioncode=="340504" & year==2012

replace regioncode="340521" if regioncode=="340521" & year==2012

replace regioncode="340602" if regioncode=="340602" & year==2012

replace regioncode="340603" if regioncode=="340603" & year==2012

replace regioncode="340604" if regioncode=="340604" & year==2012

replace regioncode="340621" if regioncode=="340621" & year==2012

replace regioncode="340702" if regioncode=="340702" & year==2012

replace regioncode="340703" if regioncode=="340703" & year==2012

replace regioncode="340711" if regioncode=="340711" & year==2012

replace regioncode="340721" if regioncode=="340721" & year==2012

replace regioncode="340802" if regioncode=="340802" & year==2012

replace regioncode="340803" if regioncode=="340803" & year==2012

replace regioncode="340811" if regioncode=="340811" & year==2012

replace regioncode="340822" if regioncode=="340822" & year==2012

replace regioncode="340823" if regioncode=="340823" & year==2012

replace regioncode="340824" if regioncode=="340824" & year==2012

replace regioncode="340825" if regioncode=="340825" & year==2012

replace regioncode="340826" if regioncode=="340826" & year==2012

replace regioncode="340827" if regioncode=="340827" & year==2012

replace regioncode="340828" if regioncode=="340828" & year==2012

replace regioncode="340881" if regioncode=="340881" & year==2012

replace regioncode="341002" if regioncode=="341002" & year==2012

replace regioncode="341003" if regioncode=="341003" & year==2012

replace regioncode="341004" if regioncode=="341004" & year==2012

replace regioncode="341021" if regioncode=="341021" & year==2012

replace regioncode="341022" if regioncode=="341022" & year==2012

replace regioncode="341023" if regioncode=="341023" & year==2012

replace regioncode="341024" if regioncode=="341024" & year==2012

replace regioncode="341102" if regioncode=="341102" & year==2012

replace regioncode="341103" if regioncode=="341103" & year==2012

replace regioncode="341122" if regioncode=="341122" & year==2012

replace regioncode="341124" if regioncode=="341124" & year==2012

replace regioncode="341125" if regioncode=="341125" & year==2012

replace regioncode="341126" if regioncode=="341126" & year==2012

replace regioncode="341181" if regioncode=="341181" & year==2012

replace regioncode="341182" if regioncode=="341182" & year==2012

replace regioncode="341202" if regioncode=="341202" & year==2012

replace regioncode="341203" if regioncode=="341203" & year==2012

replace regioncode="341204" if regioncode=="341204" & year==2012

replace regioncode="341221" if regioncode=="341221" & year==2012

replace regioncode="341222" if regioncode=="341222" & year==2012

replace regioncode="341621" if regioncode=="341621" & year==2012

replace regioncode="341622" if regioncode=="341622" & year==2012

replace regioncode="341225" if regioncode=="341225" & year==2012

replace regioncode="341226" if regioncode=="341226" & year==2012

replace regioncode="341623" if regioncode=="341623" & year==2012

replace regioncode="341282" if regioncode=="341282" & year==2012

replace regioncode="341302" if regioncode=="341302" & year==2012

replace regioncode="341321" if regioncode=="341321" & year==2012

replace regioncode="341322" if regioncode=="341322" & year==2012

replace regioncode="341323" if regioncode=="341323" & year==2012

replace regioncode="341324" if regioncode=="341324" & year==2012

replace regioncode="341402" if regioncode=="341402" & year==2012

replace regioncode="341421" if regioncode=="341421" & year==2012

replace regioncode="341422" if regioncode=="341422" & year==2012

replace regioncode="341423" if regioncode=="341423" & year==2012

replace regioncode="341424" if regioncode=="341424" & year==2012

replace regioncode="341521" if regioncode=="341521" & year==2012

replace regioncode="341522" if regioncode=="341522" & year==2012

replace regioncode="341523" if regioncode=="341523" & year==2012

replace regioncode="341524" if regioncode=="341524" & year==2012

replace regioncode="341525" if regioncode=="341525" & year==2012

replace regioncode="341802" if regioncode=="341802" & year==2012

replace regioncode="341881" if regioncode=="341881" & year==2012

replace regioncode="341821" if regioncode=="341821" & year==2012

replace regioncode="341822" if regioncode=="341822" & year==2012

replace regioncode="341823" if regioncode=="341823" & year==2012

replace regioncode="341825" if regioncode=="341825" & year==2012

replace regioncode="341824" if regioncode=="341824" & year==2012

replace regioncode="341702" if regioncode=="341702" & year==2012

replace regioncode="341721" if regioncode=="341721" & year==2012

replace regioncode="341722" if regioncode=="341722" & year==2012

replace regioncode="341723" if regioncode=="341723" & year==2012

replace regioncode="341502" if regioncode=="341502" & year==2012

replace regioncode="341602" if regioncode=="341602" & year==2012

replace regioncode="350102" if regioncode=="350102" & year==2012

replace regioncode="350103" if regioncode=="350103" & year==2012

replace regioncode="350104" if regioncode=="350104" & year==2012

replace regioncode="350105" if regioncode=="350105" & year==2012

replace regioncode="350111" if regioncode=="350111" & year==2012

replace regioncode="350121" if regioncode=="350121" & year==2012

replace regioncode="350122" if regioncode=="350122" & year==2012

replace regioncode="350123" if regioncode=="350123" & year==2012

replace regioncode="350124" if regioncode=="350124" & year==2012

replace regioncode="350125" if regioncode=="350125" & year==2012

replace regioncode="350128" if regioncode=="350128" & year==2012

replace regioncode="350181" if regioncode=="350181" & year==2012

replace regioncode="350182" if regioncode=="350182" & year==2012

replace regioncode="350203" if regioncode=="350203" & year==2012

replace regioncode="350205" if regioncode=="350205" & year==2012

replace regioncode="350206" if regioncode=="350206" & year==2012

replace regioncode="350211" if regioncode=="350211" & year==2012

replace regioncode="350212" if regioncode=="350212" & year==2012

replace regioncode="350213" if regioncode=="350213" & year==2012

replace regioncode="350302" if regioncode=="350302" & year==2012

replace regioncode="350303" if regioncode=="350303" & year==2012

replace regioncode="350321" if regioncode=="350321" & year==2012

replace regioncode="350322" if regioncode=="350322" & year==2012

replace regioncode="350402" if regioncode=="350402" & year==2012

replace regioncode="350403" if regioncode=="350403" & year==2012

replace regioncode="350421" if regioncode=="350421" & year==2012

replace regioncode="350423" if regioncode=="350423" & year==2012

replace regioncode="350424" if regioncode=="350424" & year==2012

replace regioncode="350425" if regioncode=="350425" & year==2012

replace regioncode="350426" if regioncode=="350426" & year==2012

replace regioncode="350427" if regioncode=="350427" & year==2012

replace regioncode="350428" if regioncode=="350428" & year==2012

replace regioncode="350429" if regioncode=="350429" & year==2012

replace regioncode="350430" if regioncode=="350430" & year==2012

replace regioncode="350481" if regioncode=="350481" & year==2012

replace regioncode="350502" if regioncode=="350502" & year==2012

replace regioncode="350503" if regioncode=="350503" & year==2012

replace regioncode="350504" if regioncode=="350504" & year==2012

replace regioncode="350505" if regioncode=="350505" & year==2012

replace regioncode="350521" if regioncode=="350521" & year==2012

replace regioncode="350524" if regioncode=="350524" & year==2012

replace regioncode="350525" if regioncode=="350525" & year==2012

replace regioncode="350526" if regioncode=="350526" & year==2012

replace regioncode="350581" if regioncode=="350581" & year==2012

replace regioncode="350582" if regioncode=="350582" & year==2012

replace regioncode="350583" if regioncode=="350583" & year==2012

replace regioncode="350602" if regioncode=="350602" & year==2012

replace regioncode="350603" if regioncode=="350603" & year==2012

replace regioncode="350622" if regioncode=="350622" & year==2012

replace regioncode="350623" if regioncode=="350623" & year==2012

replace regioncode="350624" if regioncode=="350624" & year==2012

replace regioncode="350625" if regioncode=="350625" & year==2012

replace regioncode="350626" if regioncode=="350626" & year==2012

replace regioncode="350627" if regioncode=="350627" & year==2012

replace regioncode="350628" if regioncode=="350628" & year==2012

replace regioncode="350629" if regioncode=="350629" & year==2012

replace regioncode="350681" if regioncode=="350681" & year==2012

replace regioncode="350702" if regioncode=="350702" & year==2012

replace regioncode="350721" if regioncode=="350721" & year==2012

replace regioncode="350722" if regioncode=="350722" & year==2012

replace regioncode="350723" if regioncode=="350723" & year==2012

replace regioncode="350724" if regioncode=="350724" & year==2012

replace regioncode="350725" if regioncode=="350725" & year==2012

replace regioncode="350781" if regioncode=="350781" & year==2012

replace regioncode="350782" if regioncode=="350782" & year==2012

replace regioncode="350783" if regioncode=="350783" & year==2012

replace regioncode="350784" if regioncode=="350784" & year==2012

replace regioncode="350802" if regioncode=="350802" & year==2012

replace regioncode="350821" if regioncode=="350821" & year==2012

replace regioncode="350822" if regioncode=="350822" & year==2012

replace regioncode="350823" if regioncode=="350823" & year==2012

replace regioncode="350824" if regioncode=="350824" & year==2012

replace regioncode="350825" if regioncode=="350825" & year==2012

replace regioncode="350881" if regioncode=="350881" & year==2012

replace regioncode="350902" if regioncode=="350902" & year==2012

replace regioncode="350981" if regioncode=="350981" & year==2012

replace regioncode="350982" if regioncode=="350982" & year==2012

replace regioncode="350921" if regioncode=="350921" & year==2012

replace regioncode="350922" if regioncode=="350922" & year==2012

replace regioncode="350923" if regioncode=="350923" & year==2012

replace regioncode="350924" if regioncode=="350924" & year==2012

replace regioncode="350925" if regioncode=="350925" & year==2012

replace regioncode="350926" if regioncode=="350926" & year==2012

replace regioncode="360102" if regioncode=="360102" & year==2012

replace regioncode="360103" if regioncode=="360103" & year==2012

replace regioncode="360104" if regioncode=="360104" & year==2012

replace regioncode="360105" if regioncode=="360105" & year==2012

replace regioncode="360111" if regioncode=="360111" & year==2012

replace regioncode="360121" if regioncode=="360121" & year==2012

replace regioncode="360122" if regioncode=="360122" & year==2012

replace regioncode="360123" if regioncode=="360123" & year==2012

replace regioncode="360124" if regioncode=="360124" & year==2012

replace regioncode="360202" if regioncode=="360202" & year==2012

replace regioncode="360203" if regioncode=="360203" & year==2012

replace regioncode="360222" if regioncode=="360222" & year==2012

replace regioncode="360281" if regioncode=="360281" & year==2012

replace regioncode="360302" if regioncode=="360302" & year==2012

replace regioncode="360313" if regioncode=="360313" & year==2012

replace regioncode="360321" if regioncode=="360321" & year==2012

replace regioncode="360322" if regioncode=="360322" & year==2012

replace regioncode="360323" if regioncode=="360323" & year==2012

replace regioncode="360402" if regioncode=="360402" & year==2012

replace regioncode="360403" if regioncode=="360403" & year==2012

replace regioncode="360421" if regioncode=="360421" & year==2012

replace regioncode="360423" if regioncode=="360423" & year==2012

replace regioncode="360424" if regioncode=="360424" & year==2012

replace regioncode="360425" if regioncode=="360425" & year==2012

replace regioncode="360426" if regioncode=="360426" & year==2012

replace regioncode="360427" if regioncode=="360427" & year==2012

replace regioncode="360428" if regioncode=="360428" & year==2012

replace regioncode="360429" if regioncode=="360429" & year==2012

replace regioncode="360430" if regioncode=="360430" & year==2012

replace regioncode="360481" if regioncode=="360481" & year==2012

replace regioncode="360502" if regioncode=="360502" & year==2012

replace regioncode="360521" if regioncode=="360521" & year==2012

replace regioncode="360602" if regioncode=="360602" & year==2012

replace regioncode="360622" if regioncode=="360622" & year==2012

replace regioncode="360681" if regioncode=="360681" & year==2012

replace regioncode="360702" if regioncode=="360702" & year==2012

replace regioncode="360721" if regioncode=="360721" & year==2012

replace regioncode="360722" if regioncode=="360722" & year==2012

replace regioncode="360723" if regioncode=="360723" & year==2012

replace regioncode="360724" if regioncode=="360724" & year==2012

replace regioncode="360725" if regioncode=="360725" & year==2012

replace regioncode="360726" if regioncode=="360726" & year==2012

replace regioncode="360727" if regioncode=="360727" & year==2012

replace regioncode="360728" if regioncode=="360728" & year==2012

replace regioncode="360729" if regioncode=="360729" & year==2012

replace regioncode="360730" if regioncode=="360730" & year==2012

replace regioncode="360731" if regioncode=="360731" & year==2012

replace regioncode="360732" if regioncode=="360732" & year==2012

replace regioncode="360733" if regioncode=="360733" & year==2012

replace regioncode="360734" if regioncode=="360734" & year==2012

replace regioncode="360735" if regioncode=="360735" & year==2012

replace regioncode="360781" if regioncode=="360781" & year==2012

replace regioncode="360782" if regioncode=="360782" & year==2012

replace regioncode="360802" if regioncode=="360802" & year==2012

replace regioncode="360821" if regioncode=="360821" & year==2012

replace regioncode="360822" if regioncode=="360822" & year==2012

replace regioncode="360823" if regioncode=="360823" & year==2012

replace regioncode="360824" if regioncode=="360824" & year==2012

replace regioncode="360825" if regioncode=="360825" & year==2012

replace regioncode="360826" if regioncode=="360826" & year==2012

replace regioncode="360827" if regioncode=="360827" & year==2012

replace regioncode="360828" if regioncode=="360828" & year==2012

replace regioncode="360829" if regioncode=="360829" & year==2012

replace regioncode="360830" if regioncode=="360830" & year==2012

replace regioncode="360881" if regioncode=="360881" & year==2012

replace regioncode="360902" if regioncode=="360902" & year==2012

replace regioncode="360921" if regioncode=="360921" & year==2012

replace regioncode="360922" if regioncode=="360922" & year==2012

replace regioncode="360923" if regioncode=="360923" & year==2012

replace regioncode="360924" if regioncode=="360924" & year==2012

replace regioncode="360925" if regioncode=="360925" & year==2012

replace regioncode="360926" if regioncode=="360926" & year==2012

replace regioncode="360981" if regioncode=="360981" & year==2012

replace regioncode="360982" if regioncode=="360982" & year==2012

replace regioncode="360983" if regioncode=="360983" & year==2012

replace regioncode="361002" if regioncode=="361002" & year==2012

replace regioncode="361021" if regioncode=="361021" & year==2012

replace regioncode="361022" if regioncode=="361022" & year==2012

replace regioncode="361023" if regioncode=="361023" & year==2012

replace regioncode="361024" if regioncode=="361024" & year==2012

replace regioncode="361025" if regioncode=="361025" & year==2012

replace regioncode="361026" if regioncode=="361026" & year==2012

replace regioncode="361027" if regioncode=="361027" & year==2012

replace regioncode="361028" if regioncode=="361028" & year==2012

replace regioncode="361029" if regioncode=="361029" & year==2012

replace regioncode="361030" if regioncode=="361030" & year==2012

replace regioncode="361102" if regioncode=="361102" & year==2012

replace regioncode="361121" if regioncode=="361121" & year==2012

replace regioncode="361122" if regioncode=="361122" & year==2012

replace regioncode="361123" if regioncode=="361123" & year==2012

replace regioncode="361124" if regioncode=="361124" & year==2012

replace regioncode="361125" if regioncode=="361125" & year==2012

replace regioncode="361126" if regioncode=="361126" & year==2012

replace regioncode="361127" if regioncode=="361127" & year==2012

replace regioncode="361128" if regioncode=="361128" & year==2012

replace regioncode="361129" if regioncode=="361129" & year==2012

replace regioncode="361130" if regioncode=="361130" & year==2012

replace regioncode="361181" if regioncode=="361181" & year==2012

replace regioncode="370102" if regioncode=="370102" & year==2012

replace regioncode="370103" if regioncode=="370103" & year==2012

replace regioncode="370104" if regioncode=="370104" & year==2012

replace regioncode="370105" if regioncode=="370105" & year==2012

replace regioncode="370112" if regioncode=="370112" & year==2012

replace regioncode="370113" if regioncode=="370113" & year==2012

replace regioncode="370124" if regioncode=="370124" & year==2012

replace regioncode="370125" if regioncode=="370125" & year==2012

replace regioncode="370126" if regioncode=="370126" & year==2012

replace regioncode="370181" if regioncode=="370181" & year==2012

replace regioncode="370202" if regioncode=="370202" & year==2012

replace regioncode="370203" if regioncode=="370203" & year==2012

replace regioncode="370205" if regioncode=="370205" & year==2012

replace regioncode="370211" if regioncode=="370211" & year==2012

replace regioncode="370212" if regioncode=="370212" & year==2012

replace regioncode="370213" if regioncode=="370213" & year==2012

replace regioncode="370214" if regioncode=="370214" & year==2012

replace regioncode="370281" if regioncode=="370281" & year==2012

replace regioncode="370282" if regioncode=="370282" & year==2012

replace regioncode="370283" if regioncode=="370283" & year==2012

replace regioncode="370284" if regioncode=="370284" & year==2012

replace regioncode="370285" if regioncode=="370285" & year==2012

replace regioncode="370302" if regioncode=="370302" & year==2012

replace regioncode="370303" if regioncode=="370303" & year==2012

replace regioncode="370304" if regioncode=="370304" & year==2012

replace regioncode="370305" if regioncode=="370305" & year==2012

replace regioncode="370306" if regioncode=="370306" & year==2012

replace regioncode="370321" if regioncode=="370321" & year==2012

replace regioncode="370322" if regioncode=="370322" & year==2012

replace regioncode="370323" if regioncode=="370323" & year==2012

replace regioncode="370402" if regioncode=="370402" & year==2012

replace regioncode="370403" if regioncode=="370403" & year==2012

replace regioncode="370404" if regioncode=="370404" & year==2012

replace regioncode="370405" if regioncode=="370405" & year==2012

replace regioncode="370406" if regioncode=="370406" & year==2012

replace regioncode="370481" if regioncode=="370481" & year==2012

replace regioncode="370502" if regioncode=="370502" & year==2012

replace regioncode="370503" if regioncode=="370503" & year==2012

replace regioncode="370521" if regioncode=="370521" & year==2012

replace regioncode="370522" if regioncode=="370522" & year==2012

replace regioncode="370523" if regioncode=="370523" & year==2012

replace regioncode="370602" if regioncode=="370602" & year==2012

replace regioncode="370611" if regioncode=="370611" & year==2012

replace regioncode="370612" if regioncode=="370612" & year==2012

replace regioncode="370613" if regioncode=="370613" & year==2012

replace regioncode="370634" if regioncode=="370634" & year==2012

replace regioncode="370681" if regioncode=="370681" & year==2012

replace regioncode="370682" if regioncode=="370682" & year==2012

replace regioncode="370683" if regioncode=="370683" & year==2012

replace regioncode="370684" if regioncode=="370684" & year==2012

replace regioncode="370685" if regioncode=="370685" & year==2012

replace regioncode="370686" if regioncode=="370686" & year==2012

replace regioncode="370687" if regioncode=="370687" & year==2012

replace regioncode="370702" if regioncode=="370702" & year==2012

replace regioncode="370703" if regioncode=="370703" & year==2012

replace regioncode="370704" if regioncode=="370704" & year==2012

replace regioncode="370705" if regioncode=="370705" & year==2012

replace regioncode="370724" if regioncode=="370724" & year==2012

replace regioncode="370725" if regioncode=="370725" & year==2012

replace regioncode="370781" if regioncode=="370781" & year==2012

replace regioncode="370782" if regioncode=="370782" & year==2012

replace regioncode="370783" if regioncode=="370783" & year==2012

replace regioncode="370784" if regioncode=="370784" & year==2012

replace regioncode="370785" if regioncode=="370785" & year==2012

replace regioncode="370786" if regioncode=="370786" & year==2012

replace regioncode="370802" if regioncode=="370802" & year==2012

replace regioncode="370811" if regioncode=="370811" & year==2012

replace regioncode="370826" if regioncode=="370826" & year==2012

replace regioncode="370827" if regioncode=="370827" & year==2012

replace regioncode="370828" if regioncode=="370828" & year==2012

replace regioncode="370829" if regioncode=="370829" & year==2012

replace regioncode="370830" if regioncode=="370830" & year==2012

replace regioncode="370831" if regioncode=="370831" & year==2012

replace regioncode="370832" if regioncode=="370832" & year==2012

replace regioncode="370881" if regioncode=="370881" & year==2012

replace regioncode="370882" if regioncode=="370882" & year==2012

replace regioncode="370883" if regioncode=="370883" & year==2012

replace regioncode="370902" if regioncode=="370902" & year==2012

replace regioncode="370903" if regioncode=="370911" & year==2012

replace regioncode="370921" if regioncode=="370921" & year==2012

replace regioncode="370923" if regioncode=="370923" & year==2012

replace regioncode="370982" if regioncode=="370982" & year==2012

replace regioncode="370983" if regioncode=="370983" & year==2012

replace regioncode="371002" if regioncode=="371002" & year==2012

replace regioncode="371081" if regioncode=="371081" & year==2012

replace regioncode="371082" if regioncode=="371082" & year==2012

replace regioncode="371083" if regioncode=="371083" & year==2012

replace regioncode="371102" if regioncode=="371102" & year==2012

replace regioncode="371121" if regioncode=="371121" & year==2012

replace regioncode="371122" if regioncode=="371122" & year==2012

replace regioncode="371202" if regioncode=="371202" & year==2012

replace regioncode="371203" if regioncode=="371203" & year==2012

replace regioncode="371302" if regioncode=="371302" & year==2012

replace regioncode="371311" if regioncode=="371311" & year==2012

replace regioncode="371312" if regioncode=="371312" & year==2012

replace regioncode="371321" if regioncode=="371321" & year==2012

replace regioncode="371322" if regioncode=="371322" & year==2012

replace regioncode="371323" if regioncode=="371323" & year==2012

replace regioncode="371324" if regioncode=="371324" & year==2012

replace regioncode="371325" if regioncode=="371325" & year==2012

replace regioncode="371326" if regioncode=="371326" & year==2012

replace regioncode="371327" if regioncode=="371327" & year==2012

replace regioncode="371328" if regioncode=="371328" & year==2012

replace regioncode="371329" if regioncode=="371329" & year==2012

replace regioncode="371402" if regioncode=="371402" & year==2012

replace regioncode="371421" if regioncode=="371421" & year==2012

replace regioncode="371422" if regioncode=="371422" & year==2012

replace regioncode="371423" if regioncode=="371423" & year==2012

replace regioncode="371424" if regioncode=="371424" & year==2012

replace regioncode="371425" if regioncode=="371425" & year==2012

replace regioncode="371426" if regioncode=="371426" & year==2012

replace regioncode="371427" if regioncode=="371427" & year==2012

replace regioncode="371428" if regioncode=="371428" & year==2012

replace regioncode="371481" if regioncode=="371481" & year==2012

replace regioncode="371482" if regioncode=="371482" & year==2012

replace regioncode="371502" if regioncode=="371502" & year==2012

replace regioncode="371521" if regioncode=="371521" & year==2012

replace regioncode="371522" if regioncode=="371522" & year==2012

replace regioncode="371523" if regioncode=="371523" & year==2012

replace regioncode="371524" if regioncode=="371524" & year==2012

replace regioncode="371525" if regioncode=="371525" & year==2012

replace regioncode="371526" if regioncode=="371526" & year==2012

replace regioncode="371581" if regioncode=="371581" & year==2012

replace regioncode="371602" if regioncode=="371602" & year==2012

replace regioncode="371621" if regioncode=="371621" & year==2012

replace regioncode="371622" if regioncode=="371622" & year==2012

replace regioncode="371623" if regioncode=="371623" & year==2012

replace regioncode="371624" if regioncode=="371624" & year==2012

replace regioncode="371625" if regioncode=="371625" & year==2012

replace regioncode="371626" if regioncode=="371626" & year==2012

replace regioncode="371702" if regioncode=="371702" & year==2012

replace regioncode="371721" if regioncode=="371721" & year==2012

replace regioncode="371722" if regioncode=="371722" & year==2012

replace regioncode="371723" if regioncode=="371723" & year==2012

replace regioncode="371724" if regioncode=="371724" & year==2012

replace regioncode="371725" if regioncode=="371725" & year==2012

replace regioncode="371726" if regioncode=="371726" & year==2012

replace regioncode="371727" if regioncode=="371727" & year==2012

replace regioncode="371728" if regioncode=="371728" & year==2012

replace regioncode="410102" if regioncode=="410102" & year==2012

replace regioncode="410103" if regioncode=="410103" & year==2012

replace regioncode="410104" if regioncode=="410104" & year==2012

replace regioncode="410105" if regioncode=="410105" & year==2012

replace regioncode="410106" if regioncode=="410106" & year==2012

replace regioncode="410108" if regioncode=="410108" & year==2012

replace regioncode="410122" if regioncode=="410122" & year==2012

replace regioncode="410181" if regioncode=="410181" & year==2012

replace regioncode="410182" if regioncode=="410182" & year==2012

replace regioncode="410183" if regioncode=="410183" & year==2012

replace regioncode="410184" if regioncode=="410184" & year==2012

replace regioncode="410185" if regioncode=="410185" & year==2012

replace regioncode="410202" if regioncode=="410202" & year==2012

replace regioncode="410203" if regioncode=="410203" & year==2012

replace regioncode="410204" if regioncode=="410204" & year==2012

replace regioncode="410205" if regioncode=="410205" & year==2012

replace regioncode="410211" if regioncode=="410211" & year==2012

replace regioncode="410221" if regioncode=="410221" & year==2012

replace regioncode="410222" if regioncode=="410222" & year==2012

replace regioncode="410223" if regioncode=="410223" & year==2012

replace regioncode="410224" if regioncode=="410224" & year==2012

replace regioncode="410225" if regioncode=="410225" & year==2012

replace regioncode="410302" if regioncode=="410302" & year==2012

replace regioncode="410303" if regioncode=="410303" & year==2012

replace regioncode="410304" if regioncode=="410304" & year==2012

replace regioncode="410305" if regioncode=="410305" & year==2012

replace regioncode="410306" if regioncode=="410306" & year==2012

replace regioncode="410307" if regioncode=="410311" & year==2012

replace regioncode="410322" if regioncode=="410322" & year==2012

replace regioncode="410323" if regioncode=="410323" & year==2012

replace regioncode="410324" if regioncode=="410324" & year==2012

replace regioncode="410325" if regioncode=="410325" & year==2012

replace regioncode="410326" if regioncode=="410326" & year==2012

replace regioncode="410327" if regioncode=="410327" & year==2012

replace regioncode="410328" if regioncode=="410328" & year==2012

replace regioncode="410329" if regioncode=="410329" & year==2012

replace regioncode="410381" if regioncode=="410381" & year==2012

replace regioncode="410402" if regioncode=="410402" & year==2012

replace regioncode="410403" if regioncode=="410403" & year==2012

replace regioncode="410404" if regioncode=="410404" & year==2012

replace regioncode="410411" if regioncode=="410411" & year==2012

replace regioncode="410421" if regioncode=="410421" & year==2012

replace regioncode="410422" if regioncode=="410422" & year==2012

replace regioncode="410423" if regioncode=="410423" & year==2012

replace regioncode="410425" if regioncode=="410425" & year==2012

replace regioncode="410481" if regioncode=="410481" & year==2012

replace regioncode="410482" if regioncode=="410482" & year==2012

replace regioncode="410502" if regioncode=="410502" & year==2012

replace regioncode="410503" if regioncode=="410503" & year==2012

replace regioncode="410504" if regioncode=="410505" & year==2012

replace regioncode="410511" if regioncode=="410506" & year==2012

replace regioncode="410522" if regioncode=="410522" & year==2012

replace regioncode="410523" if regioncode=="410523" & year==2012

replace regioncode="410526" if regioncode=="410526" & year==2012

replace regioncode="410527" if regioncode=="410527" & year==2012

replace regioncode="410581" if regioncode=="410581" & year==2012

replace regioncode="410602" if regioncode=="410602" & year==2012

replace regioncode="410603" if regioncode=="410603" & year==2012

replace regioncode="410611" if regioncode=="410611" & year==2012

replace regioncode="410621" if regioncode=="410621" & year==2012

replace regioncode="410622" if regioncode=="410622" & year==2012

replace regioncode="410702" if regioncode=="410702" & year==2012

replace regioncode="410703" if regioncode=="410703" & year==2012

replace regioncode="410704" if regioncode=="410704" & year==2012

replace regioncode="410711" if regioncode=="410711" & year==2012

replace regioncode="410721" if regioncode=="410721" & year==2012

replace regioncode="410724" if regioncode=="410724" & year==2012

replace regioncode="410725" if regioncode=="410725" & year==2012

replace regioncode="410726" if regioncode=="410726" & year==2012

replace regioncode="410727" if regioncode=="410727" & year==2012

replace regioncode="410728" if regioncode=="410728" & year==2012

replace regioncode="410781" if regioncode=="410781" & year==2012

replace regioncode="410782" if regioncode=="410782" & year==2012

replace regioncode="410802" if regioncode=="410802" & year==2012

replace regioncode="410803" if regioncode=="410803" & year==2012

replace regioncode="410804" if regioncode=="410804" & year==2012

replace regioncode="410811" if regioncode=="410811" & year==2012

replace regioncode="410821" if regioncode=="410821" & year==2012

replace regioncode="410822" if regioncode=="410822" & year==2012

replace regioncode="410823" if regioncode=="410823" & year==2012

replace regioncode="410825" if regioncode=="410825" & year==2012

replace regioncode="410881" if regioncode=="410881" & year==2012

replace regioncode="410882" if regioncode=="410882" & year==2012

replace regioncode="410883" if regioncode=="410883" & year==2012

replace regioncode="410902" if regioncode=="410902" & year==2012

replace regioncode="410922" if regioncode=="410922" & year==2012

replace regioncode="410923" if regioncode=="410923" & year==2012

replace regioncode="410926" if regioncode=="410926" & year==2012

replace regioncode="410927" if regioncode=="410927" & year==2012

replace regioncode="410928" if regioncode=="410928" & year==2012

replace regioncode="411002" if regioncode=="411002" & year==2012

replace regioncode="411023" if regioncode=="411023" & year==2012

replace regioncode="411024" if regioncode=="411024" & year==2012

replace regioncode="411025" if regioncode=="411025" & year==2012

replace regioncode="411081" if regioncode=="411081" & year==2012

replace regioncode="411082" if regioncode=="411082" & year==2012

replace regioncode="411102" if regioncode=="411102" & year==2012

replace regioncode="411123" if regioncode=="411103" & year==2012

replace regioncode="411121" if regioncode=="411121" & year==2012

replace regioncode="411122" if regioncode=="411122" & year==2012

replace regioncode="411202" if regioncode=="411202" & year==2012

replace regioncode="411221" if regioncode=="411221" & year==2012

replace regioncode="411222" if regioncode=="411222" & year==2012

replace regioncode="411224" if regioncode=="411224" & year==2012

replace regioncode="411281" if regioncode=="411281" & year==2012

replace regioncode="411282" if regioncode=="411282" & year==2012

replace regioncode="411302" if regioncode=="411302" & year==2012

replace regioncode="411303" if regioncode=="411303" & year==2012

replace regioncode="411321" if regioncode=="411321" & year==2012

replace regioncode="411322" if regioncode=="411322" & year==2012

replace regioncode="411323" if regioncode=="411323" & year==2012

replace regioncode="411324" if regioncode=="411324" & year==2012

replace regioncode="411325" if regioncode=="411325" & year==2012

replace regioncode="411326" if regioncode=="411326" & year==2012

replace regioncode="411327" if regioncode=="411327" & year==2012

replace regioncode="411328" if regioncode=="411328" & year==2012

replace regioncode="411329" if regioncode=="411329" & year==2012

replace regioncode="411330" if regioncode=="411330" & year==2012

replace regioncode="411381" if regioncode=="411381" & year==2012

replace regioncode="411402" if regioncode=="411402" & year==2012

replace regioncode="411403" if regioncode=="411403" & year==2012

replace regioncode="411421" if regioncode=="411421" & year==2012

replace regioncode="411422" if regioncode=="411422" & year==2012

replace regioncode="411423" if regioncode=="411423" & year==2012

replace regioncode="411424" if regioncode=="411424" & year==2012

replace regioncode="411425" if regioncode=="411425" & year==2012

replace regioncode="411426" if regioncode=="411426" & year==2012

replace regioncode="411481" if regioncode=="411481" & year==2012

replace regioncode="411502" if regioncode=="411502" & year==2012

replace regioncode="411503" if regioncode=="411503" & year==2012

replace regioncode="411521" if regioncode=="411521" & year==2012

replace regioncode="411522" if regioncode=="411522" & year==2012

replace regioncode="411523" if regioncode=="411523" & year==2012

replace regioncode="411524" if regioncode=="411524" & year==2012

replace regioncode="411525" if regioncode=="411525" & year==2012

replace regioncode="411526" if regioncode=="411526" & year==2012

replace regioncode="411527" if regioncode=="411527" & year==2012

replace regioncode="411528" if regioncode=="411528" & year==2012

replace regioncode="411602" if regioncode=="411602" & year==2012

replace regioncode="411621" if regioncode=="411621" & year==2012

replace regioncode="411622" if regioncode=="411622" & year==2012

replace regioncode="411623" if regioncode=="411623" & year==2012

replace regioncode="411624" if regioncode=="411624" & year==2012

replace regioncode="411625" if regioncode=="411625" & year==2012

replace regioncode="411626" if regioncode=="411626" & year==2012

replace regioncode="411627" if regioncode=="411627" & year==2012

replace regioncode="411628" if regioncode=="411628" & year==2012

replace regioncode="411681" if regioncode=="411681" & year==2012

replace regioncode="411702" if regioncode=="411702" & year==2012

replace regioncode="411721" if regioncode=="411721" & year==2012

replace regioncode="411722" if regioncode=="411722" & year==2012

replace regioncode="411723" if regioncode=="411723" & year==2012

replace regioncode="411724" if regioncode=="411724" & year==2012

replace regioncode="411725" if regioncode=="411725" & year==2012

replace regioncode="411726" if regioncode=="411726" & year==2012

replace regioncode="411727" if regioncode=="411727" & year==2012

replace regioncode="411728" if regioncode=="411728" & year==2012

replace regioncode="411729" if regioncode=="411729" & year==2012

replace regioncode="420102" if regioncode=="420102" & year==2012

replace regioncode="420103" if regioncode=="420103" & year==2012

replace regioncode="420104" if regioncode=="420104" & year==2012

replace regioncode="420105" if regioncode=="420105" & year==2012

replace regioncode="420106" if regioncode=="420106" & year==2012

replace regioncode="420107" if regioncode=="420107" & year==2012

replace regioncode="420111" if regioncode=="420111" & year==2012

replace regioncode="420112" if regioncode=="420112" & year==2012

replace regioncode="420113" if regioncode=="420113" & year==2012

replace regioncode="420114" if regioncode=="420114" & year==2012

replace regioncode="420115" if regioncode=="420115" & year==2012

replace regioncode="420116" if regioncode=="420116" & year==2012

replace regioncode="420117" if regioncode=="420117" & year==2012

replace regioncode="420202" if regioncode=="420202" & year==2012

replace regioncode="420203" if regioncode=="420203" & year==2012

replace regioncode="420204" if regioncode=="420204" & year==2012

replace regioncode="420205" if regioncode=="420205" & year==2012

replace regioncode="420222" if regioncode=="420222" & year==2012

replace regioncode="420281" if regioncode=="420281" & year==2012

replace regioncode="420302" if regioncode=="420302" & year==2012

replace regioncode="420303" if regioncode=="420303" & year==2012

replace regioncode="420321" if regioncode=="420321" & year==2012

replace regioncode="420322" if regioncode=="420322" & year==2012

replace regioncode="420323" if regioncode=="420323" & year==2012

replace regioncode="420324" if regioncode=="420324" & year==2012

replace regioncode="420325" if regioncode=="420325" & year==2012

replace regioncode="420381" if regioncode=="420381" & year==2012

replace regioncode="420502" if regioncode=="420502" & year==2012

replace regioncode="420503" if regioncode=="420503" & year==2012

replace regioncode="420504" if regioncode=="420504" & year==2012

replace regioncode="420505" if regioncode=="420505" & year==2012

replace regioncode="420506" if regioncode=="420506" & year==2012

replace regioncode="420525" if regioncode=="420525" & year==2012

replace regioncode="420526" if regioncode=="420526" & year==2012

replace regioncode="420527" if regioncode=="420527" & year==2012

replace regioncode="420528" if regioncode=="420528" & year==2012

replace regioncode="420529" if regioncode=="420529" & year==2012

replace regioncode="420581" if regioncode=="420581" & year==2012

replace regioncode="420582" if regioncode=="420582" & year==2012

replace regioncode="420583" if regioncode=="420583" & year==2012

replace regioncode="420602" if regioncode=="420602" & year==2012

replace regioncode="420606" if regioncode=="420606" & year==2012

replace regioncode="420607" if regioncode=="420607" & year==2012

replace regioncode="420624" if regioncode=="420624" & year==2012

replace regioncode="420625" if regioncode=="420625" & year==2012

replace regioncode="420626" if regioncode=="420626" & year==2012

replace regioncode="420682" if regioncode=="420682" & year==2012

replace regioncode="420683" if regioncode=="420683" & year==2012

replace regioncode="420684" if regioncode=="420684" & year==2012

replace regioncode="420702" if regioncode=="420702" & year==2012

replace regioncode="420703" if regioncode=="420703" & year==2012

replace regioncode="420704" if regioncode=="420704" & year==2012

replace regioncode="420802" if regioncode=="420802" & year==2012

replace regioncode="420804" if regioncode=="420804" & year==2012

replace regioncode="420821" if regioncode=="420821" & year==2012

replace regioncode="420822" if regioncode=="420822" & year==2012

replace regioncode="420881" if regioncode=="420881" & year==2012

replace regioncode="420902" if regioncode=="420902" & year==2012

replace regioncode="420921" if regioncode=="420921" & year==2012

replace regioncode="420922" if regioncode=="420922" & year==2012

replace regioncode="420923" if regioncode=="420923" & year==2012

replace regioncode="420981" if regioncode=="420981" & year==2012

replace regioncode="420982" if regioncode=="420982" & year==2012

replace regioncode="421381" if regioncode=="421381" & year==2012

replace regioncode="420984" if regioncode=="420984" & year==2012

replace regioncode="421002" if regioncode=="421002" & year==2012

replace regioncode="421003" if regioncode=="421003" & year==2012

replace regioncode="421022" if regioncode=="421022" & year==2012

replace regioncode="421023" if regioncode=="421023" & year==2012

replace regioncode="421024" if regioncode=="421024" & year==2012

replace regioncode="421081" if regioncode=="421081" & year==2012

replace regioncode="421083" if regioncode=="421083" & year==2012

replace regioncode="421087" if regioncode=="421087" & year==2012

replace regioncode="421102" if regioncode=="421102" & year==2012

replace regioncode="421121" if regioncode=="421121" & year==2012

replace regioncode="421122" if regioncode=="421122" & year==2012

replace regioncode="421123" if regioncode=="421123" & year==2012

replace regioncode="421124" if regioncode=="421124" & year==2012

replace regioncode="421125" if regioncode=="421125" & year==2012

replace regioncode="421126" if regioncode=="421126" & year==2012

replace regioncode="421127" if regioncode=="421127" & year==2012

replace regioncode="421181" if regioncode=="421181" & year==2012

replace regioncode="421182" if regioncode=="421182" & year==2012

replace regioncode="421202" if regioncode=="421202" & year==2012

replace regioncode="421221" if regioncode=="421221" & year==2012

replace regioncode="421222" if regioncode=="421222" & year==2012

replace regioncode="421223" if regioncode=="421223" & year==2012

replace regioncode="421224" if regioncode=="421224" & year==2012

replace regioncode="421281" if regioncode=="421281" & year==2012

replace regioncode="421302" if regioncode=="421302" & year==2012

replace regioncode="422801" if regioncode=="422801" & year==2012

replace regioncode="422802" if regioncode=="422802" & year==2012

replace regioncode="422822" if regioncode=="422822" & year==2012

replace regioncode="422823" if regioncode=="422823" & year==2012

replace regioncode="422825" if regioncode=="422825" & year==2012

replace regioncode="422826" if regioncode=="422826" & year==2012

replace regioncode="422827" if regioncode=="422827" & year==2012

replace regioncode="422828" if regioncode=="422828" & year==2012

replace regioncode="429004" if regioncode=="429004" & year==2012

replace regioncode="429005" if regioncode=="429005" & year==2012

replace regioncode="429006" if regioncode=="429006" & year==2012

replace regioncode="429021" if regioncode=="429021" & year==2012

replace regioncode="430102" if regioncode=="430102" & year==2012

replace regioncode="430103" if regioncode=="430103" & year==2012

replace regioncode="430104" if regioncode=="430104" & year==2012

replace regioncode="430105" if regioncode=="430105" & year==2012

replace regioncode="430111" if regioncode=="430111" & year==2012

replace regioncode="430121" if regioncode=="430121" & year==2012

replace regioncode="430122" if regioncode=="430122" & year==2012

replace regioncode="430124" if regioncode=="430124" & year==2012

replace regioncode="430181" if regioncode=="430181" & year==2012

replace regioncode="430202" if regioncode=="430202" & year==2012

replace regioncode="430203" if regioncode=="430203" & year==2012

replace regioncode="430204" if regioncode=="430204" & year==2012

replace regioncode="430211" if regioncode=="430211" & year==2012

replace regioncode="430221" if regioncode=="430221" & year==2012

replace regioncode="430223" if regioncode=="430223" & year==2012

replace regioncode="430224" if regioncode=="430224" & year==2012

replace regioncode="430225" if regioncode=="430225" & year==2012

replace regioncode="430281" if regioncode=="430281" & year==2012

replace regioncode="430302" if regioncode=="430302" & year==2012

replace regioncode="430304" if regioncode=="430304" & year==2012

replace regioncode="430321" if regioncode=="430321" & year==2012

replace regioncode="430381" if regioncode=="430381" & year==2012

replace regioncode="430382" if regioncode=="430382" & year==2012

replace regioncode="430405" if regioncode=="430405" & year==2012

replace regioncode="430406" if regioncode=="430406" & year==2012

replace regioncode="430407" if regioncode=="430407" & year==2012

replace regioncode="430408" if regioncode=="430408" & year==2012

replace regioncode="430412" if regioncode=="430412" & year==2012

replace regioncode="430421" if regioncode=="430421" & year==2012

replace regioncode="430422" if regioncode=="430422" & year==2012

replace regioncode="430423" if regioncode=="430423" & year==2012

replace regioncode="430424" if regioncode=="430424" & year==2012

replace regioncode="430426" if regioncode=="430426" & year==2012

replace regioncode="430481" if regioncode=="430481" & year==2012

replace regioncode="430482" if regioncode=="430482" & year==2012

replace regioncode="430502" if regioncode=="430502" & year==2012

replace regioncode="430503" if regioncode=="430503" & year==2012

replace regioncode="430511" if regioncode=="430511" & year==2012

replace regioncode="430521" if regioncode=="430521" & year==2012

replace regioncode="430522" if regioncode=="430522" & year==2012

replace regioncode="430523" if regioncode=="430523" & year==2012

replace regioncode="430524" if regioncode=="430524" & year==2012

replace regioncode="430525" if regioncode=="430525" & year==2012

replace regioncode="430527" if regioncode=="430527" & year==2012

replace regioncode="430528" if regioncode=="430528" & year==2012

replace regioncode="430529" if regioncode=="430529" & year==2012

replace regioncode="430581" if regioncode=="430581" & year==2012

replace regioncode="430602" if regioncode=="430602" & year==2012

replace regioncode="430603" if regioncode=="430603" & year==2012

replace regioncode="430611" if regioncode=="430611" & year==2012

replace regioncode="430621" if regioncode=="430621" & year==2012

replace regioncode="430623" if regioncode=="430623" & year==2012

replace regioncode="430624" if regioncode=="430624" & year==2012

replace regioncode="430626" if regioncode=="430626" & year==2012

replace regioncode="430681" if regioncode=="430681" & year==2012

replace regioncode="430682" if regioncode=="430682" & year==2012

replace regioncode="430702" if regioncode=="430702" & year==2012

replace regioncode="430703" if regioncode=="430703" & year==2012

replace regioncode="430721" if regioncode=="430721" & year==2012

replace regioncode="430722" if regioncode=="430722" & year==2012

replace regioncode="430723" if regioncode=="430723" & year==2012

replace regioncode="430724" if regioncode=="430724" & year==2012

replace regioncode="430725" if regioncode=="430725" & year==2012

replace regioncode="430726" if regioncode=="430726" & year==2012

replace regioncode="430781" if regioncode=="430781" & year==2012

replace regioncode="430802" if regioncode=="430802" & year==2012

replace regioncode="430811" if regioncode=="430811" & year==2012

replace regioncode="430821" if regioncode=="430821" & year==2012

replace regioncode="430822" if regioncode=="430822" & year==2012

replace regioncode="430902" if regioncode=="430902" & year==2012

replace regioncode="430903" if regioncode=="430903" & year==2012

replace regioncode="430921" if regioncode=="430921" & year==2012

replace regioncode="430922" if regioncode=="430922" & year==2012

replace regioncode="430923" if regioncode=="430923" & year==2012

replace regioncode="430981" if regioncode=="430981" & year==2012

replace regioncode="431002" if regioncode=="431002" & year==2012

replace regioncode="431003" if regioncode=="431003" & year==2012

replace regioncode="431021" if regioncode=="431021" & year==2012

replace regioncode="431022" if regioncode=="431022" & year==2012

replace regioncode="431023" if regioncode=="431023" & year==2012

replace regioncode="431024" if regioncode=="431024" & year==2012

replace regioncode="431025" if regioncode=="431025" & year==2012

replace regioncode="431026" if regioncode=="431026" & year==2012

replace regioncode="431027" if regioncode=="431027" & year==2012

replace regioncode="431028" if regioncode=="431028" & year==2012

replace regioncode="431081" if regioncode=="431081" & year==2012

replace regioncode="431102" if regioncode=="431102" & year==2012

replace regioncode="431103" if regioncode=="431103" & year==2012

replace regioncode="431121" if regioncode=="431121" & year==2012

replace regioncode="431122" if regioncode=="431122" & year==2012

replace regioncode="431123" if regioncode=="431123" & year==2012

replace regioncode="431124" if regioncode=="431124" & year==2012

replace regioncode="431125" if regioncode=="431125" & year==2012

replace regioncode="431126" if regioncode=="431126" & year==2012

replace regioncode="431127" if regioncode=="431127" & year==2012

replace regioncode="431128" if regioncode=="431128" & year==2012

replace regioncode="431129" if regioncode=="431129" & year==2012

replace regioncode="431202" if regioncode=="431202" & year==2012

replace regioncode="431221" if regioncode=="431221" & year==2012

replace regioncode="431222" if regioncode=="431222" & year==2012

replace regioncode="431223" if regioncode=="431223" & year==2012

replace regioncode="431224" if regioncode=="431224" & year==2012

replace regioncode="431225" if regioncode=="431225" & year==2012

replace regioncode="431226" if regioncode=="431226" & year==2012

replace regioncode="431227" if regioncode=="431227" & year==2012

replace regioncode="431228" if regioncode=="431228" & year==2012

replace regioncode="431229" if regioncode=="431229" & year==2012

replace regioncode="431230" if regioncode=="431230" & year==2012

replace regioncode="431281" if regioncode=="431281" & year==2012

replace regioncode="431302" if regioncode=="431302" & year==2012

replace regioncode="431321" if regioncode=="431321" & year==2012

replace regioncode="431322" if regioncode=="431322" & year==2012

replace regioncode="431381" if regioncode=="431381" & year==2012

replace regioncode="431382" if regioncode=="431382" & year==2012

replace regioncode="433101" if regioncode=="433101" & year==2012

replace regioncode="433122" if regioncode=="433122" & year==2012

replace regioncode="433123" if regioncode=="433123" & year==2012

replace regioncode="433124" if regioncode=="433124" & year==2012

replace regioncode="433125" if regioncode=="433125" & year==2012

replace regioncode="433126" if regioncode=="433126" & year==2012

replace regioncode="433127" if regioncode=="433127" & year==2012

replace regioncode="433130" if regioncode=="433130" & year==2012

replace regioncode="440102" if regioncode=="440115" & year==2012

replace regioncode="440103" if regioncode=="440103" & year==2012

replace regioncode="440104" if regioncode=="440104" & year==2012

replace regioncode="440105" if regioncode=="440105" & year==2012

replace regioncode="440106" if regioncode=="440106" & year==2012

replace regioncode="440107" if regioncode=="440116" & year==2012

replace regioncode="440111" if regioncode=="440111" & year==2012

replace regioncode="440112" if regioncode=="440112" & year==2012

replace regioncode="440113" if regioncode=="440113" & year==2012

replace regioncode="440114" if regioncode=="440114" & year==2012

replace regioncode="440183" if regioncode=="440183" & year==2012

replace regioncode="440184" if regioncode=="440184" & year==2012

replace regioncode="440203" if regioncode=="440203" & year==2012

replace regioncode="440204" if regioncode=="440204" & year==2012

replace regioncode="440221" if regioncode=="440205" & year==2012

replace regioncode="440222" if regioncode=="440222" & year==2012

replace regioncode="440224" if regioncode=="440224" & year==2012

replace regioncode="440229" if regioncode=="440229" & year==2012

replace regioncode="440232" if regioncode=="440232" & year==2012

replace regioncode="440233" if regioncode=="440233" & year==2012

replace regioncode="440281" if regioncode=="440281" & year==2012

replace regioncode="440282" if regioncode=="440282" & year==2012

replace regioncode="440303" if regioncode=="440303" & year==2012

replace regioncode="440304" if regioncode=="440304" & year==2012

replace regioncode="440305" if regioncode=="440305" & year==2012

replace regioncode="440306" if regioncode=="440306" & year==2012

replace regioncode="440307" if regioncode=="440307" & year==2012

replace regioncode="440308" if regioncode=="440308" & year==2012

replace regioncode="440402" if regioncode=="440402" & year==2012

replace regioncode="440403" if regioncode=="440403" & year==2012

replace regioncode="440404" if regioncode=="440404" & year==2012

replace regioncode="440507" if regioncode=="440507" & year==2012

replace regioncode="440511" if regioncode=="440511" & year==2012

replace regioncode="440512" if regioncode=="440512" & year==2012

replace regioncode="440514" if regioncode=="440514" & year==2012

replace regioncode="440515" if regioncode=="440515" & year==2012

replace regioncode="440523" if regioncode=="440523" & year==2012

replace regioncode="440604" if regioncode=="440604" & year==2012

replace regioncode="440605" if regioncode=="440605" & year==2012

replace regioncode="440606" if regioncode=="440606" & year==2012

replace regioncode="440607" if regioncode=="440607" & year==2012

replace regioncode="440608" if regioncode=="440608" & year==2012

replace regioncode="440703" if regioncode=="440703" & year==2012

replace regioncode="440704" if regioncode=="440704" & year==2012

replace regioncode="440781" if regioncode=="440781" & year==2012

replace regioncode="440705" if regioncode=="440705" & year==2012

replace regioncode="440783" if regioncode=="440783" & year==2012

replace regioncode="440784" if regioncode=="440784" & year==2012

replace regioncode="440785" if regioncode=="440785" & year==2012

replace regioncode="440802" if regioncode=="440802" & year==2012

replace regioncode="440803" if regioncode=="440803" & year==2012

replace regioncode="440804" if regioncode=="440804" & year==2012

replace regioncode="440811" if regioncode=="440811" & year==2012

replace regioncode="440823" if regioncode=="440823" & year==2012

replace regioncode="440825" if regioncode=="440825" & year==2012

replace regioncode="440881" if regioncode=="440881" & year==2012

replace regioncode="440882" if regioncode=="440882" & year==2012

replace regioncode="440883" if regioncode=="440883" & year==2012

replace regioncode="440902" if regioncode=="440902" & year==2012

replace regioncode="440903" if regioncode=="440903" & year==2012

replace regioncode="440923" if regioncode=="440923" & year==2012

replace regioncode="440981" if regioncode=="440981" & year==2012

replace regioncode="440982" if regioncode=="440982" & year==2012

replace regioncode="440983" if regioncode=="440983" & year==2012

replace regioncode="441202" if regioncode=="441202" & year==2012

replace regioncode="441203" if regioncode=="441203" & year==2012

replace regioncode="441223" if regioncode=="441223" & year==2012

replace regioncode="441224" if regioncode=="441224" & year==2012

replace regioncode="441225" if regioncode=="441225" & year==2012

replace regioncode="441226" if regioncode=="441226" & year==2012

replace regioncode="441283" if regioncode=="441283" & year==2012

replace regioncode="441284" if regioncode=="441284" & year==2012

replace regioncode="441302" if regioncode=="441302" & year==2012

replace regioncode="441303" if regioncode=="441303" & year==2012

replace regioncode="441322" if regioncode=="441322" & year==2012

replace regioncode="441323" if regioncode=="441323" & year==2012

replace regioncode="441324" if regioncode=="441324" & year==2012

replace regioncode="441402" if regioncode=="441402" & year==2012

replace regioncode="441421" if regioncode=="441421" & year==2012

replace regioncode="441422" if regioncode=="441422" & year==2012

replace regioncode="441423" if regioncode=="441423" & year==2012

replace regioncode="441424" if regioncode=="441424" & year==2012

replace regioncode="441426" if regioncode=="441426" & year==2012

replace regioncode="441427" if regioncode=="441427" & year==2012

replace regioncode="441481" if regioncode=="441481" & year==2012

replace regioncode="441502" if regioncode=="441502" & year==2012

replace regioncode="441521" if regioncode=="441521" & year==2012

replace regioncode="441523" if regioncode=="441523" & year==2012

replace regioncode="441581" if regioncode=="441581" & year==2012

replace regioncode="441602" if regioncode=="441602" & year==2012

replace regioncode="441621" if regioncode=="441621" & year==2012

replace regioncode="441622" if regioncode=="441622" & year==2012

replace regioncode="441623" if regioncode=="441623" & year==2012

replace regioncode="441624" if regioncode=="441624" & year==2012

replace regioncode="441625" if regioncode=="441625" & year==2012

replace regioncode="441702" if regioncode=="441702" & year==2012

replace regioncode="441721" if regioncode=="441721" & year==2012

replace regioncode="441723" if regioncode=="441723" & year==2012

replace regioncode="441781" if regioncode=="441781" & year==2012

replace regioncode="441802" if regioncode=="441802" & year==2012

replace regioncode="441821" if regioncode=="441821" & year==2012

replace regioncode="441823" if regioncode=="441823" & year==2012

replace regioncode="441825" if regioncode=="441825" & year==2012

replace regioncode="441826" if regioncode=="441826" & year==2012

replace regioncode="441827" if regioncode=="441827" & year==2012

replace regioncode="441881" if regioncode=="441881" & year==2012

replace regioncode="441882" if regioncode=="441882" & year==2012

replace regioncode="441900" if regioncode=="441901" & year==2012

replace regioncode="442000" if regioncode=="442001" & year==2012

replace regioncode="445102" if regioncode=="445102" & year==2012

replace regioncode="445121" if regioncode=="445121" & year==2012

replace regioncode="445122" if regioncode=="445122" & year==2012

replace regioncode="445202" if regioncode=="445202" & year==2012

replace regioncode="445221" if regioncode=="445221" & year==2012

replace regioncode="445222" if regioncode=="445222" & year==2012

replace regioncode="445224" if regioncode=="445224" & year==2012

replace regioncode="445281" if regioncode=="445281" & year==2012

replace regioncode="445302" if regioncode=="445302" & year==2012

replace regioncode="445321" if regioncode=="445321" & year==2012

replace regioncode="445322" if regioncode=="445322" & year==2012

replace regioncode="445323" if regioncode=="445323" & year==2012

replace regioncode="445381" if regioncode=="445381" & year==2012

replace regioncode="450102" if regioncode=="450102" & year==2012

replace regioncode="450103" if regioncode=="450103" & year==2012

replace regioncode="450104" if regioncode=="450107" & year==2012

replace regioncode="450105" if regioncode=="450105" & year==2012

replace regioncode="450106" if regioncode=="450108" & year==2012

replace regioncode="450121" if regioncode=="450109" & year==2012

replace regioncode="450122" if regioncode=="450122" & year==2012

replace regioncode="450123" if regioncode=="450123" & year==2012

replace regioncode="450124" if regioncode=="450124" & year==2012

replace regioncode="450125" if regioncode=="450125" & year==2012

replace regioncode="450126" if regioncode=="450126" & year==2012

replace regioncode="450127" if regioncode=="450127" & year==2012

replace regioncode="450202" if regioncode=="450202" & year==2012

replace regioncode="450203" if regioncode=="450203" & year==2012

replace regioncode="450204" if regioncode=="450204" & year==2012

replace regioncode="450205" if regioncode=="450205" & year==2012

replace regioncode="450221" if regioncode=="450221" & year==2012

replace regioncode="450222" if regioncode=="450222" & year==2012

replace regioncode="450223" if regioncode=="450223" & year==2012

replace regioncode="450224" if regioncode=="450224" & year==2012

replace regioncode="450225" if regioncode=="450225" & year==2012

replace regioncode="450226" if regioncode=="450226" & year==2012

replace regioncode="450302" if regioncode=="450302" & year==2012

replace regioncode="450303" if regioncode=="450303" & year==2012

replace regioncode="450304" if regioncode=="450304" & year==2012

replace regioncode="450305" if regioncode=="450305" & year==2012

replace regioncode="450311" if regioncode=="450311" & year==2012

replace regioncode="450321" if regioncode=="450321" & year==2012

replace regioncode="450322" if regioncode=="450322" & year==2012

replace regioncode="450323" if regioncode=="450323" & year==2012

replace regioncode="450324" if regioncode=="450324" & year==2012

replace regioncode="450325" if regioncode=="450325" & year==2012

replace regioncode="450326" if regioncode=="450326" & year==2012

replace regioncode="450327" if regioncode=="450327" & year==2012

replace regioncode="450328" if regioncode=="450328" & year==2012

replace regioncode="450329" if regioncode=="450329" & year==2012

replace regioncode="450330" if regioncode=="450330" & year==2012

replace regioncode="450331" if regioncode=="450331" & year==2012

replace regioncode="450332" if regioncode=="450332" & year==2012

replace regioncode="450403" if regioncode=="450403" & year==2012

replace regioncode="450404" if regioncode=="450404" & year==2012

replace regioncode="450421" if regioncode=="450421" & year==2012

replace regioncode="450422" if regioncode=="450422" & year==2012

replace regioncode="450423" if regioncode=="450423" & year==2012

replace regioncode="450481" if regioncode=="450481" & year==2012

replace regioncode="450502" if regioncode=="450502" & year==2012

replace regioncode="450503" if regioncode=="450503" & year==2012

replace regioncode="450512" if regioncode=="450512" & year==2012

replace regioncode="450521" if regioncode=="450521" & year==2012

replace regioncode="450602" if regioncode=="450602" & year==2012

replace regioncode="450603" if regioncode=="450603" & year==2012

replace regioncode="450621" if regioncode=="450621" & year==2012

replace regioncode="450681" if regioncode=="450681" & year==2012

replace regioncode="450702" if regioncode=="450702" & year==2012

replace regioncode="450703" if regioncode=="450703" & year==2012

replace regioncode="450721" if regioncode=="450721" & year==2012

replace regioncode="450722" if regioncode=="450722" & year==2012

replace regioncode="450802" if regioncode=="450802" & year==2012

replace regioncode="450803" if regioncode=="450803" & year==2012

replace regioncode="450804" if regioncode=="450804" & year==2012

replace regioncode="450821" if regioncode=="450821" & year==2012

replace regioncode="450881" if regioncode=="450881" & year==2012

replace regioncode="450902" if regioncode=="450902" & year==2012

replace regioncode="450921" if regioncode=="450921" & year==2012

replace regioncode="450922" if regioncode=="450922" & year==2012

replace regioncode="450923" if regioncode=="450923" & year==2012

replace regioncode="450924" if regioncode=="450924" & year==2012

replace regioncode="450981" if regioncode=="450981" & year==2012

replace regioncode="451002" if regioncode=="451002" & year==2012

replace regioncode="451021" if regioncode=="451021" & year==2012

replace regioncode="451022" if regioncode=="451022" & year==2012

replace regioncode="451023" if regioncode=="451023" & year==2012

replace regioncode="451024" if regioncode=="451024" & year==2012

replace regioncode="451025" if regioncode=="451025" & year==2012

replace regioncode="451026" if regioncode=="451026" & year==2012

replace regioncode="451027" if regioncode=="451027" & year==2012

replace regioncode="451028" if regioncode=="451028" & year==2012

replace regioncode="451029" if regioncode=="451029" & year==2012

replace regioncode="451030" if regioncode=="451030" & year==2012

replace regioncode="451031" if regioncode=="451031" & year==2012

replace regioncode="451102" if regioncode=="451102" & year==2012

replace regioncode="451121" if regioncode=="451121" & year==2012

replace regioncode="451122" if regioncode=="451122" & year==2012

replace regioncode="451123" if regioncode=="451123" & year==2012

replace regioncode="451202" if regioncode=="451202" & year==2012

replace regioncode="451221" if regioncode=="451221" & year==2012

replace regioncode="451222" if regioncode=="451222" & year==2012

replace regioncode="451223" if regioncode=="451223" & year==2012

replace regioncode="451224" if regioncode=="451224" & year==2012

replace regioncode="451225" if regioncode=="451225" & year==2012

replace regioncode="451226" if regioncode=="451226" & year==2012

replace regioncode="451227" if regioncode=="451227" & year==2012

replace regioncode="451228" if regioncode=="451228" & year==2012

replace regioncode="451229" if regioncode=="451229" & year==2012

replace regioncode="451281" if regioncode=="451281" & year==2012

replace regioncode="451302" if regioncode=="451302" & year==2012

replace regioncode="451321" if regioncode=="451321" & year==2012

replace regioncode="451322" if regioncode=="451322" & year==2012

replace regioncode="451323" if regioncode=="451323" & year==2012

replace regioncode="451324" if regioncode=="451324" & year==2012

replace regioncode="451381" if regioncode=="451381" & year==2012

replace regioncode="451402" if regioncode=="451402" & year==2012

replace regioncode="451421" if regioncode=="451421" & year==2012

replace regioncode="451422" if regioncode=="451422" & year==2012

replace regioncode="451423" if regioncode=="451423" & year==2012

replace regioncode="451424" if regioncode=="451424" & year==2012

replace regioncode="451425" if regioncode=="451425" & year==2012

replace regioncode="451481" if regioncode=="451481" & year==2012

replace regioncode="460105" if regioncode=="460105" & year==2012

replace regioncode="460106" if regioncode=="460106" & year==2012

replace regioncode="460107" if regioncode=="460107" & year==2012

replace regioncode="460108" if regioncode=="460108" & year==2012

replace regioncode="460001" if regioncode=="469001" & year==2012

replace regioncode="460002" if regioncode=="469002" & year==2012

replace regioncode="460003" if regioncode=="469003" & year==2012

replace regioncode="460005" if regioncode=="469005" & year==2012

replace regioncode="460006" if regioncode=="469006" & year==2012

replace regioncode="460007" if regioncode=="469007" & year==2012

replace regioncode="460030" if regioncode=="469025" & year==2012

replace regioncode="460031" if regioncode=="469026" & year==2012

replace regioncode="460033" if regioncode=="469027" & year==2012

replace regioncode="460034" if regioncode=="469028" & year==2012

replace regioncode="460036" if regioncode=="469030" & year==2012

replace regioncode="500101" if regioncode=="500101" & year==2012

replace regioncode="500102" if regioncode=="500102" & year==2012

replace regioncode="500103" if regioncode=="500103" & year==2012

replace regioncode="500104" if regioncode=="500104" & year==2012

replace regioncode="500105" if regioncode=="500105" & year==2012

replace regioncode="500106" if regioncode=="500106" & year==2012

replace regioncode="500107" if regioncode=="500107" & year==2012

replace regioncode="500108" if regioncode=="500108" & year==2012

replace regioncode="500109" if regioncode=="500109" & year==2012

replace regioncode="500110" if regioncode=="500110" & year==2012

replace regioncode="500111" if regioncode=="500111" & year==2012

replace regioncode="500112" if regioncode=="500112" & year==2012

replace regioncode="500113" if regioncode=="500113" & year==2012

replace regioncode="500114" if regioncode=="500114" & year==2012

replace regioncode="500115" if regioncode=="500115" & year==2012

replace regioncode="500381" if regioncode=="500116" & year==2012

replace regioncode="500382" if regioncode=="500117" & year==2012

replace regioncode="500383" if regioncode=="500118" & year==2012

replace regioncode="500384" if regioncode=="500119" & year==2012

replace regioncode="500222" if regioncode=="500222" & year==2012

replace regioncode="500223" if regioncode=="500223" & year==2012

replace regioncode="500224" if regioncode=="500224" & year==2012

replace regioncode="500225" if regioncode=="500225" & year==2012

replace regioncode="500226" if regioncode=="500226" & year==2012

replace regioncode="500227" if regioncode=="500227" & year==2012

replace regioncode="500228" if regioncode=="500228" & year==2012

replace regioncode="500229" if regioncode=="500229" & year==2012

replace regioncode="500230" if regioncode=="500230" & year==2012

replace regioncode="500231" if regioncode=="500231" & year==2012

replace regioncode="500232" if regioncode=="500232" & year==2012

replace regioncode="500233" if regioncode=="500233" & year==2012

replace regioncode="500234" if regioncode=="500234" & year==2012

replace regioncode="500235" if regioncode=="500235" & year==2012

replace regioncode="500236" if regioncode=="500236" & year==2012

replace regioncode="500237" if regioncode=="500237" & year==2012

replace regioncode="500238" if regioncode=="500238" & year==2012

replace regioncode="500240" if regioncode=="500240" & year==2012

replace regioncode="500241" if regioncode=="500241" & year==2012

replace regioncode="500242" if regioncode=="500242" & year==2012

replace regioncode="500243" if regioncode=="500243" & year==2012

replace regioncode="510104" if regioncode=="510104" & year==2012

replace regioncode="510105" if regioncode=="510105" & year==2012

replace regioncode="510106" if regioncode=="510106" & year==2012

replace regioncode="510107" if regioncode=="510107" & year==2012

replace regioncode="510108" if regioncode=="510108" & year==2012

replace regioncode="510112" if regioncode=="510112" & year==2012

replace regioncode="510113" if regioncode=="510113" & year==2012

replace regioncode="510114" if regioncode=="510114" & year==2012

replace regioncode="510115" if regioncode=="510115" & year==2012

replace regioncode="510121" if regioncode=="510121" & year==2012

replace regioncode="510122" if regioncode=="510122" & year==2012

replace regioncode="510124" if regioncode=="510124" & year==2012

replace regioncode="510129" if regioncode=="510129" & year==2012

replace regioncode="510131" if regioncode=="510131" & year==2012

replace regioncode="510132" if regioncode=="510132" & year==2012

replace regioncode="510181" if regioncode=="510181" & year==2012

replace regioncode="510182" if regioncode=="510182" & year==2012

replace regioncode="510183" if regioncode=="510183" & year==2012

replace regioncode="510184" if regioncode=="510184" & year==2012

replace regioncode="510302" if regioncode=="510302" & year==2012

replace regioncode="510303" if regioncode=="510303" & year==2012

replace regioncode="510304" if regioncode=="510304" & year==2012

replace regioncode="510311" if regioncode=="510311" & year==2012

replace regioncode="510321" if regioncode=="510321" & year==2012

replace regioncode="510322" if regioncode=="510322" & year==2012

replace regioncode="510402" if regioncode=="510402" & year==2012

replace regioncode="510403" if regioncode=="510403" & year==2012

replace regioncode="510411" if regioncode=="510411" & year==2012

replace regioncode="510421" if regioncode=="510421" & year==2012

replace regioncode="510422" if regioncode=="510422" & year==2012

replace regioncode="510502" if regioncode=="510502" & year==2012

replace regioncode="510503" if regioncode=="510503" & year==2012

replace regioncode="510504" if regioncode=="510504" & year==2012

replace regioncode="510521" if regioncode=="510521" & year==2012

replace regioncode="510522" if regioncode=="510522" & year==2012

replace regioncode="510524" if regioncode=="510524" & year==2012

replace regioncode="510525" if regioncode=="510525" & year==2012

replace regioncode="510603" if regioncode=="510603" & year==2012

replace regioncode="510623" if regioncode=="510623" & year==2012

replace regioncode="510626" if regioncode=="510626" & year==2012

replace regioncode="510681" if regioncode=="510681" & year==2012

replace regioncode="510682" if regioncode=="510682" & year==2012

replace regioncode="510683" if regioncode=="510683" & year==2012

replace regioncode="510703" if regioncode=="510703" & year==2012

replace regioncode="510704" if regioncode=="510704" & year==2012

replace regioncode="510722" if regioncode=="510722" & year==2012

replace regioncode="510723" if regioncode=="510723" & year==2012

replace regioncode="510724" if regioncode=="510724" & year==2012

replace regioncode="510725" if regioncode=="510725" & year==2012

replace regioncode="510726" if regioncode=="510726" & year==2012

replace regioncode="510727" if regioncode=="510727" & year==2012

replace regioncode="510781" if regioncode=="510781" & year==2012

replace regioncode="510802" if regioncode=="510802" & year==2012

replace regioncode="510811" if regioncode=="510811" & year==2012

replace regioncode="510812" if regioncode=="510812" & year==2012

replace regioncode="510821" if regioncode=="510821" & year==2012

replace regioncode="510822" if regioncode=="510822" & year==2012

replace regioncode="510823" if regioncode=="510823" & year==2012

replace regioncode="510824" if regioncode=="510824" & year==2012

replace regioncode="510902" if regioncode=="510903" & year==2012

replace regioncode="510921" if regioncode=="510921" & year==2012

replace regioncode="510922" if regioncode=="510922" & year==2012

replace regioncode="510923" if regioncode=="510923" & year==2012

replace regioncode="511002" if regioncode=="511002" & year==2012

replace regioncode="511011" if regioncode=="511011" & year==2012

replace regioncode="511024" if regioncode=="511024" & year==2012

replace regioncode="511025" if regioncode=="511025" & year==2012

replace regioncode="511028" if regioncode=="511028" & year==2012

replace regioncode="511102" if regioncode=="511102" & year==2012

replace regioncode="511111" if regioncode=="511111" & year==2012

replace regioncode="511112" if regioncode=="511112" & year==2012

replace regioncode="511113" if regioncode=="511113" & year==2012

replace regioncode="511123" if regioncode=="511123" & year==2012

replace regioncode="511124" if regioncode=="511124" & year==2012

replace regioncode="511126" if regioncode=="511126" & year==2012

replace regioncode="511129" if regioncode=="511129" & year==2012

replace regioncode="511132" if regioncode=="511132" & year==2012

replace regioncode="511133" if regioncode=="511133" & year==2012

replace regioncode="511181" if regioncode=="511181" & year==2012

replace regioncode="511302" if regioncode=="511302" & year==2012

replace regioncode="511303" if regioncode=="511303" & year==2012

replace regioncode="511304" if regioncode=="511304" & year==2012

replace regioncode="511321" if regioncode=="511321" & year==2012

replace regioncode="511322" if regioncode=="511322" & year==2012

replace regioncode="511323" if regioncode=="511323" & year==2012

replace regioncode="511324" if regioncode=="511324" & year==2012

replace regioncode="511325" if regioncode=="511325" & year==2012

replace regioncode="511381" if regioncode=="511381" & year==2012

replace regioncode="511402" if regioncode=="511402" & year==2012

replace regioncode="511421" if regioncode=="511421" & year==2012

replace regioncode="511422" if regioncode=="511422" & year==2012

replace regioncode="511423" if regioncode=="511423" & year==2012

replace regioncode="511424" if regioncode=="511424" & year==2012

replace regioncode="511425" if regioncode=="511425" & year==2012

replace regioncode="511502" if regioncode=="511502" & year==2012

replace regioncode="511521" if regioncode=="511521" & year==2012

replace regioncode="511522" if regioncode=="511522" & year==2012

replace regioncode="511523" if regioncode=="511523" & year==2012

replace regioncode="511524" if regioncode=="511524" & year==2012

replace regioncode="511525" if regioncode=="511525" & year==2012

replace regioncode="511526" if regioncode=="511526" & year==2012

replace regioncode="511527" if regioncode=="511527" & year==2012

replace regioncode="511528" if regioncode=="511528" & year==2012

replace regioncode="511529" if regioncode=="511529" & year==2012

replace regioncode="511602" if regioncode=="511602" & year==2012

replace regioncode="511621" if regioncode=="511621" & year==2012

replace regioncode="511622" if regioncode=="511622" & year==2012

replace regioncode="511623" if regioncode=="511623" & year==2012

replace regioncode="511681" if regioncode=="511681" & year==2012

replace regioncode="511702" if regioncode=="511702" & year==2012

replace regioncode="511721" if regioncode=="511721" & year==2012

replace regioncode="511722" if regioncode=="511722" & year==2012

replace regioncode="511723" if regioncode=="511723" & year==2012

replace regioncode="511724" if regioncode=="511724" & year==2012

replace regioncode="511725" if regioncode=="511725" & year==2012

replace regioncode="511781" if regioncode=="511781" & year==2012

replace regioncode="511802" if regioncode=="511802" & year==2012

replace regioncode="511821" if regioncode=="511821" & year==2012

replace regioncode="511822" if regioncode=="511822" & year==2012

replace regioncode="511823" if regioncode=="511823" & year==2012

replace regioncode="511824" if regioncode=="511824" & year==2012

replace regioncode="511825" if regioncode=="511825" & year==2012

replace regioncode="511826" if regioncode=="511826" & year==2012

replace regioncode="511827" if regioncode=="511827" & year==2012

replace regioncode="511902" if regioncode=="511902" & year==2012

replace regioncode="511921" if regioncode=="511921" & year==2012

replace regioncode="511922" if regioncode=="511922" & year==2012

replace regioncode="511923" if regioncode=="511923" & year==2012

replace regioncode="512002" if regioncode=="512002" & year==2012

replace regioncode="512021" if regioncode=="512021" & year==2012

replace regioncode="512022" if regioncode=="512022" & year==2012

replace regioncode="512081" if regioncode=="512081" & year==2012

replace regioncode="513221" if regioncode=="513221" & year==2012

replace regioncode="513222" if regioncode=="513222" & year==2012

replace regioncode="513223" if regioncode=="513223" & year==2012

replace regioncode="513224" if regioncode=="513224" & year==2012

replace regioncode="513225" if regioncode=="513225" & year==2012

replace regioncode="513226" if regioncode=="513226" & year==2012

replace regioncode="513227" if regioncode=="513227" & year==2012

replace regioncode="513228" if regioncode=="513228" & year==2012

replace regioncode="513229" if regioncode=="513229" & year==2012

replace regioncode="513230" if regioncode=="513230" & year==2012

replace regioncode="513231" if regioncode=="513231" & year==2012

replace regioncode="513232" if regioncode=="513232" & year==2012

replace regioncode="513233" if regioncode=="513233" & year==2012

replace regioncode="513321" if regioncode=="513321" & year==2012

replace regioncode="513322" if regioncode=="513322" & year==2012

replace regioncode="513323" if regioncode=="513323" & year==2012

replace regioncode="513324" if regioncode=="513324" & year==2012

replace regioncode="513325" if regioncode=="513325" & year==2012

replace regioncode="513326" if regioncode=="513326" & year==2012

replace regioncode="513327" if regioncode=="513327" & year==2012

replace regioncode="513328" if regioncode=="513328" & year==2012

replace regioncode="513329" if regioncode=="513329" & year==2012

replace regioncode="513330" if regioncode=="513330" & year==2012

replace regioncode="513331" if regioncode=="513331" & year==2012

replace regioncode="513332" if regioncode=="513332" & year==2012

replace regioncode="513333" if regioncode=="513333" & year==2012

replace regioncode="513334" if regioncode=="513334" & year==2012

replace regioncode="513335" if regioncode=="513335" & year==2012

replace regioncode="513336" if regioncode=="513336" & year==2012

replace regioncode="513337" if regioncode=="513337" & year==2012

replace regioncode="513338" if regioncode=="513338" & year==2012

replace regioncode="513401" if regioncode=="513401" & year==2012

replace regioncode="513422" if regioncode=="513422" & year==2012

replace regioncode="513423" if regioncode=="513423" & year==2012

replace regioncode="513424" if regioncode=="513424" & year==2012

replace regioncode="513425" if regioncode=="513425" & year==2012

replace regioncode="513426" if regioncode=="513426" & year==2012

replace regioncode="513427" if regioncode=="513427" & year==2012

replace regioncode="513428" if regioncode=="513428" & year==2012

replace regioncode="513429" if regioncode=="513429" & year==2012

replace regioncode="513430" if regioncode=="513430" & year==2012

replace regioncode="513431" if regioncode=="513431" & year==2012

replace regioncode="513432" if regioncode=="513432" & year==2012

replace regioncode="513433" if regioncode=="513433" & year==2012

replace regioncode="513434" if regioncode=="513434" & year==2012

replace regioncode="513435" if regioncode=="513435" & year==2012

replace regioncode="513436" if regioncode=="513436" & year==2012

replace regioncode="513437" if regioncode=="513437" & year==2012

replace regioncode="520102" if regioncode=="520102" & year==2012

replace regioncode="520103" if regioncode=="520103" & year==2012

replace regioncode="520111" if regioncode=="520111" & year==2012

replace regioncode="520112" if regioncode=="520112" & year==2012

replace regioncode="520113" if regioncode=="520113" & year==2012

replace regioncode="520114" if regioncode=="520114" & year==2012

replace regioncode="520121" if regioncode=="520121" & year==2012

replace regioncode="520122" if regioncode=="520122" & year==2012

replace regioncode="520123" if regioncode=="520123" & year==2012

replace regioncode="520181" if regioncode=="520181" & year==2012

replace regioncode="520201" if regioncode=="520201" & year==2012

replace regioncode="520222" if regioncode=="520222" & year==2012

replace regioncode="520203" if regioncode=="520203" & year==2012

replace regioncode="520221" if regioncode=="520221" & year==2012

replace regioncode="520302" if regioncode=="520302" & year==2012

replace regioncode="520321" if regioncode=="520321" & year==2012

replace regioncode="520322" if regioncode=="520322" & year==2012

replace regioncode="520323" if regioncode=="520323" & year==2012

replace regioncode="520324" if regioncode=="520324" & year==2012

replace regioncode="520325" if regioncode=="520325" & year==2012

replace regioncode="520326" if regioncode=="520326" & year==2012

replace regioncode="520327" if regioncode=="520327" & year==2012

replace regioncode="520328" if regioncode=="520328" & year==2012

replace regioncode="520329" if regioncode=="520329" & year==2012

replace regioncode="520330" if regioncode=="520330" & year==2012

replace regioncode="520381" if regioncode=="520381" & year==2012

replace regioncode="520382" if regioncode=="520382" & year==2012

replace regioncode="520402" if regioncode=="520402" & year==2012

replace regioncode="520421" if regioncode=="520421" & year==2012

replace regioncode="520422" if regioncode=="520422" & year==2012

replace regioncode="520423" if regioncode=="520423" & year==2012

replace regioncode="520424" if regioncode=="520424" & year==2012

replace regioncode="520425" if regioncode=="520425" & year==2012

replace regioncode="522201" if regioncode=="522201" & year==2012

replace regioncode="522222" if regioncode=="522222" & year==2012

replace regioncode="522223" if regioncode=="522223" & year==2012

replace regioncode="522224" if regioncode=="522224" & year==2012

replace regioncode="522225" if regioncode=="522225" & year==2012

replace regioncode="522226" if regioncode=="522226" & year==2012

replace regioncode="522227" if regioncode=="522227" & year==2012

replace regioncode="522228" if regioncode=="522228" & year==2012

replace regioncode="522229" if regioncode=="522229" & year==2012

replace regioncode="522230" if regioncode=="522230" & year==2012

replace regioncode="522301" if regioncode=="522301" & year==2012

replace regioncode="522322" if regioncode=="522322" & year==2012

replace regioncode="522323" if regioncode=="522323" & year==2012

replace regioncode="522324" if regioncode=="522324" & year==2012

replace regioncode="522325" if regioncode=="522325" & year==2012

replace regioncode="522326" if regioncode=="522326" & year==2012

replace regioncode="522327" if regioncode=="522327" & year==2012

replace regioncode="522328" if regioncode=="522328" & year==2012

replace regioncode="522401" if regioncode=="522401" & year==2012

replace regioncode="522422" if regioncode=="522422" & year==2012

replace regioncode="522423" if regioncode=="522423" & year==2012

replace regioncode="522424" if regioncode=="522424" & year==2012

replace regioncode="522425" if regioncode=="522425" & year==2012

replace regioncode="522426" if regioncode=="522426" & year==2012

replace regioncode="522427" if regioncode=="522427" & year==2012

replace regioncode="522428" if regioncode=="522428" & year==2012

replace regioncode="522601" if regioncode=="522601" & year==2012

replace regioncode="522622" if regioncode=="522622" & year==2012

replace regioncode="522623" if regioncode=="522623" & year==2012

replace regioncode="522624" if regioncode=="522624" & year==2012

replace regioncode="522625" if regioncode=="522625" & year==2012

replace regioncode="522626" if regioncode=="522626" & year==2012

replace regioncode="522627" if regioncode=="522627" & year==2012

replace regioncode="522628" if regioncode=="522628" & year==2012

replace regioncode="522629" if regioncode=="522629" & year==2012

replace regioncode="522630" if regioncode=="522630" & year==2012

replace regioncode="522631" if regioncode=="522631" & year==2012

replace regioncode="522632" if regioncode=="522632" & year==2012

replace regioncode="522633" if regioncode=="522633" & year==2012

replace regioncode="522634" if regioncode=="522634" & year==2012

replace regioncode="522635" if regioncode=="522635" & year==2012

replace regioncode="522636" if regioncode=="522636" & year==2012

replace regioncode="522701" if regioncode=="522701" & year==2012

replace regioncode="522702" if regioncode=="522702" & year==2012

replace regioncode="522722" if regioncode=="522722" & year==2012

replace regioncode="522723" if regioncode=="522723" & year==2012

replace regioncode="522725" if regioncode=="522725" & year==2012

replace regioncode="522726" if regioncode=="522726" & year==2012

replace regioncode="522727" if regioncode=="522727" & year==2012

replace regioncode="522728" if regioncode=="522728" & year==2012

replace regioncode="522729" if regioncode=="522729" & year==2012

replace regioncode="522730" if regioncode=="522730" & year==2012

replace regioncode="522731" if regioncode=="522731" & year==2012

replace regioncode="522732" if regioncode=="522732" & year==2012

replace regioncode="530102" if regioncode=="530102" & year==2012

replace regioncode="530103" if regioncode=="530103" & year==2012

replace regioncode="530111" if regioncode=="530111" & year==2012

replace regioncode="530112" if regioncode=="530112" & year==2012

replace regioncode="530113" if regioncode=="530113" & year==2012

replace regioncode="530121" if regioncode=="530121" & year==2012

replace regioncode="530122" if regioncode=="530122" & year==2012

replace regioncode="530124" if regioncode=="530124" & year==2012

replace regioncode="530125" if regioncode=="530125" & year==2012

replace regioncode="530126" if regioncode=="530126" & year==2012

replace regioncode="530127" if regioncode=="530127" & year==2012

replace regioncode="530128" if regioncode=="530128" & year==2012

replace regioncode="530129" if regioncode=="530129" & year==2012

replace regioncode="530181" if regioncode=="530181" & year==2012

replace regioncode="530302" if regioncode=="530302" & year==2012

replace regioncode="530321" if regioncode=="530321" & year==2012

replace regioncode="530322" if regioncode=="530322" & year==2012

replace regioncode="530323" if regioncode=="530323" & year==2012

replace regioncode="530324" if regioncode=="530324" & year==2012

replace regioncode="530325" if regioncode=="530325" & year==2012

replace regioncode="530326" if regioncode=="530326" & year==2012

replace regioncode="530328" if regioncode=="530328" & year==2012

replace regioncode="530381" if regioncode=="530381" & year==2012

replace regioncode="530402" if regioncode=="530402" & year==2012

replace regioncode="530421" if regioncode=="530421" & year==2012

replace regioncode="530422" if regioncode=="530422" & year==2012

replace regioncode="530423" if regioncode=="530423" & year==2012

replace regioncode="530424" if regioncode=="530424" & year==2012

replace regioncode="530425" if regioncode=="530425" & year==2012

replace regioncode="530426" if regioncode=="530426" & year==2012

replace regioncode="530427" if regioncode=="530427" & year==2012

replace regioncode="530428" if regioncode=="530428" & year==2012

replace regioncode="530502" if regioncode=="530502" & year==2012

replace regioncode="530521" if regioncode=="530521" & year==2012

replace regioncode="530522" if regioncode=="530522" & year==2012

replace regioncode="530523" if regioncode=="530523" & year==2012

replace regioncode="530524" if regioncode=="530524" & year==2012

replace regioncode="530602" if regioncode=="530602" & year==2012

replace regioncode="530621" if regioncode=="530621" & year==2012

replace regioncode="530622" if regioncode=="530622" & year==2012

replace regioncode="530623" if regioncode=="530623" & year==2012

replace regioncode="530624" if regioncode=="530624" & year==2012

replace regioncode="530625" if regioncode=="530625" & year==2012

replace regioncode="530626" if regioncode=="530626" & year==2012

replace regioncode="530627" if regioncode=="530627" & year==2012

replace regioncode="530628" if regioncode=="530628" & year==2012

replace regioncode="530629" if regioncode=="530629" & year==2012

replace regioncode="530630" if regioncode=="530630" & year==2012

replace regioncode="530721" if regioncode=="530721" & year==2012

replace regioncode="530722" if regioncode=="530722" & year==2012

replace regioncode="530723" if regioncode=="530723" & year==2012

replace regioncode="530724" if regioncode=="530724" & year==2012

replace regioncode="532701" if regioncode=="530802" & year==2012

replace regioncode="532722" if regioncode=="530821" & year==2012

replace regioncode="532723" if regioncode=="530822" & year==2012

replace regioncode="532724" if regioncode=="530823" & year==2012

replace regioncode="532725" if regioncode=="530824" & year==2012

replace regioncode="532726" if regioncode=="530825" & year==2012

replace regioncode="532727" if regioncode=="530826" & year==2012

replace regioncode="532728" if regioncode=="530827" & year==2012

replace regioncode="532729" if regioncode=="530828" & year==2012

replace regioncode="532730" if regioncode=="530829" & year==2012

replace regioncode="533521" if regioncode=="530902" & year==2012

replace regioncode="533522" if regioncode=="530921" & year==2012

replace regioncode="533523" if regioncode=="530922" & year==2012

replace regioncode="533524" if regioncode=="530923" & year==2012

replace regioncode="533525" if regioncode=="530924" & year==2012

replace regioncode="533526" if regioncode=="530925" & year==2012

replace regioncode="533527" if regioncode=="530926" & year==2012

replace regioncode="533528" if regioncode=="530927" & year==2012

replace regioncode="532301" if regioncode=="532301" & year==2012

replace regioncode="532322" if regioncode=="532322" & year==2012

replace regioncode="532323" if regioncode=="532323" & year==2012

replace regioncode="532324" if regioncode=="532324" & year==2012

replace regioncode="532325" if regioncode=="532325" & year==2012

replace regioncode="532326" if regioncode=="532326" & year==2012

replace regioncode="532327" if regioncode=="532327" & year==2012

replace regioncode="532328" if regioncode=="532328" & year==2012

replace regioncode="532329" if regioncode=="532329" & year==2012

replace regioncode="532331" if regioncode=="532331" & year==2012

replace regioncode="532501" if regioncode=="532501" & year==2012

replace regioncode="532502" if regioncode=="532502" & year==2012

replace regioncode="532522" if regioncode=="532522" & year==2012

replace regioncode="532523" if regioncode=="532523" & year==2012

replace regioncode="532524" if regioncode=="532524" & year==2012

replace regioncode="532525" if regioncode=="532525" & year==2012

replace regioncode="532526" if regioncode=="532526" & year==2012

replace regioncode="532527" if regioncode=="532527" & year==2012

replace regioncode="532528" if regioncode=="532528" & year==2012

replace regioncode="532529" if regioncode=="532529" & year==2012

replace regioncode="532530" if regioncode=="532530" & year==2012

replace regioncode="532531" if regioncode=="532531" & year==2012

replace regioncode="532532" if regioncode=="532532" & year==2012

replace regioncode="532621" if regioncode=="532621" & year==2012

replace regioncode="532622" if regioncode=="532622" & year==2012

replace regioncode="532623" if regioncode=="532623" & year==2012

replace regioncode="532624" if regioncode=="532624" & year==2012

replace regioncode="532625" if regioncode=="532625" & year==2012

replace regioncode="532626" if regioncode=="532626" & year==2012

replace regioncode="532627" if regioncode=="532627" & year==2012

replace regioncode="532628" if regioncode=="532628" & year==2012

replace regioncode="532801" if regioncode=="532801" & year==2012

replace regioncode="532822" if regioncode=="532822" & year==2012

replace regioncode="532823" if regioncode=="532823" & year==2012

replace regioncode="532901" if regioncode=="532901" & year==2012

replace regioncode="532922" if regioncode=="532922" & year==2012

replace regioncode="532923" if regioncode=="532923" & year==2012

replace regioncode="532924" if regioncode=="532924" & year==2012

replace regioncode="532925" if regioncode=="532925" & year==2012

replace regioncode="532926" if regioncode=="532926" & year==2012

replace regioncode="532927" if regioncode=="532927" & year==2012

replace regioncode="532928" if regioncode=="532928" & year==2012

replace regioncode="532929" if regioncode=="532929" & year==2012

replace regioncode="532930" if regioncode=="532930" & year==2012

replace regioncode="532931" if regioncode=="532931" & year==2012

replace regioncode="532932" if regioncode=="532932" & year==2012

replace regioncode="533102" if regioncode=="533102" & year==2012

replace regioncode="533103" if regioncode=="533103" & year==2012

replace regioncode="533122" if regioncode=="533122" & year==2012

replace regioncode="533123" if regioncode=="533123" & year==2012

replace regioncode="533124" if regioncode=="533124" & year==2012

replace regioncode="533321" if regioncode=="533321" & year==2012

replace regioncode="533323" if regioncode=="533323" & year==2012

replace regioncode="533324" if regioncode=="533324" & year==2012

replace regioncode="533325" if regioncode=="533325" & year==2012

replace regioncode="533421" if regioncode=="533421" & year==2012

replace regioncode="533422" if regioncode=="533422" & year==2012

replace regioncode="533423" if regioncode=="533423" & year==2012

replace regioncode="540102" if regioncode=="540102" & year==2012

replace regioncode="540121" if regioncode=="540121" & year==2012

replace regioncode="540122" if regioncode=="540122" & year==2012

replace regioncode="540123" if regioncode=="540123" & year==2012

replace regioncode="540124" if regioncode=="540124" & year==2012

replace regioncode="540125" if regioncode=="540125" & year==2012

replace regioncode="540126" if regioncode=="540126" & year==2012

replace regioncode="540127" if regioncode=="540127" & year==2012

replace regioncode="542121" if regioncode=="542121" & year==2012

replace regioncode="542122" if regioncode=="542122" & year==2012

replace regioncode="542123" if regioncode=="542123" & year==2012

replace regioncode="542124" if regioncode=="542124" & year==2012

replace regioncode="542125" if regioncode=="542125" & year==2012

replace regioncode="542126" if regioncode=="542126" & year==2012

replace regioncode="542127" if regioncode=="542127" & year==2012

replace regioncode="542128" if regioncode=="542128" & year==2012

replace regioncode="542129" if regioncode=="542129" & year==2012

replace regioncode="542132" if regioncode=="542132" & year==2012

replace regioncode="542133" if regioncode=="542133" & year==2012

replace regioncode="542221" if regioncode=="542221" & year==2012

replace regioncode="542223" if regioncode=="542223" & year==2012

replace regioncode="542224" if regioncode=="542224" & year==2012

replace regioncode="542225" if regioncode=="542225" & year==2012

replace regioncode="542227" if regioncode=="542227" & year==2012

replace regioncode="542228" if regioncode=="542228" & year==2012

replace regioncode="542229" if regioncode=="542229" & year==2012

replace regioncode="542231" if regioncode=="542231" & year==2012

replace regioncode="542232" if regioncode=="542232" & year==2012

replace regioncode="542233" if regioncode=="542233" & year==2012

replace regioncode="542301" if regioncode=="542301" & year==2012

replace regioncode="542322" if regioncode=="542322" & year==2012

replace regioncode="542323" if regioncode=="542323" & year==2012

replace regioncode="542324" if regioncode=="542324" & year==2012

replace regioncode="542325" if regioncode=="542325" & year==2012

replace regioncode="542326" if regioncode=="542326" & year==2012

replace regioncode="542327" if regioncode=="542327" & year==2012

replace regioncode="542328" if regioncode=="542328" & year==2012

replace regioncode="542329" if regioncode=="542329" & year==2012

replace regioncode="542330" if regioncode=="542330" & year==2012

replace regioncode="542331" if regioncode=="542331" & year==2012

replace regioncode="542332" if regioncode=="542332" & year==2012

replace regioncode="542334" if regioncode=="542334" & year==2012

replace regioncode="542336" if regioncode=="542336" & year==2012

replace regioncode="542338" if regioncode=="542338" & year==2012

replace regioncode="542421" if regioncode=="542421" & year==2012

replace regioncode="542422" if regioncode=="542422" & year==2012

replace regioncode="542423" if regioncode=="542423" & year==2012

replace regioncode="542424" if regioncode=="542424" & year==2012

replace regioncode="542425" if regioncode=="542425" & year==2012

replace regioncode="542426" if regioncode=="542426" & year==2012

replace regioncode="542427" if regioncode=="542427" & year==2012

replace regioncode="542429" if regioncode=="542429" & year==2012

replace regioncode="542430" if regioncode=="542430" & year==2012

replace regioncode="542523" if regioncode=="542523" & year==2012

replace regioncode="542621" if regioncode=="542621" & year==2012

replace regioncode="542622" if regioncode=="542622" & year==2012

replace regioncode="542623" if regioncode=="542623" & year==2012

replace regioncode="542625" if regioncode=="542625" & year==2012

replace regioncode="542626" if regioncode=="542626" & year==2012

replace regioncode="610102" if regioncode=="610102" & year==2012

replace regioncode="610103" if regioncode=="610103" & year==2012

replace regioncode="610104" if regioncode=="610104" & year==2012

replace regioncode="610111" if regioncode=="610111" & year==2012

replace regioncode="610112" if regioncode=="610112" & year==2012

replace regioncode="610113" if regioncode=="610113" & year==2012

replace regioncode="610114" if regioncode=="610114" & year==2012

replace regioncode="610115" if regioncode=="610115" & year==2012

replace regioncode="610116" if regioncode=="610116" & year==2012

replace regioncode="610122" if regioncode=="610122" & year==2012

replace regioncode="610124" if regioncode=="610124" & year==2012

replace regioncode="610125" if regioncode=="610125" & year==2012

replace regioncode="610126" if regioncode=="610126" & year==2012

replace regioncode="610202" if regioncode=="610202" & year==2012

replace regioncode="610203" if regioncode=="610203" & year==2012

replace regioncode="610204" if regioncode=="610204" & year==2012

replace regioncode="610222" if regioncode=="610222" & year==2012

replace regioncode="610302" if regioncode=="610302" & year==2012

replace regioncode="610303" if regioncode=="610303" & year==2012

replace regioncode="610304" if regioncode=="610304" & year==2012

replace regioncode="610322" if regioncode=="610322" & year==2012

replace regioncode="610323" if regioncode=="610323" & year==2012

replace regioncode="610324" if regioncode=="610324" & year==2012

replace regioncode="610326" if regioncode=="610326" & year==2012

replace regioncode="610327" if regioncode=="610327" & year==2012

replace regioncode="610328" if regioncode=="610328" & year==2012

replace regioncode="610329" if regioncode=="610329" & year==2012

replace regioncode="610330" if regioncode=="610330" & year==2012

replace regioncode="610331" if regioncode=="610331" & year==2012

replace regioncode="610402" if regioncode=="610402" & year==2012

replace regioncode="610403" if regioncode=="610403" & year==2012

replace regioncode="610404" if regioncode=="610404" & year==2012

replace regioncode="610422" if regioncode=="610422" & year==2012

replace regioncode="610423" if regioncode=="610423" & year==2012

replace regioncode="610424" if regioncode=="610424" & year==2012

replace regioncode="610425" if regioncode=="610425" & year==2012

replace regioncode="610426" if regioncode=="610426" & year==2012

replace regioncode="610427" if regioncode=="610427" & year==2012

replace regioncode="610428" if regioncode=="610428" & year==2012

replace regioncode="610429" if regioncode=="610429" & year==2012

replace regioncode="610430" if regioncode=="610430" & year==2012

replace regioncode="610431" if regioncode=="610431" & year==2012

replace regioncode="610481" if regioncode=="610481" & year==2012

replace regioncode="610502" if regioncode=="610502" & year==2012

replace regioncode="610521" if regioncode=="610521" & year==2012

replace regioncode="610522" if regioncode=="610522" & year==2012

replace regioncode="610523" if regioncode=="610523" & year==2012

replace regioncode="610524" if regioncode=="610524" & year==2012

replace regioncode="610525" if regioncode=="610525" & year==2012

replace regioncode="610526" if regioncode=="610526" & year==2012

replace regioncode="610527" if regioncode=="610527" & year==2012

replace regioncode="610528" if regioncode=="610528" & year==2012

replace regioncode="610581" if regioncode=="610581" & year==2012

replace regioncode="610582" if regioncode=="610582" & year==2012

replace regioncode="610602" if regioncode=="610602" & year==2012

replace regioncode="610621" if regioncode=="610621" & year==2012

replace regioncode="610622" if regioncode=="610622" & year==2012

replace regioncode="610623" if regioncode=="610623" & year==2012

replace regioncode="610624" if regioncode=="610624" & year==2012

replace regioncode="610625" if regioncode=="610625" & year==2012

replace regioncode="610626" if regioncode=="610626" & year==2012

replace regioncode="610627" if regioncode=="610627" & year==2012

replace regioncode="610628" if regioncode=="610628" & year==2012

replace regioncode="610629" if regioncode=="610629" & year==2012

replace regioncode="610630" if regioncode=="610630" & year==2012

replace regioncode="610631" if regioncode=="610631" & year==2012

replace regioncode="610632" if regioncode=="610632" & year==2012

replace regioncode="610702" if regioncode=="610702" & year==2012

replace regioncode="610721" if regioncode=="610721" & year==2012

replace regioncode="610722" if regioncode=="610722" & year==2012

replace regioncode="610723" if regioncode=="610723" & year==2012

replace regioncode="610724" if regioncode=="610724" & year==2012

replace regioncode="610725" if regioncode=="610725" & year==2012

replace regioncode="610726" if regioncode=="610726" & year==2012

replace regioncode="610727" if regioncode=="610727" & year==2012

replace regioncode="610728" if regioncode=="610728" & year==2012

replace regioncode="610729" if regioncode=="610729" & year==2012

replace regioncode="610730" if regioncode=="610730" & year==2012

replace regioncode="610802" if regioncode=="610802" & year==2012

replace regioncode="610821" if regioncode=="610821" & year==2012

replace regioncode="610822" if regioncode=="610822" & year==2012

replace regioncode="610823" if regioncode=="610823" & year==2012

replace regioncode="610824" if regioncode=="610824" & year==2012

replace regioncode="610825" if regioncode=="610825" & year==2012

replace regioncode="610826" if regioncode=="610826" & year==2012

replace regioncode="610827" if regioncode=="610827" & year==2012

replace regioncode="610828" if regioncode=="610828" & year==2012

replace regioncode="610829" if regioncode=="610829" & year==2012

replace regioncode="610830" if regioncode=="610830" & year==2012

replace regioncode="610831" if regioncode=="610831" & year==2012

replace regioncode="610902" if regioncode=="610902" & year==2012

replace regioncode="610921" if regioncode=="610921" & year==2012

replace regioncode="610922" if regioncode=="610922" & year==2012

replace regioncode="610923" if regioncode=="610923" & year==2012

replace regioncode="610924" if regioncode=="610924" & year==2012

replace regioncode="610925" if regioncode=="610925" & year==2012

replace regioncode="610926" if regioncode=="610926" & year==2012

replace regioncode="610927" if regioncode=="610927" & year==2012

replace regioncode="610928" if regioncode=="610928" & year==2012

replace regioncode="610929" if regioncode=="610929" & year==2012

replace regioncode="611002" if regioncode=="611002" & year==2012

replace regioncode="611021" if regioncode=="611021" & year==2012

replace regioncode="611022" if regioncode=="611022" & year==2012

replace regioncode="611023" if regioncode=="611023" & year==2012

replace regioncode="611024" if regioncode=="611024" & year==2012

replace regioncode="611025" if regioncode=="611025" & year==2012

replace regioncode="611026" if regioncode=="611026" & year==2012

replace regioncode="620102" if regioncode=="620102" & year==2012

replace regioncode="620103" if regioncode=="620103" & year==2012

replace regioncode="620104" if regioncode=="620104" & year==2012

replace regioncode="620105" if regioncode=="620105" & year==2012

replace regioncode="620111" if regioncode=="620111" & year==2012

replace regioncode="620121" if regioncode=="620121" & year==2012

replace regioncode="620122" if regioncode=="620122" & year==2012

replace regioncode="620123" if regioncode=="620123" & year==2012

replace regioncode="620201" if regioncode=="620201" & year==2012

replace regioncode="620302" if regioncode=="620302" & year==2012

replace regioncode="620321" if regioncode=="620321" & year==2012

replace regioncode="620402" if regioncode=="620402" & year==2012

replace regioncode="620403" if regioncode=="620403" & year==2012

replace regioncode="620421" if regioncode=="620421" & year==2012

replace regioncode="620422" if regioncode=="620422" & year==2012

replace regioncode="620423" if regioncode=="620423" & year==2012

replace regioncode="620502" if regioncode=="620502" & year==2012

replace regioncode="620503" if regioncode=="620503" & year==2012

replace regioncode="620521" if regioncode=="620521" & year==2012

replace regioncode="620522" if regioncode=="620522" & year==2012

replace regioncode="620523" if regioncode=="620523" & year==2012

replace regioncode="620524" if regioncode=="620524" & year==2012

replace regioncode="620525" if regioncode=="620525" & year==2012

replace regioncode="622301" if regioncode=="620602" & year==2012

replace regioncode="622322" if regioncode=="620621" & year==2012

replace regioncode="622323" if regioncode=="620622" & year==2012

replace regioncode="622326" if regioncode=="620623" & year==2012

replace regioncode="622201" if regioncode=="620702" & year==2012

replace regioncode="622222" if regioncode=="620721" & year==2012

replace regioncode="622223" if regioncode=="620722" & year==2012

replace regioncode="622224" if regioncode=="620723" & year==2012

replace regioncode="622225" if regioncode=="620724" & year==2012

replace regioncode="622226" if regioncode=="620725" & year==2012

replace regioncode="622701" if regioncode=="620802" & year==2012

replace regioncode="622722" if regioncode=="620821" & year==2012

replace regioncode="622723" if regioncode=="620822" & year==2012

replace regioncode="622724" if regioncode=="620823" & year==2012

replace regioncode="622725" if regioncode=="620824" & year==2012

replace regioncode="622726" if regioncode=="620825" & year==2012

replace regioncode="622727" if regioncode=="620826" & year==2012

replace regioncode="622102" if regioncode=="620902" & year==2012

replace regioncode="622123" if regioncode=="620921" & year==2012

replace regioncode="622126" if regioncode=="620922" & year==2012

replace regioncode="622124" if regioncode=="620923" & year==2012

replace regioncode="622125" if regioncode=="620924" & year==2012

replace regioncode="622101" if regioncode=="620981" & year==2012

replace regioncode="622103" if regioncode=="620982" & year==2012

replace regioncode="622801" if regioncode=="621002" & year==2012

replace regioncode="622821" if regioncode=="621021" & year==2012

replace regioncode="622822" if regioncode=="621022" & year==2012

replace regioncode="622823" if regioncode=="621023" & year==2012

replace regioncode="622824" if regioncode=="621024" & year==2012

replace regioncode="622825" if regioncode=="621025" & year==2012

replace regioncode="622826" if regioncode=="621026" & year==2012

replace regioncode="622827" if regioncode=="621027" & year==2012

replace regioncode="622421" if regioncode=="621102" & year==2012

replace regioncode="622424" if regioncode=="621121" & year==2012

replace regioncode="622425" if regioncode=="621122" & year==2012

replace regioncode="622426" if regioncode=="621123" & year==2012

replace regioncode="622427" if regioncode=="621124" & year==2012

replace regioncode="622428" if regioncode=="621125" & year==2012

replace regioncode="622429" if regioncode=="621126" & year==2012

replace regioncode="622621" if regioncode=="621202" & year==2012

replace regioncode="622624" if regioncode=="621221" & year==2012

replace regioncode="622626" if regioncode=="621222" & year==2012

replace regioncode="622623" if regioncode=="621223" & year==2012

replace regioncode="622625" if regioncode=="621224" & year==2012

replace regioncode="622627" if regioncode=="621225" & year==2012

replace regioncode="622628" if regioncode=="621226" & year==2012

replace regioncode="622630" if regioncode=="621227" & year==2012

replace regioncode="622629" if regioncode=="621228" & year==2012

replace regioncode="622901" if regioncode=="622901" & year==2012

replace regioncode="622921" if regioncode=="622921" & year==2012

replace regioncode="622922" if regioncode=="622922" & year==2012

replace regioncode="622923" if regioncode=="622923" & year==2012

replace regioncode="622924" if regioncode=="622924" & year==2012

replace regioncode="622925" if regioncode=="622925" & year==2012

replace regioncode="622926" if regioncode=="622926" & year==2012

replace regioncode="622927" if regioncode=="622927" & year==2012

replace regioncode="623001" if regioncode=="623001" & year==2012

replace regioncode="623021" if regioncode=="623021" & year==2012

replace regioncode="623022" if regioncode=="623022" & year==2012

replace regioncode="623023" if regioncode=="623023" & year==2012

replace regioncode="623024" if regioncode=="623024" & year==2012

replace regioncode="623025" if regioncode=="623025" & year==2012

replace regioncode="623026" if regioncode=="623026" & year==2012

replace regioncode="623027" if regioncode=="623027" & year==2012

replace regioncode="630102" if regioncode=="630102" & year==2012

replace regioncode="630103" if regioncode=="630103" & year==2012

replace regioncode="630104" if regioncode=="630104" & year==2012

replace regioncode="630105" if regioncode=="630105" & year==2012

replace regioncode="630121" if regioncode=="630121" & year==2012

replace regioncode="630122" if regioncode=="630122" & year==2012

replace regioncode="630123" if regioncode=="630123" & year==2012

replace regioncode="632121" if regioncode=="632121" & year==2012

replace regioncode="632122" if regioncode=="632122" & year==2012

replace regioncode="632123" if regioncode=="632123" & year==2012

replace regioncode="632126" if regioncode=="632126" & year==2012

replace regioncode="632127" if regioncode=="632127" & year==2012

replace regioncode="632128" if regioncode=="632128" & year==2012

replace regioncode="632221" if regioncode=="632221" & year==2012

replace regioncode="632222" if regioncode=="632222" & year==2012

replace regioncode="632223" if regioncode=="632223" & year==2012

replace regioncode="632224" if regioncode=="632224" & year==2012

replace regioncode="632321" if regioncode=="632321" & year==2012

replace regioncode="632322" if regioncode=="632322" & year==2012

replace regioncode="632521" if regioncode=="632521" & year==2012

replace regioncode="632522" if regioncode=="632522" & year==2012

replace regioncode="632523" if regioncode=="632523" & year==2012

replace regioncode="632524" if regioncode=="632524" & year==2012

replace regioncode="632525" if regioncode=="632525" & year==2012

replace regioncode="632621" if regioncode=="632621" & year==2012

replace regioncode="632622" if regioncode=="632622" & year==2012

replace regioncode="632623" if regioncode=="632623" & year==2012

replace regioncode="632624" if regioncode=="632624" & year==2012

replace regioncode="632625" if regioncode=="632625" & year==2012

replace regioncode="632626" if regioncode=="632626" & year==2012

replace regioncode="632721" if regioncode=="632721" & year==2012

replace regioncode="632723" if regioncode=="632723" & year==2012

replace regioncode="632724" if regioncode=="632724" & year==2012

replace regioncode="632725" if regioncode=="632725" & year==2012

replace regioncode="632726" if regioncode=="632726" & year==2012

replace regioncode="632801" if regioncode=="632801" & year==2012

replace regioncode="632802" if regioncode=="632802" & year==2012

replace regioncode="632821" if regioncode=="632821" & year==2012

replace regioncode="632822" if regioncode=="632822" & year==2012

replace regioncode="640104" if regioncode=="640104" & year==2012

replace regioncode="640105" if regioncode=="640105" & year==2012

replace regioncode="640106" if regioncode=="640106" & year==2012

replace regioncode="640121" if regioncode=="640121" & year==2012

replace regioncode="640122" if regioncode=="640122" & year==2012

replace regioncode="640181" if regioncode=="640181" & year==2012

replace regioncode="640202" if regioncode=="640202" & year==2012

replace regioncode="640203" if regioncode=="640205" & year==2012

replace regioncode="640221" if regioncode=="640221" & year==2012

replace regioncode="640302" if regioncode=="640302" & year==2012

replace regioncode="640321" if regioncode=="640502" & year==2012

replace regioncode="640322" if regioncode=="640521" & year==2012

replace regioncode="640323" if regioncode=="640323" & year==2012

replace regioncode="640324" if regioncode=="640324" & year==2012

replace regioncode="640381" if regioncode=="640381" & year==2012

replace regioncode="640402" if regioncode=="640402" & year==2012

replace regioncode="640421" if regioncode=="640522" & year==2012

replace regioncode="640422" if regioncode=="640422" & year==2012

replace regioncode="640423" if regioncode=="640423" & year==2012

replace regioncode="640424" if regioncode=="640424" & year==2012

replace regioncode="640425" if regioncode=="640425" & year==2012

replace regioncode="650102" if regioncode=="650102" & year==2012

replace regioncode="650103" if regioncode=="650103" & year==2012

replace regioncode="650104" if regioncode=="650104" & year==2012

replace regioncode="650105" if regioncode=="650105" & year==2012

replace regioncode="650106" if regioncode=="650106" & year==2012

replace regioncode="650107" if regioncode=="650107" & year==2012

replace regioncode="650108" if regioncode=="650109" & year==2012

replace regioncode="650121" if regioncode=="650121" & year==2012

replace regioncode="650202" if regioncode=="650202" & year==2012

replace regioncode="650203" if regioncode=="650203" & year==2012

replace regioncode="650204" if regioncode=="650204" & year==2012

replace regioncode="650205" if regioncode=="650205" & year==2012

replace regioncode="652101" if regioncode=="652101" & year==2012

replace regioncode="652122" if regioncode=="652122" & year==2012

replace regioncode="652123" if regioncode=="652123" & year==2012

replace regioncode="652201" if regioncode=="652201" & year==2012

replace regioncode="652222" if regioncode=="652222" & year==2012

replace regioncode="652223" if regioncode=="652223" & year==2012

replace regioncode="652301" if regioncode=="652301" & year==2012

replace regioncode="652302" if regioncode=="652302" & year==2012

replace regioncode="652303" if regioncode=="652303" & year==2012

replace regioncode="652323" if regioncode=="652323" & year==2012

replace regioncode="652324" if regioncode=="652324" & year==2012

replace regioncode="652325" if regioncode=="652325" & year==2012

replace regioncode="652327" if regioncode=="652327" & year==2012

replace regioncode="652328" if regioncode=="652328" & year==2012

replace regioncode="652701" if regioncode=="652701" & year==2012

replace regioncode="652722" if regioncode=="652722" & year==2012

replace regioncode="652723" if regioncode=="652723" & year==2012

replace regioncode="652801" if regioncode=="652801" & year==2012

replace regioncode="652822" if regioncode=="652822" & year==2012

replace regioncode="652823" if regioncode=="652823" & year==2012

replace regioncode="652824" if regioncode=="652824" & year==2012

replace regioncode="652825" if regioncode=="652825" & year==2012

replace regioncode="652826" if regioncode=="652826" & year==2012

replace regioncode="652827" if regioncode=="652827" & year==2012

replace regioncode="652828" if regioncode=="652828" & year==2012

replace regioncode="652829" if regioncode=="652829" & year==2012

replace regioncode="652901" if regioncode=="652901" & year==2012

replace regioncode="652922" if regioncode=="652922" & year==2012

replace regioncode="652923" if regioncode=="652923" & year==2012

replace regioncode="652924" if regioncode=="652924" & year==2012

replace regioncode="652925" if regioncode=="652925" & year==2012

replace regioncode="652926" if regioncode=="652926" & year==2012

replace regioncode="652928" if regioncode=="652928" & year==2012

replace regioncode="652929" if regioncode=="652929" & year==2012

replace regioncode="653001" if regioncode=="653001" & year==2012

replace regioncode="653022" if regioncode=="653022" & year==2012

replace regioncode="653023" if regioncode=="653023" & year==2012

replace regioncode="653024" if regioncode=="653024" & year==2012

replace regioncode="653101" if regioncode=="653101" & year==2012

replace regioncode="653121" if regioncode=="653121" & year==2012

replace regioncode="653122" if regioncode=="653122" & year==2012

replace regioncode="653123" if regioncode=="653123" & year==2012

replace regioncode="653124" if regioncode=="653124" & year==2012

replace regioncode="653125" if regioncode=="653125" & year==2012

replace regioncode="653126" if regioncode=="653126" & year==2012

replace regioncode="653127" if regioncode=="653127" & year==2012

replace regioncode="653128" if regioncode=="653128" & year==2012

replace regioncode="653129" if regioncode=="653129" & year==2012

replace regioncode="653130" if regioncode=="653130" & year==2012

replace regioncode="653131" if regioncode=="653131" & year==2012

replace regioncode="653201" if regioncode=="653201" & year==2012

replace regioncode="653221" if regioncode=="653221" & year==2012

replace regioncode="653222" if regioncode=="653222" & year==2012

replace regioncode="653223" if regioncode=="653223" & year==2012

replace regioncode="653224" if regioncode=="653224" & year==2012

replace regioncode="653225" if regioncode=="653225" & year==2012

replace regioncode="653226" if regioncode=="653226" & year==2012

replace regioncode="653227" if regioncode=="653227" & year==2012

replace regioncode="654002" if regioncode=="654002" & year==2012

replace regioncode="654003" if regioncode=="654003" & year==2012

replace regioncode="654021" if regioncode=="654021" & year==2012

replace regioncode="654022" if regioncode=="654022" & year==2012

replace regioncode="654023" if regioncode=="654023" & year==2012

replace regioncode="654024" if regioncode=="654024" & year==2012

replace regioncode="654025" if regioncode=="654025" & year==2012

replace regioncode="654026" if regioncode=="654026" & year==2012

replace regioncode="654027" if regioncode=="654027" & year==2012

replace regioncode="654028" if regioncode=="654028" & year==2012

replace regioncode="654201" if regioncode=="654201" & year==2012

replace regioncode="654202" if regioncode=="654202" & year==2012

replace regioncode="654221" if regioncode=="654221" & year==2012

replace regioncode="654223" if regioncode=="654223" & year==2012

replace regioncode="654224" if regioncode=="654224" & year==2012

replace regioncode="654225" if regioncode=="654225" & year==2012

replace regioncode="654226" if regioncode=="654226" & year==2012

replace regioncode="654301" if regioncode=="654301" & year==2012

replace regioncode="654321" if regioncode=="654321" & year==2012

replace regioncode="654322" if regioncode=="654322" & year==2012

replace regioncode="654323" if regioncode=="654323" & year==2012

replace regioncode="654324" if regioncode=="654324" & year==2012

replace regioncode="654325" if regioncode=="654325" & year==2012

replace regioncode="654326" if regioncode=="654326" & year==2012

replace regioncode="659001" if regioncode=="659001" & year==2012
